# The value of protein structure classification information—Surveying the scientific literature

**DOI:** 10.1002/prot.24915

**Published:** 2015-09-19

**Authors:** Naomi K. Fox, Steven E. Brenner, John‐Marc Chandonia

**Affiliations:** ^1^ Lawrence Berkeley National LaboratoryPhysical Biosciences DivisionBerkeleyCalifornia94720; ^2^ Department of Plant and Microbial BiologyUniversity of CaliforniaBerkeleyCalifornia94720

**Keywords:** SCOP, CATH, database, curation, resources

## Abstract

The Structural Classification of Proteins (SCOP) and Class, Architecture, Topology, Homology (CATH) databases have been valuable resources for protein structure classification for over 20 years. Development of SCOP (version 1) concluded in June 2009 with SCOP 1.75. The SCOPe (SCOP–extended) database offers continued development of the classic SCOP hierarchy, adding over 33,000 structures. We have attempted to assess the impact of these two decade old resources and guide future development. To this end, we surveyed recent articles to learn how structure classification data are used. Of 571 articles published in 2012–2013 that cite SCOP, 439 actually use data from the resource. We found that the type of use was fairly evenly distributed among four top categories: A) study protein structure or evolution (27% of articles), B) train and/or benchmark algorithms (28% of articles), C) augment non‐SCOP datasets with SCOP classification (21% of articles), and D) examine the classification of one protein/a small set of proteins (22% of articles). Most articles described computational research, although 11% described purely experimental research, and a further 9% included both. We examined how CATH and SCOP were used in 158 articles that cited both databases: while some studies used only one dataset, the majority used data from both resources. Protein structure classification remains highly relevant for a diverse range of problems and settings. Proteins 2015; 83:2025–2038. © 2015 The Authors. Proteins: Structure, Function, and Bioinformatics Published by Wiley Periodicals, Inc.

**Abbreviations**
ACO: absolute contact order
CATH: Class, Architecture, Topology, Homology
MPO: myeloperoxidase
PSCDB: Protein Structural Change Database
SCOP: Structural Classification of Proteins

## INTRODUCTION

Nearly all proteins have structural similarities with other proteins, and in many of these cases, share a common evolutionary origin. Protein structure classification databases, such as the Structural Classification of Proteins (SCOP) and Class, Architecture, Topology, Homology (CATH) resources, aim to provide detailed and comprehensive categorization of all proteins of known structure.^1^, ^2^, ^3^ These resources provide broad surveys of known protein folds and detailed information about the structurally characterized relatives of any classified protein.

Since the first public release of a comprehensive protein structure classification database in 1994, the size of the PDB has increased by a factor of more than 60.^4^, ^5^ Although past researchers could simply browse the SCOP or CATH hierarchies to examine proteins of interest and their close relatives, as well as to view a panorama of the entirety of protein structures, both databases have now grown to the point where simple browsing is less appealing. We therefore investigated the ways in which protein structure classification databases are currently used, including the question of why researchers might choose to use one instead of another.

**Figure 1:**
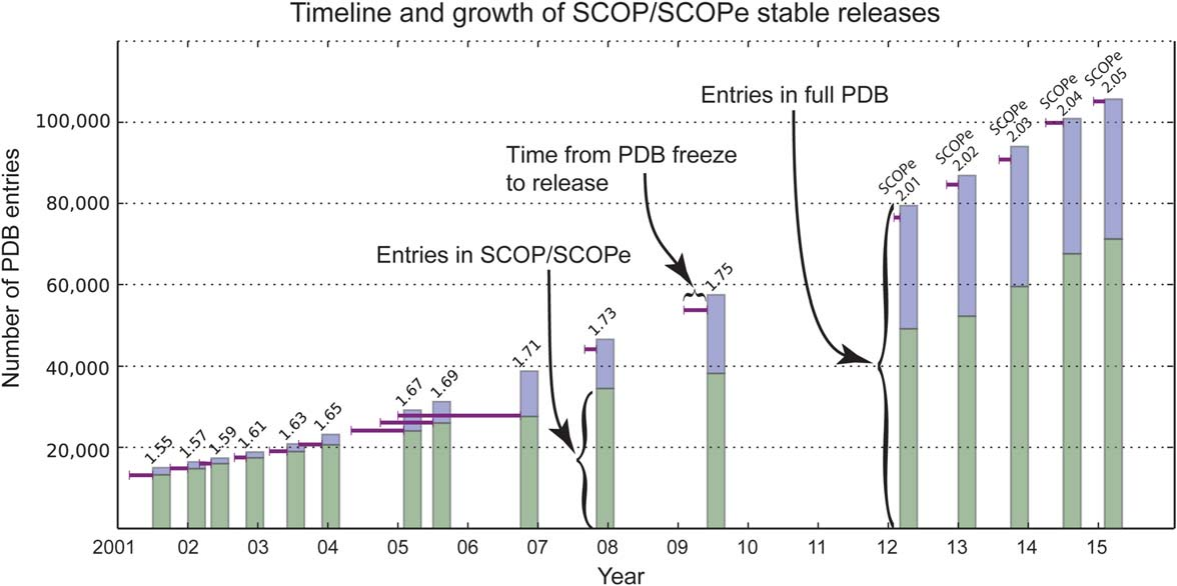
Timeline of releases showing the number of structures in the PDB and SCOP/SCOPe at each release date. The extended horizontal lines start at the PDB freeze dates for each release and indicate the time required to release each version. The height of each vertical bar corresponds to the number of structures in the PDB on the release date, and the height of the horizontal line on the bar corresponds to the number of structures in the PDB on the freeze date. The bottom portion of each bar measures the number of PDB entries classified in each version of SCOP or SCOPe. Releases through 1.71 were comprehensive—all structures at the freeze date were classified, though the last comprehensive release, 1.71, was released 21 months after the freeze date. Releases since then have not been comprehensive. [Color figure can be viewed in the online issue, which is available at wileyonlinelibrary.com.]

For historical context, SCOP and *Yahoo!* both were initiated in 1994 as manually curated hierarchical classifications.^6^ *Yahoo! Directory*, the original core of the company, was largely supplanted by crawler‐based search engines in 2002, and was shut down at the end of 2014. By analogy, now is an opportune time to assess the value of manually curated structure classifications. Although development of SCOP (version 1) concluded in June 2009 with SCOP 1.75, the SCOPe (SCOP–extended, http://scop.berkeley.edu) database, maintained by our group, offers continued development of the classic SCOP hierarchy.^7^ Since 2006, SCOP and SCOPe have augmented human expert curation with automated methods to keep up with the flood of new structures. The current release of SCOPe classifies 72,092 protein structures, 33,871 more than SCOP 1.75 (see Fig. 1).

In this manuscript, we investigate the main uses of protein structure classification data today as referenced in the literature, focusing on SCOP as an exemplar. The most straightforward way to access the SCOP classification is via the web interface. For example, a researcher might use the website to search for a protein of interest in order to show its place in the hierarchy or browse its homologs. For more in‐depth computational analyses, data files containing the entire SCOP classification, or representative subsets, may be downloaded for offline processing. These data can be used for training or benchmarking new methods, or for surveying general properties of proteins. Because several early releases of SCOPe were originally branded as new SCOP releases, some of the studies that cite SCOP are actually using SCOPe.

By analogy with taxonomy, the SCOP classification is a hierarchy of several levels where the fundamental unit of classification is a *domain* in an experimentally determined protein structure. The hierarchy of domains comprises the following levels: *species domain* representing a distinct protein sequence in an organism and its naturally occurring or artificially created variants; *protein* grouping together similar sequences of essentially the same functions that either originate from different biological species or represent different isoforms within the same species; *family* containing proteins with similar sequences but typically distinct functions and *superfamily* bridging together protein families with similar features inferred to be from a common evolutionary ancestor. Near the root, the basis of classification is purely structural: structurally similar *superfamilies* are grouped into *folds*, which are further arranged into *classes* based mainly on their secondary structure content and organization. (Further details are discussed in a prior review^2^).

Many people used SCOP for its signature feature, the structure classification. However, a large number also made use of its other aspects. We thus investigated how researchers use other resources provided by SCOP and its sister database ASTRAL.^8^, ^9^ These include domain boundaries that annotate which amino acid residues in a structure belong to which domain, for multi‐domain proteins. SCOP defines a domain as an evolutionarily conserved unit (as opposed to other common definitions of a domain, for example, based on structural compactness), so analyses of the evolution of multi‐domain structures rely on domain boundary annotations to avoid including non‐homologous regions of sequence. The ASTRAL database provides sequences and PDB‐style coordinate files for individual SCOP domains, as well as sequences of PDB chains that are classified; having SCOP data in these formats is often convenient or necessary for bioinformatic analyses. ASTRAL also provides representative subsets of proteins that span the set of classified protein structures or domains while alleviating bias toward well‐studied proteins; these subsets are commonly used for benchmarking algorithms to demonstrate their effectiveness on a representative dataset of protein structures. The representative in each subset of similar domains is chosen using AEROSPACI scores,^9^ a numeric score that estimates the quality and precision of crystallographically determined structures.

In this manuscript, we investigate the ways that SCOP and CATH are used in recent research. We have surveyed the literature to identify broad categories of SCOP use, and have classified 571 recent research articles (published in 2012–2013) into these categories. Our survey includes 158 studies that cite both SCOP and CATH, and we investigated these further in order to study how researchers used both databases, and why they may have chosen one or the other. We discuss the results of our categorization and how the lessons learned might help to direct the future of SCOPe and similar protein structure classification databases.

## MATERIALS AND METHODS

### SCOP‐citing papers

We began our literature survey by retrieving a list of all 571 articles published in 2012 and 2013 that cite a SCOP^1^, ^2^, ^5^, ^10^, ^11^, ^12^, ^13^, ^14^, ^15^ or ASTRAL^8^, ^9^, ^16^ article (henceforth, referred to as SCOP‐citing papers). This search was performed via the Web of Science (http://wokinfo.com), a curated database of publications spanning every scientific discipline. We also used Web of Science to count the number of citations to each of the SCOP‐citing articles as of 10 December 2014. We collected the 546 articles from the list that were available from the University of California, Berkeley library and manually examined each article to determine whether the SCOP classification was used in the study, or alternatively, if SCOP was merely cited for background. We also retrieved a list of the 25 most highly cited SCOP‐citing papers from the Web of Science. These articles were published between 1996 and 2009 and each has been cited >1,000 times. A list of the 158 articles from 2012–2013 that cited both a CATH article^3^, ^17^, ^18^, ^19^, ^20^, ^21^, ^22^, ^23^ and a SCOP or ASTRAL article was also retrieved from Web of Science. A list of all articles used in this study is available in the Supporting Information.

To search for articles that mention SCOP but do not cite a SCOP article, we used PubMed Central (http://www.ncbi.nlm.nih.gov/pmc/), a repository of papers from participating journals that are fully searchable. Use of PubMed Central was necessary because the Web of Science does not support full‐text searches.

### Website statistics

Access and referrer logs from the site http://scop.berkeley.edu/ were produced by the Apache HTTP server, and were used to analyze visitor patterns for three weeks from 13 January 2013 to 6 February 2013. Apache access logs from the month of March 2014 were used to identify the IP addresses of all computers used to visit the site, and the logresolve program from the Apache Software Foundation was used to resolve the IP addresses to hostnames. Robots and search engines were excluded by manual inspection of the resolved hostnames.

### Protein family statistics

To identify Pfam^24^ families that have structures classified in SCOP, and families that have been structurally characterized but do not appear in SCOP, we used HMMER version 3.1b2^25^ to search all hidden Markov models from Pfam version 27.0 against all PDB chain sequences, obtained from the SCOPe database.^7^ We considered only matches that scored at or above the trusted cutoff for each Pfam family, for which the alignment comprised at least 75% of the Pfam model.

## RESULTS AND DISCUSSION

### Six categories of SCOP use

**Figure 2:**
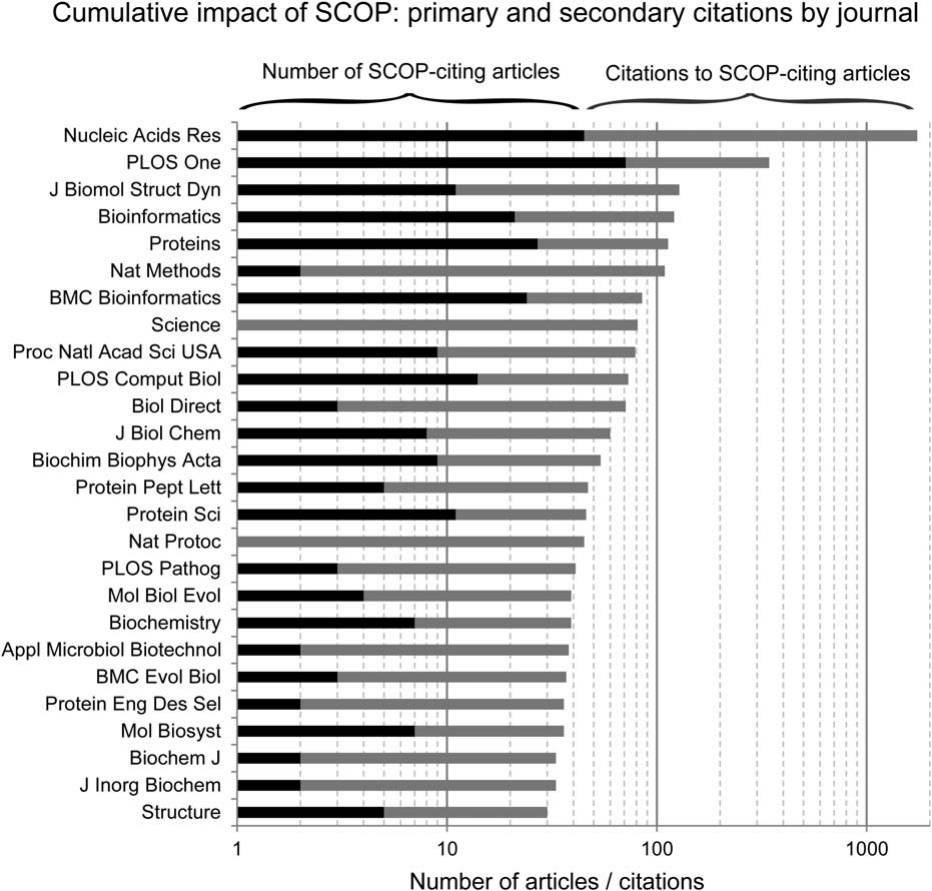
Number of SCOP‐citing articles published in journals from 2012–2013, and the total number of citations to these articles as of 10 December 2014. Journals with at least 30 cumulative citations (that is, SCOP‐citing papers and citations to such articles) are shown.

We identified 571 SCOP‐citing papers published in 2012 and 2013. These papers were written by >2,000 authors and appear in 171 unique journals. Figure 2 shows the number of papers appearing in each journal, as well as the number of citations to these papers (an approximation of each manuscript's scientific impact), for every journal that resulted in at least 30 cumulative citations. For example, there were 45 SCOP‐citing articles published in *Nucleic Acids Research* in 2012–2013 that were later cited in 1,695 articles (from any journal), resulting in the highest overall cumulative citation count of 1,740. Of the 546 articles from the list that were available from the University of California, Berkeley library, we determined that 439 (80%) of the available SCOP‐citing articles made use of the SCOP classification data.

Through an iterative process, we identified six broad categories that characterize the ways in which SCOP is used today. Our goal in designing this categorization was to get a broad sense of the different modes and types of uses of structural classification. To define categories, we first built an annotated bibliography of our corpus, noting how SCOP was used in each article. As we observed commonalities between papers, we began organizing them into a preliminary set of categories. Over several refinements, we broadened and narrowed the categories so that each contained a sufficiently representative set of papers. The article classification and annotated bibliography are available in the Supporting Information.

**Figure 3:**
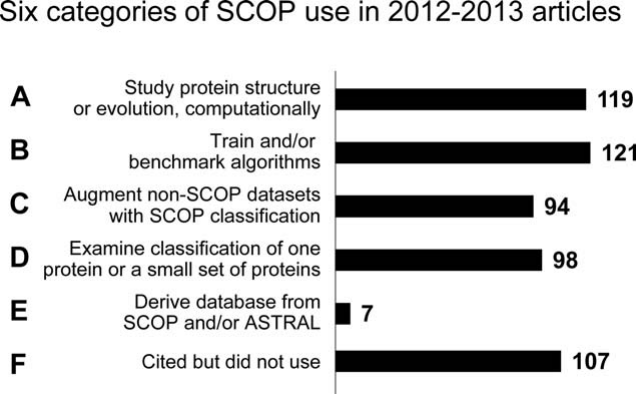
Categorization of SCOP use in 2012–2013 research articles. We devised a categorization of six major ways in which researchers used SCOP data, and placed each article into a single category, based on the primary use in that article.

We placed each of the 546 available studies that cited SCOP into exactly one of these categories, depending on the primary way in which the data were used. Our categorization is shown in Figure 3, and discussed in detail below.

#### SCOP use category A: Study protein structure or evolution, computationally

The second largest category was composed of the 119 articles that used SCOP to perform computational studies of properties that are common to, or differentiated in, different structural classes, folds, superfamilies, or families. This includes cases where the SCOP classification was examined directly to study protein structure and evolution across the complete set of known protein structures as deposited in the PDB, or within special categories, such as a particular SCOP superfamily or fold, or all two‐domain chains. A requirement for placing a article into this category was that the dataset analyzed was derived directly from SCOP or ASTRAL.

Following are some examples of studies using SCOP that we placed into this category.

**Example 1**: *Study evolution of protein folds*. Edwards, Abeln, and Deane studied fold preference in ancient and younger superfamilies using a phylogenetic‐based approach to estimate the relative age of SCOP superfamilies and categorize each as either “ancient,” “middle‐age,” or “new‐born.”^26^ ASTRAL representative sets were then used to collect structural representatives for each superfamily, and statistics about various structural properties were collected to characterize each superfamily, such as distributions of structural class, domain length, amino acid frequency, and hydrophobicity. The results led the authors to infer that, overall, a shorter evolutionary history corresponds to a less elaborate structure. They also drew the conclusion that the jelly roll motif is significantly younger than the greek key.

**Example 2:** *Study evolution of oligomer geometries*. In a study of evolution of different oligomeric states by Perica, Chothia, and Teichmann, structures were collected from 10 SCOP families that have “at least one dimer and one homologous tetramer or hexamer with the same dimeric binding mode.”^27^ Cyrus Chothia, an author of this article, is also a SCOP author. The study detected locations of mutations that were correlated with different oligomerization states and found that “such indirect, or allosteric mutations affecting intersubunit geometry via indirect mechanisms are as important as interface sequence changes for evolution of oligomeric states.”

**Figure 4:**
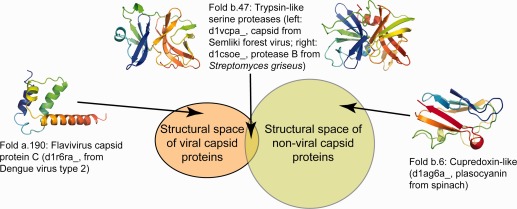
The SCOP classification and ASTRAL representative sets were used by Cheng and Brooks in a study that shows that viral capsid proteins are significantly segregated in structure space from all other proteins.^28^ The figure is adapted from Figure 2 in Cheng and Brooks's article, and shows a Venn diagram (not to scale) indicating that viral capsid structures share significantly less similarity than expected by chance with folds that contain no viral capsid structures. Examples show SCOP folds that are specific to viral capsid proteins (a.190), shared by both viral capsid and non‐capsid proteins (b.47, trypsin‐like serine proteases), and those without any structurally similar viral‐capsid folds (b.6, Cupredoxin‐like). Cheng and Brooks conclude that viral capsids evolved under distinct evolutionary constraints from non‐capsid proteins, and may provide valuable templates for protein engineering. Their study made use of two datasets: viral capsid domains derived from ViperDB and SCOP, and non‐capsid domains derived from SCOP. Domain structures were retrieved from ASTRAL.

**Example 3**: *Study viral fold specificity*. The SCOP classification was used by Cheng and Brooks to study fold diversity in viral capsid proteins.^28^ A representative set of domain structures was retrieved from ASTRAL, covering 1,047 folds, including the 21 folds that either contain or are highly similar to viral capsid structures. A structural clustering of representatives revealed that viral capsid proteins are segregated in fold space from folds with no viral capsid domains. Cheng and Brooks conclude that viral capsids evolved under distinct evolutionary constraints from non‐capsid proteins, and may provide valuable templates for protein engineering. Figure 4 illustrates how SCOP was used in the study.

**Example 4**: *Study properties of a common fold*. An article by Vijayabaskar and Vishveshwara presented a computational study of the contribution of non‐covalent interactions to (β/α)_8_ barrel fold stability^29^ The dataset was derived from all domains with the (β/α)_8_ barrel fold retrieved from ASTRAL and categorized by SCOP family. Despite possible lack of homology, the study found shared interactions among different families and therefore generalized the patterns of interactions by which the fold is maintained. Such knowledge may aid future efforts in protein design.

**Example 5**: *Search for homologs*. Many studies use SCOP to collect domain homologs. For example, the structure and function of archaeal endonuclease Nob1, which is involved in ribosome assembly, was examined in one study by Veith *et al*. through structure determination, sequence alignment, phylogenetic analysis, biochemical assays, and database comparison.^30^ A dataset of remote homologs of two important domains in Nob1, the PIN and zinc ribbon domains, was derived from the domains' respective superfamilies in SCOP. The domains were structurally aligned with the newly determined NMR structure for Nob1 from *Pyrococcus horikoshii* to find the most structurally similar domains, enabling a structural and functional comparison. These results show that both the domain structure and function of Nob1 are conserved between archaea and eukaryotes.

#### SCOP use category B: Train and/or benchmark algorithms

The most common use of the SCOP classification was to aid development of algorithms for applications such as sequence alignment and structure prediction. In most of these articles, the SCOP classification was used to benchmark the performance of algorithms, or to provide training sets to aid in setting parameter values. Most often, authors used SCOP data via ASTRAL resources. We placed 121 articles into this category. Following are examples in which SCOP was used for developing and evaluating new methods.

**Example 1**: *Measure accuracy of class, fold, superfamily, and family predictions*. Mach and Koehl used the SCOP classification in the validation of a method related to protein design.^31^ Patrice Koehl is also an ASTRAL author. Fold recognition is an especially challenging task for members of families with very few representatives in sequence databases. To address this challenge, the authors developed a method to expand the number of representatives by using a Monte Carlo‐based approach to design new sequences for these families. To evaluate whether the designed sequences increased recognition ability and accuracy, HMMER profiles were built for all families and used to predict SCOP class, fold, superfamily, and family on a test set of proteins. The set of seed sequences used for generating the designed sequences, and the test set of homologous sequences, were derived from ASTRAL representative sets. The recognition rate (that is, recall) of the profile library built with their method was 35%. To compare, using profiles built with no designed sequences resulted in a near‐0% recognition rate. The predictions were also highly accurate: 93% were correct at the structural class level, 90% at the fold level, 89% at the superfamily level, and 88% at the family level.

**Example 2**: *Train a structure refinement algorithm on a non‐redundant dataset of structures derived from ASTRAL*. A method by Moore and colleagues for converting a coarse‐grained structure to a full‐atom main‐chain structure used a training set derived from ASTRAL and SCOPe 2.01 (then called ASTRAL and SCOP 1.75A) domain structures.^32^ Lower quality structures were excluded using a cutoff on AEROSPACI scores, which are estimates of structure quality provided by ASTRAL.^9^ Each structure in the training set was then decomposed into fragments for training a Gaussian mixture model. Once trained, the method was tested on a set of 28 structures that had been recently deposited to the PDB but not yet classified in SCOP/SCOPe (and therefore not included in the training set). α‐Carbon traces were taken for each structure in the test set, and then the method was used to calculate an all‐atom main‐chain structure. RMSDs between the predicted and PDB structures were calculated and compared to those from six previously published methods. Moore's algorithm had the lowest mean RMSD across the test set.

**Example 3**: *Evaluate a protein‐interaction prediction algorithm on a non‐redundant benchmarking dataset*. The Multi‐VORFFIP method of Segura and colleagues extends a previous protein–protein interaction and binding site prediction method by also predicting binding sites for peptides, DNA, and RNA in proteins.^33^ Datasets of complexes for each interaction type were used to evaluate how well the new method discriminates between the four different types of interaction sites. For protein–protein interactions, a popular protein‐docking benchmark dataset was used that consists of 176 protein–protein complexes where no two complexes contain proteins from the same pair of SCOP families.^34^ The evaluation showed that Multi‐VORFFIP had significant discriminative power between the four interaction types.

**Example 4**: *Evaluate structure resolution method on a structurally diverse dataset*. Morimoto and colleagues presented a method for structure determination using small‐angle X‐ray scattering (SAXS) constraints combined with NMR‐derived distance restraints for local geometry. To demonstrate that their method worked on a broad range of proteins, they benchmarked it on a dataset consisting of eight proteins with different SCOP folds, from four different SCOP classes.^35^ Reference structures were calculated using full sets of NMR restraints. The authors compared the mean RMSD values calculated between the references and the ensembles produced by their method using the local geometry NMR restraints alone and combined with SAXS constraints for the 8 proteins. The results show lower mean RMSD values when SAXS data were included than when the NMR data were used alone. This result was consistent across the test set, showing that the method works well across different protein folds and structural classes.

#### SCOP use category C: Augment non‐SCOP datasets with SCOP classification

The SCOP classification can offer additional information about a dataset gathered from the literature or from another database. Structural or evolutionary diversity can be assessed by counting the number of entries that belong to different SCOP classes, folds, or superfamilies. We placed 94 articles into this category. We differentiate this from the earlier categories because the datasets were not derived directly from SCOP, but instead were compiled from a different source, then further annotated using the SCOP classification. The following are two examples of studies that made use of the SCOP classification to study properties of particular classes of proteins.

**Example 1**: *Study structural diversity of domain‐swapping structures*. Examining the SCOP classifications for a dataset of interest can assess the prevalence of properties of interest across different classes, folds, superfamilies, and so forth The SCOP classification was used in a study of domain swapping by Huang *et al*.^36^ The study investigated whether three‐dimensional domain swapping is a general property of all proteins, or is segregated in fold space. A dataset of 500 single‐domain proteins known to include domain‐swapped structures was compiled from the literature and other databases, and then each domain was labeled with its SCOP class, fold, superfamily, and family. The study found 10% of all protein folds and 5% of all families contain domain swapped structures, and found a diversity of ways domains could be swapped.

**Example 2**: *Study cotranslational folding of multi‐domain proteins using domain boundaries defined in SCOP and CATH*. SCOP and CATH domain boundary definitions were used in a computational study of cotranslational folding conducted by Ciryam and colleagues.^37^ Cotranslational folding is a phenomenon of multi‐domain chains in which one domain folds into the native state while the chain is still undergoing ribosomal translation. In the study, folding was modeled with kinetic equations to compute the probability that a domain is folded as a function of the nascent chain length. The resulting plots are termed “cotranslational folding curves.” These curves can then be compared for different translation rates. Domain boundaries for a dataset of approximately 1,300 cytosolic *E. coli* protein structures were annotated using SCOP or CATH, or domain prediction software was used when neither database had classified the structure. Since SCOP defines domains as evolutionary conserved units, not all the domains collected corresponded to autonomous folding units, so some SCOP domains were split further by the authors. The final set of domains was labeled with the structural class of each domain, which was used for setting parameters in the kinetic model. The main conclusion of the study was that about one‐third of *E. coli* cytosolic proteins exhibit cotranslational folding under *in vivo* conditions.

#### SCOP use category D: Examine classification of one protein or a small set of proteins

The SCOP website can be used to browse structural and evolutionary properties of a fold, superfamily, family, or protein of interest. This category, in which we placed 98 articles, represents the set of users that browse SCOP directly for reference. In particular, studies using laboratory experimental methods often fell under this category. One criterion we used for categorizing an article into this category is whether they likely retrieved information by browsing the SCOP website, without needing to download a dataset for further processing. Following are two examples of studies that we placed into this category.

**Example 1**: *Retrieve SCOP fold classifications for structural comparison*. A study of GRAS family proteins by Zhang and colleagues used sequence‐profile searches, structural comparisons and phylogenetic analysis to establish that GRAS family proteins belong to the Rossmann fold methyltransferase SCOP superfamily.^38^ The authors reference SCOP to dispute the results of a previous study that reported a relationship between GRAS family proteins and STAT proteins. They show that the fold adopted by STAT DNA‐binding protein domains is incompatible with the predicted secondary structure for GRAS domains. According to the article, “The STAT‐type DNA‐binding domains adopt a cytochrome f‐like β‐sandwich fold, whereas the SH2 domain adopts a β‐barrel structure, both of which are incompatible with the predicted secondary structure of the GRAS domain.”

**Example 2**: *Report SCOP classification of a protein of interest, for context*. The SCOP superfamily classification of a protein of interest is referenced by Geoghegan and colleagues in their study of myeloperoxidase (MPO) using multistage mass spectrometry.^39^ The article notes that “the structure of MPO belongs to the heme‐dependent peroxidase superfamily, consisting of 26 α‐helices arranged around the central heme moiety.” The superfamily classification provides more evolutionary context for the protein beyond its more closely related homologs.

#### SCOP use category E: Derive database from SCOP and/or ASTRAL

The final category of articles that use SCOP data includes databases derived from the SCOP classification. Although this is a substantially smaller category than the previous categories, containing just 7 articles, we chose to retain it as a separate category because of potential impact of these databases. Many articles that cite SCOP use other databases derived from SCOP data. For example, the highly cited SUPERFAMILY database is comprised of HMM models for each SCOP superfamily.^40^ The following are two examples of articles that we placed into this category.

**Example 1**: *Ensure broad coverage in a database of bound and unbound proteins*. The SCOP classification has been used to ensure broad coverage of different folds and families in the Protein Structural Change Database (PSCDB) by Amemiya and colleagues.^41^ To curate the database, ligand‐free and ligand‐bound pairs of structures of the same protein were collected from the PDB. For each SCOP family, the pair with the greatest degree of conformational change was added to the database.

**Example 2**: *Provide structural alignments of all domains in a SCOP superfamily*. Databases have been developed that augment the SCOP database with additional data that can be used in studies of structure and evolution. The PASS2 database, by Gandhimathi and colleagues, provides structure‐based sequence alignments of domains in SCOP superfamilies, as ASTRAL no longer provides these.^42^ The PASS2 database has been used to study superfamily structural outliers.^43^

##### SCOP use category F: Cited for background

Finally, we found that approximately one fifth of SCOP‐citing studies did not make use of the classification itself, but cited the database for background. For example, a study of the evolution of novel enzyme function in superfamilies^44^ cites SCOP as an exemplar of a structurally based classification of superfamilies. Another study that assesses the accuracy of structure prediction methods^45^ refers to another assessment method that used the SCOP classification as a gold standard. Another study describing a new classification of protein functional surfaces^46^ mentions SCOP as an alternative method of classifying protein structures.

### Distribution of all 2012 and 2013 SCOP‐citing articles in our categorization

Figure 3 shows the results of our categorization of all 546 SCOP‐citing articles from 2012 and 2013. Through an iterative process, we derived five categories that represent the diversity of ways that the SCOP classification is used in recent scientific literature. In our categorization, there is not one dominant mode of using the SCOP classification, showing that SCOP is an important resource for many different types of studies. The first four categories each contain roughly a quarter of the articles that used SCOP data. Although relatively few articles (7 of the 439) fell into category E (derive a database from SCOP), we have found that historically articles in this category have been among the most highly cited.

### SCOP use in experimental and computational research

Perhaps due to the ease of retrieving datasets for use in a number of applications, SCOP is a well known resource for those involved in computational structural biology research. However, researchers who primarily perform laboratory experiments may not be as well‐informed of its existence. In order to gauge whether SCOP remains a valuable tool for those working on laboratory experimental research, we categorized all articles that used the SCOP classification as either primarily computational, primarily experimental, or a containing both an experimental and computational component. For example, Suzuki and colleagues complemented results attained via experimental methods with computational methods in a study of how the membrane protein IP39 forms a lipid bilayer.^47^ They report on a low‐resolution (10 Å) structure of the membrane determined by electron crystallography. To study the arrangement of the protein in the membrane at a higher resolution, they used SCOP and another database to find high‐resolution structures of other 4‐membrane helix bundle proteins, and then used docking software to fit these into their experimentally determined EM map. The docking results were used as evidence that IP39 forms strands in a trimeric unit.

In summary, we found that the vast majority of articles using SCOP (80%) described purely computational research. However, there were still a considerable number of studies that used SCOP in the context of experimental research. 11% of the articles described purely experimental research and an additional 9% used both computational and experimental methods.

### SCOP hierarchy levels used

**Figure 5:**
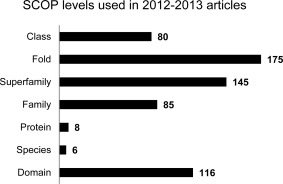
Count of studies published in 2012–2013 that made use of different SCOP levels. Studies that made use of multiple levels were counted once for each level used.

The SCOP hierarchy is organized into 7 levels: *structural class*, *fold*, *superfamily*, *family*, *protein*, *species domain*, and *domain*. We examined all articles that made use of information from one or more SCOP levels. We labeled each article by the levels that were used in the analysis, and then counted the total number of articles for each level. Often articles made use of more than one level, so these were counted more than once. Figure 5 shows the number of articles that made use of each level. The level used most often was fold (175 articles), followed by superfamily (145 articles), domain (116 articles), family (85 articles), and class (80 articles). We found the protein and species level classifications were used very infrequently, in 8 papers or fewer. SCOP also contains textual annotations of some finer details of evolutionary history that do not fit clearly into these levels (for example, annotations of similar folds in the all‐β class, or potential homology between the first seven superfamilies in the TIM (β/α)_8_ barrel fold). However, none of the articles stated that the authors made use of these details.

We expected that fold and superfamily would be used the most heavily because of their usefulness in benchmarking algorithms for fold recognition and remote homology detection. We were therefore surprised at how many studies made use of the SCOP family level classification. Since SCOP does not use a single standard for defining families, we discourage using this level for any purposes beyond a reference for naming. We also found studies that appeared to misinterpret classification of proteins as having the same SCOP fold to mean that these proteins must be homologous. However, it is possible for unrelated proteins to have the same fold: in some cases, different superfamilies in the same fold are thought to have evolved the fold independently. In other cases, different superfamilies in the same fold might share a common ancestor, but there is insufficient evidence to convince the SCOP curators to make an assertion that they are homologous. SCOP curators have historically been conservative in asserting annotations of homology^2^; such assertions of common evolutionary ancestry would be indicated by placing proteins within the same superfamily. Use of the SCOP family and fold classification levels might be especially detrimental for training and benchmarking homology detection algorithms if not done with subtlety.^48^

### Studies that use SCOP without citing a SCOP or ASTRAL article

In this manuscript, we have chosen to only survey articles that include a SCOP or ASTRAL article in the list of references. As a well‐known resource, SCOP is often used in research articles without citation, and we were interested in how many articles we might have omitted in our survey by excluding such articles. As discussed in the Methods section, we used PubMed Central to perform a second search for SCOP papers. In this database, we found 545 articles from 2012–2013 that contain the word “SCOP” in the list of references, and another 127 that mention SCOP in the body of the text, but do not contain the word “SCOP” in the list of references. We have not found any other resource named SCOP, so we believe the combined figure (672 articles) is a reasonable estimate of the number of papers that specifically reference the Structure Classification of Proteins database. We attempted a similar search for references to ASTRAL, but found that most of the results were not referencing the ASTRAL database, but rather astral microtubules formed during mitosis. Because Web of Science does not support searches on the body of articles, our methodology would have missed 127 of these 672 articles that explicitly mention SCOP in the body text but do not cite SCOP. We therefore estimate that there are at least an additional 23% of articles that reference SCOP that have been omitted from our survey because they do not cite a SCOP (or ASTRAL) article.

In addition to publications that explicitly cite or mention SCOP, a large number of scientists use SCOP to look up the classification of individual proteins (use category D) through a search on our website. In one month in 2014, we received hits to the SCOPe website from 13,362 IP addresses, of which 1,951 resolved to a name in DNS, excluding identifiable robots and search engines (Statistics for CATH are similar; personal communication from C. Orengo). Nearly half of our referrals in recent years have come from search engines such as Google, but 8% of our users were referred by websites such as the PDB, which links to SCOPe from individual structures, and by tools such as the 3d‐blast server.^49^ Each page in the SCOPe website corresponds to a single node in the classification, and provides hyperlinks to browse nodes at other levels of the hierarchy. Thus, a user might initially visit the page for a domain and then click through links to visit the domain's superfamily or fold and find neighboring superfamilies and folds. Approximately half the visitors to the SCOPe website with a resolvable IP address visited a single page, often referred by a search engine. Of those users that visited more than one page, approximately three quarters used the site to broadly explore the SCOPe classification, visiting nodes belonging to other superfamilies. This research use of SCOP may inform publications, for example to mention the name of a protein's fold and to gain clues to homologs, without yielding a citation in the manuscript.

### SCOP use in highly cited publications from the last 20 years

The main focus of this work is on recent uses of SCOP. However, in order to gain a longer‐term view of SCOP's impact, we also performed a literature survey of highly cited research published since SCOP's initial release in 1994. We retrieved the 25 most highly cited SCOP‐citing papers from the Web of Science. These articles were published between 1996 and 2009 and each has been cited >1,000 times. We examined the contents of each and found 22 of the 25 articles made heavy use of SCOP (the other three were citing SCOP for background). We categorized these 22 papers into our same five SCOP categories described above. Nine articles described using SCOP for training or benchmarking a method. These include highly used methods for sequence‐based homology detection and classification (3D‐PSSM,^50^ HHSearch,^51^ FUGUE,^52^ MUSCLE^53^), structure alignment (CE,^54^ Phyre,^55^ SSM,^56^ DaliLite^57^), and structure prediction (Jpred^58^). The second largest group comprised seven articles that augment a non‐SCOP dataset with the SCOP classification. These include publications for other domain classification databases such as Pfam,^24^, ^59^ which has its own extensive curation process for defining domain boundaries; the authors report that in some cases they consulted SCOP domain definition data to inform their decisions. In addition, the PDB.^4^, ^60^ the *Saccharomyces* Genome Database,^61^ and the UCSF Chimera software tool^62^ integrated SCOP data to provide structure classification information to their users. Two articles used SCOP primarily to investigate the classification of one protein or small set of proteins. One of these articles presented the crystal structure of α‐hemolysin, and used SCOP to determine if the fold of their new structure was novel.^63^ The other, which presented an elastic network model and demonstrated its performance on retinol‐binding protein, provided the SCOP class and superfamily classification for more context.^64^ The remaining two articles, describing SUPERFAMILY^40^ and HHpred,^65^ both used SCOP to derive databases of hidden Markov models for remote homology detection.

To summarize, we found that the majority of the 25 most highly cited SCOP‐citing articles describe highly used methods and databases. We found that SCOP played a key role in the development of each of these resources. Most of these resources are maintained continuously, or have had multiple versions made available to the public, which in turn depend on updated SCOP or SCOPe releases. Long‐term maintenance and regular updates of these SCOP‐citing resources have undoubtedly contributed to their widespread use. Although most studies that rely on these resources do not also cite SCOP, the accumulation of citations to these resources over time also reflects SCOP's ongoing impact.

### Studies that cite both SCOP and CATH

CATH is an important protein structure classification database that bears key similarities to SCOP, but uses a different hierarchy as well as different criteria for determining domain boundaries and classifying domains. CATH relies more heavily than SCOP on automation. In CATH the classification is largely algorithmic, although expert curation is used for the “architecture” level and to resolve ambiguity or disagreements in the algorithms' output, and all remote homologs are classified in CATH following considerable manual curation. By contrast, in SCOP the classification hierarchy is completely defined by expert curators, with automation used only to assign newly characterized protein structures to the hierarchy. One consequence is that SCOP often (although not always) tends to bring together more distantly related proteins in the “superfamily” level than CATH does at its “homology” level because SCOPs manual assessment draws on a broader and more idiosyncratic selection of features and literature for judgement of evolutionary relatedness.^66^ However, CATH is more consistent in its levels' meanings; and its use of advanced sequence‐based methods sometimes gives it a reach beyond SCOP. For a review of other important distinctions between the two classifications, we refer the reader to a recent study by Kolodny *et al*.^67^

We examined all 158 articles from 2012–2013 that cited both a CATH article^3^, ^17^, ^18^, ^19^, ^20^, ^21^, ^22^, ^23^ and a SCOP or ASTRAL article, to investigate how both databases were used and any justifications for using one database over the other. We found 110 papers in the set made use of structure classification information from at least one of the databases. We then found that over half of these (65 papers) made use of both databases, nine made use of only CATH, and 35 made use of only SCOP.

We first further investigated the set of 65 papers that made use of both databases. We categorized each by how the CATH classification was used, using the same five categories into which we classified SCOP‐citing articles. We found many papers used SCOP and CATH for the same purpose. 16 studies used both CATH and SCOP for performing computational studies of protein structure or evolution (Use Category A). For example, Jacob and colleagues' study of the folding times in two‐domain proteins used a computational method where domain folding time was estimated from the measurement of “absolute contact order” (ACO) defined in the article.^68^ The dataset consisted of two‐domain proteins in which both domains came from the same CATH or SCOP family. In cases where domain boundaries in CATH and SCOP differed, analysis was repeated with both definitions in order to ensure the robustness of their results: “The analysis was carried out using both CATH and SCOP in order to ensure that the ACO values that are calculated separately for each domain do not depend on the choice of domain boundaries that may differ in the two databases.” 13 studies used both the SCOP and CATH classifications for training or benchmarking algorithms (Use Category B); in 5 of these papers, this was done by using a consensus data set containing only data that was in agreement between the two databases, and in the rest, both databases were analyzed separately and results were presented for both. For example, a study by Genoni and colleagues that presented and evaluated an energy‐based method for identifying domains^69^ benchmarked the method on two datasets that consist of consensus domains from SCOP and CATH: the Benchmark_2 and Benchmark_3 datasets.^70^ The ThreaDom domain identification method developed by Xue and colleagues was mainly trained and evaluated on CATH domains, but also tested on SCOP domains to demonstrate “that the distinctive domain definitions of different databases have no impact on the training and testing procedures of domain predictions.^71^” 23 studies augmented datasets with both the SCOP and CATH classifications (Use Category C) and eight studies examined both the SCOP and CATH classifications of one protein or a small set of proteins (Use Category D). None of the studies that cite both SCOP and CATH presented a database derived from either source (Use Category E). The remaining 5 studies used CATH and SCOP for different purposes, often without specifying a reason. For example, the Pleiades method developed by Harder and colleagues used a training set of CATH domains to tune some model parameters, but then the SCOP classification was used for benchmarking the method, rather than CATH.^72^ Either database might have been used for either purpose.

We then examined articles that cited both databases but made use of only one classification. We found 35 that used SCOP rather than CATH and nine articles that used CATH rather than SCOP. In almost all cases, no reasons were given for choosing one over the other, and the CATH and SCOP databases could have been used interchangeably (with potentially different results). For example, although both SCOP and CATH are cited as examples of protein structure classifications in the study of fold preference in ancient and newer superfamilies by Edwards and colleagues that was discussed earlier in this manuscript, the authors chose to use SCOP's superfamily classification, although they could have used CATH.^26^ Many users that chose SCOP over CATH (14 of 35) were training or benchmarking an algorithm (Use Category B), and made use of the non‐redundant representative subsets of SCOP domains, which are provided in ASTRAL. For example, Yang and colleagues developed a new structure alignment method called SPalign, which they developed and tested on pairs of domains from the same SCOP fold, but with <20% sequence identity.^73^ Although the authors didn't state their reasons for choosing SCOP over CATH, a pre‐calculated SCOP domain subset in which all domain pairs have <20% sequence identity is available as part of ASTRAL, as are PDB‐style coordinate files for each domain in the subset. Because CATH has only recently made similar resources available,^74^ these authors, and others who train and benchmark algorithms on representative datasets, may have chosen SCOP for convenience, or for consistency because prior benchmarks had also used SCOP.

We found one study in which the authors used information that was available only in CATH and not in SCOP. In that study by Bukhari and Caetano‐Anollés, phylogenetic data were used to study the emergence of different CATH domain architectures.^75^ The focus of the study was on the CATH architecture level, which does not have an analogous level in SCOP. The study found ancient architectures such as the CATH *3‐layer (αβα) sandwich* (3.40) or the *orthogonal bundle* (1.10) are involved in basic cellular functions, but more recently evolved architectures such as *prism*, *propeller*, *2‐solenoid*, *super‐roll*, *clam*, *trefoil*, and *box* are not widely distributed. That study also benchmarked the phylogenetic analysis of CATH domains compared with SCOP domains, measuring the distribution of CATH architectures, topologies, and homologies, and SCOP folds, superfamilies, and families in Bacteria, Eukarya, and Archaea superkingdoms.

In summary, for studies that presented new methods, benchmarking against both resources was often used to demonstrate robustness. Although important distinctions exist, we found that for most of the 158 articles that cited both CATH and SCOP, either database could have been used interchangeably. Note however, this does not mean that the two resources are interchangeable: we expect that many studies that cite SCOP rather than CATH, and vice versa, may have had compelling reasons to do so, perhaps associated with additional information for a particular superfamily in SCOP, or the associated resources provided by ASTRAL. We also note that both databases are widely used in teaching, and both were used to guide the selection of structural genomics targets for the second phase of the Protein Structure Initiative.^76^, ^77^

### Future of protein structure classification databases

The main reason we set out to conduct a comprehensive literature survey was to gain a full and clear picture on the relevancy of protein structure classification in research today. We found 121 articles that present methods for which SCOP is used for training or benchmarking. Most of these studies used the last SCOP (version 1) release, 1.75, which classifies fewer than 40% of current PDB entries. Using an outdated dataset for developing these state of the art methods is concerning. Proteins that were structurally characterized after February 2009 have no coverage in SCOP (version 1), and only 17% of structures solved since January 2005 are classified in SCOP 1.75. Studies based on SCOP 1.75 will not have been tested on any superfamilies and folds that were structurally characterized since 2009. Currently, there are 2,023 Pfam families that contain at least one structurally characterized protein, but which do not have any members classified in SCOP 1.75. If classified in SCOP, approximately half of such structures would represent new folds and superfamilies.^78^ Over 50,000 additional structures not classified in SCOP 1.75 belong to Pfam families with members classified in SCOP, and the accuracy of computational methods might be improved if these newer structures were used in their development. We highlight each of the needs we found from our literature survey, and how we are addressing them in SCOPe.

#### Increased coverage of all known structures

We found the most pressing need across all use categories is for increased coverage of PDB structures. Studies that fell under the first two categories (“A: study protein structure or evolution computationally,” or “B: train and/or benchmark algorithms”) either use all domains from SCOP, use an ASTRAL representative set, or assemble data from particular SCOP folds, superfamilies, or families. Categorization of additional structures would improve the scientific validity of these research efforts because the newest structures for each SCOP category could be included in such studies. *Comprehensive* coverage (that is, classifying all PDB structures available on a given date, which was done in versions of SCOP through release 1.71) would be even more valuable, as researchers could draw broad conclusions about the repertoire of known structures without being biased by the interests of the curators. For studies in our third and fourth categories (“C: augment non‐SCOP datasets with SCOP classification” and “D: examine classification of one protein or small set of proteins”), increased coverage would speed the pace of research, mitigating the need to use profile‐based classification software like HHPred^65^ or SUPERFAMILY^40^ or structure alignment software such as DALI.^57^ To address the need for increased PDB coverage, we have deployed highly reliable automated classification methods in SCOPe (see Ref. 7 for details), and are also manually curating exemplar structures from the largest Pfam families not classified in SCOP. As a result, the fraction of the PDB classified in the SCOP hierarchy is 68% in SCOPe 2.05, compared with 38% of current protein structures that were classified in SCOP 1.75 (which classified 67% of structures available at the time). By contrast, SCOP2 is currently a prototype.^79^ This resource is a major redesign of SCOP that enables curators to annotate a richer set of evolutionary relationships between proteins, providing a more precise and accurate characterization of protein relationships. The prototype currently classifies 995 proteins, <1% of the PDB. These proteins were presumably selected to highlight the additional annotation capabilities of SCOP2.

#### Rapid synchronization with the PDB

One facet of increasing coverage is rapid, reliable classification of newer structures. This is primarily of use to users that are interested in learning the classification of a particular PDB structure of interest. To respond to the needs of such users, we produce periodic updates (approximately monthly), distinct from our stable SCOPe releases that supplement the latter releases with newer PDB structures.

#### Downloadable datasets in a stable format

We found that most of the studies in our survey made use of downloadable data, including parseable files that include all classification data, and ASTRAL sequences and representative subsets. To continue to support the needs of these users, SCOPe data file formats are backwards compatible with SCOP (version 1) downloadable data. In contrast, SCOP2^79^ does not provide backwards compatible datasets.

#### Web interface

We found that a considerable number of studies (22%) in our survey likely made use of the SCOP or SCOPe website to browse and search the SCOP hierarchy. Therefore, we have continued to improve the SCOPe web interface. Newer releases of ASTRAL, which are derived from the SCOPe classification, are tightly integrated into the SCOPe website. We have made the interface more user‐friendly, adding thumbnails of domains, and improved the ability to search for proteins of interest either using the site's built‐in search tool or an external search engine. Because many studies refer to both old and newer versions of SCOP and SCOPe, the SCOPe interface facilitates viewing changes to the classification, either to visualize the provenance of individual clades or the complete history of changes between subsequent stable releases of both SCOP and SCOPe.

## Supporting information

Supporting Information Figure 1.

Supporting Information Table 1.
