# Supplementary material for: The value of protein structure classification information—Surveying the scientific literature
Source: Proteins. 2015 Sep 19;83(11):2025–38. doi: 10.1002/prot.24915 (PMC4609302; doi:10.1002/prot.24915)
Supplement: Supplementary file 1 — Supporting Information Figure 1. [file PROT-83-2025-s001.html]

Zotero Report


- ## 2-Nitrobenzoate 2-Nitroreductase (NbaA) Switches Its Substrate Specificity from 2-Nitrobenzoic Acid to 2,4-Dinitrobenzoic Acid under Oxidizing Conditions

  |  |  |
  | --- | --- |
  | Type | Journal Article |
  | Author | Yong-Hak Kim |
  | Author | Woo-Seok Song |
  | Author | Hayoung Go |
  | Author | Chang-Jun Cha |
  | Author | Cheolju Lee |
  | Author | Myeong-Hee Yu |
  | Author | Peter C. K. Lau |
  | Author | Kangseok Lee |
  | Volume | 195 |
  | Issue | 2 |
  | Pages | 180-192 |
  | Publication | Journal of Bacteriology |
  | ISSN | 0021-9193 |
  | Date | JAN 2013 |
  | Extra | WOS:000316959600002 |
  | DOI | 10.1128/JB.02016-12 |
  | Abstract | 2-Nitrobenzoate 2-nitroreductase (NbaA) of Pseudomonas fluorescens strain KU-7 is a unique enzyme, transforming 2-nitrobenzoic acid (2-NBA) and 2,4-dinitrobenzoic acid (2,4-DNBA) to the 2-hydroxylamine compounds. Sequence comparison reveals that NbaA contains a conserved cysteine residue at position 141 and two variable regions at amino acids 65 to 74 and 193 to 216. The truncated mutant Delta 65-74 exhibited markedly reduced activity toward 2,4-DNBA, but its 2-NBA reduction activity was unaffected; however, both activities were abolished in the Delta 193-216 mutant, suggesting that these regions are necessary for the catalysis and specificity of NbaA. NbaA showed different lag times for the reduction of 2-NBA and 2,4-DNBA with NADPH, and the reduction of 2,4-DNBA, but not 2-NBA, failed in the presence of 1 mM dithiothreitol or under anaerobic conditions, indicating oxidative modification of the enzyme for 2,4-DNBA. The enzyme was irreversibly inhibited by 5,5'-dithio-bis-(2-nitrobenzoic acid) and ZnCl2, which bind to reactive thiol/thiolate groups, and was eventually inactivated during the formation of higher-order oligomers at high pH, high temperature, or in the presence of H2O2. SDS-PAGE and mass spectrometry revealed the formation of intermolecular disulfide bonds by involvement of the two cysteines at positions 141 and 194. Site-directed mutagenesis indicated that the cysteines at positions 39, 103, 141, and 194 played a role in changing the enzyme activity and specificity toward 2-NBA and 2,4-DNBA. This study suggests that oxidative modifications of NbaA are responsible for the differential specificity for the two substrates and further enzyme inactivation through the formation of disulfide bonds under oxidizing conditions. |
  | Date Added | 10/28/2013, 4:53:08 PM |
  | Modified | 10/28/2013, 4:53:08 PM |

  ### Notes:

  - Experimental study of a protein of interest, NbaA.

    How SCOP is used:

    Provide superfamily classification

    SCOP reference:

    NbaA (GenBank accession number BAF56676.1) is a homodimeric NADH:flavin mononucleotide (FMN) oxidoreductase-like fold protein (3). It is similar to a putative flavin-containing pro- tein (78% sequence identity; ABE46991.1) located on the Polaro- monas sp. strain JS666 plasmid 1 (GI:91790731), and it includes a flavin reductase-like domain (Pfam accession number PF01613 in the Pfam database [http://www.sanger.ac.uk/Software/Pfam/]) (6). Structurally, it is related to the NADH:FMN oxidoreductase- like structural family (SCOP accession number b.45.1.2 or 50482; http://scop.berkeley.edu/) (7).

  ### Attachments

  - J. Bacteriol.-2013-Kim-180-92.pdf
- ## 5-Methylation of Cytosine in CG:CG Base-Pair Steps: A Physicochemical Mechanism for the Epigenetic Control of DNA Nanomechanics

  |  |  |
  | --- | --- |
  | Type | Journal Article |
  | Author | Tahir I. Yusufaly |
  | Author | Yun Li |
  | Author | Wilma K. Olson |
  | Volume | 117 |
  | Issue | 51 |
  | Pages | 16436-16442 |
  | Publication | Journal of Physical Chemistry B |
  | ISSN | 1520-6106 |
  | Date | DEC 26 2013 |
  | Extra | WOS:000329331800008 |
  | DOI | 10.1021/jp409887t |
  | Abstract | van der Waals density functional theory is integrated with analysis of a non-redundant set of protein-DNA crystal structures from the Nucleic Acid Database to study the stacking energetics of CG:CG base-pair steps, specifically the role of cytosine 5-methylation. Principal component analysis of the steps reveals the dominant collective motions to correspond. to a tensile "opening" mode and two shear "sliding" and "tearing" modes in the orthogonal plane. The stacking interactions of the methyl groups globally inhibit CG:CG step overtwisting while simultaneously softening the modes locally via potential energy modulations that create metastable states. Additionally, the indirect effects of the methyl groups on possible base-pair steps neighboring CG:CG are observed to be of comparable importance to their direct effects on CG:CG. The results have implications for the epigenetic control of DNA mechanics. |
  | Date Added | 2/12/2014, 1:36:22 PM |
  | Modified | 2/12/2014, 1:36:22 PM |

  ### Notes:

  - Computational study of biophysical properties of proteins in protein-DNA structures, specifically the role of cytosine 5-methylation.

    How SCOP is used:

    Curated a non-redundant data set of 239 protein-DNA complexes, using SCOP for structural diversity.

    SCOP reference:

    The structures were filtered to exclude over-represented complexes in order to obtain a balanced sample of spatial and functional forms. The selection and classification of structures was based on sequential and structural alignment, as well as available protein classification databases, including the SCOP scheme.

  ### Attachments

  - jp409887t.pdf
- ## A 3-Dimensional Trimeric beta-Barrel Model for Chlamydia MOMP Contains Conserved and Novel Elements of Gram-Negative Bacterial Porins

  |  |  |
  | --- | --- |
  | Type | Journal Article |
  | Author | Victoria A. Feher |
  | Author | Arlo Randall |
  | Author | Pierre Baldi |
  | Author | Robin M. Bush |
  | Author | Luis M. de la Maza |
  | Author | Rommie E. Amaro |
  | Volume | 8 |
  | Issue | 7 |
  | Publication | PLoS one |
  | ISSN | 1932-6203 |
  | Date | JUL 25 2013 |
  | DOI | 10.1371/journal.pone.0068934 |
  | Language | English |
  | Abstract | Chlamydia trachomatis is the most prevalent cause of bacterial sexually transmitted diseases and the leading cause of preventable blindness worldwide. Global control of Chlamydia will best be achieved with a vaccine, a primary target for which is the major outer membrane protein, MOMP, which comprises similar to 60% of the outer membrane protein mass of this bacterium. In the absence of experimental structural information on MOMP, three previously published topology models presumed a16-stranded barrel architecture. Here, we use the latest beta-barrel prediction algorithms, previous 2D topology modeling results, and comparative modeling methodology to build a 3D model based on the 16-stranded, trimeric assumption. We find that while a 3D MOMP model captures many structural hallmarks of a trimeric 16-stranded beta-barrel porin, and is consistent with most of the experimental evidence for MOMP, MOMP residues 320-334 cannot be modeled as beta-strands that span the entire membrane, as is consistently observed in published 16-stranded beta-barrel crystal structures. Given the ambiguous results for beta-strand delineation found in this study, recent publications of membrane beta-barrel structures breaking with the canonical rule for an even number of beta-strands, findings of beta-barrels with strand-exchanged oligomeric conformations, and alternate folds dependent upon the lifecycle of the bacterium, we suggest that although the MOMP porin structure incorporates canonical 16-stranded conformations, it may have novel oligomeric or dynamic structural changes accounting for the discrepancies observed. |
  | Date Added | 10/11/2013, 10:29:15 AM |
  | Modified | 12/2/2013, 4:16:27 PM |

  ### Notes:

  - Build and study a structural model of the major outer membrane protein (MOMP) that comprises 60% of the outer membrane of the Chlamydia virus.

    How SCOP is used:

    Provide background on the structural classification of the MOMP protein.

    SCOP reference:

    MOMP, coded by the ompA gene, is considered a member of the general porin class of proteins (http://scop.mrc- lmb.cam.ac.uk/scop) [11],

  ### Attachments

  - journal.pone.0068934.pdf
- ## Aberrant 3 ` oligoadenylation of spliceosomal U6 small nuclear RNA in poikiloderma with neutropenia

  |  |  |
  | --- | --- |
  | Type | Journal Article |
  | Author | Christine Hilcenko |
  | Author | Paul J. Simpson |
  | Author | Andrew J. Finch |
  | Author | Frank R. Bowler |
  | Author | Mark J. Churcher |
  | Author | Li Jin |
  | Author | Len C. Packman |
  | Author | Adam Shlien |
  | Author | Peter Campbell |
  | Author | Michael Kirwan |
  | Author | Inderjeet Dokal |
  | Author | Alan J. Warren |
  | Volume | 121 |
  | Issue | 6 |
  | Pages | 1028-1038 |
  | Publication | BLOOD |
  | ISSN | 0006-4971 |
  | Date | FEB 7 2013 |
  | DOI | 10.1182/blood-2012-10-461491 |
  | Language | English |
  | Abstract | The recessive disorder poikiloderma with neutropenia (PN) is caused by mutations in the C16orf57 gene that encodes the highly conserved USB1 protein. Here, we present the 1.1 angstrom resolution crystal structure of human USB1, defining it as a member of the LigT-like superfamily of 2H phosphoesterases. We show that human USB1 is a distributive 3'-5' exoribonuclease that posttranscriptionally removes uridine and adenosine nucleosides from the 3' end of spliceosomal U6 small nuclear RNA (snRNA), directly catalyzing terminal 2', 3' cyclic phosphate formation. USB1 measures the appropriate length of the U6 oligo(U) tail by reading the position of a key adenine nucleotide (A102) and pausing 5 uridine residues downstream. We show that the 3' ends of U6 snRNA in PN patient lymphoblasts are elongated and unexpectedly carry nontemplated 3' oligo(A) tails that are characteristic of nuclear RNA surveillancetargets. Thus, our study reveals a novel quality control pathway in which posttranscriptional 3'-end processing by USB1 protects U6 snRNA from targeting and destruction by the nuclear exosome. Our data implicate aberrant oligoadenylation of U6 snRNA in the pathogenesis of the leukemia predisposition disorder PN. (Blood. 2013;121(6):1028-1038) |
  | Date Added | 10/11/2013, 10:29:15 AM |
  | Modified | 10/11/2013, 10:29:15 AM |

  ### Notes:

  - Present crystal structure of human USB1, placing it in the LigT-like superfamily

    How SCOP is used:

    Classify their newly crystallized structure of human USB1 into the LigT-like superfamily in SCOP.

    Mention that the H-x-S motif is highly conserved within the superfamily, despite low sequence identity overall.

    SCOP reference:

    The USB1 protein belongs to the LigT-like superfamily, defined in the SCOP database23 as a betabarrel domain with a duplicated beta/alpha/beta/alpha/beta topology (Figure 1F). The invariance of the H-x-S motif within the USB1 protein family, despite low overall amino acid sequence identity (Figure 2A), supports a critical role in catalysis.

  ### Attachments

  - Blood-2013-Hilcenko-1028-38.pdf
- ## Ab Initio structure prediction for Escherichia coli: towards genome-wide protein structure modeling and fold assignment

  |  |  |
  | --- | --- |
  | Type | Journal Article |
  | Author | Dong Xu |
  | Author | Yang Zhang |
  | URL | http://www.nature.com/srep/2013/130530/srep01895/full/srep01895.html |
  | Volume | 3 |
  | Publication | Scientific reports |
  | Date | 2013 |
  | Accessed | 9/23/2013, 10:23:40 AM |
  | Library Catalog | Google Scholar |
  | Short Title | Ab Initio structure prediction for Escherichia coli |
  | Date Added | 10/11/2013, 10:29:15 AM |
  | Modified | 10/11/2013, 10:29:15 AM |

  ### Notes:

  - Present a new pipeline for structure prediction and fold classification for a whole genome, and applied to e. coli genome.

    How SCOP is used:

    Benchmarked fold prediction on a dataset of e. coli proteins.  Validated on the SCOP fold classification, but extended to superfamily and family as well.

    SCOP reference:

    In abstract:

    For 495 unknown hard sequences, 72 are predicted to have a correct fold (TM-score . 0.5) and 321 have a substantial portion of structure correctly modeled (TM-score . 0.35). 317 sequences can be reliably assigned to a SCOP fold family based on structural analogy to existing proteins in PDB.

    ...

    SCOP fold family assignments of E. coli proteins. As an application of the genome-wide structure prediction, we assign the E. coli proteins with standard fold families by matching the ab intio models with known structures in the SCOP family database20. We first compare the top QUARK models with the proteins in the PDB using the structural alignment algorithm TM-align32. If the QUARK model includes multiple domains, DomainParser33 will be used to split the chain to domains. The PDB structures are then listed in descending order based on their TM-score value to the QUARK models. The nearest neighbor classification method34 is then used to classify the predicted models based on the TM-score list. In case that the top PDB structure has no SCOP code in the SCOP database, the code of the protein that is closest to the QUARK model is used. Here, we note that the TM-score is calculated as the average of the two TM-scores which are normalized by the target length and the analogy length separately. We found that the TM-score normalized by the target length may pick up some big proteins with artificial alignments while the use of average TM-score from both target and analog proteins help recognize the closest analogs with the similar size.

  ### Attachments

  - srep01895.pdf
- ## A bioinformatics view of zinc enzymes

  |  |  |
  | --- | --- |
  | Type | Journal Article |
  | Author | Claudia Andreini |
  | Author | Ivano Bertini |
  | URL | http://www.sciencedirect.com/science/article/pii/S0162013411003679 |
  | Volume | 111 |
  | Pages | 150–156 |
  | Publication | Journal of Inorganic Biochemistry |
  | Date | 2012 |
  | Accessed | 9/20/2013, 1:18:50 PM |
  | Library Catalog | Google Scholar |
  | Date Added | 10/11/2013, 10:29:15 AM |
  | Modified | 3/7/2014, 12:15:32 PM |

  ### Tags:

  - bioinformatics
  - Databases
  - Interesting
  - SCOP coverage insufficient
  - Zinc
  - Zinc enzymes
  - Zinc proteins

  ### Notes:

  - The paper aims to gain insight on zinc enzymes function and categorization based on bioinformatics and literature review. This is done by collecting data from different protein databases (SCOP, CATH, Pfam, etc)

    How SCOP/CATH is used:

    Use SCOP and CATH to "group" evolutionarily-related zinc sites and assign functions to the group using literature searches and EC-classification.

    Collect a data set of zinc-binding proteins from the PDB, and classify their zinc sites by SCOP and CATH superfamily.  Then the groups are annotated with functions via the literature, and non-physiological sites (those where zinc has been substituted for the native metal ion) are labeled.

    SCOP Reference:

    Zinc sites were grouped based on the CATH (http://www.cathdb.info) [19] and SCOP (http:// scop.mrc-lmb.cam.ac.uk/scop) [20] classifications of the protein do- mains containing them. In both the CATH and SCOP databases, protein domains with known structures are hierarchically classified into groups at four different levels of similarity. The superfamily level is common to both the CATH (where it corresponds to the highest level of similarity) and the SCOP (where it corresponds to the second highest level of similarity) classification schemes, and groups together protein do- mains for which there is good evidence of common ancestry and functional similarity. Each zinc site was assigned to both a CATH and a SCOP superfamily, and sites assigned either to the same CATH or to the same SCOP superfamily were grouped together. The sites of proteins that have not yet been included in the CATH or in the SCOP database were also assigned to an existing CATH and/or SCOP super- family, or left unassigned, using a procedure described in Ref. [21].

  ### Attachments

  - 1-s2.0-S0162013411003679-main.pdf
- ## Abstracting knowledge from the protein data bank

  |  |  |
  | --- | --- |
  | Type | Journal Article |
  | Author | Nicholas Furnham |
  | Author | Roman A. Laskowski |
  | Author | Janet M. Thornton |
  | URL | http://onlinelibrary.wiley.com/doi/10.1002/bip.22107/full |
  | Volume | 99 |
  | Issue | 3 |
  | Pages | 183–188 |
  | Publication | Biopolymers |
  | Date | 2013 |
  | Accessed | 9/20/2013, 1:16:24 PM |
  | Library Catalog | Google Scholar |
  | Date Added | 2/13/2014, 4:13:17 PM |
  | Modified | 3/7/2014, 12:09:46 PM |

  ### Notes:

  - Review of protein structural analysis over the past 40 years.

    How SCOP/CATH is used:

    Background on protein structure classification.

    SCOP reference:

    Two fold classification systems arose, CATH11 and SCOP,12 both of which are in use today and both of which can reveal extremely distant relationships between proteins that are not detectable by sequence comparison alone.13

  ### Attachments

  - 22107\_ftp.pdf
- ## Accurate prediction of protein structural class

  |  |  |
  | --- | --- |
  | Type | Journal Article |
  | Author | Xia-Yu Xia |
  | Author | Meng Ge |
  | Author | Zhi-Xin Wang |
  | Author | Xian-Ming Pan |
  | Volume | 7 |
  | Issue | 6 |
  | Pages | e37653 |
  | Publication | PloS one |
  | ISSN | 1932-6203 |
  | Date | 2012 |
  | Extra | PMID: 22723837 |
  | Journal Abbr | PLoS ONE |
  | DOI | 10.1371/journal.pone.0037653 |
  | Library Catalog | NCBI PubMed |
  | Language | eng |
  | Abstract | Because of the increasing gap between the data from sequencing and structural genomics, the accurate prediction of the structural class of a protein domain solely from the primary sequence has remained a challenging problem in structural biology. Traditional sequence-based predictors generally select several sequence features and then feed them directly into a classification program to identify the structural class. The current best sequence-based predictor achieved an overall accuracy of 74.1% when tested on a widely used, non-homologous benchmark dataset 25PDB. In the present work, we built a multiple linear regression (MLR) model to convert the 440-dimensional (440D) sequence feature vector extracted from the Position Specific Scoring Matrix (PSSM) of a protein domain to a 4-dimensinal (4D) structural feature vector, which could then be used to predict the four major structural classes. We performed 10-fold cross-validation and jackknife tests of the method on a large non-homologous dataset containing 8,244 domains distributed among the four major classes. The performance of our approach outperformed all of the existing sequence-based methods and had an overall accuracy of 83.1%, which is even higher than the results of those predicted secondary structure-based methods. |
  | Date Added | 10/11/2013, 10:29:15 AM |
  | Modified | 3/7/2014, 12:10:30 PM |

  ### Tags:

  - Computational Biology
  - Protein Conformation
  - Proteins
  - Protein Structure, Tertiary

  ### Notes:

  - Present a method for SCOP class prediction.

    How SCOP is used:

    Use ASTRAL 40% data set to train and validate their method for SCOP structural class prediction.

    How CATH is used:

    Mention that CATH does not differentiate between a+b and a/b classes, just the ab class.

    SCOP reference:

    In the present work, we developed an approach that predicts domains into the four major SCOP classes (all-a, all-b, a/b and a+b) by converting each domain into a discriminating 4-dimensional (4D) structural feature vector solely based on the 440- dimensional (440D) sequence feature vector extracted from the PSSM. At first, each domain in the training set was assigned to an approximate 4D structural feature vector based on the composi- tion of its secondary structural elements and to another 440D sequence feature vector based on its PSSM profile. Assuming that the domains’ 4D structural feature vectors were linear combina- tions of their 440D sequence feature vectors, the regression coefficient matrix was determined by using iterative least-squared multiple linear regression (MLR) method [35] based on the training data. Using the estimated coefficient matrix, the 4D structural vectors of the domains in the testing set were calculated according to their 440D sequence feature vectors, and then utilized to predict the four major classes. We employed 10-fold cross-validation and jackknife tests [36] to train and evaluate the model on a large, non-homologous dataset containing 8,244 domains selected from the ASTRAL SCOP40 v. 1.73 dataset [37], and an overall accuracy of 83.1% (jackknife test) was achieved. A blind test was also conducted on another dataset comprising 1,185 domains that are not included in SCOP v. 1.73 but are included in SCOP v. 1.75 to evaluate the unbiased performance of the method; an overall accuracy of 80.1% was achieved. The performance of our approach outperformed all of the existing sequence-based methods and was even better than those predicted secondary structure-based methods.

     CATH reference:

    The current version of the SCOP database, v. 1.75, includes eleven structural classes, with the four major classes (all-a, all-b, a/b and a+b) covering approximately 90% of the entries. Slightly different from SCOP, CATH does not differentiate between a/b and a+b domains at the class level (these are treated together as mixed ab) but further classifies these domains into different topologies.

  ### Attachments

  - journal.pone.0037653.pdf
- ## Accurate prediction of protein structural classes using functional domains and predicted secondary structure sequences

  |  |  |
  | --- | --- |
  | Type | Journal Article |
  | Author | Amin Ahmadi Adl |
  | Author | Abbas Nowzari-Dalini |
  | Author | Bin Xue |
  | Author | Vladimir N. Uversky |
  | Author | Xiaoning Qian |
  | URL | http://www.tandfonline.com/doi/abs/10.1080/07391102.2011.672626 |
  | Volume | 29 |
  | Issue | 6 |
  | Pages | 1127–1137 |
  | Publication | Journal of Biomolecular Structure and Dynamics |
  | Date | 2012 |
  | Accessed | 9/23/2013, 10:14:18 AM |
  | Library Catalog | Google Scholar |
  | Date Added | 10/11/2013, 10:29:15 AM |
  | Modified | 10/11/2013, 10:29:15 AM |

  ### Tags:

  - disordered proteins
  - feature selection
  - functional domains
  - predicted secondary structure sequences
  - protein secondary structure propensity
  - Protein structural class prediction
  - support vector machines (SVMs)

  ### Notes:

  - Protein structural class prediction method.  Present method for "protein structural class prediction using combinations of the novel features including secondary structure propensities as well as functional domain (FD) features extracted from the InterPro signature database."

    How SCOP is used:

    Retrieve SCOP structural class for domains in 5 data sets and validate predictions of SCOP class.

    SCOP reference:

    Introduction

    Functionalities of proteins have been commonly believed to be determined by their unique 3D (dimensional) struc- tures (Chou, 2006), which are determined by the exact spatial position of each atom. However, for simplicity, pro- teins typically are first classified into several structural folding classes, based on the type, amount, and spatial arrangement of their amino acid (AA) residues into poten- tial secondary structure elements. For example, in struc- tural classification of proteins (SCOP) (Murzin, Brenner, Hubbard, & Chot, 1995), proteins are annotated by struc- tural class labels as the first step for their 3D structure annotations, among which there are four major structural classes denoted as a, b, ab, and a þ b. These four major classes cover 82, 89, and 84% of protein folds, families, and super-families in SCOP. Proteins in the class α have α- helices as the dominant secondary structure. Similarly, sec- ondary structures of proteins in the class β are mostly dominated by β-strands. In the αβ and α + β classes, there are significant amounts of both α-helices and β-strands. In αβ, β-strands create parallel β-sheets; while in α + β class, β-strands create anti-parallel β-sheets (Murzin et al., 1995).

    ...

    Data-sets

    The proposed method is tested on three low-similarity protein data-sets that are widely used in the literature (Kurgan et al., 2008; Mizianty & Kurgan, 2009; Yang et al., 2010). The first two data-sets, referred to as 25PDB and 1189, respectively, are downloaded from RCSB Protein Data Bank (www.pdb.org) Berman, 2000 with the PDB IDs listed in the paper (Kurgan & Homaeian, 2006). The data-set 25PDB contains 1673 proteins with the pairwise sequence identity being about 25%, whereas the data-set 1189 contains 1092 proteins with 40% sequence identity. The third protein data-set, referred to as 640, was first studied in Chen et al. (2008). It contains 640 proteins with 25% sequence iden- tity. There are 76 protein sequences that overlap among three data-sets. The numbers of common sequences between each pair of data-sets are 357 (for 640 and 1189), 78 (for 640 and 25PDB), and 205 (for 1189 and 25PDB), respectively. The AA sequences in these data- sets represent protein domains rather than the complete protein AA sequences. Protein structural classification labels are retrieved from the database SCOP (Murzin et al., 1995).

    ...

    To evaluate the prediction performance of our method, we select sequences from these two data-sets that have struc- tural class annotations in SCOP with one of the four major classes, which lead to the final 415 sequences in fully structured data-set and 332 sequences in par- tially structured data-set. Note that there is no overlap between these two new data-sets and the previous three data-sets.

  ### Attachments

  - 07391102%2E2011%2E672626.pdf
- ## Accurate prediction of protein structural class using auto covariance transformation of PSI-BLAST profiles

  |  |  |
  | --- | --- |
  | Type | Journal Article |
  | Author | Taigang Liu |
  | Author | Xingbo Geng |
  | Author | Xiaoqi Zheng |
  | Author | Rensuo Li |
  | Author | Jun Wang |
  | URL | http://link.springer.com/article/10.1007/s00726-011-0964-5 |
  | Volume | 42 |
  | Issue | 6 |
  | Pages | 2243–2249 |
  | Publication | Amino acids |
  | Date | 2012 |
  | Accessed | 9/23/2013, 10:14:18 AM |
  | Library Catalog | Google Scholar |
  | Date Added | 10/11/2013, 10:29:15 AM |
  | Modified | 10/11/2013, 10:29:15 AM |

  ### Tags:

  - Auto covariance transformation
  - protein structural class
  - PSI-BLAST profile
  - support vector machine

  ### Notes:

  - Present method for protein structural class prediction based solely on sequence.

    How SCOP is used:

    Train and benchmark method for SCOP class prediction on 3rd party data sets that were derived from SCOP.

    SCOP reference:

    Introduction

    Knowledge of structural class information of a given pro- tein plays an important role in the prediction of secondary structure, tertiary structure and function analysis from the amino acid sequence (Anand et al. 2008). Based on the visual inspection of polypeptide chain topologies in a dataset of 31 globular proteins, Levitt and Chothia (1976) first introduced the concept of structural class and catego- rized the protein domains of known structure into four structural classes: all-a, all-b, a/b and a ? b. Nowadays, the most frequently used classification of protein structural classes can be found in the structural classification of proteins (SCOP) database (Murzin et al. 1995), which further divides proteins into 11 structural classes. But currently, the four major structural classes, which cover almost 90% of all SCOP entries, are still commonly adopted by many researchers.

  ### Attachments

  - s00726-011-0964-5.pdf
- ## A Combination of Feature Extraction Methods with an Ensemble of Different Classifiers for Protein Structural Class Prediction Problem.

  |  |  |
  | --- | --- |
  | Type | Journal Article |
  | Author | Abdollah Dehzangi |
  | Author | Kuldip Paliwal |
  | Author | Alok Sharma |
  | Author | Omid Dehzangi |
  | Author | Abdul Sattar |
  | URL | http://europepmc.org/abstract/MED/23713003 |
  | Publication | IEEE/ACM transactions on computational biology and bioinformatics/IEEE, ACM |
  | Date | 2013 |
  | Accessed | 9/23/2013, 10:16:36 AM |
  | Library Catalog | Google Scholar |
  | Date Added | 2/20/2014, 12:24:01 PM |
  | Modified | 2/20/2014, 12:24:01 PM |

  ### Attachments

  - Snapshot
- ## A comparative assessment and analysis of 20 representative sequence alignment methods for protein structure prediction

  |  |  |
  | --- | --- |
  | Type | Journal Article |
  | Author | Renxiang Yan |
  | Author | Dong Xu |
  | Author | Jianyi Yang |
  | Author | Sara Walker |
  | Author | Yang Zhang |
  | URL | http://www.nature.com/srep/2013/130910/srep02619/full/srep02619.html |
  | Volume | 3 |
  | Publication | Scientific reports |
  | Date | 2013 |
  | Accessed | 9/23/2013, 10:15:34 AM |
  | Library Catalog | Google Scholar |
  | Date Added | 10/11/2013, 10:29:15 AM |
  | Modified | 3/7/2014, 12:08:34 PM |

  ### Notes:

  - Assessment of 20 sequence alignment methods for protein structure prediction.

    Collect 20 sequence alignment algorithms, 10 published and 10 newly developed, which cover all representative sequence- and profile-based alignment approaches. These algorithms are benchmarked on 538 non-redundant proteins for protein fold-recognition on a uniform template library.

    How SCOP is used:

    Not using SCOP.

    Mention that most methods were benchmarked using the SCOP database, implying examples belonged to "the easy homology category".  Instead in their study, they randomly select ~500 proteins from the PDB with at most 30% sequence identity and then divide into "easy", "medium", and "hard" groups.

    SCOP reference:

    Despite the valuable insights revealed, most of the benchmark studies focused on a limited set of traditional sequence alignment algorithms and were performed nearly a decade ago. Many recent developments, e.g. structural feature integrations and HMM-HMM alignments which are important for protein structure prediction, are yet to be assessed. Meanwhile, the testing datasets used in these studies were mostly collected from the SCOP library and largely belong to the easy homology category (which represents a similar problem in the CASP experiments mentioned above), while the per- formance of the methods on detecting hard distant-homology tem- plates, which are more challenging to the field, needs to be appropriately examined.

  ### Attachments

  - [PDF] from umich.edu
  - srep02619.pdf
- ## A comparative study on filtering protein secondary structure prediction

  |  |  |
  | --- | --- |
  | Type | Journal Article |
  | Author | Petros Kountouris |
  | Author | Michalis Agathocleous |
  | Author | Vasilis J. Promponas |
  | Author | Georgia Christodoulou |
  | Author | Simos Hadjicostas |
  | Author | Vassilis Vassiliades |
  | Author | Chris Christodoulou |
  | URL | http://dl.acm.org/citation.cfm?id=2189814 |
  | Volume | 9 |
  | Issue | 3 |
  | Pages | 731–739 |
  | Publication | IEEE/ACM Transactions on Computational Biology and Bioinformatics (TCBB) |
  | Date | 2012 |
  | Accessed | 9/23/2013, 10:17:26 AM |
  | Library Catalog | Google Scholar |
  | Date Added | 10/11/2013, 10:29:15 AM |
  | Modified | 10/11/2013, 10:29:15 AM |

  ### Tags:

  - Interesting

  ### Notes:

  - Evaluate different methods that filter secondary structure predictions, removing conformations that are physiochemically unlikely.

    How SCOP is used:

    Use for training and benchmarking.  Use SCOP class-level classification to quickly evaluate whether the data subsets used for N-fold cross-validation have similar distributions of the four main SCOP classes.  Use the CB513 dataset which contains 513 non-homologous chains.

    SCOP references:

    The ultimate goal of a classification algorithm is not to achieve high training accuracy, but to classify suc- cessfully previously unseen examples. Hence, we use n-fold cross-validation to estimate the generalisation error. More specifically, we divide the training set into n subsets and, sequentially, we use n − 1 for training and the remaining one for testing. This procedure is repeated n times, until all subsets are used once for testing. In this paper, we report the results from 10-fold cross-validation on the CB513 dataset and 5- fold cross-validation on the PDB-Select25 dataset. For both datasets, the folds have similar representation of helical, extended and loop residues. Moreover, in the case of CB513, we ensure similar distributions of small/large protein chains as well as of the four main SCOP classes (all-α, all-β, α + β and α/β) [37]. The subsets are available on request.

  ### Attachments

  - [PDF] from researchgate.net
- ## A consensus view of fold space: Combining SCOP, CATH, and the Dali Domain Dictionary

  |  |  |
  | --- | --- |
  | Type | Journal Article |
  | Author | Ryan Day |
  | Author | David A.C. Beck |
  | Author | Roger S. Armen |
  | Author | Valerie Daggett |
  | URL | http://www.ncbi.nlm.nih.gov/pmc/articles/PMC2366924/ |
  | Volume | 12 |
  | Issue | 10 |
  | Pages | 2150-2160 |
  | Publication | Protein Science : A Publication of the Protein Society |
  | ISSN | 0961-8368 |
  | Date | 2003-10 |
  | Extra | PMID: 14500873 PMCID: PMC2366924 |
  | Journal Abbr | Protein Sci |
  | Accessed | 10/29/2014, 11:59:32 AM |
  | Library Catalog | PubMed Central |
  | Abstract | We have determined consensus protein-fold classifications on the basis of three classification methods, SCOP, CATH, and Dali. These classifications make use of different methods of defining and categorizing protein folds that lead to different views of protein-fold space. Pairwise comparisons of domains on the basis of their fold classifications show that much of the disagreement between the classification systems is due to differing domain definitions rather than assigning the same domain to different folds. However, there are significant differences in the fold assignments between the three systems. These remaining differences can be explained primarily in terms of the breadth of the fold classifications. Many structures may be defined as having one fold in one system, whereas far fewer are defined as having the analogous fold in another system. By comparing these folds for a nonredundant set of proteins, the consensus method breaks up broad fold classifications and combines restrictive fold classifications into metafolds, creating, in effect, an averaged view of fold space. This averaged view requires that the structural similarities between proteins having the same metafold be recognized by multiple classification systems. Thus, the consensus map is useful for researchers looking for fold similarities that are relatively independent of the method used to compare proteins. The 30 most populated metafolds, representing the folds of about half of a nonredundant subset of the PDB, are presented here. The full list of metafolds is presented on the Web. |
  | Short Title | A consensus view of fold space |
  | Date Added | 10/29/2014, 11:59:32 AM |
  | Modified | 10/29/2014, 11:59:32 AM |

  ### Attachments

  - PubMed Central Full Text PDF
  - PubMed Central Link
- ## A conserved START domain coenzyme Q-binding polypeptide is required for efficient Q biosynthesis, respiratory electron transport, and antioxidant function in Saccharomyces cerevisiae

  |  |  |
  | --- | --- |
  | Type | Journal Article |
  | Author | Christopher M. Allan |
  | Author | Shauna Hill |
  | Author | Susan Morvaridi |
  | Author | Ryoichi Saiki |
  | Author | Jarrett S. Johnson |
  | Author | Wei-Siang Liau |
  | Author | Kathleen Hirano |
  | Author | Tadashi Kawashima |
  | Author | Ziming Ji |
  | Author | Joseph A. Loo |
  | Author | Jennifer N. Shepherd |
  | Author | Catherine F. Clarke |
  | Volume | 1831 |
  | Issue | 4 |
  | Pages | 776-791 |
  | Publication | Biochimica Et Biophysica Acta-Molecular and Cell Biology of Lipids |
  | Date | APR 2013 |
  | Extra | WOS:000316438200012 |
  | DOI | 10.1016/j.bbalip.2012.12.007 |
  | Library Catalog | ISI Web of Knowledge |
  | Abstract | Coenzyme Q(n) (ubiquinone or Q(n)) is a redox active lipid composed of a fully substituted benzoquinone ring and a polyisoprenoid tail of n isoprene units. Saccharomyces cerevisiae coq1-coq9 mutants have defects in Q biosynthesis, lack Q(6), are respiratory defective, and sensitive to stress imposed by polyunsaturated fatty acids. The hallmark phenotype of the Q-less yeast coq mutants is that respiration in isolated mitochondria can be rescued by the addition of Q(2), a soluble Q analog. Yeast coq10 mutants share each of these phenotypes, with the surprising exception that they continue to produce Q(6). Structure determination of the Caulobacter crescentus Coq10 homolog (CC1736) revealed a steroidogenic acute regulatory protein-related lipid transfer (START) domain, a hydrophobic tunnel known to bind specific lipids in other START domain family members. Here we show that purified CC1736 binds Q(2), Q(3), Q(10), or demethoxy-Q(3) in an equimolar ratio, but fails to bind 3-farnesyl-4-hydroxybenzoic acid, a farnesylated analog of an early Q-intermediate. Over-expression of C crescentus CC1736 or COQ8 restores respiratory electron transport and antioxidant function of Q(6) in the yeast coq10 null mutant. Studies with stable isotope ring precursors of Q reveal that early Q-biosynthetic intermediates accumulate in the coq10 mutant and de novo Q-biosynthesis is less efficient than in the wild-type yeast or rescued coq10 mutant. The results suggest that the Coq10 polypeptide:Q (protein:ligand) complex may serve essential functions in facilitating de novo Q biosynthesis and in delivering newly synthesized Q to one or more complexes of the respiratory electron transport chain. (C) 2012 Elsevier B.V. All rights reserved. |
  | Date Added | 10/8/2014, 12:49:22 PM |
  | Modified | 10/8/2014, 1:32:30 PM |

  ### Tags:

  - Lipid autoxidation
  - Lipid binding
  - Respiratory electron transport
  - Steroidogenic acute regulatory protein
  - ubiquinone
  - Yeast mitochondria

  ### Notes:

  - How SCOP is used:

    Investigate fold of the START domain.

    SCOP reference:

    The START domain struc- ture is classified as a helix-grip type, consisting of a seven-stranded anti-parallel β-sheet with a C-terminal α-helix [17].

  ### Attachments

  - ScienceDirect Full Text PDF
- ## A context evaluation approach for structural comparison of proteins using cross entropy over n-gram modelling

  |  |  |
  | --- | --- |
  | Type | Journal Article |
  | Author | Jafar Razmara |
  | Author | Safaai B. Deris |
  | Author | Sepideh Parvizpour |
  | Volume | 43 |
  | Issue | 10 |
  | Pages | 1614-1621 |
  | Publication | Computers in Biology and Medicine |
  | ISSN | 0010-4825; 1879-0534 |
  | Date | OCT 1 2013 |
  | Extra | WOS:000325735500034 |
  | DOI | 10.1016/j.compbiomed.2013.07.022 |
  | Abstract | The structural comparison of proteins is a vital step in structural biology that is used to predict and analyse a new unknown protein function. Although a number of different techniques have been explored, the study to develop new alternative methods is still an active research area. The present paper introduces a text modelling-based technique for the structural comparison of proteins. The method models the secondary and tertiary structure of proteins in two linear sequences and then applies them to the comparison of two structures. The technique used for pairwise comparison of the sequences has been adopted from computational linguistics and its well-known techniques for analysing and quantifying textual sequences. To this end, an n-gram modelling technique is used to capture regularities between sequences, and then, the cross-entropy concept is employed to measure their similarities. Several experiments are conducted to evaluate the performance of the method and compare it with other commonly used programs. The assessments for information retrieval evaluation demonstrate that the technique has a high running speed, which is similar to other linear encoding methods, such as 3D-BLAST, SARST, and TS-AMIR, whereas its accuracy is comparable to CE and TM-align, which are high accuracy comparison tools. Accordingly, the results demonstrate that the algorithm has high efficiency compared with other state-of-the-art methods. (C) 2013 Elsevier Ltd. All rights reserved. |
  | Date Added | 2/12/2014, 2:18:08 PM |
  | Modified | 2/12/2014, 2:18:08 PM |

  ### Notes:

  - Present text-modeling based method for indexing and retrieval of protein structures.

    How SCOP is used:

    Use ASTRAL data set, filtered at 40% sequence identify. Trained and benchmarked method on fold-level classification.

    SCOP reference:

    These parameters were optimised based on maximising the number of correct fold recognitions when cross-matching the SCOP [28] domains using the PDB40 dataset from the ASTRAL database [29], as described in the results section.

    ...

    3.1. Determining the best form of the n-gram

    The first experiment is to determine the optimum size of the n-gram, to balance the accuracy and sensitivity against the computational efficiency. The experiment uses the PDB40 dataset, which corresponds to the SCOP version 1.61 from the ASTRAL database [29] to extract the 2620 domains that belong to the All Alpha, All Beta, Alpha/Beta and Alpha+Beta SCOP categories. The method was applied to an all-against-all comparison of the protein structures, except for the pairs that have similar first two levels of their SCOP numbers because, at this fold level, SCOP does not differentiate homologous and non-homologous pairs.  Thus, the dataset is reduced to 940,383 protein domain pairs. An accuracy index for a similarity database search is adopted from the Receiver Operating Characteristic (ROC) curve [30]. The index denotes false positive versus true positive rates in the ROC curve for different sizes of n-gram models, considering the SCOP database as the gold standard for indicating structural homology. Moreover, the results are compared with those of the TS-AMIR method [25], another linear encoding method that is based on n-gram modelling, which was recently developed by the authors.

    The performance of the method on different sizes of n-gram models is shown in Fig. 6. From the figure, the ability of the method to determine structural similarities among proteins within the dataset is easily observed. It is clear that 4-gram and 5-gram modelling reaches a high performance level when distinguishing structural homologies for different SCOP categories. Choosing larger sizes of n-grams in this experiment yields approximately the same accuracy. However, larger sizes of n-grams fail to distinguish protein pairs that have low similarity and low biolo- gical significance [27]. Accordingly, we used the 4-gram model as the optimum size of the n-gram in the following experiments. Additionally, the performance on the dataset computed using the ROC curve illustrates that the n-gram method with sizes of 4-gram and above gives similar results compared with the TS-AMIR method for four different SCOP categories.

    3.2. Performance test dataset

    To assess the retrieval efficiency of the method in comparison with other state-of-the-art programs, we used the dataset col- lected by Aung and Tan [31], which has 34,055 proteins from the ASTRAL SCOP 1.59; a total of 108 query proteins were selected from this dataset, which belong to four main categories (All Alpha,

    All Beta, Alpha/Beta and Alpha+Beta) with an average family size of 80. We utilised the same experiments, which were conducted by Lo et al. [16], to evaluate the n-gram method and compare the results with results from CE [5] and TM-align [6], which are two geometric algorithms; YAKUSA [14], 3D-BLAST [15], SARST [16] and TS-AMIR [25], which are four linear encoding techniques; and BLAST [32], which is a sequence search tool. The results, except for the n-gram method, are taken from [16,25].

  ### Attachments

  - 1-s2.0-S0010482513001960-main.pdf
- ## Active clustering of biological sequences

  |  |  |
  | --- | --- |
  | Type | Journal Article |
  | Author | Konstantin Voevodski |
  | Author | Maria-Florina Balcan |
  | Author | Heiko Röglin |
  | Author | Shang-Hua Teng |
  | Author | Yu Xia |
  | URL | http://dl.acm.org/citation.cfm?id=2188392 |
  | Volume | 13 |
  | Pages | 203–225 |
  | Publication | Journal of Machine Learning Research |
  | Date | 2012 |
  | Accessed | 9/20/2013, 1:17:33 PM |
  | Library Catalog | Google Scholar |
  | Date Added | 10/11/2013, 10:29:15 AM |
  | Modified | 11/11/2013, 4:57:18 PM |

  ### Tags:

  - active clustering
  - Approximation Algorithms
  - approximation stability
  - clustering
  - Clustering Accuracy
  - k-Median
  - protein sequences

  ### Notes:

  - Present a clustering algorithm for sequence clustering.

    How SCOP is used:

    Validate cluster predictions, made based on sequence alone, against Pfam and the SCOP classification at the superfamily level.  Derive one dataset from SCOP by randomly choosing several superfamilies and downloading sequences.

    SCOP reference:

    We use our algorithm to cluster proteins by sequence similarity, and compare our results to gold standard manual classifications given in the Pfam (Finn et al., 2010) and SCOP (Murzin et al., 1995) databases. These classification databases are used ubiquitously in biology to observe evolutionary relationships between proteins and to find close relatives of particular proteins. We find that for one of these sources we obtain clusterings that usually closely match the given classification, and for the other the performance of our algorithm is comparable to that of the best known algorithms using the full distance matrix. Both of these classification databases have limited coverage, so a completely automated method such as ours can be useful in clustering proteins that have yet to be classified. Moreover, our method can cluster very large data sets because it is efficient and does not require the full distance matrix as input, which may be infeasible to obtain for a very large data set.

    ...

    SCOP groups proteins on the basis of their 3D structures, so it only classiﬁes proteins whose  
    structure is known. Thus the data sets from SCOP are much smaller in size. The SCOP classiﬁcation  
    is also hierarchical: proteins are grouped by class, fold, superfamily, and family. We consider the  
    classiﬁcation at the superfamily level because this seems most appropriate given that we are only  
    using sequence information. As with the Pfam data, in each experiment we create a data set by  
    randomly choosing several superfamilies (of size between 20 and 200), retrieve the sequences of  
    the corresponding proteins, and use our Landmark-Clustering algorithm to cluster the data set.

  ### Attachments

  - p203-voevodski.pdf
- ## A daily-updated tree of (sequenced) life as a reference for genome research

  |  |  |
  | --- | --- |
  | Type | Journal Article |
  | Author | Hai Fang |
  | Author | Matt E. Oates |
  | Author | Ralph B. Pethica |
  | Author | Jenny M. Greenwood |
  | Author | Adam J. Sardar |
  | Author | Owen J. L. Rackham |
  | Author | Philip C. J. Donoghue |
  | Author | Alexandros Stamatakis |
  | Author | David A. de Lima Morais |
  | Author | Julian Gough |
  | Volume | 3 |
  | Publication | Scientific Reports |
  | ISSN | 2045-2322 |
  | Date | JUN 18 2013 |
  | Extra | WOS:000320500900012 |
  | DOI | 10.1038/srep02015 |
  | Abstract | We report a daily-updated sequenced/species Tree Of Life (sTOL) as a reference for the increasing number of cellular organisms with their genomes sequenced. The sTOL builds on a likelihood-based weight calibration algorithm to consolidate NCBI taxonomy information in concert with unbiased sampling of molecular characters from whole genomes of all sequenced organisms. Via quantifying the extent of agreement between taxonomic and molecular data, we observe there are many potential improvements that can be made to the status quo classification, particularly in the Fungi kingdom; we also see that the current state of many animal genomes is rather poor. To augment the use of sTOL in providing evolutionary contexts, we integrate an ontology infrastructure and demonstrate its utility for evolutionary understanding on: nuclear receptors, stem cells and eukaryotic genomes. The sTOL (http://supfam.org/SUPERFAMILY/sTOL) provides a binary tree of (sequenced) life, and contributes to an analytical platform linking genome evolution, function and phenotype. |
  | Date Added | 10/28/2013, 4:51:00 PM |
  | Modified | 10/28/2013, 4:51:00 PM |

  ### Notes:

  - Describe a database with a daily-updated Tree of Life.

    How SCOP is used:

    Annotate genomes dataset with SCOP domain, superfamily and family.

    SCOP reference:

    Another obstacle for building a tree of life is the presence of horizontal gene transfer (HGT), particularly in bacteria. To mitigate the impact of HGT, we utilise molecular characters in the form of SCOP structural super- families, families, supra-domains and full-length domain architec- tures. These are more tolerant to homoplasy (less HGT-sensitive) than their residual genes/proteins23,24.

    ...

    Methods

    Genomic domain assignment sources in the SUPERFAMILY database. We have compiled SCOP domain assignments over all completely sequenced genomes that are currently available (stored in the SUPERFAMILY database18). New genomes are routinely added, and are automatically annotated with domain assignments using HMMs19. The main results presented here are on a frozen data set, which at the time the work began consisted of 1,731 genomes/species (comprising 1,282 bacteria, 105 archaea, and 344 eukaryotes). The taxonomy used in this work was the subset of nodes and branches extracted from the full NCBI taxonomy relevant to those species for which completely sequenced genomes are available (those in our set). The protein sequences in these genomes were assigned to 1,919 distinct superfamilies and 3,815 distinct families from SCOP (version 1.75). In addition to the presence/absence domain occurrence information, SUPERFAMILY also provides an algorithm for unambiguously converting a protein sequence into ‘domain architecture’, a sequential order of SCOP superfamilies or gaps.

  ### Attachments

  - srep02015.pdf
- ## Adaptive Smith-Waterman residue match seeding for protein structural alignment

  |  |  |
  | --- | --- |
  | Type | Journal Article |
  | Author | Christopher M. Topham |
  | Author | Mickael Rouquier |
  | Author | Nathalie Tarrat |
  | Author | Isabelle Andre |
  | Volume | 81 |
  | Issue | 10 |
  | Pages | 1823-1839 |
  | Publication | Proteins: Structure, Function, and Bioinformatics |
  | ISSN | 0887-3585 |
  | Date | October 2013 |
  | DOI | 10.1002/prot.24327 |
  | Language | English |
  | Abstract | The POLYFIT rigid-body algorithm for automated global pairwise and multiple protein structural alignment is presented. Smith-Waterman local alignment is used to establish a set of seed equivalences that are extended using Needleman-Wunsch dynamic programming techniques. Structural and functional interaction constraints provided by evolution are encoded as one-dimensional residue physical environment strings for alignment of highly structurally overlapped protein pairs. Local structure alignment of more distantly related pairs is carried out using rigid-body conformational matching of 15-residue fragments, with allowance made for less stringent conformational matching of metal-ion and small molecule ligand-contact, disulphide bridge, and cis-peptide correspondences. Protein structural plasticity is accommodated through the stepped adjustment of a single empirical distance parameter value in the calculation of the Smith-Waterman dynamic programming matrix. Structural overlap is used both as a measure of similarity and to assess alignment quality. Pairwise alignment accuracy has been benchmarked against that of 10 widely used aligners on the Sippl and Wiederstein set of difficult pairwise structure alignment problems, and more extensively against that of Matt, SALIGN, and MUSTANG in pairwise and multiple structural alignments of protein domains with low shared sequence identity in the SCOP-ASTRAL 40% compendium. The results demonstrate the advantages of POLYFIT over other aligners in the efficient and robust identification of matching seed residue positions in distantly related protein targets and in the generation of longer structurally overlapped alignment lengths. Superposition-based application areas include comparative modeling and protein and ligand design. POLYFIT is available on the Web server at http://polyfit.insa-toulouse.fr. Proteins 2013; 81:1823-1839. (c) 2013 Wiley Periodicals, Inc. |
  | Date Added | 10/25/2013, 4:17:08 PM |
  | Modified | 3/7/2014, 12:08:45 PM |

  ### Tags:

  - ASTRAL
  - ASTRAL domain structures
  - ASTRAL sequences
  - ASTRAL subsets
  - Cite ASTRAL

  ### Notes:

  - Presents POLYFIT, a method for pairwise and multiple protein structural alignment.

    How SCOP/CATH is used:

    Perform benchmarking with two data sets:

    1. Sippl and Widerstein dataset - consisting of 6 pairs of structures: 5 from SCOP from different folds and 1 from CATH

    2. ASTRAL <=40% representative subset for SCOP 1.75

    SCOP/CATH reference:

    Preparation of domain atom coordinate sets

    Domains were extracted automatically together with nonprotein contacting molecules from remediated (ver- sion 3) RCSB PDB coordinate files84,85 according to chain and residue range specifications in host SCOP v1.7574 or CATH v3.44 database compendia.

    ...

    Following an earlier study by Kolodny et al.,31 this test set was identified by Sippl and Widerstein72 as challenging on the basis that five structure pairs (Cases A through E, Table II) were sufficiently dissimilar to have been assigned to different SCOP folds or CATH topologies, while in the sixth (Case F, Table II), the two domains were assigned to separate SCOP families within the ADP-ribosylation superfamily

    ...

    Pairwise alignment accu- racy has been benchmarked against that of 10 widely used aligners on the Sippl and Wiederstein set of difficult pairwise structure alignment problems, and more extensively against that of Matt, SALIGN, and MUSTANG in pairwise and multiple structural alignments of protein domains with low shared sequence identity in the SCOP-ASTRAL 40% compendium

  ### Attachments

  - prot24327.pdf
- ## A disease-drug-phenotype matrix inferred by walking on a functional domain network

  |  |  |
  | --- | --- |
  | Type | Journal Article |
  | Author | Hai Fang |
  | Author | Julian Gough |
  | Volume | 9 |
  | Issue | 7 |
  | Pages | 1686-1696 |
  | Publication | Molecular Biosystems |
  | ISSN | 1742-206X |
  | Date | 2013 |
  | Extra | WOS:000319882200016 |
  | DOI | 10.1039/c3mb25495j |
  | Abstract | Protein domains are classified as units of structure, evolution and function, and thus form the molecular backbone of biosphere. Although functional networks at the protein level have been reported to be of value in predicting diseases (phenotypes or drugs), they have not previously been applied at the sub-protein resolution (protein domain in this case). We herein introduce a domain network with a functional perspective. This network has nodes consisting of protein domains (at the superfamily/evolutionary level), with edges weighted by the semantic similarity according to domain-centric Gene Ontology (dcGO) annotations, which henceforth we call "dcGOnet". By globally exploring this network via a random walk, we demonstrate its predictive value on disease, drug, or phenotype-related ontologies. On cross-validation recovering ontology labels for domains, we achieve an overall area under the ROC curve of 89.0% for drugs, 87.3% for diseases, 87.6% for human phenotypes and 88.2% for mouse phenotypes. We show that the performance using global information from this network is significantly better than using local information, and also illustrate that the better performance is not sensitive to network size, or the choice of algorithm parameters, and is universal to different ontologies. Based on the dcGOnet and its global properties, we further develop an approach to build a disease-drug-phenotype matrix. The predicted interconnections are statistically supported using a novel randomization procedure, and are also empirically supported by inspection for biological relevance. Most of the high-ranking predictions recover connections that are well known, but others uncover connections that have only suggestive or obscure support in the literature; we show that these are missed by simpler methods, in particular for drug-disease connections. The value of this work is threefold: we describe a general methodology and make the software available, we provide the functional domain network itself, and the ranked drug-disease-phenotype matrix provides rich targets for investigation. All three can be found at http://supfam.org/SUPERFAMILY/dcGO/dcGOnet.html. |
  | Date Added | 2/20/2014, 12:24:01 PM |
  | Modified | 2/20/2014, 12:24:01 PM |

  ### Notes:

  - Present method for function prediction using a network-based approach (domain-centric gene ontology, DcGO).

    How SCOP is used:

    Database is built on SCOP domain data, classified at the superfamily level.

    SCOP reference:

    Protein domains, classified as units of structure, evolution and function by the Structural Classification of Proteins (SCOP)10 database, represent direct manifestations of molecular biosphere. Inspired by the multifaceted utilities of functional networks of whole proteins, we hypothesize that functional networks at a sub-protein domain resolution may also be of great value and utility.

    ...

    Domain-centric annotations of functions, diseases, phenotypes and drugs

    The latest release of the dcGO database14 contains protein domain annotations with GO17 and many other commonly used biomedical ontologies18 including diseases, phenotypes, drugs and so forth. The focus in dcGO is on domains taken from the SCOP database,10 although other domain databases are also annotated. In this study we use SCOP domains classi- fied at the superfamily level (defined as grouping together domains for which there is structure, sequence and function evidence for a common ancestor). The domain-centric annota- tions are statistically inferred from proteins with experimental evidence,19 and intuitively they can be understood as the modes-of-action underlying the protein.

  ### Attachments

  - c3mb25495j.pdf
- ## A domain-centric solution to functional genomics via dcGO Predictor

  |  |  |
  | --- | --- |
  | Type | Journal Article |
  | Author | Hai Fang |
  | Author | Julian Gough |
  | Volume | 14 |
  | Pages | S9 |
  | Publication | Bmc Bioinformatics |
  | ISSN | 1471-2105 |
  | Date | FEB 28 2013 |
  | Extra | WOS:000317187500009 |
  | DOI | 10.1186/1471-2105-14-S3-S9 |
  | Abstract | Background: Computational/manual annotations of protein functions are one of the first routes to making sense of a newly sequenced genome. Protein domain predictions form an essential part of this annotation process. This is due to the natural modularity of proteins with domains as structural, evolutionary and functional units. Sometimes two, three, or more adjacent domains (called supra-domains) are the operational unit responsible for a function, e. g. via a binding site at the interface. These supra-domains have contributed to functional diversification in higher organisms. Traditionally functional ontologies have been applied to individual proteins, rather than families of related domains and supra-domains. We expect, however, to some extent functional signals can be carried by protein domains and supra-domains, and consequently used in function prediction and functional genomics. Results: Here we present a domain-centric Gene Ontology (dcGO) perspective. We generalize a framework for automatically inferring ontological terms associated with domains and supra-domains from full-length sequence annotations. This general framework has been applied specifically to primary protein-level annotations from UniProtKB-GOA, generating GO term associations with SCOP domains and supra-domains. The resulting 'dcGO Predictor', can be used to provide functional annotation to protein sequences. The functional annotation of sequences in the Critical Assessment of Function Annotation (CAFA) has been used as a valuable opportunity to validate our method and to be assessed by the community. The functional annotation of all completely sequenced genomes has demonstrated the potential for domain-centric GO enrichment analysis to yield functional insights into newly sequenced or yet-to-be-annotated genomes. This generalized framework we have presented has also been applied to other domain classifications such as InterPro and Pfam, and other ontologies such as mammalian phenotype and disease ontology. The dcGO and its predictor are available at http://supfam.org/SUPERFAMILY/dcGO including an enrichment analysis tool. Conclusions: As functional units, domains offer a unique perspective on function prediction regardless of whether proteins are multi-domain or single-domain. The 'dcGO Predictor' holds great promise for contributing to a domain-centric functional understanding of genomes in the next generation sequencing era. |
  | Date Added | 2/20/2014, 12:24:01 PM |
  | Modified | 2/20/2014, 12:24:01 PM |

  ### Notes:

  - Present dcGO (domain-centric Gene Ontology)  SUPERFAMILY-based method for function prediction for CAFA.

    How SCOP is used:

    Annotate domains and get superfamily classification with SUPERFAMILY.

    SCOP reference:

    In abstract:

    This general framework has been applied specifically to primary protein-level annotations from UniProtKB-GOA, generating GO term associations with SCOP domains and supra-domains.

  ### Attachments

  - 1471-2105-14-S3-S9.pdf
- ## A Dynamic Data-Driven Framework for Biological Data Using 2D Barcodes

  |  |  |
  | --- | --- |
  | Type | Journal Article |
  | Author | Hui Li |
  | Author | Chunmei Liu |
  | Pages | 892098 |
  | Publication | Computational and Mathematical Methods in Medicine |
  | ISSN | 1748-670X |
  | Date | 2012 |
  | Extra | WOS:000312813200001 |
  | DOI | 10.1155/2012/892098 |
  | Abstract | Biology data is increasing exponentially from biological laboratories. It is a complicated problem for further processing the data. Processing computational data and data from biological laboratories manually may lead to potential errors in further analysis. In this paper, we proposed an efficient data-driven framework to inspect laboratory equipment and reduce impending failures. Our method takes advantage of the 2D barcode technology which can be installed on the specimen as a trigger for the data-driven system. For this end, we proposed a series of algorithms to speed up the data processing. The results show that the proposed system increases the system's scalability and flexibility. Also, it demonstrates the ability of linking a physical object with digital information to reduce the manual work related to experimental specimen. The characteristics such as high capacity of storage and data management of the 2D barcode technology provide a solution to collect experimental laboratory data in a quick and accurate fashion. |
  | Date Added | 2/20/2014, 12:24:01 PM |
  | Modified | 2/20/2014, 12:24:01 PM |

  ### Notes:

  - Present method for data management of biological data.

    How SCOP is used:

    Background on biological data classification.

    SCOP reference:

    It is necessary and urgent to propose an efficient computational approach to systematically manage and simplify the whole process to improve biology data management and to elim- inate potential errors as well as save time [1–3].

  ### Attachments

  - 892098.pdf
- ## A feature extraction technique using bi-gram probabilities of position specific scoring matrix for protein fold recognition

  |  |  |
  | --- | --- |
  | Type | Journal Article |
  | Author | Alok Sharma |
  | Author | James Lyons |
  | Author | Abdollah Dehzangi |
  | Author | Kuldip K. Paliwal |
  | URL | http://www.sciencedirect.com/science/article/pii/S0022519312006327 |
  | Publication | Journal of theoretical biology |
  | Date | 2012 |
  | Accessed | 9/23/2013, 10:16:21 AM |
  | Library Catalog | Google Scholar |
  | Date Added | 10/11/2013, 10:29:15 AM |
  | Modified | 10/11/2013, 10:29:15 AM |

  ### Tags:

  - Bi-gram features
  - Position specific scoring matrix (PSSM)
  - Protein fold recognition
  - protein sequence

  ### Notes:

  - Present method for fold recognition from sequence data.  Use a novel feature extraction technique.  Benchmark on a data set of SCOP data from a previous study.

    How SCOP is used:

    Train method on a nonredundant data set of 311 protein sequences, and validate on data set of 383 protein sequences from 27 SCOP folds representing the top 4 structural classes.

    SCOP Reference:

    3. Dataset  
    In this study,the benchmark DD protein sequence dataset  
    (Ding and Dubchak,2001) have been employed.The DD-dataset consists of 311 protein sequences in the training set where two proteins have no more than 35% of sequence identity for aligned subsequence longer than 80 residues.The test set consists of 383 protein sequences where sequence identity is less than 40%. Both the sets belong to 27 SCOP folds (Murzin etal.,1995; http://scop.  
    mrc-lmb.cam.ac.uk/scop/) which represented all major structural classes: a, b, a=b, and aþb (Ding andDubchak,2001).  The summary of DD-dataset has been given in Table 2.

  ### Attachments

  - [PDF] from griffith.edu.au
- ## A Global Characterization and Identification of Multifunctional Enzymes

  |  |  |
  | --- | --- |
  | Type | Journal Article |
  | Author | Xian-Ying Cheng |
  | Author | Wei-Juan Huang |
  | Author | Shi-Chang Hu |
  | Author | Hai-Lei Zhang |
  | Author | Hao Wang |
  | Author | Jing-Xian Zhang |
  | Author | Hong-Huang Lin |
  | Author | Yu-Zong Chen |
  | Author | Quan Zou |
  | Author | Zhi-Liang Ji |
  | Volume | 7 |
  | Issue | 6 |
  | Pages | e38979 |
  | Publication | Plos One |
  | ISSN | 1932-6203 |
  | Date | JUN 18 2012 |
  | Extra | WOS:000305583300076 |
  | DOI | 10.1371/journal.pone.0038979 |
  | Abstract | Multi-functional enzymes are enzymes that perform multiple physiological functions. Characterization and identification of multi-functional enzymes are critical for communication and cooperation between different functions and pathways within a complex cellular system or between cells. In present study, we collected literature-reported 6,799 multi-functional enzymes and systematically characterized them in structural, functional, and evolutionary aspects. It was found that four physiochemical properties, that is, charge, polarizability, hydrophobicity, and solvent accessibility, are important for characterization of multi-functional enzymes. Accordingly, a combinational model of support vector machine and random forest model was constructed, based on which 6,956 potential novel multi-functional enzymes were successfully identified from the ENZYME database. Moreover, it was observed that multi-functional enzymes are non-evenly distributed in species, and that Bacteria have relatively more multi-functional enzymes than Archaebacteria and Eukaryota. Comparative analysis indicated that the multi-functional enzymes experienced a fluctuation of gene gain and loss during the evolution from S. cerevisiae to H. sapiens. Further pathway analyses indicated that a majority of multi-functional enzymes were well preserved in catalyzing several essential cellular processes, for example, metabolisms of carbohydrates, nucleotides, and amino acids. What's more, a database of known multi-functional enzymes and a server for novel multi-functional enzyme prediction were also constructed for free access at http://bioinf.xmu.edu.cn/databases/MFEs/index.htm. |
  | Date Added | 2/20/2014, 12:24:01 PM |
  | Modified | 2/20/2014, 12:24:01 PM |

  ### Notes:

  - Computational study of multi-functional enzymes (MFEs), enzymes that perform multiple physiological functions.  Collected 6,799 such enzymes from the literature and studies structure, function, and evolutionary relationships.

    How SCOP is used:

    Annotate a data set of MFEs by SCOP class, to measure the structural diversity.

    SCOP reference:

    To have an overview of MFEs’ structural propensities, the distribution of several protein groups in Structural Classification of Proteins (SCOP) database [30] was investigated. The analysis covers 140 known MCD-MFEs, 29 known SMAD-MFEs, 2,155 enzymes and total 38,221 Protein Data Bank (PDB) Entries included in the SCOP 1.75 release database (June 2009). As illustrated in Figure 3, about 38.57% of MCD-MFEs and 44.83% of SMAD-MFEs belong to alpha and beta proteins (a/b); while only about 24.85% of total proteins in SCOP database are in a/b topology. It seems that MFEs have a structural propensity in alpha and beta topology. The propensity of a/b topology would be a general characteristic of enzyme.. Be aware that these results were achieved subject to current availability of protein structures in SCOP, which is limited and bias due to the difficulty in structure determination. However, some recent studies proposed that alpha and beta topology was common for moonlighting proteins [31,32], which would be a good case to support our finding.

  ### Attachments

  - journal.pone.0038979.pdf
- ## A Global Comparison of the Human and T. brucei Degradomes Gives Insights about Possible Parasite Drug Targets

  |  |  |
  | --- | --- |
  | Type | Journal Article |
  | Author | Susan T. Mashiyama |
  | Author | Kyriacos Koupparis |
  | Author | Conor R. Caffrey |
  | Author | James H. McKerrow |
  | Author | Patricia C. Babbitt |
  | Volume | 6 |
  | Issue | 12 |
  | Publication | Plos Neglected Tropical Diseases |
  | ISSN | 1935-2735 |
  | Date | DEC 2012 |
  | Extra | WOS:000312910200015 |
  | DOI | 10.1371/journal.pntd.0001942 |
  | Abstract | We performed a genome-level computational study of sequence and structure similarity, the latter using crystal structures and models, of the proteases of Homo sapiens and the human parasite Trypanosoma brucei. Using sequence and structure similarity networks to summarize the results, we constructed global views that show visually the relative abundance and variety of proteases in the degradome landscapes of these two species, and provide insights into evolutionary relationships between proteases. The results also indicate how broadly these sequence sets are covered by three-dimensional structures. These views facilitate cross-species comparisons and offer clues for drug design from knowledge about the sequences and structures of potential drug targets and their homologs. Two protease groups ("M32" and "C51") that are very different in sequence from human proteases are examined in structural detail, illustrating the application of this global approach in mining new pathogen genomes for potential drug targets. Based on our analyses, a human ACE2 inhibitor was selected for experimental testing on one of these parasite proteases, TbM32, and was shown to inhibit it. These sequence and structure data, along with interactive versions of the protein similarity networks generated in this study, are available at http://babbittlab.ucsf.edu/resources.html. |
  | Date Added | 10/28/2013, 4:57:32 PM |
  | Modified | 3/7/2014, 12:14:19 PM |

  ### Tags:

  - Computational Biology
  - Humans
  - Models, Molecular
  - Peptide Hydrolases
  - Protein Conformation
  - Sequence Homology, Amino Acid
  - Trypanosoma brucei brucei

  ### Notes:

  - Computational study of proteases that might be drug targets. Study proteases in human and the T. brucei parasite, which causes human African trypanosomiasis or sleeping sickness.

    How SCOP is used:

    Note that some families studied, that may or may not be evolutionary related, have similar structure and SCOP has them "annotated accordingly".

    How CATH is used:

    Not using CATH data.  Cite for background, along with SCOP.

    SCOP reference:

    The second mixed cluster (Figure 3A) contains families M14, M17, M20, M28, and C15. Unlike the first cluster discussed above, these families are assigned to different MEROPS clans (Figure 4): MC (M14), MF (M17), MH (M20 and M28), and CF (C15). This is based on differences in catalytic mechanism and non-conserved locations of metal-binding residues [20]. Structural similarity between members of these families has been detected by others and is annotated accordingly in the SCOP structural classification database [**59**], but opinions differ whether they are evolutionarily related [20,60].

    CATH reference:

    Structure similarity is often used as evidence, along with functional similarity, that proteins with divergent sequences are evolutionarily related (i.e., are homologs) [56–58].

  ### Attachments

  - journal.pntd.0001942.pdf
- ## A Glutathione Transferase from Agrobacterium tumefaciens Reveals a Novel Class of Bacterial GST Superfamily

  |  |  |
  | --- | --- |
  | Type | Journal Article |
  | Author | Katholiki Skopelitou |
  | Author | Prathusha Dhavala |
  | Author | Anastassios C. Papageorgiou |
  | Author | Nikolaos E. Labrou |
  | Volume | 7 |
  | Issue | 4 |
  | Pages | e34263 |
  | Publication | Plos One |
  | Date | April 2012 |
  | DOI | 10.1371/journal.pone.0034263 |
  | Abstract | In the present work, we report a novel class of glutathione transferases (GSTs) originated from the pathogenic soil bacterium Agrobacterium tumefaciens C58, with structural and catalytic properties not observed previously in prokaryotic and eukaryotic GST isoenzymes. A GST-like sequence from A. tumefaciens C58 (Atu3701) with low similarity to other characterized GST family of enzymes was identified. Phylogenetic analysis showed that it belongs to a distinct GST class not previously described and restricted only in soil bacteria, called the Eta class (H). This enzyme (designated as AtuGSTH1-1) was cloned and expressed in E. coli and its structural and catalytic properties were investigated. Functional analysis showed that AtuGSTH1-1 exhibits significant transferase activity against the common substrates aryl halides, as well as very high peroxidase activity towards organic hydroperoxides. The crystal structure of AtuGSTH1-1 was determined at 1.4 angstrom resolution in complex with S-(p-nitrobenzyl)-glutathione (Nb-GSH). Although AtuGSTH1-1 adopts the canonical GST fold, sequence and structural characteristics distinct from previously characterized GSTs were identified. The absence of the classic catalytic essential residues (Tyr, Ser, Cys) distinguishes AtuGSTH1-1 from all other cytosolic GSTs of known structure and function. Site-directed mutagenesis showed that instead of the classic catalytic residues, an Arg residue (Arg34), an electron-sharing network, and a bridge of a network of water molecules may form the basis of the catalytic mechanism. Comparative sequence analysis, structural information, and site-directed mutagenesis in combination with kinetic analysis showed that Phe22, Ser25, and Arg187 are additional important residues for the enzyme's catalytic efficiency and specificity. |
  | Date Added | 3/7/2014, 1:06:24 PM |
  | Modified | 3/7/2014, 1:06:24 PM |
- ## A holistic in silico approach to predict functional sites in protein structures

  |  |  |
  | --- | --- |
  | Type | Journal Article |
  | Author | Joan Segura |
  | Author | Pamela F. Jones |
  | Author | Narcis Fernandez-Fuentes |
  | URL | http://bioinformatics.oxfordjournals.org/content/28/14/1845.short |
  | Volume | 28 |
  | Issue | 14 |
  | Pages | 1845–1850 |
  | Publication | Bioinformatics |
  | Date | 2012 |
  | Accessed | 9/20/2013, 1:18:20 PM |
  | Library Catalog | Google Scholar |
  | Date Added | 10/11/2013, 10:29:15 AM |
  | Modified | 10/11/2013, 10:29:15 AM |

  ### Notes:

  - Introduces a method, Multi-VORFFIP for predicting binding sites in proteins, using structural, evolutionary, experimental, and energy-based features and a Random Forest classifier.

    How SCOP used:

    Use previously compiled datasets that have the property that no two proteins belong to the same SCOP family to benchmark their binding site prediction method.

    SCOP reference:

    Three different datasets, PEP-set, DNA-set and RNA-set, extracted from recent publications, were used to benchmark MV. Benchmark 4.0 dataset (Hwang et al., 2010), named PROT-set, was also used to assess the selectivity of the predictions. The PROT-set is a dataset of 176 protein–protein complexes specifically compiled for docking evaluation. No two single pairs of complexes belong to the same SCOP family. The PEP-set is a dataset of protein–peptides complexes compiled by Petsalaki et al. (2009) and it is composed of a non-redundant set [i.e. does not include protein–peptide complexes that belong to the same SCOP family (Murzin et al., 1995)] of 405 protein–peptides structure complexes solved both in bound and unbound conformation.

  ### Attachments

  - Full Text PDF
- ## A homology/ab initio hybrid algorithm for sampling near-native protein conformations

  |  |  |
  | --- | --- |
  | Type | Journal Article |
  | Author | Priyanka Dhingra |
  | Author | Bhyravabhotla Jayaram |
  | URL | http://onlinelibrary.wiley.com/doi/10.1002/jcc.23339/full |
  | Publication | Journal of computational chemistry |
  | Date | 2013 |
  | Accessed | 9/23/2013, 10:21:39 AM |
  | Library Catalog | Google Scholar |
  | Date Added | 10/11/2013, 10:29:15 AM |
  | Modified | 10/11/2013, 10:29:15 AM |

  ### Tags:

  - ab initio modeling
  - fold recognition
  - homology modeling
  - loop modeling
  - protein folding
  - protein tertiary structure prediction

  ### Notes:

  - Present method for protein conformational sampling for protein tertiary structure prediction. The algorithm makes use of homology and fold recognition techniques.

    How SCOP is used:

    Search for homologs in PDB, SCOP and Pfam using BLAST.  Use homologs for template-based modeling for secondary structure prediction.

    SCOP Reference:

    The overall strategy of Bhageerath-H Strgen consists of seven steps. (1) The first step involves searching the databases for sequence and family based homologs of the input amino acid sequence.(2)...

    ...

    Secondary structure prediction and database search

    Secondary structure of the input polypeptide sequence is pre- dicted using PSIPRED[60] software. The input sequence is searched in the PDB database using Blastp[61] (expectation value 1000) for finding close sequence homologs with a known structure and Pfam[62] and SCOP[63,64] databases for proteins with similar domains and family. All the hits from the database searches are used for template-based modeling in the subsequent step.

  ### Attachments

  - jcc23339.pdf
- ## A Horizontal Alignment Tool for Numerical Trend Discovery in Sequence Data: Application to Protein Hydropathy

  |  |  |
  | --- | --- |
  | Type | Journal Article |
  | Author | Omar Hadzipasic |
  | Author | James O. Wrabl |
  | Author | Vincent J. Hilser |
  | Volume | 9 |
  | Issue | 10 |
  | Pages | e1003247 |
  | Publication | Plos Computational Biology |
  | Date | October 2013 |
  | DOI | 10.1371/journal.pcbi.1003247 |
  | Abstract | An algorithm is presented that returns the optimal pairwise gapped alignment of two sets of signed numerical sequence values. One distinguishing feature of this algorithm is a flexible comparison engine (based on both relative shape and absolute similarity measures) that does not rely on explicit gap penalties. Additionally, an empirical probability model is developed to estimate the significance of the returned alignment with respect to randomized data. The algorithm's utility for biological hypothesis formulation is demonstrated with test cases including database search and pairwise alignment of protein hydropathy. However, the algorithm and probability model could possibly be extended to accommodate other diverse types of protein or nucleic acid data, including positional thermodynamic stability and mRNA translation efficiency. The algorithm requires only numerical values as input and will readily compare data other than protein hydropathy. The tool is therefore expected to complement, rather than replace, existing sequence and structure based tools and may inform medical discovery, as exemplified by proposed similarity between a chlamydial ORFan protein and bacterial colicin pore-forming domain. The source code, documentation, and a basic web-server application are available. |
  | Date Added | 3/7/2014, 1:06:24 PM |
  | Modified | 3/7/2014, 1:06:24 PM |
- ## A hybrid discriminative/generative approach to protein fold recognition

  |  |  |
  | --- | --- |
  | Type | Journal Article |
  | Author | Wies\Law Chmielnicki |
  | URL | http://www.sciencedirect.com/science/article/pii/S092523121100395X |
  | Volume | 75 |
  | Issue | 1 |
  | Pages | 194–198 |
  | Publication | Neurocomputing |
  | Date | 2012 |
  | Accessed | 9/23/2013, 10:22:14 AM |
  | Library Catalog | Google Scholar |
  | Date Added | 10/11/2013, 10:29:15 AM |
  | Modified | 10/8/2014, 12:50:25 PM |

  ### Tags:

  - Protein fold recognition
  - RDA classifier
  - Statistical classifiers
  - support vector machine

  ### Notes:

  - Authors created a hybrid classifier based on the generative and discriminative approaches. It is used for protein structure prediction and classification.

    How SCOP is used:

    Benchmarked methods on 2 different data sets derived from SCOP. The sets were each >300 sequences and from each of the major classes and folds.

    SCOP Reference:

    In experiments described in this paper two data sets derived from the structural classification of proteins (SCOP) database [14] are used. The detailed description of these sets can be found in [2]. The training set consists of 313 protein sequences and the testing set consists of 385 protein sequences. These data sets include proteins from 27 most populated different classes (pro- tein folds) representing all major structural classes: a, b, a=b, and aþb. The training set was based on PDB\_select sets [15,16] where two proteins have no more than 35% of the sequence identity. The testing set was based on PDB-40D set [17] from which represen- tatives of the same 27 largest folds are selected. The proteins that had higher than 35% identity with the proteins of the training set are removed from the testing set.

  ### Attachments

  - 1-s2.0-S092523121100395X-main.pdf
- ## Alignment of Helical Membrane Protein Sequences Using AlignMe

  |  |  |
  | --- | --- |
  | Type | Journal Article |
  | Author | Marcus Stamm |
  | Author | René Staritzbichler |
  | Author | Kamil Khafizov |
  | Author | Lucy R. Forrest |
  | URL | http://dx.plos.org/10.1371/journal.pone.0057731 |
  | Volume | 8 |
  | Issue | 3 |
  | Pages | e57731 |
  | Publication | PloS one |
  | Date | 2013 |
  | Accessed | 9/20/2013, 1:17:33 PM |
  | Library Catalog | Google Scholar |
  | Date Added | 10/11/2013, 10:29:15 AM |
  | Modified | 10/11/2013, 10:29:15 AM |

  ### Notes:

  - Present Alignment of Membrane proteins (AlignMe) method for sequence alignment and evaluate on helical membrane protein sequences.

    How SCOP is used:

    Do not use SCOP data.  Justify a choice structure similarity cutoff distance by mentioning that it is "roughly equivalent" to that found in SCOP superfamilies.

    SCOP reference:

    1.3 HOMEP2 Training and Test Set

    The original HOMEP dataset contained 36 structures [17]; in subsequent years there was a significant increase in the number of available membrane protein structures [52]. To update the database, we introduced a more automated procedure. First, structures and transmembrane definitions were collected from the PDB\_TM database (dated 17th March 2010) [53,54], and filtered to remove NMR structures, theoretical models and structures with resolution .3.5 A ̊ . Individual membrane-spanning chains were extracted and assigned to either a or b subsets, according to PDB\_TM. Next, all chains within a subset (a or b) were aligned with all other chains using a structural alignment program SKA [55,56], unless the two chains belonged to the same PDB entry. For pairs of chains with .85% identical residues (according to the structure-based alignment), only the structure with higher resolu- tion, or smaller R-factor, was retained.

    This non-redundant set was then clustered to identify families of related structures. The clustering method (File S1, Figure S1) is based on the protein structure distance (PSD) value that is calculated during SKA structural alignments [55]; **here we assume that two proteins are homologous if the PSD** **,****1.2, which is roughly equivalent to belonging to the same superfamily according to the SCOP structural classification scheme [57].** The resultant HOMEP2 data set (File S2) includes 125 structures belonging to 31 structurally distinct families. The subset of a-helical proteins used here contains 81 structures clustered into 22 families containing 177 pair-wise alignments (see File S1, Tables S1 and S2). During cross-validation, 2 of those 22 families were left out in each of 11 repetitions. The structure-based alignments obtained using the SKA program [55] were used as references against which alignment quality on the HOMEP2 set was evaluated (see legend in File S1, Table S2).

  ### Attachments

  - journal.pone.0057731.pdf
- ## A Method for WD40 Repeat Detection and Secondary Structure Prediction

  |  |  |
  | --- | --- |
  | Type | Journal Article |
  | Author | Yang Wang |
  | Author | Fan Jiang |
  | Author | Zhu Zhuo |
  | Author | Xian-Hui Wu |
  | Author | Yun-Dong Wu |
  | Volume | 8 |
  | Issue | 6 |
  | Publication | Plos One |
  | ISSN | 1932-6203 |
  | Date | JUN 11 2013 |
  | Extra | WOS:000320755400058 |
  | DOI | 10.1371/journal.pone.0065705 |
  | Abstract | WD40-repeat proteins (WD40s), as one of the largest protein families in eukaryotes, play vital roles in assembling protein-protein/DNA/RNA complexes. WD40s fold into similar beta-propeller structures despite diversified sequences. A program WDSP (WD40 repeat protein Structure Predictor) has been developed to accurately identify WD40 repeats and predict their secondary structures. The method is designed specifically for WD40 proteins by incorporating both local residue information and non-local family-specific structural features. It overcomes the problem of highly diversified protein sequences and variable loops. In addition, WDSP achieves a better prediction in identifying multiple WD40-domain proteins by taking the global combination of repeats into consideration. In secondary structure prediction, the average Q3 accuracy of WDSP in jack-knife test reaches 93.7%. A disease related protein LRRK2 was used as a representive example to demonstrate the structure prediction. |
  | Date Added | 10/28/2013, 4:53:08 PM |
  | Modified | 10/28/2013, 4:53:08 PM |

  ### Notes:

  - Present method for detecting WD40 repeats and predicting secondary structures from sequence.  WD40-repeat domains are one of the largest protein families.  They provide platforms to assemble complexes.

    How SCOP is used:

    Curate a dataset from SCOP of WD-40 and non-WD40 domains classified by structural class.

    SCOP reference:

    An Unbiased Data Set of Available WD40 Crystal Structures

    The first step of scoring function development is to establish a database of WD40 proteins with known crystal structures, which are classified by both CATH/SCOP and assignments from the literature. Every currently known WD40 protein has at least one DHSW tetrad H-bond network. By calculating their WD40 domain pairwise sequence identities, 33 WD40 proteins were selected in the training set (Table S1). These proteins have no more than 32% pairwise sequence identities in the WD40 domains. 239 WD40 repeats in 33 proteins have average 16% pairwise sequence identity (93.3% of repeats have less than 30% pairwise sequence identity). This ensures a statistically unbiased training set.

  ### Attachments

  - journal.pone.0065705.pdf
- ## Amino acid distribution rules predict protein fold

  |  |  |
  | --- | --- |
  | Type | Journal Article |
  | Author | Alexander E. Kister |
  | Author | Vladimir Potapov |
  | URL | http://212.250.180.38/bst/041/0616/0410616.pdf |
  | Volume | 41 |
  | Issue | part 2 |
  | Pages | 616–619 |
  | Publication | Biochemical Society Transactions |
  | Date | 2013 |
  | Accessed | 9/23/2013, 10:03:53 AM |
  | Library Catalog | Google Scholar |
  | Date Added | 10/11/2013, 10:29:15 AM |
  | Modified | 3/7/2014, 12:09:29 PM |

  ### Tags:

  - amino acid distribution
  - inter-residue interaction
  - protein fold
  - sequence-structure relationship
  - structure prediction
  - supersecondary structure

  ### Notes:

  - Present novel method for structure prediction which relies on statistics on amino acid distributions.

    How SCOP/CATH is used:

    Refer the reader to SCOP and CATH sites for "detailed structure classification" of Beta-sandwich like proteins.

    SCOP reference:

    Supersecondary structures of β-sandwich-like proteins  
     Spatial structures of sandwich-like proteins are composed of β-strands, which form β-sheets that pack face-to-face. The number of strands and their arrangement varies widely [15]. **Detailed structural classification of these proteins is presented in two protein structure databases, SCOP [16] and CATH [17].**

  ### Attachments

  - [PDF] from 212.250.180.38
- ## Aminoacylation of tRNA 2 '- or 3 '-hydroxyl by phosphoseryl- and pyrrolysyl-tRNA synthetases

  |  |  |
  | --- | --- |
  | Type | Journal Article |
  | Author | Markus Englert |
  | Author | Sarath Moses |
  | Author | Michael Hohn |
  | Author | Jiqiang Ling |
  | Author | Patrick O'Donoghue |
  | Author | Dieter Soell |
  | Volume | 587 |
  | Issue | 20 |
  | Pages | 3360-3364 |
  | Publication | Febs Letters |
  | ISSN | 0014-5793 |
  | Date | OCT 11 2013 |
  | Extra | WOS:000325078600012 |
  | DOI | 10.1016/j.febslet.2013.08.037 |
  | Abstract | Class I and II aminoacyl-tRNA synthetases (AARSs) attach amino acids to the 2'- and 3'-OH of the tRNA terminal adenosine, respectively. One exception is phenylalanyl-tRNA synthetase (PheRS), which belongs to Class II but attaches phenylalanine to the 2'-OH. Here we show that two Class II AARSs, O-phosphoseryl- (SepRS) and pyrrolysyl-tRNA (PylRS) synthetases, aminoacylate the 2'- and 3'-OH, respectively. Structure-based-phylogenetic analysis reveals that SepRS is more closely related to PheRS than PylRS, suggesting that the idiosyncratic feature of 2'-OH acylation evolved after the split between PheRS and PylRS. Our work completes the understanding of tRNA aminoacylation positions for the 22 natural AARSs. (C) 2013 Federation of European Biochemical Societies. Published by Elsevier B. V. All rights reserved. |
  | Date Added | 10/28/2013, 4:51:00 PM |
  | Modified | 10/28/2013, 4:51:00 PM |

  ### Notes:

  - Experimental and computational study of a family of interest: class II aminoacyl-tRNA synthetases

    How SCOP is used:

    Download all structures from the same family, and use structure alignment to build a phylogenetic tree.

    SCOP reference:

    2.4. Structure-based phylogenetic analysis

    Protein structures were downloaded from the protein databank or the SCOP database [26] and aligned using Multiseq 2.0 [27]. The tree was calculated from the structural similarity metric QH [28]. The tree was drawn based on the QH distance matrix computed in Multiseq 2.0 using Phylip 3.66 Neighbor and Drawtree programs [29].

  ### Attachments

  - 1-s2.0-S0014579313006662-main.pdf
- ## A molecular dynamics and knowledge-based computational strategy to predict native-like structures of polypeptides

  |  |  |
  | --- | --- |
  | Type | Journal Article |
  | Author | Marcio Dorn |
  | Author | Luciana S. Buriol |
  | Author | Luis C. Lamb |
  | Volume | 40 |
  | Issue | 2 |
  | Pages | 698-706 |
  | Publication | EXPERT SYSTEMS WITH APPLICATIONS |
  | ISSN | 0957-4174 |
  | Date | FEB 1 2013 |
  | DOI | 10.1016/j.eswa.2012.08.003 |
  | Language | English |
  | Abstract | One of the main research problems in structural bioinformatics is the prediction of three-dimensional structures (3-D) of polypeptides or proteins. The current rate at which amino acid sequences are identified increases much faster than the 3-D protein structure determination by experimental methods, such as X-ray diffraction and NMR techniques. The determination of protein structures is both experimentally expensive and time consuming. Predicting the correct 3-D structure of a protein molecule is an intricate and arduous task. The protein structure prediction (PSP) problem is, in computational complexity theory, an NP-complete problem. In order to reduce computing time, current efforts have targeted hybridizations between ab initio and knowledge-based methods aiming at efficient prediction of the correct structure of polypeptides. In this article we present a hybrid method for the 3-D protein structure prediction problem. An artificial neural network knowledge-based method that predicts approximated 3-D protein structures is combined with an ab initio strategy. Molecular dynamics (MD) simulation is used to the refinement of the approximated 3-D protein structures. In the refinement step, global interactions between each pair of atoms in the molecule (including non-bond interactions) are evaluated. The developed MD protocol enables us to correct polypeptide torsion angles deviation from the predicted structures and improve their stereo-chemical quality. The obtained results shows that the time to predict native-like 3-D structures is considerably reduced. We test our computational strategy with four mini proteins whose sizes vary from 19 to 34 amino acid residues. The structures obtained at the end of 32.0 nanoseconds (ns) of MD simulation were comparable topologically to their correspondent experimental structures. (C) 2012 Elsevier Ltd. All rights reserved. |
  | Date Added | 10/11/2013, 10:29:15 AM |
  | Modified | 10/11/2013, 10:29:15 AM |

  ### Tags:

  - Ab initio structure prediction
  - Molecular dynamics simulation
  - protein structure prediction
  - Structural bioinformatics

  ### Notes:

  - Present a method for protein structure prediction.

    How SCOP is used:

    Get class-levels for 4 "mini-proteins" from the PDB.  The 4 proteins are in the designed-protein, peptide, and small-protein classes.

    SCOP reference:

    2.2. Model and target proteins

    The amino acid sequence of four mini proteins are obtained from the PDB (Berman et al., 2000) and used as study cases in our exper- iments: 1ZDD (Starovasnik, Braisted, & Wells, 1997) (Fig. 2(A)/ Cyan), 1ALE (Rozek, Buchko, & Cushley, 1995) (Fig. 2(B)/Cyan), 1ARE (Hoffman, Horvath, & Klevit, 1997) (Fig. 2(C)/Cyan) and 1A11 (Opella et al., 1999) (Fig. 2(D)/Cyan). Fig. 1 presents the sec- ondary structure organization of each one of the tested proteins. Secondary structure analysis were performed by PROMOTIF (Hutchinson & Thornton, 1996). These study cases were selected in order to test our method with different classes of polypeptides with different folding patterns. These same used cases were present in Dorn and Norberto de Souza (2010).

    The polypeptide 1ZDD is a disulfide-stabilized mini protein composed of 34 amino acid residues (Fig. 1(A)) known to be ar- ranged as two a-helices connected by a turn, a structural motif known as an a-helical hairpin. 1ZDD is classified by SCOP2 (Murzin, Brenner, Hubbard, & Cothia, 1995) as a designed-protein. 1ALE is a peptide (SCOP) composed by 18 amino acid residues (Fig. 1(B)) pre- senting only a a-helix regular structure. 1ARE is a small protein (SCOP) composed by 29 amino acid residues (Fig. 1(C)) known by the arrangement of one a-helix and two b-strands. 1A11 is a peptide (SCOP) composed by 25 amino acid residues (Fig. 1(D

  ### Attachments

  - 1-s2.0-S0957417412009645-main.pdf
- ## A multi-faceted analysis of RutD reveals a novel family of alpha/beta hydrolases

  |  |  |
  | --- | --- |
  | Type | Journal Article |
  | Author | Aleksandra A. Knapik |
  | Author | Janusz J. Petkowski |
  | Author | Zbyszek Otwinowski |
  | Author | Marcin T. Cymborowski |
  | Author | David R. Cooper |
  | Author | Karolina A. Majorek |
  | Author | Maksymilian Chruszcz |
  | Author | Wanda M. Krajewska |
  | Author | Wladek Minor |
  | Volume | 80 |
  | Issue | 10 |
  | Pages | 2359-2368 |
  | Publication | Proteins-Structure Function and Bioinformatics |
  | ISSN | 0887-3585 |
  | Date | OCT 2012 |
  | Extra | WOS:000308540300003 |
  | DOI | 10.1002/prot.24122 |
  | Abstract | The rut pathway of pyrimidine catabolism is a novel pathway that allows pyrimidine bases to serve as the sole nitrogen source in suboptimal temperatures. The rut operon in E. coli evaded detection until 2006, yet consists of seven proteins named RutA, RutB, etc. through RutG. The operon is comprised of a pyrimidine transporter and six enzymes that cleave and further process the uracil ring. Herein, we report the structure of RutD, a member of the a/beta hydrolase superfamily, which is proposed to enhance the rate of hydrolysis of aminoacrylate, a toxic side product of uracil degradation, to malonic semialdehyde. Although this reaction will occur spontaneously in water, the toxicity of aminoacrylate necessitates catalysis by RutD for efficient growth with uracil as a nitrogen source. RutD has a novel and conserved arrangement of residues corresponding to the a/beta hydrolase active site, where the nucleophile's spatial position occupied by Ser, Cys, or Asp of the canonical catalytic triad is replaced by histidine. We have used a combination of crystallographic structure determination, modeling and bioinformatics, to propose a novel mechanism for this enzyme. This approach also revealed that RutD represents a previously undescribed family within the a/beta hydrolases. We compare and contrast RutD with PcaD, which is the closest structural homolog to RutD. PcaD is a 3-oxoadipate-enol-lactonase with a classic arrangement of residues in the active site. We have modeled a substrate in the PcaD active site and proposed a reaction mechanism. Proteins 2012;. (C) 2012 Wiley Periodicals, Inc. |
  | Date Added | 2/20/2014, 12:24:01 PM |
  | Modified | 2/20/2014, 12:24:01 PM |

  ### Notes:

  - Study a novel family of alpha/beta hydrolases.

    How SCOP is used:

    Look up families within superfamily of interest.

    SCOP reference:

    The a/b hydrolase fold is widely distributed in nature and the overall structure is highly conserved in evolution despite relatively low similarity on the sequence level. In the SCOP40 classification, there are 41 families within the a/b hydrolase superfamily, but proteins with new hydrolytic functions are being reported.

  ### Attachments

  - 24122\_ftp.pdf
- ## An aggregate analysis of many predicted structures to reduce errors in protein structure comparison caused by conformational flexibility

  |  |  |
  | --- | --- |
  | Type | Journal Article |
  | Author | Brian G. Godshall |
  | Author | Yisheng Tang |
  | Author | Wenjie Yang |
  | Author | Brian Y. Chen |
  | Volume | 13 |
  | Pages | S10 |
  | Publication | Bmc Structural Biology |
  | Date | November 2013 |
  | DOI | 10.1186/1472-6807-13-S1-S10 |
  | Abstract | Background: Conformational flexibility creates errors in the comparison of protein structures. Even small changes in backbone or sidechain conformation can radically alter the shape of ligand binding cavities. These changes can cause structure comparison programs to overlook functionally related proteins with remote evolutionary similarities, and cause others to incorrectly conclude that closely related proteins have different binding preferences, when their specificities are actually similar. Towards the latter effort, this paper applies protein structure prediction algorithms to enhance the classification of homologous proteins according to their binding preferences, despite radical conformational differences. Methods: Specifically, structure prediction algorithms can be used to "remodel" existing structures against the same template. This process can return proteins in very different conformations to similar, objectively comparable states. Operating on close homologs exploits the accuracy of structure predictions on closely related proteins, but structure prediction is often a nondeterministic process. Identical inputs can generate subtly different models with very different binding cavities that make structure comparison difficult. We present a first method to mitigate such errors, called "medial remodeling", that examines a large number of predicted structures to eliminate extreme models of the same binding cavity. Results: Our results, on the enolase and tyrosine kinase superfamilies, demonstrate that remodeling can enable proteins in very different conformations to be returned to states that can be objectively compared. Structures that would have been erroneously classified as having different binding preferences were often correctly classified after remodeling, while structures that would have been correctly classified as having different binding preferences almost always remained distinct. The enolase superfamily, which exhibited less sequential diversity than the tyrosine kinase superfamily, was classified more accurately after remodeling than the tyrosine kinases. Medial remodeling reduced errors from models with unusual perturbations that distort the shape of the binding site, enhancing classification accuracy. Conclusions: This paper demonstrates that protein structure prediction can compensate for conformational variety in the comparison of protein-ligand binding sites. While protein structure prediction introduces new uncertainties into the structure comparison problem, our results indicate that unusual models can be ignored through an analysis of many models, using techniques like medial remodeling. These results point to applications of protein structure comparison that extend beyond existing crystal structures. |
  | Date Added | 3/7/2014, 1:06:24 PM |
  | Modified | 3/7/2014, 1:06:24 PM |
- ## Analyses of the general rule on residue pair frequencies in local amino acid sequences of soluble, ordered proteins

  |  |  |
  | --- | --- |
  | Type | Journal Article |
  | Author | Matsuyuki Shirota |
  | Author | Kengo Kinoshita |
  | Volume | 22 |
  | Issue | 6 |
  | Pages | 725-733 |
  | Publication | PROTEIN SCIENCE |
  | ISSN | 0961-8368 |
  | Date | June 2013 |
  | DOI | 10.1002/pro.2255 |
  | Language | English |
  | Abstract | The amino acid sequences of soluble, ordered proteins with stable structures have evolved due to biological and physical requirements, thus distinguishing them from random sequences. Previous analyses have focused on extracting the features that frequently appear in protein substructures, such as -helix and -sheet, but the universal features of protein sequences have not been addressed. To clarify the differences between native protein sequences and random sequences, we analyzed 7368 soluble, ordered protein sequences, by inspecting the observed and expected occurrences of 400 amino acid pairs in local proximity, up to 10 residues along the sequence in comparison with their expected occurrence in random sequence. We found the trend that the hydrophobic residue pairs and the polar residue pairs are significantly decreased, whereas the pairs between a hydrophobic residue and a polar residue are increased. This trend was universally observed regardless of the secondary structure content but was not observed in protein sequences that include intrinsically disordered regions, indicating that it can be a general rule of protein foldability. The possible benefits of this rule are discussed from the viewpoints of protein aggregation and disorder, which are both caused by low-complexity regions of hydrophobic or polar residues. |
  | Date Added | 10/25/2013, 4:17:08 PM |
  | Modified | 10/25/2013, 4:17:08 PM |

  ### Tags:

  - protein disorder
  - protein structure
  - secondary structure
  - sequence analysis

  ### Notes:

  - Computational study of frequencies of amino acid pairings in SCOP domains.

    How SCOP is used:

    Get nonredundant data set from ASTRAL (cited) and do statistical analysis of co-occurrences of amino acid pairs.

    SCOP reference:

    Results and Discussion

    Data sets of protein sequences

    We downloaded 10,569 nonredundant amino acid sequences of protein domains from SCOP v1.75, in which the maximum sequence identity between any two sequences is below 40%.24 From these sequen- ces, we selected domains with structures solved by X-ray crystallography at a resolution better than 2.5 A ̊ , so as to focus on the amino acid sequences of ordered proteins. Membrane proteins, which were identified either by having the MeSH term “Membrane Protein” or by the SOSUI program,25 were excluded in order to focus on the sequence– structure relationship of soluble proteins. Our final dataset consisted of 7368 protein domains. From them, the amino acid sequences were obtained by reading the ATOM records, to exclude the regions without a stable structure. In addition, any short terminal sequences resembling His-tags were elimi- nated from the sequences. We referred to this data set as the “Ordered” set.

  ### Attachments

  - pro2255.pdf
- ## Analysis and consensus of currently available intrinsic protein disorder annotation sources in the MobiDB database

  |  |  |
  | --- | --- |
  | Type | Journal Article |
  | Author | Tomas Di Domenico |
  | Author | Ian Walsh |
  | Author | Silvio C. E. Tosatto |
  | Volume | 14 |
  | Pages | S3 |
  | Publication | Bmc Bioinformatics |
  | Date | April 2013 |
  | DOI | 10.1186/1471-2105-14-S7-S3 |
  | Abstract | Background: Intrinsic protein disorder is becoming an increasingly important topic in protein science. During the last few years, intrinsically disordered proteins (IDPs) have been shown to play a role in many important biological processes, e.g. protein signalling and regulation. This has sparked a need to better understand and characterize different types of IDPs, their functions and roles. Our recently published database, MobiDB, provides a centralized resource for accessing and analysing intrinsic protein disorder annotations. Results: Here, we present a thorough description and analysis of the data made available by MobiDB, providing descriptive statistics on the various available annotation sources. Version 1.2.1 of the database contains annotations for ca. 4,500,000 UniProt sequences, covering all eukaryotic proteomes. In addition, we describe a novel consensus annotation calculation and its related weighting scheme. The comparison between disorder information sources highlights how the MobiDB consensus captures the main features of intrinsic disorder and correlates well with manually curated datasets. Finally, we demonstrate the annotation of 13 eukaryotic model organisms through MobiDB's datasets, and of an example protein through the interactive user interface. Conclusions: MobiDB is a central resource for intrinsic disorder research, containing both experimental data and predictions. In the future it will be expanded to include additional information for all known proteins. |
  | Date Added | 3/7/2014, 12:08:00 PM |
  | Modified | 3/7/2014, 12:08:00 PM |
- ## Analysis of Conformational Variation in Macromolecular Structural Models

  |  |  |
  | --- | --- |
  | Type | Journal Article |
  | Author | Sandeep Kumar Srivastava |
  | Author | Savitha Gayathri |
  | Author | Babu A. Manjasetty |
  | Author | Balasubramanian Gopal |
  | Volume | 7 |
  | Issue | 7 |
  | Pages | e39993 |
  | Publication | Plos One |
  | Date | JUL 9 2012 |
  | Extra | WOS:000306354700022 |
  | DOI | 10.1371/journal.pone.0039993 |
  | Library Catalog | ISI Web of Knowledge |
  | Abstract | Experimental conditions or the presence of interacting components can lead to variations in the structural models of macromolecules. However, the role of these factors in conformational selection is often omitted by in silico methods to extract dynamic information from protein structural models. Structures of small peptides, considered building blocks for larger macromolecular structural models, can substantially differ in the context of a larger protein. This limitation is more evident in the case of modeling large multi-subunit macromolecular complexes using structures of the individual protein components. Here we report an analysis of variations in structural models of proteins with high sequence similarity. These models were analyzed for sequence features of the protein, the role of scaffolding segments including interacting proteins or affinity tags and the chemical components in the experimental conditions. Conformational features in these structural models could be rationalized by conformational selection events, perhaps induced by experimental conditions. This analysis was performed on a non-redundant dataset of protein structures from different SCOP classes. The sequence-conformation correlations that we note here suggest additional features that could be incorporated by in silico methods to extract dynamic information from protein structural models. |
  | Date Added | 10/8/2014, 12:49:22 PM |
  | Modified | 10/8/2014, 1:32:26 PM |

  ### Notes:

  - Study variations in structural models of proteins with high sequence similarity

    How SCOP is used:

    Collect a dataset of proteins from 5 structural classes in SCOP 1.73.

    SCOP reference:

    A compilation of protein structures was initially based on the SCOP (1.73 version) database. Upon the identification of candidate structural models, an advanced search in PDB was performed to obtain the corresponding protein structure deter- mined either in solution by NMR or as a part of a larger macromolecular complex. The following criteria were used to obtain the dataset for this analysis- i. Resolution cut-off for the X- ray crystal structures was set at 3.00 A ̊ (3.9 A ̊ in complexes) and ii. Only structures with a minimum overall sequence identity of 30% in a pair-wise alignment were selected. For this purpose, the EMBOSS Align program was used. PyMOL was used for the superposition of the structure pairs. The dataset of protein structural pairs had a total of 31 pairs of structures, belonging to five SCOP classes. The dataset for disordered proteins was collated from DISPROT [3]. The homologues for the disordered proteins for which PDB files were available were compiled from the PDB. The dataset for peptide structures were obtained from the PRF database within the DBGET integrated database retrieval system. In this search, the peptide length was limited to 10–40 amino acids. 110 peptide structures that contained only naturally- occurring amino acids were chosen for the study. Based on the availability of comparable sequences within large protein struc- tures, a dataset of 45 peptide structures were compiled.

  ### Attachments

  - PLoS Full Text PDF
- ## Analysis of Protein Folding using Structural Concealed Markov Model

  |  |  |
  | --- | --- |
  | Type | Journal Article |
  | Author | T. Kalai Chelvi |
  | Author | P. Rangarajan |
  | Pages | 92-97 |
  | Publication | 2013 Ieee International Conference on Smart Structures and Systems (icsss) |
  | Date | 2013 |
  | Extra | WOS:000332473600018 |
  | Library Catalog | ISI Web of Knowledge |
  | Abstract | Protein Structure Prediction (PSP) has significant applications in the fields of drug design, disease prediction and so on. Since PSP has been a great confrontation in the field of Protein Folding Research, this paper presents a novel method for protein using Structural Concealed Markov Model (SCMM). Typically, the contribution of this work has been made for appropriate mapping of protein primary structure to its 2D fold. Moreover, the model incorporates Extended Genetic Algorithm (EGA) for effectively folding the protein sequences that are having long chain lengths. The protein sequences are preprocessed, classified and then, analyzed with some parameters such as fitness, similarity and sequence gaps in order to form the optimal protein structures. The experimental results reveal the improved efficiency and accuracy of the proposed method with a performance analysis. |
  | Date Added | 10/8/2014, 12:49:22 PM |
  | Modified | 10/8/2014, 2:01:09 PM |

  ### Tags:

  - bioinformatics
  - classification
  - disease prediction
  - Drug Design
  - Educational institutions
  - EGA
  - extended genetic algorithm
  - Fitness Correlation
  - genetic algorithms
  - Genomics
  - High Dimensional Data
  - Markov processes
  - Optimization
  - pattern classification
  - Protein Folding
  - protein primary structure mapping
  - Proteins
  - protein sequences
  - protein structure prediction
  - SCMM
  - structural concealed Markov model
  - Testing
  - Training

  ### Attachments

  - IEEE Xplore Abstract Record
  - IEEE Xplore Full Text PDF
- ## Analyzing the effect of homogeneous frustration in protein folding

  |  |  |
  | --- | --- |
  | Type | Journal Article |
  | Author | V. G. Contessoto |
  | Author | D. T. Lima |
  | Author | R. J. Oliveira |
  | Author | A. T. Bruni |
  | Author | J. Chahine |
  | Author | V. B. P. Leite |
  | URL | http://onlinelibrary.wiley.com/doi/10.1002/prot.24309/abstract |
  | Publication | Proteins: Structure, Function, and Bioinformatics |
  | Date | 2013 |
  | Accessed | 9/23/2013, 10:15:34 AM |
  | Library Catalog | Google Scholar |
  | Date Added | 10/11/2013, 10:29:15 AM |
  | Modified | 2/25/2014, 12:14:17 PM |

  ### Tags:

  - C-alpha model
  - molecular dynamics
  - multivariate analysis
  - structure-based model

  ### Notes:

  - Computational study of protein folding and effects of frustration.

    How SCOP is used:

    Classified each protein in their 19-protein data set by its SCOP class.  Compared kinetics of the different classes.

    SCOP reference:

    With regard to protein motif, Figure 4 also shows the

    structural classification of proteins (SCOP) database cri-

    terion.60 Figure 4 has proteins belonging to the three

    different SCOP motifs: a (circles), a 1 b (diamonds),

    and b (triangles). In Figure 4, the blue delimited group

    with eopt50:0 (which we refer to as naturally optimized f

    protein) has only proteins with a-motif, and the red

    group with eopt > 0:0 (computationally optimized group)

    has the three protein motifs (a, a 1 b, and b). We could speculate, by inspecting these results, that in general, b proteins are those that could have their kinetics opti- mized by select mutations that create little energetic frus- tration. Evolution has selected a-proteins to be naturally optimized. a 1 b-Proteins could be the middle step in this evolutionary step and would require even less ener- getic frustration than b-proteins to have faster kinetics.

  ### Attachments

  - prot24309.pdf
- ## An Amino Acid Packing Code for alpha-Helical Structure and Protein Design

  |  |  |
  | --- | --- |
  | Type | Journal Article |
  | Author | Hyun Joo |
  | Author | Archana G. Chavan |
  | Author | Jamie Phan |
  | Author | Ryan Day |
  | Author | Jerry Tsai |
  | Volume | 419 |
  | Issue | 3-4 |
  | Pages | 234-254 |
  | Publication | JOURNAL OF MOLECULAR BIOLOGY |
  | ISSN | 0022-2836 |
  | Date | JUN 8 2012 |
  | DOI | 10.1016/j.jmb.2012.03.004 |
  | Language | English |
  | Abstract | This work demonstrates that all packing in alpha-helices can be simplified to repetitive patterns of a single motif: the knob-socket. Using the precision of Voronoi Polyhedra/Delauney Tessellations to identify contacts, the knob-socket is a four-residue tetrahedral motif: a knob residue on one alpha-helix packs into the three-residue socket on another alpha-helix. The principle of the knob-socket model relates the packing between levels of protein structure: the intra-helical packing arrangements within secondary structure that permit inter-helix tertiary packing interactions. Within an alpha-helix, the three-residue sockets arrange residues into a uniform packing lattice. Inter-helix packing results from a definable pattern of interdigitated knob-socket motifs between two alpha-helices. Furthermore, the knob-socket model classifies three types of sockets: (1) free, favoring only intra-helical packing; (2) filled, favoring inter-helical interactions; and (3) non, disfavoring alpha-helical structure. The amino acid propensities in these three socket classes essentially represent an amino acid code for structure in alpha-helical packing. Using this code, we used a novel yet straightforward approach for the design of alpha-helical structure to validate the knob-socket model. Unique sequences for three peptides were created to produce a predicted amount of alpha-helical structure: mostly helical, some helical, and no helix. These three peptides were synthesized, and helical content was assessed using CD spectroscopy. The measured alpha-helicity of each peptide was consistent with the expected predictions. These results and analysis demonstrate that the knob-socket motif functions as the basic unit of packing and presents an intuitive tool to decipher the rules governing packing in protein structure. (C) 2012 Elsevier Ltd. All rights reserved. |
  | Date Added | 10/25/2013, 4:17:08 PM |
  | Modified | 10/25/2013, 4:17:08 PM |

  ### Tags:

  - alpha-helix
  - protein design
  - protein structure
  - secondary structure packing
  - tertiary structure

  ### Notes:

  - Computational study of amino acid packing in a-helical structures.

    How SCOP is used:

    Use ASTRAL nonredundant data set of structures.  Compare packing in different SCOP classes.

    SCOP reference:

    Figure 6 displays relative probability histograms of 2240 combined XY·H sockets from an 8000 possible combinations that are either filled (Fig. 6a) or free (Fig. 6b) for all proteins in SCOP family (All), membrane proteins (Membrane), and coiled-coil proteins (Coiled coil).

    ...

    Heat maps for membrane proteins (Membrane) and coiled-coil proteins (Coiled-coil) are given for comparison along with those from all SCOP family proteins (All).

    ...

    In the development of the knob–socket model, RPCs were identified in all 15,273 domains in the ASTRAL SCOP 1.75 set of structures filtered at 95% sequence identity122 only between residues that are defined α-helical by DSSP.123

  ### Attachments

  - 1-s2.0-S0022283612002598-main.pdf
- ## An artificial neural network approach to improving the correlation between protein energetics and the backbone structure

  |  |  |
  | --- | --- |
  | Type | Journal Article |
  | Author | Timothy M. Fawcett |
  | Author | Stephanie J. Irausquin |
  | Author | Mikhail Simin |
  | Author | Homayoun Valafar |
  | URL | http://onlinelibrary.wiley.com/doi/10.1002/pmic.201200330/full |
  | Volume | 13 |
  | Issue | 2 |
  | Pages | 230–238 |
  | Publication | Proteomics |
  | Date | 2013 |
  | Accessed | 9/20/2013, 1:20:11 PM |
  | Library Catalog | Google Scholar |
  | Date Added | 10/11/2013, 10:29:15 AM |
  | Modified | 3/7/2014, 1:06:50 PM |

  ### Tags:

  - Artificial neural network
  - bioinformatics
  - Hydrogen bonding
  - Protein energetics
  - protein structure prediction
  - protein structure refinement

  ### Notes:

  - The paper details "new approach in evaluation of protein structures based on analysis of energy profiles

    produced by the SCOPE software package."

    SCOP/CATH Use

    Provide approximate number of folds in SCOP and CATH (~1500).

    SCOP Reference

     However, the Protein Data Bank  
    (PDB) [2] contains approximately 1 500-fold families as reported by CATH [3] or SCOP [4].

  ### Attachments

  - pmic7307.pdf
  - Snapshot
- ## An estimated 5% of new protein structures solved today represent a new Pfam family

  |  |  |
  | --- | --- |
  | Type | Journal Article |
  | Author | Jaina Mistry |
  | Author | Edda Kloppmann |
  | Author | Burkhard Rost |
  | Author | Marco Punta |
  | Volume | 69 |
  | Pages | 2186-2193 |
  | Publication | Acta Crystallographica Section D-Biological Crystallography |
  | ISSN | 0907-4449; 1399-0047 |
  | Date | NOV 2013 |
  | Extra | WOS:000326648900004 |
  | DOI | 10.1107/S0907444913027157 |
  | Abstract | High-resolution structural knowledge is key to understanding how proteins function at the molecular level. The number of entries in the Protein Data Bank (PDB), the repository of all publicly available protein structures, continues to increase, with more than 8000 structures released in 2012 alone. The authors of this article have studied how structural coverage of the protein-sequence space has changed over time by monitoring the number of Pfam families that acquired their first representative structure each year from 1976 to 2012. Twenty years ago, for every 100 new PDB entries released, an estimated 20 Pfam families acquired their first structure. By 2012, this decreased to only about five families per 100 structures. The reasons behind the slower pace at which previously uncharacterized families are being structurally covered were investigated. It was found that although more than 50% of current Pfam families are still without a structural representative, this set is enriched in families that are small, functionally uncharacterized or rich in problem features such as intrinsically disordered and transmembrane regions. While these are important constraints, the reasons why it may not yet be time to give up the pursuit of a targeted but more comprehensive structural coverage of the protein-sequence space are discussed. |
  | Date Added | 2/20/2014, 12:24:01 PM |
  | Modified | 2/20/2014, 12:24:01 PM |

  ### Tags:

  - coverage

  ### Notes:

  - Assess growth of structural coverage of Pfam familiies.

    How SCOP and CATH are used:

    Mention that SCOP or CATH could be used to assess structural coverage, but they suffer from two shortcomings.  First, many recent structures have not been added to the databases, and second, they only cover families for which structures that have been solved.  Instead choose to use Pfam.

    SCOP reference:

    4. Analysis of PDB structures: from individual sequences to families

    In order to better understand what the numbers reported in Fig. 1 mean in terms of progress towards more complete structural coverage of the protein sequence space, we considered PDB entries in the context of protein-sequence families (i.e. sets of homologous protein regions) and measured the increase in the number of families that are being structurally covered (i.e. that have at least one member with a known experimental structure). For this purpose we could use, in principle, the structure-based classification systems provided by SCOP (Andreeva et al., 2008) or CATH (Orengo et al., 1998). Using these resources, however, presents two problems. The first is that many of the structures released in recent years have not yet been included in the latest versions of SCOP and CATH (SCOP 1.75 and CATH v.3.5). The second is that by definition these databases only classify proteins for which structures have been solved. This means that they cannot provide us with any information on the number of protein families that are yet to be structurally characterized. To partially overcome these shortcomings, we decided to use the manually curated, mostly sequence-based Pfam database of protein families (Punta et al., 2012). Pfam provides a higher coverage of PDB structures than either CATH or SCOP, and attempts to classify all protein regions, regardless of whether they fall into a family that contains a member whose structure has been characterized.

  ### Attachments

  - ba5211.pdf
- ## A new family of proteins related to the HEAT-like repeat DNA glycosylases with affinity for branched DNA structures

  |  |  |
  | --- | --- |
  | Type | Journal Article |
  | Author | Paul H. Backe |
  | Author | Roger Simm |
  | Author | Jon K. Laerdahl |
  | Author | Bjorn Dalhus |
  | Author | Annette Fagerlund |
  | Author | Ole A. Okstad |
  | Author | Torbjorn Rognes |
  | Author | Ingrun Alseth |
  | Author | Anne-Brit Kolsto |
  | Author | Magnar Bjoras |
  | Volume | 183 |
  | Issue | 1 |
  | Pages | 66-75 |
  | Publication | Journal of Structural Biology |
  | ISSN | 1047-8477 |
  | Date | JUL 2013 |
  | Extra | WOS:000321993700008 |
  | DOI | 10.1016/j.jsb.2013.04.007 |
  | Abstract | The recently discovered HEAT-like repeat (HLR) DNA glycosylase superfamily is widely distributed in all domains of life. The present bioinformatics and phylogenetic analysis shows that HLR DNA glycosylase superfamily members in the genus Bacillus form three subfamilies: AlkC, AlkD and AlkF/AlkG. The crystal structure of AlkF shows structural similarity with the DNA glycosylases AlkC and AlkD, however neither AlkF nor AlkG display any DNA glycosylase activity. Instead, both proteins have affinity to branched DNA structures such as three-way and Holliday junctions. A unique a-hairpin in the AlkF/AlkG subfamily is most likely inserted into the DNA major groove, and could be a structural determinant regulating DNA substrate affinity. We conclude that AlkF and AlkG represent a new family of HLR proteins with affinity for branched DNA structures. (C) 2013 The Authors. Published by Elsevier Inc. All rights reserved. |
  | Date Added | 10/28/2013, 4:51:00 PM |
  | Modified | 3/7/2014, 12:10:17 PM |

  ### Notes:

  - Present a study of new DNA-glyocosylases.

    How SCOP/CATH is used:

    Background on protein structure classification.

    SCOP/CATH reference:

    4. Discussion

    We have previously shown that AlkD and AlkC are single do- main DNA glycosylases belonging to a new, fifth structural super- family of DNA glycosylases (Alseth et al., 2006; Dalhus et al., 2007). It is generally accepted that the 3D structure is more conserved than sequence in distantly related proteins. Protein domains with significant sequence similarity, usually better than roughly 30% se- quence identity, are classified as belonging to the same protein do- main family. Protein domains that have very low or insignificant sequence similarity, but still clearly are evolutonary related based on 3D structure and functional features, are classified in the same protein domain superfamily. **This protein domain classification scheme is for example employed in the most widely used domain classification hierarchies, SCOP (****Andreeva et al., 2008) and CATH (Sillitoe et al., 2013****), where a major fraction of the domain super- families comprises several families.**

  ### Attachments

  - 1-s2.0-S104784771300107X-main.pdf
- ## A new size-independent score for pairwise protein structure alignment and its application to structure classification and nucleic-acid binding prediction

  |  |  |
  | --- | --- |
  | Type | Journal Article |
  | Author | Yuedong Yang |
  | Author | Jian Zhan |
  | Author | Huiying Zhao |
  | Author | Yaoqi Zhou |
  | Volume | 80 |
  | Issue | 8 |
  | Pages | 2080-2088 |
  | Publication | Proteins: Structure, Function, and Bioinformatics |
  | ISSN | 0887-3585 |
  | Date | AUG 2012 |
  | Extra | WOS:000306132400015 |
  | DOI | 10.1002/prot.24100 |
  | Abstract | A structure alignment program aligns two structures by optimizing a scoring function that measures structural similarity. It is highly desirable that such scoring function is independent of the sizes of proteins in comparison so that the significance of alignment across different sizes of the protein regions aligned is comparable. Here, we developed a new score called SP-score that fixes the cutoff distance at 4 angstrom and removed the size dependence using a normalization prefactor. We further built a program called SPalign that optimizes SP-score for structure alignment. SPalign was applied to recognize proteins within the same structure fold and having the same function of DNA or RNA binding. For fold discrimination, SPalign improves sensitivity over TMalign for the chain-level comparison by 12% and over DALI for the domain-level comparison by 13% at the same specificity of 99.6%. The difference between TMalign and SPalign at the chain level is due to the inability of TMalign to detect single domain similarity between multidomain proteins. For recognizing nucleic acid binding proteins, SPalign consistently improves over TMalign by 12% and DALI by 31% in average value of Mathews correlation coefficients for four datasets. SPalign with default setting is 14% faster than TMalign. SPalign is expected to be useful for function prediction and comparing structures with or without domains defined. The source code for SPalign and the server are available at . Proteins 2012;. (c) 2012 Wiley Periodicals, Inc. |
  | Date Added | 2/20/2014, 12:24:01 PM |
  | Modified | 5/5/2014, 3:11:59 PM |

  ### Notes:

  - Present scoring method for structure alignment.

    How SCOP is used:

    Benchmark method on SCOP-derived  domain dataset.  Validate on fold classification.

    How CATH is used:

    CATH data is not used.

    SCOP reference:

    Here, we propose to remove the size dependence not by size-dependent d0 but by a size-dependent normalization factor. This allows us to introduce an effective alignment length that removes the need to specify a length for nor- malization. The new score with its alignment program SPalign is tested in structure classification and prediction of nucleic-acid binding proteins and compared to DALI,26 CE,19 TMalign,25 and FrTMalign.27 For recognizing structures the same SCOP fold (SCOP: Structure Classifi- cation Of Proteins), SPalign is significantly more sensitive (> 10%) in fold recognition than TMalign for chain– chain comparison and DALI for domain–domain compar- ison at the same specificity and similar in performance to TMalign for domain–domain comparison and DALI for chain–chain comparison. For predicting DNA/RNA-bind- ing proteins, SPalign consistently improves over DALI and TM-score at both chain and domain levels.

    METHODS Datasets  
     SCOP: SCOP domain dataset

    We used the dataset SCOP-20 that was used as a benchmark for testing the fold recognition program SPARKS X.28 The dataset was built using domains of sequence identity less than 20% and chain lengths greater

    than 60 from SCOP 1.75.12 After removing domains with Ca atoms only, we obtained 6367 domains.

    SCOPc: SCOP chain dataset

    To further test our scoring function with multidomain proteins, nonredundant chains for all domains contained in the SCOP-20 dataset are collected. There are a total of 5300 chains. We define that two chains are considered to be similar in structure if a domain in one chain belongs to the same fold of another domain in the other chain. This chain-level comparison is a real-world test because domains are often not defined for most newly solved structures.

    rSCOP and rSCOPc datasets

    To compare with slower structure alignment methods, we randomly chose 1058 and 1060 proteins from SCOP (rSCOP) and SCOPc (rSCOPc) datasets, respectively.

     CATH reference (11):

    Moreover, auto- matic structural comparison is complementary to manual protein structure classification11,12 that lags far behind the pace of newly determined structures due to structural genomics projects.13

  ### Attachments

  - 24100\_ftp.pdf
- ## Anisotropy of fluctuation dynamics of proteins with an elastic network model

  |  |  |
  | --- | --- |
  | Type | Journal Article |
  | Author | A R Atilgan |
  | Author | S R Durell |
  | Author | R L Jernigan |
  | Author | M C Demirel |
  | Author | O Keskin |
  | Author | I Bahar |
  | Volume | 80 |
  | Issue | 1 |
  | Pages | 505-515 |
  | Publication | Biophysical journal |
  | ISSN | 0006-3495 |
  | Date | Jan 2001 |
  | Extra | PMID: 11159421 |
  | Journal Abbr | Biophys. J. |
  | DOI | 10.1016/S0006-3495(01)76033-X |
  | Library Catalog | NCBI PubMed |
  | Language | eng |
  | Abstract | Fluctuations about the native conformation of proteins have proven to be suitably reproduced with a simple elastic network model, which has shown excellent agreement with a number of different properties for a wide variety of proteins. This scalar model simply investigates the magnitudes of motion of individual residues in the structure. To use the elastic model approach further for developing the details of protein mechanisms, it becomes essential to expand this model to include the added details of the directions of individual residue fluctuations. In this paper a new tool is presented for this purpose and applied to the retinol-binding protein, which indicates enhanced flexibility in the region of entry to the ligand binding site and for the portion of the protein binding to its carrier protein. |
  | Date Added | 10/11/2013, 10:29:15 AM |
  | Modified | 10/11/2013, 10:29:15 AM |

  ### Notes:

  - Present an elastic network model that is expanded to include direction of residue fluctuations.  Use the model to investigate dynamics of retinol binding protein.

    How SCOP is used:

    SCOP website.  To add extra details about a protein of interest.

    SCOP reference:

    It [pig plasma retinol binding protein] belongs to the super-family of lipocalins, beta-class proteins that bind hydrophobic ligands in their interior (Murzin et al., 1995).

    |  |
    | --- |
    |  |

  ### Attachments

  - PubMed entry
  - ScienceDirect Full Text PDF
  - ScienceDirect Snapshot
- ## An octamer of enolase from Streptococcus suis

  |  |  |
  | --- | --- |
  | Type | Journal Article |
  | Author | Qiong Lu |
  | Author | Hao Lu |
  | Author | Jianxun Qi |
  | Author | Guangwen Lu |
  | Author | George F. Gao |
  | Volume | 3 |
  | Issue | 10 |
  | Pages | 769–780 |
  | Publication | Protein & Cell |
  | Date | October 2012 |
  | DOI | 10.1007/s13238-012-2040-7 |
  | Abstract | Enolase is a conserved cytoplasmic metalloenzyme existing universally in both eukaryotic and prokaryotic cells. The enzyme can also locate on the cell surface and bind to plasminogen, via which contributing to the mucosal surface localization of the bacterial pathogens and assisting the invasion into the host cells. The functions of the eukaryotic enzymes on the cell surface expression (including T cells, B cells, neutrophils, monocytoes, neuronal cells and epithelial cells) are not known. Streptococcus suis serotype 2 (S. suis 2, SS2) is an important zoonotic pathogen which has recently caused two large-scale outbreaks in southern China with severe streptococcal toxic shock syndrome (STSS) never seen before in human sufferers. We recently identified the SS2 enolase as an important protective antigen which could protect mice from fatal S. suis 2 infection. In this study, a 2.4-angstrom structure of the SS2 enolase is solved, revealing an octameric arrangement in the crystal. We further demonstrated that the enzyme exists exclusively as an octamer in solution via a sedimentation assay. These results indicate that the octamer is the biological unit of SS2 enolase at least in vitro and most likely in vivo as well. This is, to our knowledge, the first comprehensive characterization of the SS2 enolase octamer both structurally and biophysically, and the second octamer enolase structure in addition to that of Streptococcus pneumoniae. We also investigated the plasminogen binding property of the SS2 enzyme. |
  | Date Added | 3/7/2014, 1:06:24 PM |
  | Modified | 3/7/2014, 1:06:24 PM |
- ## A novel algorithm combining support vector machine with the discrete wavelet transform for the prediction of protein subcellular localization

  |  |  |
  | --- | --- |
  | Type | Journal Article |
  | Author | Ru-Ping Liang |
  | Author | Shu-Yun Huang |
  | Author | Shao-Ping Shi |
  | Author | Xing-Yu Sun |
  | Author | Sheng-Bao Suo |
  | Author | Jian-Ding Qiu |
  | Volume | 42 |
  | Issue | 2 |
  | Pages | 180–187 |
  | Publication | Computers In Biology and Medicine |
  | Date | February 2012 |
  | DOI | 10.1016/j.compbiomed.2011.11.006 |
  | Abstract | Knowing the subcellular localization of proteins within the cell is an important step in elucidating its role in biological processes, its function and its potential as a drug target for disease diagnosis. As the number of complete genomes rapidly increases, accurate and efficient methods that automatically predict the subcellular localizations become more urgent. In the current paper, we developed a novel method that coupled the discrete wavelet transform with support vector machine based on the amino acid polarity to predict the subcellular localizations of prokaryotic and eukaryotic proteins. The results obtained by the jackknife test were quite promising, and indicated that the proposed method remarkably improved the prediction accuracy of subcellular locations, and could be as an effective and promising high-throughput method in the subcellular localization research. (C) 2011 Elsevier Ltd. All rights reserved. |
  | Date Added | 3/7/2014, 1:06:24 PM |
  | Modified | 3/7/2014, 1:06:24 PM |
- ## A novel neural response algorithm for protein function prediction

  |  |  |
  | --- | --- |
  | Type | Journal Article |
  | Author | Hari K. Yalamanchili |
  | Author | Quan-Wu Xiao |
  | Author | Junwen Wang |
  | URL | http://www.biomedcentral.com/1752-0509/6/S1/S19/ |
  | Volume | 6 |
  | Issue | Suppl 1 |
  | Pages | S19 |
  | Publication | BMC systems biology |
  | Date | 2012 |
  | Accessed | 9/23/2013, 10:19:41 AM |
  | Library Catalog | Google Scholar |
  | Date Added | 10/11/2013, 10:29:15 AM |
  | Modified | 3/7/2014, 12:10:27 PM |

  ### Notes:

  - Present new method for protein function prediction.

    How SCOP/CATH is used:

    Provide background on use of structural databases for function prediction.

    SCOP/CATH reference:

    Protein function assignment methods can be divided into two main categories - structure-based methods and sequence-based methods. A protein’s function is highly related to its structure. Protein structure tends to be more conserved than the amino acid sequence in the course of evolution [12,13]. Thus a variety of structure- based function prediction methods [14,15] rely on struc- ture similarities. These methods start with a predicted structure of the query protein and search for similar structural motifs in various structural classification data- bases such as CATH [16] and SCOP [17] for function prediction.

  ### Attachments

  - 1752-0509-6-S1-S19.pdf
- ## A novel protein structural classes prediction method based on predicted secondary structure

  |  |  |
  | --- | --- |
  | Type | Journal Article |
  | Author | Shuyan Ding |
  | Author | Shengli Zhang |
  | Author | Yang Li |
  | Author | Tianming Wang |
  | Volume | 94 |
  | Issue | 5 |
  | Pages | 1166-1171 |
  | Publication | Biochimie |
  | ISSN | 0300-9084 |
  | Date | May 2012 |
  | DOI | 10.1016/j.biochi.2012.01.022 |
  | Language | English |
  | Abstract | Knowledge of structural classes plays an important role in understanding protein folding patterns. In this paper, features based on the predicted secondary structure sequence and the corresponding E-H sequence are extracted. Then, an 11-dimensional feature vector is selected based on a wrapper feature selection algorithm and a support vector machine (SVM). Among the 11 selected features, 4 novel features are newly designed to model the differences between alpha/beta class and alpha + beta class, and other 7 rational features are proposed by previous researchers. To examine the performance of our method, a total of 5 datasets are used to design and test the proposed method. The results show that competitive prediction accuracies can be achieved by the proposed method compared to existing methods (SCPRED, RKS-PPSC and MODAS), and 4 new features are demonstrated essential to differentiate alpha/beta and alpha + beta classes. Standalone version of the proposed method is written in JAVA language and it can be downloaded from http://web.xidian.edu.cn/slzhang/paper.html. (C) 2012 Elsevier Masson SAS. All rights reserved. |
  | Date Added | 10/25/2013, 4:29:01 PM |
  | Modified | 3/6/2014, 4:05:00 PM |

  ### Tags:

  - feature selection
  - Protein structural classes
  - support vector machine

  ### Notes:

  - Present method for structural class prediction.

    How SCOP is used:

    Train and validate method for class prediction on ASTRAL domain representative set with <20% sequence similarity.

    SCOP reference:

    2. Materials and methods

    2.1. Materials

    A total of 5 datasets were used to design and test the new method. The ASTRAL database (version 1.73) was utilized, which is a subset of SCOP database characterized by a certain similarity threshold [30]. The ASTRAL database (including 7 classes) selected has sequence similarity lower than 20% which contains 6424 sequences [19]. In this study, only four major classes (all-a, all-b, a/ b and a þ b) that includes 5626 sequences were used. The dataset was randomly divided into two equal subsets, one was used as the training set (ASTRALtraining) and the second was used as the test set (ASTRALtest). Both of these datasets are available at http://web. xidian.edu.cn/slzhang/paper.html.

  ### Attachments

  - 1-s2.0-S0300908412000405-main.pdf
- ## A novel web server predicts amino acid residue protection against hydrogen–deuterium exchange

  |  |  |
  | --- | --- |
  | Type | Journal Article |
  | Author | Mikhail Yu Lobanov |
  | Author | Masha Yu Suvorina |
  | Author | Nikita V. Dovidchenko |
  | Author | Igor V. Sokolovskiy |
  | Author | Alexey K. Surin |
  | Author | Oxana V. Galzitskaya |
  | URL | http://bioinformatics.oxfordjournals.org/content/29/11/1375.short |
  | Volume | 29 |
  | Issue | 11 |
  | Pages | 1375–1381 |
  | Publication | Bioinformatics |
  | Date | 2013 |
  | Accessed | 9/23/2013, 10:14:50 AM |
  | Library Catalog | Google Scholar |
  | Date Added | 10/11/2013, 10:29:15 AM |
  | Modified | 2/24/2014, 4:19:55 PM |

  ### Notes:

  - Present a method to predict "the degree of protection" of particular residues from HD-exchange experiments, based on sequence alone.

    How using SCOP:

    Use a previously published database of 3769 proteins with HD exchange data.  In order to validate that the data set has good coverage, they have checked that it contains proteins that belong to all 4 SCOP classes, with <25% sequence identity.

    SCOP reference:

    The database contained proteins that belong to four main structural classification of proteins (SCOP) (Murzin et al., 1995) classes (classes a, b, c and d with all-⬚⬚, all-⬚⬚, ⬚⬚/⬚⬚ and ⬚⬚ þ ⬚⬚ proteins, respect- ively). The proteins had 525% sequence identity to one another.

  ### Attachments

  - Full Text PDF
  - PubMed entry
  - Snapshot
- ## A novel web server predicts amino acid residue protection against hydrogen–deuterium exchange

  |  |  |
  | --- | --- |
  | Type | Journal Article |
  | Author | Mikhail Yu Lobanov |
  | Author | Masha Yu Suvorina |
  | Author | Nikita V. Dovidchenko |
  | Author | Igor V. Sokolovskiy |
  | Author | Alexey K. Surin |
  | Author | Oxana V. Galzitskaya |
  | URL | http://bioinformatics.oxfordjournals.org/content/29/11/1375 |
  | Volume | 29 |
  | Issue | 11 |
  | Pages | 1375-1381 |
  | Publication | Bioinformatics |
  | ISSN | 1367-4803, 1460-2059 |
  | Date | 06/01/2013 |
  | Extra | PMID: 23620358 |
  | Journal Abbr | Bioinformatics |
  | DOI | 10.1093/bioinformatics/btt168 |
  | Accessed | 4/12/2015, 5:00:27 PM |
  | Library Catalog | bioinformatics.oxfordjournals.org |
  | Language | en |
  | Abstract | Motivation: To clarify the relationship between structural elements and polypeptide chain mobility, a set of statistical analyses of structures is necessary. Because at present proteins with determined spatial structures are much less numerous than those with amino acid sequence known, it is important to be able to predict the extent of proton protection from hydrogen–deuterium (HD) exchange basing solely on the protein primary structure. Results: Here we present a novel web server aimed to predict the degree of amino acid residue protection against HD exchange solely from the primary structure of the protein chain under study. On the basis of the amino acid sequence, the presented server offers the following three possibilities (predictors) for user’s choice. First, prediction of the number of contacts occurring in this protein, which is shown to be helpful in estimating the number of protons protected against HD exchange (sensitivity 0.71). Second, probability of H-bonding in this protein, which is useful for finding the number of unprotected protons (specificity 0.71). The last is the use of an artificial predictor. Also, we report on mass spectrometry analysis of HD exchange that has been first applied to free amino acids. Its results showed a good agreement with theoretical data (number of protons) for 10 globular proteins (correlation coefficient 0.73). We pioneered in compiling two datasets of experimental HD exchange data for 35 proteins. Availability: The H-Protection server is available for users at http://bioinfo.protres.ru/ogp/ Contact: ogalzit@vega.protres.ru Supplementary information: Supplementary data are available at Bioinformatics online. |
  | Date Added | 4/12/2015, 5:00:27 PM |
  | Modified | 4/12/2015, 5:00:27 PM |

  ### Attachments

  - Full Text PDF
  - PubMed entry
  - Snapshot
- ## Anti-viral immune responses in a primitive lung: Characterization and expression analysis of interferon-inducible immunoproteasome subunits LMP2, LMP7 and MECL-1 in a sarcopterygian fish, the Nigerian spotted lungfish (Protopterus dolloi)

  |  |  |
  | --- | --- |
  | Type | Journal Article |
  | Author | Luca Tacchi |
  | Author | Milind Misra |
  | Author | Irene Salinas |
  | Volume | 41 |
  | Issue | 4 |
  | Pages | 657-665 |
  | Publication | Developmental and Comparative Immunology |
  | ISSN | 0145-305X; 1879-0089 |
  | Date | DEC 2013 |
  | Extra | WOS:000326258500022 |
  | DOI | 10.1016/j.dci.2013.07.023 |
  | Abstract | Lungfishes (Dipnoi) represent the closest ancestor of tetrapods. Dipnoi have dual breathing modes extracting oxygen from water and air. The primitive lungs of lungfishes are exposed to external antigens including viruses. To date, the immune response of lungfishes against viruses has not been investigated. During viral immune responses, cell exposure to type I interferon induces the replacement of the constitutive proteasome with LMP2, LMP7 and MECL-1 beta subunits forming the immunoproteasome and enhancing antigen presentation to MHC class I molecules. In order to study the immune defense system of the lungfish lung, we have characterized for the first time the three immunoproteasome subunits in the sarcopterygian fish, the Nigerian spotted lungfish (Protopterus dolloi). LMP2, LMP7 and MECL-1 were identified in P. dolloi and their sequences encoded predicted proteins of 216, 275 and 278 amino acids, respectively. The mRNA of these three genes was expressed in multiple tissues, including the lung, with the highest abundance observed in kidney and post-pyloric spleen. In vitro stimulation of lungfish lung and kidney primary cell cultures with PolyI:C for 4 and 12 h resulted in increased LMP2, LMP7 and MECL-1 expression in both tissues. These results suggest a central role of these genes in the activation of an antiviral immune response in lungfish. Importantly, they indicate that the primitive lung of the common ancestor of all tetrapods is capable of inducing the expression of these genes in response to viral stimulation. (C) 2013 Elsevier Ltd. All rights reserved. |
  | Date Added | 2/12/2014, 1:36:22 PM |
  | Modified | 2/12/2014, 1:36:22 PM |

  ### Notes:

  - Experimental and computational study of immune response in lungfishes.

    How SCOP is used:

    Use SUPERFAMILY to get SCOP domains and superfamily and family classification of data set of immunoproteasome subunits.

    SCOP reference:

    2.4. Sequence analysis

    HMM (hidden Markov model) analysis was performed and six- frame translations of the sequences in the 454 reads database were scanned with HMMER version 3.1b1 (http://hmmer.org) against SUPERFAMILY version 1.75 hidden Markov models (Gough et al., 2001). SCOP (Murzin et al., 1995) superfamily, family and domain assignments were also carried out.

  ### Attachments

  - 1-s2.0-S0145305X13002127-main.pdf
- ## A pharmacological organization of G protein-coupled receptors

  |  |  |
  | --- | --- |
  | Type | Journal Article |
  | Author | Henry Lin |
  | Author | Maria F. Sassano |
  | Author | Bryan L. Roth |
  | Author | Brian K. Shoichet |
  | Volume | 10 |
  | Issue | 2 |
  | Pages | 140-146 |
  | Publication | Nature Methods |
  | ISSN | 1548-7091 |
  | Date | FEB 2013 |
  | Extra | WOS:000314623900020 |
  | DOI | 10.1038/NMETH.2324 |
  | Abstract | Protein classification typically uses structural, sequence or functional similarity. Here we introduce an orthogonal method that organizes proteins by ligand similarity, focusing on the class A G-protein-coupled receptor (GPCR) protein family. Comparing a ligand-based dendrogram to a sequence-based one, we identified GPCRs that were distantly linked by sequence but were neighbors by ligand similarity. Experimental testing of the ligands predicted to link three of these new pairs confirmed the predicted association, with potencies ranging from low nanomolar to low micromolar. We also predicted hundreds of non-GPCRs closely related to GPCRs by ligand similarity and confirmed several cases experimentally. Ligand similarities among these targets may reflect the conservation of identical ligands among unrelated receptors, which signal in different time domains. Our method integrates these apparently disparate receptors into chemically coherent circuits and suggests which of these receptors may be targeted by individual ligands. |
  | Date Added | 2/13/2014, 4:13:17 PM |
  | Modified | 3/7/2014, 12:09:20 PM |
- ## APoc: large-scale identification of similar protein pockets

  |  |  |
  | --- | --- |
  | Type | Journal Article |
  | Author | Mu Gao |
  | Author | Jeffrey Skolnick |
  | URL | http://bioinformatics.oxfordjournals.org/content/29/5/597 |
  | Volume | 29 |
  | Issue | 5 |
  | Pages | 597-604 |
  | Publication | Bioinformatics |
  | ISSN | 1367-4803, 1460-2059 |
  | Date | 03/01/2013 |
  | Extra | PMID: 23335017 |
  | Journal Abbr | Bioinformatics |
  | DOI | 10.1093/bioinformatics/btt024 |
  | Accessed | 12/9/2014, 6:05:24 AM |
  | Library Catalog | bioinformatics.oxfordjournals.org |
  | Language | en |
  | Abstract | Motivation: Most proteins interact with small-molecule ligands such as metabolites or drug compounds. Over the past several decades, many of these interactions have been captured in high-resolution atomic structures. From a geometric point of view, most interaction sites for grasping these small-molecule ligands, as revealed in these structures, form concave shapes, or ‘pockets’, on the protein’s surface. An efficient method for comparing these pockets could greatly assist the classification of ligand-binding sites, prediction of protein molecular function and design of novel drug compounds. Results: We introduce a computational method, APoc (Alignment of Pockets), for the large-scale, sequence order-independent, structural comparison of protein pockets. A scoring function, the Pocket Similarity Score (PS-score), is derived to measure the level of similarity between pockets. Statistical models are used to estimate the significance of the PS-score based on millions of comparisons of randomly related pockets. APoc is a general robust method that may be applied to pockets identified by various approaches, such as ligand-binding sites as observed in experimental complex structures, or predicted pockets identified by a pocket-detection method. Finally, we curate large benchmark datasets to evaluate the performance of APoc and present interesting examples to demonstrate the usefulness of the method. We also demonstrate that APoc has better performance than the geometric hashing-based method SiteEngine. Availability and implementation: The APoc software package including the source code is freely available at http://cssb.biology.gatech.edu/APoc. Contact: skolnick@gatech.edu Supplementary information: Supplementary data are available at Bioinformatics online. |
  | Short Title | APoc |
  | Date Added | 12/9/2014, 6:05:24 AM |
  | Modified | 12/9/2014, 6:05:24 AM |

  ### Notes:

  - How SCOP is used:

    Look up fold of two domains.

    SCOP reference:

    The ATP-binding pocket of GspS is located in the C-terminal domain, with a structural fold similar to human glutathione synthetase, whereas the ATP-binding pocket of AphA1 sits in a structural fold similar to the catalytic domain of a protein kinase. These are different structural folds according to the SCOP (Hubbard et al., 1998).

  ### Attachments

  - Full Text PDF
- ## Applications of liquid chromatography-mass spectrometry for food analysis

  |  |  |
  | --- | --- |
  | Type | Journal Article |
  | Author | Vita Di Stefano |
  | Author | Giuseppe Avellone |
  | Author | David Bongiorno |
  | Author | Vincenzo Cunsolo |
  | Author | Vera Muccilli |
  | Author | Stefano Sforza |
  | Author | Arnaldo Dossena |
  | Author | Laszlo Drahos |
  | Author | Karoly Vekey |
  | Volume | 1259 |
  | Pages | 74-85 |
  | Publication | Journal of Chromatography A |
  | ISSN | 0021-9673 |
  | Date | OCT 12 2012 |
  | Extra | WOS:000309566500006 |
  | DOI | 10.1016/j.chroma.2012.04.023 |
  | Abstract | HPLC-MS applications in the agrifood sector are among the fastest developing fields in science and industry. The present tutorial mini-review briefly describes this analytical methodology: HPLC, UHPLC, nano-HPLC on one hand, mass spectrometry (MS) and tandem mass spectrometry (MS/MS) on the other hand. Analytical results are grouped together based on the type of chemicals analyzed (lipids, carbohydrates, glycoproteins, vitamins, flavonoids, mycotoxins, pesticides, allergens and food additives). Results are also shown for various types of food (ham, cheese, milk, cereals, olive oil and wines). Although it is not an exhaustive list, it illustrates the main current directions of applications. Finally, one of the most important features, the characterization of food quality (including problems of authentication and adulteration) is discussed, together with a future outlook on future directions. (c) 2012 Elsevier B.V. All rights reserved. |
  | Date Added | 10/28/2013, 4:57:32 PM |
  | Modified | 10/28/2013, 4:57:32 PM |

  ### Notes:

  - Review of applications of liquid chromatography-mass spec for food analysis.

    How SCOP is used:

    Background on protein structure classification.

    SCOP reference:

    4.4. Cereals

    Cereals, including rice, barley and wheat, are the major crops of the global food supply, dominating world agriculture. Func- tional and nutritional properties of cereals depend largely on their protein pattern. A range of criteria [128,129] has been used to define and classify cereal proteins. The most often used classification at present subdivides cereal proteins into fami- lies/superfamilies.

  ### Attachments

  - 1-s2.0-S0021967312005808-main.pdf
- ## A Protein Block Based Fold Recognition Method for the Annotation of Twilight Zone Sequences

  |  |  |
  | --- | --- |
  | Type | Journal Article |
  | Author | V. Suresh |
  | Author | K. Ganesan |
  | Author | S. Parthasarathy |
  | URL | http://www.ingentaconnect.com/content/ben/ppl/2013/00000020/00000003/art00003 |
  | Volume | 20 |
  | Issue | 3 |
  | Pages | 249–254 |
  | Publication | Protein and peptide letters |
  | Date | 2013 |
  | Accessed | 9/23/2013, 10:18:21 AM |
  | Library Catalog | Google Scholar |
  | Date Added | 10/11/2013, 10:29:15 AM |
  | Modified | 12/2/2013, 4:24:09 PM |

  ### Tags:

  - Local protein structure
  - pairwise local alignment
  - protein block
  - protein folds recognition
  - secondary structure
  - Structural alphabet
  - twilight zone sequences

  ### Notes:

  - Paper unavailable.
- ## A proteomic Ramachandran plot (PRplot)

  |  |  |
  | --- | --- |
  | Type | Journal Article |
  | Author | Oliviero Carugo |
  | Author | Kristina Djinovic-Carugo |
  | Volume | 44 |
  | Issue | 2 |
  | Pages | 781-790 |
  | Publication | Amino Acids |
  | ISSN | 0939-4451 |
  | Date | FEB 2013 |
  | Extra | WOS:000313794600045 |
  | DOI | 10.1007/s00726-012-1402-z |
  | Abstract | Each protein structure can be characterized by the average values of the main chain torsion angles I center dot and psi and, as a consequence, be plotted on a bidimensional diagram, which resembles the Ramachandran plot. Here, we describe a proteomic I center dot-psi plot (PRplot) where each protein structure is associated with one point, allowing in this way to represent the entire protein structure universe. It was verified that the PRplot is a robust tool since it does not depend on the dimension of the proteins, on the crystallographic resolution of the structures, nor on the biological source; moreover, it is little affected by disordered and structurally uncharacterized residues. The proteins mapped on the PRplot tend to cluster in three regions that correspond to the structures rich in alpha-helices, in beta-strands, and in both helices and strands, and are distributed along a sigmoidal curve that connect these three highly populated regions. PRplots are a unique instrument to project all protein structures on a single bidimensional plane where the entire structural complexity is reduced to a striking simplicity, with the sigmoid curve clearly delineating the space fraction accessible to a stable protein. |
  | Date Added | 2/13/2014, 4:13:17 PM |
  | Modified | 3/7/2014, 12:10:09 PM |

  ### Notes:

  - Paper unavailable.
- ## Are ambivalent alpha-helices entropically driven?

  |  |  |
  | --- | --- |
  | Type | Journal Article |
  | Author | Nicholus Bhattacharjee |
  | Author | Parbati Biswas |
  | Volume | 25 |
  | Issue | 2 |
  | Pages | 73-79 |
  | Publication | PROTEIN ENGINEERING DESIGN & SELECTION |
  | ISSN | 1741-0126 |
  | Date | February 2012 |
  | DOI | 10.1093/protein/gzr059 |
  | Language | English |
  | Abstract | This work is a first attempt to characterise the conformational preference of structurally ambivalent helices in terms of their backbone conformational entropy. Ambivalent sequences conform to two different secondary structures (helix-sheet or helix-random coil or sheet-random coil, etc.) in two different proteins. For variable ambivalent helices, the helical conformations are found to possess less conformational entropy as compared with their non-helical counterparts when the f-c dihedral angle range of the entire peptide segment is used to calculate the backbone conformational entropy. The favourable number of native contacts is a primary stabilising factor for these helical conformations. However, an opposite trend is observed when the f-c angles of the individual amino acids are used to calculate the backbone conformational entropy. The results show that these peptide segments are rather reluctant to form helices, but are driven to form helices due to the favourable number of native contacts and optimum range of f-c angle of the segments. Both procedures are validated by applying on conserved helices in the non-redundant database and their corresponding counterparts in the Structural Classification of Proteins database. Although context is a major determinant in deciding conformations of ambivalent sequences, no significant difference in the conformational entropy of sequences flanking ambivalent helical sequences in helical and non-helical forms is observed in this study. The results may be useful in understanding the structural context and environmental factors which leads to the formation of ambivalent helices and designing de novo proteins. |
  | Date Added | 10/11/2013, 10:29:15 AM |
  | Modified | 10/11/2013, 10:29:15 AM |

  ### Tags:

  - ambivalent helix
  - conformational entropy
  - native contacts

  ### Attachments

  - Protein Engineering, Design and Selection-2012-Bhattacharjee-73-9.pdf
- ## A REGIONALIZABLE STATISTICAL MODEL OF INTERSECTING REGIONS IN PROTEIN-LIGAND BINDING CAVITIES

  |  |  |
  | --- | --- |
  | Type | Journal Article |
  | Author | Brian Y. Chen |
  | Author | Soutir Bandyopadhyay |
  | Volume | 10 |
  | Issue | 3 |
  | Pages | 1242004 |
  | Publication | Journal of Bioinformatics and Computational Biology |
  | ISSN | 0219-7200 |
  | Date | JUN 2012 |
  | Extra | WOS:000305482100004 |
  | DOI | 10.1142/S0219720012420048 |
  | Abstract | Finding elements of proteins that influence ligand binding specificity is an essential aspect of research in many fields. To assist in this effort,this paper presents two statistical models, based on the same theoretical foundation, for evaluating structural similarity among binding cavities. The first model specializes in the "unified" comparison of whole cavities, enabling the selection of cavities that are too dissimilar to have similar binding specificity. The second model enables a "regionalized" comparison of cavities within a user-defined region, enabling the selection of cavities that are too dissimilar to bind the same molecular fragments in the given region. We applied these models to analyze the ligand binding cavities of the serine protease and enolase superfamilies. Next, we observed that our unified model correctly separated sets of cavities with identical binding preferences from other sets with varying binding preferences, and that our regionalized model correctly distinguished cavity regions that are too dissimilar to bind similar molecular fragments in the user-defined region. These observations point to applications of statistical modeling that can be used to examine and, more importantly, identify influential structural similarities within binding site structure in order to better detect influences on protein-ligand binding specificity. |
  | Date Added | 2/13/2014, 4:13:17 PM |
  | Modified | 3/7/2014, 1:07:11 PM |
- ## A simple and efficient statistical potential for scoring ensembles of protein structures

  |  |  |
  | --- | --- |
  | Type | Journal Article |
  | Author | Pilar Cossio |
  | Author | Daniele Granata |
  | Author | Alessandro Laio |
  | Author | Flavio Seno |
  | Author | Antonio Trovato |
  | Volume | 2 |
  | Pages | 351 |
  | Publication | Scientific Reports |
  | ISSN | 2045-2322 |
  | Date | APR 3 2012 |
  | Extra | WOS:000302460800001 |
  | DOI | 10.1038/srep00351 |
  | Abstract | In protein structure prediction it is essential to score quickly and reliably large sets of models by selecting the ones that are closest to the native state. We here present a novel statistical potential constructed by Bayesian analysis measuring a few structural observables on a set of 500 experimental protein structures. Even though employing much less parameters than current state-of-the-art methods, our potential is capable of discriminating with an unprecedented reliability the native state in large sets of misfolded models of the same protein. We also introduce the new idea that thermal fluctuations cannot be neglected for scoring models that are very similar to each other. In these cases, the best structure can be recognized only by comparing the probability distributions of our potential over short finite temperature molecular dynamics simulations starting from the competing models. |
  | Date Added | 2/13/2014, 4:13:17 PM |
  | Modified | 3/7/2014, 12:15:05 PM |
- ## Assessing predictors of changes in protein stability upon mutation using self-consistency

  |  |  |
  | --- | --- |
  | Type | Journal Article |
  | Author | Grant Thiltgen |
  | Author | Richard A. Goldstein |
  | URL | http://dx.plos.org/10.1371/journal.pone.0046084 |
  | Volume | 7 |
  | Issue | 10 |
  | Pages | e46084 |
  | Publication | PloS one |
  | Date | 2012 |
  | Accessed | 9/20/2013, 1:19:04 PM |
  | Library Catalog | Google Scholar |
  | Date Added | 10/11/2013, 10:29:15 AM |
  | Modified | 10/11/2013, 10:29:15 AM |

  ### Tags:

  - Interesting

  ### Notes:

  - Assess different methods for predicting stability changes upon mutation.

    How SCOP is used:

    Use SCOP in dataset curation, to remove redundancy.  Selected at most one pair of proteins from the same SCOP family where the sequences differed by exactly one amino acid.

    SCOP reference:

    To create the dataset, all single chain PDB sequences were compared to each other and all pairs of sequences with only one amino acid change were selected. This provided 22947 pairs of proteins. To further reduce this number to a reasonable testing size and to allow for structural variability among the proteins, a pairs of proteins were randomly selected among SCOP (v1.75) families with a maximum of one pair from each family (although not all families are represented). [20]. This reduced the size of the dataset to 83 pairs of proteins.

  ### Attachments

  - journal.pone.0046084.pdf
- ## Assessing the accuracy of template-based structure prediction metaservers by comparison with structural genomics structures

  |  |  |
  | --- | --- |
  | Type | Journal Article |
  | Author | Dominik Gront |
  | Author | Marek Grabowski |
  | Author | Matthew D. Zimmerman |
  | Author | John Raynor |
  | Author | Karolina L. Tkaczuk |
  | Author | Wladek Minor |
  | URL | http://link.springer.com/article/10.1007/s10969-012-9146-2 |
  | Volume | 13 |
  | Issue | 4 |
  | Pages | 213–225 |
  | Publication | Journal of structural and functional genomics |
  | Date | 2012 |
  | Accessed | 9/23/2013, 10:14:00 AM |
  | Library Catalog | Google Scholar |
  | Date Added | 2/20/2014, 12:24:01 PM |
  | Modified | 2/20/2014, 12:24:01 PM |

  ### Notes:

  - Perform assessment of template-based structure prediction metaservers.

    How SCOP is used:

    Refer to a study by a third party that found that SCOP families could be determined by clustering at 25% sequence identity.

    SCOP reference:

    However, in a recent study, Levitt [34] determined that clustering chain sequences at the 25 % sequence identity threshold was a very good determinant for classifying proteins in SCOP families [35].

  ### Attachments

  - art%3A10.1007%2Fs10969-012-9146-2.pdf
- ## Assignment of homology to genome sequences using a library of hidden Markov models that represent all proteins of known structure

  |  |  |
  | --- | --- |
  | Type | Journal Article |
  | Author | J Gough |
  | Author | K Karplus |
  | Author | R Hughey |
  | Author | C Chothia |
  | Volume | 313 |
  | Issue | 4 |
  | Pages | 903-919 |
  | Publication | JOURNAL OF MOLECULAR BIOLOGY |
  | ISSN | 0022-2836 |
  | Date | NOV 2 2001 |
  | DOI | 10.1006/jmbi.2001.5080 |
  | Language | English |
  | Abstract | Of the sequence comparison methods, profile-based methods perform with greater selectively than those that use pairwise comparisons. Of the profile methods, hidden Markov models (HMMs) are apparently the best. The first part of this paper describes calculations that (i) improve the performance of HMMs and (ii) determine a good procedure for creating HMMs for sequences of proteins of known structure. For a family of related proteins, more homologues. are detected using multiple models built from diverse single seed sequences than from one model built from a good alignment of those sequences. A new procedure is described for detecting and correcting those errors that arise at the model-building stage of the procedure. These two improvements greatly increase selectivity and coverage. The second part of the paper describes the construction of a library of HMMs, called SUPERFAMILY, that represent essentially all proteins of known structure. The sequences of the domains in proteins of known structure, that have identifies less than 95%, are used as seeds to build the models. Using the current data, this gives a library with 4894 models. The third part of the paper describes the use of the SUPERFAMILY model library to annotate the sequences of over 50 genomes. The models match twice as many target sequences as are matched by pairwise sequence comparison methods. For each genome, close to half of the sequences are matched in all or in part and, overall, the matches cover 35% of eukaryotic genomes and 45% of bacterial genomes. On average roughly 15% of genome sequences are labelled as being hypothetical yet homologous to proteins of known structure. The annotations derived from these matches are available from a public web server at: http://stash.mrc-lmb.cam.ac.uk/SUPERFAMILY. This server also enables users to match their own sequences against the SUPERFAMILY model library. (C) 2001 Academic Press. |
  | Date Added | 10/11/2013, 10:29:15 AM |
  | Modified | 10/11/2013, 10:29:15 AM |

  ### Tags:

  - ASTRAL
  - ASTRAL sequences

  ### Notes:

  - SUPERFAMILY paper.

    SUPERFAMILY is a collection of HMMs for classifying sequences into SCOP hierarchy.

    How SCOP is used:

    Use ASTRAL sequence data to build HMMs.

  ### Attachments

  - gough-etal-JMB-2001.pdf
- ## Assignment of protein sequences to existing domain and family classification systems: Pfam and the PDB

  |  |  |
  | --- | --- |
  | Type | Journal Article |
  | Author | Qifang Xu |
  | Author | Roland L. Dunbrack |
  | URL | http://bioinformatics.oxfordjournals.org/content/28/21/2763.short |
  | Volume | 28 |
  | Issue | 21 |
  | Pages | 2763–2772 |
  | Publication | Bioinformatics |
  | Date | 2012 |
  | Accessed | 9/20/2013, 1:12:54 PM |
  | Library Catalog | Google Scholar |
  | Short Title | Assignment of protein sequences to existing domain and family classification systems |
  | Date Added | 10/11/2013, 10:29:15 AM |
  | Modified | 3/7/2014, 12:10:29 PM |

  ### Notes:

  - Motivation: Existing protein domain and family classification systems do not cover the entire PDB.

    Results: Introduce a general procedure for domain detection and classification that can be applied to any classification system, with the goal of covering the entire PDB.  Method relies on Pfam and PSI-BLAST.

     How SCOP/CATH is used:

    Negative reference.  They mention how the same procedure could have been applied to SCOP or CATH, but they decided to only do it for Pfam.

    SCOP references:

    Structure-based domain classifications of the PDB, such as SCOP (Murzin et al., 1995) and CATH (Orengo et al., 1997), are constructed by comparing the available protein structures in the PDB and creating classifications of new folds and superfamilies manually. Existing structure-based classifications cover only a portion of the PDB. The most recent SCOP release (v. 1.75A) is 2 years behind the PDB and only covers 61% of current PDB entries. CATH was last updated in November 2011 and covers 64% of the current PDB.

    The most recent SCOP release (v. 1.75A) is 2 years behind the PDB and only covers 61% of current PDB entries. CATH was last updated in November 2011 and covers 64% of the current PDB.

    To get a fair assessment of the RCSBs coverage, we used the same criterion we applied to our data—no>10 residues of over- lap between Pfam assignments. The structure protein classification systems CATH and SCOP have much lower coverages because they are built manually and updated infrequently.

    SCOP and CATH designations are sometimes provided, which solves the first problem, but SCOP and CATH represent less than two-thirds of the PDB, and their utility for this purpose is, therefore, limited.

    We can imagine a number of further applications that will be presented later, including Pfam assignments to human proteins and assignment of SCOP do- mains to the entire PDB on an ongoing basis.

  ### Attachments

  - Full Text PDF
- ## A structural model of the E. coli PhoB dimer in the transcription initiation complex

  |  |  |
  | --- | --- |
  | Type | Journal Article |
  | Author | Chang-Shung Tung |
  | Author | Benjamin H McMahon |
  | Volume | 12 |
  | Pages | 3 |
  | Publication | BMC Structural Biology |
  | ISSN | 1472-6807 |
  | Date | 2012 |
  | Extra | PMID: 22433509 |
  | Journal Abbr | BMC Struct. Biol. |
  | DOI | 10.1186/1472-6807-12-3 |
  | Library Catalog | NCBI PubMed |
  | Language | eng |
  | Abstract | BACKGROUND: There exist > 78,000 proteins and/or nucleic acids structures that were determined experimentally. Only a small portion of these structures corresponds to those of protein complexes. While homology modeling is able to exploit knowledge-based potentials of side-chain rotomers and backbone motifs to infer structures for new proteins, no such general method exists to extend our understanding of protein interaction motifs to novel protein complexes. RESULTS: We use a Motif Binding Geometries (MBG) approach, to infer the structure of a protein complex from the database of complexes of homologous proteins taken from other contexts (such as the helix-turn-helix motif binding double stranded DNA), and demonstrate its utility on one of the more important regulatory complexes in biology, that of the RNA polymerase initiating transcription under conditions of phosphate starvation. The modeled PhoB/RNAP/σ-factor/DNA complex is stereo-chemically reasonable, has sufficient interfacial Solvent Excluded Surface Areas (SESAs) to provide adequate binding strength, is physically meaningful for transcription regulation, and is consistent with a variety of known experimental constraints. CONCLUSIONS: Based on a straightforward and easy to comprehend concept, "proteins and protein domains that fold similarly could interact similarly", a structural model of the PhoB dimer in the transcription initiation complex has been developed. This approach could be extended to enable structural modeling and prediction of other bio-molecular complexes. Just as models of individual proteins provide insight into molecular recognition, catalytic mechanism, and substrate specificity, models of protein complexes will provide understanding into the combinatorial rules of cellular regulation and signaling. |
  | Date Added | 10/11/2013, 10:29:15 AM |
  | Modified | 12/2/2013, 4:19:53 PM |

  ### Tags:

  - Bacterial Proteins
  - Base Sequence
  - Binding Sites
  - DNA, Bacterial
  - DNA-Directed RNA Polymerases
  - Escherichia coli
  - Models, Molecular
  - Molecular Sequence Data
  - Promoter Regions, Genetic
  - Protein Binding
  - Protein Multimerization
  - Protein Subunits
  - Transcription, Genetic

  ### Notes:

  - Infer the structure of a protein complex (E. coli PhoB Dimer) using Motif Binding Geometries (MBG) approach which relies on a database of complexes.

    How SCOP Is used:

    Retrieve fold classification of the PhoB Receiver Domain and list other proteins in the same family.

    SCOP reference:

    The PhoB RD adopts a b-a structure [8] that can be classified as a flavodoxin-like fold according to SCOP [9]. The flavodoxin-like fold can be found in RDs of other response regulators as well as flavodoxins [10], cytochrome-P450 oxidoreductase [11] and Toll/Interleukin Receptor TIR domains [12]. These protein domains share the same structural fold with lit- tle or no sequence homology.

  ### Attachments

  - 1472-6807-12-3.pdf
- ## A systematic comparison of protein structure classifications: SCOP, CATH and FSSP

  |  |  |
  | --- | --- |
  | Type | Journal Article |
  | Author | C. Hadley |
  | Author | D. T. Jones |
  | Volume | 7 |
  | Issue | 9 |
  | Pages | 1099-1112 |
  | Publication | Structure (London, England: 1993) |
  | ISSN | 0969-2126 |
  | Date | Sep 15, 1999 |
  | Extra | PMID: 10508779 |
  | Journal Abbr | Structure |
  | Library Catalog | NCBI PubMed |
  | Language | eng |
  | Abstract | BACKGROUND: Several methods of structural classification have been developed to introduce some order to the large amount of data present in the Protein Data Bank. Such methods facilitate structural comparisons and provide a greater understanding of structure and function. The most widely used and comprehensive databases are SCOP, CATH and FSSP, which represent three unique methods of classifying protein structures: purely manual, a combination of manual and automated, and purely automated, respectively. In order to develop reliable template libraries and benchmarks for protein-fold recognition, a systematic comparison of these databases has been carried out to determine their overall agreement in classifying protein structures. RESULTS: Approximately two-thirds of the protein chains in each database are common to all three databases. Despite employing different methods, and basing their systems on different rules of protein structure and taxonomy, SCOP, CATH and FSSP agree on the majority of their classifications. Discrepancies and inconsistencies are accounted for by a small number of explanations. Other interesting features have been identified, and various differences between manual and automatic classification methods are presented. CONCLUSIONS: Using these databases requires an understanding of the rules upon which they are based; each method offers certain advantages depending on the biological requirements and knowledge of the user. The degree of discrepancy between the systems also has an impact on reliability of prediction methods that employ these schemes as benchmarks. To generate accurate fold templates for threading, we extract information from a consensus database, encompassing agreements between SCOP, CATH and FSSP. |
  | Short Title | A systematic comparison of protein structure classifications |
  | Date Added | 10/29/2014, 11:59:54 AM |
  | Modified | 10/29/2014, 11:59:54 AM |

  ### Tags:

  - Databases, Factual
  - Protein Conformation
  - Protein Folding
  - Proteins
  - Reproducibility of Results
  - Sequence Homology

  ### Attachments

  - PubMed entry
- ## A thermodynamic definition of protein domains

  |  |  |
  | --- | --- |
  | Type | Journal Article |
  | Author | Lauren L. Porter |
  | Author | George D. Rose |
  | URL | http://www.pnas.org/content/109/24/9420.short |
  | Volume | 109 |
  | Issue | 24 |
  | Pages | 9420–9425 |
  | Publication | Proceedings of the National Academy of Sciences |
  | Date | 2012 |
  | Accessed | 9/20/2013, 1:17:19 PM |
  | Library Catalog | Google Scholar |
  | Date Added | 10/11/2013, 10:29:15 AM |
  | Modified | 3/7/2014, 12:11:19 PM |

  ### Notes:

  - Present method to identify protein domains using thermodynamics experimental data (denaturing proteins with urea).  Compare results with CATH and SCOP domains.

    Motivation: inconsistency in domains among competing databases.  "seeing can be deceiving. The dependence on visual intuition introduces an unavoidable element of ambiguity into procedures for domain recognition."

     How using SCOP:

    Did not use SCOP to collect evaluation data set of 71 proteins, used CATH instead.

    Compared all domains predicted to SCOP and CATH.

    Reference to SCOP:

    Today, CATH (14) and SCOP (15) are the two most widely used domain classifications. Both are based on computational algorithms but rely ultimately on the human eye as the final arbiter of domain boundaries.

    ...

    This inherent ambiguity is reflected in conflicting domain classifications for the same protein. For example, CATH classifies human proliferating cell nuclear antigen (hPCNA) (1u7bA) as a single-domain pro- tein, but both SCOP and those who solved its structure identify two domains (17).

  ### Attachments

  - Full Text PDF
- ## A threading-based method (FINDSITE) for ligand-binding site prediction and functional annotation

  |  |  |
  | --- | --- |
  | Type | Journal Article |
  | Author | Michal Brylinski |
  | Author | Jeffrey Skolnick |
  | Volume | 105 |
  | Issue | 1 |
  | Pages | 129-134 |
  | Publication | Proceedings of the National Academy of Sciences of the United States of America |
  | ISSN | 1091-6490 |
  | Date | Jan 8, 2008 |
  | Extra | PMID: 18165317 |
  | Journal Abbr | Proc. Natl. Acad. Sci. U.S.A. |
  | DOI | 10.1073/pnas.0707684105 |
  | Library Catalog | NCBI PubMed |
  | Language | eng |
  | Abstract | The detection of ligand-binding sites is often the starting point for protein function identification and drug discovery. Because of inaccuracies in predicted protein structures, extant binding pocket-detection methods are limited to experimentally solved structures. Here, FINDSITE, a method for ligand-binding site prediction and functional annotation based on binding-site similarity across groups of weakly homologous template structures identified from threading, is described. For crystal structures, considering a cutoff distance of 4 A as the hit criterion, the success rate is 70.9% for identifying the best of top five predicted ligand-binding sites with a ranking accuracy of 76.0%. Both high prediction accuracy and ability to correctly rank identified binding sites are sustained when approximate protein models (<35% sequence identity to the closest template structure) are used, showing a 67.3% success rate with 75.5% ranking accuracy. In practice, FINDSITE tolerates structural inaccuracies in protein models up to a rmsd from the crystal structure of 8-10 A. This is because analysis of weakly homologous protein models reveals that about half have a rmsd from the native binding site <2 A. Furthermore, the chemical properties of template-bound ligands can be used to select ligand templates associated with the binding site. In most cases, FINDSITE can accurately assign a molecular function to the protein model. |
  | Date Added | 10/11/2013, 10:29:15 AM |
  | Modified | 10/11/2013, 10:29:15 AM |

  ### Tags:

  - Algorithms
  - Binding Sites
  - Biophysics
  - Computational Biology
  - Crystallography, X-Ray
  - Ligands
  - ligand screening
  - Models, Molecular
  - Models, Statistical
  - Molecular Conformation
  - pocket detection
  - Protein Binding
  - Protein Conformation
  - Protein Interaction Mapping
  - Proteins
  - protein structure prediction
  - Reproducibility of Results
  - Software

  ### Notes:

  - Present a method for binding site prediction and function annotation.  FINDSITE uses binding-site similarity across groups of weakly homologous template structures identified from threading.

    How SCOP is used:

    SCOP data is not used.  A previous study in the Sternberg lab, using SCOP, is referenced.  The study had used SCOP classification to study proteins with similar folds and determine whether binding sites were similar.

    SCOP reference:

    A systematic analysis of known protein structures grouped according to SCOP (22) reveals a general tendency of certain protein folds to bind substrates at a similar location, suggesting that analogous or very distantly homologous proteins can have common binding sites (11).

  ### Attachments

  - PNAS-2008-Brylinski-129-34.pdf
  - PubMed entry
- ## A time-interval sequence classification method

  |  |  |
  | --- | --- |
  | Type | Journal Article |
  | Author | Chieh-Yuan Tsai |
  | Author | Chih-Jung Chen |
  | Author | Chun-Ju Chien |
  | Volume | 37 |
  | Issue | 2 |
  | Pages | 251-278 |
  | Publication | Knowledge and Information Systems |
  | ISSN | 0219-1377; 0219-3116 |
  | Date | NOV 2013 |
  | Extra | WOS:000325812000002 |
  | DOI | 10.1007/s10115-012-0501-1 |
  | Abstract | Classification is one of the most popular behavior prediction tools in behavior informatics (behavior computing) to predict group membership for data instances. It has been greatly used to support customer relationship management (CRM) such as customer identification, one-to-one marketing, fraud detection, and lifetime value analysis. Although previous studies showed themselves efficient and accurate in certain CRM classification applications, most of them took demographic, RFM-type, or activity attributes as classification criteria and seldom took temporal relationship among these attributes into account. To bridge this gap, this study takes customer temporal behavior data, called time-interval sequences, as classification criteria and develops a two-stage classification framework. In the first stage, time-interval sequential patterns are discovered from customer temporal databases. Then, a time-interval sequence classifier optimized by the particle swam optimization (PSO) algorithm is developed to achieve high classification accuracy in the second stage. The experiment results indicate the proposed time-interval sequence classification framework is efficient and accurate to predict the class label of new customer temporal data. |
  | Date Added | 2/12/2014, 1:36:22 PM |
  | Modified | 2/12/2014, 1:36:22 PM |

  ### Notes:

  - Present a general classification method.

    How SCOP is used:

    Train and validate method on a data set of PDBs, classified by SCOP fold.

    SCOP reference:

    To fulfill the goal of classification accuracy comparison, a group of primary protein sequences derived from the Protein Data Bank (PDB) [5] is utilized. All data in this group correspond to a specific fold of the structural classification of proteins (SCOP) database [40]. In this validation, 1,000 proteins (sequences) belonging to 17 SCOP classes are retrieved. Two-third of them randomly selected from each class is formed as a training dataset, while the rest are formed as a testing dataset. In addition, six approaches are applied to evaluate the classification accuracy [19]:

  ### Attachments

  - art%3A10.1007%2Fs10115-012-0501-1.pdf
- ## A Topology Structure Based Outer Membrane Proteins Segment Alignment Method

  |  |  |
  | --- | --- |
  | Type | Journal Article |
  | Author | Han Wang |
  | Author | Bo Liu |
  | Author | Pingping Sun |
  | Author | Zhiqiang Ma |
  | Pages | 541359 |
  | Publication | Mathematical Problems in Engineering |
  | ISSN | 1024-123X; 1563-5147 |
  | Date | 2013 |
  | Extra | WOS:000326590000001 |
  | DOI | 10.1155/2013/541359 |
  | Abstract | Outer membrane proteins (OMPs) are transmembrane proteins (TMPs) located in outer membranes. These proteins perform diverse biochemical functions and have immediate medical relevance, so that their spatial structures are important for studying. But the special physicochemical properties of OMP make it hard to obtain their structures experimentally. For the purpose of predicting OMP structures, discriminating OMPs and aligning their sequences to native structures are indispensable steps. We developed a novelmethod OMSA (OuterMembrane Segment Alignment), which implemented both steps in one program. OMSA integratesOMP-specific topology features to implement a sequence-to-structure alignment, for example, segment type and segment orientation, while a segment-dependent gap penalty model is employed to improve the alignment. Compared to peer top-leading methods, OMSA achieved higher accuracy in bothOMPdiscrimination and alignment, whichmay further improveOMP structure studying. |
  | Date Added | 2/20/2014, 12:24:01 PM |
  | Modified | 2/20/2014, 12:24:01 PM |

  ### Notes:

  - Present method for discriminating and aligning outer membrane proteins (OMPs).

    How SCOP is used:

    Annotate data set of membrane proteins by SCOP superfamily and family, and used these to create a training and test set for their method for novel Outer Membrane Segmen Alignment (OMSA) to discriminate and align outer membrane proteins (OMPs).

    SCOP reference:

    2. Materials and Methods

    2.1.Datasets. OrientationsofProteinsinMembranes(OPM) database [28] was used in OMSA training and testing; it provides the most comprehensive collection of membrane proteins with calculated spatial arrangements. Differing to computational-based databases [29, 30], OPM database is more in agreement with the experimental data and further classifies the membrane proteins based on their main trans- membrane domains by referencing SCOP [31] and TCDB [32]. In this database, 98 entries are classified to 26 superfam- ilies, and each of them is composed of one or more protein families. Here, proteins in the same superfamily are evolu- tionarily related and with superimposable tertiary structures, but in low sequence identity, while it is high among the proteins in the same family. We randomly picked two entries from each superfamily to comprise training and testing datasets, respectively. For those superfamilies which have only one entry, the entries were selected to training dataset. Finally, the training dataset is composed of 19 nonredundant entries, while testing dataset has 28 nonredundant entries (see Table S1 in supplementary material available online at http://dx.doi.org/10.1155/2013/541359).

    For the purpose of benchmarking the performance of OMP discrimination, Gromiha and Suwa’s dataset (GS- dataset) [13] is used, which includes 377 OMPs, 268 ⬚⬚-helical

    transmembrane proteins, and 674 globular protein chains. All these well-annotated transmembrane proteins included in the dataset were obtained from PSORT-B database [33], while those globular protein chains were obtained from the PDB40D 1.37 database of SCOP [34]. In this dataset, a few transmembrane proteins are homologous, and the globular proteins have sequence identity less than 30%.

  ### Attachments

  - 541359.pdf
- ## ATP Sequestration by a Synthetic ATP-Binding Protein Leads to Novel Phenotypic Changes in Escherichia coli

  |  |  |
  | --- | --- |
  | Type | Journal Article |
  | Author | Shaleen B. Korch |
  | Author | Joshua M. Stomel |
  | Author | Megan A. Leon |
  | Author | Matt A. Hamada |
  | Author | Christine R. Stevenson |
  | Author | Brent W. Simpson |
  | Author | Sunil K. Gujulla |
  | Author | John C. Chaput |
  | Volume | 8 |
  | Issue | 2 |
  | Pages | 451–463 |
  | Publication | Acs Chemical Biology |
  | Date | February 2013 |
  | DOI | 10.1021/cb3004786 |
  | Abstract | Artificial proteins that bind key metabolites with high affinity and specificity hold great promise as new tools in synthetic biology, but little has been done to create such molecules and examine their effects on living cells. Experiments of this kind have the potential to expand our understanding of cellular systems, as certain phenotypes may be physically realistic but not yet observed in nature. Here, we examine the physiology and morphology of a population of Escherichia coli as they respond to a genetically encoded, non-biological ATP-binding protein. Unlike natural ATP-dependent proteins, which transiently bind ATP during metabolic transformations, the synthetic protein DX depletes the concentration of intracellular ATP and ADP by a mechanism of protein-mediated ligand sequestration. The resulting ATP/ADP imbalance leads to an adaptive response in which a large population of bacilli cells transition to a filamentous state with dense lipid structures that segregate the cells into compartmentalized units. A wide range of biochemical and microscopy techniques extensively characterized these novel lipid structures, which we have termed endoliposomes. We show that endoliposomes adopt well-defined box-like structures that span the full width of the cell but exclude the synthetic protein DX. We further show that prolonged DX exposure causes a large fraction of the population to enter a viable-but-non-culturable state that is not easily reversed. Both phenotypes correlate with strong intracellular changes in ATP and ADP concentration. We suggest that artificial proteins, such as DX, could be used to control and regulate specific targets in metabolic pathways. |
  | Date Added | 3/7/2014, 1:06:24 PM |
  | Modified | 3/7/2014, 1:06:24 PM |
- ## A Universal Trend among Proteomes Indicates an Oily Last Common Ancestor

  |  |  |
  | --- | --- |
  | Type | Journal Article |
  | Author | Ranjan V. Mannige |
  | Author | Charles L. Brooks |
  | Author | Eugene I. Shakhnovich |
  | URL | http://dx.plos.org/10.1371/journal.pcbi.1002839 |
  | Volume | 8 |
  | Issue | 12 |
  | Pages | e1002839 |
  | Publication | PLoS computational biology |
  | Date | 2012 |
  | Accessed | 9/20/2013, 1:18:50 PM |
  | Library Catalog | Google Scholar |
  | Date Added | 10/11/2013, 10:29:15 AM |
  | Modified | 10/11/2013, 10:29:15 AM |

  ### Tags:

  - likely ASTRAL
  - likely ASTRAL sequences
  - likely ASTRAL subsets

  ### Notes:

  - The ultimate goal is to identify the features of the last common ancestor of all lifeforms.  Toward this goal, they study the evolution of a proteome from oily (highly hydrophobic) to less oily across multiple species.

    How SCOP is used:

    Used SCOP sequences from 1.75, filtered at <=10% sequence identity, as "seed" protein domains.  Then clustered homologous sequences to do some analysis on oil escape vs. species age.

    SCOP reference:

    Here we show that ‘‘oil escape’’ occurs not only at the proteome level, but also at the individual protein composition level (which is evidenced by changes in oil content in groups of homologous, and later, orthologous, proteins over organism node space). Our ‘‘single protein’’ studies were performed on clusters of protein sequences homologous to ‘‘seed’’ protein domains listed in the SCOP database (v1.75, redundancy ƒ10%) [21]. Within a cluster, each proteome was represented at most once, and homology was ascertained by BLAST-P’s default value.

  ### Attachments

  - [HTML] from plos.org
  - journal.pcbi.1002839.pdf
  - PubMed entry
- ## Automatch: Target-binding protein design and enzyme design by automatic pinpointing potential active sites in available protein scaffolds

  |  |  |
  | --- | --- |
  | Type | Journal Article |
  | Author | Changsheng Zhang |
  | Author | Luhua Lai |
  | URL | http://onlinelibrary.wiley.com/doi/10.1002/prot.24009/full |
  | Volume | 80 |
  | Issue | 4 |
  | Pages | 1078–1094 |
  | Publication | Proteins: Structure, Function, and Bioinformatics |
  | Date | 2012 |
  | Accessed | 9/23/2013, 10:14:18 AM |
  | Library Catalog | Google Scholar |
  | Short Title | Automatch |
  | Date Added | 10/11/2013, 10:29:15 AM |
  | Modified | 10/11/2013, 10:29:15 AM |

  ### Tags:

  - active site recapitulation
  - active sites matching
  - backbone flexibility
  - enzyme design
  - target-binding protein design

  ### Notes:

  - Present a new method and program "AutoMatch" to predict good 'grafting sites' to attach an active site onto a new scaffold.  It also is used to help screen for good scaffolds.  This is an important piece of protein design and synthetic biology.

    How SCOP data is used:

    They built their own dataset, using some criteria unbeknownst to me, and then categorize by SCOP class to show structural diversity.

    SCOP reference:

    The protein name, SCOP class, 59 active sites mutation, RMSD of active atoms between the designed and native proteins, binding energy score, and full-mutation conformation score for active residues [(see definition in method part Eq. (2)] are presented in the results table. Excluding α/β proteins that are not present in gp120-binding protein design results table, and all β proteins that are not present in hemagglutinin-binding protein design table, the four major SCOP class proteins can be found in the three results tables.

  ### Attachments

  - pdf
  - Snapshot
- ## Automated identification of protein-ligand interaction features using Inductive Logic Programming: a hexose binding case study

  |  |  |
  | --- | --- |
  | Type | Journal Article |
  | Author | Jose C. A. Santos |
  | Author | Houssam Nassif |
  | Author | David Page |
  | Author | Stephen H. Muggleton |
  | Author | Michael J. E. Sternberg |
  | Volume | 13 |
  | Pages | 162 |
  | Publication | Bmc Bioinformatics |
  | ISSN | 1471-2105 |
  | Date | JUL 11 2012 |
  | Extra | WOS:000309157600001 |
  | DOI | 10.1186/1471-2105-13-162 |
  | Abstract | Background: There is a need for automated methods to learn general features of the interactions of a ligand class with its diverse set of protein receptors. An appropriate machine learning approach is Inductive Logic Programming (ILP), which automatically generates comprehensible rules in addition to prediction. The development of ILP systems which can learn rules of the complexity required for studies on protein structure remains a challenge. In this work we use a new ILP system, ProGolem, and demonstrate its performance on learning features of hexose-protein interactions. Results: The rules induced by ProGolem detect interactions mediated by aromatics and by planar-polar residues, in addition to less common features such as the aromatic sandwich. The rules also reveal a previously unreported dependency for residues CYS and LEU. They also specify interactions involving aromatic and hydrogen bonding residues. This paper shows that Inductive Logic Programming implemented in ProGolem can derive rules giving structural features of protein/ligand interactions. Several of these rules are consistent with descriptions in the literature. Conclusions: In addition to confirming literature results, ProGolem's model has a 10-fold cross-validated predictive accuracy that is superior, at the 95% confidence level, to another ILP system previously used to study protein/hexose interactions and is comparable with state-of-the-art statistical learners. |
  | Date Added | 2/13/2014, 4:13:17 PM |
  | Modified | 3/7/2014, 12:11:11 PM |
- ## Automatic alpha-helix identification in Patterson maps

  |  |  |
  | --- | --- |
  | Type | Journal Article |
  | Author | Rocco Caliandro |
  | Author | Domenica Dibenedetto |
  | Author | Giovanni Luca Cascarano |
  | Author | Annamaria Mazzone |
  | Author | Giovanni Nico |
  | Volume | 68 |
  | Pages | 1-12 |
  | Publication | Acta Crystallographica Section D-Biological Crystallography |
  | ISSN | 0907-4449 |
  | Date | JAN 2012 |
  | Extra | WOS:000298412300001 |
  | DOI | 10.1107/S0907444911046282 |
  | Abstract | alpha-Helices are peculiar atomic arrangements characterizing protein structures. Their occurrence can be used within crystallographic methods as minimal a priori information to drive the phasing process towards solution. Recently, brute-force methods have been developed which search for all possible positions of alpha-helices in the crystal cell by molecular replacement and explore all of them systematically. Knowing the alpha-helix orientations in advance would be a great advantage for this kind of approach. For this purpose, a fully automatic procedure to find alpha-helix orientations within the Patterson map has been developed. The method is based on Fourier techniques specifically addressed to the identification of helical shapes and operating on Patterson maps described in spherical coordinates. It supplies a list of candidate orientations, which are then refined by using a figure of merit based on a rotation function calculated for a template polyalanine helix oriented along the current direction. The orientation search algorithm has been optimized to work at 3 A resolution, while the candidates are refined against all measured reflections. The procedure has been applied to a large number of protein test structures, showing an overall efficiency of 77% in finding alpha-helix orientations, which decreases to 48% on limiting the number of candidate solutions (to 13 on average). The information obtained may be used in many aspects in the framework of molecular-replacement phasing, as well as to constrain the generation of models in computational modelling programs. The procedure will be accessible through the next release of IL MILIONE and could be decisive in the solution of new unknown structures. |
  | Date Added | 2/13/2014, 4:13:17 PM |
  | Modified | 3/7/2014, 12:15:20 PM |
- ## Automatic classification of protein structures relying on similarities between alignments

  |  |  |
  | --- | --- |
  | Type | Journal Article |
  | Author | Guillaume Santini |
  | Author | Henry Soldano |
  | Author | Joel Pothier |
  | Volume | 13 |
  | Publication | BMC bioinformatics |
  | ISSN | 1471-2105 |
  | Date | SEP 14 2012 |
  | DOI | 10.1186/1471-2105-13-233 |
  | Language | English |
  | Abstract | Background: Identification of protein structural cores requires isolation of sets of proteins all sharing a same subset of structural motifs. In the context of an ever growing number of available 3D protein structures, standard and automatic clustering algorithms require adaptations so as to allow for efficient identification of such sets of proteins. Results: When considering a pair of 3D structures, they are stated as similar or not according to the local similarities of their matching substructures in a structural alignment. This binary relation can be represented in a graph of similarities where a node represents a 3D protein structure and an edge states that two 3D protein structures are similar. Therefore, classifying proteins into structural families can be viewed as a graph clustering task. Unfortunately, because such a graph encodes only pairwise similarity information, clustering algorithms may include in the same cluster a subset of 3D structures that do not share a common substructure. In order to overcome this drawback we first define a ternary similarity on a triple of 3D structures as a constraint to be satisfied by the graph of similarities. Such a ternary constraint takes into account similarities between pairwise alignments, so as to ensure that the three involved protein structures do have some common substructure. We propose hereunder a modification algorithm that eliminates edges from the original graph of similarities and gives a reduced graph in which no ternary constraints are violated. Our approach is then first to build a graph of similarities, then to reduce the graph according to the modification algorithm, and finally to apply to the reduced graph a standard graph clustering algorithm. Such method was used for classifying ASTRAL-40 non-redundant protein domains, identifying significant pairwise similarities with Yakusa, a program devised for rapid 3D structure alignments. Conclusions: We show that filtering similarities prior to standard graph based clustering process by applying ternary similarity constraints i) improves the separation of proteins of different classes and consequently ii) improves the classification quality of standard graph based clustering algorithms according to the reference classification SCOP. |
  | Date Added | 10/25/2013, 4:17:08 PM |
  | Modified | 5/5/2014, 3:10:51 PM |

  ### Notes:

  - Present method for protein structure classification.

    How SCOP is used:

    Validate method on the SCOP family level classification.   For dataset, use ASTRAL representative sequences filtered at 40% sequence identity.

    How CATH is used:

    Not using CATH data.

    SCOP reference:

    In abstract:

    Conclusions: We show that filtering similarities prior to standard graph based clustering process by applying ternary similarity constraints i) improves the separation of proteins of different classes and consequently ii) improves the classification quality of standard graph based clustering algorithms according to the reference classification SCOP.

    ...

    Under "Material":

    The set of items is taken from 3D protein structure of domains of SCOP database [3]. Over the 488.567 available domain structures we restrict our search to a non-redundant subset made of the 10.569 SCOP domain representatives exhibiting less than 40% sequence identity - i.e. the ASTRAL 40 data set (version 1.75) [17].

    SCOP/CATH reference:

    Such a library can be built upon a set of representative structures taken from expert structural classifications [2,3] as SCOP [3] and CATH [4].

  ### Attachments

  - 1471-2105-13-233.pdf
- ## Automatic phylogenetic classification of bacterial beta-lactamase sequences including structural and antibiotic substrate preference information

  |  |  |
  | --- | --- |
  | Type | Journal Article |
  | Author | Jianmin Ma |
  | Author | Frank Eisenhaber |
  | Author | Sebastian Maurer-Stroh |
  | Volume | 11 |
  | Issue | 6 |
  | Pages | 1343011 |
  | Publication | Journal of Bioinformatics and Computational Biology |
  | ISSN | 0219-7200; 1757-6334 |
  | Date | DEC 2013 |
  | Extra | WOS:000329998600012 |
  | DOI | 10.1142/S0219720013430117 |
  | Abstract | Beta lactams comprise the largest and still most effective group of antibiotics, but bacteria can gain resistance through different beta lactamases that can degrade these antibiotics. We developed a user friendly tree building web server that allows users to assign beta lactamase sequences to their respective molecular classes and subclasses. Further clinically relevant information includes if the gene is typically chromosomal or transferable through plasmids as well as listing the antibiotics which the most closely related reference sequences are known to target and cause resistance against. This web server can automatically build three phylogenetic trees: the first tree with closely related sequences from a Tachyon search against the NCBI nr database, the second tree with curated reference beta lactamase sequences, and the third tree built specifically from substrate binding pocket residues of the curated reference beta lactamase sequences. We show that the latter is better suited to recover antibiotic substrate assignments through nearest neighbor annotation transfer. The users can also choose to build a structural model for the query sequence and view the binding pocket residues of their query relative to other beta lactamases in the sequence alignment as well as in the 3D structure relative to bound antibiotics. This web server is freely available at http://blac.bii.a-star.edu.sg/. |
  | Date Added | 2/20/2014, 12:24:01 PM |
  | Modified | 5/1/2015, 9:56:24 AM |

  ### Notes:

  - Develop webserver for phylogenetic analysis of beta lactamases.

    How SCOP is used:

    Validate method for beta-lactamase detection using ASTRAL sequence data, with two superfamilies removed as negative data, then used their own data set of sequence data.

    SCOP reference:

    4.1. Performance of the classi ̄cation of beta lactamase and

    nonbeta lactamase sequences

    To check the ability of the server to correctly assign class labels to potential beta lactamase sequences and correctly recognize sequences not related to beta lacta- mases, we identi ̄ed suitable positive and negative sets for performance testing. Given the exhaustive database and literature curation e®ort described above, our seed sequences represent the current best set of known and highly likely beta lactamases and were hence adopted as the positive dataset (altogether 215 sequences). Sequences of the SCOP ASTRAL subset with known 3D structures but unrelated to beta lactamase folds were adopted as the negative dataset.42,43 In detail, after the Astral SCOP 1.75b nr40 sequences were fetched from the website of SCOP, sequences belonging to the \Metallo-hydrolase/oxidoreductase" super- family and \beta-lactamase/transpeptidase-like" super-family were removed, leaving 11,152 sequences. The latter includes sequences of class A, C and D beta lacta- mases, and the former includes sequences of class B beta lactamase which comprise a di®erent structural fold compared to the other classes.

  ### Attachments

  - s0219720013430117.pdf
- ## Babesia divergens and Neospora caninum apical membrane antigen 1 structures reveal selectivity and plasticity in apicomplexan parasite host cell invasion

  |  |  |
  | --- | --- |
  | Type | Journal Article |
  | Author | Michelle L. Tonkin |
  | Author | Joanna Crawford |
  | Author | Maryse L. Lebrun |
  | Author | Martin J. Boulanger |
  | Volume | 22 |
  | Issue | 1 |
  | Pages | 114–127 |
  | Publication | Protein Science |
  | Date | January 2013 |
  | DOI | 10.1002/pro.2193 |
  | Abstract | Host cell invasion by the obligate intracellular apicomplexan parasites, including Plasmodium (malaria) and Toxoplasma (toxoplasmosis), requires a step-wise mechanism unique among known hostpathogen interactions. A key step is the formation of the moving junction (MJ) complex, a circumferential constriction between the apical tip of the parasite and the host cell membrane that traverses in a posterior direction to enclose the parasite in a protective vacuole essential for intracellular survival. The leading model of MJ assembly proposes that Rhoptry Neck Protein 2 (RON2) is secreted into the host cell and integrated into the membrane where it serves as the receptor for apical membrane antigen 1 (AMA1) on the parasite surface. We have previously demonstrated that the AMA1-RON2 interaction is an effective target for inhibiting apicomplexan invasion. To better understand the AMA1-dependant molecular recognition events that promote invasion, including the significant AMA1-RON2 interaction, we present the structural characterization of AMA1 from the apicomplexan parasites Babesia divergens (BdAMA1) and Neospora caninum (NcAMA1) by X-ray crystallography. These studies offer intriguing structural insight into the RON2-binding surface groove in the AMA1 apical domain, which shows clear evidence for receptorligand co-evolution, and the hyper variability of the membrane proximal domain, which in Plasmodium is responsible for direct binding to erythrocytes. By incorporating the structural analysis of BdAMA1 and NcAMA1 with existing AMA1 structures and complexes we were able to define conserved pockets in the AMA1 apical groove that could be targeted for the design of broadly reactive therapeutics. |
  | Date Added | 3/7/2014, 1:06:24 PM |
  | Modified | 3/7/2014, 1:06:24 PM |
- ## Bacillus cereus sphingomyelinase recognizes ganglioside GM3

  |  |  |
  | --- | --- |
  | Type | Journal Article |
  | Author | Masataka Oda |
  | Author | Aoi Fujita |
  | Author | Kensuke Okui |
  | Author | Kazuaki Miyamoto |
  | Author | Masahiro Shibutani |
  | Author | Teruhisa Takagishi |
  | Author | Masahiro Nagahama |
  | Volume | 431 |
  | Issue | 2 |
  | Pages | 164-168 |
  | Publication | Biochemical and biophysical research communications |
  | ISSN | 0006-291X |
  | Date | FEB 8 2013 |
  | DOI | 10.1016/j.bbrc.2013.01.002 |
  | Language | English |
  | Abstract | Sphingomyelinase (SMase) from Bacillus cereus (Bc-SMase) hydrolyzes sphingomyelin (SM) to phospho-choline and ceramide in a divalent metal ion-dependent manner, and is a virulence factor for septicemia. Bc-SMase has three characteristic sites, viz., the central site (catalytic site), side-edge site (membrane binding site), and beta-hairpin region (membrane binding site). Here, we show that the beta-hairpin directly binds to gangliosides, especially NeuAc alpha 2-3Gal beta 1-4Glc beta 1-1ceramide (GM3) through a carbohydrate moiety. Neuraminidase inhibited the binding of Bc-SMase to mouse peritoneal macrophages in a dose-dependent manner. SPR analysis revealed that the binding response of Bc-SMase to liposomes containing GM3 was about 15-fold higher than that to liposomes lacking GM3. Moreover, experiments with sitedirected mutants indicated that Trp-284 and Phe-285 in the beta-hairpin play an important role in the interaction with GM3. The binding of W284A and F285A mutant enzymes to mouse macrophages decreased markedly in comparison to the binding by wild-type enzymes. Therefore, we conclude that GM3 is the primary cellular receptor for Bc-SMase, and that the beta-hairpin region is the tethering region for gangliosides. Crown Copyright (C) 2013 Published by Elsevier Inc. All rights reserved. |
  | Date Added | 10/11/2013, 10:29:15 AM |
  | Modified | 11/11/2013, 3:47:01 PM |

  ### Tags:

  - Bacillus cereus
  - beta-Hairpin
  - GM3
  - Sphingomyelinase
  - Tethering

  ### Notes:

  - Study mechanism of membrane binding by Sphingomyelinase (SMase), which catalyzes the hydrolysis of sphingomyelin (SM) to produce phosphocholine and ceramide and is widely distributed throughout eukaryotes and prokaryotes.

    Experimental and computational study.

    How SCOP is used:

    Retrieve superfamily classification for SMase: DNase 1-like superfamily.

    SCOP reference:

    Bacterial SMase has been confirmed to be a member of the DNase 1-like folding superfamily [15–17], and the amino acid residues in the putative active site of bacterial SMase were found to be geometrically identical to the corresponding amino acid residues of enzymes in the DNase 1-like folding superfamily.

  ### Attachments

  - 1-s2.0-S0006291X13000387-main.pdf
- ## Backbone fractal dimension and fractal hybrid orbital of protein structure

  |  |  |
  | --- | --- |
  | Type | Journal Article |
  | Author | Xin Peng |
  | Author | Wei Qi |
  | Author | Mengfan Wang |
  | Author | Rongxin Su |
  | Author | Zhimin He |
  | URL | http://www.sciencedirect.com/science/article/pii/S1007570413002074 |
  | Publication | Communications in Nonlinear Science and Numerical Simulation |
  | Date | 2013 |
  | Accessed | 9/23/2013, 10:18:21 AM |
  | Library Catalog | Google Scholar |
  | Date Added | 10/11/2013, 10:29:15 AM |
  | Modified | 2/20/2014, 4:07:28 PM |

  ### Tags:

  - Backbone fractal dimension
  - Hybrid orbital model
  - Local fractal dimension
  - Protein

  ### Notes:

  - **Paper Summary**

    They analysized the fractal geometry (in a "local" and "backbone" dimensions) of 750 proteins, all from four different SCOP classes (alpha, beta, alpha/beta, alpha+beta). This was used for structural analysis (examining the hybrid atomic orbitals- since this is associated with the bond angles and conformation of the molecule) of the proteins.

    "Fractal theory is a very active mathematic branch of modern nonlinear science, which has been used widely to describe  
    irregular and non-differentiable geometric shapes existing in both natural world and man-made substance."

    **SCOP Use**

    Study differences in self-similarity in different SCOP classes.  Used SCOP to get structural class of the proteins.

    **SCOP Reference**

    In this paper we are mainly interested in investigating the self-similarity of 750 different protein molecules. These proteins  
    are selected from the Protein Data Bank [23] with X-ray diffraction as the structure elucidation method. We have filtered  
    out proteins exceeding 30% sequence identity and proteins that have ligands, RNA, or DNA. We have also dismissed  
    incomplete data sets that contained only the data of a-carbons. Moreover we have also removed the proteins whose sequence  
    length are less than 250 amino acids, because those are too short to be considered as fractals. The class was determined  
    according to the SCOP database [33].

  ### Attachments

  - 1-s2.0-S1007570413002074-main.pdf
  - Snapshot
- ## Bacterial GRAS domain proteins throw new light on gibberellic acid response mechanisms

  |  |  |
  | --- | --- |
  | Type | Journal Article |
  | Author | Dapeng Zhang |
  | Author | Lakshminarayan M. Iyer |
  | Author | L. Aravind |
  | Volume | 28 |
  | Issue | 19 |
  | Pages | 2407-2411 |
  | Publication | Bioinformatics |
  | ISSN | 1367-4803 |
  | Date | OCT 1 2012 |
  | Extra | WOS:000309687500001 |
  | DOI | 10.1093/bioinformatics/bts464 |
  | Abstract | Gibberellic acids (GAs) are key plant hormones, regulating various aspects of growth and development, which have been at the center of the 'green revolution'. GRAS family proteins, the primary players in GA signaling pathways, remain poorly understood. Using sequence-profile searches, structural comparisons and phylogenetic analysis, we establish that the GRAS family first emerged in bacteria and belongs to the Rossmann fold methyltransferase superfamily. All bacterial and a subset of plant GRAS proteins are likely to function as small-molecule methylases. The remaining plant versions have lost one or more AdoMet (SAM)-binding residues while preserving their substrate-binding residues. We predict that GRAS proteins might either modify or bind small molecules such as GAs or their derivatives. |
  | Date Added | 2/20/2014, 12:24:01 PM |
  | Modified | 2/20/2014, 12:24:01 PM |

  ### Notes:

  - Gibberellic acids (GAs) are key plan hormones.  GRAS family proteins are the primary players n GA signaling pathways.  Perform a bioinformatics study of the GRAS family.

    How SCOP is used:

    Look up fold classifications of STAT-type DNA-binding domain and SH2 domain.

    SCOP reference:

    The STAT-type DNA-binding domains adopt a cytochrome f-like ⬚⬚-sandwich fold, whereas the SH2 domain adopts a ⬚⬚-barrel structure (Andreeva et al., 2008), both of which are incompatible with the predicted second- ary structure of the GRAS domain.

  ### Attachments

  - Bioinformatics-2012-Zhang-2407-11.pdf
- ## BALBES: a molecular-replacement pipeline

  |  |  |
  | --- | --- |
  | Type | Journal Article |
  | Author | Fei Long |
  | Author | Alexei A Vagin |
  | Author | Paul Young |
  | Author | Garib N Murshudov |
  | Volume | 64 |
  | Issue | Pt 1 |
  | Pages | 125-132 |
  | Publication | Acta crystallographica. Section D, Biological crystallography |
  | ISSN | 0907-4449 |
  | Date | Jan 2008 |
  | Extra | PMID: 18094476 |
  | Journal Abbr | Acta Crystallogr. D Biol. Crystallogr. |
  | DOI | 10.1107/S0907444907050172 |
  | Library Catalog | NCBI PubMed |
  | Language | eng |
  | Abstract | The number of macromolecular structures solved and deposited in the Protein Data Bank (PDB) is higher than 40 000. Using this information in macromolecular crystallography (MX) should in principle increase the efficiency of MX structure solution. This paper describes a molecular-replacement pipeline, BALBES, that makes extensive use of this repository. It uses a reorganized database taken from the PDB with multimeric as well as domain organization. A system manager written in Python controls the workflow of the process. Testing the current version of the pipeline using entries from the PDB has shown that this approach has huge potential and that around 75% of structures can be solved automatically without user intervention. |
  | Short Title | BALBES |
  | Date Added | 10/11/2013, 10:29:15 AM |
  | Modified | 10/11/2013, 10:29:15 AM |

  ### Tags:

  - Algorithms
  - Computer Simulation
  - Crystallography, X-Ray
  - Databases, Protein
  - Models, Molecular
  - Protein Structure, Tertiary
  - Software

  ### Notes:

  - BALBES is a molecular-replacement pipeline.  Presents the workflow and interface for BALBES.

    How SCOP is used:

    Did not use SCOP data.  Instead used their own domain definitions.

    Listed in table 4 as an additional resource that is cross-linked.

    SCOP reference:

    Two areas relevant to this paper are the classification of domains [CATH (Pearl et al., 2005); SCOP (Murzin et al., 1995)] and the extraction of biological oligomers from crystal structures (Krissinel & Henrick, 2005). While the domains defined by both CATH and SCOP are extremely useful for the biological community in general, our attempts to use them for molecular replacement did not produce consistent results. Therefore, we undertook to redefine the domains so that they could be used for molecular replacement and structure solution routinely and consistently.

  ### Attachments

  - balbes-2008.pdf
  - PubMed entry
- ## BAYESIAN ALIGNMENT OF SIMILARITY SHAPES

  |  |  |
  | --- | --- |
  | Type | Journal Article |
  | Author | Kanti V. Mardia |
  | Author | Christopher J. Fallaize |
  | Author | Stuart Barber |
  | Author | Richard M. Jackson |
  | Author | Douglas L. Theobald |
  | Volume | 7 |
  | Issue | 2 |
  | Pages | 989-1009 |
  | Publication | Annals of Applied Statistics |
  | ISSN | 1932-6157 |
  | Date | JUN 2013 |
  | Extra | WOS:000322829800016 |
  | DOI | 10.1214/12-AOAS615 |
  | Abstract | We develop a Bayesian model for the alignment of two point configurations under the full similarity transformations of rotation, translation and scaling. Other work in this area has concentrated on rigid body transformations, where scale information is preserved, motivated by problems involving molecular data; this is known as form analysis. We concentrate on a Bayesian formulation for statistical shape analysis. We generalize the model introduced by Green and Mardia [Biometrika 93 (2006) 235-254] for the pairwise alignment of two unlabeled configurations to full similarity transformations by introducing a scaling factor to the model. The generalization is not straightforward, since the model needs to be reformulated to give good performance when scaling is included. We illustrate our method on the alignment of rat growth profiles and a novel application to the alignment of protein domains. Here, scaling is applied to secondary structure elements when comparing protein folds; additionally, we find that one global scaling factor is not in general sufficient to model these data and, hence, we develop a model in which multiple scale factors can be included to handle different scalings of shape components. |
  | Date Added | 2/13/2014, 4:13:17 PM |
  | Modified | 3/7/2014, 12:09:14 PM |
- ## BCL::Score-Knowledge Based Energy Potentials for Ranking Protein Models Represented by Idealized Secondary Structure Elements

  |  |  |
  | --- | --- |
  | Type | Journal Article |
  | Author | Nils Woetzel |
  | Author | Mert Karakas |
  | Author | Rene Staritzbichler |
  | Author | Ralf Mueller |
  | Author | Brian E. Weiner |
  | Author | Jens Meiler |
  | Volume | 7 |
  | Issue | 11 |
  | Pages | e49242 |
  | Publication | Plos One |
  | ISSN | 1932-6203 |
  | Date | NOV 16 2012 |
  | Extra | WOS:000311885300021 |
  | DOI | 10.1371/journal.pone.0049242 |
  | Abstract | The topology of most experimentally determined protein domains is defined by the relative arrangement of secondary structure elements, i.e. alpha-helices and beta-strands, which make up 50-70% of the sequence. Pairing of beta-strands defines the topology of beta-sheets. The packing of side chains between alpha-helices and beta-sheets defines the majority of the protein core. Often, limited experimental datasets restrain the position of secondary structure elements while lacking detail with respect to loop or side chain conformation. At the same time the regular structure and reduced flexibility of secondary structure elements make these interactions more predictable when compared to flexible loops and side chains. To determine the topology of the protein in such settings, we introduce a tailored knowledge-based energy function that evaluates arrangement of secondary structure elements only. Based on the amino acid C-beta atom coordinates within secondary structure elements, potentials for amino acid pair distance, amino acid environment, secondary structure element packing, beta-strand pairing, loop length, radius of gyration, contact order and secondary structure prediction agreement are defined. Separate penalty functions exclude conformations with clashes between amino acids or secondary structure elements and loops that cannot be closed. Each individual term discriminates for native-like protein structures. The composite potential significantly enriches for native-like models in three different databases of 10,000-12,000 protein models in 80-94% of the cases. The corresponding application, "BCL:: ScoreProtein," is available at www.meilerlab.org. |
  | Date Added | 2/20/2014, 12:24:01 PM |
  | Modified | 3/7/2014, 12:10:33 PM |

  ### Notes:

  - Present energy function to aid in choosing the best model for protein structure prediction.

    How SCOP/CATH is used:

    Background on protein structure classification.  Describe why they have used a non-redundant data set curated using PISCES, rather than curating using SCOP or CATH data.

    SCOP reference:

    Divergent Databank of High Resolution Crystal Structures

    Statistics have been derived from a divergent high resolution subset of the protein data bank (PDB) which was generated using the protein sequence culling server ‘‘PISCES’’ [42]. With a sequence identity limit of 25%, resolutions up to 2.0 A ̊ , a maximum R-value of 0.3, sequence lengths of 40 residues minimum only X-ray structures have been culled from the PDB. This guarantees that similar sequences are not over represented, introducing a bias to proteins that are amenable to crystallography or are of higher interest in the scientific fields. All membrane proteins have been excluded. The resulting databank has 4,379 chains in 3,409 PDB entries. This approach to create the representative protein database might leave multiple members of the more popular fold groups thereby over-representing certain secondary structure packing motifs. An alternative approach would be a non-redundant fold databank created from SCOP [43] or CATH [44] classifications. Our rational for the first approach is that a non-redundant fold database would not cover the diversity of amino acid environments and interactions that are found within similar folds of diverse sequence worsening the statistics of the amino acid centric potentials. Further we argue that secondary structure packing motifs are conserved beyond the boundaries of individual folds. The statistics describing these packing interactions should therefore not be biased by occasional repetition of one fold group.

  ### Attachments

  - journal.pone.0049242.pdf
- ## BeEP Server: using evolutionary information for quality assessment of protein structure models

  |  |  |
  | --- | --- |
  | Type | Journal Article |
  | Author | Nicolas Palopoli |
  | Author | Esteban Lanzarotti |
  | Author | Gustavo Parisi |
  | Volume | 41 |
  | Issue | W1 |
  | Pages | W398–W405 |
  | Publication | Nucleic Acids Research |
  | Date | July 2013 |
  | DOI | 10.1093/nar/gkt453 |
  | Abstract | The BeEP Server (http://www.embnet.qb.fcen.uba.ar/embnet/beep.php) is an online resource aimed to help in the endgame of protein structure prediction. It is able to rank submitted structural models of a protein through an explicit use of evolutionary information, a criterion differing from structural or energetic considerations commonly used in other assessment programs. The idea behind BeEP (Best Evolutionary Pattern) is to benefit from the substitution pattern derived from structural constraints present in a set of homologous proteins adopting a given protein conformation. The BeEP method uses a model of protein evolution that takes into account the structure of a protein to build site-specific substitution matrices. The suitability of these substitution matrices is assessed through maximum likelihood calculations from which position-specific and global scores can be derived. These scores estimate how well the structural constraints derived from each structural model are represented in a sequence alignment of homologous proteins. Our assessment on a subset of proteins from the Critical Assessment of techniques for protein Structure Prediction (CASP) experiment has shown that BeEP is capable of discriminating the models and selecting one or more native-like structures. Moreover, BeEP is not explicitly parameterized to find structural similarities between models and given targets, potentially helping to explore the conformational ensemble of the native state. |
  | Date Added | 3/7/2014, 12:08:00 PM |
  | Modified | 3/7/2014, 12:08:00 PM |
- ## beta-Bulges: Extensive structural analyses of beta-sheets irregularities

  |  |  |
  | --- | --- |
  | Type | Journal Article |
  | Author | Pierrick Craveur |
  | Author | Agnel Praveen Joseph |
  | Author | Joseph Rebehmed |
  | Author | Alexandre G. de Brevern |
  | Volume | 22 |
  | Issue | 10 |
  | Pages | 1366-1378 |
  | Publication | Protein Science |
  | ISSN | 0961-8368; 1469-896X |
  | Date | OCT 2013 |
  | Extra | WOS:000325087000008 |
  | DOI | 10.1002/pro.2324 |
  | Abstract | beta-Sheets are quite frequent in protein structures and are stabilized by regular main-chain hydrogen bond patterns. Irregularities in -sheets, named -bulges, are distorted regions between two consecutive hydrogen bonds. They disrupt the classical alternation of side chain direction and can alter the directionality of -strands. They are implicated in protein-protein interactions and are introduced to avoid -strand aggregation. Five different types of -bulges are defined. Previous studies on -bulges were performed on a limited number of protein structures or one specific family. These studies evoked a potential conservation during evolution. In this work, we analyze the -bulge distribution and conservation in terms of local backbone conformations and amino acid composition. Our dataset consists of 66 times more -bulges than the last systematic study (Chan et al. Protein Science 1993, 2:1574-1590). Novel amino acid preferences are underlined and local structure conformations are highlighted by the use of a structural alphabet. We observed that -bulges are preferably localized at the N- and C-termini of -strands, but contrary to the earlier studies, no significant conservation of -bulges was observed among structural homologues. Displacement of -bulges along the sequence was also investigated by Molecular Dynamics simulations. |
  | Date Added | 2/12/2014, 1:36:22 PM |
  | Modified | 2/12/2014, 1:36:22 PM |

  ### Notes:

  - Study of irregularities in beta-sheets, called beta-bulges.

    How SCOP is used:

    General study on protein structure.  Examine the distribution of beta-bulges across different SCOP classes.

    SCOP reference:

    Results

    Analysis of the secondary structures

    About 12,132 structures, representing 2,180,241 amino-acids, were used for this study, out of 16,712 structures in the SCOP dataset filtered at 95% sequence identity. The remaining protein chains comprise structures solved by Nuclear Magnetic Resonance, involve nonstandard PDB file formats and those structures for which PROMOTIF failed to assign backbone conformations. Table II summarizes the secondary structure assignment for the SCOP95 dataset. Three structural classes that is, a/b, a 1 b, and all-b represent a quarter of our dataset each, while all-a represents only 16.8% of the protein chains. Secondary structure assignment resulted in 35.1% of residues in a-helical conformation, 18.5% in b-sheets and rest 46.4% in coils. Similar results were found for SCOP40 dataset, and the secondary structure distributions are in agreement with previ- ous studies.4,32,33

    ...

    b-Bulge in SCOP classes  
     As seen in Tables II and Supporting Information S4, the distribution of b-bulges is not similar in all SCOP classes. b-Bulges were even found in the all-a class which by definition has a low b-sheet content. About 30.7% of these b-bulges are entirely found inside b-sheets and are mainly antiparallel G1 b-

    bulges (54.9%). As a/b class is mainly composed of parallel b-sheets, it is expected to have the highest content of parallel Special, Wide, Bent, and Classic b-bulges (3.4, 5.0, 2.9, and 20.2%, respectively). a 1 b and all-b classes exhibit roughly the same behavior with the dominance of antiparallel Classic b-bulges (60.5 and 55.2%, respectively), a significant representation of antiparallel G1 b-bulges (31.0 and 35.0%, respectively) and a limited number of b- bulges outside b-strand (⬚⬚15%), like a/b class.

    The multidomain protein and small protein classes have similar distributions with ⬚⬚30% of b- bulges in b-strands, 30% outside b-strands and 38% are partly in b-strands. The membrane associated class has lower number of b-bulges, but has the highest number of antiparallel Wide b-bulge (9.3%, which is twice the frequency in the other classes); the other 5 types of b-bulges were never observed.

    ...

    Protein superimpositions

    Analysis of b-bulges in specific protein families, for example, the WD40 family28 and the immunoglobulin family,16 has suggested that b-bulges could be more conserved than other parts of protein structures. About 950,793 structure superimpositions were car- ried out using iPBA program. Proteins placed together in the same fold category may not have a common evolutionary origin: the structural similar- ities could just arise from the physico-chemical prop- erties of proteins favoring certain packing arrangements and chain topologies. The average GDT\_TS score is 33.25 with a peak at 31 (see Sup- porting Information Fig. S2). Even though superim- positions were performed at the level of SCOP fold, a non negligible proportion of structural alignments shares a very low GDT\_TS, that is, some structures, classified in same SCOP fold cannot be properly superimposed. Hence, we selected only superimposi- tions with GDT\_TS score better than 15, a threshold already used in a previous study38; corresponding to an average RMSD lower than 2.69A ̊ (see Supporting Information Fig. S3), reflecting superimpositions of structures sharing similar global conformation. Con- sequently, 716,346 superimpositions were selected.

    ...

    Structural datasets

    Two sets of protein structures were extracted from Protein Data Bank47 based on the ASTRAL SCOP dataset,45 filtered at 40% and 95% sequence identity. The proteins were classified into folds and classes based on the SCOP classification.48 All NMR struc- tures were excluded from the analysis. SCOP95 dataset contained 16,712 structures representing 1,195 folds and 7 classes.

  ### Attachments

  - pro2324.pdf
- ## Beta-strand interfaces of non-dimeric protein oligomers are characterized by scattered charged residue patterns

  |  |  |
  | --- | --- |
  | Type | Journal Article |
  | Author | Giovanni Feverati |
  | Author | Mounia Achoch |
  | Author | Jihad Zrimi |
  | Author | Laurent Vuillon |
  | Author | Claire Lesieur |
  | Volume | 7 |
  | Issue | 4 |
  | Pages | e32558 |
  | Publication | PloS one |
  | ISSN | 1932-6203 |
  | Date | 2012 |
  | Extra | PMID: 22496732 |
  | Journal Abbr | PLoS ONE |
  | DOI | 10.1371/journal.pone.0032558 |
  | Library Catalog | NCBI PubMed |
  | Language | eng |
  | Abstract | Protein oligomers are formed either permanently, transiently or even by default. The protein chains are associated through intermolecular interactions constituting the protein interface. The protein interfaces of 40 soluble protein oligomers of stœchiometries above two are investigated using a quantitative and qualitative methodology, which analyzes the x-ray structures of the protein oligomers and considers their interfaces as interaction networks. The protein oligomers of the dataset share the same geometry of interface, made by the association of two individual β-strands (β-interfaces), but are otherwise unrelated. The results show that the β-interfaces are made of two interdigitated interaction networks. One of them involves interactions between main chain atoms (backbone network) while the other involves interactions between side chain and backbone atoms or between only side chain atoms (side chain network). Each one has its own characteristics which can be associated to a distinct role. The secondary structure of the β-interfaces is implemented through the backbone networks which are enriched with the hydrophobic amino acids favored in intramolecular β-sheets (MCWIV). The intermolecular specificity is provided by the side chain networks via positioning different types of charged residues at the extremities (arginine) and in the middle (glutamic acid and histidine) of the interface. Such charge distribution helps discriminating between sequences of intermolecular β-strands, of intramolecular β-strands and of β-strands forming β-amyloid fibers. This might open new venues for drug designs and predictive tool developments. Moreover, the β-strands of the cholera toxin B subunit interface, when produced individually as synthetic peptides, are capable of inhibiting the assembly of the toxin into pentamers. Thus, their sequences contain the features necessary for a β-interface formation. Such β-strands could be considered as 'assemblons', independent associating units, by homology to the foldons (independent folding unit). Such property would be extremely valuable in term of assembly inhibitory drug development. |
  | Date Added | 10/11/2013, 10:29:15 AM |
  | Modified | 10/11/2013, 10:29:15 AM |

  ### Tags:

  - Amino Acids
  - Electrophoresis, Polyacrylamide Gel
  - Humans
  - Hydrogen Bonding
  - Hydrophobic and Hydrophilic Interactions
  - Models, Molecular
  - Peptide Fragments
  - Protein Folding
  - Protein Multimerization
  - Proteins
  - Protein Structure, Secondary
  - Software

  ### Notes:

  - Computational study on interfaces of 40 oligomers of varying stoechiometries (i.e. dimers, trimers, etc.).

    How SCOP is used:

    Use SCOP to get superfamilies for their data set of 40 proteins.  Just provide the information but do not provide any other analysis that relies on SCOP domains or classification.

    SCOP reference:

    Properties of the whole chain proteins of the dataset

    The protein oligomers are produced by organisms from the three super-kingdoms of life with 2% of archea, 75% of bacteria and 23% of eukaryotes (Table S1). For comparison, there are 8%, 54% and 38% of archea, bacteria and eukaryotic protein oligomers for the stœchiometries from 3 to 8 in the PDB. The atomic structures (PDB) of the protein oligomers of the dataset are shown in figure 2 to illustrate the diversity of their quaternary, tertiary (folds) and secondary structures. The folds are also represented by the SCOP superfamily codes in Table S1 [42].

  ### Attachments

  - journal.pone.0032558.pdf
  - PubMed entry
- ## BetaSuperposer: superposition of protein surfaces using beta-shapes

  |  |  |
  | --- | --- |
  | Type | Journal Article |
  | Author | Jae-Kwan Kim |
  | Author | Deok-Soo Kim |
  | Volume | 30 |
  | Issue | 6 |
  | Pages | 684-700 |
  | Publication | Journal of Biomolecular Structure & Dynamics |
  | ISSN | 0739-1102 |
  | Date | 2012 |
  | Extra | WOS:000309124500006 |
  | DOI | 10.1080/07391102.2012.689700 |
  | Abstract | The comparison between two protein structures is important for understanding a molecular function. In particular, the comparison of protein surfaces to measure their similarity provides another challenge useful for studying molecular evolution, docking, and drug design. This paper presents an algorithm, called the BetaSuperposer, which evaluates the similarity between the surfaces of two structures using the beta-shape which is a geometric structure derived from the Voronoi diagram of molecule. The algorithm performs iterations of mix-and-match between the beta-shapes of two structures for the optimal superposition from which a similarity measure is computed, where each mix-and-match step attempts to solve an NP-hard problem. The devised heuristic algorithm based on the assignment problem formulation quickly produces a good superposition and an assessment of similarity. The BetaSuperposer was fully implemented and benchmarked against popular programs, the Dali and the Click, using the SCOP models. The BetaSuperposer is freely available to the public from the Voronoi Diagram Research Center (http://voronoi.hanyang.ac.kr). |
  | Date Added | 2/20/2014, 12:24:01 PM |
  | Modified | 10/8/2014, 12:50:29 PM |

  ### Notes:

  - Present a method for protein structure comparison.

    How SCOP is used:  
    Evaluate method on non-redundant data set of 24 structures, curated using class, superfamily, and family.

    SCOP reference:

    The BetaSuperposer was fully implemented and benchmarked against popular programs, the Dali and the Click, using the SCOP models.

    ...

    The BetaSuperposer was fully implemented and benchmarked against the popular programs Dali (Holm & Park, 2000) and Click (Nguyen, Tan, & Madhusud- han, 2011) using a set of 24 structures from the SCOP database.

    ...

    7.3. Benchmark test BetaSuperposer has two system parameters which are

    The test set for the benchmark test consisted of 24 PDB structures from four different superfamilies in the SCOP database so that each of the four classes (i.e. alpha, beta, alpha/beta, and alpha+beta) had a representative super- family in the test set. We chose two different families from each superfamily and three different structures from each family, where each structure was a chain in the cor- responding protein. The details of the selected structures (e.g. the name of the superfamilies, the PDB IDs, the resolution, and the numbers of residues and atoms) are given in the Table 1. The structure 1G84 (Test Code: 9) does not have the resolution value, because it was deter- mined from an NMR data. With this dataset, we con- ducted all pairwise comparisons using the three programs: the BetaSuperposer, the Dali, and the Click.

  ### Attachments

  - 07391102%2E2012%2E689700.pdf
- ## Between-strand disulfides: forbidden disulfides linking adjacent beta-strands

  |  |  |
  | --- | --- |
  | Type | Journal Article |
  | Author | Naomi L. Haworth |
  | Author | Merridee A. Wouters |
  | Volume | 3 |
  | Issue | 46 |
  | Pages | 24680-24705 |
  | Publication | Rsc Advances |
  | ISSN | 2046-2069 |
  | Date | 2013 |
  | Extra | WOS:000326745100106 |
  | DOI | 10.1039/c3ra42486c |
  | Abstract | Between-strand disulfides (BSDs) connect cysteine (Cys) residues across adjacent strands of beta-sheets. There are four BSD types which can be found in regular beta-structure: CSDs, which link residues immediately opposite each other in the b-structure (residues i and j); ETDs, which connect Cys out of register by one residue (i and j +/- 1); BDDs, which join Cys at positions i and j +/- 2; and BFDs, which link residues i and j +/- 3. Formation of these disulfides was initially predicted to be forbidden, producing too much local strain in the protein fold. However, BSDs do exist in nature. Significantly, their high levels of strain allow them to be involved in redox processes under physiological conditions. Here we characterise BSD motifs found in the Protein Data Bank (PDB), discussing important intrinsic factors, such as the disulfide conformation and torsional strain, and extrinsic factors, such as the influence of the beta-sheet environment on the disulfide and vice versa. We also discuss the biological importance of BSDs, including the prevalence of non-homologous examples in the PDB, the conservation of BSD motifs amongst related proteins (BSD clusters) and experimental evidence for BSD redox activity. For clusters of homologous BSDs we present detailed data of the disulfide properties and the variations of these properties amongst the "redundant" structures. Identification of disulfides with the potential to be involved in biological redox processes via the analysis of these data will provide important insights into the function and mechanism of BSD-containing proteins. Characterisation of thiol-based redox signalling pathways will lead to significant breakthroughs in understanding the molecular basis of oxidative stress and associated pathways, such as ageing and neurodegenerative diseases. |
  | Date Added | 2/20/2014, 12:24:01 PM |
  | Modified | 2/20/2014, 12:24:01 PM |

  ### Tags:

  - coverage
  - likely ASTRAL

  ### Notes:

  - Computational study of folds containing between-strand disulfide bonds.

    How SCOP is used:

    Use SCOP to curate a non-redundant data set of proteins with BSDs.  Use fold information to cluster domains.

    SCOP reference:

    Heterogeneity of other disulde properties can also provide indications of disulde functionality. One of the best studied protein families containing a BSD is the eukaryotic trypsin-like serine protease (eTLSP) family. This protein family illustrates the diversity which can be seen amongst BSDs belonging to the same cluster. In the SCOP (Structural Classication of Proteins) database,91 eTLSPs have a trypsin-like serine protease fold (Fold ID: 50493) consisting of two six-stranded b-barrels. There are 67 unique protein sequences belonging to the eTLSP family (SCOP family ID: 50514) in release 1.75 of the SCOP database. In 49 of these proteins an aCSDn lies just inside the mouth of one of the b-barrels, linking residues 136 and 201 of the common numbering scheme. There are 931 disuldes belonging to this cluster in our dataset, including some from proteins which are not yet classied in the SCOP database. 12 structures from proteins of different function within the family are super- imposed in Fig. 10A. The disuldes all align extremely well, differing chiey in the number of residues in the b-hairpin turn between Cys 201 and its non-CSD b-partner. These structures do vary, however, in the LonE level of the disuldes. Most disuldes (including that of trypsin) are end-aCSDns, however those of kallikrein A (black), granzyme A (dark red) and b-tryptase (red) are true-CSDs, while coagulation factor XI (yellow) has a b-bridge disulde. In some cases (such as in human b-tryptase) the level of LonE varies amongst the different structures of the same protein ($30% of these disuldes are true-aCSDns, $60% end- aCSDns). Although the function of the disulde is unknown, its presence in a subset of eTLSPs correlates with a proline at position 225.92 The disulde and proline are not found in blood coagulation eTLSPs activated by sodium.92

    ...

    Construction of a non-redundant dataset–disulde clustering As part of our analysis, populations of the various BSD motifs in the PDB are reported. To control for selection effects arising from over-representation of some proteins in the PDB, we grouped all homologous BSDs into clusters. **The main tool used to collate the various structures was SCOP.****91 The atle for SCOP release 1.75 was downloaded from http://scop.mrc-lmb.cam.ac.uk/scop/ parse/index.html for this purpose. This atle was queried by our custom program, Disulde, to identify the SCOP code(s) for the domain(s) in which each disulde is found (or between which it bridges). In the rst pass of the clustering process, structures belonging to the same SCOP family (i.e. have the same SCOP code for the rst ve levels, differing only in the sixth) and adopting the same disul****de motif were regarded as redundant.** There are some cases, however, where a protein has more than one unique instance of a particular disulde motif (for example, inuenza neuraminidase has four unique true-aCSDns arranged around the sialic acid binding site22). In order to split these into separate clusters, the residue sequence in the region of the disulde, the numbering of the Cys residues and the number of residues between the Cys were compared.

  ### Attachments

  - c3ra42486c.pdf
- ## Beyond BLASTing: Tertiary and quaternary structure analysis helps identify Major Vault Proteins

  |  |  |
  | --- | --- |
  | Type | Journal Article |
  | Author | Toni K. Daly |
  | Author | Andrew J. Sutherland-Smith |
  | Author | David Penny |
  | URL | http://gbe.oxfordjournals.org/content/5/1/217.short |
  | Volume | 5 |
  | Issue | 1 |
  | Pages | 217–232 |
  | Publication | Genome biology and evolution |
  | Date | 2013 |
  | Accessed | 9/23/2013, 10:14:36 AM |
  | Library Catalog | Google Scholar |
  | Short Title | Beyond BLASTing |
  | Date Added | 10/11/2013, 10:29:15 AM |
  | Modified | 3/7/2014, 12:10:03 PM |

  ### Tags:

  - BLAST
  - homology modeling
  - I-TASSER
  - Naegleria gruberi
  - RosettaDock

  ### Notes:

  - Vaults are large oligomeric ribonucleoproteins conserved among a variety of species, many of which contain small untranslated RNAs (vault RNA [vtRNA]) (Stadler et al. 2009).  Use structural search to find vault proteins.

    How SCOP/CATH is used:

    Not using SCOP or CATH data.

    Reference SCOP and CATH when pointing out that remote homology may be detected with structure similarity.

    SCOP reference:

    Protein structure may sometimes be minimally affected by amino acid substitutions, and sequences with limited similarity may retain homologous folding patterns (Murzin et al. 1995; Orengo et al. 1997).

  ### Attachments

  - Genome Biol Evol-2013-Daly-217-32.pdf
  - [HTML] from oxfordjournals.org
  - Snapshot
- ## Binding pocket optimization by computational protein design

  |  |  |
  | --- | --- |
  | Type | Journal Article |
  | Author | Christoph Malisi |
  | Author | Marcel Schumann |
  | Author | Nora C Toussaint |
  | Author | Jorge Kageyama |
  | Author | Oliver Kohlbacher |
  | Author | Birte Höcker |
  | Volume | 7 |
  | Issue | 12 |
  | Pages | e52505 |
  | Publication | PloS one |
  | ISSN | 1932-6203 |
  | Date | 2012 |
  | Extra | PMID: 23300688 |
  | Journal Abbr | PLoS ONE |
  | DOI | 10.1371/journal.pone.0052505 |
  | Library Catalog | NCBI PubMed |
  | Language | eng |
  | Abstract | Engineering specific interactions between proteins and small molecules is extremely useful for biological studies, as these interactions are essential for molecular recognition. Furthermore, many biotechnological applications are made possible by such an engineering approach, ranging from biosensors to the design of custom enzyme catalysts. Here, we present a novel method for the computational design of protein-small ligand binding named PocketOptimizer. The program can be used to modify protein binding pocket residues to improve or establish binding of a small molecule. It is a modular pipeline based on a number of customizable molecular modeling tools to predict mutations that alter the affinity of a target protein to its ligand. At its heart it uses a receptor-ligand scoring function to estimate the binding free energy between protein and ligand. We compiled a benchmark set that we used to systematically assess the performance of our method. It consists of proteins for which mutational variants with different binding affinities for their ligands and experimentally determined structures exist. Within this test set PocketOptimizer correctly predicts the mutant with the higher affinity in about 69% of the cases. A detailed analysis of the results reveals that the strengths of PocketOptimizer lie in the correct introduction of stabilizing hydrogen bonds to the ligand, as well as in the improved geometric complemetarity between ligand and binding pocket. Apart from the novel method for binding pocket design we also introduce a much needed benchmark data set for the comparison of affinities of mutant binding pockets, and that we use to asses programs for in silico design of ligand binding. |
  | Date Added | 10/11/2013, 10:29:15 AM |
  | Modified | 12/18/2013, 12:29:45 PM |

  ### Tags:

  - Benchmarking
  - Binding Sites
  - Computational Biology
  - Drug Design
  - Ligands
  - Protein Binding
  - Proteins
  - Software

  ### Notes:

  - Present a novel method for computational design of protein-small ligand binding interfaces named PocketOptimizer.How SCOP is used:

    How SCOP is used:

    Used SCOP to help curate data set of 12 proteins, so that no two have the same fold.

    SCOP reference:

    Benchmark Set

    We compiled a set of twelve proteins with structural and experimental affinity data for the assessment of computational design methods for protein-ligand binding. For this, we system- atically searched the PDBbind database [34], which lists high quality crystal structures of protein-ligand complexes together with experimentally determined binding data. Each protein in our set has at least two mutational variants (usually the wild type and one or more mutants) accompanied by an affinity measure (the inhibitory constant Ki or dissociation constant Kd) for the same ligand. The positions of amino acids that differ between the variants are always located in the binding pocket or active site. For each protein, there is at least one crystal structure of a variant with the ligand, for ten of the twelve there are two or more crystal structures that allow us to compare a design model of a variant with the respective crystal structure. The proteins and ligands in our benchmark set are very diverse. All ligands are shown in Figure 2. Each protein in the set belongs to a different fold as defined by SCOP [35], underscoring their structural diversity. This diversity allows to test design methods on a wide range of problems and avoids bias. Table 1 lists the benchmark proteins and their associated data.

  ### Attachments

  - journal.pone.0052505.pdf
  - PubMed entry
- ## Binding sites in membrane proteins - Diversity, druggability and prospects

  |  |  |
  | --- | --- |
  | Type | Journal Article |
  | Author | Robert Adams |
  | Author | Catherine L. Worth |
  | Author | Stefan Guenther |
  | Author | Mathias Dunkel |
  | Author | Robert Lehmann |
  | Author | Robert Preissner |
  | Volume | 91 |
  | Issue | 4 |
  | Pages | 326-339 |
  | Publication | European Journal of Cell Biology |
  | ISSN | 0171-9335 |
  | Date | APR 2012 |
  | Extra | WOS:000302881700014 |
  | DOI | 10.1016/j.ejcb.2011.06.003 |
  | Abstract | The identification of novel drug targets is one of the major challenges in proteomics. Computational methods developed over the last decade have enhanced the process of drug design in both terms of time and quality. The main task is the design of selective compounds, which bind targets more specifically, dependent on the desired mode of action of the particular drug. This makes it necessary to create compounds, which either exhibit their functions on one single protein to exclude undesired cross-reactivity or to use the advantageous effect of less selective drugs that target numerous proteins and therefore exhibit their functions on whole protein classes. Main aspects in the assignment of interactions between ligands and putative targets involve the amino acid composition of the binding site, evolutionary conservation and similarity in sequence and structure of known targets. Similarities or differences within classified protein families can be the key to their function and give first hints to functional drug design. Hereby, binding site-based classification outnumbers sequence-based classifications since similar binding sites can also be found in more distant proteins. Membrane proteins are 'difficult targets', because of their special physicochemical characteristics and the general lack of structural information. Here, we describe recent advances in modeling methods dedicated to membrane proteins. Different descriptors of similarity between compounds and the similarity between binding sites are under development and elucidate important aspects like dynamics or entropy. The importance of computational drug design is undisputable. Nevertheless, the process of design is complicated by increasing complexity, which underlines the importance of accurate knowledge about the addressed target class(es) and particularly their binding sites. One main objective by considering named topics is to predict putative side effects and errant functions (off-target effects) of novel drugs, which requires a holistic (systems biology) view on drug-target-pathway relations. In the following, we give a brief summary about the recent discussion on drug-target interactions with emphasis on membrane proteins. (C) 2011 Elsevier GmbH. All rights reserved. |
  | Date Added | 2/20/2014, 12:24:01 PM |
  | Modified | 2/20/2014, 12:24:01 PM |

  ### Notes:

  - Review of research on binding sites in membrane proteins.

    How SCOP is used:

    Amongst other tools, describe PROCOGNATE database which assigned PDB ligands to protein domains in CATH, SCOP, and Pfam.

    SCOP reference:

    The focus of the PROCOGNATE database (Bashton et al., 2008) is on ligand–domain interactions of enzymes, rather than ligand–protein interactions. PDB ligands were assigned to protein domains by CATH (Dessailly et al., 2008), SCOP (Murzin et al., 1995) and Pfam (Finn et al., 2006).

  ### Attachments

  - 1-s2.0-S0171933511001099-main.pdf
- ## Bioinformatics and Molecular Dynamics Simulation Study of L1 Stalk Non-Canonical rRNA Elements: Kink-Turns, Loops, and Tetraloops

  |  |  |
  | --- | --- |
  | Type | Journal Article |
  | Author | Miroslav Krepl |
  | Author | Kamila Réblová |
  | Author | Jaroslav Koča |
  | Author | Jiří Šponer |
  | URL | http://pubs.acs.org/doi/full/10.1021/jp401482m |
  | Volume | 117 |
  | Issue | 18 |
  | Pages | 5540–5555 |
  | Publication | The Journal of Physical Chemistry B |
  | Date | 2013 |
  | Accessed | 9/23/2013, 10:14:50 AM |
  | Library Catalog | Google Scholar |
  | Short Title | Bioinformatics and Molecular Dynamics Simulation Study of L1 Stalk Non-Canonical rRNA Elements |
  | Date Added | 10/11/2013, 10:29:15 AM |
  | Modified | 10/11/2013, 10:29:15 AM |

  ### Notes:

  - Study of the "L1 Stalk"

    How SCOP is used:

    Look up domains and SCOP class classification of  protein of interest.

    SCOP reference:

    L1 Protein. In bacteria, the L1 protein is a multidomain protein 228 (or 229) amino acids long. It belongs in the ribo- somal protein L1 family.51 The first domain is larger and belongs to the class of α+β proteins. The second domain is smaller and sequentially interrupts (72−159 a.a.) the first domain. It belongs to the class of α/β proteins (Figure 7).52

  ### Attachments

  - jp401482m.pdf
  - Snapshot
- ## Bioinformatics and Systems Biology: bridging the gap between heterogeneous student backgrounds

  |  |  |
  | --- | --- |
  | Type | Journal Article |
  | Author | Sanne Abeln |
  | Author | Douwe Molenaar |
  | Author | K. Anton Feenstra |
  | Author | Huub C. J. Hoefsloot |
  | Author | Bas Teusink |
  | Author | Jaap Heringa |
  | Volume | 14 |
  | Issue | 5 |
  | Pages | 589-598 |
  | Publication | Briefings in Bioinformatics |
  | ISSN | 1467-5463; 1477-4054 |
  | Date | SEP 2013 |
  | Extra | WOS:000327435800008 |
  | DOI | 10.1093/bib/bbt023 |
  | Abstract | Teaching students with very diverse backgrounds can be extremely challenging. This article uses the Bioinformatics and Systems Biology MSc in Amsterdam as a case study to describe how the knowledge gap for students with heterogeneous backgrounds can be bridged. We show that a mix in backgrounds can be turned into an advantage by creating a stimulating learning environment for the students. In the MSc Programme, conversion classes help to bridge differences between students, by mending initial knowledge and skill gaps. Mixing students from different backgrounds in a group to solve a complex task creates an opportunity for the students to reflect on their own abilities. We explain how a truly interdisciplinary approach to teaching helps students of all backgrounds to achieve the MSc end terms. Moreover, transferable skills obtained by the students in such a mixed study environment are invaluable for their later careers. |
  | Date Added | 2/12/2014, 2:18:08 PM |
  | Modified | 2/12/2014, 2:18:08 PM |

  ### Notes:

  - Paper on educating students in bioinformatics and systems biology.

    How SCOP is used:

    Provide an example exercise for students that uses SCOP data.

    SCOP reference:

    Project in bioinformatics

    The aim of the Bioinformatics project is to bench- mark (PSI-)BLAST [2] using the SCOP [3], GO [4] and PFAM [5] databases. To ease this task somewhat, we have selected a set of 100 proteins, with a suffi- cient number of homologues, on which students can perform the benchmarks. We also provide skeleton scripts in Python to automatically retrieve BLAST results from the web server, parse BLAST, SCOP, GO and PFAM annotation and generate ROC curves. Students have to fill in the most crucial

    parts of the scripts to get them to work, while the I/O is already written for them. This way students can focus on the major learning objectives of the project:

    (i) Understanding the need for automation— Would such a test be possible by manually using the BLAST web server?

    (ii) Understanding the fuzziness of biological data— What does a benchmark mean (e.g. function GO, structure SCOP) and how reliable are the ‘true positives’?

    (iii) Relating the research question to the method— How does the performance of BLAST depend on the reference database used (students find this very difficult)?

    (iv) Analysing large scale data in a structured way— How to generate roc-plots?

    (v) Interpreting results—What parameter settings for BLAST work best, and why?

    Students have to write a report on the project within their group. A draft report can be handed in halfway for formative feedback. Students also compare results between different groups in a final presentation. Typically, this will reveal that seem- ingly minor changes in methodology and scoring can yield quite different results.

  ### Attachments

  - Brief Bioinform-2013-Abeln-589-98.pdf
- ## Bioinformatics and variability in drug response: a protein structural perspective

  |  |  |
  | --- | --- |
  | Type | Journal Article |
  | Author | Jennifer L. Lahti |
  | Author | Grace W. Tang |
  | Author | Emidio Capriotti |
  | Author | Tianyun Liu |
  | Author | Russ B. Altman |
  | URL | http://rsif.royalsocietypublishing.org/content/9/72/1409.short |
  | Volume | 9 |
  | Issue | 72 |
  | Pages | 1409–1437 |
  | Publication | Journal of The Royal Society Interface |
  | Date | 2012 |
  | Accessed | 9/20/2013, 1:19:35 PM |
  | Library Catalog | Google Scholar |
  | Short Title | Bioinformatics and variability in drug response |
  | Date Added | 2/20/2014, 12:24:01 PM |
  | Modified | 2/20/2014, 12:24:01 PM |

  ### Notes:

  - Review of structural bioinformatics studies of drug response.

    How SCOP is used:

    Get SCOP fold classification of drug targets from the PDB.  List the 10 most common folds.

    SCOP reference:

    Similarly, some protein tertiary structures are enriched among druggable proteins. Structural classifi- cation of drug targets from the Protein Data Bank (PDB [34]) using the Structural Classification of Proteins (SCOP) database [35] showed that the 10 most commonly observed folds are, nuclear receptor ligand- binding domain, ferredoxin-like, C-terminal domain, acid protease, NAD(P)-binding Rossmann-fold domain, TIM beta/alpha-barrel, prealbumin-like, dihydrofolate reductase-like, alpha/beta-hydrolase, and DNA/RNA polymerase.

  ### Attachments

  - [HTML] from royalsocietypublishing.org
  - J. R. Soc. Interface-2012-Lahti-1409-37.pdf
  - Snapshot
- ## BioJS: an open source JavaScript framework for biological data visualization

  |  |  |
  | --- | --- |
  | Type | Journal Article |
  | Author | John Gomez |
  | Author | Leyla J. Garcia |
  | Author | Gustavo A. Salazar |
  | Author | Jose Villaveces |
  | Author | Swanand Gore |
  | Author | Alexander Garcia |
  | Author | Maria J. Martin |
  | Author | Guillaume Launay |
  | Author | Rafael Alcantara |
  | Author | Noemi del-Toro |
  | Author | Marine Dumousseau |
  | Author | Sandra Orchard |
  | Author | Sameer Velankar |
  | Author | Henning Hermjakob |
  | Author | Chenggong Zong |
  | Author | Peipei Ping |
  | Author | Manuel Corpas |
  | Author | Rafael C. Jimenez |
  | Volume | 29 |
  | Issue | 8 |
  | Pages | 1103–1104 |
  | Publication | Bioinformatics |
  | Date | April 2013 |
  | DOI | 10.1093/bioinformatics/btt100 |
  | Abstract | BioJS is an open-source project whose main objective is the visualization of biological data in JavaScript. BioJS provides an easy-to-use consistent framework for bioinformatics application programmers. It follows a community-driven standard specification that includes a collection of components purposely designed to require a very simple configuration and installation. In addition to the programming framework, BioJS provides a centralized repository of components available for reutilization by the bioinformatics community. |
  | Date Added | 3/7/2014, 12:08:00 PM |
  | Modified | 3/7/2014, 12:08:00 PM |
- ## Biological and Chemical Databases for Research into the Composition of Animal Source Foods

  |  |  |
  | --- | --- |
  | Type | Journal Article |
  | Author | Piotr Minkiewicz |
  | Author | Jan Micinski |
  | Author | Malgorzata Darewicz |
  | Author | Justyna Bucholska |
  | Volume | 29 |
  | Issue | 4 |
  | Pages | 321-351 |
  | Publication | Food Reviews International |
  | ISSN | 8755-9129 |
  | Date | OCT 2 2013 |
  | Extra | WOS:000324015200001 |
  | DOI | 10.1080/87559129.2013.818011 |
  | Abstract | Bioinformatics and cheminformatics tools such as databases play an increasingly important role in modern science. They are commonly used in biological and medical sciences and they have many applications in food science. Databases listing biologically active compounds contribute to the design of functional foods and nutraceuticals. Databases of toxic or allergenic compounds are useful for food safety evaluations. This review presents examples of freely available databases (without obligatory registration) listing major groups of bioactive components. The main categories of compounds annotated in online databases include nucleic acids, proteins, peptides, carbohydrates, lipids, and low-molecular-weight compounds. Other categories of database entries are also discussed, including enzymes, allergens and their epitopes, flavor-enhancing compounds, as well as toxic substances. The last section of the review focuses on metabases, which are Web sites that create access to multiple databases. |
  | Date Added | 10/28/2013, 4:51:00 PM |
  | Modified | 10/28/2013, 4:51:00 PM |

  ### Notes:

  - Paper unavailable.
- ## Biological Sequence Classification with Multivariate String Kernels

  |  |  |
  | --- | --- |
  | Type | Journal Article |
  | Author | Pavel P. Kuksa |
  | Volume | 10 |
  | Issue | 5 |
  | Pages | 1201-1210 |
  | Publication | Ieee-Acm Transactions on Computational Biology and Bioinformatics |
  | Date | SEP-OCT 2013 |
  | Extra | WOS:000331461400012 |
  | DOI | 10.1109/TCBB.2013.15 |
  | Library Catalog | ISI Web of Knowledge |
  | Abstract | String kernel-based machine learning methods have yielded great success in practical tasks of structured/sequential data analysis. They often exhibit state-of-the-art performance on many practical tasks of sequence analysis such as biological sequence classification, remote homology detection, or protein superfamily and fold prediction. However, typical string kernel methods rely on the analysis of discrete 1D string data (e.g., DNA or amino acid sequences). In this paper, we address the multiclass biological sequence classification problems using multivariate representations in the form of sequences of features vectors (as in biological sequence profiles, or sequences of individual amino acid physicochemical descriptors) and a class of multivariate string kernels that exploit these representations. On three protein sequence classification tasks, the proposed multivariate representations and kernels show significant 15-20 percent improvements compared to existing state-of-the-art sequence classification methods. |
  | Date Added | 10/8/2014, 12:49:22 PM |
  | Modified | 12/10/2014, 2:19:04 AM |

  ### Tags:

  - amino acid physicochemical descriptors
  - Amino Acids
  - amino acid sequences
  - biochemistry
  - bioinformatics
  - biological sequence classification
  - biological sequence profiles
  - classification
  - data analysis
  - discrete 1D string data
  - DNA
  - DNA sequences
  - fold prediction
  - Kernel
  - kernel methods
  - learning (artificial intelligence)
  - Machine learning
  - molecular biophysics
  - molecular configurations
  - multiclass biological sequence classification problems
  - multivariate string kernels
  - Proteins
  - Protein sequence
  - protein sequence classification tasks
  - protein superfamily
  - Quantization
  - remote homology detection
  - Sequence Analysis
  - Sequential analysis
  - sequential data analysis
  - string kernel-based machine learning
  - structured data analysis

  ### Attachments

  - IEEE Xplore Abstract Record
  - IEEE Xplore Full Text PDF
- ## bioNerDS: exploring bioinformatics' database and software use through literature mining

  |  |  |
  | --- | --- |
  | Type | Journal Article |
  | Author | Geraint Duck |
  | Author | Goran Nenadic |
  | Author | Andy Brass |
  | Author | David L. Robertson |
  | Author | Robert Stevens |
  | URL | http://www.biomedcentral.com/1471-2105/14/194/ |
  | Volume | 14 |
  | Issue | 1 |
  | Pages | 194 |
  | Publication | BMC bioinformatics |
  | Date | 2013 |
  | Accessed | 9/20/2013, 1:16:44 PM |
  | Library Catalog | Google Scholar |
  | Short Title | bioNerDS |
  | Date Added | 10/11/2013, 10:29:15 AM |
  | Modified | 10/11/2013, 10:29:15 AM |

  ### Notes:

  - Implement a named entity recognition system for Bioinformatics articles to identify databases and software.

    How SCOP is used:

    Not using SCOP data.

    Measure the mentions per paper for all BMC Bioinformatics articles published in the past 10 years.  SCOP ranked in the top 10, with a mean mention rate of 0.5 mentions per paper.  It doesn't rank in the top 10 for documents.

    SCOP reference:

    Table 6 provides the results obtained on the men- tion level for the top 10 resources from each journal. It features many of the same resource names listed as in Table 5, but some notable changes are that KEGG now appears in both journals’ top 10 lists, and SCOP [46] and PubMed [47] now appear in BMC Bioinformatics.

  ### Attachments

  - [PDF] from biomedcentral.com
  - Snapshot
- ## Biophysical Characterization of the Membrane-proximal Ectodomain of the Receptor-type Protein-tyrosine Phosphatase Phogrin

  |  |  |
  | --- | --- |
  | Type | Journal Article |
  | Author | Martin Noguera |
  | Author | Maria Primo |
  | Author | Laura Sosa |
  | Author | Valeria Risso |
  | Author | Edgardo Poskus |
  | Author | Mario Ermacora |
  | URL | http://www.eurekaselect.com/112939/article |
  | Volume | 20 |
  | Issue | 9 |
  | Pages | 1009-1017 |
  | Publication | Protein & Peptide Letters |
  | ISSN | 09298665 |
  | Date | 2013-07-01 |
  | DOI | 10.2174/0929866511320090007 |
  | Accessed | 12/9/2014, 5:40:46 AM |
  | Library Catalog | CrossRef |
  | Language | en |
  | Date Added | 12/9/2014, 5:40:46 AM |
  | Modified | 12/9/2014, 5:40:46 AM |

  ### Attachments

  - Biophysical Characterization of the Membrane-proximal Ectodomain of the Receptor-type Protein-tyrosine Phosphatase Phogrin | BenthamScience
- ## BioShell Threader: protein homology detection based on sequence profiles and secondary structure profiles

  |  |  |
  | --- | --- |
  | Type | Journal Article |
  | Author | Dominik Gront |
  | Author | Maciej Blaszczyk |
  | Author | Piotr Wojciechowski |
  | Author | Andrzej Kolinski |
  | URL | http://nar.oxfordjournals.org/content/40/W1/W257.short |
  | Volume | 40 |
  | Issue | W1 |
  | Pages | W257–W262 |
  | Publication | Nucleic Acids Research |
  | Date | 2012 |
  | Accessed | 2/28/2013, 1:38:04 PM |
  | Library Catalog | Google Scholar |
  | Short Title | BioShell Threader |
  | Date Added | 10/11/2013, 10:20:13 AM |
  | Modified | 10/11/2013, 10:20:13 AM |

  ### Tags:

  - likely ASTRAL
  - likely ASTRAL domain structures
  - likely ASTRAL sequences
  - mention lack of coverage in SCOP

  ### Notes:

  - Present an extension to bioshell to supports homology modeling based on sequence and secondary structure alignment.

    Ran alignments against 4 chosen domains each from a set of 423 SCOP families

    Whether their method resulted in the same family assignment as in SCOP (78.8% of cases, when using sequence and structure alignment)

    How SCOP is used:

    SCOP is used in two ways:

    1. Domain template database: Downloaded all SCOP domain data from 1.75 and used to create a database of domain templates containing sequences and 3D structures.  Do not cite ASTRAL.

    2. To derive a training and testing data set.  Filter at the family-level.  Collect all families with at least four domains with <=30% sequence similarity.  From these 423 families, select 4 domains: 2 for training and 2 for testing.

    SCOP Reference:

    **Under ABSTRACT:**

    "Careful evaluation shows that there is nearly 80%  
    chance that the query sequence belongs to the  
    same SCOP family as the top scoring template."

    **Under MATERIALS AND METHODS:**

    "(iii) aforementioned four profiles for the query sequence are aligned against an in-house database of corresponding profiles created for SCOP (24) domains."

    Mention need for better coverage in SCOP: "Unfortunately the most recent SCOP 1.75 edition that has been released in 2009 covers only a half of today’s protein data bank (PDB) content. Therefore, the SCOP-based set of templates has been extended by the PDB chain entries, which are not in the SCOP database yet"

    "The optimization and validation of all these settings were performed on a carefully selected subset (30) of the SCOP database. From all the SCOP families, only those were selected that contained at least four protein domains, similar in no more than 30% to one another."

    "Such a selection procedure resulted in a set of 423 SCOP families. Two random out of each four domains were moved to a ‘train’ set used for parameter optimization. The other pair of domains was moved to a ‘‘test’’ set, necessary for final validation. Each of these two sets, therefore, comprises the same number of SCOP domains, always two domains from the same family. The optimization goal was to maximize the chance for finding the right family member for a query."

    **Under RESULTS**

    "The results presented in Figure 3 show that the alignment of profiles already enables correct SCOP Family assignment in 74.8% of cases, whereas combined with secondary structure alignment, it yields 78.8% correct predictions."

    **Citation**

    24. Murzin,A.G., Brenner,S.E., Hubbard,T. and Chothia,C. (1995)  
    SCOP: a structural classification of proteins database for the  
    investigation of sequences and structures. J. Mol. Biol., 247,  
    536–540.

  ### Attachments

  - gks555.pdf
  - [HTML] from oxfordjournals.org
  - PubMed entry
- ## Buried and accessible surface area control intrinsic protein flexibility

  |  |  |
  | --- | --- |
  | Type | Journal Article |
  | Author | Joseph A. Marsh |
  | URL | http://arxiv.org/abs/1306.2875 |
  | Publication | Journal of Molecular Biology |
  | Date | 2013 |
  | Accessed | 9/23/2013, 10:15:34 AM |
  | Library Catalog | Google Scholar |
  | Date Added | 10/11/2013, 10:29:15 AM |
  | Modified | 3/6/2014, 11:22:11 AM |

  ### Tags:

  - monomer
  - protein dynamics
  - protein folding
  - protein structure
  - solvent-accessible surface area

  ### Notes:

  - Computational study of buried and accessible surface area and flexibility.

    How SCOP is used:

    Compare measure of "relative solvent accessible surface area" across different SCOP classes.

    Download all monomers from the PDB.  Get SCOP domains and class from 1.75 and then remove any chains that are not in SCOP or not in the first 5 classes.

    SCOP reference:

    In contrast to intrinsic disorder, there does appear to be a clear association between Arel and the secondary structure propensities of different amino acids. In particular, glutamate, leucine and lysine have strong α-helical propensities, while glycine, tyrosine and asparagine are helix destabilizing62. Therefore, given this apparent correspondence between flexibility and secondary structure propensities, the Arel values of monomer crystal structures from different SCOP classes58 were compared (Figure 4B). Consistent with the sequence trend, this analysis reveals that all-α proteins are the most flexible (mean Arel = 1.050) and all-β proteins the most rigid (mean Arel = 0.984, P < 2.2 x 10^-16^, Wilcoxon test). The mixed classes (α+β and α/β) have Arel values intermediate to α and β, although α/β and β are nearly equal. This tendency for α proteins to be more flexible than β proteins maintained when alternate measures of flexibility are considered instead of Arel (Figure S2B). Furthermore, the sequence trends and correlations between Arel and different measures of flexibility are preserved when split by structural class, demonstrating that they are largely independent of secondary structure (Figure S3 and Table S5).

    ...

    Methods

    Monomer datasets

    All monomeric crystal structure biological units containing at least 30 residues were taken from Protein Data Bank on 2012-08-08, excluding backbone-only models. The set of high-confidence monomers (used for fitting the relationship in Figure 1A) included only monomers with SCOP 1.75 domain assignments58 in order to specifically exclude structures in the classes “membrane and cell surface proteins and peptides”, “small proteins”, “coiled coil proteins”, “low resolution protein structures”, “peptides” and “designed proteins”.
  - Computational study of protein flexibility using a measure of solvent accessible service area.

    How SCOP is used:

    Computationally measured flexibility of monomer structures classified by SCOP class.  Found all-alpha was more flexible than all-beta, and mixed classes were in the middle.

    SCOP reference:

    In contrast to intrinsic disorder, there does appear to be a clear association between Arel and the secondary-structure propensities of different amino acids. In particular, glutamate, leucine, and lysine have strong α-helical propensities, while glycine, tyrosine, and asparagine are helix destabilizing [57]. Therefore, given this apparent correspondence between flexibility and secondary-structure propen- sities, the Arel values of monomer crystal structures from different SCOP classes [58] were compared (Fig. 4b). Consistent with the sequence trend, this analysis reveals that all-α proteins are the most flexible (mean Arel = 1.050) and all-β proteins are the most rigid (mean Arel = 0.984, P b 2.2 × 10^−16^, Wilcoxon test). The mixed classes (α + β and α/β have Arel values intermediate to α and β, although α/β and β are nearly equal. This tendency for α proteins to be more flexible than β proteins was maintained when alternate measures of flexibility are considered instead of Arel (Fig. S2b). Furthermore, the sequence trends and correlations between Arel and different measures of flexibility are preserved when split by structural class, demonstrating that they are largely independent of secondary structure (Fig. S3 and Table S5).

  ### Attachments

  - 1-s2.0-S0022283613003999-main.pdf
  - [PDF] from arxiv.org
  - Snapshot
- ## C7orf30 is necessary for biogenesis of the large subunit of the mitochondrial ribosome

  |  |  |
  | --- | --- |
  | Type | Journal Article |
  | Author | Joanna Rorbach |
  | Author | Payam A. Gammage |
  | Author | Michal Minczuk |
  | URL | http://nar.oxfordjournals.org/content/40/9/4097.short |
  | Volume | 40 |
  | Issue | 9 |
  | Pages | 4097–4109 |
  | Publication | Nucleic Acids Research |
  | Date | 2012 |
  | Accessed | 9/20/2013, 1:17:33 PM |
  | Library Catalog | Google Scholar |
  | Date Added | 10/11/2013, 10:20:13 AM |
  | Modified | 11/12/2013, 4:28:53 PM |

  ### Notes:

  - Paper characterizing function of the C7orf30 Protein

    How SCOP is used:

    SCOP database accessed to provide background information on the DUF143 domain.

    SCOP reference:

    **Under "Identification of C7orf30 and in silico analysis of  
    proteins containing the DUF143 domain":**

    "On the basis of structural features, DUF143 has been assigned to the superfamily of nucleotidyltransferases (NTases) (19), enzymes that transfer nucleoside monophosphate (NMP) from nucleoside triphosphate (NTP) to an acceptor hydroxyl group belonging to a protein, nucleic acid or small molecule. NTases are characterized by the presence of a common minimal core of a-b-a-b-a-b-a topology (19)  
    (Figure 1)."

    19. Murzin,A.G., Brenner,S.E., Hubbard,T. and Chothia,C. (1995)  
    SCOP: a structural classification of proteins database for the  
    investigation of sequences and structures. J. Mol. Biol., 247,  
    536–540.

  ### Attachments

  - [HTML] from oxfordjournals.org
  - Nucl. Acids Res.-2012-Rorbach-4097-109.pdf
  - PubMed entry
  - Snapshot
- ## C7orf30 specifically associates with the large subunit of the mitochondrial ribosome and is involved in translation

  |  |  |
  | --- | --- |
  | Type | Journal Article |
  | Author | Bas F. J. Wanschers |
  | Author | Radek Szklarczyk |
  | Author | Aleksandra Pajak |
  | Author | Mariel A. M. van den Brand |
  | Author | Jolein Gloerich |
  | Author | Richard J. T. Rodenburg |
  | Author | Robert N. Lightowlers |
  | Author | Leo G. Nijtmans |
  | Author | Martijn A. Huynen |
  | Volume | 40 |
  | Issue | 9 |
  | Pages | 4040-4051 |
  | Publication | Nucleic Acids Research |
  | ISSN | 0305-1048 |
  | Date | MAY 2012 |
  | Extra | WOS:000304201300031 |
  | DOI | 10.1093/nar/gkr1271 |
  | Abstract | In a comparative genomics study for mitochondrial ribosome-associated proteins, we identified C7orf30, the human homolog of the plant protein iojap. Gene order conservation among bacteria and the observation that iojap orthologs cannot be transferred between bacterial species predict this protein to be associated with the mitochondrial ribosome. Here, we show colocalization of C7orf30 with the large subunit of the mitochondrial ribosome using isokinetic sucrose gradient and 2D Blue Native polyacrylamide gel electrophoresis (BN-PAGE) analysis. We co-purified C7orf30 with proteins of the large subunit, and not with proteins of the small subunit, supporting interaction that is specific to the large mitoribosomal complex. Consistent with this physical association, a mitochondrial translation assay reveals negative effects of C7orf30 siRNA knock-down on mitochondrial gene expression. Based on our data we propose that C7orf30 is involved in ribosomal large subunit function. Sequencing the gene in 35 patients with impaired mitochondrial translation did not reveal disease-causing mutations in C7orf30. |
  | Date Added | 2/13/2014, 4:13:17 PM |
  | Modified | 3/7/2014, 12:10:37 PM |
- ## Calculating ensemble averaged descriptions of protein rigidity without sampling

  |  |  |
  | --- | --- |
  | Type | Journal Article |
  | Author | Luis C González |
  | Author | Hui Wang |
  | Author | Dennis R Livesay |
  | Author | Donald J Jacobs |
  | Volume | 7 |
  | Issue | 2 |
  | Pages | e29176 |
  | Publication | PloS one |
  | ISSN | 1932-6203 |
  | Date | 2012 |
  | Extra | PMID: 22383947 |
  | Journal Abbr | PLoS ONE |
  | DOI | 10.1371/journal.pone.0029176 |
  | Library Catalog | NCBI PubMed |
  | Language | eng |
  | Abstract | Previous works have demonstrated that protein rigidity is related to thermodynamic stability, especially under conditions that favor formation of native structure. Mechanical network rigidity properties of a single conformation are efficiently calculated using the integer body-bar Pebble Game (PG) algorithm. However, thermodynamic properties require averaging over many samples from the ensemble of accessible conformations to accurately account for fluctuations in network topology. We have developed a mean field Virtual Pebble Game (VPG) that represents the ensemble of networks by a single effective network. That is, all possible number of distance constraints (or bars) that can form between a pair of rigid bodies is replaced by the average number. The resulting effective network is viewed as having weighted edges, where the weight of an edge quantifies its capacity to absorb degrees of freedom. The VPG is interpreted as a flow problem on this effective network, which eliminates the need to sample. Across a nonredundant dataset of 272 protein structures, we apply the VPG to proteins for the first time. Our results show numerically and visually that the rigidity characterizations of the VPG accurately reflect the ensemble averaged [Formula: see text] properties. This result positions the VPG as an efficient alternative to understand the mechanical role that chemical interactions play in maintaining protein stability. |
  | Date Added | 10/11/2013, 10:29:15 AM |
  | Modified | 10/11/2013, 10:29:15 AM |

  ### Tags:

  - Algorithms
  - Animals
  - Cluster Analysis
  - Computational Biology
  - Databases, Protein
  - Disulfides
  - Humans
  - Hydrogen Bonding
  - Models, Statistical
  - Protein Conformation
  - Protein Folding
  - Proteins
  - Software
  - Thermodynamics

  ### Notes:

  - Present a method for ensemble averaged analysis of protein rigidity and flexibility.

    How SCOP is used:

    Evaluate method on 272 structures that are nonredundant at the SCOP family level.

    SCOP reference:

    Protein Structure Description

    We consider a dataset composed of 272 protein structures that are nonredundant at the SCOP [35] family level. Our dataset includes one, two and three domain proteins for PDB codes (see Table 1), that range from 50 to 764 residues.

  ### Attachments

  - [HTML] from plos.org
  - journal.pone.0029176.pdf
  - PubMed entry
- ## Camps 2.0: Exploring the sequence and structure space of prokaryotic, eukaryotic, and viral membrane proteins

  |  |  |
  | --- | --- |
  | Type | Journal Article |
  | Author | Sindy Neumann |
  | Author | Holger Hartmann |
  | Author | Antonio J. Martin-Galiano |
  | Author | Angelika Fuchs |
  | Author | Dmitrij Frishman |
  | Volume | 80 |
  | Issue | 3 |
  | Pages | 839-857 |
  | Publication | Proteins: Structure, Function, and Bioinformatics |
  | ISSN | 0887-3585 |
  | Date | MAR 2012 |
  | Extra | WOS:000300053500014 |
  | DOI | 10.1002/prot.23242 |
  | Abstract | Structural bioinformatics of membrane proteins is still in its infancy, and the picture of their fold space is only beginning to emerge. Because only a handful of three-dimensional structures are available, sequence comparison and structure prediction remain the main tools for investigating sequencestructure relationships in membrane protein families. Here we present a comprehensive analysis of the structural families corresponding to a-helical membrane proteins with at least three transmembrane helices. The new version of our CAMPS database (CAMPS 2.0) covers nearly 1300 eukaryotic, prokaryotic, and viral genomes. Using an advanced classification procedure, which is based on high-order hidden Markov models and considers both sequence similarity as well as the number of transmembrane helices and loop lengths, we identified 1353 structurally homogeneous clusters roughly corresponding to membrane protein folds. Only 53 clusters are associated with experimentally determined three-dimensional structures, and for these clusters CAMPS is in reasonable agreement with structure-based classification approaches such as SCOP and CATH. We therefore estimate that similar to 1300 structures would need to be determined to provide a sufficient structural coverage of polytopic membrane proteins. CAMPS 2.0 is available at . Proteins 2011. (c) 2012 Wiley Periodicals, Inc. |
  | Date Added | 2/20/2014, 12:24:01 PM |
  | Modified | 5/5/2014, 3:11:52 PM |

  ### Notes:

  - Present CAMPS 2.0 database, and include an analysis of structural families for alpha-helical membrane proteins.

    Found 266 structurally homogenous clusters (SC-clusters) in a class of membrane proteins.

    How SCOP/CATH are used:

    Validate SC-clusters against SCOP and CATH fold classification.

    SCOP/CATH reference:

    In Abstract:

    Only 53 clusters are associated with experimentally determined three-dimensional structures, and for these clusters CAMPS is in rea- sonable agreement with structure-based classification approaches such as SCOP and CATH.

    ...

    Comparison of SC-clusters with SCOP and CATH

    Our SC-cluster classification approach aims at identify- ing structural membrane protein families whose members share the same fold. Thus, we were particularly interested to evaluate how well our SC-clusters correlate with SCOP1 and CATH2 folds. To this end, membrane pro- teins covered by SC-clusters as well as by SCOP or CATH were identified.

    In SCOP and CATH proteins are assigned to the same fold if their structures are similar in the overall shape and connectivity of secondary structure elements. Thus, at the fold level the classification approach of SCOP and

    CATH is solely based on structure. In contrast, CAMPS is mainly based on sequence information, but also exploits structural features. The major difference here is that while SCOP and CATH rely on tertiary structure in- formation CAMPS uses predicted topology information.

    Because membrane protein structures remain scarce only 54 proteins with known structure were involved in the comparison with CATH, spread over 21 CATH folds and 31 SC-clusters (Table III). When each of the 31 SC- clusters was investigated separately and the distribution of CATH fold assignments within each of them was tested, we found a perfect agreement in all cases. By comparing the two databases in the reverse direction (i.e., by analyzing the distribution of SC-clusters within each CATH fold), 1:1 relationships were found for 16 out of 21 CATH folds. The five other folds (CATH codes 1.10.287 ‘‘Helix hairpins,’’ 1.20.120 ‘‘Four helix bundle,’’ 1.20.950 ‘‘Fumarate reductase cytochrome b subunit,’’ 1.20.1070 ‘‘Rhodopsin 7-helix transmembrane proteins,’’ and 1.20.1300 ‘‘3 helical TM bundles of succinate and fu- marate reductases’’) were associated with two to four SC- clusters (Fig. 8). Except for one case (fold 1.20.1070), all proteins involved in the disagreements had two to five TMHs (here the number of TMHs corresponds to the

    PDBTM42 annotation). One explanation for the dis- agreements between CATH and CAMPS might be the fact that membrane proteins with few helices (<6 TMHs) are difficult to classify in general, as we demon- strated in our previous study.18 Specifically, we found that the fold space of membrane proteins with less than six TMHs is rather continuous, thus complicating their structural classification. Indeed, all CATH folds except 1.20.1070 involved in the disagreements with SC-clusters (1.10.287, 1.20.120, 1.20.950, and 1.20.1300) were already found to be involved in the disagreements between CATH an SCOP reported in our previous analysis.

  ### Attachments

  - 23242\_ftp.pdf
- ## canSAR: an integrated cancer public translational research and drug discovery resource

  |  |  |
  | --- | --- |
  | Type | Journal Article |
  | Author | Mark D. Halling-Brown |
  | Author | Krishna C. Bulusu |
  | Author | Mishal Patel |
  | Author | Joe E. Tym |
  | Author | Bissan Al-Lazikani |
  | Volume | 40 |
  | Issue | D1 |
  | Pages | D947-D956 |
  | Publication | Nucleic Acids Research |
  | ISSN | 0305-1048 |
  | Date | January 2012 |
  | DOI | 10.1093/nar/gkr881 |
  | Language | English |
  | Abstract | canSAR is a fully integrated cancer research and drug discovery resource developed to utilize the growing publicly available biological annotation, chemical screening, RNA interference screening, expression, amplification and 3D structural data. Scientists can, in a single place, rapidly identify biological annotation of a target, its structural characterization, expression levels and protein interaction data, as well as suitable cell lines for experiments, potential tool compounds and similarity to known drug targets. canSAR has, from the outset, been completely use-case driven which has dramatically influenced the design of the back-end and the functionality provided through the interfaces. The Web interface at http://cansar.icr.ac.uk provides flexible, multipoint entry into canSAR. This allows easy access to the multidisciplinary data within, including target and compound synopses, bioactivity views and expert tools for chemogenomic, expression and protein interaction network data. |
  | Date Added | 10/25/2013, 4:17:08 PM |
  | Modified | 11/12/2013, 4:28:24 PM |

  ### Tags:

  - Cite ASTRAL

  ### Notes:

  - canSAR is a very broad database for cancer translational research that integrates cancer-relevant biological data such as expression, amplification, RNAi etc, together with large protein–protein interaction data, chemical screening and pharmacological activities and 3D structure"

    How SCOP is used:

    To flesh out structural data in the database.  Database provides domain specific information from Astral.  Provides structure classification from SCOP.

    Use ASTRAL data.

    Looked at the website, and found I could browse by superfamily and family classification, and these were labeled by whether a structure was available.

    SCOP reference:

    Under Data Content:

    The primary source for canSAR structural data is the RCSB PDB (31). Data from PDBe (32) helps maintain an up-to-date mapping between various databases such as UniProt (8), Pfam(10) protein family repository, SCOP structure classification database (33) and provides information in computationally parseable files.

    Domain specific information is gathered from SCOP and Astral (34).

  ### Attachments

  - Nucl. Acids Res.-2012-Halling-Brown-D947-56.pdf
- ## Capturing protein sequence-structure specificity using computational sequence design

  |  |  |
  | --- | --- |
  | Type | Journal Article |
  | Author | Paul Mach |
  | Author | Patrice Koehl |
  | Volume | 81 |
  | Issue | 9 |
  | Pages | 1556-1570 |
  | Publication | Proteins: Structure, Function, and Bioinformatics |
  | ISSN | 0887-3585 |
  | Date | September 2013 |
  | DOI | 10.1002/prot.24307 |
  | Language | English |
  | Abstract | It is well known that protein fold recognition can be greatly improved if models for the underlying evolution history of the folds are taken into account. The improvement, however, exists only if such evolutionary information is available. To circumvent this limitation for protein families that only have a small number of representatives in current sequence databases, we follow an alternate approach in which the benefits of including evolutionary information can be recreated by using sequences generated by computational protein design algorithms. We explore this strategy on a large database of protein templates with 1747 members from different protein families. An automated method is used to design sequences for these templates. We use the backbones from the experimental structures as fixed templates, thread sequences on these backbones using a self-consistent mean field approach, and score the fitness of the corresponding models using a semi-empirical physical potential. Sequences designed for one template are translated into a hidden Markov model-based profile. We describe the implementation of this method, the optimization of its parameters, and its performance. When the native sequences of the protein templates were tested against the library of these profiles, the class, fold, and family memberships of a large majority (>90%) of these sequences were correctly recognized for an E-value threshold of 1. In contrast, when homologous sequences were tested against the same library, a much smaller fraction (35%) of sequences were recognized; The structural classification of protein families corresponding to these sequences, however, are correctly recognized (with an accuracy of >88%). Proteins 2013; (c) 2013 Wiley Periodicals, Inc. |
  | Date Added | 10/25/2013, 4:29:01 PM |
  | Modified | 3/7/2014, 12:09:17 PM |

  ### Tags:

  - ASTRAL subsets
  - Cite ASTRAL

  ### Notes:

  - Computational protein sequence design method.

    How SCOP is used:

    Benchmark against SCOP fold-level classification

    Derive two pairs of data sets:

    1. D\_L: 1747 proteins.  Used ASTRAL representatives of the 3464 SCOP families in SCOP 1.73.  Filtered out structures 'with incomplete backbones', or larger than 600 residues.

    2. S\_L: all 4096 remaining members of the 1747 families.

    3. D\_S: subset of D\_L

    4. S\_S: subset of S\_L

    How CATH is used:

    Not using CATH data.

    SCOP reference:

    We generated two data sets of test proteins, namely DS (and its companion SS), a data set of proteins extracted from the structural classification of proteins (SCOP) database7 to assess the influence of fixed backbone and fixed amino acid composition on the quality of the designed sequences, and DL (and its companion SL), a large superset of DS, also from SCOP used as a test set in a large fold recognition experiment.

    The data set, DL is a comprehensive set of 1747 proteins designed to cover a large number of protein folds found in the PDB, as well as to account for structural di- versity within folds. The set of proteins is selected from SCOP version 1.73. This version of SCOP contains 1086 protein folds, representing 1777 superfamilies, themselves including 3464 families. To account for the diversity in each fold, we started with the representatives of the 3464 families, as defined by Astral.43 Structures lacking a complete backbone, or larger than 600 amino acids were removed, leading to a subset of 1747 proteins, which we name DL (for large database). These proteins vary in length from 35 to 600 amino acids with an average of 160. They correspond to 600 different folds, 1005 super- families, and 1747 families. The distribution of proteins per fold is nonuniform, with 375 folds having a single representative, whereas, for example, the fold including DNA- and RNA-binding proteins forming three helix bundles includes 61 representatives. This nonuniform distribution reflects the nonuniform distributions of pro- tein sequence families per structural fold.

    The data set DL contains a single representative for each protein family considered. We built in parallel the data set SL, which contains all 4906 remaining members of these 1747 families. Protein sequences in SL display a wide range of similarities with the sequences from their representatives in DL, in the range of 16–100%; the cor- responding differences in structure fall in the range of 0.1–5.6A ̊.

    The second data set, DS, is a subset of DL defined as follows. A representative protein R in DL is kept in DS if its family in SCOP contains another domain S whose length is similar to the length of P (within two residues)

    such that the Ca root mean square deviation (RMSD) between R and S is lower than 2 A ̊ . From the 1747 pro- teins in DL, only 157 were found to satisfy these criteria. Five more proteins corresponding to less represented protein structure classes were added (Table I). The corre- sponding pairs (R, S), where S is called the companion of R, cover a wide range of similarities, from 0.1 to 1.9 A ̊ in structural similarity (as measured by the Ca RMSD) and from 15 to 100% identity in sequence. The addi- tional 1723 sequences from other members of the corre- sponding 162 families were collected to form a test sequence set SS. The proteins in DS correspond to 107 different folds, 130 superfamilies, and 162 families.

    Both DL and DS contain proteins from all four main classes of SCOP, namely a, b, a/b, and a 1 b (Table I).

    Fold Recognition Using Designed Sequences

  ### Attachments

  - prot24307.pdf
- ## Cascaded walks in protein sequence space: use of artificial sequences in remote homology detection between natural proteins

  |  |  |
  | --- | --- |
  | Type | Journal Article |
  | Author | S. Sandhya |
  | Author | R. Mudgal |
  | Author | C. Jayadev |
  | Author | K. R. Abhinandan |
  | Author | R. Sowdhamini |
  | Author | N. Srinivasan |
  | URL | http://pubs.rsc.org/en/content/articlehtml/2012/mb/c2mb25113b |
  | Volume | 8 |
  | Issue | 8 |
  | Pages | 2076–2084 |
  | Publication | Molecular BioSystems |
  | Date | 2012 |
  | Accessed | 9/23/2013, 10:16:05 AM |
  | Library Catalog | Google Scholar |
  | Short Title | Cascaded walks in protein sequence space |
  | Date Added | 10/11/2013, 10:29:15 AM |
  | Modified | 3/7/2014, 12:11:13 PM |

  ### Notes:

  - Remote homology detection through sequence based methods is challenging because homologous sequences may diverge significantly.  Present a method to generate sequences that "bridge the gap" to aid in remote homolog detection.

    How SCOP is used:

    Validate method on SCOP fold classification.

    How CATH is used:

    Not using CATH data.

    SCOP/CATH reference:

    In this paper, we describe a novel large-scale application of computationally designed protein-like sequences in remote homology detection. In the public domain, databases such as SCOP, CATH, PFAM etc.,3,31,32 have already employed discrete evolutionary signals to group related proteins into families and superfamilies. Here, we describe a method that utilizes observed residue substitutions, as embodied in a family-specific position- specific scoring matrix (PSSM), to design sequences for that family (see Methods and Fig. 1 and 2).

    SCOP reference:

    Improved coverage in cascade PSI-BLAST searches

    Search methods are assessed for their ability to detect protein relationships by querying commonly available databases. While some methods apply direct search schemes, others are more rigorous and employ multiple steps to detect remote relationships such as PSI-BLAST or jackhammer.40 Here, the utility of designed sequences in detecting distant protein relationships was assessed by augmenting a commonly available database, PALI+. Further, comparisons of coverage of known true positives (domains of known structure belonging to the same fold as the query) in natural (PALI+) and augmented databases (DPALI) were performed using Cascade PSI-BLAST which shows better family and superfamily coverage. Following a standard procedure, we consider all hits of a query from the parent fold to be true positives and hits from different SCOP folds to be false positives.6,41 I

  ### Attachments

  - C2MB25113B.pdf
- ## CATH – a hierarchic classification of protein domain structures

  |  |  |
  | --- | --- |
  | Type | Journal Article |
  | Author | C. A. Orengo |
  | Author | A. D. Michie |
  | Author | S. Jones |
  | Author | D. T. Jones |
  | Author | M. B. Swindells |
  | Author | J. M. Thornton |
  | Volume | 5 |
  | Issue | 8 |
  | Pages | 1093–1109 |
  | Publication | Structure |
  | Date | 1997 |
  | DOI | 10.1016/S0969-2126(97)00260-8 |
  | Library Catalog | Microsoft Academic Search |
  | Date Added | 10/11/2013, 10:29:15 AM |
  | Modified | 10/11/2013, 10:29:15 AM |

  ### Notes:

  - How SCOP  is used:

    Use SCOP as an additional reference data to help in manual classification, first to validate that the architectures were consistent with manually edited ones.  Second, to adjust domain boundaries by hand, if necessary.

    SCOP references:

    Under Introduction:

    The SCOP database, developed by Murzin et al. [17], groups proteins having significant sequence similarity into homologous families, whereas more distant structural similarities are largely identified manually. This database places emphasis on evolutionary relationships and information from the literature relating to well-studied fold families is also incorporated (e.g. the β trefoils [18] and the OB fold [19]).

    Under Results and Discussion:

    For a majority of the folds (>80%) this was a simple and straightforward process and the architectural categories assigned agreed well with those given in other publicly available databases (e.g. SCOP [17]).

    At the H-level, further possible evolutionary relation- ships between sequence families can be identified by cross-checking the literature and by reference to the SCOP database [17], which contains evolutionary data extracted from a variety of sources and derived by expert consideration.

    Future developments: automatic architecture assignment: The CATH architectural groupings are currently broad, general, categories that represent a preliminary classification which should significantly aid a future, more detailed analysis of common architectural features. Although, these groups are assigned manually, other publicly available classifications have adopted a similar pragmatic approach, using a combination of automatic and manual approaches where appropriate (SCOP, DIAL [17,27]).

    Under Materials and Methods:

    Step 3: assignment of domain boundaries for multidomain proteins  
     One representative from each near-identical sequence family (N-level, >95% sequence identity) is analysed to determine the number of domains and corresponding domain boundaries. A consensus ap- proach is used whereby the assignments given by three automatic methods are compared (DETECTIVE [22], PUU [23], DOMAK [24]). If they agree in the number of domains identified and there is at least 85% overlap in residues assigned to a given domain, the boundaries given by DETECTIVE are used to chop the structure into its constituent domains. Where the algorithms disagree, the boundaries are examined by visual inspection and by reference to assignments in other data- bases, SCOP [17], 3DEE, Siddiqui and Barton. (http://speed.biop. ox.ac.uk8080/3Dee), and the literature.
  - CATH hierarchy is divided into 5 levels:

    **Class**: mainly α, mainly β and α–β.  The two  classes α/β and α+β are handled on the topology level.

    **Architecture**:for example, TIM barrel, Sandwich, Roll

    **Topology**: share the same fold.  similar number and arrangement of secondary structures and 'connectivity linking secondary structure elements is the same'.

    **Homologous superfamily**: high structure similarity and similar functions.

    **Sequence level**: domains with sequence identities >35%.  may be different examples of the same protein from different species.

    Classes:

    mainly alpha class: most distinct is 4-helix bundle.  other helix arrangements are less distinct.  continuum of folds otherwise.  includes aligned alpha hairpin, two helix and thre-helix orthogonal motifs.

    mainly beta class - more diverse.  B prism, B propellor, B solenoid.

    alpha-beta class - not as diverse.  eight regular architectures (in 1997).  12 complex folds.

    CATH is semi-automatically curated.

    Step 1: Retain only X-ray structures with resolution greater than 3 Angstroms and NMR.

    Step 2: Pairwise sequence alignments and scoring.  Protiens are goruped into sequence-based famileis.  First, complteley identical families (100% seq similarity and overlap of structures).  Second, near-identical faimiles (>95% seq. sim., 85% of larger progrein equiv to smaller.  CATH S-level generated by clustering proteins with 35% sequence ident (at least 60% larger protein equiv to smaller).  This ensures there are 'no false positives'.  A represtentative structure is chosen for each family which should never change.

    Step 3: Assign domain boundaries for multidomain proteins.

    Run three automatic methods from literature for domain decomp on a representative for each near-identical sequence family.  If there is a consensusu on number of domains and 85% overlap in resiudes assigned to given domain, DETECTIVE is used as the tie breaker.  If no consensus, a visual inspection is used.

    Step 4:

  ### Attachments

  - Link to page at linkinghub.elsevier.com
  - PDF from nook.cs.ucdavis.edu
- ## CbrA is a flavin adenine dinucleotide protein that modifies the Escherichia coli outer membrane and confers specific resistance to colicin M

  |  |  |
  | --- | --- |
  | Type | Journal Article |
  | Author | Stephanie Helbig |
  | Author | Klaus Hantke |
  | Author | Moritz Ammelburg |
  | Author | Volkmar Braun |
  | URL | http://jb.asm.org/content/194/18/4894.short |
  | Volume | 194 |
  | Issue | 18 |
  | Pages | 4894–4903 |
  | Publication | Journal of bacteriology |
  | Date | 2012 |
  | Accessed | 9/20/2013, 1:12:54 PM |
  | Library Catalog | Google Scholar |
  | Date Added | 10/11/2013, 10:29:15 AM |
  | Modified | 10/11/2013, 10:29:15 AM |

  ### Notes:

  - Analysis (in lab and bioinformatics) of the structure, homology, and function of CbrA, a protein that increases the E. coli cells' resistance to the toxin Cma.

    **SCOP Use**

    **Website use.** The sequence of Cbra was searched against the SCOP database. This determined where it would be assigned in the superfamily (?) ("Rossmann fold type of FAD-binding oxidoreductases")

    **SCOP Reference**

    Using the CbrA sequence, we searched the Protein Data Bank  
    (PDB [4]) for the closest homolog of known structure. The top hit  
    of searches available on 4 February 2012 that clustered at a maximum  
    of 70% pairwise sequence identity for proteins similar to  
    CbrA was the GGR of the archaeon Thermoplasma acidophilum  
    (PDB identifier 3OZ2) (46). HHpred retrieved a P value of  
    1.0e$49 and 20% pairwise sequence identity of CbrA and 3OZ2  
    using two iterations of PSI-BLAST for multiple-sequence alignment  
    generation and activating the realignment with the MAC  
    option. Additional searches with CbrA against the SCOP database  
    (25), version 1.75, clustered at a maximum of 70% pairwise sequence  
    identity confirmed the assignment of CbrA to the Rossmann  
    fold type of FAD-binding oxidoreductases (12).

  ### Attachments

  - [HTML] from asm.org
  - J. Bacteriol.-2012-Helbig-4894-903.pdf
  - Snapshot
- ## ccPDB: compilation and creation of data sets from Protein Data Bank

  |  |  |
  | --- | --- |
  | Type | Journal Article |
  | Author | Harinder Singh |
  | Author | Jagat Singh Chauhan |
  | Author | M. Michael Gromiha |
  | Author | Gajendra P. S. Raghava |
  | Volume | 40 |
  | Issue | D1 |
  | Pages | D486-D489 |
  | Publication | Nucleic Acids Research |
  | ISSN | 0305-1048 |
  | Date | JAN 2012 |
  | Extra | WOS:000298601300071 |
  | DOI | 10.1093/nar/gkr1150 |
  | Abstract | ccPDB (http://crdd.osdd.net/raghava/ccpdb/) is a database of data sets compiled from the literature and Protein Data Bank (PDB). First, we collected and compiled data sets from the literature used for developing bioinformatics methods to annotate the structure and function of proteins. Second, data sets were derived from the latest release of PDB using standard protocols. Third, we developed a powerful module for creating a wide range of customized data sets from the current release of PDB. This is a flexible module that allows users to create data sets using a simple six step procedure. In addition, a number of web services have been integrated in ccPDB, which include submission of jobs on PDB-based servers, annotation of protein structures and generation of patterns. This database maintains > 30 types of data sets such as secondary structure, tight-turns, nucleotide interacting residues, metals interacting residues, DNA/RNA binding residues and so on. |
  | Date Added | 2/20/2014, 12:24:01 PM |
  | Modified | 3/7/2014, 12:11:06 PM |

  ### Notes:

  - Present a database of data sets compiled from the Protein Data Bank and from the literature.

    How SCOP/CATH is used:

    Not using SCOP or CATH data.

    SCOP/CATH  reference:

    In order to facilitate protein community, a large number of secondary databases have been derived from PDB, which includes SCOP (2), CATH (3), SuperSite (4), PDB-ligand (5), PDBsum (6), etc

  ### Attachments

  - Nucl. Acids Res.-2012-Singh-D486-9.pdf
- ## Cephalosporin C acylase: dream and (/or) reality

  |  |  |
  | --- | --- |
  | Type | Journal Article |
  | Author | Loredano Pollegioni |
  | Author | Elena Rosini |
  | Author | Gianluca Molla |
  | URL | http://link.springer.com/article/10.1007/s00253-013-4741-0 |
  | Pages | 1–15 |
  | Publication | Applied microbiology and biotechnology |
  | Date | 2013 |
  | Accessed | 9/23/2013, 10:14:36 AM |
  | Library Catalog | Google Scholar |
  | Short Title | Cephalosporin C acylase |
  | Date Added | 2/20/2014, 12:24:01 PM |
  | Modified | 2/20/2014, 12:24:01 PM |

  ### Notes:

  - Paper unavailable.

  ### Attachments

  - Snapshot
- ## Chapter 15: Disease Gene Prioritization

  |  |  |
  | --- | --- |
  | Type | Journal Article |
  | Author | Yana Bromberg |
  | Volume | 9 |
  | Issue | 4 |
  | Publication | PLoS computational biology |
  | ISSN | 1553-7358 |
  | Date | April 2013 |
  | DOI | 10.1371/journal.pcbi.1002902 |
  | Language | English |
  | Abstract | Disease-causing aberrations in the normal function of a gene define that gene as a disease gene. Proving a causal link between a gene and a disease experimentally is expensive and time-consuming. Comprehensive prioritization of candidate genes prior to experimental testing drastically reduces the associated costs. Computational gene prioritization is based on various pieces of correlative evidence that associate each gene with the given disease and suggest possible causal links. A fair amount of this evidence comes from high-throughput experimentation. Thus, well-developed methods are necessary to reliably deal with the quantity of information at hand. Existing gene prioritization techniques already significantly improve the outcomes of targeted experimental studies. Faster and more reliable techniques that account for novel data types are necessary for the development of new diagnostics, treatments, and cure for many diseases. |
  | Date Added | 10/11/2013, 10:29:15 AM |
  | Modified | 10/11/2013, 3:28:34 PM |
- ## Characterisation of a cell wall-anchored protein of Staphylococcus saprophyticus associated with linoleic acid resistance

  |  |  |
  | --- | --- |
  | Type | Journal Article |
  | Author | Nathan P. King |
  | Author | Türkan Sakin\cc |
  | Author | Nouri L. Ben Zakour |
  | Author | Makrina Totsika |
  | Author | Begoña Heras |
  | Author | Pavla Simerska |
  | Author | Mark Shepherd |
  | Author | Sören G. Gatermann |
  | Author | Scott A. Beatson |
  | Author | Mark A. Schembri |
  | URL | http://www.biomedcentral.com/1471-2180/12/8/ |
  | Volume | 12 |
  | Issue | 1 |
  | Pages | 8 |
  | Publication | BMC microbiology |
  | Date | 2012 |
  | Accessed | 9/20/2013, 1:18:50 PM |
  | Library Catalog | Google Scholar |
  | Date Added | 10/11/2013, 10:29:15 AM |
  | Modified | 10/11/2013, 10:29:15 AM |

  ### Tags:

  - Anti-Bacterial Agents
  - Bacterial Proteins
  - Cell Wall
  - DNA, Bacterial
  - Drug Resistance, Bacterial
  - Gene Deletion
  - Genes, Bacterial
  - Genetic Complementation Test
  - Humans
  - Linoleic Acid
  - Membrane Proteins
  - Molecular Sequence Data
  - Molecular Weight
  - Plasmids
  - Sequence Analysis, DNA
  - Sequence Homology, Amino Acid
  - Staphylococcus aureus
  - Staphylococcus saprophyticus
  - Urinary Tract Infections

  ### Notes:

  - The paper is a study into a cell wall anchored protein of the bacterium Staphylococcus saprophyticus, which they call SssF.  It's a study into it structure and function, and how it contributes to antibacterial resistance.

    How SCOP is used:

    Use Phyre for secondary structure prediction, which uses SCOP data (domain structures) to create possible models of the protein SssF.

    SCOP Reference:

    In order to predict its three-dimensional fold  
    we carried out a fold-recognition analysis of SssF  
    sequence using Phyre [25] (Protein Homology/AnalogY  
    Recognition Engine). This server allows a pairwise alignment  
    of the SssF sequence to a library of known protein  
    structures available from the Structural Classification of  
    Proteins (SCOP) [26] and the Protein Data Bank (PDB)  
    [27] databases and generates preliminary models of the  
    protein by mapping the sequence onto the atomic coordinates  
    of different templates.

  ### Attachments

  - 1471-2180-12-8.pdf
- ## Characterization of Danio rerio Mn2+-Dependent ADP-Ribose/CDP-Alcohol Diphosphatase, the Structural Prototype of the ADPRibase-Mn-Like Protein Family

  |  |  |
  | --- | --- |
  | Type | Journal Article |
  | Author | Joaquim Rui Rodrigues |
  | Author | Ascension Fernandez |
  | Author | Jose Canales |
  | Author | Alicia Cabezas |
  | Author | Joao Meireles Ribeiro |
  | Author | Maria Jesus Costas |
  | Author | Jose Carlos Cameselle |
  | Volume | 7 |
  | Issue | 7 |
  | Pages | e42249 |
  | Publication | Plos One |
  | ISSN | 1932-6203 |
  | Date | JUL 27 2012 |
  | Extra | WOS:000306950200188 |
  | DOI | 10.1371/journal.pone.0042249 |
  | Abstract | The ADPRibase-Mn-like protein family, that belongs to the metallo-dependent phosphatase superfamily, has different functional and structural prototypes. The functional one is the Mn2+-dependent ADP-ribose/CDP-alcohol diphosphatase from Rattus norvegicus, which is essentially inactive with Mg2+ and active with low micromolar Mn2+ in the hydrolysis of the phosphoanhydride linkages of ADP-ribose, CDP-alcohols and cyclic ADP-ribose (cADPR) in order of decreasing efficiency. The structural prototype of the family is a Danio rerio protein with a known crystallographic structure but functionally uncharacterized. To estimate the structure-function correlation with the same protein, the activities of zebrafish ADPRibase-Mn were studied. Differences between zebrafish and rat enzymes are highlighted. The former showed a complex activity dependence on Mn2+, significant (approximate to 25%) Mg2+-dependent activity, but was almost inactive on cADPR (150-fold less efficient than the rat counterpart). The low cADPR hydrolase activity agreed with the zebrafish genome lacking genes coding for proteins with significant homology with cADPR-forming enzymes. Substrate-docking to zebrafish wild-type protein, and characterization of the ADPRibase-Mn H97A mutant pointed to a role of His-97 in catalysis by orientation, and to a bidentate water bridging the dinuclear metal center as the potential nucleophile. Finally, three structural elements that delimit the active site entrance in the zebrafish protein were identified as unique to the ADPRibase-Mn-like family within the metallo-dependent phosphatase superfamily. |
  | Date Added | 2/20/2014, 12:24:01 PM |
  | Modified | 2/20/2014, 12:24:01 PM |

  ### Notes:

  - Computational and experimental study of ADPRibase-Mn-like SCOP family.

    How SCOP is used:

    Perform a structural alignment of domains from SCOP superfamily "Metallo-dependent Phosphatase Superfamily" in order to discern what structural elements are unique to one SCOP familiy within the superfamily.

    SCOP reference:

    Structural Elements Unique to the ADPRibase-Mn-like Family within the Metallo-dependent Phosphatase Superfamily

    ADPRibase-Mn-like proteins are classified by SCOP as a unique family within the MDP superfamily. Like the other proteins of the superfamily, zebrafish ADPRibase-Mn contains a 4-layer a/b/b/ a fold (SCOP ID 56299), but the two babab motifs that form it are interrupted by additional elements (Fig. 8). To find out what the unique structural aspects of these proteins could be, a search for structural homologues of zebrafish ADPRibase-Mn was run in the DALI database (http://ekhidna.biocenter.helsinki.fi/dali) [48] against the PDB90 subset of the Protein Data Bank (PDB). The search returned 44 matches that are shown structurally aligned to zebrafish ADPRibase-Mn in Fig. S1. From these, a set of proteins covering all the other families of the SCOP MDP superfamily was chosen for further analysis (Table S1). Against this background, zebrafish ADPRibase-Mn showed very little sequence conserva- tion, but a high degree of structure conservation (Fig. 8A). Only a few protein parts of ADPRibase-Mn were structurally not conserved and could be unique to the ADPRibase-Mn-like proteins. Among them, three are regions with (almost) no counterpart in the other superfamily members. One corresponds to amino acids aprox. 20–35; it contains a b-hairpin motif intercalated between the left ba element of the first babab motif, forming a small independent b sheet (Fig. 8B, strands 2 and 3). Another is formed by amino acids aprox. 65–70 and folds as a small a-helix, which follows the central b element of the same motif (Fig. 8B, helix 2). The third is a domain formed by amino acids aprox. 150–195, which interrupts the second babab motif and includes a large a-helix where two metal ions different from those of the dinuclear center are bound in the crystal structure with low occupancy (Fig. 8B, helices 7 and 8). Interestingly, all these elements unique to ADPRibase-Mn-like proteins delimit the active site entrance. A BlastP search showed they are conserved in the ADPRibase-Mn orthologues in terms of sequence. They are also conserved in terms of structure in ADPRibase-Mn proteins that have been modeled by homology to the zebrafish protein (Swiss-Model repository; http://swissmodel.expasy.org/ repository/; [49]).

  ### Attachments

  - journal.pone.0042249.pdf
- ## Chemical composition is maintained in poorly conserved intrinsically disordered regions and suggests a means for their classification

  |  |  |
  | --- | --- |
  | Type | Journal Article |
  | Author | Harry Amri Moesa |
  | Author | Shunichi Wakabayashi |
  | Author | Kenta Nakai |
  | Author | Ashwini Patil |
  | Volume | 8 |
  | Issue | 12 |
  | Pages | 3262-3273 |
  | Publication | Molecular Biosystems |
  | ISSN | 1742-206X |
  | Date | 2012 |
  | Extra | WOS:000311473200016 |
  | DOI | 10.1039/c2mb25202c |
  | Abstract | Intrinsically disordered regions in proteins are known to evolve rapidly while maintaining their function. However, given their lack of structure and sequence conservation, the means through which they stay functional is not clear. Poor sequence conservation also hampers the classification of these regions into functional groups. We studied the sequence conservation of a large number of predicted and experimentally determined intrinsically disordered regions from the human proteome in 7 other eukaryotes. We determined the chemical composition of disordered regions by calculating the fraction of positive, negative, polar, hydrophobic and special (Pro, Gly) residues, and studied its maintenance in orthologous proteins. A significant number of disordered regions with low sequence conservation showed considerable similarity in their chemical composition between orthologs. Clustering disordered regions based on their chemical composition resulted in functionally distinct groups. Finally, disordered regions showed location preference within the proteins that was dependent on their chemical composition. We conclude that preserving the overall chemical composition is one of the ways through which intrinsically disordered regions maintain their flexibility and function through evolution. We propose that the chemical composition of disordered regions can be used to classify them into functional groups and, together with conservation and location, may be used to define a general classification scheme. |
  | Date Added | 2/20/2014, 12:24:01 PM |
  | Modified | 2/20/2014, 12:24:01 PM |

  ### Notes:

  - Present method to classify Intrinsically Disordered Regions (IDR).

    How SCOP is used:

    Background on protein structure classification.

    SCOP reference:

    The current domain classification techniques are based either on structure18 or on sequence conservation.17 Due to their lack of structure and sequence conservation, a large number of IDRs are not amenable to these classification techniques.

  ### Attachments

  - c2mb25202c.pdf
- ## Chemogenomics of pyridoxal 5'-phosphate dependent enzymes

  |  |  |
  | --- | --- |
  | Type | Journal Article |
  | Author | Ratna Singh |
  | Author | Francesca Spyrakis |
  | Author | Pietro Cozzini |
  | Author | Alessandro Paiardini |
  | Author | Stefano Pascarella |
  | Author | Andrea Mozzarelli |
  | URL | http://informahealthcare.com/doi/abs/10.3109/14756366.2011.643305 |
  | Volume | 28 |
  | Issue | 1 |
  | Pages | 183–194 |
  | Publication | Journal of Enzyme Inhibition and Medicinal Chemistry |
  | Date | 2013 |
  | Accessed | 9/23/2013, 10:13:41 AM |
  | Library Catalog | Google Scholar |
  | Date Added | 10/11/2013, 10:29:15 AM |
  | Modified | 3/7/2014, 12:08:51 PM |

  ### Tags:

  - biligands
  - Chemoprints
  - drug targets
  - pharmacophore
  - PLP-dependent enzymes

  ### Notes:

  - Study of PLP-dependent enzymes using bioinformatics and a chemogenomic approach. Compare structures and sequences using alignment and generated Pharmacophore models in their methods.

    How SCOP is used:

    SCOP was one of 3 databases from which data were collected to build their own structure dataset of PLP-dependent enzymes. It appears that at least the fold information was used, based on how their own dataset was organized.

    How CATH is used:

    Also collect structures from CATH.

    SCOP Reference:

    Materials and methods

    Structure database

    A database containing three-dimensional structures  
    of PLP-dependent enzymes belonging to fold types  
    I-IV was built. Using the classification found in several  
    structural databases, SCOP16, CATH17 and MMDB18, a  
    total of 683 PLP-dependent crystallographic structures  
    were retrieved from the Protein Data Bank19. From these  
    structures, 65 representative members were selected on  
    the basis of a hierarchical set of criteria: (i) engineered  
    enzymes bearing residue mutations were discarded, (ii)  
    in the presence of orthologous enzymes, the structure  
    with the highest resolution was selected. Among the 65  
    retrieved structures, 49 belong to fold type I, 9 to fold type  
    II, 4 to fold type III and 3 to fold type IV (Table S1).

  ### Attachments

  - [PDF] from researchgate.net
  - Snapshot
- ## Chicken Cytochrome P450 1A5 Is the Key Enzyme for Metabolizing T-2 Toxin to 3 ' OH-T-2

  |  |  |
  | --- | --- |
  | Type | Journal Article |
  | Author | Shufeng Shang |
  | Author | Jun Jiang |
  | Author | Yiqun Deng |
  | Volume | 14 |
  | Issue | 6 |
  | Pages | 10809-10818 |
  | Publication | International Journal of Molecular Sciences |
  | ISSN | 1422-0067 |
  | Date | JUN 2013 |
  | Extra | WOS:000320772500008 |
  | DOI | 10.3390/ijms140610809 |
  | Abstract | The transmission of T-2 toxin and its metabolites into the edible tissues of poultry has potential effects on human health. We report that T-2 toxin significantly induces CYP1A4 and CYP1A5 expression in chicken embryonic hepatocyte cells. The enzyme activity assays of CYP1A4 and CYP1A5 heterologously expressed in HeLa cells indicate that only CYP1A5 metabolizes T-2 to 3'OH-T-2 by the 3'-hydroxylation of isovaleryl groups. In vitro enzyme assays of recombinant CYP1A5 expressed in DH5 alpha further confirm that CYP1A5 can convert T-2 into TC-1 (3'OH-T-2). Therefore, CYP1A5 is critical for the metabolism of trichothecene mycotoxin in chickens. |
  | Date Added | 2/13/2014, 4:13:17 PM |
  | Modified | 3/7/2014, 12:08:55 PM |
- ## Circular Dichroism Spectral Data and Metadata in the Protein Circular Dichroism Data Bank (PCDDB): A Tutorial Guide to Accession and Deposition

  |  |  |
  | --- | --- |
  | Type | Journal Article |
  | Author | Robert W. Janes |
  | Author | A. J. Miles |
  | Author | B. Woollett |
  | Author | L. Whitmore |
  | Author | D. Klose |
  | Author | B. A. Wallace |
  | Volume | 24 |
  | Issue | 9 |
  | Pages | 751–763 |
  | Publication | Chirality |
  | Date | September 2012 |
  | DOI | 10.1002/chir.22050 |
  | Abstract | The Protein Circular Dichroism Data Bank (PCDDB) is a web-based resource containing circular dichroism (CD) and synchrotron radiation circular dichroism spectral and associated metadata located at http://pcddb.cryst.bbk.ac.uk. This resource provides a freely available, user-friendly means of accessing validated CD spectra and their associated experimental details and metadata, thereby enabling broad usage of this material and new developments across the structural biology, chemistry, and bioinformatics communities. The resource also enables researchers utilizing CD as an experimental technique to have a means of storing their data at a secure site from which it is easily retrievable, thereby making their results publicly accessible, a current requirement of many grant-funding agencies world-wide, as well as meeting the data-sharing requirements for journal publications. This tutorial provides extensive information on searching, accessing, and downloading procedures for those who wish to utilize the data available in the data bank, and detailed information on deposition procedures for creating and validating entries, including comprehensive explanations of their contents and formats, for those who wish to include their data in the data bank. Chirality 24:751763, 2012. (c) 2012 Wiley Periodicals, Inc. |
  | Date Added | 3/7/2014, 12:08:00 PM |
  | Modified | 3/7/2014, 12:08:00 PM |
- ## Cis-trans isomerization of omega dihedrals in proteins

  |  |  |
  | --- | --- |
  | Type | Journal Article |
  | Author | Pierrick Craveur |
  | Author | Agnel Praveen Joseph |
  | Author | Pierre Poulain |
  | Author | Alexandre G. de Brevern |
  | Author | Joseph Rebehmed |
  | Volume | 45 |
  | Issue | 2 |
  | Pages | 279-289 |
  | Publication | Amino Acids |
  | ISSN | 0939-4451 |
  | Date | AUG 2013 |
  | Extra | WOS:000321947700007 |
  | DOI | 10.1007/s00726-013-1511-3 |
  | Abstract | Peptide bonds in protein structures are mainly found in trans conformation with a torsion angle omega close to 180A degrees. Only a very low proportion is observed in cis conformation with omega angle around 0A degrees. Cis-trans isomerization leads to local conformation changes which play an important role in many biological processes. In this paper, we reviewed the recent discoveries and research achievements in this field. First, we presented some interesting cases of biological processes in which cis-trans isomerization is directly implicated. It is involved in protein folding and various aspect of protein function like dimerization interfaces, autoinhibition control, channel gating, membrane binding. Then we reviewed conservation studies of cis peptide bonds which emphasized evolution constraints in term of sequence and local conformation. Finally we made an overview of the numerous molecular dynamics studies and prediction methodologies already developed to take into account this structural feature in the research area of protein modeling. Many cis peptide bonds have not been recognized as such due to the limited resolution of the data and to the refinement protocol used. Cis-trans proline isomerization reactions represents a vast and promising research area that still needs to be further explored for a better understanding of isomerization mechanism and improvement of cis peptide bond predictions. |
  | Date Added | 2/20/2014, 12:24:01 PM |
  | Modified | 2/20/2014, 12:24:01 PM |

  ### Notes:

  - Paper unavailable.
- ## Cis-trans peptide variations in structurally similar proteins

  |  |  |
  | --- | --- |
  | Type | Journal Article |
  | Author | Agnel Praveen Joseph |
  | Author | Narayanaswamy Srinivasan |
  | Author | Alexandre G. de Brevern |
  | Volume | 43 |
  | Issue | 3 |
  | Pages | 1369-1381 |
  | Publication | Amino acids |
  | ISSN | 0939-4451 |
  | Date | September 2012 |
  | DOI | 10.1007/s00726-011-1211-9 |
  | Language | English |
  | Abstract | The presence of energetically less favourable cis peptides in protein structures has been observed to be strongly associated with its structural integrity and function. Inter-conversion between the cis and trans conformations also has an important role in the folding process. In this study, we analyse the extent of conservation of cis peptides among similar folds. We look at both the amino acid preferences and local structural changes associated with such variations. Nearly 34% of the Xaa-Proline cis bonds are not conserved in structural relatives; Proline also has a high tendency to get replaced by another amino acid in the trans conformer. At both positions bounding the peptide bond, Glycine has a higher tendency to lose the cis conformation. The cis conformation of more than 30% of beta turns of type VIb and IV are not found to be conserved in similar structures. A different view using Protein Block-based description of backbone conformation, suggests that many of the local conformational changes are highly different from the general local structural variations observed among structurally similar proteins. Changes between cis and trans conformations are found to be associated with the evolution of new functions facilitated by local structural changes. This is most frequent in enzymes where new catalytic activity emerges with local changes in the active site. Cis-trans changes are also seen to facilitate inter-domain and inter-protein interactions. As in the case of folding, cis-trans conversions have been used as an important driving factor in evolution. |
  | Date Added | 10/11/2013, 10:29:15 AM |
  | Modified | 12/2/2013, 4:18:52 PM |

  ### Tags:

  - Interesting

  ### Notes:

  - Study propensity of different families to adopt cis peptide bond conformations (as opposed to the much more common trans conformation)

    How SCOP is used:

    Retrieved all structures in SCOP 1.75 with resolution above some cutoff, and classified by SCOP family.  Performed multiple sequence alignment on each family.

    SCOP reference:

    Dataset

    A set of high quality protein structures solved by X-ray crystallography, with resolution better than 1.6 A ̊ and R-factor <0.25 is extracted from the PDB. The SCOP domains (version 1.75) corresponding to these structures were identified and all those domains belonging to the same SCOP family were aligned. This resulted in multiple structural alignments of 775 families. The conservation of omegadihedral angles was studied by analysing well-aligned (<30% gaps) columns in the alignment.

  ### Attachments

  - s00726-011-1211-9.pdf
- ## Classification of alpha-Helical Membrane Proteins Using Predicted Helix Architectures

  |  |  |
  | --- | --- |
  | Type | Journal Article |
  | Author | Sindy Neumann |
  | Author | Angelika Fuchs |
  | Author | Barbara Hummel |
  | Author | Dmitrij Frishman |
  | Volume | 8 |
  | Issue | 10 |
  | Pages | e77491 |
  | Publication | Plos One |
  | ISSN | 1932-6203 |
  | Date | OCT 25 2013 |
  | Extra | WOS:000326155400039 |
  | DOI | 10.1371/journal.pone.0077491 |
  | Abstract | Despite significant methodological advances in protein structure determination high-resolution structures of membrane proteins are still rare, leaving sequence-based predictions as the only option for exploring the structural variability of membrane proteins at large scale. Here, a new structural classification approach for alpha-helical membrane proteins is introduced based on the similarity of predicted helix interaction patterns. Its application to proteins with known 3D structure showed that it is able to reliably detect structurally similar proteins even in the absence of any sequence similarity, reproducing the SCOP and CATH classifications with a sensitivity of 65% at a specificity of 90%. We applied the new approach to enhance our comprehensive structural classification of alpha-helical membrane proteins (CAMPS), which is primarily based on sequence and topology similarity, in order to find protein clusters that describe the same fold in the absence of sequence similarity. The total of 151 helix architectures were delineated for proteins with more than four transmembrane segments. Interestingly, we observed that proteins with 8 and more transmembrane helices correspond to fewer different architectures than proteins with up to 7 helices, suggesting that in large membrane proteins the evolutionary tendency to re-use already available folds is more pronounced. |
  | Date Added | 2/20/2014, 12:24:01 PM |
  | Modified | 3/7/2014, 12:09:06 PM |

  ### Notes:

  - Present method for structural classification of alpha-helical membrane proteins.

    SCOP use:

    Validate against membrane protein domains that are in both CATH and SCOP.

    SCOP reference:

    Classification of Predicted Helix Architectures in Comparison to SCOP and CATH

    The similarity of predicted helix interaction graphs and the possibility of discriminating proteins with similar and different architectures based on these graphs was first evaluated using proteins with available 3D structure that are classified consistently in SCOP and CATH either to the same fold or to different folds. As four helix bundle proteins are known to pose a problem to structural classification in general [13], only proteins with at least five transmembrane helices were considered. The resulting test set contained 54 protein chains forming 211 protein pairs of which 95 had the same fold assignment in SCOP/CATH while the remaining protein pairs had the same number of transmembrane helices but different fold assignments. Helix interactions were predicted for all proteins based on helix-helix contacts obtained with TMHcon [19] using a two step filtering procedure where a large set of residue contacts is selected in the first step but only those helix pairs are predicted as interacting that make at least C residue contacts (see Materials and Methods). Similarities among these predicted helix interactions were quantified using HISS similarity scores [14] in two variations: i) treating all predicted helix interactions equally, and ii) upweighting interactions with many predicted contacts.

  ### Attachments

  - journal.pone.0077491.pdf
- ## Classification of Ligand Molecules in PDB with Fast Heuristic Graph Match Algorithm COMPLIG

  |  |  |
  | --- | --- |
  | Type | Journal Article |
  | Author | Mihoko Saito |
  | Author | Naomi Takemura |
  | Author | Tsuyoshi Shirai |
  | Volume | 424 |
  | Issue | 5 |
  | Pages | 379-390 |
  | Publication | JOURNAL OF MOLECULAR BIOLOGY |
  | ISSN | 0022-2836 |
  | Date | DEC 14 2012 |
  | DOI | 10.1016/j.jmb.2012.10.001 |
  | Language | English |
  | Abstract | A fast heuristic graph-matching algorithm, COMPLIG, was devised to classify the small-molecule ligands in the Protein Data Bank (PDB), which are currently not properly classified on structure basis. By concurrently classifying proteins and ligands, we determined the most appropriate parameter for categorizing ligands to be more than 60% identity of atoms and bonds between molecules, and we classified 11,585 types of ligands into 1946 clusters. Although the large clusters were composed of nucleotides or amino acids, a significant presence of drug compounds was also observed. Application of the system to classify the natural ligand status of human proteins in the current database suggested that, at most, 37% of the experimental structures of human proteins were in complex with natural ligands. However, protein homology- and/or ligand similarity-based modeling was implied to provide models of natural interactions for an additional 28% of the total, which might be used to increase the knowledge of intrinsic protein-metabolite interactions. (C) 2012 Elsevier Ltd. All rights reserved. |
  | Date Added | 10/11/2013, 10:29:15 AM |
  | Modified | 10/11/2013, 10:29:15 AM |

  ### Tags:

  - bioinformatics
  - graph match
  - metabolome
  - protein ligand

  ### Notes:

  - Present clustering method for fast classification of ligands based on structure.  To set parameters for ligand classification, they concurrently classified proteins and their ligands.

    How SCOP is used:

    Evaluated method on data set classified by fold.  Found that certain folds had preferences for certain ligands.

    SCOP reference:

    Structure-based classification of proteins was also examined by referring to the SCOP database.29 In this analysis, proteins were classified by their folds rather than sequence similarity. Although the figures might not be directly comparable with those de- scribed above because proteins with the same fold were not necessarily homologous and the ligands were assigned to structural domains, the table entropy was minimum at ST = 63% and 60%, which was consistent with the classification based on sequence similarity (Fig. 2d). However, the summa- tion of PCP was generally higher than that with sequence-based classification over the examined ST range and the maximum value observed at rather small ST of 40% (Fig. 2e).

    ..

    Interestingly, a lower threshold (~ 40%) was suggested when the proteins were classified by the folds defined in the SCOP database (Fig. 2e).

    ..

    The table entropy and the summation of PCP were also evaluated with the protein classification based on the SCOP database29 and the ligand classification based on the Tanimoto coefficient of MACCS fingerprint.17 In the former clustering, protein subunits were divided into structural domains according to SCOP, and ligands were assigned to the domains. The domain classes were assigned according to Class-Fold definitions in the SCOP database. In the latter clustering, the scores were evaluated as the Tanimoto coefficient of MACCS fingerprints for two molecules by using the Open Babel tool.40 The ligand molecules were clustered through a complete linkage clustering with the same threshold ST as COMPLIG score rate, M(A, B)/max{M(A, A), M(B, B)}. The correlation between COMPLIG score rate and Tanimoto coefficient was evaluated based on the comparisons among PDB ligands.

  ### Attachments

  - 1-s2.0-S0022283612008091-main.pdf
- ## Classification of protein functional surfaces using structural characteristics

  |  |  |
  | --- | --- |
  | Type | Journal Article |
  | Author | Yan Yuan Tseng |
  | Author | Wen-Hsiung Li |
  | URL | http://www.pnas.org/content/109/4/1170.short |
  | Volume | 109 |
  | Issue | 4 |
  | Pages | 1170–1175 |
  | Publication | Proceedings of the National Academy of Sciences |
  | Date | 2012 |
  | Accessed | 9/20/2013, 1:18:20 PM |
  | Library Catalog | Google Scholar |
  | Date Added | 10/11/2013, 10:29:15 AM |
  | Modified | 3/7/2014, 12:10:58 PM |

  ### Notes:

  - Present a new classification, modeled on Pfam, CATH, SCOP, etc., to classify protein space by functional surfaces.

    Use a data set of bound structures from the PDB.  The binding sits are extracted and then pairwise similarity between binding surfaces is measured with RMSD.  They clustered the surfaces using some standard clustering algorithm and created a library of ~2K surface types.

     How SCOP is used:

    Do not use SCOP data.  SCOP reference is only to point out that there are different models of protein classification.

     How CATH is used:

    Compared their method for predicting function using structural features with using CATH.  Found their classification was more consistent with function annotation than CATH.

    SCOP Reference:

    Among the best-known protein classifications are Pfam (1) by a sequence-based method and CATH (class, architecture, topology, homologous superfamily) (2) and SCOP (Structural Classification of Proteins) (3), both of which are based on the fold–domain approach. From a sequence-based classification (1, 4), one gains knowledge of the expansion of protein families and their evolutionary relationships. From a fold–domain classifica- tion (2, 3), one obtains a global view of protein fold space (5).

    CATH reference:

    Abstract:

    We found that proteins with the same enzyme nomenclature may be divided into subtypes and that two proteins in the same CATH (Class, Architec- ture, Topology, Homologous superfamily) fold may belong to two different surface types. I

    ...

    Evaluation by EC Annotations and Comparison with CATH. To assess the performance of our method, we evaluated the PSC database using the 1,145 EC annotation entries that were explicitly assigned to 15,783 bound structures (containing 16,560 chains in the PDB). All unbound forms were ignored. A positive result occurred when a classified protein matched its EC annotation. In each test entry, we matched members of PSC against EC to compute the Tani- moto coefficient, a good measure for the similarity of two classi- fications (SI Text, Performance Evaluation). As an example, we tested EC 3.4.22.56 (cysteine 3 endopeptidase), which has 33 an- notation entries. PSC could find all of the cysteine 3 endopepti- dases and correctly classified them into the same surface type (ST178), whereas CATH grouped 19 of the 33 entries into CATH ID 3.30.70.1470, 13 entries into CATH ID 3.40.50.1460, which involve 29 mixed members, and 1 entry with no CATH assignment (Table S3). For this comparison between the EC and PSC data- bases, we calculated a similarity of 0.589 [=33/(33 + 56 # 33) (i.e., 33 EC entries, 56 PSC entries in subtype ST178, and EC and PSC share 33 entries)]. For EC and CATH, we calculated a similarity of 0.576 [=19/(33 + 19 # 19)]. After evaluating the 1,145 test entries, we obtained a higher overall average similarity of 59.9% between PSC and EC than that obtained between CATH and EC (31.4%). Therefore, the PSC classification achieved a much higher corre- lation between function and structure (shape) than CATH.

  ### Attachments

  - Full Text PDF
- ## CLCAs - A Family of Metalloproteases of Intriguing Phylogenetic Distribution and with Cases of Substituted Catalytic Sites

  |  |  |
  | --- | --- |
  | Type | Journal Article |
  | Author | Anna Lenart |
  | Author | Malgorzata Dudkiewicz |
  | Author | Marcin Grynberg |
  | Author | Krzysztof Pawlowski |
  | Volume | 8 |
  | Issue | 5 |
  | Pages | e62272 |
  | Publication | Plos One |
  | ISSN | 1932-6203 |
  | Date | MAY 9 2013 |
  | Extra | WOS:000319737700010 |
  | DOI | 10.1371/journal.pone.0062272 |
  | Abstract | The zinc-dependent metalloproteases with His-Glu-x-x-His (HExxH) active site motif, zincins, are a broad group of proteins involved in many metabolic and regulatory functions, and found in all forms of life. Human genome contains more than 100 genes encoding proteins with known zincin-like domains. A survey of all proteins containing the HExxH motif shows that approximately 52% of HExxH occurrences fall within known protein structural domains (as defined in the Pfam database). Domain families with majority of members possessing a conserved HExxH motif include, not surprisingly, many known and putative metalloproteases. Furthermore, several HExxH-containing protein domains thus identified can be confidently predicted to be putative peptidases of zincin fold. Thus, we predict zincin-like fold for eight uncharacterised Pfam families. Besides the domains with the HExxH motif strictly conserved, and those with sporadic occurrences, intermediate families are identified that contain some members with a conserved HExxH motif, but also many homologues with substitutions at the conserved positions. Such substitutions can be evolutionarily conserved and non-random, yet functional roles of these inactive zincins are not known. The CLCAs are a novel zincin-like protease family with many cases of substituted active sites. We show that this allegedly metazoan family has a number of bacterial and archaeal members. An extremely patchy phylogenetic distribution of CLCAs in prokaryotes and their conserved protein domain composition strongly suggests an evolutionary scenario of horizontal gene transfer (HGT) from multicellular eukaryotes to bacteria, providing an example of eukaryote-derived xenologues in bacterial genomes. Additionally, in a protein family identified here as closely homologous to CLCA, the CLCA\_X (CLCA-like) family, a number of proteins is found in phages and plasmids, supporting the HGT scenario. |
  | Date Added | 2/20/2014, 12:24:01 PM |
  | Modified | 2/20/2014, 12:24:01 PM |

  ### Notes:

  - Bioinformatics study of a protein family of matelloproteases.

    How SCOP is used:

    background on protein structure classification.

    SCOP reference:

    The protein sequence space, recently becoming sampled more and more densely thanks to genomic and metagenomic sequencing projects, has undoubtedly ‘granular’ features, and can be classified using various algorithms and classification systems [1,2].

  ### Attachments

  - journal.pone.0062272.pdf
- ## Cloning, Baeyer-Villiger biooxidations, and structures of the camphor pathway 2-oxo-Δ(3)-4,5,5-trimethylcyclopentenylacetyl-coenzyme A monooxygenase of Pseudomonas putida ATCC 17453

  |  |  |
  | --- | --- |
  | Type | Journal Article |
  | Author | Hannes Leisch |
  | Author | Rong Shi |
  | Author | Stephan Grosse |
  | Author | Krista Morley |
  | Author | Hélène Bergeron |
  | Author | Miroslaw Cygler |
  | Author | Hiroaki Iwaki |
  | Author | Yoshie Hasegawa |
  | Author | Peter C K Lau |
  | Volume | 78 |
  | Issue | 7 |
  | Pages | 2200-2212 |
  | Publication | Applied and environmental microbiology |
  | ISSN | 1098-5336 |
  | Date | Apr 2012 |
  | Extra | PMID: 22267661 |
  | Journal Abbr | Appl. Environ. Microbiol. |
  | DOI | 10.1128/AEM.07694-11 |
  | Library Catalog | NCBI PubMed |
  | Language | eng |
  | Abstract | A dimeric Baeyer-Villiger monooxygenase (BVMO) catalyzing the lactonization of 2-oxo-Δ(3)-4,5,5-trimethylcyclopentenylacetyl-coenzyme A (CoA), a key intermediate in the metabolism of camphor by Pseudomonas putida ATCC 17453, had been initially characterized in 1983 by Ougham and coworkers (H. J. Ougham, D. G. Taylor, and P. W. Trudgill, J. Bacteriol. 153:140-152, 1983). Here we cloned and overexpressed the 2-oxo-Δ(3)-4,5,5-trimethylcyclopentenylacetyl-CoA monooxygenase (OTEMO) in Escherichia coli and determined its three-dimensional structure with bound flavin adenine dinucleotide (FAD) at a 1.95-Å resolution as well as with bound FAD and NADP(+) at a 2.0-Å resolution. OTEMO represents the first homodimeric type 1 BVMO structure bound to FAD/NADP(+). A comparison of several crystal forms of OTEMO bound to FAD and NADP(+) revealed a conformational plasticity of several loop regions, some of which have been implicated in contributing to the substrate specificity profile of structurally related BVMOs. Substrate specificity studies confirmed that the 2-oxo-Δ(3)-4,5,5-trimethylcyclopentenylacetic acid coenzyme A ester is preferred over the free acid. However, the catalytic efficiency (k(cat)/K(m)) favors 2-n-hexyl cyclopentanone (4.3 × 10(5) M(-1) s(-1)) as a substrate, although its affinity (K(m) = 32 μM) was lower than that of the CoA-activated substrate (K(m) = 18 μM). In whole-cell biotransformation experiments, OTEMO showed a unique enantiocomplementarity to the action of the prototypical cyclohexanone monooxygenase (CHMO) and appeared to be particularly useful for the oxidation of 4-substituted cyclohexanones. Overall, this work extends our understanding of the molecular structure and mechanistic complexity of the type 1 family of BVMOs and expands the catalytic repertoire of one of its original members. |
  | Date Added | 10/11/2013, 10:29:15 AM |
  | Modified | 10/11/2013, 10:29:15 AM |

  ### Tags:

  - Amino Acid Sequence
  - Camphor
  - Circular Dichroism
  - Cloning, Molecular
  - Crystallography, X-Ray
  - Cyclopentanes
  - Escherichia coli
  - Flavin-Adenine Dinucleotide
  - Molecular Sequence Data
  - NADP
  - Oxidation-Reduction
  - Oxygenases
  - Pseudomonas putida
  - Sequence Analysis, DNA
  - Substrate Specificity

  ### Notes:

  - Experimental study of Baeyer-Village monooxygenase (BVMO) enzyme.

    How SCOP is used:

    Look up superfamily and family classification of all BVMO proteins studied.

    SCOP reference:

    All of these BVMOs belong to the FAD/NAD(P)-binding domain su- perfamily and the FAD/NAD-linked reductase structural family, as classified within the SCOP database (41), a family that includes a variety of dehydrogenases and reductases.

  ### Attachments

  - Appl. Environ. Microbiol.-2012-Leisch-2200-12.pdf
- ## Cloning, In Silico Characterization and Prediction of Three Dimensional Structure of SbDof1, SbDof19, SbDof23 and SbDof24 Proteins from Sorghum [Sorghum bicolor (L.) Moench]

  |  |  |
  | --- | --- |
  | Type | Journal Article |
  | Author | Hariom Kushwaha |
  | Author | Shubhra Gupta |
  | Author | Vinay Kumar Singh |
  | Author | Naveen C. Bisht |
  | Author | Bijaya K. Sarangi |
  | Author | Dinesh Yadav |
  | Volume | 54 |
  | Issue | 1 |
  | Pages | 1–12 |
  | Publication | Molecular Biotechnology |
  | Date | May 2013 |
  | DOI | 10.1007/s12033-012-9536-5 |
  | Abstract | In the present study, four full-length Dof (DNA-binding with one finger) genes from Sorghum bicolor namely SbDof1, SbDof19, SbDof23, and SbDof24 were PCR amplified, gel eluted, cloned, and sequenced (accession number HQ540084, HQ540085, HQ540086, and HQ540087, respectively). These sequences were further characterized in silico by subjecting them to homology search, multiple sequence alignment, phylogenetic tree construction, and protein functional analysis, revealing their identity to Dof like proteins. Phylogenetic analysis of cloned SbDof genes along with other reported Dof proteins revealed existence of two major groups A and B, while group A was further bifurcated into two sub-groups (viz., I and II). Motif scan analysis of SbDof proteins revealed the presence of glycine- and alanine-rich profiles in SbDof1, while proline-rich profile was observed in SbDof23. Asparagines, methionine, and serine-rich profiles were common in case of both SbDof19 and SbDof24 proteins. The three dimensional structures of SbDof proteins were predicted by I-TASSER server based on multiple threading method. The modeled structures were refined by energy minimization and their stereo chemical qualities were validated by PROCHECK and QMEAN server indicating the acceptability of the predicted models. The final models were submitted to PMDB database with assigned PMDB IDs, i.e., PM0077395, PM0077396, PM0077397, PM0077398, and PM0076448 for SbDof1, SbDof19, SbDof23, SbDof24, and Dof domain, respectively. Based on gene ontology (GO) terms in I-TASSER server putative functions of modeled SbDof proteins were also predicted. |
  | Date Added | 3/7/2014, 12:08:00 PM |
  | Modified | 3/7/2014, 12:08:00 PM |
- ## Clustering under approximation stability

  |  |  |
  | --- | --- |
  | Type | Journal Article |
  | Author | Maria-Florina Balcan |
  | Author | Avrim Blum |
  | Author | Anupam Gupta |
  | URL | http://dl.acm.org/citation.cfm?id=2450144 |
  | Volume | 60 |
  | Issue | 2 |
  | Pages | 8 |
  | Publication | Journal of the ACM (JACM) |
  | Date | 2013 |
  | Accessed | 9/20/2013, 1:16:24 PM |
  | Library Catalog | Google Scholar |
  | Date Added | 10/11/2013, 10:29:15 AM |
  | Modified | 10/11/2013, 10:29:15 AM |

  ### Tags:

  - algorithms
  - Approximation Algorithms
  - clustering
  - Clustering Accuracy
  - k-Means
  - k-Median
  - Min-Sum
  - Theory

  ### Notes:

  - Improve computational efficiency of clustering algorithms by using approximation.

    **SCOP Use**

    SCOP data is not used in this study, but mentioned its use in a study in "subsequent work" section.

    **SCOP Reference**

    7.3. Practical Application of Approximation-Stability

    Motivated by clustering applications in computational biology, Voevodski et al. [2010; 2012] analyze (c, ✏)-approximation-stability in a model with unknown distance infor- mation where one can only make a limited number of one versus all queries. They design an algorithm that, assuming (c, ✏)-approximation-stability for the k-median ob- jective, finds a clustering that is ✏-close to the target by using only O(k) one-versus- all queries in the large cluster case, and in addition is faster than the algorithm we present here. In particular, the algorithm for the large clusters case we describe in Section 3 can be implemented in O(|S|3) time, while the one proposed in [Voevodski et al. 2010; 2012] runs in time O(|S|k(k + log |S|)). They then use their algorithm to cluster biological datasets in the Pfam [Finn et al. 2010] and SCOP [Murzin et al. 1995] databases, where the points are proteins and distances are inversely propor- tional to their sequence similarity. This setting nicely fits the one-versus all queries model because one can use a fast sequence database search program to query a se- quence against an entire dataset. The Pfam [Finn et al. 2010] and SCOP [Murzin et al. 1995] databases are used in biology to observe evolutionary relationships be- tween proteins and to find close relatives of particular proteins. Voevodski et al. [2010; 2012] show that their algorithms are not only fast on these datasets, but also achieve high accuracy. In particular, for one of these sources they obtain clusterings that al- most exactly match the given classification, and for the other, the accuracy of their algorithm comparable to that of the best known (but slower) algorithms using the full distance matrix.

  ### Attachments

  - bbg-clustering-full.pdf
  - Snapshot
- ## Clusters of ancestrally related genes that show paralogy in whole or in part are a major feature of the genomes of humans and other species

  |  |  |
  | --- | --- |
  | Type | Journal Article |
  | Author | Michael B. Walker |
  | Author | Benjamin L. King |
  | Author | Kenneth Paigen |
  | URL | http://dx.plos.org/10.1371/journal.pone.0035274 |
  | Volume | 7 |
  | Issue | 4 |
  | Pages | e35274 |
  | Publication | PloS one |
  | Date | 2012 |
  | Accessed | 9/20/2013, 1:18:50 PM |
  | Library Catalog | Google Scholar |
  | Date Added | 10/11/2013, 10:29:15 AM |
  | Modified | 2/24/2014, 4:17:37 PM |

  ### Tags:

  - Animals
  - Arabidopsis
  - Biological Evolution
  - Caenorhabditis elegans
  - Cluster Analysis
  - Databases, Genetic
  - Drosophila melanogaster
  - Genome
  - Genome, Fungal
  - Genome, Human
  - Humans
  - Immunoglobulins
  - Saccharomyces cerevisiae

  ### Notes:

  - Computational study of the extents to which ancestrally related genes are found in proximity.  Combined information with 5 protein databases, including InterPro and SCOP.

    How SCOP is used:

    Use SCOP superfamily classification to determine whether genes are paralogs.

    SCOP reference:

    Two additional datasets place their emphasis on the presence of shared functional domains, relying on Hidden Markov Models for representing structural features. Here we have imputed paralogy when two proteins share domains and are located in close proximity beyond chance expectation, which depends on the frequency of the domains across the entire genome. The SCOP superfamilies dataset uses domain classification to assert common evolutionary origin between proteins even with low sequence similarity [18,19,20]. The InterPro dataset integrates many classification systems of protein signatures or domain structures into a single source [21].

  ### Attachments

  - [HTML] from plos.org
  - journal.pone.0035274.pdf
  - PubMed entry
- ## Cocrystal structure of the ICAP1 PTB domain in complex with a KRIT1 peptide

  |  |  |
  | --- | --- |
  | Type | Journal Article |
  | Author | Weizhi Liu |
  | Author | Titus J. Boggon |
  | Volume | 69 |
  | Pages | 494–498 |
  | Publication | Acta Crystallographica Section F-structural Biology and Crystallization Communications |
  | Date | May 2013 |
  | DOI | 10.1107/S1744309113010762 |
  | Abstract | Integrin cytoplasmic domain-associated protein-1 (ICAP1) is a suppressor of integrin activation and directly binds to the cytoplasmic tail of beta 1 integrins; its binding suppresses integrin activation by competition with talin. Krev/Rap1 interaction trapped-1 (KRIT1) releases ICAP1 suppression of integrin activation by sequestering ICAP1 away from integrin cytoplasmic tails. Here, the cocrystal structure of the PTB domain of ICAP1 in complex with a 29-amino-acid fragment (residues 170-198) of KRIT1 is presented to 1.7 angstrom resolution [the resolution at which < I/sigma(I)> = 2.9 was 1.83 angstrom]. In previous studies, the structure of ICAP1 with integrin beta 1 was determined to 3.0 angstrom resolution and that of ICAP1 with the N-terminal portion of KRIT1 (residues 1-198) was determined to 2.54 angstrom resolution; therefore, this study provides the highest resolution structure yet of ICAP1 and allows further detailed analysis of the interaction of ICAP1 with its minimal binding region in KRIT1. |
  | Date Added | 3/7/2014, 1:06:24 PM |
  | Modified | 3/7/2014, 1:06:24 PM |
- ## CoDNaS: a database of conformational diversity in the native state of proteins

  |  |  |
  | --- | --- |
  | Type | Journal Article |
  | Author | Alexander Miguel Monzon |
  | Author | Ezequiel Juritz |
  | Author | Maria Silvina Fornasari |
  | Author | Gustavo Parisi |
  | Volume | 29 |
  | Issue | 19 |
  | Pages | 2512–2514 |
  | Publication | Bioinformatics |
  | Date | October 2013 |
  | DOI | 10.1093/bioinformatics/btt405 |
  | Abstract | Motivation: Conformational diversity is a key concept in the understanding of different issues related with protein function such as the study of catalytic processes in enzymes, protein-protein recognition, protein evolution and the origins of new biological functions. Here, we present a database of proteins with different degrees of conformational diversity. Conformational Diversity of Native State (CoDNaS) is a redundant collection of three-dimensional structures for the same protein derived from protein data bank. Structures for the same protein obtained under different crystallographic conditions have been associated with snapshots of protein dynamism and consequently could characterize protein conformers. CoDNaS allows the user to explore global and local structural differences among conformers as a function of different parameters such as presence of ligand, post-translational modifications, changes in oligomeric states and differences in pH and temperature. Additionally, CoDNaS contains information about protein taxonomy and function, disorder level and structural classification offering useful information to explore the underlying mechanism of conformational diversity and its close relationship with protein function. Currently, CoDNaS has 122 122 structures integrating 12 684 entries, with an average of 9.63 conformers per protein. |
  | Date Added | 3/7/2014, 12:08:00 PM |
  | Modified | 3/7/2014, 12:08:00 PM |
- ## Coexistence of Phases in a Protein Heterodimer

  |  |  |
  | --- | --- |
  | Type | Journal Article |
  | Author | Andrey Krokhotin |
  | Author | Adam Liwo |
  | Author | Antti J. Niemi |
  | Author | Harold A. Scheraga |
  | Volume | 137 |
  | Issue | 3 |
  | Publication | JOURNAL OF CHEMICAL PHYSICS |
  | ISSN | 0021-9606 |
  | Date | JUL 21 2012 |
  | DOI | 10.1063/1.4734019 |
  | Language | English |
  | Abstract | A heterodimer consisting of two or more different kinds of proteins can display an enormous number of distinct molecular architectures. The conformational entropy is an essential ingredient in the Helmholtz free energy and, consequently, these heterodimers can have a very complex phase structure. Here, it is proposed that there is a state of proteins, in which the different components of a heterodimer exist in different phases. For this purpose, the structures in the protein data bank (PDB) have been analyzed, with radius of gyration as the order parameter. Two major classes of heterodimers with their protein components coexisting in different phases have been identified. An example is the PDB structure 3DXC. This is a transcriptionally active dimer. One of the components is an isoform of the intra-cellular domain of the Alzheimer-disease related amyloid precursor protein (AICD), and the other is a nuclear multidomain adaptor protein in the Fe65 family. It is concluded from the radius of gyration that neither of the two components in this dimer is in its own collapsed phase, corresponding to a biologically active protein. The UNRES energy function has been utilized to confirm that, if the two components are separated from each other, each of them collapses. The results presented in this work show that heterodimers whose protein components coexist in different phases, can have intriguing physical properties with potentially important biological consequences. (C) 2012 American Institute of Physics. [http://dx.doi.org/10.1063/1.4734019] |
  | Date Added | 10/11/2013, 10:29:15 AM |
  | Modified | 3/7/2014, 1:07:03 PM |

  ### Notes:

  - Computational study to test hypothesis that different components of a heterodimer exist in different phases.  The "phase" can be thought of as the degree of foldedness, where the native structure or low-energy state is the "collapsed" state.

    How SCOP is used:

    Use SCOP as evidence that the number of folds in protein space is limited.  Provide count on the number of unique folds in SCOP (1400) and CATH (1300) and discuss slow rate of new folds deposited.

    SCOP reference:

    C. Soliton description of protein-backbone geometry

    Despite the apparent complexity of interactions that are described by the various molecular dynamics force fields and realistic coarse-grained energy functions, collapsed proteins display a surprisingly small variety in their shapes. There seems to be a self-organizing principle at work, that strongly limits the diversity among the biologically active protein structures. This is also reflected in Figure 2(a) that the values of the a priori highly variable R0 in Eq. (3) are very restricted. Indeed, the presence of a universal self-organizing principle in protein folding is manifested in the PDB structures.28 For example, thus far the structural classification scheme SCOP (Ref. 59) has identified around 1.400 unique folds in the PDB while, in CATH,60 there are currently around 1.300 topolo- gies. These numbers have remained largely unchanged dur-

    ing the last 3–4 years, suggesting that the number of different protein folds is quite limited, and probably most of them have already been found.59–62 The success of SCOP and CATH and other approaches such as FSSP (Ref. 63) in classifying proteins confirms that proteins are built in a modular fashion, from a relatively small number of elemental components.

  ### Attachments

  - 1.4734019.pdf
- ## Cofactor-binding sites in proteins of deviating sequence: Comparative analysis and clustering in torsion angle, cavity, and fold space

  |  |  |
  | --- | --- |
  | Type | Journal Article |
  | Author | Bjoern Stegemann |
  | Author | Gerhard Klebe |
  | Volume | 80 |
  | Issue | 2 |
  | Pages | 626-648 |
  | Publication | Proteins-Structure Function and Bioinformatics |
  | ISSN | 0887-3585 |
  | Date | FEB 2012 |
  | Extra | WOS:000298955600025 |
  | DOI | 10.1002/prot.23226 |
  | Abstract | Small molecules are recognized in protein-binding pockets through surface-exposed physicochemical properties. To optimize binding, they have to adopt a conformation corresponding to a local energy minimum within the formed proteinligand complex. However, their conformational flexibility makes them competent to bind not only to homologous proteins of the same family but also to proteins of remote similarity with respect to the shape of the binding pockets and folding pattern. Considering drug action, such observations can give rise tounexpected and undesired cross reactivity. In this study, datasets of six different cofactors (ADP, ATP, NAD(P)(H), FAD, and acetyl CoA, sharing an adenosine diphosphate moiety as common substructure), observed in multiple crystal structures of proteincofactor complexes exhibiting sequence identity below 25%, have been analyzed for the conformational properties of the bound ligands, the distribution of physicochemical properties in the accommodating protein-binding pockets, and the local folding patterns next to the cofactor-binding site. State-of-the-art clustering techniques have been applied to group the different proteincofactor complexes in the different spaces. Interestingly, clustering in cavity (Cavbase) and fold space (DALI) reveals virtually the same data structuring. Remarkable relationships can be found among the different spaces. They provide information on how conformations are conserved across the host proteins and which distinct local cavity and fold motifs recognize the different portions of the cofactors. In those cases, where different cofactors are found to be accommodated in a similar fashion to the same fold motifs, only a commonly shared substructure of the cofactors is used for the recognition process. Proteins 2012. (C) 2011 Wiley Periodicals, Inc. |
  | Date Added | 2/13/2014, 4:13:17 PM |
  | Modified | 3/7/2014, 12:11:02 PM |
- ## Comparative Analysis of Barophily-Related Amino Acid Content in Protein Domains of Pyrococcus abyssi and Pyrococcus furiosus

  |  |  |
  | --- | --- |
  | Type | Journal Article |
  | Author | Liudmila S. Yafremava |
  | Author | Massimo Di Giulio |
  | Author | Gustavo Caetano-Anolles |
  | Pages | UNSP 680436 |
  | Publication | Archaea-an International Microbiological Journal |
  | ISSN | 1472-3646 |
  | Date | 2013 |
  | Extra | WOS:000325312700001 |
  | DOI | 10.1155/2013/680436 |
  | Abstract | Amino acid substitution patterns between the nonbarophilic Pyrococcus furiosus and its barophilic relative P. abyssi confirm that hydrostatic pressure asymmetry indices reflect the extent to which amino acids are preferred by barophilic archaeal organisms. Substitution patterns in entire protein sequences, shared protein domains defined at fold superfamily level, domains in homologous sequence pairs, and domains of very ancient and very recent origin now provide further clues about the environment that led to the genetic code and diversified life. The pyrococcal proteomes are very similar and share a very early ancestor. Relative amino acid abundance analyses showed that biases in the use of amino acids are due to their shared fold superfamilies. Within these repertoires, only two of the five amino acids that are preferentially barophilic, aspartic acid and arginine, displayed this preference significantly and consistently across structure and in domains appearing in the ancestor. The more primordial asparagine, lysine and threonine displayed a consistent preference for nonbarophily across structure and in the ancestor. Since barophilic preferences are already evident in ancient domains that are at least similar to 3 billion year old, we conclude that barophily is a very ancient trait that unfolded concurrently with genetic idiosyncrasies in convergence towards a universal code. |
  | Date Added | 2/20/2014, 12:24:01 PM |
  | Modified | 2/20/2014, 12:24:01 PM |

  ### Notes:

  - Study of evolution of barophilic domains.

    SCOP reference:

    Apply method to build evolutionary trees of superfamilies in order to study evolution of barophilic (living under extreme pressure) proteins.

    2. Materials and Methods

    FSF assignments and their respective sequences were ob- tained from a structural genomic census in 749 organisms [20] that used advanced linear HMMs of structural rec- ognition in superfamily [21], probability cutoffs ⬚⬚ of 10−4, and domain definitions from SCOP version 1.73 [10] (Figure 1). FSFs were segregated into 3 classes: (1) those present only in the barophile (species-specific barophilic FSFs), (2) those present only in the nonbarophile (species-specific nonbaro- philic FSFs), and (3) those present in both species (shared FSFs).

  ### Attachments

  - 680436.pdf
- ## Comparative Analysis of Proteomes and Functionomes Provides Insights into Origins of Cellular Diversification

  |  |  |
  | --- | --- |
  | Type | Journal Article |
  | Author | Arshan Nasir |
  | Author | Gustavo Caetano-Anolles |
  | Pages | 648746 |
  | Publication | Archaea-an International Microbiological Journal |
  | ISSN | 1472-3646; 1472-3654 |
  | Date | 2013 |
  | Extra | WOS:000329737800001 |
  | DOI | 10.1155/2013/648746 |
  | Abstract | Reconstructing the evolutionary history of modern species is a difficult problem complicated by the conceptual and technical limitations of phylogenetic tree building methods. Here, we propose a comparative proteomic and functionomic inferential framework for genome evolution that allows resolving the tripartite division of cells and sketching their history. Evolutionary inferences were derived from the spread of conserved molecular features, such as molecular structures and functions, in the proteomes and functionomes of contemporary organisms. Patterns of use and reuse of these traits yielded significant insights into the origins of cellular diversification. Results uncovered an unprecedented strong evolutionary association between Bacteria and Eukarya while revealing marked evolutionary reductive tendencies in the archaeal genomic repertoires. The effects of nonvertical evolutionary processes (e.g., HGT, convergent evolution) were found to be limited while reductive evolution and molecular innovation appeared to be prevalent during the evolution of cells. Our study revealed a strong vertical trace in the history of proteins and associated molecular functions, which was reliably recovered using the comparative genomics approach. The trace supported the existence of a stem line of descent and the very early appearance of Archaea as a diversified superkingdom, but failed to uncover a hidden canonical pattern in which Bacteria was the first superkingdom to deploy superkingdom-specific structures and functions. |
  | Date Added | 2/20/2014, 12:24:01 PM |
  | Modified | 2/20/2014, 12:24:01 PM |

  ### Notes:

  - Computational study of protein structure across genomes from different taxonomies to study species-evolution.

    How SCOP is used:

    Use SUPERFAMILY to get domains and SCOP superfamily classification for proteins in a data set of 981 proteomes.  SF annotations are then used to compare proteomes of Bacteria, Eukaryotes, and Archaea.

    SCOP reference:

    The structure dataset encompasses the occurrence and abundance of 1,733 fold superfamily (FSF) domains in 981 completely sequenced proteomes. FSF domains were delimited using the Struc- tural Classification of Proteins (SCOP ver. 1.75), which is a manually curated database of structural and evolutionary information of protein domains [19, 20]. The FSF level of the SCOP hierarchy includes domains that have diverged from a common ancestor and are evolutionarily conserved [21, 22].

    ...

    2. Materials and Methods

    2.1. Data Retrieval and Manipulation. FSF domain assign- ments for 981 completely sequenced proteomes were extracted from local MySQL installation of SUPERFAMILY ver. 1.75 database [36] using a stringent ⬚⬚-value cutoff of 10−4 [37]. The SUPERFAMILY database assigns structures to protein sequences using profile hidden Markov models (HMMs) searches that are superior in detecting remote homologies [38]. The dataset included 652 bacterial, 70 archaeal, and 259 eukaryal proteomes encoding a total repertoire of 1,733 significant FSF domains. In this study, FSFs were identified using SCOP alphanumeric identifiers (e.g., c.37.1, where c represent the class of domain structure (⬚⬚, ⬚⬚, ⬚⬚ + ⬚⬚, ⬚⬚/⬚⬚, etc.), 37 the fold, and 1 the FSF). This constituted the structure dataset.

    ...

  ### Attachments

  - 648746.pdf
- ## Comparative modeling and protein-like features of hydrophobic-polar models on a two-dimensional lattice

  |  |  |
  | --- | --- |
  | Type | Journal Article |
  | Author | Sergio Moreno-Hernández |
  | Author | Michael Levitt |
  | Volume | 80 |
  | Issue | 6 |
  | Pages | 1683-1693 |
  | Publication | Proteins: Structure, Function, and Bioinformatics |
  | ISSN | 1097-0134 |
  | Date | Jun 2012 |
  | Extra | PMID: 22411636 |
  | Journal Abbr | Proteins |
  | DOI | 10.1002/prot.24067 |
  | Library Catalog | NCBI PubMed |
  | Language | eng |
  | Abstract | Lattice models of proteins have been extensively used to study protein thermodynamics, folding dynamics, and evolution. Our study considers two different hydrophobic-polar (HP) models on the 2D square lattice: the purely HP model and a model where a compactness-favoring term is added. We exhaustively enumerate all the possible structures in our models and perform the study of their corresponding folds, HP arrangements in space and shapes. The two models considered differ greatly in their numbers of structures, folds, arrangements, and shapes. Despite their differences, both lattice models have distinctive protein-like features: (1) Shapes are compact in both models, especially when a compactness-favoring energy term is added. (2) The residue composition is independent of the chain length and is very close to 50% hydrophobic in both models, as we observe in real proteins. (3) Comparative modeling works well in both models, particularly in the more compact one. The fact that our models show protein-like features suggests that lattice models incorporate the fundamental physical principles of proteins. Our study supports the use of lattice models to study questions about proteins that require exactness and extensive calculations, such as protein design and evolution, which are often too complex and computationally demanding to be addressed with more detailed models. |
  | Date Added | 10/11/2013, 10:29:15 AM |
  | Modified | 3/7/2014, 1:07:00 PM |

  ### Tags:

  - Hydrophobic and Hydrophilic Interactions
  - hydrophobicity
  - lattice models
  - Models, Molecular
  - Protein Folding
  - protein like
  - Proteins
  - protein universe
  - residue composition
  - self-avoiding walk

  ### Notes:

  - Investigate two variants of 2D lattice model for studying protein dynamics.  Validation of the use of course-grained lattice models where more precise methods are too complex and computationally demanding.

    How SCOP is used:

    Not using SCOP data.  General reference to describe protein structure space.

    SCOP reference:

    The applications of lattice models are rich and varied, for example protein design. Folds differ greatly in their designabilities in nature40–43 and in lattice mod- els.17,27,34,35

  ### Attachments

  - 24067\_ftp.pdf
- ## Comparative structural modeling of a monothiol GRX from chickpea: Insight in iron-sulfur cluster assembly

  |  |  |
  | --- | --- |
  | Type | Journal Article |
  | Author | Saurabh Yadav |
  | Author | Hemant Ritturaj Kushwaha |
  | Author | Kamal Kumar |
  | Author | Praveen Kumar Verma |
  | Volume | 51 |
  | Issue | 3 |
  | Pages | 266-273 |
  | Publication | INTERNATIONAL JOURNAL OF BIOLOGICAL MACROMOLECULES |
  | ISSN | 0141-8130 |
  | Date | October 2012 |
  | DOI | 10.1016/j.ijbiomac.2012.05.014 |
  | Language | English |
  | Abstract | Glutaredoxins (GRXs) are small, ubiquitous, multifunctional, heat-stable and glutathione-dependent thiol-disulphide oxidoreductases, classified under thioredoxin-fold superfamily. In the green lineage, GRXs constitute a complex family of proteins. Based on their active site, GRXs are classified into two subfamilies: dithiol and monothiol. Monothiol GRXs contain `CGFS' as a redox active motif and assist in maintaining redox state and iron homeostasis within the cell. Using RACE strategy, a full length cDNA of chickpea (Cicer arietinum) glutaredoxin 3 (CarGRX3) was cloned and sequenced. The cDNA contains open reading frame of 537 bp encoding 178 amino acids and exhibits features of other known `CGFS' type GRXs. Based on the multiple sequence alignment among CarGRX3 and monothiol GRXs of other photosynthetic organisms, the characteristic motif (KGX4PXCGFSX([29/30/32])KX4WPTXPQX4GX3GGXDI) with 18 invariant residues was observed. The proposed structure of CarGRX3 was compared with structurally resolved monothiol GRXs of other organisms. The CarGRX3 and nearest Arabidopsis homolog (AtGR)(cp) shares 76% sequence identity which was reflected by their 3D-structure conservation. The structure of chickpea monothiol GRX (CarGRX3) coordinates glutathione ligated [2Fe-2S] cluster in a homodimeric form, highlighting the structural basis for iron-sulfur cluster (ISC) assembly and delivery to acceptor proteins. The present study on CarGRX3 model highlighted the utility of the theoretical approaches to understand complex biological phenomena such as glutathione docking and incorporation of GSH-ligated [2Fe-2S] cluster. (C) 2012 Elsevier B.V. All rights reserved. |
  | Date Added | 10/11/2013, 10:29:15 AM |
  | Modified | 3/7/2014, 12:10:28 PM |

  ### Tags:

  - Chickpea
  - docking
  - GRX
  - homology modeling
  - Iron-sulfur cluster
  - Monothiol glutaredoxin

  ### Notes:

  - Experimental study of glutaredoxin protein.

    How SCOP/CATH is used:

    Look up classification of Glutaredoxin (GRX) proteins with known 3D structures in SCOP and CATH.

    SCOP/CATH reference:

    2.6. Foldrecognitionandsecondarystructureanalysis

    Secondary structure of the protein was predicted using JNET [28], SABLE [29,30], PREDATOR [31–33], STRIDE [34], PSIPRED [35] and SAM-T08 [36] softwares. Fold-recognition analysis was carried out using FUGUE [37], mGENETHREADER [38], FFAS03 [39] and 3DPSSM [40] softwares. The architec- tural motifs and the topology of proteins with known 3D structure were analysed according to SCOP and CATH [41,42] classifications. Topology of the modelled CarGRX3 protein was analysed using PDBSum (http://www.ebi.ac.uk/thornton-srv/ databases/pdbsum/Generate.html).

  ### Attachments

  - 1-s2.0-S0141813012001857-main.pdf
- ## Comparing proteins by their internal dynamics: Exploring structure–function relationships beyond static structural alignments

  |  |  |
  | --- | --- |
  | Type | Journal Article |
  | Author | Cristian Micheletti |
  | URL | http://www.sciencedirect.com/science/article/pii/S1571064512001327 |
  | Volume | 10 |
  | Issue | 1 |
  | Pages | 1-26 |
  | Publication | Physics of Life Reviews |
  | ISSN | 1571-0645 |
  | Date | March 2013 |
  | Journal Abbr | Physics of Life Reviews |
  | DOI | 10.1016/j.plrev.2012.10.009 |
  | Accessed | 9/19/2013, 7:16:29 PM |
  | Library Catalog | ScienceDirect |
  | Abstract | The growing interest for comparing protein internal dynamics owes much to the realisation that protein function can be accompanied or assisted by structural fluctuations and conformational changes. Analogously to the case of functional structural elements, those aspects of protein flexibility and dynamics that are functionally oriented should be subject to evolutionary conservation. Accordingly, dynamics-based protein comparisons or alignments could be used to detect protein relationships that are more elusive to sequence and structural alignments. Here we provide an account of the progress that has been made in recent years towards developing and applying general methods for comparing proteins in terms of their internal dynamics and advance the understanding of the structure–function relationship. |
  | Short Title | Comparing proteins by their internal dynamics |
  | Date Added | 2/13/2014, 4:13:41 PM |
  | Modified | 3/7/2014, 1:06:40 PM |

  ### Notes:

  - Review on what has been done to classify the space of protein dynamics.

    SCOP classifies proteins first by structural similarities, and then by evolutionary relationships implied by sequence homologies.  One might consider classifying them instead by similarity in dynamics features.

    Quantifying the dynamics of proteins in a way that means they can be compared in a meaningful manner is a relatively new ﬁeld partly due to the problem being more complex than structure alone.

    How SCOP is used:

    Mention previous study in which an ASTRAL data set was used in a dynamics study (Tobi, et Al, Proteins, 2012).

    How CATH is used:

    Look up classification of proteins of interest.

    SCOP reference:

    F. Comparison of general dynamical patterns in members of the SCOP database

    Besides the above-mentioned studies, a comparative investigation of mean-square fluctuation profiles and mode shapes was recently undertaken by Tobi [163] for an extensive set of entries from the SCOP/Astral database[6, 23]. A distinctive point of the analysis of ref. [163] is the fact that the set of amino acids over which the dynamical properties are automatically compared is not identified by sequence or structural alignments, but by matching the fluctuation (or mode) amplitude profile itself, as first envisaged by Keskin et al.[75]

    CATH reference:

    The pro- teins covered two homologous groups: the first one (CATH[122] code 3.40.190.10) included cofactor binding fragment of CysB, the lysine/arginine/ornithine-binding protein (LAO), the enzyme porphobilinogen deami- nase (PBGD), the N-terminal lobe of ovotransferrin (OVOT) while the second one (CATH code 3.40.50.2300) comprised the ribose-binding protein (RBP) and the leucine/isoleucine/valine-binding protein (LIVBP).

    ...

    FIG. 9: Examples of significant dynamics-based alignments of proteins with di↵erent degree of structural and functional similarities (captured by the CATH code and primary EC number, respectively). The examples are taken from ref. [177] and the alignments were produced with the Aladyn web- server. The aligned proteins in panel (a) have the same fold (they share the full cath code) but have di↵erent function. The pair in panel (b) have the same function but di↵erent CATH architecture. The pair in panel (c) di↵er by CATH architecture and function. The pair in panel (a) involves a haloalkane dehalogenase (PDBid 2had, CATH: 3.40.50.1820, EC: 4) and a (s)-acetone-cyanohydrin lyase (PDBid: 1yb7, CATH: 3.40.50.1820, EC: 3). The pair in panel 9b) involves a Cellobiohydrolase i (PDBid: 1dy4, CATH: 2.70.100.10, EC: 3) and a glucanase (PDBid: 2ayh, CATH: 2.60.120.200, EC: 3). The pair in panel (c) involves an exonuclease (PDBid: 1ako, CATH: 3.60.10.10, EC: 3) and an Enoyl-reductase (PDBid: 1d7o, CATH: 3.40.50.720, EC: 1). For each pair we report separately the structural superposition of the aligned regions (ribbons) and of the top three best-matching modes (arrows). Aligned elements are shown in blue for the first entry of the pair and in red for the second. The active sites are shown in cyan and pink for the first and second entry of the pair, respectively.

  ### Attachments

  - [PDF] from arxiv.org
- ## Comparison and Druggability Prediction of Protein-Ligand Binding Sites from Pharmacophore-Annotated Cavity Shapes

  |  |  |
  | --- | --- |
  | Type | Journal Article |
  | Author | Jeremy Desaphy |
  | Author | Karima Azdimousa |
  | Author | Esther Kellenberger |
  | Author | Didier Rognan |
  | Volume | 52 |
  | Issue | 8 |
  | Pages | 2287-2299 |
  | Publication | Journal of Chemical Information and Modeling |
  | ISSN | 1549-9596 |
  | Date | AUG 2012 |
  | Extra | WOS:000308254200037 |
  | DOI | 10.1021/ci300184x |
  | Abstract | Estimating the pairwise similarity of protein-ligand binding sites is a fast and efficient way of predicting cross reactivity and putative side effects of drug candidates. Among the many tools available, three-dimensional (3D) alignment dependent methods are usually slow and based on simplified representations of binding site atoms or surfaces. On the other hand, fast and efficient alignment-free methods have recently been described but suffer from a lack of interpretability. We herewith present a novel binding site description (VolSite), coupled to an alignment and comparison tool (Shaper) combining the speed of alignment-free methods with the interpretability of alignment-dependent approaches. It is based on the comparison of negative images of binding cavities encoding both shape and pharmacophoric properties at regularly spaced grid points. Shaper approximates the resulting molecular shape with a smooth Gaussian function and aligns protein binding sites by optimizing their volume overlap. Volsite and Shaper were successfully applied to compare protein-ligand binding sites and to predict their structural druggability. |
  | Date Added | 2/13/2014, 4:13:17 PM |
  | Modified | 3/7/2014, 12:15:03 PM |
- ## Comparison of tertiary structures of proteins in protein-protein complexes with unbound forms suggests prevalence of allostery in signalling proteins

  |  |  |
  | --- | --- |
  | Type | Journal Article |
  | Author | Lakshmipuram S Swapna |
  | Author | Swapnil Mahajan |
  | Author | Alexandre G de Brevern |
  | Author | Narayanaswamy Srinivasan |
  | Volume | 12 |
  | Pages | 6 |
  | Publication | BMC Structural Biology |
  | ISSN | 1472-6807 |
  | Date | 2012 |
  | Extra | PMID: 22554255 |
  | Journal Abbr | BMC Struct. Biol. |
  | DOI | 10.1186/1472-6807-12-6 |
  | Library Catalog | NCBI PubMed |
  | Language | eng |
  | Abstract | BACKGROUND: Most signalling and regulatory proteins participate in transient protein-protein interactions during biological processes. They usually serve as key regulators of various cellular processes and are often stable in both protein-bound and unbound forms. Availability of high-resolution structures of their unbound and bound forms provides an opportunity to understand the molecular mechanisms involved. In this work, we have addressed the question "What is the nature, extent, location and functional significance of structural changes which are associated with formation of protein-protein complexes?" RESULTS: A database of 76 non-redundant sets of high resolution 3-D structures of protein-protein complexes, representing diverse functions, and corresponding unbound forms, has been used in this analysis. Structural changes associated with protein-protein complexation have been investigated using structural measures and Protein Blocks description. Our study highlights that significant structural rearrangement occurs on binding at the interface as well as at regions away from the interface to form a highly specific, stable and functional complex. Notably, predominantly unaltered interfaces interact mainly with interfaces undergoing substantial structural alterations, revealing the presence of at least one structural regulatory component in every complex.Interestingly, about one-half of the number of complexes, comprising largely of signalling proteins, show substantial localized structural change at surfaces away from the interface. Normal mode analysis and available information on functions on some of these complexes suggests that many of these changes are allosteric. This change is largely manifest in the proteins whose interfaces are altered upon binding, implicating structural change as the possible trigger of allosteric effect. Although large-scale studies of allostery induced by small-molecule effectors are available in literature, this is, to our knowledge, the first study indicating the prevalence of allostery induced by protein effectors. CONCLUSIONS: The enrichment of allosteric sites in signalling proteins, whose mutations commonly lead to diseases such as cancer, provides support for the usage of allosteric modulators in combating these diseases. |
  | Date Added | 10/11/2013, 10:29:15 AM |
  | Modified | 12/2/2013, 4:19:59 PM |

  ### Tags:

  - Allosteric Site
  - Animals
  - Databases, Protein
  - Ligands
  - Models, Molecular
  - Protein Binding
  - Proteins
  - Protein Structure, Tertiary
  - Signal Transduction

  ### Notes:

  - Study structural changes between bound and unbound forms of protein complexes.

    How SCOP is used:

    Use a curated data set of protein-protein interaction complexes derived from the Benchmark 3.0 data set.  Categorize each protein by SCOP class and family.  Use Class to show that there is a good distribution across the first 4 SCOP classes.  Family is used to show that no two proteins are from the same SCOP family.

    SCOP references:

    The main dataset of our study named PPC (Protein-protein com- plexes) is an extensively curated dataset of non-obligatory proteins with their 3-D structures solved in both unbound and bound forms (Additional file 2: Table S2). It consists of 76 non-obligatory complexes representing members of di- verse functions (25 enzyme-inhibitor, 11 antigen-antibody and 40 ‘other’ complexes, which largely comprises of signal- ling proteins). The number of proteins involved in the 76 complexes represent the major SCOP (Structural Classifica- tion of Proteins) [47] classes (all α - 32, all β - 84, α/β - 57, α + β - 37).

    ...

    Protein-protein complex (PPC) dataset

    The set of curated non-obligatory protein-protein inter- action complexes solved in both unbound and bound form is taken from Benchmark 3.0 dataset [34]. The set was further pruned using PISA [102] and PDB biological unit information to exclude cases containing different non-biological oligomeric forms of a protein in the un- bound and bound forms (eg. X-X in unbound form and X-Y in bound form) and bound to other small ligands or peptides. All antibody-antigen complexes in the original dataset in which only the bound structure of the anti- body was solved were discarded since the corresponding unbound form was not available. The final dataset con- sists of 76 non-obligatory complexes (see Additional file 2: Table S2). The resolution of these entries is 3.5 Å or better. Proteins in every interacting pair in the dataset is non-redundant at the level of SCOP family [47]. Al- though a much larger dataset can be compiled if only one of the interacting proteins is available in unbound and bound form, such a dataset was not used since our objective is to compare the changes occurring in both the proteins upon complexation.

  ### Attachments

  - 1472-6807-12-6.pdf
  - [HTML] from biomedcentral.com
  - PubMed entry
- ## Composite structural motifs of binding sites for delineating biological functions of proteins

  |  |  |
  | --- | --- |
  | Type | Journal Article |
  | Author | Akira R Kinjo |
  | Author | Haruki Nakamura |
  | Volume | 7 |
  | Issue | 2 |
  | Pages | e31437 |
  | Publication | PloS one |
  | ISSN | 1932-6203 |
  | Date | 2012 |
  | Extra | PMID: 22347478 |
  | Journal Abbr | PLoS ONE |
  | DOI | 10.1371/journal.pone.0031437 |
  | Library Catalog | NCBI PubMed |
  | Language | eng |
  | Abstract | Most biological processes are described as a series of interactions between proteins and other molecules, and interactions are in turn described in terms of atomic structures. To annotate protein functions as sets of interaction states at atomic resolution, and thereby to better understand the relation between protein interactions and biological functions, we conducted exhaustive all-against-all atomic structure comparisons of all known binding sites for ligands including small molecules, proteins and nucleic acids, and identified recurring elementary motifs. By integrating the elementary motifs associated with each subunit, we defined composite motifs that represent context-dependent combinations of elementary motifs. It is demonstrated that function similarity can be better inferred from composite motif similarity compared to the similarity of protein sequences or of individual binding sites. By integrating the composite motifs associated with each protein function, we define meta-composite motifs each of which is regarded as a time-independent diagrammatic representation of a biological process. It is shown that meta-composite motifs provide richer annotations of biological processes than sequence clusters. The present results serve as a basis for bridging atomic structures to higher-order biological phenomena by classification and integration of binding site structures. |
  | Date Added | 10/11/2013, 10:29:15 AM |
  | Modified | 10/11/2013, 10:29:15 AM |

  ### Tags:

  - Amino Acid Motifs
  - Binding Sites
  - Ligands
  - Models, Biological
  - Protein Interaction Maps
  - Proteins

  ### Notes:

  - Perform all-against-all atom-level structural comparisons of all ligand-binding sites in PDB structures to identify "elementary" structural motifs, then identified "composite" motifs as combinations of elementary structural motifs.  Then studied whether composite motifs correlated with protein functions.

    How SCOP is used:

    Provide background on the fold classification for proteins in their data set.  Point out that although they share the same fold classification, the functions are "similar but different" and the differences correspond to differences in the composite motifs.

    SCOP reference (45):

    Examples of composite motifs sharing the same elementary motif and fold but with different functions

    ....

    In the example in Fig. 1, while the three proteins (LAAO [42], KDM1 [43] and PAO [44]) share the same elementary motif (N2) for FAD binding and they share the same domain folds (FAD/NAD(P)-binding domain and FAD-linked reductases C-terminal domain [45]), their biological functions are similar but different; and these differences correspond to the differences in their composite motifs.

    ...

    Glycine oxidase (GO) and glycerol-3-phosphate dehydrogenase (GlpD). GO from Bacillus subtilis (PDB 1RYI [47], chain A) and GlpD from Escherichia coli (PDB 2QCU [48], chain A) share the same elementary motif for binding the FAD cofactor, and despite the low sequence similarity (\*14% sequence identity), they share the same fold (FAD/NAD(P)-binding domain [45]) according to the Matras fold comparison program [49,50] (Fig. 4A).

    ...

    D-3-phosphoglycerate dehydrogenase (PGDH) and C- terminal-binding protein 3 (CtBP3). PGDH from E. coli (PDB 1PSD [51], chain A, EC 1.1.1.95) and CtBP3 (also called CtBP1) from rat (PDB 1HKU [52], chain A, EC 1.1.1.-) share the same elementary motif for binding the NAD cofactor and the same folds (NAD(P)-binding Rossmann-fold domain and Flavodoxin-like fold [45]) with 25% sequence identity (Fig. 4B).

  ### Attachments

  - [HTML] from plos.org
  - journal.pone.0031437.pdf
  - PubMed entry
- ## Comprehensive analysis of the HEPN superfamily: identification of novel roles in intra-genomic conflicts, defense, pathogenesis and RNA processing

  |  |  |
  | --- | --- |
  | Type | Journal Article |
  | Author | Vivek Anantharaman |
  | Author | Kira S. Makarova |
  | Author | A. Maxwell Burroughs |
  | Author | Eugene V. Koonin |
  | Author | L. Aravind |
  | Volume | 8 |
  | Publication | Biology Direct |
  | ISSN | 1745-6150 |
  | Date | JUN 15 2013 |
  | Extra | WOS:000321629500001 |
  | DOI | 10.1186/1745-6150-8-15 |
  | Abstract | Background: The major role of enzymatic toxins that target nucleic acids in biological conflicts at all levels has become increasingly apparent thanks in large part to the advances of comparative genomics. Typically, toxins evolve rapidly hampering the identification of these proteins by sequence analysis. Here we analyze an unexpectedly widespread superfamily of toxin domains most of which possess RNase activity. Results: The HEPN superfamily is comprised of all alpha-helical domains that were first identified as being associated with DNA polymerase beta-type nucleotidyltransferases in prokaryotes and animal Sacsin proteins. Using sensitive sequence and structure comparison methods, we vastly extend the HEPN superfamily by identifying numerous novel families and by detecting diverged HEPN domains in several known protein families. The new HEPN families include the RNase LS and LsoA catalytic domains, KEN domains (e.g. RNaseL and Ire1) and the RNase domains of RloC and PrrC. The majority of HEPN domains contain conserved motifs that constitute a metal-independent endoRNase active site. Some HEPN domains lacking this motif probably function as non-catalytic RNA-binding domains, such as in the case of the mannitol repressor MtlR. Our analysis shows that HEPN domains function as toxins that are shared by numerous systems implicated in intra-genomic, inter-genomic and intra-organismal conflicts across the three domains of cellular life. In prokaryotes HEPN domains are essential components of numerous toxin-antitoxin (TA) and abortive infection (Abi) systems and in addition are tightly associated with many restriction-modification (R-M) and CRISPR-Cas systems, and occasionally with other defense systems such as Pgl and Ter. We present evidence of multiple modes of action of HEPN domains in these systems, which include direct attack on viral RNAs (e.g. LsoA and RNase LS) in conjunction with other RNase domains (e.g. a novel RNase H fold domain, NamA), suicidal or dormancy-inducing attack on self RNAs (RM systems and possibly CRISPR-Cas systems), and suicidal attack coupled with direct interaction with phage components (Abi systems). These findings are compatible with the hypothesis on coupling of pathogen- targeting (immunity) and self-directed (programmed cell death and dormancy induction) responses in the evolution of robust antiviral strategies. We propose that altruistic cell suicide mediated by HEPN domains and other functionally similar RNases was essential for the evolution of kin and group selection and cell cooperation. HEPN domains were repeatedly acquired by eukaryotes and incorporated into several core functions such as endonucleolytic processing of the 5.8S-25S/28S rRNA precursor (Las1), a novel ER membrane-associated RNA degradation system (C6orf70), sensing of unprocessed transcripts at the nuclear periphery (Swt1). Multiple lines of evidence suggest that, similar to prokaryotes, HEPN proteins were recruited to antiviral, antitransposon, apoptotic systems or RNA-level response to unfolded proteins (Sacsin and KEN domains) in several groups of eukaryotes. Conclusions: Extensive sequence and structure comparisons reveal unexpectedly broad presence of the HEPN domain in an enormous variety of defense and stress response systems across the tree of life. In addition, HEPN domains have been recruited to perform essential functions, in particular in eukaryotic rRNA processing. These findings are expected to stimulate experiments that could shed light on diverse cellular processes across the three domains of life. |
  | Date Added | 10/28/2013, 4:51:00 PM |
  | Modified | 10/28/2013, 4:51:00 PM |

  ### Notes:

  - Bioinformatics study of HEPN superfamily.

    How SCOP is used:

    Looked up classification of superfamily in SCOP.

    SCOP reference:

    Structural features of the HEPN domain and the remarkable structural rearrangement in the HEPN from CRISPR-Cas systems  
     To place the identified sequence features of the HEPN domain in a three-dimensional context, we performed a systematic comparison of all available structures of HEPN domains in the PDB database. **Other than the C-terminal helical domains of nucleotidyltransferases (see SCOP database id: 81593 [64]),** we retrieved 16 distinct struc- tures of HEPN domains that come from 7 distinct families (Figure 1 and Table 1).

  ### Attachments

  - 1745-6150-8-15.pdf
- ## Comprehensive comparison of graph based multiple protein sequence alignment strategies

  |  |  |
  | --- | --- |
  | Type | Journal Article |
  | Author | Ilya Plyusnin |
  | Author | Liisa Holm |
  | Volume | 13 |
  | Pages | 64 |
  | Publication | Bmc Bioinformatics |
  | ISSN | 1471-2105 |
  | Date | APR 29 2012 |
  | Extra | WOS:000305269700001 |
  | DOI | 10.1186/1471-2105-13-64 |
  | Abstract | Background: Alignment of protein sequences (MPSA) is the starting point for a multitude of applications in molecular biology. Here, we present a novel MPSA program based on the SeqAn sequence alignment library. Our implementation has a strict modular structure, which allows to swap different components of the alignment process and, thus, to investigate their contribution to the alignment quality and computation time. We systematically varied information sources, guiding trees, score transformations and iterative refinement options, and evaluated the resulting alignments on BAliBASE and SABmark. Results: Our results indicate the optimal alignment strategy based on the choices compared. First, we show that pairwise global and local alignments contain sufficient information to construct a high quality multiple alignment. Second, single linkage clustering is almost invariably the best algorithm to build a guiding tree for progressive alignment. Third, triplet library extension, with introduction of new edges, is the most efficient consistency transformation of those compared. Alternatively, one can apply tree dependent partitioning as a post processing step, which was shown to be comparable with the best consistency transformation in both time and accuracy. Finally, propagating information beyond four transitive links introduces more noise than signal. Conclusions: This is the first time multiple protein alignment strategies are comprehensively and clearly compared using a single implementation platform. In particular, we showed which of the existing consistency transformations and iterative refinement techniques are the most valid. Our implementation is freely available at http://ekhidna. biocenter.helsinki.fi/MMSA and as a supplementary file attached to this article (see Additional file 1). |
  | Date Added | 2/13/2014, 4:13:41 PM |
  | Modified | 3/7/2014, 1:06:58 PM |
- ## Compressive genomics for protein databases

  |  |  |
  | --- | --- |
  | Type | Journal Article |
  | Author | Noah M. Daniels |
  | Author | Andrew Gallant |
  | Author | Jian Peng |
  | Author | Lenore J. Cowen |
  | Author | Michael Baym |
  | Author | Bonnie Berger |
  | URL | http://bioinformatics.oxfordjournals.org/content/29/13/i283.abstract |
  | Volume | 29 |
  | Issue | 13 |
  | Pages | i283–i290 |
  | Publication | Bioinformatics |
  | Date | 2013 |
  | Accessed | 9/23/2013, 10:16:05 AM |
  | Library Catalog | Google Scholar |
  | Date Added | 10/11/2013, 10:29:15 AM |
  | Modified | 10/11/2013, 10:29:15 AM |

  ### Tags:

  - ASTRAL
  - ASTRAL sequences
  - ASTRAL subsets

  ### Notes:

  - Motivated by the need to do homology searching on very large databases, have introduced a program called CaBLAST (Compressively accelerated BLAST) that performs much faster than PSI-BLAST and other BLAST variants.

    How SCOP data is used:

    Description: Compare their methods, 2 "compressive" BLAST-variants, against another method, HHblits.  To validate, used ASTRAL 1.75A sequence data, retaining only those that were in the same family (but not the same sequence), as sequences in another data set (HHblits, NR20 from NCBI).  Then counted the number of true positives as hits in the same superfamily and true negatives as hits in different folds.

    Levels used: fold, superfamily, family, domain

    Reference to SCOP:

    We were also interested in homology detection performance of our compressive implementations of PSI-BLAST and DELTA-BLAST with respect to HHblits (McDonnell et al., 2006). We identified all 1123 sequences from the ASTRAL subset of release 1.75A of the Structural Classifications of Proteins (SCOP) (Murzin et al., 1995) database that were not present in HHblits’ ‘NR20’ database or the August 2010 NCBI NR database, but whose SCOP families contained other homolo- gous sequences that were present in those databases. We chose the August 2010 NCBI NR database to more fairly compare with the August 2011 HHblits NR20, which is the most recent available. We then performed searches using one iteration of HHblits, one iteration of cablastp-deltasearch and two iterations of cablastp-psisearch against these databases. We chose these numbers of iterations because a single iteration of PSI-BLAST is effectively just BLASTP, whereas Boratyn et al. (2012) showed decreased accuracy with more than one iteration of DELTA-BLAST. Multiple iterations of HHblits would have resulted in slower runtime performance. **We considered results from the same SCOP superfamily (and by extension, the same SCOP family) as the query to be true positives, and results from different SCOP folds to be false positives.** We removed results from the same SCOP fold but differ-ent superfamilies, as it is not consistent across the SCOP fold classifica- tions whether those sequences are homologs. We also removed results that were not identifiable in SCOP. We plotted ROC curves based on these homology predictions. We also report the mean running times of these searches.

  ### Attachments

  - Full Text PDF
  - [HTML] from oxfordjournals.org
  - Snapshot
- ## Computational and Theoretical Methods for Protein Folding

  |  |  |
  | --- | --- |
  | Type | Journal Article |
  | Author | Mario Compiani |
  | Author | Emidio Capriotti |
  | Volume | 52 |
  | Issue | 48 |
  | Pages | 8601-8624 |
  | Publication | Biochemistry |
  | ISSN | 0006-2960 |
  | Date | DEC 3 2013 |
  | Extra | WOS:000327999300001 |
  | DOI | 10.1021/bi4001529 |
  | Abstract | A computational approach is essential whenever the complexity of the process under study is such that direct theoretical or experimental approaches are not viable. This is the case for protein folding, for which a significant amount of data are being collected. This paper reports on the essential role of in silico methods and the unprecedented interplay of computational and theoretical approaches, which is a defining point of the interdisciplinary investigations of the protein folding process. Besides giving an overview of the available computational methods and tools, we argue that computation plays not merely an ancillary role but has a more constructive function in that computational work may precede theory and experiments. More precisely, computation can provide the primary conceptual clues to inspire subsequent theoretical and experimental work even in a case where no preexisting evidence or theoretical frameworks are available. This is cogently manifested in the application of machine learning methods to come to grips with the folding dynamics. These close relationships suggested complementing the review of computational methods within the appropriate theoretical context to provide a self-contained outlook of the basic concepts that have converged into a unified description of folding and have grown in a synergic relationship with their computational counterpart. Finally, the advantages and limitations of current computational methodologies are discussed to show how the smart analysis of large amounts of data and the development of more effective algorithms can improve our understanding of protein folding. |
  | Date Added | 2/20/2014, 12:24:01 PM |
  | Modified | 3/7/2014, 12:10:04 PM |

  ### Notes:

  - Review of research into protein folding.

    How SCOP/CATH is used:

    Measure growth of structures in PDB and number of SCOP and CATH folds since 1975.

    SCOP/CATH reference:

    The complete definition of the space of protein structures is important for selecting key interactions for the stabilization of the native conformation and depends on the procedure used to classify the proteins included in the PDB. The current gold standards for the classification of protein structures are SCOP145 and CATH.146 The Structural Classification Of Proteins (SCOP) is a database composed by manually classified protein structure domains based on their similarities. It is a hierarchical classification comprised of the following levels: species, protein, family, superfamily, fold, and class. In the SCOP database, two domains that belong to the same fold have similar secondary structures in the same arrangement and with the same topological connections. CATH is a semiautomatic procedure for defining a hierarchical classification of the structures of protein domains. This classification is based on four levels: class, architecture, topology, and homologous superfamily. When two proteins have similar structural features and a high degree of sequence similarity in conjunction with similar functions, they are assumed to be evolutionarily related and, therefore, associated with the same CATH identifier.

  ### Attachments

  - bi4001529.pdf
- ## Computational design of a Diels-Alderase from a thermophilic esterase: the importance of dynamics

  |  |  |
  | --- | --- |
  | Type | Journal Article |
  | Author | Mats Linder |
  | Author | Adam Johannes Johansson |
  | Author | Tjelvar S. G. Olsson |
  | Author | John Liebeschuetz |
  | Author | Tore Brinck |
  | Volume | 26 |
  | Issue | 9 |
  | Pages | 1079-1095 |
  | Publication | JOURNAL OF COMPUTER-AIDED MOLECULAR DESIGN |
  | ISSN | 0920-654X |
  | Date | September 2012 |
  | DOI | 10.1007/s10822-012-9601-y |
  | Language | English |
  | Abstract | A novel computational Diels-Alderase design, based on a relatively rare form of carboxylesterase from Geobacillus stearothermophilus, is presented and theoretically evaluated. The structure was found by mining the PDB for a suitable oxyanion hole-containing structure, followed by a combinatorial approach to find suitable substrates and rational mutations. Four lead designs were selected and thoroughly modeled to obtain realistic estimates of substrate binding and prearrangement. Molecular dynamics simulations and DFT calculations were used to optimize and estimate binding affinity and activation energies. A large quantum chemical model was used to capture the salient interactions in the crucial transition state (TS). Our quantitative estimation of kinetic parameters was validated against four experimentally characterized Diels-Alderases with good results. The final designs in this work are predicted to have rate enhancements of a parts per thousand 10(3)-10(6) and high predicted proficiencies. This work emphasizes the importance of considering protein dynamics in the design approach, and provides a quantitative estimate of the how the TS stabilization observed in most de novo and redesigned enzymes is decreased compared to a minimal, `ideal' model. The presented design is highly interesting for further optimization and applications since it is based on a thermophilic enzyme (T (opt) = 70 A degrees C). |
  | Date Added | 10/11/2013, 10:29:15 AM |
  | Modified | 10/11/2013, 10:29:15 AM |

  ### Tags:

  - Computational enzyme design
  - DFT
  - Diels-Alder
  - molecular dynamics

  ### Notes:

  - Present a computational design of a Diels-Alderase.

    How SCOP is used:

    Look up fold-level classification of two proteins found during PDB mining for potential candidates, and discuss the vast size and "broad promiscuity" of the superfamily.

    SCOP reference:

    A set of 10 structures with diverse properties were selected for virtual screening of TS models. The hydroxynitrile lyase presented in ref. [37] was one of them, and the focus of this study, the carboxylesterase Est30 from Geobacillus stearothermophilus (PDB entry 1TQH) [98] is another. Although both structures have an a/b-hydrolase fold [99] and share the Ser–His–Asp catalytic triad, the structures differ in how the active site is accessed.

  ### Attachments

  - art%3A10.1007%2Fs10822-012-9601-y.pdf
- ## Computational methods for constructing protein structure models from 3D electron microscopy maps

  |  |  |
  | --- | --- |
  | Type | Journal Article |
  | Author | Juan Esquivel-Rodriguez |
  | Author | Daisuke Kihara |
  | Volume | 184 |
  | Issue | 1 |
  | Pages | 93–102 |
  | Publication | Journal of Structural Biology |
  | Date | October 2013 |
  | DOI | 10.1016/j.jsb.2013.06.008 |
  | Abstract | Protein structure determination by cryo-electron microscopy (EM) has made significant progress in the past decades. Resolutions of EM maps have been improving as evidenced by recently reported structures that are solved at high resolutions close to 3 angstrom. computational methods play a key role in interpreting EM data. Among many computational procedures applied to an EM map to obtain protein structure information, in this article we focus on reviewing computational methods that model protein three-dimensional (3D) structures from a 3D EM density map that is constructed from two-dimensional (2D) maps. The computational methods we discuss range from de novo methods, which identify structural elements in an EM map, to structure fitting methods, where known high resolution structures are fit into a low-resolution EM map. A list of available computational tools is also provided. (C) 2013 Elsevier Inc. All rights reserved. |
  | Date Added | 3/7/2014, 12:08:00 PM |
  | Modified | 3/7/2014, 12:08:00 PM |
- ## Computational Protein Design: The Proteus Software and Selected Applications

  |  |  |
  | --- | --- |
  | Type | Journal Article |
  | Author | Thomas Simonson |
  | Author | Thomas Gaillard |
  | Author | David Mignon |
  | Author | Marcel Schmidt Am Busch |
  | Author | Anne Lopes |
  | Author | Najette Amara |
  | Author | Savvas Polydorides |
  | Author | Audrey Sedano |
  | Author | Karen Druart |
  | Author | Georgios Archontis |
  | Volume | 34 |
  | Issue | 28 |
  | Pages | 2472-2484 |
  | Publication | Journal of Computational Chemistry |
  | ISSN | 0192-8651 |
  | Date | OCT 30 2013 |
  | Extra | WOS:000324919200007 |
  | DOI | 10.1002/jcc.23418 |
  | Abstract | We describe an automated procedure for protein design, implemented in a flexible software package, called Proteus. System setup and calculation of an energy matrix are done with the XPLOR modeling program and its sophisticated command language, supporting several force fields and solvent models. A second program provides algorithms to search sequence space. It allows a decomposition of the system into groups, which can be combined in different ways in the energy function, for both positive and negative design. The whole procedure can be controlled by editing 2-4 scripts. Two applications consider the tyrosyl-tRNA synthetase enzyme and its successful redesign to bind both O-methyl-tyrosine and D-tyrosine. For the latter, we present Monte Carlo simulations where the D-tyrosine concentration is gradually increased, displacing L-tyrosine from the binding pocket and yielding the binding free energy difference, in good agreement with experiment. Complete redesign of the Crk SH3 domain is presented. The top 10000 sequences are all assigned to the correct fold by the SUPERFAMILY library of Hidden Markov Models. Finally, we report the acid/base behavior of the SNase protein. Sidechain protonation is treated as a form of mutation; it is then straightforward to perform constant-pH Monte Carlo simulations, which yield good agreement with experiment. Overall, the software can be used for a wide range of application, producing not only native-like sequences but also thermodynamic properties with errors that appear comparable to other current software packages.Copyright (c) 2013 Wiley Periodicals, Inc. |
  | Date Added | 2/20/2014, 12:24:01 PM |
  | Modified | 2/20/2014, 12:24:01 PM |

  ### Notes:

  - Present the Proteus software for computational protein design.

    How SCOP is used:

    Annotate data set with SCOP fold using SUPERFAMILY to evaluate whether their protein design method is able to design proteins and retain a particular fold.

    SCOP reference:

    . The top 10000 sequences are all assigned to the cor- rect fold by the SUPERFAMILY library of Hidden Markov Models.

    ...

  ### Attachments

  - jcc23418.pdf
- ## Computational structural analysis of proteins of Mycobacterium tuberculosis and a resource for identifying off-targets

  |  |  |
  | --- | --- |
  | Type | Journal Article |
  | Author | Sameer Hassan |
  | Author | Abhimita Debnath |
  | Author | Vasantha Mahalingam |
  | Author | Luke Elizabeth Hanna |
  | URL | http://link.springer.com/article/10.1007/s00894-012-1412-5 |
  | Volume | 18 |
  | Issue | 8 |
  | Pages | 3993–4004 |
  | Publication | Journal of molecular modeling |
  | Date | 2012 |
  | Accessed | 9/23/2013, 10:16:36 AM |
  | Library Catalog | Google Scholar |
  | Date Added | 10/11/2013, 10:29:15 AM |
  | Modified | 10/11/2013, 10:29:15 AM |

  ### Tags:

  - Interesting
  - Off-targets
  - Proteins
  - Structural homologues
  - Structures

  ### Notes:

  - Study structures of M. tuberculosis (MTB) proteins to find good drug targets.  In particular, want to determine if any are structurally similar to those in a hosts's proteome.  This would help in determining if a drug would bind to an off-target.

    "Drug molecules can inevitably bind not only to the intended protein target but also to other off-target pro- teins. There are different approaches that can be used to identify the off targets such as sequence identity be- tween the drug target and off-target, pocket similarity, etc. In this study, we analyzed the structural similarity among proteins of MTB."

    Note that they did run into problems because so many of the structures were not classified in SCOP.

    How SCOP is used:

    Get SCOP class and fold classification for 358 MTB proteins structures.  Use to gather statistics on fold diversity in the MTB proteome.

    SCOP reference:

    Abstract:

    Majority of the MTB proteins belonged to the α/β class. 23 different protein folds are used in the MTB protein structures. Of these, the TIM barrel fold was found to be highly conserved even at very low sequence identity.

    PDB data set

    From 843 protein structures available in PDB [10] for MTB, one representative structure for each gene product was selected resulting in a set of 358 protein structures. This set was domain delineated and not based on entire chain.

    The PDB ID for the 358 protein structure data set were searched against SCOP database (1.75 release) [11] and those having SCOP classification were taken for structural analyses.

    ...

    Structural similarity of proteins

    SCOP entries for multiple chains were removed from the set of 488 entries resulting in 184 entries, and structure super- imposition analysis was performed. Of the 184 SCOP domains 78 showed structural similarity. Of the 78 domains, 65 shared structural similarity with SCOP domains and belong to the same SCOP-defined class and fold, while the remaining 13 domains shared structural architecture with SCOP domains having similar SCOP-defined class but dif- ferent folds (Fig. 3). We observed that sequence identity had a significant negative correlation (−0.834, P value < 0.01) with RMSD (Fig. 4). Majority of the structurally similar proteins had sequence identities ranging from 5 % to 25 % and RMSD ranging from 1.8 Å – 4.0 Å. The vast majority of the proteins in this cluster belonged to the α/β class of proteins. Using linear regression, the trend line was found to be y03.416 - 0.039 x and 95 % confidence interval of regression coefficient (−0.045, -0.033). Its R2 value was 0.696. The R2 value represents a measure of its goodness- of-fit (the R2 statistic can range from −1 to 1, with 1 representing perfect positive correlation and −1 representing perfect negative correlation).

  ### Attachments

  - art%3A10.1007%2Fs00894-012-1412-5.pdf
  - Snapshot

    Abstract

    ### Abstract

    Advancement in technology has helped to solve structures of several proteins including M. tuberculosis (MTB) proteins. Identifying similarity between protein structures could not only yield valuable clues to their function, but can also be employed for motif finding, protein docking and off-target identification. The current study has undertaken analysis of structures of all MTB gene products with available structures was analyzed. Majority of the MTB proteins belonged to the α/β class. 23 different protein folds are used in the MTB protein structures. Of these, the TIM barrel fold was found to be highly conserved even at very low sequence identity. We identified 21 paralogs and 27 analogs of MTB based on domains and EC classification. Our analysis revealed that many of the current drug targets share structural similarity with other proteins within the MTB genome, which could probably be off-targets. Results of this analysis have been made available in the Mycobacterium tuberculosis Structural Database (http://bmi.icmr.org.in/mtbsd/MtbSD.php/search.php) which is a useful resource for current and novel drug targets of MTB.
- ## Computer-aided antibody design

  |  |  |
  | --- | --- |
  | Type | Journal Article |
  | Author | Daisuke Kuroda |
  | Author | Hiroki Shirai |
  | Author | Matthew P. Jacobson |
  | Author | Haruki Nakamura |
  | URL | http://peds.oxfordjournals.org/content/25/10/507.short |
  | Volume | 25 |
  | Issue | 10 |
  | Pages | 507–522 |
  | Publication | Protein Engineering Design and Selection |
  | Date | 2012 |
  | Accessed | 9/20/2013, 1:19:04 PM |
  | Library Catalog | Google Scholar |
  | Date Added | 2/20/2014, 12:24:01 PM |
  | Modified | 2/20/2014, 12:24:01 PM |

  ### Notes:

  - Review of progress in computer-aided antibody design.

    How SCOP is ued:

    Look up fold classification (Ig-fold) for domains in chain.

    SCOP reference:

    An antibody consists of two types of chains, the light and heavy chains, each of which is composed of multiple domains, all of which assume the common immunoglobulin (Ig)-fold (Murzin et al., 1995; Chothia et al., 1998).

  ### Attachments

  - [HTML] from oxfordjournals.org
  - Protein Engineering, Design and Selection-2012-Kuroda-507-22.pdf
  - Snapshot
- ## Concomitant prediction of function and fold at the domain level with GO-based profiles

  |  |  |
  | --- | --- |
  | Type | Journal Article |
  | Author | Daniel Lopez |
  | Author | Florencio Pazos |
  | Volume | 14 |
  | Issue | 3 |
  | Publication | BMC BIOINFORMATICS |
  | ISSN | 1471-2105 |
  | Date | FEB 28 2013 |
  | DOI | 10.1186/1471-2105-14-S3-S12 |
  | Language | English |
  | Abstract | Predicting the function of newly sequenced proteins is crucial due to the pace at which these raw sequences are being obtained. Almost all resources for predicting protein function assign functional terms to whole chains, and do not distinguish which particular domain is responsible for the allocated function. This is not a limitation of the methodologies themselves but it is due to the fact that in the databases of functional annotations these methods use for transferring functional terms to new proteins, these annotations are done on a whole-chain basis. Nevertheless, domains are the basic evolutionary and often functional units of proteins. In many cases, the domains of a protein chain have distinct molecular functions, independent from each other. For that reason resources with functional annotations at the domain level, as well as methodologies for predicting function for individual domains adapted to these resources are required. We present a methodology for predicting the molecular function of individual domains, based on a previously developed database of functional annotations at the domain level. The approach, which we show outperforms a standard method based on sequence searches in assigning function, concomitantly predicts the structural fold of the domains and can give hints on the functionally important residues associated to the predicted function. |
  | Date Added | 10/25/2013, 4:17:08 PM |
  | Modified | 3/7/2014, 12:09:19 PM |

  ### Tags:

  - Interesting

  ### Notes:

  - Present method for function prediction that was assessed at CAFA.

    How SCOP is used:

    Use SCOP2GO to get domains and assign GO annotations.

    SCOP reference:

    The starting point is the SCOP2GO resource, which contains GO:MF annotations at the structural domain level [18]. SCOP2GO uses an automatic method for dis- cerning which particular domain of a protein chain is responsible for a GO:MF annotation originally assigned to the chain as a whole.

  ### Attachments

  - 1471-2105-14-S3-S12.pdf
- ## Conformational flexibility of the leucine binding protein examined by protein domain coarse-grained molecular dynamics

  |  |  |
  | --- | --- |
  | Type | Journal Article |
  | Author | Iwona Siuda |
  | Author | Lea Thogersen |
  | Volume | 19 |
  | Issue | 11 |
  | Pages | 4931-4945 |
  | Publication | Journal of Molecular Modeling |
  | ISSN | 1610-2940; 0948-5023 |
  | Date | NOV 2013 |
  | Extra | WOS:000326193200033 |
  | DOI | 10.1007/s00894-013-1991-9 |
  | Abstract | Periplasmic binding proteins are the initial receptors for the transport of various substrates over the inner membrane of gram-negative bacteria. The binding proteins are composed of two domains, and the substrate is entrapped between these domains. For several of the binding proteins it has been established that a closed-up conformation exists even without substrate present, suggesting a highly flexible apo-structure which would compete with the ligand-bound protein for the transporter interaction. For the leucine binding protein (LBP), structures of both open and closed conformations are known, but no closed-up structure without substrate has been reported. Here we present molecular dynamics simulations exploring the conformational flexibility of LBP. Coarse grained models based on the MARTINI force field are used to access the microsecond timescale. We show that a standard MARTINI model cannot maintain the structural stability of the protein whereas the ELNEDIN extension to MARTINI enables simulations showing a stable protein structure and nanosecond dynamics comparable to atomistic simulations, but does not allow the simulation of conformational flexibility. A modification to the MARTINI-ELNEDIN setup, referred to as domELNEDIN, is therefore presented. The domELNEDIN setup allows the protein domains to move independently and thus allows for the simulation of conformational changes. Microsecond domELNEDIN simulations starting from either the open or the closed conformations consistently show that also for LBP, the apo-structure is flexible and can exist in a closed form. |
  | Date Added | 2/13/2014, 4:13:17 PM |
  | Modified | 3/7/2014, 12:08:50 PM |

  ### Notes:

  - Use molecular dynamics to study conformation flexibility of the leucine binding protein (LBP).

    How SCOP is used:

    Background on protein structure classification.

    How CATH is used:

    Annotate data set with domain boundaries from CATH.

    SCOP reference:

    The division of a protein into structural domains will often be done in the most sensible way by a trained eye [65], and the well-established fold databases CATH [66] and SCOP [67] rely on human expertise. However, with the pace at which new protein structures are submitted to the protein data bank (PDB [68, 69]), the human expert assignments lag behind, and the development of methods for high-quality automatic assignment of domain boundaries is an active field of research [65, 70–72].

  ### Attachments

  - art%3A10.1007%2Fs00894-013-1991-9.pdf
- ## Conformations of the apo-, substrate-bound and phosphate-bound ATP-binding domain of the Cu(II) ATPase CopB illustrate coupling of domain movement to the catalytic cycle

  |  |  |
  | --- | --- |
  | Type | Journal Article |
  | Author | Samuel Jayakanthan |
  | Author | Sue A. Roberts |
  | Author | Andrzej Weichsel |
  | Author | Jose M. Argueello |
  | Author | Megan M. McEvoy |
  | Volume | 32 |
  | Issue | 5 |
  | Pages | 443-453 |
  | Publication | Bioscience Reports |
  | ISSN | 0144-8463 |
  | Date | OCT 2012 |
  | Extra | WOS:000309642200003 |
  | DOI | 10.1042/BSR20120048 |
  | Abstract | Heavy metal P-18-type ATPases play a critical role in cell survival by maintaining appropriate intracellular metal concentrations. Archaeoglobus fulgidus CopB is a member of this family that transports Cu(II) from the cytoplasm to the exterior of the cell using ATP as energy source. CopB has a 264 amino acid ATPBD (ATP-binding domain) that is essential for ATP binding and hydrolysis as well as ultimately transducing the energy to the transmembrane metal-binding site for metal occlusion and export. The relevant conformations of this domain during the different steps of the catalytic cycle are still under discussion. Through crystal structures of the apo- and phosphate-bound ATPBDs, with limited proteolysis and fluorescence studies of the apo- and substrate-bound states, we show that the isolated ATPBD of CopB cycles from an open conformation in the apo-state to a closed conformation in the substrate-bound state, then returns to an open conformation suitable for product release. The present work is the first structural report of an ATPBD with its physiologically relevant product (phosphate) bound. The solution studies we have performed help resolve questions on the potential influence of crystal packing on domain conformation. These results explain how phosphate is co-ordinated in ATPase transporters and give an insight into the physiologically relevant conformation of the ATPBD at different steps of the catalytic cycle. |
  | Date Added | 2/13/2014, 4:13:17 PM |
  | Modified | 3/7/2014, 12:14:38 PM |
- ## Conservation of complex knotting and slipknotting patterns in proteins

  |  |  |
  | --- | --- |
  | Type | Journal Article |
  | Author | Joanna I. Sulkowska |
  | Author | Eric J. Rawdon |
  | Author | Kenneth C. Millett |
  | Author | Jose N. Onuchic |
  | Author | Andrzej Stasiak |
  | Volume | 109 |
  | Issue | 26 |
  | Pages | E1715–E1723 |
  | Publication | Proceedings of the National Academy of Sciences of the United States of America |
  | Date | June 2012 |
  | DOI | 10.1073/pnas.1205918109 |
  | Abstract | While analyzing all available protein structures for the presence of knots and slipknots, we detected a strict conservation of complex knotting patterns within and between several protein families despite their large sequence divergence. Because protein folding pathways leading to knotted native protein structures are slower and less efficient than those leading to unknotted proteins with similar size and sequence, the strict conservation of the knotting patterns indicates an important physiological role of knots and slipknots in these proteins. Although little is known about the functional role of knots, recent studies have demonstrated a protein-stabilizing ability of knots and slipknots. Some of the conserved knotting patterns occur in proteins forming transmembrane channels where the slipknot loop seems to strap together the transmembrane helices forming the channel. |
  | Date Added | 3/7/2014, 1:06:24 PM |
  | Modified | 3/7/2014, 1:06:24 PM |
- ## Conservation of the three-dimensional structure in non-homologous or unrelated proteins

  |  |  |
  | --- | --- |
  | Type | Journal Article |
  | Author | Konstantinos Sousounis |
  | Author | Carl E. Haney |
  | Author | Jin Cao |
  | Author | Bharath Sunchu |
  | Author | Panagiotis A. Tsonis |
  | URL | http://www.biomedcentral.com/content/pdf/1479-7364-6-10.pdf |
  | Volume | 6 |
  | Issue | 1 |
  | Pages | 10 |
  | Publication | Human genomics |
  | Date | 2012 |
  | Accessed | 9/23/2013, 10:20:00 AM |
  | Library Catalog | Google Scholar |
  | Date Added | 2/20/2014, 12:24:01 PM |
  | Modified | 2/20/2014, 12:24:01 PM |

  ### Notes:

  - Review of 3D structure conservation in 4 non-homologous proteins.

    How SCOP is used:

    Look up fold, superfamily, and family classifications for proteins of interest.

    SCOP reference:

    Globin-like fold is an all-alpha protein fold normally consisting of six alpha helices [12].

    ...

    The heme-binding proteins are part of the actual family of globins [12].

  ### Attachments

  - [PDF] from biomedcentral.com
- ## CONTSOR--a new knowledge-based fold recognition potential, based on side chain orientation and contacts between residue terminal groups

  |  |  |
  | --- | --- |
  | Type | Journal Article |
  | Author | Boris Vishnepolsky |
  | Author | Malak Pirtskhalava |
  | Volume | 21 |
  | Issue | 1 |
  | Pages | 134-141 |
  | Publication | Protein Science |
  | ISSN | 1469-896X |
  | Date | Jan 2012 |
  | Extra | PMID: 22057923 |
  | Journal Abbr | Protein Sci. |
  | DOI | 10.1002/pro.763 |
  | Library Catalog | NCBI PubMed |
  | Language | eng |
  | Abstract | Recognizing the structural similarity without significant sequence identity (fold recognition) is an effective method for protein structure prediction. Previously, we developed a fold recognition potential called SORDIS, which incorporated side chain orientation in relation to hydrophobic core centers, distance of the residues from the protein globule center and secondary structure terms. But this potential does not include terms, based on close contacts between residues. In this paper a new fold recognition potential CONTSOR was presented, which based on SORDIS terms and the term, based on contacts between amino acid terminal groups. The performance of this potential was evaluated on SABmark benchmark for alignment accuracy and on SABmark and Lindahl benchmarks for fold recognition. The results show that CONTSOR has the best performance among other potentials on SABmark benchmark both for alignment accuracy and fold recognition and one of the best performances on Lindahl benchmark. CONTSOR software package is available for download at http://www.lifescience.org.ge/downloads/contsor.zip. |
  | Date Added | 10/11/2013, 10:29:15 AM |
  | Modified | 3/7/2014, 12:10:39 PM |

  ### Tags:

  - alignment
  - Amino Acid Sequence
  - Computational Biology
  - Protein Conformation
  - Protein Folding
  - Proteins
  - protein structure prediction
  - Sequence Alignment
  - Software
  - Structure-Activity Relationship
  - substitution matrix
  - threading
  - twilight zone

  ### Notes:

  - Present a fold-recognition method, CONTSOR

    How SCOP is used:

    Train and validate a fold-recognition method on the Twilight Zone dataset from the SABmark database.  The dataset was derived from ASTRAL, and contains low-sequence similarity sequences with the same fold.

    How CATH is used:

    Background on protein structure classification.

    SCOP reference:

    Benchmarks

    For testing performance we use Twilight Zone set of the 1.65 version of the SABmark reference alignment database,29 which contains single domain sequences with low sequence similarity. The sequences of the Twilight Zone set are taken from SCOP subset provided by the ASTRAL compendium, in which domains have a pairwise Blast E-value of at least 1, for a theoretical database size of 108 resi- dues.32,52 The Twilight Zone set contains 10,667 sequence pairs that include 1740 sequences, joined into 209 folds. For testing performance of fold recog- nition Lindahl & Elofsson benchmark,47 which con- tains 976 protein domains was also used. This benchmark was used because many fold recognition methods were tested on this dataset.

    CATH reference:

    But difficulties with classification some protein structures to certain folds in SCOP56 and CATH57 allow to say about the importance of substructures below the level of the globular domain in protein structure organization.58

  ### Attachments

  - 763\_ftp.pdf
  - PubMed entry
- ## Convergent evolution in structural elements of proteins investigated using cross profile analysis

  |  |  |
  | --- | --- |
  | Type | Journal Article |
  | Author | Kentaro Tomii |
  | Author | Yoshito Sawada |
  | Author | Shinya Honda |
  | URL | http://www.biomedcentral.com/1471-2105/13/11 |
  | Volume | 13 |
  | Issue | 1 |
  | Pages | 11 |
  | Publication | BMC bioinformatics |
  | Date | 2012 |
  | Accessed | 9/20/2013, 1:12:54 PM |
  | Library Catalog | Google Scholar |
  | Date Added | 10/11/2013, 10:29:15 AM |
  | Modified | 3/7/2014, 12:11:00 PM |

  ### Tags:

  - Evolution, Molecular
  - Humans
  - Models, Molecular
  - Peptides
  - Protein Conformation
  - Protein Folding
  - Proteins

  ### Notes:

  - Study evolution of short protein segments using FORTE profile-profile comparison method, which uses sequence-based and structure-based profiles.

    How SCOP is used:

    Train method on SCOP domain data/  Use ASTRAL 40% identity representative set of sequences to build the FORTE sequence profile library.

    Discuss fold and superfamily classifications in the analysis of the segments.

    How CATH is used:

    Look up classifications.

    SCOP references:

    The 12 profiles derived from the structural clusters for 9-residue-long segments showed correlation with sequence profiles in seven different protein folds accord- ing to the SCOP classification. Half of them showed correlation with 18 sequence profiles of segments in proteins that possess an a-a superhelix fold (SCOP ID: a.118). In Table 1 the profile of cluster #181 was appar- ently similar to the profiles of clusters #184, #246, and #247. These were the ‘adjacent-segment’ effects described above. Similarly, the profile of cluster #140 was similar to that of cluster #313 in Table 1 (and also

    ....

    Preserved sequence-structure patterns

    In the cross profile analysis of the 15-residue-long seg- ments, we identified preserved sequence-structure pat- terns that transcend protein superfamily or fold boundaries that were previously undetectable (cf. Table 2)....

    ....

    Preparation of sequence profiles

    The FORTE system (see below) holds the sequence pro- file library of representative proteins whose structures are known. The amino acid sequences of those proteins are derived mainly from the ASTRAL [53] 40% identity list according to the SCOP classification [27]. Represen- tative sequences that are not in SCOP were selected from the PDB entries [54]. The FORTE library includes 7,419 sequence-based profiles.

    CATH reference:

    In the CATH database, the three proteins possess the same a-b plaits topology (CATH ID: 3.30.70); 1p1lA and 1kr4A are classified as having CATH ID: 3.30.70.830 topology, and 1mwqA is classified as a dimeric a+b plaits protein (CATH ID: 3.30.70.1060).

    ...

    In addition, according to the CATH classification [30], most of the 1jnrA fold is in the domain that possesses the FAD/NAD(P)-binding domain topology (CATH ID: 3.50.50.60). 1kthA is cate- gorized into the factor Xa Inhibitor topology (CATH ID: 4.10.410).

    ...

    Moreover, according to SCOP, the region is assigned to other domains that belong to other folds, instead of to the spectrin repeat- like fold, as is true when other classification databases such as CATH and VAST [33] are used.

  ### Attachments

  - 1471-2105-13-11.pdf
- ## CORE: Common Region Extension Based Multiple Protein Structure Alignment for Producing Multiple Solution

  |  |  |
  | --- | --- |
  | Type | Journal Article |
  | Author | Woo-Cheol Kim |
  | Author | Sanghyun Park |
  | Author | Jung-Im Won |
  | URL | http://link.springer.com/article/10.1007/s11390-013-1365-x |
  | Volume | 28 |
  | Issue | 4 |
  | Pages | 647–656 |
  | Publication | Journal of Computer Science and Technology |
  | Date | 2013 |
  | Accessed | 9/23/2013, 10:22:27 AM |
  | Library Catalog | Google Scholar |
  | Short Title | CORE |
  | Date Added | 10/11/2013, 10:29:15 AM |
  | Modified | 10/11/2013, 10:29:15 AM |

  ### Notes:

  - The paper introduces a new technique for protein structural alignment (algorithm using multiple alignment rather than just single.) They do by locating a common region (called CORE) for multiple alignments, which results in a more accurate analysis but slower run time.

    **SCOP Use**

    SCOP is provided as an example of a database with family-level classification of close homologs.

    **SCOP Reference**

    When comparing the alignments of highly homologous proteins (e.g., the  
    family level of the hierarchical SCOP structural classi¯-  
    cation database[32]) the average length of an alignment  
    and the average lengths of the longest fragment-pair are  
    127 and 47, respectively.

  ### Attachments

  - [PDF] from ict.ac.cn
  - Snapshot
- ## Core Site-Moiety Maps Reveal Inhibitors and Binding Mechanisms of Orthologous Proteins by Screening Compound Libraries

  |  |  |
  | --- | --- |
  | Type | Journal Article |
  | Author | Kai-Cheng Hsu |
  | Author | Wen-Chi Cheng |
  | Author | Yen-Fu Chen |
  | Author | Hung-Jung Wang |
  | Author | Ling-Ting Li |
  | Author | Wen-Ching Wang |
  | Author | Jinn-Moon Yang |
  | Volume | 7 |
  | Issue | 2 |
  | Publication | Plos One |
  | ISSN | 1932-6203 |
  | Date | FEB 29 2012 |
  | Extra | WOS:000303003500038 |
  | DOI | 10.1371/journal.pone.0032142 |
  | Abstract | Members of protein families often share conserved structural subsites for interaction with chemically similar moieties despite low sequence identity. We propose a core site-moiety map of multiple proteins (called CoreSiMMap) to discover inhibitors and mechanisms by profiling subsite-moiety interactions of immense screening compounds. The consensus anchor, the subsite-moiety interactions with statistical significance, of a CoreSiMMap can be regarded as a "hot spot" that represents the conserved binding environments involved in biological functions. Here, we derive the CoreSiMMap with six consensus anchors and identify six inhibitors (IC50 < 8.0 mu M) of shikimate kinases (SKs) of Mycobacterium tuberculosis and Helicobacter pylori from the NCI database (236,962 compounds). Studies of site-directed mutagenesis and analogues reveal that these conserved interacting residues and moieties contribute to pocket-moiety interaction spots and biological functions. These results reveal that our multi-target screening strategy and the CoreSiMMap can increase the accuracy of screening in the identification of novel inhibitors and subsite-moiety environments for elucidating the binding mechanisms of targets. |
  | Date Added | 10/28/2013, 4:53:08 PM |
  | Modified | 10/28/2013, 4:53:08 PM |

  ### Notes:

  - Present method for discovering inhibitors.

    How SCOP is used:

    Background on protein structure classification.

    SCOP reference:

    Orthologous proteins often perform similar functions, despite low sequence identity. Importantly, they frequently share conserved binding environments for interacting with partners. These proteins and their interacting partners (inhibitors or substrates) can be regarded as a pharmacophore family, which is a group of protein-compound complexes that share similar physical-chemical features and interaction patterns between the proteins and their partners. Such a family is analogous to a protein sequence family [7,8] and a protein structure family [9].

  ### Attachments

  - journal.pone.0032142.pdf
- ## Correlation of genomic features with dynamic modularity in the yeast interactome: a view from the structural perspective

  |  |  |
  | --- | --- |
  | Type | Journal Article |
  | Author | Haiying Wang |
  | Author | Huiru Zheng |
  | URL | http://ieeexplore.ieee.org/xpls/abs\_all.jsp?arnumber=6298045 |
  | Volume | 11 |
  | Issue | 3 |
  | Pages | 244–250 |
  | Publication | NanoBioscience, IEEE Transactions on |
  | Date | 2012 |
  | Accessed | 9/23/2013, 10:21:39 AM |
  | Library Catalog | Google Scholar |
  | Short Title | Correlation of genomic features with dynamic modularity in the yeast interactome |
  | Date Added | 10/11/2013, 10:29:15 AM |
  | Modified | 10/11/2013, 10:29:15 AM |

  ### Tags:

  - Date and party hub proteins
  - dynamic modularity
  - interaction interfaces
  - protein interaction networks

  ### Notes:

  - Hub proteins are those with many different interactions.  Party hubs interact with most of their partners simultaneously, while date hubs bind different partners at different locations and times.  This paper aims to extend previous studies on the validity of the date/party hub distinctions by studying 3D properties and interfaces.  In particular, study whether multi- and single- interfaces are more associated with party or date hub proteins.

    How SCOP is used:

    Use Interpro database to get SCOP domain data for a PPI data set.  Then used domains to study the interfaces.

    SCOP reference:

    To create the structural interaction network of the FHC dataset, all the interactions from the dataset were mapped to SCOP (SCOP: a structural classification of proteins database) domains [21] by referring to the Interpro database [22], which provides an integrative protein signature representing protein domains, families, and functional sites. Only those interactions in which both interaction partners can be found in the database of protein structural interactome map (PSIMAP) [23] were kept. Based on the analysis of known crystal structures of proteins and complexes recorded in Protein Data Bank (PDB) [24], PSIMAP includes information about interfacial residue pairs in physical domain-domain interactions. It checks inter- actions between every possible pair of structural domains in a protein by means of the calculation of the Euclidean distance. In this study, the PSIMAP MySQL database provided by Jung et al. [26] was used. The network resulting from this analysis referred to as SFHC hereafter contains 1234 proteins linked by 2351 interactions.

  ### Attachments

  - 06298045.pdf
  - Snapshot
- ## Counterbalance of ligand- and self-coupled motions characterizes multispecificity of ubiquitin

  |  |  |
  | --- | --- |
  | Type | Journal Article |
  | Author | Bhaskar Dasgupta |
  | Author | Haruki Nakamura |
  | Author | Akira R. Kinjo |
  | Volume | 22 |
  | Issue | 2 |
  | Pages | 168-178 |
  | Publication | Protein Science |
  | ISSN | 0961-8368 |
  | Date | February 2013 |
  | DOI | 10.1002/pro.2195 |
  | Language | English |
  | Abstract | Date hub proteins are a type of proteins that show multispecificity in a time-dependent manner. To understand dynamic aspects of such multispecificity we studied Ubiquitin as a typical example of a date hub protein. Here we analyzed 9 biologically relevant Ubiquitin-protein (ligand) heterodimer structures by using normal mode analysis based on an elastic network model. Our result showed that the self-coupled motion of Ubiquitin in the complex, rather than its ligand-coupled motion, is similar to the motion of Ubiquitin in the unbound condition. The ligand-coupled motions are correlated to the conformational change between the unbound and bound conditions of Ubiquitin. Moreover, ligand-coupled motions favor the formation of the bound states, due to its in-phase movements of the contacting atoms at the interface. The self-coupled motions at the interface indicated loss of conformational entropy due to binding. Therefore, such motions disfavor the formation of the bound state. We observed that the ligand-coupled motions are embedded in the motions of unbound Ubiquitin. In conclusion, multispecificity of Ubiquitin can be characterized by an intricate balance of the ligand- and self-coupled motions, both of which are embedded in the motions of the unbound form. |
  | Date Added | 10/11/2013, 10:29:15 AM |
  | Modified | 3/7/2014, 12:10:02 PM |

  ### Tags:

  - coupling of receptor-ligand motions
  - date hub protein
  - functional modes in unbound condition
  - ligand multispecificity
  - normal mode analysis
  - Ubiquitin

  ### Notes:

  - Study of date hub proteins, which show time-dependent multispecificity.  Study ubiquitin as a typical example.

    How SCOP is used:

    Provide fold information on a protein of interest (ubiquitin)

    How CATH is used:

    Cite prior work that used CATH data.

    SCOP reference:

    Here, we took Ubiquitin as a model case for the date hub protein (Fig. 1) because it was found to be most promiscuous among the date hub proteins16 available in the Protein Data Bank (PDB).31 Ubiqui- tin is a small regulatory protein with the b-grasp fold.32

    CATH reference:

    Their anal- ysis showed good correlation between essential and normal modes for a-b CATH44,45 class of proteins, which include Ubiquitin. (3)

  ### Attachments

  - 2195\_ftp.pdf
- ## CPred: a web server for predicting viable circular permutations in proteins

  |  |  |
  | --- | --- |
  | Type | Journal Article |
  | Author | Wei-Cheng Lo |
  | Author | Li-Fen Wang |
  | Author | Yen-Yi Liu |
  | Author | Tian Dai |
  | Author | Jenn-Kang Hwang |
  | Author | Ping-Chiang Lyu |
  | Volume | 40 |
  | Issue | W1 |
  | Pages | W232-W237 |
  | Publication | Nucleic Acids Research |
  | ISSN | 0305-1048 |
  | Date | July 2012 |
  | DOI | 10.1093/nar/gks529 |
  | Language | English |
  | Abstract | Circular permutation (CP) is a protein structural rearrangement phenomenon, through which nature allows structural homologs to have different locations of termini and thus varied activities, stabilities and functional properties. It can be applied in many fields of protein research and bioengineering. The limitation of applying CP lies in its technical complexity, high cost and uncertainty of the viability of the resulting protein variants. Not every position in a protein can be used to create a viable circular permutant, but there is still a lack of practical computational tools for evaluating the positional feasibility of CP before costly experiments are carried out. We have previously designed a comprehensive method for predicting viable CP cleavage sites in proteins. In this work, we implement that method into an efficient and user-friendly web server named CPred (CP site predictor), which is supposed to be helpful to promote fundamental researches and biotechnological applications of CP. The CPred is accessible at http://sarst.life.nthu.edu.tw/CPred. |
  | Date Added | 10/25/2013, 4:17:08 PM |
  | Modified | 11/12/2013, 4:28:28 PM |

  ### Tags:

  - ASTRAL
  - ASTRAL domain structures
  - ASTRAL sequences
  - Cite ASTRAL

  ### Notes:

  - Present CPred server for predicting viable circular permutation cleavage sites in proteins.  CP is structural rearrangement in proteins where the locations of termini are varied.

    How SCOP is used:

    Permit protein structures to be input with PDB or SCOP identifier.  Not explicitly specified, but it seems that their database contains all Astral domain sequences and structures.

    SCOP reference:

     If the protein structure is input by specifying a PDB [Protein Data Bank (31)] or a Structural Classification of Proteins (32) entry identifier, the calculated feature values and final results will be cached to ensure a quick response once the same protein is queried again in the future.

  ### Attachments

  - Nucl. Acids Res.-2012-Lo-W232-7.pdf
- ## Criteria For An Updated Classification of Human Transcription Factor Dna-binding Domains

  |  |  |
  | --- | --- |
  | Type | Journal Article |
  | Author | Edgar Wingender |
  | Volume | 11 |
  | Issue | 1 |
  | Pages | 1340007 |
  | Publication | Journal of Bioinformatics and Computational Biology |
  | Date | February 2013 |
  | DOI | 10.1142/S0219720013400076 |
  | Abstract | By binding to cis-regulatory elements in a sequence-specific manner, transcription factors regulate the activity of nearby genes. Here, we discuss the criteria for a comprehensive classification of human TFs based on their DNA-binding domains. In particular, classification of basic leucine zipper (bZIP) and zinc finger factors is exemplarily discussed. The resulting classification can be used as a template for TFs of other biological species. |
  | Date Added | 3/7/2014, 12:08:00 PM |
  | Modified | 3/7/2014, 12:08:00 PM |
- ## Crowding, molecular volume and plasticity: An assessment involving crystallography, NMR and simulations

  |  |  |
  | --- | --- |
  | Type | Journal Article |
  | Author | M. Selvaraj |
  | Author | Rais Ahmad |
  | Author | Umesh Varshney |
  | Author | M. Vijayan |
  | URL | http://link.springer.com/article/10.1007/s12038-012-9276-5 |
  | Volume | 37 |
  | Issue | 1 |
  | Pages | 953–963 |
  | Publication | Journal of biosciences |
  | Date | 2012 |
  | Accessed | 9/23/2013, 10:18:03 AM |
  | Library Catalog | Google Scholar |
  | Short Title | Crowding, molecular volume and plasticity |
  | Date Added | 10/11/2013, 10:29:15 AM |
  | Modified | 10/11/2013, 10:29:15 AM |

  ### Tags:

  - Molecular crowding
  - molecular plasticity
  - molecular shape
  - peptidyl-tRNA hydrolase
  - Protein function

  ### Notes:

  - Compare NMR and Xray structures of Mycobacterium tuberculosis peptidyl-tRNA hydrolase and find discrepancy between the two, which shows the plasticity of the molecule. They also studied structure with molecular dynamics simulations and found that crowding and molecular volume were inversely related.

    **SCOP Use**

    SCOP was used just to look up the fold of their protein in the classification as well as the domain structure.

    **SCOP Reference**

    According to the SCOP classification (Murzin et al. 1995), MtPth has a  
    phosphorylase/hydrolase-like fold involving a twisted β-sheet (residues 6-9, 41-42, 49-55, 59-63,  
    90-96, 105-108 and 131-136) flanked by two helices on either side (22-35 and 156-159, and 69-  
    83 and 116-125) (Figure 1a).

  ### Attachments

  - [PDF] from ias.ac.in
  - Snapshot
- ## Crucial Protein Based Drug Targets and Potential Inhibitors for Osteoporosis: New Hope and Possibilities

  |  |  |
  | --- | --- |
  | Type | Journal Article |
  | Author | Chiranjib Chakraborty |
  | Author | C. George Priya Doss |
  | Volume | 14 |
  | Issue | 14 |
  | Pages | 1707-1713 |
  | Publication | Current Drug Targets |
  | ISSN | 1389-4501; 1873-5592 |
  | Date | DEC 2013 |
  | Extra | WOS:000329121200011 |
  | Abstract | Osteoporosis, a multifaceted bone disorder, is considered as a serious health problem throughout the world and the magnitude of diseased patients is increasing day by day. A number of successful therapeutics is available in the market for treatment. However, upon long-term administration, most of these drugs cause side-effects with some limitations. Henceforth, development of new therapeutic strategies can be a way to solve this problem as well as to develop cost effective and better tolerated therapies. Detailed understanding about the mechanistic action of the drug targets can be a great aid in the drug development process. Here in this review, we discussed the existing potential protein target class and their inhibitors related to osteoporosis. We have highlighted the existing potential protein drug targets, oestrogen receptor, calcium sensing receptor, P2Y receptor, activin receptor, calcitonin receptor, therapeutic manipulation of protease class such as cathepsin K, targeting WNT/ beta-catenin signaling and targeting RANK-RANKL signaling pathway, TNF inhibition through TNF receptor. We hope this detailed report will provide a better way of understanding towards the discovery and also for development of novel therapeutics. |
  | Date Added | 2/12/2014, 2:18:08 PM |
  | Modified | 10/8/2014, 12:50:50 PM |

  ### Notes:

  - Unavailable.
- ## Crystal and Solution Studies Reveal That the Transcriptional Regulator AcnR of Corynebacterium glutamicum Is Regulated by Citrate-Mg2+ Binding to a Non-canonical Pocket

  |  |  |
  | --- | --- |
  | Type | Journal Article |
  | Author | Javier García-Nafría |
  | Author | Meike Baumgart |
  | Author | Johan P. Turkenburg |
  | Author | Anthony J. Wilkinson |
  | Author | Michael Bott |
  | Author | Keith S. Wilson |
  | URL | http://www.jbc.org/content/288/22/15800.short |
  | Volume | 288 |
  | Issue | 22 |
  | Pages | 15800–15812 |
  | Publication | Journal of Biological Chemistry |
  | Date | 2013 |
  | Accessed | 9/20/2013, 1:18:20 PM |
  | Library Catalog | Google Scholar |
  | Date Added | 10/11/2013, 10:29:15 AM |
  | Modified | 10/11/2013, 10:29:15 AM |

  ### Notes:

  - Study regulation of AcnR,  an aconitase repressor and report on two structures of AcnR

    How SCOP is used:

    Use type: study a protein of interest

    Description: Search SCOP for homologs.  Not clear what tool was used to search SCOP with "coordinates"

    SCOP reference:

    DISCUSSION

    The AcnR Fold—The structure is typical of the TetR family of transcriptional regulators with a homodimer in an ⬚⬚-shape with a DBD and a LBD. A search in SCOP (58) with the AcnR coordinates showed as main hits two known repressors belong- ing to the TetR family, the QacR protein (Protein Data Bank code 1JTO) and the ⬚⬚-butyrolactone receptor (Protein Data Bank code 1UI5).

  ### Attachments

  - [HTML] from jbc.org
  - J. Biol. Chem.-2013-García-Nafría-15800-12.pdf
  - Snapshot
- ## Crystal structure and nucleic acid-binding activity of the CRISPR-associated protein Csx1 of Pyrococcus furiosus

  |  |  |
  | --- | --- |
  | Type | Journal Article |
  | Author | Young Kwan Kim |
  | Author | Yeon-Gil Kim |
  | Author | Byung-Ha Oh |
  | Volume | 81 |
  | Issue | 2 |
  | Pages | 261–270 |
  | Publication | Proteins-structure Function and Bioinformatics |
  | Date | February 2013 |
  | DOI | 10.1002/prot.24183 |
  | Abstract | In many prokaryotic organisms, chromosomal loci known as clustered regularly interspaced short palindromic repeats (CRISPRs) and CRISPR-associated (CAS) genes comprise an acquired immune defense system against invading phages and plasmids. Although many different Cas protein families have been identified, the exact biochemical functions of most of their constituents remain to be determined. In this study, we report the crystal structure of PF1127, a Cas protein of Pyrococcus furiosus DSM 3638 that is composed of 480 amino acids and belongs to the Csx1 family. The C-terminal domain of PF1127 has a unique beta-hairpin structure that protrudes out of an a-helix and contains several positively charged residues. We demonstrate that PF1127 binds double-stranded DNA and RNA and that this activity requires an intact beta-hairpin and involve the homodimerization of the protein. In contrast, another Csx1 protein from Sulfolobus solfataricus P2 that is composed of 377 amino acids does not have the beta-hairpin structure and exhibits no DNA-binding properties under the same experimental conditions. Notably, the C-terminal domain of these two Csx1 proteins is greatly diversified, in contrast to the conserved N-terminal domain, which appears to play a common role in the homodimerization of the protein. Thus, although P. furiosus Csx1 is identified as a nucleic acid-binding protein, other Csx1 proteins are predicted to exhibit different individual biochemical activities. Proteins 2013. (C) 2012 Wiley Periodicals, Inc. |
  | Date Added | 3/7/2014, 1:06:24 PM |
  | Modified | 3/7/2014, 1:06:24 PM |
- ## Crystal structure of a putative isochorismatase hydrolase from Oleispira antarctica

  |  |  |
  | --- | --- |
  | Type | Journal Article |
  | Author | Anna M Goral |
  | Author | Karolina L Tkaczuk |
  | Author | Maksymilian Chruszcz |
  | Author | Olga Kagan |
  | Author | Alexei Savchenko |
  | Author | Wladek Minor |
  | Volume | 13 |
  | Issue | 1 |
  | Pages | 27-36 |
  | Publication | Journal of structural and functional genomics |
  | ISSN | 1570-0267 |
  | Date | Mar 2012 |
  | Extra | PMID: 22350524 |
  | Journal Abbr | J. Struct. Funct. Genomics |
  | DOI | 10.1007/s10969-012-9127-5 |
  | Library Catalog | NCBI PubMed |
  | Language | eng |
  | Abstract | Isochorismatase-like hydrolases (IHL) constitute a large family of enzymes divided into five structural families (by SCOP). IHLs are crucial for siderophore-mediated ferric iron acquisition by cells. Knowledge of the structural characteristics of these molecules will enhance the understanding of the molecular basis of iron transport, and perhaps resolve which of the mechanisms previously proposed in the literature is the correct one. We determined the crystal structure of the apo-form of a putative isochorismatase hydrolase OaIHL (PDB code: 3LQY) from the antarctic γ-proteobacterium Oleispira antarctica, and did comparative sequential and structural analysis of its closest homologs. The characteristic features of all analyzed structures were identified and discussed. We also docked isochorismate to the determined crystal structure by in silico methods, to highlight the interactions of the active center with the substrate. The putative isochorismate hydrolase OaIHL from O. antarctica possesses the typical catalytic triad for IHL proteins. Its active center resembles those IHLs with a D-K-C catalytic triad, rather than those variants with a D-K-X triad. OaIHL shares some structural and sequential features with other members of the IHL superfamily. In silico docking results showed that despite small differences in active site composition, isochorismate binds to in the structure of OaIHL in a similar mode to its binding in phenazine biosynthesis protein PhzD (PDB code 1NF8). |
  | Date Added | 2/20/2014, 12:24:01 PM |
  | Modified | 3/7/2014, 12:14:49 PM |

  ### Tags:

  - Crystallography, X-Ray
  - Hydrolases
  - Oceanospirillaceae
  - Protein Structure, Tertiary
  - Structural Homology, Protein

  ### Notes:

  - Present a crystal structure of the apo-form of putative isochorismatase hydrolase and do comparative sequential and structural analyses of its closest homologs.

    How SCOP is use:

    Look up SCOP fold, superfamily, and family classification for protein of interest.

    How CATH is used:

    Search for similar structures in CATH database.

    SCOP reference:

    Abstract Isochorismatase-like hydrolases (IHL) constitute a large family of enzymes divided into five structural families (by SCOP). IHLs are crucial for siderophore-mediated ferric iron acquisition by cells. Knowledge of the structural char- acteristics of these molecules will enhance the understanding of the molecular basis of iron transport, and perhaps resolve which of the mechanisms previously proposed in the literature is the correct one.

    ...

    Results

    Overall structure

    The OaIHL protein consists of 190 residues. The protein crystallizes in a hexagonal P322 space group with one

    olypeptide chain in the asymmetric unit (data collection and refinement parameters are shown in Table 1). It belongs to the class of ‘alpha–beta–alpha sandwich’ structures according to both the SCOP [41] and CATCH [42] protein structural classification criteria. It adapts the isochorismatase-like hydrolase fold, characterized by a twisted 6-strand parallel b-sheet (the order of b-strands in the sheet is 3-2-1-4-5-6 relative to the primary sequence) flanked by 6 a-helices (in a Rossmann fold) and an addi- tional 2-strand antiparallel b-sheet (residues 152–154 and 157–159, which correspond to strands b6 and b7 in Fig. 2). This antiparallel b-sheet fragment may exhibit interesting stabilization properties, and is a unique feature among proteins in the IHL superfamily. Two glycerol molecules, which originate from the cryoprotectant solution, are ordered in the structure. The overall structure of OaIHL is shown in Fig. 2.

     CATH reference:

    Searches against the CATH database showed that the domain structure most similar to OaIHL is a Yecd domain (PDB code 1J2R, CATH ID: 3.40.50.850.2.1.1.1.1), which is 33% identical by sequence to OaIHL. By comparison, PhzD (CATH ID: 3.40.50.850.1.1.1.1.1) is 21% identical by sequence to OaIHL.

    SCOP/CATH reference:

    Active center

    So far 22 nonredundant structures were deposited in the PBD of members of the isochorismatase-like superfamil (IHL). 11 of them were classified by CATH, 9 by SCOP and 16 by the PFAM database [45]. Table 3 lists structures of homologs of OaIHL classified by SCOP.

  ### Attachments

  - art%3A10.1007%2Fs10969-012-9127-5.pdf
- ## Crystal Structure of the Catalytic Domain of the Bacillus cereus SleB Protein, Important in Cortex Peptidoglycan Degradation during Spore Germination

  |  |  |
  | --- | --- |
  | Type | Journal Article |
  | Author | Yunfeng Li |
  | Author | Kai Jin |
  | Author | Barbara Setlow |
  | Author | Peter Setlow |
  | Author | Bing Hao |
  | Volume | 194 |
  | Issue | 17 |
  | Pages | 4537-4545 |
  | Publication | Journal of Bacteriology |
  | ISSN | 0021-9193 |
  | Date | SEP 2012 |
  | Extra | WOS:000307683100006 |
  | DOI | 10.1128/JB.00877-12 |
  | Abstract | The SleB protein is one of two redundant cortex-lytic enzymes (CLEs) that initiate the degradation of cortex peptidoglycan (PG), a process essential for germination of spores of Bacillus species, including Bacillus anthracis. SleB has been characterized as a soluble lytic transglycosylase that specifically recognizes spore cortex PG and catalyzes the cleavage of glycosidic bonds between N-acetylmuramic acid (NAM) and N-acetylglucosamine residues with concomitant formation of a 1,6-anhydro bond in the NAM residue. We found that like the full-length Bacillus cereus SleB, the catalytic C-terminal domain (SleB(C)) exhibited high degradative activity on cortex PG in vitro, although SleB's N-terminal domain, thought to bind PG, was inactive. The 1.85-angstrom crystal structure of SleB(C) reveals an ellipsoid molecule with two distinct domains dominated by either alpha helices or beta strands. The overall fold of SleB closely resembles that of the catalytic domain of the family 1 lytic transglycosylases but with a completely different topological arrangement. Structural analysis shows that an invariant Glul 57 of SleB is in a position equivalent to that of the catalytic glutamate in other lytic transglycosylases. Indeed, SleB bearing a Glu157-to-Gln mutation lost its cortex degradative activity completely. In addition, the other redundant CLE (called Cw1J) in Bacillus species likely has a three-dimensional structure similar to that of SleB, including the invariant putative catalytic Glu residue. SleB and Cw1J may offer novel targets for the development of anti-spore agents. |
  | Date Added | 2/13/2014, 4:13:17 PM |
  | Modified | 3/7/2014, 12:14:23 PM |
- ## Crystal structure of the conserved domain of the DC lysosomal associated membrane protein: implications for the lysosomal glycocalyx

  |  |  |
  | --- | --- |
  | Type | Journal Article |
  | Author | Sonja Wilke |
  | Author | Joern Krausze |
  | Author | Konrad Büssow |
  | URL | http://www.biomedcentral.com/1741-7007/10/62/ |
  | Volume | 10 |
  | Issue | 1 |
  | Pages | 62 |
  | Publication | BMC biology |
  | Date | 2012 |
  | Accessed | 9/20/2013, 1:16:24 PM |
  | Library Catalog | Google Scholar |
  | Short Title | Crystal structure of the conserved domain of the DC lysosomal associated membrane protein |
  | Date Added | 10/11/2013, 10:29:15 AM |
  | Modified | 10/11/2013, 10:29:15 AM |

  ### Notes:

  - The crystal structure of the conserved domain of human DC-LAMP was solved.

    How SCOP is used:

    Use SCOP to verify that the studied protein has a novel fold.

    SCOP reference:

    According to the SCOP database [41], only two pro- teins are known to contain pseudo b-prism domains, a carbohydrate receptor binding protein of Lactococcus lactis phage P2 (UniProt Q71AW2, PDB 1ZRU, 2-140) [43] and a tail protein of Bordetella phage BPP-1 (Uni- Prot Q775D6, PDB 1YU0, 5-170) [44]. The topology of the b-sheets (that is, the order of the b-strands) of the DC-LAMP domain differs from the other pseudo b- prism structures. The DC-LAMP domain therefore represents a novel fold (Alexey Murzin, personal communication).

  ### Attachments

  - 1741-7007-10-62.pdf
  - [HTML] from biomedcentral.com
- ## Crystal structure of the predicted phospholipase LYPLAL1 reveals unexpected functional plasticity despite close relationship to acyl protein thioesterases

  |  |  |
  | --- | --- |
  | Type | Journal Article |
  | Author | Marco Bürger |
  | Author | Tobias J. Zimmermann |
  | Author | Yasumitsu Kondoh |
  | Author | Patricia Stege |
  | Author | Nobumoto Watanabe |
  | Author | Hiroyuki Osada |
  | Author | Herbert Waldmann |
  | Author | Ingrid R. Vetter |
  | URL | http://www.jlr.org/content/53/1/43.short |
  | Volume | 53 |
  | Issue | 1 |
  | Pages | 43–50 |
  | Publication | Journal of lipid research |
  | Date | 2012 |
  | Accessed | 9/20/2013, 1:18:20 PM |
  | Library Catalog | Google Scholar |
  | Date Added | 10/11/2013, 10:29:15 AM |
  | Modified | 10/11/2013, 10:29:15 AM |

  ### Tags:

  - alpha/beta hydrolase
  - chemical array screening
  - Crystallography, X-Ray
  - Humans
  - inhibitor
  - Lipase
  - lysophospholipase
  - Lysophospholipase
  - Models, Molecular
  - Phospholipases
  - Substrate Specificity

  ### Notes:

  - Experimental and computational study of the function and structure of the protein LYPLAL1. Also examine the relationship of its relationship LYPLAL1 to APT1.

    **SCOP Use**

    SCOP is briefly mentioned as a known database to have the crystal structures for the lysophospholipase family. These are noted to be two uncharacterized proteins under the SCOP family Carboxylesterase/thioesterase 1.

    **SCOP Reference**

    The only other crystal structures known among the lysophospholipase family are carboxylesterase from two different bacteria (e.g., PDB ID 1auo, Fig. 1 includes the corresponding sequence labeled “CARB\_H21.01\_P\_fluorescens\_1AUO”) and two uncharacterized proteins (PDB ID 3b5e and 2r8b, SCOP database, Carboxylesterase/thioesterase 1 family) (3).

  ### Attachments

  - [HTML] from jlr.org
  - J. Lipid Res.-2012-Bürger-43-50.pdf
  - PubMed entry
  - Snapshot
- ## Crystal structure of the protein from Arabidopsis thaliana gene At5g06450, a putative DnaQ-like exonuclease domain-containing protein with homohexameric assembly

  |  |  |
  | --- | --- |
  | Type | Journal Article |
  | Author | David W. Smith |
  | Author | Mi Ra Han |
  | Author | Joon Sung Park |
  | Author | Kyung Rok Kim |
  | Author | Taeho Yeom |
  | Author | Ji Yeon Lee |
  | Author | Do Jin Kim |
  | Author | Craig A. Bingman |
  | Author | Hyun-Jung Kim |
  | Author | Kyubong Jo |
  | Author | Byung Woo Han |
  | Author | George N. Phillips |
  | Volume | 81 |
  | Issue | 9 |
  | Pages | 1669-1675 |
  | Publication | Proteins-Structure Function and Bioinformatics |
  | ISSN | 0887-3585 |
  | Date | SEP 2013 |
  | Extra | WOS:000323386900015 |
  | DOI | 10.1002/prot.24315 |
  | Abstract | Arabidopsis thaliana gene At5g06450 encodes a putative DnaQ-like 3-5 exonuclease domain-containing protein (AtDECP). The DnaQ-like 3-5 exonuclease domain is often found as a proofreading domain of DNA polymerases. The overall structure of AtDECP adopts an RNase H fold that consists of a mixed -sheet flanked by -helices. Interestingly, AtDECP forms a homohexameric assembly with a central six fold symmetry, generating a central cavity. The ring-shaped structure and comparison with WRN-exo, the best structural homologue of AtDECP, suggest a possible mechanism for implementing its exonuclease activity using positively charged patch on the N-terminal side of the homohexameric assembly. The homohexameric structure of AtDECP provides unique information about the interaction between the DnaQ-like 3-5 exonuclease and its substrate nucleic acids.Proteins 2013. (c) 2013 Wiley Periodicals, Inc. |
  | Date Added | 10/28/2013, 4:51:00 PM |
  | Modified | 10/28/2013, 4:51:00 PM |

  ### Notes:

  - Describe crystal structure of a plant protein.

    How SCOP is used:

    Located their protein in SCOP, and use the SCOP description to provide further background.

    SCOP reference:

    At5g06450 (UniProtKB:Q9FNG3) has been annotated from the SCOP program6 as a putative DnaQ-like 30-50 exonuclease domain-containing protein (AtDECP), which contains all three characteristic sequence motifs, ExoI, II, and III [Fig. 1(A)].7

  ### Attachments

  - prot24315.pdf
- ## Cysteine-rich domains related to Frizzled receptors and Hedgehog-interacting proteins

  |  |  |
  | --- | --- |
  | Type | Journal Article |
  | Author | Jimin Pei |
  | Author | Nick V. Grishin |
  | URL | http://onlinelibrary.wiley.com/doi/10.1002/pro.2105/full |
  | Volume | 21 |
  | Issue | 8 |
  | Pages | 1172–1184 |
  | Publication | Protein Science |
  | Date | 2012 |
  | Accessed | 9/23/2013, 10:13:41 AM |
  | Library Catalog | Google Scholar |
  | Date Added | 10/11/2013, 10:29:15 AM |
  | Modified | 10/11/2013, 10:29:15 AM |

  ### Tags:

  - frizzled
  - FZ-CRD
  - glypican
  - Hedgehog-interacting protein
  - HFN-CRD
  - Mid1
  - RECK

  ### Notes:

  - Study of cysteine-rich domains related to Frizzled receptors and Hedgehog-interacting proteins using in-depth sequence and structural analysis.  Used computational analyses to expand the homologous set of FZ-CRDs and HFN-CRDs, providing a better understanding of their evolution and classification.

    How SCOP is used:

    Use HHpred to search SCOP, Pfam, and the eukaryotic proteome database for distant homologs of the cysteine-rich domains of interest.

    SCOP reference:

    HHpred23 was used for profile-profile-based similarity searches to identify distant homologous relationships of FZ-CRDs and HFN-CRDs (profile databases used: Pfam,25 SCOP,63 and the eukaryotic proteome databases).

  ### Attachments

  - 2105\_ftp.pdf
  - [HTML] from wiley.com
- ## Cysteine-Rich Mini-Proteins in Human Biology

  |  |  |
  | --- | --- |
  | Type | Journal Article |
  | Author | Vincent Lavergne |
  | Author | Ryan J. Taft |
  | Author | Paul F. Alewood |
  | Volume | 12 |
  | Issue | 14 |
  | Pages | 1514-1533 |
  | Publication | Current Topics in Medicinal Chemistry |
  | ISSN | 1568-0266 |
  | Date | JUL 2012 |
  | Extra | WOS:000308356300005 |
  | Abstract | Understanding the relationship between structure and function underpins both biochemistry and chemical biology, and has enabled the discovery of numerous agricultural and therapeutic agents. Small cysteine-rich proteins, which form a unique set of protein frameworks and folds, are found in all living organisms and often play crucial roles as hormones, growth factors, ion channel modulators and enzyme inhibitors in various biological pathways. Here we review secreted human cysteine-rich mini-proteins, classify them into broad families and briefly describe their structure and function. To systematically investigate this protein sub-class we designed a step-wise high throughput algorithm that is able to isolate the mature and active forms of human secreted cysteine-rich proteins (up to 200 amino acids in length) and extract their cysteine scaffolds. We limited our search to frameworks that contain an even number of cysteine residues (< 20), all of which are engaged in intra-molecular disulfide bonds. We found 53 different cysteine-rich frameworks spread over 378 secreted cysteine-rich mini-proteins. Restricting our search to those that contain >5% cysteine residues led to the identification of 22 cysteine-rich frameworks representing 21 protein families. Analysis of their molecular targets showed that these mini-proteins are frequently ligands for G protein- and enzyme-coupled receptors, transporters, extracellular enzyme inhibitors, and antimicrobial peptides. It is clear that these human secreted mini-proteins possess a wide diversity of frameworks and folds, some of which are conserved across the phylogenetic spectrum. Further study of these proteins will undoubtedly lead to insights into unresolved questions of basic biology, and the development of system-specific human therapeutics. |
  | Date Added | 2/20/2014, 12:24:01 PM |
  | Modified | 2/20/2014, 12:24:01 PM |

  ### Notes:

  - Paper unavailable.
- ## D2P2: database of disordered protein predictions

  |  |  |
  | --- | --- |
  | Type | Journal Article |
  | Author | M. E. Oates |
  | Author | P. Romero |
  | Author | T. Ishida |
  | Author | M. Ghalwash |
  | Author | M. J. Mizianty |
  | Author | B. Xue |
  | Author | Z. Dosztanyi |
  | Author | V. N. Uversky |
  | Author | Z. Obradovic |
  | Author | L. Kurgan |
  | Author | A. K. Dunker |
  | Author | J. Gough |
  | URL | http://nar.oxfordjournals.org/content/41/D1/D508.short |
  | Volume | 41 |
  | Issue | D1 |
  | Pages | D508-D516 |
  | Publication | Nucleic Acids Research |
  | ISSN | 0305-1048, 1362-4962 |
  | Date | 2012-11-29 |
  | DOI | 10.1093/nar/gks1226 |
  | Accessed | 2/28/2013, 1:36:22 PM |
  | Library Catalog | CrossRef |
  | Short Title | D2P2 |
  | Date Added | 10/11/2013, 10:20:13 AM |
  | Modified | 10/11/2013, 10:20:13 AM |

  ### Notes:

  - Present the database of Disordered Protein Prediction (D^2^P^2^), available at http://d2p2.pro

    Have classified all domains within the database into the SCOP hierarchy using SUPERFAMILY. Also provides a graphical view of their SCOP domains.

    **How using SCOP:**

     Use the SCOP hierarchy, to classify domains (using the SUPERFAMILY server).

    **Quotes**

    **Under ABSTRACT**

    "Integrated with these results are all of the predicted  
    (mostly structured) SCOP domains using the  
    SUPERFAMILY predictor. These disorder/structure  
    annotations together enable comparison of the  
    disorder predictors with each other and examination  
    of the overlap between disordered predictions and  
    SCOP domains on a large scale."

    "An interactive  
    website provides a graphical view of each protein  
    annotated with the SCOP domains..."

  ### Attachments

  - D2P2: database of disordered protein predictions

    We present the Database of Disordered Protein Prediction (D^2^P^2^), available athttp://d2p2.pro (including website source code). A battery of disorder predictors and their variants, VL-XT, VSL2b, PrDOS, PV2, Espritz and IUPred, were run on all protein sequences from 1765 complete proteomes (to be updated as more genomes are completed). Integrated with these results are all of the predicted (mostly structured) SCOP domains using the SUPERFAMILY predictor. These disorder/structure annotations together enable comparison of the disorder predictors with each other and examination of the overlap between disordered predictions and SCOP domains on a large scale. D^2^P^2^ will increase our understanding of the interplay between disorder and structure, the genomic distribution of disorder, and its evolutionary history. The parsed data are made available in a unified format for download as flat files or SQL tables either by genome, by predictor, or for the complete set. An interactive website provides a graphical view of each protein annotated with the SCOP domains and disordered regions from all predictors overlaid (or shown as a consensus). There are statistics and tools for browsing and comparing genomes and their disorder within the context of their position on the tree of life.
  - Nucl. Acids Res.-2013-Oates-D508-16.pdf
- ## Dali/FSSP classification of three-dimensional protein folds.

  |  |  |
  | --- | --- |
  | Type | Journal Article |
  | Author | L Holm |
  | Author | C Sander |
  | URL | http://www.ncbi.nlm.nih.gov/pmc/articles/PMC146389/ |
  | Volume | 25 |
  | Issue | 1 |
  | Pages | 231-234 |
  | Publication | Nucleic Acids Research |
  | ISSN | 0305-1048 |
  | Date | 1997-1-1 |
  | Extra | PMID: 9016542 PMCID: PMC146389 |
  | Journal Abbr | Nucleic Acids Res |
  | Accessed | 10/29/2014, 11:55:32 AM |
  | Library Catalog | PubMed Central |
  | Abstract | The FSSP database presents a continuously updated structural classification of three-dimensional protein folds. It is derived using an automatic structure comparison program (Dali) for the all-against-all comparison of over 6000 three-dimensional coordinate sets in the Protein Data Bank (PDB). Sequence-related protein families are covered by a representative set of 813 protein chains. Hierachical clustering based on structural similarities yields a fold tree that defines 253 fold classes. For each representative protein chain, there is a database entry containing structure-structure alignments with its structural neighbours in the PDB. The database is accessible online through World Wide Web browsers and by anonymous ftp (file transfer protocol). The overview of fold space and the individual data sets provide a rich source of information for the study of both divergent and convergent aspects of molecular evolution, and define useful test sets and a standard of truth for assessing the correctness of sequence-sequence or sequence-structure alignments. |
  | Date Added | 10/29/2014, 11:55:32 AM |
  | Modified | 10/29/2014, 11:55:32 AM |

  ### Attachments

  - PubMed Central Full Text PDF
  - PubMed Central Link
- ## DALIX: Optimal DALI protein structure alignment

  |  |  |
  | --- | --- |
  | Type | Journal Article |
  | Author | Inken Wohlers |
  | Author | Rumen Andonov |
  | Author | Gunnar W. Klau |
  | URL | http://dl.acm.org/citation.cfm?id=2491932 |
  | Volume | 10 |
  | Issue | 1 |
  | Pages | 26–36 |
  | Publication | IEEE/ACM Transactions on Computational Biology and Bioinformatics (TCBB) |
  | Date | 2013 |
  | Accessed | 9/23/2013, 10:18:03 AM |
  | Library Catalog | Google Scholar |
  | Short Title | DALIX |
  | Date Added | 10/11/2013, 10:29:15 AM |
  | Modified | 10/11/2013, 10:29:15 AM |

  ### Notes:

  - Present a mathematical model and exact algorithm for optimally aligning protein structures using the DALI scoring model.

    How SCOP is used:

    Benchmark method on the SCOPCath benchmark dataset of domains in agreement in both CATH and SCOP. Compare for structres with and without the same fold, superfamily, and family.

    SCOP reference:

    3 DATA SETS AND EXPERIMENTAL SETUP

    ...

    SCOPCath [24] is a benchmark containing 6,759 domains that are consistently classified in SCOP [30, version 1.75] and CATH [31, version 3.2.0] and that have a pairwise sequence similarity of less than 50 percent. We align all SCOPCath domains with 30 up to 50 residues that belong to the same family (386 pairs), to different families but to the same superfamily (151 pairs), and to different superfamilies but to the same fold (926 pairs). We limited the length to maximally 50 residues to obtain alignments for which our algorithm can explore multiple branch-and-bound nodes within a few minutes.

    4 RESULTS AND DISCUSSION

    We assess the capability of our algorithm to compute optimal alignments with respect to the DALI scoring function on

    1. 164 SKOLNICK alignments, 2. 1463 SCOPCath alignments, 3. 98 SISY alignments, and  
     4. 22 RIPC alignments.

    ...

    4.2 SCOPCath

    When aligning the short SCOPCath domains, for 661 (45 percent) neither DALI nor DALIX could compute an alignment with positive z-score, especially on fold level. It is likely, but unfortunately not proven by our upper bounds, that when using DALI scoring no such significant alignment exists in many cases. This situation illustrates that it is difficult to design a scoring scheme and algorithm that reliably detects gold standard structural similarities on different classification levels, ranks them correctly and discriminates them from spurious similarities. Structure alignment approaches and their scoring schemes are often benchmarked using these criteria: **The scores of their structure alignments should reproduce the SCOP hierar- chy. A “perfect” scoring function would assign a sig- nificant score to protein pairs related on family, superfamily or fold level**. In doing so, the score would decrease from family to superfamily to fold level. Protein pairs that are not related on any of these levels would not obtain a significant alignment score. In practice, probably no scoring function can meet these requirements for any protein pair. Furthermore, it is hard to evaluate whether an alignment score for a structurally related protein pair is too low because the scoring function inadequately ranks the present structural similarity or because the algorithm fails to report the top-scoring alignment. An exact algorithm maximizing a “perfect” scoring function would return a significant alignment for all protein pairs in the SCOPCath benchmark. Given the DALI score and the DALI and DALIX algorithms, this is not the case. We thus exclude protein pairs from the analysis for which no algorithm returns an alignment with positive z-score.

    ...

    Fig. 5. The barplot bins the percentages of DALI score improvement for the cases in which the DALIX alignment has positive z-score and is better than the DALI alignment. On family level, these are 278, on superfamily level 118 and on fold level 258 alignments. The improvement is computed with respect to the DALI alignment. The DALIX computation time limit is 30 CPU minutes. For most alignments, the score improvement is small. There is furthermore a large percentage of protein pairs that are entirely missed by DALI, i.e., for which DALI falsely reports that there is no structural similarity.

  ### Attachments

  - Snapshot
  - ttb2013010026.pdf
- ## Data growth and its impact on the SCOP database: new developments

  |  |  |
  | --- | --- |
  | Type | Journal Article |
  | Author | Antonina Andreeva |
  | Author | Dave Howorth |
  | Author | John-Marc Chandonia |
  | Author | Steven E Brenner |
  | Author | Tim J P Hubbard |
  | Author | Cyrus Chothia |
  | Author | Alexey G Murzin |
  | Volume | 36 |
  | Issue | Database issue |
  | Pages | D419-425 |
  | Publication | Nucleic acids research |
  | ISSN | 1362-4962 |
  | Date | Jan 2008 |
  | Extra | PMID: 18000004 |
  | Journal Abbr | Nucleic Acids Res. |
  | DOI | 10.1093/nar/gkm993 |
  | Library Catalog | NCBI PubMed |
  | Language | eng |
  | Abstract | The Structural Classification of Proteins (SCOP) database is a comprehensive ordering of all proteins of known structure, according to their evolutionary and structural relationships. The SCOP hierarchy comprises the following levels: Species, Protein, Family, Superfamily, Fold and Class. While keeping the original classification scheme intact, we have changed the production of SCOP in order to cope with a rapid growth of new structural data and to facilitate the discovery of new protein relationships. We describe ongoing developments and new features implemented in SCOP. A new update protocol supports batch classification of new protein structures by their detected relationships at Family and Superfamily levels in contrast to our previous sequential handling of new structural data by release date. We introduce pre-SCOP, a preview of the SCOP developmental version that enables earlier access to the information on new relationships. We also discuss the impact of worldwide Structural Genomics initiatives, which are producing new protein structures at an increasing rate, on the rates of discovery and growth of protein families and superfamilies. SCOP can be accessed at http://scop.mrc-lmb.cam.ac.uk/scop. |
  | Short Title | Data growth and its impact on the SCOP database |
  | Date Added | 10/11/2013, 10:29:15 AM |
  | Modified | 10/11/2013, 10:29:15 AM |

  ### Tags:

  - Databases, Protein
  - Evolution, Molecular
  - Genomics
  - Internet
  - Proteins
  - Protein Structure, Tertiary

  ### Notes:

  - SCOP 1.73 paper

  ### Attachments

  - Nucl. Acids Res.-2008-Andreeva-D419-25.pdf
  - PubMed entry
- ## DBD--taxonomically broad transcription factor predictions: new content and functionality

  |  |  |
  | --- | --- |
  | Type | Journal Article |
  | Author | Derek Wilson |
  | Author | Varodom Charoensawan |
  | Author | Sarah K Kummerfeld |
  | Author | Sarah A Teichmann |
  | Volume | 36 |
  | Issue | Database issue |
  | Pages | D88-92 |
  | Publication | Nucleic acids research |
  | ISSN | 1362-4962 |
  | Date | Jan 2008 |
  | Extra | PMID: 18073188 |
  | Journal Abbr | Nucleic Acids Res. |
  | DOI | 10.1093/nar/gkm964 |
  | Library Catalog | NCBI PubMed |
  | Language | eng |
  | Abstract | DNA-binding domain (DBD) is a database of predicted sequence-specific DNA-binding transcription factors (TFs) for all publicly available proteomes. The proteomes have increased from 150 in the initial version of DBD to over 700 in the current version. All predicted TFs must contain a significant match to a hidden Markov model representing a sequence-specific DNA-binding domain family. Access to TF predictions is provided through http://transcriptionfactor.org, where new search options are now provided such as searching by gene names in model organisms, searching for all proteins in a particular DBD family and specific organism. We illustrate the application of this type of search facility by contrasting trends of DBD family occurrence throughout the tree of life, highlighting the clear partition between eukaryotic and prokaryotic DBD expansions. The website content has been expanded to include dedicated pages for each TF containing domain assignment details, gene names, links to external databases and links to TFs with similar domain arrangements. We compare the increase in number of predicted TFs with proteome size in eukaryotes and prokaryotes. Eukaryotes follow a slower rate of increase in TFs than prokaryotes, which could be due to the presence of splice variants or an increase in combinatorial control. |
  | Short Title | DBD--taxonomically broad transcription factor predictions |
  | Date Added | 10/11/2013, 10:29:15 AM |
  | Modified | 10/11/2013, 10:29:15 AM |

  ### Tags:

  - Animals
  - Databases, Protein
  - DNA-Binding Proteins
  - Humans
  - Internet
  - Markov Chains
  - Protein Structure, Tertiary
  - Proteomics
  - Transcription Factors

  ### Notes:

  - DBD is a database of predicted sequence-specific DNA-binding transcriptions factors (TFs) for all publicly available proteomes.  The prediction method uses HMMs from SUPERFAMILY and Pfam.

    How SCOP is used:

    Use SUPERFAMILY HMMs.

    SCOP reference:

    The HMMs from SUPERFAMILY represent 37 superfamilies and 87 families according to the definitions in the SCOP database (13).

  ### Attachments

  - Nucl. Acids Res.-2008-Wilson-D88-92.pdf
  - PubMed entry
- ## DBETH: A Database of Bacterial Exotoxins for Human

  |  |  |
  | --- | --- |
  | Type | Journal Article |
  | Author | Abhijit Chakraborty |
  | Author | Sudeshna Ghosh |
  | Author | Garisha Chowdhary |
  | Author | Ujjwal Maulik |
  | Author | Saikat Chakrabarti |
  | Volume | 40 |
  | Issue | D1 |
  | Pages | D615–D620 |
  | Publication | Nucleic Acids Research |
  | Date | January 2012 |
  | DOI | 10.1093/nar/gkr942 |
  | Abstract | Pathogenic bacteria produce protein toxins to survive in the hostile environments defined by the host's defense systems and immune response. Recent progresses in high-throughput genome sequencing and structure determination techniques have contributed to a better understanding of mechanisms of action of the bacterial toxins at the cellular and molecular levels leading to pathogenicity. It is fair to assume that with time more and more unknown toxins will emerge not only by the discovery of newer species but also due to the genetic rearrangement of existing bacterial genomes. Hence, it is crucial to organize a systematic compilation and subsequent analyses of the inherent features of known bacterial toxins. We developed a Database for Bacterial ExoToxins (DBETH, http://www.hpppi.iicb.res.in/btox/), which contains sequence, structure, interaction network and analytical results for 229 toxins categorized within 24 mechanistic and activity types from 26 bacterial genuses. The main objective of this database is to provide a comprehensive knowledgebase for human pathogenic bacterial toxins where various important sequence, structure and physico-chemical property based analyses are provided. Further, we have developed a prediction server attached to this database which aims to identify bacterial toxin like sequences either by establishing homology with known toxin sequences/domains or by classifying bacterial toxin specific features using a support vector based machine learning techniques. |
  | Date Added | 3/7/2014, 12:08:00 PM |
  | Modified | 10/8/2014, 1:32:20 PM |

  ### Attachments

  - Full Text PDF
  - PubMed entry
  - Snapshot
- ## dbSNP: the NCBI database of genetic variation

  |  |  |
  | --- | --- |
  | Type | Journal Article |
  | Author | S. T. Sherry |
  | Author | M.-H. Ward |
  | Author | M. Kholodov |
  | Author | J. Baker |
  | Author | L. Phan |
  | Author | E. M. Smigielski |
  | Author | K. Sirotkin |
  | URL | http://www.ncbi.nlm.nih.gov/pmc/articles/PMC29783/ |
  | Volume | 29 |
  | Issue | 1 |
  | Pages | 308-311 |
  | Publication | Nucleic Acids Research |
  | ISSN | 0305-1048 |
  | Date | 2001-1-1 |
  | Extra | PMID: 11125122 PMCID: PMC29783 |
  | Journal Abbr | Nucleic Acids Res |
  | Accessed | 10/10/2014, 5:20:20 PM |
  | Library Catalog | PubMed Central |
  | Abstract | In response to a need for a general catalog of genome variation to address the large-scale sampling designs required by association studies, gene mapping and evolutionary biology, the National Center for Biotechnology Information (NCBI) has established the dbSNP database [S.T.Sherry, M.Ward and K.Sirotkin (1999) Genome Res., 9, 677–679]. Submissions to dbSNP will be integrated with other sources of information at NCBI such as GenBank, PubMed, LocusLink and the Human Genome Project data. The complete contents of dbSNP are available to the public at website: http://www.ncbi.nlm.nih.gov/SNP. The complete contents of dbSNP can also be downloaded in multiple formats via anonymous FTP at ftp://ncbi.nlm.nih.gov/snp/. |
  | Short Title | dbSNP |
  | Date Added | 10/10/2014, 5:20:20 PM |
  | Modified | 10/10/2014, 5:20:20 PM |

  ### Attachments

  - PubMed Central Full Text PDF
  - PubMed Central Link
- ## dcGO: database of domain-centric ontologies on functions, phenotypes, diseases and more

  |  |  |
  | --- | --- |
  | Type | Journal Article |
  | Author | Hai Fang |
  | Author | Julian Gough |
  | Volume | 41 |
  | Issue | D1 |
  | Pages | D536-D544 |
  | Publication | Nucleic Acids Research |
  | ISSN | 0305-1048 |
  | Date | JAN 2013 |
  | Extra | WOS:000312893300076 |
  | DOI | 10.1093/nar/gks1080 |
  | Abstract | We present 'dcGO' (http://supfam.org/SUPERFAMILY/dcGO), a comprehensive ontology database for protein domains. Domains are often the functional units of proteins, thus instead of associating ontological terms only with full-length proteins, it sometimes makes more sense to associate terms with individual domains. Domain-centric GO, 'dcGO', provides associations between ontological terms and protein domains at the superfamily and family levels. Some functional units consist of more than one domain acting together or acting at an interface between domains; therefore, ontological terms associated with pairs of domains, triplets and longer supra-domains are also provided. At the time of writing the ontologies in dcGO include the Gene Ontology (GO); Enzyme Commission (EC) numbers; pathways from UniPathway; human phenotype ontology and phenotype ontologies from five model organisms, including plants; anatomy ontologies from three organisms; human disease ontology and drugs from DrugBank. All ontological terms have probabilistic scores for their associations. In addition to associations to domains and supra-domains, the ontological terms have been transferred to proteins, through homology, providing annotations of > 80 million sequences covering 2414 complete genomes, hundreds of meta-genomes, thousands of viruses and so forth. The dcGO database is updated fortnightly, and its website provides downloads, search, browse, phylogenetic context and other data-mining facilities. |
  | Date Added | 2/20/2014, 12:24:01 PM |
  | Modified | 2/20/2014, 12:24:01 PM |

  ### Notes:

  - Present database for dcGO (domain-centric Gene Ontology)  SUPERFAMILY-based method for function prediction.

    How SCOP is used:

    Annotate domains and get superfamily classification with SUPERFAMILY.

    SCOP reference:

    The domain definitions used in dcGO are taken from the structural classification of proteins (SCOP) (22) clas- sified at both the superfamily and family levels. SCOP groups domains at the superfamily level if there is struc- ture, sequence and function evidence for a common evo- lutionary ancestor. Some superfamilies are sub-divided into families, which often share a higher sequence similarity and a related function. In addition to individual domains at these two different levels, dcGO also offers annotations for combinations of domains. We use the concept of supra-domains to describe combinations of two or more successive domains of known structure. In addition to providing ontology for SCOP domains, the generality of the method has enabled us to also include Pfam (23) domains in dcGO.

    ...

    Searching dcGO

    The faceted search on the dcGO website (Figure 1) is a mining hub for users, with additional bioinformatics tools hyperlinked from the search results. Full-text query is sup- ported for SCOP domains, ontologies and genomes. Identifier or accession number lookup is supported for sequences. Ontologies and SCOP domains are linked to pages for browsing their respective hierarchies. Every genome is presented within its phylogenetic context by linking to a species tree of life (called sTOL, see ‘Analysing GO terms over the species tree of life’ section). There are also links from domains and onto- logical terms to the tree of life (to see their distribution across species). Search results returning BO terms are linked to a cross-ontology comparison tool, the phenotype similarity network (PSnet, see ‘Cross-linking similar phenotypes’ section). PSnet searches for terms from other ontologies with a similar profile of associations. For lookups returning a specific genome sequence, the user is provided with the facility to submit it automatically to the ‘dcGO Predictor’ for function, phenotype and disease prediction. In conclusion, the faceted search is designed for multi-tasking; it does not just provide search results but is intended to interconnect all the tools and cross-referencing abilities of dcGO.

    Browsing the hierarchies

    The ‘BROWSE’ navigation on the website (aforemen- tioned) provides browsing for the SCOP, GO and various BO hierarchies. The hierarchy-like structure of the SCOP (or ontology) has a domain (or term) as a node and its relations to parental nodes as directed edges. To navigate this hierarchy, we display all the paths from the current node upwards to the root ordered by the shortest distances. Also, all direct children of the current node are listed underneath to enable browsing downwards. In addition to the hierarchy itself, a tabbed interface is used to aid the display of domain-centric annotations in a subject-specific manner. The SCOP-orientated hierarchy shows terms used to annotate a domain, and vice versa, the ontology- orientated hierarchy shows domains/supra-domains annotated by a term.

  ### Attachments

  - Nucl. Acids Res.-2013-Fang-D536-44.pdf
- ## Deconstruction of Activity-Dependent Covalent Modification of Heme in Human Neutrophil Myeloperoxidase by Multistage Mass Spectrometry (MS4)

  |  |  |
  | --- | --- |
  | Type | Journal Article |
  | Author | Kieran F. Geoghegan |
  | Author | Alison H. Varghese |
  | Author | Xidong Feng |
  | Author | Andrew J. Bessire |
  | Author | James J. Conboy |
  | Author | Roger B. Ruggeri |
  | Author | Kay Ahn |
  | Author | Samantha N. Spath |
  | Author | Sergey V. Filippov |
  | Author | Steven J. Conrad |
  | URL | http://pubs.acs.org/doi/abs/10.1021/bi201872j |
  | Volume | 51 |
  | Issue | 10 |
  | Pages | 2065–2077 |
  | Publication | Biochemistry |
  | Date | 2012 |
  | Accessed | 9/20/2013, 1:18:37 PM |
  | Library Catalog | Google Scholar |
  | Date Added | 10/11/2013, 10:29:15 AM |
  | Modified | 10/11/2013, 10:29:15 AM |

  ### Notes:

  - Study of myeloperoxidase (MPO) using multistage mass spectrometry.

    How SCOP is used:

    Provide details on superfamily of MPO in SCOP.

    SCOP reference:

    The structure of MPO belongs to the heme-dependent peroxidase superfamily, consisting of 26 α-helices arranged around the central heme moiety.30

  ### Attachments

  - bi201872j.pdf
  - Snapshot
- ## Deep architectures for protein contact map prediction

  |  |  |
  | --- | --- |
  | Type | Journal Article |
  | Author | Pietro Di Lena |
  | Author | Ken Nagata |
  | Author | Pierre Baldi |
  | URL | http://bioinformatics.oxfordjournals.org/content/28/19/2449.short |
  | Volume | 28 |
  | Issue | 19 |
  | Pages | 2449–2457 |
  | Publication | Bioinformatics |
  | Date | 2012 |
  | Accessed | 9/19/2013, 5:13:39 PM |
  | Library Catalog | Google Scholar |
  | Date Added | 10/11/2013, 10:29:15 AM |
  | Modified | 3/7/2014, 12:15:02 PM |

  ### Tags:

  - ASTRAL
  - ASTRAL domain structures
  - ASTRAL sequences
  - ASTRAL subsets
  - Cite ASTRAL

  ### Notes:

  - Novel machine learning algorithm for contact map prediction.

    Protein residue-residue contact map prediction aims to predict a residue contact map from sequence alone.  A new method, CMAPpro, is introduced that employs neural networks.

    How SCOP is used:

    Use a representative subset from ASTRAL 1.73 to train their contact prediction method, then use a benchmarking set derived from ASTRAL 1.75 to evaluate their method.  Compare how method performs overall and on each of the SCOP classes.

    For training, used ASTRAL 1.73 filtered at 20% sequence identity.  For benchmarking, used ASTRAL 1.75 but did their own clustering and filtering to remove redundancy.

    SCOP reference:

    2 MATERIALS AND METHODS

    2.2 Training and test sets

    The training set is derived from the ASTRAL database (Chandonia et al., 2004).  We extract from the ASTRAL release 1.73 the (precompiled) set of protein domains with less than 20% pairwise sequence identity, removing domains of length less than 50 residues, domains with multiple 3D structures, as well as non-contiguous domains (including those with missing backbone atoms). We further filter this list by selecting just one representative domain–the shortest one–per SCOP family (Murzin et al., 1995), ending up with a final set of 2,356 structures. For cross-validation purposes, this set is then partitioned into 10 disjoint groups of roughly the same size and average domain lengths, so that no domains from two distinct groups belong to the same SCOP fold. In this way, training and validation sets share neither sequence nor structural similarities.

    For performance assessment, a non-redundant test set is derived from ASTRAL release 1.75, by selecting all the new folds, with respect to version 1.73, belonging to the main SCOP classes (All-Alpha, All-Beta, Alpha/Beta, Alpha+Beta). From this set (256 new folds, 287 new families) we remove all the domains of length less than 50 residues, and those with less than L/5 long range contacts (239 new folds, 268 new families). Redundancy is filtered out by clustering each group of domains belonging to the same SCOP family at 40% of sequence similarity . The final set of 364 domains contains at least one representative for each one of the 268 new families. A BLAST search with E-value cutoff 0.01 of the test domain sequences against the set of training domain sequences returns no hit.

    3RESULTS AND DISCUSSION

    3.1 Coarse contact and orientation prediction

    We evaluate the average classification performance of the coarse contact and orientation predictor on the three classes Parallel contact (P ), Anti-parallel contact (A) and No-contact (N ) on the 364 test domains (Section 2.2).

    Table 1 reports the cross-validation average performance on the full set of protein domains (All) and as a function of the main structural domain classes: All-Alpha (mainly alpha-helices), All-Beta (mainly beta- sheets), Alpha/Beta (alpha-helices and beta-sheets, mainly parallel beta sheets) and Alpha+Beta (alpha-helices and beta-sheets, mainly anti-parallel beta sheets). As shown in Table 1, the performance of the coarse predictor on the Parallel (P) class are highly affected by the protein structural domain; in particular, the prediction precision and recall are higher for the Alpha/Beta proteins and are quite low for the All-Beta proteins. Conversely, the performance on the Anti-parallel class (A) are nearly uniform, regardless of the domain structural classification. The anti-parallel contacts appear to be easier to predict than the parallel contacts, even within the Alpha+Beta class. Though not directly comparable (due to a different definition of segment-segment contact), the coarse contact predictor has higher precision and lower recall than the 2D-BRNN developed for the same classification problem in Pollastri et al. (2006).

    ...

    3.2 Element alignment prediction

    We evaluate the contact prediction performance of the element alignment predictor at the residue level on the (predicted) strand- strand and helix-helix regions of the contact map. We use the same accuracy measure adopted for the evaluation of contact prediction performance on the entire contact map (Section 2.1).

    ...

    The average accuracy on the 364 test domains for these two probability measures and for long-range residue pairs is reported in Table 2. The prediction accuracy is reported on the full set of protein domains (All), as well as on the main structural classes (All-Alpha, All-Beta, Alpha/Beta, Alpha+Beta).

  ### Attachments

  - [PDF] from predictioncenter.org
  - Snapshot
- ## Defining and predicting structurally conserved regions in protein superfamilies

  |  |  |
  | --- | --- |
  | Type | Journal Article |
  | Author | Ivan K. Huang |
  | Author | Jimin Pei |
  | Author | Nick V. Grishin |
  | URL | http://bioinformatics.oxfordjournals.org/content/29/2/175.short |
  | Volume | 29 |
  | Issue | 2 |
  | Pages | 175–181 |
  | Publication | Bioinformatics |
  | Date | 2013 |
  | Accessed | 9/19/2013, 7:46:28 AM |
  | Library Catalog | Google Scholar |
  | Date Added | 10/11/2013, 10:29:15 AM |
  | Modified | 3/7/2014, 1:06:47 PM |

  ### Tags:

  - ASTRAL
  - ASTRAL domain structures
  - ASTRAL sequences
  - ASTRAL subsets
  - Cite ASTRAL

  ### Notes:

  - First, came up with a measurement of structural conservation (the SCI) to distinguish SCRs from non-SCRs.  Then, trained neural network models to detect SCRs from sequence alone.  Used five-fold cross validation to evaluate their method.

    See: http://prodata.swmed.edu/SCR/index.php

    How SCOP is used:

    Used SCOP data to benchmark their "conserved structure" prediction method.  Derived a data set from ASTRAL domain data filtered at 40%.  Did some filtering on the class, superfamily, and family level to remove types of domains they knew were problematic for DaliLite.  Then they removed superfamilies with fewer than five domains in the ASTRAL representative data set.

    SCOP references:

    Under Abstract:

    Results: Using pairwise DaliLite alignments among a set of homolo- gous structures, we devised a simple measure of structural conserva- tion, termed structural conservation index (SCI). SCI was used to distinguish SCRs from non-SCRs. A database of SCRs was compiled from 386 SCOP superfamilies containing 6489 protein domains.

    Under Methods:

    2.1 Compilation of the SCR database

    2.1.1 Selection of protein superfamilies Our dataset was based on the SCOP (version 1.75) database, which contains protein domain structures divided hierarchically into classes, folds, superfamilies, families, protein domains, species and PDB domains (from highest to lowest). We were particularly interested in the conservation at the superfamily level, which is the largest grouping of evolutionarily related proteins in SCOP that share common structural folds.

    To define the dataset, we only considered the structures in the ASTRAL SCOP40 database (Chandonia et al., 2004). ASTRAL contains a subset of SCOP domains with a level of non-redundancy corresponding to at most 40% sequence identity. We excluded certain superfamilies that we anticipated to have poor alignments by the DaliLite algorithm. In particular, SCOP classes g–k (small proteins, coiled coil proteins, low resolution proteins, peptides and fragments, and designed proteins) were removed. A handful of individual folds and superfamilies in the remaining six classes (all alpha proteins, all beta proteins, a/b proteins, aþb proteins, multi-domain proteins, and membrane and cell surface proteins and peptides) were also omitted from the dataset as they exhibited either high structural variability or topologies, such as repeating or duplicated domains and circular permutations, that could pose prob- lems for DaliLite (a.6.1, a.100.1, a.118, a.138.1, b.34.5, b.82.1, b.84.2, b.108.1, c.1.8, c.10.2, c.37.1, c.47.1, d.2.1, d.3.1, d.52.3, d.133, d.169.1, d.198.1, d.211.1, d.325.1, f.4.1). Finally, superfamilies with fewer than five domains were removed to ensure that there were enough members to provide meaningful structural conservation measurement. In total, 386 superfamilies with a total of 6489 protein domains were used.

  ### Attachments

  - Bioinformatics-2013-Huang-175-81.pdf
  - Full Text PDF
  - PubMed entry
  - Snapshot
- ## Defining and searching for structural motifs using DeepView/Swiss-PdbViewer

  |  |  |
  | --- | --- |
  | Type | Journal Article |
  | Author | Maria U. Johansson |
  | Author | Vincent Zoete |
  | Author | Olivier Michielin |
  | Author | Nicolas Guex |
  | URL | http://www.biomedcentral.com/1471-2105/13/173/ |
  | Volume | 13 |
  | Issue | 1 |
  | Pages | 173 |
  | Publication | BMC bioinformatics |
  | Date | 2012 |
  | Accessed | 9/20/2013, 1:19:56 PM |
  | Library Catalog | Google Scholar |
  | Date Added | 10/11/2013, 10:29:15 AM |
  | Modified | 3/7/2014, 12:14:36 PM |

  ### Notes:

  - Describe an extension of DeepView/Swiss-PdbViewer through which structural motifs may be defined and searched for in large protein structure databases.  Show that common structural motifs involved in stabilizing protein folds are present in evolutionarily and structurally unrelated proteins, also in deeply buried locations which are not obviously related to protein function.

    How SCOP is used:

    Use type: do not use SCOP data.

    In Background section.

    Paper type: software

    Description: Do not use SCOP data.

    SCOP reference:

    During the last twenty years, increasingly sophisticated methods for secondary structure prediction [5,6], fold recognition and compari- son (e.g., FSSP [7], THREADER [8], FOLDFIT [9], and others [10-12] have been developed, followed by meth- ods for fold classification, such as SCOP [13] and CATH [14].

  ### Attachments

  - 1471-2105-13-173.pdf
- ## Defining structural and evolutionary modules in proteins: a community detection approach to explore sub-domain architecture

  |  |  |
  | --- | --- |
  | Type | Journal Article |
  | Author | Jose Sergio Hleap |
  | Author | Edward Susko |
  | Author | Christian Blouin |
  | Volume | 13 |
  | Pages | 20 |
  | Publication | Bmc Structural Biology |
  | ISSN | 1472-6807 |
  | Date | OCT 16 2013 |
  | Extra | WOS:000329231500001 |
  | DOI | 10.1186/1472-6807-13-20 |
  | Abstract | Background: Assessing protein modularity is important to understand protein evolution. Still the question of the existence of a sub-domain modular architecture remains. We propose a graph-theory approach with significance and power testing to identify modules in protein structures. In the first step, clusters are determined by optimizing the partition that maximizes the modularity score. Second, each cluster is tested for significance. Significant clusters are referred to as modules. Evolutionary modules are identified by analyzing homologous structures. Dynamic modules are inferred from sets of snapshots of molecular simulations. We present here a methodology to identify sub-domain architecture robustly, biologically meaningful, and statistically supported. Results: The robustness of this new method is tested using simulated data with known modularity. Modules are correctly identified even when there is a low correlation between landmarks within a module. We also analyzed the evolutionary modularity of a data set of alpha-amylase catalytic domain homologs, and the dynamic modularity of the Niemann-Pick C1 (NPC1) protein N-terminal domain. The alpha-amylase contains an (alpha/beta)(8) barrel (TIM barrel) with the polysaccharides cleavage site and a calcium-binding domain. In this data set we identified four robust evolutionary modules, one of which forms the minimal functional TIM barrel topology. The NPC1 protein is involved in the intracellular lipid metabolism coordinating sterol trafficking. NPC1 N-terminus is the first luminal domain which binds to cholesterol and its oxygenated derivatives. Our inferred dynamic modules in the protein NPC1 are also shown to match functional components of the protein related to the NPC1 disease. Conclusions: A domain compartmentalization can be found and described in correlation space. To our knowledge, there is no other method attempting to identify sub-domain architecture from the correlation among residues. Most attempts made focus on sequence motifs of protein-protein interactions, binding sites, or sequence conservancy. We were able to describe functional/structural sub-domain architecture related to key residues for starch cleavage, calcium, and chloride binding sites in the alpha-amylase, and sterol opening-defining modules and disease-related residues in the NPC1. We also described the evolutionary sub-domain architecture of the alpha-amylase catalytic domain, identifying the already reported minimum functional TIM barrel. |
  | Date Added | 2/12/2014, 2:18:08 PM |
  | Modified | 3/7/2014, 12:09:37 PM |

  ### Notes:

  - Present method for identifying sub-domain architecture/"modules" in protein structure.

    How SCOP/CATH is used:

    Provide background on protein structure classification.

    SCOP/CATH reference:

    The domain architecture has been shown to harbor evolution- ary and structural coherence [9,29,38,89-92].

  ### Attachments

  - 1472-6807-13-20.pdf
- ## Dengue Virus Nonstructural Protein 5 Adopts Multiple Conformations in Solution

  |  |  |
  | --- | --- |
  | Type | Journal Article |
  | Author | Cecile Bussetta |
  | Author | Kyung H. Choi |
  | Volume | 51 |
  | Issue | 30 |
  | Pages | 5921-5931 |
  | Publication | Biochemistry |
  | Date | JUL 31 2012 |
  | Extra | WOS:000308262600006 |
  | DOI | 10.1021/bi300406n |
  | Library Catalog | ISI Web of Knowledge |
  | Abstract | Dengue virus (DENV) nonstructural protein 5 (NS5) is composed of two globular domains separated by a 10-residue linker. The N-terminal domain participates in the synthesis of a mRNA cap 1 structure ((7Me)GpppA(2'OMe)) at the 5' end of the viral genome and possesses guanylyltransferase, guanine-N7-methyltransferase, and nucleoside-2'O-methyltransferase activities. The C-terminal domain is an RNA-dependent RNA polymerase responsible for viral RNA synthesis. Although crystal structures of the two isolated domains have been obtained, there are no structural data for full-length NS5. It is also unclear whether the two NS5 domains interact with each other to form a stable structure in which the relative orientation of the two domains is fixed. To investigate the structure and dynamics of DENV type 3 NS5 in solution, we conducted small-angle X-ray scattering experiments with the full-length protein. NS5 was found to be monomeric and well-folded under the conditions tested. The results of these experiments also suggest that NS5 adopts multiple conformations in solution, ranging from compact to more extended forms in which the two domains do not seem to interact with each other. We interpret the multiple conformations of NS5 observed in solution as resulting from weak interactions between the two NS5 domains and flexibility of the linker in the absence of other components of the replication complex. |
  | Date Added | 10/8/2014, 12:49:22 PM |
  | Modified | 10/8/2014, 1:32:19 PM |

  ### Attachments

  - ACS Full Text PDF w/ Links
  - ACS Full Text Snapshot
- ## Dependence of alpha-helical and beta-sheet amino acid propensities on the overall protein fold type

  |  |  |
  | --- | --- |
  | Type | Journal Article |
  | Author | Kazuo Fujiwara |
  | Author | Hiromi Toda |
  | Author | Masamichi Ikeguchi |
  | Volume | 12 |
  | Pages | 18 |
  | Publication | Bmc Structural Biology |
  | Date | August 2012 |
  | DOI | 10.1186/1472-6807-12-18 |
  | Abstract | Background: A large number of studies have been carried out to obtain amino acid propensities for alpha-helices and beta-sheets. The obtained propensities for alpha-helices are consistent with each other, and the pair-wise correlation coefficient is frequently high. On the other hand, the beta-sheet propensities obtained by several studies differed significantly, indicating that the context significantly affects beta-sheet propensity. Results: We calculated amino acid propensities for alpha-helices and beta-sheets for 39 and 24 protein folds, respectively, and addressed whether they correlate with the fold. The propensities were also calculated for exposed and buried sites, respectively. Results showed that alpha-helix propensities do not differ significantly by fold, but beta-sheet propensities are diverse and depend on the fold. The propensities calculated for exposed sites and buried sites are similar for alpha-helix, but such is not the case for the beta-sheet propensities. We also found some fold dependence on amino acid frequency in beta-strands. Folds with a high Ser, Thr and Asn content at exposed sites in beta-strands tend to have a low Leu, Ile, Glu, Lys and Arg content (correlation coefficient = -0.90) and to have flat beta-sheets. At buried sites in beta-strands, the content of Tyr, Trp, Gln and Ser correlates negatively with the content of Val, Ile and Leu (correlation coefficient = -0.93). "All-beta" proteins tend to have a higher content of Tyr, Trp, Gln and Ser, whereas "alpha/beta" proteins tend to have a higher content of Val, Ile and Leu. Conclusions: The a-helix propensities are similar for all folds and for exposed and buried residues. However, beta-sheet propensities calculated for exposed residues differ from those for buried residues, indicating that the exposed-residue fraction is one of the major factors governing amino acid composition in beta-strands. Furthermore, the correlations we detected suggest that amino acid composition is related to folding properties such as the twist of a beta-strand or association between two beta sheets. |
  | Date Added | 3/7/2014, 12:08:00 PM |
  | Modified | 10/8/2014, 1:32:22 PM |

  ### Attachments

  - Full Text PDF
  - Snapshot
- ## Deriving correlated motions in proteins from X-ray structure refinement by using TLS parameters

  |  |  |
  | --- | --- |
  | Type | Journal Article |
  | Author | Yen-Yi Liu |
  | Author | Chien-Hua Shih |
  | Author | Jenn-Kang Hwang |
  | Author | Chih-Chieh Chen |
  | Volume | 518 |
  | Issue | 1, SI |
  | Pages | 52-58 |
  | Publication | GENE |
  | ISSN | 0378-1119 |
  | Date | APR 10 2013 |
  | Extra | 23rd International Conference on Genome Informatics (GIW), Tainan, TAIWAN, DEC 12-14, 2012 |
  | DOI | 10.1016/j.gene.2012.11.086 |
  | Language | English |
  | Abstract | Dynamic information in proteins may provide valuable information for understanding allosteric regulation of protein complexes or long-range effects of the mutations on enzyme activity. Experimental data such as X-ray B-factors or NMR order parameters provide a convenient estimate of atomic fluctuations (or atomic auto-correlated motions) in proteins. However, it is not as straightforward to obtain atomic cross-correlated motions in proteins - one usually resorts to more sophisticated computational methods such as Molecular Dynamics, normal mode analysis or atomic network models. In this report, we show that atomic cross-correlations can be reliably obtained directly from protein structure using X-ray refinement data. We have derived an analytic form of atomic correlated motions in terms of the original MS parameters used to refine the B-factors of X-ray structures. The correlated maps computed using this equation are well correlated with those of the method based on a mechanical model (the correlation coefficient is 0.75) for a non-homologous dataset comprising 100 structures. We have developed an approach to compute atomic cross-correlations directly from X-ray protein structure. Being in analytic form, it is fast and provides a feasible way to compute correlated motions in proteins in a high throughput way. In addition, avoiding sophisticated computational operations; it provides a quick, reliable way, especially for non-computational biologists, to obtain dynamics information directly from protein structure relevant to its function. (c) 2012 Elsevier B.V. All rights reserved. |
  | Date Added | 10/11/2013, 10:29:15 AM |
  | Modified | 10/11/2013, 10:29:15 AM |

  ### Tags:

  - Atomic cross-correlation
  - Correlated motion
  - TLS model
  - TLS parameter

  ### Notes:

  - Present computational method for studying protein dynamics.

    How SCOP is used:

    Calculate summary statistics on SCOP to count the number of single domain (80%) and multi-domain (20%) proteins.

    SCOP reference:

    Additionally, Eq. (2) uses the assumption that all atoms to be calculated are in the same domain (i.e., the same TLS group), however, there are many proteins whose structures contain more than one domain. According to our statistics for non-redundant protein structures in SCOP 1.75 (Murzin et al., 1995), 20% of the proteins are multi-domain structures, compared to 80% for single-domain structures. Therefore, it is necessary to evaluate the effect of treating a multi-domain structure as a single TLS group. Because the domain information for most protein structures in our dataset is not defined in SCOP 1.75, we ran a Protein Domain Parser (Alexandrov and Shindyalov, 2003), which is a domain prediction program, to calculate number of protein domains for each structure in our dataset. The results show that 65% of the proteins in our dataset are single-domain structures while 35% are multi-domain structures. We treat each protein chain in our dataset as a single TLS group regardless of whether there were multiple domains in the protein chain. The resulting average correlation coefficients for multi-domain structures and single-domain structures are 0.76 and 0.74, respectively. The student's t-test did not show significant difference between these two distributions (p > .05). This suggests that treating multiple domains as a single TLS group may not significantly affect our model's atomic cross-correlation computations, and implies that the dynamic properties of a TLS group adequately describe those of a protein domain.

  ### Attachments

  - 1-s2.0-S0378111912015661-main.pdf
- ## Deriving how far structural information is transmitted through parallel homodimeric coiled-coils: A correlation analysis of helical staggers

  |  |  |
  | --- | --- |
  | Type | Journal Article |
  | Author | Jerry H. Brown |
  | URL | http://onlinelibrary.wiley.com/doi/10.1002/prot.24218/full |
  | Publication | Proteins: Structure, Function, and Bioinformatics |
  | Date | 2013 |
  | Accessed | 9/23/2013, 10:17:26 AM |
  | Library Catalog | Google Scholar |
  | Short Title | Deriving how far structural information is transmitted through parallel homodimeric coiled-coils |
  | Date Added | 10/11/2013, 10:29:15 AM |
  | Modified | 10/11/2013, 10:29:15 AM |

  ### Notes:

  - Study of how distant features influence local structure on a collection of parallel homodimeric coiled-coils.

    How SCOP is used:

    Collected a dataset of proteins from the SCOP coiled-coil class, based on some geometric criteria.

    Reference to SCOP:

    In total, 75 crystal structures (of 40 different sequences, see also below) were collected as described previously 14 using SCOP24 [see Fig.2(b), Supporting Information Fig. S2].

  ### Attachments

  - Snapshot
- ## Describing sequence-ensemble relationships for intrinsically disordered proteins

  |  |  |
  | --- | --- |
  | Type | Journal Article |
  | Author | Albert H. Mao |
  | Author | Nicholas Lyle |
  | Author | Rohit V. Pappu |
  | Volume | 449 |
  | Pages | 307–318 |
  | Publication | Biochemical Journal |
  | Date | January 2013 |
  | DOI | 10.1042/BJ20121346 |
  | Abstract | Intrinsically disordered proteins participate in important protein-protein and protein-nucleic acid interactions and control cellular phenotypes through their prominence as dynamic organizers of transcriptional, post-transcriptional and signalling networks. These proteins challenge the tenets of the structure-function paradigm and their functional mechanisms remain a mystery given that they fail to fold autonomously into specific structures. Solving this mystery requires a first principles understanding of the quantitative relationships between information encoded in the sequences of disordered proteins and the ensemble of conformations they sample. Advances in quantifying sequence-ensemble relationships have been facilitated through a four-way synergy between bioinformatics, biophysical experiments, computer simulations and polymer physics theories. In the present review we evaluate these advances and the resultant insights that allow us to develop a concise quantitative framework for describing the sequence-ensemble relationships of intrinsically disordered proteins. |
  | Date Added | 3/7/2014, 12:08:00 PM |
  | Modified | 10/8/2014, 1:32:37 PM |

  ### Attachments

  - 4490307.pdf
  - Biochemical Journal (2013) 449, 307-318 - A. H. Mao, N. Lyle and R. V. Pappu - Sequence-ensemble relationships for intrinsically disordered proteins
- ## Describing some characters of serine proteinase using fractal analysis

  |  |  |
  | --- | --- |
  | Type | Journal Article |
  | Author | Xin Peng |
  | Author | Wei Qi |
  | Author | Rongxin Su |
  | Author | Zhimin He |
  | URL | http://www.sciencedirect.com/science/article/pii/S096007791200104X |
  | Volume | 45 |
  | Issue | 7 |
  | Pages | 1017–1023 |
  | Publication | Chaos, Solitons & Fractals |
  | Date | 2012 |
  | Accessed | 9/23/2013, 10:21:55 AM |
  | Library Catalog | Google Scholar |
  | Date Added | 10/11/2013, 10:29:15 AM |
  | Modified | 10/11/2013, 10:29:15 AM |

  ### Notes:

  - Calculated the fractal dimensions for 4 proteins in the serine proteinase family and its relationship to structure and function (the active site). This analysis also compared amongst the global enzymes and can be used to analysize protein evolution.

    How SCOP is used:

    SCOP class is referenced for the proteins that taken from the PDB.

    SCOP Reference:

    The proteins  
    selected from the Protein Data Bank [20] were used  
    X-ray diffraction as the structure elucidation method and  
    had a resolution less than 3.0 À. The class was assigned  
    according to the SCOP database [21] and the secondary  
    structure assignment was performed using the DSSP software  
    [22].

    The protein types are classified according to the SCOP 1.73 notation.

  ### Attachments

  - 1-s2.0-S096007791200104X-main.pdf
  - Snapshot
- ## Detecting evolutionary relationships across existing fold space, using sequence order-independent profile-profile alignments

  |  |  |
  | --- | --- |
  | Type | Journal Article |
  | Author | Lei Xie |
  | Author | Philip E Bourne |
  | Volume | 105 |
  | Issue | 14 |
  | Pages | 5441-5446 |
  | Publication | Proceedings of the National Academy of Sciences of the United States of America |
  | ISSN | 1091-6490 |
  | Date | Apr 8, 2008 |
  | Extra | PMID: 18385384 |
  | Journal Abbr | Proc. Natl. Acad. Sci. U.S.A. |
  | DOI | 10.1073/pnas.0704422105 |
  | Library Catalog | NCBI PubMed |
  | Language | eng |
  | Abstract | Here, a scalable, accurate, reliable, and robust protein functional site comparison algorithm is presented. The key components of the algorithm consist of a reduced representation of the protein structure and a sequence order-independent profile-profile alignment (SOIPPA). We show that SOIPPA is able to detect distant evolutionary relationships in cases where both a global sequence and structure relationship remains obscure. Results suggest evolutionary relationships across several previously evolutionary distinct protein structure superfamilies. SOIPPA, along with an increased coverage of protein fold space afforded by the structural genomics initiative, can be used to further test the notion that fold space is continuous rather than discrete. |
  | Date Added | 10/11/2013, 10:29:15 AM |
  | Modified | 10/11/2013, 10:29:15 AM |

  ### Tags:

  - Algorithms
  - Biological Evolution
  - Computational Biology
  - functional site
  - Models, Genetic
  - Protein Folding
  - Proteins
  - structure

  ### Notes:

  - Motivation: Would like to detect remote homologs where sequence and structure similarity is low.

    Approach: a reduced representation of the protein structure and using SOIPPA: sequence order-independent profile-profile alignments

    How SCOP is used:

    Used their own data set of 247 nonredundant protein chains known to bind an adenine containing ligand. Classified by SCOP superfamily.

    <5% of pairs (1,230 pairs) belong to the same SCOP superfamily and are the 'ground-truth' homologs.

    SCOP references:

    in Introduction:

    A central question is: What were the early protein folds and how did these folds change over long evolutionary time scales (4–7)? Comparative genomics studies and structural and phylogenetic analyses (8–10) have established that a subset of proteins, dominated by the structure classification of proteins (SCOP) (11) alpha/betaclass, were likely present in the last universal common ancestor (12, 13).

    In Results:

    To evaluate the performance of SOIPPA in detecting evolu- tionary relationships, we first see whether the algorithm can identify known sequence and structural homologous within the same SCOP superfamily. Among the 247-benchmark pairs, ⬚⬚5% of them (1,230 pairs) are from the same SCOP superfamily. If only these 1,230 pairs are considered as true positives, Fig. 2a illustrates the performance of PSI-BLAST (50), CE (51), and SOIPPA in detecting remote homologous that belong to the same SCOP superfamily. For a false-positive ratio of 0.05, the coverage of PSI-BLAST, CE, and SOIPPA is 0.55, 0.60, and 0.75, respectively. If SCOP superfamilies are taken as the gold stan- dard for defining remote evolutionary relationships, these results illustrate the well known fact that structure is more conserved than sequence. However, global structure comparison falls sig- nificantly short of SOIPPA, which takes both evolutionary profiles and structural constraints within the functional site into account. Consequently, it is more sensitive in detecting remote evolutional relationships than either PSI-BLAST or CE. The question then becomes, can SOIPPA detect functional similar- ities missed by SCOP, that is, relationships across superfamilies? In the 247-benchmark, there are 15,058 pairs aligned from different known SCOP superfamilies, and ⬚⬚30% of them are identified by SOIPPA with a false-positive ratio of 0.05 (Fig. 2b). Fig. 2b also shows that the sequence and structural similarity of these cross-superfamily pairs is not significant.

  ### Attachments

  - PNAS-2008-Xie-5441-6.pdf
  - PubMed entry
- ## Detecting mutually exclusive interactions in protein-protein interaction maps

  |  |  |
  | --- | --- |
  | Type | Journal Article |
  | Author | Carmen Sanchez Claros |
  | Author | Anna Tramontano |
  | URL | http://dx.plos.org/10.1371/journal.pone.0038765 |
  | Volume | 7 |
  | Issue | 6 |
  | Pages | e38765 |
  | Publication | PloS one |
  | Date | 2012 |
  | Accessed | 9/23/2013, 10:14:50 AM |
  | Library Catalog | Google Scholar |
  | Date Added | 2/20/2014, 12:24:01 PM |
  | Modified | 2/20/2014, 12:24:01 PM |

  ### Notes:

  - Present a method for protein-interaction prediction.  Also built a database and server for their method, which links to SCOP and other databases.

    How SCOP/CATH is used:

    Annotate interactome data in a database with links to SCOP and CATH.

    SCOP reference:

    The database can be searched both with an organism and a protein name (using a number of database identifiers, see Methods) thus allowing the user to select a sub-network of interest, in which case she or he is directed to a page containing general information about the proteins in the sub-network and links to several other databases (CATH [13], PDB [14], UniProt [15], iRefIndex [12], SCOP [16], Genbank [17] and Gene Ontology [18]).

  ### Attachments

  - [HTML] from plos.org
  - journal.pone.0038765.pdf
- ## Detection change points of triplet periodicity of gene

  |  |  |
  | --- | --- |
  | Type | Journal Article |
  | Author | Yulia M. Suvorova |
  | Author | Valentina M. Rudenko |
  | Author | Eugene V. Korotkov |
  | Volume | 491 |
  | Issue | 1 |
  | Pages | 58–64 |
  | Publication | Gene |
  | Date | January 2012 |
  | DOI | 10.1016/j.gene.2011.08.032 |
  | Abstract | The triplet periodicity (TP) is a distinguished property of protein coding sequences. There are complex genes with more than one TP type along their sequence. We say that these genes contain a triplet periodicity change point. The aim of the work is to find all genes that contain TP change point and attempt to compare the positions of change point in genes with known biological data. We have developed a mathematical method to identify triplet periodicity changes along a sequence. We have found 311221 genes with the TP change point in the KEGG/Genes database (version 48). It is about 8% from the total database volume (4013150). We showed that the repetitive sequences are not the only cause of such events. We suppose that the TP change point may indicate a fusion of genes or domains. We performed BLAST analysis to find potential ancestral genes for the parts of genes with TP change point. As a result we found that in 131323 cases sequences with TP change point have proper similarities for one or both parts. The relationship between TP change point and the fusion events in genes is discussed. The program realization of the method is available by request to authors. (C) 2011 Elsevier B.V. All rights reserved. |
  | Date Added | 3/7/2014, 12:08:00 PM |
  | Modified | 3/7/2014, 12:08:00 PM |
- ## Detection of peptide-binding sites on protein surfaces: The first step toward the modeling and targeting of peptide-mediated interactions

  |  |  |
  | --- | --- |
  | Type | Journal Article |
  | Author | Assaf Lavi |
  | Author | Chi Ho Ngan |
  | Author | Dana Movshovitz-Attias |
  | Author | Tanggis Bohnuud |
  | Author | Christine Yueh |
  | Author | Dmitri Beglov |
  | Author | Ora Schueler-Furman |
  | Author | Dima Kozakov |
  | Volume | 81 |
  | Issue | 12 |
  | Pages | 2096–2105 |
  | Publication | Proteins-structure Function and Bioinformatics |
  | Date | December 2013 |
  | DOI | 10.1002/prot.24422 |
  | Abstract | Peptide-mediated interactions, in which a short linear motif binds to a globular domain, play major roles in cellular regulation. An accurate structural model of this type of interaction is an excellent starting point for the characterization of the binding specificity of a given peptide-binding domain. A number of different protocols have recently been proposed for the accurate modeling of peptide-protein complex structures, given the structure of the protein receptor and the binding site on its surface. When no information about the peptide binding site(s) is a priori available, there is a need for new approaches to locate peptide-binding sites on the protein surface. While several approaches have been proposed for the general identification of ligand binding sites, peptides show very specific binding characteristics, and therefore, there is a need for robust and accurate approaches that are optimized for the prediction of peptide-binding sites. Here, we present PeptiMap, a protocol for the accurate mapping of peptide binding sites on protein structures. Our method is based on experimental evidence that peptide-binding sites also bind small organic molecules of various shapes and polarity. Using an adaptation of ab initio ligand binding site prediction based on fragment mapping (FTmap), we optimize a protocol that specifically takes into account peptide binding site characteristics. In a high-quality curated set of peptide-protein complex structures PeptiMap identifies for most the accurate site of peptide binding among the top ranked predictions. We anticipate that this protocol will significantly increase the number of accurate structural models of peptide-mediated interactions. Proteins 2013; 81:2096-2105. (c) 2013 Wiley Periodicals, Inc. |
  | Date Added | 3/7/2014, 12:08:00 PM |
  | Modified | 3/7/2014, 12:08:00 PM |
- ## Detection of secondary and supersecondary structures of proteins from cryo-electron microscopy

  |  |  |
  | --- | --- |
  | Type | Journal Article |
  | Author | Chandrajit Bajaj |
  | Author | Samrat Goswami |
  | Author | Qin Zhang |
  | Volume | 177 |
  | Issue | 2 |
  | Pages | 367-381 |
  | Publication | Journal of structural biology |
  | ISSN | 1095-8657 |
  | Date | Feb 2012 |
  | Extra | PMID: 22186625 |
  | Journal Abbr | J. Struct. Biol. |
  | DOI | 10.1016/j.jsb.2011.11.032 |
  | Library Catalog | NCBI PubMed |
  | Language | eng |
  | Abstract | Recent advances in three-dimensional electron microscopy (3D EM) have enabled the quantitative visualization of the structural building blocks of proteins at improved resolutions. We provide algorithms to detect the secondary structures (α-helices and β-sheets) from proteins for which the volumetric maps are reconstructed at 6-10Å resolution. Additionally, we show that when the resolution is coarser than 10Å, some of the supersecondary structures can be detected from 3D EM maps. For both these algorithms, we employ tools from computational geometry and differential topology, specifically the computation of stable/unstable manifolds of certain critical points of the distance function induced by the molecular surface. Our results connect mathematically well-defined constructions with bio-chemically induced structures observed in proteins. |
  | Date Added | 10/11/2013, 10:29:15 AM |
  | Modified | 3/7/2014, 1:07:14 PM |

  ### Tags:

  - Algorithms
  - Amino Acid Motifs
  - Animals
  - Capsid Proteins
  - Chaperonin 60
  - Critical points
  - Cryoelectron Microscopy
  - Delaunay and Voronoi objects
  - Deoxyribonuclease I
  - Electron microscopy
  - Group II Chaperonins
  - Humans
  - Models, Molecular
  - Proteins
  - Protein structures detection
  - Protein Structure, Tertiary
  - Receptor, Insulin
  - Stable/unstable manifolds
  - Surface Properties
  - Swine

  ### Notes:

  - Present a new algorithm to detect protein secondary structures from low-resolution electron microscopy maps.

    How SCOP is used:

    Annotate data set of 4 proteins used to calbrate their method with SCOP class.

    How CATH is used:

    Not using CATH data.

    SCOP Reference

    Information of various motifs of atomistic resolution protein models, is also widely available from web-based databases including SCOP (Murzin et al., 1995), CATH (Orengo et al., 1997) and DALI/FSSP (Holm and Sander, 1997).

    ...

    The calibration process is essential before we apply them to 3D EM maps of unknown atomic descriptions. We calibrate against a dataset which is similar to the one used in (Jiang et al., 2001). The results are shown in Figure 8 and statistics is given in Table 1. The model data includes bacteriorhodopsin (1C3W, all alpha), Cytochrome C’ (1BBH, all alpha), triose phosphate isomerase (1TIM, α/β), tyrosine kinase domain from the insulin receptor (1IRK, α + β) and Bluetongue Virus outer shell coat protein (1BVP, a β upper domain and an α lower domain), where the classification is based on the widely accepted Structural Classification Of Proteins (SCOP) (Murzin et al., 1995)

     CATH reference:

    The most related work is SPI-EM (Velázquez-Muriel et al., 2005) which applied a probabilistic approach to determine the homologous superfamily defined by CATH for 3D EM maps at a resolution of 8Å till 12Å. Folds or domains detecting methods are also relevant since there is a blurred distinction between super-secondary structure (motifs) and tertiary structure (folds or domains)(cf. http://swissmodel.expasy.org/course/text/chapter4.htm).

  ### Attachments

  - nihms345060.pdf
  - PubMed entry
- ## Developing a high-quality scoring function for membrane protein structures based on specific inter-residue interactions

  |  |  |
  | --- | --- |
  | Type | Journal Article |
  | Author | Andrew J. Heim |
  | Author | Zhijun Li |
  | Volume | 26 |
  | Issue | 3 |
  | Pages | 301–309 |
  | Publication | Journal of Computer-aided Molecular Design |
  | Date | March 2012 |
  | DOI | 10.1007/s10822-012-9556-z |
  | Abstract | Membrane proteins are of particular biological and pharmaceutical importance, and computational modeling and structure prediction approaches play an important role in studies of membrane proteins. Developing an accurate model quality assessment program is of significance to the structure prediction of membrane proteins. Few such programs are proposed that can be applied to a broad range of membrane protein classes and perform with high accuracy. We developed a new model scoring function Interaction-based Quality assessment (IQ), based on the analysis of four types of inter-residue interactions within the transmembrane domains of helical membrane proteins. This function was tested using three high-quality model sets: all 206 models of GPCR Dock 2008, all 284 models of GPCR Dock 2010, and all 92 helical membrane protein models of the HOMEP set. For all three sets, the scoring function can select the native structures among all of the models with the success rates of 93, 85, and 100% respectively. For comparison, these three model sets were also adopted for a recently published model assessment program for membrane protein structures, ProQM, which gave the success rates of 85, 79, and 92% separately. These results suggested that IQ outperforms ProQM when only the transmembrane regions of the models are considered. This scoring function should be useful for the computational modeling of membrane proteins. |
  | Date Added | 3/7/2014, 12:08:00 PM |
  | Modified | 3/7/2014, 12:08:00 PM |
- ## Dimensionality reduction in computational demarcation of protein tertiary structures

  |  |  |
  | --- | --- |
  | Type | Journal Article |
  | Author | Rajani R. Joshi |
  | Author | Priyabrata R. Panigrahi |
  | Author | Reshma N. Patil |
  | URL | http://link.springer.com/article/10.1007/s00894-011-1223-0 |
  | Volume | 18 |
  | Issue | 6 |
  | Pages | 2741–2754 |
  | Publication | Journal of molecular modeling |
  | Date | 2012 |
  | Accessed | 9/23/2013, 10:24:02 AM |
  | Library Catalog | Google Scholar |
  | Date Added | 10/11/2013, 10:29:15 AM |
  | Modified | 3/7/2014, 12:14:35 PM |

  ### Tags:

  - Logistic regression
  - principal component analysis
  - Protein structural classes
  - Quantitative features of tertiary folds
  - SCOP database

  ### Notes:

  - Use machine learning (logistic regression) to predict SCOP class and fold.

    How SCOP is used:

    Validate against SCOP class and fold classification.  Curate a non-redundant dataset of domains by sampling from 4 classes in SCOP.

    How CATH is used:

    Not using CATH data.

    SCOP reference:

    Abstract

    Predictive classification of major structural families and fold types of proteins is investigated deploying logistic regression. Only five to seven dimensional quantitative feature vector representations of tertiary structures are found adequate. Results for benchmark sample of non-homologous proteins from SCOP database are presented. Importance of this work as compared to homology modeling and best- known quantitative approaches is highlighted.

    ...

    Data set for common structural fold within a class

    Considering that SCOP database does finer structural classifications at different fold levels and is also the basis/ yardstick of test of the work reported by Chi et al. [13], we have considered structural families and fold types of protein (domains) as identified in this database. For exhaustive search we randomly selected maximum possible number of high-resolution structures of proteins the structural domains of which are authenticated in SCOP such that a comparable number of non-redundant observations are available from each of the four classes of interest and such that samples from each class will contain different possible sizes and orientation of the structural domain it represents.

  ### Attachments

  - art%3A10.1007%2Fs00894-011-1223-0.pdf
- ## Discovering putative prion sequences in complete proteomes using probabilistic representations of Q/N-rich domains

  |  |  |
  | --- | --- |
  | Type | Journal Article |
  | Author | Vladimir Espinosa Angarica |
  | Author | Salvador Ventura |
  | Author | Javier Sancho |
  | Volume | 14 |
  | Publication | BMC GENOMICS |
  | ISSN | 1471-2164 |
  | Date | MAY 10 2013 |
  | DOI | 10.1186/1471-2164-14-316 |
  | Language | English |
  | Abstract | Background: Prion proteins conform a special class among amyloids due to their ability to transmit aggregative folds. Prions are known to act as infectious agents in neurodegenerative diseases in animals, or as key elements in transcription and translation processes in yeast. It has been suggested that prions contain specific sequential domains with distinctive amino acid composition and physicochemical properties that allow them to control the switch between soluble and beta-sheet aggregated states. Those prion-forming domains are low complexity segments enriched in glutamine/asparagine and depleted in charged residues and prolines. Different predictive methods have been developed to discover novel prions by either assessing the compositional bias of these stretches or estimating the propensity of protein sequences to form amyloid aggregates. However, the available algorithms hitherto lack a thorough statistical calibration against large sequence databases, which makes them unable to accurately predict prions without retrieving a large number of false positives. Results: Here we present a computational strategy to predict putative prion-forming proteins in complete proteomes using probabilistic representations of prionogenic glutamine/asparagine rich regions. After benchmarking our predictive model against large sets of non-prionic sequences, we were able to filter out known prions with high precision and accuracy, generating prediction sets with few false positives. The algorithm was used to scan all the proteomes annotated in public databases for the presence of putative prion proteins. We analyzed the presence of putative prion proteins in all taxa, from viruses and archaea to plants and higher eukaryotes, and found that most organisms encode evolutionarily unrelated proteins with susceptibility to behave as prions. Conclusions: To our knowledge, this is the first wide-ranging study aiming to predict prion domains in complete proteomes. Approaches of this kind could be of great importance to identify potential targets for further experimental testing and to try to reach a deeper understanding of prions' functional and regulatory mechanisms. |
  | Date Added | 10/25/2013, 4:17:08 PM |
  | Modified | 10/25/2013, 4:17:08 PM |

  ### Tags:

  - Amyloid fibrils
  - Prion domain
  - Prion prediction
  - Protein aggregation

  ### Notes:

  - Present method to predict prion-forming proteins in a complete proteome.

    How SCOP is used:

    Evaluated method on an ASTRAL representative set in order to test scalability of method on a data set with a "high number of negative instances".

    SCOP reference:

    We also defined three additional evaluation datasets, comprising the Uniprot/Swissprot database [40] (release from February 2012), a culled list of proteins with solved tridimensional structure annotated in SCOP (version 1.75) obtained from the ASTRAL compendium [91] (in- cluding proteins with less than 95% sequence similarity) and all the intrinsically disordered proteins annotated in Disprot [42] (version 5.7). In the case of the Uniprot/ Swissprot dataset we randomly generated a million sets that were used in the benchmarking, while for the other two databases we used all the protein sequences anno- tated. In all cases the known prions were removed from the negative datasets. These three test sets were used to measure the ability of the model to handle sequence datasets with a high number of negative instances, as it is the case of the scanning of complete proteome databases.

  ### Attachments

  - 1471-2164-14-316.pdf
- ## Discovery of a new human polyomavirus associated with trichodysplasia spinulosa in an immunocompromized patient

  |  |  |
  | --- | --- |
  | Type | Journal Article |
  | Author | Els van der Meijden |
  | Author | René W A Janssens |
  | Author | Chris Lauber |
  | Author | Jan Nico Bouwes Bavinck |
  | Author | Alexander E Gorbalenya |
  | Author | Mariet C W Feltkamp |
  | Volume | 6 |
  | Issue | 7 |
  | Pages | e1001024 |
  | Publication | PLoS pathogens |
  | ISSN | 1553-7374 |
  | Date | 2010 |
  | Extra | PMID: 20686659 |
  | Journal Abbr | PLoS Pathog. |
  | DOI | 10.1371/journal.ppat.1001024 |
  | Library Catalog | NCBI PubMed |
  | Language | eng |
  | Abstract | The Polyomaviridae constitute a family of small DNA viruses infecting a variety of hosts. In humans, polyomaviruses can cause infections of the central nervous system, urinary tract, skin, and possibly the respiratory tract. Here we report the identification of a new human polyomavirus in plucked facial spines of a heart transplant patient with trichodysplasia spinulosa, a rare skin disease exclusively seen in immunocompromized patients. The trichodysplasia spinulosa-associated polyomavirus (TSV) genome was amplified through rolling-circle amplification and consists of a 5232-nucleotide circular DNA organized similarly to known polyomaviruses. Two putative "early" (small and large T antigen) and three putative "late" (VP1, VP2, VP3) genes were identified. The TSV large T antigen contains several domains (e.g. J-domain) and motifs (e.g. HPDKGG, pRb family-binding, zinc finger) described for other polyomaviruses and potentially involved in cellular transformation. Phylogenetic analysis revealed a close relationship of TSV with the Bornean orangutan polyomavirus and, more distantly, the Merkel cell polyomavirus that is found integrated in Merkel cell carcinomas of the skin. The presence of TSV in the affected patient's skin was confirmed by newly designed quantitative TSV-specific PCR, indicative of a viral load of 10(5) copies per cell. After topical cidofovir treatment, the lesions largely resolved coinciding with a reduction in TSV load. PCR screening demonstrated a 4% prevalence of TSV in an unrelated group of immunosuppressed transplant recipients without apparent disease. In conclusion, a new human polyomavirus was discovered and identified as the possible cause of trichodysplasia spinulosa in immunocompromized patients. The presence of TSV also in clinically unaffected individuals suggests frequent virus transmission causing subclinical, probably latent infections. Further studies have to reveal the impact of TSV infection in relation to other populations and diseases. |
  | Date Added | 10/11/2013, 10:29:15 AM |
  | Modified | 10/11/2013, 10:29:15 AM |

  ### Tags:

  - Carcinoma, Merkel Cell
  - DNA, Circular
  - Genes, Viral
  - Genome, Viral
  - Humans
  - Immunocompromised Host
  - Phylogeny
  - Polyomavirus
  - Skin Diseases
  - Viral Load

  ### Notes:

  - Clinical research paper.  Report on a new virus found in a human patient.

    How SCOP is used:

    Sequenced the virus proteins and did domain searches using HHsearch on the SCOP database.

    SCOP reference:

    Domain searches within the TSV large and small T antigen sequences were performed against the domain profile database SCOP [47] using the HHsearch software [48]. Hits against all 3 domains were strongly significant (E-values ,E-12).

  ### Attachments

  - journal.ppat.1001024.pdf
  - PubMed entry
- ## Discriminative modelling of context-specific amino acid substitution probabilities

  |  |  |
  | --- | --- |
  | Type | Journal Article |
  | Author | Christof Angermüller |
  | Author | Andreas Biegert |
  | Author | Johannes Söding |
  | URL | http://bioinformatics.oxfordjournals.org/content/28/24/3240.short |
  | Volume | 28 |
  | Issue | 24 |
  | Pages | 3240–3247 |
  | Publication | Bioinformatics |
  | Date | 2012 |
  | Accessed | 9/20/2013, 1:13:08 PM |
  | Library Catalog | Google Scholar |
  | Date Added | 10/11/2013, 10:29:15 AM |
  | Modified | 10/11/2013, 10:29:15 AM |

  ### Tags:

  - likely ASTRAL
  - likely ASTRAL sequences
  - likely ASTRAL subsets

  ### Notes:

  - Present a new machine learning method for sequence alignment that is "context-specific".  The context-specific substitution probability is the probability of observing a particular amino acid at some position, given the sequence window around it.

    How SCOP data is used:

    Use two data sets derived from ASTRAL sequence data for training and benchmarking of their sequence alignment algorithm. Create their own representative sets (20% through 80%) and use to evaluate sensitivity of methods. Validated method on fold and superfamily.

    SCOP reference:

    3 RESULTS

    3.1 Data sets and parameter optimization

    The SCOP database (Murzin et al., 1995) provides a hierarchical clustering of protein domains with known structures and is the de facto standard for evaluating sequence search tools. We filtered the SCOP database with a maximum pairwise sequence similarity of 20% (SCOP20) and also 30% (SCOP30), 40% (SCOP40), 60% (SCOP60) and 80% (SCOP80). We randomly assigned every fifth fold to the optimization set (1329 sequences, 215 folds in SCOP20) and all remaining folds to the test set (5287 sequences, 862 folds in SCOP20). This ensures that the optimization set does not share homologous sequences with the test set. We performed an all- against-all comparison and defined members belonging to the same fold as true positives (TPs) and those of different folds as false positives (FPs). Pairs with both proteins within the four- to eight- bladed β-propellers (SCOP fold IDs b.66 - b.70) were treated as unknown, and the same for Rossman-like folds (c.2 - c.5, c.30, c.66, c.78, c.79, c.111) and alpha-helical and 4Fe-4S ferredoxins (a.1.2, d.58.1).

  ### Attachments

  - [PDF] from researchgate.net
  - Snapshot
- ## Dissimilar sweet proteins from plants: Oddities or normal components?

  |  |  |
  | --- | --- |
  | Type | Journal Article |
  | Author | Delia Picone |
  | Author | Piero Andrea Temussi |
  | URL | http://www.sciencedirect.com/science/article/pii/S0168945212001367 |
  | Publication | Plant Science |
  | Date | 2012 |
  | Accessed | 9/23/2013, 10:14:18 AM |
  | Library Catalog | Google Scholar |
  | Short Title | Dissimilar sweet proteins from plants |
  | Date Added | 2/20/2014, 12:24:01 PM |
  | Modified | 2/20/2014, 12:24:01 PM |

  ### Tags:

  - Interesting

  ### Notes:

  - Review describes research on "nature, structure and mechanism of action of the best known sweet tasting proteins"

    How SCOP is used:

    Look up classification structural class and superfamily classification of set of "sweet proteins of plant origin".

    SCOP reference:

    See table 1 for summary of data set with SCOP classification.

    ...

    Mabinlin II is characterized by two chains linked by disulfide bridges [10]. The structure of mabinlin II has been solved by X- ray diffraction [30]. The fold (Fig. 1d) is typical of an all alpha protein, according to SCOP (Structural Classification Of Proteins) (http://scop.mrc-lmb.cam.ac.uk) [23]. The

    ...

    According to SCOP, brazzein belongs to the scorpion toxin-like superfamily [23].

  ### Attachments

  - [PDF] from researchgate.net
- ## Distance dependency and minimum amino acid alphabets for decoy scoring potentials

  |  |  |
  | --- | --- |
  | Type | Journal Article |
  | Author | Susanne Pape |
  | Author | Franziska Hoffgaard |
  | Author | Mirjam Duer |
  | Author | Kay Hamacher |
  | Volume | 34 |
  | Issue | 1 |
  | Pages | 10-20 |
  | Publication | Journal of Computational Chemistry |
  | ISSN | 0192-8651 |
  | Date | JAN 5 2013 |
  | Extra | WOS:000311440900003 |
  | DOI | 10.1002/jcc.23099 |
  | Abstract | The validity and accuracy of a proposed tertiary structure of a protein can be assessed in several ways. Scoring such a structure by a knowledge-based potential is a well-known approach in molecular biophysics, an important task in structure prediction and refinement, and a key step in several experiments on protein structures. Although several parameterizations for such models have been derived over the course of time, improvements in accuracy by explicitly using continuous distance information have not been suggested yet. We close this methodological gap by formulating the parameterization of a protein structure model as a linear program. Optimization of the parameters was performed using amino acid distances calculated for the residues in topology rich 2830 protein structures. We show the capability of our derived model to discriminate between native structures and decoys for a diverse set of proteins. In addition, we discuss the effect of reduced amino acid alphabets on the model. In contrast to studies focusing on binary contact schemes (without considering distance dependencies and proposing five symbols as optimal alphabet size), we find an accurate protein alphabet size to contain at least five symbols, preferably more, to assure a satisfactory fold recognition capability. (C) 2012 Wiley Periodicals, Inc. |
  | Date Added | 2/20/2014, 12:24:01 PM |
  | Modified | 2/20/2014, 12:24:01 PM |

  ### Notes:

  - Present protein structure scoring method for decoy detection.

    How SCOP is used:

    Evaluate method on structurally diverse set of proteins.  Collect one PDB entrie for each SCOP 'class' resulting in 2830 protein structures.

    SCOP reference:

    Data sets

    Structural Data. We prepared a structurally diverse set of pro- teins based on the Structural Classification of Proteins (SCOP) database.[31] For each SCOP class, one representative Protein Data Bank (PDB) entry with only one chain and a sequence length between 20 and 1000 amino acids was chosen. PDB files with chain breaks and unusual amino acids were elimi- nated—resulting in 2830 protein structures.[32]

  ### Attachments

  - 23099\_ftp.pdf
- ## Divergence and Convergence in Enzyme Evolution

  |  |  |
  | --- | --- |
  | Type | Journal Article |
  | Author | Michael Y. Galperin |
  | Author | Eugene V. Koonin |
  | URL | http://www.jbc.org/content/287/1/21 |
  | Volume | 287 |
  | Issue | 1 |
  | Pages | 21-28 |
  | Publication | Journal of Biological Chemistry |
  | ISSN | 0021-9258, 1083-351X |
  | Date | 01/02/2012 |
  | Extra | PMID: 22069324 |
  | Journal Abbr | J. Biol. Chem. |
  | DOI | 10.1074/jbc.R111.241976 |
  | Accessed | 9/18/2013, 4:19:42 PM |
  | Library Catalog | www.jbc.org |
  | Language | en |
  | Abstract | Comparative analysis of the sequences of enzymes encoded in a variety of prokaryotic and eukaryotic genomes reveals convergence and divergence at several levels. Functional convergence can be inferred when structurally distinct and hence non-homologous enzymes show the ability to catalyze the same biochemical reaction. In contrast, as a result of functional diversification, many structurally similar enzyme molecules act on substantially distinct substrates and catalyze diverse biochemical reactions. Here, we present updates on the ATP-grasp, alkaline phosphatase, cupin, HD hydrolase, and N-terminal nucleophile (Ntn) hydrolase enzyme superfamilies and discuss the patterns of sequence and structural conservation and diversity within these superfamilies. Typically, enzymes within a superfamily possess common sequence motifs and key active site residues, as well as (predicted) reaction mechanisms. These observations suggest that the strained conformation (the entatic state) of the active site, which is responsible for the substrate binding and formation of the transition complex, tends to be conserved within enzyme superfamilies. The subsequent fate of the transition complex is not necessarily conserved and depends on the details of the structures of the enzyme and the substrate. This variability of reaction outcomes limits the ability of sequence analysis to predict the exact enzymatic activities of newly sequenced gene products. Nevertheless, sequence-based (super)family assignments and generic functional predictions, even if imprecise, provide valuable leads for experimental studies and remain the best approach to the functional annotation of uncharacterized proteins from new genomes. |
  | Date Added | 2/20/2014, 12:24:01 PM |
  | Modified | 3/7/2014, 12:14:56 PM |

  ### Tags:

  - Catalytic Domain
  - Conserved Sequence
  - Convergence
  - Divergence
  - Enzyme Catalysis
  - Enzyme Mechanisms
  - Enzymes
  - Enzyme Structure
  - Evolution
  - Evolution, Molecular
  - Humans
  - Phosphodiesterases
  - Proteins

  ### Notes:

  - Study of function divergence (similar structures which interact with substantially different substrates) and functional convergence (non-homologous, structurally distinct enzymes that catalyze the same biochemical reactions) in four superfamilie:  the ATP-grasp, alkaline phopatase, cupin, HD hydrolase, and N-terminal nucleophile hydrolase enzyme superfamilies.

    How SCOP is used:

    1. Mention SCOP in the context of "compatibility" at the SF level between CATH, DALI, and Pfam clans.

    2. Also mention SCOP to point out an inconsistency in SF assignment of the Ntn hydrolase-like fold.

    How CATH is used:

    Not using CATH data.

    SCOP/CATH reference:

    The current classifications of protein structural (super)families, implemented in the popular SCOP, CATH, and Dali databases, are generally compatible with each other despite the differences between the underlying methodologies (11–13). Furthermore, these superfamilies often correspond to sequence-based domain families (or clans) in the Pfam database (14) and contain conserved sequence motifs that are represented in such databases as InterPro (15).

    ...

    The Ntn hydrolase-like fold is also present in the archaeal IMP cyclohydrolase PurO, which catalyzes the final step of purine biosynthesis (55). This enzyme retains all the structural features of the Ntn hydrolase superfamily but is not proteolytically processed, lacks a nucleophilic residue at the N terminus, and does not function as an amidohydrolase. Accordingly, the SCOP database assigns it to a separate superfamily (11). This enzyme is found only in a small set of methano- and haloar- chaea and represents an unusual variant of extreme divergence within the common structural core.

  ### Attachments

  - Full Text PDF
- ## Domain enhanced lookup time accelerated BLAST

  |  |  |
  | --- | --- |
  | Type | Journal Article |
  | Author | Grzegorz M Boratyn |
  | Author | Alejandro A Schäffer |
  | Author | Richa Agarwala |
  | Author | Stephen F Altschul |
  | Author | David J Lipman |
  | Author | Thomas L Madden |
  | Volume | 7 |
  | Pages | 12 |
  | Publication | Biology direct |
  | ISSN | 1745-6150 |
  | Date | 2012 |
  | Extra | PMID: 22510480 |
  | Journal Abbr | Biol. Direct |
  | DOI | 10.1186/1745-6150-7-12 |
  | Library Catalog | NCBI PubMed |
  | Language | eng |
  | Abstract | BACKGROUND: BLAST is a commonly-used software package for comparing a query sequence to a database of known sequences; in this study, we focus on protein sequences. Position-specific-iterated BLAST (PSI-BLAST) iteratively searches a protein sequence database, using the matches in round i to construct a position-specific score matrix (PSSM) for searching the database in round i + 1. Biegert and Söding developed Context-sensitive BLAST (CS-BLAST), which combines information from searching the sequence database with information derived from a library of short protein profiles to achieve better homology detection than PSI-BLAST, which builds its PSSMs from scratch. RESULTS: We describe a new method, called domain enhanced lookup time accelerated BLAST (DELTA-BLAST), which searches a database of pre-constructed PSSMs before searching a protein-sequence database, to yield better homology detection. For its PSSMs, DELTA-BLAST employs a subset of NCBI's Conserved Domain Database (CDD). On a test set derived from ASTRAL, with one round of searching, DELTA-BLAST achieves a ROC5000 of 0.270 vs. 0.116 for CS-BLAST. The performance advantage diminishes in iterated searches, but DELTA-BLAST continues to achieve better ROC scores than CS-BLAST. CONCLUSIONS: DELTA-BLAST is a useful program for the detection of remote protein homologs. It is available under the "Protein BLAST" link at http://blast.ncbi.nlm.nih.gov. |
  | Date Added | 10/11/2013, 10:29:15 AM |
  | Modified | 3/7/2014, 12:15:22 PM |

  ### Tags:

  - Algorithms
  - Computational Biology
  - Databases, Protein
  - Internet
  - Protein Structure, Tertiary
  - Reproducibility of Results
  - ROC Curve
  - Search Engine
  - Sensitivity and Specificity
  - Sequence Alignment
  - Sequence Analysis, Protein
  - Sequence Homology, Amino Acid
  - Software
  - Time Factors

  ### Notes:

  - Introduce a new variant of BLAST, called DELTA-BLAST (domain enhanced lookup time accelerated BLAST).  DELTA-BLAST employs a subset of NCBI's conserved domain database.

    How SCOP is used:

    The sequence alignment method is evaluated on ASTRAL domain sequences.  Compared across SCOP classes.

    Alignments were compared with reference structure alignments.

    SCOP reference:

    Results

    This section compares the performance of BLASTP, CS- BLAST, PSI-BLAST, and DELTA-BLAST. We assessed thehomology-detection effectiveness of these methods using search results for the ASTRAL Compendium for Sequence and Structure Analysis [30] and the Structural Classification of Proteins (SCOP) [31] databases. A database of 10,569 sequences was searched using a set of 4,852 queries. To assess not only search sensitivity but also the quality of the alignments produced, we compared program- generated alignments of 10,006 pairs of 3D domains from the superfamily subset of the SABmark set [32] to these pairs’ reference alignments. Finally, to further assess algorithm sensitivity, we analyzed the numbers of true positive results yielded by the search methods, articulated further by their CDD annotation.

    ...

    Alignment methods may show different behaviors for different protein types. Therefore, we divided the test set by SCOP class and computed ROCn score for the pooled search results for each class, with n equal to the number of queries ineach subset. DELTA-BLAST yields the lar- gest ROCn scores for all SCOP classes, except for small proteins (Table 3).

  ### Attachments

  - [PDF] from biomedcentral.com
  - PubMed entry
- ## DomHR: Accurately Identifying Domain Boundaries in Proteins Using a Hinge Region Strategy

  |  |  |
  | --- | --- |
  | Type | Journal Article |
  | Author | Xiao-yan Zhang |
  | Author | Long-jian Lu |
  | Author | Qi Song |
  | Author | Qian-qian Yang |
  | Author | Da-peng Li |
  | Author | Jiang-ming Sun |
  | Author | Tong-hua Li |
  | Author | Pei-sheng Cong |
  | Volume | 8 |
  | Issue | 4 |
  | Pages | e60559 |
  | Publication | Plos One |
  | ISSN | 1932-6203 |
  | Date | APR 11 2013 |
  | Extra | WOS:000317383200012 |
  | DOI | 10.1371/journal.pone.0060559 |
  | Abstract | Motivation: The precise prediction of protein domains, which are the structural, functional and evolutionary units of proteins, has been a research focus in recent years. Although many methods have been presented for predicting protein domains and boundaries, the accuracy of predictions could be improved. Results: In this study we present a novel approach, DomHR, which is an accurate predictor of protein domain boundaries based on a creative hinge region strategy. A hinge region was defined as a segment of amino acids that covers part of a domain region and a boundary region. We developed a strategy to construct profiles of domain-hinge-boundary (DHB) features generated by sequence-domain/hinge/boundary alignment against a database of known domain structures. The DHB features had three elements: normalized domain, hinge, and boundary probabilities. The DHB features were used as input to identify domain boundaries in a sequence. DomHR used a nonredundant dataset as the training set, the DHB and predicted shape string as features, and a conditional random field as the classification algorithm. In predicted hinge regions, a residue was determined to be a domain or a boundary according to a decision threshold. After decision thresholds were optimized, DomHR was evaluated by cross-validation, large-scale prediction, independent test and CASP (Critical Assessment of Techniques for Protein Structure Prediction) tests. All results confirmed that DomHR outperformed other well-established, publicly available domain boundary predictors for prediction accuracy. Availability: The DomHR is available at http://cal.tongji.edu.cn/domain/. |
  | Date Added | 2/13/2014, 4:13:17 PM |
  | Modified | 3/7/2014, 12:08:32 PM |
- ## DOMMINO: a database of macromolecular interactions

  |  |  |
  | --- | --- |
  | Type | Journal Article |
  | Author | Xingyan Kuang |
  | Author | Jing Ginger Han |
  | Author | Nan Zhao |
  | Author | Bin Pang |
  | Author | Chi-Ren Shyu |
  | Author | Dmitry Korkin |
  | Volume | 40 |
  | Issue | D1 |
  | Pages | D501-D506 |
  | Publication | Nucleic Acids Research |
  | ISSN | 0305-1048 |
  | Date | January 2012 |
  | DOI | 10.1093/nar/gkr1128 |
  | Language | English |
  | Abstract | With the growing number of experimentally resolved structures of macromolecular complexes, it becomes clear that the interactions that involve protein structures are mediated not only by the protein domains, but also by various non-structured regions, such as interdomain linkers, or terminal sequences. Here, we present DOMMINO (http://dommino.org), a comprehensive database of macromolecular interactions that includes the interactions between protein domains, interdomain linkers, N- and C-terminal regions and protein peptides. The database complements SCOP domain annotations with domain predictions by SUPERFAMILY and is automatically updated every week. The database interface is designed to provide the user with a three-stage pipeline to study macromolecular interactions: (i) a flexible search that can include a PDB ID, type of interaction, SCOP family of interacting proteins, organism name, interaction keyword and a minimal threshold on the number of contact pairs; (ii) visualization of subunit interaction network, where the user can investigate the types of interactions within a macromolecular assembly; and (iii) visualization of an interface structure between any pair of the interacting subunits, where the user can highlight several different types of residues within the interfaces as well as study the structure of the corresponding binary complex of subunits. |
  | Date Added | 10/25/2013, 4:23:37 PM |
  | Modified | 3/7/2014, 12:14:28 PM |

  ### Tags:

  - ASTRAL
  - Cite ASTRAL

  ### Notes:

  - DOMMINO is a comprehensive database of macromolecular interactions.  Includes the interactions between protein domains, interdomain linkers, N- and C-terminal regions and protein peptides.  
      
    How SCOP is used:  
      
    SCOP is used for domain annotation.  When available, SCOP domains are provided, otherwise, SUPERFAMILY is used.

    It's interesting that they describe protocol for dealing with non-annotated regions: linkers and C- and N- terminal regions. Cite Astral when discussing protocol for determining whether a chain is a peptide (<20 residues).

    Although they cite Astral, seems that they were not aware that Astral has updated mappings of SCOP domain definitions to residue identifiers: "Out of 110 800 SCOP protein domain definitions extracted from file dir.cla.scop.txt, a slightly reduced set of 109 942 definitions is employed for the first group of PDB struc- tures, because some SCOP domains cannot be located in the coordinate records of the PDB files from the current PDB release."

    How CATH is used:

    Not using CATH data.

    SCOP references:  
      
    Under abstract:  
      
    The database complements SCOP domain annotations with domain predictions by SUPERFAMILY and is automatically updated every week.  
      
    The database interface is designed to provide the user with a three-stage pipeline to study macro- molecular interactions: (i) a flexible search that can include a PDB ID, type of interaction, SCOP family of interacting proteins, organism name, interaction keyword and a minimal threshold on the number of contact pairs...  
      
    Under Introduction:  
      
    The database has an expanded coverage of structural domains by integrating the manual annotation of protein domains participating in the interactions using the latest version of SCOP (13) with the automated annotation using SUPERFAMILY.  
      
    Under Methods:  
      
    For domain assignment, the most recent release (June 2009) of manually curated SCOP database is used that includes the manual annotation for 38221 PDB entries. The SCOP domain definitions are extracted from file dir.cla.scop.txt. During the domain assignment, each PDB structure is assigned to one of two groups as follows. If a PDB structure has at least one assigned SCOP protein domain, according to the SCOP definition file, the structure is assigned to the first group, otherwise it is assigned to the second group of macromolecular complexes for which the constituting domains are later predicted using SUPERFAMILY software (30) (‘Domain annotation’ section).  
      
    Out of 110,800 SCOP protein domain definitions extracted from file dir.cla.scop.txt, a slightly reduced set of 109,942 definitions is employed for the first group of PDB structures, because some SCOP domains cannot be located in the coordinate records of the PDB files from the current PDB release. The definitions are stored as a new parsable file filtered.cla.scop.txt, in which the same data format as in dir.cla.scop.txt is used.  
    To assign protein domains for the PDB structures from the second group, for which no SCOP annotation is available, SUPERFAMILY software is used (30).  
      
    To assign protein domains for the PDB structures from the second group, for which no SCOP annotation is avail- able, SUPERFAMILY software is used (30). The software is designed to accurately predict the SCOP domains based on a sequence of each protein chain. It employs a collec- tion of hidden Markov models (HMMs) each correspond- ing to a structural protein domain at the SCOP family level. The prediction is done by scanning a protein sequence against the HMMs and is accepted when E-value <=0.01. In the current version of DOMMINO, we do not predict domains spanning multiple chains in a PDB structure. When two predicted domains overlap, we evenly distribute the overlapping region between the two pre- dicted domains. As a result, 101389 domains were pre- dicted for the PDB files from the second group, and their coordinates were extracted. The information on pre- dicted domains, including the corresponding regions and the SCOP classification, is summarized in a parsable file pre.cla.scop.txt.  
      
    Locating linkers, C-terminal and N-terminal regions  
      
    A predicted SCOP domain can be either a whole protein chain or its fragment. A domain annotated by SCOP can also consist of more than one fragment of a protein chain. Each protein fragment that does not belong to any protein domain is then annotated as a linker, C- or N-terminus, depending on whether it is surrounded by two domain fragments or there is just one domain fragment located to the right or to the left of it, corres- pondingly. For each defined region, its coordinates are also extracted.  
      
    Determining peptides and undefined chains  
      
    There are chains in PDB files that cannot be annotated by assigning domain using either SCOP definitions or predic- tion by SUPERFAMILY. Based on how long such protein chains, they are classified as either peptides or undefined chains. Specifically, we use a 20-residue thresh- old to determine a peptide (if the number of residues is <20) and undefined chain (otherwise). The same threshold has been used before in ASTRAL, a similar domain definition protocol (31).  
      
    SCOP/CATH reference:

    Most of the databases focus on the interactions between the protein domains. To define domains they employ either sequence-based or structure-based domain classification definitions, such as SCOP (13), CATH (14) and PFAM (15). Often,

  ### Attachments

  - Nucl. Acids Res.-2012-Kuang-D501-6.pdf
- ## DoSA: Database of Structural Alignments

  |  |  |
  | --- | --- |
  | Type | Journal Article |
  | Author | Swapnil Mahajan |
  | Author | Garima Agarwal |
  | Author | Mohammed Iftekhar |
  | Author | Bernard Offmann |
  | Author | Alexandre G. de Brevern |
  | Author | Narayanaswamy Srinivasan |
  | URL | http://www.ncbi.nlm.nih.gov/pmc/articles/PMC3708618/ |
  | Volume | 2013 |
  | Publication | Database: the journal of biological databases and curation |
  | Date | 2013 |
  | Accessed | 9/23/2013, 10:15:34 AM |
  | Library Catalog | Google Scholar |
  | Short Title | DoSA |
  | Date Added | 10/11/2013, 10:29:15 AM |
  | Modified | 10/11/2013, 10:29:15 AM |

  ### Notes:

  - Paper detailing the Database of Structural Alignments (DoSA), which provides new information based on the realigning of regions that were mislabelled as structurally variable regions (because their spatial confirmations had differed.) The implication is that is will provide new insight on protein homology and function.

    How SCOP is used:

    When they are organizing their new alignments, they based the hierarchy based on SCOP family and class. The actual data was obtained from the PALI database, which has their own database organized based on SCOP families.

    SCOP Reference**:**

    The improved structure-based sequence  
    alignments and their corresponding PB sequence  
    alignments can also be downloaded as text files.  
    The alignments are categorized according to SCOP  
    (24) families, which are further categorized as !, ",  
    !/", ! + ", small proteins and multi-domain proteins  
    classes.

    (vi) Multiple structure-based sequence alignments: Even if  
    the focus of our previous study (17) was on pairwise  
    alignments, for each protein family defined by SCOP  
    1.73, multiple structure-based sequence alignments  
    obtained using MUSTANG (29) and their corresponding  
    multiple PB sequence alignments are also available on the DoSA web site.

    The protein data set was obtained from the PALI v2.7  
    database (23), which contains structure-based sequence  
    alignments generated using DALI (9) for protein domain  
    families defined by the SCOP 1.73 database (24).  
    However, DoSA differs from PALI in a number of ways  
    (see below).

  ### Attachments

  - bat048.pdf
  - [HTML] from nih.gov
- ## DR\_bind: a web server for predicting DNA-binding residues from the protein structure based on electrostatics, evolution and geometry

  |  |  |
  | --- | --- |
  | Type | Journal Article |
  | Author | Yao Chi Chen |
  | Author | Jon D. Wright |
  | Author | Carmay Lim |
  | Volume | 40 |
  | Issue | W1 |
  | Pages | W249–W256 |
  | Publication | Nucleic Acids Research |
  | Date | July 2012 |
  | DOI | 10.1093/nar/gks481 |
  | Abstract | DR\_bind is a web server that automatically predicts DNA-binding residues, given the respective protein structure based on (i) electrostatics, (ii) evolution and (iii) geometry. In contrast to machine-learning methods, DR\_bind does not require a training data set or any parameters. It predicts DNA-binding residues by detecting a cluster of conserved, solvent-accessible residues that are electrostatically stabilized upon mutation to Asp(-)/Glu(-). The server requires as input the DNA-binding protein structure in PDB format and outputs a downloadable text file of the predicted DNA-binding residues, a 3D visualization of the predicted residues highlighted in the given protein structure, and a downloadable PyMol script for visualization of the results. Calibration on 83 and 55 non-redundant DNA-bound and DNA-free protein structures yielded a DNA-binding residue prediction accuracy/precision of 90/47% and 88/42%, respectively. Since DR\_bind does not require any training using protein-DNA complex structures, it may predict DNA-binding residues in novel structures of DNA-binding proteins resulting from structural genomics projects with no conservation data. The DR\_bind server is freely available with no login requirement at http://dnasite.limlab.ibms.sinica.edu.tw. |
  | Date Added | 3/7/2014, 12:08:00 PM |
  | Modified | 3/7/2014, 12:08:00 PM |
- ## Dual-Layer Wavelet SVM for Predicting Protein Structural Class Via the General Form of Chou's Pseudo Amino Acid Composition

  |  |  |
  | --- | --- |
  | Type | Journal Article |
  | Author | Chao Chen |
  | Author | Zhi-Bin Shen |
  | Author | Xiao-Yong Zou |
  | Volume | 19 |
  | Issue | 4 |
  | Pages | 422-429 |
  | Publication | Protein and Peptide Letters |
  | ISSN | 0929-8665 |
  | Date | APR 2012 |
  | Extra | WOS:000302157400006 |
  | Abstract | A prior knowledge of protein structural class can provide useful information about its overall structure. So, it is vitally important to develop a computational prediction method for fast and accurately determining the protein structural class. In this paper, a dual-layer wavelet support vector machine (WSVM) is presented via the general form of Chou's pseudo amino acid composition, which is featured by introducing wavelet as a kernel and making decisions by the fusion from three individual classifiers. As a demonstration, the rigorous jackknife cross-validation tests were performed on two benchmark datasets, including the more challenging 25PDB dataset. Our success rates were reliable, and it has not escaped from our notice that the present method has specific ability to predict the most difficult case of alpha+beta class. The program developed can be acquired freely on request from the authors. |
  | Date Added | 2/20/2014, 12:24:01 PM |
  | Modified | 2/20/2014, 12:24:01 PM |

  ### Notes:

  - Paper unavailable.
- ## Dynamic features of homodimer interfaces calculated by normal-mode analysis

  |  |  |
  | --- | --- |
  | Type | Journal Article |
  | Author | Yuko Tsuchiya |
  | Author | Kengo Kinoshita |
  | Author | Shigeru Endo |
  | Author | Hiroshi Wako |
  | Volume | 21 |
  | Issue | 10 |
  | Pages | 1503-1513 |
  | Publication | Protein Science |
  | ISSN | 0961-8368 |
  | Date | October 2012 |
  | DOI | 10.1002/pro.2140 |
  | Language | English |
  | Abstract | Knowledge of the dynamic features of protein interfaces is necessary for a deeper understanding of proteinprotein interactions. We performed normal-mode analysis (NMA) of 517 nonredundant homodimers and their protomers to characterize dimer interfaces from a dynamic perspective. The motion vector calculated by NMA for each atom of a dimer was decomposed into internal and external motion vectors in individual component subunits, followed by the averaging of time-averaged correlations between these vectors over atom pairs in the interface. This averaged correlation coefficient (ACC) was defined for various combinations of vectors and investigated in detail. ACCs decrease exponentially with an increasing interface area and r-value, that is, interface area divided by the entire subunit surface area. As the r-value reflects the nature of dimer formation, the result suggests that both the interface area and the nature of dimer formation are responsible for the dynamic properties of dimer interfaces. For interfaces with small or medium r-values and without intersubunit entanglements, ACCs are found to increase on dimer formation when compared with those in the protomer state. In contrast, ACCs do not increase on dimer formation for interfaces with large r-values and intersubunit entanglements such as in interwinding dimers. Furthermore, relationships between ACCs for intrasubunit atom pairs and for intersubunit atom pairs are found to significantly differ between interwinding and noninterwinding dimers for external motions. External motions are considered as an important factor for characterizing dimer interfaces. |
  | Date Added | 10/11/2013, 10:29:15 AM |
  | Modified | 12/2/2013, 4:16:55 PM |

  ### Tags:

  - correlative atomic fluctuations
  - external motion
  - homodimer interfaces
  - interface dynamics
  - internal motion
  - interwinding interfaces
  - normal-mode analysis
  - protein-protein interaction

  ### Notes:

  - Study of dynamics of homodimer interfaces

    How SCOP is used:

    Curate a non-redundant data set of homodimers for studying with NMA.  Use SCOP to select representative structures such that no pair of dimers belonged to the same family.

    SCOP reference:

    Materials and Methods

    Dataset

    We searched PDB as of January 2011 for homodi- meric interfaces whose structures have been deter- mined by X-ray crystallography at a resolution of 2.5 A ̊ or better and a sequence identity between the component subunits of at least 95%. Of these candi- date dimers, we eliminated the dimers that were extremely large in size to perform NMA [i.e., with a total number of atoms including hydrogen atoms in the two component subunits of more than 10,000 (approximately 600 residues)], as well as small dimers of less than 30 residues for each subunit. We then selected the representative structures based on the SCOP family classification so that no pair of dimers belonged to the same family.25 Finally, 517 nonredundant homodimeric interfaces were chosen for analysis.

  ### Attachments

  - 2140\_ftp.pdf
- ## Dynamic landscapes: A model of context and contingency in evolution

  |  |  |
  | --- | --- |
  | Type | Journal Article |
  | Author | David V. Foster |
  | Author | Mary M. Rorick |
  | Author | Tanja Gesell |
  | Author | Laura M. Feeney |
  | Author | Jacob G. Foster |
  | Volume | 334 |
  | Pages | 162-172 |
  | Publication | Journal of Theoretical Biology |
  | Date | OCT 7 2013 |
  | Extra | WOS:000323629500017 |
  | DOI | 10.1016/j.jtbi.2013.05.030 |
  | Library Catalog | ISI Web of Knowledge |
  | Abstract | Although the basic mechanics of evolution have been understood since Darwin, debate continues over whether macroevolutionary phenomena are driven by the fitness structure of genotype space or by ecological interaction. In this paper we propose a simple model capturing key features of fitness-landscape and ecological models of evolution. Our model describes evolutionary dynamics in a high-dimensional, structured genotype space with interspecies interaction. We find promising qualitative similarity with the empirical facts about macroevolution, including broadly distributed extinction sizes and realistic exploration of the genotype space. The abstraction of our model permits numerous applications beyond macroevolution, including protein and RNA evolution. (C) 2013 Elsevier Ltd. All rights reserved. |
  | Short Title | Dynamic landscapes |
  | Date Added | 10/8/2014, 12:49:22 PM |
  | Modified | 10/8/2014, 1:32:32 PM |

  ### Tags:

  - Ecological interaction
  - Fitness landscape
  - Macroevolution
  - Neutral networks
  - Percolation

  ### Attachments

  - ScienceDirect Full Text PDF
  - ScienceDirect Snapshot
- ## Dynamics alignment: Comparison of protein dynamics in the scop database

  |  |  |
  | --- | --- |
  | Type | Journal Article |
  | Author | Dror Tobi |
  | URL | http://onlinelibrary.wiley.com/doi/10.1002/prot.24017/full |
  | Publication | Proteins: Structure, Function, and Bioinformatics |
  | Date | 2012 |
  | Accessed | 2/28/2013, 1:38:04 PM |
  | Library Catalog | Google Scholar |
  | Short Title | Dynamics alignment |
  | Date Added | 10/11/2013, 10:29:15 AM |
  | Modified | 2/24/2014, 9:17:16 AM |

  ### Tags:

  - ASTRAL
  - ASTRAL domain structures
  - Cite ASTRAL
  - Interesting

  ### Notes:

  - Introduce a new method to compare protein dynamics.  First, the dynamics are measured using a Gaussian network model, and then a global alignment of the mode data is  built.  They show that different domains have similar global dynamics.

    Characterize dynamics of proteins based on SCOP class (all alpha, all beta, alpha and beta, and alpha/beta), as well as domain sizes.  Use GNM.

     How SCOP is used:

    Perform a comparative study of domain dynamics on the top four SCOP classes using all domains from ASTRAL 1.75.

     SCOP reference:

    "Several works have already shown that the slowest modes are conserved between structurally related proteins.Here we show that structurally dissimilar domains from different SCOP folds or classes may have similar slowest modes."

    We show that different domains may have similar global dynamics. In addition, we report that the dynamics of “all alpha proteins” domains are less specific to structural variations within a given fold or superfamily compared with the other classes.

    The SCOP/ASTRAL database release 1.75 was used in order to compare the dynamics of different domains. For each domain in the database we attempt to calculate the five GNM slowest modes. Out of the 110,791 domains in the database 179 were nonstandard domains like d1c51a and d4cata which are low resolution structures with unknown side chains identity. These domains were ignored and for another 680 domains the GNM program failed to calculate the global modes due to missing amino acids causing discontinuous structures.  
    ￼

  ### Attachments

  - 24017\_ftp.pdf
- ## ECOH: An Enzyme Commission number predictor using mutual information and a support vector machine

  |  |  |
  | --- | --- |
  | Type | Journal Article |
  | Author | Yoshihiko Matsuta |
  | Author | Masahiro Ito |
  | Author | Yukako Tohsato |
  | Volume | 29 |
  | Issue | 3 |
  | Pages | 365-372 |
  | Publication | Bioinformatics |
  | ISSN | 1367-4803 |
  | Date | FEB 1 2013 |
  | Extra | WOS:000314892000009 |
  | DOI | 10.1093/bioinformatics/bts700 |
  | Abstract | Motivation: The enzyme nomenclature system, commonly known as the enzyme commission (EC) number, plays a key role in classifying and predicting enzymatic reactions. However, numerous reactions have been described in various pathways that do not have an official EC number, and the reactions are not expected to have an EC number assigned because of a lack of articles published on enzyme assays. To predict the EC number of a non-classified enzymatic reaction, we focus on the structural similarity of its substrate and product to the substrate and product of reactions that have been classified. Results: We propose a new method to assign EC numbers using a maximum common substructure algorithm, mutual information and a support vector machine, termed the Enzyme COmmission numbers Handler (ECOH). A jack-knife test shows that the sensitivity, precision and accuracy of the method in predicting the first three digits of the official EC number (i.e. the EC sub-subclass) are 86.1%, 87.4% and 99.8%, respectively. We furthermore demonstrate that, by examining the ranking in the candidate lists of EC sub-subclasses generated by the algorithm, the method can successfully predict the classification of 85 enzymatic reactions that fall into multiple EC sub-subclasses. The better performance of the ECOH as compared with existing methods and its flexibility in predicting EC numbers make it useful for predicting enzyme function. |
  | Date Added | 2/20/2014, 12:24:01 PM |
  | Modified | 3/7/2014, 12:09:13 PM |

  ### Notes:

  - Present method for protein function prediction.

    How SCOP/CATH is used:

    Background on protein structure classification.

    SCOP/CATH reference:

    Systematic annotation systems have been provided for generating and testing biological hypotheses, although one should note any propagation of functional mis-annotation in the systems (Furnham et al., 2009). For example, Gene Ontology (GO) is a controlled vocabulary that can be used to describe gene products (Ashburner et al., 2000). Structural Classification Of Proteins (SCOP) (Andreeva et al., 2008) and Class, Architecture, Topology, Homologous superfamily (CATH) (Cuff et al., 2011) regard any pair of enzymes that share protein domains as being similar.

  ### Attachments

  - Bioinformatics-2013-Matsuta-365-72.pdf
- ## Effective inter-residue contact definitions for accurate protein fold recognition

  |  |  |
  | --- | --- |
  | Type | Journal Article |
  | Author | Chao Yuan |
  | Author | Hao Chen |
  | Author | Daisuke Kihara |
  | Volume | 13 |
  | Pages | 292 |
  | Publication | Bmc Bioinformatics |
  | ISSN | 1471-2105 |
  | Date | NOV 9 2012 |
  | Extra | WOS:000312894400001 |
  | DOI | 10.1186/1471-2105-13-292 |
  | Abstract | Background: Effective encoding of residue contact information is crucial for protein structure prediction since it has a unique role to capture long-range residue interactions compared to other commonly used scoring terms. The residue contact information can be incorporated in structure prediction in several different ways: It can be incorporated as statistical potentials or it can be also used as constraints in ab initio structure prediction. To seek the most effective definition of residue contacts for template-based protein structure prediction, we evaluated 45 different contact definitions, varying bases of contacts and distance cutoffs, in terms of their ability to identify proteins of the same fold. Results: We found that overall the residue contact pattern can distinguish protein folds best when contacts are defined for residue pairs whose C beta atoms are at 7.0 angstrom or closer to each other. Lower fold recognition accuracy was observed when inaccurate threading alignments were used to identify common residue contacts between protein pairs. In the case of threading, alignment accuracy strongly influences the fraction of common contacts identified among proteins of the same fold, which eventually affects the fold recognition accuracy. The largest deterioration of the fold recognition was observed for beta-class proteins when the threading methods were used because the average alignment accuracy was worst for this fold class. When results of fold recognition were examined for individual proteins, we found that the effective contact definition depends on the fold of the proteins. A larger distance cutoff is often advantageous for capturing spatial arrangement of the secondary structures which are not physically in contact. For capturing contacts between neighboring beta strands, considering the distance between C alpha atoms is better than the C beta-based distance because the side-chain of interacting residues on beta strands sometimes point to opposite directions. Conclusion: Residue contacts defined by C beta-C beta distance of 7.0 angstrom work best overall among tested to identify proteins of the same fold. We also found that effective contact definitions differ from fold to fold, suggesting that using different residue contact definition specific for each template will lead to improvement of the performance of threading. |
  | Date Added | 2/20/2014, 12:24:01 PM |
  | Modified | 2/20/2014, 12:24:01 PM |

  ### Notes:

  - Assess different methods for contact map prediction for protein structure prediction.

    How SCOP is used:

    Validated that contact definition methods could be used to identify proteins of the same fold.  Domain dataset was derived from SCOP.

    SCOP reference:

    Dataset of domain structures of globular proteins

    Two sets of domain structures of globular proteins were selected according to the SCOP database (release 1.73)

    [48], one for representative protein folds and another one for representative superfamilies. We selected protein folds that have at least three superfamilies, from each of which one domain structure was selected. Entries were discarded if their PDB files contain only Cα traces. In total, 194 folds were selected. The numbers of structures in each fold range from 3 to 110. In total, there are 2167 structures in the fold dataset. Similarly, a dataset of 250 representative superfamilies that contains a total of 1672 structures were selected. Each superfamily in the dataset contains at least three families, from each of which one structure was selected. In the following part, we will

    explain the experiment procedure on the fold dataset and readers should be aware that the same procedure was performed on the superfamily dataset.

  ### Attachments

  - 1471-2105-13-292.pdf
- ## Effective Moment Feature Vectors for Protein Domain Structures

  |  |  |
  | --- | --- |
  | Type | Journal Article |
  | Author | Jian-Yu Shi |
  | Author | Siu-Ming Yiu |
  | Author | Yan-Ning Zhang |
  | Author | Francis Yuk-Lun Chin |
  | Volume | 8 |
  | Issue | 12 |
  | Pages | e83788 |
  | Publication | Plos One |
  | ISSN | 1932-6203 |
  | Date | DEC 31 2013 |
  | Extra | WOS:000329325200126 |
  | DOI | 10.1371/journal.pone.0083788 |
  | Abstract | Imaging processing techniques have been shown to be useful in studying protein domain structures. The idea is to represent the pairwise distances of any two residues of the structure in a 2D distance matrix (DM). Features and/or submatrices are extracted from this DM to represent a domain. Existing approaches, however, may involve a large number of features (100-400) or complicated mathematical operations. Finding fewer but more effective features is always desirable. In this paper, based on some key observations on DMs, we are able to decompose a |
  | Date Added | 2/20/2014, 12:24:01 PM |
  | Modified | 3/7/2014, 12:08:54 PM |

  ### Notes:

  - Present distance-matrix method approach for protein profiling and classification.

    How SCOP/CATH is used:

    Train and validate against CATH, then SCOP, domains and classifications.

    Validate against SCOP class, fold, and superfamily classifications.

    SCOP reference:

    We illustrate the effectiveness of our feature vectors in the following manner. (1) Based on two well-known protein domain classification databases, CATH [17] and SCOP [18], we compare the accuracy of prediction using our moment vector versus other representations. We show that we achieve a much higher accuracy at all levels. (2) Using our moment vectors, we construct a 3D domain structure universe. We are able to show and visualize a clear and consistent distribution of domains in this universe. (3) We cluster the domains according to our moment vectors and demonstrate a relationship between structural variation and functional diversity.

    ...

    We also evaluated the moment features using another popular classification database SCOP(v 1.75). Again, the top 500 superfamilies (totally 109, 533 domains, denoted as S\_H500) were selected for evaluation. Following a similar training scheme as above, we found that the results of classification are also consistent with those of SCOP in all levels (Table 2).

    We found that Legendre moments are powerful enough to distinguish folds within a class (see Fig. S2) and superfamilies within a fold (see Fig. S3). This explains why moment features can achieve much higher classification accuracies even for more fine- grained classification levels. Note that in both evaluations of CATH and SCOP, SVM seems to perform better, so we used SVM in our experiments. In the rest of the paper, we focus on SCOP classification because SCOP treats a=b and azb domains separately whereas CATH merges them into mixed a{b class.

    ...

    scribed in Section S1).

    Using #C as a measure of structural variation across a superfamily has also been used in previous work [37]. In order to validate such measurement, another independent measure of

    structural variation is calculated by a pairwise structural alignment algorithm, called jFATCAT (freely available in http://www.rcsb. org/pdb/workbench/workbench.do). We selected two superfam- ilies, a.60.9 and a.69.1, from SCOP (V1.75) and investigated the alignment scores between domains in each superfamily respec- tively. The higher the alignment score is, the less structural variations two domains have. The detailed scores are listed in Score S1. In the proposed structural space, the superfamily a.60.9 (SCOP Name: lambda integrase-like, N-terminal domain) shows 3 clusters which contain 48, 12 and 1 domains and are rendered by red, green and blue respectively (see Fig. 5-A). The ranges ([minima, maxima]) of alignment scores within each cluster and between any cluster pair are calculated. In details, the range of alignment scores within the red cluster is [292.48, 335.52], while the score range between red and green clusters is [106.41, 128.05] and that between red and blue clusters is [153.82, 177.25]. And the score range within green cluster is [313.57, 359.92] while the score range between green and blue clusters is [114.83, 123.53]. Obviously, the scores are higher within each cluster, the scores are lower between domains in different clusters, and there is even no overlap between score ranges of domains within-cluster and between-clusters. This provides an additional independent evi- dence showing that moment features are useful to classify structural difference of domains. More importantly, three clusters just represent three different types of tyrosine recombinases (Flp recombinase, Cre recombinase and Recombinase XerD) which share conserved DNA binding mechanism in recombination reaction, but also show apparent mechanistic and regulatory differences [38]. Another superfamily a.69.1(SCOP Name: C-terminal domain of alpha and beta subunits of F1 ATP synthase) also shows similar results of structural comparison. It has 2 spatial clusters in structural space, each of which is composed of 47 domains. One cluster denotes alpha subunit of F1 ATP synthase and another denotes its beta subunit. Three beta subunits in F1 component are the ATP-ADP binding sites whereas alpha subunits are not sites but just support the F1 ATP synthases structure, even though they are placed together with the alternating arrangement in F1 ATP synthase [39]. Consequently, the above results demonstrates that structural variations of domains in a superfamily strongly related to the diversity of their functions according to the annotations in SCOP, and also illustrate that #C can capture the number of diverse functions, despite the fact that the domains can still have common conserved functional features, in a superfamily.

  ### Attachments

  - journal.pone.0083788.pdf
- ## Effects of point mutations on protein structure are nonexponentially distributed

  |  |  |
  | --- | --- |
  | Type | Journal Article |
  | Author | Tomasz Arodź |
  | Author | Przemys\law M. P\lonka |
  | URL | http://onlinelibrary.wiley.com/doi/10.1002/prot.24073/full |
  | Volume | 80 |
  | Issue | 7 |
  | Pages | 1780–1790 |
  | Publication | Proteins: Structure, Function, and Bioinformatics |
  | Date | 2012 |
  | Accessed | 9/23/2013, 10:18:03 AM |
  | Library Catalog | Google Scholar |
  | Date Added | 10/11/2013, 10:29:15 AM |
  | Modified | 10/11/2013, 10:29:15 AM |

  ### Tags:

  - amino acid substitutions
  - computational biology
  - PDB
  - protein evolution
  - protein structure change
  - SCOP coverage insufficient

  ### Notes:

  - Perform a large scale study on PDB chains differing by only a single residue.  For all such pairs in the data set,perform a structure alignment and measure the RMSDs.  They found that large structural changes due to point mutations were not uncommon.

    How used SCOP:

    To filter data set, to exclude multi-domain chains, low-res chains, Ig superfamily, etc.

    SCOP reference:

    From all PDB entries, we selected those determined using X-ray crystallography, and meeting criteria including: single amino acid chain 100–200 residues in length, ... On the basis of Structural Classification of Proteins (SCOP) classification, we excluded chains with more than one domain, proteins from the “Multidomain proteins (alpha and beta)” class, from “Membrane and cell surface proteins and peptides” class, “Immunoglobulin” superfamily, and “Low-resolution protein structures,” and “peptides” pseudo-classes. **If SCOP classification was missing for the entry, we tried to infer it based on the closest homolog with at least 40% of sequence identity in global alignment.20 We excluded..**

  ### Attachments

  - 24073\_ftp.pdf
  - Snapshot
- ## Efficient Approaches for Retrieving Protein Tertiary Structures

  |  |  |
  | --- | --- |
  | Type | Journal Article |
  | Author | Georgina Mirceva |
  | Author | Ivana Cingovska |
  | Author | Zoran Dimov |
  | Author | Danco Davcev |
  | URL | http://dl.acm.org/citation.cfm?id=2223934 |
  | Volume | 9 |
  | Issue | 4 |
  | Pages | 1166–1179 |
  | Publication | IEEE/ACM Transactions on Computational Biology and Bioinformatics (TCBB) |
  | Date | 2012 |
  | Accessed | 9/23/2013, 10:20:20 AM |
  | Library Catalog | Google Scholar |
  | Date Added | 10/11/2013, 10:29:15 AM |
  | Modified | 2/20/2014, 1:33:37 PM |

  ### Tags:

  - feature extraction
  - Information search and retrieval
  - protein databases

  ### Notes:

  - Present several approaches for fast and accurate indexing of protein tertiary structures. Compare approaches against several existing approaches for protein structure retrieval.

    How SCOP is used:

    Created a training set of domains from SCOP 1.73 and a benchmarking set of newly added domains in 1.75.

    Using fold level for validation: "A training protein is considered to be relevant if it belongs to the same SCOP domain as the query protein chain."

    SCOP references:

    4 EXPERIMENTAL RESULTS

    We have implemented a system for protein structures retrieval based on the approaches described above. Part of SCOP 1.75 database [47], [48] was used in the evaluation. The SCOP method classifies protein chains in hierarchical manner. The main level in the SCOP hierarchy is the domain level, so the accuracies of the methods for retrieving or classifying protein structures are usually evaluated according to this level.

    We used a representative data set (PDB100) [49] obtained by filtering the SCOP 1.75 protein chains, so that each pair of chains has less than 100 percent sequence similarity. Only domains with at least two representatives were considered. In this way 28,460 protein chains from 5,235 SCOP domains were taken. The protein chains are not uniformly distributed across the domains. We used two criteria to divide the set into training and test set. With the first criterion, the test data are formed from the protein chains which are classified in the SCOP 1.75, but are not classified in SCOP 1.73, or are reclassified in SCOP 1.75. All the other chains are used as a training data. In this way, we formed our data set1 which contains 26,820 training and 1,640 test data. The second criterion filters the PDB100 data set to form the test data, so that each pair of test protein chains has sequence similarity less than 10 percent [49]. In this way we obtained our data set2 which contains 25,146 training and 3,314 test data. Since the second criterion considers protein chains with a low sequence similarity, the most representative protein chains are considered as a test data. As a supplemental material, which can be found on the Computer Society Digital Library at http://doi.ieeecomputersociety.org/10.1109/TCBB. 2011.138, we provide detailed information about the protein chains which are used.

    ...

    . We have randomly selected 6,851 protein chains from SCOP 1.75 database. These chains are from the

  ### Attachments

  - Snapshot
  - ttb2012041166.pdf
- ## Efficient Methods for Robust Classification Under Uncertainty in Kernel Matrices

  |  |  |
  | --- | --- |
  | Type | Journal Article |
  | Author | Aharon Ben-Tal |
  | Author | Sahely Bhadra |
  | Author | Chiranjib Bhattacharyya |
  | Author | Arkadi Nemirovski |
  | URL | http://jmlr.org/papers/volume13/ben-tal12a/ben-tal12a.pdf |
  | Volume | 13 |
  | Pages | 2923–2954 |
  | Publication | Journal of Machine Learning Research |
  | Date | 2012 |
  | Accessed | 9/20/2013, 1:18:03 PM |
  | Library Catalog | Google Scholar |
  | Date Added | 10/11/2013, 10:29:15 AM |
  | Modified | 3/7/2014, 1:07:13 PM |

  ### Tags:

  - kernel functions
  - robust optimization
  - uncertain classification

  ### Notes:

  - Presents a model for classifying protein domains using structure alignment.

    How SCOP is used:

    They downloaded a domain structure dataset from the SCOP database on which to run their algorithm. The dataset comes from domains from 15 different superfamilies (taken from another paper).

    SCOP Reference:

    For example, consider two proteins2 d1vsra1(denote it by P) and d1gefa1(denote it by P′) belonging to protein superfamily Restrictionendo nuclease-like. The value of r for P is 1.8A ̊ and for P′ it is 2.0A ̊ respectively.

    2. One should refer to them as SCOP domains. But to lighten the discussion on the biology side we refer to them as proteins.

    We have used a data set based on the SCOP (Murzin et al., 1995) 40% sequence non-redundant data set taken from Bhadra et al. (2010). The data set has 15 classes (SCOP superfamilies), having 10 structures each. The names of these superfamilies are reported in Appendix D. To study the effect of robustness we studied the classification problem on all possible pairs, which gave rise to 105 data sets in total. E

  ### Attachments

  - ben-tal12a.pdf
- ## Efficient Parameter Estimation of Generalizable Coarse-Grained Protein Force Fields Using Contrastive Divergence: A Maximum Likelihood Approach

  |  |  |
  | --- | --- |
  | Type | Journal Article |
  | Author | Csilla Varnai |
  | Author | Nikolas S. Burkoff |
  | Author | David L. Wild |
  | Volume | 9 |
  | Issue | 12 |
  | Pages | 5718-5733 |
  | Publication | Journal of Chemical Theory and Computation |
  | ISSN | 1549-9618; 1549-9626 |
  | Date | DEC 2013 |
  | Extra | WOS:000328437500050 |
  | DOI | 10.1021/ct400628h |
  | Abstract | Maximum Likelihood (ML) optimization schemes are widely used for parameter inference. They maximize the likelihood of some experimentally observed data, with respect to the model parameters iteratively, following the gradient of the logarithm of the likelihood. Here, we employ a ML inference scheme to infer a generalizable, physics-based coarse-grained protein model (which includes G (o) over bar -like biasing terms to stabilize secondary structure elements in room-temperature simulations), using native conformations of a training set of proteins as the observed data. Contrastive divergence, a novel statistical machine learning technique, is used to efficiently approximate the direction of the gradient ascent, which enables the use of a large training set of proteins. Unlike previous work, the generalizability of the protein model allows the folding of peptides and a protein (protein G) which are not part of the training set. We compare the same force field with different van der Waals (vdW) potential forms: a hard cutoff model, and a Lennard-Jones (LJ) potential with vdW parameters inferred or adopted from the CHARMM or AMBER force fields. Simulations of peptides and protein G show that the LJ model with inferred parameters outperforms the hard cutoff potential, which is consistent with previous observations. Simulations using the LJ potential with inferred vdW parameters also outperforms the protein models with adopted vdW parameter values, demonstrating that model parameters generally cannot be used with force fields with different energy functions. The software is available at https://sites.google.com/site/crankite/. |
  | Date Added | 2/20/2014, 12:24:01 PM |
  | Modified | 2/20/2014, 12:24:01 PM |

  ### Notes:

  - Proposed a forcefield (i.e. energy function) for course-grained models.

    How SCOP is used:

    Use domain structures from SCOP 1.75 taken from ASTRAL, with less than 40% sequence identity, as a set of known protein structures representing thermal equilibrium.

    Don't reference SCOP, only ASTRAL.

    SCOP reference:

    As the data set of known protein structures representing thermodynamic equilibrium, we use a subset of the protein structures in the ASTRAL 1.75 database.66 To avoid proteins with high sequence similarity, proteins with less than 40% sequence identity were included. The ASTRAL 1.75 database contains three-dimensional (3D) structures of protein domains, classified into folding classes. For each structure, a Summary PDB ASTRAL Check Index (SPACI)67 score is assigned, indicating the reliability of crystallographically determined structures. All PDB structures from the α, β, α+β, and α/β classes of the ASTRAL 1.75 database with SPACI scores above 0.8 were included in the dataset, excluding the ones with missing residues, disulfide bonds, or unusual residues.

    Following the inference, the hydrophobic interaction strength kh needed modification. kh was increased by 0.1 RT, which was necessary for the protein folding simulations to stabilize the conformation with the hydrophobic residues in the interior of the protein. Although the hydrophobic interaction strength was sufficient to preserve the folded structure of the proteins in the database, it was not sufficiently strong for folding proteins from an unfolded state. A possible reason for the learnt value of kh being too small could be that the ASTRAL

  ### Attachments

  - ct400628h.pdf
- ## Efficient prediction algorithms for binary decomposition techniques

  |  |  |
  | --- | --- |
  | Type | Journal Article |
  | Author | Sang-Hyeun Park |
  | Author | Johannes Fürnkranz |
  | URL | http://link.springer.com/article/10.1007/s10618-011-0219-9 |
  | Volume | 24 |
  | Issue | 1 |
  | Pages | 40–77 |
  | Publication | Data Mining and Knowledge Discovery |
  | Date | 2012 |
  | Accessed | 9/23/2013, 10:15:21 AM |
  | Library Catalog | Google Scholar |
  | Date Added | 10/11/2013, 10:29:15 AM |
  | Modified | 3/7/2014, 12:14:07 PM |

  ### Tags:

  - Aggregation
  - Binary decomposition
  - Efficient decoding
  - Efficient voting
  - Multiclass classification
  - Pairwise classification
  - Ternary ECOC

  ### Notes:

  - Machine learning paper on binary decomposition method.

    "In this paper, we discuss an efficient algorithm  
    that queries only a dynamically determined subset of the trained classifiers,  
    but still predicts the same classes that would have been predicted if all classifiers  
    had been queried."

    How SCOP is used:

    **Benchmakred** method on the SCOP classification from class to family. Used ASTRAL to filter the sequences.

    SCOP Referenc:

    ASTRAL 2 & 3  
    These datasets describe protein sequences retrieved from the SCOP 1.71  
    protein database (Murzin et al. 1995). We used ASTRAL (Brenner et al.  
    2000) to filter these sequences so that no two sequences share greater than  
    95% identity. The class labels are organized in a 3-level hierarchy, consisting  
    of protein folds, superfamilies and families (in descending order). astral3  
    consists of 1,588 classes and contains the original hierarchy.

  ### Attachments

  - [PDF] from tu-darmstadt.de
  - Snapshot
- ## Efficient protein structure search using indexing methods

  |  |  |
  | --- | --- |
  | Type | Journal Article |
  | Author | Sungchul Kim |
  | Author | Lee Sael |
  | Author | Hwanjo Yu |
  | URL | http://www.biomedcentral.com/1472-6947/13/S1/S8/ |
  | Volume | 13 |
  | Issue | Suppl 1 |
  | Pages | S8 |
  | Publication | BMC medical informatics and decision making |
  | Date | 2013 |
  | Accessed | 9/23/2013, 10:20:00 AM |
  | Library Catalog | Google Scholar |
  | Date Added | 10/11/2013, 10:29:15 AM |
  | Modified | 3/7/2014, 12:09:30 PM |

  ### Notes:

  - The paper examines two different indexing techniques (iDistance and iKernel) to represent a protein structure on 3D-Zernike Descriptor. This is examine ways to reduce the time needed to search by protein structure and find similar structures.

    How SCOP/CATH is used:

    Not using SCOP or CATH data.

    They note that 3D Surfer proteins are organized similarly to SCOP. They note that searching works well based on the SCOP classification (noting families in particular).

    SCOP/CATH reference:

    There are major structure databases such as PDB,  
    CATH [14], and SCOP [15] which provides only keyword  
    search and browsing of pre-computed classification.

    It has been verified that the  
    retrieved k-nn proteins by 3D-Surfer have similar functional  
    and evolutional information in terms of SCOP classification  
    [20].

    In this section, we verify the effectiveness of indexing techniques  
    on top-k search of protein structures. Sael et al.  
    showed that 3DZD works well on finding similar proteins  
    in terms of functional and evolutionary characteristics  
    based on SCOP classification [1]. The SCOP provides the  
    ordering of all proteins of known structure according to  
    their evolutionary and structural relationships.

    We vary the partition data points  
    using different number of clusters: 121, 242, 498, and 866.  
    121 is the dimensionality of data set, and 242 is the two  
    times the dimensionality ([29] refers that this way works  
    well on iDistance). And the others are according to SCOP  
    classification hierarchy. 498 is the number of families, and  
    866 is the number of protein domains [15].

  ### Attachments

  - 1472-6947-13-S1-S8.pdf
- ## Electron Spin Density on the Axial His Ligand of High-Spin and Low-Spin Nitrophorin 2 Probed by Heteronuclear NMR Spectroscopy

  |  |  |
  | --- | --- |
  | Type | Journal Article |
  | Author | Luciano A. Abriata |
  | Author | Maria-Eugenia Zaballa |
  | Author | Robert E. Berry |
  | Author | Fei Yang |
  | Author | Hongjun Zhang |
  | Author | F. Ann Walker |
  | Author | Alejandro J. Vila |
  | Volume | 52 |
  | Issue | 3 |
  | Pages | 1285-1295 |
  | Publication | Inorganic Chemistry |
  | ISSN | 0020-1669 |
  | Date | FEB 4 2013 |
  | Extra | WOS:000314627700018 |
  | DOI | 10.1021/ic301805y |
  | Abstract | The electronic structure of heme proteins is exquisitely tuned by the interaction of the iron center with the axial ligands. NMR studies of paramagnetic heme systems have been focused on the heme signals, but signals from the axial ligands have been rather difficult to detect and assign. We report an extensive assignment of the H-1, C-13 and N-15 resonances of the axial His ligand in the NO-carrying protein nitrophorin 2 (NP2) in the paramagnetic high-spin and low-spin forms, as well as in the diamagnetic NO complex. We find that the high-spin protein has sigma spin delocalization to all atoms in the axial His57, which decreases in size as the number of bonds between Fe(III) and the atom in question increases, except that within the His57 imidazole ring the contact shifts are a balance between positive sigma and negative pi contributions. In contrast, the low-spin protein has pi spin delocalization to all atoms of the imidazole ring. Our strategy, adequately combined with a selective residue labeling scheme, represents a straightforward characterization of the electron spin density in heme axial ligands. |
  | Date Added | 2/13/2014, 4:13:17 PM |
  | Modified | 3/7/2014, 12:10:23 PM |
- ## Elementary Flux Modes Analysis of Functional Domain Networks Allows a Better Metabolic Pathway Interpretation

  |  |  |
  | --- | --- |
  | Type | Journal Article |
  | Author | Sabine Peres |
  | Author | Liza Felicori |
  | Author | Franck Molina |
  | Volume | 8 |
  | Issue | 10 |
  | Pages | UNSP e76143 |
  | Publication | Plos One |
  | ISSN | 1932-6203 |
  | Date | OCT 29 2013 |
  | Extra | WOS:000326270700003 |
  | DOI | 10.1371/journal.pone.0076143 |
  | Abstract | Metabolic network analysis is an important step for the functional understanding of biological systems. In these networks, enzymes are made of one or more functional domains often involved in different catalytic activities. Elementary flux mode (EFM) analysis is a method of choice for the topological studies of these enzymatic networks. In this article, we propose to use an EFM approach on networks that encompass available knowledge on structure-function. We introduce a new method that allows to represent the metabolic networks as functional domain networks and provides an application of the algorithm for computing elementary flux modes to analyse them. Any EFM that can be represented using the classical representation can be represented using our functional domain network representation but the fine-grained feature of functional domain networks allows to highlight new connections in EFMs. This methodology is applied to the tricarboxylic acid cycle (TCA cycle) of Bacillus subtilis, and compared to the classical analyses. This new method of analysis of the functional domain network reveals that a specific inhibition on the second domain of the lipoamide dehydrogenase (pdhD) component of pyruvate dehydrogenase complex leads to the loss of all fluxes. Such conclusion was not predictable in the classical approach. |
  | Date Added | 2/20/2014, 12:24:01 PM |
  | Modified | 3/7/2014, 12:09:04 PM |

  ### Notes:

  - Present method for metabolic network analysis.

    "We introduce a new method that allows to represent the metabolic networks as functional domain networks and provides an application of the algorithm for computing elementary flux modes to analyse them."

    How SCOP is used:

    Annotate a non-scop data set with SCOP classification (class, fold, superfamily) and domain boundaries.  Then use to domains to build a metabolic network of enzyme domains.

    How CATH is used:

    Background on protein structure classification.

    SCOP reference:

    Methods

    Domain Function Assignment

    To build a metabolic network of functional units represented by enzyme domains, we have to identify for each enzyme, its structural domains and the elementary actions they provide. To identify the structural domains we perform a systematic molecular modelling of all enzymes of the network, thanks to a pipeline dedicated to protein structure modelling using Python and Perl routines, and use the homology modelling software Modeller version 9v4 [10,11].

    Briefly said, this routine recursively takes the list of target sequences as input file (profile.py) and does a multiple (multa- lign.pl) or single alignment (salign.py), then generates the model

    (model.py). The best model of each enzyme is selected based on the lowest modeller objective function score (MOF). After this step, the structural domains of the proteins are assigned to each of the protein models using fastSCOP [12,13]. Besides, fastSCOP, PFAM [14,15], Swiss-Prot [16] and literature review were used for the functional domain assignment.

  ### Attachments

  - journal.pone.0076143.pdf
- ## Energetic selection of topology in ferredoxins

  |  |  |
  | --- | --- |
  | Type | Journal Article |
  | Author | J. Dongun Kim |
  | Author | Agustina Rodriguez-Granillo |
  | Author | David A. Case |
  | Author | Vikas Nanda |
  | Author | Paul G. Falkowski |
  | URL | http://dx.plos.org/10.1371/journal.pcbi.1002463 |
  | Volume | 8 |
  | Issue | 4 |
  | Pages | e1002463 |
  | Publication | PLoS computational biology |
  | Date | 2012 |
  | Accessed | 9/20/2013, 1:19:04 PM |
  | Library Catalog | Google Scholar |
  | Date Added | 10/11/2013, 10:29:15 AM |
  | Modified | 10/11/2013, 10:29:15 AM |

  ### Notes:

  - Study how fold-handedness is selected-for.  Analyze structural motifs in ferredoxin fold using de novo protein design methods.

    How SCOP is used:

    To look up the fold classification for proteins with a particular sequence motif (conserved heptapeptide sequence motif CXXXCXXC).

    SCOP reference:

    Among the CXXCXXC motifs, about 85% (31 out of 36) have a ferredoxin fold and approximately 15% have globin-like folds and others as defined by Structural Classification of Proteins (SCOP) [39].

  ### Attachments

  - [HTML] from plos.org
  - journal.pcbi.1002463.pdf
  - PubMed entry
- ## Enhanced genome annotation using structural profiles in the program 3D-PSSM

  |  |  |
  | --- | --- |
  | Type | Journal Article |
  | Author | Lawrence A. Kelley |
  | Author | Robert M. MacCallum |
  | Author | Michael JE Sternberg |
  | URL | http://www.sciencedirect.com/science/article/pii/S0022283600937410 |
  | Volume | 299 |
  | Issue | 2 |
  | Pages | 501–522 |
  | Publication | Journal of molecular biology |
  | Date | 2000 |
  | Accessed | 10/10/2013, 1:18:50 PM |
  | Library Catalog | Google Scholar |
  | Date Added | 10/11/2013, 10:29:15 AM |
  | Modified | 10/11/2013, 10:29:15 AM |

  ### Notes:

  - 3D-PSSM method to detect remote homologs.

    How SCOP is used:

    Use SCOP data (1) to build a profile library that is integral to their method and (2) to benchmark their predictive method.

    The profile library seems to be composed of multiple sequence alignments of all structures in each SCOP superfamily.

    The benchmark data set, derived from SCOP, consists of remote homologs that were deemed "undetectable" via PSI-BLAST.

    SCOP reference:

    Under Abstract:

    The method uses structural alignments of homologous proteins of similar three-dimenional structure in the structural classifiication of proteins (SCOP) data-base to obtain a structural equivalence of residues.

    Under Approach: Generation of profile library.

    The library of known structures is taken from the classification of proteins into homologous superfamilies in the structural classification of proteins (SCOP) database.

    Under Approach:  benchmark:

    The method uses structural alignments of homologous proteins of similar three-dimensional structure in the structural classification of proteins (SCOP) database to obtain a structural equivalence of residues.

  ### Attachments

  - 3D-PSSM-JMB-2000.pdf
  - Snapshot
- ## Enhancement of initial equivalency for protein structure alignment based on encoded local structures

  |  |  |
  | --- | --- |
  | Type | Journal Article |
  | Author | K Hung |
  | Author | J Wang |
  | Author | C Chen |
  | Author | C Chuang |
  | Author | K Tsai |
  | Author | C Chen |
  | Publication | IEEE journal of biomedical and health informatics |
  | ISSN | 2168-2208 |
  | Date | Jun 14, 2012 |
  | Extra | PMID: 22717522 |
  | Journal Abbr | IEEE J Biomed Health Inform |
  | DOI | 10.1109/TITB.2012.2204892 |
  | Library Catalog | NCBI PubMed |
  | Language | eng |
  | Abstract | Most alignment algorithms find an initial equivalent residue pair followed by an iterative optimization process to explore better near-optimal alignments in the surrounding solution space of the initial alignment. It plays a decisive role in determining the alignment quality since a poor initial alignment may make the final alignment trapped in an undesirable local optimum even with an iterative optimization. We proposed a vector-based alignment algorithm with a new initial alignment approach accounting for local structure features called MIRAGE-align. The new idea is to enhance the quality of the initial alignment based on encoded local structural alphabets to identify the protein structure pair whose sequence identity falls in or below twilight zone. The statistical analysis of alignment quality based on Match Index (MI) and computation time demonstrated that MIRAGE-align algorithm outperformed four previously published algorithms, i.e., the residue-based algorithm (CE), the vector-based algorithm (SSM), TM-align, and Fr-TM-align. MIRAGE-align yields a better estimate of initial solution to enhance the quality of initial alignment and enable the employment of a non-iterative optimization process to achieve a better alignment. |
  | Date Added | 11/11/2013, 4:55:58 PM |
  | Modified | 11/11/2013, 4:56:13 PM |

  ### Tags:

  - Initial equivalency
  - secondary structure
  - Structural alphabet
  - Structure alignment

  ### Notes:

  - The paper details a "vector-based alignment algorithm" (for protein structure alignment).

    How SCOP is used:

    They ran their algorithm against two different datasets created using SCOP data. The first set are domains that are similar in structure (same superfamilies) but have low sequence identity and the second, bigger, set is also low sequence identity, but from different superfamilies, selected at random.

    SCOP Reference:

     As the test data, 68 protein pairs of Fischer’s benchmark [25] and  
    600 protein pairs with less than 30% sequence identity were randomly  
    selected from structural classification of proteins (SCOP)  
    database [26].

    II. MATERIALS AND METHODS  
    In SCOP database, different domains of the same superfamily  
    share low-sequence identities but their structures and functional  
    features suggesting a common evolutionary origin is probable.  
    Domains in the same family are likely to have a common ancestor  
    based on sequence similarity or functional evidence [27]  
    and they are clustered in the same family based on one of the  
    two criteria. The first is that the amino acid sequence identities  
    of the family members are greater than 30% and the second is  
    the family members possess similar function and structure yet  
    with a lower level of amino acid sequence identity. For assessment  
    of performance on the proposed algorithm, two test sets  
    were selected to evaluate alignment quality and the computation  
    efficiency. One was the benchmark of Fischer et al., which  
    comprised 68 protein pairs. All pairs of the set were known  
    to have similar structures with low-sequence identity, ranging  
    from 8% to 31%. The other was a larger test data, 600 protein  
    pairs with length from 62 to 629 residues and sequence identity  
    less than 30%, were randomly selected from different protein  
    superfamilies in SCOP database.

    The training dataset was exclusively different  
    from the test data, 600 protein pairs which were randomly  
    selected from SCOP database. These training protein chains  
    were segmented into 469084 fragments of four residues in a  
    sliding-window fashion, each of which carried representative  
    local structural information.

    Results

    Two sets of test data, including 68 protein pairs of Fischer’s  
    benchmark and 600 protein pairs with less than 30% sequence  
    identity, were randomly selected from different protein superfamilies  
    in SCOP database. For each protein pair, all pairs of  
    SSEs in the protein pair were used as reference vectors individually  
    to obtain a set of possible SSE pairings and the top  
    five SSE pairings were identified at the first stage of the vectorbased  
    initial alignment for further selection. At the second stage,  
    these five SSE pairings were superimposed to determine the top  
    two SSE pairings for the alphabet code-based local structure  
    alignment.

    Conclusion

    The computation time and  
    alignment quality of the proposed algorithm were also evaluated  
    using 600 protein pairs randomly selected from SCOP with less  
    than 30% sequence identity from different superfamilies.

  ### Attachments

  - 06218183.pdf
  - PubMed entry
- ## Entropic Origin of Cobalt-Carbon Bond Cleavage Catalysis in Adenosylcobalamin-Dependent Ethanolamine Ammonia-Lyase

  |  |  |
  | --- | --- |
  | Type | Journal Article |
  | Author | Miao Wang |
  | Author | Kurt Warncke |
  | Volume | 135 |
  | Issue | 40 |
  | Pages | 15077-15084 |
  | Publication | Journal of the American Chemical Society |
  | Date | OCT 9 2013 |
  | Extra | WOS:000326356400032 |
  | DOI | 10.1021/ja404467d |
  | Library Catalog | ISI Web of Knowledge |
  | Abstract | Adenosylcobalamin-dependent enzymes accelerate the cleavage of the cobalt carbon (Co-C) bond of the bound coenzyme by >10(10)-fold. The cleavage-generated 5'-deoxyadenosyl radical initiates the catalytic cycle by abstracting a hydrogen atom from substrate. Kinetic coupling of the Co-C bond cleavage and hydrogen-atom-transfer steps at ambient temperatures has interfered with past experimental attempts to directly address the factors that govern Co-C bond cleavage catalysis. Here, we use time-resolved, full-spectrum electron paramagnetic resonance spectroscopy, with temperature-step reaction initiation, starting from the enzyme coenzyme substrate ternary complex and H-2-labeled substrate, to study radical pair generation in ethanolamine ammonia-lyase from Salmonella typhimurium at 234-248 K in a dimethylsulfoxide/Water cryosolvent system. The monoexponential kinetics of formation of the H-2- and H-1-substituted substrate radicals are the same, indicating that Co-C bond cleavage rate-limits radical pair formation. Analysis of the kinetics by using a linear, three-state model allows extraction of the microscopic rate constant for Co-C bond cleavage. Eyring analysis reveals that the activation enthalpy for Co-C bond cleavage is 32 +/- 1 kcal/mol, which is the same as for the cleavage reaction in solution. The origin of Co-C bond cleavage catalysis in the enzyme is, therefore, the large, favorable activation entropy of 61 +/- 6 cal/(mol.K) (relative to 7 +/- 1 cal/(mol.K) in solution). This represents a paradigm shift from traditional, enthalpy-based mechanisms that have been proposed for Co-C bond-breaking in B-12 enzymes. The catalysis is proposed to arise from an increase in protein configurational entropy along the reaction coordinate. |
  | Date Added | 10/8/2014, 12:47:53 PM |
  | Modified | 10/8/2014, 1:32:43 PM |

  ### Attachments

  - ACS Full Text PDF w/ Links
  - ACS Full Text Snapshot
- ## Enzyme informatics

  |  |  |
  | --- | --- |
  | Type | Journal Article |
  | Author | Rosanna G Alderson |
  | Author | Luna De Ferrari |
  | Author | Lazaros Mavridis |
  | Author | James L McDonagh |
  | Author | John B O Mitchell |
  | Author | Neetika Nath |
  | Volume | 12 |
  | Issue | 17 |
  | Pages | 1911-1923 |
  | Publication | Current topics in medicinal chemistry |
  | ISSN | 1873-4294 |
  | Date | 2012 |
  | Extra | PMID: 23116471 |
  | Journal Abbr | Curr Top Med Chem |
  | Library Catalog | NCBI PubMed |
  | Language | eng |
  | Abstract | Over the last 50 years, sequencing, structural biology and bioinformatics have completely revolutionised biomolecular science, with millions of sequences and tens of thousands of three dimensional structures becoming available. The bioinformatics of enzymes is well served by, mostly free, online databases. BRENDA describes the chemistry, substrate specificity, kinetics, preparation and biological sources of enzymes, while KEGG is valuable for understanding enzymes and metabolic pathways. EzCatDB, SFLD and MACiE are key repositories for data on the chemical mechanisms by which enzymes operate. At the current rate of genome sequencing and manual annotation, human curation will never finish the functional annotation of the ever-expanding list of known enzymes. Hence there is an increasing need for automated annotation, though it is not yet widespread for enzyme data. In contrast, functional ontologies such as the Gene Ontology already profit from automation. Despite our growing understanding of enzyme structure and dynamics, we are only beginning to be able to design novel enzymes. One can now begin to trace the functional evolution of enzymes using phylogenetics. The ability of enzymes to perform secondary functions, albeit relatively inefficiently, gives clues as to how enzyme function evolves. Substrate promiscuity in enzymes is one example of imperfect specificity in protein-ligand interactions. Similarly, most drugs bind to more than one protein target. This may sometimes result in helpful polypharmacology as a drug modulates plural targets, but also often leads to adverse side-effects. Many chemoinformatics approaches can be used to model the interactions between druglike molecules and proteins in silico. We can even use quantum chemical techniques like DFT and QM/MM to compute the structural and energetic course of enzyme catalysed chemical reaction mechanisms, including a full description of bond making and breaking. |
  | Date Added | 2/20/2014, 12:24:01 PM |
  | Modified | 3/7/2014, 12:15:33 PM |

  ### Notes:

  - Review of bioinformatics in the study of enzyme function.

    How SCOP is used:

    Background on protein structure classification.

    SCOP/CATH reference:

    One way in which to achieve this is by the use of databases such as CATH [91] and SCOP [92], in which enzymes are clustered into homologous groups aided by the conservation of protein structure, which is thought to be more conserved than sequence. Recent studies which have utilised structure to aid inference of distant evolutionary relationships include the exploration of the strictosidine synthase-like proteins [93] and the ferritin-like superfamily [94].

    The FunTree online application, released in 2011, allows for the evolution of CATH defined superfamilies (at the ‘Homologous Superfamily’ level) to be explored [95, 96, 97]. The application uses structural alignments to define ‘structurally similar groups’ (SSGs) within the superfamily. Sequences within each SSG are aligned using a structurally informed method. The resulting alignment is then used to build phylogenetic trees that integrate data from the CSA [23, 24] and MACiE [31, 32] and allow for structural superimpositions of structures at each node to be visualised using Jmol [98]. Thus, the evolution of functions in diverse superfamilies can be seen in terms of changes in structure and active site residues.

    An application such as FunTree represents an interesting and exciting way to explore evolution. However, one only has to examine the ‘example’ phylogenetic trees on the FunTree homepage [97] to see that in many cases the bifurcations at some nodes, especially more ancient ones, show a low level of bootstrap support. This is unsurprising, given the low level of homologous signal so far back in evolutionary history. The problem of low signal to noise ratio in studies of highly divergent enzymes is on-going and seemingly unavoidable; it will be of much interest to see what strategies will be developed in the future to try and overcome this.

  ### Attachments

  - emss-50852.pdf
- ## Epitopic hexapeptide sequences from Baltic cod parvalbumin beta (allergen Gad c 1) are common in the universal proteome

  |  |  |
  | --- | --- |
  | Type | Journal Article |
  | Author | Piotr Minkiewicz |
  | Author | Justyna Bucholska |
  | Author | Malgorzata Darewicz |
  | Author | Justyna Borawska |
  | Volume | 38 |
  | Issue | 1 |
  | Pages | 105–109 |
  | Publication | Peptides |
  | Date | November 2012 |
  | DOI | 10.1016/j.peptides.2012.08.011 |
  | Abstract | The aim of this study was to analyze the distribution of hexapeptide fragments considered as epitopes of Baltic cod parvalbumin beta (allergen Gad c 1) in the universal proteome. Cod (Gadus morhua subsp. callarias) parvalbumin hexapeptides cataloged in the Immune Epitope Database were used as query sequences. The UniProt database was screened using the WU-BLAST 2 program. The distribution of hexapeptide fragments was investigated in various protein families, classified according to the presence of the appropriate domains, and in proteins of plant, animal and microbial species. Hexapeptides from cod parvalbumin were found in the proteins of plants and animals which are food sources, microorganisms with various applications in food technology and biotechnology, microorganisms which are human symbionts and commensals as well as human pathogens. In the last case possible coverage between epitopes from pathogens and allergens should be avoided during vaccine design. (C) 2012 Elsevier Inc. All rights reserved. |
  | Date Added | 3/7/2014, 12:08:00 PM |
  | Modified | 3/7/2014, 12:08:00 PM |
- ## eProS-a database and toolbox for investigating protein sequence-structure-function relationships through energy profiles

  |  |  |
  | --- | --- |
  | Type | Journal Article |
  | Author | Florian Heinke |
  | Author | Stefan Schildbach |
  | Author | Daniel Stockmann |
  | Author | Dirk Labudde |
  | Volume | 41 |
  | Issue | D1 |
  | Pages | D320-D326 |
  | Publication | Nucleic Acids Research |
  | ISSN | 0305-1048; 1362-4962 |
  | Date | JAN 2013 |
  | Extra | WOS:000312893300045 |
  | DOI | 10.1093/nar/gks1079 |
  | Abstract | Gaining information about structural and functional features of newly identified proteins is often a difficult task. This information is crucial for understanding sequence-structure-function relationships of target proteins and, thus, essential in comprehending the mechanisms and dynamics of the molecular systems of interest. Using protein energy profiles is a novel approach that can contribute in addressing such problems. An energy profile corresponds to the sequence of energy values that are derived from a coarse-grained energy model. Energy profiles can be computed from protein structures or predicted from sequences. As shown, correspondences and dissimilarities in energy profiles can be applied for investigations of protein mechanics and dynamics. We developed eProS (energy profile suite, freely available at http://bioservices.hs-mittweida.de/Epros/), a database that provides similar to 76 000 pre-calculated energy profiles as well as a toolbox for addressing numerous problems of structure biology. Energy profiles can be browsed, visualized, calculated from an uploaded structure or predicted from sequence. Furthermore, it is possible to align energy profiles of interest or compare them with all entries in the eProS database to identify significantly similar energy profiles and, thus, possibly relevant structural and functional relationships. Additionally, annotations and cross-links from numerous sources provide a broad view of potential biological correspondences. |
  | Date Added | 2/20/2014, 12:24:01 PM |
  | Modified | 3/7/2014, 12:09:39 PM |

  ### Notes:

  - eProS is a database that provides pre-calucated energy profiles from a course-grained energy model, computed from structures or predicted from sequence.

    How SCOP/CATH is used:

    Annotate data set with SCOP domain boundaries and full classification.

    Also annotate with CATH data, to support reverse annotation lookup.

    SCOP reference:

    Various sources of annotation [e.g. Gene Ontology (GO) (13), PDB, CATH (14), SCOP (15) and Pfam (16)] provide a wide view on structural and func- tional features of the best hits, which can be further broadened through the reverse annotation lookup provided by eProS.

  ### Attachments

  - Nucl. Acids Res.-2013-Heinke-D320-6.pdf
- ## eThread: A Highly Optimized Machine Learning-Based Approach to Meta-Threading and the Modeling of Protein Tertiary Structures

  |  |  |
  | --- | --- |
  | Type | Journal Article |
  | Author | Michal Brylinski |
  | Author | Daswanth Lingam |
  | URL | http://dx.plos.org/10.1371/journal.pone.0050200 |
  | Volume | 7 |
  | Issue | 11 |
  | Pages | e50200 |
  | Publication | PloS one |
  | Date | 2012 |
  | Accessed | 9/20/2013, 1:19:35 PM |
  | Library Catalog | Google Scholar |
  | Short Title | eThread |
  | Date Added | 10/11/2013, 10:29:15 AM |
  | Modified | 2/25/2014, 4:38:13 PM |

  ### Tags:

  - Algorithms
  - Artificial Intelligence
  - Bayes Theorem
  - Computer Simulation
  - Databases, Protein
  - Models, Molecular
  - Protein Folding
  - Proteins
  - Protein Structure, Tertiary
  - Sequence Alignment
  - Software
  - Structural Homology, Protein

  ### Notes:

  - Present  a new homology modeling method, called eThread.  Given an input amino acid sequence, the method searches for templates in two libraries: a full chain library and a domain library.

    How SCOP is used:

    Use SCOP domains in template library for threading. Use PISCES to compile domain data set from SCOP, removing redundancy.  Obtained structures from ASTRAL.

    SCOP reference:

    Threading Libraries

    Two threading libraries are used in this study: chain and domain. Chain library comprises aforementioned 11,468 protein chains selected from the PDB by PISCES [25]. Domain library was compiled by PISCES using the Structural Classification of Proteins (SCOP) database [26]. Similarly to the chain library, the redundancy was removed at 40% pairwise sequence identity. This library contains 10,013 representative protein domains 50–600 residues in length, for which the atomic coordinates were obtained from the ASTRAL database [27].

  ### Attachments

  - [HTML] from plos.org
  - journal.pone.0050200.pdf
  - PubMed entry
- ## Eukaryotic GPN-loop GTPases paralogs use a dimeric assembly reminiscent of archeal GPN

  |  |  |
  | --- | --- |
  | Type | Journal Article |
  | Author | Béatrice Alonso |
  | Author | Carole Beraud |
  | Author | Sarra Meguellati |
  | Author | Shu W. Chen |
  | Author | Jean Luc Pellequer |
  | Author | Jean Armengaud |
  | Author | Christian Godon |
  | URL | http://www.landesbioscience.com/journals/cc/article/23367/ |
  | Volume | 12 |
  | Issue | 3 |
  | Pages | 0–9 |
  | Publication | Cell Cycle |
  | Date | 2013 |
  | Accessed | 9/23/2013, 10:17:26 AM |
  | Library Catalog | Google Scholar |
  | Date Added | 10/11/2013, 10:29:15 AM |
  | Modified | 10/11/2013, 10:29:15 AM |

  ### Tags:

  - chromatid cohesion
  - GPN-loop-GTPase
  - heterodimer
  - Interesting
  - paralogous interactions
  - P-loop NTPase

  ### Notes:

  - Experimental and computational study of the function of GPN loop GTPase. The result suggests that it has a role in sister chromatid cohesion.

    How SCOP is used:

    The SCOP database was used to note the superfamily and class that the GPN GTPase belonged to. (Just looking it up in the database).

    Also compare GPN with a remote homolog, MinD, found in the same superfamily.

    **SCOP Reference:**

    The GPN GTPases belongs to the P-loop containing nucleoside triphosphate hydrolases superfamily (class of !/à proteins, SCOP database24). In this superfamily, the cell division regulator MinD appears in many ways similar to the GPNs structure. Despite a low sequence identity between MinD and PAB0955 that was estimated to be around 6% according to a structure-based alignment made with sup3d,20 it was observed that the core of MinD structure25 superimposes onto that of PAB0955 structure with a root-mean-square deviation of 1 Å for 79 core C! atoms (Fig. S4). MinD undergoes ATP-dependent dimerization in solution,26 and the crystal structure reveals that a conserved Glu126 residue is located at the dimer interface. This residue is located right next to the G3 box (Fig. S1) and corresponds to Glu107 in PAB0955 (Fig. S4). In conclusion, convergent evolu- tionary and molecular data strongly suggest that the interactions of GPN-GTPases paralogs are of heterodimeric nature in the cell.

  ### Attachments

  - cc-12-463.pdf
  - [HTML] from nih.gov
  - Snapshot
- ## Evaluation performance of substitution matrices, based on contacts between residue terminal groups

  |  |  |
  | --- | --- |
  | Type | Journal Article |
  | Author | Boris Vishnepolsky |
  | Author | Grigol Managadze |
  | Author | Maya Grigolava |
  | Author | Malak Pirtskhalava |
  | URL | http://www.tandfonline.com/doi/abs/10.1080/07391102.2012.677769 |
  | Volume | 30 |
  | Issue | 2 |
  | Pages | 180–190 |
  | Publication | Journal of Biomolecular Structure and Dynamics |
  | Date | 2012 |
  | Accessed | 9/23/2013, 10:24:40 AM |
  | Library Catalog | Google Scholar |
  | Date Added | 10/11/2013, 10:29:15 AM |
  | Modified | 3/7/2014, 1:06:55 PM |

  ### Tags:

  - alignment
  - contact potentials
  - fold recognition
  - protein structure prediction
  - twilight zone

  ### Notes:

  - Present method for constructing contact potentials (CPs) and substitutions matrices (SMs) for protein sequence alignment.

    How SCOP is used:

    Use ASTRAL sequence data to get sequences for SABmark twilight zone data set.  Get SCOP classification and use to evaluate alignment method on full data set and separately on family, superfamily, and fold level.

    SCOP reference:

    Benchmarks

    For obtaining CP and SM elements, we use FSSP2 library (Vishnepolsky, Managadze, & Pirtskhalava, 2008) which contains 637 structures selected from FSSP (Holm, Ouzounis, Sander, Tuparev, & Vriend, 1992) by using filter criterion – Dali z-score for structural similarity between set members < 2;

    The alignment accuracy is tested on Twilight Zone set of the 1.65 version of the SABmark reference align- ment database (VanWalle, Lasters, & Wyns, 2005), which contains single domain sequences with low sequence similarity. The sequences of the Twilight Zone set are taken from a SCOP (Murzin, Brenner, Hubbard, & Chothia, 1995) subset provided by the ASTRAL com- pendium, in which domains have a pairwise Blast E- value of at least 1, for a theoretical database size of 108 residues (Altschul et al., 1997; Chandonia et al., 2004). The Twilight Zone set contains 10,667 sequence pairs, based on 1740 sequences and joined into 209 folds.

    The alignment accuracy is tested both on full Twi- light Zone set and separately on family, superfamily, and fold level according SCOP classification.

  ### Attachments

  - 07391102%2E2012%2E677769.pdf
- ## Evidence theoretic protein fold classification based on the concept of hyperfold

  |  |  |
  | --- | --- |
  | Type | Journal Article |
  | Author | Kaveh Kavousi |
  | Author | Mehdi Sadeghi |
  | Author | Behzad Moshiri |
  | Author | Babak N. Araabi |
  | Author | Ali Akbar Moosavi-Movahedi |
  | Volume | 240 |
  | Issue | 2 |
  | Pages | 148-160 |
  | Publication | MATHEMATICAL BIOSCIENCES |
  | ISSN | 0025-5564 |
  | Date | December 2012 |
  | DOI | 10.1016/j.mbs.2012.07.001 |
  | Language | English |
  | Abstract | In current computational biology, assigning a protein domain to a fold class is a complicated and controversial task. It can be more challenging in the much harder task of correct identification of protein domain fold pattern solely through using extracted information from protein sequence. To deal with such a challenging problem, the concepts of hyperfold and interlaced folds are introduced for the first time. Each hyperfold is a set of interlaced folds with a centroid fold. These concepts are used to construct a framework for handling the uncertainty involved with the fold classification problem. In this approach, an unknown query protein is assigned to a hyperfold rather than a single fold. Ten different sequence based features are used to predicting the correct hyperfold. This architecture is featured by the Dempster-Shafer theory of evidence through the bodies of evidence and Dempster's rule of combination to combine the hyperfolds. The classification architecture thus developed was applied for identifying protein folds among the 27 famous SCOP fold patterns from a stringent well-known dataset. Compared with the existing predictors tested by the same benchmark dataset, our approach might achieve the better results. (C) 2012 Elsevier Inc. All rights reserved. |
  | Date Added | 10/11/2013, 10:29:15 AM |
  | Modified | 3/7/2014, 1:07:07 PM |

  ### Tags:

  - Hyperfold
  - Interlaced folds
  - Protein fold classification
  - Sequence based feature

  ### Notes:

  - Present novel method for fold prediction.

    How SCOP is used:

    Validate fold predictions against SCOP.  Use a domain dataset that was previously curated to have folds from the 27 most populated SCOP folds (with at least 7 members) from the first four SCOP classes.

    How CATH is used:

    Not using CATH data.

    SCOP reference:

    In abstract:

    The classification architecture thus developed was applied for identifying protein folds among the 27 famous SCOP fold patterns from a stringent well-known dataset.

    ...

    2.1. Dataset and sequence based feature vectors

    The main part of the dataset used for training and testing was selected from [10]. The complete list of protein domains can be freely obtained from http://www.csbio.sjtu.edu.cn/bioinf/PFP- FunDSeqE/. In training dataset each two protein domains have no more than 35% sequence identity for domains longer than 80 res- idues. The testing dataset contains the SCOP domains having less than 40% identity with each other. For building training and testing datasets, 27 folds from most populated SCOP folds (with at least seven members) from four major structural classes all a, all b, a + b, and a/b were utilized. None of the domains in the training and testing datasets had more than 35% sequence identity to any others and 90% of the proteins of the test dataset had less than 25% sequence identity with the proteins of the training dataset.

  ### Attachments

  - 1-s2.0-S0025556412001563-main.pdf
- ## EvoDesign: de novo protein design based on structural and evolutionary profiles

  |  |  |
  | --- | --- |
  | Type | Journal Article |
  | Author | Pralay Mitra |
  | Author | David Shultis |
  | Author | Yang Zhang |
  | Volume | 41 |
  | Issue | W1 |
  | Pages | W273-W280 |
  | Publication | Nucleic Acids Research |
  | ISSN | 0305-1048 |
  | Date | JUL 2013 |
  | Extra | WOS:000323603200043 |
  | DOI | 10.1093/nar/gkt384 |
  | Abstract | Protein design aims to identify new protein sequences of desirable structure and biological function. Most current de novo protein design methods rely on physics-based force fields to search for low free-energy states following Anfinsen's thermodynamic hypothesis. A major obstacle of such approaches is the inaccuracy of the force field design, which cannot accurately describe the atomic interactions or distinguish correct folds. We developed a new web server, EvoDesign, to design optimal protein sequences of given scaffolds along with multiple sequence and structure-based features to assess the foldability and goodness of the designs. EvoDesign uses an evolution-profile-based Monte Carlo search with the profiles constructed from homologous structure families in the Protein Data Bank. A set of local structure features, including secondary structure, torsion angle and solvation, are predicted by single-sequence neural-network training and used to smooth the sequence motif and accommodate the physicochemical packing. The EvoDesign algorithm has been extensively tested in large-scale protein design experiments, which demonstrate enhanced foldability and structural stability of designed sequences compared with the physics-based designing methods. The EvoDesign server is freely available at http://zhanglab.ccmb.med.umich.edu/EvoDesign. |
  | Date Added | 10/28/2013, 4:51:00 PM |
  | Modified | 10/28/2013, 4:51:00 PM |

  ### Notes:

  - EvoDesign is a  de novo protein sequence design web server.  Sequence space search is constrained by amino acid sequence profiles of homologous structure families.

    How SCOP is used:

    Benchmarked the accuracy and efficiency of EvoDesign with a data set of 7 non-homologous proteins of varied length and SCOP classes.

    SCOP reference:

    The overall computing time of the EvoDesign server depends on the length of the scaffold and the force field selected for simulation. To test the impact of the force field selections on the accuracy and efficiency of the EvoDesign server, we arbitrarily selected seven non-homologous proteins with varied length and different SCOP class (27). Sequences of the selected scaffolds are designed using the EvoDesign server without and with physics- based force field.

  ### Attachments

  - Nucl. Acids Res.-2013-Mitra-W273-80.pdf
- ## Evolutionarily consistent families in SCOP: sequence, structure and function

  |  |  |
  | --- | --- |
  | Type | Journal Article |
  | Author | Ralph Pethica |
  | Author | Michael Levitt |
  | Author | Julian Gough |
  | URL | http://www.biomedcentral.com/1472-6807/12/27 |
  | Volume | 12 |
  | Issue | 1 |
  | Pages | 27 |
  | Publication | BMC Structural Biology |
  | ISSN | 1472-6807 |
  | Date | 2012 |
  | DOI | 10.1186/1472-6807-12-27 |
  | Abstract | BACKGROUND:SCOP is a hierarchical domain classification system for proteins of known structure. The superfamily level has a clear definition: Protein domains belong to the same superfamily if there is structural, functional and sequence evidence for a common evolutionary ancestor. Superfamilies are sub-classified into families, however, there is not such a clear basis for the family level groupings. Do SCOP families group together domains with sequence similarity, do they group domains with similar structure or by common function? It is these questions we answer, but most importantly, whether each family represents a distinct phylogenetic group within a superfamily.RESULTS:Several phylogenetic trees were generated for each superfamily: one derived from a multiple sequence alignment, one based on structural distances, and the final two from presence/absence of GO terms or EC numbers assigned to domains. The topologies of the resulting trees and confidence values were compared to the SCOP family classification.CONCLUSIONS:We show that SCOP family groupings are evolutionarily consistent to a very high degree with respect to classical sequence phylogenetics. The trees built from (automatically generated) structural distances correlate well, but are not always consistent with SCOP (hand annotated) groupings. Trees derived from functional data are less consistent with the family level than those from structure or sequence, though the majority still agree. Much of GO and EC annotation applies directly to one family or subset of the family; relatively few terms apply at the superfamily level. Maximum sequence diversity within a family is on average 22% but close to zero for superfamilies. |
  | Date Added | 10/11/2013, 10:17:54 AM |
  | Modified | 3/7/2014, 1:06:59 PM |

  ### Tags:

  - Interesting

  ### Notes:

  - Compared phylogenetic trees with SCOP hierarchy to find whether families in SCOP are 'consistent'.  Found SCOP families are highly evolutionary consistent.

    Generated 3 phylogenetic trees for each superfamily based on:

    1. multiple sequence alignment

    2. structural distances

    3. presence/absence of GO terms and EC numbers

     Found that GO and EC terms apply to family or a subset of a family, but generally do not apply at the superfamily level.

     How SCOP is used:

    Create phylogenetic trees for each SCOP superfamily and compare SCOP families with phylogenies. Use ASTRAL 1.73 95% sequences and structures.  Also use entire ASTRAL 1.73 sequence data set to compute sequence divergence for each superfamily.

    SCOP references:

    From abstract:

    Several phylogenetic trees were generated for each superfamily: one derived from a multiple sequence alignment, one based on structural distances, and the final two from presence/absence of GO terms or EC numbers assigned to domains. The topologies of the resulting trees and confidence values were compared to the SCOP family classification.

  ### Attachments

  - evolutionary-consistent.pdf
  - [HTML] from biomedcentral.com
- ## Evolutionary conservation of the polyproline II conformation surrounding intrinsically disordered phosphorylation sites

  |  |  |
  | --- | --- |
  | Type | Journal Article |
  | Author | W. Austin Elam |
  | Author | Travis P. Schrank |
  | Author | Andrew J. Campagnolo |
  | Author | Vincent J. Hilser |
  | URL | http://onlinelibrary.wiley.com/doi/10.1002/pro.2217/full |
  | Publication | Protein Science |
  | Date | 2013 |
  | Accessed | 9/20/2013, 1:20:18 PM |
  | Library Catalog | Google Scholar |
  | Date Added | 10/11/2013, 10:29:15 AM |
  | Modified | 3/7/2014, 12:09:55 PM |

  ### Tags:

  - Amino Acids
  - Amino Acid Sequence
  - Animals
  - Computer Simulation
  - Fungal Proteins
  - gene ontology
  - Humans
  - intrinsically disordered
  - Mice
  - Models, Molecular
  - Molecular Sequence Data
  - Peptides
  - Phosphoproteins
  - phosphorylation
  - Phosphorylation
  - polyproline II
  - Protein Binding
  - Protein Conformation
  - Protein Folding
  - Proteome
  - proteome

  ### Notes:

  - Study of intrinsically disordered proteins and propensity to take on the polyproline II (PII) conformation.

    How SCOP is used:

    Used several datasets in their analysis from different genomes.  One was a nonredundant set of human protein sequences extracted from the PDB consisting of proteins from each SCOP family.  Calculated PII propensity across data set and compared with other genomes.

    SCOP reference:

    Calculation of PII propensity in sequences and in silico evolution of PII propensity  
     Several protein sequence datasets39,40,52 were employed for our analysis. Several protein sequence datasets were employed for the analysis of the PII content of the proteome, including a nonredundant set of human protein sequences extracted from the PDB39 consisting of proteins from each SCOP fam- ily,57 an ID protein dataset DisProt 5.5,40 and the complete proteomes of six eukaryotes—H. sapiens (human), M. musculus (mouse), D. melanogaster (fly), C. elegans (worm), A. thaliana (plant), S. cere- visiae (yeast) obtained from the Integr8 project.52 Algorithms for calculating the PII propensities of amino acid sequences were written in Cþþ and Python, with additional data processing in Perl, the R Project, and Microsoft Excel.

  ### Attachments

  - 2217\_ftp.pdf
  - PubMed entry
- ## Evolutionary Dynamics on Protein Bi-stability Landscapes Can Potentially Resolve Adaptive Conflicts

  |  |  |
  | --- | --- |
  | Type | Journal Article |
  | Author | Tobias Sikosek |
  | Author | Erich Bornberg-Bauer |
  | Author | Hue Sun Chan |
  | Volume | 8 |
  | Issue | 9 |
  | Pages | e1002659 |
  | Publication | Plos Computational Biology |
  | Date | September 2012 |
  | DOI | 10.1371/journal.pcbi.1002659 |
  | Abstract | Experimental studies have shown that some proteins exist in two alternative native-state conformations. It has been proposed that such bi-stable proteins can potentially function as evolutionary bridges at the interface between two neutral networks of protein sequences that fold uniquely into the two different native conformations. Under adaptive conflict scenarios, bi-stable proteins may be of particular advantage if they simultaneously provide two beneficial biological functions. However, computational models that simulate protein structure evolution do not yet recognize the importance of bi-stability. Here we use a biophysical model to analyze sequence space to identify bi-stable or multi-stable proteins with two or more equally stable native-state structures. The inclusion of such proteins enhances phenotype connectivity between neutral networks in sequence space. Consideration of the sequence space neighborhood of bridge proteins revealed that bi-stability decreases gradually with each mutation that takes the sequence further away from an exactly bi-stable protein. With relaxed selection pressures, we found that bi-stable proteins in our model are highly successful under simulated adaptive conflict. Inspired by these model predictions, we developed a method to identify real proteins in the PDB with bridge-like properties, and have verified a clear bi-stability gradient for a series of mutants studied by Alexander et al. (Proc Nat Acad Sci USA 2009, 106: 21149-21154) that connect two sequences that fold uniquely into two different native structures via a bridge-like intermediate mutant sequence. Based on these findings, new testable predictions for future studies on protein bi-stability and evolution are discussed. |
  | Date Added | 3/7/2014, 12:08:00 PM |
  | Modified | 3/7/2014, 12:08:00 PM |

  ### Notes:

  - Study of proteins with more than one stable conformation and the mechanisms involved.

    How CATH is used:

    Use a database of multiple confirmations of proteins that contains only single domain proteins as annotated by CATH.

     Do not cite SCOP.

  ### Attachments

  - journal.pcbi.1002659.pdf
- ## Evolutionary history of the TBP-domain superfamily

  |  |  |
  | --- | --- |
  | Type | Journal Article |
  | Author | Bjoern Brindefalk |
  | Author | Benoit H. Dessailly |
  | Author | Corin Yeats |
  | Author | Christine Orengo |
  | Author | Finn Werner |
  | Author | Anthony M. Poole |
  | Volume | 41 |
  | Issue | 5 |
  | Pages | 2832-2845 |
  | Publication | Nucleic Acids Research |
  | ISSN | 0305-1048 |
  | Date | MAR 2013 |
  | Extra | WOS:000318062600012 |
  | DOI | 10.1093/nar/gkt045 |
  | Abstract | The TATA binding protein (TBP) is an essential transcription initiation factor in Archaea and Eucarya. Bacteria lack TBP, and instead use sigma factors for transcription initiation. TBP has a symmetric structure comprising two repeated TBP domains. Using sequence, structural and phylogenetic analyses, we examine the distribution and evolutionary history of the TBP domain, a member of the helix-grip fold family. Our analyses reveal a broader distribution than for TBP, with TBP-domains being present across all three domains of life. In contrast to TBP, all other characterized examples of the TBP domain are present as single copies, primarily within multidomain proteins. The presence of the TBP domain in the ubiquitous DNA glycosylases suggests that this fold traces back to the ancestor of all three domains of life. The TBP domain is also found in RNase HIII, and phylogenetic analyses show that RNase HIII has evolved from bacterial RNase HII via TBP-domain fusion. Finally, our comparative genomic screens confirm and extend earlier reports of proteins consisting of a single TBP domain among some Archaea. These monopartite TBP-domain proteins suggest that this domain is functional in its own right, and that the TBP domain could have first evolved as an independent protein, which was later recruited in different contexts. |
  | Date Added | 2/20/2014, 12:24:01 PM |
  | Modified | 3/7/2014, 12:10:14 PM |

  ### Notes:

  - Study evolutionary history of TATA binding protein (TBP-domain) superfamily.

    How SCOP is used:

    Look up SCOP superfamily clasification for TBP, DNA glycosylase, and RNase HIII in order to determine whether these share a common ancestor.  Found TBP and DNA glycosylase share a SCOP superfamily and Pfam clan.

    All three share a CATH fold.

    SCOP/CATH reference:

    MATERIALS AND METHODS

    Assessing remote homologies using sequence analysis

    To evaluate remote homologies between the TBP domains in TBP, DNA glycosylase and RNase HIII, we searched existing domain family classifications—SCOP (23), CATH-Gene3D (24) and Pfam (25). We also used sensi- tive sequence-based search methods, i.e. the FFAS server (26) and the HHpred server (27), to detect very remote homologies not captured in SCOP, CATH or Pfam. Structural comparisons were also performed and are described later in the text.

    SCOP, CATH and Gene3D

    The CATH (24) and SCOP (23) databases classify hom- ologous protein domain structures in superfamilies on the basis of structural, sequence and functional similarities.

    Gene3D is a sister resource of CATH containing sequence relatives for each domain structure superfamily. Hidden Markov Models (HMMs) are built using HMMer for representative sequences from each CATH domain structure family and used to scan UniProt and ENSEMBL to identify sequence relatives. We searched SCOP, CATH and Gene3D for all protein sequences con- taining TBP domains.

    ...

    First, we examined evidence of homology of TBP domains in existing databases of protein classification. **TBP domains from TBP and DNA glycosylase belong to the same superfamily in SCOP and to the same Clan (CL0407) in Pfam.**

  ### Attachments

  - Nucl. Acids Res.-2013-Brindefalk-2832-45.pdf
- ## Evolutionary inaccuracy of pairwise structural alignments

  |  |  |
  | --- | --- |
  | Type | Journal Article |
  | Author | M. I. Sadowski |
  | Author | W. R. Taylor |
  | Volume | 28 |
  | Issue | 9 |
  | Pages | 1209-1215 |
  | Publication | BIOINFORMATICS |
  | ISSN | 1367-4803 |
  | Date | MAY 1 2012 |
  | DOI | 10.1093/bioinformatics/bts103 |
  | Language | English |
  | Abstract | Motivation: Structural alignment methods are widely used to generate gold standard alignments for improving multiple sequence alignments and transferring functional annotations, as well as for assigning structural distances between proteins. However, the correctness of the alignments generated by these methods is difficult to assess objectively since little is known about the exact evolutionary history of most proteins. Since homology is an equivalence relation, an upper bound on alignment quality can be found by assessing the consistency of alignments. Measuring the consistency of current methods of structure alignment and determining the causes of inconsistencies can, therefore, provide information on the quality of current methods and suggest possibilities for further improvement. Results: We analyze the self-consistency of seven widely-used structural alignment methods (SAP, TM-align, Fr-TM-align, MAMMOTH, DALI, CE and FATCAT) on a diverse, non-redundant set of 1863 domains from the SCOP database and demonstrate that even for relatively similar proteins the degree of inconsistency of the alignments on a residue level is high (30%). We further show that levels of consistency vary substantially between methods, with two methods (SAP and Fr-TM-align) producing more consistent alignments than the rest. Inconsistency is found to be higher near gaps and for proteins of low structural complexity, as well as for helices. The ability of the methods to identify good structural alignments is also assessed using geometric measures, for which FATCAT (flexible mode) is found to be the best performer despite being highly inconsistent. We conclude that there is substantial scope for improving the consistency of structural alignment methods. |
  | Date Added | 10/25/2013, 4:29:01 PM |
  | Modified | 3/7/2014, 12:11:14 PM |

  ### Tags:

  - ASTRAL domain structures
  - ASTRAL subsets
  - Cite ASTRAL

  ### Notes:

  - Evaluated consistency of different structure alignment tools.

    How SCOP is used:

    Benchmark methods. Compare quality of scoring implemented by the methods with respect to SCOP folds, among other external annotations.

    Used ASTRAL subset of domain structures.  SCOP 1.73, Astral Scop 10.

    Used SCOP fold-level classification to evaluate consistency of the methods' scoring.  Some methods have multiple scores, while others only produce RMSD and alignment length.  Therefore, measured the rate at which score predicted that two structures were in the same SCOP fold.

    SCOP reference:

    Under Abstract:

    Results: We analyze the self-consistency of seven widely-used structural alignment methods (SAP, TM-align, Fr-TM-align, MAMMOTH, DALI, CE and FATCAT) on a diverse, non-redundant set of 1863 domains from the SCOP database and demonstrate that even for relatively similar proteins the degree of inconsistency of the alignments on a residue level is high (30%).

    Under Methods

    A set of 1863 domains was derived from the ASTRAL SCOP10 database (SCOP version 1.73; Brenner et al., 2000). The set was restricted to high quality structures by requiring a SPACI score >0.5 (roughly equivalent to requiring at least 2Å resolution) and excluding NMR structures and those with missing residues.

    We then compared the fTM scores with the methods own summary scores to determine which was likely to provide the best ranking. As a measure of correctness in ranking, we determined how well each score correctly identified pairs with the same SCOP fold in the SCOP10 dataset using a ROC statistic. The mean area under the ROC curve (AUC) was determined up to a 5% false positive rate for ten 50% partitions of the data in a bootstrap analysis to allow the significance of any differences to be assessed.

  ### Attachments

  - Full Text PDF
  - PubMed entry
  - Snapshot
- ## Evolutionary optimization of protein folding

  |  |  |
  | --- | --- |
  | Type | Journal Article |
  | Author | Cédric Debès |
  | Author | Minglei Wang |
  | Author | Gustavo Caetano-Anollés |
  | Author | Frauke Gräter |
  | URL | http://dx.plos.org/10.1371/journal.pcbi.1002861 |
  | Volume | 9 |
  | Issue | 1 |
  | Pages | e1002861 |
  | Publication | PLoS computational biology |
  | Date | 2013 |
  | Accessed | 9/20/2013, 1:17:33 PM |
  | Library Catalog | Google Scholar |
  | Date Added | 10/11/2013, 10:29:15 AM |
  | Modified | 10/11/2013, 10:29:15 AM |

  ### Tags:

  - ASTRAL
  - ASTRAL domain structures
  - Interesting

  ### Notes:

  - Study whether proteins have evolved to have shorter folding times. Using phylogenomic and structural analyses, observe an overall decrease in folding times between \*3.8 and \*1.5 billion years ago, which can be interpreted as an evolutionary optimization for rapid folding.

    How SCOP is used:

    Two uses of SCOP data:

    First, build  phylogenetic trees of SCOP domains from 1.73, including those not in the first 7 classes.   Used all ASTRAL data from that version.  Used trees to predict ages of families.

    Second, also build a phylogenetic tree using the same ASTRAL data.  Not sure why they didn't use the earlier tree.  Calculated the average SMCO for each family and superfamily.

    SCOP reference:

    Introduction:

    Specifically, phylogenomic trees that describe the history of the protein world are built from a genomic census of known protein domains defined by the Structural Classification of Proteins (SCOP) [14] and used to build timelines of domain appearance [15,16] that obey a molecular clock [17]

    Results:

    To trace protein folding in evolution, we determined the SMCO of protein domain structures at the Family (F) level of structural organization. Figure 2a shows the folding rate of each F, as measured by its average SMCO, as a function of evolutionary time. Using polynomial regression, we observed a significant decrease (p- value = 9.5e-15) in SMCO in proteins appearing between \*3.8 and \*1.5 billion years ago (Gya). Trends were maintained when excluding domains from the analysis solved in multi-domain proteins (Figure S11), and also when studying domain evolution at more or less conserved levels of structural abstraction of the SCOP hierarchy. Namely, we find a significant decrease of SMCO at the level of Superfamily (SF), p-value = 2.6e-15), and at the level of domains with less than 95% sequence identity (p-value, = 2.0e- 16, Figure S1a,b). Similarly, consistent results were obtained at the F level using linear regression (p-value = 1.0e-06, Figure S1c). Remarkably, even within a smaller data set of only 87 proteins for which folding times have been measured [24], we find that the experimental folding times exhibit a tendency to decrease early in protein evolution (Figure S2). As an additional way of validation, we repeated the analysis for \*3 million single domain sequences with predicted SMCO [25], and obtained a decrease again of SMCO up to \*1.5 Gya (p-value, = 2.0e-16, Figures S3, S4). Thus, in this initial evolutionary period, proteins tended to fold faster on average.

    ...

    We note that the resulting average chain length of three-dimensional structures in SCOP, which have been obtained from X-ray or NMR measurements, is smaller than the average length of sequences in genomes [30], apparently due to the increasing experimental difficulties when working with large proteins.

    Materials and Methods

    Phylogenomic tree

    A most parsimonious phylogenomic tree of F domain structures was reconstructed from a structural genomic census of 3,513 Fs (defined according to SCOP 1.73) in the proteomes of 989 organisms (76 Archaea, 656 Bacteria and 257 Eukarya) with genomes that have been completely sequenced [49]. Similarly, a most parsimonious phylogenomic tree of SF structures (860,497 steps; CI = 0.0255, HI = 0.9745, RI = 0.780, RC = 0.020; g1 = 20.109) was derived from a structural genomic census of 1,915 SFs (defined according to SCOP 1.73) in the proteomes of 1,096 organisms (78 Archaea, 719 Bacteria and 299 Eukarya)

    Survey of Size Modified Contact Order

    As a measure for the folding time of each protein architecture, we evaluated the size modified contact order (SMCO) of domains indexed in the SCOP database. We used the ASTRAL repositories to download the 92,470 three-dimensional structures classified in SCOP 1.73. The phylogenomic tree was built at the F level on the basis of the same protein structures, i.e. the 1.73 SCOP version. We note that the SMCO calculations are based on single protein domains from SCOP, while many proteins consist of multiple domains. Some studies showed that interactions between domains might affect folding [52]. To test if the evolutionary trends also hold for the subset of domains excluding those which have been structurally solved in multi-domain proteins, we carried out the following steps. We first downloaded the CathDomainList from the website of CATH (http://www.cathdb.info/download), and removed the PDB chains with two or more CATH domains or NMR structures or obsolete PDB entries. We then eliminated redundancy using the PISCES webserver (http://dunbrack.fccc. edu/PISCES.php) [53] using the following cut-offs: Sequence percentage identity: ,=25%, resolution: 0.0 3.0, R-factor: 0.3, sequence length: 40 10,000, Non X-ray entries: excluded, Ca-only entries: excluded, cull PDB by chain. We detected SCOP families using HMMs on the PDB chains and removed chains with long non-domain segments, i.e. the length of a segments without any domain assignment should be less than 30. Finally, we removed the chains with two or more SCOP families and the chains with two or more CATH entries. Using this dataset, we revealed the same tendencies in SMCO (Figure S11) as those of the whole dataset (compare Figure 2).

  ### Attachments

  - [HTML] from plos.org
  - journal.pcbi.1002861.pdf
- ## Evolution at the subgene level: domain rearrangements in the drosophila phylogeny

  |  |  |
  | --- | --- |
  | Type | Journal Article |
  | Author | Yi-Chieh Wu |
  | Author | Matthew D. Rasmussen |
  | Author | Manolis Kellis |
  | URL | http://mbe.oxfordjournals.org/content/29/2/689.short |
  | Volume | 29 |
  | Issue | 2 |
  | Pages | 689–705 |
  | Publication | Molecular Biology and Evolution |
  | Date | 2012 |
  | Accessed | 9/20/2013, 1:16:24 PM |
  | Library Catalog | Google Scholar |
  | Short Title | Evolution at the subgene level |
  | Date Added | 10/11/2013, 10:29:15 AM |
  | Modified | 12/2/2013, 4:23:50 PM |

  ### Notes:

  - Develop a computational model and algorithm for reconstructing gene evolution at the domain level.  Method deetects homlogous domains between genes and reconstructs evolutionary history: domain generation, duplication, loss, merge, and split events.

    How SCOP is used:

    Not using SCOP.

    Mention that alternative methods rely on domain definitions from databases such as SCOP, Pfam, InterPro, or CDD.

    SCOP reference:

    More recently, phylogenomic methods have been devel- oped to handle gene fusion and fission events or domain evolution, with initial approaches discovering domains de novo through sequence similarity (Snel et al. 2000) and later methods shifting to rely on underlying domain models us- ing databases such as InterPro (Hunter et al. 2009), Pfam (Bateman et al. 2002), SCOP (Murzin et al. 1995), SMART (Schultz et al. 1998), and CDD (Marchler-Bauer et al. 2005).

  ### Attachments

  - Mol Biol Evol-2012-Wu-689-705.pdf
- ## Evolution of Bcl-2 homology motifs: homology versus homoplasy

  |  |  |
  | --- | --- |
  | Type | Journal Article |
  | Author | Abdel Aouacheria |
  | Author | Valentine Rech de Laval |
  | Author | Christophe Combet |
  | Author | J. Marie Hardwick |
  | Volume | 23 |
  | Issue | 3 |
  | Pages | 103–111 |
  | Publication | Trends In Cell Biology |
  | Date | March 2013 |
  | DOI | 10.1016/j.tcb.2012.10.010 |
  | Abstract | Bcl-2 family proteins regulate apoptosis in animals. This protein family includes several homologous proteins and a collection of other proteins lacking sequence similarity except for a Bcl-2 homology (BH)3 motif. Thus, membership in the Bcl-2 family requires only one of the four BH motifs. On this basis, a growing number of diverse BH3-only proteins are being reported. Although compelling cell biological and biophysical evidence validates many BH3-only proteins, claims of significant BH3 sequence similarity are often unfounded. Computational and phylogenetic analyses suggest that only some BH3 motifs arose by divergent evolution from a common ancestor (homology), whereas others arose by convergent evolution or random coincidence (homoplasy), challenging current assumptions about which proteins constitute the extended Bcl-2 family. |
  | Date Added | 3/7/2014, 1:06:24 PM |
  | Modified | 3/7/2014, 1:06:24 PM |
- ## Evolution of cytochrome bc complexes: From membrane-anchored dehydrogenases of ancient bacteria to triggers of apoptosis in vertebrates

  |  |  |
  | --- | --- |
  | Type | Journal Article |
  | Author | Daria V. Dibrova |
  | Author | Dmitry A. Cherepanov |
  | Author | Michael Y. Galperin |
  | Author | Vladimir P. Skulachev |
  | Author | Armen Y. Mulkidjanian |
  | URL | http://www.sciencedirect.com/science/article/pii/S0005272813001230 |
  | Volume | 1827 |
  | Issue | 11–12 |
  | Pages | 1407-1427 |
  | Publication | Biochimica et Biophysica Acta (BBA) - Bioenergetics |
  | ISSN | 0005-2728 |
  | Date | November 2013 |
  | Journal Abbr | Biochimica et Biophysica Acta (BBA) - Bioenergetics |
  | DOI | 10.1016/j.bbabio.2013.07.006 |
  | Accessed | 9/20/2013, 11:01:16 AM |
  | Library Catalog | ScienceDirect |
  | Abstract | Abstract This review traces the evolution of the cytochrome bc complexes from their early spread among prokaryotic lineages and up to the mitochondrial cytochrome bc1 complex (complex III) and its role in apoptosis. The results of phylogenomic analysis suggest that the bacterial cytochrome b6f-type complexes with short cytochromes b were the ancient form that preceded in evolution the cytochrome bc1-type complexes with long cytochromes b. The common ancestor of the b6f-type and the bc1-type complexes probably resembled the b6f-type complexes found in Heliobacteriaceae and in some Planctomycetes. Lateral transfers of cytochrome bc operons could account for the several instances of acquisition of different types of bacterial cytochrome bc complexes by archaea. The gradual oxygenation of the atmosphere could be the key evolutionary factor that has driven further divergence and spread of the cytochrome bc complexes. On the one hand, oxygen could be used as a very efficient terminal electron acceptor. On the other hand, auto-oxidation of the components of the bc complex results in the generation of reactive oxygen species (ROS), which necessitated diverse adaptations of the b6f-type and bc1-type complexes, as well as other, functionally coupled proteins. A detailed scenario of the gradual involvement of the cardiolipin-containing mitochondrial cytochrome bc1 complex into the intrinsic apoptotic pathway is proposed, where the functioning of the complex as an apoptotic trigger is viewed as a way to accelerate the elimination of the cells with irreparably damaged, ROS-producing mitochondria. This article is part of a Special Issue entitled: Respiratory complex III and related bc complexes. |
  | Short Title | Evolution of cytochrome bc complexes |
  | Date Added | 2/20/2014, 12:24:01 PM |
  | Modified | 2/20/2014, 12:24:01 PM |

  ### Tags:

  - Bioenergetics, molecular evolution, ubiquinol:cytochrome c oxidoreductase
  - cardiolipin, cell death, photosynthesis, apoptosome
  - cytochrome c
  - plastoquinone
  - ubiquinone

  ### Notes:

  - Review of studies of evolution of cytochrome BC complexes.

    How SCOP is used:

    Look up fold and superfamily classification for protein of interest.

    SCOP reference:

    "In the SCOP database [96], the fold “heme-binding  
    four-helical bundle” comprises three superfamilies; the four-helix cytochrome b of cytochrome bc complex, together with the membrane cytochrome of the formate dehydrogenase makes the superfamily of  
    “transmembrane di-heme cytochromes”.

  ### Attachments

  - ScienceDirect Full Text PDF
- ## Evolution of oligomeric state through geometric coupling of protein interfaces

  |  |  |
  | --- | --- |
  | Type | Journal Article |
  | Author | Tina Perica |
  | Author | Cyrus Chothia |
  | Author | Sarah A. Teichmann |
  | URL | http://www.pnas.org/content/109/21/8127 |
  | Volume | 109 |
  | Issue | 21 |
  | Pages | 8127-8132 |
  | Publication | Proceedings of the National Academy of Sciences |
  | ISSN | 0027-8424, 1091-6490 |
  | Date | 05/22/2012 |
  | Extra | PMID: 22566652 |
  | Journal Abbr | PNAS |
  | DOI | 10.1073/pnas.1120028109 |
  | Accessed | 9/20/2013, 12:45:48 PM |
  | Library Catalog | www.pnas.org |
  | Language | en |
  | Abstract | Oligomerization plays an important role in the function of many proteins. Thus, understanding, predicting, and, ultimately, engineering oligomerization presents a long-standing interest. From the perspective of structural biology, protein–protein interactions have mainly been analyzed in terms of the biophysical nature and evolution of protein interfaces. Here, our aim is to quantify the importance of the larger structural context of protein interfaces in protein interaction evolution. Specifically, we ask to what extent intersubunit geometry affects oligomerization state. We define a set of structural parameters describing the overall geometry and relative positions of interfaces of homomeric complexes with different oligomeric states. This allows us to quantify the contribution of direct sequence changes in interfaces versus indirect changes outside the interface that affect intersubunit geometry. We find that such indirect, or allosteric mutations affecting intersubunit geometry via indirect mechanisms are as important as interface sequence changes for evolution of oligomeric states. |
  | Date Added | 10/11/2013, 10:29:15 AM |
  | Modified | 10/11/2013, 10:29:15 AM |

  ### Notes:

  - Study study how evolutionary changes in the geometry of complex interfaces effects oligomerization state.

    Found that the mutations which bring about geometric changes are just as likely to be outside the interface area.

    How SCOP is used:

    Filtered SCOP data, at the family-level, by extra criteria (described in excerpt below), using the 3DComplex database.  Resulted in 10 SCOP families.

    SCOP reference:

    We analyzed 10 SCOP (25) protein families, which, according to the 3DComplex database (22), have at least one dimer and one homologous tetramer or hexamer with the same dimeric binding mode and sequence identity higher than 40%.

    About multiple crystal structures:

    throughout this work, we calculate geometric variation between homologues and compare it to the variation between multiple crystal structures of the same protein wherever possible.  This allows us to distinguish geometric variation that corresponds to functional allosteric changes or simply flexibility of a protein, from genuine variation in evolution across homologues

  ### Attachments

  - Full Text PDF
- ## Evolution of Specific Protein-Protein Interaction Sites Following Gene Duplication

  |  |  |
  | --- | --- |
  | Type | Journal Article |
  | Author | Daniel Aiello |
  | Author | Daniel R. Caffrey |
  | Volume | 423 |
  | Issue | 2 |
  | Pages | 257-272 |
  | Publication | Journal of Molecular Biology |
  | Date | OCT 19 2012 |
  | Extra | WOS:000309784000010 |
  | DOI | 10.1016/j.jmb.2012.06.039 |
  | Library Catalog | ISI Web of Knowledge |
  | Abstract | Gene duplication is a common evolutionary process that leads to the expansion and functional diversification of protein subfamilies. The evolutionary events that cause paralogous proteins to bind different protein ligands (functionally diverged interfaces) are investigated and compared to paralogous proteins that bind the same protein ligand (functionally preserved interfaces). We find that functionally diverged interfaces possess more subfamily-specific residues than functionally preserved interfaces. These subfamily-specific residues are usually partially buried at the interface rim and achieve specific binding through optimized hydrogen bond geometries. In addition to optimized hydrogen bond geometries, side-chain modeling experiments suggest that steric effects are also important for binding specificity. Residues that are completely buried at the interface hub are also less conserved in functionally diverged interfaces than in functionally preserved interfaces. Consistent with this finding, hub residues contribute less to free energy of binding in functionally diverged interfaces than in functionally preserved interfaces. Therefore, we propose that protein binding is a delicate balance between binding affinity that primarily occurs at the interface hub and binding specificity that primarily occurs at the interface rim. (C) 2012 Elsevier Ltd. All rights reserved. |
  | Date Added | 10/8/2014, 12:47:53 PM |
  | Modified | 10/8/2014, 1:33:26 PM |

  ### Tags:

  - binding
  - interfaces
  - paralogs
  - protein–protein
  - specificity

  ### Attachments

  - ScienceDirect Full Text PDF
  - ScienceDirect Snapshot
- ## Exhaustive comparison and classification of ligand-binding surfaces in proteins

  |  |  |
  | --- | --- |
  | Type | Journal Article |
  | Author | Yoichi Murakami |
  | Author | Kengo Kinoshita |
  | Author | Akira R. Kinjo |
  | Author | Haruki Nakamura |
  | Volume | 22 |
  | Issue | 10 |
  | Pages | 1379-1391 |
  | Publication | Protein Science |
  | ISSN | 0961-8368; 1469-896X |
  | Date | OCT 2013 |
  | Extra | WOS:000325087000009 |
  | DOI | 10.1002/pro.2329 |
  | Abstract | Many proteins function by interacting with other small molecules (ligands). Identification of ligand-binding sites (LBS) in proteins can therefore help to infer their molecular functions. A comprehensive comparison among local structures of LBSs was previously performed, in order to understand their relationships and to classify their structural motifs. However, similar exhaustive comparison among local surfaces of LBSs (patches) has never been performed, due to computational complexity. To enhance our understanding of LBSs, it is worth performing such comparisons among patches and classifying them based on similarities of their surface configurations and electrostatic potentials. In this study, we first developed a rapid method to compare two patches. We then clustered patches corresponding to the same PDB chemical component identifier for a ligand, and selected a representative patch from each cluster. We subsequently exhaustively as compared the representative patches and clustered them using similarity score, PatSim. Finally, the resultant PatSim scores were compared with similarities of atomic structures of the LBSs and those of the ligand-binding protein sequences and functions. Consequently, we classified the patches into approximate to 2000 well-characterized clusters. We found that about 63% of these clusters are used in identical protein folds, although about 25% of the clusters are conserved in distantly related proteins and even in proteins with cross-fold similarity. Furthermore, we showed that patches with higher PatSim score have potential to be involved in similar biological processes. |
  | Date Added | 2/12/2014, 1:36:22 PM |
  | Modified | 3/7/2014, 12:09:09 PM |

  ### Notes:

  - Perform computational study to compare local surfaces of ligand-binding sites.

    How SCOP is used:

    Perform comparison of binding site geometries.  Classified the data set of chains with ligand-binding sites by SCOP class, fold, superfamily, and family.  Clustered the ligand-binding sites by similarity.

    How CATH is used:

    Perform same analysis with CATH for comparison.

    SCOP reference:

    n comparison with this pre- vious report, the ratio of the clusters classified in the same SCOP levels was smaller at surface level; that is, about 63% (1,846/2,949 clusters; psize ⬚⬚200 A ̊ 2) of the clusters were classified into these levels.

    ...

    Assignment of SCOP codes to each patch

    Protein structures are hierarchically classified into class, fold, superfamily, and family in SCOP. In this study, we only considered seven classes: (a) all-a, (b) all-b, (c) a/b (parallel b sheet; b-a-b units), (d) a1b (antiparallel b sheets; segregated a1b regions), (e) multidomain, (f) membrane and cell surface proteins and peptides, and (g) small proteins, except for (h) coiled coil proteins, (i) low resolution protein struc- tures, (j) peptides, or (k) designed proteins. A SCOP parseable file (version 1.75) was used for the assign- ment of a SCOP code(s) to each protein. For a patch extracted from multiple chains, SCOP code for a chain sharing the largest interface with a ligand was used.

    CATH reference:

    The same analysis of patch clustering was car- ried out with CATH,49 which semiautomatically clas- sifies protein domains to hierarchical groups. Consequently, overall results were not largely differ- ent from those with SCOP. In Table I, analysis with CATH topologies is also shown (see detailed analysis in the Supporting Information 4).

  ### Attachments

  - pro2329.pdf
- ## Exploring Angular Distance in Protein-Protein Docking Algorithms

  |  |  |
  | --- | --- |
  | Type | Journal Article |
  | Author | Thom Vreven |
  | Author | Howook Hwang |
  | Author | Zhiping Weng |
  | URL | http://dx.plos.org/10.1371/journal.pone.0056645 |
  | Volume | 8 |
  | Issue | 2 |
  | Pages | e56645 |
  | Publication | PloS one |
  | Date | 2013 |
  | Accessed | 9/20/2013, 1:18:50 PM |
  | Library Catalog | Google Scholar |
  | Date Added | 10/11/2013, 10:29:15 AM |
  | Modified | 10/11/2013, 10:29:15 AM |

  ### Notes:

  - Present an extension to a rigid-body protein-protein docking algorithm which performs exploration/exploitation step.

    How SCOP is used:

    Use Zdock protein-protein docking benchmark, which they note is non-redundant at the family-family pair level.

    SCOP reference:

    The complexes for testing and training were obtained from the widely used protein-protein docking benchmark developed by our lab (version 4.0) [29]. The benchmark contains 176 protein- protein complexes of which both the bound and unbound structures are available, and is non-redundant at the SCOP [30] family-family pair level. According to biochemical function, 52 complexes are of the enzyme-inhibitor type, 25 are antibody- antigen, and 99 ‘others’. In addition, the complexes are classified according to expected docking difficulty.

  ### Attachments

  - [HTML] from plos.org
  - journal.pone.0056645.pdf
- ## Exploring Early Stages of the Chemical Unfolding of Proteins at the Proteome Scale

  |  |  |
  | --- | --- |
  | Type | Journal Article |
  | Author | Michela Candotti |
  | Author | Alberto Perez |
  | Author | Carles Ferrer-Costa |
  | Author | Manuel Rueda |
  | Author | Tim Meyer |
  | Author | Josep Lluis Gelpi |
  | Author | Modesto Orozco |
  | Volume | 9 |
  | Issue | 12 |
  | Pages | e1003393 |
  | Publication | Plos Computational Biology |
  | ISSN | 1553-7358 |
  | Date | DEC 2013 |
  | Extra | WOS:000329364800018 |
  | DOI | 10.1371/journal.pcbi.1003393 |
  | Abstract | After decades of using urea as denaturant, the kinetic role of this molecule in the unfolding process is still undefined: does urea actively induce protein unfolding or passively stabilize the unfolded state? By analyzing a set of 30 proteins (representative of all native folds) through extensive molecular dynamics simulations in denaturant (using a range of force-fields), we derived robust rules for urea unfolding that are valid at the proteome level. Irrespective of the protein fold, presence or absence of disulphide bridges, and secondary structure composition, urea concentrates in the first solvation shell of quasi-native proteins, but with a density lower than that of the fully unfolded state. The presence of urea does not alter the spontaneous vibration pattern of proteins. In fact, it reduces the magnitude of such vibrations, leading to a counterintuitive slow down of the atomic-motions that opposes unfolding. Urea stickiness and slow diffusion is, however, crucial for unfolding. Long residence urea molecules placed around the hydrophobic core are crucial to stabilize partially open structures generated by thermal fluctuations. Our simulations indicate that although urea does not favor the formation of partially open microstates, it is not a mere spectator of unfolding that simply displaces to the right of the foldedunfolded equilibrium. On the contrary, urea actively favors unfolding: it selects and stabilizes partially unfolded microstates, slowly driving the protein conformational ensemble far from the native one and also from the conformations sampled during thermal unfolding. |
  | Date Added | 2/12/2014, 1:36:22 PM |
  | Modified | 2/12/2014, 1:36:22 PM |

  ### Notes:

  - Molecular dynamics study of a representative set of 30 proteins to investigate urea unfolding.

    How SCOP is used:

    Validate protocol of set of three proteins from three SCOP classes.

    Study unfolding on a representative set of proteins from 30 folds.

    SCOP reference:

    Results

    Protocol validation using three ultra-representative proteins

    We first validated our protocol using three ultra-representative proteins (in bold in Table 1), one for each of the main classes in the Structural Classification of Proteins (SCOP, [19]).

    ...

    Proteome-level study of urea unfolding

    After the validation of our protocol, we extended the chemical unfolding simulations to a larger set of proteins, to avoid any bias in the conclusion due to the native structure. We performed 1 msec of simulation in urea at high temperature (T=398K) for 30 proteins covering all the major protein folds (Table 1 and Suppl. Dataset S1).

  ### Attachments

  - journal.pcbi.1003393.pdf
- ## Exploring Fold Space Preferences of New-born and Ancient Protein Superfamilies

  |  |  |
  | --- | --- |
  | Type | Journal Article |
  | Author | Hannah Edwards |
  | Author | Sanne Abeln |
  | Author | Charlotte M. Deane |
  | Volume | 9 |
  | Issue | 11 |
  | Pages | e1003325 |
  | Publication | Plos Computational Biology |
  | ISSN | 1553-7358 |
  | Date | NOV 2013 |
  | Extra | WOS:000330357200026 |
  | DOI | 10.1371/journal.pcbi.1003325 |
  | Abstract | The evolution of proteins is one of the fundamental processes that has delivered the diversity and complexity of life we see around ourselves today. While we tend to define protein evolution in terms of sequence level mutations, insertions and deletions, it is hard to translate these processes to a more complete picture incorporating a polypeptide's structure and function. By considering how protein structures change over time we can gain an entirely new appreciation of their long-term evolutionary dynamics. In this work we seek to identify how populations of proteins at different stages of evolution explore their possible structure space. We use an annotation of superfamily age to this space and explore the relationship between these ages and a diverse set of properties pertaining to a superfamily's sequence, structure and function. We note several marked differences between the populations of newly evolved and ancient structures, such as in their length distributions, secondary structure content and tertiary packing arrangements. In particular, many of these differences suggest a less elaborate structure for newly evolved superfamilies when compared with their ancient counterparts. We show that the structural preferences we report are not a residual effect of a more fundamental relationship with function. Furthermore, we demonstrate the robustness of our results, using significant variation in the algorithm used to estimate the ages. We present these age estimates as a useful tool to analyse protein populations. In particularly, we apply this in a comparison of domains containing greek key or jelly roll motifs. |
  | Date Added | 2/20/2014, 12:24:01 PM |
  | Modified | 10/8/2014, 12:50:52 PM |

  ### Tags:

  - Interesting

  ### Notes:

  - Age annotation method

    1. Get superfamily and fold occurrence matrices. For a set of genomes, use SUPERFAMILY to annotate domains with superfamiy and fold classification. Create matrices where each row is a bit string of length k, where k is the number of superfamilies in SCOP.  The presence of absence of each superfamily is recorded as 1s and 0s.

    2. Build and/or annotate whole genome trees.  Use a the NCBI common taxonomy tree, and also built their own whole genome trees. The "distance tree" method measures the distance between the vectors of superfamiy presence/absense for each genome.  4 distance matrices were created, using both fold and superfamily and the 2 different distance functions.  Then the CONSENSE algorithm was used to build trees using the occurrence matrix and branch lengths were calculated based on the distance matrix using the FITCH algorithm. Also build trees using the "parsimony tree" method.  All trees were rooted and branch lengths normalized to between 0 and 1.

    3. Age estimation.  Perform "parsimony analysis" on each tree to infer the age of each superfamily.  Since several scenarios of gain and loss events can be used to annotate the same tree, maximum parsimony analysis is used for optimization.  Maximum parsimony attempts to find the scenario that minimizes S = lamba + g\*gamma, lambda and gamma are the number of loss and gain events.  The age of each ancestor is estimated using the height on the tree where the first event occurs.
  - Investigate relationship between superfamily age and sequence, structure, and function.

    How SCOP is used:

    Annotate SCOP superfamilies with ages.

    "Derive a database from SCOP", providing ages for each superfamily via a method based on phylogenetic analysis.

    How CATH is used:

    Background on protein structure classification.

    SCOP reference:

    Results

    1,847 SCOP superfamilies are annotated with an estimate of their age relative to a tree of life incorporating 1,014 completely sequenced genomes across the three superkingdoms (Archaea, Bacteria and Eukarya). These ages can be found online at http:// www.stats.ox.ac.uk/research/proteins/resources. The superfamily age is a relative measure of when that superfamily first appeared, calculated according to parsimonious interpretations of evolution- ary events. Figure 1 gives an outline of the age estimation procedure. These ages are used to discriminate the set of superfamilies into different age groups. There are 557 ancient superfamilies, that are predicted to have first evolved at the root of the tree (age~1) and 443 new-born superfamilies, predicted to have an ancestor nearer the leaves of the tree (agev0:4). As there is not a single standard tree of life we calculate age estimates using 8 different phylogenetic trees (see methods for descriptions of the different trees).

    SCOP/CATH reference:

    In order to visualise the landscape and diversity of structure space protein structures have been clustered within a hierarchical taxonomy [4,5].

  ### Attachments

  - journal.pcbi.1003325.pdf
- ## Exploring functionally related enzymes using radially distributed properties of active sites around the reacting points of bound ligands

  |  |  |
  | --- | --- |
  | Type | Journal Article |
  | Author | Keisuke Ueno |
  | Author | Katsuhiko Mineta |
  | Author | Kimihito Ito |
  | Author | Toshinori Endo |
  | Volume | 12 |
  | Pages | 5 |
  | Publication | BMC Structural Biology |
  | ISSN | 1472-6807 |
  | Date | 2012 |
  | Extra | PMID: 22536854 |
  | Journal Abbr | BMC Struct. Biol. |
  | DOI | 10.1186/1472-6807-12-5 |
  | Library Catalog | NCBI PubMed |
  | Language | eng |
  | Abstract | BACKGROUND: Structural genomics approaches, particularly those solving the 3D structures of many proteins with unknown functions, have increased the desire for structure-based function predictions. However, prediction of enzyme function is difficult because one member of a superfamily may catalyze a different reaction than other members, whereas members of different superfamilies can catalyze the same reaction. In addition, conformational changes, mutations or the absence of a particular catalytic residue can prevent inference of the mechanism by which catalytic residues stabilize and promote the elementary reaction. A major hurdle for alignment-based methods for prediction of function is the absence (despite its importance) of a measure of similarity of the physicochemical properties of catalytic sites. To solve this problem, the physicochemical features radially distributed around catalytic sites should be considered in addition to structural and sequence similarities. RESULTS: We showed that radial distribution functions (RDFs), which are associated with the local structural and physicochemical properties of catalytic active sites, are capable of clustering oxidoreductases and transferases by function. The catalytic sites of these enzymes were also characterized using the RDFs. The RDFs provided a measure of the similarity among the catalytic sites, detecting conformational changes caused by mutation of catalytic residues. Furthermore, the RDFs reinforced the classification of enzyme functions based on conventional sequence and structural alignments. CONCLUSIONS: Our results demonstrate that the application of RDFs provides advantages in the functional classification of enzymes by providing information about catalytic sites. |
  | Date Added | 10/11/2013, 10:29:15 AM |
  | Modified | 12/2/2013, 4:20:03 PM |

  ### Tags:

  - Biocatalysis
  - Catalytic Domain
  - Databases, Protein
  - Ligands
  - Models, Molecular
  - Molecular Sequence Annotation
  - Mutation
  - Nonlinear Dynamics
  - Oxidoreductases
  - Physicochemical Phenomena
  - Sequence Alignment
  - Transferases

  ### Notes:

  - Study active sites of functionally related enzymes.  Calculated radial distribution functions (RDFs) which are associated with local structure and properties of catalytic active sites.

    How SCOP is used:

    Cannot deduce how SCOP is used in this paper.

    Going with annotated with SCOP class.

    SCOP reference:

    Use SCOP in two tables

  ### Attachments

  - 1472-6807-12-5.pdf
- ## Exploring Protein Dynamics Space: The Dynasome as the Missing Link between Protein Structure and Function

  |  |  |
  | --- | --- |
  | Type | Journal Article |
  | Author | Ulf Hensen |
  | Author | Tim Meyer |
  | Author | Juergen Haas |
  | Author | Rene Rex |
  | Author | Gert Vriend |
  | Author | Helmut Grubmueller |
  | Volume | 7 |
  | Issue | 5 |
  | Publication | Plos One |
  | ISSN | 1932-6203 |
  | Date | MAY 11 2012 |
  | Extra | WOS:000305338200004 |
  | DOI | 10.1371/journal.pone.0033931 |
  | Abstract | Proteins are usually described and classified according to amino acid sequence, structure or function. Here, we develop a minimally biased scheme to compare and classify proteins according to their internal mobility patterns. This approach is based on the notion that proteins not only fold into recurring structural motifs but might also be carrying out only a limited set of recurring mobility motifs. The complete set of these patterns, which we tentatively call the dynasome, spans a multidimensional space with axes, the dynasome descriptors, characterizing different aspects of protein dynamics. The unique dynamic fingerprint of each protein is represented as a vector in the dynasome space. The difference between any two vectors, consequently, gives a reliable measure of the difference between the corresponding protein dynamics. We characterize the properties of the dynasome by comparing the dynamics fingerprints obtained from molecular dynamics simulations of 112 proteins but our approach is, in principle, not restricted to any specific source of data of protein dynamics. We conclude that: 1. the dynasome consists of a continuum of proteins, rather than well separated classes. 2. For the majority of proteins we observe strong correlations between structure and dynamics. 3. Proteins with similar function carry out similar dynamics, which suggests a new method to improve protein function annotation based on protein dynamics. |
  | Date Added | 10/28/2013, 4:53:08 PM |
  | Modified | 3/7/2014, 1:07:08 PM |

  ### Notes:

  - Compare dynamics across different SCOP classes.

    How SCOP is used:

    Annotate a non-redundant data set (curated using pdbfinder) of 112 proteins by SCOP class.  Plot some metrics on dynamics for each protein and show that structural classes are clustered, implying that dynamics could be used to predict structural class.

     How CATH is used:

    Do not use CATH data.  Cite only as a second example of a protein structure classification database.

    SCOP reference:

    Before addressing this question in more detail, however, we investigated the extent of the structural classification reflected in the dynamics space. Fig. 4b shows the same projections as in a) with color codes indicating the SCOP structure class. Different structure classes tend to accumulate in different regions of dynamics space. All{a proteins are, for example, predominantly found on the right, whereas most all{b proteins are found to the left. a=b-proteins overlap significantly with all{b, but are shifted slightly towards the bottom. Small proteins cover a large range from the upper left to the right. The standard deviation of the distributions of proteins of each SCOP class (large ellipses in Fig. 4b) show that the distributions overlap significantly. In contrast, the centroids of the different classes (centre of the ellipses) assume significantly different positions in dynamics space, as documented by the standard deviations of the mean (small circles).

    CATH reference:

    Figure 7 shows the distribution of protein structures (points) in the plane spanned by the first two eigenvectors obtained from this PCA. As can be seen from Fig. 7a, no clusters are evident in the space of protein structures, quite similar to our observation in the space of protein dynamics. This result supports our above conjecture that SCOP and CATH suggest a much clearer partitioning of protein structure space than is evident from our unsupervised classification from a set of 24 structural observables, and in fact also from other unsupervised approaches [63,66]. From this point of view, our finding of a rather unstructured dynasome is less surprising.

  ### Attachments

  - journal.pone.0033931.pdf
- ## Exploring the "dark matter" of a mammalian proteome by protein structure and function modeling

  |  |  |
  | --- | --- |
  | Type | Journal Article |
  | Author | Michal Brylinski |
  | Volume | 11 |
  | Pages | 47 |
  | Publication | Proteome Science |
  | ISSN | 1477-5956 |
  | Date | DEC 9 2013 |
  | Extra | WOS:000329833600001 |
  | DOI | 10.1186/1477-5956-11-47 |
  | Abstract | Background: A growing body of evidence shows that gene products encoded by short open reading frames play key roles in numerous cellular processes. Yet, they are generally overlooked in genome assembly, escaping annotation because small protein-coding genes are difficult to predict computationally. Consequently, there are still a considerable number of small proteins whose functions are yet to be characterized. Results: To address this issue, we apply a collection of structural bioinformatics algorithms to infer molecular function of putative small proteins from the mouse proteome. Specifically, we construct 1,743 confident structure models of small proteins, which reveal a significant structural diversity with a noticeably high helical content. A subsequent structure-based function annotation of small protein models exposes 178,745 putative protein-protein interactions with the remaining gene products in the mouse proteome, 1,100 potential binding sites for small organic molecules and 987 metal-binding signatures. Conclusions: These results strongly indicate that many small proteins adopt three-dimensional structures and are fully functional, playing important roles in transcriptional regulation, cell signaling and metabolism. Data collected through this work is freely available to the academic community at http://www.brylinski.org/content/databases to support future studies oriented on elucidating the functions of hypothetical small |
  | Date Added | 2/13/2014, 4:13:17 PM |
  | Modified | 3/7/2014, 12:10:13 PM |
- ## Exploring the diversity of SPRY/B30.2-mediated interactions

  |  |  |
  | --- | --- |
  | Type | Journal Article |
  | Author | Livia Perfetto |
  | Author | Pier Federico Gherardini |
  | Author | Norman E. Davey |
  | Author | Francesca Diella |
  | Author | Manuela Helmer-Citterich |
  | Author | Gianni Cesareni |
  | Volume | 38 |
  | Issue | 1 |
  | Pages | 38-46 |
  | Publication | Trends in Biochemical Sciences |
  | ISSN | 0968-0004 |
  | Date | JAN 2013 |
  | Extra | Review of studies of SPRY domain. How SCOP is used: Look up classification of SPRY domains in SCOP. SCOP reference: Structure of SPRY/B30.2 domains The core fold of the SPRY/B30.2 domain is a twisted b sandwich with anti-parallel b sheets. This fold is similar to that of carbohydrate binding lectins (SPRY domains are a superfamily of the concanavalin A-like lectin/glucanase fold topology in the structural classification of proteins (SCOP) classification [44]) and of neuralized homology repeat (NHR) domains of the neuralized family [45]. This architecture is reminiscent of the immunoglobulin fold (albeit with a completely different topology) and similarly supports several loops of variable length and sequence [46]. |
  | DOI | 10.1016/j.tibs.2012.10.001 |
  | Abstract | The SPIa/Ryanodine receptor (SPRY)/B30.2 domain is one of the most common folds in higher eukaryotes. The human genome encodes 103 SPRY/B30.2 domains, several of which are involved in the immune response. Approximately 45% of human SPRY/B30.2-containing proteins are |
  | Date Added | 2/20/2014, 12:24:01 PM |
  | Modified | 12/15/2014, 1:24:38 PM |

  ### Notes:

  - Review of studies of SPRY domain.

    How SCOP is used:

    Look up classification of SPRY domains in SCOP.

    SCOP reference:

    Structure of SPRY/B30.2 domains The core fold of the SPRY/B30.2 domain is a twisted b sandwich with anti-parallel b sheets. This fold is similar to that of carbohydrate binding lectins (SPRY domains are a superfamily of the concanavalin A-like lectin/glucanase fold topology in the structural classification of proteins (SCOP) classification [44]) and of neuralized homology repeat (NHR) domains of the neuralized family [45]. This architecture is reminiscent of the immunoglobulin fold (albeit with a completely different topology) and similarly supports several loops of variable length and sequence [46].

  ### Attachments

  - 1-s2.0-S0968000412001569-main.pdf
- ## Exploring the Energy Landscapes of Protein Folding Simulations with Bayesian Computation

  |  |  |
  | --- | --- |
  | Type | Journal Article |
  | Author | Nikolas S. Burkoff |
  | Author | Csilla Varnai |
  | Author | Stephen A. Wells |
  | Author | David L. Wild |
  | Volume | 102 |
  | Issue | 4 |
  | Pages | 878-886 |
  | Publication | BIOPHYSICAL JOURNAL |
  | ISSN | 0006-3495 |
  | Date | FEB 22 2012 |
  | DOI | 10.1016/j.bpj.2011.12.053 |
  | Language | English |
  | Abstract | Nested sampling is a Bayesian sampling technique developed to explore probability distributions localized in an exponentially small area of the parameter space. The algorithm provides both posterior samples and an estimate of the evidence (marginal likelihood) of the model. The nested sampling algorithm also provides an efficient way to calculate free energies and the expectation value of thermodynamic observables at any temperature, through a simple post processing of the output. Previous applications of the algorithm have yielded large efficiency gains over other sampling techniques, including parallel tempering. In this article, we describe a parallel implementation of the nested sampling algorithm and its application to the problem of protein folding in a GO-like force field of empirical potentials that were designed to stabilize secondary structure elements in room-temperature simulations. We demonstrate the method by conducting folding simulations on a number of small proteins that are commonly used for testing protein-folding procedures. A topological analysis of the posterior samples is performed to produce energy landscape charts, which give a high-level description of the potential energy surface for the protein folding simulations. These charts provide qualitative insights into both the folding process and the nature of the model and force field used. |
  | Date Added | 10/25/2013, 4:29:01 PM |
  | Modified | 10/25/2013, 4:29:01 PM |

  ### Notes:

  - Present method for sampling conformational space for protein folding simulation.

    How SCOP is used:

    Not completely explained, but I infer that an ASTRAL representative subset of structures was used to collect side chain dihedral angles (Chi) and determine their distribution for use in their energy model.

    SCOP reference:

    In this work, we represented other side chain atoms by one, or in the case of branched side chains, two pseudo-atoms, following (6). The side chain dihedral angles χ were permitted to vary, and take the values {±60◦,180◦}, or in the case of proline {±30◦}, with probabilities dependent on residue type, with values corresponding to the distribution of the χ angles in the same ASTRAL PDB database (7) that was used in (4), and here, to learn the potential parameters by a statistical machine learning procedure, contrastive divergence (8).

  ### Attachments

  - PIIS0006349512000550.mmc1.pdf
- ## Exploring the evolution of novel enzyme functions within structurally defined protein superfamilies

  |  |  |
  | --- | --- |
  | Type | Journal Article |
  | Author | Nicholas Furnham |
  | Author | Ian Sillitoe |
  | Author | Gemma L. Holliday |
  | Author | Alison L. Cuff |
  | Author | Roman A. Laskowski |
  | Author | Christine A. Orengo |
  | Author | Janet M. Thornton |
  | URL | http://dx.plos.org/10.1371/journal.pcbi.1002403 |
  | Volume | 8 |
  | Issue | 3 |
  | Pages | e1002403 |
  | Publication | PLoS computational biology |
  | Date | 2012 |
  | Accessed | 9/20/2013, 11:09:53 AM |
  | Library Catalog | Google Scholar |
  | Date Added | 2/20/2014, 12:24:01 PM |
  | Modified | 3/7/2014, 12:14:57 PM |

  ### Notes:

  - Computational study of enzyme function evolution.  Apply FunTree system for this task to enzyme superfamilies in CATH.

    How SCOP is used:

    Protein structure classification.  Research context reference.

    How CATH is used:

    Analyze a few superfamilies in CATH.

    SCOP/CATH reference:

    In general, it is possible to organise and classify proteins into families and superfamilies based on similarities between sequence and/or structure. Very distant relationships between proteins can usually be more successfully detected through analysis of their three- dimensional atomic structures rather than by sequence alone [5]. To this end, a number of classifications of protein three-dimensional structure have been developed to capture evolutionary relation- ships, most notably CATH [6] and SCOP [7]. Both of these classifications use protein structural domains as the discrete entity, with a protein being made up of one domain or more in which case it is described as having a multi-domain architecture (MDA). Domains often combine in multiple different ways creating different MDAs, often with different functions. Domains can be classified into superfamilies based on a detectable evolutionary relationship.

    CATH reference:

    We apply the pipeline to analyse enzyme superfamilies in CATH, using robust structurally-informed multiple sequence alignments to build phylogenetic trees, which are then annotated with structural and functional data. Relationships between metabolites, obtained by exploiting tools for comparing small molecules, are displayed on the phylogenetic tree. We have chosen two specific superfamilies to illustrate the value of combining structural and functional data to explore evolutionary changes. Analyses of these functional changes in 276 well-defined enzyme superfamilies has allowed us to present a preliminary overview of the evolution of novel enzyme functions in order to begin to gather, catalogue and classify the emergence of the catalytic reactions necessary for life.

  ### Attachments

  - [HTML] from plos.org
  - journal.pcbi.1002403.pdf
- ## Exploring the evolution of protein function in Archaea

  |  |  |
  | --- | --- |
  | Type | Journal Article |
  | Author | Alexander Goncearenco |
  | Author | Igor N. Berezovsky |
  | URL | http://www.biomedcentral.com/1471-2148/12/75 |
  | Volume | 12 |
  | Issue | 1 |
  | Pages | 75 |
  | Publication | BMC Evolutionary Biology |
  | Date | 2012 |
  | Accessed | 9/20/2013, 1:19:04 PM |
  | Library Catalog | Google Scholar |
  | Date Added | 10/11/2013, 10:29:15 AM |
  | Modified | 2/18/2014, 10:12:31 AM |

  ### Tags:

  - Amino Acyl-tRNA Synthetases
  - Archaea
  - Archaea
  - Archaeal Proteins
  - Elementary functional loops
  - evolution
  - Evolution, Molecular
  - Functional domains/folds
  - Methane
  - Models, Molecular
  - Protein Folding
  - Protein function
  - Protein Structure, Tertiary
  - Proteome
  - Structure-Activity Relationship

  ### Notes:

  - Analyze distant evolutionary connections between protein functions in Archaea based on elementary functional loops (EFLs) comprising them.  EFLs are functional units of enzymes that provide elementary reactions in biochemical transformations.

    How SCOP is used:

    Use SUPERFAMILY to detect SCOP folds in a data set from archaeal Clusters of Orthologous Groups of proteins (arCOGs).  Examine the diversity of folds found in the data set.

    SCOP references:

    Detecting domains in arCOGs

    We used HMM library from Superfamily database [50] based on ASTRAL/SCOP release 1.75 [32,51] in order to detect SCOP folds in arCOGs [26]. A complete list of detected SCOP folds in the core, shell, and orphan arCOGs is provided in Additional File 1.

    Assigning the elementary function

    Sequence profiles of elementary functional loops were used to find matches in CDD and SCOP domains with known structure [32,53]. For many protein families func- tionally important residues are known, and the role of the latter in binding [39], intermolecular interactions [54], and mechanism of catalysis [55] was used to assign the profiles their elementary functions.

  ### Attachments

  - 1471-2148-12-75.pdf
  - [HTML] from biomedcentral.com
  - PubMed entry
- ## Expression, purification and molecular modeling of the NIa protease of Cardamom mosaic virus

  |  |  |
  | --- | --- |
  | Type | Journal Article |
  | Author | T. Jebasingh |
  | Author | Eswari P. J. Pandaranayaka |
  | Author | A. Mahalakshmi |
  | Author | A. Kasin Yadunandam |
  | Author | S. Krishnaswamy |
  | Author | R. Usha |
  | Volume | 31 |
  | Issue | 6 |
  | Pages | 602-611 |
  | Publication | Journal of Biomolecular Structure & Dynamics |
  | ISSN | 0739-1102 |
  | Date | JUN 1 2013 |
  | Extra | WOS:000319323900005 |
  | DOI | 10.1080/07391102.2012.706078 |
  | Abstract | The NIa protease of Potyviridae is the major viral protease that processes potyviral polyproteins. The NIa protease coding region of Cardamom mosaic virus (CdMV) is amplified from the viral cDNA, cloned and expressed in Escherichia coli. NIa protease forms inclusion bodies in E.coli. The inclusion bodies are solubilized with 8M urea, refolded and purified by Nickel-Nitrilotriacetic acid affinity chromatography. Three-dimensional modeling of the CdMV NIa protease is achieved by threading approach using the homologous X-ray crystallographic structure of Tobacco etch mosaic virus NIa protease. The model gave an insight in to the substrate specificities of the NIa proteases and predicted the complementation of nearby residues in the catalytic triad (H42, D74 and C141) mutants in the cis protease activity of CdMV NIa protease. |
  | Date Added | 10/28/2013, 4:53:08 PM |
  | Modified | 3/7/2014, 1:06:45 PM |

  ### Notes:

  - Experimental and computational study of protein of interest: Nla protease.

    How SCOP is used:

    Annotate data set with SCOP fold.

    SCOP reference:

    Sequence analysis and molecular modeling

    Protein sequences used in this study were retrieved from the protein sequence database at NCBI (http://www.ncbi. nlm.nih.gov/). The 3D Structures of proteins were obtained from the Brookhaven protein data bank (PDB) (http://www.rcsb.org/) (Berman et al., 2000). The fold classification of proteins is retrieved from the Structural classification of proteins database (Andreeva et al., 2004). The protein sequence was compared against the non-redundant protein sequences using BLASTP. Multi- ple sequence alignment was performed with CLU- STALW (http://www.ebi.ac.uk/clustalW/index.html). The CdMV NIa protease was submitted to the fold recogni- tion servers such as FFASO3 (Jaroszewski, Rychlewski, Li, Li, & Godzik, 2005), FUGUE (Shi, Blundell, & Miz- uguchi, 2001), mGenThreader (Jones, 1999), 3D PSSM (Kelley, MacCallum, & Sternberg, 2000), Phyre2.0 (Kel- ley & Sternberg, 2009), and SAM-T02 (Karplus et al., 2003) for fold recognition.

  ### Attachments

  - 07391102%2E2012%2E706078.pdf
- ## Extending Signaling Pathways with Protein-Interaction Networks. Application to Apoptosis

  |  |  |
  | --- | --- |
  | Type | Journal Article |
  | Author | Joan Planas-Iglesias |
  | Author | Emre Guney |
  | Author | Javier Garcia-Garcia |
  | Author | Kevin A. Robertson |
  | Author | Sobia Raza |
  | Author | Tom C. Freeman |
  | Author | Peter Ghazal |
  | Author | Baldo Oliva |
  | Volume | 16 |
  | Issue | 5 |
  | Pages | 245-256 |
  | Publication | Omics-a Journal of Integrative Biology |
  | ISSN | 1536-2310 |
  | Date | MAY 2012 |
  | Extra | WOS:000303653300003 |
  | DOI | 10.1089/omi.2011.0130 |
  | Abstract | Cells exploit signaling pathways during responses to environmental changes, and these processes are often modulated during disease. Particularly, relevant human pathologies such as cancer or viral infections require downregulating apoptosis signaling pathways to progress. As a result, the identification of proteins responsible for these changes is essential for the diagnostics and development of therapeutics. Transferring functional annotation within protein interaction networks has proven useful to identify such proteins, although this is not a trivial task. Here, we used different scoring methods to transfer annotation from 53 well-studied members of the human apoptosis pathways (as known by 2005) to their protein interactors. All scoring methods produced significant predictions (compared to a random negative model), but its number was too large to be useful. Thus, we made a final prediction using specific combinations of scoring methods and compared it to the proteins related to apoptosis signaling pathways during the last 5 years. We propose 273 candidate proteins that may be relevant in apoptosis signaling pathways. Although some of them have known functions consistent with their proposed apoptotsis involvement, the majority have not been annotated yet, leaving room for further experimental studies. We provide our predictions at http://sbi.imim.es/web/Apoptosis.php |
  | Date Added | 2/20/2014, 12:24:01 PM |
  | Modified | 2/20/2014, 12:24:01 PM |

  ### Notes:

  - Paper unavailable.
- ## Extent of structural asymmetry in homodimeric proteins: prevalence and relevance

  |  |  |
  | --- | --- |
  | Type | Journal Article |
  | Author | Lakshmipuram Seshadri Swapna |
  | Author | Kuchi Srikeerthana |
  | Author | Narayanaswamy Srinivasan |
  | Volume | 7 |
  | Issue | 5 |
  | Pages | e36688 |
  | Publication | PloS one |
  | ISSN | 1932-6203 |
  | Date | 2012 |
  | Extra | PMID: 22629324 |
  | Journal Abbr | PLoS ONE |
  | DOI | 10.1371/journal.pone.0036688 |
  | Library Catalog | NCBI PubMed |
  | Language | eng |
  | Abstract | Most homodimeric proteins have symmetric structure. Although symmetry is known to confer structural and functional advantage, asymmetric organization is also observed. Using a non-redundant dataset of 223 high-resolution crystal structures of biologically relevant homodimers, we address questions on the prevalence and significance of asymmetry. We used two measures to quantify global and interface asymmetry, and assess the correlation of several molecular and structural parameters with asymmetry. We have identified rare cases (11/223) of biologically relevant homodimers with pronounced global asymmetry. Asymmetry serves as a means to bring about 2:1 binding between the homodimer and another molecule; it also enables cellular signalling arising from asymmetric macromolecular ligands such as DNA. Analysis of these cases reveals two possible mechanisms by which possible infinite array formation is prevented. In case of homodimers associating via non-topologically equivalent surfaces in their tertiary structures, ligand-dependent mechanisms are used. For stable dimers binding via large surfaces, ligand-dependent structural change regulates polymerisation/depolymerisation; for unstable dimers binding via smaller surfaces that are not evolutionarily well conserved, dimerisation occurs only in the presence of the ligand. In case of homodimers associating via interaction surfaces with parts of the surfaces topologically equivalent in the tertiary structures, steric hindrance serves as the preventive mechanism of infinite array. We also find that homodimers exhibiting grossly symmetric organization rarely exhibit either perfect local symmetry or high local asymmetry. Binding of small ligands at the interface does not cause any significant variation in interface asymmetry. However, identification of biologically relevant interface asymmetry in grossly symmetric homodimers is confounded by the presence of similar small magnitude changes caused due to artefacts of crystallisation. Our study provides new insights regarding accommodation of asymmetry in homodimers. |
  | Short Title | Extent of structural asymmetry in homodimeric proteins |
  | Date Added | 10/11/2013, 10:29:15 AM |
  | Modified | 10/11/2013, 10:29:15 AM |

  ### Tags:

  - Dimerization
  - Models, Molecular
  - Protein Binding
  - Protein Conformation
  - Protein Multimerization
  - Proteins

  ### Notes:

  - Computational study of asymmetry in homodimers.  Use a non-redundant data set of  223 crystal structures of homodimers.

    How SCOP is used:

    Collected homologs from the same SCOP family for 11 significantly assymmetric homodimers.  Used homologs in a sequence alignment to determine in the interface is conserved and to measure asymmetry across homologs.

    SCOP reference:

    Dataset of biologically relevant asymmetric homodimers

    Entries of homodimers in PiQSi are broadly categorized as symmetric or non-symmetric (termed ‘asymmetric’ in our analy- sis). The classification is based on a procedure involving the rotation of both subunits (by 360/N angles – where ‘N’ is the number of subunits in the complex) about a set of 600 axes passing through the centre of mass of the structure [10]. If the average Euclidian distance after all rotations is .7 A ̊ for all axes, then the structure is considered to be non-symmetric. From the redundant dataset of homodimers generated, entries with a global asymmetry score $7 (n = 23) were considered as a starting set of asymmetric homodimers. This set was also augmented by entries culled manually from literature (n = 6). Thorough literature analysis of these complexes (23+6) yielded a selection of 11 homodimers with pronounced asymmetry with clear functional relevance elucidated from experiments. For these 11 cases, homologues of known 3D structure, identified as members belonging to the same SCOP [64] family, were obtained for further analysis.

  ### Attachments

  - journal.pone.0036688.pdf
  - PubMed entry
- ## Extracting knowledge from protein structure geometry

  |  |  |
  | --- | --- |
  | Type | Journal Article |
  | Author | Peter Rogen |
  | Author | Patrice Koehl |
  | Volume | 81 |
  | Issue | 5 |
  | Pages | 841-851 |
  | Publication | Proteins-Structure Function and Bioinformatics |
  | ISSN | 0887-3585 |
  | Date | MAY 2013 |
  | Extra | WOS:000317288100009 |
  | DOI | 10.1002/prot.24242 |
  | Abstract | Protein structure prediction techniques proceed in two steps, namely the generation of many structural models for the protein of interest, followed by an evaluation of all these models to identify those that are native-like. In theory, the second step is easy, as native structures correspond to minima of their free energy surfaces. It is well known however that the situation is more complicated as the current force fields used for molecular simulations fail to recognize native states from misfolded structures. In an attempt to solve this problem, we follow an alternate approach and derive a new potential from geometric knowledge extracted from native and misfolded conformers of protein structures. This new potential, Metric Protein Potential (MPP), has two main features that are key to its success. Firstly, it is composite in that it includes local and nonlocal geometric information on proteins. At the short range level, it captures and quantifies the mapping between the sequences and structures of short (7-mer) fragments of protein backbones through the introduction of a new local energy term. The local energy term is then augmented with a nonlocal residue-based pairwise potential, and a solvent potential. Secondly, it is optimized to yield a maximized correlation between the energy of a structural model and its root mean square (RMS) to the native structure of the corresponding protein. We have shown that MPP yields high correlation values between RMS and energy and that it is able to retrieve the native structure of a protein from a set of high-resolution decoys. Proteins 2013. (c) 2012 Wiley Periodicals, Inc. |
  | Date Added | 2/13/2014, 4:13:17 PM |
  | Modified | 3/7/2014, 12:09:00 PM |
- ## Extracting Signatures of Spatial Organization for Biomolecular Nanostructures

  |  |  |
  | --- | --- |
  | Type | Journal Article |
  | Author | Aditya Mittal |
  | Author | Chanchal Acharya |
  | URL | http://www.ingentaconnect.com/content/asp/jnn/2012/00000012/00000011/art00004 |
  | Volume | 12 |
  | Issue | 11 |
  | Pages | 8249–8257 |
  | Publication | Journal of nanoscience and nanotechnology |
  | Date | 2012 |
  | Accessed | 9/23/2013, 10:23:40 AM |
  | Library Catalog | Google Scholar |
  | Date Added | 10/11/2013, 10:29:15 AM |
  | Modified | 3/7/2014, 12:14:17 PM |

  ### Tags:

  - Biomolecular Structure
  - Interesting
  - Nanobiology
  - Nanostructure
  - protein folding
  - Proteins
  - Structural biology

  ### Notes:

  - Present and apply a method for extracting a 2D signature from 3D structure for comparison.   Apply to >4000 crystal structures from PDB.  Find some properties are unique to proteins "regardless of structural classification".

    How SCOP is used:

    Examine structural signatures of a large data set of high-res (<2.5A) structures from SCOP database, classified by SCOP class.  Found that "soluble proteins have a universal spatial organization regardless of size, fold, structure, and function".

    SCOP Reference:

    Here, it is important to note that while a universal spatial distribu- tion for folded proteins was already established based on analysis of ∼ 4000 crystal structures from the Protein Data Bank,9⬚⬚ 10⬚⬚ 12 we decided to investigate spatial organization of backbones, for an even larger number of crys- tal structures, from the SCOP database (http://scop.mrc- lmb.cam.ac.uk/scop/) that structurally classifies proteins.  Thus, we analyzed 13550 crystal structures (PDB IDs of all the proteins are provided as additional file) of solu- ble proteins from the SCOP database (selected using only accuracy filters—only structures with a resolution of 2.5 Å or better, and without missing any coordinates for any residues were considered; No other specific “culling” of data was done). Considering each of the amino acids in the crystal structures individually, the geometrical analy- sis (described in Fig. 1) gave rise to neighbourhood data for a given protein backbone (i.e., C-alpha coordinates) in form of a 20×20 matrix at each neighbourhood dis- tance. Figure 6(a) shows the geometrical representation of the analysis of C-alpha coordinates, analogous to random points in Figure 2(a). Finally, the sigmoidal neighbour- hood data was analyzed by plotting the total number of 20 × 20 matrices (which was equal to the total number of the defined neighbourhood distances) as a function of neighbourhood distance. Thus, for each classification of proteins from the SCOP database (see legend to Fig. 6), we obtained 400 sigmoidal data sets, each sigmoidal data set representing neighbourhood between two specific amino acids out of the 20.

    ...

    Fig. 6. Soluble proteins are ellipsoidal with a signature of a universal spatial organization, regardless of shape, size or function. (a) Neighbourhood analysis for C-alpha atoms (i.e., backbones) of 13550 crystal structures in the SCOP database (Alpha—2066, Beta—3131, Alpha/Beta—4877, Alpha+ Beta—3476), was done exactly as in Figure 2. Only crystal structures of soluble proteins with a resolution of 2.5 Å or better, and without having any missing coordinates for any residue, were considered. No “culling” of data was done based on shape, size/length or function of the proteins. (

  ### Attachments

  - [PDF] from iitd.ac.in
  - Snapshot
- ## FAMILY FINGERPRINTS: A GLOBAL APPROACH TO STRUCTURAL CLASSIFICATION

  |  |  |
  | --- | --- |
  | Type | Journal Article |
  | Author | Alberto Casagrande |
  | Author | Francesco Fabris |
  | URL | http://www.worldscientific.com/doi/abs/10.1142/S0219720012420012?url\_ver=Z39.88-2003&rfr\_id=ori:rid:crossref.org&rfr\_dat=cr\_pub%3Dpubmed |
  | Volume | 10 |
  | Issue | 03 |
  | Publication | Journal of bioinformatics and computational biology |
  | Date | 2012 |
  | Accessed | 9/23/2013, 10:22:49 AM |
  | Library Catalog | Google Scholar |
  | Short Title | FAMILY FINGERPRINTS |
  | Date Added | 10/11/2013, 10:29:15 AM |
  | Modified | 3/7/2014, 12:15:09 PM |

  ### Tags:

  - BLOSUM spectrum
  - likely ASTRAL
  - likely ASTRAL domain structures
  - protein domains
  - SCOP classification

  ### Notes:

  - Present method for structural profile-based protein family classification.

    How SCOP is used:

    Curate a data set of 105 non-redundant sequences from SCOP.

    Validate their method at SCOP family classification.

    How CATH is used:

    Not using CATH data.

    SCOP reference:

    SCOP (Structural Classi ̄cation Of Proteins)1 has been proposed as a high quality hierarchical classi ̄cation for protein domains. It contains a human-guided classi ̄cation of structural domains based on sequence and structure similarities. This classi ̄cation is organized into four hierarchical levels, namely (from the lowest to the highest) the \Family" level, the \Superfamily" level, the \Fold" level and the \Class" level. The Family level contains proteins having a common evolutionary. SCOP is very accurate and, despite some di®erences concerning taxonomy and methods, the main SCOP competitors, e.g. CATH2 and FSSP,3 agree with it on most of classi ̄cations.

    Many classi ̄cation methods have been proposed so far.46 Each of them suggests a di®erent approach to solve this problem, but, as far as we know, they all exploit sequence similarity to discover analogous structures and to ̄t domains in the correct families. The most naïve technique to classify a query sequence consists in aligning such a sequence with all the fragments already included in a database, for instance by using BLAST7: the best hit would ideally suggest the most similar structure and, as a consequence, the best family classi ̄cation. Unfortunately, this is not always the case, and there exist sequences in the same SCOP family whose similarity is not discovered by BLAST.

  ### Attachments

  - s0219720012420012.pdf
- ## Fast alignment and comparison of RNA structures

  |  |  |
  | --- | --- |
  | Type | Journal Article |
  | Author | Tim Wiegels |
  | Author | Stefan Bienert |
  | Author | Andrew E. Torda |
  | Volume | 29 |
  | Issue | 5 |
  | Pages | 588–596 |
  | Publication | Bioinformatics |
  | Date | March 2013 |
  | DOI | 10.1093/bioinformatics/btt006 |
  | Abstract | Motivation: To recognize remote relationships between RNA molecules, one must be able to align structures without regard to sequence similarity. We have implemented a method, which is swift [O(n(2))], sensitive and tolerant of large gaps and insertions. Molecules are broken into overlapping fragments, which are characterized by their memberships in a probabilistic classification based on local geometry and H-bonding descriptors. This leads to a probabilistic similarity measure that is used in a conventional dynamic programming method. Results: Examples are given of database searching, the detection of structural similarities, which would not be found using sequence based methods, and comparisons with a previously published approach. |
  | Date Added | 3/7/2014, 1:06:24 PM |
  | Modified | 3/7/2014, 1:06:24 PM |
- ## Fast large-scale clustering of protein structures using Gauss integrals

  |  |  |
  | --- | --- |
  | Type | Journal Article |
  | Author | Tim Harder |
  | Author | Mikael Borg |
  | Author | Wouter Boomsma |
  | Author | Peter Røgen |
  | Author | Thomas Hamelryck |
  | URL | http://bioinformatics.oxfordjournals.org/content/28/4/510.short |
  | Volume | 28 |
  | Issue | 4 |
  | Pages | 510–515 |
  | Publication | Bioinformatics |
  | Date | 2012 |
  | Accessed | 9/20/2013, 1:13:08 PM |
  | Library Catalog | Google Scholar |
  | Date Added | 10/11/2013, 10:29:15 AM |
  | Modified | 3/7/2014, 12:14:47 PM |

  ### Tags:

  - ASTRAL
  - ASTRAL domain structures

  ### Notes:

  - Present Pleiades, a novel approach for clustering protein structures.

    How SCOP is used:

    Levels filtered on: class, family

    Performed clustering on every entry in SCOP and found that all-alpha and all-beta were well separated, and a+b and a/b were in the middle.

    Also evaluated the clustering method on all SCOP domains against SCOP itself, using sensitivity and specificity equations to measure the correctly clustered structures.

    How CATH is used:

    Use CATH domain data for tuning parameter values.  Compared  length and Gauss integral values for CATH domains and found they correlated.  Do not say why they used CATH instead of SCOP.

    SCOP reference:

    Under Discussion:

    In a second test, we investigate whether GIT can detect structural similarities in a diverse set of protein structures, in terms of both fold and protein length. First, we converted the entire Structural Classification of Proteins (SCOP) (Murzin et al., 1995) database into GIT vectors. We then removed all families with 30 or less members. The final dataset contained GIT vectors calculated from 52 876 structures. Figure 2 shows a projection of the SCOP dataset of GIT vectors after principal component analysis (Bishop, 2006). As expected, the all α and all β fold classes are indeed well separated, with the mixed α and β classes located in between the two.

    ...

    3.2 Rebuilding the SCOP hierarchy

    The SCOP database organizes known protein structures into a hierarchy describing the folds (Murzin et al., 1995). The SCOP database is organized in four hierarchy levels—class, fold, superfamily and family—and contains a total of 110 800 domains from 38 221 Protein Data Bank (PDB) (Berman et al., 2000) entries in version v1.75 from June 2009.

    In order to illustrate the capabilities of Pleiades, we start with an evaluation that involves a large number of protein structures and a clear biologically relevant goal: the automated detection of protein folds. More specifically, we compared the results of our clustering method with the fold classification in the SCOP database (Murzin et al., 1995).

    We extracted and converted all domains present in the current release of SCOP into GIT vectors. For the clustering test, we limited ourselves to domains from the main SCOP classes all α, all β, α+β and α/β, since multichain complexes as well as small peptides are beyond the scope of this algorithm. We further removed very small families, with less than five members, and structures with problems in the conversion, for example due to missing atoms. In total, we included 63 864 domains from 1436 families and 823 superfamilies.

    In order to investigate the quality of the clustering, we calculated the sensitivity, specificity, correct classification rate (CCR) and the adjusted Rand index.

    ....

    We considered a structure as correctly clustered when it was assigned to the same cluster as the majority of the members of the same family.

    CATH reference:

    Pleiades uses an enhanced version of the measure called the tuned Gauss integral (GIT). The inspection of 24000 high-resolution domains from the CATH database (Orengo et al., 1997) revealed both a correlation between different Gauss integrals as well as a dependency on the length of the amino acid chain. This analysis led to an empirical correction factor, reducing the length dependency, that is applied to each Gauss integral. Moreover, some Gauss integrals can be predicted from lower order integrals and the length of the protein. In these cases, the estimated value is removed to limit the internal correlation. Lastly, calculating a smoothed curve from the otherwise rugged Cα trace further decreases the correlation between different Gauss integrals and improves the signal to noise ratio. Overall, the use of the GIT measure leads to a significantly better correlation with the RMSD, especially when the compared proteins have different lengths (Røgen, 2005).

  ### Attachments

  - Full Text PDF
- ## Fast learning optimized prediction methodology (FLOPRED) for protein secondary structure prediction

  |  |  |
  | --- | --- |
  | Type | Journal Article |
  | Author | S. Saraswathi |
  | Author | J. L. Fernandez-Martinez |
  | Author | A. Kolinski |
  | Author | R. L. Jernigan |
  | Author | A. Kloczkowski |
  | Volume | 18 |
  | Issue | 9 |
  | Pages | 4275-4289 |
  | Publication | Journal of Molecular Modeling |
  | ISSN | 1610-2940 |
  | Date | SEP 2012 |
  | Extra | WOS:000308114000027 |
  | DOI | 10.1007/s00894-012-1410-7 |
  | Abstract | Computational methods are rapidly gaining importance in the field of structural biology, mostly due to the explosive progress in genome sequencing projects and the large disparity between the number of sequences and the number of structures. There has been an exponential growth in the number of available protein sequences and a slower growth in the number of structures. There is therefore an urgent need to develop computational methods to predict structures and identify their functions from the sequence. Developing methods that will satisfy these needs both efficiently and accurately is of paramount importance for advances in many biomedical fields, including drug development and discovery of biomarkers. A novel method called fast learning optimized prediction methodology (FLOPRED) is proposed for predicting protein secondary structure, using knowledge-based potentials combined with structure information from the CATH database. A neural network-based extreme learning machine (ELM) and advanced particle swarm optimization (PSO) are used with this data that yield better and faster convergence to produce more accurate results. Protein secondary structures are predicted reliably, more efficiently and more accurately using FLOPRED. These techniques yield superior classification of secondary structure elements, with a training accuracy ranging between 83 % and 87 % over a widerange of hidden neurons and a cross-validated testing accuracy ranging between 81 % and 84 % and a segment overlap (SOV) score of 78 % that are obtained with different sets of proteins. These results are comparable to other recently published studies, but are obtained with greater efficiencies, in terms of time and cost. |
  | Date Added | 2/13/2014, 4:13:17 PM |
  | Modified | 3/7/2014, 12:11:10 PM |
- ## Fast protein binding site comparisons using visual words representation

  |  |  |
  | --- | --- |
  | Type | Journal Article |
  | Author | Bin Pang |
  | Author | Nan Zhao |
  | Author | Dmitry Korkin |
  | Author | Chi-Ren Shyu |
  | URL | http://bioinformatics.oxfordjournals.org/content/28/10/1345.short |
  | Volume | 28 |
  | Issue | 10 |
  | Pages | 1345–1352 |
  | Publication | Bioinformatics |
  | Date | 2012 |
  | Accessed | 9/23/2013, 10:16:21 AM |
  | Library Catalog | Google Scholar |
  | Date Added | 10/11/2013, 10:29:15 AM |
  | Modified | 10/11/2013, 10:29:15 AM |

  ### Notes:

  - Present PDBword, a method for comparisons of protein binding sites using "visual words".

    Use the SCOPPI database, which provides SCOP-type classification of protein-protein interfaces based on SCOP family, and sequence and structural similarity of binding sites.

    How SCOP data is used:

    Use a nonredundant data set from SCOPPI, which is a database of PPIs based on SCOP.  Use a dataset of PPIs generated, then split into three categorized based on whether they're in the same or different SCOP families, and whether the binding sites are in different function groups.

    SCOP Reference:

    Particularly, the SCOPPI database provides an evolutionary and structural classification of PPIs based on SCOP family (Murzin et al., 1995), sequence similarity and geometric features of binding sites.

    3 RESULTS

    ...

    3.1 Protein binding site classification and retrieval

    The dataset used in this experiment, denoted as D1, is a non- redundant dataset of protein binding sites extracted from SCOPPI 1.69 and has been used to evaluate the performance of MI. Dataset D1 consists of 2819 protein binding sites clustered into 501 groups, as determined in Sommer et al. (2007). The query dataset, denoted as Q1, includes 224 binding sites from 53 groups that are selected from D1 by applying a structural alignment tool, TM-align (Zhang and Skolnick, 2005), to ensure the similarity score (i.e. TM-score) among the binding sites within one group was >0.45 [for a more detailed description of D1, see (Sommer et al., 2007)].

    4 STUDYING PROTEIN STRUCTURES FROM A GEOMETRIC PERSPECTIVE

    We have shown that PBSword can identify geometrically similar protein binding sites from the same SCOP family based on surface shape features. As the molecular shape has long been recognized as a key factor in protein–protein interactions, we further investigate whether PBSword can discover non-trivial biological connections among proteins from a geometric perspective. In this section, we first study whether our approach can help us to investigate the relationships between the geometrically similar shapes of protein binding sites participating in an interaction and the functions carried out by the interactions. Specifically, we use our approach to first retrieve geometrically similar binding sites for a ‘seed’ binding site A, and then select the top-ranked binding site B to analyze the functional similarity between the corresponding proteins. The binding partners of A and B are denoted as A′ and B′, respectively. We consider two cases: (i) A and B are from same SCOP family, whereas A′ and B′ are from different families; and (ii) A, A′, B and B′ are all from different SCOP families. We then study the relationships between the shapes of protein binding sites and functional diversity within a SCOP family. Intuitively, proteins from the same family are expected to be structurally similar and have related functions. Discovering a protein binding site from such a family would not be very biologically significant, since the binding sites from the structurally similar proteins are expected to be similar and clustered together. What would be more interesting would be the discovery of two protein binding sites which are from the same family, but have dissimilar geometric shapes and different molecular functions. For this study, we consider another case: (iii) A, A′, B and B′ are from the same SCOP family but belong to different functional groups. In this case, the binding site B is not the top-ranked, but the highest ranking result from the same family. The schematic representations for the three cases are shown in Figure 4.

  ### Attachments

  - Full Text PDF
- ## Fast Protein Binding Site Comparison via an Index-Based Screening Technology

  |  |  |
  | --- | --- |
  | Type | Journal Article |
  | Author | Mathias M. von Behren |
  | Author | Andrea Volkamer |
  | Author | Angela M. Henzler |
  | Author | Karen T. Schomburg |
  | Author | Sascha Urbaczek |
  | Author | Matthias Rarey |
  | Volume | 53 |
  | Issue | 2 |
  | Pages | 411-422 |
  | Publication | Journal of Chemical Information and Modeling |
  | ISSN | 1549-9596 |
  | Date | FEB 2013 |
  | Extra | WOS:000315478900011 |
  | Journal Abbr | J. Chem Inf. Model. |
  | DOI | 10.1021/ci300469h |
  | Library Catalog | ISI Web of Knowledge |
  | Language | English |
  | Abstract | We present TrixP, a new index-based method for fast protein binding site comparison and function prediction. TrixP determines binding site similarities based on the comparison of descriptors that encode pharmacophoric and spatial features. Therefore, it adopts the efficient core components of TrixX, a structure-based virtual screening technology for large compound libraries. TrixP expands this Nor technology by new components in order to allow a screening of protein libraries. TrixP accounts for the inherent flexibility of proteins employing a partial shape matching routine. After the identification of structures with matching pharmacophoric features and geometric shape, TrixP superimposes the binding sites and, finally, assesses their similarity according to the fit of pharmacophoric properties. TrixP is able to find analogies between closely and distantly related binding sites. Recovery rates of 81.8% for similar binding site pairs, assisted by rejecting rates of 99.5% for dissimilar pairs on a test data set containing 1331 pairs, confirm this ability. TrixP exclusively identifies members of the same protein family on top ranking positions out of a library consisting of 9802 binding sites. Furthermore, 30 predicted kinase binding sites can almost perfectly be classified into their known subfamilies. |
  | Date Added | 10/8/2014, 12:34:47 PM |
  | Modified | 10/8/2014, 1:32:42 PM |

  ### Tags:

  - active-sites
  - algorithm
  - database
  - druggability prediction
  - functional sites
  - pdb
  - server
  - shape
  - similarity
  - structural classification

  ### Attachments

  - ACS Full Text PDF w/ Links
  - ACS Full Text Snapshot
- ## Fast protein structure alignment using Gaussian overlap scoring of backbone peptide fragment similarity

  |  |  |
  | --- | --- |
  | Type | Journal Article |
  | Author | David W. Ritchie |
  | Author | Anisah W. Ghoorah |
  | Author | Lazaros Mavridis |
  | Author | Vishwesh Venkatraman |
  | URL | http://bioinformatics.oxfordjournals.org/content/28/24/3274.short |
  | Volume | 28 |
  | Issue | 24 |
  | Pages | 3274–3281 |
  | Publication | Bioinformatics |
  | Date | 2012 |
  | Accessed | 9/20/2013, 1:18:37 PM |
  | Library Catalog | Google Scholar |
  | Date Added | 10/11/2013, 10:29:15 AM |
  | Modified | 3/7/2014, 12:11:17 PM |

  ### Notes:

  - Introduce a new structure alignment method, KPax.

    How SCOP is used:

    Benchmark structure alignment method on ability to identify fold.  Use SCOP domain structures to evaluate KPax on 12 low-sequence identity pairs.  These domains were taken from previous studies of Sippl and Weiderstein (2008) and Gerstein and Levitt (1998).

    How CATH is used:

    Use CATH for training parameter values and also for benchmarking.

    SCOP references:

    2.7 Searching structural domain databases

    To allow efficient queries against structural databases such as CATH or SCOP (Murzin et al., 1995), we first pre-calculate and store the up-stream and down-stream fragment coordinates for every database residue (i.e. 6 C⬚⬚ and 6 VA coordinates per residue).

    3.3 Comparing the local and spatial scoring functions

    To investigate the strengths and weaknesses of the Kpax scoring functions, we compared the performance of Kpax’s local, spatial and local-plus-spatial scores with TM-Align using six low se- quence identity pairs of domains identified previously by Sippl and Wiederstein (2008) and six further pairs from Gerstein and Levitt (1998).

    CATH reference:

    To obtain suitable values for the parameters ⬚⬚k, we treat each ⬚⬚ as the

    standard deviation (SD) of a normal Gaussian distribution, and by con-

    sidering each residue in turn of each domain in the CATH database (Cuff

    et al., 2009), we calculated the mean and SDs of all residues at relative

    positions ⬚⬚1 to ⬚⬚3 with respect to the residue under consideration to

    obtain the values: ⬚⬚þ1 1⁄4 1.46, ⬚⬚⬚⬚1 1⁄4 1.03, ⬚⬚þ2 1⁄4 3.72, ⬚⬚⬚⬚2 1⁄4 3.54,

    ⬚⬚þ3 1⁄4 5.52 and ⬚⬚⬚⬚3 1⁄4 5.74.

  ### Attachments

  - Full Text PDF
- ## Feature Selection of Protein Structural Classification Using SVM Classifier

  |  |  |
  | --- | --- |
  | Type | Journal Article |
  | Author | Zbigniew Krajewski |
  | Author | Ewaryst Tkacz |
  | URL | http://www.sciencedirect.com/science/article/pii/S020852161370055X |
  | Volume | 33 |
  | Issue | 1 |
  | Pages | 47–61 |
  | Publication | Biocybernetics and Biomedical Engineering |
  | Date | 2013 |
  | Accessed | 9/23/2013, 10:24:56 AM |
  | Library Catalog | Google Scholar |
  | Date Added | 10/11/2013, 10:29:15 AM |
  | Modified | 10/11/2013, 10:29:15 AM |

  ### Tags:

  - feature selection
  - principal component analysis
  - pseudo amino acid composition
  - recursive feature elimination
  - SCOP database
  - support vector machine

  ### Notes:

  - Computational studies of best features to use in an SVM for rebuilding the SCOP database.

    How SCOP is used:

    Train, test, and validate their method on a non-redundant subset (<30% sequence identity) of SCOP domains.

    SCOP reference:

    2.1. Data Set

    SCOP approach based on sequence identity and structure similarity seems to be the most reliable and comprehensive. The SCOP database is organized in several levels of so called evolutionary hierarchy with the main structural classes on the top [17, 18]. The domain as a basic classification entity was used as proposed by Murzin from the SCOP database based on structural and sequential similarity and so called evolutionary relationship [19].

    The data were split into three data pools: training, test and validation. The classic 30% of paired identity threshold of significant homology was applied to avoid data redundancy and compare to the other application [20]. The composition of amino acids (AAC) and pseudo composition (PseAA) are applied as features of classifica- tion tests [21–29].

  ### Attachments

  - 1-s2.0-S020852161370055X-main.pdf
  - Snapshot

    Recursive feature elimination method (RFE), cross validation coefficient (CV) and accuracy of classification of test data are applied as a criterion of feature selection in order to find relevant features and to analyze their influence on classifier accuracy. Feature selection method was compared to principal component analysis (PCA) to understand the effectiveness of feature reduction. Support vector machine classifier with radial basis function (RBF) kernel is applied to find the best set of features using grid model selection and to select and assess relevant features. The best selected feature set is then analyzed and interpreted as the source of knowledge about the protein structure and biochemical properties of amino acids included in the protein domain sequence.
- ## Ferredoxin:thioredoxin reductase (FTR) links the regulation of oxygenic photosynthesis to deeply rooted bacteria

  |  |  |
  | --- | --- |
  | Type | Journal Article |
  | Author | Monica Balsera |
  | Author | Estefania Uberegui |
  | Author | Dwi Susanti |
  | Author | Ruth A. Schmitz |
  | Author | Biswarup Mukhopadhyay |
  | Author | Peter Schuermann |
  | Author | Bob B. Buchanan |
  | Volume | 237 |
  | Issue | 2 |
  | Pages | 619-635 |
  | Publication | Planta |
  | ISSN | 0032-0935 |
  | Date | FEB 2013 |
  | Extra | WOS:000314062500021 |
  | DOI | 10.1007/s00425-012-1803-y |
  | Abstract | Uncovered in studies on photosynthesis 35 years ago, redox regulation has been extended to all types of living cells. We understand a great deal about the occurrence, function, and mechanism of action of this mode of regulation, but we know little about its origin and its evolution. To help fill this gap, we have taken advantage of available genome sequences that make it possible to trace the phylogenetic roots of members of the system that was originally described for chloroplasts-ferredoxin, ferredoxin:thioredoxin reductase (FTR), and thioredoxin as well as target enzymes. The results suggest that: (1) the catalytic subunit, FTRc, originated in deeply rooted microaerophilic, chemoautotrophic bacteria where it appears to function in regulating CO2 fixation by the reverse citric acid cycle; (2) FTRc was incorporated into oxygenic photosynthetic organisms without significant structural change except for addition of a variable subunit (FTRv) seemingly to protect the Fe-S cluster against oxygen; (3) new Trxs and target enzymes were systematically added as evolution proceeded from bacteria through the different types of oxygenic photosynthetic organisms; (4) an oxygenic type of regulation preceded classical light-dark regulation in the regulation of enzymes of CO2 fixation by the Calvin-Benson cycle; (5) FTR is not universally present in oxygenic photosynthetic organisms, and in certain early representatives is seemingly functionally replaced by NADP-thioredoxin reductase; and (6) FTRc underwent structural diversification to meet the ecological needs of a variety of bacteria and archaea. |
  | Date Added | 2/13/2014, 4:13:41 PM |
  | Modified | 3/7/2014, 1:06:52 PM |
- ## Finding Protein Targets for Small Biologically Relevant Ligands across Fold Space Using Inverse Ligand Binding Predictions

  |  |  |
  | --- | --- |
  | Type | Journal Article |
  | Author | Gang Hu |
  | Author | Jianzhao Gao |
  | Author | Kui Wang |
  | Author | Marcin J. Mizianty |
  | Author | Jishou Ruan |
  | Author | Lukasz Kurgan |
  | Volume | 20 |
  | Issue | 11 |
  | Pages | 1815-1822 |
  | Publication | Structure |
  | ISSN | 0969-2126 |
  | Date | NOV 7 2012 |
  | DOI | 10.1016/j.str.2012.09.011 |
  | Language | English |
  | Abstract | Inverse ligand binding prediction utilizes a few protein-ligand (drug) complexes to predict other secondary therapeutic and off-targets of a given drug molecule on a proteomic scale. We adapt two binding site predictors, FINDSITE and SMAP, to perform the inverse predictions and evaluate them on over 30 representative ligands. Use of just one complex allows the identification of other protein targets; the availability of additional complexes improves the results. Both methods offer comparable quality when using three complexes with diverse proteins. SMAP is better when fewer complexes are available, while FINDSITE provides stronger predictions for smaller ligands. We propose a consensus that combines (and outperforms) the two complementary approaches implemented by FINDSITE and SMAP. Most importantly, we demonstrate that these methods successfully find distant targets that belong to structurally different folds compared to the proteins in the input complexes. |
  | Date Added | 10/11/2013, 10:29:15 AM |
  | Modified | 12/2/2013, 4:17:56 PM |

  ### Notes:

  - Adapt two binding-site predictor programs, FINDSITE and SMAP, to instead screen for proteins to bind a set of 30 ligands.

    How SCOP is used:

    Use SCOP to help evaluate how well the methods detect ligand-binding targets that are distant (belong to different folds).

    Classify a data set of ligand-binding proteins by SCOP class and fold.

    SCOP reference:

    We select three representative biologically relevant small organic ligands, NAG, ADP, and PLM, to perform detailed evaluation on a proteomic scale on two types of well-designed ligand-spe- cific benchmark data sets: a redundant data set that in- cludes all known ligand-binding proteins, and a nonredundant data set that includes a subset of diverse (in both sequence and structure) ligand-binding targets. Both data sets also include proteins that are unlikely to bind a given ligand and we use SCOP hierarchy (Murzin et al., 1995; Andreeva et al., 2008) to evaluate predictive quality when finding distant (low homology) targets, i.e., targets that belong to different folds compared to the proteins in the input/template complexes.

    Inverse Ligand Binding Predictions across the Fold Space 

    As discussed in a number of studies (Xie and Bourne, 2008; Nobeli et al., 2009; Petrey et al., 2009; Zhang et al., 2010a), the same ligand may have protein part- ners that belong to substantially different folds. This motivates an evaluation of the ability of the considered inverse ligand binding predictors to find struc- turally distant (belonging to a substantially different fold compared to the template proteins) binding proteins.

    To this end, we constructed four subsets of the redundant data sets based on the SCOP annotations (Murzin et al., 1995; Andreeva et al., 2008), with the same or different SCOP classes or SCOP folds when compared with the template protein; proteins that lack SCOP annotations were removed from this evaluation. The SCOP-annotated benchmark data sets include 957 posi- tive and 383 negative proteins for NAG, 773 positive and 250 negative for ADP, and 37 positive and 75 negative for PLM. For each template complex that constitutes input to a given inverse ligand binding prediction method, we con- structed the four subsets of the SCOP-annotated benchmark data sets. The first subset includes the proteins that are in the same SCOP class as the template protein. The second subset includes the proteins that are in different SCOP classes compared with the class of the template protein. Analogously, the third (fourth) subsets include proteins from the same (different) SCOP fold compared to the fold of the input template proteins.

  ### Attachments

  - 1-s2.0-S0969212612003450-main.pdf
- ## Finding rigid bodies in protein structures: Application to flexible fitting into cryoEM maps

  |  |  |
  | --- | --- |
  | Type | Journal Article |
  | Author | Arun Prasad Pandurangan |
  | Author | Maya Topf |
  | Volume | 177 |
  | Issue | 2 |
  | Pages | 520-531 |
  | Publication | Journal of Structural Biology |
  | ISSN | 1047-8477 |
  | Date | FEB 2012 |
  | Extra | WOS:000300755400039 |
  | DOI | 10.1016/j.jsb.2011.10.011 |
  | Abstract | We present RIBFIND, a method for detecting flexibility in protein structures via the clustering of secondary structural elements (SSEs) into rigid bodies. To test the usefulness of the method in refining atomic structures within cryoEM density we incorporated it into our flexible fitting protocol (Flex-EM). Our benchmark includes 13 pairs of protein structures in two conformations each, one of which is represented by a corresponding cryoEM map. Refining the structures in simulated and experimental maps at the 5-15 angstrom resolution range using rigid bodies identified by RIBFIND shows a significant improvement over using individual SSEs as rigid bodies. For the 15 A resolution simulated maps, using RIBFIND-based rigid bodies improves the initial fits by 40.64% on average, as compared to 26.52% when using individual SSEs. Furthermore, for some test cases we show that at the sub-nanometer resolution range the fits can be further improved by applying a two-stage refinement protocol (using RIBFIND-based refinement followed by an SSE-based refinement). The method is stand-alone and could serve as a general interactive tool for guiding flexible fitting into EM maps. (C) 2011 Elsevier Inc. All rights reserved. |
  | Date Added | 2/13/2014, 4:13:17 PM |
  | Modified | 3/7/2014, 12:14:10 PM |
- ## Fitting hidden Markov models of protein domains to a target species: application to Plasmodium falciparum

  |  |  |
  | --- | --- |
  | Type | Journal Article |
  | Author | Nicolas Terrapon |
  | Author | Olivier Gascuel |
  | Author | Éric Maréchal |
  | Author | Laurent Bréhélin |
  | URL | http://www.biomedcentral.com/1471-2105/13/67/ |
  | Volume | 13 |
  | Issue | 1 |
  | Pages | 67 |
  | Publication | BMC bioinformatics |
  | Date | 2012 |
  | Accessed | 9/20/2013, 1:19:04 PM |
  | Library Catalog | Google Scholar |
  | Short Title | Fitting hidden Markov models of protein domains to a target species |
  | Date Added | 10/11/2013, 10:29:15 AM |
  | Modified | 10/11/2013, 10:29:15 AM |

  ### Tags:

  - Algorithms
  - Amino Acid Motifs
  - Databases, Protein
  - Markov Chains
  - Molecular Sequence Annotation
  - Plasmodium falciparum
  - Proteins
  - Proteome
  - Proteomics
  - Sequence Alignment
  - Software

  ### Notes:

  - Present method to customize HMMs for domain detection to species.  In spite of their high specificity, HMMs may lack sensitivity when searching for domains in divergent organisms. 

    How SCOP is used:

    Not using SCOP data. 

    Mention SCOP when providing motivation for using HMMs for domain prediction.

    SCOP references:

    Background:

    approaches have been developed to define and identify protein domains. Some are based on a structural clas- sification scheme [2],

  ### Attachments

  - 1471-2105-13-67.pdf

    <div class="page" title="Page 1">
    <div class="layoutArea"></div>
    </div>
- ## Folding Mechanism of an Extremely Thermostable (beta alpha)(8)-Barrel Enzyme: A High Kinetic Barrier Protects the Protein from Denaturation

  |  |  |
  | --- | --- |
  | Type | Journal Article |
  | Author | Linn Carstensen |
  | Author | Gabriel Zoldak |
  | Author | Franz-Xaver Schmid |
  | Author | Reinhard Sterner |
  | Volume | 51 |
  | Issue | 16 |
  | Pages | 3420-3432 |
  | Publication | Biochemistry |
  | ISSN | 0006-2960 |
  | Date | APR 24 2012 |
  | DOI | 10.1021/bi300189f |
  | Language | English |
  | Abstract | HisF, the cyclase subunit of imidazole glycerol phosphate synthase (ImGPS) from Thermotoga maritima, is an extremely thermostable (beta alpha)(8)-barrel protein. We elucidated the unfolding and refolding mechanism of HisF. Its unfolding transition is reversible and adequately described by the two-state model, but 6 weeks is necessary to reach equilibrium (at 25 degrees C). During refolding, initially a burst-phase off-pathway intermediate is formed. The subsequent productive folding occurs in two kinetic phases with time constants of similar to 3 and similar to 20 s. They reflect a sequential process via an on-pathway intermediate, as revealed by stopped-flow double-mixing experiments. The final step leads to native HisF, which associates with the glutaminase subunit HisH to form the functional ImGPS complex. The conversion of the on-pathway intermediate to the native protein results in a 10(6)-fold increase of the time constant for unfolding from 89 ms to 35 h (at 4.0 M GdmCl) and thus establishes a high energy barrier to denaturation. We conclude that the extra stability of HisF is used for kinetic protection against unfolding. In its refolding mechanism, HisF resembles other (beta alpha)(8)-barrel proteins. |
  | Date Added | 10/11/2013, 10:29:15 AM |
  | Modified | 11/11/2013, 3:47:59 PM |

  ### Notes:

  - Study folding mechanism of an enzyme with (B alpha)-barrel fold using experimental methods.

    How SCOP is used:

    Use case: website.

    Description: General reference to SCOP to point out prominence of a particular fold.  Note that "Approximately 10% of all proteins with known three-dimensional structure contain at least one (βα)8-barrel domain."

    SCOP reference:

    Approximately 1200 different protein folds have been identified to date [Structural Classification of Proteins (SCOP) release 1.75, February 2009],1 each of them being characterized by a distinct toplogical orientation of secondary structure elements. Whereas many folds are represented by only a few members of the protein database, others have been recruited extensively in the course of evolution. A prominent example is the (βα)8-barrel, which is among the most ancient, frequent, and versatile folds.2−4 Approximately 10% of all proteins with known three-dimensional structure contain at least one (βα)8-barrel domain. With very few exceptions, all known (βα)8-barrels are enzymes, and SCOP distinguishes 33 superfamilies that catalyze more than 60 different reactions. They occur in five of the six Enzyme Commission (EC) classes, acting as oxidoreductases, transferases, lyases, hydrolases, and isomerases, and many of them are engaged in essential metabolic pathways.5,6

  ### Attachments

  - bi300189f.pdf
- ## Folding of an all-helical Greek-key protein monitored by quenched-flow hydrogen-deuterium exchange and NMR spectroscopy

  |  |  |
  | --- | --- |
  | Type | Journal Article |
  | Author | Lesley H. Greene |
  | Author | Hai Li |
  | Author | Junyan Zhong |
  | Author | Guoxia Zhao |
  | Author | Khym Wilson |
  | Volume | 41 |
  | Issue | 1 |
  | Pages | 41–51 |
  | Publication | European Biophysics Journal With Biophysics Letters |
  | Date | January 2012 |
  | DOI | 10.1007/s00249-011-0756-6 |
  | Abstract | To advance our understanding of the protein folding process, we use stopped-flow far-ultraviolet (far-UV) circular dichroism and quenched-flow hydrogen-deuterium exchange coupled with nuclear magnetic resonance (NMR) spectroscopy to monitor the formation of hydrogen-bonded secondary structure in the C-terminal domain of the Fas-associated death domain (Fadd-DD). The death domain superfamily fold consists of six alpha-helices arranged in a Greek-key topology, which is shared by the all-beta-sheet immunoglobulin and mixed alpha/beta-plait superfamilies. Fadd-DD is selected as our model death domain protein system because the structure of this protein has been solved by NMR spectroscopy, and both thermodynamic and kinetic analysis indicate it to be a stable, monomeric protein with a rapidly formed hydrophobic core. Stopped-flow far-UV circular dichroism spectroscopy revealed that the folding process was monophasic and the rate is 23.4 s(-1). Twenty-two amide hydrogens in the backbone of the helices and two in the backbone of the loops were monitored, and the folding of all six helices was determined to be monophasic with rate constants between 19 and 22 s(-1). These results indicate that the formation of secondary structure is largely cooperative and concomitant with the hydrophobic collapse. This study also provides unprecedented insight into the formation of secondary structure within the highly populated Greek-key fold more generally. Additional insights are gained by calculating the exchange rates of 23 residues from equilibrium hydrogen-deuterium exchange experiments. The majority of protected amide protons are found on helices 2, 4, and 5, which make up core structural elements of the Greek-key topology. |
  | Date Added | 3/7/2014, 12:08:00 PM |
  | Modified | 3/7/2014, 12:08:00 PM |
- ## Folding of multidomain proteins: Biophysical consequences of tethering even in apparently independent folding

  |  |  |
  | --- | --- |
  | Type | Journal Article |
  | Author | Oshrit Arviv |
  | Author | Yaakov Levy |
  | URL | http://onlinelibrary.wiley.com/doi/10.1002/prot.24161/full |
  | Volume | 80 |
  | Issue | 12 |
  | Pages | 2780–2798 |
  | Publication | Proteins: Structure, Function, and Bioinformatics |
  | Date | 2012 |
  | Accessed | 9/23/2013, 10:14:36 AM |
  | Library Catalog | Google Scholar |
  | Short Title | Folding of multidomain proteins |
  | Date Added | 10/11/2013, 10:29:15 AM |
  | Modified | 10/11/2013, 10:29:15 AM |

  ### Tags:

  - coarse-grained simulation
  - conjugated protein
  - energy landscape
  - flexible linker
  - multidomain protein

  ### Notes:

  - Study of how "tethering" affects the (apparently) independent folding of protein domains in multi-domain chains.

    Use course-grained molecular dynamics to study folding in two two-domain proteins.

    How used SCOP:

    Didn't use SCOP data.  Refer to SCOP when defining protein domains.

    Reference to SCOP:

    Although a unique, consistent definition of a protein domain remains elusive,6 it is frequently referred to as ‘‘a structural, functional, and evolutionary component of proteins, which can often be expressed as a single unit.’’7,8 Therefore, domains sharing functional and structural features suggesting a common evolutionary origin can be grouped into superfamilies.7

  ### Attachments

  - [PDF] from weizmann.ac.il
  - Snapshot
- ## Folding Properties of Cytosine Monophosphate Kinase from E-coli Indicate Stabilization through an Additional Insert in the NMP Binding Domain

  |  |  |
  | --- | --- |
  | Type | Journal Article |
  | Author | Thorsten Beitlich |
  | Author | Thorsten Lorenz |
  | Author | Jochen Reinstein |
  | Volume | 8 |
  | Issue | 10 |
  | Pages | e78384 |
  | Publication | Plos One |
  | ISSN | 1932-6203 |
  | Date | OCT 30 2013 |
  | Extra | WOS:000326334500114 |
  | DOI | 10.1371/journal.pone.0078384 |
  | Abstract | The globular 25 kDa protein cytosine monophosphate kinase (CMPK, EC ID: 2.7.4.14) from E. coli belongs to the family of nucleoside monophosphate (NMP) kinases (NMPK). Many proteins of this family share medium to high sequence and high structure similarity including the frequently found alpha/beta topology. A unique feature of CMPK in the family of NMPKs is the positioning of a single cis-proline residue in the CORE-domain (cis-Pro124) in conjunction with a large insert in the NMP binding domain. This insert is not found in other well studied NMPKs such as AMPK or UMP/CMPK. We have analyzed the folding pathway of CMPK using time resolved tryptophan and FRET fluorescence as well as CD. Our results indicate that unfolding at high urea concentrations is governed by a single process, whereas refolding in low urea concentrations follows at least a three step process which we interpret as follows: Pro124 in the CORE-domain is in cis in the native state (N-c) and equilibrates with its trans-isomer in the unfolded state (U-c-U-t). Under refolding conditions, at least the U-t species and possibly also the U-c species undergo a fast initial collapse to form intermediates with significant amount of secondary structure, from which the trans-Pro124 fraction folds to the native state with a 100-fold lower rate constant than the cis-Pro124 species. CMPK thus differs from homologous NMP kinases like UMP/CMP kinase or AMP kinase, where folding intermediates show much lower content of secondary structure. Importantly also unfolding is up to 100-fold faster compared to CMPK. We therefore propose that the stabilizing effect of the long NMP-domain insert in conjunction with a subtle twist in the positioning of a single cis-Pro residue allows for substantial stabilization compared to other NMP kinases with alpha/beta topology. |
  | Date Added | 2/12/2014, 1:36:22 PM |
  | Modified | 2/12/2014, 1:36:22 PM |

  ### Notes:

  - Experimental study of folding kinetics of CMKP protein.

    How SCOP is used:

    Look up SCOP class classification of CMPK protein.

    SCOP reference:

    The 25 kDa protein CMP kinase from E. coli (CMPK) also belongs to this family and its structure alone and in complex with CDP was solved by Briozzo et al. [12] and classified as a/b- protein, including a P-loop motif, which is typical for these phosphoryl group transferring enzymes [24].

  ### Attachments

  - journal.pone.0078384.pdf
- ## FRAN and RBF-PSO as two components of a hyper framework to recognize protein folds

  |  |  |
  | --- | --- |
  | Type | Journal Article |
  | Author | Elham Abbasi |
  | Author | Mehdi Ghatee |
  | Author | M. E. Shiri |
  | Volume | 43 |
  | Issue | 9 |
  | Pages | 1182-1191 |
  | Publication | Computers in Biology and Medicine |
  | ISSN | 0010-4825 |
  | Date | SEP 1 2013 |
  | Extra | WOS:000324154700011 |
  | Journal Abbr | Comput. Biol. Med. |
  | DOI | 10.1016/j.compbiomed.2013.05.017 |
  | Library Catalog | ISI Web of Knowledge |
  | Language | English |
  | Abstract | In this paper, an intelligent hyper framework is proposed to recognize protein folds from its amino acid sequence which is a fundamental problem in bioinformatics. This framework includes some statistical and intelligent algorithms for proteins classification. The main components of the proposed framework are the Fuzzy Resource-Allocating Network (FRAN) and the Radial Bases Function based on Particle Swarm Optimization (RBF-PSO). FRAN applies a dynamic method to tune up the RBF network parameters. Due to the patterns complexity captured in protein dataset, FRAN classifies the proteins under fuzzy conditions. Also, RBF-PSO applies PSO to tune up the RBF classifier. Experimental results demonstrate that FRAN improves prediction accuracy up to 51% and achieves acceptable multi-class results for protein fold prediction. Although RBF-PSO provides reasonable results for protein fold recognition up to 48%, it is weaker than FRAN in some cases. However the proposed hyper framework provides an opportunity to use a great range of intelligent methods and can learn from previous experiences. Thus it can avoid the weakness of some intelligent methods in terms of memory, computational time and static structure. Furthermore, the performance of this system can be enhanced throughout the system life-cycle. (C) 2013 Elsevier Ltd. All rights reserved. |
  | Date Added | 10/8/2014, 12:34:47 PM |
  | Modified | 10/8/2014, 1:32:29 PM |

  ### Tags:

  - classification
  - classifiers
  - Instance-based method
  - Machine learning
  - mlp
  - neural-networks
  - particle swarm optimizer
  - prediction
  - Protein Fold Recognition
  - pso
  - rbf
  - sequence

  ### Attachments

  - ScienceDirect Full Text PDF
  - ScienceDirect Snapshot
- ## From Protein Structure to Function via Computational Tools and Approaches

  |  |  |
  | --- | --- |
  | Type | Journal Article |
  | Author | Rachel Kolodny |
  | Author | Mickey Kosloff |
  | Volume | 53 |
  | Issue | 3-4 |
  | Pages | 147-156 |
  | Publication | Israel Journal of Chemistry |
  | ISSN | 0021-2148; 1869-5868 |
  | Date | APR 2013 |
  | Extra | WOS:000317859800004 |
  | DOI | 10.1002/ijch.201200078 |
  | Abstract | The three-dimensional structures of proteins are often considered fundamental for understanding their function. Yet, because of the complexity of protein structure, extracting specific functional information from structures can be a considerable challenge. Here, we present selected approaches and tools that we have developed to study and connect protein sequence, structure, and function spaces. First, we consider a global perspective of structure space and view the protein data bank (PDB) as a database. We highlight challenges in searching protein structure space and in using the PDB as the starting point for computational structural studies. Then we describe a function-oriented view and show examples of how multiple protein structures can be used to extract insights about the function and specificity of proteins at the family level. |
  | Date Added | 2/13/2014, 4:13:17 PM |
  | Modified | 10/8/2014, 1:32:35 PM |

  ### Tags:

  - bioinformatics
  - molecular recognition
  - protein structures
  - structure-activity relationships

  ### Attachments

  - Snapshot
- ## FUGUE: sequence-structure homology recognition using environment-specific substitution tables and structure-dependent gap penalties

  |  |  |
  | --- | --- |
  | Type | Journal Article |
  | Author | Jiye Shi |
  | Author | Tom L Blundell |
  | Author | Kenji Mizuguchi |
  | URL | http://www.sciencedirect.com/science/article/pii/S002228360194762X |
  | Volume | 310 |
  | Issue | 1 |
  | Pages | 243-257 |
  | Publication | Journal of Molecular Biology |
  | ISSN | 0022-2836 |
  | Date | June 29, 2001 |
  | Journal Abbr | Journal of Molecular Biology |
  | DOI | 10.1006/jmbi.2001.4762 |
  | Accessed | 8/2/2013, 4:43:54 PM |
  | Library Catalog | ScienceDirect |
  | Abstract | FUGUE, a program for recognizing distant homologues by sequence-structure comparison (http://www-cryst.bioc.cam.ac.uk/fugue/), has three key features. (1) Improved environment-specific substitution tables. Substitutions of an amino acid in a protein structure are constrained by its local structural environment, which can be defined in terms of secondary structure, solvent accessibility, and hydrogen bonding status. The environment-specific substitution tables have been derived from structural alignments in the HOMSTRAD database (http://www-cryst.bioc.cam.ac.uk/homstrad/). (2) Automatic selection of alignment algorithm with detailed structure-dependent gap penalties. FUGUE uses the global-local algorithm to align a sequence-structure pair when they greatly differ in length and uses the global algorithm in other cases. The gap penalty at each position of the structure is determined according to its solvent accessibility, its position relative to the secondary structure elements (SSEs) and the conservation of the SSEs. (3) Combined information from both multiple sequences and multiple structures. FUGUE is designed to align multiple sequences against multiple structures to enrich the conservation/variation information. We demonstrate that the combination of these three key features implemented in FUGUE improves both homology recognition performance and alignment accuracy. |
  | Short Title | FUGUE |
  | Date Added | 10/11/2013, 10:29:15 AM |
  | Modified | 10/11/2013, 10:29:15 AM |

  ### Notes:

  - Fugue is a method for sequence-structure homology recognition.

    How SCOP is used:

    Evaluated method on datasets derived from SCOP.  Validated on Fold,Superfamily, and Family levels.  Used the HOMSTRAD database, rather than the

    Derived a few different benchmark data sets from SCOP data.  Derived their data set from Lindahl's test-set", published in a previous paper, which derived a non-redundant test of 976 sequences from SCOP 1.37.

    SCOP references:

    Under Introduction:

    According to the SCOP database,2,3 where proteins are classifieded hierarchically to reflect both structural and evolutionary relationships, proteins in the same family have ``clear evolutionary relationship''; proteins that share only the same superfamily have ``probable common evolutionary origin''; those classi®ed as sharing only the same fold are of ``major structure similarity'' and mostly ``do not have evolutionary relationships''.4 Thus, sequence-structure homology recognition is differ- ent from fold recognition: the former aims at recog- nising the similarity at both family and superfamily levels while the latter covers all three levels, with particular emphasis on the fold level.

    Under Benchmarking:

    *Recognition performance:* To compare the sequence-structure homology recognition performance of FUGUE with other methods, we used the extensive benchmark23 derived from the SCOP database.

    *Multiple-structure profiles versus single- structure profiles:* The performances of profiles derived from mul- tiple structures and of those derived from single structures were compared using the SCOP-HOM- STRAD test-set, which was derived as follows. Firstly, 597 representative SCOP domains, which belong to different SCOP families, were selected from Lindahl's benchmark.23 These domains were then mapped onto families in the HOMSTRAD database. The mapping is successful only if the domain definition in the HOMSTRAD family is consistent with the SCOP domain and at least one of the members in the HOMSTRAD family has significant sequence similarity to the SCOP domain (BLAST,7 E-value <0.001). In total, 209 HOM- STRAD families were mapped and profiles gener- ated for both the mapped SCOP domains and the mapped HOMSTRAD multiple structure align- ments. Finally, we used PSI-BLAST alignments of the sequences of those 597 SCOP domains to search against two structure libraries of the same size: one for single-structure pro®les and the other for multiple-structure ones. Performance was eval- uated as described above.

    *Stability of substitution tables*:  A subset (SCOP474), which consisted of 474 structures, was selected from Lindahl's test-set23 to perform a true jack-knife test. These structures were selected such that none of them shares obvious sequence similarity (BLAST, E-value <0.1) to any of the structures in either SUB177 or SUB371. The two sets of substitution tables derived from SUB177 and SUB371 were benchmarked on homology recognition performance using this 474 ⬚⬚ 474 test-set. PSI-BLAST was also tested.

  ### Attachments

  - ScienceDirect Full Text PDF
  - Snapshot
- ## Functional analysis of the distal region of the third intracellular loop of PROKR2

  |  |  |
  | --- | --- |
  | Type | Journal Article |
  | Author | Xiao-Tao Zhou |
  | Author | Dan-Na Chen |
  | Author | Zhi-Qun Xie |
  | Author | Zhen Peng |
  | Author | Kai-De Xia |
  | Author | Hua-Die Liu |
  | Author | Wei Liu |
  | Author | Bing Su |
  | Author | Jia-Da Li |
  | Volume | 439 |
  | Issue | 1 |
  | Pages | 12-17 |
  | Publication | Biochemical and Biophysical Research Communications |
  | ISSN | 0006-291X |
  | Date | SEP 13 2013 |
  | Extra | WOS:000324791600003 |
  | DOI | 10.1016/j.bbrc.2013.08.039 |
  | Abstract | Mutations in the G-protein-coupled receptor PROKR2 have been identified in patients with idiopathic hypogonadotropic hypogonadism (IHH) and Kallmann syndrome (KS) manifesting with delayed puberty and infertility. Recently, the homozygous mutation V274D was identified in a man displaying KS with an apparent reversal of hypogonadism. The affected amino acid, valine 274, is located at the junction region of the third intracellular loop (IL3) and the sixth transmembrane domain (TM6). In this study, we first studied the effect of V274D and related mutations (V274A, V274T, and V274R) on the signaling activity and cell surface expression of PROKR2. Our data indicate that a charged amino acid substitution at residue 274 of PROKR2 results in low cell surface expression and loss-of-function. Furthermore, we studied the effects of two clusters of basic amino acids located at the proximal region of Val274 on the cell surface expression and function of PROKR2. The deletion of RRK (270-272) resulted in undetectable cell surface expression, whereas RKR (264-266)-deleted PROKR2 was expressed normally on the cell surface but showed loss-of-function due to a deficiency in G-protein coupling. Our data indicate that the distal region of the IL3 of PROKR2 may differentially influence receptor trafficking and G-protein coupling. (c) 2013 Elsevier Inc. All rights reserved. |
  | Date Added | 2/12/2014, 2:18:08 PM |
  | Modified | 2/12/2014, 2:18:08 PM |

  ### Notes:

  - Experimental and computational study of region in the G-protein-coupled receptor PROKR2.

    How SCOP is used:

    Use Phyre2 and HHSearch to find remote homologs in SCOP for molecular modeling, and list the SCOP family classification for each of the six templates found.

    SCOP reference:

    2.7. Molecular modeling

    Because there was no homology structure with sequence iden- tity of at least 40% in the PDB database [24], the intensive mode of the Phyre2 server [25] was utilized to predict the three-dimen- sional structures of PROKR2. The Phyre2 server uses the alignment of hidden Markov models via HHsearch [26] to significantly im- prove the accuracy of alignment and the detection rate. The se- quences of WT and V274D PROKR2 were submitted to the Phyre2 server, and six templates (SCOP codes [27]: c2ksaA [human Substance-P receptor], c2rh1A [human b2-adrenergic receptor], c3emlA [Human Adenosine A2A receptor], c3uonA [human M2 muscarinic acetylcholine receptor], c4djhA [human j-type opioid receptor], and c3pdsA [human b2-adrenergic receptor]) were se- lected to model the protein based on heuristics to maximize the confidence, percentage identity, and alignment coverage.

  ### Attachments

  - 1-s2.0-S0006291X1301365X-main.pdf
- ## Functional Annotation of Conserved Hypothetical Proteins from Haemophilus influenzae Rd KW20

  |  |  |
  | --- | --- |
  | Type | Journal Article |
  | Author | Mohd. Shahbaaz |
  | Author | Md. Imtaiyaz Hassan |
  | Author | Faizan Ahmad |
  | Volume | 8 |
  | Issue | 12 |
  | Pages | e84263 |
  | Publication | Plos One |
  | Date | December 2013 |
  | DOI | 10.1371/journal.pone.0084263 |
  | Abstract | Haemophilus influenzae is a Gram negative bacterium that belongs to the family Pasteurellaceae, causes bacteremia, pneumonia and acute bacterial meningitis in infants. The emergence of multi-drug resistance H. influenzae strain in clinical isolates demands the development of better/new drugs against this pathogen. Our study combines a number of bioinformatics tools for function predictions of previously not assigned proteins in the genome of H. influenzae. This genome was extensively analyzed and found 1,657 functional proteins in which function of 429 proteins are unknown, termed as hypothetical proteins (HPs). Amino acid sequences of all 429 HPs were extensively annotated and we successfully assigned the function to 296 HPs with high confidence. We also characterized the function of 124 HPs precisely, but with less confidence. We believed that sequence of a protein can be used as a framework to explain known functional properties. Here we have combined the latest versions of protein family databases, protein motifs, intrinsic features from the amino acid sequence, pathway and genome context methods to assign a precise function to hypothetical proteins for which no experimental information is available. We found these HPs belong to various classes of proteins such as enzymes, transporters, carriers, receptors, signal transducers, binding proteins, virulence and other proteins. The outcome of this work will be helpful for a better understanding of the mechanism of pathogenesis and in finding novel therapeutic targets for H. influenzae. |
  | Date Added | 3/7/2014, 12:08:00 PM |
  | Modified | 10/8/2014, 1:32:39 PM |

  ### Attachments

  - PLoS Full Text PDF
  - PLoS Snapshot
- ## Functional Determinants of Temperature Adaptation in Enzymes of Cold- versus Warm-Adapted Mussels (Genus Mytilus)

  |  |  |
  | --- | --- |
  | Type | Journal Article |
  | Author | Brent L. Lockwood |
  | Author | George N. Somero |
  | URL | http://mbe.oxfordjournals.org/content/29/10/3061 |
  | Volume | 29 |
  | Issue | 10 |
  | Pages | 3061-3070 |
  | Publication | Molecular Biology and Evolution |
  | ISSN | 0737-4038, 1537-1719 |
  | Date | 10/01/2012 |
  | Extra | PMID: 22491035 |
  | Journal Abbr | Mol Biol Evol |
  | DOI | 10.1093/molbev/mss111 |
  | Accessed | 9/20/2013, 11:23:15 AM |
  | Library Catalog | mbe.oxfordjournals.org |
  | Language | en |
  | Abstract | Temperature is a strong selective force on the evolution of proteins due to its effects on higher orders of protein structure and, thereby, on critical protein functions like ligand binding and catalysis. Comparisons among orthologous proteins from differently thermally adapted species show consistent patterns of adaptive variation in function, but few studies have examined functional adaptation among multiple structural families of proteins. Thus, with our present state of knowledge, it is difficult to predict what fraction of the proteome will exhibit adaptive variation in the face of temperature increases of a few to several degrees Celsius, that is, temperature increases of the magnitude predicted by models of global warming. Here, we compared orthologous enzymes of the warm-adapted Mediterranean mussel Mytilus galloprovincialis and the cold-adapted Mytilus trossulus, a native of the North Pacific Ocean, species whose physiologies exhibit significantly different responses to temperature. We measured the effects of temperature on the kinetics (Michaelis–Menten constant—Km) of five enzymes that are important for ATP generation and that represent distinct protein structural families. Among phosphoglucomutase (PGM), phosphoglucose isomerase (PGI), pyruvate kinase (PK), phosphoenolpyruvate carboxykinase (GTP) (PEPCK), and isocitrate dehydrogenase (NADP) (IDH), only IDH orthologs showed significantly different thermal responses of Km between the two species. The Km of isocitrate of M. galloprovincialis-IDH was intrinsically lower and more thermally stable than that of M. trossulus-IDH and thus had higher substrate affinity at high temperatures. Two amino acid substitutions account for the functional differences between IDH orthologs, one of which allows for more hydrogen bonds to form near the mobile region of the active site in M. galloprovincialis-IDH. Taken together, our findings cast light on the targets of adaptive evolution in the context of climate change; only a minority of proteins might adapt to small changes in temperature, and these adaptations may involve only small changes in sequence. |
  | Date Added | 10/11/2013, 10:29:15 AM |
  | Modified | 2/26/2014, 2:41:58 PM |

  ### Tags:

  - adaptation
  - adaptation
  - invasive species
  - invasive species
  - Michaelis–Menten constants (Km)
  - Michaelis-Menten constants (K-m)
  - Mytilus
  - Mytilus
  - protein evolution
  - Protein Evolution
  - structure–function relationships
  - structure-function relationships
  - temperature
  - temperature

  ### Notes:

  - Structural and functional adaptations in response to temperature environment have been somewhat well-studied in enzymes that share the NAD(P)-binding Rossman fold, but studies in other structural classes has been limited.  
      
    Presents a comparative study of thermophilic homologues in two mussel species from 5 different SCOP folds.  
      
    How SCOP is used:  
      
    Use SCOP to select 5 enzymes for a study that were "structurally diverse", each from a different fold.  
      
    SCOP references:  
    PGM    Monomer    PGM, first three domains  
    PGI    Homodimer    SIS domain  
    PK    Homotetramer    PK beta-barrel domain like  
    PEPCK    Monomer    PEP carboxykinase like  
    IDH    Homodimer    Isocitrate/isopropylmalate dehydrogenase like  
    cMDH    Homodimer    NAD(P)-binding Rossmann-fold domains  
      
       
    "The structure of IDH, characterized by an isocitrate/isopropylmalate dehydrogenase–like fold, is a structural family closely related to the NAD(P)-binding Rossmann-fold domain of cMDH and A4-LDH (Murzin et al. 1995; Fields and Somero 1998; Andreeva et al. 2004, 2008). This suggests that the similar trends in temperature adaptation for IDH and cMDH in Mytilus (Fields et al. 2006) and for A4-LDHs of differently adapted vertebrates (Fields and Somero 1998) are due to the shared aspects of their 3D structures."

  ### Attachments

  - Full Text PDF
- ## Functional inference by ProtoNet family tree: the uncharacterized proteome of Daphnia pulex

  |  |  |
  | --- | --- |
  | Type | Journal Article |
  | Author | Nadav Rappoport |
  | Author | Michal Linial |
  | Volume | 14 |
  | Publication | BMC bioinformatics |
  | ISSN | 1471-2105 |
  | Date | FEB 28 2013 |
  | Extra | WOS:000317187500011 |
  | DOI | 10.1186/1471-2105-14-S3-S11 |
  | Abstract | Background: Daphnia pulex (Water flea) is the first fully sequenced crustacean genome. The crustaceans and insects have diverged from a common ancestor. It is a model organism for studying the molecular makeup for coping with the environmental challenges. In the complete proteome, there are 30,550 putative proteins. However, about 10,000 of them have no known homologues. Currently, the UniProtoKB reports on 95% of the Daphnia's proteins as putative and uncharacterized proteins. Results: We have applied ProtoNet, an unsupervised hierarchical protein clustering method that covers about 10 million sequences, for automatic annotation of the Daphnia's proteome. 98.7% (26,625) of the Daphnia full-length proteins were successfully mapped to 13,880 ProtoNet stable clusters, and only 1.3% remained unmapped. We compared the properties of the Daphnia's protein families with those of the mouse and the fruitfly proteomes. Functional annotations were successfully assigned for 86% of the proteins. Most proteins (61%) were mapped to only 2953 clusters that contain Daphnia's duplicated genes. We focused on the functionality of maximally amplified paralogs. Cuticle structure components and a variety of ion channels protein families were associated with a maximal level of gene amplification. We focused on gene amplification as a leading strategy of the Daphnia in coping with environmental toxicity. Conclusions: Automatic inference is achieved through mapping of sequences to the protein family tree of ProtoNet 6.0. Applying a careful inference protocol resulted in functional assignments for over 86% of the complete proteome. We conclude that the scaffold of ProtoNet can be used as an alignment-free protocol for large-scale annotation task of uncharacterized proteomes. |
  | Date Added | 10/28/2013, 4:53:08 PM |
  | Modified | 10/8/2014, 12:51:00 PM |

  ### Notes:

  - Present ProtoNet method to perform function annotation of the Water flea genome.

    How SCOP/CATH is used:

    Use SCOP/CATH annotations in their method to map Water flea proteome to known protein superfamilies and families.

     SCOP/CATH reference:

    The DB including all the external expert annotations (e.g., SCOP, Pfam, GO) will be updated each year.

    ...

    Annotation inference

    We focused only on the following dominating annotations: UniProt Keywords, EC, GO, InterPro and the structural classifications from CATH [32] and SCOP [33] (see data- base description in [8]). For each one of these keywords we looked for the one with the highest Correspondence Score (CS) index that reflects the size of the intersection (number of proteins with a specific annotation in the clus- ter) divided by the size of the union (number of proteins with the specific annotation in the tree). We eliminate annotations that are based on uninformative terms such as ‘complete proteome’, ‘taxonomy’ and ‘hypothetical protein’.

  ### Attachments

  - 1471-2105-14-S3-S11.pdf
- ## Functional prediction of binding pockets

  |  |  |
  | --- | --- |
  | Type | Journal Article |
  | Author | Maria Kontoyianni |
  | Author | Christopher B. Rosnick |
  | URL | http://pubs.acs.org/doi/abs/10.1021/ci2005912 |
  | Volume | 52 |
  | Issue | 3 |
  | Pages | 824–833 |
  | Publication | Journal of chemical information and modeling |
  | Date | 2012 |
  | Accessed | 9/23/2013, 10:14:36 AM |
  | Library Catalog | Google Scholar |
  | Date Added | 10/11/2013, 10:29:15 AM |
  | Modified | 10/8/2014, 12:34:58 PM |

  ### Tags:

  - active-sites
  - cavities
  - classification
  - Drug Discovery
  - druggable genome
  - enzyme function
  - protein-function prediction
  - scop database
  - structural genomics
  - structure alignment

  ### Notes:

  - Present method for binding pocket function prediction.  The binding pockets of a data set of proteins were described with structural, thermodynamics, and geometric attributes.

    How SCOP is used:

    Collect a dataset of proteins grouped by common function using the PDB, SCOP, and EC annotations.

    Use data set to validate their method for function prediction.

    Mention inconsistencies between their classification and SCOP's family and protein-level classification.

    SCOP references:

    Complex and Site Preparation. For all computations, Discovery Studio 2.5 was employed within the Accelrys suite of programs (Accelrys Inc., San Diego, CA 92121). The data set was compiled using the protein databank, the Structural Classification of Proteins (**SCOP**),68 the Enzyme Classification (EC) system,69 and the Washington University Basic Local Alignment Search Tool Version 2.0 (WU-Blast2).70,71 

    ...

    Complexes included in this study are presented in Table 1. The criteria used for target selection were rather straightforward: sufficient data points per protein family, preference for human species, exclusion of isozymes and mutants, noncovalent binding between the ligand and respective protein, resolution of the crystallographic complex should be less than 3.0 Å, and the bound ligand was preferably an inhibitor. It can be seen that not only representatives from each of the main divisions (classes) of the enzyme classification system are included, but we have also covered the majority of subclasses within each of these divisions. Hydrolases and gamma-carboxylases have the lowest number of representatives, with 15 and 16 complexes, respectively. The reason for the reduced representation of these two families in our data set stems from their relatively limited presence in the protein databank. If we use SCOP’s nomenclature, it also becomes obvious that our classification is not consistent hierarchically that is, certain categories are at the ‘family’ tree of SCOP, while others are at the ‘protein’ level. For example, the ‘isomerase’ class consists of a variety of proteins whose function is similar; however, they are geometrically and structurally very distinct. In contrast, families such as dihydrofolate reductase (DHFR) or HIV have representatives which are homologous proteins both functionally and structurally. We were also conflicted about the transferases (methyl-acyl and aryl-alkyl), which are grouped separately in our data set (Table 1) from the thymidylate synthases and kinases, although the latter two are both under the umbrella of transferases, based on the EC convention. We chose this division because there is enough structural identity in these individual classes to be independent categories, while the

  ### Attachments

  - ci2005912.pdf
- ## Functional site plasticity in domain superfamilies

  |  |  |
  | --- | --- |
  | Type | Journal Article |
  | Author | Benoit H. Dessailly |
  | Author | Natalie L. Dawson |
  | Author | Kenji Mizuguchi |
  | Author | Christine A. Orengo |
  | Volume | 1834 |
  | Issue | 5 |
  | Pages | 874-889 |
  | Publication | Biochimica Et Biophysica Acta-Proteins and Proteomics |
  | ISSN | 1570-9639 |
  | Date | MAY 2013 |
  | Extra | WOS:000318388300008 |
  | DOI | 10.1016/j.bbapap.2013.02.042 |
  | Abstract | We present, to our knowledge, the first quantitative analysis of functional site diversity in homologous domain superfamilies. Different types of functional sites are considered separately. Our results show that most diverse superfamilies are very plastic in terms of the spatial location of their functional sites. This is especially true for protein-protein interfaces. In contrast, we confirm that catalytic sites typically occupy only a very small number of topological locations. Small-ligand binding sites are more diverse than expected, although in a more limited manner than protein-protein interfaces. In spite of the observed diversity, our results also confirm the previously reported preferential location of functional sites. We identify a subset of homologous domain superfamilies where diversity is particularly extreme, and discuss possible reasons for such plasticity, i.e. structural diversity. Our results do not contradict previous reports of preferential co-location of sites among homologues, but rather point at the importance of not ignoring other sites, especially in large and diverse superfamilies. Data on sites exploited by different relatives, within each well annotated domain superfamily, has been made accessible from the CATH website in order to highlight versatile superfamilies or superfamilies with highly preferential sites. This information is valuable for system biology and knowledge of any constraints on protein interactions could help in understanding the dynamic control of networks in which these proteins participate. The novelty of our work lies in the comprehensive nature of the analysis - we have used a significantly larger dataset than previous studies - and the fact that in many superfamilies we show that different parts of the domain surface are exploited by different relatives for ligand/protein interactions, particularly in superfamilies which are diverse in sequence and structure, an observation not previously reported on such a large scale. This article is part of a Special Issue entitled: The emerging dynamic view of proteins: Protein plasticity in allostery, evolution and self-assembly. (C) 2013 Elsevier B.V. All rights reserved. |
  | Date Added | 2/20/2014, 12:24:01 PM |
  | Modified | 3/7/2014, 12:10:01 PM |

  ### Notes:

  - Computational study of variance of functional site locations within domain superfamilies.

    How SCOP is used:

    Cite two previous studies that used SCOP.

    First study counted presence of ligand binding sites in all members of the same SCOP superfamily.

    Second study confirmed searched for the presence of the same catalytic domain found in NAD(P)-binding Rossmann superfamily, and found in at least 7 different SCOP superfamilies.

    How CATH is used:

    Use CATH superfamilies, rather than SCOP.  One "advantage" of using CATH is that data on functional sites is readily available: "Data on sites exploited by different relatives, within each well annotated domain superfamily, has been made accessible from the CATH website in order to highlight versatile superfamilies or superfamilies with highly preferential sites."

    SCOP reference:

    Previous studies have attempted to look at how diverse the func- tional sites of different types are between sets of homologous pro- teins, genes or domains. For example, it was reported that domains within SCOP families (the family level in SCOP groups together do- mains that are clearly evolutionarily related, generally with pairwise sequence identities of 30% or greater), generally have their binding sites in similar locations [17]. As part of a review on challenges to pre- dict macromolecular interactions, Wass et al. succinctly reported a count of ligand-binding sites in SCOP superfamilies and described that most superfamilies have a small number of such sites, and that these sites tend to be found in most superfamily members [11].

    ...

    3.3.3. Superfamily with high site coverage and high sequence diversity

    Fig. 5 illustrates the protein–protein interface coverage for the NAD(P)-binding Rossmann superfamily. This superfamily is extremely large with 402 60% sequence identity clusters. The NAD(P)-binding Rossmann domains bind the coenzyme nicotinamide adenine dinucleo- tide (NAD+) and a large selection of catalytic domains, which have been shown to come from at least seven different SCOP superfamilies [52].

  ### Attachments

  - 1-s2.0-S1570963913001131-main.pdf
- ## Function prediction from networks of local evolutionary similarity in protein structure

  |  |  |
  | --- | --- |
  | Type | Journal Article |
  | Author | Serkan Erdin |
  | Author | Eric Venner |
  | Author | Andreas M. Lisewski |
  | Author | Olivier Lichtarge |
  | URL | http://www.biomedcentral.com/1471-2105/14/S3/S6/ |
  | Volume | 14 |
  | Issue | Suppl 3 |
  | Pages | S6 |
  | Publication | BMC bioinformatics |
  | Date | 2013 |
  | Accessed | 9/20/2013, 1:19:04 PM |
  | Library Catalog | Google Scholar |
  | Date Added | 10/11/2013, 10:29:15 AM |
  | Modified | 3/7/2014, 12:09:53 PM |

  ### Notes:

  - Improve on Evolutionary Trace Annotation (ETA) method for protein function prediction.  ETA diffuses annotations over a network of template matches.  Because structural similarity alone is insufficient for inferring function, template-based instead use the presence of identical residues in identical geometries.

    How SCOP/CATH is used:

    Not using the SCOP or CATH classification.

    Mention SCOP and CATH as structure classification databases.

    SCOP reference:

    any global (described by CATH [7] or SCOP [8] codes) similarities that exist between structures may indicate functional similarities that are not recognizable from sequence comparisons alone [9

  ### Attachments

  - 1471-2105-14-S3-S6.pdf
- ## Further Evidence for the Likely Completeness of the Library of Solved Single Domain Protein Structures

  |  |  |
  | --- | --- |
  | Type | Journal Article |
  | Author | Jeffrey Skolnick |
  | Author | Hongyi Zhou |
  | Author | Michal Brylinski |
  | Volume | 116 |
  | Issue | 23 |
  | Pages | 6654-6664 |
  | Publication | Journal of Physical Chemistry B |
  | ISSN | 1520-6106 |
  | Date | JUN 14 2012 |
  | Extra | WOS:000305356100009 |
  | DOI | 10.1021/jp211052j |
  | Abstract | Recent studies questioned whether the Protein Data Bank (PDB) contains all compact, single domain protein structures. Here, we show that all quasi-spherical, QS, random protein structures devoid of secondary structure are in the PDB and are excellent templates for all native PDB proteins up to 250 residues. Because QS templates have a similar global contour as native, TASSER can refine 98% (90%) of those whose TM-score is 0.4 (0.35) to structures greater than or equal to the 0.5 TM-score threshold (0.74 (0.64) mean TM-score) for CATH/SCOP assignment. On the basis of this and the fact that, at a TM-score of 0.4, 83% (90%) of all (internal) core secondary structure elements are recovered, a 0.40 TM-score is an appropriate fold similarity assignment threshold. Despite the claims of Taylor, Trovato, and Zhou that many of their structures lack a PDB counterpart, using fr-TM-align, at a 0.45 (0.5) TM-score threshold, essentially all (most) are found in the PDB. Thus, the conclusion that the PDB is likely complete is further supported. |
  | Date Added | 10/28/2013, 4:53:08 PM |
  | Modified | 3/7/2014, 12:11:05 PM |

  ### Notes:

  - Investigate whether the PDB is complete: that is the set of solved folds for single-domain protein structures is complete.

    How SCOP/CATH is used:

    Refer to previous work using SCOP and CATH.

    SCOP reference:

    In practice, the traditional way of addressing this issue is to ask if two proteins have the same “fold” or “topology”; for example, whether they have the same SCOP14 and CATH15 fold assignment.

    ...

    Xu and Yang showed that above a TM-score of 0.5, the fold as assessed by CATH17 and SCOP29 is likely the same.28

    ...

    Another study that examined the completeness of the PDB is due to Dai and Zhou,31 who extended the existing library of PDB structures by permuting loops and considered a maximum of 5 loop permutations on 2936 SCOP domains.14 For proteins between 60 and 200 residues, using the original version of TM- align,26 they conclude that at a TM-score threshold of 0.5, 82% of the loop permuted structures between 180 and 200 residues belong to new fold clusters and are absent in the PDB. We shall explore whether this conclusion holds on further analysis when fr-TM-align is used.

  ### Attachments

  - jp211052j.pdf
- ## Gene3D: a domain-based resource for comparative genomics, functional annotation and protein network analysis

  |  |  |
  | --- | --- |
  | Type | Journal Article |
  | Author | Jonathan Lees |
  | Author | Corin Yeats |
  | Author | James Perkins |
  | Author | Ian Sillitoe |
  | Author | Robert Rentzsch |
  | Author | Benoit H. Dessailly |
  | Author | Christine Orengo |
  | Volume | 40 |
  | Issue | D1 |
  | Pages | D465–D471 |
  | Publication | Nucleic Acids Research |
  | Date | January 2012 |
  | DOI | 10.1093/nar/gkr1181 |
  | Abstract | Gene3D http://www.w3.org/1999/xlink">http://gene3d.biochem.ucl.ac.uk is a comprehensive database of protein domain assignments for sequences from the major sequence databases. Domains are directly mapped from structures in the CATH database or predicted using a library of representative profile HMMs derived from CATH superfamilies. As previously described, Gene3D integrates many other protein family and function databases. These facilitate complex associations of molecular function, structure and evolution. Gene3D now includes a domain functional family (FunFam) level below the homologous superfamily level assignments. Additions have also been made to the interaction data. More significantly, to help with the visualization and interpretation of multi-genome scale data sets, we have developed a new, revamped website. Searching has been simplified with more sophisticated filtering of results, along with new tools based on Cytoscape Web, for visualizing protein-protein interaction networks, differences in domain composition between genomes and the taxonomic distribution of individual superfamilies. |
  | Date Added | 3/7/2014, 12:08:00 PM |
  | Modified | 3/7/2014, 12:08:00 PM |
- ## Generalized order-value optimization

  |  |  |
  | --- | --- |
  | Type | Journal Article |
  | Author | Jose Mario Martinez |
  | Volume | 20 |
  | Issue | 1 |
  | Pages | 75–98 |
  | Publication | Top |
  | Date | April 2012 |
  | DOI | 10.1007/s11750-010-0169-1 |
  | Abstract | Generalized Order-Value Optimization (GOVO) problems involve functions whose evaluation depends on order relations on some representation functional set. We give examples of GOVO problems that may be analyzed in the context of Piecewise-Smooth Optimization. Generalizations of algorithms that have been proved to be effective for proving special classes of GOVO problems are introduced. The case of Low Order-Value Optimization (LOVO) is considered as an example of GOVO in which one needs specialized algorithms with stronger convergence results. Applications of constrained LOVO problems and problems with OVO constraints are presented. The state-of-the-art of Protein Alignment problems from the LOVO point of view are discussed. |
  | Date Added | 3/7/2014, 1:06:24 PM |
  | Modified | 3/7/2014, 1:06:24 PM |
- ## Genes under positive selection in a model plant pathogenic fungus, Botrytis

  |  |  |
  | --- | --- |
  | Type | Journal Article |
  | Author | Gabriela Aguileta |
  | Author | Juliette Lengelle |
  | Author | Helene Chiapello |
  | Author | Tatiana Giraud |
  | Author | Muriel Viaud |
  | Author | Elisabeth Fournier |
  | Author | Francois Rodolphe |
  | Author | Sylvain Marthey |
  | Author | Aurelie Ducasse |
  | Author | Annie Gendrault |
  | Author | Julie Poulain |
  | Author | Patrick Wincker |
  | Author | Lilian Gout |
  | Volume | 12 |
  | Issue | 5 |
  | Pages | 987–996 |
  | Publication | Infection Genetics and Evolution |
  | Date | July 2012 |
  | DOI | 10.1016/j.meegid.2012.02.012 |
  | Abstract | The rapid evolution of particular genes is essential for the adaptation of pathogens to new hosts and new environments. Powerful methods have been developed for detecting targets of selection in the genome. Here we used divergence data to compare genes among four closely related fungal pathogens adapted to different hosts to elucidate the functions putatively involved in adaptive processes. For this goal, ESTs were sequenced in the specialist fungal pathogens Botrytis tulipae and Botrytis ficariarum, and compared with genome sequences of Botrytis cinerea and Sclerotinia sclerotiorum, responsible for diseases on over 200 plant species. A maximum likelihood-based analysis of 642 predicted orthologs detected 21 genes showing footprints of positive selection. These results were validated by resequencing nine of these genes in additional Botrytis species, showing they have also been rapidly evolving in other related species. Twenty of the 21 genes had not previously been identified as pathogenicity factors in B. cinerea, but some had functions related to plant-fungus interactions. The putative functions were involved in respiratory and energy metabolism, protein and RNA metabolism, signal transduction or virulence, similarly to what was detected in previous studies using the same approach in other pathogens. Mutants of B. cinerea were generated for four of these genes as a first attempt to elucidate their functions. (C) 2012 Elsevier B.V. All rights reserved. |
  | Date Added | 3/7/2014, 12:08:00 PM |
  | Modified | 3/7/2014, 12:08:00 PM |
- ## Genetic and structural characterization of the growth hormone gene and protein from tench, Tinca tinca

  |  |  |
  | --- | --- |
  | Type | Journal Article |
  | Author | R. Panicz |
  | Author | J. Sadowski |
  | Author | R. Drozd |
  | Volume | 38 |
  | Issue | 6 |
  | Pages | 1645–1653 |
  | Publication | Fish Physiology and Biochemistry |
  | Date | December 2012 |
  | DOI | 10.1007/s10695-012-9661-x |
  | Abstract | The analysis of the tench growth hormone gene structure revealed a comparable organization of coding and non-coding regions than other from cyprinid species. Based on the performed mRNA and amino acid sequence alignments, gh tench is related to Asian than to European representatives of Cyprinidae family. Second aim of the work was to characterize and predict protein structure of the tench growth hormone. Tinca tinca GH share many common features with human GH molecule. The Tench GH protein binds to the growth hormone receptor (GHR) using two regions I and II that are situated at opposite sites of molecule. Binding site I is placed in the central part of T. tinca GH and H 189 amino acid in the middle region of the IV helix is crucial for GH-GHR interactions. |
  | Date Added | 3/7/2014, 12:08:00 PM |
  | Modified | 3/7/2014, 12:08:00 PM |
- ## Genome3D: a UK collaborative project to annotate genomic sequences with predicted 3D structures based on SCOP and CATH domains

  |  |  |
  | --- | --- |
  | Type | Journal Article |
  | Author | Tony E. Lewis |
  | Author | Ian Sillitoe |
  | Author | Antonina Andreeva |
  | Author | Tom L. Blundell |
  | Author | Daniel WA Buchan |
  | Author | Cyrus Chothia |
  | Author | Alison Cuff |
  | Author | Jose M. Dana |
  | Author | Ioannis Filippis |
  | Author | Julian Gough |
  | URL | http://nar.oxfordjournals.org/content/41/D1/D499.short |
  | Volume | 41 |
  | Issue | D1 |
  | Pages | D499–D507 |
  | Publication | Nucleic Acids Research |
  | Date | 2013 |
  | Accessed | 9/20/2013, 10:46:32 AM |
  | Library Catalog | Google Scholar |
  | Short Title | Genome3D |
  | Date Added | 2/20/2014, 12:24:01 PM |
  | Modified | 5/5/2014, 3:12:47 PM |

  ### Notes:

  - Genome3D is a genome annotation database, with a focus on SCOP and CATH annotations.

    How SCOP is used:

    Annotate by SCOP domain, and full classification.

    SCOP/CATH reference:

    There are 1429 consensus superfamily pairs between CATH v3.5.0 and SCOP v1.75, and these are grouped into ‘Bronze Standard’ (532 pairs), ‘Silver Standard’ (527 pairs) and ‘Gold Standard’ (370 pairs) according to their degree of similarity.

  ### Attachments

  - [HTML] from oxfordjournals.org
  - Nucl. Acids Res.-2013-Lewis-D499-507.pdf
  - Snapshot
- ## Genome-wide structural modelling of TCR-pMHC interactions

  |  |  |
  | --- | --- |
  | Type | Journal Article |
  | Author | I.-Hsin Liu |
  | Author | Yu-Shu Lo |
  | Author | Jinn-Moon Yang |
  | Volume | 14 |
  | Pages | S5 |
  | Publication | Bmc Genomics |
  | ISSN | 1471-2164 |
  | Date | OCT 16 2013 |
  | Extra | WOS:000329440600004 |
  | DOI | 10.1186/1471-2164-14-S5-S5 |
  | Abstract | Background: The adaptive immune response is antigen-specific and triggered by pathogen recognition through T cells. Although the interactions and mechanisms of TCR-peptide-MHC (TCR-pMHC) have been studied over three decades, the biological basis for these processes remains controversial. As an increasing number of high-throughput binding epitopes and available TCR-pMHC complex structures, a fast genome-wide structural modelling of TCR-pMHC interactions is an emergent task for understanding immune interactions and developing peptide vaccines. Results: We first constructed the PPI matrices and iMatrix, using 621 non-redundant PPI interfaces and 398 non-redundant antigen-antibody interfaces, respectively, for modelling the MHC-peptide and TCR-peptide interfaces, respectively. The iMatrix consists of four knowledge-based scoring matrices to evaluate the hydrogen bonds and van der Waals forces between sidechains or backbones, respectively. The predicted energies of iMatrix are high correlated (Pearson's correlation coefficient is 0.6) to 70 experimental free energies on antigen-antibody interfaces. To further investigate iMatrix and PPI matrices, we inferred the 701,897 potential peptide antigens with significant statistic from 389 pathogen genomes and modelled the TCR-pMHC interactions using available TCR-pMHC complex structures. These identified peptide antigens keep hydrogen-bond energies and consensus interactions and our TCR-pMHC models can provide detailed interacting models and crucial binding regions. Conclusions: Experimental results demonstrate that our method can achieve high precision for predicting binding affinity and potential peptide antigens. We believe that iMatrix and our template-based method can be useful for the binding mechanisms of TCR-pMHC complexes and peptide vaccine designs. |
  | Date Added | 2/20/2014, 12:24:01 PM |
  | Modified | 2/20/2014, 12:24:01 PM |

  ### Notes:

  - "developed the iMatrix, PPI-scoring matrices and a template-based approach for modelling of TCR- pMHC interactions in a genome-wide scale."

    How SCOP is used:

    Look up family of proteins of interest.

    SCOP reference:

    Local structural alignment of binding domains

    TCR and antibody are composed of six variable loops (CDRs) and have the same domain annotation (i.e. V set domains (antibody variable domain-like)) based on SCOP [36] database.

  ### Attachments

  - 1471-2164-14-S5-S5.pdf
- ## GFam: a platform for automatic annotation of gene families

  |  |  |
  | --- | --- |
  | Type | Journal Article |
  | Author | Rajkumar Sasidharan |
  | Author | Tamás Nepusz |
  | Author | David Swarbreck |
  | Author | Eva Huala |
  | Author | Alberto Paccanaro |
  | URL | http://nar.oxfordjournals.org/content/40/19/e152.short |
  | Volume | 40 |
  | Issue | 19 |
  | Pages | e152–e152 |
  | Publication | Nucleic Acids Research |
  | Date | 2012 |
  | Accessed | 9/20/2013, 11:10:25 AM |
  | Library Catalog | Google Scholar |
  | Short Title | GFam |
  | Date Added | 10/11/2013, 10:20:13 AM |
  | Modified | 3/7/2014, 12:11:09 PM |

  ### Notes:

  - One objectives behind developing GFam was to provide consensus domain architecture that maximizes annotation coverage provided by InterPro member databases in a meaningful way for a given sequence.

    -Automatically annotate gene/protein families

    -build families based on common domain  architecture and annotate with function

     How SCOP/CATH is used:

    Gfam integrates SUPERFAMILY and Gene3D, which are collections of HMMs built for SCOP and CATH.  Also uses other methods to annotate domains and then creates "consensus domain architectures".

    SCOP reference:

    "SUPERFAMILY (12) and Gene3D (14) are  
    based on a collection of HMMs derived using protein domains at the superfamily/Hlevel based on the hierarchical protein structure classification schemes SCOP (47,48) and  
    CATH (49), respectively. The annotation resource PIRSF (20) usesHMMs over the full length of a protein rather than on the component domains. By integrating these individual resources, InterPro (17) capitalizes on their specific advantages,  
    producing a powerful integrated database along with a search tool InterProScan (40). To assess how well GFam captures such integrated annotations, we calculated  
    sequence and residue coverage for TAIR9 and TAIR10 proteins for all member resources in InterPro and from GFam."

    **47. Andreeva,A., Howorth,D., Chandonia,J.M., Brenner,S.E.,  
    Hubbard,T.J., Chothia,C. and Murzin,A.G. (2008) Data growth  
    and its impact on the SCOP database: new developments. Nucleic  
    Acids Res., 36, D419–D425.  
    48. Murzin,A.G., Brenner,S.E., Hubbard,T. and Chothia,C. (1995)  
    SCOP: a structural classification of proteins database for the  
    investigation of sequences and structures. J. Mol. Biol., 247,  
    536–540.**

  ### Attachments

  - Nucl. Acids Res.-2012-Sasidharan-e152.pdf
- ## Giant viruses coexisted with the cellular ancestors and represent a distinct supergroup along with superkingdoms Archaea, Bacteria and Eukarya

  |  |  |
  | --- | --- |
  | Type | Journal Article |
  | Author | Arshan Nasir |
  | Author | Kyung M. Kim |
  | Author | Gustavo Caetano-Anolles |
  | URL | http://www.biomedcentral.com/1471-2148/12/156/ |
  | Volume | 12 |
  | Issue | 1 |
  | Pages | 156 |
  | Publication | BMC Evolutionary Biology |
  | Date | 2012 |
  | Accessed | 9/20/2013, 1:19:56 PM |
  | Library Catalog | Google Scholar |
  | Date Added | 10/11/2013, 10:29:15 AM |
  | Modified | 12/2/2013, 4:19:36 PM |

  ### Tags:

  - Interesting

  ### Notes:

  - Study evolution of giant viruses.  Reconstruct phylogenies describing the evolution of proteomes and protein domain structures of cellular organisms and double-stranded DNA viruses with medium-to-very-large proteomes (giant viruses).

    How SCOP is used:

    Retrieved SCOP superfamily classification for their dataset using SUPERFAMILY and study abundance of various superfamilies to study how viruses are distributed in various superfamilies.

    SCOP references:

    Background

    ...

    The structural hierarchy defined in the Structural Classification of Proteins (SCOP) groups protein domains with high se- quence conservation (>30% identities) into fold families (FFs), FFs with structural and functional evidence of common origin into fold superfamilies (FSFs), FSFs with common topologies (i.e., same major secondary struc- ture in same arrangement) into folds (Fs) and Fs with similar secondary structure (e.g., alpha helix, beta sheet etc.) into protein classes [25,26]. A total of 110,800 domains that are indexed in SCOP ver. 1.75 correspond to 38,221 protein data bank (PDB) entries and are grouped into 1,195 Fs, 1,962 FSFs and 3,902 FFs.

    ...

    Here we make evolutionary statements from a census of abundance of 1,830 FSFs (defined in SCOP ver. 1.75) in a total of 1,037 proteomes.

    ...

    Methods

    Data retrieval

    We downloaded the FSF assignments for a total of 981 organisms with publically available sequenced genomes (70 Archaea, 652 Bacteria, and 259 Eukarya) from the SUPERFAMILY ver. 1.75 MySQL database (release: 08/ 29/2010)[48,49]. We retrieved the protein sequences encoded by 56 viral genomes including 51 NCLDV and 5 viruses from Archaea, Bacteria and Eukarya (united by the presence of capsid) from the NCBI viral genome re- source homepage (link: http://www.ncbi.nlm.nih.gov/ genomes/GenomesHome.cgi?taxid=10239) and assigned structural domains corresponding to 1,830 FSFs using the hidden Markov Models (HMMs) of structural recog- nition in SUPERFAMILY at a probability cutoff E value of 0.0001 [50]. This defined a total dataset of 1,037 pro- teomes (56 viruses, 70 Archaea, 652 Bacteria, and 259 Eukarya) with a total FSF repertoire of 1,739 FSFs (91 out of 1,830 FSFs had no representation in our dataset and were excluded from the analysis). In these studies, domains were identified using concise classification strings (css) (e.g., c.26.1.5, where c represents the protein class, 26 the F, 1 the FSF and 5 the FF).

  ### Attachments

  - 1471-2148-12-156.pdf
  - [HTML] from biomedcentral.com
- ## Global analysis of chaperone effects using a reconstituted cell-free translation system

  |  |  |
  | --- | --- |
  | Type | Journal Article |
  | Author | Tatsuya Niwa |
  | Author | Takashi Kanamori |
  | Author | Takuya Ueda |
  | Author | Hideki Taguchi |
  | URL | http://www.pnas.org/content/109/23/8937.short |
  | Volume | 109 |
  | Issue | 23 |
  | Pages | 8937–8942 |
  | Publication | Proceedings of the National Academy of Sciences |
  | Date | 2012 |
  | Accessed | 9/20/2013, 1:17:19 PM |
  | Library Catalog | Google Scholar |
  | Date Added | 10/11/2013, 10:29:15 AM |
  | Modified | 10/11/2013, 10:29:15 AM |

  ### Notes:

  - Study of propensity of some proteins to aggregate (stick-together) by secondary-structure content. (Protein folding is often hampered by protein aggregation, which can be prevented by a variety of chaperones in the cell.)

    How SCOP is used:

    They already had a dataset of proteins, and just used SCOP to classify the secondary-structure composition.

    Use SCOP at the class level, in order to study if there is a correlation between aggregation propensity and secondary-structure composition.

    SCOP reference:

    "Earlier works revealed that some of structural motifs were correlated with the aggregation propensity (6) and were enriched in GroE substrates (30, 31). Then, to address the correlation between the chaperone effects and the tertiary or quaternary structures, the Structural Classification of Proteins (SCOP) database (class and fold) (32) and the oligomeric states of proteins were compared, although only a small number of proteins was analyzed, because of the limited database size. When classified by the SCOP classes (all-α, all-β, α/β, and α+β), DnaKJE was effective for the α+β class, whereas GroE was not effective for the all-α class (Fig. 4*B*). Furthermore, we found some biases for DnaKJE and GroE in several SCOP folds (Fig. 4*C*). GroE was biased toward the c1 (TIM barrel) -fold, which is plausible because the most abundant fold in the in vivo obligate GroE substrates is the TIM barrel-fold (30, 31). Neither DnaKJE nor GroE was effective for the a4 (DNA/RNA-binding 3-helical bundle-fold) and c94 (periplasmic binding protein-like II) -folds (Fig. 4*C*)."

  ### Attachments

  - PNAS-2012-Niwa-8937-42.pdf
- ## Golden triangle for folding rates of globular proteins

  |  |  |
  | --- | --- |
  | Type | Journal Article |
  | Author | Sergiy O. Garbuzynskiy |
  | Author | Dmitry N. Ivankov |
  | Author | Natalya S. Bogatyreva |
  | Author | Alexei V. Finkelstein |
  | URL | http://www.pnas.org/content/110/1/147 |
  | Volume | 110 |
  | Issue | 1 |
  | Pages | 147-150 |
  | Publication | Proceedings of the National Academy of Sciences |
  | ISSN | 0027-8424, 1091-6490 |
  | Date | 01/02/2013 |
  | Journal Abbr | PNAS |
  | DOI | 10.1073/pnas.1210180110 |
  | Accessed | 2/28/2013, 1:35:26 PM |
  | Library Catalog | www.pnas.org |
  | Language | en |
  | Abstract | The ability of protein chains to spontaneously form their spatial structures is a long-standing puzzle in molecular biology. Experimentally measured rates of spontaneous folding of single-domain globular proteins range from microseconds to hours: the difference (11 orders of magnitude) is akin to the difference between the life span of a mosquito and the age of the universe. Here, we show that physical theory with biological constraints outlines a “golden triangle” limiting the possible range of folding rates for single-domain globular proteins of various size and stability, and that the experimentally measured folding rates fall within this narrow triangle built without any adjustable parameters, filling it almost completely. In addition, the golden triangle predicts the maximal size of protein domains that fold under solely thermodynamic (rather than kinetic) control. It also predicts the maximal allowed size of the “foldable” protein domains, and the size of domains found in known protein structures is in a good agreement with this limit. |
  | Date Added | 10/11/2013, 10:29:15 AM |
  | Modified | 3/7/2014, 12:09:45 PM |

  ### Notes:

  - There is a wide range in folding rates of proteins.

    Show bounds on folding rates based on particular parameters such as stability and size.

    "Here, we show that physical theory with biological constraints outlines a “golden trian- gle” limiting the possible range of folding rates for single-domain globular proteins of various size and stability, and that the experi- mentally measured folding rates fall within this narrow triangle built without any adjustable parameters, filling it almost completely.  
    "

     How SCOP/CATH is used:

    Mainly, they need single-domain chain proteins with kinetic experimental data.

    Calculated statistics on SCOP and CATH to confirm an estimate of maximum domain size.  Used only the first 4 SCOP classes.

    Reference to SCOP:

    The analysis of domains listed in the comprehensive protein structure databases SCOP (35) and CATH (36) confirms this estimate of the maximal domain size: a few SCOP domains, <1% of 4,861 (Fig. 3B), have more than 500 residues; 30% of this 1% contain 2 or even more structural domains according to CATH, whereas all of the rest (70% of the 1%) are either significantly oblate, or significantly oblong, or composed of several compact, domain-like structural repeats [like Armadillo repeats (37) and à-propeller blades (38)].

    Materials and Methods

    Protein domains shown in Fig. 3 B and C belong to four main SCOP (35) structural classes (", à, "/à, " + à). The SCOP “domains” that consist of more than one domain, according to the SCOP remarks, are not taken into ac- count. All of the other single-chain SCOP domains with sequence identity below 80% (40) have been selected.

  ### Attachments

  - Full Text PDF
- ## Golgi localization of glycosyltransferases requires a Vps74p oligomer

  |  |  |
  | --- | --- |
  | Type | Journal Article |
  | Author | Karl R Schmitz |
  | Author | Jingxuan Liu |
  | Author | Shiqing Li |
  | Author | Thanuja Gangi Setty |
  | Author | Christopher S Wood |
  | Author | Christopher G Burd |
  | Author | Kathryn M Ferguson |
  | Volume | 14 |
  | Issue | 4 |
  | Pages | 523-534 |
  | Publication | Developmental cell |
  | ISSN | 1878-1551 |
  | Date | Apr 2008 |
  | Extra | PMID: 18410729 |
  | Journal Abbr | Dev. Cell |
  | DOI | 10.1016/j.devcel.2008.02.016 |
  | Library Catalog | NCBI PubMed |
  | Language | eng |
  | Abstract | The mechanism of glycosyltransferase localization to the Golgi apparatus is a long-standing question in secretory cell biology. All Golgi glycosyltransferases are type II membrane proteins with small cytosolic domains that contribute to Golgi localization. To date, no protein has been identified that recognizes the cytosolic domains of Golgi enzymes and contributes to their localization. Here, we report that yeast Vps74p directly binds to the cytosolic domains of cis and medial Golgi mannosyltransferases and that loss of this interaction correlates with loss of Golgi localization of these enzymes. We have solved the X-ray crystal structure of Vps74p and find that it forms a tetramer, which we also observe in solution. Deletion of a critical structural motif disrupts tetramer formation and results in loss of Vps74p localization and function. Vps74p is highly homologous to the human GMx33 Golgi matrix proteins, suggesting a conserved function for these proteins in the Golgi enzyme localization machinery. |
  | Date Added | 10/11/2013, 10:29:15 AM |
  | Modified | 10/11/2013, 10:29:15 AM |

  ### Tags:

  - Carrier Proteins
  - Crystallography, X-Ray
  - Glycosylation
  - Glycosyltransferases
  - Golgi Apparatus
  - HeLa Cells
  - Humans
  - Models, Molecular
  - Molecular Chaperones
  - Molecular Sequence Data
  - Protein Structure, Quaternary
  - Recombinant Fusion Proteins
  - Saccharomyces cerevisiae
  - Saccharomyces cerevisiae Proteins

  ### Notes:

  - describe function and structural studies of yeast Vps74p protein.

    How SCOP is used:

    Use downloaded SCOP data.  Search SCOP, CATH, and use DALI to find if there are any known proteins with similar folds to Vps74p, which is involved in glycosyltransferase localization to the Golgi apparatus.

    SCOP reference:

    The overall structure of Vps74p appears to be novel, bearing no strong homology to known protein folds as assessed by the DALI server (Holm et al., 1992), automated comparison against the CATH structural database (Pearl et al., 2005), and SSM compar- ison against the SCOP database (Murzin et al., 1995).

  ### Attachments

  - 1-s2.0-S153458070800110X-main.pdf
  - PubMed entry
- ## Graph-based methods for protein structure comparison

  |  |  |
  | --- | --- |
  | Type | Journal Article |
  | Author | Thomas Fober |
  | Author | Marco Mernberger |
  | Author | Gerhard Klebe |
  | Author | Eyke Huellermeier |
  | Volume | 3 |
  | Issue | 5 |
  | Pages | 307-320 |
  | Publication | Wiley Interdisciplinary Reviews-Data Mining and Knowledge Discovery |
  | ISSN | 1942-4787 |
  | Date | SEP 2013 |
  | Extra | WOS:000323256400001 |
  | DOI | 10.1002/widm.1099 |
  | Abstract | While sequence-based methods are widely used as reliable tools for protein function prediction in general, these methods are likely to fail in cases of low sequence similarity. This is due to the fact that proteins with low sequence similarity may nevertheless have similar functions and exhibit similar structures. In such cases, structure-based comparison methods can help to provide further insights owing to the widely accepted paradigm that structure mirrors function. Moreover, thanks to the steady increase in structural information with the advent of structural genomic projects and the steady improvements in structure prediction, these methods are becoming more and more applicable. Many structure-based approaches to the comparative analysis of proteins and the inference of protein function rely on graph formalisms for modeling protein structures and, correspondingly, employ graph-theoretic algorithms for analyzing and comparing such structures. This review is devoted to approaches of that kind and presents an overview of the most important graph-based algorithms. (C) 2013 Wiley Periodicals, Inc. |
  | Date Added | 2/12/2014, 1:36:22 PM |
  | Modified | 10/8/2014, 12:50:53 PM |

  ### Tags:

  - active-sites
  - automated-method
  - binding-sites
  - edit distance
  - functional sites
  - function prediction
  - maximal common subgraph
  - structure alignment
  - subgraph isomorphism detection
  - theoretic approach

  ### Notes:

  - Unavailable.
- ## Growth of novel protein structural data

  |  |  |
  | --- | --- |
  | Type | Journal Article |
  | Author | Michael Levitt |
  | Volume | 104 |
  | Issue | 9 |
  | Pages | 3183-3188 |
  | Publication | Proceedings of the National Academy of Sciences of the United States of America |
  | ISSN | 0027-8424 |
  | Date | Feb 27, 2007 |
  | Extra | PMID: 17360626 PMCID: PMC1802002 |
  | Journal Abbr | Proc. Natl. Acad. Sci. U.S.A. |
  | DOI | 10.1073/pnas.0611678104 |
  | Library Catalog | NCBI PubMed |
  | Language | eng |
  | Abstract | Contrary to popular assumption, the rate of growth of structural data has slowed, and the Protein Data Bank (PDB) has not been growing exponentially since 1995. Reaching such a dramatic conclusion requires careful measurement of growth of novel structures, which can be achieved by clustering entry sequences, or by using a novel index to down-weight entries with a higher number of sequence neighbors. These measures agree, and growth rates are very similar for entire PDB files, clusters, and weighted chains. The overall sizes of Structural Classification of Proteins (SCOP) categories (number of families, superfamilies, and folds) appear to be directly proportional to the number of deposited PDB files. Using our weighted chain count, which is most correlated to the change in the size of each SCOP category in any time period, shows that the rate of increase of SCOP categories is actually slowing down. This enables the final size of each of these SCOP categories to be predicted without examining or comparing protein structures. In the last 3 years, structures solved by structural genomics (SG) initiatives, especially the United States National Institutes of Health Protein Structure Initiative, have begun to redress the slowing growth of the PDB. Structures solved by SG are 3.8 times less sequence-redundant than typical PDB structures. Since mid-2004, SG programs have contributed half the novel structures measured by weighted chain counts. Our analysis does not rely on visual inspection of coordinate sets: it is done automatically, providing an accurate, up-to-date measure of the growth of novel protein structural data. |
  | Date Added | 10/29/2014, 11:43:05 AM |
  | Modified | 10/29/2014, 11:43:05 AM |

  ### Tags:

  - Amino Acid Sequence
  - Cluster Analysis
  - Databases, Protein
  - Proteins
  - Proteomics
  - Sequence Homology

  ### Attachments

  - PubMed entry
- ## Guardians of the actin monomer

  |  |  |
  | --- | --- |
  | Type | Journal Article |
  | Author | Bo Xue |
  | Author | Robert C. Robinson |
  | Volume | 92 |
  | Issue | 10-11 |
  | Pages | 316-332 |
  | Publication | European Journal of Cell Biology |
  | ISSN | 0171-9335; 1618-1298 |
  | Date | OCT-NOV 2013 |
  | Extra | WOS:000329260500002 |
  | DOI | 10.1016/j.ejcb.2013.10.012 |
  | Abstract | Actin is a universal force provider in eukaryotic cells. Biological processes harness the pressure generated from actin polymerization through dictating the time, place and direction of filament growth. As such, polymerization is initiated and maintained via tightly controlled filament nucleation and elongation machineries. Biological systems integrate force into their activities through recruiting and activating these machineries. In order that actin function as a common force generating polymerization motor, cells must maintain a pool of active, polymerization-ready monomeric actin, and minimize extemporaneous polymerization. Maintenance of the active monomeric actin pool requires the recycling of actin filaments, through depolymerization, nucleotide exchange and reloading of the polymerization machineries, while the levels of monomers are constantly monitored and supplemented, when needed, via the access of a reserve pool of monomers and through gene expression. Throughout its monomeric life, actin needs to be protected against gratuitous nucleation events. Here, we review the proteins that act as custodians of monomeric actin. We estimate their levels on a tissue scale, and calculate the implied concentrations of each actin complex based on reported binding affinities. These estimations predict that monomeric actin is rarely, if ever, alone. Thus, the guardians keep the volatility of actin in check, so that its explosive power is only released in the controlled environments of the nucleation and polymerization machineries. (C) 2013 Elsevier GmbH. All rights reserved. |
  | Date Added | 2/20/2014, 12:24:01 PM |
  | Modified | 2/20/2014, 12:24:01 PM |

  ### Notes:

  - Review of research on actin regulating families.

    How SCOP is used:

    Retrieve classification for cofilins in SCOP (cofilin-like family).

    How CATH is used:

    Retrieve classification for cofilins (severin superfamily)

    SCOP/CATH reference:

    Cofilins possess a unique fold called the ADF-homology domain (ADF-H, Poukkula et al., 2011) (Fig. 2d), which is also classified as the cofilin-like family by SCOP (Andreeva et al., 2008), and the severin superfamily by CATH (Sillitoe et al., 2012).

  ### Attachments

  - 1-s2.0-S017193351300071X-main.pdf
- ## Half a century of Ramachandran plots

  |  |  |
  | --- | --- |
  | Type | Journal Article |
  | Author | Oliviero Carugo |
  | Author | Kristina Djinovic Carugo |
  | Volume | 69 |
  | Pages | 1333-1341 |
  | Publication | Acta Crystallographica Section D-Biological Crystallography |
  | ISSN | 0907-4449 |
  | Date | AUG 2013 |
  | Extra | WOS:000322445100001 |
  | DOI | 10.1107/S090744491301158X |
  | Abstract | On the occasion of their fiftieth birthday, it is opportune to review the first half century of Ramachandran plots. In the present review, some of the most relevant aspects of this fifty-year history are summarized, from the original ideas of Gopalasamudram Narayana Ramachandran to subsequent revisions and to applications in structural biology. This will not be a guided walk through five decades of Ramachandran plots, but a commented summary of the lines along which the original ideas evolved and continue to develop, and of their applications. |
  | Date Added | 10/28/2013, 4:51:00 PM |
  | Modified | 10/28/2013, 4:51:00 PM |

  ### Notes:

  - Review of research with Ramachandran plots.

    How SCOP is used:

    General study on protein structure.  Use ASTRAL representative structures to plot phi and psi averages.  Found that they are distributed on a sigmoid function.

    SCOP reference:

    An extension of the concept that supports the Ramachandran plot has recently been proposed. While each residue of a protein is represented by a point on the Ramachandran plot, each protein of an ensemble of proteins is represented by a point on the proteomic Ramachandran plot (PRplot; Carugo & Djinovic ́-Carugo, 2013).

    This is achieved by computing the circular average of the ’ and dihedral angles for each protein and by plotting the corresponding point on the map. By using a nonredundant set of protein structures taken from the PBSelect database (Griep & Hobohm, 2010), it was possible to verify that proteins are distributed around a sigmoid function such as, for example,

    1⁄4 ⬚⬚165:7 exp1⁄2⬚⬚ð88:7=xÞ⬚⬚14 ⬚⬚ þ 126:9; ð1Þ

    with a correlation coefficient equal to 0.936 (see Fig. 6). Closely similar expressions were obtained by using other nonredundant sets of proteins obtained from the SCOP (Andreeva et al., 2008) database of protein structural domains or built using the PISCES web server (Wang & Dunbrack, 2003), or by using other sigmoid functions.

  ### Attachments

  - dz5282 (1).pdf
- ## HELANAL-Plus: a web server for analysis of helix geometry in protein structures

  |  |  |
  | --- | --- |
  | Type | Journal Article |
  | Author | Prasun Kumar |
  | Author | Manju Bansal |
  | Volume | 30 |
  | Issue | 6 |
  | Pages | 773-783 |
  | Publication | JOURNAL OF BIOMOLECULAR STRUCTURE & DYNAMICS |
  | ISSN | 0739-1102 |
  | Date | 2012 |
  | DOI | 10.1080/07391102.2012.689705 |
  | Language | English |
  | Date Added | 10/25/2013, 4:29:01 PM |
  | Modified | 10/25/2013, 4:29:01 PM |

  ### Tags:

  - alpha-helix
  - ASTRAL
  - ASTRAL domain structures
  - geometry of the helix
  - helix axis
  - JmolApplet
  - UP-DOWN ring conformation of Proline

  ### Notes:

  - Present HELANAL web server for alpha helix geometry analysis.

    How SCOP is used:

    Evaluate method on non-redundant data set.  1195 representative domain structures for different folds were downloaded from ASTRAL-1.75.  Used SPACI to filter.

    SCOP reference:

    2.4. Data-set preparation

    The HELANAL geometry algorithm has been tested on a diverse set of protein structures. We used a common fold benchmark, viz. the Structural Classification of Pro- teins (SCOP) (Andreeva et al., 2008) database. SCOP classifies all protein domains of known structure into a hierarchy with four levels: class, fold, superfamily, and family. In our study, we work at the fold level which groups the proteins with the same major secondary struc- tures in the same arrangement and with the same topo- logical connections. Total of 1195 representative folds were downloaded from ASTRAL-1.75 (Brenner, Koehl, & Levitt, 2000) release database. This database is based on SCOP 1.75 for all protein structures in PDB released before February 2009. The data-set was further refined by following steps: (i) domains with SPACI score <.4 were excluded (total 266 folds removed), (ii) proteins which had a missing ATOM record for any of the resi- dues were removed (total 1 fold removed), and (iii) pro- teins which do not have any STRIDE assigned α-helix of lengthP9 residues were also discarded (total 133 folds removed). Final data-set includes 795 representa- tive protein folds. STRIDE program was used for sec- ondary structure assignment and a total of 4517 α- helices were identified. HELANAL-Plus can assign heli- cal parameters to α-helix of length > 5 residues, but geometry is assigned only to the helix with length P 9 residues. Hence in present analysis α-helices with length P 9 residues were selected. Cut-off values described above for the geometry assignments are based on the detailed analysis of STRIDE defined α-helices.

  ### Attachments

  - 07391102%2E2012%2E689705.pdf
- ## Helical ambivalency induced by point mutations

  |  |  |
  | --- | --- |
  | Type | Journal Article |
  | Author | Nicholus Bhattacharjee |
  | Author | Parbati Biswas |
  | URL | http://www.biomedcentral.com/1472-6807/13/9 |
  | Volume | 13 |
  | Issue | 1 |
  | Pages | 9 |
  | Publication | BMC Structural Biology |
  | Date | 2013 |
  | Accessed | 9/23/2013, 10:22:14 AM |
  | Library Catalog | Google Scholar |
  | Date Added | 10/11/2013, 10:29:15 AM |
  | Modified | 12/2/2013, 4:19:48 PM |

  ### Tags:

  - Interesting
  - likely ASTRAL
  - likely ASTRAL domain structures
  - likely ASTRAL sequences

  ### Notes:

  - Study effects of point mutations on helices.  Collect a data set of helices from a non-redundant data set from the PDB, then collect all sequences from helices and create a database of mutated sequences.  Search the SCOP database for non-helical regions that are identical to the mutated sequences.  Then use this to infer the amino acid types and conditions that are most associated with the loss of a helix.

    How SCOP is used:

    Used sequences from helices from a non-redundant dataset of proteins from PDB-select.  Mapped sequences to non-helical regions in SCOP domains.  Measured propensity of a mutation to create a helix.

    Used SCOP to retrieve structures with the same sequence from each SCOP class.

    SCOP references:

    In Abstract:

    Results: Sequences generated by point mutations of helices are mapped onto their non-helical counterparts in the SCOP database.

    In Background:

    This work presents a detailed study of helical ambiva- lency induced by point mutations. Helices from the non-redundant database are point mutated at all residue positions and the resulting sequences are mapped onto the SCOP database to obtain completely non-helical con- formations.

    In Methods:

    Database and mutation

    The database used in the present study comprises the crystal structures from May-2008 release of PDB-select [41], which were compiled to create a database of non- redundant proteins from PDB [22] (Protein Data Bank). The database comprises protein chains with a sequence identity of 25% or less. All protein chains considered in this study have resolution ≤ 3Å and crystallographic R- factor, R ≤ 0.3. The selected database consists of 2586 non-redundant protein chains from 2466 protein struc- tures. Secondary structures are annotated residue-wise with the help of DSSP software [42]. According to the widely used definition, H and G denote helical conforma- tions while all other classes (B, E, I, S, T, -) are considered to be non-helical [43-45]. Neglecting helices with less than 5 residues long, there are 11592 helices in the non- redundant database. These helices were point mutated at each position by all 20 amino acids (excluding the amino acid present at the given position in wild type helix). So for a helix of length 5 amino acids this method will gen- erate (5X19) 95 mutated sequences. The total number of mutated helices generated from 11592 helices is 2662375 which constitute the database of mutated sequences.

    Proteins from nine SCOP (Structural Classification of Proteins) [46] classes viz. (I)All alpha proteins, (II)All beta proteins, (III)Alpha and beta proteins(a+b), (IV)Alpha and beta proteins(a/b), (V)Coiled coiled pro- teins, (VI)Membrane and cell surface proteins and peptides, (VII)Multi-domain proteins(alpha and beta), (VIII)Peptides and (IX)Small proteins were compiled to obtain sequences identical to the mutated sequences. A structural cut-off resolution ≤ 3Å and R ≤ 0.3 were applied on these proteins with the PISCES server [47]. The resultant SCOP database consists of 48244 protein chains from 22309 protein structures.

    Both the non-redundant protein and SCOP database used in the present study are similar to our recent work on ambivalent helices [34] which helps in comparison between both the works.

    Identical sequence search

    The method for identifying the sequences identical to

    the mutated sequences in SCOP database in non-helical

    conformations is similar to previous method of search-

    ing variable and conserved helices [34].

  ### Attachments

  - [PDF] from biomedcentral.com
  - Snapshot
- ## HEMD: An Integrated Tool of Human Epigenetic Enzymes and Chemical Modulators for Therapeutics

  |  |  |
  | --- | --- |
  | Type | Journal Article |
  | Author | Zhimin Huang |
  | Author | Haiming Jiang |
  | Author | Xinyi Liu |
  | Author | Yingyi Chen |
  | Author | Jiemin Wong |
  | Author | Qi Wang |
  | Author | Wenkang Huang |
  | Author | Ting Shi |
  | Author | Jian Zhang |
  | Volume | 7 |
  | Issue | 6 |
  | Pages | e39917 |
  | Publication | Plos One |
  | ISSN | 1932-6203 |
  | Date | JUN 25 2012 |
  | Extra | WOS:000305781700073 |
  | DOI | 10.1371/journal.pone.0039917 |
  | Abstract | Background: Epigenetic mechanisms mainly include DNA methylation, post-translational modifications of histones, chromatin remodeling and non-coding RNAs. All of these processes are mediated and controlled by enzymes. Abnormalities of the enzymes are involved in a variety of complex human diseases. Recently, potent natural or synthetic chemicals are utilized to establish the quantitative contributions of epigenetic regulation through the enzymes and provide novel insight for developing new therapeutics. However, the development of more specific and effective epigenetic therapeutics requires a more complete understanding of the chemical epigenomic landscape. Description: Here, we present a human epigenetic enzyme and modulator database (HEMD), the database which provides a central resource for the display, search, and analysis of the structure, function, and related annotation for human epigenetic enzymes and chemical modulators focused on epigenetic therapeutics. Currently, HEMD contains 269 epigenetic enzymes and 4377 modulators in three categories (activators, inhibitors, and regulators). Enzymes are annotated with detailed description of epigenetic mechanisms, catalytic processes, and related diseases, and chemical modulators with binding sites, pharmacological effect, and therapeutic uses. Integrating the information of epigenetic enzymes in HEMD should allow for the prediction of conserved features for proteins and could potentially classify them as ideal targets for experimental validation. In addition, modulators curated in HEMD can be used to investigate potent epigenetic targets for the query compound and also help chemists to implement structural modifications for the design of novel epigenetic drugs. Conclusions: HEMD could be a platform and a starting point for biologists and medicinal chemists for furthering research on epigenetic therapeutics. HEMD is freely available at http://mdl.shsmu.edu.cn/HEMD/. |
  | Date Added | 2/20/2014, 12:24:01 PM |
  | Modified | 3/7/2014, 12:14:44 PM |

  ### Notes:

  - Present HEMD: "an integrated database of human epigenetic enzymes and their modulators focused on epigenetic therapeutics"

    How SCOP is used:

    Annotate a data set curated from the literature on proteins associated with: ‘‘DNA methylation’’, ‘‘histones modifica- tion’’, ‘‘chromatin remodeling’’, and ‘‘non-coding RNA’’.

    SCOP reference:

    An up-to-date synchronization on available structures of epigenetic enzymes from PDB [26] is present and their structural classification SCOP [27] and CATH [28] based on the PDB ID are also labeled.

  ### Attachments

  - journal.pone.0039917.pdf
- ## HHblits: lightning-fast iterative protein sequence searching by HMM-HMM alignment

  |  |  |
  | --- | --- |
  | Type | Journal Article |
  | Author | Michael Remmert |
  | Author | Andreas Biegert |
  | Author | Andreas Hauser |
  | Author | Johannes Soeding |
  | Volume | 9 |
  | Issue | 2 |
  | Pages | 173-175 |
  | Publication | Nature Methods |
  | ISSN | 1548-7091 |
  | Date | FEB 2012 |
  | Extra | WOS:000300029600027 |
  | DOI | 10.1038/NMETH.1818 |
  | Abstract | Sequence-based protein function and structure prediction depends crucially on sequence-search sensitivity and accuracy of the resulting sequence alignments. We present an open-source, general-purpose tool that represents both query and database sequences by profile hidden Markov models (HMMs): 'HMM-HMM based lightning-fast iterative sequence search' (HHblits; http://toolkit.genzentrum.lmu.de/hhblits/). Compared to the sequence-search tool PSI-BLAST, HHblits is faster owing to its discretized-profile prefilter, has 50-100% higher sensitivity and generates more accurate alignments. |
  | Date Added | 2/20/2014, 12:24:01 PM |
  | Modified | 2/20/2014, 12:24:01 PM |

  ### Notes:

  - Present HHblits method for homolog detection.

    How SCOP is used:

    Validate homolog detection using SCOP fold annotation.  Use a representative domain sequence dataset derived from SCOP.  To assess quality of pairwise alignments, randomly selected domain pairs from each superfamily.

    SCOP reference:

    ...

    Figure 1 caption:

    (c) True  
     positive pairs (same SCOP fold) compared to  
     false positive pairs (different SCOP fold) for one  
     and three search iterations in an all-against-all comparison. FDR, false discovery rate. (

    ...

    We compared the sensitivity of HHblits to that of PSI-BLAST and HMMER3 in detecting homologous proteins (to rank true positive, homologous pairs above false positive, unrelated pairs) (Fig. 1c). We performed an all-against-all comparison of 5,287 representative domain sequences from the Structural Classification of Proteins (SCOP) database11. After one itera- tion, HHblits detected 107% more true positive pairs than PSI- BLAST and 53% more than HMMER3 at 1% false discovery rate, and after three iterations, the improvement was 147% over PSI- BLAST and 69% over HMMER3. We obtained similar values in a receiver operating curve 5 (ROC5) analysis (Online Methods and Supplementary Fig. 7). Furthermore, HHblits reported more reliable E values than PSI-BLAST (Supplementary Fig. 8).

    To assess the quality of the pairwise alignments (Fig. 1d), we randomly selected from each SCOP superfamily up to ten pairs of domains with <30% sequence identity and a TM-align (Online Methods) structural similarity score of >0.6 (Supplementary Data 2). For each method, we built MSAs for the queries using two search iterations through UniProt and aligned the resulting query MSAs with their corresponding templates. We determined correctly aligned residues through comparison with the structural alignments. Compared to PSI-BLAST and HMMER3, HHblits sensitivity per residue using default parameters (mact 0.5) was 12 and 2 percentage points higher and the precision per residue was 15 and 10 percentage points higher, respectively (Fig. 1d). The higher precision of HHblits alignments explains its robustness against homologous overextension (tested on a benchmark with multidomain proteins; Supplementary Fig. 9), which is the main cause of corrupted PSI-BLAST alignments12.

  ### Attachments

  - nmeth.1818.pdf
- ## Hidden Relationship between Conserved Residues and Locally Conserved Phosphate-Binding Structures in NAD(P)-Binding Proteins

  |  |  |
  | --- | --- |
  | Type | Journal Article |
  | Author | Chih Yuan Wu |
  | Author | Yun Hao Hwa |
  | Author | Yao Chi Chen |
  | Author | Carmay Lim |
  | Volume | 116 |
  | Issue | 19 |
  | Pages | 5644–5652 |
  | Publication | Journal of Physical Chemistry B |
  | Date | May 2012 |
  | DOI | 10.1021/jp3014332 |
  | Abstract | A one-dimensional (1D) motif usually comprises conserved essential residues involved in catalysis, ligand binding, or maintaining a specific structure. However, it cannot be easily detected in proteins with low sequence identity because it is difficult to (1) identify protein sequences suspected to contain the motif, and (2) align sequences with little sequence identity to spot the conserved residues. Here, we present a strategy for discovering phosphate-binding ID motifs in NAD(P)-binding proteins sharing low sequence identity that overcomes these two hurdles by determining all distinct locally conserved pyrophosphate-binding structures and aligning the same-length sequences comprising each of these structures to identify the conserved residues. We show that the sequence motifs derived from the distinct pyrophosphate-binding structures yield different numbers/spacing of conserved Gly residues. We also show that they depend on the side chain orientations and cofactor type (NAD or NADP). Thus, sequence motifs derived from local similarity of backbone structures without consideration of the cofactor type and/or side chain orientations would reduce their reliability in annotating protein function from sequence alone. The three-dimensional (3D) and 1D motifs comprising conserved residues in nonredundant proteins reveal hidden relationships between the protein structure/function and sequence as well as protein-cofactor interactions. |
  | Date Added | 3/7/2014, 12:08:00 PM |
  | Modified | 3/7/2014, 12:08:00 PM |
- ## Hierarchical Classification of Protein Folds Using a Novel Ensemble Classifier

  |  |  |
  | --- | --- |
  | Type | Journal Article |
  | Author | Chen Lin |
  | Author | Ying Zou |
  | Author | Ji Qin |
  | Author | Xiangrong Liu |
  | Author | Yi Jiang |
  | Author | Caihuan Ke |
  | Author | Quan Zou |
  | URL | http://www.ncbi.nlm.nih.gov/pmc/articles/PMC3577917/ |
  | Volume | 8 |
  | Issue | 2 |
  | Publication | PloS one |
  | Date | 2013 |
  | Accessed | 9/23/2013, 10:17:26 AM |
  | Library Catalog | Google Scholar |
  | Date Added | 10/11/2013, 10:29:15 AM |
  | Modified | 10/11/2013, 10:29:15 AM |

  ### Tags:

  - likely ASTRAL
  - likely ASTRAL sequences

  ### Notes:

  - Present method for SCOP class and fold prediction.

    How SCOP is used:

    Train and validate their fold prediction method on SCOP domains.

    SCOP references:

    Using the latest SCOP release (version 1.75) [22], we deleted similar protein domains and reduced the homology of dataset to train a highly reliable model.

  ### Attachments

  - pone.0056499.pdf
- ## Highly Abundant Proteins Favor More Stable 3D Structures in Yeast

  |  |  |
  | --- | --- |
  | Type | Journal Article |
  | Author | Adrian WR Serohijos |
  | Author | S. Y. Lee |
  | Author | Eugene I. Shakhnovich |
  | URL | http://www.sciencedirect.com/science/article/pii/S000634951205148X |
  | Volume | 104 |
  | Issue | 3 |
  | Pages | L1–L3 |
  | Publication | Biophysical journal |
  | Date | 2013 |
  | Accessed | 9/20/2013, 1:12:35 PM |
  | Library Catalog | Google Scholar |
  | Date Added | 10/11/2013, 10:29:15 AM |
  | Modified | 10/11/2013, 10:29:15 AM |

  ### Notes:

  - Study to test hypothesis that proteins that are more abundant tend to be more stable.  Study this hypothesis in yeast proteins.

    How SCOP is used:

    Identify domains in a curated data set of yeast proteins, yielding 302 domains.  Calculate hydrogen bonding and vdw interactions in each domain as a measure of stability.

    SCOP reference:

    To demonstrate this prediction more unambiguously, we extracted all of the yeast proteins from the Protein Data Bank, partitioned them into domains as defined by SCOP (11), and then mapped their experimentally measured abundance (9). Also, we excluded domains with gaps in the structure. This procedure yielded 302 domains on which we performed a structural analysis (Fig. 2 and Table S1 in the Supporting Material).

  ### Attachments

  - [PDF] from harvard.edu
- ## Highly covarying residues have a functional role in antibody constant domains

  |  |  |
  | --- | --- |
  | Type | Journal Article |
  | Author | Elizabeth A. Proctor |
  | Author | Pradeep Kota |
  | Author | Stephen J. Demarest |
  | Author | Justin A. Caravella |
  | Author | Nikolay V. Dokholyan |
  | URL | http://onlinelibrary.wiley.com/doi/10.1002/prot.24247/full |
  | Publication | Proteins: Structure, Function, and Bioinformatics |
  | Date | 2013 |
  | Accessed | 9/23/2013, 10:14:18 AM |
  | Library Catalog | Google Scholar |
  | Date Added | 10/11/2013, 10:29:15 AM |
  | Modified | 10/11/2013, 10:29:15 AM |

  ### Tags:

  - covariation networks
  - multiple sequence alignment
  - protein design
  - protein domains
  - protein evolution

  ### Notes:

  - Studying variability among antibodies.  Highlight how, although there is high variability in sequence and function, the immunoglobulin superfamily has only 4 families/folding sets in SCOP.

    How using SCOP:

    To lookup families/folding sets of immunoglobulin superfamily.

    Reference to SCOP:

    Despite the remarkable variations in sequence and function, the members of the Ig superfamily are grouped into only four folding ‘‘sets’’ (V, C1, C2, and I) in the database of structural classification of proteins (SCOP).11

  ### Attachments

  - [PDF] from unc.edu
- ## High-quality protein backbone reconstruction from alpha carbons using gaussian mixture models

  |  |  |
  | --- | --- |
  | Type | Journal Article |
  | Author | Benjamin L. Moore |
  | Author | Lawrence A. Kelley |
  | Author | James Barber |
  | Author | James W. Murray |
  | Author | James T. MacDonald |
  | Volume | 34 |
  | Issue | 22 |
  | Pages | 1881-1889 |
  | Publication | Journal of Computational Chemistry |
  | ISSN | 0192-8651 |
  | Date | AUG 15 2013 |
  | DOI | 10.1002/jcc.23330 |
  | Language | English |
  | Abstract | Coarse-grained protein structure models offer increased efficiency in structural modeling, but these must be coupled with fast and accurate methods to revert to a full-atom structure. Here, we present a novel algorithm to reconstruct mainchain models from C traces. This has been parameterized by fitting Gaussian mixture models (GMMs) to short backbone fragments centered on idealized peptide bonds. The method we have developed is statistically significantly more accurate than several competing methods, both in terms of RMSD values and dihedral angle differences. The method produced Ramachandran dihedral angle distributions that are closer to that observed in real proteins and better Phaser molecular replacement log-likelihood gains. Amino acid residue sidechain reconstruction accuracy using SCWRL4 was found to be statistically significantly correlated to backbone reconstruction accuracy. Finally, the PD2 method was found to produce significantly lower energy full-atom models using Rosetta which has implications for multiscale protein modeling using coarse-grained models. A webserver and C++ source code is freely available for noncommercial use from: http://www.sbg.bio.ic.ac.uk/phyre2/PD2\_ca2main/. (c) 2013 Wiley Periodicals, Inc. |
  | Date Added | 10/25/2013, 4:17:08 PM |
  | Modified | 11/20/2014, 9:58:14 AM |

  ### Tags:

  - ASTRAL
  - ASTRAL domain structures
  - Coarse-grained model
  - multiscale protein modeling
  - protein backbone
  - protein structure modeling
  - webserver

  ### Notes:

  - Present method for converting a course-grained model of a protein backbone back to a full all-atom main-chain model.

    How SCOP is used:

    Train method on representative data set from ASTRAL from SCOPe 2.01.  Use structures not in SCOPe 2.01 in a test set.

    SCOP reference:

    A large dataset of fragments was derived from ASTRAL SCOP 1.75A,[29] filtered by 40% sequence identity and retaining only X-ray crystallography-derived structures with high reliabil- ity and precision, as defined by an AEROSPACI quality score >0.5 (this score is approximately the reciprocal of the resolu- tion).[29] Finally, PSI-BLAST[30] was used to filter structures that were homologous to those in our first test set (Table 1) to avoid overfitting. This left approximately 2800 high-resolution structures in our training set.

    The training set of PDB structures was then decomposed into fragments.

    ...

    The second test set was made up of a filtered set of struc- tures deposited to the RCSB PBD between 15=03=2012 and 08=06=2012, after the date ASTRAL-SCOP 1.75A was released. The structures in this set were required to be better than 2 A ̊ resolution X-ray crystallography-derived protein structures with no more than 30% sequence similarity to each other. Addition- ally, this set was filtered for proteins with backbone chain breaks, unusual backbone bond lengths or multiple chains. This left 28 structures in the second test set.

  ### Attachments

  - jcc23330.pdf
- ## High-throughput analytical gel filtration screening of integral membrane proteins for structural studies

  |  |  |
  | --- | --- |
  | Type | Journal Article |
  | Author | Christian Low |
  | Author | Per Moberg |
  | Author | Esben M. Quistgaard |
  | Author | Marie Hedren |
  | Author | Fatma Guettou |
  | Author | Jens Frauenfeld |
  | Author | Lars Haneskog |
  | Author | Par Nordlund |
  | Volume | 1830 |
  | Issue | 6 |
  | Pages | 3497–3508 |
  | Publication | Biochimica Et Biophysica Acta-general Subjects |
  | Date | June 2013 |
  | DOI | 10.1016/j.bbagen.2013.02.001 |
  | Abstract | Background: Structural studies of integral membrane proteins (IMPs) are often hampered by difficulties in producing stable homogenous samples for crystallization. To overcome this hurdle it has become common practice to screen large numbers of target proteins to find suitable candidates for crystallization. For such an approach to be effective, an efficient screening strategy is imperative. To this end, strategies have been developed that involve the use of green fluorescent protein (GFP) fusion constructs. However, these approaches suffer from two drawbacks: proteins with a translocated C-terminus cannot be tested and scale-up from analytical to preparative purification is often non-trivial and may require re-cloning. Methods: Here we present a screening approach that prioritizes IMP targets based on three criteria: expression level, detergent solubilization yield and homogeneity as determined by high-throughput small-scale immobilized metal affinity chromatography (IMAC) and automated size-exclusion chromatography (SEC). Results: To validate the strategy, we screened 48 prokaryotic IMPs in two different vectors and two Escherichia coli strains. A set of 11 proteins passed all preset quality control checkpoints and was subjected to crystallization trials. Four of these crystallized directly in initial sparse matrix screens, highlighting the robustness of the strategy. Conclusions: We have developed a rapid and cost efficient screening strategy that can be used for all IMPs regardless of topology. The analytical steps have been designed to be a good mimic of preparative purification, which greatly facilitates scale-up. General significance: The screening approach presented here is intended and expected to help drive forward structural biology of membrane proteins. (C) 2013 Elsevier B.V. All rights reserved. |
  | Date Added | 3/7/2014, 12:08:00 PM |
  | Modified | 3/7/2014, 12:08:00 PM |
- ## H-InvDB in 2013: an omics study platform for human functional gene and transcript discovery

  |  |  |
  | --- | --- |
  | Type | Journal Article |
  | Author | Jun-ichi Takeda |
  | Author | Chisato Yamasaki |
  | Author | Katsuhiko Murakami |
  | Author | Yoko Nagai |
  | Author | Miho Sera |
  | Author | Yuichiro Hara |
  | Author | Nobuo Obi |
  | Author | Takuya Habara |
  | Author | Takashi Gojobori |
  | Author | Tadashi Imanishi |
  | Volume | 41 |
  | Issue | D1 |
  | Pages | D915-D919 |
  | Publication | Nucleic Acids Research |
  | ISSN | 0305-1048 |
  | Date | JAN 2013 |
  | Extra | WOS:000312893300129 |
  | DOI | 10.1093/nar/gks1245 |
  | Abstract | H-InvDB (http://www.h-invitational.jp/) is a comprehensive human gene database started in 2004. In the latest version, H-InvDB 8.0, a total of 244 709 human complementary DNA was mapped onto the hg19 reference genome and 43 829 gene loci, including nonprotein-coding ones, were identified. Of these loci, 35 631 were identified as potential protein-coding genes, and 22 898 of these were identical to known genes. In our analysis, 19 309 annotated genes were specific to H-InvDB and not found in RefSeq and Ensembl. In fact, 233 genes of the 19 309 turned out to have protein functions in this version of H-InvDB; they were annotated as unknown protein functions in the previous version. Furthermore, 11 genes were identified as known Mendelian disorder genes. It is advantageous that many biologically functional genes are hidden in the H-InvDB unique genes. As large-scale proteomic projects have been conducted to elucidate the functions of all human proteins, we have enhanced the proteomic information with an advanced protein view and new subdatabase of protein complexes (Protein Complex Database with quality index). We propose that H-InvDB is an important resource for finding novel candidate targets for medical care and drug development. |
  | Date Added | 2/20/2014, 12:24:01 PM |
  | Modified | 2/20/2014, 12:24:01 PM |

  ### Notes:

  - Present update to H-InvDB database for gene annotation and transcript discovery.

    How SCOP is used:

    Use HEAT annotation tool to annotate sequences with SCOP annotations, amongst other annotations.

    SCOP reference:

    Among the H-Inv satellite databases, H-InvDB Enrichment Analysis Tool (HEAT) (8) was con- siderably upgraded. HEAT is a tool for gene-set enrich- ment analysis based on various annotation in H-InvDB, such as InterPro (18), GO (19), KEGG pathway (20), SCOP (21), subcellular localization, chromosomal band, gene family and tissue specific expression in H-ANGEL (10). It searches for H-InvDB annotations that are signifi- cantly enriched in a user-defined gene sets as compared with the entire H-InvDB representative protein-coding transcripts.

  ### Attachments

  - Nucl. Acids Res.-2013-Takeda-D915-9.pdf
- ## History of biological metal utilization inferred through phylogenomic analysis of protein structures

  |  |  |
  | --- | --- |
  | Type | Journal Article |
  | Author | Christopher L Dupont |
  | Author | Andrew Butcher |
  | Author | Ruben E Valas |
  | Author | Philip E Bourne |
  | Author | Gustavo Caetano-Anollés |
  | Volume | 107 |
  | Issue | 23 |
  | Pages | 10567-10572 |
  | Publication | Proceedings of the National Academy of Sciences of the United States of America |
  | ISSN | 1091-6490 |
  | Date | Jun 8, 2010 |
  | Extra | PMID: 20498051 |
  | Journal Abbr | Proc. Natl. Acad. Sci. U.S.A. |
  | DOI | 10.1073/pnas.0912491107 |
  | Library Catalog | NCBI PubMed |
  | Language | eng |
  | Abstract | The fundamental chemistry of trace elements dictates the molecular speciation and reactivity both within cells and the environment at large. Using protein structure and comparative genomics, we elucidate several major influences this chemistry has had upon biology. All of life exhibits the same proteome size-dependent scaling for the number of metal-binding proteins within a proteome. This fundamental evolutionary constant shows that the selection of one element occurs at the exclusion of another, with the eschewal of Fe for Zn and Ca being a defining feature of eukaryotic proteomes. Early life lacked both the structures required to control intracellular metal concentrations and the metal-binding proteins that catalyze electron transport and redox transformations. The development of protein structures for metal homeostasis coincided with the emergence of metal-specific structures, which predominantly bound metals abundant in the Archean ocean. Potentially, this promoted the diversification of emerging lineages of Archaea and Bacteria through the establishment of biogeochemical cycles. In contrast, structures binding Cu and Zn evolved much later, providing further evidence that environmental availability influenced the selection of the elements. The late evolving Zn-binding proteins are fundamental to eukaryotic cellular biology, and Zn bioavailability may have been a limiting factor in eukaryotic evolution. The results presented here provide an evolutionary timeline based on genomic characteristics, and key hypotheses can be tested by alternative geochemical methods. |
  | Date Added | 10/11/2013, 10:29:15 AM |
  | Modified | 10/11/2013, 10:29:15 AM |

  ### Tags:

  - Interesting

  ### Notes:

  - Study protein evolution in terms of adopting metal-binding sites.

    How SCOP is used:

    Use SCOP 1.69.  Manually inspect each SF for structures containing covalently bonded metal ions.

    Retrieve PDB structures and label SFs and families as 'metal-binding' if they contain only metal-binding proteins.

    SCOP reference:

    Results  
     Overview. The evolutionary units in our study are protein struc- tural domains: more specifically, compact and independently folding 3D architectures. The structural classification of proteins (SCOP) uses structural similarity to group domain fold-struc- tures into fold superfamilies (FSF), which are one or more evolutionarily related protein fold-families (FF) with little se- quence similarity. FFs are sequence clusters nested within each FSF (20). SCOP (20) provides a hierarchical classification of all protein domains published in the Protein Data Bank (PDB) (21) and version 1.69 of SCOP sorts 70,800 domains into 945 defined folds that are assigned to 1,539 FSFs, which are further sub- divided into 2,845 FFs. In the simplest scenario, a protein con- tains one domain belonging to a FF and its parent FSF. More complex proteins contain multiple domains from one or more FF and FSF. Here, we examine the distribution of metal-binding FFs in extant genomes, simultaneously examining the temporal evolution of the parent category, the FSFs.

  ### Attachments

  - PNAS-2010-Dupont-10567-72.pdf
  - PubMed entry
- ## How many drug targets are there?

  |  |  |
  | --- | --- |
  | Type | Journal Article |
  | Author | John P. Overington |
  | Author | Bissan Al-Lazikani |
  | Author | Andrew L. Hopkins |
  | URL | http://www.nature.com/nrd/journal/v5/n12/abs/nrd2199.html |
  | Volume | 5 |
  | Issue | 12 |
  | Pages | 993–996 |
  | Publication | Nature reviews Drug discovery |
  | Date | 2006 |
  | Accessed | 10/10/2013, 1:19:17 PM |
  | Library Catalog | Google Scholar |
  | Date Added | 10/11/2013, 10:29:15 AM |
  | Modified | 10/11/2013, 10:29:15 AM |

  ### Notes:

  - How SCOP is used:

    Annotate a data set of drug targets with SCOP and Pfam classification.  Found very few families (130) are represented.

    SCOP reference:

    In order to identify the familial relation- ships between all drug targets, we analysed the presence of domains, using the SCOP19 and PFAM20 databases. Approximately 130 ‘privileged druggable domains’ cover all current drug targets. This number is in stark contrast to the projected number of protein families and folds (10,000 folds21 and more than 16,000 families22).

  ### Attachments

  - [PDF] from ksu.edu.sa
  - Snapshot
- ## How Many Protein-Protein Interactions Types Exist in Nature?

  |  |  |
  | --- | --- |
  | Type | Journal Article |
  | Author | Leonardo Garma |
  | Author | Srayanta Mukherjee |
  | Author | Pralay Mitra |
  | Author | Yang Zhang |
  | URL | http://dx.doi.org/10.1371/journal.pone.0038913 |
  | Volume | 7 |
  | Issue | 6 |
  | Pages | e38913 |
  | Publication | PLoS one |
  | Date | June 13, 2012 |
  | Journal Abbr | PLoS ONE |
  | DOI | 10.1371/journal.pone.0038913 |
  | Accessed | 9/20/2013, 12:41:20 PM |
  | Library Catalog | PLoS Journals |
  | Abstract | “Protein quaternary structure universe” refers to the ensemble of all protein-protein complexes across all organisms in nature. The number of quaternary folds thus corresponds to the number of ways proteins physically interact with other proteins. This study focuses on answering two basic questions: Whether the number of protein-protein interactions is limited and, if yes, how many different quaternary folds exist in nature. By all-to-all sequence and structure comparisons, we grouped the protein complexes in the protein data bank (PDB) into 3,629 families and 1,761 folds. A statistical model was introduced to obtain the quantitative relation between the numbers of quaternary families and quaternary folds in nature. The total number of possible protein-protein interactions was estimated around 4,000, which indicates that the current protein repository contains only 42% of quaternary folds in nature and a full coverage needs approximately a quarter century of experimental effort. The results have important implications to the protein complex structural modeling and the structure genomics of protein-protein interactions. |
  | Date Added | 10/11/2013, 10:29:15 AM |
  | Modified | 3/7/2014, 12:14:54 PM |

  ### Tags:

  - Databases, Protein
  - Models, Theoretical
  - Protein Binding
  - Proteins

  ### Notes:

  - Computational study to cluster protein complexes in PDB by structure similarity using a specialized complex alignment method, MM-Align.

    Notes that the protein tertiary space is limited, and that SCOP has 1200 folds.  In contrast to the extensive studies on tertiary structure space, quaternary structure space studies have been limited.

    How SCOP is used:

    Background information on previous assessment of TM-score on SCOP and CATH data.

    SCOP reference:

    Introduction

    The protein universe refers to a collection of all proteins across all organisms in nature [1]. In 1992, there were only 887 protein structures in the Protein Data Bank (PDB) which could be categorized into 120 different tertiary folds. Chothia [2] noticed that about 1/4 of the entries at the EMBL/SwissProt sequence databank were homologous to the 120 folds, and 1/3 of the genome sequences presented in the sequence databank. He thereby suggested that the number of protein tertiary folds in the protein universe should be limited and around 1500 (,1206364). Amazingly, this simple estimation stood well the test of time and lies at the center of the subsequent estimation range (1000–2000) using more elaborate methods based on much larger datasets [3,4,5,6]. At present, the PDB has over 70 k structures, which has been argued to be structurally complete [1,7,8,9]. The structure set has been categorized into 1,195 folds by SCOP [10] in the 2009 release, consistent with the Chothia’s original estimation.

    ...

    Assessment of Complex Structure Similarity

    ...

    Quantitatively, for tertiary protein structures, it has been shown [25] that the posterior probability of TM-score of random protein structure pairs has a rapid phase transition at TM-score = 0.5 and the structures of TM-score .0.5 approximately corresponds to the same protein folds as defined by SCOP [10] and CATH [26] databases.

  ### Attachments

  - PLoS Full Text PDF
- ## How old is my gene?

  |  |  |
  | --- | --- |
  | Type | Journal Article |
  | Author | John A. Capra |
  | Author | Maureen Stolzer |
  | Author | Dannie Durand |
  | Author | Katherine S. Pollard |
  | Volume | 29 |
  | Issue | 11 |
  | Pages | 659-668 |
  | Publication | Trends in Genetics |
  | ISSN | 0168-9525 |
  | Date | NOV 2013 |
  | Extra | WOS:000326902000007 |
  | DOI | 10.1016/j.tig.2013.07.001 |
  | Abstract | Gene functions, interactions, disease associations, and ecological distributions are all correlated with gene age. However, it is challenging to estimate the intricate series of evolutionary events leading to a modern-day gene and then to reduce this history to a single age estimate. Focusing on eukaryotic gene families, we introduce a framework that can be used to compare current strategies for quantifying gene age, discuss key differences between these methods, and highlight several common problems. We argue that genes with complex evolutionary histories do not have a single well-defined age. As a result, care must be taken to articulate the goals and assumptions of any analysis that uses gene age estimates. Recent algorithmic advances offer the promise of gene age estimates that are fast, accurate, and consistent across gene families. This will enable a shift to integrated genome-wide analyses of all events in gene evolutionary histories in the near future. |
  | Date Added | 2/12/2014, 1:36:22 PM |
  | Modified | 3/7/2014, 12:10:10 PM |

  ### Notes:

  - Review of method for predicting gene age.

    How SCOP/CATH is used:

    Background on protein structure classification.

    SCOP/CATH reference:

    Many additional sequence-comparison methods have been developed that are able to detect more remote homology by building statistical profiles from multiple alignments of sequences related to the query (e.g., [59–61]) or analyzing known and predicted structural similarities (e.g, SCOP [62,63]).

  ### Attachments

  - 1-s2.0-S016895251300111X-main.pdf
- ## How significant is a protein structure similarity with TM-score = 0.5?

  |  |  |
  | --- | --- |
  | Type | Journal Article |
  | Author | Jinrui Xu |
  | Author | Yang Zhang |
  | Volume | 26 |
  | Issue | 7 |
  | Pages | 889-895 |
  | Publication | Bioinformatics (Oxford, England) |
  | ISSN | 1367-4811 |
  | Date | Apr 1, 2010 |
  | Extra | PMID: 20164152 |
  | Journal Abbr | Bioinformatics |
  | DOI | 10.1093/bioinformatics/btq066 |
  | Library Catalog | NCBI PubMed |
  | Language | eng |
  | Abstract | MOTIVATION: Protein structure similarity is often measured by root mean squared deviation, global distance test score and template modeling score (TM-score). However, the scores themselves cannot provide information on how significant the structural similarity is. Also, it lacks a quantitative relation between the scores and conventional fold classifications. This article aims to answer two questions: (i) what is the statistical significance of TM-score? (ii) What is the probability of two proteins having the same fold given a specific TM-score? RESULTS: We first made an all-to-all gapless structural match on 6684 non-homologous single-domain proteins in the PDB and found that the TM-scores follow an extreme value distribution. The data allow us to assign each TM-score a P-value that measures the chance of two randomly selected proteins obtaining an equal or higher TM-score. With a TM-score at 0.5, for instance, its P-value is 5.5 x 10(-7), which means we need to consider at least 1.8 million random protein pairs to acquire a TM-score of no less than 0.5. Second, we examine the posterior probability of the same fold proteins from three datasets SCOP, CATH and the consensus of SCOP and CATH. It is found that the posterior probability from different datasets has a similar rapid phase transition around TM-score=0.5. This finding indicates that TM-score can be used as an approximate but quantitative criterion for protein topology classification, i.e. protein pairs with a TM-score >0.5 are mostly in the same fold while those with a TM-score <0.5 are mainly not in the same fold. |
  | Date Added | 10/11/2013, 10:29:15 AM |
  | Modified | 10/11/2013, 10:29:15 AM |

  ### Notes:

  - investigates protein-structure similarity scoring significance, in particular the template modeling score (TM-score).

    How SCOP is used:

    Use SCOP for benchmarking TM-scoring method.  Create a data set of protein pairs with the same and different folds.

    Use SCOP 1.73. Filter by >95% sequence identity and also remove chains with fewer than 80 residues.  Resulted in ~750K pairs of proteins that are deemed to share the same fold.  Also use a consensus data set built from SCOP and CATH.

    SCOP reference:

    Under Abstract:

    Second, we examine the posterior probability of the same fold proteins from three datasets SCOP, CATH and the consensus of SCOP and CATH.

    Dataset of proteins with same/different folds

    To estimate the posterior probability for structure pairs at given TM-scores sharing the same topology, a collection of protein pairs in both the same and the different folds is necessary. For this purpose, we borrow the Fold and Topology definition from the standard protein structure classification databases: SCOP (Andreeva et al., 2008) and CATH (Cuff et al., 2009) to generate the same and different fold datasets.

    .3.1 Three sets of same fold structure pairs The first set of protein domains (Set-I) are collected from the SCOP 1.73 database. After filtering out the redundant proteins with a sequence identity >95% and the small proteins with length below 80 amino acids, 11 239 protein domains remain, which cover 551 main Fold families in SCOP. An all-to-all pairing is then carried out for the proteins within the same Fold family and ends up with a total of 746 420 protein pairs which are considered as sharing same folds in SCOP.

    The second set of protein domains (Set-II) are from CATH 3.2.0. The structure pairs are generated from the proteins in the same ‘Topology’, a structural level equivalent to the ‘Fold’ in SCOP (Hadley and Jones, 1999). After the same redundancy and length filtering, 14 830 domains covering 700 main Topologies in CATH are obtained. An all-to-all pairing among proteins of the same Topology families results in 2 769 868 domain pairs. The reason for Set-II being much bigger than Set-I is due to the fact that some CATH families have a dominantly large size.

    The third protein pair set (Set-III) is a consensus of the SCOP and CATH databases where the proteins are of the same fold in both SCOP and CATH. Due to the different domain splitting system, SCOP and CATH may have protein domains with the same ID (the same PDB names and chains) but having different sequence segments. To ensure that SCOP and CATH deal with the same structures, we filter out those inconsistent domains and collect only the structures which have the same IDs in the SCOP and CATH and meanwhile have the identical regions covering >90% of both the SCOP and CATH domains. By these criteria, 5105 domain structures are culled from SCOP with a counterpart in CATH, which cover 328 different fold families. An all-to-all pairing is carried out among the proteins which are consistently defined by SCOP and CATH as being of the same fold, resulting in 186 359 protein pairs.

  ### Attachments

  - Bioinformatics-2010-Xu-889-95.pdf
  - PubMed entry
- ## How to evaluate performance of prediction methods? Measures and their interpretation in variation effect analysis

  |  |  |
  | --- | --- |
  | Type | Journal Article |
  | Author | Mauno Vihinen |
  | Volume | 13 |
  | Pages | S2 |
  | Publication | Bmc Genomics |
  | ISSN | 1471-2164 |
  | Date | JUN 18 2012 |
  | Extra | WOS:000306145100002 |
  | DOI | 10.1186/1471-2164-13-S4-S2 |
  | Abstract | Background: Prediction methods are increasingly used in biosciences to forecast diverse features and characteristics. Binary two-state classifiers are the most common applications. They are usually based on machine learning approaches. For the end user it is often problematic to evaluate the true performance and applicability of computational tools as some knowledge about computer science and statistics would be needed. Results: Instructions are given on how to interpret and compare method evaluation results. For systematic method performance analysis is needed established benchmark datasets which contain cases with known outcome, and suitable evaluation measures. The criteria for benchmark datasets are discussed along with their implementation in VariBench, benchmark database for variations. There is no single measure that alone could describe all the aspects of method performance. Predictions of genetic variation effects on DNA, RNA and protein level are important as information about variants can be produced much faster than their disease relevance can be experimentally verified. Therefore numerous prediction tools have been developed, however, systematic analyses of their performance and comparison have just started to emerge. Conclusions: The end users of prediction tools should be able to understand how evaluation is done and how to interpret the results. Six main performance evaluation measures are introduced. These include sensitivity, specificity, positive predictive value, negative predictive value, accuracy and Matthews correlation coefficient. Together with receiver operating characteristics (ROC) analysis they provide a good picture about the performance of methods and allow their objective and quantitative comparison. A checklist of items to look at is provided. Comparisons of methods for missense variant tolerance, protein stability changes due to amino acid substitutions, and effects of variations on mRNA splicing are presented. |
  | Date Added | 2/13/2014, 4:13:17 PM |
  | Modified | 10/8/2014, 1:32:27 PM |

  ### Attachments

  - Full Text PDF
  - Snapshot
- ## Human Cytomegalovirus Gene UL76 Induces IL-8 Expression through Activation of the DNA Damage Response

  |  |  |
  | --- | --- |
  | Type | Journal Article |
  | Author | Helena Costa |
  | Author | Rute Nascimento |
  | Author | John Sinclair |
  | Author | Robert Michael Evans Parkhouse |
  | Volume | 9 |
  | Issue | 9 |
  | Pages | e1003609 |
  | Publication | Plos Pathogens |
  | ISSN | 1553-7374 |
  | Date | SEP 2013 |
  | Extra | WOS:000324922300028 |
  | DOI | 10.1371/journal.ppat.1003609 |
  | Abstract | Human cytomegalovirus (HCMV), a beta-herpesvirus, has evolved many strategies to subvert both innate and adaptive host immunity in order to ensure its survival and propagation within the host. Induction of IL-8 is particularly important during HCMV infection as neutrophils, primarily attracted by IL-8, play a key role in virus dissemination. Moreover, IL-8 has a positive effect in the replication of HCMV. This work has identified an HCMV gene (UL76), with the relevant property of inducing IL-8 expression at both transcriptional and protein levels. Up-regulation of IL-8 by UL76 results from activation of the NF-kB pathway as inhibition of both IKK-beta activity or degradation of Ikb alpha abolishes the IL-8 induction and, concomitantly, expression of UL76 is associated with the translocation of p65 to the nucleus where it binds to the IL-8 promoter. Furthermore, the UL76-mediated induction of IL-8 requires ATM and is correlated with the phosphorylation of NEMO on serine 85, indicating that UL76 activates NF-kB pathway by the DNA Damage response, similar to the impact of genotoxic drugs. More importantly, a UL76 deletion mutant virus was significantly less efficient in stimulating IL-8 production than the wild type virus. In addition, there was a significant reduction of IL-8 secretion when ATM -/- cells were infected with wild type HCMV, thus, indicating that ATM is also involved in the induction of IL-8 by HCMV. In conclusion, we demonstrate that expression of UL76 gene induces IL-8 expression as a result of the DNA damage response and that both UL76 and ATM have a role in the mechanism of IL-8 induction during HCMV infection. Hence, this work characterizes a new role of the activation of DNA Damage response in the context of host-pathogen interactions. |
  | Date Added | 2/12/2014, 1:36:22 PM |
  | Modified | 2/12/2014, 1:36:22 PM |

  ### Notes:

  - Study function of a herpesvirus gene, UL76.

    How SCOP is used:

    Look at functions in related families within the same superfamily  as UL76, the protein of interest.

    SCOP reference:

    This superfamily of restriction endonuclease- like fold proteins includes several restriction endonucleases (e.g. EcoRI, EcoRII, BamHI, BglI, Cfr10I, NaeI), DNA repair enzymes (MutH and Vsr), Holliday junction resolvases (Hjc and Hje) and other nucleotide-cleaving enzymes [15].

  ### Attachments

  - journal.ppat.1003609.pdf
- ## Human Lipoxygenase: Developments in its Structure, Function, Relevance to Diseases and Challenges in Drug Development

  |  |  |
  | --- | --- |
  | Type | Journal Article |
  | Author | E. Skrzypczak-Jankun |
  | Author | J. Jankun |
  | Author | A. Al-Senaidy |
  | Volume | 19 |
  | Issue | 30 |
  | Pages | 5122–5127 |
  | Publication | Current Medicinal Chemistry |
  | Date | October 2012 |
  | Abstract | Human lipoxygenases (LOXs) are the enzymes participating in the metabolism of the polyunsaturated fatty acids and catalyzing their oxidation to a variety of eicosanoids, which as the secondary signal transducers have a major impact on human homeostasis. They are involved in many diseases such as inflammatory responses, cancers, cardiovascular and kidney diseases, neurodegenerative disorders and metabolic syndrome. This review summarizes recent developments concerning human 12S-LOX and rabbit 15-LOX projected upon available structural data of LOX and COX oxidoreductases, with conclusions that might apply to LOX family of enzymes in general. Namely: (i) Human lipoxygenases might act as oligomers consisting of active and apo monomers. (ii) Sequential homodimers might act as structural heterodimers with the dimeric interface formed by the interactions resembling the leucine zipper in the coiled-coil superstructure. (iii) Two commonly recognized domains are not sufficient to explain LOX flexibility. Molecular architecture should contain assignment of another regulatory domain of alpha-beta character, possibly important in molecular signaling, which might provide another avenue for targeted drug development. (iv) Allosteric mechanism might involve orchestrated conformational changes and flexibility of the coils connecting the structured elements and ligands binding in more than one monomer. |
  | Date Added | 3/7/2014, 12:08:00 PM |
  | Modified | 3/7/2014, 12:08:00 PM |
- ## Human Prostatic Acid Phosphatase: Structure, Function and Regulation

  |  |  |
  | --- | --- |
  | Type | Journal Article |
  | Author | Sakthivel Muniyan |
  | Author | Nagendra K. Chaturvedi |
  | Author | Jennifer G. Dwyer |
  | Author | Chad A. LaGrange |
  | Author | William G. Chaney |
  | Author | Ming-Fong Lin |
  | URL | http://www.mdpi.com/1422-0067/14/5/10438 |
  | Volume | 14 |
  | Issue | 5 |
  | Pages | 10438–10464 |
  | Publication | International journal of molecular sciences |
  | Date | 2013 |
  | Accessed | 9/23/2013, 10:15:34 AM |
  | Library Catalog | Google Scholar |
  | Short Title | Human Prostatic Acid Phosphatase |
  | Date Added | 2/20/2014, 12:24:01 PM |
  | Modified | 2/20/2014, 12:24:01 PM |

  ### Notes:

  - Review of research on human prostatic acid phosphatase (hPAcP).

    How SCOP is used:

    Look up structural class of protein.

    SCOP reference:

    Secondary structural analyses demonstrated that hPAcP is composed of 44% α-helix (16 helices; 158 residues) [77], 12% β-strand (ten strands; 45 residues) and the rest are loops and β-turns [78].

  ### Attachments

  - [PDF] from mdpi.com
- ## Hybrid Sequencing Approach Applied to Human Fecal Metagenomic Clone Libraries Revealed Clones with Potential Biotechnological Applications

  |  |  |
  | --- | --- |
  | Type | Journal Article |
  | Author | Maria Dzunkova |
  | Author | Giuseppe D'Auria |
  | Author | David Perez-Villarroya |
  | Author | Andres Moya |
  | Volume | 7 |
  | Issue | 10 |
  | Pages | e47654 |
  | Publication | Plos One |
  | Date | October 2012 |
  | DOI | 10.1371/journal.pone.0047654 |
  | Abstract | Natural environments represent an incredible source of microbial genetic diversity. Discovery of novel biomolecules involves biotechnological methods that often require the design and implementation of biochemical assays to screen clone libraries. However, when an assay is applied to thousands of clones, one may eventually end up with very few positive clones which, in most of the cases, have to be "domesticated” for downstream characterization and application, and this makes screening both laborious and expensive. The negative clones, which are not considered by the selected assay, may also have biotechnological potential; however, unfortunately they would remain unexplored. Knowledge of the clone sequences provides important clues about potential biotechnological application of the clones in the library; however, the sequencing of clones one-by-one would be very time-consuming and expensive. In this study, we characterized the first metagenomic clone library from the feces of a healthy human volunteer, using a method based on 454 pyrosequencing coupled with a clone-by-clone Sanger end-sequencing. Instead of whole individual clone sequencing, we sequenced 358 clones in a pool. The medium-large insert (7-15 kb) cloning strategy allowed us to assemble these clones correctly, and to assign the clone ends to maintain the link between the position of a living clone in the library and the annotated contig from the 454 assembly. Finally, we found several open reading frames (ORFs) with previously described potential medical application. The proposed approach allows planning ad-hoc biochemical assays for the clones of interest, and the appropriate sub-cloning strategy for gene expression in suitable vectors/hosts. |
  | Date Added | 3/7/2014, 12:08:00 PM |
  | Modified | 3/7/2014, 12:08:00 PM |
- ## IDEAL: Intrinsically Disordered proteins with Extensive Annotations and Literature

  |  |  |
  | --- | --- |
  | Type | Journal Article |
  | Author | Satoshi Fukuchi |
  | Author | Shigetaka Sakamoto |
  | Author | Yukiko Nobe |
  | Author | Seiko D. Murakami |
  | Author | Takayuki Amemiya |
  | Author | Kazuo Hosoda |
  | Author | Ryotaro Koike |
  | Author | Hidekazu Hiroaki |
  | Author | Motonori Ota |
  | Volume | 40 |
  | Issue | D1 |
  | Pages | D507-D511 |
  | Publication | Nucleic Acids Research |
  | ISSN | 0305-1048 |
  | Date | JAN 2012 |
  | Extra | WOS:000298601300075 |
  | DOI | 10.1093/nar/gkr884 |
  | Abstract | IDEAL, Intrinsically Disordered proteins with Extensive Annotations and Literature (http://www.ideal.force.cs.is.nagoya-u.ac.jp/IDEAL/), is a collection of knowledge on experimentally verified intrinsically disordered proteins. IDEAL contains manual annotations by curators on intrinsically disordered regions, interaction regions to other molecules, post-translational modification sites, references and structural domain assignments. In particular, IDEAL explicitly describes protean segments that can be transformed from a disordered state to an ordered state. Since in most cases they can act as molecular recognition elements upon binding of partner proteins, IDEAL provides a data resource for functional regions of intrinsically disordered proteins. The information in IDEAL is provided on a user-friendly graphical view and in a computer-friendly XML format. |
  | Date Added | 2/20/2014, 12:24:01 PM |
  | Modified | 3/7/2014, 12:14:58 PM |

  ### Notes:

  - Present IDEAL database of intrinsically disordered proteins with extensive annotations and literature.

    How SCOP is used:

    Annotate sequences with SCOP and Pfam domains.  For SCOP, use both SCOP RPS-BLAST and SCOP Hmmer.

    How CATH is used:

    Not using CATH data.

    SCOP reference:

    MISCELLANEOUS INFORMATION

    We integrate the miscellaneous information from UniProt, namely, regions interacting with other molecules, motifs and post-translational modifications. During the annota- tion process, the curators find interaction sites, sequence motifs or other information that has not been described in UniProt, the new information is included in IDEAL. IDEAL also provides SCOP (version 1.75) and Pfam (23) (version 24.0) domain assignments using reverse PSI-Blast (24) and HMMer (25). Note that ordered regions assigned in the order/disorder annotation process are experimentally verified ordered regions, while the structural domain assignments were done using homology searches.

    SCOP/CATH reference:

    onsidering that the protein 3D structural databases such as PDB, SCOP (Structural Classification of Proteins) (8) and CATH (9), have played important roles in deepening our understand- ing of the nature of protein structures and functions, the development of IDP databases are essential to the progress of IDP research.

  ### Attachments

  - Nucl. Acids Res.-2012-Fukuchi-D507-11.pdf
- ## Identification and characterization of a previously undescribed family of sequence-specific DNA-binding domains

  |  |  |
  | --- | --- |
  | Type | Journal Article |
  | Author | Matthew B. Lohse |
  | Author | Aaron D. Hernday |
  | Author | Polly M. Fordyce |
  | Author | Liron Noiman |
  | Author | Trevor R. Sorrells |
  | Author | Victor Hanson-Smith |
  | Author | Clarissa J. Nobile |
  | Author | Joseph L. DeRisi |
  | Author | Alexander D. Johnson |
  | URL | http://www.pnas.org/content/110/19/7660.short |
  | Volume | 110 |
  | Issue | 19 |
  | Pages | 7660–7665 |
  | Publication | Proceedings of the National Academy of Sciences |
  | Date | 2013 |
  | Accessed | 9/20/2013, 1:12:22 PM |
  | Library Catalog | Google Scholar |
  | Date Added | 10/11/2013, 10:29:15 AM |
  | Modified | 10/11/2013, 10:29:15 AM |

  ### Notes:

  - Study a sequence-specific DNA-binding protein that has no detectable homology with any previously studied RNA- or DNA-binding protein.  Their observations imply that there may be many small clades for sequence-specific DNA-binding proteins.  
      
     How using SCOP:  
      
    SCOP is used to highlight the diversity of superfamilies in which DNA-binding proteins are found.  
      
    Reference to SCOP:  
      
    Regulation of gene expression by sequence-specific DNA-binding proteins underlies many biological processes, from environmental responses in single-celled organisms to the development of multicellular structures in animals and plants.  
      
    Between 5% and 10% of the coding capacity of most genomes is dedicated to these proteins, and they can be arranged into numerous families and superfamilies based on their amino acid sequences and the structural motifs through which DNA is recognized (1).

  ### Attachments

  - Full Text PDF
  - [HTML] from pnas.org
  - PubMed entry
  - Snapshot
- ## Identification of Catalytic Residues Using a Novel Feature that Integrates the Microenvironment and Geometrical Location Properties of Residues

  |  |  |
  | --- | --- |
  | Type | Journal Article |
  | Author | Lei Han |
  | Author | Yong-Jun Zhang |
  | Author | Jiangning Song |
  | Author | Ming S. Liu |
  | Author | Ziding Zhang |
  | Volume | 7 |
  | Issue | 7 |
  | Publication | PLOS ONE |
  | ISSN | 1932-6203 |
  | Date | JUL 19 2012 |
  | DOI | 10.1371/journal.pone.0041370 |
  | Language | English |
  | Abstract | Enzymes play a fundamental role in almost all biological processes and identification of catalytic residues is a crucial step for deciphering the biological functions and understanding the underlying catalytic mechanisms. In this work, we developed a novel structural feature called MEDscore to identify catalytic residues, which integrated the microenvironment (ME) and geometrical properties of amino acid residues. Firstly, we converted a residue's ME into a series of spatially neighboring residue pairs, whose likelihood of being located in a catalytic ME was deduced from a benchmark enzyme dataset. We then calculated an ME-based score, termed as MEscore, by summing up the likelihood of all residue pairs. Secondly, we defined a parameter called Dscore to measure the relative distance of a residue to the center of the protein, provided that catalytic residues are typically located in the center of the protein structure. Finally, we defined the MEDscore feature based on an effective nonlinear integration of MEscore and Dscore. When evaluated on a well-prepared benchmark dataset using fivefold cross-validation tests, MEDscore achieved a robust performance in identifying catalytic residues with an AUC1.0 of 0.889. At a <= 10% false positive rate control, MEDscore correctly identified approximately 70% of the catalytic residues. Remarkably, MEDscore achieved a competitive performance compared with the residue conservation score (e.g. CONscore), the most informative singular feature predominantly employed to identify catalytic residues. To the best of our knowledge, MEDscore is the first singular structural feature exhibiting such an advantage. More importantly, we found that MEDscore is complementary with CONscore and a significantly improved performance can be achieved by combining CONscore with MEDscore in a linear manner. As an implementation of this work, MEDscore has been made freely accessible at http://protein.cau. edu.cn/mepi/. |
  | Date Added | 10/25/2013, 4:17:08 PM |
  | Modified | 10/25/2013, 4:17:08 PM |

  ### Notes:

  - Present a novel method to predict catalytic residues.

    How SCOP is used:

    Get domains and SCOP classification for data set of enzymes.  Retrieve structures from ASTRAL.  Use SCOP classification to filter by class and count the number of folds, superfamilies, and families present in the remaining 223 enzymes.

    SCOP reference:

    Materials and Methods

    Benchmark enzyme dataset

    The benchmark enzyme dataset used in this study was extracted from the Catalytic Site Atlas (CSA) database (version 2.2.12) [71]. **Total of 7,124 entries with catalytic residues annotated directly in the literature were extracted. These entries were mapped onto the SCOP database (version 1.75) [72] and the corresponding PDB files were downloaded from the ASTRAL database (http://astral. berkeley.edu/pdbstyle-1.75.html) [73].** These enzymes were further filtered based on the following criteria: a) the sequence identity between any two sequences should be less than 30%; b) the sequence length of any enzyme should be larger than 100; c) the PDB structures with 10 consecutive missing residues were excluded; d) only the PDB structures belonging to four SCOP structural classes (i.e. all-a, all-b, a+b and a/b) were included; e) if an enzyme had two or more NMR structure models in our dataset, only the first model was retained; and f) some enzymes were discarded because that the number of homologous sequences of the enzymes was insufficient to permit an accurate calculation of residue conservation scores. Based on the above criteria, 223 enzyme catalytic domains were retained in our final dataset, covering six top levels of the EC classifications. T**hese 223 enzymes cover 112 folds, 139 superfamilies and 185 families in terms of the SCOP classification**. In this non-redundant benchmark enzyme dataset, 630 residues are defined as catalytic residues according to the CSA annotation, while the remaining 60,658 residues are regarded as non-catalytic residues. The details about the enzyme dataset are listed in Supporting Information Text S1.

    ...

    In particular, the benchmark enzyme dataset was randomly divided into five subsets and each subset contained roughly equal number of protein domains (the SCOP entries of these five subsets are available in Text S1).

    ...

    We further investigated the performance of MEscore in different structural folds. As shown in Figure S5, the performance of MEscore varies in different folds.

  ### Attachments

  - journal.pone.0041370.pdf
- ## Identification of domains in protein structures from the analysis of intramolecular interactions

  |  |  |
  | --- | --- |
  | Type | Journal Article |
  | Author | Alessandro Genoni |
  | Author | Giulia Morra |
  | Author | Giorgio Colombo |
  | URL | http://pubs.acs.org/doi/abs/10.1021/jp210568a |
  | Volume | 116 |
  | Issue | 10 |
  | Pages | 3331–3343 |
  | Publication | The Journal of Physical Chemistry B |
  | Date | 2012 |
  | Accessed | 9/23/2013, 10:14:36 AM |
  | Library Catalog | Google Scholar |
  | Date Added | 10/11/2013, 10:29:15 AM |
  | Modified | 3/7/2014, 12:14:51 PM |

  ### Notes:

  - Present method for domain predictions based on analysis of intramolecular interactions

    How SCOP/CATH is used:

    Benchmark domain prediction method on the benchmark\_2 and benchmark\_3 datasets, which were derived from SCOP and CATH data.

    SCOP reference:

    Among the strategies based on the human expertise, SCOP4 and CATH5 are the most relevant and popular.

    ...

    This approach has been implemented in a computer program and it has been tested using the comprehensive benchmark data sets assembled by Bourne and co-workers (i.e., Benchmark\_2 and Bench- mark\_3) to assess the capabilities of novel or already existing algorithmic methods.31

  ### Attachments

  - jp210568a.pdf
- ## Identification of novel components of NAD-utilizing metabolic pathways and prediction of their biochemical functions

  |  |  |
  | --- | --- |
  | Type | Journal Article |
  | Author | Robson Francisco de Souza |
  | Author | L. Aravind |
  | Volume | 8 |
  | Issue | 6 |
  | Pages | 1661-1677 |
  | Publication | Molecular Biosystems |
  | ISSN | 1742-206X |
  | Date | 2012 |
  | Extra | WOS:000303776200007 |
  | DOI | 10.1039/c2mb05487f |
  | Abstract | Nicotinamide adenine dinucleotide (NAD) is a ubiquitous cofactor participating in numerous redox reactions. It is also a substrate for regulatory modifications of proteins and nucleic acids via the addition of ADP-ribose moieties or removal of acyl groups by transfer to ADP-ribose. In this study, we use in-depth sequence, structure and genomic context analysis to uncover new enzymes and substrate-binding proteins in NAD-utilizing metabolic and macromolecular modification systems. We predict that Escherichia coli YbiA and related families of domains from diverse bacteria, eukaryotes, large DNA viruses and single strand RNA viruses are previously unrecognized components of NAD-utilizing pathways that probably operate on ADP-ribose derivatives. Using contextual analysis we show that some of these proteins potentially act in RNA repair, where NAD is used to remove 2'-3' cyclic phosphodiester linkages. Likewise, we predict that another family of YbiA-related enzymes is likely to comprise a novel NAD-dependent ADP-ribosylation system for proteins, in conjunction with a previously unrecognized ADP-ribosyltransferase. A similar ADP-ribosyltransferase is also coupled with MACRO or ADP-ribosylglycohydrolase domain proteins in other related systems, suggesting that all these novel systems are likely to comprise pairs of ADP-ribosylation and ribosylglycohydrolase enzymes analogous to the DraG-DraT system, and a novel group of bacterial polymorphic toxins. We present evidence that some of these coupled ADP-ribosyltransferases/ribosylglycohydrolases are likely to regulate certain restriction modification enzymes in bacteria. The ADP-ribosyltransferases found in these, the bacterial polymorphic toxin and host-directed toxin systems of bacteria such as Waddlia also throw light on the evolution of this fold and the origin of eukaryotic polyADP-ribosyltransferases and NEURL4-like ARTs, which might be involved in centrosomal assembly. We also infer a novel biosynthetic pathway that might be involved in the synthesis of a nicotinate-derived compound in conjunction with an asparagine synthetase and AMPylating peptide ligase. We use the data derived from this analysis to understand the origin and early evolutionary trajectories of key NAD-utilizing enzymes and present targets for future biochemical investigations. |
  | Date Added | 2/20/2014, 12:24:01 PM |
  | Modified | 2/20/2014, 12:24:01 PM |

  ### Notes:

  - Computational study of NAD-utilizing pathways.

    "use in-depth sequence, structure and genomic context analysis to uncover new enzymes and substrate-binding proteins in NAD-utilizing metabolic and macromolecular modification systems."

    How SCOP is used:

    Look up fold for NAD-dependent enzymes in SCOP.

    SCOP reference:

    Comparative genomics suggests that the most ancient group of NAD-dependent enzymes is that possessing the classical Rossmann fold.89,90

  ### Attachments

  - c2mb05487f.pdf
- ## Identification of Nucleotide-Binding Sites in Protein Structures: A Novel Approach Based on Nucleotide Modularity

  |  |  |
  | --- | --- |
  | Type | Journal Article |
  | Author | Luca Parca |
  | Author | Pier Federico Gherardini |
  | Author | Mauro Truglio |
  | Author | Iolanda Mangone |
  | Author | Fabrizio Ferrè |
  | Author | Manuela Helmer-Citterich |
  | Author | Gabriele Ausiello |
  | URL | http://dx.plos.org/10.1371/journal.pone.0050240 |
  | Volume | 7 |
  | Issue | 11 |
  | Pages | e50240 |
  | Publication | PloS one |
  | Date | 2012 |
  | Accessed | 9/20/2013, 1:18:20 PM |
  | Library Catalog | Google Scholar |
  | Short Title | Identification of Nucleotide-Binding Sites in Protein Structures |
  | Date Added | 10/11/2013, 10:29:15 AM |
  | Modified | 10/11/2013, 10:29:15 AM |

  ### Tags:

  - Animals
  - Bacterial Proteins
  - Binding Sites
  - Carbohydrates
  - Computational Biology
  - Databases, Protein
  - Humans
  - Nucleotides
  - Phosphates
  - Plant Proteins
  - Protein Binding
  - Protein Folding
  - Proteins
  - Reproducibility of Results
  - Software
  - Solvents

  ### Notes:

  - Present method for nucleotide-binding site prediction from protein structure by searching a reference set  of template binding site for nucleotide "modules".

    How SCOP is used:

    Evaluate template-matching method using SCOP.  Assess how often the top-ranked result in the template search was in the same fold as the query.  Performed for 410 structures out of 924 in their dataset (limited by the structures classified in SCOP).

    SCOP reference:

    When searching for structural similarities between the query protein and the template binding sites, the method discards matches involving potentially homologous proteins. Similarly it is interesting to quantify how many times a query nucleotide-binding site is identified by a template binding site having a different protein fold. To this end we analyzed the 410 (out of 924) proteins in the dataset that are classified in the SCOP database [32]. Moreover we could not consider predictions made by template binding sites that do not have a SCOP record. The top ranked, correct, prediction has a SCOP fold different from the query protein in 50% and 52% of cases for the nucleobase and carbohydrate respectively. This percentage rises to 86% for phosphate. These results confirm that similar binding motifs for these modules occur in different protein folds [16,19].

  ### Attachments

  - journal.pone.0050240.pdf
- ## Identification of related proteins on family, superfamily and fold level

  |  |  |
  | --- | --- |
  | Type | Journal Article |
  | Author | E Lindahl |
  | Author | A Elofsson |
  | Volume | 295 |
  | Issue | 3 |
  | Pages | 613-625 |
  | Publication | Journal of molecular biology |
  | ISSN | 0022-2836 |
  | Date | Jan 21, 2000 |
  | Extra | PMID: 10623551 |
  | Journal Abbr | J. Mol. Biol. |
  | DOI | 10.1006/jmbi.1999.3377 |
  | Library Catalog | NCBI PubMed |
  | Language | eng |
  | Abstract | Proteins might have considerable structural similarities even when no evolutionary relationship of their sequences can be detected. This property is often referred to as the proteins sharing only a "fold". Of course, there are also sequences of common origin in each fold, called a "superfamily", and in them groups of sequences with clear similarities, designated "family". Developing algorithms to reliably identify proteins related at any level is one of the most important challenges in the fast growing field of bioinformatics today. However, it is not at all certain that a method proficient at finding sequence similarities performs well at the other levels, or vice versa.Here, we have compared the performance of various search methods on these different levels of similarity. As expected, we show that it becomes much harder to detect proteins as their sequences diverge. For family related sequences the best method gets 75% of the top hits correct. When the sequences differ but the proteins belong to the same superfamily this drops to 29%, and in the case of proteins with only fold similarity it is as low as 15%. We have made a more complete analysis of the performance of different algorithms than earlier studies, also including threading methods in the comparison. Using this method a more detailed picture emerges, showing multiple sequence information to improve detection on the two closer levels of relationship. We have also compared the different methods of including this information in prediction algorithms. For lower specificities, the best scheme to use is a linking method connecting proteins through an intermediate hit. For higher specificities, better performance is obtained by PSI-BLAST and some procedures using hidden Markov models. We also show that a threading method, THREADER, performs significantly better than any other method at fold recognition. |
  | Date Added | 10/22/2013, 1:01:28 PM |
  | Modified | 10/22/2013, 1:01:28 PM |

  ### Tags:

  - Algorithms
  - Evolution, Molecular
  - Protein Folding
  - Proteins
  - Sequence Homology, Amino Acid

  ### Attachments

  - PubMed entry
- ## Identification of Similar Binding Sites to Detect Distant Polypharmacology

  |  |  |
  | --- | --- |
  | Type | Journal Article |
  | Author | Xavier Jalencas |
  | Author | Jordi Mestres |
  | Volume | 32 |
  | Issue | 11-12 |
  | Pages | 976-990 |
  | Publication | Molecular Informatics |
  | ISSN | 1868-1743; 1868-1751 |
  | Date | DEC 2013 |
  | Extra | WOS:000330109500010 |
  | DOI | 10.1002/minf.201300082 |
  | Date Added | 2/20/2014, 12:24:01 PM |
  | Modified | 3/7/2014, 12:09:32 PM |

  ### Tags:

  - Interesting

  ### Notes:

  - Review of research in detecting polypharmacology using binding site similarity.

    How SCOP is used:

    1. Classify data set of 2655 drug-binding structures by SCOP classification.

    2. Classify data set of kinases by SCOP family.

    How CATH is used:

    Not using CATH data.

    SCOP reference:

    Indeed, the PDB offers structural evidence of distant pol- ypharmacology examples.[22] We took a set of 1358 ap- proved drugs (excluding nutraceuticals) from DrugBank 3.0[8] and searched them using their InChI keys in Ligand Expo.[63] Of those, 387 compounds involving 2655 struc- tures were located in the PDB, 234 of them present in more than one PDB entry. Detection of distant polypharma- cology examples was performed by assigning structur- al[165,166] and functional[24,167] classification codes to each structure. A total of 138 drugs were found to be co-crystal- lized in at least two structurally and functionally unrelated proteins.

    ...

    CavBase was used to compare and cluster a set of ATP binding sites from 258 protein kinases spanning 48 SCOP families.

     CATH reference:

    (CATH is reference number 25.)

    There is still a strong bias for recognised therapeutic targets,[23,24] but it is slowly being corrected thanks to recent structural genomics initiatives.[25,26] Conse-

  ### Attachments

  - 976\_ftp.pdf
- ## Identifying protein quaternary structural attributes by incorporating physicochemical properties into the general form of Chou's PseAAC via discrete wavelet transform

  |  |  |
  | --- | --- |
  | Type | Journal Article |
  | Author | Xing-Yu Sun |
  | Author | Shao-Ping Shi |
  | Author | Jian-Ding Qiu |
  | Author | Sheng-Bao Suo |
  | Author | Shu-Yun Huang |
  | Author | Ru-Ping Liang |
  | Volume | 8 |
  | Issue | 12 |
  | Pages | 3178–3184 |
  | Publication | Molecular Biosystems |
  | Date | 2012 |
  | DOI | 10.1039/c2mb25280e |
  | Abstract | In vivo, some proteins exist as monomers and others as oligomers. Oligomers can be further classified into homo-oligomers (formed by identical subunits) and hetero-oligomers (formed by different subunits), and they form the structural components of various biological functions, including cooperative effects, allosteric mechanism and ion-channel gating. Therefore, with the avalanche of protein sequences generated in the post-genomic era, it is very important for both basic research and the pharmaceutical industry to acquire the possible knowledge about quaternary structural attributes of their proteins of interest. In view of this, a high throughput method (DWT\_DT), a 2-layer approach by fusing discrete wavelet transform (DWT) and decision-tree algorithm (DT) with physicochemical features, has been developed to predict protein quaternary structures. The 1st layer is to assign a query protein to one of the 10 main quaternary structural attributes. The 2nd layer is to evaluate whether the protein in question is composed of homo- or hetero-oligomers. The overall accuracy by jackknife test for the 1st layer identification was 89.60%. The overall accuracy of the 2nd layer varies from 88.23 to 100%. The results suggest that this newly developed protocol (DWT\_DT) is very promising in predicting quaternary structures with complicated composition. |
  | Date Added | 3/7/2014, 1:06:24 PM |
  | Modified | 3/7/2014, 1:06:24 PM |
- ## Identifying structural domains of proteins using clustering

  |  |  |
  | --- | --- |
  | Type | Journal Article |
  | Author | Howard J. Feldman |
  | URL | http://www.biomedcentral.com/1471-2105/13/286/ |
  | Volume | 13 |
  | Issue | 1 |
  | Pages | 286 |
  | Publication | BMC bioinformatics |
  | Date | 2012 |
  | Accessed | 9/23/2013, 10:16:05 AM |
  | Library Catalog | Google Scholar |
  | Date Added | 10/11/2013, 10:29:15 AM |
  | Modified | 3/7/2014, 12:15:00 PM |

  ### Tags:

  - ASTRAL
  - ASTRAL sequences
  - ASTRAL subsets

  ### Notes:

  - Present a clustering-based method for domain identification

    How SCOP is used:

    Used ASTRAL 30% for both training and benchmarking a domain prediction algorithm.  Also used benchmarking\_2 and 3 data sets, which only includes domains that agree in both SCOP and CATH.

    SCOP references:

    Abstract:

    The method is competitive with others, achieving 70% agreement with SCOP on a large non-redundant data set, and 80% on a set more heavily weighted in multi-domain proteins on which both SCOP and CATH agree.

    Background:

    ...

    As a result of these different paradigms, there still does not exist a precise definition for a protein domain, nor do experts always agree on the number or location of domains within a given structure. This makes it extremely difficult to come up with a fully automated algorithm, then, to assign domain boundaries. That said, the SCOP [4] and CATH [5] databases are typically used for the problem. We found that these agree only 80% of the time on number of domains however, over 75,500 chains that they have in common (SCOP 1.75 and CATH 3.4.0, data not shown)!

    Results:

    ...

    The main data set used to optimize the algorithms was the ASTRAL30 set, con- sisting of 8792 domains in 7178 non-redundant protein chains. Only 7076 of these chains actually still existed in the current Protein Data Bank (PDB) however and so comprised the training set used in this study for the CA algorithm.

    ...

    An assignment was considered correct when it agreed with SCOP, since ASTRAL is based on the SCOP domain database.

  ### Attachments

  - 1471-2105-13-286.pdf
- ## iLoops: a protein-protein interaction prediction server based on structural features

  |  |  |
  | --- | --- |
  | Type | Journal Article |
  | Author | Joan Planas-Iglesias |
  | Author | Manuel A. Marin-Lopez |
  | Author | Jaume Bonet |
  | Author | Javier Garcia-Garcia |
  | Author | Baldo Oliva |
  | Volume | 29 |
  | Issue | 18 |
  | Pages | 2360-2362 |
  | Publication | Bioinformatics |
  | ISSN | 1367-4803 |
  | Date | SEP 15 2013 |
  | Extra | WOS:000323943200021 |
  | DOI | 10.1093/bioinformatics/btt401 |
  | Abstract | Protein-protein interactions play a critical role in many biological processes. Despite that, the number of servers that provide an easy and comprehensive method to predict them is still limited. Here, we present iLoops, a web server that predicts whether a pair of proteins can interact using local structural features. The inputs of the server are as follows: (i) the sequences of the query proteins and (ii) the pairs to be tested. Structural features are assigned to the query proteins by sequence similarity. Pairs of structural features (formed by loops or domains) are classified according to their likelihood to favor or disfavor a protein-protein interaction, depending on their observation in known interacting and non-interacting pairs. The server evaluates the putative interaction using a random forest classifier. |
  | Date Added | 10/28/2013, 4:51:00 PM |
  | Modified | 10/28/2013, 4:51:00 PM |

  ### Tags:

  - Integrated SCOP data into method
  - likely ASTRAL
  - likely ASTRAL sequences

  ### Notes:

  - iLoops is a web server that predicts whether a pair of proteins can interact using local structural features.  The server takes a pair of sequences as input.  It determines structural features (loops and domains) by running BLAST against databases of loops (ArchDB) and domains (SCOP).  Then it creates a profile of structural features for each chain and uses a random forest to infer whether the two proteins have an interaction.  
      
    How SCOP is used:

    iLoops server method uses SCOP domain data and ArchDB loop data in a machine learning-based method for protein interaction prediction.

    SCOP reference:

    We use the classification of loops from ArchDB (Espadaler et al., 2004) and domains from SCOP (Andreeva et al., 2008) to define the local structural features; hence, structural features are domains or loops.

  ### Attachments

  - Bioinformatics-2013-Planas-Iglesias-2360-2.pdf
- ## Immunoglobulin domains in Escherichia coli and other enterobacteria: from pathogenesis to applications in antibody technologies

  |  |  |
  | --- | --- |
  | Type | Journal Article |
  | Author | Gustavo Bodelon |
  | Author | Carmen Palomino |
  | Author | Luis Angel Fernandez |
  | Volume | 37 |
  | Issue | 2 |
  | Pages | 204-250 |
  | Publication | Fems Microbiology Reviews |
  | ISSN | 0168-6445 |
  | Date | MAR 2013 |
  | Extra | WOS:000314750700006 |
  | DOI | 10.1111/j.1574-6976.2012.00347.x |
  | Abstract | The immunoglobulin (Ig) protein domain is widespread in nature having a well-recognized role in proteins of the immune system. In this review, we describe the proteins containing Ig-like domains in Escherichia coli and enterobacteria, reporting their structural and functional properties, protein folding, and diverse biological roles. In addition, we cover the expression of heterologous Ig domains in E.coli owing to its biotechnological application for expression and selection of antibody fragments and full-length IgG molecules. Ig-like domains in E.coli and enterobacteria are frequently found in cell surface proteins and fimbrial organelles playing important functions during host cell adhesion and invasion of pathogenic strains, being structural components of pilus and nonpilus fimbrial systems and members of the intimin/invasin family of outer membrane (OM) adhesins. Ig-like domains are also found in periplasmic chaperones and OM usher proteins assembling fimbriae, in oxidoreductases and hydrolytic enzymes, ATP-binding cassette transporters, sugar-binding and metal-resistance proteins. The folding of most E.coli Ig-like domains is assisted by periplasmic chaperones, peptidylprolyl cis/trans isomerases and disulfide bond catalysts that also participate in the folding of antibodies expressed in this bacterium. The technologies for expression and selection of recombinant antibodies in E.coli are described along with their biotechnological potential. |
  | Date Added | 2/13/2014, 4:13:17 PM |
  | Modified | 3/7/2014, 12:10:16 PM |

  ### Notes:

  - Review of research on Immunoglobulin domains.

    How SCOP is used:

    Manual search for proteins with similar structures.  List SCOP sunid for a superfamily.

    How CATH is used:

    Not using CATH data.  Just citing paper.

    SCOP reference:

    Ig-like domains in E. coli and enterobacteria

    Ig-like domains have been reported in a good number of E. coli and enterobacterial proteins, as will be reviewed in the following sections. To find known E. coli and entero- bacterial proteins bearing an Ig-like fold we performed bibliographic and bioinformatic searches to screen current protein databases. **We searched in the structural classifica- tion of proteins (SCOP, version 1.75; Andreeva** **et al., 2008) and in the conserved domain protein family (Pfam; Finn et al.****, 2008) databases.**

    **...**

    The reported structures of the polypeptides comprising the extracellular C- region of 497 amino acids of invasin (Inv497) from Yersinia pseudotuberculosis (Hamburger et al., 1999), the C-termi- nal 280 amino acids of intimin (Int280) from EPEC (Kelly et al., 1999; Luo et al., 2000) and 188 amino acids (Int188) from EHEC (Yi et al., 2010), along with struc- tural predictions of the rest of the C-region, show a rod- like structure consisting of four (invasin D1–D4) or three (intimin D0–D3) Ig-like domains (SCOP 49373) followed by a C-type lectin-like domain (invasin D5 and intimin D4) responsible for receptor-binding and located at the C-terminal tip of the molecule (Fig. 6b).

    CATH reference:

    Indeed, the number of possible stable configurations of globular proteins may be limited, and thus, a considerable number of known proteins adopt one of 10 favorable ‘superfold’ configurations (Orengo et al., 1994, 1997), one of which is the Ig fold (Steiner, 1996).

  ### Attachments

  - fmr347.pdf
- ## Implementation of a Parallel Protein Structure Alignment Service on Cloud

  |  |  |
  | --- | --- |
  | Type | Journal Article |
  | Author | Che-Lun Hung |
  | Author | Yaw-Ling Lin |
  | Pages | 439681 |
  | Publication | International Journal of Genomics |
  | ISSN | 2314-436X |
  | Date | 2013 |
  | Extra | WOS:000318272700001 |
  | DOI | 10.1155/2013/439681 |
  | Abstract | Protein structure alignment has become an important strategy by which to identify evolutionary relationships between protein sequences. Several alignment tools are currently available for online comparison of protein structures. In this paper, we propose a parallel protein structure alignment service based on the Hadoop distribution framework. This service includes a protein structure alignment algorithm, a refinement algorithm, and a MapReduce programming model. The refinement algorithm refines the result of alignment. To process vast numbers of protein structures in parallel, the alignment and refinement algorithms are implemented using MapReduce. We analyzed and compared the structure alignments produced by different methods using a dataset randomly selected from the PDB database. The experimental results verify that the proposed algorithm refines the resulting alignments more accurately than existing algorithms. Meanwhile, the computational performance of the proposed service is proportional to the number of processors used in our cloud platform. |
  | Date Added | 2/13/2014, 4:13:17 PM |
  | Modified | 3/7/2014, 12:09:34 PM |
- ## Implicit Solvation Parameters Derived from Explicit Water Forces in Large-Scale Molecular Dynamics Simulations

  |  |  |
  | --- | --- |
  | Type | Journal Article |
  | Author | Jens Kleinjung |
  | Author | Walter R. P. Scott |
  | Author | Jane R. Allison |
  | Author | Wilfred F. van Gunsteren |
  | Author | Franca Fraternali |
  | Volume | 8 |
  | Issue | 7 |
  | Pages | 2391-2403 |
  | Publication | JOURNAL OF CHEMICAL THEORY AND COMPUTATION |
  | ISSN | 1549-9618 |
  | Date | July 2012 |
  | DOI | 10.1021/ct200390j |
  | Language | English |
  | Abstract | Implicit solvation is a mean force approach to model solvent forces acting on a solute molecule. It is frequently used in molecular simulations to reduce the computational cost of solvent treatment. In the first instance, the free energy of solvation and the associated solvent-solute forces can be approximated by a function of the solvent-accessible surface area (SASA) of the solute and differentiated by an atom-specific salvation parameter sigma(SASA)(i). A procedure for the determination of values for the sigma(SASA)(i) parameters through matching of explicit and implicit solvation forces is proposed. Using the results of Molecular Dynamics simulations of 188 topologically diverse protein structures in water and in implicit solvent, values for the sigma(SASA)(i) parameters for atom types i of the standard amino acids in the GROMOS force field have been determined. A simplified representation based on groups of atom types sigma(SASA)(g) was obtained via partitioning of the atom-type sigma(SASA)(i) distributions by dynamic programming. Three groups of atom types with well separated parameter ranges were obtained, and their performance in implicit versus explicit simulations was assessed. The solvent forces are available at http://mathbio.nimr.mrc.ac.uk/wild/Solvent\_Forces. |
  | Date Added | 10/25/2013, 4:29:01 PM |
  | Modified | 10/25/2013, 4:29:01 PM |

  ### Tags:

  - ASTRAL
  - ASTRAL domain structures

  ### Notes:

  - Present a method for implicit solvation for MD simulations.

    How SCOP is used:

    Evaluate method on non-redundant dataset of domains from ASTRAL.

    SCOP reference:

    Selection of the Reference Protein Set..Using this alphabet, we translated a selected set of 2559 well-resolved protein domains of the SCOP ASTRAL40 database38 to topological strings by assigning a topological alphabet character to each supersecondary structure. The concatenated characters form a topological string that characterizes basic features of the protein fold.

    The selected SCOP set was reduced to less than 10% of its size by applying the MinSet method,39 so as to derive a database subset that was amenable to MD simulations but maximally informative in terms of topological composition.

  ### Attachments

  - ct200390j.pdf
- ## Improved Detection of Remote Homologues Using Cascade PSI-BLAST: Influence of Neighbouring Protein Families on Sequence Coverage

  |  |  |
  | --- | --- |
  | Type | Journal Article |
  | Author | Swati Kaushik |
  | Author | Eshita Mutt |
  | Author | Ajithavalli Chellappan |
  | Author | Sandhya Sankaran |
  | Author | Narayanaswamy Srinivasan |
  | Author | Ramanathan Sowdhamini |
  | URL | http://dx.plos.org/10.1371/journal.pone.0056449 |
  | Volume | 8 |
  | Issue | 2 |
  | Pages | e56449 |
  | Publication | PloS one |
  | Date | 2013 |
  | Accessed | 9/23/2013, 10:15:21 AM |
  | Library Catalog | Google Scholar |
  | Short Title | Improved Detection of Remote Homologues Using Cascade PSI-BLAST |
  | Date Added | 10/11/2013, 10:29:15 AM |
  | Modified | 10/11/2013, 10:29:15 AM |

  ### Notes:

  - Propose method called "Cascade PSI-BLAST", a "generalized strategy" for sequence-based remote homology detection using multiple query sequences.

    How SCOP is used:

    Evaluated method of 13 protein families representing every structural class in the SCOP database.

    Validated on the family, superfamily, and fold levels.

    SCOP reference:

    Implementation of Multiple Queries on other SCOP Classes

    SCOP database provides detailed information about the evolutionary relationships of known proteins on the basis of structure and function. Determining such protein relationships

    using computer algorithms is very helpful in assigning functions to hypothetical proteins and those whose structures are not yet determined. Therefore, to test the wide applicability of our findings, the above analysis was also carried out on random families chosen from all the classes of SCOP database. Some of highly populated superfamilies were selected from SCOP database and comparison of coverage of PSI-BLAST and Cascade PSI- BLAST was performed (using single query sequence) (Table S3). We found that cascaded search could cover 22%, 31% and 27% more average coverage at family, superfamily and fold level respectively, for diverse protein superfamilies, with an average precision score of 71% which is very assuring. By cascading PSI- BLAST searches on multiple protein families of unrelated folds, 36% increase in coverage at the family level was observed, along with a 43% decrease in precision score (please see Table ST3). At the superfamily level, there is an observed 58% increase in coverage and 23% decrease in precision score. At the fold level, however, there is a 65% increase in coverage by cascading the sequence searches accompanied by 26% decrease in precision score. These observations clearly reveal that there is a trade-off between coverage and precision score in applying sensitive sequence search algorithms. At higher levels of structural hierarchy, the extent of ‘loss’ in precision score is fairly minimal by the cascaded approach, suggesting that there is little chance that the specificity is lost in making connections at the fold level.

    Two highly populated folds were chosen from each class, from which ten queries of each family were selected at random to perform coverage analysis of each query sequence at family, superfamily and fold level (Table S4). We observed that with Cascade PSI-BLAST there was appreciable increase in coverage with precision score 81%. Use of multiple queries of a family was found to be promising as different queries and displayed differences in finding its own family members. Numbers of remote connections were also dependent on type of query used, as some queries picked members from other families while others did not. These results show a wide applicability of our approaches.

  ### Attachments

  - [HTML] from plos.org
  - journal.pone.0056449.pdf
- ## Improved estimates of coordinate error for molecular replacement

  |  |  |
  | --- | --- |
  | Type | Journal Article |
  | Author | Robert D. Oeffner |
  | Author | Gabor Bunkoczi |
  | Author | Airlie J. McCoy |
  | Author | Randy J. Read |
  | Volume | 69 |
  | Pages | 2209-2215 |
  | Publication | Acta Crystallographica Section D-Biological Crystallography |
  | ISSN | 0907-4449; 1399-0047 |
  | Date | NOV 2013 |
  | Extra | WOS:000326648900007 |
  | DOI | 10.1107/S0907444913023512 |
  | Abstract | The estimate of the root-mean-square deviation (r.m.s.d.) in coordinates between the model and the target is an essential parameter for calibrating likelihood functions for molecular replacement (MR). Good estimates of the r.m.s.d. lead to good estimates of the variance term in the likelihood functions, which increases signal to noise and hence success rates in the MR search. Phaser has hitherto used an estimate of the r.m.s.d. that only depends on the sequence identity between the model and target and which was not optimized for the MR likelihood functions. Variance-refinement functionality was added to Phaser to enable determination of the effective r.m.s.d. that optimized the log-likelihood gain (LLG) for a correct MR solution. Variance refinement was subsequently performed on a database of over 21000 MR problems that sampled a range of sequence identities, protein sizes and protein fold classes. Success was monitored using the translation-function Z-score (TFZ), where a TFZ of 8 or over for the top peak was found to be a reliable indicator that MR had succeeded for these cases with one molecule in the asymmetric unit. Good estimates of the r.m.s.d. are correlated with the sequence identity and the protein size. A new estimate of the r.m.s.d. that uses these two parameters in a function optimized to fit the mean of the refined variance is implemented in Phaser and improves MR outcomes. Perturbing the initial estimate of the r.m.s.d. from the mean of the distribution in steps of standard deviations of the distribution further increases MR success rates. |
  | Date Added | 2/12/2014, 2:18:08 PM |
  | Modified | 2/12/2014, 2:18:08 PM |

  ### Tags:

  - coverage

  ### Notes:

  - Present a method for rmsd estimate refinement in molecular replacement.  Classify a data set by SCOP class and then evaluate the behavior of their method on each class.

    How SCOP is used:

    Collect a data set of biological monomers from the PDB.  Classified each "target" by its SCOP class.  Excluded targets that were not in the top four SCOP classes or were not yet classified in SCOP.

    SCOP reference:

    2.1. Target structures

    2862 structures were selected from the PDB using the criteria that they were biological monomers, that they had one monomer in the asymmetric unit and that the associated X-ray data had been deposited. Twinned structures were excluded, as were structures for which the published R factor could not be reproduced.

    The number of entries in the PDB varies drastically across the range of protein sizes from very small (fewer than 50 residues) to large (more than 1000 residues). The vast majority of proteins are in the moderate-size range of between 100 and 500 residues. Targets were chosen across the range of sizes in the PDB. All PDB structures with 600 residues or more that met the selection criteria were retained, but nonetheless the relatively small number of large structures available limited the quality of the statistics for the largest proteins. The distribution of sizes used is shown in Fig. 1(a).

    Targets were chosen across the range of SCOP classes (Murzin et al., 1995). There are ten SCOP classes, of which we focused only on the four main classes: ‘all-alpha’, ‘all-beta’, ‘alpha and beta proteins’ and ‘alpha and beta proteins’. The current SCOP database, from 23 February 2009, annotates 38 221 PDB entries. This is about half of the number of PDB entries as of the commencement of this study and so a significant fraction of the target structures was uncategorized. The number of proteins belonging to the SCOP classes varies according to the number of residues in the protein (Fig. 1b). Very small proteins of 50 or fewer residues do not belong to any of the four SCOP classes under consid- eration. Proteins in the moderate-size range are uniformly distributed across the SCOP classes.

    ...

    3.5. Dependence on SCOP class

    We also investigated the dependence of the VRMS on the SCOP class. Fig. 5(b) shows the distributions of the VRMS/ eVRMS values for the four SCOP classes of moderate-sized proteins under consideration in this study. From these distri- butions we can deduce the means and standard deviations listed in Table 3.

    Proteins belonging to the ‘all-⬚⬚’ class have a VRMS that is overestimated by about 5% on average, whereas those for ‘all-⬚⬚’ proteins are underestimated by about 9% on average. This suggests that the overall folds for proteins dominated by ⬚⬚-sheets are better conserved than those composed of ⬚⬚-helices. Apart from the ‘all-⬚⬚’ class, which is more variable, the standard deviations show that the distributions separated into fold categories are slightly narrower than the total distribution that combines all fold categories. However, this analysis has not been used to further refine estimates of the VRMS based on fold class in Phaser because there is still a very large overlap among the distributions for different fold

    classes compared with the standard deviations of the distri- butions and hence it is likely that little would be gained compared with sampling the estimates of the VRMS in frac- tions of ⬚⬚(VRMS/eVRMS). At the same time, there would be much added complication in determining and passing information about the fold class to Phaser.

  ### Attachments

  - ba5212.pdf
- ## Improved method for predicting protein fold patterns with ensemble classifiers

  |  |  |
  | --- | --- |
  | Type | Journal Article |
  | Author | Weicheng Chen |
  | Author | Xiangrong Liu |
  | Author | Yong Huang |
  | Author | Yi Jiang |
  | Author | Quan Zou |
  | Author | Chen Lin |
  | URL | http://www.funpecrp.com.br/gmr/year2012/vol11-1/pdf/gmr1674.pdf |
  | Volume | 11 |
  | Issue | 1 |
  | Pages | 174–181 |
  | Publication | Genetics and Molecular Research |
  | Date | 2012 |
  | Accessed | 9/23/2013, 10:16:36 AM |
  | Library Catalog | Google Scholar |
  | Date Added | 10/11/2013, 10:29:15 AM |
  | Modified | 10/11/2013, 10:29:15 AM |

  ### Tags:

  - bioinformatics
  - Ensemble classifier
  - machine learning
  - Protein folding pattern

  ### Notes:

  - Predicting protein folding based on 2 different methods, feature extracting and ensemble classification. They compared their method two different databases and found high degree of accuracy.

    **SCOP Use:**

    Evaluated method for fold prediction on two datasets:

    1. all SCOP data from one release (got ~54% accuracy).

    2. PFP-Pred and PFP-FunDSeqE databases (311 proteins for training, 383 for testing).  Classified these into 27 SCOP folds. (got 71% accuracy).

    **SCOP References:** 

    We chose two datasets for the experiments. One was the latest data in the SCOP database (Murzin et al., 1995) and the other was the same dataset as that in the PFP-Pred and PFP-FunDSeqE databases, which contain 311 proteins for training and 383 proteins for testing. None of the proteins in the testing dataset has more than 35% sequence similarity to those in the training dataset. According to the SCOP database these proteins can be further categorized into the following 27-fold types: 1) globin-like, 2) cytochrome c, 3) DNA-bind- ing 3-helical bundle, 4) 4-helical up-and-down bundle, 5) 4-helical cytokines, 6) EF-hand, 7) immunoglobulin-like, 8) cupredoxins, 9) viral coat and capsid proteins, 10) concanavalin A- like lectin/glucanases, 11) SH3-like barrel, 12) oligonucleotide/oligosaccharide-binding-fold, 13) β-trefoil, 14) trypsin-like serine proteases, 15) lipocalins, 16) triosephosphate isomerase barrel, 17) flavin adenine dinucleotide (also nicotinamide adenine dinucleotide-binding mo- tif), 18) flavodoxin-like, 19) nicotinamide adenine dinucleotide phosphate-binding Rossmann fold, 20) P-loop, 21) thioredoxin-like, 22) ribonuclease H-like motif, 23) hydrolases, 24) peri- plasmic binding protein-like, 25) β-grasp, 26) ferredoxin-like, and 27) small inhibitors, toxins, and lectins. Of these fold types, types 1-6 belong to the α structural class, types 7-15 to the β class, types 16-24 to the α/β class, and types 25-27 to the α+β class.

    The latest data contain 1067 α structural proteins, 1034 β structural proteins, 1471 α/β proteins, and 1588 α+β proteins (data from SCOP, August 20, 2011).

  ### Attachments

  - gmr1674.pdf
- ## Incorporating Secondary Structural Features into Sequence Information for Predicting Protein Structural Class

  |  |  |
  | --- | --- |
  | Type | Journal Article |
  | Author | Bo Liao |
  | Author | Ting Peng |
  | Author | Haowen Chen |
  | Author | Yaping Lin |
  | Volume | 20 |
  | Issue | 10 |
  | Pages | 1079-1087 |
  | Publication | Protein and peptide letters |
  | ISSN | 0929-8665 |
  | Date | October 2013 |
  | Language | English |
  | Abstract | Knowledge of structural classes is applied in numerous important predictive tasks that address structural and functional features of proteins, although the prediction accuracy of the protein structural classes is not high. In this study, 45 different features were rationally designed to model the differences between protein structural classes, among which, 30 of them reflect the combined protein sequence information. In terms of correlation function, the protein sequence can be converted to a digital signal sequence, from which we can generate 20 discrete Fourier spectrum numbers. According to the segments of amino with different characteristics occurring in protein sequences, the frequencies of the 10 kinds of segments of amino acid (motifs) in protein are calculated. Other features include the secondary structural information :10 features were proposed to model the strong adjacent correlations in the secondary structural elements and capture the long-range spatial interactions between secondary structures, other 5 features were designed to differentiate alpha/beta from alpha+beta classes, which is a major problem of the existing algorithm. The methods were proposed based on a large set of low-identity sequences for which secondary structure is predicted from their sequence (based on PSI-PRED). By means of this method, the overall prediction accuracy of four benchmark datasets were all improved. Especially for the dataset FC699, 25PDB and D1189 which are 1.26%, 1% and 0.85% higher than the best previous method respectively. |
  | Date Added | 10/11/2013, 10:29:15 AM |
  | Modified | 3/7/2014, 12:09:23 PM |

  ### Tags:

  - Discrete fourier spectrum
  - long-range spatial interaction
  - motifs
  - PSI-PRED

  ### Notes:

  - Paper unavailable.
- ## Increasing Sequence Search Sensitivity with Transitive Alignments

  |  |  |
  | --- | --- |
  | Type | Journal Article |
  | Author | Ketil Malde |
  | Author | Tomasz Furmanek |
  | URL | http://dx.plos.org/10.1371/journal.pone.0054422 |
  | Volume | 8 |
  | Issue | 2 |
  | Pages | e54422 |
  | Publication | PloS one |
  | Date | 2013 |
  | Accessed | 9/23/2013, 10:18:03 AM |
  | Library Catalog | Google Scholar |
  | Date Added | 10/11/2013, 10:29:15 AM |
  | Modified | 10/11/2013, 10:29:15 AM |

  ### Notes:

  - Present a method for more sensitive sequence search and alignments in small, curated, databases by leveraging larger databases.

    How SCOP is used:

    Used ASTRAL 40% representative subset of sequences to evaluate their alignment method.  Validated on remote homology detection at the superfamily and fold levels.

    SCOP reference:

    We downloaded SCOP 40, version 1.75A. This contains 11 211 proteins from SCOP with at most 40% identity, classified into SCOP’s categories of class, fold, superfamily, and family. We used BLASTP to align this set of proteins against itself, and we also generated transitive alignments by using BLASTP to match the SCOP proteins to UniRef 50, and BLASTP alignments from UniRef 50 to SCOP 40, in both cases with an E-value threshold of 0.1. The results were sorted according to alignment score, and classified as true positives if the query and target had the same classification, false positives if they had different classification. The resulting ROC curves for the SCOP superfamily and fold levels are shown in Figure 2.

  ### Attachments

  - journal.pone.0054422.pdf
- ## IndelFR: a database of indels in protein structures and their flanking regions

  |  |  |
  | --- | --- |
  | Type | Journal Article |
  | Author | Zheng Zhang |
  | Author | Cheng Xing |
  | Author | Lushan Wang |
  | Author | Bin Gong |
  | Author | Hui Liu |
  | Volume | 40 |
  | Issue | D1 |
  | Pages | D512-D518 |
  | Publication | Nucleic Acids Research |
  | ISSN | 0305-1048 |
  | Date | January 2012 |
  | DOI | 10.1093/nar/gkr1107 |
  | Language | English |
  | Abstract | Insertion/deletion (indel) is one of the most common methods of protein sequence variation. Recent studies showed that indels could affect their flanking regions and they are important for protein function and evolution. Here, we describe the Indel Flanking Region Database (IndelFR, http://indel.bioinfo.sdu.edu.cn), which provides sequence and structure information about indels and their flanking regions in known protein domains. The indels were obtained through the pairwise alignment of homologous structures in SCOP superfamilies. The IndelFR database contains 2 925 017 indels with flanking regions extracted from 373 402 structural alignment pairs of 12 573 non-redundant domains from 1053 superfamilies. IndelFR provides access to information about indels and their flanking regions, including amino acid sequences, lengths, locations, secondary structure constitutions, hydrophilicity/hydrophobicity, domain information, 3D structures and so on. IndelFR has already been used for molecular evolution studies and may help to promote future functional studies of indels and their flanking regions. |
  | Date Added | 10/25/2013, 4:17:08 PM |
  | Modified | 11/12/2013, 4:28:12 PM |

  ### Tags:

  - ASTRAL
  - ASTRAL domain structures
  - ASTRAL subsets
  - Cite ASTRAL

  ### Notes:

  - IndelFR: Indel Flanking Region Database, provides sequence and structure information about Insertion/Deletion (indels) and their flanking regions in 'known' protein domains.

    How SCOP is used:

    Indels were obtained through pairwise structure alignment in SCOP superfamilies.  Used SCOP 1.73 and Astral 95% representative subset.  Website tree interface permits browsing through SCOP tree, and augments domain data with indel data.

    SCOP references:

    Under Abstract:  
      
    The indels were obtained through the pairwise align- ment of homologous structures in SCOP superfamilies.   
      
    Under Introduction:  
      
    The classification of homologous domains in IndelFR is based on SCOP superfamilies (28). Structure files were obtained from the ASTRAL95 non-redundant structural database (29).  
      
    Under data collection and database construction:  
    the data about superfamilies were obtained from the structural classification database SCOP 1.73 (28). The IndelFR database contains all of the superfamilies that have two or more non-redundant struc- tures in the first five SCOP classes. The five SCOP classes include: all alpha proteins, all beta proteins, alpha and beta proteins (a/b), alpha and beta proteins (a+b) and multi-domain proteins. Due to its enormous size, the immunoglobulin superfamily is temporarily excluded from the current version of the IndelFR database. The data regarding non-redundant protein domains in each superfamily was obtained from the ASTRAL95 database, in which the percent sequence identity between any two structures is always <95% (29).  
      
    We introduced the superfamily-match-indel relationship, and included this into the tree-graph catalog according to SCOP classification.  
      
    Under user interface design: browse  
      
    In the IndelFR database, indels with flanking regions are classified according to their SCOP superfamilies. In the ‘SCOP Tree’ interface, users can explore any of the superfamilies through the entire SCOP Tree or five subtrees corresponding to the five structure classes (Figure 2A).   
      
    Under Search:  
      
    In addition to browsing indels and matches in super- families through the ‘SCOP Tree’ interface, users are allowed to retrieve indels and matches.  
      
    Under Future Directions:  
      
    In the future, we plan to provide conserved indels within one SCOP superfamily by multiple sequence alignment based on structure. However, one superfamily may con- tain many distant-related homologous proteins, so they may be quite different from each other in both sequence and structure. Consequently, current algorithms for multiple sequence alignment based on structure may not be able to conduct multiple sequence alignment in every superfamily.  
    In the future, we will keep updating our database fol- lowing SCOP updates. In order to facilitate users, we are considering adding Sequence BLAST Search in the future and enabling Indel Fuzzy Search that will allow users to search for target indels from millions of indels in the IndelFR database.   
      
    Under Figure 2 legend:  
      
    Samples of the IndelFR interfaces. (A) The ‘SCOP Tree’ interface. All indels and matches in the superfamilies can be browsed through this interface.

  ### Attachments

  - Nucl. Acids Res.-2012-Zhang-D512-8.pdf
- ## Inferences from structural comparison: flexibility, secondary structure wobble and sequence alignment optimization

  |  |  |
  | --- | --- |
  | Type | Journal Article |
  | Author | Gaihua Zhang |
  | Author | Zhen Su |
  | Volume | 13 |
  | Pages | S12 |
  | Publication | Bmc Bioinformatics |
  | ISSN | 1471-2105 |
  | Date | SEP 11 2012 |
  | Extra | WOS:000309358800012 |
  | DOI | 10.1186/1471-2105-13-S15-S12 |
  | Abstract | Background: Work on protein structure prediction is very useful in biological research. To evaluate their accuracy, experimental protein structures or their derived data are used as the 'gold standard'. However, as proteins are dynamic molecular machines with structural flexibility such a standard may be unreliable. Results: To investigate the influence of the structure flexibility, we analysed 3,652 protein structures of 137 unique sequences from 24 protein families. The results showed that (1) the three-dimensional (3D) protein structures were not rigid: the root-mean-square deviation (RMSD) of the backbone C-alpha of structures with identical sequences was relatively large, with the average of the maximum RMSD from each of the 137 sequences being 1.06 angstrom; (2) the derived data of the 3D structure was not constant, e. g. the highest ratio of the secondary structure wobble site was 60.69%, with the sequence alignments from structural comparisons of two proteins in the same family sometimes being completely different. Conclusion: Proteins may have several stable conformations and the data derived from resolved structures as a 'gold standard' should be optimized before being utilized as criteria to evaluate the prediction methods, e. g. sequence alignment from structural comparison. Helix/beta-sheet transition exists in normal free proteins. The coil ratio of the 3D structure could affect its resolution as determined by X-ray crystallography. |
  | Date Added | 2/20/2014, 12:24:01 PM |
  | Modified | 2/20/2014, 12:24:01 PM |

  ### Notes:

  - Computational study of protein flexibility.

    How SCOP is used:

    Annotate data set curated with CD-Hit with SCOP classification (fold)

    SCOP reference:

    Materials and methods

    Data collection

    CD-HIT [23] was utilized for clustering the protein sequences from the PDB database [18], the sequence identity threshold used was 0.99 as we tried to analyse the structures with few mutations, because these mutated sites are in or around the functional important region that have often been altered by researchers in mechan- isms studies. HMMER3 was utilized to categorize the protein family with an E-value cut-off of 0.0001 [24]. The structures were selected using the following rules:

    1. The sequential structures were determined by X-ray crystallography with resolution < 3.5Å;

    2. There were > 4 structures for each identical sequence;

    3. In each protein family, there were at least three unique proteins.

    In general, structures with resolution < 2.5 Å are con- sidered reliable. However, analysis of structures with low resolution may supply some interesting information about protein flexibility. In the present study, 1,956 PDB entries were collected, with 1,588 having resolution < 2.5 Å (Additional file 1: Figure S1 and Additional file 2).

    Structures with identical sequences were defined as a ‘structural group’. We obtained 3,652 structures from 137 unique sequences and distributed in 24 protein families; and 62 structural groups contained mutations. The detailed protein families can be seen in Additional file 3; the PDB entries and mutation sites are shown in Additional file 4. **The structural folding types were annotated by the SCOP 1.75 database [25] and shown in Additional file 5.** The functional divisions are shown in Additional file 6. The dataset includes free proteins, pro- tein-ligand complexes and protein-protein complexes.

  ### Attachments

  - 1471-2105-13-S15-S12.pdf
- ## Inherent Relationships among Different Biophysical Prediction Methods for Intrinsically Disordered Proteins

  |  |  |
  | --- | --- |
  | Type | Journal Article |
  | Author | Fan Jin |
  | Author | Zhirong Liu |
  | URL | http://www.sciencedirect.com/science/article/pii/S0006349512051247 |
  | Volume | 104 |
  | Issue | 2 |
  | Pages | 488–495 |
  | Publication | Biophysical journal |
  | Date | 2013 |
  | Accessed | 9/23/2013, 10:14:50 AM |
  | Library Catalog | Google Scholar |
  | Date Added | 10/11/2013, 10:29:15 AM |
  | Modified | 10/11/2013, 10:29:15 AM |

  ### Notes:

  - Evaluation of different biophysical prediction methods for instrinsically disordered proteins

    How SCOP is used:

    Use SCOP domains from the first four classes, filtered to have at most 30% sequence identity, to train method for disorder prediction.  DisProt data were used for positive examples and SCOP domains for negative (ordered) examples.

    SCOP references:

    Experimental SCOP dataset

    The experimental dataset of ordered proteins was obtained from the SCOP database (Ver. 1.75, June 2009) (41). Four SCOP classes (all a; all b; aþb; a/b) were considered and redundant sequences with >30% sequence iden- tity were removed. The final SCOP dataset contained 2005 proteins.

    Proteins in DisProt with sequences longer than 100 residues and disordered ratio larger than 0.5 were used as the positive set (disordered set), while the SCOP dataset was adopted as the negative set (ordered set). To make order/disorder prediction using a specified residue property, we calculated the average of the property over the sequence residues (without consid- ering factors such as window averaging) for every protein and used it as the order/disorder indicator.

  ### Attachments

  - 1-s2.0-S0006349512051247-main.pdf
- ## Insights into polypharmacology from drug-domain associations

  |  |  |
  | --- | --- |
  | Type | Journal Article |
  | Author | Aurelio A. Moya-Garcia |
  | Author | Juan A. G. Ranea |
  | Volume | 29 |
  | Issue | 16 |
  | Pages | 1934-1937 |
  | Publication | Bioinformatics |
  | ISSN | 1367-4803 |
  | Date | AUG 15 2013 |
  | Extra | WOS:000322337400002 |
  | DOI | 10.1093/bioinformatics/btt321 |
  | Abstract | Motivation: Polypharmacology (the ability of a single drug to affect multiple targets) is a key feature that may explain part of the decreasing success of conventional drug discovery strategies driven by the quest for drugs to act selectively on a single target. Most drug targets are proteins that are composed of domains (their structural and functional building blocks). Results: In this work, we model drug-domain networks to explore the role of protein domains as drug targets and to explain drug polypharmacology in terms of the interactions between drugs and protein domains. We find that drugs are organized around a privileged set of druggable domains. Conclusions: Protein domains are a good proxy for drug targets, and drug polypharmacology emerges as a consequence of the multidomain composition of proteins. |
  | Date Added | 2/13/2014, 4:13:17 PM |
  | Modified | 3/7/2014, 12:09:10 PM |
- ## Insights into the evolution of sorbitol metabolism: phylogenetic analysis of SDR196C family

  |  |  |
  | --- | --- |
  | Type | Journal Article |
  | Author | Agustin Sola Carvajal |
  | Author | Maria Inmaculada Garcia Garcia |
  | Author | Francisco Garcia Carmona |
  | Author | Alvaro Sanchez Ferrer |
  | Volume | 12 |
  | Pages | 147 |
  | Publication | Bmc Evolutionary Biology |
  | Date | August 2012 |
  | DOI | 10.1186/1471-2148-12-147 |
  | Abstract | Background: Short chain dehydrogenases/reductases (SDR) are NAD(P)(H)-dependent oxidoreductases with a highly conserved 3D structure and of an early origin, which has allowed them to diverge into several families and enzymatic activities. The SDR196C family (http://www.sdr-enzymes.org) groups bacterial sorbitol dehydrogenases (SDH), which are of great industrial interest. In this study, we examine the phylogenetic relationship between the members of this family, and based on the findings and some sequence conserved blocks, a new and a more accurate classification is proposed. Results: The distribution of the 66 bacterial SDH species analyzed was limited to Gram-negative bacteria. Six different bacterial families were found, encompassing alpha-, beta- and gamma-proteobacteria. This broad distribution in terms of bacteria and niches agrees with that of SDR, which are found in all forms of life. A cluster analysis of sorbitol dehydrogenase revealed different types of gene organization, although with a common pattern in which the SDH gene is surrounded by sugar ABC transporter proteins, another SDR, a kinase, and several gene regulators. According to the obtained trees, six different lineages and three sublineages can be discerned. The phylogenetic analysis also suggested two different origins for SDH in beta-proteobacteria and four origins for gamma-proteobacteria. Finally, this subdivision was further confirmed by the differences observed in the sequence of the conserved blocks described for SDR and some specific blocks of SDH, and by a functional divergence analysis, which made it possible to establish new consensus sequences and specific fingerprints for the lineages and sub lineages. Conclusion: SDH distribution agrees with that observed for SDR, indicating the importance of the polyol metabolism, as an alternative source of carbon and energy. The phylogenetic analysis pointed to six clearly defined lineages and three sub lineages, and great variability in the origin of this gene, despite its well conserved 3D structure. This suggests that SDH are very old and emerged early during the evolution. This study also opens up a new and more accurate classification of SDR196C family, introducing two numbers at the end of the family name, which indicate the lineage and the sublineage of each member, i.e, SDR196C6.3. |
  | Date Added | 3/7/2014, 12:08:00 PM |
  | Modified | 3/7/2014, 12:08:00 PM |
- ## Insights into the Fold Organization of TIM Barrel from Interaction Energy Based Structure Networks

  |  |  |
  | --- | --- |
  | Type | Journal Article |
  | Author | M. S. Vijayabaskar |
  | Author | Saraswathi Vishveshwara |
  | URL | http://dx.plos.org/10.1371/journal.pcbi.1002505 |
  | Volume | 8 |
  | Issue | 5 |
  | Pages | e1002505 |
  | Publication | PLoS computational biology |
  | Date | 2012 |
  | Accessed | 9/20/2013, 1:18:37 PM |
  | Library Catalog | Google Scholar |
  | Date Added | 10/11/2013, 10:29:15 AM |
  | Modified | 2/24/2014, 11:48:45 AM |

  ### Tags:

  - ASTRAL
  - ASTRAL domain structures
  - Cite ASTRAL

  ### Notes:

  - Study of the contributions of noncovalent interactions to stabilizing TIM barrel folds, using Protein Energy Networks (PENs).  Have also developed a novel network-based phylogenetic analysis, and claim that it agrees better with the SCOP classification than sequence-based phylogeny.

    How SCOP is used:

    Derived a dataset from the TIM fold from SCOP.   Used ASTRAL structure data, but not for representative sets.  Sort domains by family, then then use cd-hit program to limit to 30% sequence identity.  Resulted in 19 families with 81 domains.

    SCOP references:

    "Despite high sequence diversity we find common patterns of interactions of equivalent energies emerged when investigated at the family level. The family level classification of the TIM fold was obtained from the SCOP database [39]."

    "It can be readily seen that the interaction conservation based method clusters proteins of the same family under the same clade better than the sequence conservation based method. It should be noted that the SCOP classification of families is based on sequence or structure or functional similarities. The interaction based phylogeny matches very well with the SCOP classification than the sequence based method for the same dataset."

    "The dataset used in this analysis is composed of domains from the TIM fold given by Structural Classification Of Proteins (SCOP) [39]. The coordinates for the domains are obtained from ASTRAL [45]. The domains are sorted into their respective families as given in SCOP. The sequence identity within the members of each family is less than 30%."

  ### Attachments

  - [HTML] from plos.org
  - journal.pcbi.1002505.pdf
  - PubMed entry
- ## In-silico prediction of an uncharacterized protein generated from heat responsive SSH library in wheat (Triticum aestivum L.)

  |  |  |
  | --- | --- |
  | Type | Journal Article |
  | Author | Jasdeep Chatrath Padaria |
  | Author | Deepesh Bhatt Koushik Biswas |
  | Author | Gagandeep Singh |
  | Author | Rajkumar Raipuria |
  | Volume | 6 |
  | Issue | 2 |
  | Pages | 150-156 |
  | Publication | Plant Omics |
  | ISSN | 1836-0661 |
  | Date | MAR 2013 |
  | Extra | WOS:000317716300009 |
  | Abstract | Wheat is exposed to various abiotic stresses at different stages of its life cycle leading to severe decline in productivity. With rapid climate changes, high temperature stress is a major limitation to wheat production. Certain cultivars of wheat display a tolerant response to heat stress. Studies on differential expression in response to heat stress leads to identification of genes involved in molecular mechanism of thermo tolerance. Large-scale differential display analysis generates a large number of transcripts, of which a few are stress responsive whereas, many are of unknown or uncharacterized functional identity. The present study was done to identify a transcript of uncharacterized function obtained from heat responsive subtractive library generated from anthesis stage of thermo-tolerant wheat cv. Raj3765. Real time PCR analysis showed a four-fold increase in expression of the identified transcript at a stress of 37 C at the anthesis stage, indicating its role in facilitating the plant to cope the deleterious effects of high temperature at anthesis stage. Protparam tool analysis revealed that the leucine (Leu) is dominant amino acid present in the sequence, involving 15.5% of total amino acids. In-silico analysis revealed the existence of conserved domain region similar to leucine rich repeat (LRR) motif, an important DNA-binding domain. The presence of LRR motif in the protein predicted from the transcript under study indicates that this protein has a role as a signaling molecule involved in stress responses. Functional validation of the identified transcript in a model plant system shall confirm its role in heat stress tolerance. |
  | Date Added | 2/13/2014, 4:13:17 PM |
  | Modified | 3/7/2014, 12:09:05 PM |
- ## In silico studies of Echinococcus granulosus FABPs

  |  |  |
  | --- | --- |
  | Type | Journal Article |
  | Author | Adriana Esteves |
  | Author | Margot Paulino Zunini |
  | URL | http://www.tandfonline.com/doi/abs/10.1080/07391102.2012.698246 |
  | Volume | 31 |
  | Issue | 2 |
  | Pages | 224–239 |
  | Publication | Journal of Biomolecular Structure and Dynamics |
  | Date | 2013 |
  | Accessed | 9/23/2013, 10:20:20 AM |
  | Library Catalog | Google Scholar |
  | Date Added | 10/11/2013, 10:29:15 AM |
  | Modified | 3/7/2014, 12:09:52 PM |

  ### Tags:

  - docking
  - FABPs
  - fatty acids
  - molecular dynamic simulation
  - platyhelminthes

  ### Notes:

  - Study two fatty acid binding proteins using homology modeling and molecular dynamics.

    How SCOP/CATH is used:

    look up superfamily classification of fatty acid binding proteins (FABPs).

    SCOP/CATH reference:

    These proteins are members of the calycin, lipocalin or FABP superfamilies, according to the criteria considered for clas- sification (Murzin, Brenner, Hubbard, & Chothia, 1995; Orengo, Michie, Jones, Swindells, & Thornton, 1997; Ono & Odani, 2010; Sigrist, Ono, et al., 2010).

  ### Attachments

  - 07391102%2E2012%2E698246.pdf
- ## In silico study on the effect of surface lysines and arginines on the electrostatic interactions and protein stability

  |  |  |
  | --- | --- |
  | Type | Journal Article |
  | Author | Sriram Sokalingam |
  | Author | Bharat Madan |
  | Author | Govindan Raghunathan |
  | Author | Sun-Gu Lee |
  | URL | http://link.springer.com/article/10.1007/s12257-012-0516-1 |
  | Volume | 18 |
  | Issue | 1 |
  | Pages | 18–26 |
  | Publication | Biotechnology and Bioprocess Engineering |
  | Date | 2013 |
  | Accessed | 9/23/2013, 10:16:05 AM |
  | Library Catalog | Google Scholar |
  | Date Added | 10/11/2013, 10:29:15 AM |
  | Modified | 10/11/2013, 10:29:15 AM |

  ### Tags:

  - arginine
  - charged amino acids
  - electrostatic interactions
  - hydrogen bonds
  - lysine
  - molecular dynamics simulations
  - protein stability
  - salt-bridge

  ### Notes:

  - Computational study of the effect of surface lysines and arginines on electrostatic interactions and protein stability.

    How SCOP is used:

    Retrieve fold classification of 10 proteins in their dataset.  Shows that each entry has a different fold.

    SCOP reference:

    In this study, ten commercially important mesophilic proteins with known structures were chosen for in silico structural analysis. Table 1 lists the organism, protein name, size, fold classification by SCOP database [21], PDB ID [22] and total number of arginines and lysines of the model proteins analyzed.

  ### Attachments

  - art%3A10.1007%2Fs12257-012-0516-1.pdf
- ## Integrating Structure to Protein-Protein Interaction Networks That Drive Metastasis to Brain and Lung in Breast Cancer

  |  |  |
  | --- | --- |
  | Type | Journal Article |
  | Author | H. Billur Engin |
  | Author | Emre Guney |
  | Author | Ozlem Keskin |
  | Author | Baldo Oliva |
  | Author | Attila Gursoy |
  | Volume | 8 |
  | Issue | 11 |
  | Pages | UNSP e81035 |
  | Publication | Plos One |
  | Date | November 2013 |
  | DOI | 10.1371/journal.pone.0081035 |
  | Abstract | Blocking specific protein interactions can lead to human diseases. Accordingly, protein interactions and the structural knowledge on interacting surfaces of proteins (interfaces) have an important role in predicting the genotype-phenotype relationship. We have built the phenotype specific sub-networks of protein-protein interactions (PPIs) involving the relevant genes responsible for lung and brain metastasis from primary tumor in breast cancer. First, we selected the PPIs most relevant to metastasis causing genes (seed genes), by using the "guilt-by-association" principle. Then, we modeled structures of the interactions whose complex forms are not available in Protein Databank (PDB). Finally, we mapped mutations to interface structures (real and modeled), in order to spot the interactions that might be manipulated by these mutations. Functional analyses performed on these sub-networks revealed the potential relationship between immune system-infectious diseases and lung metastasis progression, but this connection was not observed significantly in the brain metastasis. Besides, structural analyses showed that some PPI interfaces in both metastasis sub-networks are originating from microbial proteins, which in turn were mostly related with cell adhesion. Cell adhesion is a key mechanism in metastasis, therefore these PPIs may be involved in similar molecular pathways that are shared by infectious disease and metastasis. Finally, by mapping the mutations and amino acid variations on the interface regions of the proteins in the metastasis sub-networks we found evidence for some mutations to be involved in the mechanisms differentiating the type of the metastasis. |
  | Date Added | 3/7/2014, 12:08:00 PM |
  | Modified | 3/7/2014, 12:08:00 PM |
- ## Integration of related sequences with protein three-dimensional structural families in an updated version of PALI database

  |  |  |
  | --- | --- |
  | Type | Journal Article |
  | Author | V. S. Gowri |
  | Author | Shashi B. Pandit |
  | Author | P. S. Karthik |
  | Author | N. Srinivasan |
  | Author | S. Balaji |
  | Volume | 31 |
  | Issue | 1 |
  | Pages | 486-488 |
  | Publication | Nucleic Acids Research |
  | ISSN | 1362-4962 |
  | Date | Jan 1, 2003 |
  | Extra | PMID: 12520058 PMCID: PMC165510 |
  | Journal Abbr | Nucleic Acids Res. |
  | Library Catalog | NCBI PubMed |
  | Language | eng |
  | Abstract | The database of Phylogeny and ALIgnment of homologous protein structures (PALI) contains three-dimensional (3-D) structure-dependent sequence alignments as well as structure-based phylogenetic trees of protein domains in various families. The latest updated version (Release 2.1) comprises of 844 families of homologous proteins involving 3863 protein domain structures with each of these families having at least two members. Each member in a family has been structurally aligned with every other member in the same family using two proteins at a time. In addition, an alignment of multiple structures has also been performed using all the members in a family. Every family with at least three members is associated with two dendrograms, one based on a structural dissimilarity metric and the other based on similarity of topologically equivalenced residues for every pairwise alignment. Apart from these multi-member families, there are 817 single member families in the updated version of PALI. A new feature in the current release of PALI is the integration, with 3-D structural families, of sequences of homologues from the sequence databases. Alignments between homologous proteins of known 3-D structure and those without an experimentally derived structure are also provided for every family in the enhanced version of PALI. The database with several web interfaced utilities can be accessed at: http://pauling.mbu.iisc.ernet.in/~pali. |
  | Date Added | 11/3/2014, 3:37:54 PM |
  | Modified | 11/3/2014, 3:37:54 PM |

  ### Tags:

  - Animals
  - Databases, Protein
  - Phylogeny
  - Proteins
  - Protein Structure, Tertiary
  - Sequence Alignment
  - Structural Homology, Protein
  - User-Computer Interface

  ### Attachments

  - PubMed entry
- ## Interaction between shrimp and white spot syndrome virus through PmRab7-VP28 complex: an insight using simulation and docking studies

  |  |  |
  | --- | --- |
  | Type | Journal Article |
  | Author | Arunima Kumar Verma |
  | Author | Shipra Gupta |
  | Author | Sharad Verma |
  | Author | Abha Mishra |
  | Author | N. S. Nagpure |
  | Author | Shivesh Pratap Singh |
  | Author | Ajey Kumar Pathak |
  | Author | Uttam Kumar Sarkar |
  | Author | Shri Prakash Singh |
  | Author | Mahender Singh |
  | Author | Prahlad Kishore Seth |
  | Volume | 19 |
  | Issue | 3 |
  | Pages | 1285-1294 |
  | Publication | Journal of Molecular Modeling |
  | ISSN | 1610-2940 |
  | Date | MAR 2013 |
  | Extra | WOS:000315349800030 |
  | DOI | 10.1007/s00894-012-1672-0 |
  | Abstract | White spot disease is a devastating disease of shrimp Penaeus monodon in which the shrimp receptor protein PmRab7 interacts with viral envelop protein VP28 to form PmRab7-VP28 complex, which causes initiation of the disease. The molecular mechanism implicated in the disease, the dynamic behavior of proteins as well as interaction between both the biological counterparts that crafts a micro-environment feasible for entry of virus into the shrimp is still unknown. In the present study, we applied molecular modeling (MM), molecular dynamics (MD) and docking to compute surface mapping of infective amino acid residues between interacting proteins. Our result showed that alpha-helix of PmRab7 (encompassing Ser74, Ile143, Thr184, Arg53, Asn144, Thr184, Arg53, Arg79) interacts with beta-sheets of VP28 (containing Ser74, Ile143, Thr184, Arg53, Asn144, Thr184, Arg53, Arg79) and Arg69-Ser74, Val75-Ile143, Leu73-Ile143, Arg79-Asn144, Ala198-Ala182 bonds contributed in the formation of PmRab7-VP28 complex. Further studies on the amino acid residues and bonds may open new possibilities for preventing PmRab7-VP28 complex formation, thus reducing chances of WSD. The quantitative predictions provide a scope for experimental testing in future as well as endow with a straightforward evidence to comprehend cellular mechanisms underlying the disease. |
  | Date Added | 2/13/2014, 4:13:17 PM |
  | Modified | 3/7/2014, 12:08:39 PM |
- ## Interaction signatures stabilizing the NAD(P)-binding Rossmann fold: a structure network approach

  |  |  |
  | --- | --- |
  | Type | Journal Article |
  | Author | Moitrayee Bhattacharyya |
  | Author | Roopali Upadhyay |
  | Author | Saraswathi Vishveshwara |
  | Volume | 7 |
  | Issue | 12 |
  | Pages | e51676 |
  | Publication | PloS one |
  | ISSN | 1932-6203 |
  | Date | 2012 |
  | Extra | PMID: 23284738 |
  | Journal Abbr | PLoS ONE |
  | DOI | 10.1371/journal.pone.0051676 |
  | Library Catalog | NCBI PubMed |
  | Language | eng |
  | Abstract | The fidelity of the folding pathways being encoded in the amino acid sequence is met with challenge in instances where proteins with no sequence homology, performing different functions and no apparent evolutionary linkage, adopt a similar fold. The problem stated otherwise is that a limited fold space is available to a repertoire of diverse sequences. The key question is what factors lead to the formation of a fold from diverse sequences. Here, with the NAD(P)-binding Rossmann fold domains as a case study and using the concepts of network theory, we have unveiled the consensus structural features that drive the formation of this fold. We have proposed a graph theoretic formalism to capture the structural details in terms of the conserved atomic interactions in global milieu, and hence extract the essential topological features from diverse sequences. A unified mathematical representation of the different structures together with a judicious concoction of several network parameters enabled us to probe into the structural features driving the adoption of the NAD(P)-binding Rossmann fold. The atomic interactions at key positions seem to be better conserved in proteins, as compared to the residues participating in these interactions. We propose a "spatial motif" and several "fold specific hot spots" that form the signature structural blueprints of the NAD(P)-binding Rossmann fold domain. Excellent agreement of our data with previous experimental and theoretical studies validates the robustness and validity of the approach. Additionally, comparison of our results with statistical coupling analysis (SCA) provides further support. The methodology proposed here is general and can be applied to similar problems of interest. |
  | Short Title | Interaction signatures stabilizing the NAD(P)-binding Rossmann fold |
  | Date Added | 10/11/2013, 10:29:15 AM |
  | Modified | 3/7/2014, 12:15:25 PM |

  ### Tags:

  - Binding Sites
  - Hydrogen Bonding
  - Models, Molecular
  - Models, Theoretical
  - NADP
  - Protein Folding
  - Proteins
  - Protein Structure, Secondary

  ### Notes:

  - Computational study to determine the "consensus structural features" that drive formation of the NAD(P)-binding Rossman fold/superfamily.  The aim is to determine the key factors that lead to the formation of a particular fold from diverse sequences.

    How SCOP is used:

    Curate data set of 8 families from the NAD(P)-binding Rossman fold/superfamily.  Use the Pisces server to cull the data set to only the high-resolution structures.  Perform structural alignments and build graph representations of the structures in order to find conserved structural interactions, as opposed to just sequence conservation.

    SCOP reference:

    Methods

    Creation of the Dataset

    The NAD(P)-binding Rossmann fold domains (SCOP\_id: 51734 (fold) and 51735 (superfamily)), as classified in Structural Classification of Proteins [35], contains 12 families. The coordi- nates of the NAD(P)-binding Rossmann fold domains for all these structures are obtained from ASTRAL in the PDB format [36]. Culling of this full dataset is done using the Pisces server (http:// dunbrack.fccc.edu/PISCES.php) [37] to obtain high resolution (,2A ̊ ) structures with good R-factor values (,0.25). We further chose only those families which had at least more than three structures available after the culling of the dataset. This finally produced 84 high resolution structures from 8 different families hosting the NAD(P)-binding Rossmann fold domain (summarized in Table 1 and Table S1 in Supporting Information S2) with an average sequence identity of 38.8% (std dev = 20.85; summarized in Table S7 in Supporting Information S2and Fig. S5 in Supporting Information S1).

  ### Attachments

  - journal.pone.0051676.pdf
- ## Inter-domain movements in polyketide synthases: a molecular dynamics study

  |  |  |
  | --- | --- |
  | Type | Journal Article |
  | Author | Swadha Anand |
  | Author | Debasisa Mohanty |
  | Volume | 8 |
  | Issue | 4 |
  | Pages | 1157-1171 |
  | Publication | MOLECULAR BIOSYSTEMS |
  | ISSN | 1742-206X |
  | Date | 2012 |
  | DOI | 10.1039/c2mb05425f |
  | Language | English |
  | Abstract | Insights into the structure and dynamics of modular polyketide synthases (PKS) are essential for understanding the mechanistic details of the biosynthesis of a large number of pharmaceutically important secondary metabolites. The crystal structures of the KS-AT di-domain from erythromycin synthase have revealed the relative orientation of various catalytic domains in a minimal PKS module. However, the relatively large distance between catalytic centers of KS and AT domains in the static structure has posed certain intriguing questions regarding mechanistic details of substrate transfer during polyketide biosynthesis. In order to investigate the role of inter-domain movements in substrate channeling, we have carried out a series of explicit solvent MD simulations for time periods ranging from 10 to 15 ns on the KS-AT di-domain and its sub-fragments. Analyses of these MD trajectories have revealed that both the catalytic domains and the structured inter-domain linker region remain close to their starting structures. Inter-domain movements at KS-linker and linker-AT interfaces occur around hinge regions which connect the structured linker region to the catalytic domains. The KS-linker interface was found to be more flexible compared to the linker-AT interface. However, inter-domain movements observed during the timescale of our simulations do not significantly reduce the distance between catalytic centers of KS and AT domains for facilitating substrate channeling. Based on these studies and prediction of intrinsic disorder we propose that the intrinsically unstructured linker stretch preceding the ACP domain might be facilitating movement of ACP domains to various catalytic centers. |
  | Date Added | 10/25/2013, 4:29:01 PM |
  | Modified | 10/25/2013, 4:29:01 PM |

  ### Notes:

  - Computational study, using molecular dynamics, of polyketide synthases.

    How SCOP is used:

    Study uses Jpred for secondary structure prediction in linker region.  Mentions that Jpred used SCOP/ASTRAL data from 2000..

    SCOP reference:

    JPred44 uses a HMM profile based on a non-redundant dataset from the Astral compendium of SCOP domain data45 which was taken in 2000.

  ### Attachments

  - c2mb05425f.pdf
- ## InterEvol database: exploring the structure and evolution of protein complex interfaces

  |  |  |
  | --- | --- |
  | Type | Journal Article |
  | Author | Guilhem Faure |
  | Author | Jessica Andreani |
  | Author | Raphael Guerois |
  | Volume | 40 |
  | Issue | D1 |
  | Pages | D847-D856 |
  | Publication | Nucleic Acids Research |
  | ISSN | 0305-1048 |
  | Date | JAN 2012 |
  | Extra | WOS:000298601300127 |
  | DOI | 10.1093/nar/gkr845 |
  | Abstract | Capturing how the structures of interacting partners evolved at their binding interfaces is a fundamental issue for understanding interactomes evolution. In that scope, the InterEvol database was designed for exploring 3D structures of homologous interfaces of protein complexes. For every chain forming a complex in the protein data bank (PDB), close and remote structural interologs were identified providing essential snapshots for studying interfaces evolution. The database provides tools to retrieve and visualize these structures. In addition, pre-computed multiple sequence alignments of most likely interologs retrieved from a wide range of species can be downloaded to enrich the analysis. The database can be queried either directly by pdb code or keyword but also from the sequence of one or two partners. Interologs multiple sequence alignments can also be recomputed online with tailored parameters using the InterEvolAlign facility. Last, an InterEvol PyMol plugin was developed to improve interactive exploration of structures versus sequence alignments at the interfaces of complexes. Based on a series of automatic methods to extract structural and sequence data, the database will be monthly updated. Structures coordinates and sequence alignments can be queried and downloaded from the InterEvol web interface at http://biodev.cea.fr/interevol/. |
  | Date Added | 10/28/2013, 4:53:08 PM |
  | Modified | 3/7/2014, 12:15:01 PM |

  ### Notes:

  - An interolog is a conserved reaction between two binding partners.  The InterEvol database contains close and remote structural interologs for every chain forming a complex in the PDB.

    How SCOP is used:

    HHSearch is used to search for homologs in the SCOP database and classify a data set by superfamily.

    MATRAS program, which was trained with SCOP data, is used to score 3D structure similarity.

    How CATH is used:

    Not using CATH data.

    SCOP/CATH references:

    Under Introduction

    Different strategies for clustering the inter- faces were proposed depending on whether the entire chains (as in PRISM, IBIS) or the domains (as in SCOWLP, PIBASE, 3did, ProtCID) defined by either SCOP (22), CATH (23) or PFAM (24) were considered in the comparison process.

    Under CLUSTERING CLOSE AND REMOTE STRUCTURAL INTEROLOGS:

    To identify structural interologs below 70% sequence identity, including remotely related ones, the set of ‘CHAIN>70’ had to be further clustered to the super- family level. The profile–profile comparison algorithm HHsearch is well suited for that purpose since it was calibrated against the SCOP database to detect superfamilies relationships between sequences with high sensitivity (37).

    The fold similarity probability was defined using the reliability score calculated by Matras (42) which was calibrated by all-vs-all comparison of protein domains in SCOP 1.59 database.

  ### Attachments

  - Nucl. Acids Res.-2012-Faure-D847-56.pdf
- ## Intrinsically disordered proteins in human mitochondria

  |  |  |
  | --- | --- |
  | Type | Journal Article |
  | Author | Masahiro Ito |
  | Author | Yukako Tohsato |
  | Author | Hitoshi Sugisawa |
  | Author | Shohei Kohara |
  | Author | Satoshi Fukuchi |
  | Author | Ikuko Nishikawa |
  | Author | Ken Nishikawa |
  | URL | http://onlinelibrary.wiley.com/doi/10.1111/gtc.12000/full |
  | Volume | 17 |
  | Issue | 10 |
  | Pages | 817–825 |
  | Publication | Genes to Cells |
  | Date | 2012 |
  | Accessed | 9/23/2013, 10:18:03 AM |
  | Library Catalog | Google Scholar |
  | Date Added | 10/11/2013, 10:29:15 AM |
  | Modified | 10/11/2013, 10:29:15 AM |

  ### Notes:

  - Computational study of intrinsically disordered proteins (IDPs) in mitochondria proteins. This was done through a bioinformatics search.

    IDPs are abundant in eukaryotes, but scarce in prokaryotes.  Mitochondria in prokaryotes are known to have evolved from a eukaryotic ancestor.  Study ID ratios in mitochondria proteins with (B-type) and without (E-type) bacterial homologs.

    SCOP use:

    Use SCOP to help search for homologs.  They are interested in determining whether mitochondrial proteins have bacterial homologs.  They use BLAST to find homologs, and when the evalue is week, they referred to SCOP and Pfa**.**

    SCOP Reference:

    As described in the Methods  
    section, the proteins with a clear homologue in a-proteobacteria  
    were defined as B-type; those with weaker  
    homology in a-proteobacteria were classified as B-type  
    only if the same SCOP/Pfam domains were found.

    We found 99 proteins with SCOP/Pfam  
    domains unique to eukaryotes and defined these proteins  
    as E-type.

    Classification of proteins into B-type/E-type

    Mitochondrial proteins in the dataset selected above were clas- sified into the bacterial type (B-type) or the eukaryotic type (E-type) depending on the presence or absence, respectively, of homologues in a-proteobacteria. We used all 124 species of a-proteobacteria with a known genome, registered in the GTOP database (released in October 2010) (Fukuchi et al. 2009b). Existence of a homologue was defined by a hit in a BLAST search (Altschul et al. 1997) with an E-value less than 10"10. For weaker hits, with E-values less than 10"3, we used an additional criterion—whether the same structural/functional domains, that is, SCOP (Murzin et al. 1995) and Pfam (Finn et al. 2008) provided by GTOP, were detected in a pair of proteins. If the query protein contained multiple domains, bacterial homologues should also have the same types of domains, in the same order, along the sequence. The criterion of using domains was capable of distinguishing proteins into B-type/E-type. For instance, we reasoned that if a query pro- tein had a SCOP/Pfam domain that was detected only in eukaryotic proteins, but never in prokaryotic proteins, the query protein must be of the E-type.

  ### Attachments

  - gtc12000.pdf
  - Snapshot
- ## In vivo translation rates can substantially delay the cotranslational folding of the Escherichia coli cytosolic proteome

  |  |  |
  | --- | --- |
  | Type | Journal Article |
  | Author | Prajwal Ciryam |
  | Author | Richard I. Morimoto |
  | Author | Michele Vendruscolo |
  | Author | Christopher M. Dobson |
  | Author | Edward P. O'Brien |
  | Volume | 110 |
  | Issue | 2 |
  | Pages | E132-E140 |
  | Publication | Proceedings of the National Academy of Sciences of the United States of America |
  | ISSN | 0027-8424 |
  | Date | JAN 8 2013 |
  | Extra | WOS:000313906600007 |
  | DOI | 10.1073/pnas.1213624110 |
  | Abstract | A question of fundamental importance concerning protein folding in vivo is whether the kinetics of translation or the thermodynamics of the ribosome nascent chain (RNC) complex is the major determinant of cotranslational folding behavior. This is because translation rates can reduce the probability of cotranslational folding below that associated with arrested ribosomes, whose behavior is determined by the equilibrium thermodynamics of the RNC complex. Here, we combine a chemical kinetic equation with genomic and proteomic data to predict domain folding probabilities as a function of nascent chain length for Escherichia coli cytosolic proteins synthesized on both arrested and continuously translating ribosomes. Our results indicate that, at in vivo translation rates, about one-third of the Escherichia coli cytosolic proteins exhibit cotranslational folding, with at least one domain in each of these proteins folding into its stable native structure before the full-length protein is released from the ribosome. The majority of these cotranslational folding domains are influenced by translation kinetics which reduces their probability of cotranslational folding and consequently increases the nascent chain length at which they fold into their native structures. For about 20% of all cytosolic proteins this delay in folding can exceed the length of the completely synthesized protein, causing one or more of their domains to switch from co- to posttranslational folding solely as a result of the in vivo translation rates. These kinetic effects arise from the difference in time scales of folding and amino-acid addition, and they represent a source of metastability in Escherichia coli's proteome. |
  | Date Added | 2/20/2014, 12:24:01 PM |
  | Modified | 3/7/2014, 12:10:05 PM |

  ### Notes:

  - Computational study of translation and folding in the e. coli proteome.

    How SCOP/CATH is used:

    Used SCOP or CATH domain definitions to annotate data set.  Used DomainParser if the SCOP or CATH domain definition was not available.

    SCOP reference:

    Full details of the construction of this database are provided in Supplemental Information. Briefly, for the 4,319 unique coding sequences in the E. coli transcriptome,

    we identified those that had an X-ray structure in the Research Collabo- ratory for Structural Bioinformatics (RCSB) Protein Data Bank (PDB) (14) with a resolution of less than or equal to 3 Å or an NMR entry, and also had domain definitions in the Structural Classification of Proteins (SCOP) (15) or CATH (16) database. This procedure resulted in 802 proteins. For the 489 additional proteins that had an RCSB structure but no SCOP or CATH entry, we used Domain Parser (DP) (17) to identify their domains; the latter algo- rithm was able to identify domains in 264 of these proteins, and the remaining proteins were not included in the database. A small percentage of SCOP and CATH domains were reported to be made up of multiple seg- ments. Given that the sequence separation between segments is quite large (Fig. S1), we treated these discontiguous segments as separate domains, provided they were at least 50 residues in length. We then used PSORTb 3.0.2 (18) to classify proteins on the basis of their subcellular localization;

  ### Attachments

  - PNAS-2013-Ciryam-E132-40.pdf
- ## IS-Dom: a dataset of independent structural domains automatically delineated from protein structures

  |  |  |
  | --- | --- |
  | Type | Journal Article |
  | Author | Teppei Ebina |
  | Author | Yuki Umezawa |
  | Author | Yutaka Kuroda |
  | Volume | 27 |
  | Issue | 5 |
  | Pages | 419-426 |
  | Publication | Journal of Computer-Aided Molecular Design |
  | ISSN | 0920-654X |
  | Date | MAY 2013 |
  | Extra | WOS:000320505400003 |
  | DOI | 10.1007/s10822-013-9654-6 |
  | Abstract | Protein domains that can fold in isolation are significant targets in diverse area of proteomics research as they are often readily analyzed by high-throughput methods. Here, we report IS-Dom, a dataset of Independent Structural Domains (ISDs) that are most likely to fold in isolation. IS-Dom was constructed by filtering domains from SCOP, CATH, and DomainParser using quantitative structural measures, which were calculated by estimating inter-domain hydrophobic clusters and hydrogen bonds from the full length protein's atomic coordinates. The ISD detection protocol is fully automated, and all of the computed interactions are stored in the server which enables rapid update of IS-Dom. We also prepared a standard IS-Dom using parameters optimized by maximizing the Youden's index. The standard IS-Dom, contained 54,860 ISDs, of which 25.5 % had high sequence identity and termini overlap with a Protein Data Bank (PDB) cataloged sequence and are thus experimentally shown to fold in isolation [coined autonomously folded domain (AFDs)]. Furthermore, our ISD detection protocol missed less than |
  | Date Added | 2/20/2014, 12:24:01 PM |
  | Modified | 3/7/2014, 12:09:58 PM |

  ### Notes:

  - Present update to IS-Dom database of independent structural domains.

    How SCOP/CATH is used:

    Validate their method on a 'control group' of SCOP and CATH domain definitions.

    SCOP/CATH rereference:

    IS-Dom construction

    We constructed IS-Dom, a dataset of ISDs, using all of the 213,010 PDB chains as of January 15th 2013 (Fig. 1) which contained 16,601 SCOP, 28,030 CATH, and 37,753 DomainParser multi-domain proteins. We calculated ISDs for 21 different threshold numbers (hydrophobic cluster, MC–MC, MC–SC and SC–SC H-bonds; from 0 to 20) and 18 distance thresholds for the hydrophobic cluster (from 1.0 to 10.0 by step of 0.5A ̊). This yielded 21 9 21 9 21 9 21 9 19 = 3,695,139 ISD datasets in IS-Dom corre- sponding to all possible combinations of interaction num- bers and distance thresholds. ISDs with both N- and C-termini within 10 residues were considered identical, and the largest ISD was listed in IS-Dom.

    ...

  ### Attachments

  - art%3A10.1007%2Fs10822-013-9654-6.pdf
- ## Is Renalase a Novel Player in Catecholaminergic Signaling? The Mystery of the Catalytic Activity of an Intriguing New Flavoenzyme

  |  |  |
  | --- | --- |
  | Type | Journal Article |
  | Author | Sara Baroni |
  | Author | Mario Milani |
  | Author | Vittorio Pandini |
  | Author | Giulio Pavesi |
  | Author | David Horner |
  | Author | Alessandro Aliverti |
  | Volume | 19 |
  | Issue | 14 |
  | Pages | 2540-2551 |
  | Publication | Current Pharmaceutical Design |
  | ISSN | 1381-6128 |
  | Date | APR 2013 |
  | Extra | WOS:000316460200005 |
  | Abstract | Renalase is a flavoprotein recently discovered in humans, preferentially expressed in the proximal tubules of the kidney and secreted in blood and urine. It is highly conserved in vertebrates, with homologs identified in eukaryotic and prokaryotic organisms. Several genetic, epidemiological, clinical and experimental studies show that renalase plays a role in the modulation of the functions of the cardiovascular system, being particularly active in decreasing the catecholaminergic tone, in lowering blood pressure and in exerting a protective action against myocardial ischemic damage. Deficient renalase synthesis might be the cause of the high occurrence of hypertension and adverse cardiac events in kidney disease patients. Very recently, recombinant human renalase has been structurally and functionally characterized in vitro. Results show that it belongs to the p-hydroxybenzoate hydroxylase structural family of flavoenzymes, contains non-covalently bound FAD with redox features suggestive of a dehydrogenase activity, and is not a catecholamine-degrading enzyme, either through oxidase or NAD(P)H-dependent monooxygenase reactions. The biochemical data now available will hopefully provide the basis for a systematic and rational quest toward the identification of the reaction catalyzed by renalase and of the molecular mechanism of its physiological action, which in turn are expected to favor the development of novel therapeutic tools for the treatment of kidney and cardiovascular diseases. |
  | Date Added | 2/20/2014, 12:24:01 PM |
  | Modified | 2/20/2014, 12:24:01 PM |

  ### Notes:

  - Paper unavailable.
- ## J-TM Align: Efficient Comparison of Protein Structure Based on TM-Align

  |  |  |
  | --- | --- |
  | Type | Journal Article |
  | Author | Pietro H. Guzzi |
  | Author | Pierangelo Veltri |
  | Author | Mario Cannataro |
  | Volume | 8 |
  | Issue | 2 |
  | Pages | 220-225 |
  | Publication | Current Bioinformatics |
  | ISSN | 1574-8936 |
  | Date | April 2013 |
  | Language | English |
  | Abstract | Proteins interact among them and different interactions are represented as graphs named Protein to Protein Interaction (PPI) networks. From a physical point of view, interactions are performed by contacts among protein structure. Consequently, the study and the comparison of protein structure is an important field in Bioinformatics and Computational Biology. The TM-Align algorithm is a method that presents one of the best performance but is currently available only as a stand alone application with a simple command-line interface available only on Linux platforms. We provide a comprehensive tool, (J-TMAlign) allowing a graphical, easy to use, interface to access J-TMAlign functions and the possibility to visualize compared structure. Finally, J-TMAlign is based on a multi-threaded architecture enables user to submit multiple jobs that are executed in a concurrent and time-efficient way. |
  | Date Added | 10/11/2013, 10:29:15 AM |
  | Modified | 3/7/2014, 12:09:40 PM |

  ### Tags:

  - Distributed processing
  - docking
  - multithreads
  - protein analysis
  - protein structure
  - protein structure comparison
  - wrappers

  ### Notes:

  - No access to article.
- ## KB-Rank: efficient protein structure and functional annotation identification via text query

  |  |  |
  | --- | --- |
  | Type | Journal Article |
  | Author | Elchin S Julfayev |
  | Author | Ryan J McLaughlin |
  | Author | Yi-Ping Tao |
  | Author | William A McLaughlin |
  | Volume | 13 |
  | Issue | 2 |
  | Pages | 101-110 |
  | Publication | Journal of structural and functional genomics |
  | ISSN | 1570-0267 |
  | Date | Jun 2012 |
  | Extra | PMID: 22270457 |
  | Journal Abbr | J. Struct. Funct. Genomics |
  | DOI | 10.1007/s10969-012-9125-7 |
  | Library Catalog | NCBI PubMed |
  | Language | eng |
  | Abstract | The KB-Rank tool was developed to help determine the functions of proteins. A user provides text query and protein structures are retrieved together with their functional annotation categories. Structures and annotation categories are ranked according to their estimated relevance to the queried text. The algorithm for ranking first retrieves matches between the query text and the text fields associated with the structures. The structures are next ordered by their relative content of annotations that are found to be prevalent across all the structures retrieved. An interactive web interface was implemented to navigate and interpret the relevance of the structures and annotation categories retrieved by a given search. The aim of the KB-Rank tool is to provide a means to quickly identify protein structures of interest and the annotations most relevant to the queries posed by a user. Informational and navigational searches regarding disease topics are described to illustrate the tool's utilities. The tool is available at the URL http://protein.tcmedc.org/KB-Rank. |
  | Short Title | KB-Rank |
  | Date Added | 2/20/2014, 12:24:01 PM |
  | Modified | 3/7/2014, 12:14:34 PM |

  ### Tags:

  - Algorithms
  - Animals
  - Computational Biology
  - Databases, Protein
  - Humans
  - Internet
  - Models, Molecular
  - Molecular Sequence Annotation
  - Protein Conformation
  - Proteins
  - Search Engine
  - Sequence Analysis, Protein
  - Software
  - Structure-Activity Relationship

  ### Notes:

  - Present KB-Rank server to retrieve structure and functional annotation via text queries.

    How SCOP/CATH is used:

    Include SCOP and CATH domains and superfamily annotation for protein structures in database.

    SCOP reference:

    Structural domains assignments were provided through the CATH [27] and SCOP [28] databases.

    ...

    Annotation catego- ries that are available for browsing include cellular path- ways retrieved from the National Cancer Institute’s Pathway Interaction Database [15]; superfamily designa- tions provided from the SCOP database [28]; ...

  ### Attachments

  - art%3A10.1007%2Fs10969-012-9125-7.pdf
  - PubMed entry
- ## kClust: fast and sensitive clustering of large protein sequence databases

  |  |  |
  | --- | --- |
  | Type | Journal Article |
  | Author | Maria Hauser |
  | Author | Christian E. Mayer |
  | Author | Johannes Soeding |
  | Volume | 14 |
  | Pages | UNSP 248 |
  | Publication | Bmc Bioinformatics |
  | Date | AUG 15 2013 |
  | Extra | WOS:000323789700001 |
  | DOI | 10.1186/1471-2105-14-248 |
  | Library Catalog | ISI Web of Knowledge |
  | Abstract | Background: Fueled by rapid progress in high-throughput sequencing, the size of public sequence databases doubles every two years. Searching the ever larger and more redundant databases is getting increasingly inefficient. Clustering can help to organize sequences into homologous and functionally similar groups and can improve the speed, sensitivity, and readability of homology searches. However, because the clustering time is quadratic in the number of sequences, standard sequence search methods are becoming impracticable. Results: Here we present a method to cluster large protein sequence databases such as UniProt within days down to 20%-30% maximum pairwise sequence identity. kClust owes its speed and sensitivity to an alignment-free prefilter that calculates the cumulative score of all similar 6-mers between pairs of sequences, and to a dynamic programming algorithm that operates on pairs of similar 4-mers. To increase sensitivity further, kClust can run in profile-sequence comparison mode, with profiles computed from the clusters of a previous kClust iteration. kClust is two to three orders of magnitude faster than clustering based on NCBI BLAST, and on multidomain sequences of 20%-30% maximum pairwise sequence identity it achieves comparable sensitivity and a lower false discovery rate. It also compares favorably to CD-HIT and UCLUST in terms of false discovery rate, sensitivity, and speed. Conclusions: kClust fills the need for a fast, sensitive, and accurate tool to cluster large protein sequence databases to below 30% sequence identity. kClust is freely available under GPL at ftp://toolkit.lmb.uni-muenchen.de/pub/kClust/. |
  | Short Title | kClust |
  | Date Added | 10/8/2014, 12:49:22 PM |
  | Modified | 10/8/2014, 1:32:34 PM |

  ### Attachments

  - Full Text PDF
  - Snapshot
- ## KD4v: comprehensible knowledge discovery system for missense variant

  |  |  |
  | --- | --- |
  | Type | Journal Article |
  | Author | Tien-Dao Luu |
  | Author | Alin Rusu |
  | Author | Vincent Walter |
  | Author | Benjamin Linard |
  | Author | Laetitia Poidevin |
  | Author | Raymond Ripp |
  | Author | Luc Moulinier |
  | Author | Jean Muller |
  | Author | Wolfgang Raffelsberger |
  | Author | Nicolas Wicker |
  | Author | Odile Lecompte |
  | Author | Julie D. Thompson |
  | Author | Olivier Poch |
  | Author | Hoan Nguyen |
  | Volume | 40 |
  | Issue | W1 |
  | Pages | W71-W75 |
  | Publication | Nucleic Acids Research |
  | ISSN | 0305-1048 |
  | Date | JUL 2012 |
  | Extra | WOS:000306670900012 |
  | DOI | 10.1093/nar/gks474 |
  | Abstract | A major challenge in the post-genomic era is a better understanding of how human genetic alterations involved in disease affect the gene products. The KD4v (Comprehensible Knowledge Discovery System for Missense Variant) server allows to characterize and predict the phenotypic effects (deleterious/neutral) of missense variants. The server provides a set of rules learned by Induction Logic Programming (ILP) on a set of missense variants described by conservation, physico-chemical, functional and 3D structure predicates. These rules are interpretable by non-expert humans and are used to accurately predict the deleterious/neutral status of an unknown mutation. The web server is available at http://decrypthon.igbmc.fr/kd4v. |
  | Date Added | 2/20/2014, 12:24:01 PM |
  | Modified | 2/20/2014, 12:24:01 PM |

  ### Notes:

  - Present KD4v web server for predicting phenotypic effects of missense variants.

    How SCOP is used:

    Annotate sequence data set with SCOP fold classification.

    SCOP reference:

    In KD4v, this multi-level sequence-based charac- terization of nsSNPs is complemented by parameters related to 3D models or the 3D Fold classification in SCOP (13). This results in pre-computed annotations for over 63000 known nsSNPs in the 10713 proteins with known or modelled 3D structures currently available.

  ### Attachments

  - Nucl. Acids Res.-2012-Luu-W71-5.pdf
- ## KIDFamMap: a database of kinase-inhibitor-disease family maps for kinase inhibitor selectivity and binding mechanisms

  |  |  |
  | --- | --- |
  | Type | Journal Article |
  | Author | Yi-Yuan Chiu |
  | Author | Chih-Ta Lin |
  | Author | Jhang-Wei Huang |
  | Author | Kai-Cheng Hsu |
  | Author | Jen-Hu Tseng |
  | Author | Syuan-Ren You |
  | Author | Jinn-Moon Yang |
  | Volume | 41 |
  | Issue | D1 |
  | Pages | D430-D440 |
  | Publication | Nucleic Acids Research |
  | ISSN | 0305-1048 |
  | Date | JAN 2013 |
  | Extra | WOS:000312893300061 |
  | DOI | 10.1093/nar/gks1218 |
  | Abstract | Kinases play central roles in signaling pathways and are promising therapeutic targets for many diseases. Designing selective kinase inhibitors is an emergent and challenging task, because kinases share an evolutionary conserved ATP-binding site. KIDFamMap (http://gemdock.life.nctu.edu.tw/KIDFamMap/) is the first database to explore kinase-inhibitor families (KIFs) and kinase-inhibitor-disease (KID) relationships for kinase inhibitor selectivity and mechanisms. This database includes 1208 KIFs, 962 KIDs, 55 603 kinase-inhibitor interactions (KIIs), 35 788 kinase inhibitors, 399 human protein kinases, 339 diseases and 638 disease allelic variants. Here, a KIF can be defined as follows: (i) the kinases in the KIF with significant sequence similarity, (ii) the inhibitors in the KIF with significant topology similarity and (iii) the KIIs in the KIF with significant interaction similarity. The KIIs within a KIF are often conserved on some consensus KIDFamMap anchors, which represent conserved interactions between the kinase subsites and consensus moieties of their inhibitors. Our experimental results reveal that the members of a KIF often possess similar inhibition profiles. The KIDFamMap anchors can reflect kinase conformations types, kinase functions and kinase inhibitor selectivity. We believe that KIDFamMap provides biological insights into kinase inhibitor selectivity and binding mechanisms. |
  | Date Added | 10/28/2013, 4:53:08 PM |
  | Modified | 10/28/2013, 4:53:08 PM |

  ### Notes:

  - Present KIDFamMap database of kinase-inhibtor-disease family maps.

    How SCOP is used:

    Background on protein structure classification.

    SCOP reference:

    As a result, a group of KIIs with consensus anchors can constitute a kinase- inhibitor family (KIF), which is analogous to a protein sequence family (18,19), a structure family (20) and a protein–protein interaction family (21).

  ### Attachments

  - Nucl. Acids Res.-2013-Chiu-D430-40.pdf
- ## Kinetic properties of alternatively spliced isoforms of laccase-2 from Tribolium castaneum and Anopheles gambiae

  |  |  |
  | --- | --- |
  | Type | Journal Article |
  | Author | Maureen J Gorman |
  | Author | Lucinda I Sullivan |
  | Author | Thi D T Nguyen |
  | Author | Huaien Dai |
  | Author | Yasuyuki Arakane |
  | Author | Neal T Dittmer |
  | Author | Lateef U Syed |
  | Author | Jun Li |
  | Author | Duy H Hua |
  | Author | Michael R Kanost |
  | Volume | 42 |
  | Issue | 3 |
  | Pages | 193-202 |
  | Publication | Insect biochemistry and molecular biology |
  | ISSN | 1879-0240 |
  | Date | Mar 2012 |
  | Extra | PMID: 22198355 |
  | Journal Abbr | Insect Biochem. Mol. Biol. |
  | DOI | 10.1016/j.ibmb.2011.11.010 |
  | Library Catalog | NCBI PubMed |
  | Language | eng |
  | Abstract | Laccase-2 is a highly conserved multicopper oxidase that functions in insect cuticle pigmentation and tanning. In many species, alternative splicing gives rise to two laccase-2 isoforms. A comparison of laccase-2 sequences from three orders of insects revealed eleven positions at which there are conserved differences between the A and B isoforms. Homology modeling suggested that these eleven residues are not part of the substrate binding pocket. To determine whether the isoforms have different kinetic properties, we compared the activity of laccase-2 isoforms from Tribolium castaneum and Anopheles gambiae. We partially purified the four laccases as recombinant enzymes and analyzed their ability to oxidize a range of laccase substrates. The predicted endogenous substrates tested were dopamine, N-acetyldopamine (NADA), N-β-alanyldopamine (NBAD) and dopa, which were detected in T. castaneum previously and in A. gambiae as part of this study. Two additional diphenols (catechol and hydroquinone) and one non-phenolic substrate (2,2'-azino-bis(3-ethylbenzthiazoline-6-sulphonic acid)) were also tested. We observed no major differences in substrate specificity between the A and B isoforms. Dopamine, NADA and NBAD were oxidized with catalytic efficiencies ranging from 51 to 550 min⁻¹ mM⁻¹. These results support the hypothesis that dopamine, NADA and NBAD are endogenous substrates for both isoforms of laccase-2. Catalytic efficiencies associated with dopa oxidation were low, ranging from 8 to 30 min⁻¹ mM⁻¹; in comparison, insect tyrosinase oxidized dopa with a catalytic efficiency of 201 min⁻¹ mM⁻¹. We found that dopa had the highest redox potential of the four endogenous substrates, and this property of dopa may explain its poor oxidation by laccase-2. We conclude that laccase-2 splice isoforms are likely to oxidize the same substrates in vivo, and additional experiments will be required to discover any isoform-specific functions. |
  | Date Added | 10/11/2013, 10:29:15 AM |
  | Modified | 10/11/2013, 10:29:15 AM |

  ### Tags:

  - Alternative Splicing
  - Amino Acid Sequence
  - Animals
  - Anopheles gambiae
  - Catecholamines
  - Cuticle
  - Female
  - Hydrogen-Ion Concentration
  - Insect
  - Insect Proteins
  - Isoenzymes
  - Kinetics
  - Laccase
  - Laccase
  - Male
  - Molecular Sequence Data
  - Multicopper oxidase
  - Oxidation-Reduction
  - Recombinant Proteins
  - Substrate
  - Substrate Specificity
  - Tribolium

  ### Notes:

  - Computational study of two laccase-2 isoforms across different species.  Most laccases consiste of three cupredoxin-like domains.

    How SCOP is used:

    To get domain boundaries for a cupredoxin-like domains for some of their isoforms.  Define boundaries of putative  cupredoxin-like domains by aligning with homolog in SCOP.

    SCOP reference:

    Boundaries of the putative cupredoxin-like domains were estimated by aligning laccase-2 sequences with the sequence of a fungal laccase, Trametes versicolor laccaseIIIb (TvLacIIIb, PDB ID: 1KYA), which has a solved crystal structure, and then using SCOP (Murzin et al., 1995) to define the boundaries of the cupredoxin-like domains of TvLacIIIb (Figure S1).

  ### Attachments

  - 1-s2.0-S0965174811002141-main.pdf
  - PubMed entry
- ## Large-scale analysis of conserved rare codon clusters suggests an involvement in co-translational molecular recognition events

  |  |  |
  | --- | --- |
  | Type | Journal Article |
  | Author | Matthieu Chartier |
  | Author | Francis Gaudreault |
  | Author | Rafael Najmanovich |
  | Volume | 28 |
  | Issue | 11 |
  | Pages | 1438-1445 |
  | Publication | Bioinformatics |
  | ISSN | 1367-4803 |
  | Date | JUN 1 2012 |
  | Extra | WOS:000304537000004 |
  | DOI | 10.1093/bioinformatics/bts149 |
  | Abstract | Motivation: An increasing amount of evidence from experimental and computational analysis suggests that rare codon clusters are functionally important for protein activity. Most of the studies on rare codon clusters were performed on a limited number of proteins or protein families. In the present study, we present the Sherlocc program and how it can be used for large scale protein family analysis of evolutionarily conserved rare codon clusters and their relation to protein function and structure. This large-scale analysis was performed using the whole Pfam database covering over 70% of the known protein sequence universe. Our program Sherlocc, detects statistically relevant conserved rare codon clusters and produces a user-friendly HTML output. Results: Statistically significant rare codon clusters were detected in a multitude of Pfam protein families. The most statistically significant rare codon clusters were predominantly identified in N-terminal Pfam families. Many of the longest rare codon clusters are found in membrane-related proteins which are required to interact with other proteins as part of their function, for example in targeting or insertion. We identified some cases where rare codon clusters can play a regulating role in the folding of catalytically important domains. Our results support the existence of a widespread functional role for rare codon clusters across species. Finally, we developed an online filter-based search interface that provides access to Sherlocc results for all Pfam families. |
  | Date Added | 2/13/2014, 4:13:17 PM |
  | Modified | 3/7/2014, 12:15:08 PM |
- ## Large-Scale Modelling of the Divergent Spectrin Repeats in Nesprins: Giant Modular Proteins

  |  |  |
  | --- | --- |
  | Type | Journal Article |
  | Author | Flavia Autore |
  | Author | Mark Pfuhl |
  | Author | Xueping Quan |
  | Author | Aisling Williams |
  | Author | Roland G. Roberts |
  | Author | Catherine M. Shanahan |
  | Author | Franca Fraternali |
  | Volume | 8 |
  | Issue | 5 |
  | Publication | PLoS one |
  | ISSN | 1932-6203 |
  | Date | MAY 6 2013 |
  | DOI | 10.1371/journal.pone.0063633 |
  | Language | English |
  | Abstract | Nesprin-1 and nesprin-2 are nuclear envelope (NE) proteins characterized by a common structure of an SR (spectrin repeat) rod domain and a C-terminal transmembrane KASH [Klarsicht-ANC-Syne-homology] domain and display N-terminal actin-binding CH (calponin homology) domains. Mutations in these proteins have been described in Emery-Dreifuss muscular dystrophy and attributed to disruptions of interactions at the NE with nesprins binding partners, lamin A/C and emerin. Evolutionary analysis of the rod domains of the nesprins has shown that they are almost entirely composed of unbroken SR-like structures. We present a bioinformatical approach to accurate definition of the boundaries of each SR by comparison with canonical SR structures, allowing for a large-scale homology modelling of the 74 nesprin-1 and 56 nesprin-2 SRs. The exposed and evolutionary conserved residues identify important pbs for protein-protein interactions that can guide tailored binding experiments. Most importantly, the bioinformatics analyses and the 3D models have been central to the design of selected constructs for protein expression. 1D NMR and CD spectra have been performed of the expressed SRs, showing a folded, stable, high content a-helical structure, typical of SRs. Molecular Dynamics simulations have been performed to study the structural and elastic properties of consecutive SRs, revealing insights in the mechanical properties adopted by these modules in the cell. |
  | Date Added | 10/11/2013, 10:29:15 AM |
  | Modified | 12/2/2013, 4:16:25 PM |

  ### Notes:

  - Study spectrin repeat (SR) domains in Nesprins (nuclear envelope proteins).

    How SCOP is used:

    Collect a data set of SR-containing proteins and then assign boundaries to SR domains using SCOP in order to get single domain SRs.

    SCOP reference:

    ### Methods

    #### Extraction of single domain SR Structures and Boundary Assignments

    Protein Structures containing SRs were extracted by BLAST [43] from the Protein Data Bank (PDB). The selected structures are: human α-actinin 1 (P12814), α-actinin 2 (P35609), α-actinin 3 (Q08043), α-actinin 4 (Q43707), chicken α-actinin 1 (P05094), human α-spectrin 1 (P02549), α-spectrin 2(Q13813), chicken α-spectrin 2 (P07751), human β-spectrin 1 (P11277), β-spectrin 2 (Q01082), chicken β-spectrin 2 (P07751), fruit fly α-spectrin (P13395), human utrophin (P46939), human dystrophin (P11532) and human nesprin-1 (Q8NF91) as reported inTable 1. To be noted that some of the selected structures contain more than a single SR. To extract single SR domain structures from templates with multiple SRs to be used in the modelling procedure, we had to assign the correct boundaries that define the ‘canonical’ SR topology.

    The single domain SR templates were extracted by Perl scripts following the SR assignments on their protein sequences by SCOP [44] and SWISSPROT database [45]. The sequences of these single domain SRs, were used with HMMER2.3 [46] to construct a tailored SR seed alignment based on the alignment (PF00435) [20]. Newly defined boundaries were assigned based on SCOP assignment for SR, which divides adjacent SRs into two triple-helix structure units. Structural alignments of these assigned domain structures were carried out with web server MAMMOTH-mult [47].

    3DCoffee software [48] has been used to generate the high-quality multiple sequence alignments of nesprins SRs (Figure S1). The sequences of SR templates extracted from SCOP were used to guide the multiple alignments since the structural information extract from the templates can help to improve the quality of the alignments.

  ### Attachments

  - journal.pone.0063633.pdf
- ## LB3D: A Protein Three-Dimensional Substructure Search Program Based on the Lower Bound of a Root Mean Square Deviation Value

  |  |  |
  | --- | --- |
  | Type | Journal Article |
  | Author | Genki Terashi |
  | Author | Tetsuo Shibuya |
  | Author | Mayuko Takeda-Shitaka |
  | Volume | 19 |
  | Issue | 5 |
  | Pages | 493-503 |
  | Publication | JOURNAL OF COMPUTATIONAL BIOLOGY |
  | ISSN | 1066-5277 |
  | Date | May 2012 |
  | DOI | 10.1089/cmb.2011.0230 |
  | Language | English |
  | Abstract | Searching for protein structure-function relationships using three-dimensional (3D) structural coordinates represents a fundamental approach for determining the function of proteins with unknown functions. Since protein structure databases are rapidly growing in size, the development of a fast search method to find similar protein substructures by comparison of protein 3D structures is essential. In this article, we present a novel protein 3D structure search method to find all substructures with root mean square deviations (RMSDs) to the query structure that are lower than a given threshold value. Our new algorithm runs in O(m + N/m(0.5)) time, after O(N log N) preprocessing, where N is the database size and m is the query length. The new method is 1.8-41.6 times faster than the practically best known O(N) algorithm, according to computational experiments using a huge database (i.e., > 20,000,000 C-alpha coordinates). |
  | Date Added | 10/25/2013, 4:17:08 PM |
  | Modified | 10/25/2013, 4:17:08 PM |

  ### Tags:

  - algorithms
  - computational molecular biology
  - Databases
  - Protein
  - protein families
  - protein folding
  - protein structure

  ### Notes:

  - Present a method for rapidly searching a database for similar structures.

    How SCOP is used:

    Evaluate method on SCOP 1.75.They downloaded all domain structures for SCOP 1.75 from ASTRAL and randomly selected 100 as query domains.

    SCOP reference:

    3.1 Computational experiments on the SCOP1.75 database

    To test the performance of the new algorithm, we used the SCOP database (Andreeva et al., 2008) release 1.75 (denoted as SCOP1.75), which contains 110,799 domains with a total of 20,429,263 C-alpha coordinates. We randomly selected 100 domains from SCOP1.75 as query domains for this experiment. These 100 domains contained a total of 6,710–22,692 substructures depending on the length of sub- structures (10–200). All the 3D coordinates are taken from the ASTRAL database (Chandonia et al., 2004).

  ### Attachments

  - cmb%2E2011%2E0230.pdf
- ## lDDT: a local superposition-free score for comparing protein structures and models using distance difference tests

  |  |  |
  | --- | --- |
  | Type | Journal Article |
  | Author | Valerio Mariani |
  | Author | Marco Biasini |
  | Author | Alessandro Barbato |
  | Author | Torsten Schwede |
  | Volume | 29 |
  | Issue | 21 |
  | Pages | 2722–2728 |
  | Publication | Bioinformatics |
  | Date | November 2013 |
  | DOI | 10.1093/bioinformatics/btt473 |
  | Abstract | Motivation: The assessment of protein structure prediction techniques requires objective criteria to measure the similarity between a computational model and the experimentally determined reference structure. Conventional similarity measures based on a global superposition of carbon alpha atoms are strongly influenced by domain motions and do not assess the accuracy of local atomic details in the model. Results: The Local Distance Difference Test (lDDT) is a superpositionfree score that evaluates local distance differences of all atoms in a model, including validation of stereochemical plausibility. The reference can be a single structure, or an ensemble of equivalent structures. We demonstrate that lDDT is well suited to assess local model quality, even in the presence of domain movements, while maintaining good correlation with global measures. These properties make lDDT a robust tool for the automated assessment of structure prediction servers without manual intervention. |
  | Date Added | 3/7/2014, 12:08:00 PM |
  | Modified | 3/7/2014, 12:08:00 PM |
- ## LigSearch: a knowledge-based web server to identify likely ligands for a protein target

  |  |  |
  | --- | --- |
  | Type | Journal Article |
  | Author | Tjaart A. P. de Beer |
  | Author | Roman A. Laskowski |
  | Author | Mark-Eugene Duban |
  | Author | A. W. Edith Chan |
  | Author | Wayne F. Anderson |
  | Author | Janet M. Thornton |
  | Volume | 69 |
  | Pages | 2395–2402 |
  | Publication | Acta Crystallographica Section D-biological Crystallography |
  | Date | December 2013 |
  | DOI | 10.1107/S0907444913022294 |
  | Abstract | Identifying which ligands might bind to a protein before crystallization trials could provide a significant saving in time and resources. LigSearch, a web server aimed at predicting ligands that might bind to and stabilize a given protein, has been developed. Using a protein sequence and/or structure, the system searches against a variety of databases, combining available knowledge, and provides a clustered and ranked output of possible ligands. LigSearch can be accessed at http://www.ebi.ac.uk/thornton-srv/databases/LigSearch. |
  | Date Added | 3/7/2014, 12:08:00 PM |
  | Modified | 3/7/2014, 12:08:00 PM |
- ## Limitations of Gene Duplication Models: Evolution of Modules in Protein Interaction Networks

  |  |  |
  | --- | --- |
  | Type | Journal Article |
  | Author | Frank Emmert-Streib |
  | Volume | 7 |
  | Issue | 4 |
  | Pages | e35531 |
  | Publication | Plos One |
  | Date | April 2012 |
  | DOI | 10.1371/journal.pone.0035531 |
  | Abstract | It has been generally acknowledged that the module structure of protein interaction networks plays a crucial role with respect to the functional understanding of these networks. In this paper, we study evolutionary aspects of the module structure of protein interaction networks, which forms a mesoscopic level of description with respect to the architectural principles of networks. The purpose of this paper is to investigate limitations of well known gene duplication models by showing that these models are lacking crucial structural features present in protein interaction networks on a mesoscopic scale. This observation reveals our incomplete understanding of the structural evolution of protein networks on the module level. |
  | Date Added | 3/7/2014, 1:06:24 PM |
  | Modified | 3/7/2014, 1:06:24 PM |
- ## LNA: Fast Protein Structural Comparison Using a Laplacian Characterization of Tertiary Structure

  |  |  |
  | --- | --- |
  | Type | Journal Article |
  | Author | Nicolas Bonnel |
  | Author | Pierre-Francois Marteau |
  | Volume | 9 |
  | Issue | 5 |
  | Pages | 1451-1458 |
  | Publication | IEEE-ACM TRANSACTIONS ON COMPUTATIONAL BIOLOGY AND BIOINFORMATICS |
  | ISSN | 1545-5963 |
  | Date | SEP-OCT 2012 |
  | DOI | 10.1109/TCBB.2012.64 |
  | Language | English |
  | Abstract | In the last two decades, a lot of protein 3D shapes have been discovered, characterized, and made available thanks to the Protein Data Bank (PDB), that is nevertheless growing very quickly. New scalable methods are thus urgently required to search through the PDB efficiently. This paper presents an approach entitled LNA (Laplacian Norm Alignment) that performs a structural comparison of two proteins with dynamic programming algorithms. This is achieved by characterizing each residue in the protein with scalar features. The feature values are calculated using a Laplacian operator applied on the graph corresponding to the adjacency matrix of the residues. The weighted Laplacian operator we use estimates, at various scales, local deformations of the topology where each residue is located. On some benchmarks, which are widely shared by the community, we obtain qualitatively similar results compared to other competing approaches, but with an algorithm one or two order of magnitudes faster. 180,000 protein comparisons can be done within 1 second with a single recent Graphical Processing Unit (GPU), which makes our algorithm very scalable and suitable for real-time database querying across the web. |
  | Date Added | 10/25/2013, 4:17:08 PM |
  | Modified | 10/8/2014, 12:50:22 PM |

  ### Tags:

  - classification
  - GPU implementation
  - Laplacian
  - Proteins
  - structural comparison

  ### Notes:

  - Present method for structure alignment.

    How SCOP is used:

    1. Use a representative set from ASTRAL to help train parameters for the method.

    2. Validate on a 3rd-party data non-redundant data set which was annotated with SCOP fold, superfamily, and family data.  Test if similar structure are in the same family.

    How CATH is used:

    Not using CATH data.

    SCOP reference:

    3 RESULTS AND DISCUSSION

    Dynamic programming algorithms require low memory usage. Both LNANWk and LNASWk algorithms are imple- mented on both CPU using C/C++ and GPU using the OpenCL language [21]. Experiments are performed on a 2 GHz Intel Xeon CPU and Nvidia Tesla M2050 GPU. We first use a subset of SCOP [18] to determines optimal parameters. We then evaluate our approach on ranking and classification tasks against two different data sets: COPS [12] and proteus300 [2], [11]. We also measure speed performance on the whole PDB database to see how our approach scales when applied to real databases.

    3.1 Optimization of the Parameter Values

    Both of the dynamic algorithms, we propose require the adjustment of a few parameters. We determine optimal sets of parameter values using a subset of SCOP downloaded on the Astral compendium website [7]. From the 40 percent ID filtered subset of SCOP 1.75, among 640 families having at least four members, we select at random 4 proteins from each family. We then build 4 subsets (one protein from each family in each subset) and perform a fourfold cross validation to optimize the set of parameters. For each query set, we record the top three returned results and check whether they belong to the same family of the query or not. Thus, the best possible score is 3 ⬚⬚ 4 ⬚⬚ 640 1⁄4 7;680.

    ...

    3.3 Proteus300

    The proteus300 data set was first used in [2] and later in [11]. It contains 300 protein domains evenly distributed across 30 SCOP families (27 super families and 24 folds). The number of residues in the proteins ranges from 64 to 455. Protein length are quite homogeneous inside families, and we expect that global alignment methods perform well on this task.

    3.3.1 Structural Comparison Performances

    We measure classification, Area Under the ROC Curve (AUC) [4], maximum accuracy, clustering, and speed performances. We compare our approach to the same methods as those in Section 3.2: [40], [22], [6] using the same settings. We also add MAMMOTH [29] to the comparison and report results from [11]. Results are summarized in Table 3. AUC and accuracy measures were computed using the ROCR package [34].

    For each protein, we compute its similarity score with all other proteins and assign the family of the most similar protein in the data set (Nearest Neighbor rule). We then measure the number of correctly assigned families for the

    whole data set. YAKUZA and GOSSIP obtain the worst results. All other presented approaches reach similar classification performances (Table 3, col. 4): all approaches have at most three errors on the 300 protein domains and 6 of them (LNANW1, LNANW2, FAST, TM-align, A\_purva+sse, and Eig\_7) have no error.

    CATH reference:

    Orengo and Taylor [28] use double dynamic programming on vector of C⬚⬚ distances and are used to build the CATH [8] classification.

  ### Attachments

  - 06193093.pdf
- ## Local Conformational Changes in the DNA Interfaces of Proteins

  |  |  |
  | --- | --- |
  | Type | Journal Article |
  | Author | Tomoko Sunami |
  | Author | Hidetoshi Kono |
  | URL | http://dx.plos.org/10.1371/journal.pone.0056080 |
  | Volume | 8 |
  | Issue | 2 |
  | Pages | e56080 |
  | Publication | PloS one |
  | Date | 2013 |
  | Accessed | 9/20/2013, 1:20:18 PM |
  | Library Catalog | Google Scholar |
  | Date Added | 10/11/2013, 10:29:15 AM |
  | Modified | 10/11/2013, 10:29:15 AM |

  ### Tags:

  - SCOP coverage insufficient

  ### Notes:

  - Computational study of of DNA-binding protein interfaces, and their conformational changes between the bound and unbound forms.

    How SCOP is used:

    Get SCOP class for their data set for statistics.

    SCOP reference:

    Dataset Preparation and Structural Alphabet Assignment

    One hundred and twenty-six representative pairs of clusters in the DNA-free (DBfree) and DNA-bound (DBbound) forms were obtained with a sequence similarity of less than 30%. The representative clusters were a set of subclusters with the largest members within each cluster (Table 1 and Table S1). The proteins of the 126 clusters had dsDNA binding domains that belonged to different structural classes according to the SCOP classification (version 1.75)[36]: 43 all alpha proteins, 12 all beta proteins, 30 alpha and beta proteins (a/b), 22 alpha and beta proteins (a+b), 11 multi-domain proteins (a andb), 1 small protein, and 1 coiled coil protein. The remaining 32 proteins were not classified in the SCOP database.

  ### Attachments

  - [HTML] from plos.org
  - journal.pone.0056080.pdf
- ## Local Network Patterns in Protein-Protein Interfaces

  |  |  |
  | --- | --- |
  | Type | Journal Article |
  | Author | Qiang Luo |
  | Author | Rebecca Hamer |
  | Author | Gesine Reinert |
  | Author | Charlotte M. Deane |
  | URL | http://dx.plos.org/10.1371/journal.pone.0057031 |
  | Volume | 8 |
  | Issue | 3 |
  | Pages | e57031 |
  | Publication | PloS one |
  | Date | 2013 |
  | Accessed | 9/23/2013, 10:15:04 AM |
  | Library Catalog | Google Scholar |
  | Date Added | 10/11/2013, 10:29:15 AM |
  | Modified | 10/11/2013, 10:29:15 AM |

  ### Notes:

  - Computational study of contact networks within protein-protein binding interfaces.

    How SCOP is used:

    Analyzed three data sets of homodimers, heterodimers, and domain-domain (multidomain proteins) interfaces.  Used SCOP to collect domain annotations.

    SCOP reference:

    Data sets

    The main data sets used in this study were built as described in [23]; here is a brief review. Three data sets of 1150 two domain proteins, 583 homodimers, and 94 heterodimers were used in this paper to discover the local network patterns at interfaces. Each entry in the database has a 3-D structure with a resolution better than 2.5A ̊ . The interface residues were identified as residues which are 4.5A ̊ or less away from a residue on the other protein or domain. Domain annotations were collected from SCOP [28], while the complexes were gathered by querying the PDB [29]. Sequence identity in the database is less than 70%, the change in the accessible surface area (ASA) on binding for all proteins is greater than 175A ̊ 2 , and the sequence length of each chain is more than 100. Notably, the crystal contacts are difficult to be excluded completely from the database since a crystal contact may bury as much as 800A ̊ 2 of the surface area [30]. However, in this paper, the possible crystal contacts in the protein complexes can only be a small portion of the whole data set of interfaces since we have chosen the oligomeric state of 2 in PDB for the complexes. The set of two-domain proteins comes from SCOP and will not have crystal contacts. While the count numbers of different types 4- cliques at interfaces of the combined database including all three types of interfaces and the corresponding numbers counted at the domain-domain interfaces have a correlation coefficient of 0.9988, a chi-square test rejects the hypothesis that the distributions are the same across the three databases. The domain-domain interfaces being the largest of our three data sets, we first use the data set of the domain-domain interfaces to discover the local network patterns for interfaces, and then compare the findings to that of the homodimer interfaces and heterodimer interfaces to reveal more subtle characteristics for the interfaces. In the results section, unless specified explicitly the data set mentioned in the Result section means the data set of the domain-domain interfaces.

  ### Attachments

  - [HTML] from plos.org
  - journal.pone.0057031.pdf
- ## Local Structural Differences in Homologous Proteins: Specificities in Different SCOP Classes

  |  |  |
  | --- | --- |
  | Type | Journal Article |
  | Author | Agnel Praveen Joseph |
  | Author | Hélène Valadié |
  | Author | Narayanaswamy Srinivasan |
  | Author | Alexandre G. de Brevern |
  | URL | http://dx.plos.org/10.1371/journal.pone.0038805 |
  | Volume | 7 |
  | Issue | 6 |
  | Pages | e38805 |
  | Publication | PloS one |
  | Date | 2012 |
  | Accessed | 2/28/2013, 1:38:04 PM |
  | Library Catalog | Google Scholar |
  | Abstract | The constant increase in the number of solved protein structures is of great help in understanding the basic principles behind protein folding and evolution. 3-D structural knowledge is valuable in designing and developing methods for comparison, modelling and prediction of protein structures. These approaches for structure analysis can be directly implicated in studying protein function and for drug design. The backbone of a protein structure favours certain local conformations which include α-helices, β-strands and turns. Libraries of limited number of local conformations (Structural Alphabets) were developed in the past to obtain a useful categorization of backbone conformation. Protein Block (PB) is one such Structural Alphabet that gave a reasonable structure approximation of 0.42 Å. In this study, we use PB description of local structures to analyse conformations that are preferred sites for structural variations and insertions, among group of related folds. This knowledge can be utilized in improving tools for structure comparison that work by analysing local structure similarities. Conformational differences between homologous proteins are known to occur often in the regions comprising turns and loops. Interestingly, these differences are found to have specific preferences depending upon the structural classes of proteins. Such class-specific preferences are mainly seen in the all-β class with changes involving short helical conformations and hairpin turns. A test carried out on a benchmark dataset also indicates that the use of knowledge on the class specific variations can improve the performance of a PB based structure comparison approach. The preference for the indel sites also seem to be confined to a few backbone conformations involving β-turns and helix C-caps. These are mainly associated with short loops joining the regular secondary structures that mediate a reversal in the chain direction. Rare β-turns of type I’ and II’ are also identified as preferred sites for insertions. |
  | Short Title | Local Structural Differences in Homologous Proteins |
  | Date Added | 10/11/2013, 10:29:15 AM |
  | Modified | 10/11/2013, 10:29:15 AM |

  ### Tags:

  - Amino Acid Sequence
  - Proteins
  - Protein Structure, Secondary

  ### Notes:

  - An alternative way to describe a protein's backbone conformation is using protein blocks (PBs) taken from a structural alphabet.  In this work, the PBs are a set of 16 prototypes of main chain conformations that are 5 residues long and described by 8 dihedral angles (2 for each res, minus the first phi and last psi angles?).  The PB description can be used for structural comparison.

    In this paper, conformation of homologues are compared, and it is found that the diversity of conformations has some dependency on the class.  They show that the knowledge of SCOP class-specific variations can improve performance of a PB based structure comparison tool.

    Use a special non-redundant dataset derived from SCOP: the PALI data set (1,922 domain families and 231,000 domain pairs aligned with MUSTANG).  Used the SCOP classification at the class level for their characterization.

    How SCOP is used:

    1. Do statistical analysis of protein block signatures to find correlations in different SCOP classes.

    2. Evaluate use of class-specific PB substitution matrices for structure alignment. Curate a non-redundant data set from PALI dataset by categorizing by SCOP superfamily and selecting 2 families from each superfamily, and finally 2 domains with <40% sequence identity from each family.

    SCOP reference:

    As the secondary structure content and topology varies between structural classes of proteins (as defined by SCOP [75]), we check whether there are class-specific specificities for changes in local pentapeptide conformations.

    ...

    Dataset

    The dataset of protein structure alignments used in the study is the recent version of PALI dataset V 2.8a [78,79,80]. It consists of 1,922 domain families comprising of 231,000 domain pairs aligned using MUSTANG [81]. The domains are classified based on SCOP definitions [75]. SCOP classifies domain structures into four major classes. All-a class consists of proteins with mainly a- helical content while all-b proteins are composed of mainly strand conformation. a/b contains both helical and strand conformations that are mixed in the structure, while they are segregated in the case of a+b class.

    ...

    Test Dataset for Alignments

    The gain in the quality of superposition (quantified as the difference in rmsd of superimposition) obtained using the class specific PB substitution matrices was checked on a smaller dataset. From each SCOP superfamily in the PALI dataset (with two or more families), two families were randomly chosen and from each of these families, a domain pair with sequence identity less than 40%, was chosen. It represents 1,050 domains (comprising of 188,760 residues) from 263 families.

    ...

    Class Specific PB Substitutions

    The distribution of domain structures in different SCOP classes is based on the secondary structure content and topology. As a result, the background distribution of PBs also varies between the SCOP classes. For instance, the all-a class has very low percentage of strand associated PBs while all-b has a low percentage of helix associated PBs (Figure S5).

    The PB substitution scores observed in the different SCOP classes were compared to the scores observed in the global distribution. The PB substitution patterns show variations across different SCOP classes. Clustering PBs based on the substitution patterns reflect different behaviours in each structural class.

    ...

    The preferences for the site of insertions, has variations across different SCOP classes. A few class specific preferences could be found for the all-a and all-b classes, especially for short inserts of length less than 4 (Table 2). Perhaps, many of the preferred sites for insertions/deletions are class-independent. b-turns and the C- capping region of a-helices are largely found as indel sites. These preferred sites are associated with loops that mediate the reversal in the direction of the backbone. Across the different SCOP classes, the two major PB bounds for insertions, are ‘h-i’ and ‘p-a’. The di-PB ‘p-a’ characterizes helix-helix and helix-strand transi- tions (Figures 8A and D). This local fold is characteristic of the C- cap motif of a-helices. Both short and long insertions are found associated with this site. In the all-b class, this site is preferred for single residue insertions with an association with beta turn of type I (Figure 8B). These di-PB ‘hi’ on the other hand, mainly characterizes region of strand-strand transitions (Figures 8B to 8D). Long insertions are found to occur at this site. The local structural region involving ‘hi’ is dominated by beta turn of type I’ (Figures 8B to 8D).

    ...

    Variations in the patterns of local structural changes are observed across different SCOP classes (Figure 5). Specific conformational changes are also preferred in certain SCOP classes (Figure 6). This is most evident in the case of all-b class, where the preferred local structure substitutions are found associated with short helical regions and b-turns. The preferred substitutions involving central helix PB m is rather unexpected. Short helices dominate the helical conformations found in the all-b class (Figure S7). About 69.2% of the PB m series occurring in this class are of length 3 or lesser. They are often seen in the region of transition between beta strands. Preferred substitutions with the PBs seen in the N-cap of strands (a & c), usually occur in such regions. Other structural elements associated with preferred local structural differences in the all- b class, are the b-hairpins. This local fold has a very high frequency of occurrence in the all-b class. It is interesting to see that the type IV b-turns are the predominant ones with class specific conformational changes. As they are uncharacterized, they encompass a wide range of conformations.

    Structural Alignment

    The knowledge on the substitution preferences observed in different SCOP classes could be utilized to improve structural comparisons based on PB sequence alignment [67,72,73]. PB based structural alignment method, iPBA, was shown to perform better than other established methods like DALI [92], MUS- TANG [81], VAST [93], CE [94] and GANGSTA+ [95]. About 82% of the alignments had better quality when compared to DALI in benchmark tests. Comparable performance could be observed with respect to TMALIGN [96] and FATCAT [97].

    ...

    Hot-spots for Insertions

    The relative frequency of occurrence of insertions is similar across different SCOP classes. The distribution of insertion of different lengths in the classes follows similar pattern (Figure S8). However, single residue insertions have a relatively low frequency in the all-b class. The preferred sites of insertions are highly specific in terms of local conformation. Though some class-specific insert sites are observed, the different SCOP classes share many

    insert sites. Helix C-caps and hairpin turns mainly constitute the sites favourable for occurrence of indels (Table 2).

  ### Attachments

  - [HTML] from plos.org
  - journal.pone.0038805.pdf
  - PubMed entry
- ## Low-Density Lipoprotein Receptor Gene Familial Hypercholesterolemia Variant Database: Update and Pathological Assessment

  |  |  |
  | --- | --- |
  | Type | Journal Article |
  | Author | Ebele Usifo |
  | Author | Sarah E. A. Leigh |
  | Author | Ros A. Whittall |
  | Author | Nicholas Lench |
  | Author | Alison Taylor |
  | Author | Corin Yeats |
  | Author | Christine A. Orengo |
  | Author | Andrew C. R. Martin |
  | Author | Jacopo Celli |
  | Author | Steve E. Humphries |
  | Volume | 76 |
  | Pages | 387–401 |
  | Publication | Annals of Human Genetics |
  | Date | September 2012 |
  | DOI | 10.1111/j.1469-1809.2012.00724.x |
  | Abstract | Familial hypercholesterolemia (FH) is caused predominately by variants in the low-density lipoprotein receptor gene (LDLR). We report here an update of the UCL LDLR variant database to include variants reported in the literature and in-house between 2008 and 2010, transfer of the database to LOVDv.2.0 platform (https://grenada.lumc.nl/LOVD2/UCL-Heart/home.php?select\_db=LDLR) and pathogenicity analysis. The database now contains over 1288 different variants reported in FH patients: 55% exonic substitutions, 22% exonic small rearrangements (<100 bp), 11% large rearrangements (>100 bp), 2% promoter variants, 10% intronic variants and 1 variant in the 3' untranslated sequence. The distribution and type of newly reported variants closely matches that of the 2008 database, and we have used these variants (n= 223) as a representative sample to assess the utility of standard open access software (PolyPhen, SIFT, refined SIFT, Neural Network Splice Site Prediction Tool, SplicePort and NetGene2) and additional analyses (Single Amino Acid Polymorphism database, analysis of conservation and structure and Mutation Taster) for pathogenicity prediction. In combination, these techniques have enabled us to assign with confidence pathogenic predictions to 8/8 in-frame small rearrangements and 8/9 missense substitutions with previously discordant results from PolyPhen and SIFT analysis. Overall, we conclude that 79% of the reported variants are likely to be disease causing. |
  | Date Added | 3/7/2014, 12:08:00 PM |
  | Modified | 3/7/2014, 12:08:00 PM |
- ## LUCApedia: a database for the study of ancient life

  |  |  |
  | --- | --- |
  | Type | Journal Article |
  | Author | Aaron David Goldman |
  | Author | Tess M. Bernhard |
  | Author | Egor Dolzhenko |
  | Author | Laura F. Landweber |
  | Volume | 41 |
  | Issue | D1 |
  | Pages | D1079-D1082 |
  | Publication | Nucleic Acids Research |
  | ISSN | 0305-1048 |
  | Date | JAN 2013 |
  | Extra | WOS:000312893300153 |
  | DOI | 10.1093/nar/gks1217 |
  | Abstract | Organisms represented by the root of the universal evolutionary tree were most likely complex cells with a sophisticated protein translation system and a DNA genome encoding hundreds of genes. The growth of bioinformatics data from taxonomically diverse organisms has made it possible to infer the likely properties of early life in greater detail. Here we present LUCApedia, (http://eeb.princeton.edu/lucapedia), a unified framework for simultaneously evaluating multiple data sets related to the Last Universal Common Ancestor (LUCA) and its predecessors. This unification is achieved by mapping eleven such data sets onto UniProt, KEGG and BioCyc IDs. LUCApedia may be used to rapidly acquire evidence that a certain gene or set of genes is ancient, to examine the early evolution of metabolic pathways, or to test specific hypotheses related to ancient life by corroborating them against the rest of the database. |
  | Date Added | 2/20/2014, 12:24:01 PM |
  | Modified | 2/20/2014, 12:24:01 PM |

  ### Notes:

  - Database for the study of the last universal common ancestor.

    How SCOP is used:

    Other.

    SCOP reference:

    Six of the early life data sets are derived from studies in which features of LUCA were inferred by surveying a taxonomically broad range of organisms for universal traits:

    ...

    Yang et al. (25)’. A phylogeny of 174 taxonomically diverse organisms was produced using a quantitative classification system based on protein domain content. The method identified 66 universal protein superfamilies (defined by SCOP) (26).

  ### Attachments

  - Nucl. Acids Res.-2013-Goldman-D1079-82.pdf
- ## LUD, a new protein domain associated with lactate utilization

  |  |  |
  | --- | --- |
  | Type | Journal Article |
  | Author | William C. Hwang |
  | Author | Constantina Bakolitsa |
  | Author | Marco Punta |
  | Author | Penelope C. Coggill |
  | Author | Alex Bateman |
  | Author | Herbert L. Axelrod |
  | Author | Neil D. Rawlings |
  | Author | Mayya Sedova |
  | Author | Scott N. Peterson |
  | Author | Ruth Y. Eberhardt |
  | Author | L. Aravind |
  | Author | Jaime Pascual |
  | Author | Adam Godzik |
  | Volume | 14 |
  | Pages | 341 |
  | Publication | Bmc Bioinformatics |
  | ISSN | 1471-2105 |
  | Date | NOV 26 2013 |
  | Extra | WOS:000327532700001 |
  | DOI | 10.1186/1471-2105-14-341 |
  | Abstract | Background: A novel highly conserved protein domain, DUF162 [Pfam: PF02589], can be mapped to two proteins: LutB and LutC. Both proteins are encoded by a highly conserved LutABC operon, which has been implicated in lactate utilization in bacteria. Based on our analysis of its sequence, structure, and recent experimental evidence reported by other groups, we hereby redefine DUF162 as the LUD domain family. Results: JCSG solved the first crystal structure [PDB:2G40] from the LUD domain family: LutC protein, encoded by ORF DR\_1909, of Deinococcus radiodurans. LutC shares features with domains in the functionally diverse ISOCOT superfamily. We have observed that the LUD domain has an increased abundance in the human gut microbiome. Conclusions: We propose a model for the substrate and cofactor binding and regulation in LUD domain. The significance of LUD-containing proteins in the human gut microbiome, and the implication of lactate metabolism in the radiation-resistance of Deinococcus radiodurans are discussed. |
  | Date Added | 2/12/2014, 2:18:08 PM |
  | Modified | 2/12/2014, 2:18:08 PM |

  ### Notes:

  - Present structure of LutC protein, and study function of LUD domain, which is associated with lactate utlization.

    How SCOP is used:

    Provide fold and superfamily classification of the LutC protein.

    SCOP reference:

    Structural alignment with other protein structures present in the Protein Data Bank, using the program DALI [13,14], suggests LutC protein is structurally akin to proteins found in the ISOCOT superfamily [15]. This is consistent with its classification in SCOP [16] as part of the NagB/RpiA/CoA transferase-like fold and super- family.

  ### Attachments

  - 1471-2105-14-341.pdf
- ## Lysine acetylation is a highly abundant and evolutionarily conserved modification in Escherichia coli

  |  |  |
  | --- | --- |
  | Type | Journal Article |
  | Author | Junmei Zhang |
  | Author | Robert Sprung |
  | Author | Jimin Pei |
  | Author | Xiaohong Tan |
  | Author | Sungchan Kim |
  | Author | Heng Zhu |
  | Author | Chuan-Fa Liu |
  | Author | Nick V Grishin |
  | Author | Yingming Zhao |
  | Volume | 8 |
  | Issue | 2 |
  | Pages | 215-225 |
  | Publication | Molecular & cellular proteomics: MCP |
  | ISSN | 1535-9484 |
  | Date | Feb 2009 |
  | Extra | PMID: 18723842 |
  | Journal Abbr | Mol. Cell Proteomics |
  | DOI | 10.1074/mcp.M800187-MCP200 |
  | Library Catalog | NCBI PubMed |
  | Language | eng |
  | Abstract | Lysine acetylation and its regulatory enzymes are known to have pivotal roles in mammalian cellular physiology. However, the extent and function of this modification in prokaryotic cells remain largely unexplored, thereby presenting a hurdle to further functional study of this modification in prokaryotic systems. Here we report the first global screening of lysine acetylation, identifying 138 modification sites in 91 proteins from Escherichia coli. None of the proteins has been previously associated with this modification. Among the identified proteins are transcriptional regulators, as well as others with diverse functions. Interestingly, more than 70% of the acetylated proteins are metabolic enzymes and translation regulators, suggesting an intimate link of this modification to energy metabolism. The new dataset suggests that lysine acetylation could be abundant in prokaryotic cells. In addition, these results also imply that functions of lysine acetylation beyond regulation of gene expression are evolutionarily conserved from bacteria to mammals. Furthermore, we demonstrate that bacterial lysine acetylation is regulated in response to stress stimuli. |
  | Date Added | 10/11/2013, 10:29:15 AM |
  | Modified | 10/11/2013, 10:29:15 AM |

  ### Tags:

  - ASTRAL
  - ASTRAL sequences
  - ASTRAL subsets

  ### Notes:

  - Lysine acetylation is a post-translational modification and is known to have an important role in mammalian cellular function.  Present research of lysine acetylation in prokaryotic cells, in particular in 91 proteins from e. coli that have modification sites.

    How SCOP is used:

    Used SCOP90 representative set from ASTRAL 1.71.

    Ran BLAST against the sequence database.  Collected all hits with e-values less than 10^-3.

    Mapped the position of acetylated lysines to the hit sequences and visually inspected structures to determine role of conserved lysine in binding or catalytic activity.

    SCOP reference:

    Structure Analysis of E. coli Lysine Acetylation Sites—For each acetylated E. coli protein, BLAST was run against a database of domain sequences with known structures from the SCOP90 representative set of ASTRAL compendium (version 1.71) (36, 37). Data- base size of BLAST was set to the size of the protein nr database as of April 6th, 2007 (39,280,211,952 letters) to impose a stringent E- value cutoff. Hits with an E-value less than 0.001 were analyzed. We identified homologous structures for 69 of the 91 acetylated proteins (⬚⬚76%). For these proteins, we mapped the positions of acetylated lysines to the model structures using BLAST local alignments and visually inspected the crystal structures to determine the role of the conserved lysine in substrate and protein binding or catalytic activity.

  ### Attachments

  - Mol Cell Proteomics-2009-Zhang-215-25.pdf
  - PubMed entry
- ## MACiE: exploring the diversity of biochemical reactions

  |  |  |
  | --- | --- |
  | Type | Journal Article |
  | Author | Gemma L. Holliday |
  | Author | Claudia Andreini |
  | Author | Julia D. Fischer |
  | Author | Syed Asad Rahman |
  | Author | Daniel E. Almonacid |
  | Author | Sophie T. Williams |
  | Author | William R. Pearson |
  | Volume | 40 |
  | Issue | D1 |
  | Pages | D783–D789 |
  | Publication | Nucleic Acids Research |
  | Date | January 2012 |
  | DOI | 10.1093/nar/gkr799 |
  | Abstract | MACiE (which stands for Mechanism, Annotation and Classification in Enzymes) is a database of enzyme reaction mechanisms, and can be accessed from http://www.ebi.ac.uk/thornton-srv/databases/MACiE/. This article presents the release of Version 3 of MACiE, which not only extends the dataset to 335 entries, covering 182 of the EC sub-subclasses with a crystal structure available (similar to 90%), but also incorporates greater chemical and structural detail. This version of MACiE represents a shift in emphasis for new entries, from non-homologous representatives covering EC reaction space to enzymes with mechanisms of interest to our users and collaborators with a view to exploring the chemical diversity of life. We present new tools for exploring the data in MACiE and comparing entries as well as new analyses of the data and new searches, many of which can now be accessed via dedicated Perl scripts. |
  | Date Added | 3/7/2014, 12:08:00 PM |
  | Modified | 3/7/2014, 12:08:00 PM |
- ## Macromolecular structure modeling from 3D EM using VolRover 2.0

  |  |  |
  | --- | --- |
  | Type | Journal Article |
  | Author | Qin Zhang |
  | Author | Radhakrishna Bettadapura |
  | Author | Chandrajit Bajaj |
  | Volume | 97 |
  | Issue | 9 |
  | Pages | 709-731 |
  | Publication | Biopolymers |
  | ISSN | 0006-3525 |
  | Date | SEP 2012 |
  | Extra | WOS:000305183000007 |
  | DOI | 10.1002/bip.22052 |
  | Abstract | We review tools for structure identification and model-based refinement from three-dimensional electron microscopy implemented in our in-house software package, VOLROVER 2.0. For viral density maps with icosahedral symmetry, we segment the capsid, polymeric, and monomeric subunits using techniques based on automatic symmetry detection and multidomain fast marching. For large biomolecules without symmetry information, we again use our multidomain fast-marching method with manual or fit-based multiseeding to segment meaningful substructures. In either case, we subject the resulting segmented subunit to secondary structure detection when the EM resolution is sufficiently high, and rigid-body structure fitting when the corresponding X-ray structure is available. Secondary structure elements are identified by three techniques: our earlier volume-based and boundary-based skeletonization methods as well as a new method, currently in development, based on solving the grassfire flow equation. For rigid-body fitting, we adapt our earlier fast Fourier-based correlation scheme F2Dock. Our reported segmentation, secondary structure elements identification, and rigid-body fitting techniques, implemented in VOLROVER 2.0 are applied to the PSB 2011 cryo-EM modeling challenge data, and our results are briefly compared to similar results submitted from other research groups. The comparisons show that our techniques are equally capable of segmenting relatively accurate subunits from a viral or protein assembly, and that high segmentation quality leads in turn to higher-quality results of secondary structure elements identification and correlation-based rigid-body fitting. (c) 2012 Wiley Periodicals, Inc. Biopolymers 97: 709731, 2012. |
  | Date Added | 2/13/2014, 4:13:17 PM |
  | Modified | 3/7/2014, 12:10:24 PM |
- ## Mapping small molecule binding data to structural domains

  |  |  |
  | --- | --- |
  | Type | Journal Article |
  | Author | Felix A Kruger |
  | Author | Raghd Rostom |
  | Author | John P Overington |
  | Volume | 13 Suppl 17 |
  | Pages | S11 |
  | Publication | BMC bioinformatics |
  | ISSN | 1471-2105 |
  | Date | 2012 |
  | Extra | PMID: 23282026 |
  | Journal Abbr | BMC Bioinformatics |
  | DOI | 10.1186/1471-2105-13-S17-S11 |
  | Library Catalog | NCBI PubMed |
  | Language | eng |
  | Abstract | BACKGROUND: Large-scale bioactivity/SAR Open Data has recently become available, and this has allowed new analyses and approaches to be developed to help address the productivity and translational gaps of current drug discovery. One of the current limitations of these data is the relative sparsity of reported interactions per protein target, and complexities in establishing clear relationships between bioactivity and targets using bioinformatics tools. We detail in this paper the indexing of targets by the structural domains that bind (or are likely to bind) the ligand within a full-length protein. Specifically, we present a simple heuristic to map small molecule binding to Pfam domains. This profiling can be applied to all proteins within a genome to give some indications of the potential pharmacological modulation and regulation of all proteins. RESULTS: In this implementation of our heuristic, ligand binding to protein targets from the ChEMBL database was mapped to structural domains as defined by profiles contained within the Pfam-A database. Our mapping suggests that the majority of assay targets within the current version of the ChEMBL database bind ligands through a small number of highly prevalent domains, and conversely the majority of Pfam domains sampled by our data play no currently established role in ligand binding. Validation studies, carried out firstly against Uniprot entries with expert binding-site annotation and secondly against entries in the wwPDB repository of crystallographic protein structures, demonstrate that our simple heuristic maps ligand binding to the correct domain in about 90 percent of all assessed cases. Using the mappings obtained with our heuristic, we have assembled ligand sets associated with each Pfam domain. CONCLUSIONS: Small molecule binding has been mapped to Pfam-A domains of protein targets in the ChEMBL bioactivity database. The result of this mapping is an enriched annotation of small molecule bioactivity data and a grouping of activity classes following the Pfam-A specifications of protein domains. This is valuable for data-focused approaches in drug discovery, for example when extrapolating potential targets of a small molecule with known activity against one or few targets, or in the assessment of a potential target for drug discovery or screening studies. |
  | Date Added | 10/11/2013, 10:29:15 AM |
  | Modified | 3/7/2014, 12:14:29 PM |

  ### Tags:

  - Binding Sites
  - Computational Biology
  - Drug Discovery
  - Humans
  - Ligands
  - Proteins
  - Protein Structure, Tertiary
  - Small Molecule Libraries
  - Structure-Activity Relationship

  ### Notes:

  - Present method for predicting a mapping for possible binding interactions between a database of small molecules and Pfam domains.

    How SCOP/CATH is used:

    Not using SCOP or CATH data.

    SCOP reference:

    Domain assignment information is available from a number of publicly available resources. SCOP [20] and CATH [21] are databases that define pro- tein architecture based on hierarchical definitions of 3D structural domains.

  ### Attachments

  - 1471-2105-13-S17-S11.pdf
- ## Mapping the Anopheles gambiae Odorant Binding Protein 1 (AgamOBP1) using modeling techniques, site directed mutagenesis, circular dichroism and ligand binding assays

  |  |  |
  | --- | --- |
  | Type | Journal Article |
  | Author | B. Rusconi |
  | Author | A. C. Maranhao |
  | Author | J. P. Fuhrer |
  | Author | P. Krotee |
  | Author | S. H. Choi |
  | Author | F. Grun |
  | Author | T. Thireou |
  | Author | S. D. Dimitratos |
  | Author | D. F. Woods |
  | Author | O. Marinotti |
  | Author | M. F. Walter |
  | Author | E. Eliopoulos |
  | Volume | 1824 |
  | Issue | 8 |
  | Pages | 947-953 |
  | Publication | Biochimica et Biophysica Acta (BBA)-Proteins and Proteomics |
  | ISSN | 1570-9639 |
  | Date | August 2012 |
  | DOI | 10.1016/j.bbapap.2012.04.011 |
  | Language | English |
  | Abstract | The major malaria vector in Sub-Saharan Africa is the Anopheles gambiae mosquito. This species is a key target of malaria control measures. Mosquitoes find humans primarily through olfaction, yet the molecular mechanisms associated with host-seeking behavior remain largely unknown. To further understand the functionality of A. gambiae odorant binding protein 1 (AgamOBP1), we combined in silico protein structure modeling and site-directed mutagenesis to generate 16 AgamOBP1 protein analogues containing single point mutations of interest. Circular dichroism (CD) and ligand-binding assays provided data necessary to probe the effects of the point mutations on ligand binding and the overall structure of AgamOBP1. Far-UV CD spectra of mutated AgamOBP1 variants displayed both substantial decreases to ordered alpha-helix structure ( up to22%) and increases to disordered alpha-helix structure(up to 15%) with only minimal changes in random coil (unordered) structure. In mutations Y54A, Y122A and W114Q aromatic side chain removal from the binding site significantly reduced N-phenyl-1-naphthylamine binding. Several non-aromatic mutations (L15T, L19T, L58T, L58Y, M84Q, M84K, H111A, Y122A and L124T) elicited changes to protein conformation with subsequent effects on ligand binding. This study provides empirical evidence for the in silico predicted functions of specific amino acids in AgamOBP1 folding and ligand binding characteristics. (C) 2012 Elsevier B.V. All rights reserved. |
  | Date Added | 10/11/2013, 10:29:15 AM |
  | Modified | 11/11/2013, 3:48:58 PM |

  ### Tags:

  - 3D modeling
  - Anopheles gambiae
  - Circular dichroism spectroscopy
  - Fluorescence spectroscopy
  - Odorant Binding Protein
  - Site directed mutagenesis

  ### Notes:

  - Experimental and computational studies of Anopheles gambiae Oderant Binding Protein 1, which is involved with the process mosquitoes use to find humans.

    How SCOP is used:

    Perform structure-based sequence alignment of a protein family to determine "topohydrophobic" residues.  Used the SCOP family for odorant binding proteins to gather domains for alignment.

    SCOP reference:

    TH residues of AgamOBP1 were determined following the structure-based sequence alignment of crystallographically determined structures of the insect pheromone/odorant-binding proteins SCOP family [17,18] (PDB code 2ERB, 3CZ1, 1OOH, 1OW4 and 1DQE ) using the Dali server [19] .

  ### Attachments

  - 1-s2.0-S1570963912000817-main.pdf
- ## Mapping the protein universe

  |  |  |
  | --- | --- |
  | Type | Journal Article |
  | Author | Liisa Holm |
  | Author | Chris Sander |
  | URL | http://www.sciencemag.org/content/273/5275/595.short |
  | Volume | 273 |
  | Issue | 5275 |
  | Pages | 595–602 |
  | Publication | Science |
  | Date | 1996 |
  | Accessed | 10/10/2013, 1:19:17 PM |
  | Library Catalog | Google Scholar |
  | Date Added | 10/11/2013, 10:29:15 AM |
  | Modified | 10/11/2013, 10:29:15 AM |

  ### Notes:

  - Article describing potential and algorithmic complexities of protein shape comparison.

    How SCOP data is used:

    Do not use SCOP data.

    Refer to SCOP as one of many databases that are applying structure techniques to map the protein landscape.

  ### Attachments

  - PubMed entry
  - Science-1996-Holm-595-602.pdf
  - Snapshot
- ## Mass spectrometry in the proteome analysis of mature cereal kernels

  |  |  |
  | --- | --- |
  | Type | Journal Article |
  | Author | Vincenzo Cunsolo |
  | Author | Vera Muccilli |
  | Author | Rosaria Saletti |
  | Author | Salvatore Foti |
  | Volume | 31 |
  | Issue | 4 |
  | Pages | 448-465 |
  | Publication | Mass Spectrometry Reviews |
  | ISSN | 0277-7037 |
  | Date | JUL-AUG 2012 |
  | Extra | WOS:000305388100002 |
  | DOI | 10.1002/mas.20347 |
  | Abstract | In the last decade, the improved performance and versatility of the mass spectrometers together with the increasing availability of gene and genomic sequence database, led the mass spectrometry to become an indispensable tool for either protein and proteome analyses in cereals. Mass spectrometric works on prolamins have rapidly evolved from the determination of the molecular masses of proteins to the proteomic approaches aimed to a large-scale protein identification and study of functional and regulatory aspects of proteins. Mass spectrometry coupled with electrophoresis, chromatographic methods, and bioinformatics tools is currently making significant contributions to a better knowledge of the composition and structure of the cereal proteins and their structurefunction relationships. Results obtained using mass spectrometry, including characterization of prolamins, investigation of the gluten toxicity for coeliac patients, identification of proteins responsible of cereal allergies, determination of the protein pattern and its modification under environmental or stress effects, investigation of genetically modified varieties by proteomic approaches, are summarized here, to illustrate current trends, analytical troubles and challenges, and suggest possible future perspectives. (c) 2011 Wiley Periodicals, Inc. Mass Spec Rev 31:448465, 2012 |
  | Date Added | 10/28/2013, 4:57:32 PM |
  | Modified | 10/28/2013, 4:57:32 PM |

  ### Notes:

  - Review of research using mass spectrometry on cereal proteins.

    How SCOP is used:

    Background on protein structure classification.  Use superfamily classifications for cereal proteins, but don't seem to have gotten these out of SCOP.

    SCOP reference:

    Families whose members have low sequence identities but whose structures and functional properties sug- gest a probable common evolutionary origin are placed togeth- er in superfamilies (Murzin et al., 1995; Lo Conte et al., 2002). C

  ### Attachments

  - 20347\_ftp.pdf
- ## Mdm10 is an ancient eukaryotic porin co-occurring with the ERMES complex

  |  |  |
  | --- | --- |
  | Type | Journal Article |
  | Author | Nadine Flinner |
  | Author | Lars Ellenrieder |
  | Author | Sebastian B. Stiller |
  | Author | Thomas Becker |
  | Author | Enrico Schleiff |
  | Author | Oliver Mirus |
  | Volume | 1833 |
  | Issue | 12 |
  | Pages | 3314-3325 |
  | Publication | Biochimica Et Biophysica Acta-Molecular Cell Research |
  | ISSN | 0167-4889; 0006-3002 |
  | Date | DEC 2013 |
  | Extra | WOS:000329596200074 |
  | DOI | 10.1016/j.bbamcr.2013.10.006 |
  | Abstract | Mitochondrial beta-barrel proteins fulfill central functions in the outer membrane like metabolite exchange catalyzed by the voltage-dependent anion channel (VDAC) and protein biogenesis by the central components of the preprotein translocase of the outer membrane (Tom40) or of the sorting and assembly machinery (Sam50). The mitochondrial division and morphology protein Mdm10 is another essential outer membrane protein with proposed beta-barrel fold, which has so far only been found in Fungi. Mdm10 is part of the endoplasmic reticulum mitochondria encounter structure (ERMES), which tethers the ER to mitochondria and associates with the SAM complex. In here, we provide evidence that Mdm10 phylogenetically belongs to the VDAC/Tom40 superfamily. Contrary to Tom40 and VDAC, Mdm10 exposes long loops towards both sides of the membrane. Analyses of single loop deletion mutants of Mdm10 in the yeast Saccharomyces cerevisiae reveal that the loops are dispensable for Mdm10 function. Sequences similar to fungal Mdm10 can be found in species from Excavates to Fungi, but neither in Metazoa nor in plants. Strikingly, the presence of Mdm10 coincides with the appearance of the other ERMES components. Mdm10's presence in both unikonts and bikonts indicates an introduction at an early time point in eukaryotic evolution. (C) 2013 Elsevier B.V. All rights reserved. |
  | Date Added | 2/12/2014, 2:18:08 PM |
  | Modified | 2/12/2014, 2:18:08 PM |

  ### Notes:

  - Experimental and computational study to Investigate what superfamily Mdm10 (an outer membrane mitochondrial division and morphology protein found in fungi) belongs to.

    How SCOP is used:

    Propose an extension to multiple sequence alignment that allows the alignment of divergent sequences of Mdm10 and two other outer-membrane proteins with related function (VDAC and Tom40).

    Evaluated their method by collecting protein domains from the same SCOP superfamily that belonged to different Pfam families (had high sequence divergence).

    SCOP reference:

    We evaluated the performance of our method by aligning various pairs of protein domains from the SCOP/ASTRAL database [82,83] (http://scop.berkeley.edu/) belonging to different PFAM families, which were selected to reflect the situation within the eukaryotic porin superfamily; i.e. we specifically searched for pairs of protein domains with known structures, which have the same fold but strongly divergent sequences (Supplementary Fig. S3, Table S4).

  ### Attachments

  - 1-s2.0-S0167488913003510-main.pdf
- ## Measuring and comparing structural fluctuation patterns in large protein datasets

  |  |  |
  | --- | --- |
  | Type | Journal Article |
  | Author | Edvin Fuglebakk |
  | Author | Julián Echave |
  | Author | Nathalie Reuter |
  | URL | http://bioinformatics.oxfordjournals.org/content/28/19/2431.short |
  | Volume | 28 |
  | Issue | 19 |
  | Pages | 2431–2440 |
  | Publication | Bioinformatics |
  | Date | 2012 |
  | Accessed | 9/20/2013, 1:19:56 PM |
  | Library Catalog | Google Scholar |
  | Date Added | 10/11/2013, 10:29:15 AM |
  | Modified | 3/7/2014, 12:14:59 PM |

  ### Tags:

  - ASTRAL
  - ASTRAL domain structures
  - ASTRAL sequences
  - ASTRAL subsets
  - Cite ASTRAL
  - Interesting

  ### Notes:

  - "comparative dynamics" study

    Study protein dynamics by measuring root-mean square fluctuations of aligned residues.  Study whether there is a correlation between conserved RMSF values and conserved residues in SCOP families.

    How SCOP is used:

    Benchmark method on four datasets derived from ASTRAL 1.75, filtered at 95% sequence identity.  Each dataset was taken from a different SCOP class.  Then ensured good superfamily and family representation by included domans for at least two different families for each superfamily, and that each family had at least 6 domains, for a talk dataset of 189 domains.

    References to SCOP:

    (From abstract:)

    We performed a systematic assessment of several scores that quantify the (dis)similarity between protein fluctuation patterns. We show that the best scores perform as well as or better than structural dissimilarity, as assessed by their consistency with the SCOP classification.

    2 METHODS

    2.1 Datasets

    We choose four datasets from the ASTRAL compendium (Chandonia et al., 2004) (version 1.75), one for each of the four main SCOP classes (Murzin et al., 1995). All domains were chosen from the subset of the ASTRAL compendium that has at most 95% sequence identity between domains. Each dataset is composed of protein domains that belong to two different superfamilies of the same fold. In addition, for each super- family we included domains from two different families. We made sure to make the selection so that all families are represented by at least 6 domains, for a total set of 189 domains.

    2.5 Assessment

    We assessed the performance of all the (dis)similarity scores studied, by evaluating their consistency with the SCOP classification at the superfam- ily level. SCOP is an expert classification-based mainly on visual inspec- tion of protein structures (Murzin et al., 1995). It is a hierarchical system with four levels: class, fold, superfamily and family. Fold-related proteins are structurally similar but not homologous, whereas superfamily and family-related proteins are, respectively, probably homologous and clearly homologous, as suggested by sequence or functional similarity.

    To quantify the performance of a given (dis)similarity score, we calcu- late the proportion of cases for which it ranks domains in agreement with SCOP. Consider a triplet of protein domains (d1, d2 and d3) with the first two being members of the same superfamily and the third of a different superfamily within the same fold. A given measure is consistent with SCOP for this triplet if the (dis)similarity between d1 and d2 is (smaller) larger than between d1 and d3.

  ### Attachments

  - Full Text PDF

    "comparative dynamics" study

    The study of conserved dynamics is a new area that may help to reveal more interesting remote evolutionary relationships than using structure similarity alone.

    Presents study of conservation of protein dynamics by measuring root-mean square fluctuations (RMSF) of aligned residues. Evaluated several RMSF-similarity and structural similarity score on 'consistency with SCOP classification'  Found that the best RMSF scores perform as well or better than structural similarity scores.

    How SCOP is used:

    Use type: benchmarking

    Levels used: fold, superfamily, domain

    Filtered on:

    Representative subset: Used ASTRAL 1.75 filtered at 95% sequence identity

    Description:

    Used SCOP to evaluate whether there is a correlation between

    Using ASTRAL data directly, from 1.75. Use <=95% ID rep sequences.  Filtered to only first four classes, then chose 2 SFs per fold, then chose 2 families per SF.  Resulted in a set of 189 domains.

    References to SCOP:

    From Abstract:

    We performed a systematic assessment of several scores that quantify the (dis)similarity between protein fluctuation patterns. We show that the best scores perform as well as or better than structural dissimilarity, as assessed by their consistency with the SCOP classification.

    2 METHODS

    2.1 Datasets

    We choose four datasets from the ASTRAL compendium (Chandonia et al., 2004) (version 1.75), one for each of the four main SCOP classes (Murzin et al., 1995). All domains were chosen from the subset of the ASTRAL compendium that has at most 95% sequence identity between domains. Each dataset is composed of protein domains that belong to two different superfamilies of the same fold. In addition, for each super- family we included domains from two different families. We made sure to make the selection so that all families are represented by at least 6 domains, for a total set of 189 domains.

    2.5 Assessment

    We assessed the performance of all the (dis)similarity scores studied, by evaluating their consistency with the SCOP classification at the superfam- ily level. SCOP is an expert classification-based mainly on visual inspec- tion of protein structures (Murzin et al., 1995). It is a hierarchical system with four levels: class, fold, superfamily and family. Fold-related proteins are structurally similar but not homologous, whereas superfamily and family-related proteins are, respectively, probably homologous and clearly homologous, as suggested by sequence or functional similarity.

    To quantify the performance of a given (dis)similarity score, we calcu- late the proportion of cases for which it ranks domains in agreement with SCOP. Consider a triplet of protein domains (d1, d2 and d3) with the first two being members of the same superfamily and the third of a different superfamily within the same fold. A given measure is consistent with SCOP for this triplet if the (dis)similarity between d1 and d2 is (smaller) larger than between d1 and d3.

    3 RESULTS

    We performed a comparative assessment of different scores of (dis)similarity of protein fluctuation patterns. To this end, and as described in Section 2, we

    1. (1)  chose four datasets, each of them including protein domains from 1-fold represented by two different SCOP superfamilies;
    2. (2)  obtained a multiple structural alignment of all proteins in each dataset from which we extract the conserved struc- tural core;
    3. (3)  produced pairwise superimpositions of all proteins within a dataset;
    4. (4)  calculated properties that characterise the fluctuations of the aligned core of each protein;
    5. (5)  quantified the similarity of such properties with different (dis)similarity measures and
    6. (6)  calculated the consistency of each measure with the SCOP classification.
  - supplementary\_information.doc
- ## Mechanisms Involved in the Functional Divergence of Duplicated GroEL Chaperonins in Myxococcus xanthus DK1622

  |  |  |
  | --- | --- |
  | Type | Journal Article |
  | Author | Yan Wang |
  | Author | Wen-yan Zhang |
  | Author | Zheng Zhang |
  | Author | Jian Li |
  | Author | Zhi-feng Li |
  | Author | Zai-gao Tan |
  | Author | Tian-tian Zhang |
  | Author | Zhi-hong Wu |
  | Author | Hong Liu |
  | Author | Yue-zhong Li |
  | Volume | 9 |
  | Issue | 2 |
  | Pages | e1003306 |
  | Publication | Plos Genetics |
  | Date | February 2013 |
  | DOI | 10.1371/journal.pgen.1003306 |
  | Abstract | The gene encoding the GroEL chaperonin is duplicated in nearly 30% of bacterial genomes; and although duplicated groEL genes have been comprehensively determined to have distinct physiological functions in different species, the mechanisms involved have not been characterized to date. Myxococcus xanthus DK1622 has two copies of the groEL gene, each of which can be deleted without affecting cell viability; however, the deletion of either gene does result in distinct defects in the cellular heat-shock response, predation, and development. In this study, we show that, from the expression levels of different groELs, the distinct functions of groEL1 and groEL2 in predation and development are probably the result of the substrate selectivity of the paralogous GroEL chaperonins, whereas the lethal effect of heat shock due to the deletion of groEL1 is caused by a decrease in the total groEL expression level. Following a bioinformatics analysis of the composition characteristics of GroELs from different bacteria, we performed region-swapping assays in M. xanthus, demonstrating that the differences in the apical and the C-terminal equatorial regions determine the substrate specificity of the two GroELs. Site-directed mutagenesis experiments indicated that the GGM repeat sequence at the C-terminus of GroEL1 plays an important role in functional divergence. Divergent functions of duplicated GroELs, which have similar patterns of variation in different bacterial species, have thus evolved mainly via alteration of the apical and the C-terminal equatorial regions. We identified the specific substrates of strain DK1622's GroEL1 and GroEL2 using immunoprecipitation and mass spectrometry techniques. Although 68 proteins bound to both GroEL1 and GroEL2, 83 and 46 proteins bound exclusively to GroEL1 or GroEL2, respectively. The GroEL-specific substrates exhibited distinct molecular sizes and secondary structures, providing an encouraging indication for GroEL evolution for functional divergence. |
  | Date Added | 3/7/2014, 12:08:00 PM |
  | Modified | 3/7/2014, 12:08:00 PM |
- ## Mechanisms of Protein Sequence Divergence and Incompatibility

  |  |  |
  | --- | --- |
  | Type | Journal Article |
  | Author | Alon Wellner |
  | Author | Maria Raitses Gurevich |
  | Author | Dan S. Tawfik |
  | Volume | 9 |
  | Issue | 7 |
  | Publication | Plos Genetics |
  | ISSN | 1553-7404 |
  | Date | JUL 2013 |
  | Extra | WOS:000322321100056 |
  | DOI | 10.1371/journal.pgen.1003665 |
  | Abstract | Alignments of orthologous protein sequences convey a complex picture. Some positions are utterly conserved whilst others have diverged to variable degrees. Amongst the latter, many are non-exchangeable between extant sequences. How do functionally critical and highly conserved residues diverge? Why and how did these exchanges become incompatible within contemporary sequences? Our model is phosphoglycerate kinase (PGK), where lysine 219 is an essential active-site residue completely conserved throughout Eukaryota and Bacteria, and serine is found only in archaeal PGKs. Contemporary sequences tested exhibited complete loss of function upon exchanges at 219. However, a directed evolution experiment revealed that two mutations were sufficient for human PGK to become functional with serine at position 219. These two mutations made position 219 permissive not only for serine and lysine, but also to a range of other amino acids seen in archaeal PGKs. The identified trajectories that enabled exchanges at 219 show marked sign epistasis - a relatively small loss of function with respect to one amino acid (lysine) versus a large gain with another (serine, and other amino acids). Our findings support the view that, as theoretically described, the trajectories underlining the divergence of critical positions are dominated by sign epistatic interactions. Such trajectories are an outcome of rare mutational combinations. Nonetheless, as suggested by the laboratory enabled K219S exchange, given enough time and variability in selection levels, even utterly conserved and functionally essential residues may change. |
  | Date Added | 10/28/2013, 4:53:08 PM |
  | Modified | 10/28/2013, 4:53:08 PM |

  ### Notes:

  - Study evolution of a protein of interest: phosphoglycerate kinase  (PGK).

    How SCOP is used:

    Collect a structure from the same fold, but different family from studied family, to serve as the "outgroup" for phylogenetic analysis

    SCOP reference:

    Phylogenetic analysis

    PGK sequences were collected by combining three BLAST searches using human, E. coli and M. mazei PGK sequences as queries. Each of the three BLAST searches contained sequences from all three kingdoms of life, suggesting that the searches were exhaustive. **Glycerate kinase from** **Neisseria meningitides** **was structurally aligned with the PGK sequences to serve as an outgroup, in agreement with the protein fold classification [39].**

  ### Attachments

  - journal.pgen.1003665.pdf
- ## Membrane protein structural bioinformatics

  |  |  |
  | --- | --- |
  | Type | Journal Article |
  | Author | Timothy Nugent |
  | Author | David T. Jones |
  | Volume | 179 |
  | Issue | 3 |
  | Pages | 327–337 |
  | Publication | Journal of Structural Biology |
  | Date | September 2012 |
  | DOI | 10.1016/j.jsb.2011.10.008 |
  | Abstract | Despite the increasing number of recently solved membrane protein strictures, coverage of membrane protein fold space remains relatively sparse. This necessitates the use of computational strategies to investigate membrane protein structure, allowing us to further our understanding of how membrane proteins carry out their diverse range of functions, while aiding the development of novel predictive tools with which to probe uncharacterised folds. Analysis of known structures, the application of machine learning techniques, molecular dynamics simulations and protein structure prediction have enabled significant advances to be made in the field of membrane protein research. In this communication, the key bioinformatic methods that allow the characterisation of membrane proteins are reviewed, the tools available for the structural analysis of membrane proteins are presented and the contribution these tools have made to expanding our understanding of membrane protein structure, function and stability is discussed. (C) 2011 Elsevier Inc. All rights reserved. |
  | Date Added | 3/7/2014, 12:08:00 PM |
  | Modified | 3/7/2014, 12:08:00 PM |
- ## Membrane Topology and Predicted RNA-Binding Function of the 'Early Responsive to Dehydration (ERD4)' Plant Protein

  |  |  |
  | --- | --- |
  | Type | Journal Article |
  | Author | Archana Rai |
  | Author | Penna Suprasanna |
  | Author | Stanislaus F. D'Souza |
  | Author | Vinay Kumar |
  | Volume | 7 |
  | Issue | 3 |
  | Pages | e32658 |
  | Publication | Plos One |
  | ISSN | 1932-6203 |
  | Date | MAR 14 2012 |
  | Extra | WOS:000303198600022 |
  | DOI | 10.1371/journal.pone.0032658 |
  | Abstract | Functional annotation of uncharacterized genes is the main focus of computational methods in the post genomic era. These tools search for similarity between proteins on the premise that those sharing sequence or structural motifs usually perform related functions, and are thus particularly useful for membrane proteins. Early responsive to dehydration (ERD) genes are rapidly induced in response to dehydration stress in a variety of plant species. In the present work we characterized function of Brassica juncea ERD4 gene using computational approaches. The ERD4 protein of unknown function possesses ubiquitous DUF221 domain (residues 312-634) and is conserved in all plant species. We suggest that the protein is localized in chloroplast membrane with at least nine transmembrane helices. We detected a globular domain of 165 amino acid residues (183-347) in plant ERD4 proteins and expect this to be posited inside the chloroplast. The structural-functional annotation of the globular domain was arrived at using fold recognition methods, which suggested in its sequence presence of two tandem RNA-recognition motif (RRM) domains each folded into beta alpha beta beta alpha beta topology. The structure based sequence alignment with the known RNA-binding proteins revealed conservation of two non-canonical ribonucleoprotein sub-motifs in both the putative RNA-recognition domains of the ERD4 protein. The function of highly conserved ERD4 protein may thus be associated with its RNA-binding ability during the stress response. This is the first functional annotation of ERD4 family of proteins that can be useful in designing experiments to unravel crucial aspects of stress tolerance mechanism. |
  | Date Added | 2/20/2014, 12:24:01 PM |
  | Modified | 2/20/2014, 12:24:01 PM |

  ### Notes:

  - Computational investigation of the function of the Brassica juncea ERD4 protein using a combination of advanced sequence profile searches and structure prediction bioinformatics approaches like fold recogni- tion and comparative modeling.

    How SCOP is used:

    Annotate a non-SCOP data set with SCOP classification.

    SCOP reference:

    The SCOP superfamily classification (DNA-binding protein) is listed for each of the 5 chains.

  ### Attachments

  - journal.pone.0032658.pdf
- ## MESSA: MEta-server for protein sequence analysis

  |  |  |
  | --- | --- |
  | Type | Journal Article |
  | Author | Qian Cong |
  | Author | Nick V. Grishin |
  | URL | http://www.biomedcentral.com/1741-7007/10/82/ |
  | Volume | 10 |
  | Issue | 1 |
  | Pages | 82 |
  | Publication | BMC biology |
  | Date | 2012 |
  | Accessed | 9/20/2013, 1:19:35 PM |
  | Library Catalog | Google Scholar |
  | Short Title | MESSA |
  | Date Added | 10/11/2013, 10:29:15 AM |
  | Modified | 10/11/2013, 10:29:15 AM |

  ### Notes:

  - MESSA is a web server that analyzes amino acid sequence to predict the structure and function of the protein. It also detects homology and assigns a family. Also includes manual curation.

    How SCOP is used:

    SCOP is referenced as one their outputs (Section VII) as a resource that shows the homologous protein in the database that was inputted (detected through BLAST, RPS -BLAST, and HHpred). Also notes where in the SCOP hierarchy this protein is found.

    SCOP Reference:

    This sec- tion shows homologous structures in the Protein Data Bank (PDB) [38] and structure domains in the Structure Classification Of Protein (SCOP) database [39] detected by BLAST (e-value below 0.001), RPS-BLAST (e-value below 0.001) and HHpred server (probability higher than 80%). For each detected protein and protein domain, the alignment and the corresponding structure displayed by Jmol [40]) can be retrieved. The conservation of protein structures among homologs allows these structures, in most cases, to represent the general fold of the query protein and to be suitable templates for structure model- ing. For structure domains detected in SCOP, we provide their classification hierarchy to highlight the evolutionary history and suggest similarities to other proteins.

    Seventh, to detect evolutionarily related proteins with available three-dimensional structures and reveal domain architectures, we use three protocols: first, BLAST against PDB (e-value cut-off: 0.001); second, RPS-BLAST (e-value cut-off: 0.01); and third, HHpred server (prob- ability cut-off: 80%) against the 70% sequence identity representatives of all PDB and SCOP (version 1.75) entries.

  ### Attachments

  - 1741-7007-10-82.pdf
  - [HTML] from biomedcentral.com
- ## Meta-Analysis of General Bacterial Subclades in Whole-Genome Phylogenies Using Tree Topology Profiling

  |  |  |
  | --- | --- |
  | Type | Journal Article |
  | Author | Thomas Meinel |
  | Author | Antje Krause |
  | Volume | 8 |
  | Pages | 489-525 |
  | Publication | Evolutionary Bioinformatics |
  | ISSN | 1176-9343 |
  | Date | 2012 |
  | Extra | WOS:000308500000001 |
  | DOI | 10.4137/EBO.S9642 |
  | Abstract | In the last two decades, a large number of whole-genome phylogenies have been inferred to reconstruct the Tree of Life (ToL). Underlying data models range from gene or functionality content in species to phylogenetic gene family trees and multiple sequence alignments of concatenated protein sequences. Diversity in data models together with the use of different tree reconstruction techniques, disruptive biological effects and the steadily increasing number of genomes have led to a huge diversity in published phylogenies. Comparison of those and, moreover, identification of the impact of inference properties (underlying data model, inference technique) on particular reconstructions is almost impossible. In this work, we introduce tree topology profiling as a method to compare already published whole-genome phylogenies. This method requires visual determination of the particular topology in a drawn whole-genome phylogeny for a set of particular bacterial clans. For each clan, neighborhoods to other bacteria are collected into a catalogue of generalized alternative topologies. Particular topology alternatives found for an ordered list of bacterial clans reveal a topology profile that represents the analyzed phylogeny. To simulate the inhomogeneity of published gene content phylogenies we generate a set of seven phylogenies using different inference techniques and the SYSTERS-PhyloMatrix data model. After tree topology profiling on in total 54 selected published and newly inferred phylogenies, we separate artefactual from biologically meaningful phylogenies and associate particular inference results (phylogenies) with inference background (inference techniques as well as data models). Topological relationships of particular bacterial species groups are presented. With this work we introduce tree topology profiling into the scientific field of comparative phylogenomics. |
  | Date Added | 10/28/2013, 4:53:08 PM |
  | Modified | 10/28/2013, 4:53:08 PM |

  ### Notes:

  - Present a method for comparing phylogenetic tree topologies.

    How SCOP is used:

    SCOP data are not used directly in this paper.  SCOP data has been used to curate some of the trees of life that were compared.

    SCOP reference:

    See table 1.

  ### Attachments

  - f\_3303-EBO-Meta-Analysis-of-General-Bacterial-Subclades-in-Whole-Genome-Phylogeni.pdf\_4469.pdf
- ## MetaBase-the wiki-database of biological databases

  |  |  |
  | --- | --- |
  | Type | Journal Article |
  | Author | Dan M. Bolser |
  | Author | Pierre-Yves Chibon |
  | Author | Nicolas Palopoli |
  | Author | Sungsam Gong |
  | Author | Daniel Jacob |
  | Author | Victoria Dominguez Del Angel |
  | Author | Dan Swan |
  | Author | Sebastian Bassi |
  | Author | Virginia Gonzalez |
  | Author | Prashanth Suravajhala |
  | Author | Seungwoo Hwang |
  | Author | Paolo Romano |
  | Author | Rob Edwards |
  | Author | Bryan Bishop |
  | Author | John Eargle |
  | Author | Timur Shtatland |
  | Author | Nicholas J. Provart |
  | Author | Dave Clements |
  | Author | Daniel P. Renfro |
  | Author | Daeui Bhak |
  | Author | Jong Bhak |
  | Volume | 40 |
  | Issue | D1 |
  | Pages | D1250-D1254 |
  | Publication | Nucleic Acids Research |
  | ISSN | 0305-1048 |
  | Date | JAN 2012 |
  | Extra | WOS:000298601300187 |
  | DOI | 10.1093/nar/gkr1099 |
  | Abstract | Biology is generating more data than ever. As a result, there is an ever increasing number of publicly available databases that analyse, integrate and summarize the available data, providing an invaluable resource for the biological community. As this trend continues, there is a pressing need to organize, catalogue and rate these resources, so that the information they contain can be most effectively exploited. MetaBase (MB) (http://MetaDatabase.Org) is a community-curated database containing more than 2000 commonly used biological databases. Each entry is structured using templates and can carry various user comments and annotations. Entries can be searched, listed, browsed or queried. The database was created using the same MediaWiki technology that powers Wikipedia, allowing users to contribute on many different levels. The initial release of MB was derived from the content of the 2007 Nucleic Acids Research (NAR) Database Issue. Since then, approximately 100 databases have been manually collected from the literature, and users have added information for over 240 databases. MB is synchronized annually with the static Molecular Biology Database Collection provided by NAR. To date, there have been 19 significant contributors to the project; each one is listed as an author here to highlight the community aspect of the project. |
  | Date Added | 2/20/2014, 12:24:01 PM |
  | Modified | 3/7/2014, 12:15:24 PM |

  ### Notes:

  - Present a database of databases.

    How SCOP is used:

    Background on protein structure classification.

    SCOP reference:

    When discussing biological databases, there are simply too many different resources to comprehensively cover the topic in a short introduction...

    There are classification databases (13,14),

  ### Attachments

  - Nucl. Acids Res.-2012-Bolser-D1250-4.pdf
- ## Metal binding properties and structure of a type III metallothionein from the metal hyperaccumulator plant Noccaea caerulescens

  |  |  |
  | --- | --- |
  | Type | Journal Article |
  | Author | Lucia Rubio Fernandez |
  | Author | Guy Vandenbussche |
  | Author | Nancy Roosens |
  | Author | Cedric Govaerts |
  | Author | Erik Goormaghtigh |
  | Author | Nathalie Verbruggen |
  | Volume | 1824 |
  | Issue | 9 |
  | Pages | 1016-1023 |
  | Publication | Biochimica Et Biophysica Acta-Proteins and Proteomics |
  | ISSN | 1570-9639 |
  | Date | SEP 2012 |
  | Extra | WOS:000307369500002 |
  | DOI | 10.1016/j.bbapap.2012.05.010 |
  | Abstract | Metallothioneins (MT) are low molecular weight proteins with cysteine-rich sequences that bind heavy metals with remarkably high affinities. Plant MTs differ from animal ones by a peculiar amino acid sequence organization consisting of two short Cys-rich terminal domains (containing from 4 to 8 Cys each) linked by a Cys free region of about |
  | Date Added | 2/13/2014, 4:13:17 PM |
  | Modified | 3/7/2014, 12:15:35 PM |

  ### Notes:

  - Experimental study of a Metallothioen (MT) protein.

    How SCOP/CATH is used:

    Annotate a non-SCOP non-CATH data set with SCOP and CATH classification in order to get good coverage of structural folds.

    SCOP reference:

    An analysis of the secondary structure was carried out as described in [39,40]. Results are reported in Table 1. This analysis is in agreement with a largely disordered protein. In another attempt we compared the spectrum of NcMT3 with an ATR-FTIR protein spectra present in a FTIR database [41] optimized to cover as well as possible the different struc- tural folds as described by CATH and SCOP [41,42]. A hierarchical clus- tering (not shown) indicated that the closest spectrum in the entire database was the spectrum of a rabbit metallothionein II (Swiss-Prot MT2A\_RABIT, PDB 4mt2) introduced into the database to represent a fully unstructured protein.

  ### Attachments

  - 1-s2.0-S1570963912000994-main.pdf
- ## MetalPDB: a database of metal sites in biological macromolecular structures

  |  |  |
  | --- | --- |
  | Type | Journal Article |
  | Author | Claudia Andreini |
  | Author | Gabriele Cavallaro |
  | Author | Serena Lorenzini |
  | Author | Antonio Rosato |
  | Volume | 41 |
  | Issue | D1 |
  | Pages | D312-D319 |
  | Publication | Nucleic Acids Research |
  | ISSN | 0305-1048 |
  | Date | JAN 2013 |
  | Extra | WOS:000312893300044 |
  | DOI | 10.1093/nar/gks1063 |
  | Abstract | We present here MetalPDB (freely accessible at http://metalweb.cerm.unifi.it), a novel resource aimed at conveying the information available on the three-dimensional (3D) structures of metal-binding biological macromolecules in a consistent and effective manner. This is achieved through the systematic and automated representation of metal-binding sites in proteins and nucleic acids by way of Minimal Functional Sites (MFSs). MFSs are 3D templates that describe the local environment around the metal(s) independently of the larger context of the macromolecular structure embedding the site(s), and are the central objects of MetalPDB design. MFSs are grouped into equistructural (broadly defined as sites found in corresponding positions in similar structures) and equivalent sites (equistructural sites that contain the same metals), allowing users to easily analyse similarities and variations in metal-macromolecule interactions, and to link them to functional information. The web interface of MetalPDB allows access to a comprehensive overview of metal-containing biological structures, providing a basis to investigate the basic principles governing the properties of these systems. MetalPDB is updated monthly in an automated manner. |
  | Date Added | 2/20/2014, 12:24:01 PM |
  | Modified | 3/7/2014, 12:10:22 PM |

  ### Notes:

  - Present the MetalPDB database of metal-binding sites in biological macromolecules.

    How SCOP/CATH is used:

    Annotate every PDB structure in the database with the SCOP sccs, as well as CATH, Pfam, and Uniprot classification.

    Then group MFSs using CATH, SCOP, or Pfam classification.

    SCOP/CATH reference:

    (6) For each protein chain in a PDB structure, identify the 50% sequence identity group in the PDB, the EC number, if relevant, as well as the UniProt (http://www.uniprot.org/) (15), CATH (http://www .cathdb.info/) (16), SCOP (http://scop.mrc-lmb.cam .ac.uk/scop/) (17) and Pfam (http://pfam.sanger.ac .uk/) (18) codes. Each MFS is then associated with the CATH, SCOP and Pfam code(s) of the protein domain(s) that contain the ligands.

    (7) Group MFSs into sets of ‘equivalent’ and ‘equistructural’ MFSs. Two MFSs are defined to be ‘equivalent’ when they satisfy the following condi- tions: (i) they have the same CATH, SCOP or Pfam classification; alternatively, the sequence identity between the two PDB chains that contain them is ⬚⬚50% (effectively meaning that the two chains have the same fold (19)); (ii) after structural superposition of the PDB chains containing them, the two MFSs are superimposed (i.e. the distance between their geometric centers is <3.5 A ̊ ); and (iii) after structural superposition of the PDB chains con- taining them, the two MFSs have the same metal elements in the same positions. For the latter condi- tion to be fulfilled, equivalent sites must have the same nuclearity. Two MFSs are defined to be ‘equistructural’ when they satisfy conditions (i) and (ii) above, while condition (iii) does not need to be fulfilled. This implies that two equivalent sites are also equistructural, but the converse is not necessar- ily true. All equivalent and equistructural MFSs are grouped into clusters of equivalent and equistructural MFSs, respectively, by using a single linkage clustering strategy. For each group of equiva- lent MFSs, a representative MFS is chosen by select- ing the PDB structure with the highest resolution. The present step is applied to metalloproteins only as CATH, SCOP and Pfam classifications are not available for nucleic acids. Hence, no equivalent or equistructural site is defined for nucleic acids.

  ### Attachments

  - Nucl. Acids Res.-2013-Andreini-D312-9.pdf
- ## MetalS(2): A Tool for the Structural Alignment of Minimal Functional Sites in Metal-Binding Proteins and Nucleic Acids

  |  |  |
  | --- | --- |
  | Type | Journal Article |
  | Author | Claudia Andreini |
  | Author | Gabriele Cavallaro |
  | Author | Antonio Rosato |
  | Author | Yana Valasatava |
  | Volume | 53 |
  | Issue | 11 |
  | Pages | 3064-3075 |
  | Publication | Journal of Chemical Information and Modeling |
  | ISSN | 1549-9596; 1549-960X |
  | Date | NOV 2013 |
  | Extra | WOS:000327747200026 |
  | DOI | 10.1021/ci400459w |
  | Abstract | We developed a new software tool, MetalS(2), for the structural alignment of Minimal Functional Sites (MFSs) in metal-binding biological macromolecules. MFSs are 3D templates that describe the local environment around the metal(s) independently of the larger context of the macromolecular structure. Such local environment has a determinant role in tuning the chemical reactivity of the metal, ultimately contributing to the functional properties of the whole system. On our example data sets, MetalS2 unveiled structural similarities that other programs for protein structure comparison do not consistently point out and overall identified a larger number of structurally similar MFSs. MetalS2 supports the comparison of MFSs harboring different metals and/or with different nuclearity and is available both as a stand-alone program and a Web tool (http://metalweb.cerm.unifi.it/tools/metals2/). |
  | Date Added | 2/20/2014, 12:24:01 PM |
  | Modified | 3/7/2014, 12:10:20 PM |

  ### Notes:

  - Present a method, MetalS2, for metal binding site alignment.

    How SCOP/CATH are used:

    Use  SCOP/CATH superfamily to remove redundancy in metal-binding data sets.

    SCOP/CATH reference:

    The Fe-data set contains 86 proteins; its relatively small size allowed us to manually analyze the results. The Zn-data set contains 367 proteins, resulting in 67161 pairwise comparisons, which constitute a large enough basis for statistical analysis. **Both data sets are nonredundant, i.e. for all proteins belonging to the same SCOP****32 or CATH33** **superfamily only one representative was kept.** In this way, we minimized the number of homologous proteins in the data set, whose structures are expected to be very similar34 and thus would result, if included, in a less stringent testing of the program.

    ...

    Figure 4. An example of functionally relevant MFS alignments. 1dmh is a catechol dioxygenase; 2b5h is a cysteine dioxygenase; 2fiy is a protein of unknown function. Fold classification according to three different databases is reported in the CATH, SCOP, and Pfam columns. The EC column specifies the Enzyme Commission classification, where known. Site names in the first column correspond to those adopted in the MetalPDB database.21

  ### Attachments

  - ci400459w.pdf
- ## MICAN : a protein structure alignment algorithm that can handle Multiple-chains, Inverse alignments, C-alpha only models, Alternative alignments, and Non-sequential alignments

  |  |  |
  | --- | --- |
  | Type | Journal Article |
  | Author | Shintaro Minami |
  | Author | Kengo Sawada |
  | Author | George Chikenji |
  | Volume | 14 |
  | Publication | BMC bioinformatics |
  | ISSN | 1471-2105 |
  | Date | JAN 18 2013 |
  | DOI | 10.1186/1471-2105-14-24 |
  | Language | English |
  | Abstract | Background: Protein pairs that have the same secondary structure packing arrangement but have different topologies have attracted much attention in terms of both evolution and physical chemistry of protein structures. Further investigation of such protein relationships would give us a hint as to how proteins can change their fold in the course of evolution, as well as a insight into physico-chemical properties of secondary structure packing. For this purpose, highly accurate sequence order independent structure comparison methods are needed. Results: We have developed a novel protein structure alignment algorithm, MICAN (a structure alignment algorithm that can handle Multiple-chain complexes, Inverse direction of secondary structures, C-alpha only models, Alternative alignments, and Non-sequential alignments). The algorithm was designed so as to identify the best structural alignment between protein pairs by disregarding the connectivity between secondary structure elements (SSE). One of the key feature of the algorithm is utilizing the multiple vector representation for each SSE, which enables us to correctly treat bent or twisted nature of long SSE. We compared MICAN with other 9 publicly available structure alignment programs, using both reference-dependent and reference-independent evaluation methods on a variety of benchmark test sets which include both sequential and non-sequential alignments. We show that MICAN outperforms the other existing methods for reproducing reference alignments of non-sequential test sets. Further, although MICAN does not specialize in sequential structure alignment, it showed the top level performance on the sequential test sets. We also show that MICAN program is the fastest non-sequential structure alignment program among all the programs we examined here. Conclusions: MICAN is the fastest and the most accurate program among non-sequential alignment programs we examined here. These results suggest that MICAN is a highly effective tool for automatically detecting non-trivial structural relationships of proteins, such as circular permutations and segment-swapping, many of which have been identified manually by human experts so far. The source code of MICAN is freely download-able at http://www.tbp.cse.nagoya-u.ac.jp/MICAN. |
  | Date Added | 10/11/2013, 10:29:15 AM |
  | Modified | 12/2/2013, 4:19:24 PM |

  ### Notes:

  - Present MICAN protein structure alignment method.

    Applied to examples with non-sequential structural similarity, which means that structurally equivalent regions occur in a different order in the sequence of the compared proteins.

    How SCOP is used:

    Use type: calculate statistics on SCOP  
       
     Application: non-sequential structure similarity  
       
     Filtered on:   N/A  
     Filtering type:  N/A  
       
     Benchmarking type:  
     Levels used in analysis: family, superfamily  
     Representative set: All pairs of SCOP family representatives within the same superfamily (12,603 pairs).

    Description: Determine that 39% of pairs in structurally similar pairs data set derived from SCOP have non-sequential alignments.

    SCOP reference:

    Another example is a pair of Carboxylic esterase (PDB entry 1YAS) and Hydroxynitrile lyase (PDB entry 1QLW) [10]. Both belong to the same superfam- ily (alpha/beta-Hydrolases superfamily) defined in SCOP database [12], suggesting that they share a common evo- lutionary ancestor.

    ...

    Homologous protein pairs that have non-sequential structural similarity  
     Recently, some homologous protein pairs that show non- sequential structure similarity have been reported [10,11].

    However, it is unclear how abundant such pairs are. In order to address the issue, we compared homologous pro- tein structures by MICAN. We considered all the pairs of SCOP family representatives within the same superfamily (12,603 pairs). For these 12,603 pairs, pairwise structural alignments were generated using MICAN. Here, only the alignments with significantly high structural similarity, TM-score ≥ 0.5 [42], were collected, resulting in total of 8,335 structurally similar protein pairs. Among them, the overall proportion of protein pairs that show non- sequential alignments was 39% (3,284 pairs).

  ### Attachments

  - 1471-2105-14-24.pdf
- ## Microsecond folding experiments and simulations: a match is made

  |  |  |
  | --- | --- |
  | Type | Journal Article |
  | Author | M. B. Prigozhin |
  | Author | M. Gruebele |
  | URL | http://pubs.rsc.org/en/content/articlehtml/2013/cp/c3cp43992e |
  | Volume | 15 |
  | Issue | 10 |
  | Pages | 3372–3388 |
  | Publication | Physical Chemistry Chemical Physics |
  | Date | 2013 |
  | Accessed | 9/23/2013, 10:03:53 AM |
  | Library Catalog | Google Scholar |
  | Short Title | Microsecond folding experiments and simulations |
  | Date Added | 10/11/2013, 10:29:15 AM |
  | Modified | 10/11/2013, 10:29:15 AM |

  ### Notes:

  - Survey paper on microsecond fold experiments and simulations.

    How SCOP is used:

    Provide background on protein structure classification.

    SCOP reference:

    Many folds are intuitively related, even if they differ in quanti- tative detail, and structural classes and families have been identified.134

  ### Attachments

  - C3CP43992E.pdf
- ## Mimicking the action of folding chaperones by Hamiltonian replica-exchange molecular dynamics simulations: Application in the refinement of de novo models

  |  |  |
  | --- | --- |
  | Type | Journal Article |
  | Author | Hao Fan |
  | Author | Xavier Periole |
  | Author | Alan E. Mark |
  | URL | http://onlinelibrary.wiley.com/doi/10.1002/prot.24068/full |
  | Volume | 80 |
  | Issue | 7 |
  | Pages | 1744–1754 |
  | Publication | Proteins: Structure, Function, and Bioinformatics |
  | Date | 2012 |
  | Accessed | 9/20/2013, 1:17:33 PM |
  | Library Catalog | Google Scholar |
  | Short Title | Mimicking the action of folding chaperones by Hamiltonian replica-exchange molecular dynamics simulations |
  | Date Added | 10/11/2013, 10:29:15 AM |
  | Modified | 10/11/2013, 10:29:15 AM |

  ### Tags:

  - chaperone
  - protein structure prediction
  - protein structure refinement
  - replica-exchange molecular dynamics
  - statistical potential

  ### Notes:

  - Ran molecular dynamics experiments on a test set of 15 proteins that had been used in a previous study.

    Use SCOP to describe secondary structure composition of each protein in the test set.

    How used SCOP:

    Categorized a data set of 15 proteins by their class.

    Reference to SCOP:

    According to the SCOP classification system,40 of the 15 proteins, 7 are all-α, 4 are all-β, and 4 are α+β

  ### Attachments

  - 24068\_ftp.pdf
- ## Mining Tertiary Structural Motifs for Assessment of Designability

  |  |  |
  | --- | --- |
  | Type | Journal Article |
  | Author | Jian Zhang |
  | Author | Gevorg Grigoryan |
  | Volume | 523 |
  | Pages | 21–40 |
  | Publication | Methods In Protein Design |
  | Date | 2013 |
  | DOI | 10.1016/B978-0-12-394292-0.00002-3 |
  | Abstract | The observation of a limited secondary-structural alphabet in native proteins, with significant sequence preferences, has profoundly influenced the fields of protein design and structure prediction (Simons, Kooperberg, Huang, & Baker, 1997; Verschueren et al., 2011). In the era of structural genomics, as the size of the structural dataset continues to grow rapidly, it is becoming possible to extend this analysis to tertiary structural motifs and their sequences. For a hypothetical tertiary motif, the rate of its utilization in natural proteins may be used to assess its designability-the ease with which the motif can be realized with natural amino acids. This requires a structural similarity search methodology, which rather than looking for global topological agreement (more appropriate for categorization of full proteins or domains), identifies detailed geometric matches. In this chapter, we introduce such a method, called MaDCaT, and demonstrate its use by assessing the designability landscapes of two tertiary structural motifs. We also show that such analysis can establish structure/sequence links by providing the sequence constraints necessary to encode designable motifs. As logical extension of their secondary-structure counterparts, tertiary structural preferences will likely prove extremely useful in de novo protein design and structure prediction. |
  | Date Added | 3/7/2014, 12:08:00 PM |
  | Modified | 3/7/2014, 1:06:34 PM |
- ## Mistletoe lectin has a shiga toxin-like structure and should be combined with other Toll-like receptor ligands in cancer therapy

  |  |  |
  | --- | --- |
  | Type | Journal Article |
  | Author | Claudia Maletzki |
  | Author | Michael Linnebacher |
  | Author | Rajkumar Savai |
  | Author | Uwe Hobohm |
  | Volume | 62 |
  | Issue | 8 |
  | Pages | 1283–1292 |
  | Publication | Cancer Immunology Immunotherapy |
  | Date | August 2013 |
  | DOI | 10.1007/s00262-013-1455-1 |
  | Abstract | Mistletoe extract (ME) is applied as an adjuvant treatment in cancer therapy in thousands of patients each year in Europe. The main immunostimulating component of mistletoe extract, mistletoe lectin, recently has been shown to be a pattern recognition receptor ligand and hence is binding to an important class of pathogen-sensing receptors. Pattern recognition receptor ligands are potent activators of dendritic cells. This activation is a prerequisite for a full-blown T-cell response against cancer cells. Pattern recognition receptor ligands are increasingly recognized as important players in cancer immunotherapy. We collect evidence from case studies on spontaneous regression, from epidemiology, from experiments in a mouse cancer model, and from protein structure comparisons to argue that a combination of mistletoe therapy with other pattern recognition receptor ligand substances leads to an increased immune stimulatory effect. We show that mistletoe lectin is a plant protein of bacterial origin with a 3D structure very similar to shiga toxin from Shigella dysenteriae, which explains the remarkable immunogenicity of mistletoe lectin. Secondly, we show that a combination of pattern recognition receptor ligands applied metronomically in a cancer mouse model leads to complete remission, while single pattern recognition receptor ligands slowed tumor growth. Taken together, we propose to combine mistletoe drugs with other pattern recognition receptor ligand drugs to increase its efficacy in adjuvant or even primary cancer therapy. |
  | Date Added | 3/7/2014, 12:08:00 PM |
  | Modified | 3/7/2014, 12:08:00 PM |
- ## Modeling of folds and folding pathways for some protein families of (alpha+beta)- and (alpha/beta)-classes

  |  |  |
  | --- | --- |
  | Type | Journal Article |
  | Author | Alexey B. Gordeev |
  | Author | Alexander V. Efimov |
  | Volume | 31 |
  | Issue | 1, SI |
  | Pages | 4-16 |
  | Publication | Journal of Biomolecular Structure and Dynamics |
  | ISSN | 0739-1102 |
  | Date | JAN 1 2013 |
  | DOI | 10.1080/07391102.2012.691341 |
  | Language | English |
  | Abstract | In this paper, updated structural trees for a/beta-proteins containing five- and seven-segment (a/beta)-motifs are represented. Novel structural motifs occurring in some families of (a?+beta)- and (a/beta)-proteins are also characterized. Databases of these proteins have been compiled from the Protein Data Bank (PDB) and Structural Classification of Proteins (SCOP) and the corresponding structural trees have been constructed. The classification of these proteins has been developed and organized as an extension of the PCBOST database, which is available at http://strees.protres.ru. In total, the updated Protein Classification Based on Structural Trees database contains 11 structural trees, 106 levels, 635 folds, 4911 proteins and domains, and 14,202 PDB entries. |
  | Date Added | 10/11/2013, 10:29:15 AM |
  | Modified | 3/7/2014, 12:09:43 PM |

  ### Tags:

  - classification
  - handedness
  - structural motif
  - structural tree

  ### Notes:

  - Extend an alternative structure classification database, PDBOST, which is based on the similarity of spatial structures and common folding pathways simulated with trees, and does not use any functional or evolutionary information.

    How SCOP is used:

    Mention SCOP for background on protein structure classificaition.

    How CATH is used:

    Not using CATH data.

    SCOP reference:

    Abstract:

    In this paper, updated structural trees for α/β-proteins containing five- and seven-segment (α/β)-motifs are represented. Novel structural motifs occurring in some families of (α+β)- and (α/β)-proteins are also characterized. Databases of these proteins have been compiled from the Protein Data Bank (PDB) and Structural Classification of Proteins (SCOP) and the corresponding structural trees have been constructed. The classification of these proteins has been developed and organized as an extension of the PCBOST database, which is available at http://strees.protres.ru. In total, the updated Protein Classification Based on Structural Trees database contains 11 structural trees, 106 levels, 635 folds, 4911 proteins and domains, and 14,202 PDB entries.

    ...

    Based on the structural trees, we have developed a novel Structural Classifica- tion of Proteins (SCOP) referred to as Protein Classification Based on Structural Trees (PCBOST) (Gordeev, Kargatov, & Efimov, 2010), which is available at http://strees.protres.ru/. This classification is based primarily on the similarity of spatial structures and common folding pathways simulated with the trees, thereby differing from other known protein classifica- tions like Structural Classification of Proteins (SCOP) (Murzin, Brenner, Hubbard, & Chothia, 1995), Class- Architecture-Topology-Homologous superfamily (CATH) (Orengo et al., 1997), and others (Dietmann et al., 2001; Sowdhamini, Rufino, & Blundell, 1996; Przytycka, Aur- ora, & Rose, 1999). Our classification disregards the amino acid sequences, functions, and evolutionary rela- tionships of proteins which are taken into account in other known classifications.

  ### Attachments

  - 07391102%2E2012%2E691341.pdf
- ## Modeling Proteins Using a Super-Secondary Structure Library and NMR Chemical Shift Information

  |  |  |
  | --- | --- |
  | Type | Journal Article |
  | Author | Vilas Menon |
  | Author | Brinda K. Vallat |
  | Author | Joseph M. Dybas |
  | Author | Andras Fiser |
  | Volume | 21 |
  | Issue | 6 |
  | Pages | 891-899 |
  | Publication | Structure |
  | ISSN | 0969-2126 |
  | Date | JUN 4 2013 |
  | Extra | WOS:000320739800006 |
  | DOI | 10.1016/j.str.2013.04.012 |
  | Abstract | A remaining challenge in protein modeling is to predict structures for sequences with no sequence similarity to any experimentally solved structure. Based on earlier observations, the library of protein backbone supersecondary structure motifs (Smotifs) saturated about a decade ago. Therefore, it should be possible to build any structure from a combination of existing Smotifs with the help of limited experimental data that are sufficient to relate the backbone conformations of Smotifs between target proteins and known structures. Here, we present a hybrid modeling algorithm that relies on an exhaustive Smotif library and on nuclear magnetic resonance chemical shift patterns without any input of primary sequence information. In a test of 102 proteins, the algorithm delivered 90 homology-model-quality models, among them 24 high-quality ones, and a topologically correct solution for almost all cases. The current approach opens a venue to address the modeling of larger protein structures for which chemical shifts are available. |
  | Date Added | 2/20/2014, 12:24:01 PM |
  | Modified | 2/20/2014, 12:24:01 PM |

  ### Notes:

  - Present method to assist in solving NMR structures using "Smotifs", a database of secondary structure motifs.

    How SCOP is used:

    Evaluate method on non-redundant data set from the Biological Magnetic Resonance Data Bank (BMRB), which distributes a test set where each structure has a different SCOP fold.

    SCOP reference:

    Benchmarking the Algorithm

    We implemented our prediction method on a data set of 102 pro- teins obtained from the Biological Magnetic Resonance Data Bank (BMRB) (Ulrich et al., 2008) database (Table S1 available online). **The test set is currently the largest nonredundant data set of experimentally known structures for which CS data are publically available and where all structures represent a different SCOP fold category (****Andreeva et al., 2008****).** This selection en- sures that the largest possible varieties of proteins are tested with respect to secondary structure composition and topologies. The results are presented as a distribution of GDT\_TS scores (Zemla, 2003) of the superposed backbone atoms for the entire lengths of the experimental structure and the top-ranked model (Figure 2). The top-ranked models have GDT\_TS scores in the range of 20%–80%. The number of proteins where the best- sampled models have GDT\_TS R 50%, is 47 (Figure 2). This means that a high-quality homology model is generated for about half of the cases and for almost all cases at least a topo- logically correct fold is produced. The 102 proteins can be broken down in different SCOP classes, with a slight difference in terms of performance. The best-performing classes in terms of median GDT\_TS scores are the all-a class (44%) followed by the a/b class (40%), while the all-b class (37%) and a+b class (36%) lag behind slightly and the class of small proteins are in the middle (39%). The only two designed proteins in our set perform the best, albeit the statistics are very limited. We also employed a smaller, separate set of ten proteins for exploring some of the computationally intensive aspects of the method.

    ...

    Test Data Set of Experimentally Known Proteins

    Entries were extracted from the BMRB database (out of a current total of 7,881) that had either been deposited simultaneously with a corresponding PDB entry or which had a corresponding solution NMR PDB entry with a BMRB ‘‘compar- ison score’’ less than or equal to 9. Entries with identical sequence to the corresponding PDB file and with complete CS data were retained. In order to select the widest possible range of protein architectures, all entries were cross-referenced with SCOP (Andreeva et al., 2008). From the remaining set we selected 102 proteins, which did not generate errors when running TALOS+ (Table S1). I**n terms of SCOP class definition, the test set contained 42 all-****a, 8 all-b, 3 a/b, 33 a+b****, 14 small proteins, and 2 designed proteins, all belonging to a unique fold category.** The length of the proteins ranged between 56 and 130 with a median length of 88 residues, and these proteins are composed of two to eight Smotifs. Due to the intensity of the computation, the algorithm itself was parameterized, trained, and developed on a smaller set of ten pro- teins solved by NMR and disjoint from the above-described 102 member test set: 2KL8, 2KCl, 2KD1, 2KPO, 2KYS, 2JMO, 2JUA, 2JVE, 2JVF, and 2L2N.

  ### Attachments

  - 1-s2.0-S096921261300124X-main.pdf
- ## Modeling regionalized volumetric differences in protein-ligand binding cavities

  |  |  |
  | --- | --- |
  | Type | Journal Article |
  | Author | Brian Y. Chen |
  | Author | Soutir Bandyopadhyay |
  | Volume | 10 |
  | Pages | S6 |
  | Publication | Proteome Science |
  | Date | June 2012 |
  | DOI | 10.1186/1477-5956-10-S1-S6 |
  | Abstract | Identifying elements of protein structures that create differences in protein-ligand binding specificity is an essential method for explaining the molecular mechanisms underlying preferential binding. In some cases, influential mechanisms can be visually identified by experts in structural biology, but subtler mechanisms, whose significance may only be apparent from the analysis of many structures, are harder to find. To assist this process, we present a geometric algorithm and two statistical models for identifying significant structural differences in protein-ligand binding cavities. We demonstrate these methods in an analysis of sequentially nonredundant structural representatives of the canonical serine proteases and the enolase superfamily. Here, we observed that statistically significant structural variations identified experimentally established determinants of specificity. We also observed that an analysis of individual regions inside cavities can reveal areas where small differences in shape can correspond to differences in specificity. |
  | Date Added | 3/7/2014, 1:06:24 PM |
  | Modified | 3/7/2014, 1:06:24 PM |
- ## Modular Evolution and the Origins of Symmetry: Reconstruction of a Three-Fold Symmetric Globular Protein

  |  |  |
  | --- | --- |
  | Type | Journal Article |
  | Author | Aron Broom |
  | Author | Andrew C. Doxey |
  | Author | Yuri D. Lobsanov |
  | Author | Lisa G. Berthin |
  | Author | David R. Rose |
  | Author | P. Lynne Howell |
  | Author | Brendan J. McConkey |
  | Author | Elizabeth M. Meiering |
  | Volume | 20 |
  | Issue | 1 |
  | Pages | 161-171 |
  | Publication | Structure |
  | ISSN | 0969-2126 |
  | Date | JAN 11 2012 |
  | DOI | 10.1016/j.str.2011.10.021 |
  | Language | English |
  | Abstract | The high frequency of internal structural symmetry in common protein folds is presumed to reflect their evolutionary origins from the repetition and fusion of ancient peptide modules, but little is known about the primary sequence and physical determinants of this process. Unexpectedly, a sequence and structural analysis of symmetric subdomain modules within an abundant and ancient globular fold, the beta-trefoil, reveals that modular evolution is not simply a relic of the ancient past, but is an ongoing and recurring mechanism for regenerating symmetry, having occurred independently in numerous existing beta-trefoil proteins. We performed a computational reconstruction of a beta-trefoil subdomain module and repeated it to form a newly three-fold symmetric globular protein, Three Foil. In addition to its near perfect structural identity between symmetric modules, Three Foil is highly soluble, performs multivalent carbohydrate binding, and has remarkably high thermal stability. These findings have far-reaching implications for understanding the evolution and design of proteins via subdomain modules. |
  | Date Added | 10/11/2013, 10:29:15 AM |
  | Modified | 12/2/2013, 4:17:54 PM |

  ### Notes:

  - Study of B-trefoil domain, an ancient fold adopted by many proteins with great diversity of sequence and function.

    How SCOP is used:

    Used SCOP, CDD, and Pfam to build a data set of B-trefoil domain sequences.

    Collect all families in SCOP that are 'annotated as beta-trefoil'.

    SCOP reference:

    EXPERIMENTAL PROCEDURES

    Sequence Dataset Construction and Analysis

    All annotated b-trefoil domain sequences were retrieved from the National Center for Biotechnology Information (NCBI) using the Conserved Domain Database (CDD). All families annotated as b-trefoils by SCOP (Murzin et al., 1995) and Pfam (Finn et al., 2010) with an available structure in the Protein Data Bank (http://www.pdb.org) (Berman et al., 2000) were included. See Table 1 for statistics on construction of the dataset.

  ### Attachments

  - 1-s2.0-S0969212611004102-main.pdf
- ## Molecular and Biochemical Analyses of the GH44 Module of CbMan5B/Cel44A, a Bifunctional Enzyme from the Hyperthermophilic Bacterium Caldicellulosiruptor bescii

  |  |  |
  | --- | --- |
  | Type | Journal Article |
  | Author | Libin Ye |
  | Author | Xiaoyun Su |
  | Author | George E. Schmitz |
  | Author | Young Hwan Moon |
  | Author | Jing Zhang |
  | Author | Roderick I. Mackie |
  | Author | Isaac KO Cann |
  | URL | http://aem.asm.org/content/78/19/7048.short |
  | Volume | 78 |
  | Issue | 19 |
  | Pages | 7048–7059 |
  | Publication | Applied and environmental microbiology |
  | Date | 2012 |
  | Accessed | 9/20/2013, 1:19:04 PM |
  | Library Catalog | Google Scholar |
  | Date Added | 10/11/2013, 10:29:15 AM |
  | Modified | 10/11/2013, 10:29:15 AM |

  ### Notes:

  - Experimental study of the GH44 enzyme (glycosyl hydrogenase).

    How SCOP is used:

    Look up the family-level classification of proteins studied (Composite domain of glycosyl hydrolase families 5, 30, 39, and 51) when giving background on the structure of the GH44 catalytic modules.

    SCOP reference:

    The Beta-sandwich domain is required for proper folding of the GH44 module. The GH44 catalytic modules are composed of a TIM-like domain with an accompanying beta-sandwich domain. The co-occurrence of a TIM-like domain with a beta-sandwich do- main is also found in GH5, GH30, GH39, and GH51 proteins (8, 13, 19, 25, 39). The beta-sandwich domain is regarded as “a composite domain” for these proteins in the Structural Classification of Proteins database (32).

  ### Attachments

  - Appl. Environ. Microbiol.-2012-Ye-7048-59.pdf
- ## Molecular characterization of an alpha-N-acetylgalactosaminidase from Clonorchis sinensis

  |  |  |
  | --- | --- |
  | Type | Journal Article |
  | Author | Myoung-Ro Lee |
  | Author | Won Gi Yoo |
  | Author | Yu-Jung Kim |
  | Author | Dae-Won Kim |
  | Author | Shin-Hyeong Cho |
  | Author | Kwang Yeon Hwang |
  | Author | Jung-Won Ju |
  | Author | Won-Ja Lee |
  | Volume | 111 |
  | Issue | 5 |
  | Pages | 2149–2156 |
  | Publication | Parasitology Research |
  | Date | November 2012 |
  | DOI | 10.1007/s00436-012-3063-y |
  | Abstract | The alpha-N-acetylgalactosaminidase (alpha-NAGAL) is an exoglycosidase that selectively cleaves terminal alpha-linked N-acetylgalactosamines from a variety of sugar chains. A complementary DNA (cDNA) clone encoding a novel Clonorchis sinensis alpha-NAGAL (Cs-alpha-NAGAL) was identified in the expressed sequence tags database of the adult C. sinensis liver fluke. The complete coding sequence was 1,308 bp long and encoded a 436-residue protein. The selected glycosidase was manually curated as alpha-NAGAL (EC 3.2.1.49) based on a composite bioinformatics analysis including a search for orthologues, comparative structure modeling, and the generation of a phylogenetic tree. One orthologue of Cs-alpha-NAGAL was the Rattus norvegicus alpha-NAGAL (accession number: NP\_001012120) that does not exist in C. sinensis. Cs-alpha-NAGAL belongs to the GH27 family and the GH-D clan. A phylogenetic analysis revealed that the GH27 family of Cs-alpha-NAGAL was distinct from GH31 and GH36 within the GH-D clan. The putative 3D structure of Cs-alpha-NAGAL was built using SWISS-MODEL with a Gallus gallus alpha-NAGAL template (PDB code 1ktb chain A); this model demonstrated the superimposition of a TIM barrel fold (alpha/beta) structure and substrate binding pocket. Cs-alpha-NAGAL transcripts were detected in the adult worm and egg cDNA libraries of C. sinensis but not in the metacercaria. Recombinant Cs-alpha-NAGAL (rCs-alpha-NAGAL) was expressed in Escherichia coli, and the purified rCs-alpha-NAGAL was recognized specifically by the C. sinensis-infected human sera. This is the first report of an alpha-NAGAL protein in the Trematode class, suggesting that it is a potential diagnostic or vaccine candidate with strong antigenicity. |
  | Date Added | 3/7/2014, 12:08:00 PM |
  | Modified | 3/7/2014, 12:08:00 PM |
- ## Molecular dynamics simulations of the Bcl-2 protein to predict the structure of its unordered flexible loop domain

  |  |  |
  | --- | --- |
  | Type | Journal Article |
  | Author | Pawan Kumar Raghav |
  | Author | Yogesh Kumar Verma |
  | Author | Gurudutta U. Gangenahalli |
  | Volume | 18 |
  | Issue | 5 |
  | Pages | 1885–1906 |
  | Publication | Journal of Molecular Modeling |
  | Date | May 2012 |
  | DOI | 10.1007/s00894-011-1201-6 |
  | Abstract | B-cell lymphoma (Bcl-2) protein is an anti-apoptotic member of the Bcl-2 family. It is functionally demarcated into four Bcl-2 homology (BH) domains: BH1, BH2, BH3, BH4, one flexible loop domain (FLD), a transmembrane domain (TM), and an X domain. Bcl-2's BH domains have clearly been elucidated from a structural perspective, whereas the conformation of FLD has not yet been predicted, despite its important role in regulating apoptosis through its interactions with JNK-1, PKC, PP2A phosphatase, caspase 3, MAP kinase, ubiquitin, PS1, and FKBP38. Many important residues that regulate Bcl-2 anti-apoptotic activity are present in this domain, for example Asp34, Thr56, Thr69, Ser70, Thr74, and Ser87. The structural elucidation of the FLD would likely help in attempts to accurately predict the effect of mutating these residues on the overall structure of the protein and the interactions of other proteins in this domain. Therefore, we have generated an increased quality model of the Bcl-2 protein including the FLD through modeling. Further, molecular dynamics (MD) simulations were used for FLD optimization, to predict the flexibility, and to determine the stability of the folded FLD. In addition, essential dynamics (ED) was used to predict the collective motions and the essential subspace relevant to Bcl-2 protein function. The predicted average structure and ensemble of MD-simulated structures were submitted to the Protein Model Database (PMDB), and the Bcl-2 structures obtained exhibited enhanced quality. This study should help to elucidate the structural basis for Bcl-2 anti-apoptotic activity regulation through its binding to other proteins via the FLD. |
  | Date Added | 3/7/2014, 1:06:24 PM |
  | Modified | 3/7/2014, 1:06:24 PM |
- ## Molecular Evolution of Translin Superfamily Proteins Within the Genomes of Eubacteria, Archaea and Eukaryotes

  |  |  |
  | --- | --- |
  | Type | Journal Article |
  | Author | Gagan D. Gupta |
  | Author | Avinash Kale |
  | Author | Vinay Kumar |
  | Volume | 75 |
  | Issue | 5-6 |
  | Pages | 155-167 |
  | Publication | Journal of Molecular Evolution |
  | ISSN | 0022-2844 |
  | Date | DEC 2012 |
  | Extra | WOS:000312263300001 |
  | DOI | 10.1007/s00239-012-9534-z |
  | Abstract | Translin and its interacting partner protein, TRAX, are members of the translin superfamily. These proteins are involved in mRNA regulation and in promoting RISC activity by removing siRNA passenger strand cleavage products, and have been proposed to play roles in DNA repair and recombination. Both homomeric translin and heteromeric translin-TRAX complex bind to ssDNA and RNA; however, the heteromeric complex is a key activator in siRNA-mediated silencing in human and drosophila. The residues critical for RNase activity of the complex reside in TRAX sequence. Both translin and TRAX are well conserved in eukaryotes. In present work, a single translin superfamily protein is detected in Chloroflexi eubacteria, in the known phyla of archaea and in some unicellular eukaryotes. The prokaryotic proteins essentially share unique sequence motifs with eukaryotic TRAX, while the proteins possessing both the unique sequences and conserved indels of TRAX or translin can be identified from protists. Intriguingly, TRAX protein in all the known genomes of extant Chloroflexi share high sequence similarity and conserved indels with the archaeal protein, suggesting occurrence of TRAX at least at the time of Chloroflexi divergence as well as evolutionary relationship between Chloroflexi and archaea. The mirror phylogeny in phylogenetic tree, constructed using diverse translin and TRAX sequences, indicates gene duplication event leading to evolution of translin in unicellular eukaryotes, prior to divergence of multicellular eukayrotes. Since Chloroflexi has been debated to be near the last universal common ancestor, the present analysis indicates that TRAX may be useful to understand the tree of life. |
  | Date Added | 2/20/2014, 12:24:01 PM |
  | Modified | 2/20/2014, 12:24:01 PM |

  ### Notes:

  - Perform phylogenetic analysis to study evolution of Translin and its interacting partner protein, TRAX, both members of the translin superfamily.

    How SCOP is used:

    Look up SCOP superfamily classification for proteins of interest.

    SCOP reference:

    However, clustering of translin and TRAX proteins as distinct families is not conspicuous in databases, like SCOP (Andreeva et al. 2008), PFAM (Punta et al. 2012), and CDD (Marchler-Bauer et al. 2009). This prompted us to study the evolutionary relationship of both the proteins as well as to determine their ancestry.

    ...

    Also all the hits identified by various fold-recognition servers used in the meta-server job predicted high confidence homology of the bacterial protein with either TRAX or translin pro- tein (SCOP ID 74784).

    ...

    The tertiary structure of translin and TRAX monomers is also very similar and both the proteins belong to translin superfamily (SCOP ID 74784).

  ### Attachments

  - art%3A10.1007%2Fs00239-012-9534-z.pdf
- ## Molecular Modeling Comparison of the Performance of NS5b Polymerase Inhibitor (PSI-7977) on Prevalent HCV Genotypes

  |  |  |
  | --- | --- |
  | Type | Journal Article |
  | Author | Abdo A. Elfiky |
  | Author | Wael M. Elshemey |
  | Author | Wissam A. Gawad |
  | Author | Omar S. Desoky |
  | URL | http://link.springer.com/article/10.1007/s10930-013-9462-9 |
  | Volume | 32 |
  | Issue | 1 |
  | Pages | 75–80 |
  | Publication | The protein journal |
  | Date | 2013 |
  | Accessed | 9/23/2013, 10:25:16 AM |
  | Library Catalog | Google Scholar |
  | Date Added | 10/11/2013, 10:29:15 AM |
  | Modified | 3/7/2014, 12:09:54 PM |

  ### Tags:

  - DAA
  - HCV
  - Molecular modeling
  - NS5b
  - Nucleoside inhibitor
  - PSI-7977

  ### Notes:

  - Use homology modeling to study structure and function of PSI-7977, a drug for the treatment of Hepatitis C virus.

    How SCOP is used:

    Look up classification of their protein of interest: class, fold,superfamily, and family.

    SCOP reference:

    According to structure classification of proteins (SCOP) database [1, 2, 11, 17, 31], HCV Non-structural 5b poly- merase is classified as follow:

    Class: Multi-domain proteins (alpha and beta). Fold: DNA/RNA polymerases.  
     Superfamily: DNA/RNA polymerases.  
     Family: RNA dependent RNA polymerase.

  ### Attachments

  - art%3A10.1007%2Fs10930-013-9462-9.pdf
- ## Molecular replacement then and now

  |  |  |
  | --- | --- |
  | Type | Journal Article |
  | Author | Giovanna Scapin |
  | Volume | 69 |
  | Pages | 2266-2275 |
  | Publication | Acta Crystallographica Section D-Biological Crystallography |
  | ISSN | 0907-4449; 1399-0047 |
  | Date | NOV 2013 |
  | Extra | WOS:000326648900014 |
  | DOI | 10.1107/S0907444913011426 |
  | Abstract | The `phase problem' in crystallography results from the inability to directly measure the phases of individual diffracted X-ray waves. While intensities are directly measured during data collection, phases must be obtained by other means. Several phasing methods are available (MIR, SAR, MAD, SAD and MR) and they all rely on the premise that phase information can be obtained if the positions of marker atoms in the unknown crystal structure are known. This paper is dedicated to the most popular phasing method, molecular replacement (MR), and represents a personal overview of the development, use and requirements of the methodology. The first description of noncrystallographic symmetry as a tool for structure determination was explained by Rossmann and Blow [Rossmann & Blow (1962), Acta Cryst.15, 24-31]. The term `molecular replacement' was introduced as the name of a book in which the early papers were collected and briefly reviewed [Rossmann (1972), The Molecular Replacement Method. New York: Gordon & Breach]. Several programs have evolved from the original concept to allow faster and more sophisticated searches, including six-dimensional searches and brute-force approaches. While careful selection of the resolution range for the search and the quality of the data will greatly influence the outcome, the correct choice of the search model is probably still the main criterion to guarantee success in solving a structure using MR. Two of the main parameters used to define the `best' search model are sequence identity (25% or more) and structural similarity. Another parameter that may often be undervalued is the quality of the probe: there is clearly a relationship between the quality and the correctness of the chosen probe and its usefulness as a search model. Efforts should be made by all structural biologists to ensure that their deposited structures, which are potential search probes for future systems, are of the best possible quality. |
  | Date Added | 2/12/2014, 2:18:08 PM |
  | Modified | 3/7/2014, 12:08:56 PM |

  ### Tags:

  - Interesting

  ### Notes:

  - Review of molecular replacement methods.

    How SCOP is used:

    Examine the number of new SCOP folds and CATH topologies added each year and also the number of new structures per fold that have been added.  More structures per fold increases accuracy of molecular replacement.

    Why is CATH cited:

    Provide summary statistics from both SCOP and CATH to compare the two databases.

    SCOP reference:

    Figure 4

    (a) Distribution of ‘new’ and total SCOP folds (red and yellow) and ‘new’ and total CATH topologies (purple and green) in the PDB. This graph was generated using the tools available in the ‘PDB Statistics’ page of the RSCB PDB (http://www.rcsb.org; Berman et al., 2000). There has been no new fold reported since 2008 and no new topology since 2009. (b) Distribution of new ‘all-⬚⬚’ folds over the years: the large majority were discovered between 1990 and 2000, and between then and now the distribution of folds is basically unchanged. (c) Yearly and total reports for the ⬚⬚–⬚⬚ superhelix fold (as defined in SCOP). Even if the total number of folds has not changed, the number of structures within the fold has increased.

    ...

    3. Search models  
     3.1. Sequence identity versus structure similarity

    It is well known that the more similar, both in primary and tertiary structure, the search model is to the target, the more likely it is that a solution to an MR problem can be found. It has generally been accepted that a 1.5 A ̊ r.m.s.d. between the search model and the target is the lowest limit at which a related structure can be used as a search model. However, the r.m.s.d. is a post mortem evaluation of how similar the probe and target are, and the only initially available indicator of closeness is the sequence identity. According to the Chothia equation (Chothia & Lesk, 1986), a 1.5 A ̊ r.m.s.d. corresponds to ⬚⬚29% sequence identity, which loosely translates into saying that a search model has to be at least 30% identical to the target to be a good search model. This is not always the case, however: a high sequence identity can still lead to a high r.m.s.d. if relative domain movements or variations in loop positions are present. On the other hand, molecules with a much lower identity can be good search models if three- dimensional similarity is retained. Fig. 4(a) shows the distri- bution of ‘new’ and total folds in the PDB both as SCOP (Murzin et al., 1995) folds and CATH (Orengo et al., 1997) topologies. No new folds have been reported since 2008 and no new topologies since 2009, but for MR purposes the important fact is that even if the total number of folds has not changed, the number of structures within a fold has increased. Figs. 4(b) and 4(c) show, for example, that even if the large majority of new ‘all-⬚⬚’ folds were discovered between 1990 and 2000 and the distribution of folds is basically unchanged between then and now, the number of structure within each fold (the ⬚⬚–⬚⬚ superhelix fold in Fig. 4c being just an example), is much higher now than just five years ago. In 2000 there were about 50 examples of the ⬚⬚–⬚⬚ superhelix fold; today there are over 350. This provides a much finer sampling of the three- dimensional space, and even if the primary-sequence identity

    between the target and the probe is much lower that the desired 25–35%, the chances of finding a probe with a similar three-dimensional structure are increasing. Most of the programs have ways to take this three-dimensional sampling into consideration, either by using structural alignments or multiple models or other knowledge-based modification of the search models. Various programs in CCP4 can be used to perform probe modification: for example, CHAINSAW (Stein, 2008) can be used to prune side chains based on a given alignment, PDBCUR provides various analyses and manip- ulations of PDB files, including B-factor analysis and ways to cut out residues/loops if their B factors are above an accep- table threshold, and PDBSET allows the removal of waters and other small molecules. The Rosetta suite (Das & Baker, 2008), which was initially developed for de novo protein structure prediction, has methods for homology modeling and protein design that can modify the starting probe and has been proven to be efficacious in solving complex molecular- replacement problems (Kaufmann et al., 2010; DiMaio et al., 2011).

  ### Attachments

  - ba5202.pdf
- ## MoNetFamily: a web server to infer homologous modules and module-module interaction networks in vertebrates

  |  |  |
  | --- | --- |
  | Type | Journal Article |
  | Author | Chun-Yu Lin |
  | Author | Yi-Wei Lin |
  | Author | Shang-Wen Yu |
  | Author | Yu-Shu Lo |
  | Author | Jinn-Moon Yang |
  | Volume | 40 |
  | Issue | W1 |
  | Pages | W263-W270 |
  | Publication | Nucleic Acids Research |
  | ISSN | 0305-1048 |
  | Date | JUL 2012 |
  | Extra | WOS:000306670900043 |
  | DOI | 10.1093/nar/gks541 |
  | Abstract | A module is a fundamental unit forming with highly connected proteins and performs a certain kind of biological functions. Modules and module-module interaction (MMI) network are essential for understanding cellular processes and functions. The MoNetFamily web server can identify the modules, homologous modules (called module family) and MMI networks across multiple species for the query protein(s). This server first finds module candidates of the query by using BLASTP to search the module template database (1785 experimental and 1252 structural templates). MoNetFamily then infers the homologous modules of the selected module candidate using protein-protein interaction (PPI) families. According to homologous modules and PPIs, we statistically calculated MMIs and MMI networks across multiple species. For each module candidate, MoNetFamily identifies its neighboring modules and their MMIs in module networks of Homo sapiens, Mus musculus and Danio rerio. Finally, MoNetFamily shows the conserved proteins, PPI profiles and functional annotations of the module family. Our results indicate that the server can be useful for MMI network (e.g. 1818 modules and 9678 MMIs in H. sapiens) visualizations and query annotations using module families and neighboring modules. We believe that the server is able to provide valuable insights to determine homologous modules and MMI networks across multiple species for studying module evolution and cellular processes. The MoNetFamily sever is available at http://monetfamily.life.nctu.edu.tw. |
  | Date Added | 10/28/2013, 4:53:08 PM |
  | Modified | 10/28/2013, 4:53:08 PM |

  ### Notes:

  - MoNetFamily is a server to predict homologous modules and module-module interaction networks.

    How SCOP is used:

    SCOP is cited as a model for a protein classification database.  Compare concept of module families with SCOP families.

    SCOP reference:

    The concept of the module family is analogous to the concepts of protein sequence family (11) and protein structure family **(12)** and protein–protein interactions (PPI) family (13).

  ### Attachments

  - Nucl. Acids Res.-2012-Lin-W263-70.pdf
- ## MSV3d: database of human MisSense variants mapped to 3D protein structure

  |  |  |
  | --- | --- |
  | Type | Journal Article |
  | Author | Tien-Dao Luu |
  | Author | Alin-Mihai Rusu |
  | Author | Vincent Walter |
  | Author | Raymond Ripp |
  | Author | Luc Moulinier |
  | Author | Jean Muller |
  | Author | Thierry Toursel |
  | Author | Julie D. Thompson |
  | Author | Olivier Poch |
  | Author | Hoan Nguyen |
  | Pages | bas018 |
  | Publication | Database-the Journal of Biological Databases and Curation |
  | ISSN | 1758-0463 |
  | Date | APR 3 2012 |
  | Extra | WOS:000304919000001 |
  | DOI | 10.1093/database/bas018 |
  | Abstract | The elucidation of the complex relationships linking genotypic and phenotypic variations to protein structure is a major challenge in the post-genomic era. We present MSV3d (Database of human MisSense Variants mapped to 3D protein structure), a new database that contains detailed annotation of missense variants of all human proteins (20 199 proteins). The multi-level characterization includes details of the physico-chemical changes induced by amino acid modification, as well as information related to the conservation of the mutated residue and its position relative to functional features in the available or predicted |
  | Date Added | 2/20/2014, 12:24:01 PM |
  | Modified | 2/20/2014, 12:24:01 PM |

  ### Notes:

  - Present MSV3d: a database of MisSense variants mapped to 3D protein structure.

    How SCOP is used:

    Annotate structures in database with SCOP fold classification.  Collect stats on distribution of missense variants in SCOP folds.

    SCOP reference:

    Mutant information. This level involves data related to the gene and its associated protein, the chromosome position, the OMIM disease and genotype population ref- erence. Pathogenicity prediction scores from external tools are provided by locally running the latest version of SIFT (21) and Polyphen-2 (9) to predict damaging effects of all missense variants in MSV3d. The SCOP fold classification (22) is also identified.

    ...

    Database statistics

    MSV3d currently contains more than 445574 missense variants mapped to 20 199 human proteins. Of these mis- sense variants, 58159 were found in SwissVar, 424541 in dbSNP (build 135) and 37 209 in both SwissVar and dbSNP. A total of 24379 the missense variants are considered as disease-causing variants and 421 195 as VUS.

    Concerning the structural data, 10713 structural tem- plates from the PDB database have been identified allow- ing the mapping of 63528 variants to a 3D structure. **Among those mapped variants, 13421 are identified in 265 SCOP fold classif**ications and 8023 variants are asso- ciated with 1479 OMIM diseases. Concerning gene conser- vation and function, 49 164 variants are mapped to one of the 2342 functional domains identified in the database (extracted from the Pfam protein family database (31), vali- dated and propagated by MACSIMS) and 1799 HPO ontol- ogy terms from the HPO (Human Phenotype Ontology) database (32).

    Up-to-date statistics concerning the physico-chemical changes induced by the amino acid substitutions, the con- servation patterns, the localization in a secondary structure and/or functional domain are available on the ‘Statistics’ page of the website. **Distributions of missense variants in SCOP folds or Pfam domains are also provid**ed. As an example, Figure 2 illustrates the top 20 SCOP folds enriched in missense variants. By default, these statistics take into account the missense variants of all genes in the database. However, the user can also submit his own gene list in order to personalize the statistics analysis.

  ### Attachments

  - Database-2012-Luu-database-bas018.pdf
- ## MulPSSM: a database of multiple position-specific scoring matrices of protein domain families

  |  |  |
  | --- | --- |
  | Type | Journal Article |
  | Author | V. S. Gowri |
  | Author | O. Krishnadev |
  | Author | C. S. Swamy |
  | Author | N. Srinivasan |
  | Volume | 34 |
  | Issue | Database issue |
  | Pages | D243-246 |
  | Publication | Nucleic Acids Research |
  | ISSN | 1362-4962 |
  | Date | Jan 1, 2006 |
  | Extra | PMID: 16381855 PMCID: PMC1347406 |
  | Journal Abbr | Nucleic Acids Res. |
  | DOI | 10.1093/nar/gkj043 |
  | Library Catalog | NCBI PubMed |
  | Language | eng |
  | Abstract | Representation of multiple sequence alignments of protein families in terms of position-specific scoring matrices (PSSMs) is commonly used in the detection of remote homologues. A PSSM is generated with respect to one of the sequences involved in the multiple sequence alignment as a reference. We have shown recently that the use of multiple PSSMs corresponding to an alignment, with several sequences in the family used as reference, improves the sensitivity of the remote homology detection dramatically. MulPSSM contains PSSMs for a large number of sequence and structural families of protein domains with multiple PSSMs for every family. The approach involves use of a clustering algorithm to identify most distinct sequences corresponding to a family. With each one of the distinct sequences as reference, multiple PSSMs have been generated. The current release of MulPSSM contains approximately 33,000 and approximately 38,000 PSSMs corresponding to 7868 sequence and 2625 structural families. A RPS\_BLAST interface allows sequence search against PSSMs of sequence or structural families or both. An analysis interface allows display and convenient navigation of alignments and domain hits. MulPSSM can be accessed at http://crick.mbu.iisc.ernet.in/~mulpssm. |
  | Short Title | MulPSSM |
  | Date Added | 11/3/2014, 3:38:14 PM |
  | Modified | 11/3/2014, 3:38:14 PM |

  ### Tags:

  - Databases, Protein
  - Internet
  - Protein Structure, Tertiary
  - Sequence Alignment
  - Sequence Analysis, Protein
  - User-Computer Interface

  ### Attachments

  - PubMed entry
- ## Multicopper oxidase-3 is a laccase associated with the peritrophic matrix of Anopheles gambiae

  |  |  |
  | --- | --- |
  | Type | Journal Article |
  | Author | Minglin Lang |
  | Author | Michael R. Kanost |
  | Author | Maureen J. Gorman |
  | URL | http://dx.plos.org/10.1371/journal.pone.0033985 |
  | Volume | 7 |
  | Issue | 3 |
  | Pages | e33985 |
  | Publication | PloS one |
  | Date | 2012 |
  | Accessed | 9/20/2013, 1:19:56 PM |
  | Library Catalog | Google Scholar |
  | Date Added | 10/11/2013, 10:29:15 AM |
  | Modified | 10/11/2013, 10:29:15 AM |

  ### Tags:

  - Amino Acid Sequence
  - Animals
  - Anopheles gambiae
  - Catalysis
  - Ceruloplasmin
  - Female
  - Humans
  - Hydrogen-Ion Concentration
  - Kinetics
  - Laccase
  - Molecular Sequence Data
  - Protein Transport
  - Recombinant Proteins
  - Sequence Alignment
  - Substrate Specificity

  ### Notes:

  - Experimental and computational study of the multicopper oxydase (MCO) family of enzymes.

    How SCOP is used:

    Use SCOP to get domain boundaries for a domain of interest in MCO sequences from 3 species.

    SCOP reference:

    Sequence analyses

    ...

    Boundaries of the putative cupredoxin- like domains were estimated by aligning MCO3 sequences with the sequence of a fungal laccase, Trametes versicolor laccaseIIIb (TvLacIIIb, PDB ID: 1KYA), which has a solved crystal structure, and using SCOP [36] to define the boundaries of the cupredoxin- like domains of TvLacIIIb.

    ...

  ### Attachments

  - [HTML] from plos.org
  - journal.pone.0033985.pdf
  - PubMed entry
- ## Multiple graph regularized protein domain ranking

  |  |  |
  | --- | --- |
  | Type | Journal Article |
  | Author | Jim Jing-Yan Wang |
  | Author | Halima Bensmail |
  | Author | Xin Gao |
  | Volume | 13 |
  | Publication | BMC BIOINFORMATICS |
  | ISSN | 1471-2105 |
  | Date | NOV 19 2012 |
  | DOI | 10.1186/1471-2105-13-307 |
  | Language | English |
  | Abstract | Background: Protein domain ranking is a fundamental task in structural biology. Most protein domain ranking methods rely on the pairwise comparison of protein domains while neglecting the global manifold structure of the protein domain database. Recently, graph regularized ranking that exploits the global structure of the graph defined by the pairwise similarities has been proposed. However, the existing graph regularized ranking methods are very sensitive to the choice of the graph model and parameters, and this remains a difficult problem for most of the protein domain ranking methods. Results: To tackle this problem, we have developed the Multiple Graph regularized Ranking algorithm, MultiG-Rank. Instead of using a single graph to regularize the ranking scores, MultiG-Rank approximates the intrinsic manifold of protein domain distribution by combining multiple initial graphs for the regularization. Graph weights are learned with ranking scores jointly and automatically, by alternately minimizing an objective function in an iterative algorithm. Experimental results on a subset of the ASTRAL SCOP protein domain database demonstrate that MultiG-Rank achieves a better ranking performance than single graph regularized ranking methods and pairwise similarity based ranking methods. Conclusion: The problem of graph model and parameter selection in graph regularized protein domain ranking can be solved effectively by combining multiple graphs. This aspect of generalization introduces a new frontier in applying multiple graphs to solving protein domain ranking applications. |
  | Date Added | 10/25/2013, 4:17:08 PM |
  | Modified | 10/25/2013, 4:17:08 PM |

  ### Notes:

  - Present method for protein structure classification/fold recognition.

    How SCOP is used:

    Use ASTRAL 40% data set as their "domain database" for use in clustering method with the query protein.

    Validate folds of retrieved domains match the query domain.

    SCOP reference:

    Protein domain database and query set

    We used the SCOP 1.75A database [21] to construct the database and query set. In the SCOP 1.75A database, there are 49,219 protein domain PDB entries and 135,643 domains, belonging to 7 classes and 1,194 SCOP fold types.

    Protein domain database

    Our protein domain database was selected from ASTRAL SCOP 1.75A set [21], a subset of the SCOP (Struc- tural Classification of Proteins)1.75A database which was released in March 15, 2012 [21]. ASTRAL SCOP 1.75A40%) [21], a genetic domain sequence subset, was used as our protein domain database D. This database was selected from SCOP 1.75A database so that the selected domains have less than 40% identity to each other. There are a total of 11,212 protein domains in the ASTRAL SCOP 1.75A 40% database belonging to 1,196 SCOP fold types. The ASTRAL database is available on-line at http:// scop.berkeley.edu. The number of protein domains in each SCOP fold varies from 1 to 402. The distribution of protein domains with the different fold types is shown in Figure 1. Many previous studies evaluated ranking per- formances using the older version of the ASTRAL SCOP dataset (ASTRAL SCOP 1.73 95%) that was released in 2008 [3].

    Query set

    We also randomly selected 540 protein domains from the SCOP 1.75A database to construct a query set. For each query protein domain that we selected we ensured that there was at least one protein domain belonging to the same SCOP fold type in the ASTRAL SCOP 1.75A 40% database, so that for each query, there was at least one ”positive” sample in the protein domain database. How- ever, it should be noted that the 540 protein domains in the query data set were randomly selected and do not necessarily represent 540 different folds. Here we call our query set the 540 query dataset because it contains 540 protein domains from the SCOP 1.75A database.

  ### Attachments

  - 1471-2105-13-307.pdf
- ## Multiple molecule effects on the cooperativity of protein folding transitions in simulations

  |  |  |
  | --- | --- |
  | Type | Journal Article |
  | Author | Jacob I. Lewis |
  | Author | Devin J. Moss |
  | Author | Thomas A. Knotts |
  | Volume | 136 |
  | Issue | 24 |
  | Pages | 245101 |
  | Publication | Journal of Chemical Physics |
  | ISSN | 0021-9606 |
  | Date | JUN 28 2012 |
  | Extra | WOS:000305881100046 |
  | DOI | 10.1063/1.4729604 |
  | Abstract | Though molecular simulation of proteins has made notable contributions to the study of protein folding and kinetics, disagreement between simulation and experiment still exists. One of the criticisms levied against simulation is its failure to reproduce cooperative protein folding transitions. This weakness has been attributed to many factors such as a lack of polarizability and adequate capturing of solvent effects. This work, however, investigates how increasing the number of proteins simulated simultaneously can affect the cooperativity of folding transitions - a topic that has received little attention previously. Two proteins are studied in this work: phage T4 lysozyme (Protein Data Bank (PDB) ID: 7LZM) and phage 434 repressor (PDB ID: 1R69). The results show that increasing the number of proteins molecules simulated simultaneously leads to an increase in the macroscopic cooperativity for transitions that are inherently cooperative on the molecular level but has little effect on the cooperativity of other transitions. Taken as a whole, the results identify one area of consideration to improving simulations of protein folding. (C) 2012 American Institute of Physics. [http://dx.doi.org/10.1063/1.4729604] |
  | Date Added | 2/13/2014, 4:13:17 PM |
  | Modified | 3/7/2014, 12:14:25 PM |
- ## Multiple structure alignment with msTALI

  |  |  |
  | --- | --- |
  | Type | Journal Article |
  | Author | Paul Shealy |
  | Author | Homayoun Valafar |
  | Volume | 13 |
  | Pages | 105 |
  | Publication | Bmc Bioinformatics |
  | ISSN | 1471-2105 |
  | Date | MAY 20 2012 |
  | Extra | WOS:000309906900001 |
  | DOI | 10.1186/1471-2105-13-105 |
  | Abstract | Background: Multiple structure alignments have received increasing attention in recent years as an alternative to multiple sequence alignments. Although multiple structure alignment algorithms can potentially be applied to a number of problems, they have primarily been used for protein core identification. A method that is capable of solving a variety of problems using structure comparison is still absent. Here we introduce a program msTALI for aligning multiple protein structures. Our algorithm uses several informative features to guide its alignments: torsion angles, backbone C-a atom positions, secondary structure, residue type, surface accessibility, and properties of nearby atoms. The algorithm allows the user to weight the types of information used to generate the alignment, which expands its utility to a wide variety of problems. Results: msTALI exhibits competitive results on 824 families from the Homstrad and SABmark databases when compared to Matt and Mustang. We also demonstrate success at building a database of protein cores using 341 randomly selected CATH domains and highlight the contribution of msTALI compared to the CATH classifications. Finally, we present an example applying msTALI to the problem of detecting hinges in a protein undergoing rigid-body motion. Conclusions: msTALI is an effective algorithm for multiple structure alignment. In addition to its performance on standard comparison databases, it utilizes clear, informative features, allowing further customization for domain-specific applications. The C++ source code for msTALI is available for Linux on the web at http://ifestos.cse.sc.edu/mstali. |
  | Date Added | 2/13/2014, 4:13:17 PM |
  | Modified | 3/7/2014, 12:11:07 PM |
- ## MUSCLE: multiple sequence alignment with high accuracy and high throughput

  |  |  |
  | --- | --- |
  | Type | Journal Article |
  | Author | Robert C Edgar |
  | Volume | 32 |
  | Issue | 5 |
  | Pages | 1792-1797 |
  | Publication | Nucleic acids research |
  | ISSN | 1362-4962 |
  | Date | 2004 |
  | Extra | PMID: 15034147 |
  | Journal Abbr | Nucleic Acids Res. |
  | DOI | 10.1093/nar/gkh340 |
  | Library Catalog | NCBI PubMed |
  | Language | eng |
  | Abstract | We describe MUSCLE, a new computer program for creating multiple alignments of protein sequences. Elements of the algorithm include fast distance estimation using kmer counting, progressive alignment using a new profile function we call the log-expectation score, and refinement using tree-dependent restricted partitioning. The speed and accuracy of MUSCLE are compared with T-Coffee, MAFFT and CLUSTALW on four test sets of reference alignments: BAliBASE, SABmark, SMART and a new benchmark, PREFAB. MUSCLE achieves the highest, or joint highest, rank in accuracy on each of these sets. Without refinement, MUSCLE achieves average accuracy statistically indistinguishable from T-Coffee and MAFFT, and is the fastest of the tested methods for large numbers of sequences, aligning 5000 sequences of average length 350 in 7 min on a current desktop computer. The MUSCLE program, source code and PREFAB test data are freely available at http://www.drive5. com/muscle. |
  | Short Title | MUSCLE |
  | Date Added | 10/11/2013, 10:29:15 AM |
  | Modified | 10/11/2013, 10:29:15 AM |

  ### Tags:

  - ASTRAL sequences
  - ASTRAL subsets

  ### Notes:

  - MUSCLE is a method for multiple sequence alignment.

    How SCOP is used:

    Benchmark method using SABmark data, which was derived from ASTRAL.

    Use representative data sets taken from ASTRAL, then classified by SCOP folds and superfamilies.

    SCOP reference:

    SABmark. We used version 1.63 of the SABmark reference alignments, which consists of two subsets: Superfamily and Twilight. All sequences have known structure. The Twilight set contains 1994 domains from the Astral database (26) with pairwise sequence similarity e-values `1, divided into 236 folds according to the SCOP classification (27). The Superfamily set contains sequences of pairwise identity `50%, divided into 462 SCOP superfamilies. Each pair of structures was aligned with two structural aligners: SOFI (28) and CE (29), producing a sequence alignment from the consensus in which only high-con®dence regions are retained. Input sets range from three to 25 sequences, with an average of eight and an average sequence length of 179.

  ### Attachments

  - Full Text PDF
- ## MUSTER: Improving protein sequence profile-profile alignments by using multiple sources of structure information

  |  |  |
  | --- | --- |
  | Type | Journal Article |
  | Author | Sitao Wu |
  | Author | Yang Zhang |
  | Volume | 72 |
  | Issue | 2 |
  | Pages | 547-556 |
  | Publication | Proteins |
  | ISSN | 1097-0134 |
  | Date | Aug 2008 |
  | Extra | PMID: 18247410 |
  | Journal Abbr | Proteins |
  | DOI | 10.1002/prot.21945 |
  | Library Catalog | NCBI PubMed |
  | Language | eng |
  | Abstract | We develop a new threading algorithm MUSTER by extending the previous sequence profile-profile alignment method, PPA. It combines various sequence and structure information into single-body terms which can be conveniently used in dynamic programming search: (1) sequence profiles; (2) secondary structures; (3) structure fragment profiles; (4) solvent accessibility; (5) dihedral torsion angles; (6) hydrophobic scoring matrix. The balance of the weighting parameters is optimized by a grading search based on the average TM-score of 111 training proteins which shows a better performance than using the conventional optimization methods based on the PROSUP database. The algorithm is tested on 500 nonhomologous proteins independent of the training sets. After removing the homologous templates with a sequence identity to the target >30%, in 224 cases, the first template alignment has the correct topology with a TM-score >0.5. Even with a more stringent cutoff by removing the templates with a sequence identity >20% or detectable by PSI-BLAST with an E-value <0.05, MUSTER is able to identify correct folds in 137 cases with the first model of TM-score >0.5. Dependent on the homology cutoffs, the average TM-score of the first threading alignments by MUSTER is 5.1-6.3% higher than that by PPA. This improvement is statistically significant by the Wilcoxon signed rank test with a P-value < 1.0 x 10(-13), which demonstrates the effect of additional structural information on the protein fold recognition. The MUSTER server is freely available to the academic community at http://zhang.bioinformatics.ku.edu/MUSTER. |
  | Short Title | MUSTER |
  | Date Added | 10/11/2013, 10:29:15 AM |
  | Modified | 10/11/2013, 10:29:15 AM |

  ### Tags:

  - likely ASTRAL
  - likely ASTRAL domain structures

  ### Notes:

  - MUSTER is a new threading algorithm.

    How SCOP is used:

    Used SCOP data to train 9 parameters of MUSTER method.

    Derived a dataset of pairs of structures from 558  randomly chosen SCOP families.  Each pair has a TM-score>0.5.  120 pairs are in the same fold, but not SF.  70 pairs are in the same SF, but not family.

     SCOP reference:

    Because the number of protein pairs in PROSUP is rel- atively small (110 with TM-score >0.5) compared with the number of free parameters in MUSTER, we add a new set of 190 nonredundant protein structure pairs to our training set. These 190 pairs are selected with a TM-score >0.5 from 558 randomly chosen SCOP families,59 where 120 pairs share the same ‘‘class’’ and ‘‘fold’’ but different ‘‘super-family’’ and 70 pairs share the same ‘‘class,’’ ‘‘fold,’’ and ‘‘super-family’’ but different ‘‘family.’’

  ### Attachments

  - muster\_2008.pdf
  - PubMed entry
- ## Mutational Analysis of the Binding Pockets of the Diketo Acid Inhibitor L-742,001 in the Influenza Virus PA Endonuclease

  |  |  |
  | --- | --- |
  | Type | Journal Article |
  | Author | Annelies Stevaert |
  | Author | Roberto Dallocchio |
  | Author | Alessandro Dessi |
  | Author | Nicolino Pala |
  | Author | Dominga Rogolino |
  | Author | Mario Sechi |
  | Author | Lieve Naesens |
  | Volume | 87 |
  | Issue | 19 |
  | Pages | 10524–10538 |
  | Publication | Journal of Virology |
  | Date | October 2013 |
  | DOI | 10.1128/JVI.00832-13 |
  | Abstract | The influenza virus PA endonuclease, which cleaves capped host pre-mRNAs to initiate synthesis of viral mRNA, is a prime target for antiviral therapy. The diketo acid compound L-742,001 was previously identified as a potent inhibitor of the influenza virus endonuclease reaction, but information on its precise binding mode to PA or potential resistance profile is limited. Computer-assisted docking of L-742,001 into the crystal structure of inhibitor-free N-terminal PA (PA-Nter) indicated a binding orientation distinct from that seen in a recent crystallographic study with L-742,001-bound PA-Nter (R. M. DuBois et al., PLoS Pathog. 8:e1002830, 2012). A comprehensive mutational analysis was performed to determine which amino acid changes within the catalytic center of PA or its surrounding hydrophobic pockets alter the antiviral sensitivity to L-742,001 in cell culture. Marked (up to 20-fold) resistance to L-742,001 was observed for the H41A, I120T, and G81F/V/T mutant forms of PA. Two- to 3-fold resistance was seen for the T20A, L42T, and V122T mutants, and the R124Q and Y130A mutants were 3-fold more sensitive to L-742,001. Several mutations situated at noncatalytic sites in PA had no or only marginal impact on the enzymatic functionality of viral ribonucleoprotein complexes reconstituted in cell culture, consistent with the less conserved nature of these PA residues. Our data provide relevant insights into the binding mode of L-742,001 in the PA endonuclease active site. In addition, we predict some potential resistance sites that should be taken into account during optimization of PA endonuclease inhibitors toward tight binding in any of the hydrophobic pockets surrounding the catalytic center of the enzyme. |
  | Date Added | 3/7/2014, 1:06:24 PM |
  | Modified | 3/7/2014, 1:06:24 PM |
- ## Native N-Terminus Nitrophorin 2 from the Kissing Bug: Similarities to and Differences from NP2(D1A)

  |  |  |
  | --- | --- |
  | Type | Journal Article |
  | Author | Robert E. Berry |
  | Author | Dhanasekaran Muthu |
  | Author | Tatiana K. Shokhireva |
  | Author | Sarah A. Garrett |
  | Author | Hongjun Zhang |
  | Author | F. Ann Walker |
  | Volume | 9 |
  | Issue | 9 |
  | Pages | 1739-1755 |
  | Publication | Chemistry & Biodiversity |
  | ISSN | 1612-1872 |
  | Date | SEP 2012 |
  | Extra | WOS:000308715800010 |
  | DOI | 10.1002/cbdv.201100449 |
  | Abstract | The first amino acid of mature native nitrophorin 2 is aspartic acid, and when expressed in E. coli, the wild-type gene of the mature protein retains the methionine-0, which is produced by translation of the start codon. This form of NP2, (M0)NP2, has been found to have different properties from its D1A mutant, for which the Met0 is cleaved by the methionine aminopeptidase of E. coli (R. E. Berry, T. K. Shokhireva, I. Filippov, M. N. Shokhirev, H. Zhang, F. A. Walker, Biochemistry 2007, 46, 6830). Native N-terminus nitrophorin 2 ((?M0)NP2) has been prepared by employing periplasmic expression of NP2 in E. coli using the pelB leader sequence from Erwinia carotovora, which is present in the pET-26b expression plasmid (Novagen). This paper details the similarities and differences between the three different N-terminal forms of nitrophorin 2, (M0)NP2, NP2(D1A), and (?M0)NP2. It is found that the NMR spectra of high- and low-spin (?M0)NP2 are essentially identical to those of NP2(D1A), but the rate and equilibrium constants for histamine and NO dissociation/association of the two are different. |
  | Date Added | 2/13/2014, 4:13:17 PM |
  | Modified | 3/7/2014, 12:15:27 PM |
- ## Nature of the Protein Universe

  |  |  |
  | --- | --- |
  | Type | Journal Article |
  | Author | Michael Levitt |
  | URL | http://www.jstor.org/stable/40483751 |
  | Volume | 106 |
  | Issue | 27 |
  | Pages | 11079-11084 |
  | Publication | Proceedings of the National Academy of Sciences of the United States of America |
  | ISSN | 0027-8424 |
  | Date | July 07, 2009 |
  | Extra | ArticleType: research-article / Full publication date: Jul. 7, 2009 / Copyright © 2009 National Academy of Sciences |
  | Accessed | 11/7/2012, 2:00:33 PM |
  | Library Catalog | JSTOR |
  | Abstract | The protein universe is the set of all proteins of all organisms. Here, all currently known sequences are analyzed in terms of families that have single-domain or multidomain architectures and whether they have a known three-dimensional structure. Growth of new single-domain families is very slow: Almost all growth comes from new multidomain architectures that are combinations of domains characterized by ≈ 15,000 sequence profiles. Singledomain families are mostly shared by the major groups of organisms, whereas multidomain architectures are specific and account for species diversity. There are known structures for a quarter of the single-domain families, and > 70% of all sequences can be partially modeled thanks to their membership in these families. |
  | Date Added | 10/11/2013, 10:29:15 AM |
  | Modified | 10/11/2013, 10:29:15 AM |

  ### Tags:

  - Amino Acid Sequence
  - Databases, Protein
  - domain architecture
  - Evolution, Molecular
  - Models, Molecular
  - Multigene Family
  - Proteins
  - protein sequence
  - protein structure
  - Protein Structure, Tertiary
  - structural genomics
  - Structural Homology, Protein

  ### Notes:

  - This paper discusses what is known and not know about the distribution of the protein universe into protein families.  
      
    First, the distribution of protein families in single-domain architecture (SDA) and multi-domain architecture (MDA) families is compared.  Almost all growth comes from new MDAs.  SDAs are commonly shared between the three major organism groups of life: prokaryotes, eukaryotes, and viruses, while MDAs tend to be unique to the group.  
      
    Another interesting topic discussed in the paper is the prevalence of 'dark matter'.  78% of all known sequences longer than 50 aa can be classified into a family, by matching all or part of the sequence to a sequence profile.  The remaining 22% is uncharacterized and considered as 'dark matter'.  The dark matter may be a result of (1) DNA-deduced protein sequences are not real; (2) these are low-complexity, non-globular protein sequences (3) sequences may belong to a known family, but pattern matching methods are unable to detect them (4) new  
    families within the dark matter remain to be discovered  
      
    Levitt's list of the main implications of this study is:

    1. Because homology modeling is now well developed, improved ability to recognize and model sequence would reduce the amount of additional experimental structure determinations.
    2. Dark matter needs to be analyzed for new sequence profiles.
    3. Frequent updates of sequence profile databases are needed to keep up with rapid growth of the number of sequences, doubling every 28 months.

    How SCOP is used:

    Points to some  earlier work that investigates the growth of single-domain architectures in SCOP.

  ### Attachments

  - JSTOR Full Text PDF
  - PubMed entry
- ## Negatively Cooperative Binding Properties of Human Cytochrome P450 2E1 with Monocyclic Substrates

  |  |  |
  | --- | --- |
  | Type | Journal Article |
  | Author | Jie Ping |
  | Author | Ya-Jun Wang |
  | Author | Jing-Fang Wang |
  | Author | Xuan Li |
  | Author | Yi-Xue Li |
  | Author | Pei Hao |
  | Volume | 13 |
  | Issue | 7 |
  | Pages | 1024-1031 |
  | Publication | Current Drug Metabolism |
  | ISSN | 1389-2002 |
  | Date | SEP 2012 |
  | Extra | WOS:000307743000017 |
  | Abstract | Human CYP2E1 accounts for almost 2% of total CYP enzymes in the liver cells, and plays a crucial role in the metabolism of small molecular weight compounds. This enzyme is associated with the nearly 6% metabolisms of the currently clinical drugs. However, it is found that CYP2E1 has a non-hyperbolic kinetic profile that can not be explained by the common Michaelis-Menten mechanism. Further studies show that the non-hyperbolic kinetic behaviors are associated with multiple substrate binding, which is also known as the cooperative binding properties. However, the detailed mechanism for the cooperative binding is not clear by now. In this paper, we summarized the experimental and theoretical studies on the cooperative binding mechanism. Based on the structural analysis, a second substrate binding site is confirmed in human CYP2E1, which is located neither in the region near Leu103, Leu210 and Phe478, nor far from the active site. Additionally, two important residues Thr303 and Phe478 are also identified to be the key factors in the cooperative binding on the short-range and long-range effects, respectively. The former plays a crucial role in the positioning of substrates and in proton delivery to the active site; the latter is located between the substrate access channel and the active site, and exhibits directly effects on substrate access or on substrate positioning in the active site. All these points can provide useful information for the cooperative binding in human CYP2E1, revealing the detailed mechanism for the non-hyperbolic kinetic behaviors. |
  | Date Added | 2/13/2014, 4:13:17 PM |
  | Modified | 3/7/2014, 12:14:05 PM |
- ## Networks of Protein-Protein Interactions: From Uncertainty to Molecular Details

  |  |  |
  | --- | --- |
  | Type | Journal Article |
  | Author | Javier Garcia-Garcia |
  | Author | Jaume Bonet |
  | Author | Emre Guney |
  | Author | Oriol Fornes |
  | Author | Joan Planas |
  | Author | Baldo Oliva |
  | Volume | 31 |
  | Issue | 5 |
  | Pages | 342-362 |
  | Publication | Molecular Informatics |
  | Date | MAY 2012 |
  | Extra | WOS:000303857900002 |
  | DOI | 10.1002/minf.201200005 |
  | Library Catalog | ISI Web of Knowledge |
  | Abstract | Proteins are the bricks and mortar of cells. The work of proteins is structural and functional, as they are the principal element of the organization of the cell architecture, but they also play a relevant role in its metabolism and regulation. To perform all these functions, proteins need to interact with each other and with other bio-molecules, either to form complexes or to recognize precise targets of their action. For instance, a particular transcription factor may activate one gene or another depending on its interactions with other proteins and not only with DNA. Hence, the ability of a protein to interact with other bio-molecules, and the partners they have at each particular time and location can be crucial to characterize the role of a protein. Proteins rarely act alone; they rather constitute a mingled network of physical interactions or other types of relationships (such as metabolic and regulatory) or signaling cascades. In this context, understanding the function of a protein implies to recognize the members of its neighborhood and to grasp how they associate, both at the systemic and atomic level. The network of physical interactions between the proteins of a system, cell or organism, is defined as the interactome. The purpose of this review is to deepen the description of interactomes at different levels of detail: from the molecular structure of complexes to the global topology of the network of interactions. The approaches and techniques applied experimentally and computationally to attain each level are depicted. The limits of each technique and its integration into a model network, the challenges and actual problems of completeness of an interactome, and the reliability of the interactions are reviewed and summarized. Finally, the application of the current knowledge of protein-protein interactions on modern network medicine and protein function annotation is also explored. |
  | Short Title | Networks of Protein-Protein Interactions |
  | Date Added | 10/8/2014, 12:49:22 PM |
  | Modified | 10/8/2014, 1:32:23 PM |

  ### Tags:

  - Computational methods
  - Experimental methods
  - Interactome
  - Interface
  - Protein interactions

  ### Notes:

  - Review on research on protein-protein interaction networks.

    How SCOP/CATH is used:

    For background on networks that rely on SCOP.

    SCOP reference:

    SCOPPI, SCOWLP and InterPare are based on SCOP domains [156]. PIBASE is based relies on SCOP and CATH [157] domains.

  ### Attachments

  - 342\_ftp.pdf
- ## New families of carboxyl peptidases: serine-carboxyl peptidases and glutamic peptidases

  |  |  |
  | --- | --- |
  | Type | Journal Article |
  | Author | Kohei Oda |
  | URL | http://jb.oxfordjournals.org/content/151/1/13.short |
  | Volume | 151 |
  | Issue | 1 |
  | Pages | 13–25 |
  | Publication | Journal of biochemistry |
  | Date | 2012 |
  | Accessed | 9/23/2013, 10:15:49 AM |
  | Library Catalog | Google Scholar |
  | Short Title | New families of carboxyl peptidases |
  | Date Added | 2/20/2014, 12:24:01 PM |
  | Modified | 2/20/2014, 12:24:01 PM |

  ### Notes:

  - Review of two studies on recently discovered peptidase families.

    How SCOP is used:

    Look for similar structures using 3D structure comparison.

    SCOP reference:

    Other unique features revealed by the structural analysis are as follows: (i) Topological and three- dimensional structural comparisons reveal that the b-sandwich fold of eqolisin is similar to the members of the concanavalin A-like lectins/glucanases super- family (69).

  ### Attachments

  - J Biochem-2012-Oda-13-25.pdf
  - Snapshot
- ## New functional families (FunFams) in CATH to improve the mapping of conserved functional sites to 3D structures

  |  |  |
  | --- | --- |
  | Type | Journal Article |
  | Author | Ian Sillitoe |
  | Author | Alison L Cuff |
  | Author | Benoit H Dessailly |
  | Author | Natalie L Dawson |
  | Author | Nicholas Furnham |
  | Author | David Lee |
  | Author | Jonathan G Lees |
  | Author | Tony E Lewis |
  | Author | Romain A Studer |
  | Author | Robert Rentzsch |
  | Author | Corin Yeats |
  | Author | Janet M Thornton |
  | Author | Christine A Orengo |
  | Volume | 41 |
  | Issue | Database issue |
  | Pages | D490-498 |
  | Publication | Nucleic Acids Research |
  | ISSN | 1362-4962 |
  | Date | Jan 2013 |
  | Extra | PMID: 23203873 |
  | Journal Abbr | Nucleic Acids Res. |
  | DOI | 10.1093/nar/gks1211 |
  | Library Catalog | NCBI PubMed |
  | Language | eng |
  | Abstract | CATH version 3.5 (Class, Architecture, Topology, Homology, available at http://www.cathdb.info/) contains 173 536 domains, 2626 homologous superfamilies and 1313 fold groups. When focusing on structural genomics (SG) structures, we observe that the number of new folds for CATH v3.5 is slightly less than for previous releases, and this observation suggests that we may now know the majority of folds that are easily accessible to structure determination. We have improved the accuracy of our functional family (FunFams) sub-classification method and the CATH sequence domain search facility has been extended to provide FunFam annotations for each domain. The CATH website has been redesigned. We have improved the display of functional data and of conserved sequence features associated with FunFams within each CATH superfamily. |
  | Date Added | 2/20/2014, 12:24:01 PM |
  | Modified | 5/5/2014, 3:12:50 PM |

  ### Tags:

  - Databases, Protein
  - Genomics
  - Internet
  - Molecular Sequence Annotation
  - Protein Folding
  - Proteins
  - Protein Structure, Tertiary
  - Sequence Alignment
  - Sequence Analysis, Protein
  - Structural Homology, Protein

  ### Notes:

  - Present latest release of CATH.

    How SCOP is used:

    Examine consistency of SCOP and CATH data.  Discuss why they are different.

    SCOP reference:

    COMPARISONS BETWEEN CATH AND SCOP

    CATH and Structural Classifications of Proteins (SCOP) (3) are the two most comprehensive protein structure classification resources. Both are in active development. The latest release of SCOP (v1.75) classifies 110800 domains (38,221 PDB entries) compared with over 173000 (51,334 PDB entries) for CATH. Currently, CATH has 1313 folds classified compared with 1195 for SCOP, but comparisons at this level are problematic, as more subjective criteria are used in fold classification.

    Recent analysis has shown that, if one applies relatively conservative thresholds to identify equivalent superfamilies between the two resources (i.e. a 60% overlap between matching domains identified in the same PDB chain and 60% of these matching domains grouped into equivalent superfamilies), ⬚⬚800 superfamilies correspond between SCOP and CATH. A new initiative, Genome3D, is enabling collaboration between the SCOP and CATH groups to refine the iden- tification of equivalent superfamilies and to present infor- mation on philosophical differences between the resources that lead to alternative ways of grouping relatives. There is much less agreement at the fold level, again because of the subjective manner in which fold is defined.

  ### Attachments

  - gks1211.pdf
- ## New sub-family of lysozyme-like proteins shows no catalytic activity: crystallographic and biochemical study of STM3605 protein from Salmonella Typhimurium

  |  |  |
  | --- | --- |
  | Type | Journal Article |
  | Author | Karolina Michalska |
  | Author | Roslyn N. Brown |
  | Author | Hui Li |
  | Author | Robert Jedrzejczak |
  | Author | George S. Niemann |
  | Author | Fred Heffron |
  | Author | John R. Cort |
  | Author | Joshua N. Adkins |
  | Author | Gyorgy Babnigg |
  | Author | Andrzej Joachimiak |
  | Volume | 14 |
  | Issue | 1 |
  | Pages | 1–10 |
  | Publication | Journal of Structural and Functional Genomics |
  | Date | March 2013 |
  | DOI | 10.1007/s10969-013-9151-0 |
  | Abstract | Phage viruses that infect prokaryotes integrate their genome into the host chromosome; thus, microbial genomes typically contain genetic remnants of both recent and ancient phage infections. Often phage genes occur in clusters of atypical G+C content that reflect integration of the foreign DNA. However, some phage genes occur in isolation without other phage gene neighbors, probably resulting from horizontal gene transfer. In these cases, the phage gene product is unlikely to function as a component of a mature phage particle, and instead may have been co-opted by the host for its own benefit. The product of one such gene from Salmonella enterica serovar Typhimurium, STM3605, encodes a protein with modest sequence similarity to phage-like lysozyme (N-acetylmuramidase) but appears to lack essential catalytic residues that are strictly conserved in all lysozymes. Close homologs in other bacteria share this characteristic. The structure of the STM3605 protein was characterized by X-ray crystallography, and functional assays showed that it is a stable, folded protein whose structure closely resembles lysozyme. However, this protein is unlikely to hydrolyze peptidoglycan. Instead, STM3605 is presumed to have evolved an alternative function because it shows some lytic activity and partitions to micelles. |
  | Date Added | 3/7/2014, 12:08:00 PM |
  | Modified | 3/7/2014, 12:08:00 PM |
- ## NMR Structure of Lipoprotein YxeF from Bacillus subtilis Reveals a Calycin Fold and Distant Homology with the Lipocalin Blc from Escherichia coli

  |  |  |
  | --- | --- |
  | Type | Journal Article |
  | Author | Yibing Wu |
  | Author | Marco Punta |
  | Author | Rong Xiao |
  | Author | Thomas B. Acton |
  | Author | Bharathwaj Sathyamoorthy |
  | Author | Fabian Dey |
  | Author | Markus Fischer |
  | Author | Arne Skerra |
  | Author | Burkhard Rost |
  | Author | Gaetano T. Montelione |
  | Author | Thomas Szyperski |
  | Volume | 7 |
  | Issue | 6 |
  | Pages | e37404 |
  | Publication | Plos One |
  | ISSN | 1932-6203 |
  | Date | JUN 5 2012 |
  | Extra | WOS:000305343900007 |
  | DOI | 10.1371/journal.pone.0037404 |
  | Abstract | The soluble monomeric domain of lipoprotein YxeF from the Gram positive bacterium B. subtilis was selected by the Northeast Structural Genomics Consortium (NESG) as a target of a biomedical theme project focusing on the structure determination of the soluble domains of bacterial lipoproteins. The solution NMR structure of YxeF reveals a calycin fold and distant homology with the lipocalin Blc from the Gram-negative bacterium E. coli. In particular, the characteristic beta-barrel, which is open to the solvent at one end, is extremely well conserved in YxeF with respect to Blc. The identification of YxeF as the first lipocalin homologue occurring in a Gram-positive bacterium suggests that lipocalins emerged before the evolutionary divergence of Gram positive and Gram negative bacteria. Since YxeF is devoid of the alpha-helix that packs in all lipocalins with known structure against the beta-barrel to form a second hydrophobic core, we propose to introduce a new lipocalin sub-family named 'slim lipocalins', with YxeF and the other members of Pfam family PF11631 to which YxeF belongs constituting the first representatives. The results presented here exemplify the impact of structural genomics to enhance our understanding of biology and to generate new biological hypotheses. |
  | Date Added | 2/20/2014, 12:24:01 PM |
  | Modified | 3/7/2014, 12:10:31 PM |

  ### Notes:

  - Present NMR structure of lipoprotein.

    How SCOP/CATH is used:

    Look up proteins of interest in SCOP and CATH.

    SCOP reference:

    Current Classification of YxeF Structure in the CATH, SCOP and Pfam Databases

    Inspection of the YxeF structure (Figure 2) shows that it resembles b-barrel proteins belonging to the ‘calycin superfamily’ which includes lipocalins, fatty acid binding proteins, triabin, avidins/streptavidins and a class of metalloprotease inhibitors. All calycins contain a calyx-like b-barrel characterized by a +1 up- and-down topology (Figure 4), with triabin being the only exception due to a b-strand swap, and fatty acid-binding proteins featuring two additional b-strands in the barrel with respect to other calycins (i.e., 10-stranded instead of 8-stranded) [5,6]. The b- barrels structurally characterizing calycins are open to the solvent on one side and often harbor a ligand-binding site [6,7].

    Accordingly, our YxeF structure (Figure 2) has been incorpo- rated in the CATH (class architecture topology homologous superfamily) and SCOP (structurally classification of proteins) databases [8,9]. In CATH, it is part of the Homology sub-level 2.40.128.20 within the ‘lipocalin’ Topology. This Homology sub- level incorporates lipocalins, fatty acid binding proteins and triabin, while the ‘lipocalin’ Topology includes all other calycins together with additional members such as some outer membrane proteins. **In SCOP, YxeF is assigned to the ‘retinol binding protein-like’ family, containing all lipocalins of known structure. This family is found within the ‘lipocalin’ SCOP** **super****family further including fatty acid binding proteins and triabin.** **Avidin/ streptavidin and metalloprotease inhibitors are instead assigned to a different SCOP fold (****i.e.****, ‘streptavidin-like’).** Finally, in the Pfam sequence database lipocalins are grouped with fatty acid-binding proteins in several families within the ‘calycin superfamily’ clan [10], which additionally includes triabin. Avidins/streptavidins and metalloprotease inhibitors are not considered to be part of the ‘calycin superfamily’. These classifications are (i) based on both sequence and structure comparisons, (ii) rely, at least to some degree, on manual curation, and (iii) favor the hypothesis that an evolutionary link exists between lipocalins, fatty acid binding proteins and triabin. They leave, however, the tetrameric avidins/ streptavidins and some metalloprotease inhibitors in limbo with respect to their relationship to the other proteins alluded to above.

    **SCOP further identifies lipocalins as a sub-group of more closely related proteins and places YxeF among them.** Lipocalins are extracellular (sometimes membrane anchored) proteins known to generally transport and store small, largely hydrophobic compounds within a ligand pocket surrounded by four loops at the open end of the b-barrel [5,11]. Despite sharing with lipocalins the same b-barrel topology YxeF lacks a C-terminal a-helix (Figure 4A,B) which, in all lipocalins with known structure, packs against one side of the b-barrel. This observation raises the question of whether and how YxeF is evolutionary related to lipocalins. One of the key challenges associated with classifying calycin2/lipocalin-like proteins is their typically very low (i.e., insignificant) sequence identity, so that quite often homology cannot be inferred from sequence alone [5,6]. Furthermore, the manifold of known eight stranded b-barrels appears to form what has been named a structural ‘quasi-continuum’ [12]. This greatly impedes the identification of boundaries between divergent and convergent evolutionary links. In the following, we present a structural bioinformatics analysis aimed at resolving the YxeF structure classification and elucidating YxeF’s evolutionary origin.

    ...

    Staphostatin B is classified in SCOP as having a ‘Streptavidin-like’ fold but high structural similarity to lipocalins was recognized previously [18].

  ### Attachments

  - journal.pone.0037404.pdf
- ## Novel approach for selecting the best predictor for identifying the binding sites in DNA binding proteins

  |  |  |
  | --- | --- |
  | Type | Journal Article |
  | Author | R. Nagarajan |
  | Author | Shandar Ahmad |
  | Author | M. Michael Gromiha |
  | Volume | 41 |
  | Issue | 16 |
  | Pages | 7606-7614 |
  | Publication | Nucleic Acids Research |
  | ISSN | 0305-1048 |
  | Date | SEP 2013 |
  | Extra | WOS:000325173300011 |
  | DOI | 10.1093/nar/gkt544 |
  | Abstract | Protein-DNA complexes play vital roles in many cellular processes by the interactions of amino acids with DNA. Several computational methods have been developed for predicting the interacting residues in DNA-binding proteins using sequence and/or structural information. These methods showed different levels of accuracies, which may depend on the choice of data sets used in training, the feature sets selected for developing a predictive model, the ability of the models to capture information useful for prediction or a combination of these factors. In many cases, different methods are likely to produce similar results, whereas in others, the predictors may return contradictory predictions. In this situation, a priori estimates of prediction performance applicable to the system being investigated would be helpful for biologists to choose the best method for designing their experiments. In this work, we have constructed unbiased, stringent and diverse data sets for DNA-binding proteins based on various biologically relevant considerations: (i) seven structural classes, (ii) 86 folds, (iii) 106 superfamilies, (iv) 194 families, (v) 15 binding motifs, (vi) single/double-stranded DNA, (vii) DNA conformation (A, B, Z, etc.), (viii) three functions and (ix) disordered regions. These data sets were culled as non-redundant with sequence identities of 25 and 40% and used to evaluate the performance of 11 different methods in which online services or standalone programs are available. We observed that the best performing methods for each of the data sets showed significant biases toward the data sets selected for their benchmark. Our analysis revealed important data set features, which could be used to estimate these context-specific biases and hence suggest the best method to be used for a given problem. We have developed a web server, which considers these features on demand and displays the best method that the investigator should use. The web server is freely available at http://www.biotech.iitm.ac.in/DNA-protein/. Further, we have grouped the methods based on their complexity and analyzed the performance. The information gained in this work could be effectively used to select the best method for designing experiments. |
  | Date Added | 2/12/2014, 1:36:22 PM |
  | Modified | 2/12/2014, 1:36:22 PM |

  ### Notes:

  - Present method for selecting the best context-specific method for predicting DNA-binding sites.

    How SCOP is used:

    Evaluate methods on a data set classified by structural class, folds, superfamilies, and families.

    SCOP reference:

    Classification based on protein structure

    We have used the SCOP database (52) for structural classification of proteins based on their structural classes, folding types, superfamilies and families. Our final data set contains 260 protein chains from seven classes, 86 folds, 106 superfamilies and 194 families with the sequence identity of <25%.

    Further, we have identified the disordered regions by comparing the structures of proteins in free and complex forms and analyzed the performance of different methods in disordered regions.

  ### Attachments

  - Nucl. Acids Res.-2013-Nagarajan-7606-14.pdf
- ## Novel autoproteolytic and DNA-damage sensing components in the bacterial SOS response and oxidized methylcytosine-induced eukaryotic DNA demethylation systems

  |  |  |
  | --- | --- |
  | Type | Journal Article |
  | Author | L. Aravind |
  | Author | Swadha Anand |
  | Author | Lakshminarayan M. Iyer |
  | Volume | 8 |
  | Publication | Biology Direct |
  | ISSN | 1745-6150 |
  | Date | AUG 15 2013 |
  | Extra | WOS:000323577100001 |
  | DOI | 10.1186/1745-6150-8-20 |
  | Abstract | The bacterial SOS response is an elaborate program for DNA repair, cell cycle regulation and adaptive mutagenesis under stress conditions. Using sensitive sequence and structure analysis, combined with contextual information derived from comparative genomics and domain architectures, we identify two novel domain superfamilies in the SOS response system. We present evidence that one of these, the SOS response associated peptidase (SRAP; Pfam: DUF159) is a novel thiol autopeptidase. Given the involvement of other autopeptidases, such as LexA and UmuD, in the SOS response, this finding suggests that multiple structurally unrelated peptidases have been recruited to this process. The second of these, the ImuB-C superfamily, is linked to the Y-family DNA polymerase-related domain in ImuB, and also occurs as a standalone protein. We present evidence using gene neighborhood analysis that both these domains function with different mutagenic polymerases in bacteria, such as Pol |
  | Date Added | 10/28/2013, 4:51:00 PM |
  | Modified | 10/28/2013, 4:51:00 PM |

  ### Notes:

  - Computational study of proteins in bacterial SOS response.  Identify two novel superfamilies in the SOS response system.

    How SCOP is used:

    look up fold classification in SCOP

    SCOP reference:

    Examination of the structure of the SRAP domain re- veals that it assumes a unique fold (BB1717-like fold in the SCOP database) that appears to have been constituted from 5 repeats of a β-hairpin [19].

  ### Attachments

  - 1745-6150-8-20.pdf
- ## Novel inositol catabolic pathway in Thermotoga maritima

  |  |  |
  | --- | --- |
  | Type | Journal Article |
  | Author | Irina A. Rodionova |
  | Author | Semen A. Leyn |
  | Author | Michael D. Burkart |
  | Author | Nathalie Boucher |
  | Author | Kenneth M. Noll |
  | Author | Andrei L. Osterman |
  | Author | Dmitry A. Rodionov |
  | URL | http://onlinelibrary.wiley.com/doi/10.1111/1462-2920.12096/full |
  | Publication | Environmental microbiology |
  | Date | 2013 |
  | Accessed | 9/20/2013, 1:17:19 PM |
  | Library Catalog | Google Scholar |
  | Date Added | 10/11/2013, 10:29:15 AM |
  | Modified | 10/11/2013, 10:29:15 AM |

  ### Notes:

  - Find a new path in the MI catabolic pathway in Thermotoga maritima. The study is the characterization of 4 enzymes through genome analysis, bioinformatics, and experimental research and detail the 3 new reactions that are found in this novel pathway.

    How SCOP is used:

    Searched SCOP for remote homologs using hhpred.

    SCOP Reference:

    The long range homology analysis and tertiary structure modelling  
    were performed using HHpred (Soding et al., 2005) and  
    I-TASSER (Roy et al., 2010) web tools, the Pfam database of  
    protein families (Punta et al., 2012) and SCOP database of  
    protein structures (Murzin et al., 1995).

  ### Attachments

  - emi12096.pdf
- ## NPIDB: nucleic acid-protein interaction database

  |  |  |
  | --- | --- |
  | Type | Journal Article |
  | Author | Dmitry D. Kirsanov |
  | Author | Olga N. Zanegina |
  | Author | Evgeniy A. Aksianov |
  | Author | Sergei A. Spirin |
  | Author | Anna S. Karyagina |
  | Author | Andrei V. Alexeevski |
  | Volume | 41 |
  | Issue | D1 |
  | Pages | D517-D523 |
  | Publication | Nucleic Acids Research |
  | ISSN | 0305-1048 |
  | Date | JAN 2013 |
  | Extra | WOS:000312893300073 |
  | DOI | 10.1093/nar/gks1199 |
  | Abstract | The Nucleic acid-Protein Interaction DataBase (http://npidb.belozersky.msu.ru/) contains information derived from structures of DNA-protein and RNA-protein complexes extracted from the Protein Data Bank (3846 complexes in October 2012). It provides a web interface and a set of tools for extracting biologically meaningful characteristics of nucleoprotein complexes. The content of the database is updated weekly. The current version of the Nucleic acid-Protein Interaction DataBase is an upgrade of the version published in 2007. The improvements include a new web interface, new tools for calculation of intermolecular interactions, a classification of SCOP families that contains DNA-binding protein domains and data on conserved water molecules on the DNA-protein interface. |
  | Date Added | 2/20/2014, 12:24:01 PM |
  | Modified | 2/20/2014, 12:24:01 PM |

  ### Notes:

  - Present update to a database: "The Nucleic acid—Protein Interaction DataBase (http://npidb.belozersky.msu.ru/) contains information derived from structures of DNA–protein and RNA–protein complexes extracted from the Protein Data Bank (3846 complexes in October 2012)."

    How SCOP is used:

    Annotate all proteins in the database with SCOP domains, class, fold, superfamily, and family.

    SCOP reference:

    Under abstract:

    The improvements include a new web interface, new tools for calculation of intermolecular interactions, a classification of SCOP families that contains DNA–binding protein domains and data on conserved water molecules on the DNA–protein interface.

    ...

    Information on SCOP domains is extracted by Perl scripts from SCOP parsable files (http://scop.mrc-lmb. cam.ac.uk/scop/parse/index.html), release 1.75.

    For each (Pfam or SCOP) domain, a structure file in PDB format is created. This file contains description of the domain itself and of segments of nucleic acid chains that are in contact with the domain. Sets of representatives of Pfam and SCOP families (one complex for each family containing at least one domain with a known X-ray structure) are created and stored. These representatives are chosen from the complexes with best resolution among all complexes representing each particular family.

    The NPIDB database contains comparative structural information on some SCOP families. Namely, there are 1847 SCOP domains in contact with double-stranded DNA with at least 10 complementary base pairs. Those 1847 domains represent 110 SCOP families. For each of these 110 families, all their representatives were extracted from the PDB, including those that were solved in the absence of DNA.

    ...

    For each interaction class, a set of representatives of families of the type (the subset of best resolution representatives of all SCOP families) is available.

    ...

    1. The list of SCOP families is designed as a tree of SCOP classes, folds, superfamilies and families. A hyperlink from each family name leads to a page containing a table analogous to a table of a Pfam family, and, for a number of families of DNA-recognizing domains contacting with a long (>10bp) double-stranded DNA, a description of the family. The description includes a structural superposition of all representatives of the family in the PDB, the corresponding multiple amino acid sequence alignment and an information on conserved water bridges on the protein–DNA interface.

       The list of interaction classes of DNA-recognizing SCOP domains contains hyperlinks to lists of SCOP families whose representatives demonstrate the certain mode of DNA–protein interaction.

  ### Attachments

  - Nucl. Acids Res.-2013-Kirsanov-D517-23.pdf
- ## N-Terminal Domains in Two-Domain Proteins Are Biased to Be Shorter and Predicted to Fold Faster Than Their C-Terminal Counterparts

  |  |  |
  | --- | --- |
  | Type | Journal Article |
  | Author | Etai Jacob |
  | Author | Ron Unger |
  | Author | Amnon Horovitz |
  | URL | http://www.sciencedirect.com/science/article/pii/S221112471300154X |
  | Publication | Cell reports |
  | Date | 2013 |
  | Accessed | 9/20/2013, 1:19:56 PM |
  | Library Catalog | Google Scholar |
  | Date Added | 10/11/2013, 10:29:15 AM |
  | Modified | 3/7/2014, 12:09:33 PM |

  ### Notes:

  - Study into the misfolding of proteins. They found that in protein with 2 or more domains, the N-terminal domain is smaller and tends to fold faster than C-terminal domain. This bias is more prominent in prokaryotes than eukaryotes. The study was done through computational analysis of the protein domains.

    How SCOP/CATH is used:

    Collect a data set of 2-domain proteins from SCOP and CATH.

    They restricted their data set to all proteins that had at least 2 domains in which both domains belonged to the same family.  The used domains from SCOP and CATH.  If there was more entry per family, results were averaged.

    The 2 domains combined had to make up at least 80% of the full protein and the linker region had to be 30 amino acids or less.

    SCOP Reference:

    UNDER METHODS:

    We restricted our analysis to two domain  
    proteins with known structure in which both domains  
    belong to the same family (as defined by the CATH [Orengo  
    et al., 1997] and SCOP [Murzin et al., 1995] databases) so that  
    the strong dependence of ACO and chain length on topology  
    would not mask a signal that arises from the domain order.  
    The analysis was carried out using both CATH and SCOP in  
    order to ensure that the ACO values that are calculated separately  
    for each domain do not depend on the choice of domain  
    boundaries that may differ in the two databases. We also  
    required that each domain is formed by a continuous sequence  
    of 50–300 residues and is, thus, in the range where ACO and  
    chain length were shown to have predictive value. Finally, we  
    only considered two-domain proteins in which the combined  
    length of the two domains is >80% of the length of the full protein,  
    and the linker connecting the two domains is less than 30  
    amino acids. Data for families with more than one member  
    were included in the analysis using their average so that large  
    families would not be overrepresented.  
    A significant tendency is observed for the ACO values of the  
    N-terminal domains in two-domain proteins (satisfying the  
    criteria described above) to be smaller than those of their neighboring  
    C-terminal domains (Figure 2). The values of the ratio  
    between the number of all two-domain proteins in SCOP and  
    CATH with a predicted faster-folding N-terminal domain and  
    the number of all those with a predicted faster-folding C-terminal

    domain (nACO(Nt) < ACO(Ct)/nACO(Ct) < ACO(Nt)) are 1.4 and 1.7,  
    respectively, with respective binomial test p values of 0.04  
    and 0.016. This tendency is observed for all domain classes  
    (a, b, a/b, and a+b) in both SCOP and CATH. The values of  
    nACO(Nt) < ACO(Ct)/nACO(Ct) < ACO(Nt) are 1.7, 1.5, and 1.8 for the  
    19, 28, and 44 respective members of the a, b, a/b, and a+b  
    classes in CATH, and 1.8, 1.3, and 1.4 for the 31, 46, and 88  
    members of these classes in SCOP. Importantly, the bias for  
    ACO values of the N-terminal domains in two-domain proteins  
    to be smaller than those of their neighboring C-terminal domains  
    is not due to differences in domain lengths because it is  
    observed also for proteins with domains of similar size (Table  
    S1). For example, the values of the ratio nACO(Nt) < ACO(Ct)/  
    nACO(Ct) < ACO(Nt) for all the two-domain proteins in CATH and  
    SCOP, when those with a difference of more than ten amino  
    acids in their domain lengths were excluded from the analysis,  
    are 1.6 and 1.4, respectively. The corresponding p values of  
    0.053 and 0.078 are, however, somewhat higher owing to the  
    smaller sizes of the data sets when only two-domain proteins  
    comprising domains with similar lengths are considered. We  
    also calculated Fisher’s exact test of independence p values to  
    determine to what extent ACO values contain information  
    beyond that which is provided by domain length. The respective  
    Fisher’s exact test p values of 0.32 and 0.798 for the case above  
    indicate that the bias in ACO values is not due to differences in  
    domain lengths (Table S1). In the case of relative contact order  
    (RCO) calculations (see Experimental Procedures) for twodomain  
    proteins with a difference of less than ten amino acids  
    in their domain lengths, the values of the ratio nRCO(Nt) < RCO(Ct)/  
    nRCO(Ct) < RCO(Nt) for the two-domain proteins in CATH and  
    SCOP are, as expected, similar to the corresponding values of  
    nACO(Nt) < ACO(Ct)/nACO(Ct) < ACO(Nt), but the dependence of the  
    bias on domain length is greater as reflected in the respective  
    Fisher’s exact test p values of 0.1 and 0.077 (Table S1). In summary,  
    therefore, two predictors of folding rate, domain length  
    and ACO, indicate independently of each other that N-terminal  
    domains in two-domain proteins tend to fold faster than their  
    neighboring C-terminal domains.

    Contact Order Analysis  
    Two databases of structural classification of proteins were used in the  
    analysis: (1) version 1.75A of SCOP (Murzin et al., 1995) that was downloaded  
    from http://scop.berkeley.edu/astral; and (2) version 3.4 of CATH (Orengo  
    et al., 1997) that was downloaded from http://release.cathdb.info. Only proteins  
    that contain two domains belonging to the same family (the lowest level  
    in the structural hierarchy as defined by CATH and SCOP) were included in the  
    analysis. In addition, we considered only proteins in which the length of each  
    domain is between 50 and 300 residues and where the combined lengths of  
    the two domains are >80% of the length of the PDB entry and that the length  
    of the linker is less than 30 amino acids. In cases where different two-domain

    proteins contain the same domain, we required in order to avoid redundancy  
    that the nonshared domains differ in sequence by at least 5% (using other cutoffs  
    did not alter the results). This process yielded 454 entries for 174 domain  
    families in SCOP and 1,247 entries (808 of which belong to the immunoglobulins)  
    for 92 domain families in CATH. Data for families with more than one  
    member were included in the analysis using their average so that large families  
    (e.g., the immunoglobulins) would not be overrepresented.  
    ACO was calculated as described by Plaxco et al. (1998) and Galzitskaya  
    et al. (2003) using the script written by Erik Alm that was downloaded from  
    the website http://depts.washington.edu/bakerpg/contact\_order. ACO is the  
    average sequence separation between contacting residues in the native structure  
    and is given by  
    ACO=  
    1  
    N  
    X  
    N  
    DSi; j ;  
    where N is the number of contacts in the native structure, and DSi;j is the  
    number of amino acids between residues i and j that are in contact. The  
    RCO is equal to ACO/L, where L is the length of the protein.

  ### Attachments

  - 1-s2.0-S221112471300154X-main.pdf
- ## Octarellin VI: Using Rosetta to Design a Putative Artificial (beta/alpha)(8) Protein

  |  |  |
  | --- | --- |
  | Type | Journal Article |
  | Author | Maximiliano Figueroa |
  | Author | Nicolas Oliveira |
  | Author | Annabelle Lejeune |
  | Author | Kristian W. Kaufmann |
  | Author | Brent M. Dorr |
  | Author | Andre Matagne |
  | Author | Joseph A. Martial |
  | Author | Jens Meiler |
  | Author | Cecile Van de Weerdt |
  | Volume | 8 |
  | Issue | 8 |
  | Publication | PLoS one |
  | ISSN | 1932-6203 |
  | Date | AUG 19 2013 |
  | DOI | 10.1371/journal.pone.0071858 |
  | Language | English |
  | Abstract | The computational protein design protocol Rosetta has been applied successfully to a wide variety of protein engineering problems. Here the aim was to test its ability to design de novo a protein adopting the TIM-barrel fold, whose formation requires about twice as many residues as in the largest proteins successfully designed de novo to date. The designed protein, Octarellin VI, contains 216 residues. Its amino acid composition is similar to that of natural TIM-barrel proteins. When produced and purified, it showed a far-UV circular dichroism spectrum characteristic of folded proteins, with alpha-helical and beta-sheet secondary structure. Its stable tertiary structure was confirmed by both tryptophan fluorescence and circular dichroism in the near UV. It proved heat stable up to 70 degrees C. Dynamic light scattering experiments revealed a unique population of particles averaging 4 nm in diameter, in good agreement with our model. Although these data suggest the successful creation of an artificial alpha/beta protein of more than 200 amino acids, Octarellin VI shows an apparent noncooperative chemical unfolding and low solubility. |
  | Date Added | 10/11/2013, 10:29:15 AM |
  | Modified | 12/2/2013, 4:16:09 PM |

  ### Notes:

  - Test Rosetta's ability to design *de novo* a protein adopting the TIM-barrel fold, whose formation requires about twice as many residues as in the largest proteins successfully designed *de novo* to date.

    Paper application: protein design

    How SCOP is used:

    Mention that TIM-barrel fold has at least 23 superfamilies in SCOP.

    SCOP reference:

    The (β/α)_8_ fold, also known as the TIM-barrel fold, is a very widespread protein topology. It is shared by at least 23 superfamilies in the Structural Classification Of Proteins (SCOP) database [9] and is the most common enzyme fold in the Protein Data Bank (PDB) [10].

  ### Attachments

  - journal.pone.0071858.pdf
- ## Oligomerization Interface of RAGE Receptor Revealed by MS-Monitored Hydrogen Deuterium Exchange

  |  |  |
  | --- | --- |
  | Type | Journal Article |
  | Author | Ewa Sitkiewicz |
  | Author | Krzysztof Tarnowski |
  | Author | Jaroslaw Poznanski |
  | Author | Magdalena Kulma |
  | Author | Michal Dadlez |
  | Volume | 8 |
  | Issue | 10 |
  | Pages | e76353 |
  | Publication | Plos One |
  | ISSN | 1932-6203 |
  | Date | OCT 1 2013 |
  | Extra | WOS:000325427100061 |
  | DOI | 10.1371/journal.pone.0076353 |
  | Abstract | Activation of the receptor for advanced glycation end products ( RAGE) leads to a chronic proinflammatory signal, affecting patients with a variety of diseases. Potentially beneficial modification of RAGE activity requires understanding the signal transduction mechanism at the molecular level. The ligand binding domain is structurally uncoupled from the cytoplasmic domain, suggesting receptor oligomerization is a requirement for receptor activation. In this study, we used hydrogen-deuterium exchange and mass spectrometry to map structural differences between the monomeric and oligomeric forms of RAGE. Our results indicated the presence of a region shielded from exchange in the oligomeric form of RAGE and led to the identification of a new oligomerization interface localized at the linker region between domains |
  | Date Added | 2/12/2014, 1:36:22 PM |
  | Modified | 2/12/2014, 1:36:22 PM |

  ### Notes:

  - HD-exchange study of RAGE receptor.

    How SCOP is used:

    Search for heterogeneous parallel-antiparallel beta-sheet topologies in SCOP and find that they are rare in the PDB.

    SCOP reference:

    These residues are located in a two-stranded parallel β-sheet 4 (3CJJ), and propagation of the β-sheet in this region is assumed to accompany protein oligomerization. Heterogeneous parallel- antiparallel β-sheet topologies are rarely reported in PDB records (as identified in the SCOP database, http:// scop.berkeley.edu [60], Accessed 2013 Sep 4), and it was assumed to be unlikely.

  ### Attachments

  - journal.pone.0076353.pdf
- ## Oligomerization of the reversibly glycosylated polypeptide: its role during rice plant development and in the regulation of self-glycosylation

  |  |  |
  | --- | --- |
  | Type | Journal Article |
  | Author | Veronica De Pino |
  | Author | Cristina Marino Busjle |
  | Author | Silvia Moreno |
  | Volume | 250 |
  | Issue | 1 |
  | Pages | 111-119 |
  | Publication | Protoplasma |
  | ISSN | 0033-183X |
  | Date | FEB 2013 |
  | Extra | WOS:000314186400011 |
  | DOI | 10.1007/s00709-012-0382-x |
  | Abstract | A multigenic family of self-glycosylating proteins named reversibly glycosylated polypeptides, designated as RGPs, have been usually associated with carbohydrate metabolism, although they are an enigma both at the functional, as well as at the structural level. In this work, we used biochemical approaches to demonstrate that complex formation is linked to rice plant development, in which class 1 Oryza sativa RGP (OsRGP) would be involved in an early stage of growing plants, while class 2 OsRGP would be associated with a late stage linked to an active polysaccharide synthesis that occurs during the elongation of plant. Here, a further investigation of the complex formation of the Solanum tuberosum RGP (StRGP) was performed. Results showed that disulfide bonds are at least partially responsible for maintaining the oligomeric protein structure, so that the nonreduced StRGP protein showed an apparent higher molecular weight and a lower radioglycosylation of the monomer with respect to its reduced form. Hydrophobic cluster analysis and secondary structure prediction revealed that class 2 RGPs no longer maintained the Rossman fold described for class 1 RGP. A 3D structure of the StRGP protein resolved by homology modeling supports the possibility of intercatenary disulfide bridges formed by exposed cysteines residues C79, C303 and C251 and they are most probably involved in complex formation occurring into the cell cytoplasm. |
  | Date Added | 2/20/2014, 12:24:01 PM |
  | Modified | 2/20/2014, 12:24:01 PM |

  ### Notes:

  - Experimental study of reversibly glycosylated polypeptides (RGPs).

    How SCOP is used:

    Look up superfamily and family of template structure for homology modeling.

    SCOP reference:

    The template structure (1XHB) belongs to the nucleotide-diphospho-sugar transferases superfamily, polypeptide N-acetylgalactosaminyltransferase 1, N- terminal domain family, according to Scop classification (Andreeva et al. 2008).

  ### Attachments

  - art%3A10.1007%2Fs00709-012-0382-x.pdf
- ## On the Difference in Quality between Current Heuristic and Optimal Solutions to the Protein Structure Alignment Problem

  |  |  |
  | --- | --- |
  | Type | Journal Article |
  | Author | Mauricio Arriagada |
  | Author | Aleksandar Poleksic |
  | Pages | 459248 |
  | Publication | Biomed Research International |
  | Date | 2013 |
  | DOI | 10.1155/2013/459248 |
  | Abstract | The importance of pairwise protein structural comparison in biomedical research is fueling the search for algorithms capable of finding more accurate structural match of two input proteins in a timely manner. In recent years, we have witnessed rapid advances in the development of methods for approximate and optimal solutions to the protein structure matching problem. Albeit slow, these methods can be extremely useful in assessing the accuracy of more efficient, heuristic algorithms. We utilize a recently developed approximation algorithm for protein structure matching to demonstrate that a deep search of the protein superposition space leads to increased alignment accuracy with respect to many well-established measures of alignment quality. The results of our study suggest that a large and important part of the protein superposition space remains unexplored by current techniques for protein structure alignment. |
  | Date Added | 3/7/2014, 1:06:24 PM |
  | Modified | 3/7/2014, 1:06:24 PM |
- ## On the origin and evolution of thermophily: reconstruction of functional precambrian enzymes from ancestors of Bacillus

  |  |  |
  | --- | --- |
  | Type | Journal Article |
  | Author | Joanne K. Hobbs |
  | Author | Charis Shepherd |
  | Author | David J. Saul |
  | Author | Nicholas J. Demetras |
  | Author | Svend Haaning |
  | Author | Colin R. Monk |
  | Author | Roy M. Daniel |
  | Author | Vickery L. Arcus |
  | URL | http://mbe.oxfordjournals.org/content/29/2/825.short |
  | Volume | 29 |
  | Issue | 2 |
  | Pages | 825–835 |
  | Publication | Molecular Biology and Evolution |
  | Date | 2012 |
  | Accessed | 9/20/2013, 1:16:44 PM |
  | Library Catalog | Google Scholar |
  | Short Title | On the origin and evolution of thermophily |
  | Date Added | 10/11/2013, 10:29:15 AM |
  | Modified | 12/2/2013, 4:23:52 PM |

  ### Tags:

  - Interesting

  ### Notes:

  - Reconstruct ancestral sequences of increasing age of four enzymes using Bayesian methods.  Investigate the evolution of thermophily by studying proteins with kinetic methods and also crystallize to study structure.

    How SCOP is used:

    use type: study

    Description: Used SCOP to find structurally similar neigbors to protein of interest (ANC4).  Collect all structures from the same fold (dehyodrogenase-like) and perform structural alignment to compare the structures.  Found that "close structural homologs span the prokaryotic tree" and list two "noteworthy" structural homologs.

    SCOP reference:

    Three-Dimensional Structure of ML LeuB from the Last Common Ancestor of Bacillus  
     To investigate the structural evolution of LeuB and com- pare the structure of an ancestral LeuB with its contempo- rary homologs, we used X-ray crystallography to determine the 3D structure of ML ANC4 (fig. 5). The data collection and refinement statistics for this structure can be found in supplementary table S3 (Supplementary Material online).

    The ANC4 structure shows that, like contemporary LeuB enzymes, the ancestral enzyme is dimeric and has a similar topology and fold to the LeuB structure from B. coagulans (PDB no. 1V53). A sequence-independent structural com- parison (using root mean square deviation; RMSD) between ANC4 and other structures from the isocitrate/isopropylma- late dehydrogenase-like fold (Murzin et al. 1995) showed that close structural homologs span the prokaryotic tree. Two close structural homologs are noteworthy: LeuB from the deeply branching organisms (Battistuzzi et al. 2004) Acidithiobacillus ferrooxidans (RMSD 5 1.22 A ̊ , 339 aligned residues) and Thermotoga maritima (RMSD 5 1.35 A ̊ , 341 aligned residues). Interestingly, ANC4 is more closely aligned with these two structures than the B. coagulans structure (RMSD 5 1.44 A ̊ , 336 aligned residues). These structural com- parisons are reinforced by sequence comparisons for the Bacillus LeuB ancestors; the ANC1–ANC4 sequences move progressively closer to the T. maritima enzyme (59.8–62.5% sequence identity, respectively) despite the fact that the T. maritima sequence was not used in the ASR process. This sequence trend is also seen for ANC1–ANC4 and LeuB from the ancient bacterium Aquifex aeolicus (Battistuzzi et al. 2004).

  ### Attachments

  - [HTML] from oxfordjournals.org
  - Mol Biol Evol-2012-Hobbs-825-35.pdf
- ## On the role of thermal backbone fluctuations in myoglobin ligand gate dynamics

  |  |  |
  | --- | --- |
  | Type | Journal Article |
  | Author | Andrey Krokhotin |
  | Author | Antti J. Niemi |
  | Author | Xubiao Peng |
  | URL | http://link.aip.org/link/?JCPSA6/138/175101/1 |
  | Volume | 138 |
  | Pages | 175101 |
  | Publication | JOURNAL OF CHEMICAL PHYSICS |
  | Date | 2013 |
  | Accessed | 9/23/2013, 10:16:36 AM |
  | Library Catalog | Google Scholar |
  | Date Added | 10/11/2013, 10:29:15 AM |
  | Modified | 3/7/2014, 1:06:42 PM |

  ### Notes:

  - Present an energy function to characterize protein folding and unfolding dynamics.  Apply function to study thermodynamics of the myoglobin backbone, in particular to model the response of the structure to heating and cooling cycles.

    How SCOP/CATH is used:

    SCOP nor CATH data are being used. The databases are just mentioned as classification systems based on protein structure.

    SCOP Reference:

    The interpretation of the protein backbone in terms of solitons can be used as a basis  
    for a quantitative, purely geometric secondary structure classication31. This classication  
    scheme can be developed as a complement to existing schemes such as CATH50 and SCOP51.

  ### Attachments

  - [PDF] from arxiv.org
- ## On the Universe of Protein Folds

  |  |  |
  | --- | --- |
  | Type | Journal Article |
  | Author | Rachel Kolodny |
  | Author | Leonid Pereyaslavets |
  | Author | Abraham O. Samson |
  | Author | Michael Levitt |
  | URL | http://www.annualreviews.org/doi/abs/10.1146/annurev-biophys-083012-130432 |
  | Volume | 42 |
  | Pages | 559–582 |
  | Publication | Annual review of biophysics |
  | Date | 2013 |
  | Accessed | 9/20/2013, 1:12:22 PM |
  | Library Catalog | Google Scholar |
  | Date Added | 2/13/2014, 4:13:17 PM |
  | Modified | 10/8/2014, 12:50:56 PM |

  ### Notes:

  - Review of uses and differences of structural classification databases. Very relevant to the SCOP literature review.

    How SCOP is used:

    Provide in-depth assessment of differences between SCOP and CATH.

    SCOP reference:

    In Abstract:

    Classification of proteins structures, which started in the 1970s with about a dozen structures, has continued with increasing en- thusiasm, leading to two main fold classifications, SCOP and CATH, as well as many additional databases.

  ### Attachments

  - annurev-biophys-083012-130432.pdf
- ## OPM database and PPM web server: resources for positioning of proteins in membranes

  |  |  |
  | --- | --- |
  | Type | Journal Article |
  | Author | Mikhail A. Lomize |
  | Author | Irina D. Pogozheva |
  | Author | Hyeon Joo |
  | Author | Henry I. Mosberg |
  | Author | Andrei L. Lomize |
  | Volume | 40 |
  | Issue | D1 |
  | Pages | D370-D376 |
  | Publication | Nucleic Acids Research |
  | ISSN | 0305-1048 |
  | Date | JAN 2012 |
  | Extra | WOS:000298601300054 |
  | DOI | 10.1093/nar/gkr703 |
  | Abstract | The Orientations of Proteins in Membranes (OPM) database is a curated web resource that provides spatial positions of membrane-bound peptides and proteins of known three-dimensional structure in the lipid bilayer, together with their structural classification, topology and intracellular localization. OPM currently contains more than 1200 transmembrane and peripheral proteins and peptides from approximately 350 organisms that represent approximately 3800 Protein Data Bank entries. Proteins are classified into classes, superfamilies and families and assigned to 21 distinct membrane types. Spatial positions of proteins with respect to the lipid bilayer are optimized by the PPM 2.0 method that accounts for the hydrophobic, hydrogen bonding and electrostatic interactions of the proteins with the anisotropic water-lipid environment described by the dielectric constant and hydrogen-bonding profiles. The OPM database is freely accessible at http://opm.phar.umich.edu. Data can be sorted, searched or retrieved using the hierarchical classification, source organism, localization in different types of membranes. The database offers downloadable coordinates of proteins and peptides with membrane boundaries. A gallery of protein images and several visualization tools are provided. The database is supplemented by the PPM server (http://opm.phar.umich.edu/server.php) which can be used for calculating spatial positions in membranes of newly determined proteins structures or theoretical models. |
  | Date Added | 2/20/2014, 12:24:01 PM |
  | Modified | 2/20/2014, 12:24:01 PM |

  ### Notes:

  - Present OPM - Orientation of Proteins in Membranes - database.

    How SCOP is used:

    Annotate non-SCOP data set with SCOP classification.

    Use SCOP and Pfam to curate superfamilies and families.  Also provide links to SCOP.

    SCOP reference:

    PROTEIN CLASSIFICATION

    The classification has four-level hierarchy: type (TM, per- ipheral/monotopic protein and peptides), class (a-helical polytopic, a-helical bitopic, b-barrel TM proteins; and all-a, all-b, a+b, a/b peripheral/monotopic proteins), superfamily (evolutionarily related proteins) and family (proteins with clear sequence homology). Multi-domain proteins and their complexes are classified based on Pfam (32), SCOP (33) and TCDB (6) classification of their largest membrane-associated domain. OPM super- families usually correspond to Pfam clans and SCOP superfamilies, whereas OPM families correspond to Pfam, SCOP and TCDB families.

    ...

    The database provides links to TCDB (6), Pfam (32) from family and superfamily pages and to SCOP (33), PDB (3), PDBsum (39), PDBe (29), OCA (40), MMDB (41) from protein pages.

    ...

  ### Attachments

  - Nucl. Acids Res.-2012-Lomize-D370-6.pdf
- ## Optimization of Model Parameters for Describing the Amide I Spectrum of a Large Set of Proteins

  |  |  |
  | --- | --- |
  | Type | Journal Article |
  | Author | Eeva-Liisa Karjalainen |
  | Author | Tore Ersmark |
  | Author | Andreas Barth |
  | URL | http://pubs.acs.org/doi/abs/10.1021/jp301095v |
  | Volume | 116 |
  | Issue | 16 |
  | Pages | 4831–4842 |
  | Publication | The Journal of Physical Chemistry B |
  | Date | 2012 |
  | Accessed | 9/20/2013, 1:11:53 PM |
  | Library Catalog | Google Scholar |
  | Date Added | 10/11/2013, 10:29:15 AM |
  | Modified | 10/11/2013, 10:29:15 AM |

  ### Notes:

  - Present a biophysical simulation method for prediction of infrared absorption of the amide I vibration of proteins.

    How SCOP is used:

    Used the "rationally selected proteins (RaSP)" basis set, a data set of 44 proteins that has been curated in a previous study to have diverse folds as defined by CATH and SCOP.

    SCOP reference:

    Protein Set. The rationally selected proteins (RaSP) set developed by Goormaghtigh and co-workers40 was used for the simulations described in this manuscript. The RaSP set was constructed to represent the maximum range of structural variation exhibited in proteins by including as many different protein folds as defined in CATH75 and SCOP76,77 as possible, as well as structures representing different α-helix and β-sheet contents. Also, only proteins for which high quality crystal structures were available and that also were generally commercially available qualified for the set. The set is described in detail in the original publication, and the included proteins are listed in Table 1. More detailed information on the main characteristics of these proteins can be found in the Supporting Information.

  ### Attachments

  - jp301095v.pdf
  - Snapshot
- ## Optimization of profile-to-profile alignment parameters for one-dimensional threading

  |  |  |
  | --- | --- |
  | Type | Journal Article |
  | Author | Pawel Gniewek |
  | Author | Andrzej Kolinski |
  | Author | Dominik Gront |
  | URL | http://online.liebertpub.com/doi/abs/10.1089/cmb.2011.0307 |
  | Volume | 19 |
  | Issue | 7 |
  | Pages | 879–886 |
  | Publication | Journal of Computational Biology |
  | Date | 2012 |
  | Accessed | 9/23/2013, 10:18:21 AM |
  | Library Catalog | Google Scholar |
  | Date Added | 10/11/2013, 10:29:15 AM |
  | Modified | 3/7/2014, 12:14:50 PM |

  ### Tags:

  - algorithms

  ### Notes:

  - Present threading-based approach to sequence alignment and evaluate on SCOP family prediction.

    How SCOP is used:

    Use type: training and benchmarking  
       
     Application: sequence alignment and family classification  
       
     Filtered on: family  (families with 4 or more domains)  
     Filtering type:  b (representative set.  chose top 4 domains in families with 4 or more domains.  placed 2 in training set and 2 in benchmarking set)  
       
     Benchmarking type: a (validation)  
     Levels used in benchmarking: family   
     Representative set: Used ASTRAL 40% subsets

    Description:

    Trained their method using training set then benchmarked for family classification using the other data set.

    SCOP references:

    2.1. Benchmark set

    The optimization process described in this contribution has been based on the most recent Astral database (Chandonia et al., 2004). The database consists of protein domain structures extracted from the PDB content according to the SCOP classification (Murzin et al., 1995). Redundancy had already been removed from the set in such a way that amino acid sequences of any two domains from the database are identical in at most 40%. In order to transform the Astral database into a well-balanced benchmark set, we performed the following steps:

    (i) We considered only these SCOP families that are represented in Astral by at least 4 domains. Any domain that belongs to a Family which does not satisfy this condition was excluded from the benchmark. In this way, we could easily divide the dataset into a train set and a test set.

    (ii) Then, the best four Family representing structures (according AreoSpaci score) were divided into training and testing sets by putting two randomly selected domains into the first set and the remaining two into the other.

    The procedure resulted in a benchmark set comprising two subsets: train and test (1082 domains each). The former set was used to determine the optimal values for the necessary parameters and the latter one to assess the quality of our method. Any of these 1082 domains may be used as a query in a search for homologues domains. The way that the benchmark set was constructed ensured that there was exactly one correct answer that shared the same SCOP Family with the query. Moreover, the training and testing sets were of the same size, which helped to avoid any bias during the optimization procedure.

    In the course of this study, to provide homology sequence redundancy on the 30% level, several additional domains were excluded from the benchmark. In order to keep the benchmark consistent, we also removed all other domains that belong to the same SCOP Family as the problematic ones. The reduced benchmark set therefore comprised two subsets of 935 domains.

  ### Attachments

  - [PDF] from uw.edu.pl
  - Snapshot
- ## Origin and Evolution of Protein Fold Designs Inferred from Phylogenomic Analysis of CATH Domain Structures in Proteomes

  |  |  |
  | --- | --- |
  | Type | Journal Article |
  | Author | Syed Abbas Bukhari |
  | Author | Gustavo Caetano-Anollés |
  | URL | http://dx.plos.org/10.1371/journal.pcbi.1003009 |
  | Volume | 9 |
  | Issue | 3 |
  | Pages | e1003009 |
  | Publication | PLoS computational biology |
  | Date | 2013 |
  | Accessed | 9/20/2013, 1:16:59 PM |
  | Library Catalog | Google Scholar |
  | Date Added | 10/11/2013, 10:29:15 AM |
  | Modified | 3/7/2014, 12:10:55 PM |

  ### Notes:

  - Studies the evolutionary emergence of different CATH topologies such as sandwiches, bundles, barrels, prisms, solenoids, and propellors.

    Both CATH and SCOP are hierarchical classifications of protein domains by structural and evolutionary relationships, but CATH focuses more on structural similarities.  In particular, SCOP doesn't have an equivalent level to the CATH topology level.  In previous studies, phylogenies of SCOP fold, sf, and family levels were studied.  Now their focus is on CATH.

    Additionally, the presented study "benchmarks the phylogenomic analysis of CATH domains with SCOP domains."

    How SCOP/CATH is used:

    Present a qualitative comparison of CATH and SCOP.

    Count the distribution of CATH and SCOP domains with representatives from different taxonomic groups.  For example, over 50% of SCOP folds have reps from arhaea, bacteria, and eukarya.  Around 8% of folds only have bacteria reps.

    SCOP/CATH reference:

    SCOP [5] is a largely manual collection of protein structural domains that aims to provide a detailed and compre- hensive description of the structural and evolutionary relationships of proteins with known structures. In contrast, CATH [6] uses a combination of automated and manual techniques, which include computational algorithms, empirical and statistical evidence, literature review and expert analysis. Both classifications are hierarchical but dissect 3D structure differently, focusing more on either evolutionary or structural considerations [4]. SCOP unifies domain structures that are evolutionarily related at sequence level (.30% pairwise residue identities) and are unambiguously linked to specific molecular functions into fold families (FFs), FFs with common structures and functions with a common evolutionary...

     ..

    The study benchmarks previous phylogenetic analysis of SCOP-defined domains and again reveals the early origin of the archaeal superkingdom.

  ### Attachments

  - journal.pcbi.1003009.pdf
- ## Overcoming sequence misalignments with weighted structural superposition

  |  |  |
  | --- | --- |
  | Type | Journal Article |
  | Author | Nickolay A. Khazanov |
  | Author | Kelly L. Damm-Ganamet |
  | Author | Daniel X. Quang |
  | Author | Heather A. Carlson |
  | Volume | 80 |
  | Issue | 11 |
  | Pages | 2523-2535 |
  | Publication | Proteins: Structure, Function, and Bioinformatics |
  | ISSN | 0887-3585 |
  | Date | November 2012 |
  | DOI | 10.1002/prot.24134 |
  | Language | English |
  | Abstract | An appropriate structural superposition identifies similarities and differences between homologous proteins that are not evident from sequence alignments alone. We have coupled our Gaussian-weighted RMSD (wRMSD) tool with a sequence aligner and seed extension (SE) algorithm to create a robust technique for overlaying structures and aligning sequences of homologous proteins (HwRMSD). HwRMSD overcomes errors in the initial sequence alignment that would normally propagate into a standard RMSD overlay. SE can generate a corrected sequence alignment from the improved structural superposition obtained by wRMSD. HwRMSD's robust performance and its superiority over standard RMSD are demonstrated over a range of homologous proteins. Its better overlay results in corrected sequence alignments with good agreement to HOMSTRAD. Finally, HwRMSD is compared to established structural alignment methods: FATCAT, secondary-structure matching, combinatorial extension, and Dalilite. Most methods are comparable at placing residue pairs within 2 angstrom, but HwRMSD places many more residue pairs within 1 angstrom, providing a clear advantage. Such high accuracy is essential in drug design, where small distances can have a large impact on computational predictions. This level of accuracy is also needed to correct sequence alignments in an automated fashion, especially for omics-scale analysis. HwRMSD can align homologs with low-sequence identity and large conformational differences, cases where both sequence-based and structural-based methods may fail. The HwRMSD pipeline overcomes the dependency of structural overlays on initial sequence pairing and removes the need to determine the best sequence-alignment method, substitution matrix, and gap parameters for each unique pair of homologs. Proteins 2012. (c) 2012 Wiley Periodicals, Inc. |
  | Date Added | 10/25/2013, 4:17:08 PM |
  | Modified | 3/7/2014, 1:07:06 PM |

  ### Tags:

  - Cite ASTRAL

  ### Notes:

  - HwRMSD: structure-based sequence alignment tool

    How SCOP/CATH is used:

    Do not use SCOP or CATH data.

    SCOP reference:

    Many databases exist that classify proteins into fam- ilies by their structures, including but not limited to SCOP,4 CATH,5 DaliDB,6 PASS2,7 MMDB,8 ASTRAL,9

  ### Attachments

  - 24134\_ftp.pdf
- ## Overlapping correlation clustering

  |  |  |
  | --- | --- |
  | Type | Journal Article |
  | Author | Francesco Bonchi |
  | Author | Aristides Gionis |
  | Author | Antti Ukkonen |
  | URL | http://link.springer.com/article/10.1007/s10115-012-0522-9 |
  | Pages | 1–32 |
  | Publication | Knowledge and information systems |
  | Date | 2013 |
  | Accessed | 9/23/2013, 10:14:36 AM |
  | Library Catalog | Google Scholar |
  | Date Added | 10/11/2013, 10:29:15 AM |
  | Modified | 10/11/2013, 10:29:15 AM |

  ### Tags:

  - algorithms
  - clustering
  - Correlation clustering
  - Overlapping clustering
  - Pregel

  ### Notes:

  - The paper proposes an algorithm to address overlapping in clustering data sets. This is applied to animal trajectories from zoological data, as well as to and protein sequence clustering by from sequence similarity.

    **SCOP Use:**

    Evaluate method first on SCOP superfamily classification, to compare with another algorithm (SCPS).   Then also evaluated on the full classification, using a similarity function based on the depth of the lowest common ancestory (lca) in the SCOP tree.

    **SCOP Reference**

    An important problem in genomics is the study of evolution- ary relatedness of proteins. We use our algorithms to cluster proteins to homologous groups given pairwise similarities of their amino-acid sequences. Such similarities are computed by the sequence alignment tool BLAST [12]. We follow the approach of Paccanaro et al. [13] and Nepusz et al. [14], and compare the computed clustering against a ground truth given by SCOP, a manually crafted taxonomy of proteins [15]. The SCOP taxonomy is a tree with proteins at the leaf nodes. The ground truth clusters used in the experiments are subsets of the leafs, that is, proteins, rooted at different SCOP superfamilies. These are nodes on the 3rd level below the root.

    We also conduct a more fine-grained analysis of the results using the SCOP taxonomy. Intuitively the cost of a clustering errors should take distances induced by the taxonomy into account. If two proteins are placed in the same cluster, they should contribute more (less) to the clustering cost if their distance in the taxonomy is higher (lower). Consequently, we define the SCOP similarity between two proteins as follows:

    sim(u, v) = d(lca(u, v)) , (8) max(d(u), d(v)) − 1

    where d(u) is the depth of a node in the tree (the root is at depth 0), and lca(u, v) denotes the lowest common ancestor of the nodes u and v. We then define the cost of a clustering to be 1 − sim(u, v) for two proteins that are assigned to the same cluster, and sim(u,v) for two proteins assigned to different clusters.

  ### Attachments

  - OCC.pdf
  - Snapshot
- ## p42.3 gene expression in gastric cancer cell and its protein regulatory network analysis

  |  |  |
  | --- | --- |
  | Type | Journal Article |
  | Author | Jianhua Zhang |
  | Author | Chunlei Lu |
  | Author | Zhigang Shang |
  | Author | Rui Xing |
  | Author | Li Shi |
  | Author | Youyong Lv |
  | Volume | 9 |
  | Pages | 53 |
  | Publication | Theoretical Biology and Medical Modelling |
  | Date | December 2012 |
  | DOI | 10.1186/1742-4682-9-53 |
  | Abstract | Background: To analyze the p42.3 gene expression in gastric cancer (GC) cell, find the relationship between protein structure and function, establish the regulatory network of p42.3 protein molecule and then to obtain the optimal regulatory pathway. Methods: The expression of p42.3 gene was analyzed by RT-PCR, Western Blot and other biotechnologies. The relationship between the spatial conformation of p42.3 protein molecule and its function was analyzed using bioinformatics, MATLAB and related knowledge about protein structure and function. Furthermore, based on similarity algorithm of spatial layered spherical coordinate, we compared p42.3 molecule with several similar structured proteins which are known for the function, screened the characteristic nodes related to tumorigenesis and development, and established the multi variable relational model between p42.3 protein expression, cell cycle regulation and biological characteristics in the level of molecular regulatory networks. Finally, the optimal regulatory network was found by using Bayesian network. Results: (1) The expression amount of p42.3 in G1 and M phase was higher than that in S and G2 phase; (2) The space coordinate systems of different structural domains of p42.3 protein were established in Matlab7.0 software; (3) The optimal pathway of p42.3 gene in protein regulatory network in gastric cancer is Ras protein, Raf 1 protein, MEK, MAPK kinase, MAPK, tubulin, spindle protein, centromere protein and tumor. Conclusion: It is of vital significance for mechanism research to find out the action pathway of p42.3 in protein regulatory network, since p42.3 protein plays an important role in the generation and development of GC. |
  | Date Added | 3/7/2014, 1:06:24 PM |
  | Modified | 3/7/2014, 1:06:24 PM |
- ## PACSY, a relational database management system for protein structure and chemical shift analysis

  |  |  |
  | --- | --- |
  | Type | Journal Article |
  | Author | Woonghee Lee |
  | Author | Wookyung Yu |
  | Author | Suhkmann Kim |
  | Author | Iksoo Chang |
  | Author | Weontae Lee |
  | Author | John L. Markley |
  | URL | http://link.springer.com/article/10.1007/s10858-012-9660-3 |
  | Volume | 54 |
  | Issue | 2 |
  | Pages | 169–179 |
  | Publication | Journal of biomolecular NMR |
  | Date | 2012 |
  | Accessed | 9/23/2013, 10:19:41 AM |
  | Library Catalog | Google Scholar |
  | Date Added | 10/11/2013, 10:29:15 AM |
  | Modified | 3/7/2014, 12:14:26 PM |

  ### Tags:

  - bioinformatics
  - BMRB
  - database
  - NMR
  - PACSY
  - PDB
  - SCOP
  - Structural biology

  ### Notes:

  - It details a database management system called PACSY (Protein structure And Chemical Shift NMR spectroscopY), which integrates data from PDB and other protein databases (including SCOP). The information taken from these databases are used to "provide three-dimensional coordinates and chemical shifts of atoms along with derived information such as torsion angles, solvent accessible surface areas, and hydrophobicity scales."

    How SCOP is used:

    The entire SCOP database was downloaded (1.75) and used. The information used is the class level only, which was used to annotate the PDB entries that were downloaded.

    SCOP reference:

    The data format of the PDB has been extended, and the current Worldwide Protein  
    Data Bank (wwPDB) now encompasses structural data from NMR spectroscopy as well as X-ray crystallography  
    (Berman et al. 2007). Comparisons of three-dimensional structures provide information on evolutionary relationships,  
    and analyses of this kind are available from the SCOP database (Murzin et al. 1995) and the CATH database (a  
    hierarchic classification of protein domain structures, Orengo et al. 1997).

    Although clear relationships have been found between 3D structure and NMR parameters (e.g., chemical shifts,  
    J-coupling constants, RDC values), tools are lacking that enable the combined analysis of data from the PDB, BMRB,  
    and SCOP databases. One of the reasons for this is that PDB and BMRB data are stored in flat-file formats, versions of  
    the Self-defining Text Archive and Retrieval (STAR) file format (Hall and Spadaccini 1994). As an aid to easier and  
    faster handling of the huge information content of these databases, we have developed the PACSY (Protein structure  
    And Chemical Shift spectroscopY) database, which utilizes a relational database management system (RDBMS), to  
    manage information derived from the PDB, BMRB, and SCOP databases. We describe how information from each  
    database is extracted and processed to make them cross-related one another to enable queries.

    The PACSY Maker  
    software then processes these data with STRIDE (Frishman and Argos 1995), combines them with SCOP data, and  
    parses the resulting data into a set of tables and fields in the prepared RDBMS server.

    It has the simple  
    graphical user interface (GUI) shown in Fig. 3a, which is used to set up a working directory to store downloaded files  
    from the PDB, BMRB, and SCOP databases along with processed files, such as SQL dump files and an insertion script  
    file. Once a root of the working directory is set up, other directories for storage and processes are created automatically  
    as relative directories. The user can modify those directories for more detailed setup. PACSY Maker downloads  
    dbmatch.csv from the BMRB ftp archive when it is executed (Fig. 2). The file, dbmatch.csv, contains information on  
    how BMRB entries are related to entries in other databases such as PDB, Swiss-Prot, and EMBL. PACSY Maker  
    processes the file to contain only information from PDB and BMRB submitted by a common author, and checks for  
    needed updates by comparing the results to a recently processed dbmatch.csv file. Next, PACSY Maker downloads the  
    SCOP database, and parses it to add structural classification information to each PDB entry. Finally, PACSY Maker  
    downloads PDB and BMRB files from the respective web archive that match the update list made by comparing the  
    new and old processed dbmatch.csv files.

    Under Results

    The PACSY database was built and installed for testing at the National Magnetic Resonance Facility at Madison  
    (NMRFAM). PACSY Maker ran on a 64-bit CentOS 5.5 developmental server for an entire day to build and upload  
    SQL dump script files for the initial database. The number of downloaded PDB and BMRB files were both 3745, and a  
    data file was downloaded for SCOP. 473 Mb were consumed by BMRB files, whereas 18 Gb were consumed by PDB  
    files. The size of SCOP database was only 5.8 Mb.

    Statistics were collected from PACSY to confirm both the availability and feasibility of database queries. Because the  
    PACSY database employs a client–server concept, it supports many different options, including remote operation  
    (Fig. 1). Because PACSY Analyzer utilizes an ODBC connection to the database server, in our case MySQL 5.0, we  
    first installed and set up ODBC Connector. Next, we used PACSY Analyzer to determine the structural classification of  
    PACSY entries as defined by the SCOP database (Table 2). SCOP does not cover all PDB entries, because full  
    classification is not automated. Csaba’s study in 2009 revealed that the SCOP database version 1.73 covered 35.5 % of  
    all PDB entries whereas CATH database version 3.1.0 covered 32.0 % (Csaba et al. 2009). Furthermore, Jefferson and  
    co-workers found that for single domain classifications of the type commonly found in NMR structures, coverage of  
    CATH by SCOP was greater than that of SCOP by CATH (Jefferson et al. 2008). We found that the SCOP 1.73  
    database provided 43 % coverage. Because PACSY contains structural classification information, it is possible to  
    investigate proteins by fold class. Apart from unclassified entries, the largest class of PDB and BMRB entries were for  
    all-alpha proteins (745 entries, Table 2). Other major classes are well represented, except for multi-domain proteins (no  
    entries, Table 2).

    All the work performed in this paper is based on the  
    versions of PDB and BMRB available on February 7, 2012 and on the 1.75 version of the SCOP database.

  ### Attachments

  - PACSY, a relational database management system for protein structure and chemical shift analysis - Springer.pdf
- ## PainNetworks: A web-based resource for the visualisation of pain-related genes in the context of their network associations

  |  |  |
  | --- | --- |
  | Type | Journal Article |
  | Author | James R. Perkins |
  | Author | Jonathan Lees |
  | Author | Ana Antunes-Martins |
  | Author | Ilhem Diboun |
  | Author | Stephen B. McMahon |
  | Author | David L. H. Bennett |
  | Author | Christine Orengo |
  | Volume | 154 |
  | Issue | 12 |
  | Publication | Pain |
  | Date | December 2013 |
  | DOI | 10.1016/j.pain.2013.09.003 |
  | Abstract | Hundreds of genes are proposed to contribute to nociception and pain perception. Historically, most studies of pain-related genes have examined them in isolation or alongside a handful of other genes. More recently the use of systems biology techniques has enabled us to study genes in the context of the biological pathways and networks in which they operate. Here we describe a Web-based resource, available at http://www.PainNetworks.org. It integrates interaction data from various public databases with information on known pain genes taken from several sources (eg, The Pain Genes Database) and allows the user to examine a gene (or set of genes) of interest alongside known interaction partners. This information is displayed by the resource in the form of a network. The user can enrich these networks by using data from pain-focused gene expression studies to highlight genes that change expression in a given experiment or pairs of genes showing correlated expression patterns across different experiments. Genes in the networks are annotated in several ways including biological function and drug binding. The Web site can be used to find out more about a gene of interest by looking at the function of its interaction partners. It can also be used to interpret the results of a functional genomics experiment by revealing putative novel pain-related genes that have similar expression patterns to known pain-related genes and by ranking genes according to their network connections with known pain genes. We expect this resource to grow over time and become a valuable asset to the pain community. (C) 2013 International Association for the Study of Pain. Published by Elsevier B. V. All rights reserved. |
  | Date Added | 3/7/2014, 12:08:00 PM |
  | Modified | 3/7/2014, 12:08:00 PM |
- ## PASS2 version 4: An update to the database of structure-based sequence alignments of structural domain superfamilies

  |  |  |
  | --- | --- |
  | Type | Journal Article |
  | Author | A. Gandhimathi |
  | Author | Anu G. Nair |
  | Author | R. Sowdhamini |
  | Volume | 40 |
  | Issue | D1 |
  | Pages | D531-D534 |
  | Publication | Nucleic Acids Research |
  | ISSN | 0305-1048 |
  | Date | January 2012 |
  | DOI | 10.1093/nar/gkr1096 |
  | Language | English |
  | Abstract | Accurate structure-based sequence alignments of distantly related proteins are crucial in gaining insight about protein domains that belong to a superfamily. The PASS2 database provides alignments of proteins related at the superfamily level and are characterized by low sequence identity. We thus report an automated, updated version of the superfamily alignment database known as PASS2.4, consisting of 1961 superfamilies and 10 569 protein domains, which is in direct correspondence with SCOP (1.75) database. Database organization, improved methods for efficient structure-based sequence alignments and the analysis of extreme distantly related proteins within superfamilies formed the focus of this update. Alignment of family-specific functional residues can be realized using such alignments and is shown using one superfamily as an example. The database of alignments and other related features can be accessed at http://caps.ncbs.res.in/pass2/. |
  | Date Added | 10/25/2013, 4:17:08 PM |
  | Modified | 11/12/2013, 4:28:19 PM |

  ### Tags:

  - Cite ASTRAL

  ### Notes:

  - PASS2 provides structure-based sequence alignments of all SCOP superfamilies.

    This paper reports recent improvements to PASS2, especially the recent upgrade to using SCOP 1.75 data.

    How SCOP is used:

    Provide alignments for each SCOP superfamily.  Don't explicitly say if they are using ASTRAL sequences or domains, but it would make sense if they did.

    SCOP reference:

    SCOP (5) database provides a detailed and comprehensive descrip- tion about protein structures organized at different hierarchies of structural and functional similarities. ASTRAL (6) provides an explicit mapping between the PDB ATOM and SEQRES records within PDB files, which is used to derive databases of sequences corresponding to the SCOP domains.

    We thus report an automated, updated version of the superfamily alignment database known as PASS2.4, consisting of 1961 superfamilies and 10569 protein domains, which is in direct correspondence with SCOP (1.75) database.

    The idea of structure-based sequence alignment and analysis of protein domain superfamilies originally started with CAMPASS (10), The automated version of CAMPASS, called as PASS2 (11), which we now refer to as PASS2.1, contained 613 superfamilies in direct correspondence with SCOP 1.53. The subsequent versions of PASS2 [PASS2.2 and PASS2.3 (12,13)] have been updated in direct correspondence with SCOP1.63 and SCOP 1.73, respectively. In most PASS2 versions, we have classified the superfamilies into single-member (SMS), two-member (TMS) and multi-member (MMS) superfamilies, which directly implies the number of domains with <40% identity with other domains in the superfamily. TMS and MMS are aligned using specific alignment method from PASS2 version 3 onwards. The statistics of all the four versions are reported in Figure 1. The current version of PASS2, PASS2.4, holds 10569 protein domains (at a 40% sequence identity cut-off) belonging to 1961 superfamilies and is in direct correspondence with SCOP 1.75.

  ### Attachments

  - Nucl. Acids Res.-2012-Gandhimathi-D531-4.pdf
- ## PBSword: a web server for searching similar protein-protein binding sites

  |  |  |
  | --- | --- |
  | Type | Journal Article |
  | Author | Bin Pang |
  | Author | Xingyan Kuang |
  | Author | Nan Zhao |
  | Author | Dmitry Korkin |
  | Author | Chi-Ren Shyu |
  | Volume | 40 |
  | Issue | W1 |
  | Pages | W428-W434 |
  | Publication | Nucleic Acids Research |
  | ISSN | 0305-1048 |
  | Date | July 2012 |
  | DOI | 10.1093/nar/gks527 |
  | Language | English |
  | Abstract | PBSword is a web server designed for efficient and accurate comparisons and searches of geometrically similar protein-protein binding sites from a large-scale database. The basic idea of PBSword is that each protein binding site is first represented by a high-dimensional vector of `visual words', which characterizes both the global and local shape features of the binding site. It then uses a scalable indexing technique to search for those binding sites whose visual words representations are similar to that of the query binding site. Our system is able to return ranked results of binding sites in short time from a database of 194 322 domain-domain binding sites. PBSword supports query by protein ID and by new structures uploaded by users. PBSword is a useful tool to investigate functional connections among proteins based on the local structures of binding site and has potential applications to protein-protein docking and drug discovery. The system is hosted at http://pbs.rnet.missouri.edu. |
  | Date Added | 10/11/2013, 10:29:15 AM |
  | Modified | 5/5/2014, 3:12:43 PM |

  ### Notes:

  - Present a web server, PBSword, for indexing and searching for similar protein-protein binding sites.  Use SCOP domain definitions.

    How SCOP is used:

    Index all binding sites by the SCOP domains.  Provide ability to search database by SCOP or PDB  ID.

    How CATH is used:

    Not using CATH data.  Just cited as an example.

    SCOP reference:

    The key features of PBSword server include the follow- ing: (i) The binding site comparison method introduces a novel feature extraction algorithm and online database indexing; (ii) the database of binding site is based on the interactions between domains which are defined using the latest SCOP version (24); (iii) for each retrieved binding site from the database, a 3-dimensional (3D) view of struc- ture and surface, as well as physicochemical properties are presented; (iv) the efficiency has been significantly enhanced to meet the requirements of large-scale protein binding site database searching.

    ...

    Database management and preprocessing

    The database of PBSword contains domain–domain binding sites of known protein structures. The structural data are extracted from Protein Data Bank (PDB) (25). If a PDB entry has more than one structure model, the first model is used in the database’s current implementa- tion. For domain assignment, the most recent release (June 2009) of manually curated SCOP database is used. For each PDB structure, each pair of determined subunits (i.e. domains) is analysed to determine whether they interact with each other using the following definition.

    ...

    Currently, the entire PBSword database contains 194 322 redundant binding sites selected from 3123 SCOP families.

  ### Attachments

  - Nucl. Acids Res.-2012-Pang-W428-34.pdf
- ## PDB-2-PB: a curated online protein block sequence database

  |  |  |
  | --- | --- |
  | Type | Journal Article |
  | Author | V. Suresh |
  | Author | K. Ganesan |
  | Author | S. Parthasarathy |
  | Volume | 45 |
  | Issue | 1 |
  | Pages | 127-129 |
  | Publication | JOURNAL OF APPLIED CRYSTALLOGRAPHY |
  | ISSN | 0021-8898 |
  | Date | February 2012 |
  | DOI | 10.1107/S0021889811052356 |
  | Language | English |
  | Abstract | This article describes the development of a curated online protein block sequence database, PDB-2-PB. The protein block sequences for protein structures with complete backbone coordinates have been encoded using the encoding procedure of de Brevern, Etchebest & Hazout [Proteins (2000), 41, 271287]. In the current release of the PDB-2-PB database (version 1.0), the protein entries from a recent release of the World Wide Protein Data Bank (wwPDB), which has 74 297 solved PDB entries as of 7 July 2011, have been used as a primary source. The PDB-2-PB database stores the protein block sequences for all the chains present in a protein structure. PDB-2-PB version 1.0 has the curated protein block sequences for 103 252 PDB chain entries (93 547 X-ray, 7033 NMR and 2672 other experimental chain entries). From the PDB-2-PB database, users can extract the curated protein block sequence and its corresponding amino acid sequence, which is extracted from the PDB ATOM records. Users can download these sequences either by using the PDB code or by using various parameters listed in the database. The PDB-2-PB database is freely available at . |
  | Date Added | 10/11/2013, 10:29:15 AM |
  | Modified | 10/11/2013, 10:29:15 AM |

  ### Tags:

  - Databases
  - local protein structures
  - protein block sequences
  - Protein Data Bank

  ### Notes:

  - Present a database of protein block sequences: PDB-2-PB.

    How SCOP is used:

    Mentioned in previous work on a database for predicting fold.  The PredictFold-PB  database contains a protein-block sequence for each SCOP fold from 1.75, excluding 242 folds where PDB entries were problematic.

    SCOP reference:

    Our recent work with the PB is a development of the web-based fold recognition server called PredictFold-PB (Suresh et al., 2012). In this method, we align the predicted PB sequence of a query with a library of assigned PB sequences of 953 known folds using a local pairwise alignment program. Our method uses a PB fold library with 953 assigned PB sequences that belong to 953 folds out of the 1195 folds in the SCOP 1.75 release (Murzin et al., 1995). The remaining folds were not considered because their PDB entries are affected by one of the following: (i) missing amino acids in the ATOM records, (ii) ATOM records interrupted by HETATM records, (iii) non- standard amino acids in ATOM records, (iv) PDB entries with only C⬚⬚ coordinate information and (v) PDB entries without any back- bone coordinate information.

  ### Attachments

  - aj5185.pdf
- ## PDBe: Protein Data Bank in Europe

  |  |  |
  | --- | --- |
  | Type | Journal Article |
  | Author | S. Velankar |
  | Author | Y. Alhroub |
  | Author | C. Best |
  | Author | S. Caboche |
  | Author | M. J. Conroy |
  | Author | J. M. Dana |
  | Author | M. A. Fernandez Montecelo |
  | Author | G. van Ginkel |
  | Author | A. Golovin |
  | Author | S. P. Gore |
  | Author | A. Gutmanas |
  | Author | P. Haslam |
  | Author | P. M. S. Hendrickx |
  | Author | E. Heuson |
  | Author | M. Hirshberg |
  | Author | M. John |
  | Author | I. Lagerstedt |
  | Author | S. Mir |
  | Author | L. E. Newman |
  | Author | T. J. Oldfield |
  | Author | A. Patwardhan |
  | Author | L. Rinaldi |
  | Author | G. Sahni |
  | Author | E. Sanz-Garcia |
  | Author | S. Sen |
  | Author | R. Slowley |
  | Author | A. Suarez-Uruena |
  | Author | G. J. Swaminathan |
  | Author | M. F. Symmons |
  | Author | W. F. Vranken |
  | Author | M. Wainwright |
  | Author | G. J. Kleywegt |
  | Volume | 40 |
  | Issue | D1 |
  | Pages | D445-D452 |
  | Publication | Nucleic Acids Research |
  | ISSN | 0305-1048 |
  | Date | JAN 2012 |
  | Extra | WOS:000298601300065 |
  | DOI | 10.1093/nar/gkr998 |
  | Abstract | The Protein Data Bank in Europe (PDBe; pdbe.org) is a partner in the Worldwide PDB organization (wwPDB; www.wwpdb.org) and as such actively involved in managing the single global archive of biomacromolecular structure data, the PDB. In addition, PDBe develops tools, services and resources to make structure-related data more accessible to the biomedical community. Here we describe recently developed, extended or improved services, including an animated structure-presentation widget (PDBportfolio), a widget to graphically display the coverage of any UniProt sequence in the PDB (UniPDB), chemistry- and taxonomy-based PDB-archive browsers (PDBeXplore), and a tool for interactive visualization of NMR structures, corresponding experimental data as well as validation and analysis results (Vivaldi). |
  | Date Added | 2/20/2014, 12:24:01 PM |
  | Modified | 3/7/2014, 12:10:42 PM |

  ### Notes:

  - Present PDBe.

    How SCOP is used:

    Annotate PDBe with SCOP domains using SIFTS.

    SCOP reference:

    Domain structure—separate images show SCOP (13),

    CATH (14) and Pfam (15) domains as annotated by the SIFTS resource (16).

  ### Attachments

  - Nucl. Acids Res.-2012-Velankar-D445-52.pdf
- ## PDB-scale analysis of known and putative ligand-binding sites with structural sketches

  |  |  |
  | --- | --- |
  | Type | Journal Article |
  | Author | Jun-Ichi Ito |
  | Author | Yasuo Tabei |
  | Author | Kana Shimizu |
  | Author | Kentaro Tomii |
  | Author | Koji Tsuda |
  | URL | http://onlinelibrary.wiley.com/doi/10.1002/prot.23232/full |
  | Volume | 80 |
  | Issue | 3 |
  | Pages | 747–763 |
  | Publication | Proteins: Structure, Function, and Bioinformatics |
  | Date | 2012 |
  | Accessed | 9/20/2013, 1:17:19 PM |
  | Library Catalog | Google Scholar |
  | Date Added | 10/11/2013, 10:29:15 AM |
  | Modified | 3/7/2014, 12:14:41 PM |

  ### Tags:

  - ligand-binding site
  - neighbor search algorithm
  - pocketome
  - structure and function

  ### Notes:

  - Present a new, fast, method for binding site prediction that does not rely on 3D structure alignment.  Instead, it represents the structures in a bit-string called a "structural sketch".

    How used SCOP/CATH:

    Didn't use SCOP or CATH data.  SCOP and CATH are cited to show that there are a number of popular tools that for categorize protein domains by structure and sequence similarities, but not for binding site similarities.

    Reference to SCOP:

    In databases such as SCOP4 and CATH,5 structures are divided into relatively large units, that is, domains, and then hierarchically classified according to their global structure and sequence.  However, proteins that do not exhibit any overall sequence or structural similarity can share common functions. Well-known instances include the Ser-His-Asp catalytic triad found in serine proteases6, 7 and the P-loop containing nucleotide-binding proteins.8 In these cases, only a few key residues close to the ligand are highly conserved, whereas their folds are distinct from one another. Given that most proteins exhibit their functions through interactions with other molecules (so-called ligands), most protein functions can be characterized by ligand-binding sites, that is, a set of residues directly involved in interaction with a ligand. Therefore, the pairwise comparison of ligand-binding sites, across different families or folds, is an appropriate approach to gaining functional and evolutionary knowledge about proteins.

  ### Attachments

  - 23232\_ftp.pdf
- ## PepBind: A Comprehensive Database and Computational Tool for Analysis of Protein-peptide Interactions

  |  |  |
  | --- | --- |
  | Type | Journal Article |
  | Author | Arindam Atanu Das |
  | Author | Om Prakash Sharma |
  | Author | Muthuvel Suresh Kumar |
  | Author | Ramadas Krishna |
  | Author | Premendu P. Mathur |
  | Volume | 11 |
  | Issue | 4 |
  | Pages | 241-246 |
  | Publication | Genomics Proteomics & Bioinformatics |
  | ISSN | 1672-0229; 2210-3244 |
  | Date | AUG 2013 |
  | Extra | BCI:BCI201300745874 |
  | DOI | 10.1016/j.gpb.2013.03.002 |
  | Abstract | Protein-peptide interactions, where one partner is a globular protein (domain) and the other is a flexible linear peptide, are key components of cellular processes predominantly in signaling and regulatory networks, hence are prime targets for drug design. To derive the details of the protein-peptide interaction mechanism is often a cumbersome task, though it can be made easier with the availability of specific databases and tools. The Peptide Binding Protein Database (PepBind) is a curated and searchable repository of the structures, sequences and experimental observations of 3100 protein-peptide complexes. The web interface contains a computational tool, protein inter-chain interaction (PICI), for computing several types of weak or strong interactions at the protein-peptide interaction interface and visualizing the identified interactions between residues in Jmol viewer. This initial database release focuses on providing protein-peptide interface information along with structure and sequence information for protein-peptide complexes deposited in the Protein Data Bank (PDB). Structures in PepBind are classified based on their cellular activity. More than 40% of the structures in the database are found to be involved in different regulatory pathways and nearly 20% in the immune system. These data indicate the importance of protein-peptide complexes in the regulation of cellular processes. PepBind is freely accessible at http://pepbind.bicpu.edu.in/. |
  | Date Added | 2/20/2014, 12:24:01 PM |
  | Modified | 2/20/2014, 12:24:01 PM |

  ### Notes:

  - Database of protein-peptide interactions.

    How SCOP is used:

    Search for proteins with similar structures using the FATCAT service from the PDB.

    SCOP reference:

    For a structure similarity search, we take advantage of the web ser- vice of PDB, which employs the FATCAT algorithm [31] to recognize homologous domains available at PepBind, SCOP [32] and PDP [33].

    ...

    Links to other related databases and servers for the queried protein are provided for further analysis of the structures. These resources include PDB [8], PDBsum [34], Pfam [35],

    CASTp [36], OCA Browser (http://bip.weizmann.ac.il/oca/), PSI/KB (http://sbkb.org/kb/), SRS [37], MMDB [38], PQS [39], SCOP [32], CATH [40], Proteopedia [41], Jena Library [42] and UniProt [43].

  ### Attachments

  - 1-s2.0-S1672022913000739-main.pdf
- ## Pfam 10 years on: 10 000 families and still growing

  |  |  |
  | --- | --- |
  | Type | Journal Article |
  | Author | Stephen John Sammut |
  | Author | Robert D. Finn |
  | Author | Alex Bateman |
  | Volume | 9 |
  | Issue | 3 |
  | Pages | 210-219 |
  | Publication | BRIEFINGS IN BIOINFORMATICS |
  | ISSN | 1467-5463 |
  | Date | May 2008 |
  | DOI | 10.1093/bib/bbn010 |
  | Language | English |
  | Abstract | Classifications of proteins into groups of related sequences are in some respects like a periodic table for biology, allowing us to understand the underlying molecular biology of any organism. Pfam is a large collection of protein domains and families. Its scientific goal is to provide a complete and accurate classification of protein families and domains. The next release of the database will contain over 10 000 entries, which leads us to reflect on how far we are from completing this work. Currently Pfam matches 72 of known protein sequences, but for proteins with known structure Pfam matches 95, which we believe represents the likely upper bound. Based on our analysis a further 28 000 families would be required to achieve this level of coverage for the current sequence database. We also show that as more sequences are added to the sequence databases the fraction of sequences that Pfam matches is reduced, suggesting that continued addition of new families is essential to maintain its relevance. |
  | Date Added | 10/11/2013, 10:29:15 AM |
  | Modified | 10/11/2013, 10:29:15 AM |

  ### Tags:

  - classification
  - coverage
  - hidden Markov model
  - Pfam
  - protein families

  ### Notes:

  - Discusses state of Pfam.

    How SCOP is used:

    Gives summary statistics on SCOP.

    SCOP reference:

    Using known structures to group families together into larger superfamilies, the SCOP data- base [8] has now identified almost 1600 distinct evolutionary families, and growth appears to be increasing in a linear fashion.

  ### Attachments

  - Brief Bioinform-2008-Sammut-210-9.pdf
- ## Pfam: a comprehensive database of protein domain families based on seed alignments

  |  |  |
  | --- | --- |
  | Type | Journal Article |
  | Author | Erik LL Sonnhammer |
  | Author | Sean R. Eddy |
  | Author | Richard Durbin |
  | URL | http://www1.cs.columbia.edu/~rkuang/candidacy/pfam.pdf |
  | Volume | 28 |
  | Issue | 3 |
  | Pages | 405–420 |
  | Publication | Proteins Structure Function and Genetics |
  | Date | 1997 |
  | Accessed | 2/28/2013, 3:33:53 PM |
  | Library Catalog | Google Scholar |
  | Short Title | Pfam |
  | Date Added | 10/11/2013, 10:29:15 AM |
  | Modified | 10/11/2013, 10:29:15 AM |

  ### Notes:

  - First Pfam paper.

    How SCOP is used:

    Provides links to SCOP families from the PDB's mappings.

    SCOP reference:

    These families are cross-referenced to the protein structure database PDB, which is used to link them to the structural classification database SCOP12 from the Pfam WWW servers.

  ### Attachments

  - [PDF] from columbia.edu
- ## PFClust: a novel parameter free clustering algorithm

  |  |  |
  | --- | --- |
  | Type | Journal Article |
  | Author | Lazaros Mavridis |
  | Author | Neetika Nath |
  | Author | John BO Mitchell |
  | URL | http://link.springer.com/article/10.1186/1471-2105-14-213 |
  | Volume | 14 |
  | Issue | 1 |
  | Pages | 1–21 |
  | Publication | BMC bioinformatics |
  | Date | 2013 |
  | Accessed | 9/23/2013, 10:15:21 AM |
  | Library Catalog | Google Scholar |
  | Short Title | PFClust |
  | Date Added | 10/11/2013, 10:29:15 AM |
  | Modified | 3/7/2014, 12:09:12 PM |

  ### Notes:

  - Present PFClust, a novel parameter free clustering algorithm, and evaluate on synthetic data sets and CATH data.

    How SCOP is used:

    Use type: Do not use SCOP data.

    Description: Negative reference.  Use CATH instead of SCOP to evaluate clustering method on structure classification.

    How CATH is used:

    Benchmark an algorithm.

    SCOP reference:

    Extending these ideas to three-dimensional (3D) pro- tein structure provides the interesting task of clustering and classifying protein domain folds. During the early 1990s the Protein Data Bank (PDB) [18] held only a few thousand 3D crystal structures, and several initiatives for protein fold classification were proposed with CATH [19] and SCOP [20] being the best known. These were based on either manual curation (SCOP) or computer- aided manual curation (CATH). Common to both ap- proaches is that the human curator has the final word in the classification decision. With the exponential growth of the number of 3D high resolution structures depos- ited in the PDB during the last decade [21], reaching 87,085 structures at the beginning of 2013, the rate- limiting manual part of the curation process restricts our capacity to understand the full structural diversity of proteins. Hence it would be ideal if a fully automated process could classify protein domains and cluster them into structurally similar groups.

    ....

    Mavridis et al. proposed in the same paper a novel structure-based indexing for existing classification schemes such as CATH [19] and SCOP [20]. Their proposed consensus algorithm works well for only some of the cases it was tested on, because of the structural diversity of a number of protein domains assigned to the same super- families [28].

    CATH reference:

    We also demonstrate the ability of PFClust to classify the three dimensional structures of protein domains, using a set of folds taken from the structural bioinformatics database CATH.

    ...

    Conclusions: We show that PFClust is able to cluster the test datasets a little better, on average, than any of the other algorithms, and furthermore is able to do this without the need to specify any external parameters. Results on the synthetic datasets demonstrate that PFClust generates meaningful clusters, while our algorithm also shows excellent agreement with the correct assignments for a dataset extracted from the CATH part-manually curated classification of protein domain structures.

  ### Attachments

  - 1471-2105-14-213.pdf
- ## Pharmacophore Binding Motifs for Nicotinamide Adenine Dinucleotide Analogues Across Multiple Protein Families: A Detailed Contact-Based Analysis of the Interaction between Proteins and NAD (P) Cofactors

  |  |  |
  | --- | --- |
  | Type | Journal Article |
  | Author | Ilenia Giangreco |
  | Author | Martin J. Packer |
  | URL | http://pubs.acs.org/doi/abs/10.1021/jm400644z |
  | Publication | Journal of medicinal chemistry |
  | Date | 2013 |
  | Accessed | 9/20/2013, 1:12:22 PM |
  | Library Catalog | Google Scholar |
  | Short Title | Pharmacophore Binding Motifs for Nicotinamide Adenine Dinucleotide Analogues Across Multiple Protein Families |
  | Date Added | 10/11/2013, 10:29:15 AM |
  | Modified | 3/7/2014, 12:09:44 PM |

  ### Notes:

  - Computational study of protein-binding pharmacophore of NAD and its close analogs in all protein-ligand structures in the PDB. Analyze a dataset of all 1932 NAD(P)-containing proteins in the PDB.

    How SCOP is used:

    1. Mainly, provide summary statistics on the breakdown of folds in their data set (mostly NAD(P)-binding Rossmand-fold and TIM beta/alpha barrel).

    2. Get domain data and classification for each protein in their data set from SCOP, Pfam, and CATH and provide in supporting information.

    How CATH is used:

    See 2 above.

    SCOP reference:

    Data Set. The present work describes a detailed analysis of all NAD(P)-containing proteins deposited into the RCSB PDB. A total number of 1932 structures were downloaded after browsing the database to find structures complexed with the NAD(P). All data are updated as of 23/02/2012 when we first started working on this project. Table 1 shows how many structures we found for each possible form of the NAD that we used as a query for the field ligand ID. Although the cofactor can be indicated by seven different three letter codes, we will use NAD to refer to all of them.

    For each search, we generated a summary report including structure details (e.g., resolution, experimental technique), domain details (e.g., PFAM ID,23 SCOP ID,24 CATH ID25), and biological details (e.g., macromolecule’s name, EC number) which can be used to classify proteins in the data set. As an example, by grouping the 1932 structures based on the PFAM ID, we found 130 different possible combinations including 101 structures without the PFAM ID specified. The top four protein families are, instead, the short-chain dehydrogenase, the aldo- keto reductase, the dihydrofolatereductase, and the epimerase, respectively. **Alternatively, the two most populated folds based on the SCOP ID are the NAD(P)-binding Rossmann-fold domains and the TIM** **β/α****-barrel.** Tables collecting the full classification based on the PFAM ID, the SCOP ID, and others, are provided as Supporting Information.

  ### Attachments

  - jm400644z.pdf
  - Snapshot

    Abstract

    We have analyzed the protein-binding pharmacophore of NAD and its close analogues in all protein–ligand structures available in the RCSB database as of February 2012; this analysis has then been used to assess the novelty of structures emerging after that date. We show that proteins have evolved diverse pharmacophore motifs for binding the adenine moiety, fewer, but still diverse, motifs for nicotinamide, and a very limited set of motifs for binding the pyrophosphate linker. Our exhaustive analysis includes a pharmacophore contact analysis for over 1900 protein–ligand structures containing NAD analogues; we have benchmarked this set of contacts against nearly 27 000 protein–ligand structures to demonstrate that the diversity of interactions seen with NAD is very similar to that seen for all other ligands. Hence, variation in binding motifs for NAD is not distinct from that observed for other ligands and they show significant variation across protein families.
- ## Pine nut allergy: Clinical features and major allergens characterization

  |  |  |
  | --- | --- |
  | Type | Journal Article |
  | Author | Beatriz Cabanillas |
  | Author | Hsiaopo Cheng |
  | Author | Casey C. Grimm |
  | Author | Barry K. Hurlburt |
  | Author | Julia Rodriguez |
  | Author | Jesus F. Crespo |
  | Author | Soheila J. Maleki |
  | Volume | 56 |
  | Issue | 12 |
  | Pages | 1884-1893 |
  | Publication | MOLECULAR NUTRITION & FOOD RESEARCH |
  | ISSN | 1613-4125 |
  | Date | December 2012 |
  | DOI | 10.1002/mnfr.201200245 |
  | Language | English |
  | Abstract | Scope The aims of this study were to evaluate IgE-mediated hypersensitivity to pine nut with details of clinical reactions and to characterize major pine nut allergens. Methods and results The study included ten consecutive teenagers and adults diagnosed with IgE-mediated clinical allergy to pine nut. Two major pine nut allergens were purified and identified and the secondary structures and susceptibility to digestion were characterized. Severe reactions represent 80% of allergic reactions to pine nut in this study. Moreover, 70% of the patients were monosensitized to this nut. Two major allergens with molecular weights of 6 and 50 kDa were purified and identified as albumin and vicilin, respectively. The 6 kDa protein (albumin), rich in a-helix content, was far more stable to peptic and tryptic digestion as compared with 50 kDa protein (vicilin), which was quickly broken down. The secondary structure of the purified 50 kDa protein showed 41% beta-sheet, 5% alpha-helix, and 54% random coil and/or loops. Conclusion Eighty percent of allergic reactions to pine nut in the ten patients included in this study were severe. Most patients (70%) were monosensitized to this nut. Two major allergens with molecular weights of 6 and 50 kDa were purified and identified as albumin and vicilin, respectively. |
  | Date Added | 10/11/2013, 10:29:15 AM |
  | Modified | 10/11/2013, 10:29:15 AM |

  ### Tags:

  - Albumin
  - Allergens
  - Anaphylaxis
  - Pine nut allergy
  - Vicilin

  ### Notes:

  - Experimental study of allergens in the pine nut. They took 2 of the allergens and purified it and tested against digestion using pepsin and trypsin.

    **SCOP Use**

    SCOP was just used to look up the structure of the protein (class level).

    **SCOP Reference**

    CD analysis demonstrated that purified 6 kDa protein was rich in ⬚⬚-helix content and did not have ⬚⬚-sheet structure. Ac- cording to numerous studies, 2S albumins are characterized by a similar three-dimensional structure enriched in ⬚⬚-helix structure [18].

  ### Attachments

  - mnfr1854.pdf
- ## Pleiotropic Roles of a Ribosomal Protein in Dictyostelium discoideum

  |  |  |
  | --- | --- |
  | Type | Journal Article |
  | Author | Smita Amarnath |
  | Author | Trupti Kawli |
  | Author | Smita Mohanty |
  | Author | Narayanaswamy Srinivasan |
  | Author | Vidyanand Nanjundiah |
  | Volume | 7 |
  | Issue | 2 |
  | Pages | e30644 |
  | Publication | Plos One |
  | ISSN | 1932-6203 |
  | Date | FEB 17 2012 |
  | Extra | WOS:000302853600047 |
  | DOI | 10.1371/journal.pone.0030644 |
  | Abstract | The cell cycle phase at starvation influences post-starvation differentiation and morphogenesis in Dictyostelium discoideum. We found that when expressed in Saccharomyces cerevisiae, a D. discoideum cDNA that encodes the ribosomal protein S4 (DdS4) rescues mutations in the cell cycle genes cdc24, cdc42 and bem1. The products of these genes affect morphogenesis in yeast via a coordinated moulding of the cytoskeleton during bud site selection. D. discoideum cells that over-or under-expressed DdS4 did not show detectable changes in protein synthesis but displayed similar developmental aberrations whose intensity was graded with the extent of over-or under-expression. This suggested that DdS4 might influence morphogenesis via a stoichiometric effect - specifically, by taking part in a multimeric complex similar to the one involving Cdc24p, Cdc42p and Bem1p in yeast. In support of the hypothesis, the S. cerevisiae proteins Cdc24p, Cdc42p and Bem1p as well as their D. discoideum cognates could be co-precipitated with antibodies to DdS4. Computational analysis and mutational studies explained these findings: a C-terminal domain of DdS4 is the functional equivalent of an SH3 domain in the yeast scaffold protein Bem1p that is central to constructing the bud site selection complex. Thus in addition to being part of the ribosome, DdS4 has a second function, also as part of a multi-protein complex. We speculate that the existence of the second role can act as a safeguard against perturbations to ribosome function caused by spontaneous variations in DdS4 levels. |
  | Date Added | 2/20/2014, 12:24:01 PM |
  | Modified | 2/20/2014, 12:24:01 PM |

  ### Notes:

  - Experimental and computational study of important cell-cycle genes in two organisms.

    How SCOP is used:

    get superfamily classification and domain boundaries for a data set.

    SCOP reference:

    Computational sequence analysis and structure prediction

    Sequences of ScBem1p, DdS4, ScS4 and their related proteins were been obtained from Uniprot [62]. Domain assignments based on functional similarity was obtained from Pfam [26] family database by doing a HMM based search in the protein family database. Structural domain assignments were obtained by using 3D-Jigzaw [63], an automated server for comparative modeling and Superfmaily database system [64] which queries the sequence against SCOP [65] families and superfamilies. 

    ...

    Supporting Information

    Figure S1 Structural insights into DdS4 interactions. (A) Domain organization of ScBem1p (I) and DdS4p (II). SCOP superfamilies were obtained from Superfamily.org database [77]. Definitions for SCOP ids are as follows: b.34.2 : All Beta protein, SH3 like barrel, SH3 domain; b.34.5 : All Beta, SH3-like barrel, Translation proteins SH3- like domain; d.189.1 -: Alpha and beta proteins, PX domain; d.15.2 -: Alpha and beta proteins, beta- Grasp(ubiquitin like), CAD & PB1 domain.

  ### Attachments

  - journal.pone.0030644.pdf
- ## PocketAnnotate: towards site-based function annotation

  |  |  |
  | --- | --- |
  | Type | Journal Article |
  | Author | Praveen Anand |
  | Author | Kalidas Yeturu |
  | Author | Nagasuma Chandra |
  | Volume | 40 |
  | Issue | W1 |
  | Pages | W400-W408 |
  | Publication | Nucleic Acids Research |
  | Date | JUL 2012 |
  | Extra | WOS:000306670900066 |
  | DOI | 10.1093/nar/gks421 |
  | Library Catalog | ISI Web of Knowledge |
  | Abstract | A computational pipeline PocketAnnotate for functional annotation of proteins at the level of binding sites has been proposed in this study. The pipeline integrates three in-house algorithms for site-based function annotation: PocketDepth, for prediction of binding sites in protein structures; PocketMatch, for rapid comparison of binding sites and PocketAlign, to obtain detailed alignment between pair of binding sites. A novel scheme has been developed to rapidly generate a database of non-redundant binding sites. For a given input protein structure, putative ligand-binding sites are identified, matched in real time against the database and the query substructure aligned with the promising hits, to obtain a set of possible ligands that the given protein could bind to. The input can be either whole protein structures or merely the substructures corresponding to possible binding sites. Structure-based function annotation at the level of binding sites thus achieved could prove very useful for cases where no obvious functional inference can be obtained based purely on sequence or fold-level analyses. An attempt has also been made to analyse proteins of no known function from Protein Data Bank. PocketAnnotate would be a valuable tool for the scientific community and contribute towards structure-based functional inference. The web server can be freely accessed at http://proline.biochem.iisc.ernet.in/pocketannotate/. |
  | Short Title | PocketAnnotate |
  | Date Added | 10/8/2014, 12:49:22 PM |
  | Modified | 10/8/2014, 1:33:27 PM |

  ### Attachments

  - Full Text PDF
  - PubMed entry
  - Snapshot
- ## Porphyrin and heme metabolism and the porphyrias

  |  |  |
  | --- | --- |
  | Type | Journal Article |
  | Author | Herbert L. Bonkovsky |
  | Author | Jun-Tao Guo |
  | Author | Weihong Hou |
  | Author | Ting Li |
  | Author | Tarun Narang |
  | Author | Manish Thapar |
  | URL | http://onlinelibrary.wiley.com/doi/10.1002/cphy.c120006/full |
  | Publication | Comprehensive Physiology |
  | Date | 2013 |
  | Accessed | 9/18/2013, 1:43:01 PM |
  | Library Catalog | Google Scholar |
  | Date Added | 10/11/2013, 10:29:15 AM |
  | Modified | 10/11/2013, 10:29:15 AM |

  ### Notes:

  - This article, from a journal on physiology, discusses porphyrias, a disorder that results from defects in heme synthesis.  SCOP is cited in order to inform the reader of the diversity of hemoprotein structures, and lists the top four superfamilies that hemoproteins are found in.

    How SCOP is used:

    Provide background on a protein of interest.

    SCOP reference:

    "Hemoproteins are found in at least 31 different structural fold conformations in all the four major classes based on structural classification of proteins (SCOP) (170) and are dominated by the all-α-fold structures with globin-like (a.1), cytochrome P450 (a.104), cytochrome c (a.3), and multiheme cytochromes (a.138) as the top four in terms of frequency of occurrence (139)."

  ### Attachments

  - c120006.pdf
- ## PoSSuM: a database of similar protein-ligand binding and putative pockets

  |  |  |
  | --- | --- |
  | Type | Journal Article |
  | Author | Jun-Ichi Ito |
  | Author | Yasuo Tabei |
  | Author | Kana Shimizu |
  | Author | Koji Tsuda |
  | Author | Kentaro Tomii |
  | Volume | 40 |
  | Issue | D1 |
  | Pages | D541-D548 |
  | Publication | Nucleic Acids Research |
  | ISSN | 0305-1048 |
  | Date | JAN 2012 |
  | Extra | WOS:000298601300081 |
  | DOI | 10.1093/nar/gkr1130 |
  | Abstract | Numerous potential ligand-binding sites are available today, along with hundreds of thousands of known binding sites observed in the PDB. Exhaustive similarity search for such vastly numerous binding site pairs is useful to predict protein functions and to enable rapid screening of target proteins for drug design. Existing databases of ligand-binding sites offer databases of limited scale. For example, SitesBase covers only similar to 33 000 known binding sites. Inferring protein function and drug discovery purposes, however, demands a much more comprehensive database including known and putative-binding sites. Using a novel algorithm, we conducted a large-scale all-pairs similarity search for 1.8 million known and potential binding sites in the PDB, and discovered over 14 million similar pairs of binding sites. Here, we present the results as a relational database Pocket Similarity Search using Multiple-sketches (PoSSuM) including all the discovered pairs with annotations of various types. PoSSuM enables rapid exploration of similar binding sites among structures with different global folds as well as similar ones. Moreover, PoSSuM is useful for predicting the binding ligand for unbound structures, which provides important clues for characterizing protein structures with unclear functions. The PoSSuM database is freely available at http://possum.cbrc.jp/PoSSuM/. |
  | Date Added | 2/20/2014, 12:24:01 PM |
  | Modified | 3/7/2014, 12:14:40 PM |

  ### Notes:

  - Present PoSSum database that classifies similar binding sites.

    How SCOP/CATH is used:

    Annotate with both CATH and SCOP classification.  Annotate SCOP class,fold,superfamily,and family.

    SCOP reference:

    Because, all sites were annotated with in- formation of various types such as CATH (19), SCOP (20), EC numbers (21) and Gene Ontology (GO) terms (22), users can easily scrutinize similar binding sites between proteins with different folds or similar catalytic sites between enzymes with different EC numbers.

    ...

    Annotation to binding sites

    To facilitate subsequent analyses, all binding sites were annotated to the greatest degree possible using CATH (version 3.4) codes, SCOP (version 1.75) domain classifi- cation codes EC commission numbers, and three biologic- al domains of GO terms (molecular functions, biological processes and cellular components).

    Each site was annotated with four levels of CATH codes, i.e. Class, Architecture, Topology and Homology, and of SCOP codes, i.e. Class, Fold, Superfamily and Family, by matching the binding site residues against the domain region defined by CATH or SCOP. One binding site can reside between multiple domains. In such a case, we found all domains involved with the binding site, and annotated the site with the multiple CATH and SCOP codes. Among those domains, many had not been defined in CATH or SCOP. In our study, if the number of binding site residues that were overlapped with undefined domains was >70% of all binding site residues, then we regarded the binding site as an undefined one and assigned ‘0.0.0.0’ to it. Furthermore, we assigned EC numbers and GO terms to binding sites. Because, an EC number and/or a GO term was assigned to each protein chain, we detected the largest overlapped protein chain for the binding site, and assigned the corresponding EC number/GO term to the site.

    ...

    All of the obtained 14 million pairs were compiled into a relational database, POSSUM, along with their corres- ponding annotations such as CATH, SCOP, EC and GO.

    ...

    Only a CATH and SCOP code that accounts for the largest part of the binding site is displayed if the binding site has multiple CATH or SCOP codes.

  ### Attachments

  - Nucl. Acids Res.-2012-Ito-D541-8.pdf
- ## PPM-Dom: A novel method for domain position prediction

  |  |  |
  | --- | --- |
  | Type | Journal Article |
  | Author | Jing Sun |
  | Author | Runyu Jing |
  | Author | Yuelong Wang |
  | Author | Tuanfei Zhu |
  | Author | Menglong Li |
  | Author | Yizhou Li |
  | Volume | 47 |
  | Pages | 8-15 |
  | Publication | Computational Biology and Chemistry |
  | ISSN | 1476-9271; 1476-928X |
  | Date | DEC 2013 |
  | Extra | WOS:000329270700003 |
  | DOI | 10.1016/j.compbiolchem.2013.06.002 |
  | Abstract | Domains are the structural basis of the physiological functions of proteins, and the prediction of which is an advantageous process on the study of protein structure and function. This article proposes a new complete automatic prediction method, PPM-Dom (Domain Position Prediction Method), for predicting the particular positions of domains in a target protein via its atomic coordinate. The presented method integrates complex networks, community division, and fuzzy mean operator (FMO). The whole sequences are divided into potential domain regions by the complex network and community division, and FMO allows the final determination for the domain position. This method will suffice to predict regions that will form a domain structure and those that are unstructured based on completely new atomic coordinate information of the query sequence, and be able to separate different domains in the same query sequence from each other. On evaluating the performance using an independent testing dataset, PPM-Dom reached 91.41% for prediction accuracy, 96.12% for sensitivity and 92.86% for specificity. The tool bag of PPM-Dom is freely available at http://cic.scu.edu.cn/bioinformatics/PPMDom.zip. (C) 2013 The Authors. Published by Elsevier Ltd. All rights reserved. |
  | Date Added | 2/13/2014, 4:13:17 PM |
  | Modified | 3/7/2014, 12:08:47 PM |

  ### Notes:

  - Present PPM-Dom method for domain boundary prediction given sequence.

    How SCOP/CATH is used:

    Validate domains predicted against both SCOP and CATH data.

    SCOP reference:

    Furthermore, the prediction performances based on both SCOP (Andreeva et al., 2004, 2008; Brenner et al., 2000) and CATH (Greene et al., 2007; Orengo et al., 1997) were compared. Although SCOP database is considered the standard for protein structure classification (Day et al., 2003), there is not a single or true domain assignments but only different assignments that can dif- fer on some case. When taken the domain information of SCOP as reference dataset, while the domain information of CATH as the measure of the prediction results. On evaluating the perfor- mance using the same independent testing dataset of 100 proteins, the prediction accuracy, sensibility and specificity of PPM-Dom reached 30.81%, 33.50% and 30.98%, respectively. If the domain information of SCOP and CATH are resemble, then the prediction performance of the two parts would not have too much differ- ence. However, CATH and SCOP show great differences on domain assignments, especially for the position and number of domains in protein sequences, since the same protein sequence may be defined have one domain in SCOP and eight domains in the CATH at the same time. That is, the low results may attributed to the different domain information and classification between the two databanks.

  ### Attachments

  - 1-s2.0-S1476927113000595-main.pdf
- ## PredictFold-PSS-3D1D: A Protein Fold Recognition Server for Predicting Folds from the Twilight Zone Sequences

  |  |  |
  | --- | --- |
  | Type | Journal Article |
  | Author | Kaliappan Ganesan |
  | Author | Subbiah Parthasarathy |
  | Volume | 8 |
  | Issue | 5 |
  | Pages | 552-556 |
  | Publication | Current Bioinformatics |
  | ISSN | 1574-8936; 2212-392X |
  | Date | NOV 2013 |
  | Extra | WOS:000325754000005 |
  | Abstract | The PredictFold-PSS-3D1D is an online protein fold recognition web server used to predict the possible folds from the twilight zone protein sequences. In this server, an improved 3D1D profile method (Ganesan and Parthasarathy, J. Struct. Funct. Genomics, 12, 181-189, 2011) is employed, wherein, the inclusion of predicted secondary structure information improves fold recognition. The PredictFold-PSS-3D1D server accepts amino acid sequences and their predicted secondary structure data as input and aligns them with the 3D1D profiles of known SCOP folds in a database. The alignments are ranked by the z-values and P-values. The top 5 ranks of the SCOP folds from the database are listed along with a link to 'View SCOP details'. The folds with z-values >= 3.0 and P-values <= 0.05 are indicated as 'Predicted Fold' for the given query twilight zone protein sequence. This server is available in our PredictFold web server at http://bioinfo.bdu.ac.in/pss3d1d/. |
  | Date Added | 2/12/2014, 2:18:08 PM |
  | Modified | 2/12/2014, 2:18:08 PM |

  ### Notes:

  - Unavailable.
- ## Predicting enzymatic function from global binding site descriptors

  |  |  |
  | --- | --- |
  | Type | Journal Article |
  | Author | Andrea Volkamer |
  | Author | Daniel Kuhn |
  | Author | Friedrich Rippmann |
  | Author | Matthias Rarey |
  | URL | http://onlinelibrary.wiley.com/doi/10.1002/prot.24205/abstract |
  | Rights | Copyright © 2012 Wiley Periodicals, Inc. |
  | Volume | 81 |
  | Issue | 3 |
  | Pages | 479-489 |
  | Publication | Proteins: Structure, Function, and Bioinformatics |
  | ISSN | 1097-0134 |
  | Date | 2013 |
  | Journal Abbr | Proteins |
  | DOI | 10.1002/prot.24205 |
  | Accessed | 12/9/2014, 5:32:42 AM |
  | Library Catalog | Wiley Online Library |
  | Language | en |
  | Abstract | Due to the rising number of solved protein structures, computer-based techniques for automatic protein functional annotation and classification into families are of high scientific interest. DoGSiteScorer automatically calculates global descriptors for self-predicted pockets based on the 3D structure of a protein. Protein function predictors on three levels with increasing granularity are built by use of a support vector machine (SVM), based on descriptors of 26632 pockets from enzymes with known structure and enzyme classification. The SVM models represent a generalization of the available descriptor space for each enzyme class, subclass, and substrate-specific sub-subclass. Cross-validation studies show accuracies of 68.2% for predicting the correct main class and accuracies between 62.8% and 80.9% for the six subclasses. Substrate-specific recall rates for a kinase subset are 53.8%. Furthermore, application studies show the ability of the method for predicting the function of unknown proteins and gaining valuable information for the function prediction field. Proteins 2013. © 2012 Wiley Periodicals, Inc. |
  | Date Added | 12/9/2014, 5:32:42 AM |
  | Modified | 12/9/2014, 5:32:42 AM |

  ### Tags:

  - descriptor-based
  - DoGSite
  - EC number
  - enzyme classification
  - function prediction
  - pocket prediction
  - structure-based
  - support vector machine

  ### Attachments

  - Full Text PDF
  - Snapshot
- ## Predicting enzymatic function from global binding site descriptors

  |  |  |
  | --- | --- |
  | Type | Journal Article |
  | Author | Andrea Volkamer |
  | Author | Daniel Kuhn |
  | Author | Friedrich Rippmann |
  | Author | Matthias Rarey |
  | Volume | 81 |
  | Issue | 3 |
  | Pages | 479-489 |
  | Publication | Proteins-Structure Function and Bioinformatics |
  | ISSN | 0887-3585 |
  | Date | MAR 2013 |
  | Extra | WOS:000314179600011 |
  | DOI | 10.1002/prot.24205 |
  | Abstract | Due to the rising number of solved protein structures, computer-based techniques for automatic protein functional annotation and classification into families are of high scientific interest. DoGSiteScorer automatically calculates global descriptors for self-predicted pockets based on the 3D structure of a protein. Protein function predictors on three levels with increasing granularity are built by use of a support vector machine (SVM), based on descriptors of 26632 pockets from enzymes with known structure and enzyme classification. The SVM models represent a generalization of the available descriptor space for each enzyme class, subclass, and substrate-specific sub-subclass. Cross-validation studies show accuracies of 68.2% for predicting the correct main class and accuracies between 62.8% and 80.9% for the six subclasses. Substrate-specific recall rates for a kinase subset are 53.8%. Furthermore, application studies show the ability of the method for predicting the function of unknown proteins and gaining valuable information for the function prediction field. Proteins 2013. (C) 2012 Wiley Periodicals, Inc. |
  | Date Added | 2/13/2014, 4:13:17 PM |
  | Modified | 10/8/2014, 1:32:41 PM |

  ### Tags:

  - 3d coordinate templates
  - active-sites
  - alignment
  - catalytic residues
  - database
  - descriptor-based
  - DoGSite
  - EC number
  - enzyme classification
  - Enzymes
  - function prediction
  - ligand-binding
  - pocket prediction
  - protein function prediction
  - sequence
  - structural classification
  - structure-based
  - support vector machine

  ### Attachments

  - 24205\_ftp.pdf
  - Snapshot
- ## Predicting metal-binding sites from protein sequence

  |  |  |
  | --- | --- |
  | Type | Journal Article |
  | Author | Andrea Passerini |
  | Author | Marco Lippi |
  | Author | Paolo Frasconi |
  | URL | http://dl.acm.org/citation.cfm?id=2077958 |
  | Volume | 9 |
  | Issue | 1 |
  | Pages | 203–213 |
  | Publication | IEEE/ACM Transactions on Computational Biology and Bioinformatics (TCBB) |
  | Date | 2012 |
  | Accessed | 9/23/2013, 10:14:36 AM |
  | Library Catalog | Google Scholar |
  | Date Added | 10/11/2013, 10:29:15 AM |
  | Modified | 10/11/2013, 10:29:15 AM |

  ### Tags:

  - Interesting

  ### Notes:

  - How SCOP is used:

    Collected a data set of proteins with a metal-binding sites from the PDB, removing structures not classified in SCOP.  Filtered with 90% sequence identity threshold using CD-HIT

    Performed leave-one-out superfamily and fold training and benchmarking in order to evaluate whether their algorithm would work for domains within superfamilies or folds that had not been included in the training set.

    SCOP reference:

    ...

    The second data set was built using a more stringent criterion to remove redundancy, by taking into account the Structural Classification of Proteins (SCOP) hierarchy [40]: in this case, in fact, we aim at measuring the ability of the predictor in identifying metal binding sites within proteins belonging to SCOP superfamilies – or folds – which are not observed in the training set. First, we extracted from the December 2009 release of PDB 17,783 protein chains with at least a CYS or HIS bonded to a metal ion3. We detected ligands using a cutoff of 3A ̊ on the distance between the metal ion (or complex) and the sulfur or nitrogen atoms for cysteines and histidines respectively. We then discarded 6,090 entries not mapped in the 1.75 release (June 2009) of the SCOP database. We also removed very few cases in which the number of metal binding sites was greater than five. Finally, we obtained a sequence-unique subset of 1,824 protein chains by running CD-HIT v4.0 [41] with sequence identity threshold set to 0.9 (default value). The data set contained 12,323 HIS and 8,290 CYS. 54% of the resulting chains were bonded to zinc, 14% to heme groups, 7% to cadmium, 7% to iron, 7% to iron- solfur groups, 5% to copper. Following the procedure described above, we found 122 CYS and 12 HIS coordinating multiple ions. In these cases we kept in the data set only the closest ligand-ion pair.\...

    5.2 SCOP-based data set

    When using the SCOP-based data set, we employed a different strategy to perform the experiments: in this case, the goal is to measure the performance of the predictor on SCOP superfamilies – or folds – which are not observed in the training set. We refer to this procedure as leave-k-superfamilies-out, or leave- k-folds-out, where folds here are intended as SCOP hierarchy folds, and should not be confused with folds of the standard k-fold-cross-validation procedure. We partitioned the data set in k = 10 subsets of chains, maintaining the same average percentage of ligands in each subset, and with the additional constraint that no pair of chains in different subsets belonged to the same SCOP superfamily. We also prepared a second version of this data set, where we considered SCOP folds instead of superfamilies: in this case, we discarded multi-domain chains, as building the partition would have been otherwise unfeasible. This version of the data set was therefore reduced to 1,466 chains.

  ### Attachments

  - [PDF] from unitn.it
  - Snapshot
- ## Predicting new indications for approved drugs using a proteochemometric method

  |  |  |
  | --- | --- |
  | Type | Journal Article |
  | Author | Sivanesan Dakshanamurthy |
  | Author | Naiem T. Issa |
  | Author | Shahin Assefnia |
  | Author | Ashwini Seshasayee |
  | Author | Oakland J. Peters |
  | Author | Subha Madhavan |
  | Author | Aykut Uren |
  | Author | Milton L. Brown |
  | Author | Stephen W. Byers |
  | URL | http://pubs.acs.org/doi/abs/10.1021/jm300576q |
  | Volume | 55 |
  | Issue | 15 |
  | Pages | 6832–6848 |
  | Publication | Journal of Medicinal Chemistry |
  | Date | 2012 |
  | Accessed | 9/23/2013, 10:13:41 AM |
  | Library Catalog | Google Scholar |
  | Date Added | 10/11/2013, 10:29:15 AM |
  | Modified | 10/11/2013, 10:29:15 AM |

  ### Tags:

  - Interesting
  - Very interesting

  ### Notes:

  - The paper describes a novel method called "Train, Match, Fit, Streamline," which predicts interactions with drugs and their targets. The method was tested on >3500 drugs across >2000 human proteins with a high degree of accuracy. 

    **SCOP Use**

    Used for measuring "value of promiscuity" for tested drugs.  They downloaded the entire SCOP database and created each domain entry with a reference to its fold and family.  For each drug, counted the folds and families in which interacting domains were found.   Used to compile a list of the "5 most promiscuous" drugs.

    **SCOP Reference**

    Drug promiscuity and overlap of protein family and fold

    In drug development, it is important that molecules reach and interact with their desired targets while minimizing cross-target interactions. However, many FDA approved drugs have notable side effects that consumers are warned about prior to their administration. Thus, we were interested in investigating whether our method could more formally predict the extent of drug promiscuity/non-specificity. We evaluated the extent of promiscuity in terms of protein family and fold classifications. We used the entire SCOP database and parsed it to create a CSV file that matches PDB IDs with their corresponding fold and family keys (41). For each molecule in the drug data set, we then determined the targets for which they are considered the top 1 hit and used those PDB IDs to determine the folds and families they correspond to. Using this information, we were able to determine the numbers of unique folds and families that the drugs are targeting. To objectively quantify the “promiscuity” of a molecule, we devised a numerical score to create the “value of promiscuity”. This value is the combined sum of the number of unique folds and the number of unique families that a particular molecule is predicted to hit. The greater this value is, the greater the extent of promiscuity. According to Figure 6, the three most promiscuous compounds (DB02197, DB03869 and staurosporine) are kinase inhibitors. As indicated above, staurosporine is a “broad–specificity kinase inhibitor” targeting multiple families especially kinases (40,42). Furthermore, Tables 2 and 3 show that the five most promiscuous drugs are predicted to interact with proteins that have many overlapping folds/families. Intrigued by this result, we further explored whether shape similarities of protein binding pockets may exhibit drug promiscuity.

    41. Murzin AG, Brenner SE, Hubbard T, Chothia C. SCOP: a structural classification of proteins database for the investigation of sequences and structures. J Mol Biol. 1995; 247:536–540. [PubMed: 7723011]

  ### Attachments

  - nihms394208.pdf
  - Snapshot
- ## Predicting protein contact map using evolutionary and physical constraints by integer programming

  |  |  |
  | --- | --- |
  | Type | Journal Article |
  | Author | Zhiyong Wang |
  | Author | Jinbo Xu |
  | Volume | 29 |
  | Issue | 13 |
  | Pages | 266-273 |
  | Publication | Bioinformatics |
  | ISSN | 1367-4803 |
  | Date | JUL 1 2013 |
  | Extra | Joint 21st Annual Meeting of Intelligent Systems for Molecular Biology (ISMB) / 12th European Conference on Computational Biology (ECCB), Berlin, GERMANY, JUL 21-23, 2013 |
  | DOI | 10.1093/bioinformatics/btt211 |
  | Language | English |
  | Abstract | Motivation: Protein contact map describes the pairwise spatial and functional relationship of residues in a protein and contains key information for protein 3D structure prediction. Although studied extensively, it remains challenging to predict contact map using only sequence information. Most existing methods predict the contact map matrix element-by-element, ignoring correlation among contacts and physical feasibility of the whole-contact map. A couple of recent methods predict contact map by using mutual information, taking into consideration contact correlation and enforcing a sparsity restraint, but these methods demand for a very large number of sequence homologs for the protein under consideration and the resultant contact map may be still physically infeasible. Results: This article presents a novel method PhyCMAP for contact map prediction, integrating both evolutionary and physical restraints by machine learning and integer linear programming. The evolutionary restraints are much more informative than mutual information, and the physical restraints specify more concrete relationship among contacts than the sparsity restraint. As such, our method greatly reduces the solution space of the contact map matrix and, thus, significantly improves prediction accuracy. Experimental results confirm that PhyCMAP outperforms currently popular methods no matter how many sequence homologs are available for the protein under consideration. |
  | Date Added | 10/25/2013, 4:29:01 PM |
  | Modified | 11/12/2013, 4:23:03 PM |

  ### Tags:

  - ASTRAL subsets
  - Cite ASTRAL
  - Interesting

  ### Notes:

  - Present PhyCMAP method for contact map prediction, based on machine learning and linear programming.  
      
    How SCOP is used:

    Used a different tool, PDB25 from the Dunbrack lab, to compile a non-redundant data set of 601 proteins.  In order to fairly compare with CMapPro, which had been trained on Astral data, they removed all sequences with >=90% sequence identity with a sequence in the Astral 1.73 subset.

    SCOP reference:  
      
    Test data II: Set600. This set contains 601 proteins randomly extracted from PDB25 (Brenner et al., 2000) and was constructed before CASP10 started. The test proteins have the following properties: (i) they share 525% sequence identity with the training proteins; (iii) all proteins have at least 50 residues and an X-ray structure with resolution better than 1.9 A ̊ ; and (iii) all the proteins have at least five residues with pre- dicted secondary structure being alpha-helix or beta-strand.  
      
    Both the training set and Set600 are sampled from PDB25 (Wang and Dunbrack, 2003), in which any two proteins share 525% sequence identity. Sequence identity is calculated using the method in (Brenner et al., 2000).  
      
    It should be noticed that CMAPpro used Astral 1.73 (Brenner et al., 2000; Di Lena et al., 2012) as its training set, which shares 490% sequence identity with 226 proteins in Set600 (180 with Meff4100 and 46 with Meff ⬚⬚ 100). To more fairly compare the prediction methods, we exclude the 226 proteins from Set600 that share >90% sequence identity with the CMAPpro training set.

  ### Attachments

  - Bioinformatics-2013-Wang-i266-73.pdf
- ## Predicting protein residue-residue contacts using deep networks and boosting

  |  |  |
  | --- | --- |
  | Type | Journal Article |
  | Author | Jesse Eickholt |
  | Author | Jianlin Cheng |
  | Volume | 28 |
  | Issue | 23 |
  | Pages | 3066–3072 |
  | Publication | Bioinformatics |
  | Date | December 2012 |
  | DOI | 10.1093/bioinformatics/bts598 |
  | Abstract | Motivation: Protein residue-residue contacts continue to play a larger and larger role in protein tertiary structure modeling and evaluation. Yet, while the importance of contact information increases, the performance of sequence-based contact predictors has improved slowly. New approaches and methods are needed to spur further development and progress in the field. Results: Here we present DNCON, a new sequence-based residue-residue contact predictor using deep networks and boosting techniques. Making use of graphical processing units and CUDA parallel computing technology, we are able to train large boosted ensembles of residue-residue contact predictors achieving state-of-the-art performance. |
  | Date Added | 3/7/2014, 12:08:00 PM |
  | Modified | 3/7/2014, 12:08:00 PM |
- ## Predicting Protein Structural Class by Incorporating Patterns of Over-Represented k-mers into the General form of Chou's PseAAC

  |  |  |
  | --- | --- |
  | Type | Journal Article |
  | Author | Yu-Fang Qin |
  | Author | Chun-Hua Wang |
  | Author | Xiao-Qing Yu |
  | Author | Jie Zhu |
  | Author | Tai-Gang Liu |
  | Author | Xiao-Qi Zheng |
  | Volume | 19 |
  | Issue | 4 |
  | Pages | 388-397 |
  | Publication | Protein and peptide letters |
  | ISSN | 0929-8665 |
  | Date | April 2012 |
  | Language | English |
  | Abstract | Computational prediction of protein structural class based on sequence data remains a challenging problem in current protein science. In this paper, a new feature extraction approach based on relative polypeptide composition is introduced. This approach could take into account the background distribution of a given k-mer under a Markov model of order k-2, and avoid the curse of dimensionality with the increase of k by using a T-statistic feature selection strategy. The selected features are then fed to a support vector machine to perform the prediction. To verify the performance of our method, jackknife cross-validation tests are performed on four widely used benchmark datasets. Comparison of our results with existing methods shows that our method provides satisfactory performance for structural class prediction. |
  | Date Added | 10/11/2013, 10:29:15 AM |
  | Modified | 11/11/2013, 4:52:21 PM |

  ### Tags:

  - Markov model
  - protein structural class
  - relative polypeptide composition
  - support vector machine
  - T-statistic

  ### Notes:

  - Paper unavailable.
- ## Predicting the Binding Patterns of Hub Proteins: A Study Using Yeast Protein Interaction Networks

  |  |  |
  | --- | --- |
  | Type | Journal Article |
  | Author | Carson M. Andorf |
  | Author | Vasant Honavar |
  | Author | Taner Z. Sen |
  | Volume | 8 |
  | Issue | 2 |
  | Publication | Plos One |
  | ISSN | 1932-6203 |
  | Date | FEB 19 2013 |
  | Extra | WOS:000315182800036 |
  | DOI | 10.1371/journal.pone.0056833 |
  | Abstract | Background: Protein-protein interactions are critical to elucidating the role played by individual proteins in important biological pathways. Of particular interest are hub proteins that can interact with large numbers of partners and often play essential roles in cellular control. Depending on the number of binding sites, protein hubs can be classified at a structural level as singlish-interface hubs (SIH) with one or two binding sites, or multiple-interface hubs (MIH) with three or more binding sites. In terms of kinetics, hub proteins can be classified as date hubs (i.e., interact with different partners at different times or locations) or party hubs (i.e., simultaneously interact with multiple partners). Methodology: Our approach works in 3 phases: Phase I classifies if a protein is likely to bind with another protein. Phase II determines if a protein-binding (PB) protein is a hub. Phase III classifies PB proteins as singlish-interface versus multiple-interface hubs and date versus party hubs. At each stage, we use sequence-based predictors trained using several standard machine learning techniques. Conclusions: Our method is able to predict whether a protein is a protein-binding protein with an accuracy of 94% and a correlation coefficient of 0.87; identify hubs from non-hubs with 100% accuracy for 30% of the data; distinguish date hubs/party hubs with 69% accuracy and area under ROC curve of 0.68; and SIH/MIH with 89% accuracy and area under ROC curve of 0.84. Because our method is based on sequence information alone, it can be used even in settings where reliable protein-protein interaction data or structures of protein-protein complexes are unavailable to obtain useful insights into the functional and evolutionary characteristics of proteins and their interactions. Availability: We provide a web server for our three-phase approach: http://hybsvm.gdcb.iastate.edu. |
  | Date Added | 10/28/2013, 4:53:08 PM |
  | Modified | 10/28/2013, 4:53:08 PM |

  ### Notes:

  - Present method for classifying whether a protein is a hub protein, and if so, whether it is a single- or multiple-interface hub.

    How SCOP is used:

    Get domain boundaries for a data set of proteins. Use as a baseline for the number of binding interfaces.

    SCOP reference:

    We compare the results of predictors trained using machine learning methods with two baseline methods: the first baseline method classifies proteins based on the number of SCOP [47,48] and PFAM [49] domains (domain-based method) present in the sequence.

    ...

    Domain-based Method. The domain-based method builds a classifier by using a class-conditional probability distribution based on the frequency of SCOP [47,48] and PFAM [49] domains in the following manner. For each protein, the count for each type of domain was determined by the number of domains listed at the Saccharomyces Genome Database (SGD) [75]. This method was used to rule out a simple direct correlation between the number of domains and the number of interaction sites on a hub protein.

  ### Attachments

  - journal.pone.0056833.pdf
- ## Prediction of Enzymatic Activity of Proteins Based on Structural and Functional Domains

  |  |  |
  | --- | --- |
  | Type | Journal Article |
  | Author | Theodoros G. Koutsandreas |
  | Author | Eleftherios D. Pilalis |
  | Author | Aristotelis A. Chatziioannou |
  | Publication | 2013 Ieee 13th International Conference on Bioinformatics and Bioengineering (bibe) |
  | Date | 2013 |
  | Extra | WOS:000335217700033 |
  | Library Catalog | ISI Web of Knowledge |
  | Abstract | The prediction of the putative enzymatic function of uncharacterized proteins is a major problem in the field of metagenomic research, where large amounts of sequences can be rapidly determined. In this work a machine-learning approach was developed, that attempts the prediction of enzymatic activity based on three protein domain databases, PFAM, CATH and SCOP, which contain functional and structural information of proteins as Hidden Markov Models. Separate and combined classifiers were trained by well-annotated data and their performance was assessed in order to compare the predictive power of different attribute sets corresponding to the three protein domain databases. All classifiers performed well, with an average accuracy of similar to 96% and an average AUC score of 0.84. As a conclusion, the classification procedure can be integrated to more extended metagenomic analysis workflows. |
  | Date Added | 10/8/2014, 12:49:22 PM |
  | Modified | 10/8/2014, 1:57:27 PM |

  ### Tags:

  - accuracy
  - AUC score
  - biochemistry
  - biology computing
  - Biotechnology
  - CATH
  - classification procedure
  - Databases
  - enzymatic activity
  - Enzymes
  - functional domains
  - functional information
  - Genomics
  - hidden Markov models
  - learning (artificial intelligence)
  - machine-learning approach
  - metagenomic research
  - molecular biophysics
  - molecular configurations
  - pattern classification
  - PFAM
  - protein domain databases
  - Proteins
  - putative enzymatic function
  - scop
  - structural domains
  - structural information
  - Training

  ### Attachments

  - IEEE Xplore Abstract Record
  - IEEE Xplore Full Text PDF
- ## Prediction of inter domain interactions in modular polyketide synthases by docking and correlated mutation analysis

  |  |  |
  | --- | --- |
  | Type | Journal Article |
  | Author | Gitanjali Yadav |
  | Author | Swadha Anand |
  | Author | Debasisa Mohanty |
  | Volume | 31 |
  | Issue | 1 |
  | Pages | 17–29 |
  | Publication | Journal of Biomolecular Structure & Dynamics |
  | Date | January 2013 |
  | DOI | 10.1080/07391102.2012.691342 |
  | Abstract | Polyketide synthases (PKSs) are huge multi-enzymatic protein complexes involved in the biosynthesis of one of the largest families of bioactive natural products, namely polyketides. The specificity of interactions between various catalytic domains of these megasynthases is one of the pivotal factors which control the precise order in which the extender units are joined during the biosynthetic process. Hence, understanding the molecular details of proteinprotein interactions in the PKS megasynthases would be crucial for rational design of novel polyketides by domain swapping experiments involving engineered combinations of PKS catalytic domains. We have developed a computational method for exploring the binding interface between two proteins, and used it to identify the interacting residue pairs, which govern the specificity of recognition between acyl carrier protein (ACP) domain and two core catalytic domains, namely the ketosynthase (KS) and acyl transferase (AT). Both of these domain interactions i.e. the KSACP and the ATACP, are likely to play a major role in channelling of substrates and control of specificity during polyketide biosynthesis. The method, called interface scan, uses a combination of geometric docking and evolutionary information for the identification of the most appropriate mode of association between two proteins. The parameters of interface scan have been standardized based on analysis of contacts in the crystal structure of ACP in complex with ACP synthase (AcpS). Many of the contacts predicted for PKS domains are in agreement with available experiments. |
  | Date Added | 3/7/2014, 1:06:24 PM |
  | Modified | 3/7/2014, 1:06:24 PM |
- ## Prediction of metal ion-binding sites in proteins using the fragment transformation method

  |  |  |
  | --- | --- |
  | Type | Journal Article |
  | Author | Chih-Hao Lu |
  | Author | Yu-Feng Lin |
  | Author | Jau-Ji Lin |
  | Author | Chin-Sheng Yu |
  | Volume | 7 |
  | Issue | 6 |
  | Pages | e39252 |
  | Publication | PloS one |
  | ISSN | 1932-6203 |
  | Date | 2012 |
  | Extra | PMID: 22723976 |
  | Journal Abbr | PLoS ONE |
  | DOI | 10.1371/journal.pone.0039252 |
  | Library Catalog | NCBI PubMed |
  | Language | eng |
  | Abstract | The structure of a protein determines its function and its interactions with other factors. Regions of proteins that interact with ligands, substrates, and/or other proteins, tend to be conserved both in sequence and structure, and the residues involved are usually in close spatial proximity. More than 70,000 protein structures are currently found in the Protein Data Bank, and approximately one-third contain metal ions essential for function. Identifying and characterizing metal ion-binding sites experimentally is time-consuming and costly. Many computational methods have been developed to identify metal ion-binding sites, and most use only sequence information. For the work reported herein, we developed a method that uses sequence and structural information to predict the residues in metal ion-binding sites. Six types of metal ion-binding templates- those involving Ca(2+), Cu(2+), Fe(3+), Mg(2+), Mn(2+), and Zn(2+)-were constructed using the residues within 3.5 Å of the center of the metal ion. Using the fragment transformation method, we then compared known metal ion-binding sites with the templates to assess the accuracy of our method. Our method achieved an overall 94.6 % accuracy with a true positive rate of 60.5 % at a 5 % false positive rate and therefore constitutes a significant improvement in metal-binding site prediction. |
  | Date Added | 10/11/2013, 10:29:15 AM |
  | Modified | 10/11/2013, 10:29:15 AM |

  ### Tags:

  - Amino Acids
  - Binding Sites
  - Ions
  - Metalloproteins
  - Metals
  - Models, Molecular
  - Peptides
  - Protein Binding
  - Protein Conformation
  - ROC Curve

  ### Notes:

  - Present method for metal ion-binding site prediction.

    How SCOP is used:

    Remove redundancy in their data set.  Use SCOP to classify their data set by superfamily (those not classified in SCOP were just removed), then use sequence ID filtering. Used data set to evaluate their method for ion-binding site prediction.

    SCOP reference:

    Dataset containing the metal ion–binding proteins

    The proteins in the final dataset were extracted from the PDB and contain at least one Ca2+, Cu2+, Fe3+, Mg2+, Mn2+, or Zn2+ ion. At the time of our study, approximately one-fourth of all PDB entries (20094 of 77294 proteins) contained a metal ion(s). The following criteria were applied to these proteins as filters. If the structures did not contain any polypeptide chain, those structures were excluded. For proteins containing more than one polypeptide chains, we included only the chains with residues involved in metal ion–binding. The length of the polypeptide chain was required to be more than 50 residues. DNA and/or RNA components were removed, leaving only the polypeptide chain.

    To ensure that many different types of proteins were included in the dataset, proteins were grouped according to their superfamily by SCOP (version 1.67) [27]. Proteins that could not be classified by in this manner were removed. Finally, BLASTClust, in the standalone BLAST package (version 2.2.10) [28], was used to align the sequences in a pairwise fashion so that the remaining proteins could be sorted into groups that had sequence identities $ 25%. This step was performed to remove the redundant structures from the dataset because sequences with at least 25 % identity usually have similar conformations. For each cluster we retained the first entry as representative of the cluster. The final dataset is composed of 1,109 polypeptides representing 361 SCOP superfamilies.

  ### Attachments

  - journal.pone.0039252.pdf
  - PubMed entry
- ## Prediction of protein domain boundaries from inverse covariances

  |  |  |
  | --- | --- |
  | Type | Journal Article |
  | Author | Michael I. Sadowski |
  | Volume | 81 |
  | Issue | 2 |
  | Pages | 253-260 |
  | Publication | Proteins: Structure, Function, and Bioinformatics |
  | ISSN | 0887-3585 |
  | Date | FEB 2013 |
  | Extra | WOS:000313811700006 |
  | DOI | 10.1002/prot.24181 |
  | Abstract | It has been known even since relatively few structures had been solved that longer protein chains often contain multiple domains, which may fold separately and play the role of reusable functional modules found in many contexts. In many structural biology tasks, in particular structure prediction, it is of great use to be able to identify domains within the structure and analyze these regions separately. However, when using sequence data alone this task has proven exceptionally difficult, with relatively little improvement over the naive method of choosing boundaries based on size distributions of observed domains. The recent significant improvement in contact prediction provides a new source of information for domain prediction. We test several methods for using this information including a kernel smoothing-based approach and methods based on building alpha-carbon models and compare performance with a length-based predictor, a homology search method and four published sequence-based predictors: DOMCUT, DomPRO, DLP-SVM, and SCOOBY-DOmain. We show that the kernel-smoothing method is significantly better than the other ab initio predictors when both single-domain and multidomain targets are considered and is not significantly different to the homology-based method. Considering only multidomain targets the kernel-smoothing method outperforms all of the published methods except DLP-SVM. The kernel smoothing method therefore represents a potentially useful improvement to ab initio domain prediction. Proteins 2013. (C) 2012 Wiley Periodicals, Inc. |
  | Date Added | 2/20/2014, 12:24:01 PM |
  | Modified | 5/5/2014, 3:11:56 PM |

  ### Notes:

  - Present method for domain boundary prediction and evaluate on CASP data set that uses different criteria than SCOP for defining domain boundary.

    How SCOP/CATH are used:

    Do not use SCOP data.

    Benchmark on CATH data instead.

    SCOP reference:

    Since that point there have been many substantial advances in the analysis, delineation, and classification of protein domains using sequence (SMART11; PFam12) and structure (SCOP13; CATH14), with important insights into their functional promiscuity and evolu- tion15,16 as well as the folding of individual domains17 and multidomain proteins.

    ...

    As comparison measures we implemented two alterna- tive methods of domain prediction which have previously been shown to perform well: a naive predictor using only length information inspired by the DGS method21 and a homology search-based method which identified end- points of alignments to CATH domain HMMs (v. 3.2)14 and used a simple smoothing protocol to derive predic- tions.

  ### Attachments

  - 24181\_ftp.pdf
- ## Prediction of protein domain with mRMR feature selection and analysis

  |  |  |
  | --- | --- |
  | Type | Journal Article |
  | Author | Bi-Qing Li |
  | Author | Le-Le Hu |
  | Author | Lei Chen |
  | Author | Kai-Yan Feng |
  | Author | Yu-Dong Cai |
  | Author | Kuo-Chen Chou |
  | URL | http://dx.plos.org/10.1371/journal.pone.0039308 |
  | Volume | 7 |
  | Issue | 6 |
  | Pages | e39308 |
  | Publication | PloS one |
  | Date | 2012 |
  | Accessed | 9/20/2013, 1:12:54 PM |
  | Library Catalog | Google Scholar |
  | Date Added | 10/11/2013, 10:29:15 AM |
  | Modified | 3/7/2014, 12:14:24 PM |

  ### Tags:

  - Protein Conformation
  - Proteins
  - Solvents

  ### Notes:

  - Present method for protein domain prediction using machine learning.

    How SCOP/CATH is used:

    Provide background on protein domain classification.

    Negative reference?  Did evaluate their method on a non-redundant data set of 9,409 protein sequences with "clear experimental domain annotations" (unclear how the domains were collected).

    SCOP/CATH reference:

    The concreted techniques involved in the ab-initio methods are the machine learning algorithms [35,39], artificial neural networks [40], and support vector machines [41,42], along with the high quality domain databases such as CATH [43], SCOP [44] and DALI [45].

  ### Attachments

  - journal.pone.0039308.pdf
- ## Prediction of protein-protein binding free energies

  |  |  |
  | --- | --- |
  | Type | Journal Article |
  | Author | Thom Vreven |
  | Author | Howook Hwang |
  | Author | Brian G Pierce |
  | Author | Zhiping Weng |
  | Volume | 21 |
  | Issue | 3 |
  | Pages | 396-404 |
  | Publication | Protein Science |
  | ISSN | 1469-896X |
  | Date | Mar 2012 |
  | Extra | PMID: 22238219 |
  | Journal Abbr | Protein Sci. |
  | DOI | 10.1002/pro.2027 |
  | Library Catalog | NCBI PubMed |
  | Language | eng |
  | Abstract | We present an energy function for predicting binding free energies of protein-protein complexes, using the three-dimensional structures of the complex and unbound proteins as input. Our function is a linear combination of nine terms and achieves a correlation coefficient of 0.63 with experimental measurements when tested on a benchmark of 144 complexes using leave-one-out cross validation. Although we systematically tested both atomic and residue-based scoring functions, the selected function is dominated by residue-based terms. Our function is stable for subsets of the benchmark stratified by experimental pH and extent of conformational change upon complex formation, with correlation coefficients ranging from 0.61 to 0.66. |
  | Date Added | 10/11/2013, 10:29:15 AM |
  | Modified | 12/2/2013, 4:24:26 PM |

  ### Tags:

  - affinity
  - affinity
  - Algorithms
  - Antigen-Antibody Complex
  - binding
  - binding
  - computational
  - computational
  - energy function
  - energy function
  - Entropy
  - Protein Binding
  - Protein Conformation
  - protein–protein interaction
  - protein-protein interaction
  - Proteins
  - Thermodynamics

  ### Notes:

  - Present computational method for predicting protein-protein binding free energies.

    How SCOP is used:

    Train and benchmark their method on a protein-protein docking benchmark that is "non-redundant at the SCOP family level".

    SCOP reference:

    Dataset

    For training and testing our functions, we used the Affinity Benchmark that was based on our protein– protein docking benchmark and recently published by us and other groups.11,29 All the hetero-atoms were removed from the structures, so that we did not bias potentials that are parameterized for non- amino acid atom over potentials that are not. Some of the terms need hydrogen atoms present, which were added using Rosetta.22 We did not refine the structures; the positions of the non-hydrogen atoms were kept the same as in the X-ray structures. The Benchmark is non-redundant at the SCOP family level,30 and has nine cognate/noncognate pairs. Each pair consists of complexes that have similar geome- try, but very different affinity. Robust prediction algorithms should be able to predict the correct order of affinities for the cognate/noncognate pairs.

  ### Attachments

  - 2027\_ftp.pdf
  - PubMed entry
  - Snapshot
- ## Prediction of RNA binding proteins comes of age from low resolution to high resolution

  |  |  |
  | --- | --- |
  | Type | Journal Article |
  | Author | Huiying Zhao |
  | Author | Yuedong Yang |
  | Author | Yaoqi Zhou |
  | URL | http://pubs.rsc.org/en/content/articlehtml/2013/mb/c3mb70167k |
  | Publication | Mol. BioSyst. |
  | Date | 2013 |
  | Accessed | 9/23/2013, 10:15:04 AM |
  | Library Catalog | Google Scholar |
  | Date Added | 2/20/2014, 12:24:01 PM |
  | Modified | 2/20/2014, 12:24:01 PM |

  ### Notes:

  - Review compares traditional machine-learning based approaches with template-based methods for RNA-binding prediction.

    How SCOP is used:

    Get the fold classification for all RNA-binding proteins to get a count of the number of folds.

    SCOP reference:

    For example, the Structural Classification Of Proteins (SCOP)21 has 44 folds shared by both RNA and non-RNA binding proteins.22

  ### Attachments

  - C3MB70167K.pdf
- ## Predictive sequence analysis of the Candidatus Liberibacter asiaticus proteome

  |  |  |
  | --- | --- |
  | Type | Journal Article |
  | Author | Qian Cong |
  | Author | Lisa N Kinch |
  | Author | Bong-Hyun Kim |
  | Author | Nick V Grishin |
  | Volume | 7 |
  | Issue | 7 |
  | Pages | e41071 |
  | Publication | PloS one |
  | ISSN | 1932-6203 |
  | Date | 2012 |
  | Extra | PMID: 22815919 |
  | Journal Abbr | PLoS ONE |
  | DOI | 10.1371/journal.pone.0041071 |
  | Library Catalog | NCBI PubMed |
  | Language | eng |
  | Abstract | Candidatus Liberibacter asiaticus (Ca. L. asiaticus) is a parasitic gram-negative bacterium that is closely associated with Huanglongbing (HLB), a worldwide citrus disease. Given the difficulty in culturing the bacterium and thus in its experimental characterization, computational analyses of the whole Ca. L. asiaticus proteome can provide much needed insights into the mechanisms of the disease and guide the development of treatment strategies. In this study, we applied state-of-the-art sequence analysis tools to every Ca. L. asiaticus protein. Our results are available as a public website at http://prodata.swmed.edu/liberibacter\_asiaticus/. In particular, we manually curated the results to predict the subcellular localization, spatial structure and function of all Ca. L. asiaticus proteins (http://prodata.swmed.edu/liberibacter\_asiaticus/curated/). This extensive information should facilitate the study of Ca. L. asiaticus proteome function and its relationship to disease. Pilot studies based on the information from our website have revealed several potential virulence factors, discussed herein. |
  | Date Added | 10/11/2013, 10:29:15 AM |
  | Modified | 10/11/2013, 10:29:15 AM |

  ### Tags:

  - Alphaproteobacteria
  - Amino Acid Motifs
  - Amino Acid Sequence
  - Databases, Factual
  - Genome, Bacterial
  - Gram-Negative Bacteria
  - Models, Molecular
  - Molecular Conformation
  - Molecular Sequence Data
  - Pilot Projects
  - Plants
  - Protein Conformation
  - Proteome
  - Proteomics
  - Sequence Homology, Amino Acid
  - Virulence Factors

  ### Notes:

  - Computational study of sequences of Candidatus Liberibacter asiaticus proteome.  Created a database integrating their sequence data and data and results from various other databases and methods.

    How SCOP is used:

    To detect homologs to proteins to the studied sequences, used HHsearch and search SCOP among other databases.

    Also augment their database with SCOP sccs classification data when available.

    SCOP reference:

    Fourth, to detect evolutionarily related protein structures and reveal domain architectures, we used three protocols: 1) PSI- BLAST (e-value cutoff 0.005) against the NR database (05/22/ 2011), starting from the sequence profiles built by the buildali.pl script in the HHsearch package, 2) RPS-BLAST (e-value cutoff 0.005) and 3) HHsearch (probability cutoff 90%) against the 70% sequence identity representatives of all PDB entries (up to Jun, 2011), the Structure Classification of Proteins (SCOP, version 1.75) database [37] and the Molecular Modeling DataBase (MMDB, up to Jan, 2011) from NCBI [38], with each single protein sequence as a query.

    ...

    Description of the Website

    ...

    Section V. Homologous structures and domains (illustrated in Fig. 1E). Homology modeling remains the most reliable and effective way to predict protein 3D structure [43,44]. This section is designed for structure modeling. Homologous structures and structure domains detected by PSI-BLAST (e-value cutoff 0.005), RPS-BLAST (e-value cutoff 0.005) and HHsearch (probability cutoff 90.0%) are presented in similar format as described in Section III. For each hit, the alignment and the corresponding structure displayed by Jmol (an open-source Java viewer for chemical structures in 3D, http://www.jmol.org/) can be easily retrieved. These protein structures can be used as templates to generate a 3D structural model. For structure domains detected in SCOP, we provide their classification hierarchy, which places them in an evolutionary context and suggests similarities to other proteins.

  ### Attachments

  - [HTML] from plos.org
  - journal.pone.0041071.pdf
  - PubMed entry
- ## Prescont: Predicting protein-protein interfaces utilizing four residue properties

  |  |  |
  | --- | --- |
  | Type | Journal Article |
  | Author | Hermann Zellner |
  | Author | Martin Staudigel |
  | Author | Thomas Trenner |
  | Author | Meik Bittkowski |
  | Author | Vincent Wolowski |
  | Author | Christian Icking |
  | Author | Rainer Merkl |
  | Volume | 80 |
  | Issue | 1 |
  | Pages | 154-168 |
  | Publication | Proteins-Structure Function and Bioinformatics |
  | ISSN | 0887-3585 |
  | Date | JAN 2012 |
  | Extra | WOS:000298598800013 |
  | DOI | 10.1002/prot.23172 |
  | Abstract | An important task of computational biology is to identify those parts of a polypeptide chain, which are involved in interactions with other proteins. For this purpose, we have developed the program PresCont, which predicts in a robust manner amino acids that constitute protein-protein interfaces (PPIs). PresCont reaches state-of-the-art classification quality on the basis of only four residue properties that can be readily deduced from the 3D structure of an individual protein and a multiple sequence alignment (MSA) composed of homologs. The core of PresCont is a support vector machine, which assesses solvent-accessible surface area, hydrophobicity, conservation, and the local environment of each amino acid on the protein surface. For training and performance testing, we compiled three nonoverlapping datasets consisting of permanently formed or transient complexes, respectively. A comparison with SPPIDER, ProMate, and meta-PPISP showed that PresCont compares favorably with these highly sophisticated programs, and that its prediction quality is less dependent on the type of protein complex being considered. This balance is due to a mutual compensation of classification weaknesses observed for individual properties: For PPIs of permanent complexes, solvent-accessible surface and hydrophobicity contribute most to classification quality, for PPIs of transient complexes, the assessment of the local environment is most significant. Moreover, we show that for permanent complexes a segmentation of PPIs into core and rim residues has only a moderate influence on prediction quality. PresCont is available as a web service at . Proteins 2012; (C) 2011 Wiley Periodicals, Inc. |
  | Date Added | 2/20/2014, 12:24:01 PM |
  | Modified | 2/20/2014, 12:24:01 PM |

  ### Notes:

  - Present PresCont method for predicting protein-protein interfaces.  Evaluate on a non-redundant data set of homo and hetero-dimers.

    How SCOP is used:

    Evaluate method on non-redundant data set.

    Compiled their own dataset from the PDB, and then used SCOP classification to help avoid 'spatial redundancy'.

    I assume that means removing extra copies of proteins with the same fold.

    SCOP reference:

    MATERIALS AND METHODS

    PlaneDimers and Dimers: Redundancy free sets of dimeric proteins

    A comprehensive, redundancy-free set of homo- and heterodimers, whose structures have been deposited in the PDB database has been compiled by the group of R. Nussinov.28 The authors have utilized PQS29 and the SCOP classification of proteins30 to eliminate multiple copies of the same complex and to avoid spatial redun- dancy.28 The resulting dataset consists of 2582 clusters, each of which contains one or more instances of a spe- cific dimeric complex. To compile a representative set of globular proteins, we removed membrane proteins, anti- gen-antibody complexes, and virus capsids. For each of the remaining clusters we selected as a typical example that complex being on sequence level most similar to all other ones. We named this dataset Dimers and used it to deduce scores for the occurrence of intramolecular resi- due-pairs, see Eq. (9). This dataset as well as all other ones introduced below can be downloaded from our webserver http://www-bioinf.uni-regensburg.de/.

  ### Attachments

  - 23172\_ftp.pdf
- ## Probing the protein space for extending the detection of weak homology folds

  |  |  |
  | --- | --- |
  | Type | Journal Article |
  | Author | Danilo Gullotto |
  | Author | Mario Salvatore Nolassi |
  | Author | Andrea Bernini |
  | Author | Ottavia Spiga |
  | Author | Neri Niccolai |
  | URL | http://www.sciencedirect.com/science/article/pii/S0022519312006297 |
  | Publication | Journal of theoretical biology |
  | Date | 2012 |
  | Accessed | 9/23/2013, 10:16:36 AM |
  | Library Catalog | Google Scholar |
  | Date Added | 10/11/2013, 10:29:15 AM |
  | Modified | 3/7/2014, 12:09:42 PM |

  ### Tags:

  - protein fold
  - Protein motif
  - Remote homology
  - Structural bioinformatics
  - structure prediction

  ### Notes:

  - Present an algorithm for protein structure prediction, "Building Block Structure Predictor (BBSP)", a hybrid divide-and-conquer algorithm based on a number of other structure prediction techniques, including template-based detection of "domain building motifs".

    How SCOP/CATH is used:

    Construct a protein motif library using a nonredundant data set of sequences from PDB for homology modeling.  SCOP and CATH were used to verify that "several common SCOP superfamilies" were represented.

    SCOP Referenc:

    2.1. Construction of protein motif library

    ...

    The motif library has been constructed on the basis of a non- redundant set (sequence identity lower than 30%) of 1104 proteins taken from the PDB. Thus, protein domains belonging to several common Structural Classification of Proteins (SCOP) superfamilies (Murzin et al., 1995) and Class Architecture Topol- ogy Homologous superfamily (CATH) superfolds (Orengo et al., 1997) have been included and 7960 sub-structures, ranging from 3 to 145 mers, are currently present in the regularly updated database.

  ### Attachments

  - 1-s2.0-S0022519312006297-main.pdf
- ## ProBiS-database: precalculated binding site similarities and local pairwise alignments of PDB structures

  |  |  |
  | --- | --- |
  | Type | Journal Article |
  | Author | Janez Konc |
  | Author | Tomo Cesnik |
  | Author | Joanna Trykowska Konc |
  | Author | Matej Penca |
  | Author | Dušanka Janežič |
  | Volume | 52 |
  | Issue | 2 |
  | Pages | 604-612 |
  | Publication | Journal of chemical information and modeling |
  | ISSN | 1549-960X |
  | Date | Feb 27, 2012 |
  | Extra | PMID: 22268964 |
  | Journal Abbr | J Chem Inf Model |
  | DOI | 10.1021/ci2005687 |
  | Library Catalog | NCBI PubMed |
  | Language | eng |
  | Abstract | ProBiS-Database is a searchable repository of precalculated local structural alignments in proteins detected by the ProBiS algorithm in the Protein Data Bank. Identification of functionally important binding regions of the protein is facilitated by structural similarity scores mapped to the query protein structure. PDB structures that have been aligned with a query protein may be rapidly retrieved from the ProBiS-Database, which is thus able to generate hypotheses concerning the roles of uncharacterized proteins. Presented with uncharacterized protein structure, ProBiS-Database can discern relationships between such a query protein and other better known proteins in the PDB. Fast access and a user-friendly graphical interface promote easy exploration of this database of over 420 million local structural alignments. The ProBiS-Database is updated weekly and is freely available online at http://probis.cmm.ki.si/database. |
  | Short Title | ProBiS-database |
  | Date Added | 10/11/2013, 10:29:15 AM |
  | Modified | 10/11/2013, 10:29:15 AM |

  ### Tags:

  - Algorithms
  - Binding Sites
  - Databases, Protein
  - Interesting
  - Internet
  - Protein Conformation
  - Proteins
  - Structural Homology, Protein

  ### Notes:

  - Present ProBiS-Database, which provides precalculated binding site similarities from local pairwise alignments of PDB structures.

    How SCOP is used:

    Refer to the SCOP fold classification in a use case example, to show how two proteins with low sequence similarity and different folds may have similar binding sites.

    SCOP reference:

    (SCOP paper is reference 23.)

    Example 2: Local Pairwise Alignments of PDB Structures.

    An interactive table of similar proteins appears on the right side of Figure 2. Each of these similar proteins may have many different local pairwise alignments with the query protein; they are ranked by the Z-Score of their highest scoring local pairwise alignment. Similar proteins marked with a red star are “Hot”, which means they are of a different protein family according to the Protein Family (Pfam) classification system than the query protein.22 In the Local Structural Similarity Profile page for cytochrome c in Figure 2, there are 61 “hot” similar proteins; **many of these have a fold different from that of the query protein (cytochrome c fold)**.23 Among similar proteins are various differently folded proteins, e.g., multiheme cytochrome, cytochrome f, etc. It should be noted that these proteins have no backbone or sequence similarities and thus will not be detected by structural alignment algorithms, which compare protein backbones or secondary structure elements.6 In the majority of these differently folded proteins, the detected pairwise alignments correspond to amino acids in the heme binding sites of these proteins, and below we present one such example.

  ### Attachments

  - ci2005687.pdf
  - [HTML] from acs.org
  - PubMed entry
- ## ProCoCoA: A quantitative approach for analyzing protein core composition

  |  |  |
  | --- | --- |
  | Type | Journal Article |
  | Author | Silvia Bottini |
  | Author | Andrea Bernini |
  | Author | Matteo De Chiara |
  | Author | Diego Garlaschelli |
  | Author | Ottavia Spiga |
  | Author | Marco Dioguardi |
  | Author | Elisa Vannuccini |
  | Author | Anna Tramontano |
  | Author | Neri Niccolai |
  | Volume | 43 |
  | Pages | 29-34 |
  | Publication | Computational Biology and Chemistry |
  | ISSN | 1476-9271 |
  | Date | APR 2013 |
  | Extra | WOS:000319493000005 |
  | DOI | 10.1016/j.compbiolchem.2012.12.007 |
  | Abstract | Defining the amino acid composition of protein cores is fundamental for understanding protein folding, as different architectures might achieve structural stability only in the presence of specific amino acid networks. Quantitative characterization of protein cores in relation to the corresponding structures and dynamics is needed to increase the reliability of protein engineering procedures. Unambiguous criteria based on atom depth considerations were established to assign amino acid residues to protein cores and, hence, for classifying inner and outer molecular moieties. These criteria were summarized in a new tool named ProCoCoA, Protein Core Composition Analyzer. An user-friendly web interface was developed, available at the URL: http://www.sbl.unisi.it/prococoa. An accurate estimate of protein core composition for six protein architectures selected from the CATH database of solved structures has been carried out, and the obtained results indicate the presence of specific patterns of amino acid core composition in different protein folds. (C) 2012 Elsevier Ltd. All rights reserved. |
  | Date Added | 2/13/2014, 4:13:17 PM |
  | Modified | 3/7/2014, 12:10:15 PM |
- ## ProDis-ContSHC: learning protein dissimilarity measures and hierarchical context coherently for protein-protein comparison in protein database retrieval

  |  |  |
  | --- | --- |
  | Type | Journal Article |
  | Author | Jingyan Wang |
  | Author | Xin Gao |
  | Author | Quanquan Wang |
  | Author | Yongping Li |
  | URL | http://www.biomedcentral.com/1471-2105/13/S7/S2/ |
  | Volume | 13 |
  | Issue | Suppl 7 |
  | Pages | S2 |
  | Publication | BMC bioinformatics |
  | Date | 2012 |
  | Accessed | 9/20/2013, 1:19:35 PM |
  | Library Catalog | Google Scholar |
  | Short Title | ProDis-ContSHC |
  | Date Added | 10/11/2013, 10:29:15 AM |
  | Modified | 3/7/2014, 1:06:54 PM |

  ### Tags:

  - ASTRAL
  - ASTRAL domain structures
  - ASTRAL sequences
  - ASTRAL subsets

  ### Notes:

  - Propose a novel protein-protein dissimilarity learning algorithm, ProDis-ContSHC.

    How SCOP is used:

    Benchmarked method on ASTRAL 1.73 filtered at 95% sequence similariy.  Validated fold predictions against the SCOP classification.

    SCOP reference:

    In Abstract:

    We test the performance of ProDis-ContSHC on two benchmark sets, i.e., the ASTRAL 1.73 database and the FSSP/ DALI database. Experimental results demonstrate that plugging our supervised contextual dissimilarity measures into the retrieval systems significantly outperforms the context-free dissimilarity/similarity measures and other unsupervised contextual dissimilarity measures that do not use the class label information.

    Benchmark sets

    To evaluate the proposed ProDis-ContSHC algorithm, we conduct experiments on two different benchmark sets, i.e., the ones used in [21] and [26] respectively. ASTRAL 1.73 protein domain dataset

    Following [26], we use the following database and queries as our first benchmark set:  
     Database The ASTRAL 1.73 [48] 95% sequence-identity non-redundant data set is used as the protein database. We generate our index database from the tableau data set published by Stivala et al. [49], which contains 15,169 entries.

    Results and discussion

    Results on ASTRAL 1.73 dataset

    To compare a query protein x0 to a protein xi in the ASTRAL 1.73 dataset, we compute the cosine similarity [27] as the baseline similarity measure as in [26]. Cosine similarity [27] simply calculates the cosine of the angle between the two vectors xi and xj.

    ...

    ROC curve and precision-recall curve performance

    SCOP [53] fold classification is used as the ground truth to evaluate the performance of the different methods. To fairly compare the accuracy, we use the receiver operating characteristic (ROC) curve [54], the area under this ROC curve (AUC) [54], and the precision-recall curve [55]. Given a query protein x0 which belongs to the SCOP fold l0, the top k proteins returned by the search algorithms are considered as the hits. The remaining proteins are consid- ered as the misses. For the i-th protein xi belonging to the SCOPfoldli,ifli =l0 andi≤k,theproteinxi isdefinedas a true positive (TP). On the other hand, if li ≠ l0 and i ≤ k, xi isdefinedasafalsepositive(FP).Ifli ≠l0 andi>k,xi is defined as a true negative (TN). Otherwise, xi is a false negative (FN). Using these definitions, we can then com- pute the true positive rate (TPR or recall), the false posi- tive rate (FPR), recall and precision as follows:

  ### Attachments

  - 1471-2105-13-S7-S2.pdf
  - [HTML] from biomedcentral.com
  - PubMed entry
- ## Progress in computational studies of host-pathogen interactions

  |  |  |
  | --- | --- |
  | Type | Journal Article |
  | Author | Hufeng Zhou |
  | Author | Jingjing Jin |
  | Author | Limsoon Wong |
  | Volume | 11 |
  | Issue | 2 |
  | Pages | 1230001 |
  | Publication | Journal of Bioinformatics and Computational Biology |
  | ISSN | 0219-7200 |
  | Date | APR 2013 |
  | Extra | WOS:000321005100002 |
  | DOI | 10.1142/S0219720012300018 |
  | Abstract | Host-pathogen interactions are important for understanding infection mechanism and developing better treatment and prevention of infectious diseases. Many computational studies on host-pathogen interactions have been published. Here, we review recent progress and results in this field and provide a systematic summary, comparison and discussion of computational studies on host-pathogen interactions, including prediction and analysis of host-pathogen protein-protein interactions; basic principles revealed from host-pathogen interactions; and database and software tools for host-pathogen interaction data collection, integration and analysis. |
  | Date Added | 2/20/2014, 12:24:01 PM |
  | Modified | 12/15/2014, 12:21:39 PM |

  ### Notes:

  - Review of computational studies of host-pathogen interactions.

    How SCOP is used:

    Not using SCOP data.  Reference another work that used annotated non-SCOP data set with SCOP data (superfamily).

    SCOP reference:

    2.2.1. Comparative modeling

    Prediction by comparative modeling is a representative structure-based approach. For example, in Davis et al.,6 an automated pipeline for large-scale comparative protein structure modeling, MODPIPE, is applied to model the structure of host and pathogen proteins based on their sequences and corresponding template struc- tures. Given the computed model of a protein, the SCOP34 superfamilies that the protein belongs to are identi ̄ed. A database of protein structural interfaces, PIBASE, is then scanned. If a SCOP superfamily of a host protein and a SCOP superfamily of a pathogen protein are both involved in the same PIBASE35 protein structural interface, then the host protein and the pathogen protein are predicted as a putative PPI.

  ### Attachments

  - s0219720012300018.pdf
- ## PROMALS3D: a tool for multiple protein sequence and structure alignments

  |  |  |
  | --- | --- |
  | Type | Journal Article |
  | Author | Jimin Pei |
  | Author | Bong-Hyun Kim |
  | Author | Nick V Grishin |
  | Volume | 36 |
  | Issue | 7 |
  | Pages | 2295-2300 |
  | Publication | Nucleic acids research |
  | ISSN | 1362-4962 |
  | Date | Apr 2008 |
  | Extra | PMID: 18287115 |
  | Journal Abbr | Nucleic Acids Res. |
  | DOI | 10.1093/nar/gkn072 |
  | Library Catalog | NCBI PubMed |
  | Language | eng |
  | Abstract | Although multiple sequence alignments (MSAs) are essential for a wide range of applications from structure modeling to prediction of functional sites, construction of accurate MSAs for distantly related proteins remains a largely unsolved problem. The rapidly increasing database of spatial structures is a valuable source to improve alignment quality. We explore the use of 3D structural information to guide sequence alignments constructed by our MSA program PROMALS. The resulting tool, PROMALS3D, automatically identifies homologs with known 3D structures for the input sequences, derives structural constraints through structure-based alignments and combines them with sequence constraints to construct consistency-based multiple sequence alignments. The output is a consensus alignment that brings together sequence and structural information about input proteins and their homologs. PROMALS3D can also align sequences of multiple input structures, with the output representing a multiple structure-based alignment refined in combination with sequence constraints. The advantage of PROMALS3D is that it gives researchers an easy way to produce high-quality alignments consistent with both sequences and structures of proteins. PROMALS3D outperforms a number of existing methods for constructing multiple sequence or structural alignments using both reference-dependent and reference-independent evaluation methods. |
  | Short Title | PROMALS3D |
  | Date Added | 10/11/2013, 10:29:15 AM |
  | Modified | 10/11/2013, 10:29:15 AM |

  ### Tags:

  - ASTRAL
  - ASTRAL domain structures
  - ASTRAL sequences
  - ASTRAL subsets

  ### Notes:

  - Present a tool for multiple sequence and structure alignment: PROMALS3D.

    How SCOP is used:

    1. As part of their workflow: SCOP data is used as part of their workflow, in the step to detect homologs with 3D structure.  Used ASTRAL 1.69 40% representative set.  Used sequence and structure data.

    2. To benchmark their method.  Used the SABmark database, which derives a benchmarking dataset from SCOP 1.65.

  ### Attachments

  - Nucl. Acids Res.-2008-Pei-2295-300.pdf
  - PubMed entry
- ## Promiscuous domains: facilitating stability of the yeast protein-protein interaction network

  |  |  |
  | --- | --- |
  | Type | Journal Article |
  | Author | Erli Pang |
  | Author | Tao Tan |
  | Author | Kui Lin |
  | Volume | 8 |
  | Issue | 3 |
  | Pages | 766-771 |
  | Publication | Molecular Biosystems |
  | ISSN | 1742-206X |
  | Date | 2012 |
  | Extra | WOS:000300048500010 |
  | DOI | 10.1039/c1mb05364g |
  | Abstract | Domain-domain interactions are a critical type of the mechanisms mediating protein-protein interactions (PPIs). For a given protein domain, its ability to combine with distinct domains is usually referred to as promiscuity or versatility. Interestingly, a previous study has reported that a domain's promiscuity may reflect its ability to interact with other domains in human proteins. In this work, promiscuous domains were first identified from the yeast genome. Then, we sought to determine what roles promiscuous domains might play in the PPI network. Mapping the promiscuous domains onto the proteins in this network revealed that, consistent with the previous knowledge, the hub proteins were significantly enriched with promiscuous domains. We also found that the set of hub proteins were not the same set as those proteins with promiscuous domains, although there was some overlap. Analysis of the topological properties of this yeast PPI network showed that the characteristic path length of the network increased significantly after deleting proteins with promiscuous domains. This indicated that communication between two proteins was longer and the network stability decreased. These observations suggested that, as the hub proteins, proteins with promiscuous domains might play a role in maintaining network stability. In addition, functional analysis revealed that proteins with promiscuous domains mainly participated in the "Folding, Sorting, and Degradation" and "Replication and Repair" biological pathways, and that they significantly execute key molecular functions, such as "nucleoside-triphosphatase activity (GO: 0017111)." |
  | Date Added | 2/20/2014, 12:24:01 PM |
  | Modified | 3/7/2014, 12:14:08 PM |

  ### Notes:

  - Computational study of promiscuous domains in yeast PPIs.

    How SCOP/CATH is used:

    Background on protein structure classification.

    SCOP/CATH reference:

    Proteins in general consist of domains which are evolution- ary units,14–16 and which are usually classified into structure- based domains, such as SCOP17 and CATH,18 and sequence-based domains, such as Pfam.15

  ### Attachments

  - c1mb05364g.pdf
- ## Protein Conformational Diversity Modulates Sequence Divergence

  |  |  |
  | --- | --- |
  | Type | Journal Article |
  | Author | Ezequiel Juritz |
  | Author | Nicolas Palopoli |
  | Author | Maria Silvina Fornasari |
  | Author | Sebastian Fernandez-Alberti |
  | Author | Gustavo Parisi |
  | Volume | 30 |
  | Issue | 1 |
  | Pages | 79–87 |
  | Publication | Molecular Biology and Evolution |
  | Date | January 2013 |
  | DOI | 10.1093/molbev/mss080 |
  | Abstract | It is well established that the conservation of protein structure during evolution constrains sequence divergence. The conservation of certain physicochemical environments to preserve protein folds and then the biological function originates a site-specific structurally constrained substitution pattern. However, protein native structure is not unique. It is known that the native state is better described by an ensemble of conformers in a dynamic equilibrium. In this work, we studied the influence of conformational diversity in sequence divergence and protein evolution. For this purpose, we derived a set of 900 proteins with different degrees of conformational diversity from the PCDB database, a conformer database. With the aid of a structurally constrained protein evolutionary model, we explored the influence of the different conformations on sequence divergence. We found that the presence of conformational diversity strongly modulates the substitution pattern. Although the conformers share several of the structurally constrained sites, 30% of them are conformer specific. Also, we found that in 76% of the proteins studied, a single conformer outperforms the others in the prediction of sequence divergence. It is interesting to note that this conformer is usually the one that binds ligands participating in the biological function of the protein. The existence of a conformer-specific site-substitution pattern indicates that conformational diversity could play a central role in modulating protein evolution. Furthermore, our findings suggest that new evolutionary models and bioinformatics tools should be developed taking into account this substitution bias. |
  | Date Added | 3/7/2014, 12:08:00 PM |
  | Modified | 3/7/2014, 12:08:00 PM |
- ## Protein design by fusion: implications for protein structure prediction and evolution

  |  |  |
  | --- | --- |
  | Type | Journal Article |
  | Author | Katarzyna Skorupka |
  | Author | Seong Kyu Han |
  | Author | Hyun-Jun Nam |
  | Author | Sanguk Kim |
  | Author | Salem Faham |
  | Volume | 69 |
  | Pages | 2451-2460 |
  | Publication | Acta Crystallographica Section D-Biological Crystallography |
  | ISSN | 0907-4449; 1399-0047 |
  | Date | DEC 2013 |
  | Extra | WOS:000328370400018 |
  | DOI | 10.1107/S0907444913022701 |
  | Abstract | Domain fusion is a useful tool in protein design. Here, the structure of a fusion of the heterodimeric flagella-assembly proteins FliS and FliC is reported. Although the ability of the fusion protein to maintain the structure of the heterodimer may be apparent, threading-based structural predictions do not properly fuse the heterodimer. Additional examples of naturally occurring heterodimers that are homologous to full-length proteins were identified. These examples highlight that the designed protein was engineered by the same tools as used in the natural evolution of proteins and that heterodimeric structures contain a wealth of information, currently unused, that can improve structural predictions. |
  | Date Added | 2/12/2014, 2:18:08 PM |
  | Modified | 3/7/2014, 12:08:49 PM |

  ### Notes:

  - Experimental and computational study of domain fusion for protein design.  Report on the structure of a fusion of two heterodimers: FliS and FliC.

    How SCOP/CATH is used:

    Used PDBefold to search SCOP and the entire PDB for proteins of similar structure.  Also used CATH directly (because CATH website supports searches by PDB structure) to search for similar structures.

    SCOP/CATH reference:

    3.2. Homology search

    We evaluated the final structure using a number of struc- tural databases and programs, including CATH (Greene et al., 2007), VAST (Gibrat et al., 1996), DALI (Holm & Rosen- stro ̈m, 2010) and PDBeFold (Krissinel & Henrick, 2004) tested both against SCOP categories (Murzin et al., 1995) as well as all PDB entries.

    ...

    CATH also identified the C chain of cytochrome c oxidase as the closest structural homolog.

  ### Attachments

  - yt5059.pdf
- ## Protein docking using case-based reasoning

  |  |  |
  | --- | --- |
  | Type | Journal Article |
  | Author | Anisah W. Ghoorah |
  | Author | Marie-Dominique Devignes |
  | Author | Malika Smail-Tabbone |
  | Author | David W. Ritchie |
  | Volume | 81 |
  | Issue | 12 |
  | Pages | 2150-2158 |
  | Publication | Proteins-Structure Function and Bioinformatics |
  | ISSN | 0887-3585; 1097-0134 |
  | Date | DEC 2013 |
  | Extra | WOS:000327344300010 |
  | DOI | 10.1002/prot.24433 |
  | Abstract | Protein docking algorithms aim to calculate the three-dimensional (3D) structure of a protein complex starting from its unbound components. Although ab initio docking algorithms are improving, there is a growing need to use homology modeling techniques to exploit the rapidly increasing volumes of structural information that now exist. However, most current homology modeling approaches involve finding a pair of complete single-chain structures in a homologous protein complex to use as a 3D template, despite the fact that protein complexes are often formed from one or more domain-domain interactions (DDIs). To model 3D protein complexes by domain-domain homology, we have developed a case-based reasoning approach called KBDOCK which systematically identifies and reuses domain family binding sites from our database of nonredundant DDIs. When tested on 54 protein complexes from the Protein Docking Benchmark, our approach provides a near-perfect way to model single-domain protein complexes when full-homology templates are available, and it extends our ability to model more difficult cases when only partial or incomplete templates exist. These promising early results highlight the need for a new and diverse docking benchmark set, specifically designed to assess homology docking approaches. Proteins 2013; 81:2150-2158. (c) 2013 Wiley Periodicals, Inc. |
  | Date Added | 2/12/2014, 1:36:22 PM |
  | Modified | 2/12/2014, 1:36:22 PM |

  ### Notes:

  - Present protein docking method.

    How SCOP is used:

    Background on protein structure classification.

    SCOP reference:

    Because protein domains may often be identified as structural and functional units, the 3D structures of pro- tein complexes are often analyzed in terms of their com- ponent domain–domain interactions (DDIs). In recent years, several protein structure interaction databases have been described.8 Some of these collect interactions between protein chains,9–13 whereas others14–19 anno- tate interactions using the Pfam,20 SCOP,21 or CDD22 domain classifications.

  ### Attachments

  - prot24433.pdf
- ## Protein domain definition should allow for conditional disorder

  |  |  |
  | --- | --- |
  | Type | Journal Article |
  | Author | Kavestri Yegambaram |
  | Author | Esther M. M. Bulloch |
  | Author | Richard L. Kingston |
  | Volume | 22 |
  | Issue | 11 |
  | Pages | 1502-1518 |
  | Publication | Protein Science |
  | ISSN | 0961-8368; 1469-896X |
  | Date | NOV 2013 |
  | Extra | WOS:000326025100005 |
  | DOI | 10.1002/pro.2336 |
  | Abstract | Proteins are often classified in a binary fashion as either structured or disordered. However this approach has several deficits. Firstly, protein folding is always conditional on the physiochemical environment. A protein which is structured in some circumstances will be disordered in others. Secondly, it hides a fundamental asymmetry in behavior. While all structured proteins can be unfolded through a change in environment, not all disordered proteins have the capacity for folding. Failure to accommodate these complexities confuses the definition of both protein structural domains and intrinsically disordered regions. We illustrate these points with an experimental study of a family of small binding domains, drawn from the RNA polymerase of mumps virus and its closest relatives. Assessed at face value the domains fall on a structural continuum, with folded, partially folded, and near unstructured members. Yet the disorder present in the family is conditional, and these closely related polypeptides can access the same folded state under appropriate conditions. Any heuristic definition of the protein domain emphasizing conformational stability divides this domain family in two, in a way that makes no biological sense. Structural domains would be better defined by their ability to adopt a specific tertiary structure: a structure that may or may not be realized, dependent on the circumstances. This explicitly allows for the conditional nature of protein folding, and more clearly demarcates structural domains from intrinsically disordered regions that may function without folding. PDB Code(s): 4KYC 4KYD 4KYE |
  | Date Added | 2/12/2014, 1:36:22 PM |
  | Modified | 3/7/2014, 12:08:33 PM |

  ### Notes:

  - Experimental and computational study of intrinsically disordered domains.

    How SCOP/CATH is used:

    Background on protein structure classification.

    SCOP/CATH reference:

    Because of the repeated occurrence of similar domains in differing structural contexts, and their linkage with function, domain identification and classification underpins protein taxonomy and functional annotation.5–8

  ### Attachments

  - pro2336.pdf
- ## Protein domain recurrence and order can enhance prediction of protein functions

  |  |  |
  | --- | --- |
  | Type | Journal Article |
  | Author | Mario Abdel Messih |
  | Author | Meghana Chitale |
  | Author | Vladimir B. Bajic |
  | Author | Daisuke Kihara |
  | Author | Xin Gao |
  | Volume | 28 |
  | Issue | 18 |
  | Pages | Swiss Inst Bioinformat (SIB) |
  | Publication | Bioinformatics |
  | Date | September 2012 |
  | DOI | 10.1093/bioinformatics/bts398 |
  | Abstract | Motivation: Burgeoning sequencing technologies have generated massive amounts of genomic and proteomic data. Annotating the functions of proteins identified in this data has become a big and crucial problem. Various computational methods have been developed to infer the protein functions based on either the sequences or domains of proteins. The existing methods, however, ignore the recurrence and the order of the protein domains in this function inference. Results: We developed two new methods to infer protein functions based on protein domain recurrence and domain order. Our first method, DRDO, calculates the posterior probability of the Gene Ontology terms based on domain recurrence and domain order information, whereas our second method, DRDO-NB, relies on the naive Bayes methodology using the same domain architecture information. Our large-scale benchmark comparisons show strong improvements in the accuracy of the protein function inference achieved by our new methods, demonstrating that domain recurrence and order can provide important information for inference of protein functions. |
  | Date Added | 3/7/2014, 12:08:00 PM |
  | Modified | 3/7/2014, 12:08:00 PM |
- ## Protein domain structure uncovers the origin of aerobic metabolism and the rise of planetary oxygen

  |  |  |
  | --- | --- |
  | Type | Journal Article |
  | Author | Kyung Mo Kim |
  | Author | Tao Qin |
  | Author | Ying-Ying Jiang |
  | Author | Ling-Ling Chen |
  | Author | Min Xiong |
  | Author | Derek Caetano-Anollés |
  | Author | Hong-Yu Zhang |
  | Author | Gustavo Caetano-Anollés |
  | URL | http://www.sciencedirect.com/science/article/pii/S0969212611004163 |
  | Volume | 20 |
  | Issue | 1 |
  | Pages | 67–76 |
  | Publication | Structure |
  | Date | 2012 |
  | Accessed | 9/20/2013, 1:19:04 PM |
  | Library Catalog | Google Scholar |
  | Date Added | 10/11/2013, 10:29:15 AM |
  | Modified | 10/11/2013, 10:29:15 AM |

  ### Tags:

  - Interesting

  ### Notes:

  - Use a structural census in nearly 1,000 genomes and a molecular clock of SCOP folds and families to define a timeline of appearance of protein families linked to single-domain enzymes.

    Calculate 'geological ages' of domains at the family and superfamily level.

     How SCOP is used:

    Create a dataset derived from SCOP of single-domain enzymes with "unambigous enzymatic activities" defined using EC classification.

    Use SCOP family classification is to calculate ages and define a timeline.

    SCOP reference:

    INTRODUCTION

    We calculated the evolutionary age of protein domain structures at FF (Caetano-Anolle ́s et al., 2012, 2011), FSF (Wang et al., 2007), and F (Caetano-Anolle ́s and Caetano- Anolle ́ s, 2003) levels from intrinsically rooted phylogenies reconstructed from a census of domain structures in hundreds of genomes that have been completely sequenced.

    EXPERIMENTAL PROCEDURES

    Phylogenomic Methods

    A timeline of domain discovery was derived from a universal phylogenomic tree of FF domain structure. Protein structural domains corresponding to 3,513 FFs (out of 3,902 defined by SCOP 1.75) were assigned to proteomes of 989 organisms whose genomes were completely sequenced (76 Archaea, 656 Bacteria, and 257 Eukarya). This structural genomic census used the iterative Sequence Alignment and Modeling System (SAM) method to scan genomic sequences (with probability cutoffs E of 104) against a library of advanced linear hidden Markov models (HMMs) of structural recognition in SUPERFAMILY (Gough et al., 2001). The census produced a data matrix with columns representing proteomes (phylogenetic characters) and rows representing FFs (phylogenetic taxa). This matrix was used to build a phylo- genetic tree of FF domain structure using the maximum parsimony (MP) method in PAUP\* version 4.0b10 (Swofford, 2002) and a combined parsimony ratchet (PR) and iterative search approach (Wang and Caetano-Anolle ́ s, 2009) to facilitate tree reconstruction and avoid the risk for optimal trees being trapped in suboptimal regions of tree space. A single MP reconstruction was retained following 300 ratchet iterations (10 3 30 chains) with 1,000 replicates of random taxon addition, tree bisection reconnection (TBR) branch swapping, and maxtrees unrestricted. Concise classification strings (ccs) defined SCOP domains at FF level (e.g., c.37.1.12, where c represents the protein class, 37 the F, 1 the FSF, and 12 the FF) and were used to identify taxa in trees. Finally, the relative age of protein architectures (nd) was calculated directly from the phylogenomic tree using a PERL script that counts the number of nodes from the ancestral architecture at the base of the tree to each leaf and provides it in a relative zero to one scale. A recent review summarizes the general approach and the progression of census data and tree reconstruction in recent years (Caetano-Anolle ́ s et al., 2009a). In addition the phylogenomic approach based on a genomic census is robust against uneven sampling of genomes across the three superkingdoms (Kim and Caetano-Anolle ́ s, 2011).

    Identification of Single-Domain Enzyme FFs

    The 3,902 FFs defined by SCOP 1.75 (February 2009) cover 38,221 PDB entries. These proteins include 19,038 enzymes harboring 1,421 enzyme activities defined at 4 levels of EC classification (February 2011). Out of these enzymes, 4,138 consist of single-domain proteins corresponding to 416 FFs and 711 EC 4-level activities. To guarantee a tight link between FF and enzyme function, FFs with unambiguous enzymatic activities were identified by select- ing enzymes with activities defined in at least three levels of EC classification. Out of the initial 416 FFs, 276 FFs were unambiguously linked to 347 EC numbers. These FFs catalyze 658 biochemical reactions recorded in KEGG (Kanehisa et al., 2010). Reaction directions and main reaction pairs were used to identify substrates and products of enzymatic activities.

  ### Attachments

  - 1-s2.0-S0969212611004163-main.pdf
- ## Protein Folding in the 2D Hydrophobic-Hydrophilic (HP) Square Lattice Model is Chaotic

  |  |  |
  | --- | --- |
  | Type | Journal Article |
  | Author | Jacques M. Bahi |
  | Author | Nathalie Cote |
  | Author | Christophe Guyeux |
  | Author | Michel Salomon |
  | Volume | 4 |
  | Issue | 1 |
  | Pages | 98-114 |
  | Publication | Cognitive Computation |
  | ISSN | 1866-9956 |
  | Date | MAR 2012 |
  | Extra | WOS:000305213200010 |
  | DOI | 10.1007/s12559-011-9118-z |
  | Abstract | Among the unsolved problems in computational biology, protein folding is one of the most interesting challenges. To study this folding, tools like neural networks and genetic algorithms have received a lot of attention, mainly due to the NP completeness of the folding process. The background idea that has given rise to the use of these algorithms is obviously that the folding process is predictable. However, this important assumption is disputable as chaotic properties of such a process have been recently highlighted. In this paper, which is an extension of a former work accepted to the 2011 International Joint Conference on Neural Networks (IJCNN11), the topological behavior of a well-known dynamical system used for protein folding prediction is evaluated. It is mathematically established that the folding dynamics in the 2D hydrophobic-hydrophilic (HP) square lattice model, simply called the 2D model in this document, is indeed a chaotic dynamical system as defined by Devaney. Furthermore, the chaotic behavior of this model is qualitatively and quantitatively deepened, by studying other mathematical properties of disorder, namely: the indecomposability, instability, strong transitivity, and constants of expansivity and sensitivity. Some consequences for both biological paradigms and structure prediction using this model are then discussed. In particular, it is shown that some neural networks seems to be unable to predict the evolution of this model with accuracy, due to its complex behavior. |
  | Date Added | 10/28/2013, 4:53:08 PM |
  | Modified | 10/28/2013, 4:53:08 PM |

  ### Notes:

  - Paper unavailable.
- ## Protein folding: is it simply surface to volume minimization?

  |  |  |
  | --- | --- |
  | Type | Journal Article |
  | Author | Aditya Mittal |
  | Author | Chanchal Acharya |
  | Volume | 31 |
  | Issue | 9 |
  | Pages | 953-955 |
  | Publication | Journal of Biomolecular Structure & Dynamics |
  | ISSN | 0739-1102 |
  | Date | SEP 1 2013 |
  | Extra | WOS:000323027400001 |
  | DOI | 10.1080/07391102.2012.748526 |
  | Date Added | 10/28/2013, 4:51:00 PM |
  | Modified | 10/28/2013, 4:51:00 PM |

  ### Notes:

  - Study of protein folding.

    How SCOP is used:

    Seem to be using ASTRAL domain structures.  Study backbone configurations of downloaded structures.

    Categorize into first 4 classes to count distrubiotions by SCOP class.

    SCOP reference:

    Figure 1 shows our results after analyzing backbones of a total of 13240 crystal structures of folded proteins from the Structural Classification of Proteins (SCOP) database (http://www.rcsb.org/pdb/, Andreeva et al., 2008; Berman, Henrick, Nakamura, & Markley, 2007).

  ### Attachments

  - 07391102%2E2012%2E748526.pdf
- ## Protein Fold Prediction using Cluster Merging

  |  |  |
  | --- | --- |
  | Type | Journal Article |
  | Author | Ngyuen Quang Phuoc |
  | Author | Sung-Ryul Kim |
  | Editor | F. I. S. Ko |
  | Editor | K. DalKwack |
  | Editor | S. Hwang |
  | Editor | S. Kawata |
  | Editor | Y. W. Chen |
  | Publication | 2011 6TH INTERNATIONAL CONFERENCE ON COMPUTER SCIENCES AND CONVERGENCE INFORMATION TECHNOLOGY (ICCIT) |
  | Date | 2012 |
  | Extra | WOS:000309767600059 |
  | Library Catalog | ISI Web of Knowledge |
  | Language | English |
  | Abstract | Protein folding prediction, also called protein structure prediction, is one of the most important issues for understanding living organisms. Therefore, predicting the folding structure of proteins from their linear sequence is a very big challenge in biology. Despite years of research and the wide variety of approaches, protein folding still remains a difficult problem. One of the main difficulties is controlling the over-fitting and under-fitting behavior of classifiers in the prediction systems. In this paper we propose a new learning method to improve the accuracy of protein folding prediction by balancing between over-fitting and under-fitting. The key of this method is based on a special way for analyzing the distance among training data points in order to cluster them into spaces which have high density of data points. By this, the over fitting and under fitting can be controlled in a comprehensive manner. Some experimental results seem to indicate that the proposed method has a significant potential on improve the accuracy of protein folding prediction. |
  | Date Added | 10/8/2014, 12:57:51 PM |
  | Modified | 10/8/2014, 12:59:02 PM |

  ### Tags:

  - accuracy
  - Algorithms
  - classification
  - Cluster
  - Decision Trees
  - ensemble classifier
  - Over-fitting
  - Protein folding prediction
  - recognition
  - scop
  - secondary structure
  - support vector machines
  - Under-fitting

  ### Notes:

  - Not available.
- ## Protein function prediction using domain families

  |  |  |
  | --- | --- |
  | Type | Journal Article |
  | Author | Robert Rentzsch |
  | Author | Christine A. Orengo |
  | Volume | 14 |
  | Pages | S5 |
  | Publication | Bmc Bioinformatics |
  | ISSN | 1471-2105 |
  | Date | FEB 28 2013 |
  | Extra | WOS:000317187500005 |
  | DOI | 10.1186/1471-2105-14-S3-S5 |
  | Abstract | Here we assessed the use of domain families for predicting the functions of whole proteins. These 'functional families' (FunFams) were derived using a protocol that combines sequence clustering with supervised cluster evaluation, relying on available high-quality Gene Ontology (GO) annotation data in the latter step. In essence, the protocol groups domain sequences belonging to the same superfamily into families based on the GO annotations of their parent proteins. An initial test based on enzyme sequences confirmed that the FunFams resemble enzyme (domain) families much better than do families produced by sequence clustering alone. For the CAFA 2011 experiment, we further associated the FunFams with GO terms probabilistically. All target proteins were first submitted to domain superfamily assignment, followed by FunFam assignment and, eventually, function assignment. The latter included an integration step for multi-domain target proteins. The CAFA results put our domain-based approach among the top ten of 31 competing groups and |
  | Date Added | 2/13/2014, 4:13:17 PM |
  | Modified | 3/7/2014, 12:09:01 PM |
- ## Protein homology detection by HMM-HMM comparison

  |  |  |
  | --- | --- |
  | Type | Journal Article |
  | Author | J Soding |
  | Volume | 21 |
  | Issue | 7 |
  | Pages | 951-960 |
  | Publication | Bioinformatics |
  | ISSN | 1367-4803 |
  | Date | APR 1 2005 |
  | DOI | 10.1093/bioinformatics/bti125 |
  | Language | English |
  | Abstract | Motivation: Protein homology detection and sequence alignment are at the basis of protein structure prediction, function prediction and evolution. Results: We have generalized the alignment of protein sequences with a profile hidden Markov model (HMM) to the case of pairwise alignment of profile HMMs. We present a method for detecting distant homologous relationships between proteins based on this approach. The method (HHsearch) is benchmarked together with BLAST, PSI-BLAST, HMMER and the profile-profile comparison tools PROF\_SIM and COMPASS, in an all-against-all comparison of a database of 3691 protein domains from SCOP 1.63 with pairwise sequence identities below 20%. Sensitivity: When the predicted secondary structure is included in the HMMs, HHsearch is able to detect between 2.7 and 4.2 times more homologs than PSI-BLAST or HMMER and between 1.44 and 1.9 times more than COMPASS or PROF\_SIM for a rate of false positives of 10%. Approximately half of the improvement over the profile-profile comparison methods is attributable to the use of profile HMMs in place of simple profiles. Alignment quality: Higher sensitivity is mirrored by an increased alignment quality. HHsearch produced 1.2, 1.7 and 3.3 times more good alignments ('balanced' score > 0.3) than the next best method (COMPASS), and 1.6, 2.9 and 9.4 times more than PSI-BLAST, at the family, superfamily and fold level, respectively. Speed: HHsearch scans a query of 200 residues against 3691 domains in 33 s on an AMD64 2GHz PC. This is 10 times faster than PROF\_SIM and 17 times faster than COMPASS. |
  | Date Added | 10/11/2013, 10:29:15 AM |
  | Modified | 11/9/2014, 1:56:16 PM |

  ### Notes:

  - HHSearch method for detecting remote homologies with HMMs.

    How SCOP is used:

    Use ASTRAL representative subset (<=80%) sequence data from SCOP 1.63 to benchmark their method.

    SCOP references:

    In Abstract:

    The method (HHsearch) is benchmarked together with BLAST, PSI- BLAST, HMMER and the profile–profile comparison tools PROF\_SIM and COMPASS, in an all-against-all comparison of a database of 3691 protein domains from SCOP 1.63 with pairwise sequence identities below 20%.

    Under Methods: Scoring secondary structure

    We predicted the secondary structure for all domains in SCOP (version 1.63, filtered to a maximum sequence identity of 20%) and compared the PSIPRED predictions for each residue with the DSSP assignments.

    Under Results and Discussion:

    The 3691 sequences of the SCOP database (Murzin et al., 1995) (version 1.63) filtered to a maximum sequence identity of 20% (‘SCOP-20’) were obtained from the ASTRAL server (Chandonia et al., 2004). Each sequence corresponds to a single structural domain, except for 73 sequences from the SCOP class of multi-domain proteins.

    Following SCOP, we classify each pair of domains as homologous if they are members of the same superfam- ily. Domains from different classes are classified as non-homologous. All other pairs are considered as ‘unknown’ in the benchmark since their evolutionary relationship cannot be ascertained.

    In an analysis of the complete data we found many pairs of sequences from different superfamilies and sometimes even different folds that HHsearch predicts as homologs with high confidence. In most cases their structures are also very similar, either in parts or globally. This convinced us that many superfamilies that are classi- fied by SCOP into different folds are in fact homologous. We name just two examples, the TIM barrels (Henn-Sax et al., 2001) (SCOP superfamilies c.1.1 – c.1.25) and the beta propellers (SCOP folds b.66 – b.70). To test how well the various methods detect these cases of structural similarity and putative homology, we analyze the data with a second, alternative definition of true and false positives. A pair is now defined as true positive if the domains belong to the same SCOP superfamily or if the sequence-based alignment yields a structural alignment with a MaxSub score (Siew et al., 2000) of at least 0.1. Pairs of sequences from different classes and with zero MaxSub score are classified as non-homologous. All other relationships are classified as unknown. Roughly speaking, the MaxSub score tells us what fraction of the query residues can be structurally superposed with the aligned residues from the other structure. It is defined such that a score >0 occurs rarely by chance.8

  ### Attachments

  - Full Text PDF
- ## Protein interactions in 3D: From interface evolution to drug discovery

  |  |  |
  | --- | --- |
  | Type | Journal Article |
  | Author | Christof Winter |
  | Author | Andreas Henschel |
  | Author | Anne Tuukkanen |
  | Author | Michael Schroeder |
  | URL | http://www.sciencedirect.com/science/article/pii/S1047847712001128 |
  | Volume | 179 |
  | Issue | 3 |
  | Pages | 347–358 |
  | Publication | Journal of Structural Biology |
  | Date | 2012 |
  | Accessed | 9/20/2013, 1:18:03 PM |
  | Library Catalog | Google Scholar |
  | Short Title | Protein interactions in 3D |
  | Date Added | 2/13/2014, 4:13:17 PM |
  | Modified | 3/7/2014, 12:10:34 PM |

  ### Notes:

  - Review of research on 3D protein interactions.

    How SCOP/CATH is used:

    Review a number of databases, methods, and studies that make use of SCOP or CATH data.

    SCOP/CATH reference:

    The above definitions of interfaces led to various databases pro- viding access to all structural protein interactions in the PDB such as 3did, iPfam, PIBASE, SCOPPI,1 SCOWLP, PRISM, PSIBASE, DOMINE and PSIMAP (Stein et al., 2005; Finn et al., 2005; Davis and Sali, 2005; Winter et al., 2006; Teyra et al., 2006; Ogmen et al., 2005; Gong et al., 2005b; Raghavachari et al., 2008; Park et al., 2001). These databases are usually based on domain–domain interactions. Domain defini- tions are taken from SCOP, the structural classification of proteins (Murzin et al., 1995), from CATH (Orengo et al., 1997), from Pfam (Bateman et al., 2004), or from the conserved domain database, CDD (Marchler-Bauer et al., 2005).

  ### Attachments

  - 1-s2.0-S1047847712001128-main.pdf
- ## Protein loops, solitons, and side-chain visualization with applications to the left-handed helix region

  |  |  |
  | --- | --- |
  | Type | Journal Article |
  | Author | Martin Lundgren |
  | Author | Antti J. Niemi |
  | Author | Fan Sha |
  | Volume | 85 |
  | Issue | 6 |
  | Pages | 061909 |
  | Publication | Physical Review E |
  | ISSN | 1539-3755 |
  | Date | JUN 11 2012 |
  | Extra | WOS:000305128000007 |
  | DOI | 10.1103/PhysRevE.85.061909 |
  | Abstract | Folded proteins have a modular assembly. They are constructed from regular secondary structures like alpha helices and beta strands that are joined together by loops. Here we develop a visualization technique that is adapted to describe this modular structure. In complement to the widely employed Ramachandran plot that is based on toroidal geometry, our approach utilizes the geometry of a two sphere. Unlike the more conventional approaches that describe only a given peptide unit, ours is capable of describing the entire backbone environment including the neighboring peptide units. It maps the positions of each atom to the surface of the two-sphere exactly how these atoms are seen by an observer who is located at the position of the central C-alpha atom. At each level of side-chain atoms we observe a strong correlation between the positioning of the atom and the underlying local secondary structure with very little if any variation between the different amino acids. As a concrete example we analyze the left-handed helix region of nonglycyl amino acids. This region corresponds to an isolated and highly localized residue independent sector in the direction of the C-beta carbons on the two-sphere. We show that the residue independent localization extends to C gamma and C-delta carbons and to side-chain oxygen and nitrogen atoms in the case of asparagine and aspartic acid. When we extend the analysis to the side-chain atoms of the neighboring residues, we observe that left-handed beta turns display a regular and largely amino acid independent structure that can extend to seven consecutive residues. This collective pattern is due to the presence of a backbone soliton. We show how one can use our visualization techniques to analyze and classify the different solitons in terms of selection rules that we describe in detail. |
  | Date Added | 2/13/2014, 4:13:17 PM |
  | Modified | 3/7/2014, 12:14:21 PM |
- ## Protein profile in vascular wall of atherosclerotic mice analyzed ex vivo using FT-IR spectroscopy

  |  |  |
  | --- | --- |
  | Type | Journal Article |
  | Author | Tomasz P. Wrobel |
  | Author | Katarzyna Majzner |
  | Author | Malgorzata Baranska |
  | Volume | 96 |
  | Pages | 940-945 |
  | Publication | Spectrochimica Acta Part a-Molecular and Biomolecular Spectroscopy |
  | ISSN | 1386-1425 |
  | Date | OCT 2012 |
  | Extra | WOS:000311248500117 |
  | DOI | 10.1016/j.saa.2012.07.103 |
  | Abstract | The structure of proteins in a tissue can undergo changes on account of disease state such as diabetes or atherosclerosis. In this work the protein profile in atherosclerotic tissue is monitored by FT-IR imaging coupled with Hierarchical Cluster Analysis (HCA). Additionally, a model for prediction of secondary structure of proteins content based on amide I and II range is used to show the distribution of analyzed proteins. A new protein class emerged in atherosclerotic tissue in the region of the plaque and additionally the plaque was found to be strongly mixed with smooth muscle cell. The calculated secondary structure contents of proteins in atherosclerotic tissue in comparison to healthy tissue showed an increase of structures related to beta-sheet (E and T) and a decrease of helical (H) and unassigned arrangements. (c) 2012 Elsevier B.V. All rights reserved. |
  | Date Added | 2/13/2014, 4:13:17 PM |
  | Modified | 3/7/2014, 12:10:32 PM |
- ## Protein-protein docking benchmark version 3.0

  |  |  |
  | --- | --- |
  | Type | Journal Article |
  | Author | Howook Hwang |
  | Author | Brian Pierce |
  | Author | Julian Mintseris |
  | Author | Joël Janin |
  | Author | Zhiping Weng |
  | Volume | 73 |
  | Issue | 3 |
  | Pages | 705-709 |
  | Publication | Proteins |
  | ISSN | 1097-0134 |
  | Date | Nov 15, 2008 |
  | Extra | PMID: 18491384 |
  | Journal Abbr | Proteins |
  | DOI | 10.1002/prot.22106 |
  | Library Catalog | NCBI PubMed |
  | Language | eng |
  | Abstract | We present version 3.0 of our publicly available protein-protein docking benchmark. This update includes 40 new test cases, representing a 48% increase from Benchmark 2.0. For all of the new cases, the crystal structures of both binding partners are available. As with Benchmark 2.0, Structural Classification of Proteins (Murzin et al., J Mol Biol 1995;247:536-540) was used to remove redundant test cases. The 124 unbound-unbound test cases in Benchmark 3.0 are classified into 88 rigid-body cases, 19 medium-difficulty cases, and 17 difficult cases, based on the degree of conformational change at the interface upon complex formation. In addition to providing the community with more test cases for evaluating docking methods, the expansion of Benchmark 3.0 will facilitate the development of new algorithms that require a large number of training examples. Benchmark 3.0 is available to the public at http://zlab.bu.edu/benchmark. |
  | Date Added | 10/11/2013, 10:29:15 AM |
  | Modified | 10/11/2013, 10:29:15 AM |

  ### Tags:

  - Algorithms
  - Animals
  - complex structure
  - Databases, Protein
  - Internet
  - protein complexes
  - Protein Interaction Mapping
  - protein-protein docking
  - protein-protein interactions

  ### Notes:

  - Released updates to the Zdock docking benchmarking data set.

    How SCOP is used:

    Use SCOP data in semiautomated dataset retrieval and curation workflow. First, collected complexes from the PDB.  Then used SCOP to filter down a dataset, so that no two test cases belong to the same family-family pair.

    SCOP reference:

    For the remaining protein complexes, we utilized Structural Classification of Proteins (SCOP)1 to examine protein family–family pair redundancy within the new cases and against the existing cases from Benchmark 2.0. In addition to the latest version of SCOP (1.71), which was released in Oct. 2006, we used its preclassification version, Pre-SCOP (http://www.mrc-lmb.cam.ac.uk/agm/pre-scop/), for the structures deposited in PDB since the SCOP 1.71 release. Nonredundancy was set at the family level of SCOP, that is, no two test cases in Benchmark 3.0 are allowed to belong to the same family–family pair. The users who are interested in developing statistical potentials with our benchmark may also want to exclude test cases that belong to the same superfamily–superfamily pairs. This would affect two pairs of test cases: 1EZU/1N8O and 1GRN/1WQ1 (labeled with ‘‘\*’’ in Table I). To avoid this level of redundancy, one test case from each of these pairs can be removed. We then eliminated the test cases for which the unbound structures had less than 96% sequence identity to the corresponding bound structures, as defined by BLAST.16 For the remaining test cases with multiple crystal structures of the unbound proteins, we chose the unbound structure with the highest sequence similarity, highest structure resolution, and fewest missing residues. Finally, we discarded test cases that present unusual diffi- culties for docking algorithms, for example, three or more residues in the binding site were missing in the unbound structure, or the bound and the unbound structures have different cofactors at the binding site. The cofactors included in structures are listed in the table at the bench- mark website (http://zlab.bu.edu/benchmark).

  ### Attachments

  - PubMed entry
  - zdock\_2008.pdf
- ## Protein-protein docking benchmark version 4.0

  |  |  |
  | --- | --- |
  | Type | Journal Article |
  | Author | Howook Hwang |
  | Author | Thom Vreven |
  | Author | Joël Janin |
  | Author | Zhiping Weng |
  | Volume | 78 |
  | Issue | 15 |
  | Pages | 3111-3114 |
  | Publication | Proteins |
  | ISSN | 1097-0134 |
  | Date | Nov 15, 2010 |
  | Extra | PMID: 20806234 |
  | Journal Abbr | Proteins |
  | DOI | 10.1002/prot.22830 |
  | Library Catalog | NCBI PubMed |
  | Language | eng |
  | Abstract | We updated our protein-protein docking benchmark to include complexes that became available since our previous release. As before, we only considered high-resolution complex structures that are nonredundant at the family-family pair level, for which the X-ray or NMR unbound structures of the constituent proteins are also available. Benchmark 4.0 adds 52 new complexes to the 124 cases of Benchmark 3.0, representing an increase of 42%. Thus, benchmark 4.0 provides 176 unbound-unbound cases that can be used for protein-protein docking method development and assessment. Seventeen of the newly added cases are enzyme-inhibitor complexes, and we found no new antigen-antibody complexes. Classifying the new cases according to expected difficulty for protein-protein docking algorithms gives 33 rigid body cases, 11 cases of medium difficulty, and 8 cases that are difficult. Benchmark 4.0 listings and processed structure files are publicly accessible at http://zlab.umassmed.edu/benchmark/. |
  | Date Added | 10/11/2013, 10:29:15 AM |
  | Modified | 10/11/2013, 10:29:15 AM |

  ### Tags:

  - Algorithms
  - complex structure
  - Computational Biology
  - Crystallography, X-Ray
  - Databases, Protein
  - Models, Chemical
  - Models, Molecular
  - Nuclear Magnetic Resonance, Biomolecular
  - protein complexes
  - Protein Conformation
  - Protein Interaction Mapping
  - protein-protein docking
  - protein-protein interactions
  - Proteins
  - Software

  ### Notes:

  - Describe release of zdock bench marking dataset version 4.0.

    How SCOP is used:

    Use SCOP as previously, to filter a data set of complexes collected from the PDB so there are no test cases from the same family-family pair.

    SCOP reference:

    The unbound forms of both binding partners were available for 1667 complex structures, and we used the Structural Classifica- tion of Proteins (SCOP)16 database (version 1.75) to check this set for redundancy at the family level. Two complexes were deemed redun- dant if both proteins in one complex were in the same SCOP families as the two proteins in the other complex, respectively. This yielded 109 complexes that were non- redundant with the complexes in the previous release of the Benchmark and amongst themselves. (PDB entries without SCOP unique identifier sunid17 were excluded from the bound candidate list to remove possible redun- dancy.) Finally, we used literature information to elimi- nate obligate complexes,18 which further reduced the list to 52 complexes.

  ### Attachments

  - PubMed entry
  - zdock-2010.pdf
- ## Protein Remote Homology Detection by Combining Chou's Pseudo Amino Acid Composition and Profile-Based Protein Representation

  |  |  |
  | --- | --- |
  | Type | Journal Article |
  | Author | Bin Liu |
  | Author | Xiaolong Wang |
  | Author | Quan Zou |
  | Author | Qiwen Dong |
  | Author | Qingcai Chen |
  | Volume | 32 |
  | Issue | 9-10 |
  | Pages | 775-782 |
  | Publication | Molecular Informatics |
  | ISSN | 1868-1743; 1868-1751 |
  | Date | OCT 2013 |
  | Extra | WOS:000330179500002 |
  | DOI | 10.1002/minf.201300084 |
  | Abstract | Protein remote homology detection is a key problem in bioinformatics. Currently the discriminative methods, such as Support Vector Machine (SVM) can achieve the best performance. The most efficient approach to improve the performance of SVM-based methods is to find a general protein representation method that is able to convert proteins with different lengths into fixed length vectors and captures the different properties of the proteins for the discrimination. The bottleneck of designing the protein representation method is that native proteins have different lengths. Motivated by the success of the pseudo amino acid composition (PseAAC) proposed by Chou, we applied this approach for protein remote homology detection. Some new indices derived from the amino acid index (AAIndex) database are incorporated into the PseAAC to improve the generalization ability of this method. Finally, the performance is further improved by combining the modified PseAAC with profile-based protein representation containing the evolutionary information extracted from the frequency profiles. Our experiments on a well-known benchmark show this method achieves superior or comparable performance with current state-of-the-art methods. |
  | Date Added | 2/20/2014, 12:24:01 PM |
  | Modified | 2/20/2014, 12:24:01 PM |

  ### Notes:

  - Present method for remote homology detection using.

    How SCOP is used:

    Use SCOP 1.53 data from Astral to train and benchmark their method.  Use family and superfamily levels.

    SCOP reference:

    The benchmark contains 4352 proteins selected from SCOP version 1.53. These proteins are extracted from the Astral database[45] and include no pair with a sequence sim- ilarity higher than an E-value of 10⬚⬚25. The 4352 proteins can be classified into 853 superfamilies and 1356 families. For readers’ convenience, the codes of the 4352 proteins and their sequence as well as the attributes of their families and superfamilies are given in Supporting Information S1. 54 families with significant number of proteins are selected as the target families from the 1356 families in order to val- idate the performance of the proposed method. For each target family, the proteins within the family are taken as positive test samples, and the proteins outside the family but within the same superfamily are taken as positive train- ing samples. Negative samples are selected from outside of the superfamily and are separated into training and test sets. The 54 training and testing datasets thus obtained are given in the Online Supporting Information S2 and Sup- porting Information S3, respectively.

    ...

    3.2 Comparison with Other Sequence-Based Methods

    In order to compare the proposed PseAACIndex method with other relevant protein remote homology detection methods, the PseAACIndex was evaluated on the widely used SCOP 1.53 dataset to give an unbiased comparison with prior methods that are based on sequence composi- tion information.

  ### Attachments

  - 775\_ftp.pdf
- ## Protein Secondary Structure Prediction with SPARROW

  |  |  |
  | --- | --- |
  | Type | Journal Article |
  | Author | Francesco Bettella |
  | Author | Dawid Rasinski |
  | Author | Ernst Walter Knapp |
  | Volume | 52 |
  | Issue | 2 |
  | Pages | 545-556 |
  | Publication | JOURNAL OF CHEMICAL INFORMATION AND MODELING |
  | ISSN | 1549-9596 |
  | Date | February 2012 |
  | DOI | 10.1021/ci200321u |
  | Language | English |
  | Abstract | A first step toward predicting the structure of a protein is to determine its secondary structure. The secondary structure information is generally used as starting point to solve protein crystal structures. In the present study, a machine learning approach based on a complete set of two-class scoring function:; was used. Such functions discriminate between two specific structural classes or between a single specific class and the rest. The approach uses a hierarchical scheme of scoring functions and a neural network. The parameters are determined by optimizing the recall of learning data. Quality control performed by predicting separate independent test data. A first set of scoring functions is trained to correlate the secondary structures of residues with profiles of sequence windows of width 15, centered at these residues. The sequence profiles are obtained by multiple sequence alignment with PSI-BLAST. A second set of scoring functions is trained to correlate the secondary structures of the center residues with the secondary structures of all other residues in the sequence windows used in the first step. Finally, a neural network is trained using the results from the second set of scoring functions as input to make a decision on the secondary structure class of the residue in the center of the sequence window, Here, we consider the three-class problem of helix, strand, and other secondary structures. The corresponding prediction scheme “SPARROW” was trained with the ASTRAL40 database, which contains protein domain structures with less than 40% sequence identity. The secondary structures were determined with DSSP. In a loose assignment, the helix class contains all DSSP helix types (alpha, 3-10, pi), the strand class contains beta-strand and beta-bridge, and the third class contain.; the other structures. In a tight assignment, the helix and strand classes contain only alpha-helix and beta-strand classes, respectively. A 10-fold cross validation showed less than 0.8% deviation in the fraction of correct structure assignments between true prediction and recall of data used for training. Using sequences of 140,000 residues as a test data set, 80.46% +/- 0.35% of secondary structures are predicted correctly in the loose assignment, a prediction performance, which is very close to the best results in the field. Most applications are done with the loose assignment. However, the tight assignment yields 2.25% better prediction performance. With each individual prediction, we also provide a confidence measure providing the probability that the prediction is correct. The SPARROW software can be used and downloaded on the Web page http://agknapp.chemie.fu-berlin.de/sparrow/. |
  | Date Added | 10/25/2013, 4:23:37 PM |
  | Modified | 3/7/2014, 12:15:26 PM |

  ### Notes:

  - Present SPARROW method for protein secondary structure prediction.

    How SCOP is used:

    Evaluate method on non-redundant data set.

    Downloaded a dataset from ASTRAL, filtered at 40% sequence identity.

    SCOP reference:

    Secondary Structure Databases. The data sets of protein domain structures used for secondary structure prediction are based on ASTRAL40 from the SCOP database.85−87 Proteins in the ASTRAL40 sets have sequence identities of less than 40%. General information on number of protein domains, number of residues, and release dates of the different versions of ASTRAL40 used in the present study are listed in Table 1.

  ### Attachments

  - ci200321u.pdf
- ## Protein sequence comparison based on K-string dictionary

  |  |  |
  | --- | --- |
  | Type | Journal Article |
  | Author | Chenglong Yu |
  | Author | Rong L. He |
  | Author | Stephen S.-T. Yau |
  | Volume | 529 |
  | Issue | 2 |
  | Pages | 250-256 |
  | Publication | Gene |
  | Date | OCT 2013 |
  | Extra | WOS:000325122800008 |
  | DOI | 10.1016/j.gene.2013.07.092 |
  | Library Catalog | ISI Web of Knowledge |
  | Abstract | The current K-string-based protein sequence comparisons require large amounts of computer memory because the dimension of the protein vector representation grows exponentially with K. In this paper, we propose a novel concept, the "K-string dictionary", to solve this high-dimensional problem. It allows us to use a much lower dimensional K-string-based frequency or probability vector to represent a protein, and thus significantly reduce the computer memory requirements for their implementation. Furthermore, based on this new concept we use Singular Value Decomposition to analyze real protein datasets, and the improved protein vector representation allows us to obtain accurate gene trees. (C) 2013 Elsevier B.V. All rights reserved. |
  | Date Added | 10/8/2014, 12:49:22 PM |
  | Modified | 10/8/2014, 1:32:44 PM |

  ### Tags:

  - Cardinality
  - Frequency vector
  - K-string
  - Sequence comparison
  - Singular Value Decomposition

  ### Attachments

  - ScienceDirect Full Text PDF
  - ScienceDirect Snapshot
- ## Protein Similarity Networks Reveal Relationships among Sequence, Structure, and Function within the Cupin Superfamily

  |  |  |
  | --- | --- |
  | Type | Journal Article |
  | Author | Richard Uberto |
  | Author | Ellen W. Moomaw |
  | Volume | 8 |
  | Issue | 9 |
  | Publication | Plos One |
  | ISSN | 1932-6203 |
  | Date | SEP 6 2013 |
  | Extra | WOS:000324856500083 |
  | DOI | 10.1371/journal.pone.0074477 |
  | Abstract | The cupin superfamily is extremely diverse and includes catalytically inactive seed storage proteins, sugar-binding metal-independent epimerases, and metal-dependent enzymes possessing dioxygenase, decarboxylase, and other activities. Although numerous proteins of this superfamily have been structurally characterized, the functions of many of them have not been experimentally determined. We report the first use of protein similarity networks (PSNs) to visualize trends of sequence and structure in order to make functional inferences in this remarkably diverse superfamily. PSNs provide a way to visualize relatedness of structure and sequence among a given set of proteins. Structure- and sequence-based clustering of cupin members reflects functional clustering. Networks based only on cupin domains and networks based on the whole proteins provide complementary information. Domain-clustering supports phylogenetic conclusions that the N- and C-terminal domains of bicupin proteins evolved independently. Interestingly, although many functionally similar enzymatic cupin members bind the same active site metal ion, the structure and sequence clustering does not correlate with the identity of the bound metal. It is anticipated that the application of PSNs to this superfamily will inform experimental work and influence the functional annotation of databases. |
  | Date Added | 10/28/2013, 4:53:08 PM |
  | Modified | 3/7/2014, 12:08:44 PM |

  ### Notes:

  - Computational study of structure and sequence similarity in Cupin Superfamily.

    How SCOP is used:

    Look up information on 'cupin' superfamily

    SCOP reference:

    Inconsistencies exist in the usage of the term ‘cupin’. According to the Structural Classification of Proteins (SCOP) database [12,13,14], cupin proteins are members of the ‘RmlC-like Cupins’ superfamily within the double-stranded b-helix (DSBH) multi- catalytic fold [15]. The term ‘cupin superfamily’ has often been used to refer to those proteins defined by the SCOP database as well as the 2-oxoglutarate-Fe2+-dependent dioxygenase superfam- ily that also possesses the DSBH fold [1,5]. However defined, the cupin superfamily is extremely diverse and includes catalytically inactive seed storage and sugar-binding metal-independent pro- teins as well as metal-dependent enzymes possessing dioxygenase, decarboxylase, and other activities [10].

  ### Attachments

  - journal.pone.0074477.pdf
- ## Protein space: A natural method for realizing the nature of protein universe

  |  |  |
  | --- | --- |
  | Type | Journal Article |
  | Author | Chenglong Yu |
  | Author | Mo Deng |
  | Author | Shiu-Yuen Cheng |
  | Author | Shek-Chung Yau |
  | Author | Rong L. He |
  | Author | Stephen S. -. T. Yau |
  | Volume | 318 |
  | Pages | 197–204 |
  | Publication | Journal of Theoretical Biology |
  | Date | February 2013 |
  | DOI | 10.1016/j.jtbi.2012.11.005 |
  | Abstract | Current methods cannot tell us what the nature of the protein universe is concretely. They are based on different models of amino acid substitution and multiple sequence alignment which is an NP-hard problem and requires manual intervention. Protein structural analysis also gives a direction for mapping the protein universe. Unfortunately, now only a minuscule fraction of proteins' 3-dimensional structures are known. Furthermore, the phylogenetic tree representations are not unique for any existing tree construction methods. Here we develop a novel method to realize the nature of protein universe. We show the protein universe can be realized as a protein space in 60-dimensional Euclidean space using a distance based on a normalized distribution of amino acids. Every protein is in one-to-one correspondence with a point in protein space, where proteins with similar properties stay close together. Thus the distance between two points in protein space represents the biological distance of the corresponding two proteins. We also propose a natural graphical representation for inferring phylogenies. The representation is natural and unique based on the biological distances of proteins in protein space. This will solve the fundamental question of how proteins are distributed in the protein universe. (C) 2012 Elsevier Ltd. All rights reserved. |
  | Date Added | 3/7/2014, 1:06:24 PM |
  | Modified | 3/7/2014, 1:06:24 PM |
- ## Protein structural classification based on pseudo amino acid composition using SVM classifier

  |  |  |
  | --- | --- |
  | Type | Journal Article |
  | Author | Zbigniew Krajewski |
  | Author | Ewaryst Tkacz |
  | URL | http://www.sciencedirect.com/science/article/pii/S020852161300003X |
  | Publication | Biocybernetics and Biomedical Engineering |
  | Date | 2013 |
  | Accessed | 9/23/2013, 10:24:56 AM |
  | Library Catalog | Google Scholar |
  | Date Added | 10/11/2013, 10:29:15 AM |
  | Modified | 3/6/2014, 4:04:33 PM |

  ### Tags:

  - Minimal-distance methods
  - protein structural class
  - pseudo amino acid composition
  - SCOP database
  - support vector machine

  ### Notes:

  - Propose machine-learning method for protein structure classification at the structural class level.

    How SCOP is used:

    Train and validate on SCOP data from ASTRAL non-redundant set.

    SCOP reference:

    In abstract:

    The SCOP database and the ASTRAL tool were a source of non-homologous data to avoid the redundancy and to ensure a maximal amount of available data.

    ...

    2.1. Data set

    Classification method of structural class used in this paper is the method of learning with teacher using training data pool. The data was split into three data pools: training, test and validation. To overcome the problem of data redundancy, the classic 30% of paired identity threshold of significant homolo- gy was applied. Certainly, the choice based on e-value much better realize homology distinction with respect to twilight zone in the great database. In spite of this, we used the traditional threshold in order to compare with other applica- tion which would seem to be reliable from our point of view [30]. The domain as a basic classification entity was used as proposed by Murzin from the SCOP database based on structural and sequential similarity and so called evolutionary relationship [31]. We chose 7702 non-homologous domains using the ASTRAL application meant for some processing with the SCOP database, issue of 1.75 [32]. From this group of domains the tree data pools was chosen.

  ### Attachments

  - 1-s2.0-S020852161300003X-main.pdf
  - Snapshot

    ## Abstract

    This paper deals with a structural classification by the aid of support vector machine (SVM) classifier. Amino acid composition (AAC) and pseudo amino acid composition (PseAA) features were applied with different variants. Additionally the feature reflecting the length of protein chain was taken into consideration. The SVM classifier was compared to minimal-length classifiers with respect to the AAC features. The best model of SVM classifier was chosen using grid method on the basis of cross-validation (CV) as criterion. The best model of SVM classifier is evaluated with respect to proper evaluation rates. The SCOP database and the ASTRAL tool were a source of non-homologous data to avoid the redundancy and to ensure a maximal amount of available data.

    ## Keywords

    - Pseudo amino acid composition;
    - Support vector machine;
    - Minimal-distance methods;
    - Protein structural class;
    - SCOP database

    ---
- ## Protein Structural Statistics with PSS

  |  |  |
  | --- | --- |
  | Type | Journal Article |
  | Author | Thomas Gaillard |
  | Author | Benjamin B. L. Schwarz |
  | Author | Yassmine Chebaro |
  | Author | Roland H. Stote |
  | Author | Annick Dejaegere |
  | Volume | 53 |
  | Issue | 9 |
  | Pages | 2471–2482 |
  | Publication | Journal of Chemical Information and Modeling |
  | Date | September 2013 |
  | DOI | 10.1021/ci400233j |
  | Abstract | Characterizing the variability within an ensemble of protein structures is a common requirement in structural biology and bioinformatics. With the increasing number of protein structures becoming available, there is a need for new tools capable of automating the structural comparison of large ensemble of structures. We present Protein Structural Statistics (PSS), a command-line program written in Perl for Unix-like environments, dedicated to the calculation of structural statistics for a set of proteins. PSS can perform multiple sequence alignments, structure superpositions, calculate Cartesian and dihedral coordinate statistics, and execute cluster analyses. An HTML report that contains a convenient summary of results with figures, tables, and hyperlinks can also be produced. PSS is a new tool providing an automated way to compare multiple structures. It integrates various types of structural analyses through an user-friendly and flexible interface, facilitating the access to powerful but more specialized programs. PSS is easy to modify and extend and is distributed under a free and open source license. The relevance of PSS is illustrated by examples of application to pertinent biological problems. |
  | Date Added | 3/7/2014, 1:06:24 PM |
  | Modified | 3/7/2014, 1:06:24 PM |
- ## Protein structure alignment beyond spatial proximity

  |  |  |
  | --- | --- |
  | Type | Journal Article |
  | Author | Sheng Wang |
  | Author | Jianzhu Ma |
  | Author | Jian Peng |
  | Author | Jinbo Xu |
  | URL | http://www.nature.com/srep/2013/130314/srep01448/full/srep01448.html?WT.ec\_id=SREP-20130319 |
  | Volume | 3 |
  | Publication | Scientific reports |
  | Date | 2013 |
  | Accessed | 9/23/2013, 10:20:20 AM |
  | Library Catalog | Google Scholar |
  | Date Added | 10/11/2013, 10:29:15 AM |
  | Modified | 3/7/2014, 1:06:36 PM |

  ### Notes:

  - This paper presents a novel method DeepAlign for automatic pairwise protein structure alignment. DeepAlign aligns two protein structures using not only spatial proximity of equivalent residues (after rigid-body superposition), but also evolutionary relationship and hydrogen-bonding similarity.

    How SCOP is used:

    Evaluate DeepAlign on harder cases where structures are analogous, but come from different Folds.  Used Non-SCOP  previously published dataset (MALISAM) which has 130 protein pairs that are "structural analogs" from different SCOP folds.

    SCOP reference:   

    The benchmarks. We use three manually-curated benchmarks: (i) A subset of CDD (Conserved Domain Database)31 used in20; (ii) MALIDUP32; and (iii) MALISAM33. The CDD set contains 3591 manually-curated pairwise structure alignments. The human- curated alignments for CDD contain only the alignments of core residues. The CDD set has already been used to evaluate a bunch of pairwise structure alignment algorithms34, including CE4, FAST8, LOCK235, MATRAS36, VAST10 and SHEBA9. MALIDUP has 241 manually-curated pairwise structure alignments for homologous domains originated from internal duplication within the same polypeptide chain. About half of the pairs in MALIDUP are re- mote homologs. MALISAM contains 130 protein pairs and the two proteins in any pair are structural analogs with different SCOP37 folds. There is strong evidence indicating that proteins in a MALIDUP pair are not homologs38. Therefore, MALIDUP are the most challenging benchmark among these three. The alignments in these three databases are manually-curated, taking into consider- ation not only geometric similarity, but also evolutionary and func- tional relationship. Therefore, the manually-curated alignments make more biological sense and it is reasonable to use them as reference to judge automatically-generated alignments.

  ### Attachments

  - srep01448.pdf
- ## Protein structure alignment by incremental combinatorial extension (CE) of the optimal path.

  |  |  |
  | --- | --- |
  | Type | Journal Article |
  | Author | Ilya N. Shindyalov |
  | Author | Philip E. Bourne |
  | URL | http://peds.oxfordjournals.org/content/11/9/739.short |
  | Volume | 11 |
  | Issue | 9 |
  | Pages | 739–747 |
  | Publication | Protein engineering |
  | Date | 1998 |
  | Accessed | 10/10/2013, 1:18:50 PM |
  | Library Catalog | Google Scholar |
  | Date Added | 10/11/2013, 10:29:15 AM |
  | Modified | 10/11/2013, 10:29:15 AM |

  ### Notes:

  - Incremental Combinatorial Extension (CE) method for protein structure alignment.

    How SCOP is used:

    Used SCOP in one task for "detecting a protein fold",  In particular, they wanted to detect the 4-helical up-and-down bundle.  Collected a data set from SCOP of all 54 chains with 4-helical up-and-down bundle fold and removed redundant sequences, limiting down to 24 chains.

    SCOP reference:

    PDF is locked.

  ### Attachments

  - [PDF] from oxfordjournals.org
  - Snapshot
- ## Protein structure database search and evolutionary classification

  |  |  |
  | --- | --- |
  | Type | Journal Article |
  | Author | Jinn-Moon Yang |
  | Author | Chi-Hua Tung |
  | Volume | 34 |
  | Issue | 13 |
  | Pages | 3646-3659 |
  | Publication | Nucleic Acids Research |
  | ISSN | 1362-4962 |
  | Date | 2006 |
  | Extra | PMID: 16885238 PMCID: PMC1540718 |
  | Journal Abbr | Nucleic Acids Res. |
  | DOI | 10.1093/nar/gkl395 |
  | Library Catalog | NCBI PubMed |
  | Language | eng |
  | Abstract | As more protein structures become available and structural genomics efforts provide structural models in a genome-wide strategy, there is a growing need for fast and accurate methods for discovering homologous proteins and evolutionary classifications of newly determined structures. We have developed 3D-BLAST, in part, to address these issues. 3D-BLAST is as fast as BLAST and calculates the statistical significance (E-value) of an alignment to indicate the reliability of the prediction. Using this method, we first identified 23 states of the structural alphabet that represent pattern profiles of the backbone fragments and then used them to represent protein structure databases as structural alphabet sequence databases (SADB). Our method enhanced BLAST as a search method, using a new structural alphabet substitution matrix (SASM) to find the longest common substructures with high-scoring structured segment pairs from an SADB database. Using personal computers with Intel Pentium4 (2.8 GHz) processors, our method searched more than 10 000 protein structures in 1.3 s and achieved a good agreement with search results from detailed structure alignment methods. [3D-BLAST is available at http://3d-blast.life.nctu.edu.tw]. |
  | Date Added | 10/10/2014, 4:37:17 PM |
  | Modified | 10/10/2014, 4:37:17 PM |

  ### Tags:

  - Acetyltransferases
  - Databases, Protein
  - Data Interpretation, Statistical
  - Evolution, Molecular
  - Protein Conformation
  - Proteins
  - Sequence Alignment
  - Sequence Analysis, Protein
  - Software
  - Structural Homology, Protein

  ### Attachments

  - PubMed entry
- ## Protein structure fitting and refinement guided by cryo-EM density

  |  |  |
  | --- | --- |
  | Type | Journal Article |
  | Author | Maya Topf |
  | Author | Keren Lasker |
  | Author | Ben Webb |
  | Author | Haim Wolfson |
  | Author | Wah Chiu |
  | Author | Andrej Sali |
  | Volume | 16 |
  | Issue | 2 |
  | Pages | 295-307 |
  | Publication | Structure (London, England: 1993) |
  | ISSN | 0969-2126 |
  | Date | Feb 2008 |
  | Extra | PMID: 18275820 |
  | Journal Abbr | Structure |
  | DOI | 10.1016/j.str.2007.11.016 |
  | Library Catalog | NCBI PubMed |
  | Language | eng |
  | Abstract | For many macromolecular assemblies, both a cryo-electron microscopy map and atomic structures of its component proteins are available. Here we describe a method for fitting and refining a component structure within its map at intermediate resolution (<15 A). The atomic positions are optimized with respect to a scoring function that includes the crosscorrelation coefficient between the structure and the map as well as stereochemical and nonbonded interaction terms. A heuristic optimization that relies on a Monte Carlo search, a conjugate-gradients minimization, and simulated annealing molecular dynamics is applied to a series of subdivisions of the structure into progressively smaller rigid bodies. The method was tested on 15 proteins of known structure with 13 simulated maps and 3 experimentally determined maps. At approximately 10 A resolution, Calpha rmsd between the initial and final structures was reduced on average by approximately 53%. The method is automated and can refine both experimental and predicted atomic structures. |
  | Date Added | 10/11/2013, 10:29:15 AM |
  | Modified | 10/11/2013, 10:29:15 AM |

  ### Notes:

  - Describe a method for fitting and refining a structure (known from other experimental data) to a cryo-em map.

    How SCOP is used:

    Used SCOP website data to assign domains to the two multidomain proteins that make up their test set.

    SCOP reference:

    Both the GroEL monomer and EF-Tu were assigned three domains each, based on SCOP (Murzin et al., 1995).

  ### Attachments

  - 1-s2.0-S0969212608000130-main.pdf
  - PubMed entry
- ## Protein structure networks

  |  |  |
  | --- | --- |
  | Type | Journal Article |
  | Author | Lesley H. Greene |
  | URL | http://bfg.oxfordjournals.org/content/11/6/469.short |
  | Volume | 11 |
  | Issue | 6 |
  | Pages | 469–478 |
  | Publication | Briefings in functional genomics |
  | Date | 2012 |
  | Accessed | 9/23/2013, 10:15:34 AM |
  | Library Catalog | Google Scholar |
  | Date Added | 10/11/2013, 10:29:15 AM |
  | Modified | 3/7/2014, 12:14:48 PM |

  ### Tags:

  - allostery
  - graph theory
  - long-range interactions
  - networks
  - protein folding
  - protein structure

  ### Notes:

  - Review of how network science has furthered understanding of protein relationships. Lists various research examples in protein structure and organization.

    How SCOP/CATH is used:

    SCOP is referenced as an example of a database that organizes protein structures hierarchically. The article notes all the SCOP's levels in order, but doesn't actually make use of SCOP data.

    SCOP/CATH Reference:

    There are two leading  
    databases which organize this vast collection of structures  
    using a hierarchical approach. These are the  
    CATH [54] and SCOP [55] databases. Both databases  
    initially group proteins according to secondary  
    structure content such as mainly a-helical, mainly  
    b-sheet or a combination of a and b structure  
    which is termed ‘Class’ and then move down the  
    hierarchy. In the CATH databases the primary  
    levels in order of hierarchy are: Class, Architecture,  
    Topology (fold family) and Homologous superfamily.  
    In the SCOP database the primary levels in descending  
    order are: Class, Fold, Superfamily and  
    Family.

  ### Attachments

  - Briefings in Functional Genomics-2012-Greene-469-78.pdf
- ## Protein structure prediction on the Web: a case study using the Phyre server

  |  |  |
  | --- | --- |
  | Type | Journal Article |
  | Author | Lawrence A. Kelley |
  | Author | Michael JE Sternberg |
  | URL | http://www.nature.com/nprot/journal/v4/n3/abs/nprot.2009.2.html |
  | Volume | 4 |
  | Issue | 3 |
  | Pages | 363–371 |
  | Publication | Nature protocols |
  | Date | 2009 |
  | Accessed | 10/10/2013, 1:19:17 PM |
  | Library Catalog | Google Scholar |
  | Short Title | Protein structure prediction on the Web |
  | Date Added | 10/11/2013, 10:29:15 AM |
  | Modified | 10/11/2013, 10:29:15 AM |

  ### Tags:

  - likely ASTRAL domain structures
  - likely ASTRAL sequences

  ### Notes:

  - Phyre is a 3D structure prediction web server.  Since the protein folding problem is not solved, and homology modeling-based prediction is imperfect, this paper serves as a general guideline for interpreting results from homology modeling methods.  To do this, they have focused on the step-by-step procedure of their system, Phyre.

    How SCOP is used:

    Include profiles of all SCOP domains in the fold library. All structures and sequences are used, and then augmented with newer PDB data.

    SCOP reference:

    The Phyre server uses a library of known protein structures taken from the Structural Classification of Proteins (SCOP) database11 and augmented with newer depositions in the Protein Data Bank (PDB)12. The sequence of each of these structures is scanned against a nonredundant sequence database and a profile constructed and deposited in the ‘fold library’. The known and predicted secondary structure of these proteins is also stored in the fold library.

  ### Attachments

  - [PDF] from imperial.ac.uk
  - Snapshot
- ## Protein Structure Validation and Identification from Unassigned Residual Dipolar Coupling Data Using 2D-PDPA

  |  |  |
  | --- | --- |
  | Type | Journal Article |
  | Author | Arjang Fahim |
  | Author | Rishi Mukhopadhyay |
  | Author | Ryan Yandle |
  | Author | James H. Prestegard |
  | Author | Homayoun Valafar |
  | URL | http://www.mdpi.com/1420-3049/18/9/10162 |
  | Volume | 18 |
  | Issue | 9 |
  | Pages | 10162–10188 |
  | Publication | Molecules |
  | Date | 2013 |
  | Accessed | 9/20/2013, 1:16:24 PM |
  | Library Catalog | Google Scholar |
  | Date Added | 2/20/2014, 12:24:01 PM |
  | Modified | 3/7/2014, 1:06:51 PM |

  ### Notes:

  - Present 2D-PDPA method for protein structure determination.

    How SCOP is used:

    Get the count of the total number of folds in SCOP (1393).

    SCOP reference:

    SCOP [15], CATH [16], and FSSP [59] report the total number of family folds as 1393, 1233 and 2860 respectively.

  ### Attachments

  - [PDF] from mdpi.com
  - Snapshot
- ## ProtoNet 6.0: organizing 10 million protein sequences in a compact hierarchical family tree

  |  |  |
  | --- | --- |
  | Type | Journal Article |
  | Author | Nadav Rappoport |
  | Author | Solange Karsenty |
  | Author | Amos Stern |
  | Author | Nathan Linial |
  | Author | Michal Linial |
  | Volume | 40 |
  | Issue | D1 |
  | Pages | D313-D320 |
  | Publication | Nucleic Acids Research |
  | ISSN | 0305-1048 |
  | Date | JAN 2012 |
  | Extra | WOS:000298601300046 |
  | DOI | 10.1093/nar/gkr1027 |
  | Abstract | ProtoNet 6.0 (http://www.protonet.cs.huji.ac.il) is a data structure of protein families that cover the protein sequence space. These families are generated through an unsupervised bottom-up clustering algorithm. This algorithm organizes large sets of proteins in a hierarchical tree that yields high-quality protein families. The 2012 ProtoNet (Version 6.0) tree includes over 9 million proteins of which 5.5% come from UniProtKB/SwissProt and the rest from UniProtKB/TrEMBL. The hierarchical tree structure is based on an all-against-all comparison of 2.5 million representatives of UniRef50. Rigorous annotation-based quality tests prune the tree to most informative 162 088 clusters. Every high-quality cluster is assigned a ProtoName that reflects the most significant annotations of its proteins. These annotations are dominated by GO terms, UniProt/Swiss-Prot keywords and InterPro. ProtoNet 6.0 operates in a default mode. When used in the advanced mode, this data structure offers the user a view of the family tree at any desired level of resolution. Systematic comparisons with previous versions of ProtoNet are carried out. They show how our view of protein families evolves, as larger parts of the sequence space become known. ProtoNet 6.0 provides numerous tools to navigate the hierarchy of clusters. |
  | Date Added | 2/20/2014, 12:24:01 PM |
  | Modified | 3/7/2014, 12:11:18 PM |

  ### Notes:

  - Present ProtoNet database.  Protonet performs protein classification with a clustering algorithm.

    How SCOP/CATH is used:

    Assign SCOP and CATH classification, amongst others, to clusters determined by ProtoNet.

    Appears to just provide SCOP superfamily.

    SCOP/CATH reference:

    Each of the ⬚⬚162000 stable clusters was assigned a ProtoName. On average, a cluster is associated with 9.7 possible names. Most names are derived from Taxonomy (33%), UniProt (19%), GO (18%), InterPro (17%) and the rest includes information from structural classifications [e.g. SCOP (21) and CATH (22)] or ENZYME-based annotations (31).

  ### Attachments

  - Nucl. Acids Res.-2012-Rappoport-D313-20.pdf
- ## ProtoNet: charting the expanding universe of protein sequences

  |  |  |
  | --- | --- |
  | Type | Journal Article |
  | Author | Nadav Rappoport |
  | Author | Nathan Linial |
  | Author | Michal Linial |
  | Volume | 31 |
  | Issue | 4 |
  | Pages | 290-292 |
  | Publication | Nature Biotechnology |
  | ISSN | 1087-0156 |
  | Date | APR 2013 |
  | Extra | WOS:000317195500014 |
  | DOI | 10.1038/nbt.2553 |
  | Date Added | 10/28/2013, 4:53:08 PM |
  | Modified | 10/28/2013, 4:53:08 PM |
- ## PSCDB: a database for protein structural change upon ligand binding

  |  |  |
  | --- | --- |
  | Type | Journal Article |
  | Author | Takayuki Amemiya |
  | Author | Ryotaro Koike |
  | Author | Akinori Kidera |
  | Author | Motonori Ota |
  | Volume | 40 |
  | Issue | D1 |
  | Pages | D554-D558 |
  | Publication | Nucleic Acids Research |
  | ISSN | 0305-1048 |
  | Date | JAN 2012 |
  | Extra | WOS:000298601300083 |
  | DOI | 10.1093/nar/gkr966 |
  | Abstract | Proteins are flexible molecules that undergo structural changes to function. The Protein Data Bank contains multiple entries for identical proteins determined under different conditions, e.g. with and without a ligand molecule, which provides important information for understanding the structural changes related to protein functions. We gathered 839 protein structural pairs of ligand-free and ligand-bound states from monomeric or homo-dimeric proteins, and constructed the Protein Structural Change DataBase (PSCDB). In the database, we focused on whether the motions were coupled with ligand binding. As a result, the protein structural changes were classified into seven classes, i.e. coupled domain motion (59 structural changes), independent domain motion (70), coupled local motion (125), independent local motion (135), burying ligand motion (104), no significant motion (311) and other type motion (35). PSCDB provides lists of each class. On each entry page, users can view detailed information about the motion, accompanied by a morphing animation of the structural changes. PSCDB is available at http://idp1.force.cs.is.nagoya-u.ac.jp/pscdb/. |
  | Date Added | 2/20/2014, 12:24:01 PM |
  | Modified | 2/20/2014, 12:24:01 PM |

  ### Notes:

  - Present PSCDB database for protein structural change upon ligand binding.

    How SCOP is used:

    Derive a database from SCOP.

    SCOP reference:

    The representative pairs were selected according to the SCOP (structural classification of proteins) family (26), or based on clustering with 40% sequence identity.

    ...

    Future directions

    In PSCDB, the structural changes are presented only for representative proteins of the SCOP families. The repre- sentative protein was selected as the pair of ligand-free and ligand-bound structures of an identical protein with the largest RMSD value in each protein family (20).

  ### Attachments

  - Nucl. Acids Res.-2012-Amemiya-D554-8.pdf
- ## PSC: protein surface classification

  |  |  |
  | --- | --- |
  | Type | Journal Article |
  | Author | Yan Yuan Tseng |
  | Author | Wen-Hsiung Li |
  | Volume | 40 |
  | Issue | Web Server issue |
  | Pages | W435-439 |
  | Publication | Nucleic Acids Research |
  | ISSN | 1362-4962 |
  | Date | Jul 2012 |
  | Extra | PMID: 22669905 |
  | Journal Abbr | Nucleic Acids Res. |
  | DOI | 10.1093/nar/gks495 |
  | Library Catalog | NCBI PubMed |
  | Language | eng |
  | Abstract | We recently proposed to classify proteins by their functional surfaces. Using the structural attributes of functional surfaces, we inferred the pairwise relationships of proteins and constructed an expandable database of protein surface classification (PSC). As the functional surface(s) of a protein is the local region where the protein performs its function, our classification may reflect the functional relationships among proteins. Currently, PSC contains a library of 1974 surface types that include 25,857 functional surfaces identified from 24,170 bound structures. The search tool in PSC empowers users to explore related surfaces that share similar local structures and core functions. Each functional surface is characterized by structural attributes, which are geometric, physicochemical or evolutionary features. The attributes have been normalized as descriptors and integrated to produce a profile for each functional surface in PSC. In addition, binding ligands are recorded for comparisons among homologs. PSC allows users to exploit related binding surfaces to reveal the changes in functionally important residues on homologs that have led to functional divergence during evolution. The substitutions at the key residues of a spatial pattern may determine the functional evolution of a protein. In PSC (http://pocket.uchicago.edu/psc/), a pool of changes in residues on similar functional surfaces is provided. |
  | Short Title | PSC |
  | Date Added | 10/11/2013, 10:20:13 AM |
  | Modified | 3/7/2014, 12:10:57 PM |

  ### Tags:

  - Alcohol Dehydrogenase
  - Cluster Analysis
  - Humans
  - Internet
  - Proteins
  - Software
  - Structural Homology, Protein
  - Surface Properties

  ### Notes:

  - The PSC web server aims to classify proteins based on their functional surfaces.

    How SCOP is used:

    Noted in the introduction for background information, just as an example of a database that classifies proteins based on structure and homology. However, the paper goes on to say that proteins may have diverged enough that homology isn't evident by structure.

    How CATH is used:

    Annotate proteins in data set of complexes downloaded from the PDB with their CATH identifiers.  This data is presented to the user.

     SCOP reference:

    Well-known classifications, such as Pfam (1),  
    COG (2), structural classification of proteins (SCOP) (3)  
    and class, architecture, topology, homologous superfamily  
    (CATH) (4) have provided biological insights into protein  
    structure, function and evolution. However, two proteins  
    may have diverged so much, such that their homology is  
    no longer evident at the sequence or global structural  
    level, making it challenging to decide if the two proteins  
    are functionally related. This underscores the importance  
    of identifying local structural regions that are well  
    conserved in evolution (5,6).

    CATH reference:

    For example, oxophytodienoate reductase and NADPH dehydrogenase have the same fold identifi- cation of CATH 3.20.20.70 (Aldolase class I). However, their Enzyme Commission (EC) annotations are EC 1.3.1.42 and EC 1.6.99.1, so they actually have different enzymatic functions.

    ...

    SC LIBARAY AND DATA ACCESS

    The PSC database was constructed as follows. First, we collected the bound structures from 24170 entries of Protein Data Bank (PDB) (13), which included a total of 25857 chains. Then, using an automated pipeline, we identified the binding surfaces of each bound form (9,14) and calculated their geometric measurements, including the composition of a spatial pattern, solvent accessible area and molecular volume. In addition, we provided bio- logical annotations via cross-links to UniProt (15). Enzyme annotations from EC (16) and fold terms from CATH are provided. We also allow users to access all putative binding surfaces along with their corresponding evolutionary conservation and geometric measurements. Most importantly, structurally similar or functionally related binding surfaces across species are associated with each other and characterized by structural attributes.

    ...

    Finally, a comparison of surface members with EC annota- tions and CATH identifications allows users to gain struc- tural insights into the relationship between shape and function.

  ### Attachments

  - Nucl. Acids Res.-2012-Tseng-W435-9.pdf
- ## Pseudomonas aeruginosa PA1006 is a Persulfide-Modified Protein that is Critical for Molybdenum Homeostasis

  |  |  |
  | --- | --- |
  | Type | Journal Article |
  | Author | Gregory Tombline |
  | Author | Johanna M. Schwingel |
  | Author | John D. Lapek Jr |
  | Author | Alan E. Friedman |
  | Author | Thomas Darrah |
  | Author | Michael Maguire |
  | Author | Nadine E. Van Alst |
  | Author | Melanie J. Filiatrault |
  | Author | Barbara H. Iglewski |
  | URL | http://dx.plos.org/10.1371/journal.pone.0055593 |
  | Volume | 8 |
  | Issue | 2 |
  | Pages | e55593 |
  | Publication | PloS one |
  | Date | 2013 |
  | Accessed | 9/20/2013, 1:16:24 PM |
  | Library Catalog | Google Scholar |
  | Date Added | 10/11/2013, 10:29:15 AM |
  | Modified | 10/11/2013, 10:29:15 AM |

  ### Notes:

  - Experimental study of structure and function of Pae PA1006 protein.

    How SCOP is used:

    Look up fold of an ortholog of PA1006 protein, E. coli YhhP/TusA protein.  List other superfamilies with the same fold.

    SCOP reference:

    The structure of the E. coli YhhP/TusA protein was determined by NMR [12] and the Structural Classification of Protein (SCOP) database [13,14] classifies the YhhP/TusA structure as displaying an IF3-like fold (translation Initiation Factor-3) consisting of two alpha helices that rest upon four beta strands (beta-alpha-beta-alpha-beta2). This fold occurs in several protein superfamilies including the C-terminal domains of IF3 and ProRS, YhbY, SirA, AlbA, RH3, and EPT/RTPC proteins.

  ### Attachments

  - [HTML] from plos.org
  - journal.pone.0055593.pdf
- ## PSimScan: Algorithm and Utility for Fast Protein Similarity Search

  |  |  |
  | --- | --- |
  | Type | Journal Article |
  | Author | Anna Kaznadzey |
  | Author | Natalia Alexandrova |
  | Author | Vladimir Novichkov |
  | Author | Denis Kaznadzey |
  | Volume | 8 |
  | Issue | 3 |
  | Publication | PLOS ONE |
  | ISSN | 1932-6203 |
  | Date | MAR 7 2013 |
  | DOI | 10.1371/journal.pone.0058505 |
  | Language | English |
  | Abstract | In the era of metagenomics and diagnostics sequencing, the importance of protein comparison methods of boosted performance cannot be overstated. Here we present PSimScan (Protein Similarity Scanner), a flexible open source protein similarity search tool which provides a significant gain in speed compared to BLASTP at the price of controlled sensitivity loss. The PSimScan algorithm introduces a number of novel performance optimization methods that can be further used by the community to improve the speed and lower hardware requirements of bioinformatics software. The optimization starts at the lookup table construction, then the initial lookup table-based hits are passed through a pipeline of filtering and aggregation routines of increasing computational complexity. The first step in this pipeline is a novel algorithm that builds and selects `similarity zones' aggregated from neighboring matches on small arrays of adjacent diagonals. PSimScan performs 5 to 100 times faster than the standard NCBI BLASTP, depending on chosen parameters, and runs on commodity hardware. Its sensitivity and selectivity at the slowest settings are comparable to the NCBI BLASTP's and decrease with the increase of speed, yet stay at the levels reasonable for many tasks. PSimScan is most advantageous when used on large collections of query sequences. Comparing the entire proteome of Streptocuccus pneumoniae (2,042 proteins) to the NCBI's non-redundant protein database of 16,971,855 records takes 6.5 hours on a moderately powerful PC, while the same task with the NCBI BLASTP takes over 66 hours. We describe innovations in the PSimScan algorithm in considerable detail to encourage bioinformaticians to improve on the tool and to use the innovations in their own software development. |
  | Date Added | 10/25/2013, 4:17:08 PM |
  | Modified | 10/25/2013, 4:17:08 PM |

  ### Notes:

  - Present PSimScan homology detection method.

    How SCOP is used:

    Train and validate against SCOP/ASTRAL data.  Use fold classification.

    SCOP reference:

    Test Arrangement

    The following sequence sets were used:

    PDB90 subset of the Astral SCOP database ver.1.75, containing 15545 sequences of the total length of 2683934 amino acid residues, classified into 3901 unique folds.

    ...

    Figure S1 Selectivity and Sensitivity of PSimScan at different parameters versus other similarity search tools, calculated on a normalized database. All proteins from a subset of the PDB90 database with balanced representation of protein families were compared with each other using PSimScan, SSEARCH, BLAST, USEARCH, RAPSearch and BLAT. PSimScan was tested at different combinations of kthresh (similarity zone detection threshold) and approx (tuple diversifica- tion level) parameters. For SSEARCH, BLAST, USEARCH, RAPSearch and BLAT, the Coverage vs Error graphs were plotted as described by Brenner et al [47]. Similarities between proteins of the same SCOP fold were treated as true positives, while similarities between proteins of different folds – as false positives (errors). The

    ...

  ### Attachments

  - journal.pone.0058505.pdf
- ## PTS phosphorylation of Mga modulates regulon expression and virulence in the group A streptococcus

  |  |  |
  | --- | --- |
  | Type | Journal Article |
  | Author | Elise R. Hondorp |
  | Author | Sherry C. Hou |
  | Author | Lara L. Hause |
  | Author | Kanika Gera |
  | Author | Ching-En Lee |
  | Author | Kevin S. McIver |
  | Volume | 88 |
  | Issue | 6 |
  | Pages | 1176-1193 |
  | Publication | Molecular Microbiology |
  | ISSN | 0950-382X |
  | Date | JUN 2013 |
  | Extra | WOS:000320174300012 |
  | DOI | 10.1111/mmi.12250 |
  | Abstract | The ability of a bacterial pathogen to monitor available carbon sources in host tissues provides a clear fitness advantage. In the group A streptococcus (GAS), the virulence regulator Mga contains homology to phosphotransferase system (PTS) regulatory domains (PRDs) found in sugar operon regulators. Here we show that Mga was phosphorylated in vitro by the PTS components EI/HPr at conserved PRD histidines. A ptsI (EI-deficient) GAS mutant exhibited decreased Mga activity. However, PTS-mediated phosphorylation inhibited Mga-dependent transcription of emmin vitro. Using alanine (unphosphorylated) and aspartate (phosphomimetic) mutations of PRD histidines, we establish that a doubly phosphorylated PRD1 phosphomimetic (D/DMga4) is completely inactive in vivo, shutting down expression of the Mga regulon. Although D/DMga4 is still able to bind DNAin vitro, homo-multimerization of Mga is disrupted and the protein is unable to activate transcription. PTS-mediated regulation of Mga activity appears to be important for pathogenesis, as bacteria expressing either non-phosphorylated (A/A) or phosphomimetic (D/D) PRD1 Mga mutants were attenuated in a model of GAS invasive skin disease. Thus, PTS-mediated phosphorylation of Mga may allow the bacteria to modulate virulence gene expression in response to carbohydrate status. Furthermore, PRD-containing virulence regulators (PCVRs) appear to be widespread in Gram-positive pathogens. |
  | Date Added | 10/28/2013, 4:53:08 PM |
  | Modified | 10/28/2013, 4:53:08 PM |

  ### Notes:

  - Experimental and computational study of virulence regulator Mga in group A streptococcus.

    How SCOP is used:

    Search for 'structural homologs' in SCOP (this work was done previously).

    SCOP reference:

    Results

    Mga shares homology to PRD-containing regulators

    To identify structural homologues to domains within Mga, we previously undertook an in silico analysis to compare

    Mga to proteins of known structure in the SCOP database (Andreeva et al., 2004; Deutscher et al., 2005; Hondorp and McIver, 2007). The central region of Mga was pre- dicted to have strong structural homology to PTS regula- tory domains (PRDs) in the B. subtilis antiterminator LicT, the only PRD-containing protein for which a structure had been determined (Deutscher et al., 2005; Hondorp and McIver, 2007).

  ### Attachments

  - mmi12250.pdf
- ## PUTRACER: A NOVEL METHOD FOR IDENTIFICATION OF CONTINUOUS-DOMAINS IN MULTI-DOMAIN PROTEINS

  |  |  |
  | --- | --- |
  | Type | Journal Article |
  | Author | Seyed Shahriar Arab |
  | Author | Mohammadbagher Parsa Gharamaleki |
  | Author | Zaiddodine Pashandi |
  | Author | Rezvan Mobasseri |
  | URL | http://www.worldscientific.com/doi/abs/10.1142/S021972001340012X |
  | Volume | 11 |
  | Issue | 01 |
  | Publication | Journal of bioinformatics and computational biology |
  | Date | 2013 |
  | Accessed | 9/23/2013, 10:14:00 AM |
  | Library Catalog | Google Scholar |
  | Short Title | PUTRACER |
  | Date Added | 10/11/2013, 10:29:15 AM |
  | Modified | 3/7/2014, 12:10:19 PM |

  ### Tags:

  - likely ASTRAL

  ### Notes:

  - Present Protein Unit Tracer (PUTracer) method for domain prediction in multi-domain proteins using sequence and structural features.

    How SCOP/CATH is used:

    Validate domain prediction method on a benchmark dataset derived from SCOP and CATH (Balanced Domain Benchmark 2).

    SCOP/CATH references:

    Performance of the program was assessed by a comprehensive benchmark dataset of 124 protein chains, which is based on agreement among experts (e.g. CATH, SCOP) and was expanded to include structures with di®erent types of domain combinations.

    3.1. Dataset properties

    Since \55 chain" dataset19 and the one introduced by Islam18 seem to have bias to 1- domain proteins, Balanced Domain Benchmark 2 was selected.20 This benchmark includes proteins in agreement with domain assignment by three expert methods: SCOP,21 CATH22 and AUTHORS.18 Moreover, this benchmark was used by other automatic methods for domain partitioning such as PDP,11 DomainParser,6 NCBI,23 DALI,24 and PUU.5 Therefore, the comparison of PUTracer with others can be feasible.

  ### Attachments

  - s021972001340012x.pdf
- ## Quality assessment of protein model-structures based on structural and functional similarities

  |  |  |
  | --- | --- |
  | Type | Journal Article |
  | Author | Bogumil M. Konopka |
  | Author | Jean-Christophe Nebel |
  | Author | Malgorzata Kotulska |
  | URL | http://www.biomedcentral.com/1471-2105/13/242/ |
  | Volume | 13 |
  | Issue | 1 |
  | Pages | 242 |
  | Publication | BMC bioinformatics |
  | Date | 2012 |
  | Accessed | 9/23/2013, 10:18:03 AM |
  | Library Catalog | Google Scholar |
  | Date Added | 10/11/2013, 10:29:15 AM |
  | Modified | 10/11/2013, 10:29:15 AM |

  ### Tags:

  - Interesting
  - likely ASTRAL
  - likely ASTRAL domain structures
  - likely ASTRAL sequences
  - likely ASTRAL subsets

  ### Notes:

  - Evaluate GOBA - Gene Ontology-Based Assessment for protein model quality assessment.  GOBA makes the assumption that a high quality model is structurally similar to proteins that are functionally similar to the prediction target.

    How SCOP is used:

    Use SCOP classification to validate their hypothesis that proteins which are more functionally similar tend to be more structurally similar.  Used representative set of 5901 structures from SCOP.  Computed and compared Dali Z-score and Functional Similarity score for each pair.

    Seem to actually be using ASTRAL, but ASTRAL was not cited.

    SCOP references:

    This is also supported by the fact that the number of known unique folds (as defined by SCOP [30]) from Protein Data Bank (PDB) [31] equals 1393 (as of 11.2011), while the number of all non-redundant structures (below 30% of sequence similarity) is 18,132. The ratio leads to the conclusion that individ- ual domains of different proteins can adopt very similar shapes.

    ...

    Results

    The concept underlying our method was validated using a representative set of protein native structures, while the accuracy of model quality predictions was tested using model-structures of protein targets issued and assessed in the CASP8 and CASP9 contests (see Methods for a full description of datasets). In addition, comparisons were conducted with all MQAPs that took part in the CASP8 and CASP9 events.

    Functional and structural similarities

    Our method is based on the assumption that there is a good correlation between similarity metrics of protein structure and function. This relationship was investigated on a representative, non-redundant set of 5901 native structures from SCOP database [30]. Dali Z-scores of Structural Neighbors (SNs, see Methods) of each protein were plotted against their corresponding Functional Similarity (FS) scores (Figure 1). Over 700,000 protein pairs were compared.

  ### Attachments

  - 1471-2105-13-242.pdf
  - [HTML] from biomedcentral.com
- ## Quantifying protein modularity and evolvability: A comparison of different techniques

  |  |  |
  | --- | --- |
  | Type | Journal Article |
  | Author | Mary Rorick |
  | URL | http://www.sciencedirect.com/science/article/pii/S0303264712001232 |
  | Publication | Biosystems |
  | Date | 2012 |
  | Accessed | 9/23/2013, 10:23:40 AM |
  | Library Catalog | Google Scholar |
  | Short Title | Quantifying protein modularity and evolvability |
  | Date Added | 10/11/2013, 10:29:15 AM |
  | Modified | 3/7/2014, 12:11:15 PM |

  ### Tags:

  - Coevolution
  - Evolvability
  - Modularity
  - protein evolution
  - Protein module
  - Robustness

  ### Notes:

  - Review examines different techniques in protein research (particularly the modularity and evolution of proteins) and its future uses - engineering new proteins, new models for evolution, etc.

    How SCOP/CATH is used:

    Not using SCOP or CATH data.

    Just listed as a database that organizes protein domains.

    SCOP reference:

    Where domains are identified with strictly geometric  
    or topographical requirements (e.g., Go, 1981), domain identification  
    methods can be classified as relying on suppositional criteria.  
    In practice, however, domains are more commonly identified as  
    regions of conserved structure existing in different protein contexts  
    (e.g., SCOP, CATH, PDP, SMART and PFAM (Alexandrov and  
    Shindyalov, 2003; Andreeva et al., 2004; Copley et al., 2002; Lo  
    Conte et al., 2002; Murzin et al., 1995; Pearl et al., 2003; Xu et al.,  
    2000)).

  ### Attachments

  - 1-s2.0-S0303264712001232-main.pdf
- ## Raman spectroscopy of proteins: a review

  |  |  |
  | --- | --- |
  | Type | Journal Article |
  | Author | A. Rygula |
  | Author | K. Majzner |
  | Author | K. M. Marzec |
  | Author | A. Kaczor |
  | Author | M. Pilarczyk |
  | Author | M. Baranska |
  | URL | http://onlinelibrary.wiley.com/doi/10.1002/jrs.4335/full |
  | Publication | Journal of Raman Spectroscopy |
  | Date | 2013 |
  | Accessed | 9/23/2013, 10:21:39 AM |
  | Library Catalog | Google Scholar |
  | Short Title | Raman spectroscopy of proteins |
  | Date Added | 2/20/2014, 12:24:01 PM |
  | Modified | 2/20/2014, 12:24:01 PM |

  ### Notes:

  - Experimental study of 26 protein structures using Raman spectroscopy.

    How SCOP is used:

    Annotate a non-SCOP data set with SCOP structural class.

    SCOP reference:

    In this work, 26 proteins of different structure, function and properties are investigated by Raman spectroscopy with 488, 532 and 1064 nm laser lines. To discuss their spectral properties, proteins were divided, according to the Structural Classification of Proteins (SCOP),[13] into four classes according to their second- ary structure, i.e. a-helical (a), b-sheet (b), mixed structures (a/b, a+b, s) and others. Several reviews (e.g.[3,6,14]) showing the potential of Raman (and IR, e.g.[15]) spectroscopy for the measure- ment and analysis of proteins have been published; however, to the best of our knowledge, such a large collection of individual protein Raman spectra was not offered in any paper. This work can serve as a review and comprehensive vibrational spectra library, based on our and previous Raman measurements, with detailed analysis.

  ### Attachments

  - jrs4335.pdf
- ## Rampant Exchange of the Structure and Function of Extramembrane Domains between Membrane and Water Soluble Proteins

  |  |  |
  | --- | --- |
  | Type | Journal Article |
  | Author | Hyun-Jun Nam |
  | Author | Seong Kyu Han |
  | Author | James U. Bowie |
  | Author | Sanguk Kim |
  | Volume | 9 |
  | Issue | 3 |
  | Pages | e1002997 |
  | Publication | Plos Computational Biology |
  | ISSN | 1553-7358 |
  | Date | MAR 2013 |
  | Extra | WOS:000316864200063 |
  | DOI | 10.1371/journal.pcbi.1002997 |
  | Abstract | Of the membrane proteins of known structure, we found that a remarkable 67% of the water soluble domains are structurally similar to water soluble proteins of known structure. Moreover, 41% of known water soluble protein structures share a domain with an already known membrane protein structure. We also found that functional residues are frequently conserved between extramembrane domains of membrane and soluble proteins that share structural similarity. These results suggest membrane and soluble proteins readily exchange domains and their attendant functionalities. The exchanges between membrane and soluble proteins are particularly frequent in eukaryotes, indicating that this is an important mechanism for increasing functional complexity. The high level of structural overlap between the two classes of proteins provides an opportunity to employ the extensive information on soluble proteins to illuminate membrane protein structure and function, for which much less is known. To this end, we employed structure guided sequence alignment to elucidate the functions of membrane proteins in the human genome. Our results bridge the gap of fold space between membrane and water soluble proteins and provide a resource for the prediction of membrane protein function. A database of predicted structural and functional relationships for proteins in the human genome is provided at sbi.postech.ac.kr/emdmp. |
  | Date Added | 2/20/2014, 12:24:01 PM |
  | Modified | 2/20/2014, 12:24:01 PM |

  ### Notes:

  - Computational study of "domain sharing between membrane and soluble proteins": the predominance of membrane and soluble homologs.

    How SCOP is used:

    Study whether any of the structural classes are more likely to contain soluble/membrane homologs.  Annotate a non-SCOP data set of 558 membrane and ~44000 soluble proteins with class and fold.

    SCOP reference:

    The structural relatives do not appear to be restricted to any particular type of fold as they span many SCOP classes, including all alpha, all beta, alpha+beta and alpha/beta classes (Figure 2). The aligned pairs share 352 different fold types (Table S1) which is roughly a quarter of the 1,200 total fold types in SCOP [9]. These results indicate that diverse fold types performing various biological functions are shared between membrane and soluble proteins.

    ...

    We examined how frequently shared domains between mem- brane and soluble proteins were found from same SCOP folds. Of 87 structurally similar domains, 60 (68.9%) extramembrane domains and soluble protein domain shared same SCOP folds, whereas 27 (31.1%) domains appeared in different SCOP folds (Figure S9A and Table S5). The number of fold types annotated for membrane proteins is much smaller than that of soluble proteins (Figure S9B). Specifically, structural pairs that share same SCOP fold were usually found from the extramembrane regions of membrane proteins. Meanwhile, structural pairs with different SCOP folds were mostly found from fold annotations assigned to whole membrane protein structures including both transmem- brane and extramembrane regions.

    ...

    Materials and Methods

    Data sets of membrane and soluble protein structures

    We collected 558 membrane and 43547 soluble protein structures from the PDB library [29]. We included only structures solved by X-ray and NMR, and excluded structures solved by EM (electron microscopy and cryo-electron diffraction), Fiber (fiber diffraction), IR (infrared spectroscopy), Model (predicted models), Neutron (neutron diffraction). Only experimentally confirmed membrane protein structures from the SwissProt and PDB databases were included. Proteins annotated as single-/multi-pass membrane proteins or membrane proteins were included, but peripheral membrane proteins were excluded. We collected soluble protein structures by excluding membrane proteins and putative membrane proteins. The SCOP database (release 1.75) was used to examine the fold and class diversity of structures. The current SCOP database lists only 58 folds of membrane proteins, whereas more than 1000 folds are listed for soluble proteins.

    ...

    Class, fold and domain identification of aligned structural pairs

    We classified structurally similar membrane and soluble proteins into four classes; all alpha, all beta, alpha+beta, and alpha/beta based on SCOP classifications [9]. SCOP database is a comprehensive ordering of all proteins of known structures according to their structural relationships. Because structural information of membrane proteins is lacking, we utilized class information of soluble proteins to identify the class of structurally aligned membrane and soluble protein pairs. We used the domain information from the SCOP database to assign domain bound- aries of the structurally aligned regions of membrane and soluble proteins. We assigned a domain annotation if an aligned region covered more than the 90% of domain length.

    ...

  ### Attachments

  - journal.pcbi.1002997.pdf
- ## Random field model reveals structure of the protein recombinational landscape

  |  |  |
  | --- | --- |
  | Type | Journal Article |
  | Author | Philip A. Romero |
  | Author | Frances H. Arnold |
  | URL | http://dx.plos.org/10.1371/journal.pcbi.1002713 |
  | Volume | 8 |
  | Issue | 10 |
  | Pages | e1002713 |
  | Publication | PLoS computational biology |
  | Date | 2012 |
  | Accessed | 9/20/2013, 1:18:03 PM |
  | Library Catalog | Google Scholar |
  | Date Added | 10/11/2013, 10:29:15 AM |
  | Modified | 10/11/2013, 10:29:15 AM |

  ### Notes:

  - Present and study a model of protein recombination.

    How SCOP is used:

    The authors' lab has constructed and tested 8 recombination libraries from various protein families.  They present summary statistics on their libraries and use SCOP to retrieve the fold class (i.e. all-alpha, alpha+beta, etc).

    SCOP reference:

    (In table legend:)

    "The fold class was retrieved from the SCOP structural database [53]. The fraction of functional sequences and additivity were calculated as described in Methods."

  ### Attachments

  - [HTML] from plos.org
  - journal.pcbi.1002713.pdf

    SCOP reference:

    In table legend:

    "The fold class was retrieved from the SCOP structural database [53]. The fraction of functional sequences and additivity were calculated as described in Methods."
  - PubMed entry
- ## Random Matrix Theory Analysis of Cross Correlations in Molecular Dynamics Simulations of Macro-Biomolecules

  |  |  |
  | --- | --- |
  | Type | Journal Article |
  | Author | Masanori Yamanaka |
  | Volume | 82 |
  | Issue | 8 |
  | Publication | JOURNAL OF THE PHYSICAL SOCIETY OF JAPAN |
  | ISSN | 0031-9015 |
  | Date | August 2013 |
  | DOI | 10.7566/JPSJ.82.083801 |
  | Language | English |
  | Abstract | We apply the random matrix theory to analyze the molecular dynamics simulation of macromolecules, such as proteins. The eigensystem of the cross-correlation matrix for the time series of the atomic coordinates is analyzed. We study a data set with seven different sampling intervals to observe the characteristic motion at each time scale. In all cases, the unfolded eigenvalue spacings are in agreement with the predictions of random matrix theory. In the short-time scale, the cross-correlation matrix has the universal properties of the Gaussian orthogonal ensemble. The eigenvalue distribution and inverse participation ratio have a crossover behavior between the universal and nonuniversal classes, which is distinct from the known results such as the financial time series. Analyzing the inverse participation ratio, we extract the correlated cluster of atoms and decompose it to subclusters. |
  | Date Added | 10/11/2013, 10:29:15 AM |
  | Modified | 3/7/2014, 1:06:35 PM |

  ### Tags:

  - biomolecules
  - cross correlation matrix
  - domain
  - gaussian orthogonal ensemble
  - level statistics
  - molecular dynamics
  - Protein
  - proteomics
  - random matrix

  ### Notes:

  - Present "random matrix theory analysis method" which examines molecular dynamics data.  Apply to a data set of molecular dynamics data.  Present method as a possible way to classify proteins by dynamics, as opposed to 3D static structures.

    How SCOP/CATH is used:

    Not using SCOP or CATH data.

    SCOP reference:

    Knowledge of the structure and function of the proteome is central to the exploitation of the wealth of biological information available in the postgenomic era. Classification of the three-dimensional structures of protein is one of the central issues in molecular biology. Protein structures can be classified in terms of their similarity or a common evolutionary origin. CATH,1) FSSP,2) PFAM,3) and SCOP4) are well-known databases. These classifications are based on the static properties of the molecular structures obtained by the X-ray crystallographic or nuclear magnetic resonance analysis. Recently, research on the dynamical properties of biomolecules has attracted considerable attention. It is important for understanding organisms and drug discovery. Principal component analysis (PCA) is a well-known method of studying the fluctuation from the static properties and classifying the structures into some groups.5) However, fully understanding the dynamical properties is still difficult in general.

  ### Attachments

  - jpsj%2E82%2E083801.pdf
- ## RAPHAEL: recognition, periodicity and insertion assignment of solenoid protein structures

  |  |  |
  | --- | --- |
  | Type | Journal Article |
  | Author | Ian Walsh |
  | Author | Francesco G. Sirocco |
  | Author | Giovanni Minervini |
  | Author | Tomas Di Domenico |
  | Author | Carlo Ferrari |
  | Author | Silvio C. E. Tosatto |
  | Volume | 28 |
  | Issue | 24 |
  | Pages | 3257–3264 |
  | Publication | Bioinformatics |
  | Date | December 2012 |
  | DOI | 10.1093/bioinformatics/bts550 |
  | Abstract | MOTIVATION: Repeat proteins form a distinct class of structures where folding is greatly simplified. Several classes have been defined, with solenoid repeats of periodicity between ca. 5 and 40 being the most challenging to detect. Such proteins evolve quickly and their periodicity may be rapidly hidden at sequence level. From a structural point of view, finding solenoids may be complicated by the presence of insertions or multiple domains. To the best of our knowledge, no automated methods are available to characterize solenoid repeats from structure. RESULTS: Here we introduce RAPHAEL, a novel method for the detection of solenoids in protein structures. It reliably solves three problems of increasing difficulty: (1) recognition of solenoid domains, (2) determination of their periodicity and (3) assignment of insertions. RAPHAEL uses a geometric approach mimicking manual classification, producing several numeric parameters that are optimized for maximum performance. The resulting method is very accurate, with 89.5% of solenoid proteins and 97.2% of non-solenoid proteins correctly classified. RAPHAEL periodicities have a Spearman correlation coefficient of 0.877 against the manually established ones. A baseline algorithm for insertion detection in identified solenoids has a Q(2) value of 79.8%, suggesting room for further improvement. RAPHAEL finds 1931 highly confident repeat structures not previously annotated as solenoids in the Protein Data Bank records. |
  | Date Added | 3/7/2014, 12:08:00 PM |
  | Modified | 3/7/2014, 12:08:00 PM |
- ## Rapid catalytic template searching as an enzyme function prediction procedure

  |  |  |
  | --- | --- |
  | Type | Journal Article |
  | Author | Jerome P. Nilmeier |
  | Author | Daniel A. Kirshner |
  | Author | Sergio E. Wong |
  | Author | Felice C. Lightstone |
  | URL | http://dx.plos.org/10.1371/journal.pone.0062535 |
  | Volume | 8 |
  | Issue | 5 |
  | Pages | e62535 |
  | Publication | PloS one |
  | Date | 2013 |
  | Accessed | 9/23/2013, 10:15:34 AM |
  | Library Catalog | Google Scholar |
  | Date Added | 10/11/2013, 10:29:15 AM |
  | Modified | 10/11/2013, 10:29:15 AM |

  ### Notes:

  - Present an enzyme protein function prediction algorithm, Catalytic Site Identification (CatSId), based on identification of catalytic residues.

    How SCOP is used:

    Mentioned in background as a database with "enhanced annotation" for a sequence of interest that can be used to infer function.

    SCOP reference:

    One approach is to infer function by focusing on global sequence or structural similarity. Global structural alignment procedures, e.g. LGA [2], PINTS [3,4], and CE [3,4], and sequence annotation approaches that indicate a structural or functional context, e.g. SCOP [5], CATH [6], GO [7,8], or KEGG [9], successfully provide an enhanced annotation of the sequence of interest. I

  ### Attachments

  - [HTML] from plos.org
  - journal.pone.0062535.pdf
- ## Rebelling for a Reason: Protein Structural "Outliers''

  |  |  |
  | --- | --- |
  | Type | Journal Article |
  | Author | Gandhimathi Arumugam |
  | Author | Anu G. Nair |
  | Author | Sridhar Hariharaputran |
  | Author | Sowdhamini Ramanathan |
  | Volume | 8 |
  | Issue | 9 |
  | Publication | Plos One |
  | ISSN | 1932-6203 |
  | Date | SEP 20 2013 |
  | Extra | WOS:000324768000018 |
  | DOI | 10.1371/journal.pone.0074416 |
  | Abstract | Analysis of structural variation in domain superfamilies can reveal constraints in protein evolution which aids protein structure prediction and classification. Structure-based sequence alignment of distantly related proteins, organized in PASS2 database, provides clues about structurally conserved regions among different functional families. Some superfamily members show large structural differences which are functionally relevant. This paper analyses the impact of structural divergence on function for multi-member superfamilies, selected from the PASS2 superfamily alignment database. Functional annotations within superfamilies, with structural outliers or 'rebels', are discussed in the context of structural variations. Overall, these data reinforce the idea that functional similarities cannot be extrapolated from mere structural conservation. The implication for fold-function prediction is that the functional annotations can only be inherited with very careful consideration, especially at low sequence identities. |
  | Date Added | 10/28/2013, 4:53:08 PM |
  | Modified | 10/8/2014, 12:50:47 PM |

  ### Notes:

  - Computational study of "structural outliers" in SCOP superfamilies.  Use structural alignments from PASS2 database to determine outliers.

    How SCOP is used:

    Examine PASS2 alignments of all multi-membered superfamilies to detect structural outliers.

    How CATH is used:

    Background on protein structure classification.

    SCOP reference:

    Structure-based Sequence Alignment of Superfamily Domains

    PASS2 [28] database contains structure-based sequence align- ment of protein domain superfamilies in correspondence with SCOP 1.75. A PASS2 superfamily is a subset of corresponding SCOP superfamily, with no member sharing more than 40% sequence identity with any of the other members. We have mainly focused on multi-member superfamily (MMS; which implies multiple number of superfamily members) with ,40% identity with other domains in the superfamily.

    ...

    Structurally Deviant Members of PASS2

    Here, we emphasize that using an appropriate structure alignment protocol even on protein domains with low sequence identity, one can identify structural differences which occur due to a functional reason. After the structural alignment of 731 multi- membered superfamilies, 159 superfamilies show one or more structurally deviant members within the superfamily. Figure 1 shows the total multi-member superfamilies and superfamilies having outliers, grouped according to structural class. These outliers generally exhibit high RMSD .5.5 and they are again confirmed by visual inspection.

  ### Attachments

  - journal.pone.0074416.pdf
- ## Recognition Rules for Binding of Homeodomains to Operator DNA

  |  |  |
  | --- | --- |
  | Type | Journal Article |
  | Author | Yu N. Chirgadze |
  | Author | V. S. Sivozhelezov |
  | Author | R. V. Polozov |
  | Author | V. A. Stepanenko |
  | Author | V. V. Ivanov |
  | URL | http://www.tandfonline.com/doi/full/10.1080/073911012010525019 |
  | Volume | 29 |
  | Issue | 4 |
  | Pages | 715–731 |
  | Publication | Journal of Biomolecular Structure and Dynamics |
  | Date | 2012 |
  | Accessed | 9/23/2013, 10:20:20 AM |
  | Library Catalog | Google Scholar |
  | Date Added | 10/11/2013, 10:29:15 AM |
  | Modified | 10/11/2013, 10:29:15 AM |

  ### Tags:

  - Binding interface
  - Homeodomain family
  - Invariant contacts
  - Protein-DNA recognition
  - transcription factor
  - Variable contacts

  ### Notes:

  - Computational study of protein-dna interfaces.

    How SCOP is used:

    To gather statistics on the number of SCOP families containing protein-dna complexes.

    SCOP reference:

    There are about two thousand protein-DNA complexes of known 3D structure which belong to 207 families, as reported in the SCOP database (37).

  ### Attachments

  - [PDF] from jbsdonline.com
- ## Recurrent Structural Motifs in Non-Homologous Protein Structures

  |  |  |
  | --- | --- |
  | Type | Journal Article |
  | Author | Maria U. Johansson |
  | Author | Vincent Zoete |
  | Author | Nicolas Guex |
  | URL | http://www.mdpi.com/1422-0067/14/4/7795/pdf |
  | Volume | 14 |
  | Issue | 4 |
  | Pages | 7795–7814 |
  | Publication | International journal of molecular sciences |
  | Date | 2013 |
  | Accessed | 9/23/2013, 10:15:34 AM |
  | Library Catalog | Google Scholar |
  | Date Added | 10/11/2013, 10:29:15 AM |
  | Modified | 3/7/2014, 12:09:31 PM |

  ### Tags:

  - Delaunay triangulation
  - long-range contacts
  - protein folding
  - protein fragments
  - protein structure
  - Structural motifs
  - structure comparison/similarity
  - structure prediction

  ### Notes:

  - Computational study analyzes recurrent structural motifs by their amino acid composition in relation to their stability (calculate the free folding energy). They examine the changes amongst alanine mutations as well (higher free folding energy). It was found that the highest energy was found in motifs with Phe, Ile, Leu, Val, Tyr, Met, and Trp.

    How SCOP s used:

    First, mention that an advantage of their method is that it does not rely on a multi-level fold classification.

    Use SCOP to describe distribution of particular motifs detected in their study, for 3 case study examples.  For example, mention a particular motif is found in "36 different SCOP families (and 33 superfamilies, 32 folds and four classes)".

    How CATH is used:

    Do not use CATH data.  Only cite as an example database.

    **SCOP Reference**

    Furthermore,  
    given the nature of our motifs, a precise multi-level fold classification, such as Structural Classification  
    of Proteins (SCOP) [38] or CATH [39], was not required, and to report our results, protein chains were  
    instead classified as belonging to exactly one of the four categories: helix, strand, helix-strand mixture  
    and “other”, depending on the fraction of residues in the corresponding secondary structure  
    conformation in each protein chain, as described in Section 3.2.

    The RSM shown in Figure 2 is the  
    one with the highest value of recurrence among all our RSMs. This motif is found in chains classified  
    into 36 different SCOP families (and 33 superfamilies, 32 folds and four classes). However, only 33%  
    of the supporting chains have a SCOP classification, but it is nonetheless clear that it is a very widely  
    spread structural motif. The twist of the beta-sheet in Figure 2 is caused by the interactions between  
    the side chains of the valines; this was first described by Chou and Scheraga [42].

    For 2dbs-A, the similar structure is 1a79 (max sequence identity: 8%, RMSD 3.16 Å) and  
    for 2in5-A, the similar structure is 3fzx (max sequence identity: 9%, RMSD 3.29 Å). Both 2dbs-A and  
    2in5-A belong to the New Fold category in SCOP.  
    Both structures are covered by RSMs (Figure 9). In total, 2dbs-A is covered by 35 RSMs and  
    2in5-A is covered by 82 RSMs. The coverage of 2dbs-A is supported by chains belonging to  
    42 different SCOP families (and 33 superfamilies, 30 folds and four classes) and most of the  
    supporting chains belong to the SCOP family c.82.1.1. The coverage of 2in5-A is supported by chains  
    belonging to 67 different SCOP families (and 58 superfamilies, 53 folds and five classes), and of these  
    SCOP families, most of the supporting chains belong to either b.42.1.1 or d.22.1.1. Furthermore, far  
    from all chains in the search set have been classified in SCOP. For 2dbs-A and 2in5-A only 34% and  
    38%, respectively, of the supporting chains have a SCOP classification.

  ### Attachments

  - ijms-14-07795.pdf
- ## Reduced false positives in PDZ binding prediction using sequence and structural descriptors

  |  |  |
  | --- | --- |
  | Type | Journal Article |
  | Author | John C. Hawkins |
  | Author | Hongbo Zhu |
  | Author | Joan Teyra |
  | Author | M. Teresa Pisabarro |
  | URL | http://dl.acm.org/citation.cfm?id=2353085 |
  | Volume | 9 |
  | Issue | 5 |
  | Pages | 1492–1503 |
  | Publication | IEEE/ACM Transactions on Computational Biology and Bioinformatics (TCBB) |
  | Date | 2012 |
  | Accessed | 9/23/2013, 10:14:50 AM |
  | Library Catalog | Google Scholar |
  | Date Added | 10/11/2013, 10:29:15 AM |
  | Modified | 10/11/2013, 10:29:15 AM |

  ### Tags:

  - machine learning
  - PDZ binding
  - protein binding prediction
  - protein structure classification

  ### Notes:

  - PDZ domain binding partner prediction.  The PDZ domain is one of the most widely utilized protein binding domains in nature, and has been shown to be highly selective of its binding partners, on the basis of at least the final four residues.

    How SCOP is used:

    Use type: other  
       
     Application: protein binding partner prediction

    Description: Get domain boundaries for their data set of proteins with PDZ domains.

    SCOP reference:

    In order to obtain a significant amount of data on the binding specificities of a wide range of PDZ domains, we made use of a number of different data sets....

    The domains in these data sets were mapped to known PDZ domains using SCOP version 1.75 [24]. The overlap between the data sets for those domains with experimentally resolved structures is shown in Table 1. In total, we have 25 unique domains, with some of them having binding data coming from multiple sources.

  ### Attachments

  - Snapshot
  - ttb2012051492.pdf
- ## Reduced Polymorphism in Domains Involved in Protein-Protein Interactions

  |  |  |
  | --- | --- |
  | Type | Journal Article |
  | Author | Zohar Itzhaki |
  | Author | Hanah Margalit |
  | Volume | 7 |
  | Issue | 4 |
  | Pages | e34503 |
  | Publication | Plos One |
  | Date | April 2012 |
  | DOI | 10.1371/journal.pone.0034503 |
  | Abstract | Genome sequencing of various individuals or isolates of the same species allows studying the polymorphism level of specific proteins and protein domains. Here we ask whether domains that are known to be involved in mediating protein-protein interactions show lower polymorphism than other domains. To this end we take advantage of a recent genome sequence dataset of 39 Saccahromyces cerevisiae strains and the experimentally determined protein interaction network of the laboratory strain. We analyze the polymorphism in domain residues involved in interactions at various levels of resolution, depending on their likelihood to be interaction mediators. We find that domains involved in interactions are less polymorphic than other domains. Furthermore, as the likelihood of a residue to be involved in interaction increases, its polymorphism decreases. Our results suggest that purifying selection operates on domains capable of mediating protein interactions to maintain their function. |
  | Date Added | 3/7/2014, 12:08:00 PM |
  | Modified | 3/7/2014, 12:08:00 PM |
- ## Relation between sequence and structure in membrane proteins

  |  |  |
  | --- | --- |
  | Type | Journal Article |
  | Author | Mireia Olivella |
  | Author | Angel Gonzalez |
  | Author | Leonardo Pardo |
  | Author | Xavier Deupi |
  | URL | http://bioinformatics.oxfordjournals.org/content/29/13/1589.abstract |
  | Volume | 29 |
  | Issue | 13 |
  | Pages | 1589–1592 |
  | Publication | Bioinformatics |
  | Date | 2013 |
  | Accessed | 9/20/2013, 1:17:33 PM |
  | Library Catalog | Google Scholar |
  | Date Added | 10/11/2013, 10:29:15 AM |
  | Modified | 10/11/2013, 10:29:15 AM |

  ### Notes:

  - Performed sequence and structure alignments to study sequence and structure conservation in membrane proteins.

    How SCOP data is used:

    Use type: benchmarking  
       
     Benchmarking application: study conservation in membrane proteins  
       
     Filtered on:   N/A  
       
     Benchmarking type: c

    Levels used in benchmarking: Family

    Representative set: N/A

    Description: Collected a membrane protein data set from the PDB, then classified into SCOP families to get pairs of homologous domains.  Then compare sequence similarity and RMSD.

    SCOP reference:

    2 METHODS

    2.1 Membrane protein dataset

    The coordinates of polytopic TM proteins with three or more homolo- gous structures and resolution 54.0 A ̊ were obtained from the Protein Data Bank (Berman et al., 2000). Selected proteins were classified accord- ing to the SCOP (Murzin et al., 1995) and OPM (Lomize et al., 2006) databases and include receptors, energy transfer molecules, transporters and channels from different phyla. The native inactive state (i.e. without mutations or activating ligands) was selected for those proteins with more than one structure available. A total of 159 membrane proteins (111 ⬚⬚-helix bundles and 48 b-barrels) representing 25 different families were analyzed (Supplementary Table S1). This resulted in a comparison of 432 pairs (250 in ⬚⬚-helix bundles and 182 in b-barrels) of homologous TM protein subunits.

  ### Attachments

  - [PDF] from researchgate.net
  - Snapshot
  - Supplementary\_material.docx
- ## Relocating the active-site lysine in rhodopsin and implications for evolution of retinylidene proteins

  |  |  |
  | --- | --- |
  | Type | Journal Article |
  | Author | Erin L. Devine |
  | Author | Daniel D. Oprian |
  | Author | Douglas L. Theobald |
  | URL | http://www.pnas.org/content/110/33/13351.short |
  | Volume | 110 |
  | Issue | 33 |
  | Pages | 13351–13355 |
  | Publication | Proceedings of the National Academy of Sciences |
  | Date | 2013 |
  | Accessed | 9/20/2013, 1:12:54 PM |
  | Library Catalog | Google Scholar |
  | Date Added | 10/11/2013, 10:29:15 AM |
  | Modified | 3/7/2014, 12:10:00 PM |

  ### Notes:

  - Experimental study of type I and II rhodopsins.

    How SCOP is used:

    Use SCOP to place protein/family of interest in context.  Seem to be referring to an old version of SCOP.

    How CATH is used:

    Look up bacteriorhodpsin's fold in both SCOP and CATH, and find consensus.

    Reference to SCOP:

    SCOP reference: The GPCR fold comprises seven transmembrane α-helices oriented in a particular spatial arrangement with a specific connectivity (SCOP classification scop.b.g.c.A; ref. 3).

    ..

    Like rhodopsin, bacteriorhodopsin adopts the GPCR fold (1, 3, 6). B

  ### Attachments

  - Full Text PDF
- ## Representing and comparing protein folds and fold families using three-dimensional shape-density representations

  |  |  |
  | --- | --- |
  | Type | Journal Article |
  | Author | Lazaros Mavridis |
  | Author | Anisah W. Ghoorah |
  | Author | Vishwesh Venkatraman |
  | Author | David W. Ritchie |
  | URL | http://onlinelibrary.wiley.com/doi/10.1002/prot.23218/full |
  | Volume | 80 |
  | Issue | 2 |
  | Pages | 530–545 |
  | Publication | Proteins: Structure, Function, and Bioinformatics |
  | Date | 2012 |
  | Accessed | 9/20/2013, 1:17:33 PM |
  | Library Catalog | Google Scholar |
  | Date Added | 10/11/2013, 10:29:15 AM |
  | Modified | 3/7/2014, 12:14:18 PM |

  ### Tags:

  - protein alignment
  - protein classification
  - protein clustering
  - protein comparison
  - protein indexing
  - three-dimensional protein shapes
  - three-dimensional superpositions

  ### Notes:

  - Introduce a 3D structure alignment method, 3DBlast, that relies only on shape-density, and no other information on sequence or other geometry.  The method uses consensus shapes to represent an entire fold family.

    How SCOP is used:

    Did not use SCOP data.

    The method is evaluated against CATH data.

    Just mention SCOP as another classification scheme, and how shape-based indexing could enhance SCOP.

    How CATH is used:

    Evaluate method on CATH data.

    Reference to SCOP:

    To classify the ever growing number of protein fold families and to represent these in a convenient way for automatic indexing and searching, we believe it will be necessary to perform large scale clustering calculations to provide an additional shape-based indexing scheme which will enhance existing classifications schemes such as CATH and SCOP.

     Reference to CATH:

    The utility of this approach is compared with several well-known protein structure align- ment algorithms using receiver-operator-char- acteristic plots of queries against the ‘‘gold standard’’ CATH database. Despite being com- pletely independent of protein sequences and using no information about the internal geom- etry of proteins, our results from searching the CATH database show that 3D-Blast is highly competitive compared to current state- of-the-art protein structure alignment algo- rithms.

    ...

    SPF representation of the CATH database

    This study uses version 3.2 of the CATH database (June 2009; http://www.cathdb.info) consisting of 12,287 nonredundant protein domain structures which have been classified into 2178 homologous super-families (i.e., which belong to the same ‘‘H’’ level in the CATH classifi- cation). These H-level CATH super-families are here called ‘‘fold families’’ for brevity.

    One limitation of the SPF representation is that the Gauss–Laguerre radial basis functions need to be scaled to a given distance range, and any shapes which extend beyond this range are represented only very poorly. Figure 1 shows the distribution of the sizes of the protein domains in the CATH database, as estimated by their maximum radii. This figure shows that most domains have radii in the range 25– 30 A ̊ .

  ### Attachments

  - 23218\_ftp.pdf
- ## Residue mutations and their impact on protein structure and function: detecting beneficial and pathogenic changes

  |  |  |
  | --- | --- |
  | Type | Journal Article |
  | Author | Romain A. Studer |
  | Author | Benoit H. Dessailly |
  | Author | Christine A. Orengo |
  | Volume | 449 |
  | Pages | 581-594 |
  | Publication | Biochemical Journal |
  | ISSN | 0264-6021 |
  | Date | FEB 1 2013 |
  | Extra | WOS:000313776000002 |
  | DOI | 10.1042/8J20121221 |
  | Abstract | The present review focuses on the evolution of proteins and the impact of amino acid mutations on function from a structural perspective. Proteins evolve under the law of natural selection and undergo alternating periods of conservative evolution and of relatively rapid change. The likelihood of mutations being fixed in the genome depends on various factors, such as the fitness of the phenotype or the position of the residues in the three-dimensional structure. For example, co-evolution of residues located close together in three-dimensional space can occur to preserve global stability. Whereas point mutations can fine-tune the protein function, residue insertions and deletions ('decorations' at the structural level) can sometimes modify functional sites and protein interactions more dramatically. We discuss recent developments and tools to identify such episodic mutations, and examine their applications in medical research. Such tools have been tested on simulated data and applied to real data such as viruses or animal sequences. Traditionally, there has been little if any crosstalk between the fields of protein biophysics, protein structure function and molecular evolution. However, the last several years have seen some exciting developments in combining these approaches to obtain an in-depth understanding of how proteins evolve. For example, a better understanding of how structural constraints affect protein evolution will greatly help us to optimize our models of sequence evolution. The present review explores this new synthesis of perspectives. |
  | Date Added | 2/20/2014, 12:24:01 PM |
  | Modified | 3/7/2014, 12:08:48 PM |

  ### Notes:

  - Review of studies on protein evolution and impact of amino acid substitutions on function and structure.

    How SCOP/CATH is used:

    Cite a previous studoes that used SCOP or CATH.

    The previous study with SCOP examined local structural variations in indel-flanking regions within SCOP families.

    SCOP reference:

    Relationship of indels and nucleotide substitutions

    Indels tend to occur in hotspot regions, which are prone to higher substitution rates of amino acids [70]. This phenomenon has been observed in both eukaryota [71] and bacteria [72]. Analysis of SCOP families revealed structural shifts in the flanking region of indels [73]. This correlation of indels with hotspots can be partly explained by the fact that both elevated rates of amino acid substitutions and indels occur in regions containing amino acid repeats, and these could act as mutagenic drivers [74] especially in the case of repeated hydrophilic residues [75].

    CATH reference:

    Methods for detecting adaptive substitutions

    Applied at the amino acid level

    A plethora of different algorithms have been developed to identify sites under functional divergence (see Table 1). The sites predicted can vary greatly between these tools, depending on the definitions used for conservation and similarity [97,98]. A number of resources also provide information about sites that are conserved within functional families. These include FunShift [99], the SDR (specificity-determining residue) database [100], CATH and its sister site Gene3D [59,101] and Cube-DB [102].

  ### Attachments

  - 4490581.pdf
- ## Restrictions to protein folding determined by the protein size

  |  |  |
  | --- | --- |
  | Type | Journal Article |
  | Author | Alexei V. Finkelstein |
  | Author | Natalya S. Bogatyreva |
  | Author | Sergiy O. Garbuzynskiy |
  | URL | http://www.sciencedirect.com/science/article/pii/S0014579313003360 |
  | Publication | FEBS letters |
  | Date | 2013 |
  | Accessed | 9/20/2013, 1:18:50 PM |
  | Library Catalog | Google Scholar |
  | Date Added | 2/13/2014, 4:13:17 PM |
  | Modified | 3/7/2014, 12:09:48 PM |

  ### Notes:

  - Computational study of factors that contribute to differences in folding times, such as size, shape, and stability of the fold.

    How SCOP/CATH is used:

    Examine distribution of domain sizes in SCOP and CATH (likely used ASTRAL representative set without a citation).  Found that of the 1% of domains in the SCOP subset of 4861 domains have more than 500 residues, of those 1%, the majority are composed of structural repeats (like Armadillo or beta-propeller blades).

    SCOP/CATH reference:

    7. Materials and methods

    In this study, we consider single-domain proteins (or separate domains) without disulfide bonds or covalently bound ligands.

    ...

    The analysis of domains listed in the comprehensive protein structure databases SCOP [59] and CATH [60] confirms this esti- mate of the maximal domain size: a few SCOP-domains,<1% of 4861 (see Fig. 3A, B), have more than 500 residues; 30% of this 1% contain two or even more structural domains according to CATH while all the rest (70% of the 1%) are either significantly ob- late, or significantly oblong, or composed of several compact, do- main-like structural repeats (like Armadillo repeats [61] and beta-propeller blades [62]).

    ...

    Protein domains in Fig. 3A and B belong to four main SCOP [59] structural classes (a, b, a/b, a + b). The SCOP ‘‘domains’’ that con- sist of more than one domain, according to the SCOP remarks, are not taken into account. All of the other single-chain SCOP do- mains with sequence identity below 80% [65] have been selected.

  ### Attachments

  - 1-s2.0-S0014579313003360-main.pdf
- ## Retinoid-Binding Proteins: Similar Protein Architectures Bind Similar Ligands via Completely Different Ways

  |  |  |
  | --- | --- |
  | Type | Journal Article |
  | Author | Yu-Ru Zhang |
  | Author | Yu-Qi Zhao |
  | Author | Jing-Fei Huang |
  | Volume | 7 |
  | Issue | 5 |
  | Pages | e36772 |
  | Publication | Plos One |
  | ISSN | 1932-6203 |
  | Date | MAY 4 2012 |
  | Extra | WOS:000305349800121 |
  | DOI | 10.1371/journal.pone.0036772 |
  | Abstract | Background: Retinoids are a class of compounds that are chemically related to vitamin A, which is an essential nutrient that plays a key role in vision, cell growth and differentiation. In vivo, retinoids must bind with specific proteins to perform their necessary functions. Plasma retinol-binding protein (RBP) and epididymal retinoic acid binding protein (ERABP) carry retinoids in bodily fluids, while cellular retinol-binding proteins (CRBPs) and cellular retinoic acid-binding proteins (CRABPs) carry retinoids within cells. Interestingly, although all of these transport proteins possess similar structures, the modes of binding for the different retinoid ligands with their carrier proteins are different. Methodology/Principal Findings: In this work, we analyzed the various retinoid transport mechanisms using structure and sequence comparisons, binding site analyses and molecular dynamics simulations. Our results show that in the same family of proteins and subcellular location, the orientation of a retinoid molecule within a binding protein is same, whereas when different families of proteins are considered, the orientation of the bound retinoid is completely different. In addition, none of the amino acid residues involved in ligand binding is conserved between the transport proteins. However, for each specific binding protein, the amino acids involved in the ligand binding are conserved. The results of this study allow us to propose a possible transport model for retinoids. Conclusions/Significance: Our results reveal the differences in the binding modes between the different retinoid-binding proteins. |
  | Date Added | 2/20/2014, 12:24:01 PM |
  | Modified | 2/20/2014, 12:24:01 PM |

  ### Notes:

  - Computational study of binding in retinoid proteins.

    How SCOP is used:

    Look up superfamily and family classification of retinoid proteins in SCOP.

    SCOP reference:

    RBP, ERABP, CRBPs (CRBP I, II, III, and IV) and CRABPs (CRABP I and CRABP II) belong to the lipocalins superfamily in the Structural Classification of Proteins (SCOP) database [3]. Although they differ both in sequence and function, all members of the lipocalins superfamily contain a six- or eight-stranded b- barrel as part of their tertiary structure and a highly conservative motif, the short conserved region (SCR), as part of their amino acid sequence [4]. In the SCOP, RBP and ERABP belong to the retinol-binding protein-like (RBP) family. CRBPs and CRABPs belong to the fatty acid-binding protein-like (FABP) family.

    ....

  ### Attachments

  - journal.pone.0036772.pdf
- ## Retrieving backbone string neighbors provides insights into structural modeling of membrane proteins

  |  |  |
  | --- | --- |
  | Type | Journal Article |
  | Author | Jiang-Ming Sun |
  | Author | Tong-Hua Li |
  | Author | Pei-Sheng Cong |
  | Author | Sheng-Nan Tang |
  | Author | Wen-Wei Xiong |
  | Volume | 11 |
  | Issue | 7 |
  | Pages | M111.016808 |
  | Publication | Molecular & cellular proteomics: MCP |
  | ISSN | 1535-9484 |
  | Date | Jul 2012 |
  | Extra | PMID: 22415040 |
  | Journal Abbr | Mol. Cell Proteomics |
  | DOI | 10.1074/mcp.M111.016808 |
  | Library Catalog | NCBI PubMed |
  | Language | eng |
  | Abstract | Identification of protein structural neighbors to a query is fundamental in structure and function prediction. Here we present BS-align, a systematic method to retrieve backbone string neighbors from primary sequences as templates for protein modeling. The backbone conformation of a protein is represented by the backbone string, as defined in Ramachandran space. The backbone string of a query can be accurately predicted by two innovative technologies: a knowledge-driven sequence alignment and encoding of a backbone string element profile. Then, the predicted backbone string is employed to align against a backbone string database and retrieve a set of backbone string neighbors. The backbone string neighbors were shown to be close to native structures of query proteins. BS-align was successfully employed to predict models of 10 membrane proteins with lengths ranging between 229 and 595 residues, and whose high-resolution structural determinations were difficult to elucidate both by experiment and prediction. The obtained TM-scores and root mean square deviations of the models confirmed that the models based on the backbone string neighbors retrieved by the BS-align were very close to the native membrane structures although the query and the neighbor shared a very low sequence identity. The backbone string system represents a new road for the prediction of protein structure from sequence, and suggests that the similarity of the backbone string would be more informative than describing a protein as belonging to a fold. |
  | Date Added | 10/11/2013, 10:29:15 AM |
  | Modified | 10/11/2013, 10:29:15 AM |

  ### Tags:

  - Algorithms
  - Amino Acid Sequence
  - Computational Biology
  - Databases, Protein
  - Humans
  - Membrane Proteins
  - Models, Molecular
  - Molecular Sequence Data
  - Protein Conformation
  - Proteus mirabilis
  - Sequence Alignment
  - Sequence Analysis, Protein
  - Sequence Homology, Amino Acid
  - Structural Homology, Protein

  ### Notes:

  - Present the BS-align method for protein structure prediction using a threading-type approach.  Encode the 3D backbone structure as a "backbone string", and use to build a database on which their method relies.

    Evaluated method on the nr3PDB database.

    How SCOP is used:

    Classify a non-redundant data set (nr3PDB) that is used to build the "backbone string" database by SCOP fold.  When sequence redundancy was removed from their backbone string database, they found that there was approximately one string per SCOP fold, implying that these backbone strings could be a used for fold-classification.

    SCOP Reference:

    Backbone String Database—We utilized the actual back- bone string of all known structural proteins in the nr3PDB da- tabase (NCBI MMDB 2009 Dec, three-level nonredundancy, 40849 entries in total) and constructed the backbone string database (BSD), which served as a benchmark alignment da- tabase. When we reduced the redundancy of the BSD by CD- HIT (29), the number of entries decreased quickly (Fig. 2), which confirmed the fact that the backbone string was more con- served than the sequence. These observations indicated that the backbone string maintained strong structural integrity and could be considered as the bridge between sequence- based and structure-based methods. When the backbone string identity was reduced to 50%, the number of left entries was approximately equal to the number of the folds in SCOP (1193, V1.75, 2009) (30). This finding implied that the backbone string may be a good criterion of protein classification. Moreover, the similarity of the backbone strings was the foundation of BS-align and was especially useful when sequences alignment was unfeasible.

    ...

    DISCUSSION

    ...

    The second ad- vantage was that the backbone string was more conservative than the sequence. For BSD, when the backbone string iden- tity was reduced to 40% (Fig. 2), only 74 entries (72 proteins) remained, which indicated that cross fold similarities were abundant in geometrically similar proteins. Based on the SCOP classification system, there were 24 all-beta proteins, 14 alpha and beta proteins (a⬚⬚b), 14 small proteins, 12 alpha and beta proteins (a/b), 10 all-alpha proteins, four multido- main proteins, three membrane and cell surface proteins, four peptides and one coiled coil protein found in these entries with lengths varying between 54 and 2512 residues. This phenomenon implied that the protein structures were fairly conserved and suggested that the backbone string may be a suitable criterion of taxonomy and a backbone string-based library may be more reasonable and compact than existing libraries.

  ### Attachments

  - Mol Cell Proteomics-2012-Sun-.pdf
  - PubMed entry
- ## Re-visiting protein-centric two-tier classification of existing DNA-protein complexes

  |  |  |
  | --- | --- |
  | Type | Journal Article |
  | Author | Sony Malhotra |
  | Author | Ramanathan Sowdhamini |
  | URL | http://www.biomedcentral.com/1471-2105/13/165/ |
  | Volume | 13 |
  | Issue | 1 |
  | Pages | 165 |
  | Publication | BMC bioinformatics |
  | Date | 2012 |
  | Accessed | 9/23/2013, 10:18:03 AM |
  | Library Catalog | Google Scholar |
  | Date Added | 10/11/2013, 10:29:15 AM |
  | Modified | 10/11/2013, 10:29:15 AM |

  ### Tags:

  - classification
  - DNA
  - DNA-protein interactions
  - Genome-wide survey
  - Sequence searches

  ### Notes:

  - Updated a 2-tier classification of DNA-protein complexes that was introduced, and last curated, in 2000.  Added new families and groups.  The first level is the group, indicating the type of DNA binding motif in the protein partner.  The second level is the family, corresponding to the functional role of the protein.

    How SCOP is used:

    Annotate folds of newly classified structures in their database, in order to report on fold diversity and growth.

    SCOP references:

    In abstract:

    There were 34 SCOP folds which were observed to be present in the complexes of both old and new classifications, whereas 28 folds are present exclusively in the new complexes.

    ...

    The new families were also examined for their folds as ascribed to them by SCOP 1.75 [16], and the folds were recorded [see Additional file 1]. Although SCOP is a highly updated database, we realised that ~30% of the entries (PDB IDs) were not included in SCOP 1.75 due to newer PDB entries. 34 SCOP folds were common to both new and old classification and they experienced an expansion in the number of complexes. The fold change in these 34 common folds is represented in Figure 8. The number of members, belonging to both old and new classification possessing each of the common 34 folds is summarised [see Additional file 4]. The top three folds, experiencing maximum expansion in terms of members possessing them, were Histone, Homing endo- nuclease and DNA/RNA Polymerase - truly reflecting the maximum increase in the number of members and families in enzymes group. Therefore, expansion in the existing families was seen to a maximum extent in the families of enzyme group which have property to bind to DNA and then carry out an enzymatic activity.

    Figure 8 Common SCOP folds in old and new classification34 common folds in both old and new classification complexes. Number of members possessing these folds expanded in new classification compared to old classification. The fold increase in the number of members with each of these 34 folds is plotted. Maximum fold increase of 27 was observed in Histone family.

    Figure 9 SCOP folds only in new classification28 folds present only in newly classified complexes. The fold exhibited by maximum number of newly classified complexes are those which are involved in DNA damage repair functions like Lesion bypass DNA Polymerase, MutS domain, Glycosylase. Numbers represent the respective names of SCOP fold in the Figure 1. ATP-dependent DNA ligase DNA-binding domain, 2. Cryptochrome/photolyase FAD-binding domain, 3. DNA-clamp, 4. Double-stranded β-helix, 5. GCM domain, 6. Hcp1-like, 7. Metallo-dependent phosphatases, 8. Phage replication organizer domain, 9. SPOC domain-like, vWA-like, 10. Thioredoxin fold, 11. Type II DNA topoisomerase ,12. DNA-binding domain of intron-encoded endonucleases, 13. Phospholipase D/nuclease, 14. Replication modulator SeqA, C-terminal DNA-binding domain, 15. SMAD MH1 domain, 16. UDP-Glycosyltransferase/glycogen phosphorylase, 17. N-terminal domain of MutM-like DNA repair  
     proteins, 18. P-loop containing nucleoside triphosphate hydrolases, 19. SAM domain-like, 20. SRF-like, 21. Zinc finger design, 22. Origin of replication-binding domain, RBD-like, 23. Ribonuclease H-like motif, 24. PUA domain-like, 25. DNA-glycosylase, 26. Putative DNA-binding domain, 27. DNA-repair protein MutS, domain III, 28. Lesion bypass DNA polymerase (Y-family).

  ### Attachments

  - 1471-2105-13-165.pdf
  - [HTML] from biomedcentral.com
- ## Ribosomal history reveals origins of modern protein synthesis

  |  |  |
  | --- | --- |
  | Type | Journal Article |
  | Author | Ajith Harish |
  | Author | Gustavo Caetano-Anollés |
  | URL | http://dx.plos.org/10.1371/journal.pone.0032776 |
  | Volume | 7 |
  | Issue | 3 |
  | Pages | e32776 |
  | Publication | PLoS one |
  | Date | 2012 |
  | Accessed | 9/20/2013, 1:16:44 PM |
  | Library Catalog | Google Scholar |
  | Date Added | 10/11/2013, 10:29:15 AM |
  | Modified | 12/2/2013, 4:16:19 PM |

  ### Tags:

  - Evolution, Molecular
  - Likelihood Functions
  - Models, Molecular
  - Phylogeny
  - Protein Biosynthesis
  - Proteins
  - Ribonucleoproteins
  - Ribosomes
  - RNA, Ribosomal

  ### Notes:

  - Phylogenetic analysis of ribosomal proteins.

    How SCOP is used:

    Create a data set of protein sequences from 749 completely sequenced organisms and detect structural domains using SUPERFAMILY with models from SCOP 1.73.  Assign each domain a SCOP sccs, then build matrices to count the occurrence of each superfamily in a proteome.  Built phylogenetic trees using the matrices.

    SCOP reference:

    Phylogenomic Analysis of Protein Domain Structure and Ancestry of r-Proteins

    The general scheme applied to the evolutionary study of rRNA structure has been applied to the evolutionary study of protein domain structures [15,33]. The scheme is illustrated in Figure 1. We first conducted a census of genomic sequence in 749 organisms that have been completely sequenced (52 archaeal, 478 bacterial, and 219 eukaryal species) assigning protein structural domains at FSF level of structural complexity to protein sequences using linear HMMs of structural recognition in SUPERFAMILY [114] and probability cutoffs E of 1024. Domains were defined by SCOP version 1.73 [115,116] and described using SCOP concise classification strings (ccs). ccs descriptors are widely used symbolic representations of domains within the hierarchy of structural classification (e.g., the P-loop hydrolase FSF is named c.37.1, where c represents the protein class, 37 the fold and 1 the FSF). Features that numerically characterize the genomic abundance of each FSF (g) were used as characters to build data matrices for phylogenetic analysis. g indicates the number of multiple occurrences of an FSF domain in a proteome. Empirically, g values range from 0 to thousands and resemble morphometric data with a large variance [116,117].

  ### Attachments

  - [HTML] from plos.org
  - journal.pone.0032776.pdf
  - PubMed entry
- ## Ric-8: Different cellular roles for a heterotrimeric G-protein GEF

  |  |  |
  | --- | --- |
  | Type | Journal Article |
  | Author | M. V. Hinrichs |
  | Author | M. Torrejon |
  | Author | M. Montecino |
  | Author | J. Olate |
  | Volume | 113 |
  | Issue | 9 |
  | Pages | 2797-2805 |
  | Publication | Journal of Cellular Biochemistry |
  | ISSN | 0730-2312 |
  | Date | SEP 2012 |
  | Extra | WOS:000306292700001 |
  | DOI | 10.1002/jcb.24162 |
  | Abstract | Signaling via heterotrimeric G-proteins is evoked by agonist-mediated stimulation of seven transmembrane spanning receptors (GPCRs). During the last decade it has become apparent that Ga subunits can be activated by receptor-independent mechanisms. Ric-8 belongs to a highly conserved protein family that regulates heterotrimeric G-protein function, acting as a non-canonical guanine nucleotide exchange factors (GEF) over a subset of Ga subunits. In this review we discuss the roles of Ric-8 in the regulation of diverse cell functions, emphasizing the contribution of its multiple domain protein structure in these diverse functions. J. Cell. Biochem. 113: 27972805, 2012. (C) 2012 Wiley Periodicals, Inc. |
  | Date Added | 2/20/2014, 12:24:01 PM |
  | Modified | 2/20/2014, 12:24:01 PM |

  ### Notes:

  - Review discusses roles of Ric-8, a multi-domain protein, in regulation of diverse functions.

    How SCOP is used:

    Perform modeling to determine a potential structure for Ric-8.  Look up the fold for the best template found.

    SCOP reference:

    Ric-8 PROTEIN STRUCTURE

    One way of understanding the different functions associated with a regulatory protein is by knowing its three-dimensional structure. Nevertheless, until now no group has experimentally assessed and reported with significant detail the structural features of the Ric-8 proteins. Recently, our research team carried out a structural characterization of Ric-8B in silico by building a model of its putative three-dimensional structure. Because Ric-8B has no homology to any other known protein, we utilized different bioinformatic methods that are based on folding recognition motifs (threading) to construct a structural model for Xenopus laevis Ric-8 (xRic-8) in the absence of a template. **The structural model obtained for Ric-8B shows an alpha–alpha superhelix folding that corre- sponds to the armadillo structure according to SCOP classification [Andreeva et al., 2008].** Based on this folding, we subsequently built a refined model using as templates proteins that are known to contain the armadillo structure [Coates, 2003]. We propose that the xRic-8 structure is formed by 10 armadillo folding motifs, organized in a right-twisted alpha–alpha super helix (Fig. 5).

  ### Attachments

  - 24162\_ftp.pdf
- ## Rice heterotrimeric G-protein alpha subunit (RGA1): In silico analysis of the gene and promoter and its upregulation under abiotic stress

  |  |  |
  | --- | --- |
  | Type | Journal Article |
  | Author | Dinesh K. Yadav |
  | Author | Devesh Shukla |
  | Author | Narendra Tuteja |
  | Volume | 63 |
  | Pages | 262–271 |
  | Publication | Plant Physiology and Biochemistry |
  | Date | February 2013 |
  | DOI | 10.1016/j.plaphy.2012.11.031 |
  | Abstract | Heterotrimeric G-protein complexes (G alpha, G beta and G gamma) operate at the apex of diverse signal transduction systems along with their cognate transmembrane G-protein coupled receptors (GPCRs) and appropriate downstream effectors in the plant Rice G alpha in response to stress has not been well studied. Here, we report the in silico analysis of G alpha subunit from Oryza sativa cv. Indica group Swarna [RGA1(I), accession number HQ634688], its promoter and its transcript upregulation in response to abiotic stresses. Genomic sequence of RGA1(I) contains thirteen exonic and twelve intronic segments. Phylogenetic analysis of RGA1(I) demonstrated high homology with Sorghum and maize and is distantly related to barley and wheat Promoter sequence analysis of RGA1(I) confirms the presence of stress-related cis-regulatory elements viz. ABA, MeJAE, ARE, GT-1 boxes and LTR suggesting its active and possible independent roles in abiotic stress signalling. Expasy PROSITE database of protein families and domains revealed important motifs, patterns and biologically significant sites in RGA1(I). Three dimensional structure of RGA1(I) protein predicted by I-TASSER server and its stereochemical qualities were validated by PROCHECK and QMEAN server indicating the acceptability of the predicted model. The transcript profiling of RGA1(I) showed upregulation following NaCl, cold and drought stress. Under elevated temperature, its transcript was down regulated. Heavy metal(loid)s stress showed rhythmic and strong upregulation. It showed a rhythmic response in ABA stress. These findings provide a critical evidence for its active role in regulation of abiotic stresses in rice. These findings suggest its possible exploitation in the development of abiotic stress tolerance in crops. (c) 2012 Elsevier Masson SAS. All rights reserved. |
  | Date Added | 3/7/2014, 12:08:00 PM |
  | Modified | 3/7/2014, 12:08:00 PM |
- ## Rice transglutaminase gene: Identification, protein expression, functionality, light dependence and specific cell location

  |  |  |
  | --- | --- |
  | Type | Journal Article |
  | Author | N. Campos |
  | Author | S. Castañón |
  | Author | I. Urreta |
  | Author | M. Santos |
  | Author | J. M. Torné |
  | URL | http://www.sciencedirect.com/science/article/pii/S016894521300023X |
  | Publication | Plant Science |
  | Date | 2013 |
  | Accessed | 9/23/2013, 10:03:53 AM |
  | Library Catalog | Google Scholar |
  | Short Title | Rice transglutaminase gene |
  | Date Added | 10/11/2013, 10:29:15 AM |
  | Modified | 10/11/2013, 10:29:15 AM |

  ### Tags:

  - Cell location
  - Cloning
  - Light dependence
  - Protein expression
  - Rice
  - Transglutaminase

  ### Notes:

  - The paper details the characterization and functions of transglutaminases in rice as part of study to determine TGase use in plants. The protein expression was found to be light dependent.

    How SCOP is used:

    SCOP is mentioned briefly to note which superfamily their proteins are classified in.

    SCOP reference:

    TGases, papain- like thiol proteases, peptide:N-glycanases (PNGases) and N-acetyl transferases are classified within the same protein superfamily (TGase-like) according to the structural classification of proteins (SCOP) database (http://scop.mrc-Imb.cam.ac.uk/scop) [4,5].

  ### Attachments

  - 1-s2.0-S016894521300023X-main.pdf
- ## Right-and left-handed three-helix proteins. I. Experimental and simulation analysis of differences in folding and structure

  |  |  |
  | --- | --- |
  | Type | Journal Article |
  | Author | Anna V. Glyakina |
  | Author | Leonid B. Pereyaslavets |
  | Author | Oxana V. Galzitskaya |
  | URL | http://onlinelibrary.wiley.com/doi/10.1002/prot.24301/full |
  | Publication | Proteins: Structure, Function, and Bioinformatics |
  | Date | 2013 |
  | Accessed | 9/23/2013, 10:20:00 AM |
  | Library Catalog | Google Scholar |
  | Date Added | 10/11/2013, 10:29:15 AM |
  | Modified | 2/20/2014, 9:52:20 AM |

  ### Tags:

  - likely ASTRAL
  - likely ASTRAL domain structures
  - likely ASTRAL sequences

  ### Notes:

  - Investigation of folding rates of 3-helix protein folding and the influence of handedness.  Modeled folding using Monte Carlo.

    How SCOP is used:

    Use SCOP database to download all domains from all-alpha class.  Then reduced the size of the data set by examining the tertiary structures. Ended up with data set of 385 "proteins".

    Did not use ASTRAL representative set.  "We reduced our research to protein domains that have no more than 85% homology between them, and chose those with best resolution in the case of crystallography or with bigger number models in the case of NMR study, preferring the former to the latter."

    Reference to SCOP:

    For construction of the second dataset, we took from the SCOP database (version 1.75)27,28 all domains which are considered as alpha-helical folds and assigned all secondary structures with DSSP.29 All residues assigned as a-helical, p-helical, and 3/10 helical were considered as helical. Only helices with at least six helical residues were considered. If two helices have a common direction within 45⬚⬚ and have up to four residues between them which are not classified as helical by DSSP, but lay in the conventional helical region, these two or more regions are considered as one whole helix. We reduced our research to protein domains that have no more than 85% homology between them, and chose those with best resolution in the case of crystallography or with bigger number models in the case of NMR study, preferring the former to the latter. All domains were verified manually and finally we got 385 proteins which are considered with the abovementioned rules as three- helical. For some of them, we cut tails which do not affect domain stability or have huge flexibility either uncertainty, in the case of NMR models.

  ### Attachments

  - prot24301.pdf
- ## RNA structure and dynamics: A base pairing perspective

  |  |  |
  | --- | --- |
  | Type | Journal Article |
  | Author | Sukanya Halder |
  | Author | Dhananjay Bhattacharyya |
  | Volume | 113 |
  | Issue | 2 |
  | Pages | 264-283 |
  | Publication | Progress in Biophysics & Molecular Biology |
  | ISSN | 0079-6107 |
  | Date | NOV 2013 |
  | Extra | WOS:000328806300003 |
  | DOI | 10.1016/j.pbiomolbio2013.07.003 |
  | Abstract | RNA is now known to possess various structural, regulatory and enzymatic functions for survival of cellular organisms. Functional RNA structures are generally created by three-dimensional organization of small structural motifs, formed by base pairing between self-complementary sequences from different parts of the RNA chain. In addition to the canonical Watson Crick or wobble base pairs, several non-canonical base pairs are found to be crucial to the structural organization of RNA molecules. They appear within different structural motifs and are found to stabilize the molecule through long-range intra-molecular interactions between basic structural motifs like double helices and loops. These base pairs also impart functional variation to the minor groove of A-form RNA helices, thus forming anchoring site for metabolites and ligands. Non-canonical base pairs are formed by edge-to-edge hydrogen bonding interactions between the bases. A large number of theoretical studies have been done to detect and analyze these non-canonical base pairs within crystal or NMR derived structures of different functional RNA. Theoretical studies of these isolated base pairs using ab initio quantum chemical methods as well as molecular dynamics simulations of larger fragments have also established that many of these non-canonical base pairs are as stable as the canonical Watson Crick base pairs. This review focuses on the various structural aspects of non-canonical base pairs in the organization of RNA molecules and the possible applications of these base pairs in predicting RNA structures with more accuracy. (C) 2013 Elsevier Ltd. All rights reserved. |
  | Date Added | 2/12/2014, 1:36:22 PM |
  | Modified | 10/8/2014, 12:50:54 PM |

  ### Tags:

  - Detection of non-canonical base pairs
  - g-center-dot
  - higher-order structures
  - intervening sequence rna
  - large ribosomal-subunit
  - Non-canonical base pair
  - nucleic-acid structures
  - protein data-bank
  - quantum-chemical calculations
  - RNA secondary structure
  - small nucleolar rnas
  - Structural characterization of non-canonical base pairs
  - structure database analysis
  - watson-crick/sugar-edge

  ### Notes:

  - Review of methods to analyze RNA structure and dynamics.

    How SCOP is used:

    background on protein structure classification.

    SCOP reference:

    Considering the need of classification of these proteins, there are a number of methods available, such as SCOP (Murzin et al., 1995; Hubbard et al., 1997), FSSP (Holm and Sander, 1997), Pisces (Wang and Dunbrack, 2005), BIPA (Lee and Blundell, 2009) etc. These methods can classify a protein struc- ture based on its structural class, source organism, secondary structure content, resolution, etc. In a similar manner, it is also necessary to organize the available RNA structures to determine different structureefunction relationships.

    ...

    However, similar to the structural classi- fication of proteins into a, b, a/b, (a þ b) categories by SCOP, the classification of RNA based on these structural motifs is difficult as the structural motifs are few and common to all types of RNA.

  ### Attachments

  - 1-s2.0-S0079610713000588-main.pdf
- ## Robust, high-throughput solution structural analyses by small angle X-ray scattering (SAXS)

  |  |  |
  | --- | --- |
  | Type | Journal Article |
  | Author | Greg L Hura |
  | Author | Angeli L Menon |
  | Author | Michal Hammel |
  | Author | Robert P Rambo |
  | Author | Farris L, 2nd Poole |
  | Author | Susan E Tsutakawa |
  | Author | Francis E, Jr Jenney |
  | Author | Scott Classen |
  | Author | Kenneth A Frankel |
  | Author | Robert C Hopkins |
  | Author | Sung-Jae Yang |
  | Author | Joseph W Scott |
  | Author | Bret D Dillard |
  | Author | Michael W W Adams |
  | Author | John A Tainer |
  | Volume | 6 |
  | Issue | 8 |
  | Pages | 606-612 |
  | Publication | Nature methods |
  | ISSN | 1548-7105 |
  | Date | Aug 2009 |
  | Extra | PMID: 19620974 |
  | Journal Abbr | Nat. Methods |
  | DOI | 10.1038/nmeth.1353 |
  | Library Catalog | NCBI PubMed |
  | Language | eng |
  | Abstract | We present an efficient pipeline enabling high-throughput analysis of protein structure in solution with small angle X-ray scattering (SAXS). Our SAXS pipeline combines automated sample handling of microliter volumes, temperature and anaerobic control, rapid data collection and data analysis, and couples structural analysis with automated archiving. We subjected 50 representative proteins, mostly from Pyrococcus furiosus, to this pipeline and found that 30 were multimeric structures in solution. SAXS analysis allowed us to distinguish aggregated and unfolded proteins, define global structural parameters and oligomeric states for most samples, identify shapes and similar structures for 25 unknown structures, and determine envelopes for 41 proteins. We believe that high-throughput SAXS is an enabling technology that may change the way that structural genomics research is done. |
  | Date Added | 10/11/2013, 10:29:15 AM |
  | Modified | 10/11/2013, 10:29:15 AM |

  ### Tags:

  - Bacterial Proteins
  - Equipment Design
  - Models, Molecular
  - Protein Conformation
  - Proteins
  - Pyrococcus furiosus
  - Scattering, Small Angle
  - X-Ray Diffraction

  ### Notes:

  - Present a pipeline for high-throughput analysis of protein structure in solution using small angle X-ray scattering.

    How SCOP is used:

    SCOP data is not used.  SCOP is referenced to point out that crystallography and NMR spectroscopy have provided a 'deep and broad survey' of structural properties.

    SCOP reference:

    both crystallography and NMR spectroscopy have provided a deep and broad survey of macro- molecular structural properties at high resolution6–8.

  ### Attachments

  - nmeth.1353.pdf
  - PubMed entry
- ## Roles of residues in the interface of transient protein-protein complexes before complexation

  |  |  |
  | --- | --- |
  | Type | Journal Article |
  | Author | Lakshmipuram S Swapna |
  | Author | Ramachandra M Bhaskara |
  | Author | Jyoti Sharma |
  | Author | Narayanaswamy Srinivasan |
  | Volume | 2 |
  | Pages | 334 |
  | Publication | Scientific reports |
  | ISSN | 2045-2322 |
  | Date | 2012 |
  | Extra | PMID: 22451863 |
  | Journal Abbr | Sci Rep |
  | DOI | 10.1038/srep00334 |
  | Library Catalog | NCBI PubMed |
  | Language | eng |
  | Abstract | Transient protein-protein interactions play crucial roles in all facets of cellular physiology. Here, using an analysis on known 3-D structures of transient protein-protein complexes, their corresponding uncomplexed forms and energy calculations we seek to understand the roles of protein-protein interfacial residues in the unbound forms. We show that there are conformationally near invariant and evolutionarily conserved interfacial residues which are rigid and they account for ∼65% of the core interface. Interestingly, some of these residues contribute significantly to the stabilization of the interface structure in the uncomplexed form. Such residues have strong energetic basis to perform dual roles of stabilizing the structure of the uncomplexed form as well as the complex once formed while they maintain their rigid nature throughout. This feature is evolutionarily well conserved at both the structural and sequence levels. We believe this analysis has general bearing in the prediction of interfaces and understanding molecular recognition. |
  | Date Added | 10/11/2013, 10:29:15 AM |
  | Modified | 10/11/2013, 10:29:15 AM |

  ### Notes:

  - Biophysical study of protein-protein complex formation.

    How SCOP Is used:

    Derive a data set from the Benchmark4 protein docking benchmarking dataset, which was derived from SCOP.

    Proteins in dataset are "nonredundant at the level of their SCOP families"

    SCOP reference:

    Methods

    Dataset of 3D-structures of bound & unbound forms of transient protein-protein complexes. A curated dataset of structures of proteins involved in transient interactions, solved in both unbound and bound forms, were taken from Benchmark4 dataset35. Out of the 176 transient protein-protein complexes available, only those structures of unbound forms solved at a resolution better than 2A ̊ were considered for the analysis. Further, only entries containing single chain in asymmetric unit and biological unit, with no other macromolecular ligand bound were considered, to ensure that there was no bias due to crystal contacts and ligand-binding. This dataset was further pruned by removing entries belonging to the class of antigen-antibody interactions owing to their specialized nature of interaction. The remaining entries were clustered at 25% sequence identity using BLASTCLUST algorithm (http:// www.csc.fi/english/research/sciences/bioscience/programs/blast/blastclust) to remove redundant sequence information. Finally, a non-redundant dataset of 67 structures of unbound forms solved at high resolution was obtained. This dataset consists of proteins performing diverse functions, ranging from enzyme-substrates/ inhibitors, signalling proteins, and other proteins involved in cellular processes. **Interacting proteins of each binary complex of dataset are non-redundant at the level of their SCOP families****36**. The PDB accession codes for the high-resolution unbound forms, the corresponding bound forms and the interacting partner in the bound form are provided in Supplementary Table S1 online.

  ### Attachments

  - srep00334.pdf
- ## Rooted Phylogeny of the Three Superkingdoms

  |  |  |
  | --- | --- |
  | Type | Journal Article |
  | Author | Ajith Harish |
  | Author | Anders Tunlid |
  | Author | Charles G. Kurland |
  | URL | http://www.sciencedirect.com/science/article/pii/S030090841300134X |
  | Publication | Biochimie |
  | Date | 2013 |
  | Accessed | 9/20/2013, 1:18:20 PM |
  | Library Catalog | Google Scholar |
  | Date Added | 10/11/2013, 10:29:15 AM |
  | Modified | 10/11/2013, 10:29:15 AM |

  ### Tags:

  - Gene duplication
  - Genome content/protein domain
  - Innocuous HGT
  - Reductive evolution
  - Rooted phylogeny

  ### Notes:

  - **Paper Summary**

    The paper claims to find evidence that bacteria and archaea have a common ancestor, LACA, and that eukarya diverged earlier on from these two based on analysis of protein domains. The common ancestor of this tree was very complex.

    **SCOP Use**

    They pulled the information of protein domains at the superfamily level from SCOP's database from SUPERFAMILY. All the superfamilies where sorted based on whether they appeared in Archaea, Bacteria, and/or Eukaryotes.

    **SCOP Reference**

    The data supporting these interpretations were obtained by  
    phylogenetic analysis of roughly 1700 compact protein domains,  
    each representing a cohort of structural and functional homologs  
    that were identified by hidden Markov annotation at the level of  
    superfamily in hundreds of genomes [5,6].

     Structural and functional annotations of proteins from  
    completely sequenced genomes were obtained from the SUPERFAMILY  
    (1.75) database. Here, annotations are based on hidden  
    Markov models (HMM) that identify recurrent protein domains at  
    the superfamily level of the SCOP (Structural Classification of Proteins)  
    hierarchy [22]. In this hierarchy, the domains correspond to  
    stable tertiary folds that have been identified by X-ray crystallographic  
    and/orNMRspectroscopic methods [22].

  ### Attachments

  - 1-s2.0-S030090841300134X-main.pdf
  - Snapshot
- ## RSARF: prediction of residue solvent accessibility from protein sequence using Random Forest method

  |  |  |
  | --- | --- |
  | Type | Journal Article |
  | Author | Ganesan Pugalenthi |
  | Author | Krishna Kumar Kandaswamy |
  | Author | Kuo-Chen Chou |
  | Author | Saravanan Vivekanandan |
  | Author | Prasanna Kolatkar |
  | URL | http://www.ingentaconnect.com/content/ben/ppl/2012/00000019/00000001/art00008 |
  | Volume | 19 |
  | Issue | 1 |
  | Pages | 50–56 |
  | Publication | Protein and peptide letters |
  | Date | 2012 |
  | Accessed | 9/20/2013, 1:16:24 PM |
  | Library Catalog | Google Scholar |
  | Short Title | RSARF |
  | Date Added | 10/11/2013, 10:29:15 AM |
  | Modified | 10/11/2013, 10:29:15 AM |

  ### Tags:

  - Accessible surface area
  - conserved residue
  - functional residue
  - hydrophobic core
  - protein interface
  - protein structure prediction

  ### Notes:

  - Paper unavailable.
- ## SAS-Pro: simultaneous residue assignment and structure superposition for protein structure alignment

  |  |  |
  | --- | --- |
  | Type | Journal Article |
  | Author | Shweta B Shah |
  | Author | Nikolaos V Sahinidis |
  | Volume | 7 |
  | Issue | 5 |
  | Pages | e37493 |
  | Publication | PloS one |
  | ISSN | 1932-6203 |
  | Date | 2012 |
  | Extra | PMID: 22662161 |
  | Journal Abbr | PLoS ONE |
  | DOI | 10.1371/journal.pone.0037493 |
  | Library Catalog | NCBI PubMed |
  | Language | eng |
  | Abstract | Protein structure alignment is the problem of determining an assignment between the amino-acid residues of two given proteins in a way that maximizes a measure of similarity between the two superimposed protein structures. By identifying geometric similarities, structure alignment algorithms provide critical insights into protein functional similarities. Existing structure alignment tools adopt a two-stage approach to structure alignment by decoupling and iterating between the assignment evaluation and structure superposition problems. We introduce a novel approach, SAS-Pro, which addresses the assignment evaluation and structure superposition simultaneously by formulating the alignment problem as a single bilevel optimization problem. The new formulation does not require the sequentiality constraints, thus generalizing the scope of the alignment methodology to include non-sequential protein alignments. We employ derivative-free optimization methodologies for searching for the global optimum of the highly nonlinear and non-differentiable RMSD function encountered in the proposed model. Alignments obtained with SAS-Pro have better RMSD values and larger lengths than those obtained from other alignment tools. For non-sequential alignment problems, SAS-Pro leads to alignments with high degree of similarity with known reference alignments. The source code of SAS-Pro is available for download at http://eudoxus.cheme.cmu.edu/saspro/SAS-Pro.html. |
  | Short Title | SAS-Pro |
  | Date Added | 10/11/2013, 10:29:15 AM |
  | Modified | 3/7/2014, 1:06:57 PM |

  ### Tags:

  - Amino Acids
  - Computational Biology
  - Internet
  - Protein Conformation
  - Proteins
  - Software

  ### Notes:

  - Present a new structure alignment method, SAS-Pro.

    How SCOP is used:

    Evaluate alignment method on a previously published data set of 40 proteins from four different SCOP folds.  Doesn't appear that they are using SCOP classification in their validation though.

    How CATH is used:

    Background on protein structure classification.

    SCOP/CATH reference:

    These tools have been instrumental in the development of various protein structure databases like FSSP [16], SCOP [17], CATH [18] and HOMSTRAD [19], which provide extensive information on classification of protein folds and domains.

    ...

    We performed computational experiments based on three data sets:

    - the Sokol data set [44], which is a set of 9 small size proteins with proteins from three different fold families,
    - the Skolnick data set [20], which is a set of 40 large globular proteins from four different fold families from the SCOP data base, and
    - the RIPC data set [45], which is a set of 23 complex structure alignment problems.

  ### Attachments

  - journal.pone.0037493.pdf
- ## Scalable web services for the PSIPRED Protein Analysis Workbench

  |  |  |
  | --- | --- |
  | Type | Journal Article |
  | Author | Daniel W. A. Buchan |
  | Author | Federico Minneci |
  | Author | Tim C. O. Nugent |
  | Author | Kevin Bryson |
  | Author | David T. Jones |
  | Volume | 41 |
  | Issue | W1 |
  | Pages | W349–W357 |
  | Publication | Nucleic Acids Research |
  | Date | July 2013 |
  | DOI | 10.1093/nar/gkt381 |
  | Abstract | Here, we present the new UCL Bioinformatics Group's PSIPRED Protein Analysis Workbench. The Workbench unites all of our previously available analysis methods into a single web-based frame-work. The new web portal provides a greatly streamlined user interface with a number of new features to allow users to better explore their results. We offer a number of additional services to enable computationally scalable execution of our prediction methods; these include SOAP and XML-RPC web server access and new HADOOP packages. All software and services are available via the UCL Bioinformatics Group website at http://bioinf.cs.ucl.ac.uk/. |
  | Date Added | 3/7/2014, 12:08:00 PM |
  | Modified | 3/7/2014, 12:08:00 PM |
- ## Scaling laws in simple and complex proteins: size scaling effects associated with domain number and folding class

  |  |  |
  | --- | --- |
  | Type | Journal Article |
  | Author | Parker Rogerson |
  | Author | Gustavo A. Arteca |
  | URL | http://link.springer.com/article/10.1007/s10910-012-0010-1 |
  | Volume | 50 |
  | Issue | 7 |
  | Pages | 1901–1919 |
  | Publication | Journal of Mathematical Chemistry |
  | Date | 2012 |
  | Accessed | 9/23/2013, 10:20:20 AM |
  | Library Catalog | Google Scholar |
  | Short Title | Scaling laws in simple and complex proteins |
  | Date Added | 10/11/2013, 10:29:15 AM |
  | Modified | 3/7/2014, 12:11:16 PM |

  ### Tags:

  - Folding families
  - Polymer size
  - protein domains
  - Protein folds
  - SCOP database

  ### Notes:

  - Study of domain compactness.

    How SCOP is used:

    Curate a non-redundant data set using their own criteria and SCOP data so they retain at most one entry per "SCOP classification" (assume to be sccs).

    Calculate statistics on the "summary of gyration" to compare domains from single and multi-domain proteins.  Extensively make use of SCOP class in this equation.

    SCOP reference:

    Our objective is to determine if there is a difference in the packing arrangement of domains derived from single-domain proteins and domains located within multi- domain proteins, and to evaluate these differences in terms of the four major folding classes. To this end, we rely on a subclass of independent, nonreduntant domains extracted from the PDB, and organized by the SCOP data base, in order to study their size-scaling regimes.

    The SCOP and PDB archives contain a enormous number of entries, and, at the same time, they are highly redundant. This redundancy may take the form of closely related sequences, or multiple structural entries associated with distinct resolutions, experimental methodology, temperature, or type of ligand binding. In order to avoid biases in the scaling behaviour, it is necessary to curtail this multiplicity. Duplication is eliminated by using an appropriate set of criteria to ensure only one entry per domain type. The following protocol was used to filter out redundancy and create our data set:

    1. (a)  Only single chains are considered (that is, quaternary structure is omitted in our analysis). If multiple data are available for the same chain, we chose the structure with the best resolution. In the case of X-ray data, structures with resolution above 3.2 Å were omitted. Proteins with missing residues or poorly-resolved areas were also excluded from our set.
    2. (b)  Chains under 35 residues are omitted, as they tend to resemble unstructured polypeptides.
    3. (c)  Proteins with the same chain length and over 90 % sequence identity are represented by a single entry.
    4. (d)  Domains with > 90 % sequence identity, yet differing in more than 15 amino acids in chain length, are considered distinct entries.
    5. (e)  Due to the fact that termini regions are often labile, we allow chains to differ by up to a 12-residue segment at one end of the protein.

    Using these criteria, we retain only one entry within such an ensemble for domains and chains belonging to the same SCOP classification.

    We started our analysis with a total of 85,686 protein domains in the four major folding classes. After reorganizing the structures in the SCOP data base with the above criteria, we ended up with an ensemble of 8,614 non-redundant individual domains with the following breakdown in terms of folding classes (FC): all-α (1,741 out of 14,824), all-β (2,527 out of 23,547), α + β (2,099 out of 21,499), and α/β (2,247 out of 25,816). Finally, we have reclassified these units according to provenance, i.e., the number of domains in the original intact protein chain. In order to study size scal- ing, each of these subgroups is reclassified in turn according to their Rg-value by the binning processes explained in the next section.

    Note that while the SCOP data base includes a “multi-domain protein” section, it does not link these complex proteins to their constituent domains. This mapping is a necessary step in our analysis, permitting us to study the effect of domain provenance on size scaling. Multi-domain single-chain analysis was carried out after subjecting this data set to the same redundancy elimination protocol used for single domains.

  ### Attachments

  - art%3A10.1007%2Fs10910-012-0010-1.pdf
  - Snapshot

    ## Abstract

    The native states of the most compact globular proteins have been described as being in the so-called “collapsed-polymer regime,” characterized by the scaling law *R*  _*g*_  ~ *n* ^ν^, where *R*  _*g*_  is radius of gyration, *n* is the number of residues, and ν ≈ 1/3. However, the diversity of folds and the plasticity of native states suggest that this law may not be universal. In this work, we study the scaling regimes of: (*i*) one to four-domain protein chains, and (*ii*) their constituent domains, in terms of the four major folding classes. In the case of complete chains, we show that size scaling is influenced by the number of domains. For the set of domains belonging to the all-*α*, all-*β, α*/*β*, and *α + β* folding classes, we find that size-scaling exponents vary between 0.3 ≤ ν ≤ 0.4. Interestingly, even domains in the same folding class show scaling regimes that are sensitive to domain provenance, *i.e*., the number of domains present in the original intact chain. We demonstrate that the level of compactness, as measured by monomer density, decreases when domains originate from increasingly complex proteins.
- ## SCO4008, a Putative TetR Transcriptional Repressor from Streptomyces coelicolor A3(2), Regulates Transcription of sco4007 by Multidrug Recognition

  |  |  |
  | --- | --- |
  | Type | Journal Article |
  | Author | Takeshi Hayashi |
  | Author | Yoshikazu Tanaka |
  | Author | Naoki Sakai |
  | Author | Ui Okada |
  | Author | Min Yao |
  | Author | Nobuhisa Watanabe |
  | Author | Tomohiro Tamura |
  | Author | Isao Tanaka |
  | Volume | 425 |
  | Issue | 18 |
  | Pages | 3289–3300 |
  | Publication | Journal of Molecular Biology |
  | Date | September 2013 |
  | DOI | 10.1016/j.jmb.2013.06.013 |
  | Abstract | SCO4008 from Streptomyces coelicolor A3(2) is a member of the TetR family. However, its precise function is not yet clear. In this study, the crystal structure of SCO4008 was determined at a resolution of 2.3 angstrom, and its DNA-binding properties were analyzed. Crystal structure analysis showed that SCO4008 forms an Omega-shaped homodimer in which the monomer is composed of an N-terminal DNA-binding domain containing a helix-turn-helix and a C-terminal dimerization and regulatory domain possessing a ligand-binding cavity. The genomic systematic evolution of ligands by exponential enrichment and electrophoretic mobility shift assay revealed that four SCO4008 dimers bind to the two operator regions located between sco4008 and sco4007, a secondary transporter belonging to the major facilitator superfamily. Ligand screening analysis showed that SCO4008 recognizes a wide range of structurally dissimilar cationic and hydrophobic compounds. These results suggested that SCO4008 is a transcriptional repressor of sco4007 responsible for the multidrug resistance system in S. coelicolor A3(2). (C) 2013 Elsevier Ltd. All rights reserved. |
  | Date Added | 3/7/2014, 1:06:24 PM |
  | Modified | 3/7/2014, 1:06:24 PM |
- ## SCOP: a Structural Classification of Proteins database

  |  |  |
  | --- | --- |
  | Type | Journal Article |
  | Author | T. J. Hubbard |
  | Author | B. Ailey |
  | Author | S. E. Brenner |
  | Author | A. G. Murzin |
  | Author | C. Chothia |
  | Volume | 27 |
  | Issue | 1 |
  | Pages | 254-256 |
  | Publication | Nucleic Acids Research |
  | ISSN | 0305-1048 |
  | Date | Jan 1, 1999 |
  | Extra | PMID: 9847194 PMCID: PMC148149 |
  | Journal Abbr | Nucleic Acids Res. |
  | Library Catalog | NCBI PubMed |
  | Language | eng |
  | Abstract | The Structural Classification of Proteins (SCOP) database provides a detailed and comprehensive description of the relationships of all known proteins structures. The classification is on hierarchical levels: the first two levels, family and superfamily, describe near and far evolutionary relationships; the third, fold, describes geometrical relationships. The distinction between evolutionary relationships and those that arise from the physics and chemistry of proteins is a feature that is unique to this database, so far. The database can be used as a source of data to calibrate sequence search algorithms and for the generation of population statistics on protein structures. The database and its associated files are freely accessible from a number of WWW sites mirrored from URL http://scop. mrc-lmb.cam.ac.uk/scop/ |
  | Short Title | SCOP |
  | Date Added | 12/9/2014, 3:54:22 AM |
  | Modified | 12/9/2014, 3:54:22 AM |

  ### Tags:

  - Algorithms
  - Databases, Factual
  - Evolution, Molecular
  - Information Storage and Retrieval
  - Internet
  - Protein Conformation
  - Protein Folding
  - Proteins
  - Sequence Alignment
  - Sequence Homology, Amino Acid
  - Statistics as Topic

  ### Attachments

  - PubMed entry
- ## SCOP: a structural classification of proteins database

  |  |  |
  | --- | --- |
  | Type | Journal Article |
  | Author | T. J. Hubbard |
  | Author | A. G. Murzin |
  | Author | S. E. Brenner |
  | Author | C. Chothia |
  | Volume | 25 |
  | Issue | 1 |
  | Pages | 236-239 |
  | Publication | Nucleic Acids Research |
  | ISSN | 0305-1048 |
  | Date | Jan 1, 1997 |
  | Extra | PMID: 9016544 PMCID: PMC146380 |
  | Journal Abbr | Nucleic Acids Res. |
  | Library Catalog | NCBI PubMed |
  | Language | eng |
  | Abstract | The Structural Classification of Proteins (SCOP) database provides a detailed and comprehensive description of the relationships of all known proteins structures. The classification is on hierarchical levels: the first two levels, family and superfamily, describe near and far evolutionary relationships; the third, fold, describes geometrical relationships. The distinction between evolutionary relationships and those that arise from the physics and chemistry of proteins is a feature that is unique to this database, so far. SCOP also provides for each structure links to atomic co-ordinates, images of the structures, interactive viewers, sequence data, data on any conformational changes related to function and literature references. The database is freely accessible on the World Wide Web (WWW) with an entry point at URL http://scop.mrc-lmb.cam.ac.uk/scop/ |
  | Short Title | SCOP |
  | Date Added | 12/9/2014, 3:54:42 AM |
  | Modified | 12/9/2014, 3:54:42 AM |

  ### Tags:

  - Amino Acid Sequence
  - Databases, Factual
  - Protein Folding
  - Proteins
  - Protein Structure, Secondary
  - Protein Structure, Tertiary

  ### Attachments

  - PubMed entry
- ## SCOP: a structural classification of proteins database

  |  |  |
  | --- | --- |
  | Type | Journal Article |
  | Author | L. Lo Conte |
  | Author | B. Ailey |
  | Author | T. J. Hubbard |
  | Author | S. E. Brenner |
  | Author | A. G. Murzin |
  | Author | C. Chothia |
  | Volume | 28 |
  | Issue | 1 |
  | Pages | 257-259 |
  | Publication | Nucleic Acids Research |
  | ISSN | 0305-1048 |
  | Date | Jan 1, 2000 |
  | Extra | PMID: 10592240 PMCID: PMC102479 |
  | Journal Abbr | Nucleic Acids Res. |
  | Library Catalog | NCBI PubMed |
  | Language | eng |
  | Abstract | The Structural Classification of Proteins (SCOP) database provides a detailed and comprehensive description of the relationships of known protein structures. The classification is on hierarchical levels: the first two levels, family and superfamily, describe near and distant evolutionary relationships; the third, fold, describes geometrical relationships. The distinction between evolutionary relationships and those that arise from the physics and chemistry of proteins is a feature that is unique to this database so far. The sequences of proteins in SCOP provide the basis of the ASTRAL sequence libraries that can be used as a source of data to calibrate sequence search algorithms and for the generation of statistics on, or selections of, protein structures. Links can be made from SCOP to PDB-ISL: a library containing sequences homologous to proteins of known structure. Sequences of proteins of unknown structure can be matched to distantly related proteins of known structure by using pairwise sequence comparison methods to find homologues in PDB-ISL. The database and its associated files are freely accessible from a number of WWW sites mirrored from URL http://scop.mrc-lmb.cam.ac.uk/scop/ |
  | Short Title | SCOP |
  | Date Added | 12/9/2014, 3:53:49 AM |
  | Modified | 12/9/2014, 3:53:49 AM |

  ### Tags:

  - Databases, Factual
  - Evolution, Molecular
  - Information Storage and Retrieval
  - Internet
  - Protein Conformation
  - Proteins

  ### Attachments

  - PubMed entry
- ## SCOP: a structural classification of proteins database for the investigation of sequences and structures

  |  |  |
  | --- | --- |
  | Type | Journal Article |
  | Author | A. G. Murzin |
  | Author | S. E. Brenner |
  | Author | T. Hubbard |
  | Author | C. Chothia |
  | Volume | 247 |
  | Issue | 4 |
  | Pages | 536-540 |
  | Publication | Journal of Molecular Biology |
  | ISSN | 0022-2836 |
  | Date | Apr 7, 1995 |
  | Extra | PMID: 7723011 |
  | Journal Abbr | J. Mol. Biol. |
  | DOI | 10.1006/jmbi.1995.0159 |
  | Library Catalog | NCBI PubMed |
  | Language | eng |
  | Abstract | To facilitate understanding of, and access to, the information available for protein structures, we have constructed the Structural Classification of Proteins (scop) database. This database provides a detailed and comprehensive description of the structural and evolutionary relationships of the proteins of known structure. It also provides for each entry links to co-ordinates, images of the structure, interactive viewers, sequence data and literature references. Two search facilities are available. The homology search permits users to enter a sequence and obtain a list of any structures to which it has significant levels of sequence similarity. The key word search finds, for a word entered by the user, matches from both the text of the scop database and the headers of Brookhaven Protein Databank structure files. The database is freely accessible on World Wide Web (WWW) with an entry point to URL http: parallel scop.mrc-lmb.cam.ac.uk magnitude of scop. |
  | Short Title | SCOP |
  | Date Added | 11/3/2014, 2:45:55 PM |
  | Modified | 11/3/2014, 2:45:55 PM |

  ### Tags:

  - Amino Acid Sequence
  - Databases, Factual
  - Protein Folding
  - Proteins
  - Sequence Analysis

  ### Attachments

  - PubMed entry
- ## SCOPe: Structural Classification of Proteins--extended, integrating SCOP and ASTRAL data and classification of new structures

  |  |  |
  | --- | --- |
  | Type | Journal Article |
  | Author | Naomi K. Fox |
  | Author | Steven E. Brenner |
  | Author | John-Marc Chandonia |
  | Volume | 42 |
  | Issue | Database issue |
  | Pages | D304-309 |
  | Publication | Nucleic Acids Research |
  | ISSN | 1362-4962 |
  | Date | Jan 2014 |
  | Extra | PMID: 24304899 PMCID: PMC3965108 |
  | Journal Abbr | Nucleic Acids Res. |
  | DOI | 10.1093/nar/gkt1240 |
  | Library Catalog | NCBI PubMed |
  | Language | eng |
  | Abstract | Structural Classification of Proteins-extended (SCOPe, http://scop.berkeley.edu) is a database of protein structural relationships that extends the SCOP database. SCOP is a manually curated ordering of domains from the majority of proteins of known structure in a hierarchy according to structural and evolutionary relationships. Development of the SCOP 1.x series concluded with SCOP 1.75. The ASTRAL compendium provides several databases and tools to aid in the analysis of the protein structures classified in SCOP, particularly through the use of their sequences. SCOPe extends version 1.75 of the SCOP database, using automated curation methods to classify many structures released since SCOP 1.75. We have rigorously benchmarked our automated methods to ensure that they are as accurate as manual curation, though there are many proteins to which our methods cannot be applied. SCOPe is also partially manually curated to correct some errors in SCOP. SCOPe aims to be backward compatible with SCOP, providing the same parseable files and a history of changes between all stable SCOP and SCOPe releases. SCOPe also incorporates and updates the ASTRAL database. The latest release of SCOPe, 2.03, contains 59 514 Protein Data Bank (PDB) entries, increasing the number of structures classified in SCOP by 55% and including more than 65% of the protein structures in the PDB. |
  | Short Title | SCOPe |
  | Date Added | 10/13/2014, 12:15:03 PM |
  | Modified | 10/13/2014, 12:15:03 PM |

  ### Tags:

  - Databases, Protein
  - Internet
  - Proteins
  - Protein Structure, Tertiary
  - Systems Integration

  ### Attachments

  - PubMed entry
- ## SCOP, Structural Classification of Proteins database: applications to evaluation of the effectiveness of sequence alignment methods and statistics of protein structural data

  |  |  |
  | --- | --- |
  | Type | Journal Article |
  | Author | T. J. Hubbard |
  | Author | B. Ailey |
  | Author | S. E. Brenner |
  | Author | A. G. Murzin |
  | Author | C. Chothia |
  | Volume | 54 |
  | Issue | Pt 6 Pt 1 |
  | Pages | 1147-1154 |
  | Publication | Acta Crystallographica. Section D, Biological Crystallography |
  | ISSN | 0907-4449 |
  | Date | Nov 1, 1998 |
  | Extra | PMID: 10089491 |
  | Journal Abbr | Acta Crystallogr. D Biol. Crystallogr. |
  | Library Catalog | NCBI PubMed |
  | Language | eng |
  | Abstract | The Structural Classification of Proteins (SCOP) database provides a detailed and comprehensive description of the relationships of all known protein structures. The classification is on hierarchical levels: the first two levels, family and superfamily, describe near and far evolutionary relationships; the third, fold, describes geometrical relationships. The distinction between evolutionary relationships and those that arise from the physics and chemistry of proteins is a feature that is unique to this database, so far. The database can be used as a source of data to calibrate sequence search algorithms and for the generation of population statistics on protein structures. The database and its associated files are freely accessible from a number of WWW sites mirrored from URL http://scop. mrc-lmb.cam.ac.uk/scop/. |
  | Short Title | SCOP, Structural Classification of Proteins database |
  | Date Added | 12/9/2014, 3:54:06 AM |
  | Modified | 12/9/2014, 3:54:06 AM |

  ### Tags:

  - Algorithms
  - Amino Acid Sequence
  - Database Management Systems
  - Databases, Factual
  - Evaluation Studies as Topic
  - Molecular Sequence Data
  - Protein Conformation
  - Protein Folding
  - Sequence Alignment

  ### Attachments

  - PubMed entry
- ## ScrewFit: combining localization and description of protein secondary structure

  |  |  |
  | --- | --- |
  | Type | Journal Article |
  | Author | Paolo A. Calligari |
  | Author | Gerald R. Kneller |
  | URL | http://scripts.iucr.org/cgi-bin/paper?S0907444912039029 |
  | Volume | 68 |
  | Issue | 12 |
  | Pages | 1690–1693 |
  | Publication | Acta Crystallographica Section D: Biological Crystallography |
  | Date | 2012 |
  | Accessed | 9/23/2013, 10:23:40 AM |
  | Library Catalog | Google Scholar |
  | Short Title | ScrewFit |
  | Date Added | 10/11/2013, 10:29:15 AM |
  | Modified | 3/7/2014, 12:15:10 PM |

  ### Notes:

  - Extend the ScrewFit algorithm for providing a 'geometrical description' of protein conformation to also perform secondary structure prediction.

    How SCOP is used:

    Use ASTRAL dataset filtered at 40% sequence identity, to train parameters of the ScrewFit algorithm.  The motivation is to get structural variety in secondary structure geometries in order to compute confidence intervals for the ScrewFit parameters associated with different structural motifs.

    SCOP reference:

    2. Secondary-structure assignments

    Secondary-structure motifs are generally defined with respect to the regular winding of the main chain in model polypeptides, which is associated with specific hydrogen-bond patterns. However, significant deviations from the ideal conformations of these motifs are found in experimentally determined protein structures. This structural variety can be used to establish confidence intervals for the ScrewFit para- meters which are associated with a given structural motif. For this purpose, we analyzed 1027 ⬚⬚-helices and 1336 ⬚⬚-strands from the SCOP+ASTRAL database (Chandonia et al., 2004), which contains the coordinates of secondary-structure elements for each domain classified according to the SCOP fold classes (Murzin et al., 1995). The motifs are taken from proteins with less than 40% identity in the amino-acid sequence. In the following, we refer to the coordinate subsets for ⬚⬚-helices and ⬚⬚-strands as A and B, respectively.

  ### Attachments

  - [PDF] from cnrs-orleans.fr
- ## Searching for Likeness in a Database of Macromolecular Complexes

  |  |  |
  | --- | --- |
  | Type | Journal Article |
  | Author | Jeffrey R. Van Voorst |
  | Author | Barry C. Finzel |
  | Volume | 53 |
  | Issue | 10 |
  | Pages | 2634–2647 |
  | Publication | Journal of Chemical Information and Modeling |
  | Date | October 2013 |
  | DOI | 10.1021/ci4002537 |
  | Abstract | A software tool and workflow based on distance geometry is presented that can be used to search for local similarity in substructures in a comprehensive database of experimentally derived macromolecular structure. The method does not rely on fold annotation, specific secondary structure assignments, or sequence homology and may be used to locate compound substructures of multiple segments spanning different macromolecules that share a queried backbone geometry. This generalized substructure searching capability is intended to allow users to play an active part in exploring the role specific substructures play in larger protein domains, quaternary assemblies of proteins, and macromolecular complexes of proteins and polynucleotides. The user may select any portion or portions of an existing structure or complex to serve as a template for searching, and other structures that share the same structural features are identified, retrieved and overlaid to emphasize substructural likeness. Matching structures may be compared using a variety of integrated tools including molecular graphics for structure visualization and matching substructure sequence logos. A number of examples are provided that illustrate how generalized substructure searching may be used to understand both the similarity, and individuality of specific macromolecular structures. Web-based access to our substructure searching services is freely available at https://drugsite.msi.umn.edu. |
  | Date Added | 3/7/2014, 1:06:24 PM |
  | Modified | 10/8/2014, 1:32:40 PM |

  ### Attachments

  - ACS Full Text PDF w/ Links
  - ACS Full Text Snapshot
- ## Searching for protein signatures using a multilevel alphabet

  |  |  |
  | --- | --- |
  | Type | Journal Article |
  | Author | Ronit Hod |
  | Author | Refael Kohen |
  | Author | Yael Mandel-Gutfreund |
  | URL | http://onlinelibrary.wiley.com/doi/10.1002/prot.24261/full |
  | Publication | Proteins: Structure, Function, and Bioinformatics |
  | Date | 2013 |
  | Accessed | 9/23/2013, 10:15:21 AM |
  | Library Catalog | Google Scholar |
  | Date Added | 10/11/2013, 10:29:15 AM |
  | Modified | 10/11/2013, 10:29:15 AM |

  ### Tags:

  - motif search
  - multilevel alphabet
  - protein disorder
  - secondary structure
  - surface accessibility

  ### Notes:

  - Present a new method to represent amino acid sequence with a new alphabet, reflecting "instrinsic structural and chemical properties" then apply MEME search algorithm to detect motifs.

    Demonstrate that they can detect the amphipathic helix structural motif.

    How SCOP data is used:

    In order to assess the  specificity and sensitivity at detecting alpha helices, they tested their method on domain datasets of "all-beta" folds, as classified by SCOP.  Downloaded datasets from PDB that are categorized by SCOP as all-beta, to serve "as the control".  Found that many of the all-beta folds indeed contained alpha-helices.

    Reference to SCOP:

    In addition, 10 control sets were extracted, each including 20 protein domains from the PDB annotated in SCOP (Version 1.75)43 as ‘‘all beta’’ domains.

     ...

    To further evaluate the statistical power of our method, we selected ten random control sets of 20 protein domains from the PDB that were defined in SCOP as ‘‘all beta’’ and were thus not expected to contain AHs.

  ### Attachments

  - [PDF] from technion.ac.il
- ## Searching protein structure databases with DaliLite v.3

  |  |  |
  | --- | --- |
  | Type | Journal Article |
  | Author | L Holm |
  | Author | S Kääriäinen |
  | Author | P Rosenström |
  | Author | A Schenkel |
  | Volume | 24 |
  | Issue | 23 |
  | Pages | 2780-2781 |
  | Publication | Bioinformatics |
  | ISSN | 1367-4811 |
  | Date | Dec 1, 2008 |
  | Extra | PMID: 18818215 |
  | Journal Abbr | Bioinformatics |
  | DOI | 10.1093/bioinformatics/btn507 |
  | Library Catalog | NCBI PubMed |
  | Language | eng |
  | Abstract | The Red Queen said, 'It takes all the running you can do, to keep in the same place.' Lewis Carrol MOTIVATION: Newly solved protein structures are routinely scanned against structures already in the Protein Data Bank (PDB) using Internet servers. In favourable cases, comparing 3D structures may reveal biologically interesting similarities that are not detectable by comparing sequences. The number of known structures continues to grow exponentially. Sensitive-thorough but slow-search algorithms are challenged to deliver results in a reasonable time, as there are now more structures in the PDB than seconds in a day. The brute-force solution would be to distribute the individual comparisons on a massively parallel computer. A frugal solution, as implemented in the Dali server, is to reduce the total computational cost by pruning search space using prior knowledge about the distribution of structures in fold space. This note reports paradigm revisions that enable maintaining such a knowledge base up-to-date on a PC. AVAILABILITY: The Dali server for protein structure database searching at http://ekhidna.biocenter.helsinki.fi/dali\_server is running DaliLite v.3. The software can be downloaded for academic use from http://ekhidna.biocenter.helsinki.fi/dali\_lite/downloads/v3. |
  | Date Added | 10/11/2013, 10:29:15 AM |
  | Modified | 10/29/2013, 9:33:45 AM |

  ### Notes:

  - DALI server for 3D structure alignment.

    How SCOP is used:

    Benchmark the method on a curated representative set of first four SCOP classes.

    SCOP reference:

    The utility of a protein structure database search method (i.e. similarity measure and optimization algorithm) must depend on its ability to report back ‘interesting’ matches. As an illustration, we chose query and target structures representing diverse super- families from the four main structural classes in SCOP: cytochromes c and winged helix DNA-binding domains from the all-alpha class, cupredoxins and PUA-like domains from the all-beta class, metallo- dependent hydrolases and alpha/beta hydrolases from the alpha/beta class, and lysozyme-likes and nucleotidyltransferases from the alpha + beta class (Table 1).

  ### Attachments

  - Full Text PDF
- ## Secondary-structure matching (SSM), a new tool for fast protein structure alignment in three dimensions

  |  |  |
  | --- | --- |
  | Type | Journal Article |
  | Author | E. Krissinel |
  | Author | K. Henrick |
  | URL | http://scripts.iucr.org/cgi-bin/paper?S0907444904026460 |
  | Volume | 60 |
  | Issue | 12 |
  | Pages | 2256–2268 |
  | Publication | Acta Crystallographica Section D: Biological Crystallography |
  | Date | 2004 |
  | Accessed | 10/10/2013, 1:18:50 PM |
  | Library Catalog | Google Scholar |
  | Date Added | 10/11/2013, 10:29:15 AM |
  | Modified | 10/11/2013, 10:29:15 AM |

  ### Tags:

  - likely ASTRAL
  - likely ASTRAL domain structures
  - likely ASTRAL sequences

  ### Notes:

  - SSM: secondary-structure matching algorithm that improves accuracy of 3D structure alignment methods.

    How SCOP is used:

    Use SCOP data to help train parameters for fold identification method.  Used SCOP folds from SCOP 1.61.

    SCOP reference:

    This recurrence starts from the function &1⬚⬚x⬚⬚, which is calculated empirically by running SSM on all pairs of non-redundant protein structures [we used SCOP folds as found in SCOP Version 1.61 (Murzin et al., 1995)].

  ### Attachments

  - [PDF] from ebi.ac.uk
  - Snapshot
- ## Secondary-structure matching (SSM), a new tool for fast protein structure alignment in three dimensions

  |  |  |
  | --- | --- |
  | Type | Journal Article |
  | Author | E. Krissinel |
  | Author | K. Henrick |
  | URL | http://scripts.iucr.org/cgi-bin/paper?ba5056 |
  | Volume | 60 |
  | Issue | 12 |
  | Pages | 2256-2268 |
  | Publication | Acta Crystallographica Section D Biological Crystallography |
  | ISSN | 0907-4449 |
  | Date | 2004-12-01 |
  | DOI | 10.1107/S0907444904026460 |
  | Accessed | 10/28/2014, 2:35:51 PM |
  | Library Catalog | CrossRef |
  | Date Added | 10/28/2014, 2:36:05 PM |
  | Modified | 10/28/2014, 2:36:05 PM |

  ### Attachments
- ## Secondary structure of proteins analyzed ex vivo in vascular wall in diabetic animals using FT-IR spectroscopy

  |  |  |
  | --- | --- |
  | Type | Journal Article |
  | Author | Katarzyna Majzner |
  | Author | Tomasz P. Wrobel |
  | Author | Andrzej Fedorowicz |
  | Author | Stefan Chlopicki |
  | Author | Malgorzata Baranska |
  | Volume | 138 |
  | Issue | 24 |
  | Pages | 7400-7410 |
  | Publication | Analyst |
  | ISSN | 0003-2654; 1364-5528 |
  | Date | 2013 |
  | Extra | WOS:000326988500019 |
  | DOI | 10.1039/c3an00455d |
  | Abstract | In recent years many methods for ex vivo tissue analysis or diagnosis of diseases have been applied, including infrared absorption spectroscopy. Fourier-transform infrared (FT-IR) absorption microspectroscopy allows the simultaneous monitoring of the content of various chemical compounds in tissues with both high selectivity and resolution. Imaging of tissue samples in very short time can be performed using a spectrometer equipped with a Focal Plane Array (FPA) detector. Additionally, a detection of minor components or subtle changes associated with the functional status of a tissue sample is possible when advanced methods of data analysis, such as chemometric techniques, are applied. Monitoring of secondary structures of proteins has already proved to be useful in the analysis of animal tissues in disease states. The aim of this work was to build a mathematical model based on FT-IR measurements for the prediction of alterations in the content of secondary structures of proteins analyzed by FT-IR in the vascular wall of diabetic animals. For that purpose a spectral database of proteins of known crystallography and secondary structures was assembled. Thirty-seven proteins were measured by means of two FT-IR techniques: transflection and Attenuated Total Reflectance (ATR). The obtained model was tested on cross-sections of rat tail, for which the content of proteins and their secondary structures was well characterized. Then, the model was applied for the detection of possible alterations in the secondary structures of proteins in the vascular wall of diabetic rats and mice. The obtained results suggest a prominent increase in E-and S-structures and a decrease in the content of H-structures in the vascular wall from diabetic mice and rats. FT-IR-based studies of secondary structures of proteins may be a novel approach to study complex processes ongoing in the vascular wall. The obtained results are satisfactory; however, the existing limitations of the method are also discussed. |
  | Date Added | 2/13/2014, 4:13:17 PM |
  | Modified | 3/7/2014, 12:09:15 PM |
- ## Secreted Proteases Control Autolysin-mediated Biofilm Growth of Staphylococcus aureus

  |  |  |
  | --- | --- |
  | Type | Journal Article |
  | Author | Chen Chen |
  | Author | Vengadesan Krishnan |
  | Author | Kevin Macon |
  | Author | Kartik Manne |
  | Author | Sthanam V. L. Narayana |
  | Author | Olaf Schneewind |
  | Volume | 288 |
  | Issue | 41 |
  | Pages | 29440–29452 |
  | Publication | Journal of Biological Chemistry |
  | Date | October 2013 |
  | DOI | 10.1074/jbc.M113.502039 |
  | Abstract | Staphylococcus epidermidis, a commensal of humans, secretes Esp protease to prevent Staphylococcus aureus biofilm formation and colonization. Blocking S. aureus colonization may reduce the incidence of invasive infectious diseases; however, the mechanism whereby Esp disrupts biofilms is unknown. We show here that Esp cleaves autolysin (Atl)-derived murein hydrolases and prevents staphylococcal release of DNA, which serves as extracellular matrix in biofilms. The three-dimensional structure of Esp was revealed by x-ray crystallography and shown to be highly similar to that of S. aureus V8 (SspA). Both atl and sspA are necessary for biofilm formation, and purified SspA cleaves Atl-derived murein hydrolases. Thus, S. aureus biofilms are formed via the controlled secretion and proteolysis of autolysin, and this developmental program appears to be perturbed by the Esp protease of S. epidermidis. |
  | Date Added | 3/7/2014, 1:06:24 PM |
  | Modified | 3/7/2014, 1:06:24 PM |
- ## Selective constraint on human pre-mRNA splicing by protein structural properties

  |  |  |
  | --- | --- |
  | Type | Journal Article |
  | Author | Jean-Christophe Gelly |
  | Author | Hsuan-Yu Lin |
  | Author | Alexandre G de Brevern |
  | Author | Trees-Juen Chuang |
  | Author | Feng-Chi Chen |
  | Volume | 4 |
  | Issue | 9 |
  | Pages | 966-975 |
  | Publication | Genome biology and evolution |
  | ISSN | 1759-6653 |
  | Date | 2012 |
  | Extra | PMID: 22936073 |
  | Journal Abbr | Genome Biol Evol |
  | DOI | 10.1093/gbe/evs071 |
  | Library Catalog | NCBI PubMed |
  | Language | eng |
  | Abstract | Alternative splicing (AS) is a major mechanism of increasing proteome diversity in complex organisms. Different AS transcript isoforms may be translated into peptide sequences of significantly different lengths and amino acid compositions. One important question, then, is how AS is constrained by protein structural requirements while peptide sequences may be significantly changed in AS events. Here, we address this issue by examining whether the intactness of three-dimensional protein structural units (compact units in protein structures, namely protein units [PUs]) tends to be preserved in AS events in human. We show that PUs tend to occur in constitutively spliced exons and to overlap constitutive exon boundaries. Furthermore, when PUs are located at the boundaries between two alternatively spliced exons (ASEs), these neighboring ASEs tend to co-occur in different transcript isoforms. In addition, such PU-spanned ASE pairs tend to have a higher frequency of being included in transcript isoforms. ASE regions that overlap with PUs also have lower nonsynonymous-to-synonymous substitution rate ratios than those that do not overlap with PUs, indicating stronger negative selection pressure in PU-overlapped ASE regions. Of note, we show that PUs have protein domain- and structural orderness-independent effects on messenger RNA (mRNA) splicing. Overall, our results suggest that fine-scale protein structural requirements have significant influences on the splicing patterns of human mRNAs. |
  | Date Added | 10/11/2013, 10:29:15 AM |
  | Modified | 3/7/2014, 1:07:09 PM |

  ### Tags:

  - Alternative Splicing
  - Alternative Splicing
  - Evolution, Molecular
  - Exons
  - Gene Frequency
  - Humans
  - protein structural constraint
  - Protein Structure, Quaternary
  - Protein Structure, Tertiary
  - protein unit
  - Proteomics
  - RNA, Messenger
  - RNA Precursors
  - Selection, Genetic

  ### Notes:

  - Study alternative splicing.

    How SCOP/CATH is used:

    Use type: do not use SCOP or CATH data

    Description: Cite SCOP/CATH as examples of protein structure databases.

    SCOP reference:

    Introduction

    Correct folding of a protein into its native three-dimensional (3D) structure is critical for normal protein functions. The mo- lecular mechanism responsible for protein folding is not fully understood and remains one of the most fundamental prob- lems in biological sciences. Nowadays, more than 1,000 different structural domains have been identified and deposited in protein structural databases, for example, SCOP (Structural Classification of Proteins) (Murzin et al. 1995;

    Andreeva et al. 2008), DDBASE (DIAL Derived Domain Database) (Vinayagam et al. 2003), PDP (Protein Domain Parser) (Alexandrov and Shindyalov 2003), CATH (Class, Archi- tecture, Topology, and Homologous superfamily) (Orengo et al. 1997; Cuff et al. 2011), or FSSP (Families of Structurally Similar Proteins) (Holm and Sander 1994). S

  ### Attachments

  - Genome Biol Evol-2012-Gelly-966-75.pdf
- ## Self-complementarity within proteins: bridging the gap between binding and folding

  |  |  |
  | --- | --- |
  | Type | Journal Article |
  | Author | Sankar Basu |
  | Author | Dhananjay Bhattacharyya |
  | Author | Rahul Banerjee |
  | Volume | 102 |
  | Issue | 11 |
  | Pages | 2605-2614 |
  | Publication | Biophysical journal |
  | ISSN | 1542-0086 |
  | Date | Jun 6, 2012 |
  | Extra | PMID: 22713576 |
  | Journal Abbr | Biophys. J. |
  | DOI | 10.1016/j.bpj.2012.04.029 |
  | Library Catalog | NCBI PubMed |
  | Language | eng |
  | Abstract | Complementarity, in terms of both shape and electrostatic potential, has been quantitatively estimated at protein-protein interfaces and used extensively to predict the specific geometry of association between interacting proteins. In this work, we attempted to place both binding and folding on a common conceptual platform based on complementarity. To that end, we estimated (for the first time to our knowledge) electrostatic complementarity (Em) for residues buried within proteins. Em measures the correlation of surface electrostatic potential at protein interiors. The results show fairly uniform and significant values for all amino acids. Interestingly, hydrophobic side chains also attain appreciable complementarity primarily due to the trajectory of the main chain. Previous work from our laboratory characterized the surface (or shape) complementarity (Sm) of interior residues, and both of these measures have now been combined to derive two scoring functions to identify the native fold amid a set of decoys. These scoring functions are somewhat similar to functions that discriminate among multiple solutions in a protein-protein docking exercise. The performances of both of these functions on state-of-the-art databases were comparable if not better than most currently available scoring functions. Thus, analogously to interfacial residues of protein chains associated (docked) with specific geometry, amino acids found in the native interior have to satisfy fairly stringent constraints in terms of both Sm and Em. The functions were also found to be useful for correctly identifying the same fold for two sequences with low sequence identity. Finally, inspired by the Ramachandran plot, we developed a plot of Sm versus Em (referred to as the complementarity plot) that identifies residues with suboptimal packing and electrostatics which appear to be correlated to coordinate errors. |
  | Short Title | Self-complementarity within proteins |
  | Date Added | 10/11/2013, 10:29:15 AM |
  | Modified | 11/18/2013, 10:12:24 AM |

  ### Tags:

  - Amino Acids
  - Crystallography, X-Ray
  - Databases, Protein
  - Models, Molecular
  - Protein Binding
  - Protein Folding
  - Proteins
  - Reproducibility of Results
  - Static Electricity

  ### Notes:

  - Present an electrostatic potential for buried residues.

    Shape and electrostatic potential complementarity, used for studying binding interfaces, may have practical use in folding and packing.  Apply shape and electrostatic potential complementarity analysis to fold recognition and to detect local regions of suboptimal packing and/or electrostatics in a native fold with a Ramachandran-style plot.

    How SCOP is used:

    Used data set, derived from the REFAB4.0 database, of low-sequence identity pairs of proteins in the same SCOP fold, distributed over a number of classes, for evaluating scoring functions on fold recognition.  Cross-threaded the sequences and found that scores were higher (better) than when threaded on decoys.

    SCOP reference:

    DB2 (composed of 65 all a, 70 all b, 106 ajb, 124 aþb, and 35 multidomain proteins) was used in the calculation of Em of amino acid residues and their related statistics.

    Fold recognition by cross-threading

    The scoring functions were also tested for protein pairs that

    belonged to the same fold but had low sequence identity

    upon alignment. We selected 100 such pairs (sequence iden-

    tities ranging from 6% to 30%) sampling diverse folds from

    the PREFAB4.0 database (40). The sequence identities upon

    structural alignment for each pair were determined by Dali

    Server (38) and their folds assigned according to the SCOP

    database (41) (data set S2). For every pair, we aligned the

    two native sequences using CLUSTAL W (42).

  ### Attachments

  - 1-s2.0-S0006349512005036-main.pdf
  - PubMed Central Link
  - PubMed entry
- ## Self consistency grouping: a stringent clustering method

  |  |  |
  | --- | --- |
  | Type | Journal Article |
  | Author | Bong-Hyun Kim |
  | Author | Bhadrachalam Chitturi |
  | Author | Nick V. Grishin |
  | URL | http://www.biomedcentral.com/1471-2105/13/S13/S3 |
  | Volume | 13 |
  | Issue | Suppl 13 |
  | Pages | S3 |
  | Publication | BMC bioinformatics |
  | Date | 2012 |
  | Accessed | 9/20/2013, 1:19:56 PM |
  | Library Catalog | Google Scholar |
  | Short Title | Self consistency grouping |
  | Date Added | 10/11/2013, 10:29:15 AM |
  | Modified | 10/11/2013, 10:29:15 AM |

  ### Notes:

  - Present a clustering method and evaluate it on SCOP domain structures and fold level classification.

    How SCOP is used:

    Benchmarking method.  Created a representative set of 9528 domains from SCOP 1.75 filtered at 40% sequence identity, then used DALI to get z-scores between all pairs.  Applied their own clustering algorithm to the data.

    SCOP references:

    Results

    We compare SCG to well-known agglomerative cluster- ing algorithms: complete linkage (CL), single linkage (SL), and average linkage (AL). We compared the meth- ods with simulated data and with SCOP datasets. Among these methods, CL is the most similar method to SCG.

    ....

    Comparison of methods in clustering protein structure

    In general, protein structures were considered hard to cluster with conventional clustering methods without human intervention [4]. We classified protein structures with SCG-fast, CL, AL, and SL (similar to the comparison of the previous section). We selected 9528 representative protein domain structures at 40% sequence identity hav- ing all alpha, all beta, alpha/beta, and alpha+beta from Structural Classifications Of Proteins (SCOP) ver. 1.75 [11]. Then Z-scores were measured for all pairs (~50,000,000) of the structures among the selected SCOP domain structures with DALI [12], one of most widely used structural comparison program. The similarity scores measured by DALI can be found at http://prodata. swmed.edu/scg/dali/. SCG identified 4965 independent clusters. In contrast to the test based on simulated data of the previous section, we set the parameter that

    determines the number of clusters for all other methods to this value, 4965 (table 2), in order to compare different clustering methods without a bias.

    The SCG clustering of SCOP domains shows that many of clusters are very small, ~1/3 of total protein domains form singleton clusters (3039 domains) and only few domains form relatively bigger clusters (see Fig 4). CL, AL and SL showed similar distributions, although CL shows the most similar cluster size distribution to SCG. SL shows the largest number of singletons. Among the three methods, CL is the most similar to SCG as shown in table 3. Compared to SCG other methods produce more singletons and lower numbers of clusters with sizes ranging from two to four. Clusters of protein structures constructed by these different methods were compared to SCOP folds built by experts and the number of correct and incorrect pairs were calculated same way as in Fig 3. (c). Similar to the simulation results (Fig 3. (c)), SL and AL show a larger percentage of incorrect pairs (3.7% and 3.5% respectively, see Table 2), whereas SCG and CL show only 0.2% and 0.4% incorrect pairs respectively. This reflects the fact that both SCG and CL are more conservative than others are. 

    We would expect similar results to the clustering done on simulated data if the scores were perfect and the grouping was done objectively. Note that we used Eucli- dian distance in simulation (Fig 3. (a)), which is consid- ered as a perfect measure, and SCG yielded no incorrect pairs. However, the DALI Z-score is a structural similar- ity score as measured by an approximate algorithm and the SCOP database is manually curated. Thus, one may reasonably expect that these two phenomena are not in perfect synchronization. We attribute the incorrect pairs found by SCG to the differences between the metric used for classification done by SCOP, i.e. human curation and the DALI Z-scores.

  ### Attachments

  - 1471-2105-13-S13-S3.pdf
  - [HTML] from biomedcentral.com
  - PubMed entry
- ## Sequence determinants of protein architecture

  |  |  |
  | --- | --- |
  | Type | Journal Article |
  | Author | S. Rackovsky |
  | Volume | 81 |
  | Issue | 10 |
  | Pages | 1681-1685 |
  | Publication | Proteins-Structure Function and Bioinformatics |
  | ISSN | 0887-3585 |
  | Date | OCT 2013 |
  | Extra | WOS:000324115400001 |
  | DOI | 10.1002/prot.24328 |
  | Abstract | Delineation of the relationship between sequence and structure in proteins has proven elusive. Most studies of this problem use alignment methods and other approaches based on the characteristics of individual residues. It is demonstrated herein that the sequence-structure relationship is determined in significant part by global characteristics of sequence organization. Information encoded in complete sequences is required to distinguish proteins in different architectural groups. It is found that the statistically significant differences between sequences encoding different architectures are encoded in a surprisingly small set of low-wave-number sequence periodicities. It would therefore appear that unexpected simplicity in an appropriately defined Fourier space may be an inherent characteristic of the sequences of folded proteins. Proteins 2013; 81:1681-1685. (c) 2013 Wiley Periodicals, Inc. |
  | Date Added | 2/13/2014, 4:13:17 PM |
  | Modified | 3/7/2014, 12:09:03 PM |
- ## Sequence, structure and functional diversity of PD-(D/E) XK phosphodiesterase superfamily

  |  |  |
  | --- | --- |
  | Type | Journal Article |
  | Author | Kamil Steczkiewicz |
  | Author | Anna Muszewska |
  | Author | Lukasz Knizewski |
  | Author | Leszek Rychlewski |
  | Author | Krzysztof Ginalski |
  | URL | http://nar.oxfordjournals.org/content/40/15/7016.short |
  | Volume | 40 |
  | Issue | 15 |
  | Pages | 7016–7045 |
  | Publication | Nucleic Acids Research |
  | Date | 2012 |
  | Accessed | 9/20/2013, 1:12:35 PM |
  | Library Catalog | Google Scholar |
  | Date Added | 10/11/2013, 10:20:13 AM |
  | Modified | 11/12/2013, 4:28:45 PM |

  ### Notes:

  - Investigation of the PD-(D/E)XK phosphodiesterases superfamily.

    Proteins under the superfamily PD-(D/E)XK phosphodiesterases are extremely diverse and homology is very complicated.  In SCOP, there are over 100 PD-(D/E)XK nucleases, found in four main "groups": restriction endonuclease-like enzymes, tRNA–intron splicing endo- nucleases, eukaryotic RPB5 N-terminal domain and TBP-interacting protein-like.

    Paper reclassifying proteins containing a PD-(D/E)XK domain

    -Multiple sequence alignment, structural alignment, taxonomy, function, used

    -Fold and class as classified by SCOP is noted when discussing the structure of PD-(D/E)XK phosphodiesterases

    -Also notes the fold level: Fold c.52: Restriction endonuclease-like, as well as the four superfamilies below it:

    1. |  |  |
       | --- | --- |
       |  | c.52.1: Restriction endonuclease-like [52980] (34 families) |
    2. |  |  |
       | --- | --- |
       |  | c.52.2: tRNA-intron endonuclease catalytic domain-like [53032] (1 family) |
    3. |  |  |
       | --- | --- |
       |  | c.52.3: Eukaryotic RPB5 N-terminal domain [53036] (1 family) |
    4. |  |  |
       | --- | --- |
       |  | c.52.4: TBP-interacting protein-like [159612] (1 family) |

    How SCOP is used:

    Built a data set from 44 Pfam famililies and 60 proteins from the "restriction endonuclease-like" fold in SCOP.

    **Quotes**

    **Under INTRODUCTION**

    "The common conserved structural core of PD-(D/E)XK  
    phosphodiesterases consists of a central, four-stranded,  
    mixed b-sheet flanked by two a-helices on both sides  
    (with abbbab topology), forming a scaffold adopted for the active site formation (11) (Figures 1 and 2). This architecture  
    and topology are classified in SCOP (Structural  
    Classification of Proteins) database (12) as a restriction  
    endonuclease-like fold."

    " In addition, there are over 100 structures of  
    PD-(D/E)XK nucleases cataloged in SCOP database (12)  
    clustered into four main groups, encompassing restriction  
    endonuclease-like enzymes, tRNA–intron splicing endonucleases,  
    eukaryotic RPB5 N-terminal domain and  
    TBP-interacting protein-like."

    **Under RESULTS**"In order to broaden the repertoire of PD-(D/E)XK  
    proteins we performed sensitive distant homology  
    searches using as the initial dataset 44 Pfam 25 families  
    and 60 representative restriction endonuclease-like  
    proteins of known structure cataloged in SCOP database."

    **Under DISCUSSION**

    'The PD-(D/E)XK fold can be described as gregarious  
    (161) referring to its presence in several evolutionary unrelated  
    protein structures. N-acetyltransferases, lipases,  
    dehydrogenases containing the PD-(D/E)XK domain as  
    a substructure represent different folds (even fold  
    classes) according to SCOP database. This finding  
    provides novel challenges to protein structure classification  
    that should probably describe structural space for  
    the a/b sandwich architecture as the continuum rather  
    than distinct folds."

    **CITATION**

    **12. Murzin,A.G., Brenner,S.E., Hubbard,T. and Chothia,C. (1995)  
    SCOP: a structural classification of proteins database for the  
    investigation of sequences and structures. J. Mol. Biol., 247,  
    536–540.**

  ### Attachments

  - Nucl. Acids Res.-2012-Steczkiewicz-7016-45.pdf
  - Snapshot
- ## SGD: Saccharomyces Genome Database

  |  |  |
  | --- | --- |
  | Type | Journal Article |
  | Author | J M Cherry |
  | Author | C Adler |
  | Author | C Ball |
  | Author | S A Chervitz |
  | Author | S S Dwight |
  | Author | E T Hester |
  | Author | Y Jia |
  | Author | G Juvik |
  | Author | T Roe |
  | Author | M Schroeder |
  | Author | S Weng |
  | Author | D Botstein |
  | Volume | 26 |
  | Issue | 1 |
  | Pages | 73-79 |
  | Publication | Nucleic acids research |
  | ISSN | 0305-1048 |
  | Date | Jan 1, 1998 |
  | Extra | PMID: 9399804 |
  | Journal Abbr | Nucleic Acids Res. |
  | Library Catalog | NCBI PubMed |
  | Language | eng |
  | Abstract | The Saccharomyces Genome Database (SGD) provides Internet access to the complete Saccharomyces cerevisiae genomic sequence, its genes and their products, the phenotypes of its mutants, and the literature supporting these data. The amount of information and the number of features provided by SGD have increased greatly following the release of the S.cerevisiae genomic sequence, which is currently the only complete sequence of a eukaryotic genome. SGD aids researchers by providing not only basic information, but also tools such as sequence similarity searching that lead to detailed information about features of the genome and relationships between genes. SGD presents information using a variety of user-friendly, dynamically created graphical displays illustrating physical, genetic and sequence feature maps. SGD can be accessed via the World Wide Web at http://genome-www.stanford.edu/Saccharomyces/ |
  | Short Title | SGD |
  | Date Added | 10/11/2013, 10:29:15 AM |
  | Modified | 10/11/2013, 10:29:15 AM |

  ### Tags:

  - Base Sequence
  - Databases, Bibliographic
  - Databases, Factual
  - Genes, Fungal
  - Genome, Fungal
  - Information Storage and Retrieval
  - Saccharomyces cerevisiae
  - Sequence Homology, Nucleic Acid
  - Terminology as Topic

  ### Notes:

  - SGD is a special database for Saccharomyces cerevisiae genomic data.

    How SCOP is used:

    Provide link to SCOP database.

    SCOP reference:

    For each homolog, hyperlinks to interactive 3D viewers [RasMol (40), Java viewer and Cn3D (41,42)] as well as external structural databases [PDB, MMDB (43), SCOP (44,45), CATH (46), Swiss-Model (47,48), PDBsum (49)] are offered for learning more about any particular protein structure listed.

  ### Attachments

  - Nucl. Acids Res.-1998-Cherry-73-9.pdf
- ## SHEEP: A TOOL FOR DESCRIPTION OF beta-SHEETS IN PROTEIN 3D STRUCTURES

  |  |  |
  | --- | --- |
  | Type | Journal Article |
  | Author | Evgeniy Aksianov |
  | Author | Andrei Alexeevski |
  | Volume | 10 |
  | Issue | 2, SI |
  | Publication | Journal of bioinformatics and computational biology |
  | ISSN | 0219-7200 |
  | Date | April 2012 |
  | DOI | 10.1142/S021972001241003X |
  | Language | English |
  | Abstract | The description of a protein fold is a hard problem due to significant variability of main structural units, beta-sheets and alpha-helixes, and their mutual arrangements. An adequate description of the structural units is an important step in objective protein structure classification, which to date is based on expert judgment in a number of cases. Explicit determination and description of structural units is more complicated for beta-sheets than for alpha-helixes due to beta-sheets variability both in composition and geometry. We have developed an algorithm that can significantly modify beta-sheets detected by commonly used DSSP and Stride algorithms and represent the result as a “beta-sheet map,” a table describing certain beta-sheet features. In our approach, beta-sheets (rather than beta-strands) are considered as holistic objects. Both hydrogen bonds and geometrical restrains are explored for the determination of beta-sheets. The algorithm is implemented in SheeP program. It was tested for prediction architectures of domains from 93 well-defined all-beta and alpha/beta SCOP protein domain families, and showed 93% of correct results. The Web-service http://mouse.belozersky.msu.ru/sheep allows to detect beta-sheets in a given protein structure, visualize beta-sheet maps, as well as input three-dimensional structures with highlighted beta-sheets and their structural features. |
  | Date Added | 10/11/2013, 10:29:15 AM |
  | Modified | 3/7/2014, 12:15:34 PM |

  ### Tags:

  - Interesting

  ### Notes:

  - Present SHEEP method, for creating a profile of beta-sheets in 3D structures that has applications in protein structure indexing and classification.

    How SCOP is used:

    Used SCOP annotations to help select a data set of SCOP families in which B-sheets are "explicitly described".

    Used data set for training and validation on detection of SCOP fold, superfamily, and family levels.

    Why is CATH cited:

    Additionally provide CATH arcitecture classification for protein of interest.

    SCOP reference:

    The algorithm is implemented in SheeP program. It was tested for prediction architectures of domains from 93 well-defined all-beta and alpha/betaSCOP protein domain families, and showed 93% of correct results.

    ...

    SheeP program is a part of the automatic protein architecture detector, results of which will be published elsewhere. The correct determination of -sheets in input protein structure by SheeP is more important for this purpose than the percentage of correct individual residue assignments in comparison with other protein sec- ondary structure detectors. We would like to avoid such mistakes as joining two -sheets of -sandwiches into one, splitting evident for the expert -barrel into two -sheets, etc. This is why, besides testing on the set of selected structures, we have applied SheeP to protein domain structures from several SCOP families. We had chosen those families for which the number of -sheets and their arrangement were clearly described in SCOP family or fold annotations. Nevertheless, we were forced to analyze manually all tested structures to confirm or correct family annotations for particular family members.

    SheeP results for 93 SCOP families showed 7% of essential mistakes in com- parison with human judgment.

    SCOP/CATH reference:

    The problem of choosing decision on -sheet compound could be demonstrated in a domain from PDB entry 1JMX, residues 364"494 of chain A. This domain is classified as immunoglobulin-like -sandwich (seven strands, two sheets) in SCOP database and as -sandwich, immunoglobulin-like architecture, in CATH.

  ### Attachments

  - S021972001241003X
  - s021972001241003x.pdf
- ## Shrimp invertebrate lysozyme i-lyz: Gene structure, molecular model and response of c and i lysozymes to lipopolysaccharide (LPS)

  |  |  |
  | --- | --- |
  | Type | Journal Article |
  | Author | Alma B. Peregrino-Uriarte |
  | Author | Adriana T. Muhlia-Almazan |
  | Author | Aldo A. Arvizu-Flores |
  | Author | Gracia Gomez-Anduro |
  | Author | Teresa Gollas-Galvan |
  | Author | Gloria Yepiz-Plascencia |
  | Author | Rogerio R. Sotelo-Mundo |
  | Volume | 32 |
  | Issue | 1 |
  | Pages | 230-236 |
  | Publication | Fish & Shellfish Immunology |
  | ISSN | 1050-4648 |
  | Date | JAN 2012 |
  | Extra | WOS:000299979100028 |
  | DOI | 10.1016/j.fsi.2011.10.026 |
  | Abstract | The invertebrate lysozyme (i-lyz or destabilase) is present in shrimp. This protein may have a function as a peptidoglycan-breaking enzyme and as a peptidase. Shrimp is commonly infected with Vibrio sp., a Gram-negative bacteria, and it is known that the c-lyz (similar to chicken lysozyme) is active against these bacteria. To further understand the regulation of lysozymes, we determined the gene sequence and modeled the protein structure of i-lyz. In addition, the expression of i-lyz and c-lyz in response to lipopolysaccharide (LPS) was studied. The shrimp i-lyz gene is interrupted by two introns with canonical splice junctions. The expression of the shrimp i-lyz was transiently down-regulated after LPS injection followed by induction after 6 h in hepatopancreas. In contrast, c-lyz was up-regulated in hepatopancreas 4 h post-injection and slightly down-regulated in gills. The L vannamei i-lyz does not contain the catalytic residues for muramidase (glycohydrolase) neither isopeptidase activities; however, it is known that the antibacterial activity does not solely rely on the enzymatic activity of the protein. The study of invertebrate lysozyme will increase our understanding of the regulatory process of the defense mechanisms. (C) 2011 Elsevier Ltd. All rights reserved. |
  | Date Added | 2/13/2014, 4:13:17 PM |
  | Modified | 3/7/2014, 12:14:06 PM |
- ## Shuttling happens: soluble flavin mediators of extracellular electron transfer in Shewanella

  |  |  |
  | --- | --- |
  | Type | Journal Article |
  | Author | Evan D. Brutinel |
  | Author | Jeffrey A. Gralnick |
  | URL | http://link.springer.com/article/10.1007/s00253-011-3653-0 |
  | Volume | 93 |
  | Issue | 1 |
  | Pages | 41-48 |
  | Publication | Applied Microbiology and Biotechnology |
  | ISSN | 0175-7598, 1432-0614 |
  | Date | 2012/01/01 |
  | Journal Abbr | Appl Microbiol Biotechnol |
  | DOI | 10.1007/s00253-011-3653-0 |
  | Accessed | 12/9/2014, 6:51:19 AM |
  | Library Catalog | link.springer.com |
  | Language | en |
  | Abstract | The genus Shewanella contains Gram negative γ-proteobacteria capable of reducing a wide range of substrates, including insoluble metals and carbon electrodes. The utilization of insoluble respiratory substrates by bacteria requires a strategy that is quite different from a traditional respiratory strategy because the cell cannot take up the substrate. Electrons generated by cellular metabolism instead must be transported outside the cell, and perhaps beyond, in order to reduce an insoluble substrate. The primary focus of research in model organisms such as Shewanella has been the mechanisms underlying respiration of insoluble substrates. Electrons travel from the menaquinone pool in the cytoplasmic membrane to the surface of the bacterial cell through a series of proteins collectively described as the Mtr pathway. This review will focus on respiratory electron transfer from the surface of the bacterial cell to extracellular substrates. Shewanella sp. secrete redox-active flavin compounds able to transfer electrons between the cell surface and substrate in a cyclic fashion—a process termed electron shuttling. The production and secretion of flavins as well as the mechanisms of cell-mediated reduction will be discussed with emphasis on the experimental evidence for a shuttle-based mechanism. The ability to reduce extracellular substrates has sparked interest in using Shewanella sp. for applications in bioremediation, bioenergy, and synthetic biology. |
  | Short Title | Shuttling happens |
  | Date Added | 12/9/2014, 6:51:19 AM |
  | Modified | 12/9/2014, 6:51:19 AM |

  ### Tags:

  - Biotechnology
  - Electron shuttle
  - Flavin
  - Microbial Genetics and Genomics
  - Microbiology
  - Respiration
  - Shewanella

  ### Notes:

  - Review of research on soluble flavin mediators of extracellular electron transfer in Shewanella

    How SCOP is used:

    Investigate classification of one protein or a small set of proteins

    SCOP reference:

    Hemes 2 and 7 are located at the sides of the protein near a β-barrel of extended Greek-key split barrel domains (Clarke et al. 2011), common in flavin binding proteins (Hubbard et al. 1999).

  ### Attachments

  - Full Text PDF
- ## SIFTS: Structure Integration with Function, Taxonomy and Sequences resource

  |  |  |
  | --- | --- |
  | Type | Journal Article |
  | Author | Sameer Velankar |
  | Author | Jose M. Dana |
  | Author | Julius Jacobsen |
  | Author | Glen van Ginkel |
  | Author | Paul J. Gane |
  | Author | Jie Luo |
  | Author | Thomas J. Oldfield |
  | Author | Claire O'Donovan |
  | Author | Maria-Jesus Martin |
  | Author | Gerard J. Kleywegt |
  | Volume | 41 |
  | Issue | D1 |
  | Pages | D483-D489 |
  | Publication | Nucleic Acids Research |
  | ISSN | 0305-1048 |
  | Date | JAN 2013 |
  | Extra | WOS:000312893300069 |
  | DOI | 10.1093/nar/gks1258 |
  | Abstract | The Structure Integration with Function, Taxonomy and Sequences resource (SIFTS; http://pdbe.org/sifts) is a close collaboration between the Protein Data Bank in Europe (PDBe) and UniProt. The two teams have developed a semi-automated process for maintaining up-to-date cross-reference information to UniProt entries, for all protein chains in the PDB entries present in the UniProt database. This process is carried out for every weekly PDB release and the information is stored in the SIFTS database. The SIFTS process includes cross-references to other biological resources such as Pfam, SCOP, CATH, GO, InterPro and the NCBI taxonomy database. The information is exported in XML format, one file for each PDB entry, and is made available by FTP. Many bioinformatics resources use SIFTS data to obtain cross-references between the PDB and other biological databases so as to provide their users with up-to-date information. |
  | Date Added | 2/20/2014, 12:24:01 PM |
  | Modified | 3/7/2014, 12:08:40 PM |

  ### Notes:

  - Database paper,  presents update of SIFTS, a weekly-updated database that maps multiple databases.  SIFTS has been used by other databases o obtain cross-references for PDB files between many different databases.

    How SCOP is used:

    Annotate chains in the database with SCOP domains and classification, amongst other annotations.

    SCOP reference:

    Under Abstract:

    The SIFTS process includes cross- references to other biological resources such as Pfam, SCOP, CATH, GO, InterPro and the NCBI taxonomy database.

  ### Attachments

  - Nucl. Acids Res.-2013-Velankar-D483-9.pdf
- ## Simultaneous prediction of protein secondary structure and transmembrane spans

  |  |  |
  | --- | --- |
  | Type | Journal Article |
  | Author | Julia Koehler Leman |
  | Author | Ralf Mueller |
  | Author | Mert Karakas |
  | Author | Nils Woetzel |
  | Author | Jens Meiler |
  | Volume | 81 |
  | Issue | 7 |
  | Pages | 1127–1140 |
  | Publication | Proteins-structure Function and Bioinformatics |
  | Date | July 2013 |
  | DOI | 10.1002/prot.24258 |
  | Abstract | Prediction of transmembrane spans and secondary structure from the protein sequence is generally the first step in the structural characterization of (membrane) proteins. Preference of a stretch of amino acids in a protein to form secondary structure and being placed in the membrane are correlated. Nevertheless, current methods predict either secondary structure or individual transmembrane states. We introduce a method that simultaneously predicts the secondary structure and transmembrane spans from the protein sequence. This approach not only eliminates the necessity to create a consensus prediction from possibly contradicting outputs of several predictors but bears the potential to predict conformational switches, i.e., sequence regions that have a high probability to change for example from a coil conformation in solution to an -helical transmembrane state. An artificial neural network was trained on databases of 177 membrane proteins and 6048 soluble proteins. The output is a 3 x 3 dimensional probability matrix for each residue in the sequence that combines three secondary structure types (helix, strand, coil) and three environment types (membrane core, interface, solution). The prediction accuracies are 70.3% for nine possible states, 73.2% for three-state secondary structure prediction, and 94.8% for three-state transmembrane span prediction. These accuracies are comparable to state-of-the-art predictors of secondary structure (e.g., Psipred) or transmembrane placement (e.g., OCTOPUS). The method is available as web server and for download at www.meilerlab.org. Proteins 2013; 81:1127-1140. (c) 2013 Wiley Periodicals, Inc. |
  | Date Added | 3/7/2014, 1:06:24 PM |
  | Modified | 3/7/2014, 1:06:24 PM |
- ## Single molecule force spectroscopy using polyproteins

  |  |  |
  | --- | --- |
  | Type | Journal Article |
  | Author | Toni Hoffmann |
  | Author | Lorna Dougan |
  | URL | http://pubs.rsc.org/en/content/articlehtml/2012/cs/c2cs35033e |
  | Volume | 41 |
  | Issue | 14 |
  | Pages | 4781–4796 |
  | Publication | Chemical Society Reviews |
  | Date | 2012 |
  | Accessed | 9/20/2013, 1:13:08 PM |
  | Library Catalog | Google Scholar |
  | Date Added | 2/20/2014, 12:24:01 PM |
  | Modified | 2/20/2014, 12:24:01 PM |

  ### Notes:

  - Present tutorial on an experimental method: single molecule force spectroscopy.

    How SCOP is used:

    Annotate a data set of polyproteins with SCOP class.

    SCOP reference:

    Table 1 Selected homopolyproteins and chimeric polyproteins. This table contains a small selection of mainly recombinant homopolyproteins and proteins that have been mechanically analysed using chimeric polyproteins. Lower arabic numbers indicate the number of respective protein domains or tandem repeats in the polyprotein. If available, the pulling velocity is given in round brackets next to the peak unfolding force. The general classification is taken from the SCOP database.60

  ### Attachments

  - [PDF] from researchgate.net
- ## SitEx: a computer system for analysis of projections of protein functional sites on eukaryotic genes

  |  |  |
  | --- | --- |
  | Type | Journal Article |
  | Author | Irina Medvedeva |
  | Author | Pavel Demenkov |
  | Author | Nikolay Kolchanov |
  | Author | Vladimir Ivanisenko |
  | Volume | 40 |
  | Issue | Database issue |
  | Pages | D278-283 |
  | Publication | Nucleic Acids Research |
  | ISSN | 1362-4962 |
  | Date | Jan 2012 |
  | Extra | PMID: 22139920 |
  | Journal Abbr | Nucleic Acids Res. |
  | DOI | 10.1093/nar/gkr1187 |
  | Library Catalog | NCBI PubMed |
  | Language | eng |
  | Abstract | Search of interrelationships between the structural-functional protein organization and exon structure of encoding gene provides insights into issues concerned with the function, origin and evolution of genes and proteins. The functions of proteins and their domains are defined mostly by functional sites. The relation of the exon-intron structure of the gene to the protein functional sites has been little studied. Development of resources containing data on projections of protein functional sites on eukaryotic genes is needed. We have developed SitEx, a database that contains information on functional site amino acid positions in the exon structure of encoding gene. SitEx is integrated with the BLAST and 3DExonScan programs. BLAST is used for searching sequence similarity between the query protein and polypeptides encoded by single exons stored in SitEx. The 3DExonScan program is used for searching for structural similarity of the given protein with these polypeptides using superimpositions. The developed computer system allows users to analyze the coding features of functional sites by taking into account the exon structure of the gene, to detect the exons involved in shuffling in protein evolution, also to design protein-engineering experiments. SitEx is accessible at http://www-bionet.sscc.ru/sitex/. Currently, it contains information about 9994 functional sites presented in 2021 proteins described in proteomes of 17 organisms. |
  | Short Title | SitEx |
  | Date Added | 10/11/2013, 10:20:13 AM |
  | Modified | 11/12/2013, 4:28:56 PM |

  ### Notes:

  - Database that has information on "functional site amino acid positions in the exon structure of  
    encoding gene."

    Their data set is composed of 2021 structures with non-redundant sequences whose PDB descriptions contained functional sites.

    How SCOP is used:

    -Annotated data set with SCOP domain boundaries and fold

    -Can search through the SitEx database using SCOP identifiers

    SCOP reference: **Under CONSTRUCTION AND CONTENT**

    "Also, using ClustalW alignments, amino acid positions  
    of functional site, exon, Pfam domains and SCOP structural region boundaries were identified in polypeptide chain. Information on folds was retrieved from the SCOP database (22)."

    **Under UTILITY**

    "The web service SitEx consists of a database covering the protein functional sites, Pfam and SCOP domain projections..."

    "Search queries are performed through the PDB, SCOP  
    and Ensembl identifiers....."

    **Citation**

    22. Murzin,A.G., Brenner,S.E., Hubbard,T. and Chothia,C. (1995)  
    SCOP: a structural classification of proteins database for the  
    investigation of sequences and structures. J. Mol. Biol., 247,  
    536–540.

  ### Attachments

  - [HTML] from oxfordjournals.org
  - Nucl. Acids Res.-2012-Medvedeva-D278-83.pdf
  - PubMed entry
- ## Size scaling behaviour in protein domains belonging to the all-alpha, all-beta, alpha/beta, and alpha plus beta folding classes

  |  |  |
  | --- | --- |
  | Type | Journal Article |
  | Author | Parker Rogerson |
  | Author | Gustavo A. Arteca |
  | Volume | 50 |
  | Issue | 1 |
  | Pages | 169-186 |
  | Publication | Journal of Mathematical Chemistry |
  | ISSN | 0259-9791 |
  | Date | January 2012 |
  | DOI | 10.1007/s10910-011-9904-6 |
  | Language | English |
  | Abstract | We studied the size scaling behaviour in an ensemble of 8,614 non-redundant protein domains belonging to the all-alpha, all-beta, alpha / beta, and alpha + beta folding classes. We find that the most compact structural domains can be characterized by an effective exponent nu (eff) = 0.39 +/- 0.01, which is larger than the value for “collapsed-polymers,” i.e., nu = 1/3. We also show that the global nu (eff) -exponent is an average of the scaling regimes for short and long compact chains, where the values change from nu (eff) a parts per thousand 0.37 to nu (eff) a parts per thousand 0.45 at chain length of ca. 269. A transition from short-chain to long-chain scaling behaviour is found in all major folding classes, over a window of chain lengths between 216 and 269 residues. In addition, variations in scaling exponent with respect to folding class indicates that the smallest domains in the (all-beta) and (alpha / beta) families appear to be more compact structures than the smallest (all-alpha)- and (alpha + beta)-domains. |
  | Date Added | 10/11/2013, 10:29:15 AM |
  | Modified | 3/7/2014, 12:14:04 PM |

  ### Tags:

  - likely ASTRAL
  - likely ASTRAL domain structures

  ### Notes:

  - Computational study of "size scaling behavior" in domains from all-alpha, all-beta, alpha/beta, and alpha+beta classes.  Size and density is characterized by the "radius of gyration" which is analagous to the standard deviation of distances between the center of the domain and the CAs.

    How SCOP is used:

    Collect a dataset of domain structures form each of the top 4 classes, then remove redundancy.  Calculate "radius of gyration" for each domain and then compare statistics on each of the classes.

    SCOP reference:

    2.2 Selection of non-redundant single domains

    We used the SCOP data base as the basis to organize our selected ensemble of pro- tein domains. This data base organizes domains into lineages of “common folds” within larger “folding classes” based on similarities in folding topology [14–17]. Entries in SCOP are manually curated; domains are inspected visually and classified according to consensual, albeit subjective criteria. In this work, we consider the four principal folding classes (or root nodes), corresponding to the (all-α), (all-β), (α + β), and (α / β)-folds. The all-α and all-β folds consist almost exclusively of helical and β-sheet structures, respectively, while the α+β and α/β classes contain varying degrees of both secondary-structural elements. In (α + β)-domains, helices and anti- parallel-sheets are spatially segregated; in the (α / β)-folds, helices and β-sheets typi- cally alternate, allowing the β-strands to organize in a parallel fashion, e.g., the TIM- barrels [26,27].

    Given the high level of redundancy in the PDB and SCOP data bases, it is important to avoid biasing the size-scaling analysis by eliminating all duplicate entries from our data set. We devised the following selection protocol to ensure one entry per domain type:

    1. (a)  Only one structure was used per domain among entries with no missing residues and at least a 3.2Å-resolution.
    2. (b)  Domains with more than 90% sequence homology were represented by a single entry, unless they differed in chain length by more than fifteen residues, in which case they are considered distinct entries.
    3. (c)  Very short chains were deemed poorly-structured peptides and often omitted; typically, but not always, protein chains with less than 35 residues appear as “outliers” in our analysis.

    The present study began with an ensemble of 85,686 single domains in the SCOP data base, with the following breakdown in terms of folding class: 14,824 for F C = α (i.e., (all-α)-domains), 23,547 for FC = β, 21,499 for FC = α+β, and 25,816 for FC = α/β. When subject to the above screening procedure, the set is reduced

    roughly to about 10% of the total entries. Specifically, we retain 8,614 non-redundant structures, with the following distribution according to folding class: 1,741 (all-α)- domains, 2,527 all-β, 2,099 α + β, and 2,247 (α / β)-domains. This list includes indi- vidual domains associated with both single- and multi-domain proteins. The radius of gyration of each chain was computed from the α-carbon coordinates extracted from entries in the PDB archive. In the next section, we use these results to analyze the size-scaling behaviour in isolated protein domains.

    ...

    4 Effect of folding class on size-scaling behaviour

    Using the SCOP classification, we have extended the previous analysis to determine the effect of folding class on the ν ̄FC-exponents introduced in Eq. (4). As in Sect. 3, we determine the domains with the smallest radius of gyration within a given bin of chain lengths. The set of molecular sizes for the most compact entries within a given folding class is denoted as {[rg]∗j,FC}, corresponding to the ensemble of jth-bins for FC-domains for a particular bin selection. Here, we use ⬚⬚n = 10 for all folding classes.

  ### Attachments

  - art%3A10.1007%2Fs10910-011-9904-6.pdf
- ## S-linked protein homocysteinylation: identifying targets based on structural, physicochemical and protein-protein interactions of homocysteinylated proteins

  |  |  |
  | --- | --- |
  | Type | Journal Article |
  | Author | Yumnam Silla |
  | Author | Elayanambi Sundaramoorthy |
  | Author | Puneet Talwar |
  | Author | Shantanu Sengupta |
  | Volume | 44 |
  | Issue | 5 |
  | Pages | 1307-1316 |
  | Publication | Amino acids |
  | ISSN | 0939-4451 |
  | Date | May 2013 |
  | DOI | 10.1007/s00726-013-1465-5 |
  | Language | English |
  | Abstract | An elevated level of homocysteine, a thiol-containing amino acid is associated with a wide spectrum of disease conditions. A majority (> 80 %) of the circulating homocysteine exist in protein-bound form. Homocysteine can bind to free cysteine residues in the protein or could cleave accessible cysteine disulfide bonds via thiol disulfide exchange reaction. Binding of homocysteine to proteins could potentially alter the structure and/or function of the protein. To date only 21 proteins have been experimentally shown to bind homocysteine. In this study we attempted to identify other proteins that could potentially bind to homocysteine based on the criteria that such proteins will have significant 3D structural homology with the proteins that have been experimentally validated and have solvent accessible cysteine residues either with high dihedral strain energy (for cysteine-cysteine disulfide bonds) or low pKa (for free cysteine residues). This analysis led us to the identification of 78 such proteins of which 68 proteins had 154 solvent accessible disulfide cysteine pairs with high dihedral strain energy and 10 proteins had free cysteine residues with low pKa that could potentially bind to homocysteine. Further, protein-protein interaction network was built to identify the interacting partners of these putative homocysteine binding proteins. We found that the 21 experimentally validated proteins had 174 interacting partners while the 78 proteins identified in our analysis had 445 first interacting partners. These proteins are mainly involved in biological activities such as complement and coagulation pathway, focal adhesion, ECM-receptor, ErbB signalling and cancer pathways, etc. paralleling the disease-specific attributes associated with hyperhomocysteinemia. |
  | Date Added | 10/11/2013, 10:29:15 AM |
  | Modified | 12/2/2013, 4:18:55 PM |

  ### Tags:

  - Dihedral strain energy
  - Disulfide
  - Homocysteine
  - Homologous structure
  - pKa
  - protein-protein interaction

  ### Notes:

  - Identify potential homocystein-binding targets.  Elevated levels of homocysteine, a thio-containing amino acid, is associated with a number of disease conditions.  Binding of homocystein to proteins could alter structure and/or function.  Only 21 proteins have been experimentally determined to bind to homocysteine.

     How SCOP is used:

    Search SCOP 1.75 database for proteins with structural similarity using iPBA (protein block profile based method).

    SCOP reference:

    Materials and methods

    Homology structure search

    To identify the proteins that have structural similarity with the 21 proteins that have been experimentally proven to bind Hcy, the PDB ids of these proteins were searched using iPBA (Gelly et al. 2011). The iPBA provides struc- turally related protein using SCOP version 1.75 (Murzin et al. 1995) as the structure data set. Based on the Protein Block (PB) alignment score the top 100 hits are reported and values [1.5 are considered to be structurally related with high confidence. Hence in our analysis we have considered all the human proteins that have score greater than 1.5. To quantify the PB sequence alignment score, iPBA further provides a GDT\_PB score which is similar to the Global distance test total score (GDT\_TS) (Zemla 2003) for the top hundred hits.

  ### Attachments

  - s00726-013-1465-5.pdf
- ## Small-angle X-ray scattering constraints and local geometry like secondary structures can construct a coarse-grained protein model at amino acid residue resolution

  |  |  |
  | --- | --- |
  | Type | Journal Article |
  | Author | Yasumasa Morimoto |
  | Author | Takashi Nakagawa |
  | Author | Masaki Kojima |
  | URL | http://www.sciencedirect.com/science/article/pii/S0006291X12024394 |
  | Publication | Biochemical and biophysical research communications |
  | Date | 2013 |
  | Accessed | 9/23/2013, 10:16:05 AM |
  | Library Catalog | Google Scholar |
  | Date Added | 10/11/2013, 10:29:15 AM |
  | Modified | 10/11/2013, 10:29:15 AM |

  ### Tags:

  - Coarse-grained model
  - Restrained molecular dynamics
  - secondary structure
  - Small-angle X-ray scattering

  ### Notes:

  - Present a new methodology to determine protein structure using small-angle X-ray scattering (SAXS) data.

    How SCOP is used:

    Use SCOP to guide selection of small data set of 8 proteins to ensure fold diversity. Data set is used to validate method.

    SCOP reference:

    2.2. SAXS and NMR data

    We used RNase T1 as the first model protein, because its NMR- derived three-dimensional structure was once determined in our laboratory [14] and the SAXS data measured under the same experimental condition were also available [6].

    In addition to RNase T1, eight proteins with different folding topologies based on the Structure Classification Of Proteins (SCOP) [15] were selected according to the following requirements:

    1. (i)  The atomic coordinates determined by NMR are available from Protein Data Bank (PDB).
    2. (ii)  The NMR-derived distance information obtained experimen- tally is also available from BioMagResBank (BMRB).
    3. (iii)  The same structure as in PDB can be constructed correctly from the NMR-derived structural information in BMRB using the EMBOSS program used in this study.

    Among eight proteins thus selected, ATC2521 from Agrobacte- rium tumefaciens (PDB ID: 2JQ4) and steril-a-motif of human de- leted in liver cancer 2 (2JW2) are classified as all-a proteins, Filamin-B (2DIA) and putative lipoprotein from Bacillus cereus (2K5W) as all-b proteins, pyruvate phosphate dikinase (2FM4 [16]) and eukaryotic translation termination factor eRF1 (2HST [17]) as a/b proteins, and Ral guanosine dissociation stimulator (2B3A) and NE1680 from Nitrosomonas europaea (2HFQ) as a+b proteins.

  ### Attachments

  - 1-s2.0-S0006291X12024394-main.pdf
- ## Smolign: A Spatial Motifs-Based Protein Multiple Structural Alignment Method

  |  |  |
  | --- | --- |
  | Type | Journal Article |
  | Author | Hong Sun |
  | Author | Ahmet Sacan |
  | Author | Hakan Ferhatosmanoglu |
  | Author | Yusu Wang |
  | URL | http://ieeexplore.ieee.org/xpls/abs\_all.jsp?arnumber=6078455 |
  | Volume | 9 |
  | Issue | 1 |
  | Pages | 249–261 |
  | Publication | Computational Biology and Bioinformatics, IEEE/ACM Transactions on |
  | Date | 2012 |
  | Accessed | 9/20/2013, 1:18:03 PM |
  | Library Catalog | Google Scholar |
  | Short Title | Smolign |
  | Date Added | 10/11/2013, 10:29:15 AM |
  | Modified | 3/7/2014, 12:11:01 PM |

  ### Tags:

  - contact map
  - distance map
  - HOMSTRAD
  - multiple structure alignment
  - partial order curve comparison
  - protein structure
  - secondary structure elements (SSE)
  - structural motif library

  ### Notes:

  - Paper detailing a new method for multiple structure alignment based on common alignments from a built library of motifs.

    "We introduce a novel strategy based on: building a contact-window based motif library from the protein structural data, discovery and extension of common alignment seeds from this library, and optimal superimposition of multiple structures according to these alignment seeds by an enhanced partial order curve comparison method."

    How SCOP is used:

    SCOP data not being used. They just note that the organization of the dataset being used is similar to SCOP's family level.

    How CATH is used:

    Look up classification of two proteins in a data set, for more background.

    SCOP reference:

    Homstrad [28] benchmark dataset contains manually  
    curated pairwise and multiple alignments of highly  
    homologous proteins. The similarity of the aligned  
    proteins is comparable to that of the family level  
    in the SCOP [44] hierarchical classification database.

    CATH reference:

    Set 2 has only 3 proteins (PDB: 1cnx, 1jfjA, and 2sas), but the aligned motifs are very diverse. CATH [40] classifies 1ncx and 2sas to have one alpha helical domain and 1jfjA to have two alpha helical domains.

  ### Attachments

  - [PDF] from metu.edu.tr
- ## S-MOTIFS AS A NEW APPROACH TO SECONDARY STRUCTURE PREDICTION: COMPARISON WITH STATE OF THE ART METHODS

  |  |  |
  | --- | --- |
  | Type | Journal Article |
  | Author | Ivan Popov |
  | URL | http://www.diagnosisp.com/dp/journals/view\_pdf.php?journal\_id=1&archive=1&issue\_id=39&article\_id=1330 |
  | Volume | 26 |
  | Issue | 3 |
  | Pages | 3016–3020 |
  | Publication | BIOTECHNOLOGY & BIOTECHNOLOGICAL EQUIPMENT |
  | Date | 2012 |
  | Accessed | 9/23/2013, 10:24:40 AM |
  | Library Catalog | Google Scholar |
  | Short Title | S-MOTIFS AS A NEW APPROACH TO SECONDARY STRUCTURE PREDICTION |
  | Date Added | 10/11/2013, 10:29:15 AM |
  | Modified | 10/11/2013, 10:29:15 AM |

  ### Tags:

  - proteins structure prediction
  - secondary structure
  - s-motifs

  ### Notes:

  - **Paper Summary**

    It is detailing a method for predicting secondary structure based on the protein sequence. It does so by matching S-motifs to the sequences.

    **SCOP Use** SCOP data not being used. Just mentioned as a database that also classifies motifs.

    **SCOP Reference**

    They were first used to  
    explore the diversity of the loop regions in available protein  
    structures (5), and later, to characterize the novel protein  
    folds that were added to the databases concerned with the  
    classification of protein structures (SCOP, CATH) (4, 11, 13)

  ### Attachments

  - [PDF] from diagnosisp.com
- ## SMURFLite: combining simplified Markov random fields with simulated evolution improves remote homology detection for beta-structural proteins into the twilight zone

  |  |  |
  | --- | --- |
  | Type | Journal Article |
  | Author | Noah M. Daniels |
  | Author | Raghavendra Hosur |
  | Author | Bonnie Berger |
  | Author | Lenore J. Cowen |
  | URL | http://bioinformatics.oxfordjournals.org/content/28/9/1216.short |
  | Volume | 28 |
  | Issue | 9 |
  | Pages | 1216–1222 |
  | Publication | Bioinformatics |
  | Date | 2012 |
  | Accessed | 9/20/2013, 1:16:59 PM |
  | Library Catalog | Google Scholar |
  | Short Title | SMURFLite |
  | Date Added | 10/11/2013, 10:29:15 AM |
  | Modified | 10/11/2013, 10:29:15 AM |

  ### Tags:

  - likely ASTRAL
  - likely ASTRAL domains
  - likely ASTRAL sequences

  ### Notes:

  - I emailed authors to ask how exactly they compiled their data set.
  - Present SMURFLite, a HMMER-type program for sequence alignment, that incorporates features on hydrogen bond patterns in beta strands.

    SMURF uses Markov Random Fields, rather than HMMs.  HMMs are limited in their power to detect remote homologs because they do not model statistical dependencies between amino acid residues that are close in space but far apart in sequence.  MRFs use an auxiliary dependency graph that allows them to model more complex statistical dependencies.

    How SCOP is used:

    Use SCOP for training and benchmarking.

    1. Beta-propeller data set:  Downloaded a selection of SCOP folds and superfamilies that are mostly-beta from SCOP version 1.75.  Then created the data set by downloading the sequences and structures of the full chains from the PDB.  (This was verified with the SMURF authors.)

    2. All-beta proteins: Filtered by superfamily, removing superfamilies that were deemed 'structurally inconsistent' using a structural alignment program.

    Used the all-beta data set to demonstrate scalability of their method, and conduct a whole-genome search on T. maritima.

    (Beta-propeller training and benchmarking data set: fold, superfamily), (All-beta proteins benchmarking data set: class, superfamily )

    Reference to SCOP:

    Abstract:

    We test SMURFLite on all propeller and barrel folds in the mainly-beta class of the SCOP hierarchy in stringent cross-validation experiments.

    Introduction:

    We first test SMURFLite on all propeller and barrel folds in the mainly-beta class of the SCOP hierarchy in stringent cross- validation experiments.

    We demonstrate SMURFLite’s ability to scale to whole genomes by running a SMURFLite library of 207 beta-structural SCOP superfamilies against the entire genome of T.maritima, and make over a 100 new fold predictions (available at http://smurf.cs.tufts.edu/smurflite). The majority of these predictions are for genes that display very little sequence similarity with any proteins of known structure, demonstrating the power of SMURFlite to recognize remote homologs.

    Methods: Datasets:

    From SCOP (Murzin et al., 1995) version 1.75, we chose the folds ‘5-bladed Beta-Propellers’, ‘6-bladed Beta-Propellers’, ‘7-bladed Beta- Propellers’ and ‘8-bladed Beta-Propellers’. We also chose superfamilies from all of the mostly-beta folds containing the word ‘barrel’ in their description, whether open or closed, restricted to those superfamilies comprising at least four families (in order to facilitate leave-family- out cross-validation). These superfamilies were: ‘Nucleic acid-binding proteins’ (50249), ‘Translation proteins’ (50447), ‘Trypsin-like serine proteases’ (50494), ‘Barwin-like endoglucanases’ (50685), ‘Cyclophilin- like’ (50891), ‘Sm-like ribonucleoproteins’ (50182), ‘PDZ domain-like’ (50156), ‘Prokaryotic SH3-related domain’ (82057), ‘Tudor/PWWP/MBT’ (63748), ‘Electron Transport accessory proteins’ (50090), ‘Translation proteins SH3-like domain’ (50104), ‘Lipocalins’ (50814) and ‘FMN-binding split barrel’ (50475). Of these, we removed the superfamilies ‘Lipocalins’ and ‘Trypsin-like serine proteases,’ which were not structurally consistent enough to permit a multiple structure alignment for training HMMER or the SMURF variants, and which were broken into distinct superfamilies by (Daniels et al., 2012), with the result that 11 superfamilies containing barrels were selected. In addition, for the whole-genome search on T.maritima, out of 354 total superfamilies within the SCOP class ‘All beta proteins’, 288 (81%) of which contain at least two protein chains, 207 superfamilies (71%) were structurally consistent enough to be aligned using the Matt (Menke et al., 2008) structural alignment program. We built SMURFLite templates for these 207 superfamilies, and obtained from UniProt the protein sequences for T.maritima, comprising 1852 genes.

    Methods: Training and testing process:

    For the beta-propeller folds, strict leave-superfamily-out cross-validation was performed. The propeller folds are structurally highly consistent (Menke et al., 2010), and thus high-quality Matt (Menke et al., 2008) multiple structure alignments were possible without descending to the superfamily level. For each propeller fold, its constituent superfamilies were identified. Each superfamily was left out, a training set was established from the protein chains in the remaining superfamilies, with duplicate sequences removed. An HMM (in the case of HMMER and HHPred) or MRF (in the case of SMURF and SMURFLite) were trained on the training set (HMMER parameter settings are discussed below). Protein chains from the left-out superfamily were used as positive test examples. Negative test examples were protein chains from all other folds in SCOP classes 1, 2, 3 and 4 (including propeller folds with differing blade counts), indicated as representatives from the non-redundant Protein Data Bank repository (nr-PDB) (Berman et al., 2000) database with non-redundancy set to a BLAST E-value of 10−7.

    The beta propellers are atypical of most beta-structural SCOP folds, in that they structurally align well at the fold level of the SCOP hierarchy. For the beta-barrel superfamilies, strict leave-family-out cross-validation was performed. The barrel superfamilies are distinguished by strand number and shear as well as other structural features (Murzin et al., 1995), and so like most beta-structural motifs they do not align well structurally at the fold level. For this reason, the superfamily level was chosen for training. For each superfamily, its constituent families were identified. Each family was left out, a training set was established from the protein chains in the remaining families, with duplicate sequences removed. An HMM (in the case of HMMER and HHPred) or MRF (in the case of SMURF and SMURFLite) were trained on the training set. Protein chains from the left-out family were used as positive test examples. Negative test examples were protein chains from all other superfamilies in SCOP classes 1, 2, 3 and 4 (including other barrel superfamilies), indicated as representatives from the nr-PDB (Berman et al., 2000) database with non-redundancy set to a BLAST E-value of 10−7.

    Each test example was aligned to the trained HMM (from HMMER and HHPred) and MRF, and was also threaded, using RAPTOR, against each individual chain in the training set (RAPTOR parameters are discussed below). The score reported for HMMER and HHPred was the output HMM score, and the score reported for SMURF and SMURFLite was the combined HMM and pairwise score from the MRF. For RAPTOR, the score reported for a test example was the highest score from all the scores resulting from threading that test example onto each chain in the training set. For each training set, the scores for each method were collected and a ROC curve (a plot of true positive rate versus false positive rate) computed. We report the area under the curve (AUC statistic) from this ROC curve (Sonego and Pongor, 2008).

    Methods: P-values:

    SMURFLite computes the P-value for an alignment similarly to HMMER, using an extreme value distribution (EVD) (Eddy, 1998). An EVD is fitted to the distribution of raw scores over a random sampling of 5000 protein chains from across the SCOP hierarchy. The P-value is then simply computed as 1−cdf(x) for any raw SmurfLite score x

    More references in Methods and Results

  ### Attachments

  - Full Text PDF
  - [HTML] from oxfordjournals.org
  - Snapshot
- ## Soliton driven relaxation dynamics and protein collapse in the villin headpiece

  |  |  |
  | --- | --- |
  | Type | Journal Article |
  | Author | Andrey Krokhotin |
  | Author | Martin Lundgren |
  | Author | Antti J. Niemi |
  | Author | Xubiao Peng |
  | URL | http://iopscience.iop.org/0953-8984/25/32/325103 |
  | Volume | 25 |
  | Issue | 32 |
  | Pages | 325103 |
  | Publication | Journal of Physics: Condensed Matter |
  | Date | 2013 |
  | Accessed | 9/23/2013, 10:21:39 AM |
  | Library Catalog | Google Scholar |
  | Date Added | 10/11/2013, 10:29:15 AM |
  | Modified | 3/7/2014, 12:09:25 PM |

  ### Notes:

  - Proposed a thermodynamics-based model of protein folding and unfolding.  As an example, apply the model to the HP35 chicken villin headpiece subdomain.

    How SCOP/CATH is used:

    Not using SCOP or CATH data.

    SCOP/CATH reference:

    1. Introduction

    Structural classification schemes disclose that folded proteins  
    have a modular structure. For example SCOP [1] and  
    CATH [2], see also [3], identify around 1200–1300 different  
    folds and topologies; these numbers have changed very  
    little during the past few years.

  ### Attachments

  - 0953-8984\_25\_32\_325103.pdf
- ## Solution NMR of a 463-residue phosphohexomutase: domain 4 mobility, substates, and phosphoryl transfer defect

  |  |  |
  | --- | --- |
  | Type | Journal Article |
  | Author | Akella VS Sarma |
  | Author | Asokan Anbanandam |
  | Author | Allek Kelm |
  | Author | Ritcha Mehra-Chaudhary |
  | Author | Yirui Wei |
  | Author | Peiwu Qin |
  | Author | Yingying Lee |
  | Author | Mark V. Berjanskii |
  | Author | Jacob A. Mick |
  | Author | Lesa J. Beamer |
  | URL | http://pubs.acs.org/doi/abs/10.1021/bi201609n |
  | Volume | 51 |
  | Issue | 3 |
  | Pages | 807–819 |
  | Publication | Biochemistry |
  | Date | 2012 |
  | Accessed | 9/20/2013, 1:18:50 PM |
  | Library Catalog | Google Scholar |
  | Short Title | Solution NMR of a 463-residue phosphohexomutase |
  | Date Added | 10/11/2013, 10:29:15 AM |
  | Modified | 10/11/2013, 10:29:15 AM |

  ### Tags:

  - Catalytic Domain
  - Crystallography, X-Ray
  - Nuclear Magnetic Resonance, Biomolecular
  - Phosphoglucomutase
  - Phosphorylation
  - Phosphotransferases (Phosphomutases)
  - Protein Binding
  - Protein Transport
  - Pseudomonas aeruginosa
  - Substrate Specificity

  ### Notes:

  - NMR study of mobility and function of domain 4 of phosphohexomutase which catalyzes transfer of a phosphoryl group across sugar substrates.

    How SCOP is used:

    Use case: website search

    Description: provide details on superfamily that a domain is found in.

    SCOP reference:

    Bacterial PMM/PGM comprises four mixed α/β-domains encompassing a deep and positively charged catalytic cleft.16 The first three domains share a common fold of a four-stranded β-sheet between two helices.16 Domain 4 is topologically distinct and is classified as a member of the TATA-box binding protein-like superfamily.17

  ### Attachments

  - bi201609n.pdf
  - [HTML] from nih.gov
  - PubMed entry
- ## Solution NMR structure of the helicase associated domain BVU\_0683(627-691) from Bacteroides vulgatus provides first structural coverage for protein domain family PF03457 and indicates domain binding to DNA

  |  |  |
  | --- | --- |
  | Type | Journal Article |
  | Author | Jeffrey L. Mills |
  | Author | Thomas B. Acton |
  | Author | Rong Xiao |
  | Author | John K. Everett |
  | Author | Gaetano T. Montelione |
  | Author | Thomas Szyperski |
  | Volume | 14 |
  | Issue | 1 |
  | Pages | 19-24 |
  | Publication | Journal of Structural and Functional Genomics |
  | ISSN | 1345-711X; 1570-0267 |
  | Date | MAR 2013 |
  | Extra | BCI:BCI201300412186 |
  | DOI | 10.1007/s10969-012-9148-0 |
  | Abstract | A high-quality NMR structure of the helicase associated (HA) domain comprising residues 627-691 of the 753-residue protein BVU\_0683 from Bacteroides vulgatus exhibits an all alpha-helical fold. The structure presented here is the first representative for the large protein domain family PF03457 (currently 742 members) of HA domains. Comparison with structurally similar proteins supports the hypothesis that HA domains bind to DNA and that binding specificity varies greatly within the family of HA domains constituting PF03457. |
  | Date Added | 2/20/2014, 12:24:01 PM |
  | Modified | 2/20/2014, 12:24:01 PM |

  ### Notes:

  - Present first structural representative of Pfam family PF03457.

    How SCOP is used:

    background on protein structure classification.

    SCOP reference:

    The domain BVU\_0683(627–691) was selected as a target of the Protein Structure Initiative and assigned to the Northeast Structural Genomics Consortium (NESG; http://www.nesg.org) for structure determination (NESG Target ID BvR106A) as part of a cooperative inter-center effort aimed at providing structural coverage of large, uncharacterized protein domain families [4]. Initial structural representatives of such fami- lies exhibit high modeling leverage [5], expand our under- standing of protein evolution [6], and generally expand our knowledge of fundamental relationships between protein sequences, three-dimensional structures, and protein func- tion.

  ### Attachments

  - art%3A10.1007%2Fs10969-012-9148-0.pdf
- ## Solution NMR structure of the ribosomal protein RP-L35Ae from Pyrococcus furiosus

  |  |  |
  | --- | --- |
  | Type | Journal Article |
  | Author | David A Snyder |
  | Author | James M Aramini |
  | Author | Bomina Yu |
  | Author | Yuanpeng J Huang |
  | Author | Rong Xiao |
  | Author | John R Cort |
  | Author | Ritu Shastry |
  | Author | Li-Chung Ma |
  | Author | Jinfeng Liu |
  | Author | Burkhard Rost |
  | Author | Thomas B Acton |
  | Author | Michael A Kennedy |
  | Author | Gaetano T Montelione |
  | Volume | 80 |
  | Issue | 7 |
  | Pages | 1901-1906 |
  | Publication | Proteins: Structure, Function, and Bioinformatics |
  | ISSN | 1097-0134 |
  | Date | Jul 2012 |
  | Extra | PMID: 22422653 |
  | Journal Abbr | Proteins |
  | DOI | 10.1002/prot.24071 |
  | Library Catalog | NCBI PubMed |
  | Language | eng |
  | Abstract | The ribosome consists of small and large subunits each composed of dozens of proteins and RNA molecules. However, the functions of many of the individual protomers within the ribosome are still unknown. In this article, we describe the solution NMR structure of the ribosomal protein RP-L35Ae from the archaeon Pyrococcus furiosus. RP-L35Ae is buried within the large subunit of the ribosome and belongs to Pfam protein domain family PF01247, which is highly conserved in eukaryotes, present in a few archaeal genomes, but absent in bacteria. The protein adopts a six-stranded anti-parallel β-barrel analogous to the "tRNA binding motif" fold. The structure of the P. furiosus RP-L35Ae presented in this article constitutes the first structural representative from this protein domain family. |
  | Date Added | 10/11/2013, 10:29:15 AM |
  | Modified | 3/7/2014, 12:11:03 PM |

  ### Tags:

  - Amino Acid Sequence
  - Archaeal Proteins
  - eEF-1A
  - EF-Tu
  - L35Ae
  - Models, Molecular
  - Molecular Sequence Data
  - Nuclear Magnetic Resonance, Biomolecular
  - PF01247
  - Protein Structure, Tertiary
  - Pyrococcus furiosus
  - Recombinant Proteins
  - ribosomal protein
  - Ribosomal Proteins
  - Sequence Alignment
  - solution NMR
  - Static Electricity
  - structural genomics
  - tRNA binding

  ### Notes:

  - Describe solution NMR structure of a ribosomal protein.

    How SCOP/CATH is used:

    Look up SCOP fold classification and list a few examples of proteins with similar folds.

    Reference to SCOP:

    "SCOP34 and CATH35 classify the RP-L35Ae structure to a fold/topology class including such tRNA binding domains as the β-barrel domains of EF-Tu/eEF-1A36 and Gar137 [Fig. 1(e)]."

  ### Attachments

  - nihms364817.pdf
- ## Solution structure, copper binding and backbone dynamics of recombinant Ber e 1-the major allergen from Brazil nut

  |  |  |
  | --- | --- |
  | Type | Journal Article |
  | Author | Louise Rundqvist |
  | Author | Tobias Tengel |
  | Author | Janusz Zdunek |
  | Author | Erik Björn |
  | Author | Jürgen Schleucher |
  | Author | Marcos J C Alcocer |
  | Author | Göran Larsson |
  | Volume | 7 |
  | Issue | 10 |
  | Pages | e46435 |
  | Publication | PloS one |
  | ISSN | 1932-6203 |
  | Date | 2012 |
  | Extra | PMID: 23056307 |
  | Journal Abbr | PLoS ONE |
  | DOI | 10.1371/journal.pone.0046435 |
  | Library Catalog | NCBI PubMed |
  | Language | eng |
  | Abstract | BACKGROUND: The 2S albumin Ber e 1 is the major allergen in Brazil nuts. Previous findings indicated that the protein alone does not cause an allergenic response in mice, but the addition of components from a Brazil nut lipid fraction were required. Structural details of Ber e 1 may contribute to the understanding of the allergenic properties of the protein and its potential interaction partners. METHODOLOGY/PRINCIPAL FINDINGS: The solution structure of recombinant Ber e 1 was solved using NMR spectroscopy and measurements of the protein back bone dynamics at a residue-specific level were extracted using (15)N-spin relaxation. A hydrophobic cavity was identified in the structure of Ber e 1. Using the paramagnetic relaxation enhancement property of Cu(2+) in conjunction with NMR, it was shown that Ber e 1 is able to specifically interact with the divalent copper ion and the binding site was modeled into the structure. The IgE binding region as well as the copper binding site show increased dynamics on both fast ps-ns timescale as well as slower µs-ms timescale. CONCLUSIONS/SIGNIFICANCE: The overall fold of Ber e 1 is similar to other 2S albumins, but the hydrophobic cavity resembles that of a homologous non-specific lipid transfer protein. Ber e 1 is the first 2S albumin shown to interact with Cu(2+) ions. This Cu(2+) binding has minimal effect on the electrostatic potential on the surface of the protein, but the charge distribution within the hydrophobic cavity is significantly altered. As the hydrophobic cavity is likely to be involved in a putative lipid interaction the Cu(2+) can in turn affect the interaction that is essential to provoke an allergenic response. |
  | Date Added | 10/11/2013, 10:29:15 AM |
  | Modified | 10/11/2013, 10:29:15 AM |

  ### Tags:

  - 2S Albumins, Plant
  - Antigens, Plant
  - Bertholletia
  - Copper
  - Interesting
  - Models, Molecular
  - Nuclear Magnetic Resonance, Biomolecular
  - Protein Binding
  - Protein Conformation
  - Recombinant Proteins

  ### Notes:

  - Experimental study (NMR) of structure of Ber e 1, an allergen in Brazil nuts.

    How SCOP is used:

    Search the SCOP database for proteins that share the same fold as Ber e 1.  They tend to have in common extreme temperature tolerance, even though they are non-homologous and not all allergens.

    SCOP reference:

    Comparison with other proteins

    The three-dimensional structure of Ber e 1 was compared to other known protein structures using the DALI server [36], which compares protein three-dimensional structures without taking sequence homology as a prerequisite. As expected from the SCOP database [27], the structure of Ber e 1 has a fold similar to other 2 s albumins, ns-LTPs and amylase inhibitors. However, a vast majority of proteins found to be structurally similar to Ber e 1 are non-homologous and have not been identified as allergens. For example, the 2S albumin fold can appear as a single domain, as in the case of mabinlin, an artificial sweetener, or as a domain in a larger protein such as the C-terminal domain of Thermosynechococcus elongatus circadian clock protein KaiA. Aside from the similarity in their tertiary structure, many of these proteins have the common trait of extreme temperature tolerance.

  ### Attachments

  - journal.pone.0046435.pdf
- ## Solution Structure of a Phytocystatin from Ananas comosus and Its Molecular Interaction with Papain

  |  |  |
  | --- | --- |
  | Type | Journal Article |
  | Author | Deli Irene |
  | Author | Tse-Yu Chung |
  | Author | Bo-Jiun Chen |
  | Author | Ting-Hang Liu |
  | Author | Feng-Yin Li |
  | Author | Jason T. C. Tzen |
  | Author | Cheng-I. Wang |
  | Author | Chia-Lin Chyan |
  | Volume | 7 |
  | Issue | 11 |
  | Pages | e47865 |
  | Publication | Plos One |
  | ISSN | 1932-6203 |
  | Date | NOV 6 2012 |
  | Extra | WOS:000311315300015 |
  | DOI | 10.1371/journal.pone.0047865 |
  | Abstract | The structure of a recombinant pineapple cystatin (AcCYS) was determined by NMR with the RMSD of backbone and heavy atoms of twenty lowest energy structures of 0.56 and 1.11 angstrom, respectively. It reveals an unstructured N-terminal extension and a compact inhibitory domain comprising a four-stranded antiparallel beta-sheet wrapped around a central alpha-helix. The three structural motifs (G(45), Q(89)XVXG, and W-120) putatively responsible for the interaction with papain-like proteases are located in one side of AcCYS. Significant chemical shift perturbations in two loop regions, residues 45 to 48 (GIYD) and residues |
  | Date Added | 2/13/2014, 4:13:17 PM |
  | Modified | 3/7/2014, 12:14:42 PM |
- ## Space-related pharma-motifs for fast search of protein binding motifs and polypharmacological targets

  |  |  |
  | --- | --- |
  | Type | Journal Article |
  | Author | Yi-Yuan Chiu |
  | Author | Chun-Yu Lin |
  | Author | Chih-Ta Lin |
  | Author | Kai-Cheng Hsu |
  | Author | Li-Zen Chang |
  | Author | Jinn-Moon Yang |
  | Volume | 13 |
  | Pages | S21 |
  | Publication | BMC Genomics |
  | ISSN | 1471-2164 |
  | Date | DEC 13 2012 |
  | Extra | WOS:000317183100001 |
  | DOI | 10.1186/1471-2164-13-S7-S21 |
  | Abstract | Background: To discover a compound inhibiting multiple proteins (i.e. polypharmacological targets) is a new paradigm for the complex diseases (e.g. cancers and diabetes). In general, the polypharmacological proteins often share similar local binding environments and motifs. As the exponential growth of the number of protein structures, to find the similar structural binding motifs (pharma-motifs) is an emergency task for drug discovery (e. g. side effects and new uses for old drugs) and protein functions. Results: We have developed a Space-Related Pharmamotifs (called SRPmotif) method to recognize the binding motifs by searching against protein structure database. SRPmotif is able to recognize conserved binding environments containing spatially discontinuous pharma-motifs which are often short conserved peptides with specific physico-chemical properties for protein functions. Among 356 pharma-motifs, 56.5% interacting residues are highly conserved. Experimental results indicate that 81.1% and 92.7% polypharmacological targets of each protein-ligand complex are annotated with same biological process (BP) and molecular function (MF) terms, respectively, based on Gene Ontology (GO). Our experimental results show that the identified pharma-motifs often consist of key residues in functional (active) sites and play the key roles for protein functions. The SRPmotif is available at http://gemdock.life.nctu.edu.tw/SRP/. Conclusions: SRPmotif is able to identify similar pharma-interfaces and pharma-motifs sharing similar binding environments for polypharmacological targets by rapidly searching against the protein structure database. Pharmamotifs describe the conservations of binding environments for drug discovery and protein functions. Additionally, these pharma-motifs provide the clues for discovering new sequence-based motifs to predict protein functions from protein sequence databases. We believe that SRPmotif is useful for elucidating protein functions and drug discovery. |
  | Date Added | 2/20/2014, 12:24:01 PM |
  | Modified | 5/5/2014, 3:10:59 PM |

  ### Notes:

  - Present method for binding site prediction.

    How SCOP/CATH is used:

     Annotate data set.

    SCOP/CATH reference:

    Final, we selected 89 non-redundant protein- ligand complexes (called FDA89) with structure-based classifications (i.e. SCOP [22] and CATH [23]) and all of the proteins are recorded in the UniProt database [24] (Table S1 in additional file 1).

    ...

    . In addition, the structural classifications, SCOP (version 1.75) and CATH (version 3.4), were also used to annotate the polypharmacological targets.

    ...

    **Precision and recall rates were utilized to assess the similarity of biological functions (i.e. BP and MF) and structural classifications (i.e. SCOP and CATH) between polypharmacological targets and their query proteins of protein-ligand complexes.** Based on BP and MF annota- tions, the precision rates are 81.1% and 92.7%, respec- tively (Table 1). These experimental results show that polypharmacological targets not only are involved in the similar cellular process but also perform similar biologi- cal functions. Moreover, the precision rates are 55.2% and 79.7% for SCOP and CATH, respectively (Table 1). In the above results, the polypharmacological targets without annotations are considered as negatives. The precision rates are more than 90% when the polypharma- cological targets without any annotations are removed. The high precision rates show that polypharmacological targets of each protein-ligand complex are usually recorded in the same structure family.

    However, the results with low recall rates may imply that proteins with the same annotation (i.e. biological function or the structure family) sometimes have the key difference in protein-ligand binding environments. For example, viral neuraminidase (NA) of influenza virus is a drug target for prevention of influenza infection and has several homologous proteins (e.g. Sialidase 2 (NEU2)) in the human genome. Both of NA and NEU2 are a type of glycoside hydrolase enzymes (Enzyme Commission num- ber (EC) 3.2.1.18). NA (PDB code: 1NNC[26], chain A) and NEU2 (PDB code: 2F0Z, chain A) have crystal struc- tures with the same drug, Zanamivir (ZMR, listing name Relenza), and are classified as identical structure family in SCOP (b.68.1.1) and CATH (2.120.10.10).

  ### Attachments

  - 1471-2164-13-S7-S21.pdf
- ## Specialized Dynamical Properties of Promiscuous Residues Revealed by Simulated Conformational Ensembles

  |  |  |
  | --- | --- |
  | Type | Journal Article |
  | Author | Arianna Fornili |
  | Author | Alessandro Pandini |
  | Author | Hui-Chun Lu |
  | Author | Franca Fraternali |
  | Volume | 9 |
  | Issue | 11 |
  | Pages | 5127-5147 |
  | Publication | Journal of Chemical Theory and Computation |
  | ISSN | 1549-9618; 1549-9626 |
  | Date | NOV 2013 |
  | Extra | WOS:000327044500043 |
  | DOI | 10.1021/ct400486p |
  | Abstract | The ability to interact with different partners is one of the most important features in proteins. Proteins that bind a large number of partners (hubs) have been often associated with intrinsic disorder. However, many examples exist of hubs with an ordered structure, and evidence of a general mechanism promoting promiscuity in ordered proteins is still elusive. An intriguing hypothesis is that promiscuous binding sites have specific dynamical properties, distinct from the rest of the interface and pre-existing in the protein isolated state. Here, we present the first comprehensive study of the intrinsic dynamics of promiscuous residues in a large protein data set. Different computational methods, from coarse-grained elastic models to geometry-based sampling methods and to full-atom Molecular Dynamics simulations, were used to generate conformational ensembles for the isolated proteins. The flexibility and dynamic correlations of interface residues with a different degree of binding promiscuity were calculated and compared considering side chain and backbone motions, the latter both on a local and on a global scale. The study revealed that (a) promiscuous residues tend to be more flexible than nonpromiscuous ones, (b) this additional flexibility has a higher degree of organization, and (c) evolutionary conservation and binding promiscuity have opposite effects on intrinsic dynamics. Findings on simulated ensembles were also validated on ensembles of experimental structures extracted from the Protein Data Bank (PDB). Additionally, the low occurrence of single nucleotide polymorphisms observed for promiscuous residues indicated a tendency to preserve binding diversity at these positions. A case study on two ubiquitin-like proteins exemplifies how binding promiscuity in evolutionary related proteins can be modulated by the fine-tuning of the interface dynamics. The interplay between promiscuity and flexibility highlighted here can inspire new directions in protein-protein interaction prediction and design methods. |
  | Date Added | 2/20/2014, 12:24:01 PM |
  | Modified | 2/20/2014, 12:24:01 PM |

  ### Notes:

  - Computational study of dynamics of hub proteins.

    How SCOP is used:

    Annotate non-SCOP data set of families prepared using the PiSite database, with SCOP family.  Retain only those families with unambiougous SCOP domain classification across all the members.  Then further narrowed down to those that were single-domain chains.

    Used ASTRAL.

    SCOP reference:

    For each family in nonredundant PiSite (Figure 1), we selected the members with known UniProtKB and SCOP IDs using the PDBSWS PDB/UniProt mapping39 and the SCOP IDs using the Astral SCOP database40 (v 1.75). Only the families with unambiguous SCOP domain classification across all the members and with at least one member with known UniProtKB ID were retained. We then selected 251 families that satisfied the following requirements:

    (1) TheirmembershaveonlyoneSCOPdomain.  
     (2) The sequences of the resolved structures in the family cover at least 75% of the corresponding UniProtKB

    sequences.  
     (3) They have at least one partner with known structure.  
     (4) There is at least one structure in the family with no gaps in

    the resolved main chain. The ungapped X-ray structure with the best resolution in the family was selected as the structural representative (SR) to be used in the simulations and structural analyses. When no crystallographic structure with a complete main chain was found, an ungapped NMR structure was selected as SR if available.

    The 251 families define our full data set SFull (Supporting Information (SI) Table S1). Each family includes on average ∼20 members, for a total of 4917 PDB chains.

    ...

    ■ RESULTS

    A data set of 251 monodomain proteins (SFull) was extracted from the PDB and partitioned into 151 monopartner and 100 multipartner proteins using the PiSite database38 (Methods). The composition of the data set in terms of 7 general protein function categories (SI Figure S1) was obtained using a functional annotation of SCOP superfamilies.104,105 As ex- pected,2,12,33 monopartner proteins (cyan) showed an enrich-

    ment in the metabolism and general categories, while multi- partner proteins (magenta) were particularly rich in the categories related to extra- and intracellular processes, information, and regulation.

  ### Attachments

  - ct400486p.pdf
- ## Specificity and affinity quantification of protein-protein interactions

  |  |  |
  | --- | --- |
  | Type | Journal Article |
  | Author | Zhiqiang Yan |
  | Author | Liyong Guo |
  | Author | Liang Hu |
  | Author | Jin Wang |
  | Volume | 29 |
  | Issue | 9 |
  | Pages | 1127-1133 |
  | Publication | Bioinformatics |
  | ISSN | 1367-4803 |
  | Date | MAY 1 2013 |
  | Extra | WOS:000318573900004 |
  | DOI | 10.1093/bioinformatics/btt121 |
  | Abstract | Motivation: Most biological processes are mediated by the protein-protein interactions. Determination of the protein-protein structures and insight into their interactions are vital to understand the mechanisms of protein functions. Currently, compared with the isolated protein structures, only a small fraction of protein-protein structures are experimentally solved. Therefore, the computational docking methods play an increasing role in predicting the structures and interactions of protein-protein complexes. The scoring function of protein-protein interactions is the key responsible for the accuracy of the computational docking. Previous scoring functions were mostly developed by optimizing the binding affinity which determines the stability of the protein-protein complex, but they are often lack of the consideration of specificity which determines the discrimination of native protein-protein complex against competitive ones. Results: We developed a scoring function (named as SPA-PP, specificity and affinity of the protein-protein interactions) by incorporating both the specificity and affinity into the optimization strategy. The testing results and comparisons with other scoring functions show that SPA-PP performs remarkably on both predictions of binding pose and binding affinity. Thus, SPA-PP is a promising quantification of protein-protein interactions, which can be implemented into the protein docking tools and applied for the predictions of protein-protein structure and affinity. |
  | Date Added | 2/20/2014, 12:24:01 PM |
  | Modified | 2/20/2014, 12:24:01 PM |

  ### Notes:

  - Present a new scoring function for protein-protein interactions for use in PPI predictions.

    How SCOP is used:

    background on protein structure classification.

    SCOP reference:

    In reality, there is only finite number of protein folds (⬚⬚1300) in nature (Andreeva et al., 2008).

  ### Attachments

  - Bioinformatics-2013-Yan-1127-33.pdf
- ## Stability and rigidity/flexibility-two sides of the same coin?

  |  |  |
  | --- | --- |
  | Type | Journal Article |
  | Author | Tatyana B. Mamonova |
  | Author | Anna V. Glyakina |
  | Author | Oxana V. Galzitskaya |
  | Author | Maria G. Kurnikova |
  | URL | http://www.sciencedirect.com/science/article/pii/S1570963913000745 |
  | Publication | Biochimica et Biophysica Acta (BBA)-Proteins and Proteomics |
  | Date | 2013 |
  | Accessed | 9/23/2013, 10:22:14 AM |
  | Library Catalog | Google Scholar |
  | Date Added | 10/11/2013, 10:29:15 AM |
  | Modified | 10/11/2013, 10:29:15 AM |

  ### Tags:

  - Flexible region
  - Hydrogen bond
  - Molecular dynamics simulation
  - Proteins from thermophilic and mesophilic organisms
  - Salt bridge

  ### Notes:

  - Compare rigidity/flexibility predictions of a fully sequence-based method (FoldUnfold) with a 3D-structure based method (MDFirst).

    How SCOP is used:

    1. To ensure structural diversity of their 4 protein data set,  each protein from a different SCOP class.

    2. In a previous publications, parameters for the FoldUnfold program were trained using a data set from SCOP.

    SCOP reference:

    2.1. Dataset of proteins

    ...

    In this work we use four pairs of homologous proteins cho- sen such that each pair belongs to one of the four main SCOP structural classes [36] of water-soluble globular proteins.

    ...

    2.2. FoldUnfold for prediction of flexible regions

    The FoldUnfold program [26] is used in this work to predict flexible regions in four pairs of proteins with a known structure. The FoldUnfold program is accessible at http://bioinfo.protres.ru/ogu/. The principle of its operation is described elsewhere [26–28]. Such a property of resi- dues as the observed average number of contacts in a globular state is used in the program. The average number of contacts for each of the 20 types of amino acid residues was calculated for 5829 protein struc- tures from the SCOP protein database [27].

  ### Attachments

  - 1-s2.0-S1570963913000745-main.pdf
  - Snapshot
- ## Statistical significance of threading scores

  |  |  |
  | --- | --- |
  | Type | Journal Article |
  | Author | Afshin Fayyaz Movaghar |
  | Author | Guillaume Launay |
  | Author | Sophie Schbath |
  | Author | Jean-François Gibrat |
  | Author | François Rodolphe |
  | Volume | 19 |
  | Issue | 1 |
  | Pages | 13-29 |
  | Publication | Journal of Computational Biology |
  | ISSN | 1557-8666 |
  | Date | Jan 2012 |
  | Extra | PMID: 22149633 |
  | Journal Abbr | J. Comput. Biol. |
  | DOI | 10.1089/cmb.2011.0236 |
  | Library Catalog | NCBI PubMed |
  | Language | eng |
  | Abstract | We present a general method for assessing threading score significance. The threading score of a protein sequence, thread onto a given structure, should be compared with the threading score distribution of a random amino-acid sequence, of the same length, thread on the same structure; small p-values point significantly high scores. We claim that, due to general protein contact map properties, this reference distribution is a Weibull extreme value distribution whose parameters depend on the threading method, the structure, the length of the query and the random sequence simulation model used. These parameters can be estimated off-line with simulated sequence samples, for different sequence lengths. They can further be interpolated at the exact length of a query, enabling the quick computation of the p-value. |
  | Date Added | 10/11/2013, 10:29:15 AM |
  | Modified | 12/2/2013, 4:23:37 PM |

  ### Tags:

  - Algorithms
  - Amino Acid Sequence
  - Computational Biology
  - computational molecular biology
  - Computer Simulation
  - Markov chains
  - Markov Chains
  - Models, Statistical
  - Protein Conformation
  - Proteins
  - Sequence Alignment
  - Sequence Analysis
  - sequence analysis
  - Statistical Distributions
  - statistics
  - stochastic process

  ### Notes:

  - Investigate method for determining the "significance" of a threading score, and whether threading score correlates with the likelihood that a query sequence belongs to some fold.

    How SCOP is used:

    Validate method on data sets curated from SCOP.  Validate that sequences from the same family and superfamily have smaller p-values.

    1. All proteins within the same SCOP family as 1gtvA

    2. All proteins within the same superfamily as 1gtvA

    3. Proteins with distinct SCOP folds from 1gtvA

    SCOP reference:

    3.2.1. Threading different sequences onto a fold

    Data. Four sets of real protein sequences were constituted representing different similarity classes with respect to the structure of 1gtvA.

    The first set (‘‘psi-blast homologs’’) consists of proteins collected via psi-blast, thus sharing a significant sequence similarity with 1gtvA. Their structure is unknown, but almost certainly similar to that of 1gtvA, owing to the clear sequence similarity.

    The next three sets are disjoint and their structures are experimentally known.

    The second set (‘‘SCOP family’’) contains all proteins belonging to the same SCOP family as 1gtvA. These proteins share a structural and functional similarity with 1gtvA, and are certainly homologous.

    The third set (‘‘SCOP superfamily’’) contains all proteins belonging to the same SCOP superfamily than 1gtvA, but to a different family. These proteins present only a moderate sequence similarity with 1gtvA, less than 20% sequence identity, although they are structurally related.

    The fourth set (‘‘other SCOP folds’’) collects proteins belonging to other folds, thus presenting no structural similarity with 1gtvA, and less than 20% sequence identity with 1gtvA. These are in fact 44 ‘‘alpha-beta’’ protein sequences of length lying between 187 and 226, thus belonging to the ‘‘global regime.’’

  ### Attachments

  - cmb.2011.0236.pdf
  - [HTML] from nih.gov
- ## Structural alphabet motif discovery and a structural motif database

  |  |  |
  | --- | --- |
  | Type | Journal Article |
  | Author | Shih-Yen Ku |
  | Author | Yuh-Jyh Hu |
  | URL | http://www.sciencedirect.com/science/article/pii/S0010482511002095 |
  | Volume | 42 |
  | Issue | 1 |
  | Pages | 93–105 |
  | Publication | Computers in Biology and Medicine |
  | Date | 2012 |
  | Accessed | 9/23/2013, 10:22:49 AM |
  | Library Catalog | Google Scholar |
  | Date Added | 10/11/2013, 10:29:15 AM |
  | Modified | 3/7/2014, 1:07:02 PM |

  ### Tags:

  - Motif-finding tools
  - Protein structures
  - Sequence motifs
  - Structural alphabets
  - Structural motifs

  ### Notes:

  - Presents structural motif database called SA-Motifbase. It's based on the conserved structural motifs found at different levels in SCOP.

    How SCOP is used:

    The organization of the SA-Motifbase database is based on SCOP's hierarchy, specifically for fold, superfamily, and family. In analyzing the motifs, the organization is based on the recurring times under each of these levels.

    SCOP Reference:

    We identified the conserved structural motifs for each fold,  
    superfamily, and family in SCOP [7].

    We divided the structural motif discovery process into two  
    stages. First, for a protein group, such as a SCOP family, we transformed  
    the protein in its 3D structure into a structural alphabet  
    sequence. Various structural alphabets have been developed based  
    on different design strategies and domain knowledge [17–23]. Their  
    size can vary from a dozen to nearly a hundred.

    Numerous sequence motif-finding tools exist [8–14], which use different search strategies  
    and objective functions to identify and evaluate motifs. We  
    selected MEME [7] to discover the motifs from the proteins in each  
    fold, superfamily, and family of SCOP because MEME is freely  
    accessible, and it provides a convenient web-based interface. In  
    MEME, a motif is represented as a position weight matrix. It is more  
    expressive, and can be converted easily into a regular expression  
    based on specified weight thresholds.

    SA-Motifbase stores the structural alphabet motifs that characterize  
    the local structural segments that are conserved in the  
    SCOP protein hierarchy. For each motif, SA-Motifbase records its  
    alphabet letter preference, the alphabet letter frequency distribution,  
    and the significance.

    We summarized in Table 1 the statistics of the motifs identified  
    from the folds, superfamilies, and families in the SCOP  
    database. The statistics include the number of proteins containing  
    the motifs, the average number of motifs in a protein, the total  
    number of motifs, as well as the mean size of the motifs. There are  
    83% and 82% of the proteins at the fold and superfamily levels that  
    contain the structural alphabet motifs, respectively.

    To demonstrate that  
    the proposed approach is capable of characterizing the structural  
    bba-unit, we analyzed the structural motifs discovered from the  
    g.37.1 superfamily in SCOP. A motif was considered to match a  
    subdomain correctly if over half the residues in the subdomain  
    were included in the motif.

  ### Attachments

  - 1-s2.0-S0010482511002095-main.pdf
  - Snapshot
- ## Structural and Biochemical Basis of Yos9 Protein Dimerization and Possible Contribution to Self-association of 3-Hydroxy-3-methylglutaryl-Coenzyme A Reductase Degradation Ubiquitin-Ligase Complex

  |  |  |
  | --- | --- |
  | Type | Journal Article |
  | Author | Jennifer Hanna |
  | Author | Anja Schuetz |
  | Author | Franziska Zimmermann |
  | Author | Joachim Behlke |
  | Author | Thomas Sommer |
  | Author | Udo Heinemann |
  | Volume | 287 |
  | Issue | 11 |
  | Pages | 8633–8640 |
  | Publication | Journal of Biological Chemistry |
  | Date | March 2012 |
  | DOI | 10.1074/jbc.M111.317644 |
  | Abstract | In yeast, the membrane-bound HMG-CoA reductase degradation (HRD) ubiquitin-ligase complex is a key player of the ER-associated protein degradation pathway that targets misfolded proteins for proteolysis. Yos9, a component of the luminal submodule of the ligase, scans proteins for specific oligosaccharide modifications, which constitute a critical determinant of the degradation signal. Here, we report the crystal structure of the Yos9 domain that was previously suggested to confer binding to Hrd3, another component of the HRD complex. We observe an alpha beta-roll domain architecture and a dimeric assembly which are confirmed by analytical ultracentrifugation of both the crystallized domain and full-length Yos9. Our binding studies indicate that, instead of this domain, the N-terminal part of Yos9 including the mannose 6-phosphate receptor homology domain mediates the association with Hrd3 in vitro. Our results support the model of a dimeric state of the HRD complex and provide first-time evidence of self-association on its luminal side. |
  | Date Added | 3/7/2014, 12:08:00 PM |
  | Modified | 3/7/2014, 12:08:00 PM |
- ## Structural and Biochemical Characterization of Phage lambda FI Protein (gpFI) Reveals a Novel Mechanism of DNA Packaging Chaperone Activity

  |  |  |
  | --- | --- |
  | Type | Journal Article |
  | Author | Ana Popovic |
  | Author | Bin Wu |
  | Author | Cheryl H. Arrowsmith |
  | Author | Aled M. Edwards |
  | Author | Alan R. Davidson |
  | Author | Karen L. Maxwell |
  | Volume | 287 |
  | Issue | 38 |
  | Pages | 32085-32095 |
  | Publication | Journal of Biological Chemistry |
  | ISSN | 0021-9258 |
  | Date | SEP 14 2012 |
  | DOI | 10.1074/jbc.M112.378349 |
  | Language | English |
  | Abstract | One of the final steps in the morphogenetic pathway of phage lambda is the packaging of a single genome into a preformed empty head structure. In addition to the terminase enzyme, the packaging chaperone, FI protein (gpFI), is required for efficient DNA packaging. In this study, we demonstrate an interaction between gpFI and the major head protein, gpE. Amino acid substitutions in gpFI that reduced the strength of this interaction also decreased the biological activity of gpFI, implying that this head binding activity is essential for the function of gpFI. We also show that gpFI is a two-domain protein, and the C-terminal domain is responsible for the head binding activity. Using nuclear magnetic resonance spectroscopy, we determined the three-dimensional structure of the C-terminal domain and characterized the helical nature of the N-terminal domain. Through structural comparisons, we were able to identify two previously unannotated prophage-encoded proteins with tertiary structures similar to gpFI, although they lack significant pairwise sequence identity. Sequence analysis of these diverse homologues led us to identify related proteins in a variety of myo- and siphophages, revealing that gpFI function has a more highly conserved role in phage morphogenesis than was previously appreciated. Finally, we present a novel model for the mechanism of gpFI chaperone activity in the DNA packaging reaction of phage lambda. |
  | Date Added | 10/11/2013, 10:29:15 AM |
  | Modified | 11/11/2013, 4:46:17 PM |

  ### Notes:

  - Study of Phage lambada F1 Protein (gpF1)

    How SCOP is used:

    Use SCOP to look up family-level of two structural homologs of protein of interest.

    SCOP reference:

    Putative gpFI Homologues Are Found in Contractile and Non- contractile Tailed Phages and Prophages—To identify structural homologues of the gpFI C-terminal domain fold, we performed a DALI (27) search. This search yielded significant hits to two prophage-encoded proteins: Bacillus subtilis YqbF (Pro- tein Data Bank code 2HJQ) and Haemophilus influenzae HI1506 (Protein Data Bank code 2OUT). The SCOP database (28) classifies both YqbF and HI1506 as belonging to the GINS/ PriA/YqbF domain family.

  ### Attachments

  - J. Biol. Chem.-2012-Popovic-32085-95.pdf
- ## Structural and dynamic aspects of Ca2+ and Mg2+ binding of the regulatory domains of the Na/Ca2+ exchanger

  |  |  |
  | --- | --- |
  | Type | Journal Article |
  | Author | Vincent Breukels |
  | Author | Wouter G. Touw |
  | Author | Geerten W. Vuister |
  | URL | http://31.24.0.70/bst/040/0409/0400409.pdf |
  | Volume | 40 |
  | Issue | part 2 |
  | Publication | Biochemical Society Transactions |
  | Date | 2012 |
  | Accessed | 9/23/2013, 10:16:21 AM |
  | Library Catalog | Google Scholar |
  | Date Added | 10/11/2013, 10:29:15 AM |
  | Modified | 10/11/2013, 10:29:15 AM |

  ### Tags:

  - calcium binding
  - Calx-beta
  - Interesting
  - magnesium binding
  - protein dynamics
  - protein stability
  - sodium/calcium exchanger (NCX)

  ### Notes:

  - Describe fold and binding activity of the NCX (Na+/CA2+ exchanger) membrane protein.

    How SCOP is used:

    Discuss how two homologous domains in NCX are classified as two different domains in domain databases. The two domains have some structural differences.

    SCOP reference:

    The Calx-β domains belong to the superclass of immunoglobulin-like folds, which also comprises other members such as C2, cadherin and immunoglobulin domains. The C2-domains, present in phospholipases, protein kinases C and synaptotagmins, are not only structurally homologous, but also bind Ca2 + . **Although the Calx-****β** **and C2-domains bear structural homology, the domains are usually classified as different motifs in domain databases such as SCOP [23].**   The β-sandwich of C2-domains is composed of eight strands instead of the seven strands that build up the sandwich of the Calx-β domains. Furthermore, a multiple structural alignment of several representative C2- and Calx-β domains using MUSTANG [24] shows that the Ca2 + binding sites are located at opposite sites of the β-sandwich. The ‘BC’ (L2) and ‘FG’ loops (L5) of C2 bind Ca2 + , while the AB, CD and EFloopsbindCa2+ inCalx-βdomains.

  ### Attachments

  - [PDF] from 31.24.0.70
- ## Structural and functional analysis of multi-interface domains

  |  |  |
  | --- | --- |
  | Type | Journal Article |
  | Author | Liang Zhao |
  | Author | Steven C H Hoi |
  | Author | Limsoon Wong |
  | Author | Tobias Hamp |
  | Author | Jinyan Li |
  | Volume | 7 |
  | Issue | 12 |
  | Pages | e50821 |
  | Publication | PloS one |
  | ISSN | 1932-6203 |
  | Date | 2012 |
  | Extra | PMID: 23272073 |
  | Journal Abbr | PLoS ONE |
  | DOI | 10.1371/journal.pone.0050821 |
  | Library Catalog | NCBI PubMed |
  | Language | eng |
  | Abstract | A multi-interface domain is a domain that can shape multiple and distinctive binding sites to contact with many other domains, forming a hub in domain-domain interaction networks. The functions played by the multiple interfaces are usually different, but there is no strict bijection between the functions and interfaces as some subsets of the interfaces play the same function. This work applies graph theory and algorithms to discover fingerprints for the multiple interfaces of a domain and to establish associations between the interfaces and functions, based on a huge set of multi-interface proteins from PDB. We found that about 40% of proteins have the multi-interface property, however the involved multi-interface domains account for only a tiny fraction (1.8%) of the total number of domains. The interfaces of these domains are distinguishable in terms of their fingerprints, indicating the functional specificity of the multiple interfaces in a domain. Furthermore, we observed that both cooperative and distinctive structural patterns, which will be useful for protein engineering, exist in the multiple interfaces of a domain. |
  | Date Added | 10/11/2013, 10:29:15 AM |
  | Modified | 10/11/2013, 10:29:15 AM |

  ### Tags:

  - Algorithms
  - Animals
  - Binding Sites
  - Cluster Analysis
  - Crystallography, X-Ray
  - Databases, Protein
  - Humans
  - Ligands
  - Models, Molecular
  - Models, Statistical
  - Molecular Conformation
  - Proteasome Endopeptidase Complex
  - Protein Binding
  - Protein Conformation
  - Protein Interaction Mapping
  - Proteins
  - Protein Structure, Secondary
  - Protein Structure, Tertiary

  ### Notes:

  - Computational study of proteins with multiple interfaces.

    A multi-interface domain is a domain that can shape multiple and distinctive binding sites to contact with many other domains, forming a hub in domain-domain interaction networks.

    How SCOP is used:

    Get SCOP classification for a database of multi-interface proteins.  If the SCOP classifcaition is unavailable, attempt to get from similar structures using PDBeFold.

    Examine the distribution of multiple interface at different SCOP classification levels.

    SCOP reference:

    Aggregation of multi-interface proteins

    To explore which domains have multiple interfaces as well as their distributions in PDB, we aggregate all these 5,222 multi- interface proteins according to their structural annotations.

    Protein structural annotations are obtained through following steps. First we directly retrieve each multi-interface protein’s structural annotations from SCOP [1]. Then, for those proteins that do not have SCOP annotations, we employ PDBeFold [34] to search for annotations of similar proteins stored in SCOP. Among the results generated by PDBeFold for a given protein, we chose the one with the best Q-score as the target domain and retrieve the complete information of the protein containing this domain from SCOP.

    Based on structural annotations, multi-interface proteins are further aggregated into several groups in accordance with SCOP classification, as per the following steps: (i) Align each multi- interface protein sequence to its target domain sequence. (ii) Categorize each interface to a domain by the interface residues’ position and domain range. If the entire set of interface residues fall into one domain for a given interface then it is annotated by that domain identifier; otherwise, multiple domain identifiers are tagged to that interface. (iii) Aggregate multi-interface proteins into clusters at different SCOP classification levels, i.e., class, fold, superfamily, family, and domain, according to their annotations.

    ...

    Figure 4 is produced by PHYLIP [44] based on 2,517 of the 5,222 multi-interface proteins that have SCOP annotations. It shows the number distribution of multi-interface proteins at different SCOP classification levels. Obviously, multi-interface domains can appear in a broad range of clusters in terms of SCOP classification. Among all the eleven classes in SCOP, a=b proteins, azb proteins, all-b proteins, and all-a proteins account for 90.3% of all the multi-interface proteins. Figure 4 also indicates that all-b proteins, or at least part of them, are less conservable since they have the largest number of multi-interface proteins in one domain. It can be also seen that multi-interface proteins with a large variability tend to aggregate to a small number of clusters instead of uniformly spread out to each cluster as shown in Figure 4.

    Table 2 gives the distribution of the 2,517 multi-interface proteins at different levels of SCOP classification. The complete number of sub-levels for each classification level is retrieved from SCOP [1], while the number of sub-levels with multiple interfaces for each level is determined by the number of multi-interface domains ‘‘upgraded’’ from the domain level to the class level. It can be seen from Figure 4 that, while multi-interface proteins exist over all classes of SCOP classification, they clearly favor a few of the sub-levels. In particular, although there are more than 110,000 domains with annotation in SCOP [1], only a very small proportion of these domains (1,730/97,178) have the multi- interface property. This phenomenon also suggests that all biological processes have their own small set of pivotal proteins [45].

  ### Attachments

  - [HTML] from plos.org
  - journal.pone.0050821.pdf
  - PubMed entry
- ## Structural and functional analysis of the archaeal endonuclease Nob1

  |  |  |
  | --- | --- |
  | Type | Journal Article |
  | Author | Thomas Veith |
  | Author | Roman Martin |
  | Author | Jan P. Wurm |
  | Author | Benjamin L. Weis |
  | Author | Elke Duchardt-Ferner |
  | Author | Charlotta Safferthal |
  | Author | Raoul Hennig |
  | Author | Oliver Mirus |
  | Author | Markus T. Bohnsack |
  | Author | Jens Wöhnert |
  | URL | http://nar.oxfordjournals.org/content/40/7/3259.short |
  | Volume | 40 |
  | Issue | 7 |
  | Pages | 3259–3274 |
  | Publication | Nucleic Acids Research |
  | Date | 2012 |
  | Accessed | 9/20/2013, 1:19:35 PM |
  | Library Catalog | Google Scholar |
  | Date Added | 10/11/2013, 10:20:13 AM |
  | Modified | 11/12/2013, 4:28:36 PM |

  ### Notes:

  - Analysis of the structure and function of Nob1, involved in ribosome assembly, through sequence alignment, phylogenetic analysis, biochem assays, structure determination, database comparison, etc.

    How SCOP is used:

    For structure comparison: Used the PIN and zinc ribbon domain found in many nucleases and got their structures as classified by the SCOP database.

    Sunids that are listed are for superfamilies.

    I looked through the section describing the results of structure comparison and found that they were only able to find differences.  For the PIN domain, the domain that they found that was most similar "Unfortunately, the function of this factor is currently unknown and thus, no functional comparison of the two proteins is possible."

    SCOP reference:

    **Under MATERIALS AND METHODS**

    **Structure comparison**  
    "Structures of PIN (scop:88723) and zinc ribbon domains  
    (scop:57783 and scop:144206) were identified by their  
    SCOP (61) classification and downloaded from the protein  
    data bank (PDB) (www.pdb.org) (62). Structures were  
    loaded in YASARA (www.yasara.org) and superimposed  
    with its MUSTANG plugin (63) onto the domains of  
    PhNob1 to calculate the root mean square deviation and  
    sequence (RMSD) similarity."

  ### Attachments

  - Nucl. Acids Res.-2012-Veith-3259-74.pdf
  - PubMed entry
- ## Structural and Functional Characterization of Bc28.1, Major Erythrocyte-binding Protein from Babesia canis Merozoite Surface

  |  |  |
  | --- | --- |
  | Type | Journal Article |
  | Author | Yin-Shan Yang |
  | Author | Brice Murciano |
  | Author | Karina Moubri |
  | Author | Prisca Cibrelus |
  | Author | Theo Schetters |
  | Author | Andre Gorenflot |
  | Author | Stephane Delbecq |
  | Author | Christian Roumestand |
  | Volume | 287 |
  | Issue | 12 |
  | Pages | 9495-9508 |
  | Publication | Journal of Biological Chemistry |
  | ISSN | 0021-9258 |
  | Date | MAR 16 2012 |
  | Extra | WOS:000301797800078 |
  | DOI | 10.1074/jbc.M111.260745 |
  | Abstract | Babesiosis (formerly known as piroplasmosis) is a tick-borne disease caused by the intraerythrocytic development of protozoa parasites from the genus Babesia. Like Plasmodium falciparum, the agent of malaria, or Toxoplasma gondii, responsible for human toxoplasmosis, Babesia belongs to the Apicomplexa family. Babesia canis is the agent of the canine babesiosis in Europe. Clinical manifestations of this disease range from mild to severe and possibly lead to death by multiple organ failure. The identification and characterization of parasite surface proteins represent major goals, both for the understanding of the Apicomplexa invasion process and for the vaccine potential of such antigens. Indeed, we have already shown that Bd37, the major antigenic adhesion protein from Babesia divergens, the agent of bovine babesiosis, was able to induce complete protection against various parasite strains. The major merozoite surface antigens of Babesia canis have been described as a 28-kDa membrane protein family, anchored at the surface of the merozoite. Here, we demonstrate that Bc28.1, a major member of this multigenic family, is expressed at high levels at the surface of the merozoite. This protein is also found in the parasite in vitro culture supernatants, which are the basis of effective vaccines against canine babesiosis. We defined the erythrocyte binding function of Bc28.1 and determined its high resolution solution structure using NMR spectroscopy. Surprisingly, although these proteins are thought to play a similar role in the adhesion process, the structure of Bc28.1 from B. canis appears unrelated to the previously published structure of Bd37 from B. divergens. Site-directed mutagenesis experiments also suggest that the mechanism of the interaction with the erythrocyte membrane could be different for the two proteins. The resolution of the structure of Bc28 represents a milestone for the characterization of the parasite erythrocyte binding and its interaction with the host immune system. |
  | Date Added | 2/13/2014, 4:13:17 PM |
  | Modified | 3/7/2014, 12:10:26 PM |
- ## Structural and functional characterization of the N-terminal domain of the yeast Mg2+ channel Mrs2

  |  |  |
  | --- | --- |
  | Type | Journal Article |
  | Author | Muhammad Bashir Khan |
  | Author | Gerhard Sponder |
  | Author | Bjoern Sjoeblom |
  | Author | Sona Svidova |
  | Author | Rudolf J. Schweyen |
  | Author | Oliviero Carugo |
  | Author | Kristina Djinovic-Carugo |
  | Volume | 69 |
  | Pages | 1653–1664 |
  | Publication | Acta Crystallographica Section D-biological Crystallography |
  | Date | September 2013 |
  | DOI | 10.1107/S0907444913011712 |
  | Abstract | Mg2+ translocation across cellular membranes is crucial for a myriad of physiological processes. Eukaryotic Mrs2 transporters are distantly related to the major bacterial Mg2+ transporter CorA, the structure of which displays a bundle of giant alpha-helices forming a long pore that extends beyond the membrane before widening into a funnel-shaped cytosolic domain. Here, a functional and structural analysis of the regulatory domain of the eukaryotic Mg2+ channel Mrs2 from the yeast inner mitochondrial membrane is presented using crystallography, genetics, biochemistry and fluorescence spectroscopy. Surprisingly, the fold of the Mrs2 regulatory domain bears notable differences compared with the related bacterial channel CorA. Nevertheless, structural analysis showed that analogous residues form functionally critical sites, notably the hydrophobic gate and the Mg2+-sensing site. Validation of candidate residues was performed by functional studies of mutants in isolated yeast mitochondria. Measurements of the Mg2+ influx into mitochondria confirmed the involvement of Met309 as the major gating residue in Mrs2, corresponding to Met291 in CorA. |
  | Date Added | 3/7/2014, 1:06:24 PM |
  | Modified | 3/7/2014, 1:06:24 PM |
- ## Structural and functional insights into alphavirus polyprotein processing and pathogenesis

  |  |  |
  | --- | --- |
  | Type | Journal Article |
  | Author | Gyehwa Shin |
  | Author | Samantha A. Yost |
  | Author | Matthew T. Miller |
  | Author | Elizabeth J. Elrod |
  | Author | Arash Grakoui |
  | Author | Joseph Marcotrigiano |
  | Volume | 109 |
  | Issue | 41 |
  | Pages | 16534–16539 |
  | Publication | Proceedings of the National Academy of Sciences of the United States of America |
  | Date | October 2012 |
  | DOI | 10.1073/pnas.1210418109 |
  | Abstract | Alphaviruses, a group of positive-sense RNA viruses, are globally distributed arboviruses capable of causing rash, arthritis, encephalitis, and death in humans. The viral replication machinery consists of four nonstructural proteins (nsP1-4) produced as a single polyprotein. Processing of the polyprotein occurs in a highly regulated manner, with cleavage at the P2/3 junction influencing RNA template use during genome replication. Here, we report the structure of P23 in a precleavage form. The proteins form an extensive interface and nsP3 creates a ring structure that encircles nsP2. The P2/3 cleavage site is located at the base of a narrow cleft and is not readily accessible, suggesting a highly regulated cleavage. The nsP2 protease active site is over 40 angstrom away from the P2/3 cleavage site, supporting a trans cleavage mechanism. nsP3 contains a previously uncharacterized protein fold with a zinc-coordination site. Known mutations in nsP2 that result in formation of noncytopathic viruses or a temperature sensitive phenotype cluster at the nsP2/nsP3 interface. Structure-based mutations in nsP3 opposite the location of the nsP2 noncytopathic mutations prevent efficient cleavage of P23, affect RNA infectivity, and alter viral RNA production levels, highlighting the importance of the nsP2/nsP3 interaction in pathogenesis. A potential RNA-binding surface, spanning both nsP2 and nsP3, is proposed based on the location of ion-binding sites and adaptive mutations. These results offer unexpected insights into viral protein processing and pathogenesis that may be applicable to other polyprotein-encoding viruses such as HIV, hepatitis C virus (HCV), and Dengue virus. |
  | Date Added | 3/7/2014, 1:06:24 PM |
  | Modified | 3/7/2014, 1:06:24 PM |
- ## Structural and functional insights into (S)-ureidoglycolate dehydrogenase, a metabolic branch point enzyme in nitrogen utilization

  |  |  |
  | --- | --- |
  | Type | Journal Article |
  | Author | Myung-Il Kim |
  | Author | Inchul Shin |
  | Author | Suhee Cho |
  | Author | Jeehyun Lee |
  | Author | Sangkee Rhee |
  | Volume | 7 |
  | Issue | 12 |
  | Pages | e52066 |
  | Publication | PloS one |
  | ISSN | 1932-6203 |
  | Date | 2012 |
  | Extra | PMID: 23284870 |
  | Journal Abbr | PLoS ONE |
  | DOI | 10.1371/journal.pone.0052066 |
  | Library Catalog | NCBI PubMed |
  | Language | eng |
  | Abstract | Nitrogen metabolism is one of essential processes in living organisms. The catabolic pathways of nitrogenous compounds play a pivotal role in the storage and recovery of nitrogen. In Escherichia coli, two different, interconnecting metabolic routes drive nitrogen utilization through purine degradation metabolites. The enzyme (S)-ureidoglycolate dehydrogenase (AllD), which is a member of l-sulfolactate dehydrogenase-like family, converts (S)-ureidoglycolate, a key intermediate in the purine degradation pathway, to oxalurate in an NAD(P)-dependent manner. Therefore, AllD is a metabolic branch-point enzyme for nitrogen metabolism in E. coli. Here, we report crystal structures of AllD in its apo form, in a binary complex with NADH cofactor, and in a ternary complex with NADH and glyoxylate, a possible spontaneous degradation product of oxalurate. Structural analyses revealed that NADH in an extended conformation is bound to an NADH-binding fold with three distinct domains that differ from those of the canonical NADH-binding fold. We also characterized ligand-induced structural changes, as well as the binding mode of glyoxylate, in the active site near the NADH nicotinamide ring. Based on structural and kinetic analyses, we concluded that AllD selectively utilizes NAD(+) as a cofactor, and further propose that His116 acts as a general catalytic base and that a hydride transfer is possible on the B-face of the nicotinamide ring of the cofactor. Other residues conserved in the active sites of this novel l-sulfolactate dehydrogenase-like family also play essential roles in catalysis. |
  | Date Added | 10/11/2013, 10:29:15 AM |
  | Modified | 10/11/2013, 10:29:15 AM |

  ### Tags:

  - Alcohol Oxidoreductases
  - Amino Acid Sequence
  - Binding Sites
  - Enzyme Activation
  - Glyoxylates
  - Models, Molecular
  - Molecular Docking Simulation
  - Molecular Sequence Data
  - NAD
  - Nitrogen
  - Protein Binding
  - Protein Conformation
  - Protein Multimerization
  - Sequence Alignment

  ### Notes:

  - Present crystal structure of an enzyme, E. coli AllD.

    How SCOP is used:

    Describe SCOP classification of the family for protein studied.

    SCOP reference:

    The enzyme (S)-ureidoglycolate dehydrogenase (EC 1.1.1.154) was designated as the gene product of allD in E. coli [17] and belongs to a member of L-sulfolactate dehydrogenase-like protein family [18].

  ### Attachments

  - [HTML] from plos.org
  - journal.pone.0052066.pdf
  - PubMed entry
- ## Structural and functional studies of S-adenosyl-L-methionine binding proteins: a ligand-centric approach

  |  |  |
  | --- | --- |
  | Type | Journal Article |
  | Author | Rajaram Gana |
  | Author | Shruti Rao |
  | Author | Hongzhan Huang |
  | Author | Cathy Wu |
  | Author | Sona Vasudevan |
  | Volume | 13 |
  | Publication | Bmc Structural Biology |
  | ISSN | 1472-6807 |
  | Date | APR 25 2013 |
  | Extra | WOS:000319457100001 |
  | DOI | 10.1186/1472-6807-13-6 |
  | Abstract | Background: The post-genomic era poses several challenges. The biggest is the identification of biochemical function for protein sequences and structures resulting from genomic initiatives. Most sequences lack a characterized function and are annotated as hypothetical or uncharacterized. While homology-based methods are useful, and work well for sequences with sequence identities above 50%, they fail for sequences in the twilight zone (<30%) of sequence identity. For cases where sequence methods fail, structural approaches are often used, based on the premise that structure preserves function for longer evolutionary time-frames than sequence alone. It is now clear that no single method can be used successfully for functional inference. Given the growing need for functional assignments, we describe here a systematic new approach, designated ligand-centric, which is primarily based on analysis of ligand-bound/unbound structures in the PDB. Results of applying our approach to S-adenosyl-L-methionine (SAM) binding proteins are presented. Results: Our analysis included 1,224 structures that belong to 172 unique families of the Protein Information Resource Superfamily system. Our ligand-centric approach was divided into four levels: residue, protein/domain, ligand, and family levels. The residue level included the identification of conserved binding site residues based on structure-guided sequence alignments of representative members of a family, and the identification of conserved structural motifs. The protein/domain level included structural classification of proteins, Pfam domains, domain architectures,and protein topologies. The ligand level included ligand conformations, ribose sugar puckering, and the identification of conserved ligand-atom interactions. The family level included phylogenetic analysis. Conclusion: We found that SAM bound to a total of 18 different fold types (I-XVIII). We identified 4 new fold types and 11 additional topological arrangements of strands within the well-studied Rossmann fold Methyltransferases (MTases). This extends the existing structural classification of SAM binding proteins. A striking correlation between fold type and the conformation of the bound SAM (classified as types) was found across the 18 fold types. Several site-specific rules were created for the assignment of functional residues to families and proteins that do not have a bound SAM or a solved structure. |
  | Date Added | 10/28/2013, 4:53:08 PM |
  | Modified | 10/28/2013, 4:53:08 PM |

  ### Tags:

  - coverage

  ### Notes:

  - Present a method for protein function prediction and appliy to S-adenosyl-L-methionine (SAM) binding proetines.

    How SCOP is used:

    Annotate dataset with SCOP fold.

    Analysis includes 1,224 structures from 172 families taken from Protein Information Resource Superfamily system.

    SCOP reference:

    Here, we describe a systematic ligand-centric approach to protein annotation that is primarily based on ligand- bound structures from the Protein Data Bank (PDB). Our approach is multi-pronged, and is divided into four levels: residue, protein/domain, ligand, and family levels (Figure 1). Our analysis at the residue level includes the identification of conserved binding site residues based on structure-guided sequence alignments of representative members of a family and the identification of conserved structural motifs. **Our protein/domain level analysis in- cludes identification of Structural Classification of Proteins (SCOP) folds, Pfam domains, domain architecture, and protein topologies.** Our analysis of the ligand level in- cludes examination of ligand conformations, ribose sugar puckering (when applicable), and the identifica- tion of conserved ligand-atom interactions. Finally, our family level analysis includes phylogenetic analysis. Our approach can be used as a platform for function iden- tification, drug design, homology modeling, and other applications. We have applied our method to analyze 1,224 protein structures that are SAM binding proteins. Our results indicate that application of this ligand- centric approach allows making accurate protein func- tion predictions.

    ...

    Structural fold information

    Initial fold information was obtained primarily from SCOP [34]. For structures that did not have any SCOP information, the SUPERFAMILY database that is based on SCOP HMMs [35], was used for structural fold as- signment purposes. If no classification existed using either one of the databases, we assigned our own classifi- cations based on manual inspection and other functional attributes (Additional file 1: Table S1, column labeled SCOP fold).

    ...

    SCOP classifies all of the above topologies into the SAM-dependent MTase superfamily (Additional file 1: Table S1 column labeled SCOP folds). We suggest classifi- cation of the major arrangements into sub-classes, because these different arrangements may have functional con- sequences. Topological arrangements have previously been shown to be important for identifying the substrate specificities for these enzymes. For example, MTases with small molecules as substrates do not have any C- terminal additions, while MTases with protein substrates contain C-terminal additions [45].

    Several structures were not yet classified in SCOP, and in some cases, the SUPERFAMILY database was used, although for several structures, the SUPERFAMILY data- base yielded only weak hits to unrelated families. In these cases, the structures were manually inspected for classification. For example, the Core Protein VP4 (PDB-ID: 2JHP) had no significant hits at the time of this analysis, but manual inspection revealed that this protein belonged to fold type I and had an interesting topological arrange- ment comprised of both fold types Ia and Ib (Figure 3). This protein contained two SAM binding sites (one per domain). Topological arrangement 3 2 1 4 5 7 6 (fold type Ia) is inserted between β2 and β3 of the other SAM-binding domain that has the topology 6 7 5 4 1 2 3 (fold Ib). Results of topological analysis for the remainder fold types (II-XVIII) are provided in Additional file 2: Table S2 (column labeled Topology and Topological Class).

  ### Attachments

  - 1471-2164-13-S7-S21.pdf
  - 1472-6807-13-6.pdf
  - 1472-6807-13-6-s1.xlsx
  - 1472-6807-13-6-s3.pdf
- ## Structural and genomic DNA analysis of a putative transcription factor SCO5550 from Streptomyces coelicolor A3(2): Regulating the expression of gene sco5551 as a transcriptional activator with a novel dimer shape

  |  |  |
  | --- | --- |
  | Type | Journal Article |
  | Author | Takeshi Hayashi |
  | Author | Yoshikazu Tanaka |
  | Author | Naoki Sakai |
  | Author | Nobuhisa Watanabe |
  | Author | Tomohiro Tamura |
  | Author | Isao Tanaka |
  | Author | Min Yao |
  | Volume | 435 |
  | Issue | 1 |
  | Pages | 28–33 |
  | Publication | Biochemical and Biophysical Research Communications |
  | Date | May 2013 |
  | DOI | 10.1016/j.bbrc.2013.04.017 |
  | Abstract | SCO5550 from the model actinomycete Streptomyces coelicolor A3(2) was identified as a putative transcriptional regulator, and classified into the MerR family by sequence analysis. Recombined SCO5550 was successfully produced in Rhodococcus erythropolis, which can be used to stably express recombinant protein by optimizing the temperature over a wide range (4-35 degrees C). Crystal structure analysis showed that the dimerization domain (C-terminal domain) of SCO5550 has a novel fold and forms a new dimer shape, whereas the DNA-binding domain (N-terminal domain) is very similar to those of MerR family members. Such the new dimer form suggests that SCO5550 may define a new subfamily as a new member of the MerR family. Binding DNA sequence analysis of SCO5550 using the genomic systematic evolution of ligands by exponential enrichment (gSELEX) and electrophoretic mobility shift assay (EMSA) indicated that SCO5550 regulates the expression of the immediately upstream gene sco5551 encoding a putative protein, probably as a transcriptional activator. (C) 2013 Elsevier Inc. All rights reserved. |
  | Date Added | 3/7/2014, 1:06:24 PM |
  | Modified | 3/7/2014, 1:06:24 PM |
- ## Structural and mechanistic studies of the orf12 gene product from the clavulanic acid biosynthesis pathway

  |  |  |
  | --- | --- |
  | Type | Journal Article |
  | Author | Karin Valegard |
  | Author | Aman Iqbal |
  | Author | Nadia J. Kershaw |
  | Author | David Ivison |
  | Author | Catherine Genereux |
  | Author | Alain Dubus |
  | Author | Cecilia Blikstad |
  | Author | Marina Demetriades |
  | Author | Richard J. Hopkinson |
  | Author | Adrian J. Lloyd |
  | Author | David I. Roper |
  | Author | Christopher J. Schofield |
  | Author | Inger Andersson |
  | Author | Michael A. McDonough |
  | Volume | 69 |
  | Issue | 8 |
  | Pages | 1567-1579 |
  | Publication | Acta Crystallographica Section D: Biological Crystallography |
  | ISSN | 0907-4449 |
  | Date | August 2013 |
  | DOI | 10.1107/S0907444913011013 |
  | Language | English |
  | Abstract | Structural and biochemical studies of the orf12 gene product (ORF12) from the clavulanic acid (CA) biosynthesis gene cluster are described. Sequence and crystallographic analyses reveal two domains: a C-terminal penicillin-binding protein (PBP)/beta-lactamase-type fold with highest structural similarity to the class A beta-lactamases fused to an N-terminal domain with a fold similar to steroid isomerases and polyketide cyclases. The C-terminal domain of ORF12 did not show beta-lactamase or PBP activity for the substrates tested, but did show low-level esterase activity towards 3'-O-acetyl cephalosporins and a thioester substrate. Mutagenesis studies imply that Ser173, which is present in a conserved SXXK motif, acts as a nucleophile in catalysis, consistent with studies of related esterases, beta-lactamases and d-Ala carboxypeptidases. Structures of wild-type ORF12 and of catalytic residue variants were obtained in complex with and in the absence of clavulanic acid. The role of ORF12 in clavulanic acid biosynthesis is unknown, but it may be involved in the epimerization of (3S,5S)-clavaminic acid to (3R,5R)-clavulanic acid. |
  | Date Added | 10/11/2013, 10:29:15 AM |
  | Modified | 12/2/2013, 4:18:29 PM |

  ### Notes:

  - Structural and biochemical studies of the orf12 gene product (ORF12) from the clavulanic acid (CA) biosynthesis gene cluster are described.

    How SCOP is used:

    Use DALI to search for homologs in SCOP and locate the fold, superfamily, and family.  Describe the common functions of the 'structural family'.

    SCOP reference:

    3.2.3. Structural similarity to b-lactamases/D-Ala carboxy- peptidases and a family VIII esterase. The results from the DALI search for structural homologues of ORF12 imply that the C-terminal domain fold is related to the beta-lactamase/d-Ala carboxypeptidase-like fold, the beta-lactamase/d-Ala carboxy- peptidase superfamily and the beta-lactamase class A family as defined in the Structural Classification of Proteins (SCOP) database (Murzin et al., 1995; Holm et al., 2008). Many reported structures are available for this fold (824 hits using DALI). The members of this structural family have been shown to catalyse a variety of different reactions, including (but not limited to) beta-lactam hydrolysis (beta-lactamases), carboxypeptidation/transpeptidation (PBPs), thioester/ester hydrolysis (EstB) and deamidation (DAA) (Asano et al., 1989; Ghuysen, 1991; Petersen et al., 2001).

  ### Attachments

  - kw5065.pdf
- ## Structural bioinformatics of the human spliceosomal proteome

  |  |  |
  | --- | --- |
  | Type | Journal Article |
  | Author | Iga Korneta |
  | Author | Marcin Magnus |
  | Author | Janusz M. Bujnicki |
  | URL | http://nar.oxfordjournals.org/content/40/15/7046.short |
  | Volume | 40 |
  | Issue | 15 |
  | Pages | 7046–7065 |
  | Publication | Nucleic Acids Research |
  | Date | 2012 |
  | Accessed | 9/20/2013, 1:18:37 PM |
  | Library Catalog | Google Scholar |
  | Date Added | 10/11/2013, 10:20:13 AM |
  | Modified | 11/12/2013, 4:29:02 PM |

  ### Tags:

  - Interesting
  - SCOP coverage insufficient

  ### Notes:

  - "systematic structural bioinformatics analysis of the proteins of the human spliceosomal proteome".

    In particular, classified data set of 252 human splicing proteins by fold.  And built a non-redundant data set of spliceosome proteins with structures, using modeling where experimental data was not available.

    How SCOP is used:

    1. Where possible, retrieved domains for 252 spliceosome proteins from SCOP 1.75.  Note that they used parseable files.

    2. Classify data sets by SCOP class and superfamily.

    **Notes**

    -SCOP database used for structural identification of domains (v. 1.75)

    -Downloaded the parseable files

    -Noted the class the all domains (mostly from d)

    -Also noted the SCOP superfamily and description of each domain

    **QUOTES**

    **Under MATERIALS AND METHODS**

    Identification and description of structural  
    regions of proteins

    "SCOP database (49) IDs used for the purposed of structural  
    domain identification were either extracted from the  
    Protein Data Bank or from the SCOP parseable files on the  
    SCOP website (http://scop.mrc-lmb.cam.ac.uk/scop/parse/  
    index.html) or assigned using the fastSCOP server (http://  
    fastscop.life.nctu.edu.tw/) (50). "

    **Under RESULTS AND DISCUSSION**

    "Ordered domains of splicing proteins classified in the  
    SCOP (49) catalogue belong to classes a–e and g, with  
    an over-representation of class d, which contains superfamily d.58.7 (RNA-binding domain, RRM (RBD), which usually corresponds to PFAM domain PF00076, RRM\_1; Table 2)."

    "Table 2.  Statistics  of ordered  structural domains  of the human"  
    "spliceosome according  to  the SCOP  classiﬁcation"  
    "SCOP  ID               Description                                     Number  of domains"  
    "a"                        "All a"                                                        "79"  
    "b"                        "All b"                                                        "83"  
    "c"                        "a  and  b (a/b)"                                          "53"  
    "d"                        "a  and  b (a+b)"                                        "159"  
    "e"                        "Multi-domain (a  and  b)"                            "1"  
    "g"                        "Small"                                                        "49"

    Chart on 7060 noting the SCOP superfamily and description

    an over-representation of class d, which contains super- family d.58.7 (RNA-binding domain, RRM (RBD), which usually corresponds to PFAM domain PF00076, RRM\_1; Table 2). RRM is present in the 252 proteins in as many as 117 copies. This means that roughly each fourth to fifth domain in the spliceosomal proteome is an RRM. As RRM is a small domain that usually binds single-stranded RNA (63,64), this reflects the key charac- ter of protein–RNA interactions in the splicing process.

    Other common types of ordered protein regions found in the human spliceosomal proteome include other small RNA-binding domains, large a- and b-repeat-based protein-binding domains, small protein disorder-binding domains, ubiquitin-related domains and stable multidomain RNA helicase architectures (Table 3). Repeat-based domains are often found as building blocks of protein complexes, while some of the ubiquitin-related domains have been shown to be part of a putative ubiquitin-based system of controlling spliceosome assembly and dynamics (22,65).

    **Citation**

    49. Murzin,A.G., Brenner,S.E., Hubbard,T. and Chothia,C. (1995)  
    SCOP: a structural classification of proteins database for the  
    investigation of sequences and structures. J. Mol. Biol., 247,  
    536–540.

  ### Attachments

  - [HTML] from oxfordjournals.org
  - Nucl. Acids Res.-2012-Korneta-7046-65.pdf
  - Snapshot
- ## Structural Characterization of HP1264 Reveals a Novel Fold for the Flavin Mononucleotide Binding Protein

  |  |  |
  | --- | --- |
  | Type | Journal Article |
  | Author | Ki-Young Lee |
  | Author | Ji-Hun Kim |
  | Author | Kyu-Yeon Lee |
  | Author | Jiyun Lee |
  | Author | Ingyun Lee |
  | Author | Ye-Ji Bae |
  | Author | Bong-Jin Lee |
  | Volume | 52 |
  | Issue | 9 |
  | Pages | 1583-1593 |
  | Publication | Biochemistry |
  | ISSN | 0006-2960 |
  | Date | MAR 5 2013 |
  | Extra | WOS:000315844500008 |
  | DOI | 10.1021/bi301714a |
  | Abstract | Complex I (NADH-quinone oxidoreductase) is an enzyme that catalyzes the initial electron transfer from nicotinamide adenine dinucleotide (NADH) to flavin mononucleotide (FMN) bound at the tip of the hydrophilic domain of complex I. The electron flow into complex I is coupled to the generation of a proton gradient across the membrane that is essential for the synthesis of ATP. However, Helicobacter pylori has an unusual complex I that lacks typical NQO1 and NQO2 subunits, both of which are generally included in the NADH dehydrogenase domain of complex I. Here, we determined the solution structure of HP 1264, one of the unusual subunits of complex I from H. pylori, which is located in place of NQO2, by three-dimensional nuclear magnetic resonance (NMR) spectroscopy and revealed that HP1264 can bind to FMN through UV-visible, fluorescence, and NMR titration experiments. This result suggests that FMN-bound HP1264 could be involved in the initial electron transfer step of complex I. In addition, HP1264 is structurally most similar to Escherichia coli TusA, which belongs to the SirA-like superfamily having an IF3-like fold in the SCOP database, implying that HP1264 adopts a novel fold for FMN binding. On the basis of the NMR titration data, we propose the candidate residues Ile32, Met34, Leu58, Trp68, and Val71 of HP1264 for the interaction with FMN. Notably, these residues are not conserved in the FMN binding site of any other flavoproteins with known structure. This study of the relationship between the structure and FMN binding property of HP1264 will contribute to improving our understanding of flavoprotein structure and the electron transfer mechanism of complex I. |
  | Date Added | 2/20/2014, 12:24:01 PM |
  | Modified | 2/20/2014, 12:24:01 PM |

  ### Notes:

  - Experimental and computational study of HP1264, also known as complex I subunit E from H. pylori.

    How SCOP is used:

    Search for similar structures to get fold and superfamily classification.

    SCOP reference:

    In abstract:

    In addition, HP1264 is structurally  
     most similar to Escherichia coli TusA, which belongs to the SirA-like superfamily having an  
     IF3-like fold in the SCOP database, implying that HP1264 adopts a novel fold for FMN binding.

    ...

    The following servers were used for the sequence and structural analyses: BLAST (http://blast.ncbi.nlm.nih.gov/ Blast.cgi), DALI (http://ekhidna.biocenter.helsink.fi/dali- server), and SCOP (http://scop.mrc-lmb.cam.ac.uk/scop).

    ...

    HP1264 folds into a compact two-layer α/β-sandwich structure with a β1α1β2α2β3β4 topology, comprising a mixed four-stranded β-sheet stacked against two α-helices, both of which are nearly parallel to the strands of the β-sheet (Figure 1b). The β-strands correspond to residues 3−5 (β1), 28−35 (β2), 55−57 (β3), and 68−74 (β4), while the α-helices correspond to residues 20−24 (α1) and 40−50 (α2). The secondary structures of HP1264 are packed and stabilized by forming an interior extensive hydrophobic core with 11 hydrophobic residues. On the basis of the SCOP database,35 it is revealed that HP1264 adopts an IF3 (translation initiation factor 3)-like fold. In common, this fold has a core β1α1β2α2β3β4 topology with two layers and antiparallel strand 4.

    Structural Comparisons of HP1264. The past decade has been marked by outstanding advances in understanding the structure of bacterial complex I at an atomic level.16,18 In particular, the only determined structure of the hydrophilic domain of bacterial complex I was that from T. thermophilus. NQO2 is one of the eight subunits of this structure and might correspond to HP1264, which is also known as complex I subunit E from H. pylori. However, we found that HP1264 has a low degree of structural similarity to NQO2. In the SCOP database, NQO2 is divided into an N-terminal helical bundle and C-terminal thioredoxin fold. This fact supports the idea that HP1264 may play different structural and functional roles in the electron transfer mechanism of complex I compared to that of NQO2.

    Novel Site and Fold for FMN Binding. It has recently been reported that a variety of FMN binding protein folds are widely distributed throughout all kingdoms of life. Because HP1264 was thought to be the complex I subunit capable of FMN binding, the structure of HP1264 was preferentially compared with the previously determined structure of the NQO1 subunit of complex I from T. thermophilus,16 which can bind to FMN. Notably, the Rossmann fold-like domain of NQO1 has a frequently occurring motif that binds to nucleotides such as FMN, FAD, NADH, etc.45 **In the SCOP database, this domain of NQO1 is classifi****ed as a NQO1 FMN binding domain-like fold (Table 1 of the Supporting Information).** However, little structural similarity was observed between the IF3-like fold of HP1264 and the NQO1 FMN binding domain-like fold. Therefore, it is conceivable that HP1264 has structurally evolved in a different manner to have a new fold with the FMN binding function when compared to NQO1. The FMN binding of NQO1 is mostly achieved by a hydrogen bond network at the end of a solvent-exposed cavity.16 Figure 5b shows that various residues of NQO1 are involved in FMN binding at the cavity. FMN interacts mainly with the α3−α4 loop (Gly64 and Gly66), the β1−α5 loop (Asp94, Glu95, Ser96, and Glu97), α8 (Tyr180 and Gly183), and β4 (Ile218, Asn219, and Asn220), suggesting that the FMN binding mechanism of NQO1 is different from that of HP1264.

    I**n the SCOP database, FMN binding folds from** **H. pylori have recently been classified into a fl****avodoxin-like fold and chorismate synthase fold, respectively (Table 1 of the Supporting Information).** The flavodoxin-like fold from H. pylori can bind to the phosphoribityl tail of FMN by hydrogen bonds and to the isoalloxazine ring of FMN by hydrophobic interactions (Figure 5c).46 The phosphate group of FMN is bound by the loop motif (Thr-Asp-Ser-Gly-Asn-Ala), and the ribityl part of FMN is bound by the side chain atoms of Asn14 and Asp142. The isoalloxazine ring is bound by residues Tyr92 and Ala55 at the si-face and the re-face of the isoalloxazine ring, respectively. The chorismate synthase fold from H. pylori exhibits a highly positive electrostatic potential at the surface of the FMN binding pocket.47 Diverse regions, including α2 (His104), the α2−α3 loop (Arg123), α3 (Ser125), β11 (Asn241), β16 (Lys296), the β16−β17 loop (Thr298, Pro299, and Ser300), and α8 (Ile327 and Arg330), contribute to the formation of this FMN binding pocket (Figure 5d). This bioinformatics study revealed that the previously known FMN binding sites and folds from H. pylori were not conserved in HP1264.

    Furthermore, we found that the IF3-like fold and the proposed FMN binding site of HP1264 are not similar to those of any other FMN binding proteins described so far in the literature or in the bioinformatics database. **A systematic study of the SCOP database revealed that 14 protein folds in complex with FMN are structurally determined (Table 1 of the Supporting Information).** These folds show distinctive features according to the spatial arrangements of the secondary structure elements. The HP1264 fold presented here could be classified into the 15th protein fold for FMN binding. However, it remains unclear whether HP1264 and its neighboring subunits cooperatively form a FMN binding fold of complex I. Further study is necessary to determine the overall structure and exact FMN binding mode of complex I from H. pylori.

  ### Attachments

  - bi301714a.pdf
  - bi301714a\_si\_001.pdf
- ## Structural differences between soluble and membrane bound cytochrome P450s

  |  |  |
  | --- | --- |
  | Type | Journal Article |
  | Author | I. G. Denisov |
  | Author | A. Y. Shih |
  | Author | S. G. Sligar |
  | Volume | 108 |
  | Pages | 150-158 |
  | Publication | Journal of Inorganic Biochemistry |
  | ISSN | 0162-0134 |
  | Date | MAR 2012 |
  | Extra | WOS:000302205600022 |
  | DOI | 10.1016/j.jinorgbio.2011.11.026 |
  | Abstract | The superfamily of cytochrome P450s forms a large class of heme monooxygenases with more than 13,000 enzymes represented in organisms from all biological kingdoms. Despite impressive variability in sizes, sequences, location, and function, all cytochrome P450s from various organisms have very similar tertiary structures within the same fold. Here we show that systematic comparison of all available X-ray structures of cytochrome P450s reveals the presence of two distinct structural classes of cytochrome P450s. For all membrane bound enzymes, except the CYP51 family, the beta-domain and the A-propionate heme side chain are shifted towards the proximal side of the heme plane, which may result in an increase of the volume of the substrate binding pocket and an opening of a potential channel for the substrate access and/or product escape directly into the membrane. This structural feature is also observed in several soluble cytochrome P450s, such as CYP108, CYP151, and CYP158A2, which catalyze transformations of bulky substrates. Alternatively, both beta-domains and the A-propionate side chains in the soluble isozymes extend towards the distal site of the heme. This difference between the structures of soluble and membrane bound cytochrome P450s can be rationalized through the presence of several amino acid inserts in the latter class which are involved in direct interactions with the membrane, namely the F'- and G'-helices. Molecular dynamics using the most abundant human cytochrome P450, CYP3A4, incorporated into a model POPC bilayer reveals the facile conservation of a substrate access channel, directed into the membrane between the B-C loop and the beta domain, and the closure of the peripheral substrate access channel directed through the B-C loop. This is in contrast to the case when the same simulation is run in buffer, where no such channel closing occurs. Taken together, these results reveal a key structural difference between membrane bound and soluble cytochrome P450s with important functional implications induced by the lipid bilayer. (c) 2011 Elsevier Inc. All rights reserved. |
  | Date Added | 2/20/2014, 12:24:01 PM |
  | Modified | 2/20/2014, 12:24:01 PM |

  ### Notes:

  - Computational study of the Cytochrome P450s superfamily. Use structure analysis and molecular dynamics.

    How SCOP is used:

    Cite that all cytochrome P450s structures have the same SCOP fold.

    SCOP reference:

    However, despite these differ- ences, all known structures of cytochrome P450s from a variety of or- ganisms have essentially the same tertiary structure and belong to the same protein fold [3].

  ### Attachments

  - 1-s2.0-S0162013411003746-main.pdf
- ## Structural engineering of a phage lysin that targets Gram-negative pathogens

  |  |  |
  | --- | --- |
  | Type | Journal Article |
  | Author | Petra Lukacik |
  | Author | Travis J. Barnard |
  | Author | Paul W. Keller |
  | Author | Kaveri S. Chaturvedi |
  | Author | Nadir Seddiki |
  | Author | JamesW. Fairman |
  | Author | Nicholas Noinaj |
  | Author | Tara L. Kirby |
  | Author | Jeffrey P. Henderson |
  | Author | Alasdair C. Steven |
  | Author | B. Joseph Hinnebusch |
  | Author | Susan K. Buchanan |
  | Volume | 109 |
  | Issue | 25 |
  | Pages | 9857–9862 |
  | Publication | Proceedings of the National Academy of Sciences of the United States of America |
  | Date | June 2012 |
  | DOI | 10.1073/pnas.1203472109 |
  | Abstract | Bacterial pathogens are becoming increasingly resistant to antibiotics. As an alternative therapeutic strategy, phage therapy reagents containing purified viral lysins have been developed against Gram-positive organisms but not against Gram-negative organisms due to the inability of these types of drugs to cross the bacterial outer membrane. We solved the crystal structures of a Yersinia pestis outer membrane transporter called FyuA and a bacterial toxin called pesticin that targets this transporter. FyuA is a beta-barrel membrane protein belonging to the family of TonB dependent transporters, whereas pesticin is a soluble protein with two domains, one that binds to FyuA and another that is structurally similar to phage T4 lysozyme. The structure of pesticin allowed us to design a phage therapy reagent comprised of the FyuA binding domain of pesticin fused to the N-terminus of T4 lysozyme. This hybrid toxin kills specific Yersinia and pathogenic E. coli strains and, importantly, can evade the pesticin immunity protein (Pim) giving it a distinct advantage over pesticin. Furthermore, because FyuA is required for virulence and is more common in pathogenic bacteria, the hybrid toxin also has the advantage of targeting primarily disease-causing bacteria rather than indiscriminately eliminating natural gut flora. |
  | Date Added | 3/7/2014, 1:06:24 PM |
  | Modified | 3/7/2014, 1:06:24 PM |
- ## Structural features that predict real-value fluctuations of globular proteins

  |  |  |
  | --- | --- |
  | Type | Journal Article |
  | Author | Michal Jamroz |
  | Author | Andrzej Kolinski |
  | Author | Daisuke Kihara |
  | Volume | 80 |
  | Issue | 5 |
  | Pages | 1425-1435 |
  | Publication | Proteins-Structure Function and Bioinformatics |
  | ISSN | 0887-3585 |
  | Date | MAR 2012 |
  | Extra | WOS:000302541900015 |
  | DOI | 10.1002/prot.24040 |
  | Abstract | It is crucial to consider dynamics for understanding the biological function of proteins. We used a large number of molecular dynamics (MD) trajectories of nonhomologous proteins as references and examined static structural features of proteins that are most relevant to fluctuations. We examined correlation of individual structural features with fluctuations and further investigated effective combinations of features for predicting the real value of residue fluctuations using the support vector regression (SVR). It was found that some structural features have higher correlation than crystallographic B-factors with fluctuations observed in MD trajectories. Moreover, SVR that uses combinations of static structural features showed accurate prediction of fluctuations with an average Pearson's correlation coefficient of 0.669 and a root mean square error of 1.04 angstrom. This correlation coefficient is higher than the one observed in predictions by the Gaussian network model (GNM). An advantage of the developed method over the GNMs is that the former predicts the real value of fluctuation. The results help improve our understanding of relationships between protein structure and fluctuation. Furthermore, the developed method provides a convienient practial way to predict fluctuations of proteins using easily computed static structural features of proteins. Proteins 2012; (c) 2012 Wiley Periodicals, Inc. |
  | Date Added | 2/13/2014, 4:13:17 PM |
  | Modified | 3/7/2014, 12:14:39 PM |
- ## Structural, functional and molecular docking study to characterize GMI1 from Arabidopsis thaliana

  |  |  |
  | --- | --- |
  | Type | Journal Article |
  | Author | Md Rezaul Islam |
  | Author | Md Ismail Hosen |
  | Author | Aubhishek Zaman |
  | Author | Md Ohedul Islam |
  | URL | http://link.springer.com/article/10.1007/s12539-013-0153-1 |
  | Volume | 5 |
  | Issue | 1 |
  | Pages | 13–22 |
  | Publication | Interdisciplinary Sciences: Computational Life Sciences |
  | Date | 2013 |
  | Accessed | 9/23/2013, 10:21:55 AM |
  | Library Catalog | Google Scholar |
  | Date Added | 10/11/2013, 10:29:15 AM |
  | Modified | 3/7/2014, 1:06:46 PM |

  ### Tags:

  - Arabidopsis thaliana
  - docking
  - GMI1
  - in silico modeling

  ### Notes:

  - I couldn't get access to the paper.

  ### Attachments

  - Snapshot

    ## Abstract

    *γ*-irradiation and Mitomycin C Induced 1 (GMI1), is a member of the SMC-hinge domain-containing protein family that takes part in double stranded break repair mechanism in eukaryotic cells. In this study we hypothesize a small molecule-Adenosine Tri Phosphate (ATP) binding region of novel SMC like GM1 protein in model organism Arabidopsis *thaliana* using *in silico* modeling. Initially, analyzing sequence information for the protein indicated presence of motifs — ‘Walker A nucleotide-binding domain’ that are required to interact with nucleotides along with ‘Walker B’ motif and ABC signature sequences. This was further proven through GMI1-ATP docking experiment and results were verified by comparing the values with controls. In negative control, no binding was seen in the same binding region of GMI1 structure for small molecules randomly selected form PubChem database, whereas in positive control binding affinity of other known proteins with ATP binding potential resembled GMI1-ATP binding affinity of −5.4 kcal/mol. Furthermore we also docked small molecules that shares structural similarity with ATP to GMI1 and found that Purine Mononucleotide bound the region with the best affinity, which implies that the compound may bind the protein with strong binding and can work as a potential agonist/antagonist to GMI1. We believe that the study would shed more light into the GM1 mechanism of action. Although the computational predictions made here are based on concrete confidence, it should be mentioned that *in vitro* experimentation does not fall into the scopes of this study and thus the results found here have to be further validated *in vitro*.
- ## Structural modelling and dynamics of proteins for insights into drug interactions

  |  |  |
  | --- | --- |
  | Type | Journal Article |
  | Author | Tim Werner |
  | Author | Michael B. Morris |
  | Author | Siavoush Dastmalchi |
  | Author | W. Bret Church |
  | URL | http://www.sciencedirect.com/science/article/pii/S0169409X11002912 |
  | Volume | 64 |
  | Issue | 4 |
  | Pages | 323–343 |
  | Publication | Advanced drug delivery reviews |
  | Date | 2012 |
  | Accessed | 9/20/2013, 1:16:59 PM |
  | Library Catalog | Google Scholar |
  | Date Added | 2/20/2014, 12:24:01 PM |
  | Modified | 2/20/2014, 12:24:01 PM |

  ### Notes:

  - Review of the application of structural modelling and dynamics to the study of drug interactions.

    How SCOP is used:

    1. To show that fold space is more limited than sequence space.  Mention number of folds, superfamilies, and families in SCOP 1.75.

    2. Describe a previous study where small-domain sequences (<150 residues) that were non-homologous to any others in SCOP were first modeled with Rosetta, then classified into SCOP superfamilies using structure and function information.  Found they could classify only about 1/5th of the sequences with this method (with whatever criteria they deemed as high-confidence), implying that the success of de novo prediction methods was limited.

    SCOP reference:

    By their nature, threading methods are limited to a search of known folds and are unable to correctly predict the structure of the target if, in reality, it adopts a novel fold. That said, there appears to be a limited number of folds: estimations of protein folds for water-soluble proteins vary from 400 to 10,000 [6–11], with 2700 folds being a relatively recent estimate [11] derived by analysing the Structural Classification of Proteins (SCOP) [12,13] database. The current SCOP release 1.75 (June 2009) contains 1195 different folds belonging to 1962 superfamilies and 3902 families.

    ...

    In a 2007 study [101], small protein domains (b150 residues), which were not homologous to known structures, were selected from the yeast genome. 3D structures of these 3338 domains were predicted using ROSETTA and then classified into the SCOP superfam- ilies by a structure-based comparison of the predicted models with the SCOP structures, and also by integration of Gene Ontology (GO) data [102]. Out of the 3338 models, 404 could be assigned using only structure-comparison methods and a further 177 were success- fully assigned after integrating GO data. The results show that the de novo prediction methods might be useful for special problems but one cannot expect a high success rate.

  ### Attachments

  - 1-s2.0-S0169409X11002912-main.pdf
  - Snapshot
- ## Structural modelling and mutant cycle analysis predict pharmacoresponsiveness of a Na(V)1.7 mutant channel

  |  |  |
  | --- | --- |
  | Type | Journal Article |
  | Author | Yang Yang |
  | Author | Sulayman D Dib-Hajj |
  | Author | Jian Zhang |
  | Author | Yang Zhang |
  | Author | Lynda Tyrrell |
  | Author | Mark Estacion |
  | Author | Stephen G Waxman |
  | Volume | 3 |
  | Pages | 1186 |
  | Publication | Nature communications |
  | ISSN | 2041-1723 |
  | Date | 2012 |
  | Extra | PMID: 23149731 |
  | Journal Abbr | Nat Commun |
  | DOI | 10.1038/ncomms2184 |
  | Library Catalog | NCBI PubMed |
  | Language | eng |
  | Abstract | Sodium channel Na(V)1.7 is critical for human pain signalling. Gain-of-function mutations produce pain syndromes including inherited erythromelalgia, which is usually resistant to pharmacotherapy, but carbamazepine normalizes activation of Na(V)1.7-V400M mutant channels from a family with carbamazepine-responsive inherited erythromelalgia. Here we show that structural modelling and thermodynamic analysis predict pharmacoresponsiveness of another mutant channel (S241T) that is located 159 amino acids distant from V400M. Structural modelling reveals that Na(v)1.7-S241T is ~2.4 Å apart from V400M in the folded channel, and thermodynamic analysis demonstrates energetic coupling of V400M and S241T during activation. Atomic proximity and energetic coupling are paralleled by pharmacological coupling, as carbamazepine (30 μM) depolarizes S214T activation, as previously reported for V400M. Pharmacoresponsiveness of S241T to carbamazepine was further evident at a cellular level, where carbamazepine normalized the hyperexcitability of dorsal root ganglion neurons expressing S241T. We suggest that this approach might identify variants that confer enhanced pharmacoresponsiveness on a variety of channels. |
  | Date Added | 10/11/2013, 10:29:15 AM |
  | Modified | 10/11/2013, 10:29:15 AM |

  ### Tags:

  - Action Potentials
  - Animals
  - Carbamazepine
  - Dimethyl Sulfoxide
  - Ganglia, Spinal
  - HEK293 Cells
  - Humans
  - Ion Channel Gating
  - Models, Molecular
  - Mutant Proteins
  - Mutation
  - NAV1.7 Voltage-Gated Sodium Channel
  - Neurons
  - Protein Structure, Tertiary
  - Rats
  - Rats, Sprague-Dawley
  - Thermodynamics

  ### Notes:

  - Use homology modeling to attain a structure for Nav1.7 channel, and the analyze the properties.

    NaV1.7 is a neuronal protein and a central player in pain transduction.  Various mutations are associated with indifference to pain or several painful syndromes.  Recent findings suggest the possibility of personalized pharmacotherapy based on a pharmacogenomics approach.

    How SCOP is used:

    Not using SCOP data.  Mention SCOP in the context of homology modeling, giving background on values of TM-scores for proteins with similar structure.

    SCOP reference:

    A model with TM-score >0.5 indicates a high similarity between the predicted model and the native structure18, 19.

  ### Attachments

  - nihms-418252.pdf
  - PubMed entry
- ## Structural Modelling Pipelines in Next Generation Sequencing Projects

  |  |  |
  | --- | --- |
  | Type | Journal Article |
  | Author | Jonathan G. L. Mullins |
  | Editor | M. I. Rees |
  | Volume | 89 |
  | Pages | 117-167 |
  | Publication | Challenges and Opportunities of Next-Generation Sequencing for Biomedical Research |
  | Date | 2012 |
  | Extra | WOS:000314729700006 |
  | Abstract | Our capacity to reliably predict protein structure from sequence is steadily improving due to the increased numbers and better targeting of protein structures being experimentally determined by structural genomics projects, along with the development of better modeling methodologies. Template-based (homology) modeling and de novo modeling methods are being combined to fill in remaining gaps in template coverage, and powerful automated structural modeling pipelines are being applied to large data sets of protein sequences. The improved quality of 3D models of proteins has led to their routine use in assessing the functional impact of nonsynonymous single nucleotide polymorphisms (nsSNPs) in specific protein systems, with the development of approaches that may be applied in a predictive fashion to nsSNPs emerging from next-generation sequencing projects. The challenges encountered in deriving functionally meaningful deductions from structural modeling can be quite different for proteins of different protein functional classes. The specific challenges to the assessment of the structural and functional impact of nsSNPs in globular proteins such as binding and regulatory proteins, structural proteins, and enzymes are discussed, as well as membrane transport proteins and ion channels. The mapping of reliable predictions of the structural and functional impact of SNPs, generated from automated modeling pipelines, on to protein-protein interaction networks will facilitate new approaches to understanding complex polygenic disorders and predisposition to disease. |
  | Date Added | 2/13/2014, 4:13:17 PM |
  | Modified | 3/7/2014, 1:07:55 PM |

  ### Notes:

  - Paper unavailable.
- ## Structural patterns in globular proteins

  |  |  |
  | --- | --- |
  | Type | Journal Article |
  | Author | M. Levitt |
  | Author | C. Chothia |
  | Volume | 261 |
  | Issue | 5561 |
  | Pages | 552-558 |
  | Publication | Nature |
  | ISSN | 0028-0836 |
  | Date | Jun 17, 1976 |
  | Extra | PMID: 934293 |
  | Journal Abbr | Nature |
  | Library Catalog | NCBI PubMed |
  | Language | eng |
  | Abstract | A simple diagrammatic representation has been used to show the arrangement of alpha helices and beta sheets in 31 globular proteins, which are classified into four clearly separated classes. The observed arrangements are significantly non-random in that pieces of secondary structure adjacent in sequence along the polypeptide chain are also often in contact in three dimensions. |
  | Date Added | 11/3/2014, 2:50:22 PM |
  | Modified | 11/3/2014, 2:50:22 PM |

  ### Tags:

  - Models, Structural
  - Protein Conformation

  ### Attachments

  - PubMed entry
- ## Structural Phylogenomics Retrodicts the Origin of the Genetic Code and Uncovers the Evolutionary Impact of Protein Flexibility

  |  |  |
  | --- | --- |
  | Type | Journal Article |
  | Author | Gustavo Caetano-Anollés |
  | Author | Minglei Wang |
  | Author | Derek Caetano-Anollés |
  | URL | http://dx.plos.org/10.1371/journal.pone.0072225 |
  | Volume | 8 |
  | Issue | 8 |
  | Pages | e72225 |
  | Publication | PloS one |
  | Date | 2013 |
  | Accessed | 9/20/2013, 1:19:56 PM |
  | Library Catalog | Google Scholar |
  | Date Added | 2/20/2014, 12:24:01 PM |
  | Modified | 2/20/2014, 12:24:01 PM |

  ### Notes:

  - Computational study of evolution of "aminoacyl-tRNA synthetase enzymes".

    How SCOP is used:

    Build a phylogenomic tree using SCOP family information.

    SCOP reference:

    Structural Phylogenomic Analysis

    In this study we mapped the evolution of aaRS domains in a published evolutionary timeline of domain appearance at fold family (FF) level of structural abstraction [6,7]. This timeline was selected for a number of reasons: FFs generally provide structures with unambiguous assignments of molecular functions, the timeline is well annotated, and results can be benchmarked to a description of the rise of early structures and functions [7]. The timeline was derived from a phylogenomic tree of 2,397 FF structures (out of 3,464 defined by the STRUCTURAL CLASSIFICATION

    OF PROTEINS (SCOP) 1.73; [8]) reconstructed from a structural census in the genomes of 420 free-living organisms from all three cellular superkingdoms (FL420). The timeline was for all purposes congruent to a timeline derived from a phylogenomic tree of 3,513 FFs (out of 3,902 defined by SCOP 1.75) reconstructed from a census of 989 genomes (A989) [14].

  ### Attachments

  - [HTML] from plos.org
  - journal.pone.0072225.pdf
- ## Structural phylogenomics reveals gradual evolutionary replacement of abiotic chemistries by protein enzymes in purine metabolism

  |  |  |
  | --- | --- |
  | Type | Journal Article |
  | Author | Kelsey Caetano-Anollés |
  | Author | Gustavo Caetano-Anollés |
  | URL | http://dx.plos.org/10.1371/journal.pone.0059300 |
  | Volume | 8 |
  | Issue | 3 |
  | Pages | e59300 |
  | Publication | PloS one |
  | Date | 2013 |
  | Accessed | 9/20/2013, 1:20:11 PM |
  | Library Catalog | Google Scholar |
  | Date Added | 10/11/2013, 10:29:15 AM |
  | Modified | 3/7/2014, 12:10:11 PM |

  ### Notes:

  - Study the emergence of purine metabolism.  Determine the ages of protein domains from fold families.

    How SCOP is used:

    Collect domains and families and create phylogenetic tree.

    How CATH is used:

    Background on protein structure classification.

    SCOP reference:

    The relative age of domains with molecular structures defined according to the STRUCTURAL CLASSIFICATION OF PROTEINS (SCOP) [6] are first obtained from phylogenetic trees, explicit statements of domain history built from a census of protein domain structure in the proteomes of hundreds to thousands of organisms that have been sequenced [2,3,7–10]. The age of domains is then mapped onto the enzymes and associated enzymatic functions that delimit network structure in illustrations of each and every subnetwork of metabolism [4]

    ...

    Taxonomies such as SCOP [6] and CATH [12] use protein domain building blocks as units of classification [13]. In SCOP, domains that are evolutionarily closely related at the sequence level are clustered into fold families (FFs). Domains belonging to different families that exhibit low sequence identities but that share structural and functional features suggesting a common origin are further unified into fold superfamilies (FSFs). Finally, FSFs sharing secondary structures that are similarly arranged and topologically connected are unified into protein folds. These folds sometimes have peripheral regions of secondary structure that add peripheral structural complexity to the central core fold architecture [14].

    ...

    MANET 1.0 and 2.0 use domains structures defined at fold level. This is problematic. Folds are ambiguously associated with molecular functions and cannot dissect recruitment patterns without additional information [1,5]. In contrast, FFs are generally unambiguously linked to molecular functions and are more powerful in their ability to uncover the history of early biochemistry [17]. This power has been made evident in the study of the most ancient proteins [18], the rise of translation [19], the protein repertoire of the last universal common ancestor [9], the first amino acid biosynthetic pathways and the origin of aerobic metabolism and planet oxygenation [20,21]. We therefore assigned ages to the FF domains of the purine metabolic subnetwork using phylogenomic trees reconstructed from an analysis of domain abundance in the proteomes of 989 organisms that have been completely sequenced (A989) [20] and a subset of 420 that are free-living (FL420) [19]. The rooted trees describe the evolution of 3,513 FFs (out of the 3,902 defined by SCOP 1.75).

  ### Attachments

  - [HTML] from plos.org
  - journal.pone.0059300.pdf
- ## Structural phylogenomics uncovers the early and concurrent origins of cysteine biosynthesis and iron-sulfur proteins

  |  |  |
  | --- | --- |
  | Type | Journal Article |
  | Author | Hong-Yu Zhang |
  | Author | Tao Qin |
  | Author | Ying-Ying Jiang |
  | Author | Gustavo Caetano-Anollés |
  | URL | http://www.tandfonline.com/doi/abs/10.1080/07391102.2012.687520 |
  | Volume | 30 |
  | Issue | 5 |
  | Pages | 542–545 |
  | Publication | Journal of Biomolecular Structure and Dynamics |
  | Date | 2012 |
  | Accessed | 9/23/2013, 10:20:00 AM |
  | Library Catalog | Google Scholar |
  | Date Added | 10/11/2013, 10:29:15 AM |
  | Modified | 10/11/2013, 10:29:15 AM |

  ### Tags:

  - cysteine biosynthesis
  - evolution
  - iron-sulfur proteins
  - molecular clock
  - protein structure

  ### Notes:

  - Computational study using a phylogenomic tree built with SCOP data "to explore the origins and evolution of Cys biosynthesis and possible evolution- ary association to metallochemistry".

    How SCOP is used:

    Use SCOP to build a phylogenomic tree to study the emergence of different families.

    SCOP reference:

    Standard molecular clocks based on rates of change in protein and/or nucleic acid sequences are widely used for establishing evolutionary timescales (Kumar, 2005). However, the use of these conventional tools of evolu- tionary biology is restricted by the time span of the pro- teins that are studied. Recently, we established timescales of protein domains by embarking on a systematic evolu- tionary analysis of their structures (Wang et al., 2011). These timescales are embodied in phylogenies that describe the evolution of domain structures and encom- pass the entire history of the protein world, from its putative origin 3.8 billion years ago (giga-annum, Ga) to the present (Caetano-Anollés & Caetano-Anollés, 2003). In these studies, we use Hidden Markov Models of struc- tural recognition to survey domain structures defined at fold and fold superfamily (FSF) levels of the Structural Classification of Proteins (Murzin, Brenner, Hubbard, & Chothia, 1995) in hundreds of organisms belonging to the three superkingdoms of life. Folds group FSF domains that have similar arrangements of secondary structures in three-dimensional space but that may not be evolutionarily related. In turn, FSFs unify fold family (FF) domains with sequences that share a common evo- lutionary origin and similar functions (Murzin et al., 1995). To establish timescales, we then reconstruct phy- logenomic trees of folds and FSFs using a cladistic method widely used in morphometrics (Caetano-Anollés, Wang, Caetano-Anollés, & Mittenthal, 2009). Since these trees are highly unbalanced, the relative age of domain structures can be established by counting the number of molecular speciation events (splits or nodes) along lin- eages of the trees. The ages of domains were thus char- acterized by node distances for folds (ndF) and FSFs (ndFSF) along branches of the trees (from root to leaf) on a relative 0–1 scale and were used to build timelines of domain discovery that scaled proportional to geological age (Wang et al., 2011). The resulting molecular clocks have been used to trace important and very ancient evolu- tionary events, including the rise of oxygen in our planet, the evolutionary history of metabolism, and the origins of organismal diversification (Kim et al., 2012; Wang et al., 2011). Here we use these methods to explore the origins and evolution of Cys biosynthesis and possible evolution- ary association to metallochemistry. Since cysteinyl sulfur is crucially linked to iron-sulfur (Fe-S) clusters in metal- loenzyme active sites (Meyer, 2008), we use timelines of domain discovery to explore the origin and evolution of Fe-S proteins. These proteins are widely distributed in life and harbor a multitude of molecular functions, including crucial participation in nitrogen fixation, cata- lytic generation of radicals, oxidation–reduction reactions, mitochondrial electron transport, and sulfur donation in cofactor and lipid metabolic networks.

  ### Attachments

  - 07391102%2E2012%2E687520.pdf
- ## Structural Propensities of Human Ubiquitination Sites: Accessibility, Centrality and Local Conformation

  |  |  |
  | --- | --- |
  | Type | Journal Article |
  | Author | Yuan Zhou |
  | Author | Sixue Liu |
  | Author | Jiangning Song |
  | Author | Ziding Zhang |
  | Volume | 8 |
  | Issue | 12 |
  | Pages | UNSP e83167 |
  | Publication | Plos One |
  | ISSN | 1932-6203 |
  | Date | DEC 11 2013 |
  | Extra | WOS:000328730300135 |
  | DOI | 10.1371/journal.pone.0083167 |
  | Abstract | The existence and function of most proteins in the human proteome are regulated by the ubiquitination process. To date, tens of thousands human ubiquitination sites have been identified from high-throughput proteomic studies. However, the mechanism of ubiquitination site selection remains elusive because of the complicated sequence pattern flanking the ubiquitination sites. In this study, we perform a systematic analysis of 1,330 ubiquitination sites in 505 protein structures and quantify the significantly high accessibility and unexpectedly high centrality of human ubiquitination sites. Further analysis suggests that the higher centrality of ubiquitination sites is associated with the multi-functionality of ubiquitination sites, among which protein-protein interaction sites are common targets of ubiquitination. Moreover, we demonstrate that ubiquitination sites are flanked by residues with non-random local conformation. Finally, we provide quantitative and unambiguous evidence that most of the structural propensities contain specific information about ubiquitination site selection that is not represented by the sequence pattern. Therefore, the hypothesis about the structural level of the ubiquitination site selection mechanism has been substantially approved. |
  | Date Added | 2/12/2014, 1:36:22 PM |
  | Modified | 2/12/2014, 1:36:22 PM |

  ### Notes:

  - Computational study of structural geometries of ubiquitination sites.

    How SCOP is used:

    Get summary statistics on data set of 505 PDB structures on number of folds and familiies.

    SCOP reference:

    As a result, 1,330 Ubsites and 5,465 Non-Ubsites were mapped onto the 505 PDB structures (Table S1), which cover 151 folds and 229 families according to the latest SCOP [28] annotations.

  ### Attachments

  - journal.pone.0083167.pdf
- ## Structural SCOP Superfamily Level Classification Using Unsupervised Machine Learning

  |  |  |
  | --- | --- |
  | Type | Journal Article |
  | Author | Ulavappa B. Angadi |
  | Author | M. Venkatesulu |
  | URL | http://dl.acm.org/citation.cfm?id=2122456 |
  | Volume | 9 |
  | Issue | 2 |
  | Pages | 601–608 |
  | Publication | IEEE/ACM Transactions on Computational Biology and Bioinformatics (TCBB) |
  | Date | 2012 |
  | Accessed | 2/28/2013, 1:38:04 PM |
  | Library Catalog | Google Scholar |
  | Date Added | 10/11/2013, 10:29:15 AM |
  | Modified | 3/7/2014, 1:07:15 PM |

  ### Tags:

  - ART2 neural network
  - protein classification
  - SCOP
  - unsupervised learning

  ### Notes:

  - Applies some machine learning techniques on BLAST data to automatically classify proteins into superfamilies.  Evaluates against other published methods for doing this.

    How SCOP is used:

    Benchmarked method for superfamily classification.

    Used ASTRAL representative set for dataset.

    How CATH is used:

    Not using CATH data.

    SCOP reference:

    3 DATA SET

    To evaluate the performance of our approach, we used three different sets of data from nonredundant (less than 40 percent sequence identity) ASTRAL [24] SCOP 1.75 (http://astral.berkeley.edu/). For the first data set, we selected the data set of 507 sequences used by Paccanaro et al. [8] to classify the protein domains into SCOP superfamily using spectral clustering. This data set was found to have very low similarity measures between the sequences within the superfamilies. In the data set containing 507 sequences, the domains were found to belong to six superfamilies, namely, Globin-like (88), EF- hand (83), Cupredoxins (78), (Trans) glycosidases (83), Thioredoxin-like (81), and Membrane all-alpha (94). For the second data set, we selected all domains (1,170) of eight SCOP 1.75 superfamilies: a.4.1 (102), .a.4.5 (181), b.1.1 (139), b.40.4 (101), c.2.1 (195), c.37.1 (239), c.47.1 (110), and c.66.1 (103). The third data set comprised all domains (1,375) of 14 superfamilies: a.4.5 (181), b.40.4 (101), c.2.1 (195), c.37.1 (239), c.47.1 (110), c.66.1 (103), a.39.1 (51), b.1.18 (76), b.18.1 (56), b.29.1 (57), c.108.1 (56), c.52.1 (39), c.55.1 (68), and c.55.3 (43).

     SCOP/CATH reference:

    Several classification databases such as SCOP [1], CATH [2], and Dali Domain Dictionary [3] have been developed based on 3D structural information to build protein domains in a hierarchical manner and to reflect structural, functional, and evolu- tionary relatedness.

  ### Attachments

  - ttb2012020601.pdf

    One of the major research directions in bioinformatics is that of assigning superfamily classification to a given set of  
    proteins. The classification reflects the structural, evolutionary, and functional relatedness. These relationships are embodied in a  
    hierarchical classification, such as the Structural Classification of Protein (SCOP), which is mostly manually curated. Such a  
    classification is essential for the structural and functional analyses of proteins. Yet a large number of proteins remain unclassified. In  
    this study, we have proposed an unsupervised machine learning approach to classify and assign a given set of proteins to SCOP  
    superfamilies. In the method, we have constructed a database and similarity matrix using P-values obtained from an all-against-all  
    BLAST run and trained the network with the ART2 unsupervised learning algorithm using the rows of the similarity matrix as input  
    vectors, enabling the trained network to classify the proteins from 0.82 to 0.97 f-measure accuracy. The performance of ART2 has  
    been compared with that of spectral clustering, Random forest, SVM, and HHpred. ART2 performs better than the others except  
    HHpred. HHpred performs better than ART2 and the sum of errors is smaller than that of the other methods evaluated.
- ## Structural studies of large nucleoprotein particles, vaults

  |  |  |
  | --- | --- |
  | Type | Journal Article |
  | Author | Hideaki Tanaka |
  | Author | Tomitake Tsukihara |
  | Volume | 88 |
  | Issue | 8 |
  | Pages | 416–433 |
  | Publication | Proceedings of the Japan Academy Series B-physical and Biological Sciences |
  | Date | October 2012 |
  | DOI | 10.2183/pjab.88.416 |
  | Abstract | Vault is the largest nonicosahedral cytosolic nucleoprotein particle ever described. The widespread presence and evolutionary conservation of vaults suggest important biologic roles, although their functions have not been fully elucidated. X-ray structure of vault from rat liver was determined at 3.5 angstrom resolution. It exhibits an ovoid shape with a size of 40 x 40 x 67 nm(3). The cage structure of vault consists of a dimer of half-vaults, with each half-vault comprising 39 identical major vault protein (MVP) chains. Each MVP monomer folds into 12 domains: nine structural repeat domains, a shoulder domain, a cap-helix domain and a cap-ring domain. Interactions between the 42-turn-long cap-helix domains are key to stabilizing the particle. The other components of vaults, telomerase-associated proteins, poly(ADP-ribose) polymerases and small RNAs, are in location in the vault particle by electron microscopy. |
  | Date Added | 3/7/2014, 1:06:24 PM |
  | Modified | 3/7/2014, 1:06:24 PM |
- ## Structural updates of alignment of protein domains and consequences on evolutionary models of domain superfamilies

  |  |  |
  | --- | --- |
  | Type | Journal Article |
  | Author | Eshita Mutt |
  | Author | Sudha Sane Rani |
  | Author | Ramanathan Sowdhamini |
  | Volume | 6 |
  | Pages | 20 |
  | Publication | Biodata Mining |
  | ISSN | 1756-0381 |
  | Date | NOV 15 2013 |
  | Extra | WOS:000328955700001 |
  | DOI | 10.1186/1756-0381-6-20 |
  | Abstract | Background: Influx of newly determined crystal structures into primary structural databases is increasing at a rapid pace. This leads to updation of primary and their dependent secondary databases which makes large scale analysis of structures even more challenging. Hence, it becomes essential to compare and appreciate replacement of data and inclusion of new data that is critical between two updates. PASS2 is a database that retains structure-based sequence alignments of protein domain superfamilies and relies on SCOP database for its hierarchy and definition of superfamily members. Since, accurate alignments of distantly related proteins are useful evolutionary models for depicting variations within protein superfamilies, this study aims to trace the changes in data in between PASS2 updates. Results: In this study, differences in superfamily compositions, family constituents and length variations between different versions of PASS2 have been tracked. Studying length variations in protein domains, which have been introduced by indels (insertions/deletions), are important because theses indels act as evolutionary signatures in introducing variations in substrate specificity, domain interactions and sometimes even regulating protein stability. With this objective of classifying the nature and source of variations in the superfamilies during transitions (between the different versions of PASS2), increasing length-rigidity of the superfamilies in the recent version is observed. In order to study such length-variant superfamilies in detail, an improved classification approach is also presented, which divides the superfamilies into distinct groups based on their extent of length variation. Conclusions: An objective study in terms of transition between the database updates, detailed investigation of the new/old members and examination of their structural alignments is non-trivial and will help researchers in designing experiments on specific superfamilies, in various modelling studies, in linking representative superfamily members to rapidly expanding sequence space and in evaluating the effects of length variations of new members in drug target proteins. The improved objective classification scheme developed here would be useful in future for automatic analysis of length variation in cases of updates of databases or even within different secondary databases. |
  | Date Added | 2/12/2014, 2:18:08 PM |
  | Modified | 3/7/2014, 12:09:08 PM |

  ### Notes:

  - Study the changes between releases in the PASS2 database, which provides multiple structure alignnments for each SCOP superfamily.  Mainly, look at the differences in lengths within the superfamily.

    Note: Not sure why they didn't just study SCOP directly,

    How SCOP is used:

    Computational study of all SCOP domains classified at the superfamily level.

    How CATH is used:

    Background on protein structure classification.

    SCOP reference:

    In abstract:

    PASS2 is a database that retains structure-based sequence alignments of protein domain superfamilies and relies on SCOP database for its hierarchy and definition of superfamily members.

     SCOP/CATH reference:

    Protein domains are one of the fundamental building blocks of protein structures and have been used as a unit for structural classification of proteins. Protein domains in primary structural databases such as PDB (Protein Data Bank) [1] have been grouped according to structural hierarchy such as protein folds, superfamilies and families in databases like CATH (Class, Architecture, Topology, Homologous superfamily) [2] and SCOP (Structural Classification of Proteins) [3].

  ### Attachments

  - 1756-0381-6-20.pdf
- ## Structure and Activity of NADPH-Dependent Reductase Q1EQE0 from Streptomyces kanamyceticus, which Catalyses the R-Selective Reduction of an Imine Substrate

  |  |  |
  | --- | --- |
  | Type | Journal Article |
  | Author | Maria Rodriguez-Mata |
  | Author | Annika Frank |
  | Author | Elizabeth Wells |
  | Author | Friedemann Leipold |
  | Author | Nicholas J. Turner |
  | Author | Sam Hart |
  | Author | Johan P. Turkenburg |
  | Author | Gideon Grogan |
  | Volume | 14 |
  | Issue | 11 |
  | Pages | 1372–1379 |
  | Publication | Chembiochem |
  | Date | July 2013 |
  | DOI | 10.1002/cbic.201300321 |
  | Abstract | NADPH-dependent oxidoreductase Q1EQE0 from Streptomyces kanamyceticus catalyzes the asymmetric reduction of the prochiral monocyclic imine 2-methyl-1-pyrroline to the chiral amine (R)-2-methylpyrrolidine with >99% ee, and is thus of interest as a potential biocatalyst for the production of optically active amines. The structures of Q1EQE0 in native form, and in complex with the nicotinamide cofactor NADPH have been solved and refined to a resolution of 2.7 angstrom. Q1EQE0 functions as a dimer in which the monomer consists of an N-terminal Rossman-fold motif attached to a helical C-terminal domain through a helix of 28 amino acids. The dimer is formed through reciprocal domain sharing in which the C-terminal domains are swapped, with a substrate-binding cleft formed between the N-terminal subunit of monomer A and the C-terminal subunit of monomer B. The structure is related to those of known -hydroxyacid dehydrogenases, except that the essential lysine, which serves as an acid/base in the (de)protonation of the nascent alcohol in those enzymes, is replaced by an aspartate residue, Asp187 in Q1EQE0. Mutation of Asp187 to either asparagine or alanine resulted in an inactive enzyme. |
  | Date Added | 3/7/2014, 1:06:24 PM |
  | Modified | 3/7/2014, 1:06:24 PM |
- ## Structure and assembly of a trans-periplasmic channel for type IV pili in Neisseria meningitidis

  |  |  |
  | --- | --- |
  | Type | Journal Article |
  | Author | Jamie-Lee Berry |
  | Author | Marie M. Phelan |
  | Author | Richard F. Collins |
  | Author | Tomas Adomavicius |
  | Author | Tone Tønjum |
  | Author | Stefan A. Frye |
  | Author | Louise Bird |
  | Author | Ray Owens |
  | Author | Robert C. Ford |
  | Author | Lu-Yun Lian |
  | URL | http://dx.plos.org/10.1371/journal.ppat.1002923 |
  | Volume | 8 |
  | Issue | 9 |
  | Pages | e1002923 |
  | Publication | PLoS Pathogens |
  | Date | 2012 |
  | Accessed | 9/20/2013, 1:13:08 PM |
  | Library Catalog | Google Scholar |
  | Date Added | 10/11/2013, 10:29:15 AM |
  | Modified | 10/11/2013, 10:29:15 AM |

  ### Notes:

  - Present structure of PilQ periplasmic domains (using NMR and homology modeling).  The paper details the function and structure of PilQ domains in determining their role in the secretion of pili by pathogenic bacteria. It also generates the structure of PilP:PilQ complex by NMR and its role.

    Use NMR to get structure, and computational method for studying docking.

    "We conclude that passage of the pilus fiber requires disassembly of both the membrane-spanning and the b-domain regions in PilQ, and that PilP plays an important role in stabilising the PilQ assembly during secretion, through its anchorage in the inner membrane."

    How SCOP is used:

    **L**ook up the fold of the PilQ b-domains. (Just the fold where the domain was under was mentioned).

    SCOP Reference:

    The most similar fold identified within the SCOP database [33] is the CS domain from the human Sgt1 kinetochore complex [34]. The b-domain fold is larger, however, and includes two additional b-strands, such that b5 is paired with b6, rather than b4, as is the case with the CS domain (Figure S1).

    Characterization of the domain fold was carried out using SCOP [33];

  ### Attachments

  - [HTML] from plos.org
  - journal.ppat.1002923.pdf
- ## Structure and Catalytic Mechanism of 3-Ketosteroid-Delta 4-(5 alpha)-dehydrogenase from Rhodococcus jostii RHA1 Genome

  |  |  |
  | --- | --- |
  | Type | Journal Article |
  | Author | Niels van Oosterwijk |
  | Author | Jan Knol |
  | Author | Lubbert Dijkhuizen |
  | Author | Robert van der Geize |
  | Author | Bauke W. Dijkstra |
  | Volume | 287 |
  | Issue | 37 |
  | Pages | 30975–30983 |
  | Publication | Journal of Biological Chemistry |
  | Date | September 2012 |
  | DOI | 10.1074/jbc.M112.374306 |
  | Abstract | 3-Ketosteroid Delta 4-(5 alpha)-dehydrogenases ( Delta 4-(5 alpha)-KSTDs) are enzymes that introduce a double bond between the C4 and C5 atoms of 3-keto-(5 alpha)-steroids. Here we show that the ro05698 gene from Rhodococcus jostii RHA1 codes for a flavoprotein with Delta 4-(5 alpha)-KSTD activity. The 1.6 angstrom resolution crystal structure of the enzyme revealed three conserved residues ( Tyr-319, Tyr-466, and Ser-468) in a pocket near the isoalloxazine ring system of the FAD co-factor. Site-directed mutagenesis of these residues confirmed that they are absolutely essential for catalytic activity. A crystal structure with bound product 4-androstene-3,17-dione showed that Ser-468 is in a position in which it can serve as the base abstracting the 4 beta-proton from the C4 atom of the substrate. Ser-468 is assisted by Tyr-319, which possibly is involved in shuttling the proton to the solvent. Tyr-466 is at hydrogen bonding distance to the C3 oxygen atom of the substrate and can stabilize the keto-enol intermediate occurring during the reaction. Finally, the FAD N5 atom is in a position to be able to abstract the 5 alpha-hydrogen of the substrate as a hydride ion. These features fully explain the reaction catalyzed by Delta 4-(5 alpha)-KSTDs. |
  | Date Added | 3/7/2014, 1:06:24 PM |
  | Modified | 3/7/2014, 1:06:24 PM |
- ## Structure and dynamics of a primordial catalytic fold generated by in vitro evolution

  |  |  |
  | --- | --- |
  | Type | Journal Article |
  | Author | Fa-An Chao |
  | Author | Aleardo Morelli |
  | Author | John C. Haugner III |
  | Author | Lewis Churchfield |
  | Author | Leonardo N. Hagmann |
  | Author | Lei Shi |
  | Author | Larry R. Masterson |
  | Author | Ritimukta Sarangi |
  | Author | Gianluigi Veglia |
  | Author | Burckhard Seelig |
  | URL | http://www.nature.com/nchembio/journal/v9/n2/abs/nchembio.1138.html |
  | Volume | 9 |
  | Issue | 2 |
  | Pages | 81–83 |
  | Publication | Nature chemical biology |
  | Date | 2012 |
  | Accessed | 9/20/2013, 1:11:53 PM |
  | Library Catalog | Google Scholar |
  | Date Added | 10/11/2013, 10:29:15 AM |
  | Modified | 10/11/2013, 10:29:15 AM |

  ### Notes:

  - The authors created an artificial RNA ligase in vitro which adopted a new form.

    "This artificial enzyme lost its original fold and adopted an entirely novel structure with dramatically enhanced conformational dynamics, demonstrating that a primordial fold with suitable flexibility is sufficient to carry out enzymatic function."

    **SCOP Use**

    SCOP isn't mentioned by name. The paper just notes that proteins structures can be classified under different folds with a reference to the SCOP authors. (No SCOP info/data used).

    **SCOP Reference:**

    The known structures of naturally occurring proteins can be assigned to an apparently finite number of different fold families1,2

    2. Murzin AG, Brenner SE, Hubbard T, Chothia C. J Mol Biol. 1995; 247:536–540. [PubMed: 7723011]

  ### Attachments

  - [HTML] from europepmc.org
  - nihms419141.pdf
  - Snapshot
- ## Structure and Function of a Novel LD-Carboxypeptidase A Involved in Peptidoglycan Recycling

  |  |  |
  | --- | --- |
  | Type | Journal Article |
  | Author | Debanu Das |
  | Author | Mireille Herve |
  | Author | Marc-Andre Elsliger |
  | Author | Rameshwar U. Kadam |
  | Author | Joanna C. Grant |
  | Author | Hsiu-Ju Chiu |
  | Author | Mark W. Knuth |
  | Author | Heath E. Klock |
  | Author | Mitchell D. Miller |
  | Author | Adam Godzik |
  | Author | Scott A. Lesley |
  | Author | Ashley M. Deacon |
  | Author | Dominique Mengin-Lecreulx |
  | Author | Ian A. Wilson |
  | Volume | 195 |
  | Issue | 24 |
  | Pages | 5555-5566 |
  | Publication | Journal of Bacteriology |
  | ISSN | 0021-9193; 1098-5530 |
  | Date | DEC 2013 |
  | Extra | WOS:000327546900014 |
  | DOI | 10.1128/JB.00900-13 |
  | Abstract | Approximately 50% of cell wall peptidoglycan in Gram-negative bacteria is recycled with each generation. The primary substrates used for peptidoglycan biosynthesis and recycling in the cytoplasm are GlcNAc-MurNAc(anhydro)-tetrapeptide and its degradation product, the free tetrapeptide. This complex process involves similar to 15 proteins, among which the cytoplasmic enzyme LD-carboxypeptidase A (LdcA) catabolizes the bond between the last two L- and D-amino acid residues in the tetrapeptide to form the tripeptide, which is then utilized as a substrate by murein peptide ligase (Mpl). LdcA has been proposed as an antibacterial target. The crystal structure of Novosphingobium aromaticivorans DSM 12444 LdcA (NaLdcA) was determined at 1.89-angstrom resolution. The enzyme was biochemically characterized and its interactions with the substrate modeled, identifying residues potentially involved in substrate binding. Unaccounted electron density at the dimer interface in the crystal suggested a potential site for disrupting protein-protein interactions should a dimer be required to perform its function in bacteria. Our analysis extends the identification of functional residues to several other homologs, which include enzymes from bacteria that are involved in hydrocarbon degradation and destruction of coral reefs. The NaLdcA crystal structure provides an alternate system for investigating the structure-function relationships of LdcA and increases the structural coverage of the protagonists in bacterial cell wall recycling. |
  | Date Added | 2/12/2014, 1:36:22 PM |
  | Modified | 2/12/2014, 1:36:22 PM |

  ### Notes:

  - Present crystal structure of a LdcA protein and studied function.

    How SCOP is used:

    look up domains and fold-level classification of LdcA protein.

    SCOP reference:

    The NaLdcA monomer is composed of ⬚⬚-helices H1 to H11 and ⬚⬚-strands ⬚⬚1 to ⬚⬚10 (Fig. 1). Both molecules in the asu are very similar in structure: chain A can be superimposed onto chain B, with an RMSD of 0.2 Å over 270 C⬚⬚ atoms. According to SCOP (59), the LdcA architecture has an N-terminal domain with a fla- vodoxin-like fold (residues ⬚⬚3 to 169 in PaLdcA) and a C-termi- nal domain with a “swiveling” ⬚⬚/⬚⬚/⬚⬚ fold (residues ⬚⬚170 to 307 in PaLdcA). The active site comprises a Ser104-His261-Glu191 cat- alytic triad located in a cleft between the two domains.

  ### Attachments

  - J. Bacteriol.-2013-Das-5555-66.pdf
- ## Structure and Function of Enzymes of Shikimate Pathway

  |  |  |
  | --- | --- |
  | Type | Journal Article |
  | Author | Aditya Dev |
  | Author | Satya Tapas |
  | Author | Shivendra Pratap |
  | Author | Pravindra Kumar |
  | URL | http://www.ingentaconnect.com/content/ben/cbio/2012/00000007/00000004/art00005 |
  | Volume | 7 |
  | Issue | 4 |
  | Pages | 374–391 |
  | Publication | Current Bioinformatics |
  | Date | 2012 |
  | Accessed | 9/23/2013, 10:22:27 AM |
  | Library Catalog | Google Scholar |
  | Date Added | 10/11/2013, 10:29:15 AM |
  | Modified | 10/11/2013, 10:29:15 AM |

  ### Tags:

  - Aromatic amino acid
  - crystal structure
  - enzyme
  - homology model
  - mycobacterium tuberculosis
  - shikimate pathway

  ### Notes:

  - No access to article.

  ### Attachments

  - Snapshot
- ## Structure and function of the DUF2233 domain in bacteria and in the human mannose 6-phosphate uncovering enzyme

  |  |  |
  | --- | --- |
  | Type | Journal Article |
  | Author | Debanu Das |
  | Author | Wang-Sik Lee |
  | Author | Joanna C. Grant |
  | Author | Hsiu-Ju Chiu |
  | Author | Carol L. Farr |
  | Author | Julie Vance |
  | Author | Heath E. Klock |
  | Author | Mark W. Knuth |
  | Author | Mitchell D. Miller |
  | Author | Marc-André Elsliger |
  | URL | http://www.jbc.org/content/288/23/16789.short |
  | Volume | 288 |
  | Issue | 23 |
  | Pages | 16789–16799 |
  | Publication | Journal of Biological Chemistry |
  | Date | 2013 |
  | Accessed | 9/20/2013, 1:12:35 PM |
  | Library Catalog | Google Scholar |
  | Date Added | 10/11/2013, 10:29:15 AM |
  | Modified | 10/11/2013, 10:29:15 AM |

  ### Tags:

  - Interesting

  ### Notes:

  - Experimental study of structure and function of the DUF2233 domain.

    How SCOP is used:

    Used FATCAT alignment search tool on SCOP to find folds similar to that of their crystal structure.  Found domains "bear some resemblance" to the cystatin fold in SCOP, and that one of the domains is belongs to a novel fold.

    SCOP reference:

    BACOVA\_00430 consist of four domains, each of which bears some resemblance to the cystatin fold (SCOP code 54402) (41), which consists of a curved antiparallel ⬚⬚-sheet wrapped around an ⬚⬚-helix (Fig. 1).

    ...

    Structural Comparisons—A search for other proteins of sim- ilar structure was carried out using FATCAT (42) (flexible alignment mode) against the SCOP database (43) and DALI (44). When queried using the entire BACOVA\_00430 struc- ture, FATCAT returned only two hits with significant p value scores (⬚⬚0.05): human latexin (Protein Data Bank code 2bo9; p ⬚⬚ 0.0286; C⬚⬚ r.m.s.d., ⬚⬚3 Å; sequence identity, ⬚⬚3%) and a protein of unknown function, YpmB, from Bacillus subtilis (Protein Data Bank code 2gu3; p ⬚⬚ 0.04; C⬚⬚ r.m.s.d., ⬚⬚2 Å;

    sequence identity, ⬚⬚3%). However, in both cases, the coverage is restricted to domain 1 of BACOVA\_00430 because latexin and YpmB are both ⬚⬚ and ⬚⬚ (⬚⬚ ⬚⬚ ⬚⬚) proteins belonging to the cystatin-like fold (and cystatin/monellin superfamily). No sig- nificant hits were found by FATCAT when the search was restricted to the DUF2233 domain. Similar results were obtained with a DALI search for the full BACOVA\_00430 structure; all structural similarities were again limited to the N-terminal domain, which most closely resembles the proto- typical cystatin-like fold. Thus, BACOVA\_00430 is the first structural representative of the novel DUF2233 domain architecture.

  ### Attachments

  - J. Biol. Chem.-2013-Das-16789-99.pdf
- ## Structure and function of tripeptidyl peptidase II, a giant cytosolic protease

  |  |  |
  | --- | --- |
  | Type | Journal Article |
  | Author | Beate Rockel |
  | Author | Klaus O. Kopec |
  | Author | Andrei N. Lupas |
  | Author | Wolfgang Baumeister |
  | Volume | 1824 |
  | Issue | 1 |
  | Pages | 237–245 |
  | Publication | Biochimica Et Biophysica Acta-proteins and Proteomics |
  | Date | January 2012 |
  | DOI | 10.1016/j.bbapap.2011.07.002 |
  | Abstract | Tripeptidyl peptidase II is the largest known eukaryotic peptidase. It has been described as a multi-purpose peptidase, which, in addition to its house-keeping function in intracellular protein degradation, plays a role in several vital cellular processes such as antigen processing, apoptosis, or cell division, and is involved in diseases like muscle wasting, obesity, and in cancer. Biochemical studies and bioinformatics have identified TPPII as a subtilase, but its structure is very unusual: it forms a large homooligomeric complex (6 MDa) with a spindle-like shape. Recently, the high-resolution structure of TPPII homodimers (300 kDa) was solved and a hybrid structure of the holocomplex built of 20 dimers was obtained by docking it into the EM-density. Here, we summarize our current knowledge about TPPII with a focus on structural aspects. This article is part of a Special Issue entitled: Proteolysis 50 years after the discovery of lysosome. (C) 2011 Elsevier B.V. All rights reserved. |
  | Date Added | 3/7/2014, 1:06:24 PM |
  | Modified | 3/7/2014, 1:06:24 PM |
- ## Structure and mechanisms of Escherichia coli aspartate transcarbamoylase

  |  |  |
  | --- | --- |
  | Type | Journal Article |
  | Author | William N Lipscomb |
  | Author | Evan R Kantrowitz |
  | Volume | 45 |
  | Issue | 3 |
  | Pages | 444-453 |
  | Publication | Accounts of chemical research |
  | ISSN | 1520-4898 |
  | Date | Mar 20, 2012 |
  | Extra | PMID: 22011033 |
  | Journal Abbr | Acc. Chem. Res. |
  | DOI | 10.1021/ar200166p |
  | Library Catalog | NCBI PubMed |
  | Language | eng |
  | Abstract | Enzymes catalyze a particular reaction in cells, but only a few control the rate of this reaction and the metabolic pathway that follows. One specific mechanism for such enzymatic control of a metabolic pathway involves molecular feedback, whereby a metabolite further down the pathway acts at a unique site on the control enzyme to alter its activity allosterically. This regulation may be positive or negative (or both), depending upon the particular system. Another method of enzymatic control involves the cooperative binding of the substrate, which allows a large change in enzyme activity to emanate from only a small change in substrate concentration. Allosteric regulation and homotropic cooperativity are often known to involve significant conformational changes in the structure of the protein. Escherichia coli aspartate transcarbamoylase (ATCase) is the textbook example of an enzyme that regulates a metabolic pathway, namely, pyrimidine nucleotide biosynthesis, by feedback control and by the cooperative binding of the substrate, L-aspartate. The catalytic and regulatory mechanisms of this enzyme have been extensively studied. A series of X-ray crystal structures of the enzyme in the presence and absence of substrates, products, and analogues have provided details, at the molecular level, of the conformational changes that the enzyme undergoes as it shifts between its low-activity, low-affinity form (T state) to its high-activity, high-affinity form (R state). These structural data provide insights into not only how this enzyme catalyzes the reaction between l-aspartate and carbamoyl phosphate to form N-carbamoyl-L-aspartate and inorganic phosphate, but also how the allosteric effectors modulate this activity. In this Account, we summarize studies on the structure of the enzyme and describe how these structural data provide insights into the catalytic and regulatory mechanisms of the enzyme. The ATCase-catalyzed reaction is regulated by nucleotide binding some 60 Å from the active site, inducing structural alterations that modulate catalytic activity. The delineation of the structure and function in this particular model system will help in understanding the molecular basis of cooperativity and allosteric regulation in other systems as well. |
  | Date Added | 2/20/2014, 12:24:01 PM |
  | Modified | 2/20/2014, 12:24:01 PM |

  ### Tags:

  - Allosteric Regulation
  - Aspartate Carbamoyltransferase
  - Crystallography, X-Ray
  - Escherichia coli
  - Models, Molecular
  - Structure-Activity Relationship

  ### Notes:

  - Paper unavailable.

  ### Attachments

  - PubMed entry
- ## Structure-based functional inference of hypothetical proteins from Mycoplasma hyopneumoniae

  |  |  |
  | --- | --- |
  | Type | Journal Article |
  | Author | Marbella Maria da Fonseca |
  | Author | Arnaldo Zaha |
  | Author | Ernesto R. Caffarena |
  | Author | Ana Tereza Ribeiro Vasconcelos |
  | Volume | 18 |
  | Issue | 5 |
  | Pages | 1917-1925 |
  | Publication | Journal of Molecular Modeling |
  | ISSN | 1610-2940 |
  | Date | MAY 2012 |
  | Extra | WOS:000303541900021 |
  | DOI | 10.1007/s00894-011-1212-3 |
  | Abstract | Enzootic pneumonia caused by Mycoplasma hyopneumoniae is a major constraint to efficient pork production throughout the world. This pathogen has a small genome with 716 coding sequences, of which 418 are homologous to proteins with known functions. However, almost 42% of the 716 coding sequences are annotated as hypothetical proteins. Alternative methodologies such as threading and comparative modeling can be used to predict structures and functions of such hypothetical proteins. Often, these alternative methods can answer questions about the properties of a model system faster than experiments. In this study, we predicted the structures of seven proteins annotated as hypothetical in M. hyopneumoniae, using the structure-based approaches mentioned above. Three proteins were predicted to be involved in metabolic processes, two proteins in transcription and two proteins where no function could be assigned. However, the modeled structures of the last two proteins suggested experimental designs to identify their functions. Our findings are important in diminishing the gap between the lack of annotation of important metabolic pathways and the great number of hypothetical proteins in the M. hyopneumoniae genome. |
  | Date Added | 2/20/2014, 12:24:01 PM |
  | Modified | 2/20/2014, 12:24:01 PM |

  ### Notes:

  - Computational study.  Use structure-based approaches to predict function of seven proteins annotated as hypothetical in pneumonia virus genome.

    How SCOP is used:

    background on protein structure classification.

    SCOP reference:

    These methods are possible because biological processes such as gene duplication and evolution- ary divergence occur in many distantly related organisms [8], giving rise to structurally and functionally similar families of proteins.

  ### Attachments

  - art%3A10.1007%2Fs00894-011-1212-3.pdf
- ## Structure-Based Function Prediction of Uncharacterized Protein Using Binding Sites Comparison

  |  |  |
  | --- | --- |
  | Type | Journal Article |
  | Author | Janez Konc |
  | Author | Milan Hodoscek |
  | Author | Mitja Ogrizek |
  | Author | Joanna Trykowska Konc |
  | Author | Dusanka Janezic |
  | Volume | 9 |
  | Issue | 11 |
  | Pages | e1003341 |
  | Publication | Plos Computational Biology |
  | ISSN | 1553-7358 |
  | Date | NOV 2013 |
  | Extra | WOS:000330357200040 |
  | DOI | 10.1371/journal.pcbi.1003341 |
  | Abstract | A challenge in structural genomics is prediction of the function of uncharacterized proteins. When proteins cannot be related to other proteins of known activity, identification of function based on sequence or structural homology is impossible and in such cases it would be useful to assess structurally conserved binding sites in connection with the protein's function. In this paper, we propose the function of a protein of unknown activity, the Tm1631 protein from Thermotoga maritima, by comparing its predicted binding site to a library containing thousands of candidate structures. The comparison revealed numerous similarities with nucleotide binding sites including specifically, a DNA-binding site of endonuclease IV. We constructed a model of this Tm1631 protein with a DNA-ligand from the newly found similar binding site using ProBiS, and validated this model by molecular dynamics. The interactions predicted by the Tm1631-DNA model corresponded to those known to be important in endonuclease IV-DNA complex model and the corresponding binding free energies, calculated from these models were in close agreement. We thus propose that Tm1631 is a DNA binding enzyme with endonuclease activity that recognizes DNA lesions in which at least two consecutive nucleotides are unpaired. Our approach is general, and can be applied to any protein of unknown function. It might also be useful to guide experimental determination of function of uncharacterized proteins. |
  | Date Added | 2/20/2014, 12:24:01 PM |
  | Modified | 2/20/2014, 12:24:01 PM |

  ### Notes:

  - Present method for function prediction.

    Apply to an uncharacterized protein, Tm1631 in T. maritima, and predict function using ProBiS MD simulation to find structurally conserved bindng sites.

    How SCOP is used:

    Search for homologs.  Look at families within the same fold of protein studied (TM1631).

    SCOP reference [15]:

    To refine the search and narrow down possible functions of Tm1631 protein, we compare this newly identified phosphate binding site with the binding sites in endonuclease IV nucleic acids binding proteins, which are the closest relatives of Tm1631 according to sequence identity, in the a8b8 triose phosphate isomerase (TIM) barrel fold [15] of which the Tm1631 is a member. A similarity is detected with endonuclease IV DNA-binding site, one of the TIM barrel folds.

  ### Attachments

  - journal.pcbi.1003341.pdf
- ## Structure-based redesign of proteins for minimal T-cell epitope content

  |  |  |
  | --- | --- |
  | Type | Journal Article |
  | Author | Yoonjoo Choi |
  | Author | Karl E. Griswold |
  | Author | Chris Bailey-Kellogg |
  | Volume | 34 |
  | Issue | 10 |
  | Pages | 879-891 |
  | Publication | Journal of Computational Chemistry |
  | ISSN | 0192-8651 |
  | Date | APR 5 2013 |
  | Extra | WOS:000316328600009 |
  | DOI | 10.1002/jcc.23213 |
  | Abstract | The protein universe displays a wealth of therapeutically relevant activities, but T-cell driven immune responses to non-self biological agents present a major impediment to harnessing the full diversity of these molecular functions. Mutagenic T-cell epitope deletion seeks to mitigate the immune response, but can typically address only a small number of epitopes. Here, we pursue a bottom-up approach that redesigns an entire protein to remain native-like but contain few if any immunogenic epitopes. We do so by extending the Rosetta flexible-backbone protein design software with an epitope scoring mechanism and appropriate constraints. The method is benchmarked with a diverse panel of proteins and applied to three targets of therapeutic interest. We show that the deimmunized designs indeed have minimal predicted epitope content and are native-like in terms of various quality measures, and moreover that they display levels of native sequence recovery comparable to those of non-deimmunized designs. (c) 2013 Wiley Periodicals, Inc. |
  | Date Added | 2/20/2014, 12:24:01 PM |
  | Modified | 3/7/2014, 12:10:07 PM |

  ### Notes:

  - Extend the rosetta flexilbe-backbone protein design method with an epitope scoring.

    How SCOP/CATH is used:

    Cite previous work that a TM-score > 0.8 implies agreement with the SCOP and CATH fold classification.

    SCOP/CATH reference:

    Design evaluation

    In addition to having minimal epitope content (for strategies with deimmunization), a redesigned protein should ‘look natu- ral’. We evaluate how well that goal has been achieved with several measures:

    • energy, according to the Rosetta energy

    • packing quality, according to the packstat score in Rosetta; very high resolution X-ray structures (sub 1.0 A ̊) have scores > 0.6.

    **• structural distortion from target backbone, according to TM-score;[51] > 0.5 generally indicates the same fold and > 0.8**

    **almost perfectly agrees with SCOP [52] and CATH [53] fold classi- fications** **[54]**

    • core hydrophobicity compared to wild-type proteins, according to the Kyte–Doolittle measure.[55] Core residues are determined as those with absolute solvent accessiblity < 20 according to the DSSP program.[56]

    • charge conservation, according to counts of charged residues

    • native sequence recovery (i.e., sequence identity to wild- type), as implemented by sequence\_recovery in Rosetta; the initial work by Kuhlman and Baker showed 27% native sequence recovery overall and 51% in the core[35]

  ### Attachments

  - 23213\_ftp.pdf
- ## Structure determination through homology modelling and torsion-angle simulated annealing: application to a polysaccharide deacetylase from Bacillus cereus

  |  |  |
  | --- | --- |
  | Type | Journal Article |
  | Author | Vasiliki E. Fadouloglou |
  | Author | Maria Kapanidou |
  | Author | Athanasia Agiomirgianaki |
  | Author | Sofia Arnaouteli |
  | Author | Vassilis Bouriotis |
  | Author | Nicholas M. Glykos |
  | Author | Michael Kokkinidis |
  | Volume | 69 |
  | Pages | 276–283 |
  | Publication | Acta Crystallographica Section D-biological Crystallography |
  | Date | February 2013 |
  | DOI | 10.1107/S0907444912045829 |
  | Abstract | The structure of BC0361, a polysaccharide deacetylase from Bacillus cereus, has been determined using an unconventional molecular-replacement procedure. Tens of putative models of the C-terminal domain of the protein were constructed using a multitude of homology-modelling algorithms, and these were tested for the presence of signal in molecular-replacement calculations. Of these, only the model calculated by the SAM-T08 server gave a consistent and convincing solution, but the resulting model was too inaccurate to allow phase determination to proceed to completion. The application of slow-cooling torsion-angle simulated annealing (started from a very high temperature) drastically improved this initial model to the point of allowing phasing through cycles of model building and refinement to be initiated. The structure of the protein is presented with emphasis on the presence of a C-alpha-modified proline at its active site, which was modelled as an alpha-hydroxy-L-proline. |
  | Date Added | 3/7/2014, 12:08:00 PM |
  | Modified | 3/7/2014, 12:08:00 PM |
- ## Structure-function analysis of human TYW2 enzyme required for the biosynthesis of a highly modified Wybutosine (yW) base in phenylalanine-tRNA

  |  |  |
  | --- | --- |
  | Type | Journal Article |
  | Author | Virginia Rodriguez |
  | Author | Sona Vasudevan |
  | Author | Akiko Noma |
  | Author | Bradley A. Carlson |
  | Author | Jeffrey E. Green |
  | Author | Tsutomu Suzuki |
  | Author | Settara C. Chandrasekharappa |
  | URL | http://dx.plos.org/10.1371/journal.pone.0039297 |
  | Volume | 7 |
  | Issue | 6 |
  | Pages | e39297 |
  | Publication | PloS one |
  | Date | 2012 |
  | Accessed | 9/20/2013, 1:18:50 PM |
  | Library Catalog | Google Scholar |
  | Date Added | 10/11/2013, 10:29:15 AM |
  | Modified | 10/11/2013, 10:29:15 AM |

  ### Tags:

  - Animals
  - Female
  - Humans
  - Mammary Glands, Animal
  - Mice
  - Nucleosides
  - RNA, Transfer, Phe
  - Saccharomyces cerevisiae
  - Structure-Activity Relationship

  ### Notes:

  - Experiment and computational study to characterize hTYW2 enzyme.

    How SCOP is used:

    Get SCOP fold classification for the studied enzyme.

    SCOP reference:

    The hTYW2 belongs to AdoMet-dependent methyltransferase SCOP (http://scop.mrc-lmb.cam.ac.uk/scop) fold with a topology consistent with Class I methyltransferases. The arrangement of beta strands is in the order 6754123 with strand 7 anti-parallel to rest of the strands as is typically seen in this fold. It belongs to the class of alpha/beta proteins as per SCOP classifications [22].

  ### Attachments

  - [HTML] from plos.org
  - journal.pone.0039297.pdf
  - PubMed entry
- ## Structure homology and interaction redundancy for discovering virus-host protein interactions

  |  |  |
  | --- | --- |
  | Type | Journal Article |
  | Author | Benoit de Chassey |
  | Author | Laurene Meyniel-Schicklin |
  | Author | Anne Aublin-Gex |
  | Author | Vincent Navratil |
  | Author | Thibaut Chantier |
  | Author | Patrice Andre |
  | Author | Vincent Lotteau |
  | Volume | 14 |
  | Issue | 10 |
  | Pages | 938-944 |
  | Publication | Embo Reports |
  | ISSN | 1469-221X |
  | Date | OCT 2013 |
  | Extra | WOS:000325253100019 |
  | DOI | 10.1038/embor.2013.130 |
  | Abstract | Virus-host interactomes are instrumental to understand global perturbations of cellular functions induced by infection and discover new therapies. The construction of such interactomes is, however, technically challenging and time consuming. Here we describe an original method for the prediction of high-confidence interactions between viral and human proteins through a combination of structure and high-quality interactome data. Validation was performed for the NS1 protein of the influenza virus, which led to the identification of new host factors that control viral replication. |
  | Date Added | 2/12/2014, 1:36:22 PM |
  | Modified | 2/12/2014, 1:36:22 PM |

  ### Notes:

  - Present a method for the prediction of virus-host protein interactions.

    How SCOP is used:

    Integrate SCOP database into method.  Search for proteins with similar structures to the virus and host proteins in SCOP.  Use these in the method.

    SCOP reference:

    RESULTS AND DISCUSSION  
     Principle of the method  
     Several methods have proposed to use structural information to predict protein–protein interactions [8–10]. The method described here relies on the assumption that when two proteins are structurally homologous, they are more likely to have interactors in common (Fig 1A). Using the Structural Classification of Proteins from SCOP database [11] and the high-quality data sets of protein–protein interactions from the VirHostNet database [2], we showed that this assumption is a general feature of human and viral proteins (supplementary information online). Briefly, for a viral protein having a solved structure, structurally homologous human and viral proteins are first selected. Inter- actors of these homologous proteins are then identified. These proteins are considered as putative interactors and ranked according to a score that favors proteins independently identified from multiple structural homologues.

  ### Attachments

  - 938.full.pdf
- ## Structure Motivator: A tool for exploring small three-dimensional elements in proteins

  |  |  |
  | --- | --- |
  | Type | Journal Article |
  | Author | David P. Leader |
  | Author | E. J. Milner-White |
  | URL | http://www.biomedcentral.com/1472-6807/12/26/abstract |
  | Rights | 2012 Leader and Milner-White; licensee BioMed Central Ltd. |
  | Volume | 12 |
  | Issue | 1 |
  | Pages | 26 |
  | Publication | BMC Structural Biology |
  | ISSN | 1472-6807 |
  | Date | 2012-10-16 |
  | Extra | PMID: 23067391 |
  | DOI | 10.1186/1472-6807-12-26 |
  | Accessed | 12/9/2014, 6:57:34 AM |
  | Library Catalog | www.biomedcentral.com |
  | Language | en |
  | Abstract | Protein structures incorporate characteristic three-dimensional elements defined by some or all of hydrogen bonding, dihedral angles and amino acid sequence. The software application, Structure Motivator, allows interactive exploration and analysis of such elements, and their resolution into sub-classes. PMID: 23067391 |
  | Short Title | Structure Motivator |
  | Date Added | 12/9/2014, 6:57:34 AM |
  | Modified | 12/9/2014, 6:57:34 AM |

  ### Tags:

  - Dihedral angle
  - Protein motif
  - Ramachandran plot
  - Relational database

  ### Attachments

  - Full Text PDF
  - Snapshot
- ## Structure of a thermophilic cyanobacterial b6f-type Rieske protein

  |  |  |
  | --- | --- |
  | Type | Journal Article |
  | Author | Sebastian Veit |
  | Author | Kazuki Takeda |
  | Author | Yuichi Tsunoyama |
  | Author | Dorothea Rexroth |
  | Author | Matthias Roegner |
  | Author | Kunio Miki |
  | Volume | 68 |
  | Pages | 1400–1408 |
  | Publication | Acta Crystallographica Section D-biological Crystallography |
  | Date | October 2012 |
  | DOI | 10.1107/S0907444912034129 |
  | Abstract | The `Rieske protein' PetC is one of the key subunits of the cytochrome b6f complex. Its Rieske-type [2Fe2S] cluster participates in the photosynthetic electron-transport chain. Overexpression and careful structure analysis at 2.0 angstrom resolution of the extrinsic soluble domain of PetC from the thermophilic cyanobacterium Thermosynechococcus elongatus BP-1 enabled in-depth spectroscopic and structural characterization and suggested novel structural features. In particular, both the protein structure and the positions of the internal water molecules unexpectedly showed a higher similarity to eukaryotic PetCs than to other prokaryotic PetCs. The structure also revealed a deep pocket on the PetC surface which is oriented towards the membrane surface in the whole complex. Its surface properties suggest a binding site for a hydrophobic compound and the complete conservation of the pocket-forming residues in all known PetC sequences indicates the functional importance of this pocket in the cytochrome b6f complex. |
  | Date Added | 3/7/2014, 1:06:24 PM |
  | Modified | 3/7/2014, 1:06:24 PM |
- ## Structure of isochorismate synthase DhbC from Bacillus anthracis

  |  |  |
  | --- | --- |
  | Type | Journal Article |
  | Author | M. J. Domagalski |
  | Author | K. L. Tkaczuk |
  | Author | M. Chruszcz |
  | Author | T. Skarina |
  | Author | O. Onopriyenko |
  | Author | M. Cymborowski |
  | Author | M. Grabowski |
  | Author | A. Savchenko |
  | Author | W. Minor |
  | URL | http://scripts.iucr.org/cgi-bin/paper?S1744309113021246 |
  | Volume | 69 |
  | Issue | 9 |
  | Pages | 0–0 |
  | Publication | Acta Crystallographica Section F: Structural Biology and Crystallization Communications |
  | Date | 2013 |
  | Accessed | 9/23/2013, 10:22:14 AM |
  | Library Catalog | Google Scholar |
  | Date Added | 10/11/2013, 10:29:15 AM |
  | Modified | 2/18/2014, 12:26:16 PM |

  ### Notes:

  - Present X-ray crystallography structure of isocochorismate synthase DhbC from Bacillus anthracis.

    How SCOP is used:

    Use DALI to find the SCOP family classification.  Lists sccs.  Also found that all other structures of isochorismate-utilizing enzymes belonged to the same family.

    SCOP reference:

    Structural similarity searches using DALI (Holm & Rosenstro ̈ m, 2010) suggest that DhbC belongs to the aminodeoxychorismate (ADC) synthase domain family according to the SCOP classification (SCOP class d.161.1.1; Murzin et al., 1995) and the PF00425 family (which contains chorismate-binding enzymes) in the Pfam classification (Bateman et al., 2004). DhbC adopts the ADC synthase-like fold

    ...

    The structures of several isochorismate-utilizing enzymes have previously been solved by X-ray crystallography (Table 2)...All of these belong to the same structural family according to SCOP and they all take part in the conversion of chorismate to isochorismate (EntC and MenF), salicylate (Irp9 and Mbtl), anthranilate (TrpE) or ADIC (PhzE).

  ### Attachments

  - kw5067.pdf
- ## Structure of MMACHC Reveals an Arginine-Rich Pocket and a Domain-Swapped Dimer for Its B-12 Processing Function

  |  |  |
  | --- | --- |
  | Type | Journal Article |
  | Author | D. Sean Froese |
  | Author | Tobias Krojer |
  | Author | Xuchu Wu |
  | Author | Roshi Shrestha |
  | Author | Wasim Kiyani |
  | Author | Frank von Delft |
  | Author | Roy A. Gravel |
  | Author | Udo Oppermann |
  | Author | Wyatt W. Yue |
  | Volume | 51 |
  | Issue | 25 |
  | Pages | 5083-5090 |
  | Publication | Biochemistry |
  | ISSN | 0006-2960 |
  | Date | JUN 26 2012 |
  | Extra | WOS:000305661800011 |
  | Journal Abbr | Biochemistry |
  | DOI | 10.1021/bi300150y |
  | Library Catalog | ISI Web of Knowledge |
  | Language | English |
  | Abstract | Defects in the MMACHC gene represent the most common disorder of cobalamin (Cbl) metabolism, affecting synthesis of the enzyme cofactors adenosyl-Cbl and methyl-Cbl. The encoded MMACHC protein binds intracellular Cbl derivatives with different upper axial ligands and exhibits flavin mononucleotide (FMN)-dependent decyanase activity toward cyano-Cbl as well as glutathione (GSH)-dependent dealkylase activity toward alkyl-Cbls. We determined the structure of human MMACHC.adenosyl-Cbl complex, revealing a tailor-made nitroreductase scaffold which binds adenosyl-Cbl in a "base-off, five-coordinate" configuration for catalysis. We further identified an arginine-rich pocket close to the Cbl binding site responsible for GSH binding and dealkylation activity. Mutation of these highly conserved arginines, including a replication of the prevalent MMACHC missense mutation, Arg161Gln, disrupts GSH binding and dealkylation. We further showed that two Cbl-binding monomers dimerize to mediate the reciprocal exchange of a conserved "PNRRP" loop from both subunits, serving as a protein cap for the upper axial ligand in trans and required for proper dealkylation activity. Our dimeric structure is supported by solution studies, where dimerization is triggered upon binding its substrate adenosyl-Cbl or cofactor FMN. Together our data provide a structural framework to understanding catalytic function and disease mechanism for this multifunctional enzyme. |
  | Date Added | 10/8/2014, 12:29:53 PM |
  | Modified | 10/8/2014, 1:32:21 PM |

  ### Tags:

  - activation
  - adenosylcobalamin
  - cblc
  - enzyme
  - glutathione
  - homocystinuria
  - methylmalonic aciduria
  - protein
  - vitamin-b-12

  ### Attachments

  - ACS Full Text PDF w/ Links
  - ACS Full Text Snapshot
- ## Structure of Myoglobin: A Three-Dimensional Fourier Synthesis at 2 |[angst]|. Resolution

  |  |  |
  | --- | --- |
  | Type | Journal Article |
  | Author | J. C. Kendrew |
  | Author | R. E. Dickerson |
  | Author | B. E. Strandberg |
  | Author | R. G. Hart |
  | Author | D. R. Davies |
  | Author | D. C. Phillips |
  | Author | V. C. Shore |
  | URL | http://www.nature.com/nature/journal/v185/n4711/abs/185422a0.html |
  | Rights | © 1960 Nature Publishing Group |
  | Volume | 185 |
  | Issue | 4711 |
  | Pages | 422-427 |
  | Publication | Nature |
  | Date | February 13, 1960 |
  | Journal Abbr | Nature |
  | DOI | 10.1038/185422a0 |
  | Accessed | 10/29/2014, 11:40:05 AM |
  | Library Catalog | www.nature.com |
  | Language | en |
  | Short Title | Structure of Myoglobin |
  | Date Added | 10/29/2014, 11:40:20 AM |
  | Modified | 10/29/2014, 11:40:20 AM |

  ### Attachments

  - Full Text PDF
  - Snapshot
- ## Structure of ribose 5-phosphate isomerase from the probiotic bacterium Lactobacillus salivarius UCC118

  |  |  |
  | --- | --- |
  | Type | Journal Article |
  | Author | Carina M. C. Lobley |
  | Author | Pierre Aller |
  | Author | Alice Douangamath |
  | Author | Yamini Reddivari |
  | Author | Mario Bumann |
  | Author | Louise E. Bird |
  | Author | Joanne E. Nettleship |
  | Author | Jose Brandao-Neto |
  | Author | Raymond J. Owens |
  | Author | Paul W. O'Toole |
  | Author | Martin A. Walsh |
  | Volume | 68 |
  | Issue | 12 |
  | Pages | 1427-1433 |
  | Publication | Acta Crystallographica Section F: Structural Biology and Crystallization Communications |
  | ISSN | 1744-3091 |
  | Date | December 2012 |
  | DOI | 10.1107/S174430911204273X |
  | Language | English |
  | Abstract | The structure of ribose 5-phosphate isomerase from the probiotic bacterium Lactobacillus salivarius UCC188 has been determined at 1.72 angstrom resolution. The structure was solved by molecular replacement, which identified the functional homodimer in the asymmetric unit. Despite only showing 57% sequence identity to its closest homologue, the structure adopted the typical alpha and beta D-ribose 5-phosphate isomerase fold. Comparison to other related structures revealed high homology in the active site, allowing a model of the substrate-bound protein to be proposed. The determination of the structure was expedited by the use of in situ crystallization-plate screening on beamline I04-1 at Diamond Light Source to identify well diffracting protein crystals prior to routine cryocrystallo-graphy. |
  | Date Added | 10/11/2013, 10:29:15 AM |
  | Modified | 12/2/2013, 4:18:43 PM |

  ### Notes:

  - Describe structure of ribose 5-phosphate isomerase

    How SCOP is used:

    Use case: provide background on structure classification of protein of interest.

    Description: Provide SCOP family for L. salvarius.

    SCOP reference:

    3.2. RpiA structure and comparison with the D-ribose-5-phosphate isomerase family

    The overall topology of L. salivarius RpiA (Figs. 2 and 3) is broadly similar to other known RpiA structures. The N-terminal domain consists of ⬚⬚-helices 1–5 and ⬚⬚-strands 1–3, 6, 12 and 13. The C-terminal domain consists of ⬚⬚-helices 6 and 7 and ⬚⬚-strands 7–10. The remaining three ⬚⬚-strands form the interface between the two domains. In RpiA structures the domain interface typically consists of four ⬚⬚-strands. From a structural superposition (not shown) we see that the fourth strand, which would have been made by residues 124– 126, is in fact an extended loop in the L. salivarius structure. While there is this very subtle change in topology, the extended loop occupies broadly the same position as the short ⬚⬚-strand that it replaces. The L. salivarius structure fits well into the SCOP ⬚⬚ and ⬚⬚ family of d-ribose-5-phosphate isomerase (RpiA) catalytic domains, as would be expected.

  ### Attachments

  - hv5226.pdf
- ## Structure of staphylococcal alpha-hemolysin, a heptameric transmembrane pore

  |  |  |
  | --- | --- |
  | Type | Journal Article |
  | Author | LZ Song |
  | Author | MR Hobaugh |
  | Author | C Shustak |
  | Author | S Cheley |
  | Author | H Bayley |
  | Author | JE Gouaux |
  | Volume | 274 |
  | Issue | 5294 |
  | Pages | 1859-1866 |
  | Publication | SCIENCE |
  | ISSN | 0036-8075 |
  | Date | DEC 13 1996 |
  | DOI | 10.1126/science.274.5294.1859 |
  | Language | English |
  | Abstract | The structure of the Staphylococcus aureus alpha-hemolysin pore has been determined to 1.9 Angstrom resolution. Contained within the mushroom-shaped homo-oligomeric heptamer is a solvent-filled channel, 100 Angstrom in length, that runs along the sevenfold axis and ranges from 14 Angstrom to 46 Angstrom in diameter. The lytic, transmembrane domain comprises the lower half of a 14-strand antiparallel beta barrel, to which each protomer contributes two beta strands, each 65 Angstrom long. The interior of the beta barrel is primarily hydrophilic, and the exterior has a hydrophobic belt 28 Angstrom wide. The structure proves the heptameric subunit stoichiometry of the alpha-hemolysin oligomer, shows that a glycine-rich and solvent-exposed region of a water-soluble protein can self-assemble to form a transmembrane pore of defined structure, and provides insight into the principles of membrane interaction and transport activity of beta barrel pore-forming toxins. |
  | Date Added | 10/11/2013, 10:29:15 AM |
  | Modified | 10/11/2013, 10:29:15 AM |

  ### Notes:

  - Crystallography paper describing structure of alpha-hemolysin.

    How SCOP is used:

    Have examined SCOP to find if there are any folds similar to the protomers that they are studying.  Verify that their structure does indeed have a novel fold.

    SCOP reference:

    The protomers adopt a tertiary fold that is distinct from folds previously described.

  ### Attachments

  - Science-1996-Song-1859-65.pdf
- ## Structure of the Chlamydia trachomatis Immunodominant Antigen Pgp3

  |  |  |
  | --- | --- |
  | Type | Journal Article |
  | Author | Ahmad Galaleldeen |
  | Author | Alexander B. Taylor |
  | Author | Ding Chen |
  | Author | Jonathan P. Schuermann |
  | Author | Stephen P. Holloway |
  | Author | Shuping Hou |
  | Author | Siqi Gong |
  | Author | Guangming Zhong |
  | Author | P. John Hart |
  | Volume | 288 |
  | Issue | 30 |
  | Pages | 22068-22079 |
  | Publication | Journal of Biological Chemistry |
  | Date | JUL 26 2013 |
  | Extra | WOS:000328841900052 |
  | DOI | 10.1074/jbc.M113.475012 |
  | Library Catalog | ISI Web of Knowledge |
  | Abstract | Chlamydia trachomatis infection is the most common sexually transmitted bacterial disease. Left untreated, it can lead to ectopic pregnancy, pelvic inflammatory disease, and infertility. Here we present the structure of the secreted C. trachomatis protein Pgp3, an immunodominant antigen and putative virulence factor. The approximate to 84-kDa Pgp3 homotrimer, encoded on a cryptic plasmid, consists of globular N- and C-terminal assemblies connected by a triple-helical coiled-coil. The C-terminal domains possess folds similar to members of the TNF family of cytokines. The closest Pgp3 C-terminal domain structural homologs include a lectin from Burkholderia cenocepacia, the C1q component of complement, and a portion of the Bacillus anthracis spore surface protein BclA, all of which play roles in bioadhesion. The N-terminal domain consists of a concatenation of structural motifs typically found in trimeric viral proteins. The central parallel triple-helical coiled-coil contains an unusual alternating pattern of apolar and polar residue pairs that generate a rare right-handed superhelical twist. The unique architecture of Pgp3 provides the basis for understanding its role in chlamydial pathogenesis and serves as the platform for its optimization as a potential vaccine antigen candidate. |
  | Date Added | 10/8/2014, 12:49:22 PM |
  | Modified | 10/8/2014, 1:32:33 PM |

  ### Tags:

  - Bacterial Pathogenesis
  - Beta-Helix
  - Beta-Propeller
  - Chlamydia
  - Helical Coiled-coil
  - Immunology
  - Pgp3
  - Sexually Transmitted Disease
  - Tumor Necrosis Factor (TNF)
  - X-ray Crystallography

  ### Notes:

  - Present strucure of Pgp3, an immunogenic protein secreted by Chlamydia trachomatis.

    How SCOP is used:

    Use website to search and browse for structures of interest.

    SCOP reference:

    Concatenated structural motifs similar to those typically found in viral proteins (Fig. 5) were discovered by eye during perusal of the Structural Classification

    of Proteins (SCOP) database (39).

  ### Attachments

  - Full Text PDF
- ## Structure of the Mtb CarD/RNAP beta-Lobes Complex Reveals the Molecular Basis of Interaction and Presents a Distinct DNA-Binding Domain for Mtb CarD

  |  |  |
  | --- | --- |
  | Type | Journal Article |
  | Author | Gulcin Gulten |
  | Author | James C. Sacchettini |
  | Volume | 21 |
  | Issue | 10 |
  | Pages | 1859-1869 |
  | Publication | Structure |
  | ISSN | 0969-2126; 1878-4186 |
  | Date | OCT 8 2013 |
  | Extra | WOS:000326413500016 |
  | DOI | 10.1016/j.str.2013.08.014 |
  | Abstract | CarD from Mycobacterium tuberculosis (Mtb) is an essential protein shown to be involved in stringent response through downregulation of rRNA and ribosomal protein genes. CarD interacts with the beta-subunit of RNAP and this interaction is vital for Mtb's survival during the persistent infection state. We have determined the crystal structure of CarD in complex with the RNAP beta-subunit beta 1 and beta 2 domains at 2.1 angstrom resolution. The structure reveals the molecular basis of CarD/RNAP interaction, providing a basis to further our understanding of RNAP regulation by CarD. The structural fold of the CarD N-terminal domain is conserved in RNAP interacting proteins such as TRCF-RID and CdnL, and displays similar interactions to the predicted homology model based on the TRCF/RNAP beta 1 structure. Interestingly, the structure of the C-terminal domain, which is required for complete CarD function in vivo, represents a distinct DNA-binding fold. |
  | Date Added | 2/12/2014, 1:36:22 PM |
  | Modified | 2/12/2014, 1:36:22 PM |

  ### Notes:

  - Determine crystal structure of CarD/RNAP.

    How SCOP is used:

    Look up structural class classification of Mtb CarD protein, and get domains.

    SCOP reference:

    Overall Structure of Mtb CarD  
     Mtb CarD belongs to the a + b protein class (SCOP) (Murzin et al., 1995). The structure is composed of two distinct domains: an all b-stranded N-terminal domain (residues 1–49) and an all a-helical C-terminal domain (residues 63–160; Figures 1A and 2A).

  ### Attachments

  - 1-s2.0-S0969212613003067-main.pdf
- ## Structure of the nucleotide-binding domain of a dipeptide ABC transporter reveals a novel iron-sulfur cluster-binding domain

  |  |  |
  | --- | --- |
  | Type | Journal Article |
  | Author | Xiaolu Li |
  | Author | Wei Zhuo |
  | Author | Jie Yu |
  | Author | Jingpeng Ge |
  | Author | Jinke Gu |
  | Author | Yue Feng |
  | Author | Maojun Yang |
  | Author | Linfang Wang |
  | Author | Na Wang |
  | Volume | 69 |
  | Pages | 256–265 |
  | Publication | Acta Crystallographica Section D-biological Crystallography |
  | Date | February 2013 |
  | DOI | 10.1107/S0907444912045180 |
  | Abstract | Dipeptide permease (Dpp), which belongs to an ABC transport system, imports peptides consisting of two or three l-amino acids from the matrix to the cytoplasm in microbes. Previous studies have indicated that haem competes with dipeptides to bind DppA in vitro and in vivo and that the Dpp system can also translocate haem. Here, the crystal structure of DppD, the nucleotide-binding domain (NBD) of the ABC-type dipeptide/oligopeptide/nickel-transport system from Thermoanaerobacter tengcongensis, bound with ATP, Mg2+ and a [4Fe-4S] iron-sulfur cluster is reported. The N-terminal domain of DppD shares a similar structural fold with the NBDs of other ABC transporters. Interestingly, the C-terminal domain of DppD contains a [4Fe-4S] cluster. The UV-visible absorbance spectrum of DppD was consistent with the presence of a [4Fe-4S] cluster. A search with DALI revealed that the [4Fe-4S] cluster-binding domain is a novel structural fold. Structural analysis and comparisons with other ABC transporters revealed that this iron-sulfur cluster may act as a mediator in substrate (dipeptide or haem) binding by electron transfer and may regulate the transport process in Dpp ABC transport systems. The crystal structure provides a basis for understanding the properties of ABC transporters and will be helpful in investigating the functions of NBDs in the regulation of ABC transporter activity. |
  | Date Added | 3/7/2014, 1:06:24 PM |
  | Modified | 3/7/2014, 1:06:24 PM |
- ## Structure Prediction and Analysis of DNA Transposon and LINE Retrotransposon Proteins

  |  |  |
  | --- | --- |
  | Type | Journal Article |
  | Author | Gyoergy Abrusan |
  | Author | Yang Zhang |
  | Author | Andras Szilagyi |
  | Volume | 288 |
  | Issue | 22 |
  | Pages | 16127-16138 |
  | Publication | Journal of Biological Chemistry |
  | ISSN | 0021-9258 |
  | Date | MAY 31 2013 |
  | Extra | WOS:000319822300063 |
  | DOI | 10.1074/jbc.M113.451500 |
  | Abstract | Despite the considerable amount of research on transposable elements, no large-scale structural analyses of the TE proteome have been performed so far. We predicted the structures of hundreds of proteins from a representative set of DNA and LINE transposable elements and used the obtained structural data to provide the first general structural characterization of TE proteins and to estimate the frequency of TE domestication and horizontal transfer events. We show that 1) ORF1 and Gag proteins of retrotransposons contain high amounts of structural disorder; thus, despite their very low conservation, the presence of disordered regions and probably their chaperone function is conserved. 2) The distribution of SCOP classes in DNA transposons and LINEs indicates that the proteins of DNA transposons are more ancient, containing folds that already existed when the first cellular organisms appeared. 3) DNA transposon proteins have lower contact order than randomly selected reference proteins, indicating rapid folding, most likely to avoid protein aggregation. 4) Structure-based searches for TE homologs indicate that the overall frequency of TE domestication events is low, whereas we found a relatively high number of cases where horizontal transfer, frequently involving parasites, is the most likely explanation for the observed homology. |
  | Date Added | 2/20/2014, 12:24:01 PM |
  | Modified | 2/20/2014, 12:24:01 PM |

  ### Notes:

  - Computational study of transposon elements (TE) proteome.

    How SCOP is used:

    1. Annotate data set of transposon element proteins with SCOP class and fold.  study whether the folds tend to be more 'ancient'.

    SCOP reference:

    The distribution of SCOP classes in DNA trans- posons and LINEs indicates that the proteins of DNA trans- posons are more ancient, containing folds that already existed when the first cellular organisms appeared.

    ...

    Briefly, FiefDom generates a PSSM using a query sequence and a refer- ence database (nr) and searches for domain boundaries using the distribution of hits from a structure database (SCOP (21) or PDB (22)). Combining the coordinates of conserved domains and the distribution of SCOP/PDB hits on the TE protein sequence, we identified domain boundaries at the regions with the lowest sequence coverage, and when these boundaries col- lided with conserved domains, we adjusted them manually (Fig. 1).

    ...

    Identification of SCOP Domains in the TE Structures and Their Enrichment—To provide a functional characterization of the TE proteins, we searched the SCOP database (21) to identify domains that are similar to the predicted TE proteins with a minimum TM score of 0.5. We searched SCOP for structurally similar domains, excluding the sequences with higher sequence similarity than 95% (ASTRAL95) in an all versus all manner: all TE structures were compared with all SCOP structures with TMfold (24). From the hits, we kept only those with a TM score higher than 0.5 and a minimum number of aligned residues higher than 80, as the probabilistic background of detecting shorter matches is not well understood (24). Because there is large structural redundancy within SCOP domains, we applied a further filtering step; from the overlapping SCOP matches, we kept only those most similar to the query TE structure, i.e. with the highest TM score, and the highest number of aligned resi- dues closer than 5 Å. This step removed the redundant hits and resulted in 403 different SCOP hits to DNA transposon struc- tures and 521 SCOP domains that are similar to LINE struc- tures (see supplemental Tables 3 and 4).

  ### Attachments

  - J. Biol. Chem.-2013-Abrusán-16127-38.pdf
- ## Structures of a gamma-aminobutyrate (GABA) transaminase from the s-triazine-degrading organism Arthrobacter aurescens TC1 in complex with PLP and with its external aldimine PLP-GABA adduct

  |  |  |
  | --- | --- |
  | Type | Journal Article |
  | Author | Heather Bruce |
  | Author | Anh Nguyen Tuan |
  | Author | Juan Mangas Sanchez |
  | Author | Charlotte Leese |
  | Author | Jennifer Hopwood |
  | Author | Ralph Hyde |
  | Author | Sam Hart |
  | Author | Johan P. Turkenburg |
  | Author | Gideon Grogan |
  | Volume | 68 |
  | Pages | 1175–1180 |
  | Publication | Acta Crystallographica Section F-structural Biology and Crystallization Communications |
  | Date | October 2012 |
  | DOI | 10.1107/S1744309112030023 |
  | Abstract | Two complex structures of the gamma-aminobutyrate (GABA) transaminase A1R958 from Arthrobacter aurescens TC1 are presented. The first, determined to a resolution of 2.80 angstrom, features the internal aldimine formed by reaction between the epsilon-amino group of Lys295 and the cofactor pyridoxal phosphate (PLP); the second, determined to a resolution of 2.75 angstrom, features the external aldimine adduct formed between PLP and GABA in the first half-reaction. This is the first structure of a microbial GABA transaminase in complex with its natural external aldimine and reveals the molecular determinants of GABA binding in this enzyme. |
  | Date Added | 3/7/2014, 1:06:24 PM |
  | Modified | 3/7/2014, 1:06:24 PM |
- ## Structures of human primase reveal design of nucleotide elongation site and mode of Pol alpha tethering

  |  |  |
  | --- | --- |
  | Type | Journal Article |
  | Author | Mairi Louise Kilkenny |
  | Author | Michael Anthony Longo |
  | Author | Rajika L. Perera |
  | Author | Luca Pellegrini |
  | Volume | 110 |
  | Issue | 40 |
  | Pages | 15961-15966 |
  | Publication | Proceedings of the National Academy of Sciences of the United States of America |
  | ISSN | 0027-8424 |
  | Date | OCT 1 2013 |
  | Extra | WOS:000325105500040 |
  | DOI | 10.1073/pnas.1311185110 |
  | Abstract | Initiation of DNA synthesis in genomic duplication depends on primase, the DNA-dependent RNA polymerase that synthesizes de novo the oligonucleotides that prime DNA replication. Due to the discontinuous nature of DNA replication, primase activity on the lagging strand is required throughout the replication process. In eukaryotic cells, the presence of primase at the replication fork is secured by its physical association with DNA polymerase a (Pol a), which extends the RNA primer with deoxynucleotides. Our knowledge of the mechanism that primes DNA synthesis is very limited, as structural information for the eukaryotic enzyme has proved difficult to obtain. Here, we describe the crystal structure of human primase in heterodimeric form consisting of full-length catalytic subunit and a C-terminally truncated large subunit. We exploit the crystallographic model to define the architecture of its nucleotide elongation site and to show that the small subunit integrates primer initiation and elongation within the same set of functional residues. Furthermore, we define in atomic detail the mode of association of primase to Pol a, the critical interaction that keeps primase tethered to the eukaryotic replisome. |
  | Date Added | 2/12/2014, 1:36:22 PM |
  | Modified | 2/12/2014, 1:36:22 PM |

  ### Notes:

  - Report crystal structures of human primase in unliganded form and bound to UTP.

    How SCOP is used:

    look up superfamily classification of PriS.

    SCOP reference:

    PriS adopts the expected “prim” fold char- acteristic of the prim-pol superfamily (26), fused to a smaller all- helical domain, which confers to PriS a rather flat, slab-like ap- pearance.

  ### Attachments

  - PNAS-2013-Kilkenny-15961-6.pdf
- ## Subcellular localization of extracytoplasmic proteins in monoderm bacteria: rational secretomics-based strategy for genomic and proteomic analyses

  |  |  |
  | --- | --- |
  | Type | Journal Article |
  | Author | Sandra Renier |
  | Author | Pierre Micheau |
  | Author | Régine Talon |
  | Author | Michel Hébraud |
  | Author | Mickaël Desvaux |
  | URL | http://dx.plos.org/10.1371/journal.pone.0042982 |
  | Volume | 7 |
  | Issue | 8 |
  | Pages | e42982 |
  | Publication | PloS one |
  | Date | 2012 |
  | Accessed | 9/20/2013, 1:20:18 PM |
  | Library Catalog | Google Scholar |
  | Short Title | Subcellular localization of extracytoplasmic proteins in monoderm bacteria |
  | Date Added | 10/11/2013, 10:29:15 AM |
  | Modified | 10/11/2013, 10:29:15 AM |

  ### Tags:

  - Bacterial Proteins
  - Bacterial Secretion Systems
  - Cell Wall
  - Computational Biology
  - Decision Trees
  - Genomics
  - Listeria monocytogenes
  - Proteomics
  - Software Design
  - Subcellular Fractions

  ### Notes:

  - Present method for genome-wide prediction of subcellular localization (SCL).  Method is a workflow of existing bioinformatics tools.

    How SCOP is used:

    Search for homologs using SUPERFAMILY on SCOP 1.73.

    SCOP reference:

    Searches against various databases were performed using different tools, namely RPS-BLAST v2.2.19 (Reverse Position- Specific BLAST) [70], HMMER v2.3.2 for hidden Markov models (HMM) [71], InterProScan v4.3 [72], or ScanProsite v1.0 [73]. Interrogated databases included InterPro (IPR) v32.0 [74], Pfam (PF) v24.0 [75], SMART (SM) v6.1 [76], TIGRfam (TIGR) v10.1 [77], SuperFamily (SSF) SCOP v1.73 [78,79], PIRSF v2.74 [80], PRK v3.0 [81], COG v1.0 [82] and Prosite (PS) v20.7 [83].

  ### Attachments

  - journal.pone.0042982.pdf
- ## Subpocket Analysis Method for Fragment-Based Drug Discovery

  |  |  |
  | --- | --- |
  | Type | Journal Article |
  | Author | Tuomo Kalliokoski |
  | Author | Tjelvar SG Olsson |
  | Author | Anna Vulpetti |
  | URL | http://pubs.acs.org/doi/abs/10.1021/ci300523r |
  | Volume | 53 |
  | Issue | 1 |
  | Pages | 131–141 |
  | Publication | Journal of chemical information and modeling |
  | Date | 2013 |
  | Accessed | 9/23/2013, 10:15:21 AM |
  | Library Catalog | Google Scholar |
  | Date Added | 10/11/2013, 10:29:15 AM |
  | Modified | 10/11/2013, 10:29:15 AM |

  ### Notes:

  - Present SubCav method for comparing and aligning subpockets.  Evaluated on a data set of nonredundant PDB complexes that "host identical fragments".

    How SCOP is used:

    1. Integrate SCOP classification, among other annotations (uniprot, Pfam, etc) into method to help facilitate analysis

    2. Illustrate a use case of SubCav that involves searching for structures with similar binding pockets and removing results within the same SCOP superfamily in order to identify "fragment-like bioisoteric replacements" that are not related.

    SCOP reference:

    2.1.4. Additional Protein Annotations. Sequence similarity and protein annotations were used together with SubCav similarity to facilitate the analysis of the results in order to highlight nontrivial matches. The sequence similarity of the proteins was calculated using the Needleman-Wunsch method26 implemented in EMBOSS.27 The default values for opening penalty of 10.0 and extension penalty 0.5 were used.

    Uniprot annotations for the protein structures were extracted from Uniprot Knowledgebase.28 Two commonly structural classifications for proteins were also used (SCOP29,30 family annotation and PFAM31).

    ..

    3.2. Prospective Screen with HSP90 and FRAGPDB. To illustrate the potential of using subpocket searches to identify fragment-like bioisosteric replacements, a prospective screen on

    FRAGPDB was carried out using the adenine portion of ACP bound to HSP90 (PDB code 3t10). The PDB structure 3t10 was recently published and did not form part of the LigandExpo database used to build FRAGPDB. A subpocket query was manually defined with PyMol’s graphical user interface53 to include all protein atoms at a 5 Å distance from any atoms of the adenine fragment. The longer query distance was selected as it is one of the presets in PyMol.

    An Osc value of greater than 0.50 was used to select the most promising cases for further inspection. By using the UNIPROT annotation, all the subpockets corresponding to other HSP90 PDB structures present in FRAGPDB were removed, which were correctly top ranked based on Osc. The subpockets corresponding to proteins with the same SCOP family name (i.e., d.122.1.1) and with a sequence similarity higher than 30% were discarded, and the remaining 1416 subpocket pairs were then manually checked. Some of the retrieved PDBs are highlighted in Table 4. Despite the low sequence similarity other proteins belonging to the ATPase/kinase superfamily were identified, such as the pyruvate and alpha-ketoacid dehydrogenase kinase (entries 1−5 of Table 4) and DNA topoisomerase gyrase B (entry 6) as well as various topoisomerases (entries 7−9), histidine kinases (entries 10− 11), and the DNA repair enzyme MutL-like proteins (entries 9−12). It is worth pointing out that the interactions with the ribose and the phosphates are not conserved among protein histidine kinases and the other ATPases.54 The cytosolic paralog of HSP90 (entry 16) was also found. Good overlap was also observed with the nucleotide-binding site of the anti-σ and serine kinase spoIIAB bound to ADP and to the anti-σ spoIIAA protein (entry 18), with an extremely low sequence similarity of 4.1%.

  ### Attachments

  - ci300523r.pdf
- ## SUPERFAMILY 1.75 including a domain-centric gene ontology method

  |  |  |
  | --- | --- |
  | Type | Journal Article |
  | Author | David A. de Lima Morais |
  | Author | Hai Fang |
  | Author | Owen J. L. Rackham |
  | Author | Derek Wilson |
  | Author | Ralph Pethica |
  | Author | Cyrus Chothia |
  | Author | Julian Gough |
  | Volume | 39 |
  | Issue | Database issue |
  | Pages | D427-434 |
  | Publication | Nucleic Acids Research |
  | ISSN | 1362-4962 |
  | Date | Jan 2011 |
  | Extra | PMID: 21062816 PMCID: PMC3013712 |
  | Journal Abbr | Nucleic Acids Res. |
  | DOI | 10.1093/nar/gkq1130 |
  | Library Catalog | NCBI PubMed |
  | Language | eng |
  | Abstract | The SUPERFAMILY resource provides protein domain assignments at the structural classification of protein (SCOP) superfamily level for over 1400 completely sequenced genomes, over 120 metagenomes and other gene collections such as UniProt. All models and assignments are available to browse and download at http://supfam.org. A new hidden Markov model library based on SCOP 1.75 has been created and a previously ignored class of SCOP, coiled coils, is now included. Our scoring component now uses HMMER3, which is in orders of magnitude faster and produces superior results. A cloud-based pipeline was implemented and is publicly available at Amazon web services elastic computer cloud. The SUPERFAMILY reference tree of life has been improved allowing the user to highlight a chosen superfamily, family or domain architecture on the tree of life. The most significant advance in SUPERFAMILY is that now it contains a domain-based gene ontology (GO) at the superfamily and family levels. A new methodology was developed to ensure a high quality GO annotation. The new methodology is general purpose and has been used to produce domain-based phenotypic ontologies in addition to GO. |
  | Date Added | 10/10/2014, 3:07:45 PM |
  | Modified | 10/10/2014, 3:07:45 PM |

  ### Tags:

  - Databases, Protein
  - Genes
  - Phenotype
  - Phylogeny
  - Proteins
  - Protein Structure, Tertiary
  - Sequence Analysis, Protein
  - Software

  ### Attachments

  - PubMed entry
- ## Supervised Protein Family Classification and New Family Construction

  |  |  |
  | --- | --- |
  | Type | Journal Article |
  | Author | Gangman Yi |
  | Author | Michael R. Thon |
  | Author | Sing-Hoi Sze |
  | URL | http://www.ncbi.nlm.nih.gov/pmc/articles/PMC3415071/ |
  | Volume | 19 |
  | Issue | 8 |
  | Pages | 957-967 |
  | Publication | Journal of Computational Biology |
  | ISSN | 1066-5277 |
  | Date | 2012-8 |
  | Extra | PMID: 22876787 PMCID: PMC3415071 |
  | Journal Abbr | J Comput Biol |
  | DOI | 10.1089/cmb.2011.0044 |
  | Accessed | 11/14/2013, 5:08:53 PM |
  | Library Catalog | PubMed Central |
  | Abstract | The goal of protein family classification is to group proteins into families so that proteins within the same family have common function or are related by ancestry. While supervised classification algorithms are available for this purpose, most of these approaches focus on assigning unclassified proteins to known families but do not allow for progressive construction of new families from proteins that cannot be assigned. Although unsupervised clustering algorithms are also available, they do not make use of information from known families. By computing similarities between proteins based on pairwise sequence comparisons, we develop supervised classification algorithms that achieve improved accuracy over previous approaches while allowing for construction of new families. We show that our algorithm has higher accuracy rate and lower mis-classification rate when compared to algorithms that are based on the use of multiple sequence alignments and hidden Markov models, and our algorithm performs well even on families with very few proteins and on families with low sequence similarity. A software program implementing the algorithm (SClassify) is available online (http://faculty.cse.tamu.edu/shsze/sclassify). |
  | Date Added | 11/14/2013, 5:08:53 PM |
  | Modified | 11/14/2013, 5:09:15 PM |

  ### Tags:

  - algorithms
  - gene clusters
  - protein families

  ### Notes:

  - Present machine-learning methods for family classification and clustering.

     How SCOP is used:

    Use SCOP for training and benchmarking their method for family classification.  Use 10-fold cross validation.  Do not specify how the data set was derived from SCOP.

    SCOP references:

    3.1. Data sets

    We apply our algorithm to a few large-scale data sets, including curated families from the Pfam database (Bateman et al., 2000), protein families from the SCOP database (Murzin et al., 1995), full length pro- karyotic sequences from the ProtClustDB database (Klimke et al., 2009), and curated proteins from the Swiss-Prot subset of the UniProt database (Apweiler et al., 2004). To compare the performance of our algorithm to slower algorithms, we use families within individual species from Pfam, including Arabi- dopsis thaliana, Caenorhabditis elegans, Drosophila melanogaster, Escherichia coli, Homo sapiens, Mus musculus, and Saccharomyces cerevisiae, with the proteins that are within each species forming a data set.

  ### Attachments

  - PubMed Central Full Text PDF
- ## SUPFAM: a database of sequence superfamilies of protein domains

  |  |  |
  | --- | --- |
  | Type | Journal Article |
  | Author | Shashi B. Pandit |
  | Author | Rana Bhadra |
  | Author | V. S. Gowri |
  | Author | S. Balaji |
  | Author | B. Anand |
  | Author | N. Srinivasan |
  | Volume | 5 |
  | Pages | 28 |
  | Publication | BMC bioinformatics |
  | ISSN | 1471-2105 |
  | Date | Mar 15, 2004 |
  | Extra | PMID: 15113407 PMCID: PMC394316 |
  | Journal Abbr | BMC Bioinformatics |
  | DOI | 10.1186/1471-2105-5-28 |
  | Library Catalog | NCBI PubMed |
  | Language | eng |
  | Abstract | BACKGROUND: SUPFAM database is a compilation of superfamily relationships between protein domain families of either known or unknown 3-D structure. In SUPFAM, sequence families from Pfam and structural families from SCOP are associated, using profile matching, to result in sequence superfamilies of known structure. Subsequently all-against-all family profile matches are made to deduce a list of new potential superfamilies of yet unknown structure. DESCRIPTION: The current version of SUPFAM (release 1.4) corresponds to significant enhancements and major developments compared to the earlier and basic version. In the present version we have used RPS-BLAST, which is robust and sensitive, for profile matching. The reliability of connections between protein families is ensured better than before by use of benchmarked criteria involving strict e-value cut-off and a minimal alignment length condition. An e-value based indication of reliability of connections is now presented in the database. Web access to a RPS-BLAST-based tool to associate a query sequence to one of the family profiles in SUPFAM is available with the current release. In terms of the scientific content the present release of SUPFAM is entirely reorganized with the use of 6190 Pfam families and 2317 structural families derived from SCOP. Due to a steep increase in the number of sequence and structural families used in SUPFAM the details of scientific content in the present release are almost entirely complementary to previous basic version. Of the 2286 families, we could relate 245 Pfam families with apparently no structural information to families of known 3-D structures, thus resulting in the identification of new families in the existing superfamilies. Using the profiles of 3904 Pfam families of yet unknown structure, an all-against-all comparison involving sequence-profile match resulted in clustering of 96 Pfam families into 39 new potential superfamilies. CONCLUSION: SUPFAM presents many non-trivial superfamily relationships of sequence families involved in a variety of functions and hence the information content is of interest to a wide scientific community. The grouping of related proteins without a known structure in SUPFAM is useful in identifying priority targets for structural genomics initiatives and in the assignment of putative functions. Database URL: http://pauling.mbu.iisc.ernet.in/~supfam. |
  | Short Title | SUPFAM |
  | Date Added | 11/3/2014, 3:38:38 PM |
  | Modified | 11/3/2014, 3:38:38 PM |

  ### Tags:

  - Amino Acid Sequence
  - Computational Biology
  - Databases, Protein
  - Peptides
  - Proteins
  - Protein Structure, Tertiary

  ### Attachments

  - PubMed entry
- ## Surface-imprinted polymers in microfluidic devices

  |  |  |
  | --- | --- |
  | Type | Journal Article |
  | Author | Schirhagl Romana |
  | Author | Ren KangNing |
  | Author | N. Zare Richard |
  | Volume | 55 |
  | Issue | 4 |
  | Pages | 469-483 |
  | Publication | Science China-Chemistry |
  | ISSN | 1674-7291 |
  | Date | APR 2012 |
  | Extra | WOS:000303184300003 |
  | DOI | 10.1007/s11426-012-4544-7 |
  | Abstract | Molecularly imprinted polymers are generated by curing a cross-linked polymer in the presence of a template. During the curing process, noncovalent bonds form between the polymer and the template. The interaction sites for the noncovalent bonds become "frozen" in the cross-linking polymer and maintain their shape even after the template is removed. The resulting cavities reproduce the size and shape of the template and can selectively reincorporate the template when a mixture containing it flows over the imprinted surface. In the last few decades the field of molecular imprinting has evolved from being able to selectively capture only small molecules to dealing with all kinds of samples. Molecularly imprinted polymers (MIPs) have been generated for analytes as diverse as metal ions, drug molecules, environmental pollutants, proteins and viruses to entire cells. We review here the relatively new field of surface imprinting, which creates imprints of large, biologically relevant templates. The traditional bulk imprinting, where a template is simply added to a prepolymer before curing, cannot be applied if the analyte is too large to diffuse from the cured polymer. Special methods must be used to generate binding sites only on a surface. Those techniques have solved crucial problems in separation science as well as chemical and biochemical sensing. The implementation of imprinted polymers into microfluidic chips has greatly improved the applicability of microfluidics. We present the latest advances and different approaches of surface imprinting and their applications for microfluidic devices. |
  | Date Added | 2/20/2014, 12:24:01 PM |
  | Modified | 2/20/2014, 12:24:01 PM |

  ### Notes:

  - Review of method of molecularly imprinting polymers.

    How SCOP is used:

    Background on protein structure classification.  Say its a hard task.

    SCOP reference:

    The disadvantage of the method is that one needs to know which substructure of an analyte is present on the surface, which is often nontrivial [136–139].

  ### Attachments

  - art%3A10.1007%2Fs11426-012-4544-7.pdf
- ## Surprising similarities in structure comparison

  |  |  |
  | --- | --- |
  | Type | Journal Article |
  | Author | Jean-Francois Gibrat |
  | Author | Thomas Madej |
  | Author | Stephen H. Bryant |
  | URL | http://www.sciencedirect.com/science/article/pii/S0959440X96800583 |
  | Volume | 6 |
  | Issue | 3 |
  | Pages | 377–385 |
  | Publication | Current opinion in structural biology |
  | Date | 1996 |
  | Accessed | 10/10/2013, 1:19:17 PM |
  | Library Catalog | Google Scholar |
  | Date Added | 10/11/2013, 10:29:15 AM |
  | Modified | 10/11/2013, 10:29:15 AM |

  ### Notes:

  - Survey paper discussing the state-of-the art in structure comparison methods as of 1996.

    How SCOP is used:

    Not using SCOP data.

    SCOP reference:

    Listed in a table as a "wonderful resource".

  ### Attachments

  - gibrat-1996.pdf

    These are wonderful resources, well worth a stop on a biologist's tour of the Interned

    In Table 2 we list the World Wide Web addresses of sites that provide access to the results of structural similarity search and classification. These include the results of the new algorithms described here [33••,35"], and results of other comprehensive similarity search and human expert classifications [39,40",59]. A brief description of the information available at each site is provided in Table 2. These are wonderful resources, well worth a stop on a biologist's tour of the Interned
  - Snapshot
- ## Systematic Mutational Analysis of the Putative Hydrolase PqsE: Toward a Deeper Molecular Understanding of Virulence Acquisition in Pseudomonas aeruginosa

  |  |  |
  | --- | --- |
  | Type | Journal Article |
  | Author | Benjamin Folch |
  | Author | Eric Deziel |
  | Author | Nicolas Doucet |
  | Volume | 8 |
  | Issue | 9 |
  | Pages | e73727 |
  | Publication | Plos One |
  | ISSN | 1932-6203 |
  | Date | SEP 10 2013 |
  | Extra | WOS:000327538600040 |
  | DOI | 10.1371/journal.pone.0073727 |
  | Abstract | Pseudomonas aeruginosa is an important opportunistic human pathogen that can establish bacterial communication by synchronizing the behavior of individual cells in a molecular phenomenon known as "quorum sensing''. Through an elusive mechanism involving gene products of the pqs operon, the PqsE enzyme is absolutely required for the synthesis of extracellular phenazines, including the toxic blue pigment pyocyanin, effectively allowing cells to achieve full-fledged virulence. Despite several functional and structural attempts at deciphering the role of this relevant enzymatic drug target, no molecular function has yet been ascribed to PqsE. In the present study, we report a series of alanine scanning experiments aimed at altering the biological function of PqsE, allowing us to uncover key amino acid positions involved in the molecular function of this enzyme. We use sequence analysis and structural overlays with members of homologous folds to pinpoint critical positions located in the vicinity of the ligand binding cleft and surrounding environment, revealing the importance of a unique C-terminal a-helical motif in the molecular function of PqsE. Our results suggest that the active site of the enzyme involves residues that extend further into the hydrophobic core of the protein, advocating for a lid-like movement of the two terminal helices. This information should help design virtual libraries of PqsE inhibitors, providing means to counter P. aeruginosa virulence acquisition and helping to reduce nosocomial infections. |
  | Date Added | 2/12/2014, 1:36:22 PM |
  | Modified | 2/12/2014, 1:36:22 PM |

  ### Notes:

  - Computational study of function of PqsE.

    How SCOP is used:

    Retrieve all structures from the same fold as PqsE, and perform a structural alignment to compare their structures.

    SCOP reference:

    We know from the PqsE structure [20] and the SCOP database [27] that PqsE belongs to the metallo-hydrolase/oxidoreductase superfamily, a functional enzyme clan encompassing at least 13 interspecies protein families playing various biological functions.

    ...

    Identification of two unique structural motifs in PqsE

    PqsE adopts a very common three-dimensional structure shared by many proteins of the metallo-b-lactamase fold [29]. We retrieved all proteins adopting such a fold from the SCOP database [27], as well as members of known structure from b- lactamase families 2, B, B2, B3, and B4 (395 hits in the Pfam database [30]). The pairwise superposition of each member with PqsE was analyzed and the strong conservation among structural homologues allowed the unambiguous identification of the active- site cavity (Figure 2A). Two structural elements that differentiate PqsE from other members of this protein fold could be identified from this pairwise superposition.

  ### Attachments

  - journal.pone.0073727.pdf
- ## Tagaturonate-fructuronate epimerase UxaE, a novel enzyme in the hexuronate catabolic network in Thermotoga maritima

  |  |  |
  | --- | --- |
  | Type | Journal Article |
  | Author | Irina A. Rodionova |
  | Author | David A. Scott |
  | Author | Nick V. Grishin |
  | Author | Andrei L. Osterman |
  | Author | Dmitry A. Rodionov |
  | Volume | 14 |
  | Issue | 11 |
  | Pages | 2920-2934 |
  | Publication | ENVIRONMENTAL MICROBIOLOGY |
  | ISSN | 1462-2912 |
  | Date | November 2012 |
  | DOI | 10.1111/j.1462-2920.2012.02856.x |
  | Language | English |
  | Abstract | Thermotoga maritima is a marine hyperthermophilic microorganism that degrades a wide range of simple and complex carbohydrates including pectin and produces fermentative hydrogen at high yield. Galacturonate and glucuronate, two abundant hexuronic acids in pectin and xylan, respectively, are catabolized via committed metabolic pathways to supply carbon and energy for a variety of microorganisms. By a combination of bioinformatics and experimental techniques we identified a novel enzyme family (named UxaE) catalysing a previously unknown reaction in the hexuronic acid catabolic pathway, epimerization of tagaturonate to fructuronate. The enzymatic activity of the purified recombinant tagaturonate epimerase from T. maritima was directly confirmed and kinetically characterized. Its function was also confirmed by genetic complementation of the growth of the Escherichia coli uxaB knockout mutant strain on galacturonate. An inferred novel galacturonate to mannonate catabolic pathway in T. maritima was reconstituted in vitro using a mixture of recombinant purified enzymes UxaE, UxaC and UxuB. Members of the newly identified UxaE family were identified in similar to 50 phylogenetically diverse heterotrophic bacteria from aquatic and soil environments. The genomic context of respective genes and reconstruction of associated pathways suggest that UxaE enzymatic and biological function remains conserved in all of these species. |
  | Date Added | 10/11/2013, 10:29:15 AM |
  | Modified | 10/11/2013, 10:29:15 AM |

  ### Notes:

  - Identify a novel enzyme family (UxaE) involved in catalyzing a reaction in the hexuronic acid catabolic pathway.  Computational and experimental study.

    How SCOP is used:

    Classify their UxaE proteins in SCOP using HHPred.  Found hits to proteins in the aldolase superfamily in the   
    TIM b/a barrel fold.

    SCOP reference:

    We used the HHpred server to search for distant homo- logues of T. maritima UxaE among proteins with experi- mentally determined spatial structures (pdb70\_17Mar12 database). The first hit, with probability above 99%, was to the tagatose-bisphosphate aldolase AgaY from E. coli (pdb entry 1gvf). According to structural classification of proteins in the SCOP database, AgaY belongs to the aldolase superfamily in the TIM b/a barrel fold. Moreover, all hits with probability above 95% are class II aldolases (e.g. fructose-bisphosphate aldolase), and all hits above 70% probability are TIM barrels (e.g. arginine decarboxy- lase). This consistency of the results combined with a very high score of the first hit and conservation of metal ligands in the sequence alignment (Fig. S1) strongly suggests that UxaE is homologous to class II aldolases and adopts a TIM barrel structure.

  ### Attachments

  - emi2856.pdf
- ## Template-based protein structure modeling using the RaptorX web server

  |  |  |
  | --- | --- |
  | Type | Journal Article |
  | Author | Morten Källberg |
  | Author | Haipeng Wang |
  | Author | Sheng Wang |
  | Author | Jian Peng |
  | Author | Zhiyong Wang |
  | Author | Hui Lu |
  | Author | Jinbo Xu |
  | URL | http://www.nature.com/nprot/journal/v7/n8/full/nprot.2012.085.html%3FWT.mc\_id%3DTWT\_NatureProtocols |
  | Volume | 7 |
  | Issue | 8 |
  | Pages | 1511–1522 |
  | Publication | Nature Protocols |
  | Date | 2012 |
  | Accessed | 9/23/2013, 10:14:00 AM |
  | Library Catalog | Google Scholar |
  | Date Added | 10/11/2013, 10:29:15 AM |
  | Modified | 10/11/2013, 10:29:15 AM |

  ### Notes:

  - Present RaptorX protein homology modeling web server.

    How SCOP is used:

    Database provides statistics on SCOP classifications of top-ranked templates.

    SCOP reference:

    Function annotation of structure models. Similarity in the fold of two proteins may indicate the existence of an evolutionary relation- ship, which in turn may imply a shared functional role. The Structural Classification of Proteins (SCOP) database provides a description of the structural and evolutionary relation of most proteins in the PDB45,46. **Whenever a structure model is constructed, RaptorX pro- vides a distribution statistic of the ‘class’, ‘fold’, ‘super-family’, ‘family, and ‘protein type’ from some or all of ten top-ranked templates as identified in the SCOP database version 1.75, with each template contribution weighted by its predicted alignment quality (normalized among the ten structures).** Only the templates with a predicted align- ment quality of at least 85% of the highest predicted quality are used, as in most cases the predicted alignment quality error is less than 15%. The SCOP distribution of high-ranked templates, in addition to the 3D model of the target sequence, will give the user an initial feel for the nature of the protein being modeled and thus provide a starting point for further exploration of the structure in question.

  ### Attachments

  - nprot.2012.pdf
- ## Tertiary Structure-Function Analysis Reveals the Pathogenic Signaling Potentiation Mechanism of Helicobacter pylori Oncogenic Effector CagA

  |  |  |
  | --- | --- |
  | Type | Journal Article |
  | Author | Takeru Hayashi |
  | Author | Miki Senda |
  | Author | Hiroko Morohashi |
  | Author | Hideaki Higashi |
  | Author | Masafumi Horio |
  | Author | Yui Kashiba |
  | Author | Lisa Nagase |
  | Author | Daisuke Sasaya |
  | Author | Tomohiro Shimizu |
  | Author | Nagarajan Venugopalan |
  | Author | Hiroyuki Kumeta |
  | Author | Nobuo N. Noda |
  | Author | Fuyuhiko Inagaki |
  | Author | Toshiya Senda |
  | Author | Masanori Hatakeyama |
  | Volume | 12 |
  | Issue | 1 |
  | Pages | 20–33 |
  | Publication | Cell Host & Microbe |
  | Date | July 2012 |
  | DOI | 10.1016/j.chom.2012.05.010 |
  | Abstract | The Helicobacter pylori type IV secretion effector CagA is a major bacterial virulence determinant and critical for gastric carcinogenesis. Upon delivery into gastric epithelial cells, CagA localizes to the inner face of the plasma membrane, where it acts as a pathogenic scaffold/hub that promiscuously recruits host proteins to potentiate oncogenic signaling. We find that CagA comprises a structured N-terminal region and an intrinsically disordered C-terminal region that directs versatile protein interactions. X-ray crystallographic analysis of the N-terminal CagA fragment (residues 1-876) revealed that the region has a structure comprised of three discrete domains. Domain I constitutes a mobile CagA N terminus, while Domain II tethers CagA to the plasma membrane by interacting with membrane phosphatidylserine. Domain III interacts intramolecularly with the intrinsically disordered C-terminal region, and this interaction potentiates the pathogenic scaffold/hub function of CagA. The present work provides a tertiary-structural basis for the pathophysiological/oncogenic action of H. pylori CagA. |
  | Date Added | 3/7/2014, 1:06:24 PM |
  | Modified | 3/7/2014, 1:06:24 PM |
- ## Text mining improves prediction of protein functional sites

  |  |  |
  | --- | --- |
  | Type | Journal Article |
  | Author | Karin M Verspoor |
  | Author | Judith D Cohn |
  | Author | Komandur E Ravikumar |
  | Author | Michael E Wall |
  | Volume | 7 |
  | Issue | 2 |
  | Pages | e32171 |
  | Publication | PloS one |
  | ISSN | 1932-6203 |
  | Date | 2012 |
  | Extra | PMID: 22393388 |
  | Journal Abbr | PLoS ONE |
  | DOI | 10.1371/journal.pone.0032171 |
  | Library Catalog | NCBI PubMed |
  | Language | eng |
  | Abstract | We present an approach that integrates protein structure analysis and text mining for protein functional site prediction, called LEAP-FS (Literature Enhanced Automated Prediction of Functional Sites). The structure analysis was carried out using Dynamics Perturbation Analysis (DPA), which predicts functional sites at control points where interactions greatly perturb protein vibrations. The text mining extracts mentions of residues in the literature, and predicts that residues mentioned are functionally important. We assessed the significance of each of these methods by analyzing their performance in finding known functional sites (specifically, small-molecule binding sites and catalytic sites) in about 100,000 publicly available protein structures. The DPA predictions recapitulated many of the functional site annotations and preferentially recovered binding sites annotated as biologically relevant vs. those annotated as potentially spurious. The text-based predictions were also substantially supported by the functional site annotations: compared to other residues, residues mentioned in text were roughly six times more likely to be found in a functional site. The overlap of predictions with annotations improved when the text-based and structure-based methods agreed. Our analysis also yielded new high-quality predictions of many functional site residues that were not catalogued in the curated data sources we inspected. We conclude that both DPA and text mining independently provide valuable high-throughput protein functional site predictions, and that integrating the two methods using LEAP-FS further improves the quality of these predictions. |
  | Date Added | 10/11/2013, 10:29:15 AM |
  | Modified | 10/11/2013, 10:29:15 AM |

  ### Tags:

  - Animals
  - Binding Sites
  - Catalytic Domain
  - Computational Biology
  - Crystallography, X-Ray
  - Databases, Protein
  - Data Mining
  - Humans
  - Models, Molecular
  - Models, Statistical
  - Molecular Conformation
  - Proteins
  - Protein Structure, Tertiary
  - Sequence Analysis, Protein
  - Software

  ### Notes:

  - Present Literature Enhanced Automated Prediction of Functional Sites (LEAP-FS)  method for protein functional site prediction that integrates protein structure analysis and text mining.  Structure analysis was carried out using Dynamics Perturbation Analysis (DPA), which predicts functional sites.

    How SCOP is used:

    Data set is comprised of all domains from SCOP 1.75.

    Apply three approaches for functional site prediction:

    1. database-based approach: use MSAs of structures from the same SCOP protein-level nodes to transfer functional site annotations to aligned residues.

    2. biophysical structure-based approach

    3. text-mining-based approach.

    Methods 2 and 3 were validated against 1.

    For database-based approach, performed "transitive function annotation".  Used SCOP protein and family-level nodes to transfer functions in order to create a data set

    SCOP reference:

    Fast DPA enabled a typical protein domain to be analyzed in less than a minute using a single core of a desktop computer, bringing analysis of all ,100,000 protein domains in version 1.75 of the SCOP database [13] within easy reach. Our preliminary application of DPA to ,50,000 domains in an earlier version of SCOP confirmed the feasibility of this task [14].

    ...

    Here we demonstrate the integration of high-throughput structure-based and text-based functional site predictions. We applied DPA to a comprehensive set of ,100,000 domains in the SCOP database. We found the predictions recapitulated much of the information about functional sites in the databases, but many of the predictions were left unvalidated.

    ...

    To perform text-based predictions, we compiled a corpus C from a set of MEDLINE abstracts linked to the domains in S (Methods). In all we retrieved 17,595 abstracts representing primary references for 30,816 PDB entries, covering 88,707 SCOP domains.

    ...

    Protein and family annotations. Multiple sequence alignments (MSAs) were used to transfer NSM, NSM-valid, and CSA annotations to residues at equivalent positions across SCOP families (Methods). Two types of transfers were performed, differing in their scope. Highly conservative protein level transfers were performed just between domains that correspond to the same protein. Family level transfers were performed between any two domains within the same SCOP family. The MSAs were also used to compute residue conservation scores, e.g., for identifying when annotations and predictions were associated with highly conserved residues (Methods).

    ...

    Multiple sequence alignments for annotation transfer

    For each of the 3,462 SCOP families containing at least two domains (covering 101,545 of the domains in S), we performed one or two levels of multiple sequence alignment (MSA) using MUSCLE version 3.7 with default settings [53]. The first MSA level was based on assigning each domain in a SCOP family a unique protein id based on database references from the struct\_ref category of the mmCIF data. In order of preference, a domain was assigned the Uniprot entry name, Genbank id or PDB id of the mmCIF entity associated with the domain. For each of 3,300 families, we were able to perform a protein-level MSA for each protein ID with more than one domain in the family. These alignments, covering 96,137 domains, included all domains within a family associated with a particular protein ID. The second level of MSA was performed on a non-redundant set of domains within each family with at least two distinct protein IDs. This was accomplished by selecting the longest (or the first in SID alphabetical order if there was a tie) domain for each unique protein ID within a family. 2,320 families included at least two distinct protein sequence IDs, covering 87,057 domains in S. By merging the protein-level and the non-redundant family-level MSAs, we were able to generate a virtual alignment of all domains in the family by transferring family-level alignment positions from the non- redundant domains to their cohorts in the appropriate protein-level MSA.

    ...

    Dynamics Perturbation Analysis (DPA) of SCOP domains

    Fast DPA [8] was performed on the subset SX of S, which consisted of 98,934 SCOP domains determined by X-ray crystallography and containing a single chain identifier. Given an input PDB structure, MSMS [54] was run with a 1.5 A ̊ probe radius and a triangulation density of 1 vertex per A ̊ 2 to generate test points on the surface of the protein. The cutoff rc for interactions between protein Ca atoms was 10.5 A ̊ . The cutoff rs for interactions between a test point and the protein was 15.5 A ̊ , and the interaction strength between a test point and protein atoms was cs = 12c, or 12 times the strength of the interaction between two protein atoms. To predict functional sites, the

  ### Attachments

  - [HTML] from plos.org
  - journal.pone.0032171.pdf
  - PubMed entry
- ## TFinDit: transcription factor-DNA interaction data depository

  |  |  |
  | --- | --- |
  | Type | Journal Article |
  | Author | Daniel Turner |
  | Author | RyangGuk Kim |
  | Author | Jun-tao Guo |
  | URL | http://www.biomedcentral.com/1471-2105/13/220/ |
  | Volume | 13 |
  | Issue | 1 |
  | Pages | 220 |
  | Publication | BMC bioinformatics |
  | Date | 2012 |
  | Accessed | 9/23/2013, 10:14:50 AM |
  | Library Catalog | Google Scholar |
  | Short Title | TFinDit |
  | Date Added | 10/11/2013, 10:29:15 AM |
  | Modified | 3/7/2014, 12:10:43 PM |

  ### Tags:

  - Binding site prediction
  - database
  - Interaction potential
  - transcription factor

  ### Notes:

  - TFinDit is a specialized structural database with annotated transcription factor-DNA complex structures and other related data.

    How SCOP/CATH is used:

    Annotate structures in data set with SCOP classifications (sccs) and CATH classification.  Provide links to databases.

    SCOP/CATH reference:

    In addition, links of the TFinDit entry to other useful web services are also provided (Red Box in Figure 3). These include PDB [2], WebPDA [23], PDIdb [27], 3D-footprint [26], BIPA [24], NDB [43], and NPIDB [22] and to structural classifications websites CATH [44] and SCOP [45].

  ### Attachments

  - 1471-2105-13-220.pdf
  - [HTML] from biomedcentral.com
- ## TGM2 and implications for human disease: role of alternative splicing.

  |  |  |
  | --- | --- |
  | Type | Journal Article |
  | Author | Thung-S. Lai |
  | Author | Charles S. Greenberg |
  | URL | http://www.researchgate.net/publication/234019335\_TGM2\_and\_implications\_for\_human\_disease\_role\_of\_alternative\_splicing\_/file/3deec51524a7f5363a.pdf |
  | Volume | 18 |
  | Pages | 504–519 |
  | Publication | Frontiers in bioscience: a journal and virtual library |
  | Date | 2012 |
  | Accessed | 9/23/2013, 10:14:00 AM |
  | Library Catalog | Google Scholar |
  | Short Title | TGM2 and implications for human disease |
  | Date Added | 10/11/2013, 10:29:15 AM |
  | Modified | 10/11/2013, 10:29:15 AM |

  ### Tags:

  - Alternative Splicing
  - Cancer
  - Interesting
  - Neurodegenerative
  - Review
  - Transglutaminase
  - Wound Healing

  ### Notes:

  - The paper is a review of studies on Tissue transglutaminase (TGM2), which is an enzyme that plays a role in alternative splicing. The study examines its biochemistry, structure and function, biology, and alternative spliced forms to see its role "in cancer, neurodegeneration, inflammation and wound healing."

    How SCOP is used:

    SCOP data was used to note which families had a similar protein structure of TGM2. (Domain structure and family data used - probably looked it up on the website). It's noted specifically under TGM2's role in cell adhesion (by noting that its structure is similar to a family of adhesive proteins).

    SCOP Referenc**:**

    TGM2 also functions as an adhesion molecule  
    that contributes to cell-cell and cell-ECM interactions (2).  
    The folding of the N-terminal β-sandwich and two C terminal  
    β-barrel domains of TGM2 is similar to the  
    immunoglobulin like (IgF)-folding domain, a major family  
    of adhesive proteins that are predicted to be involved in  
    protein-protein interaction (146, 147).

  ### Attachments

  - [PDF] from researchgate.net
- ## The ASTRAL compendium for protein structure and sequence analysis

  |  |  |
  | --- | --- |
  | Type | Journal Article |
  | Author | S. E. Brenner |
  | Author | P. Koehl |
  | Author | M. Levitt |
  | Volume | 28 |
  | Issue | 1 |
  | Pages | 254-256 |
  | Publication | Nucleic Acids Research |
  | ISSN | 0305-1048 |
  | Date | Jan 1, 2000 |
  | Extra | PMID: 10592239 PMCID: PMC102434 |
  | Journal Abbr | Nucleic Acids Res. |
  | Library Catalog | NCBI PubMed |
  | Language | eng |
  | Abstract | The ASTRAL compendium provides several databases and tools to aid in the analysis of protein structures, particularly through the use of their sequences. The SPACI scores included in the system summarize the overall characteristics of a protein structure. A structural alignments database indicates residue equivalencies in superimposed protein domain structures. The PDB sequence-map files provide a linkage between the amino acid sequence of the molecule studied (SEQRES records in a database entry) and the sequence of the atoms experimentally observed in the structure (ATOM records). These maps are combined with information in the SCOPdatabase to provide sequences of protein domains. Selected subsets of the domain database, with varying degrees of similarity measured in several different ways, are also available. ASTRALmay be accessed at http://astral.stanford.edu/ |
  | Date Added | 11/3/2014, 3:20:05 PM |
  | Modified | 11/3/2014, 3:20:05 PM |

  ### Tags:

  - Amino Acid Sequence
  - Database Management Systems
  - Databases, Factual
  - Protein Conformation
  - Sequence Homology, Amino Acid

  ### Attachments

  - PubMed entry
- ## The ASTRAL Compendium in 2004

  |  |  |
  | --- | --- |
  | Type | Journal Article |
  | Author | John-Marc Chandonia |
  | Author | Gary Hon |
  | Author | Nigel S. Walker |
  | Author | Loredana Lo Conte |
  | Author | Patrice Koehl |
  | Author | Michael Levitt |
  | Author | Steven E. Brenner |
  | Volume | 32 |
  | Issue | Database issue |
  | Pages | D189-192 |
  | Publication | Nucleic Acids Research |
  | ISSN | 1362-4962 |
  | Date | Jan 1, 2004 |
  | Extra | PMID: 14681391 PMCID: PMC308768 |
  | Journal Abbr | Nucleic Acids Res. |
  | DOI | 10.1093/nar/gkh034 |
  | Library Catalog | NCBI PubMed |
  | Language | eng |
  | Abstract | The ASTRAL Compendium provides several databases and tools to aid in the analysis of protein structures, particularly through the use of their sequences. Partially derived from the SCOP database of protein structure domains, it includes sequences for each domain and other resources useful for studying these sequences and domain structures. The current release of ASTRAL contains 54,745 domains, more than three times as many as the initial release 4 years ago. ASTRAL has undergone major transformations in the past 2 years. In addition to several complete updates each year, ASTRAL is now updated on a weekly basis with preliminary classifications of domains from newly released PDB structures. These classifications are available as a stand-alone database, as well as integrated into other ASTRAL databases such as representative subsets. To enhance the utility of ASTRAL to structural biologists, all SCOP domains are now made available as PDB-style coordinate files as well as sequences. In addition to sequences and representative subsets based on SCOP domains, sequences and subsets based on PDB chains are newly included in ASTRAL. Several search tools have been added to ASTRAL to facilitate retrieval of data by individual users and automated methods. ASTRAL may be accessed at http://astral.stanford. edu/. |
  | Date Added | 11/3/2014, 3:20:14 PM |
  | Modified | 11/3/2014, 3:20:14 PM |

  ### Tags:

  - Animals
  - Computational Biology
  - Databases, Protein
  - Humans
  - Information Storage and Retrieval
  - Internet
  - Proteins
  - Protein Structure, Tertiary
  - Software

  ### Attachments

  - PubMed entry
- ## The challenge of increasing Pfam coverage of the human proteome

  |  |  |
  | --- | --- |
  | Type | Journal Article |
  | Author | Jaina Mistry |
  | Author | Penny Coggill |
  | Author | Ruth Y. Eberhardt |
  | Author | Antonio Deiana |
  | Author | Andrea Giansanti |
  | Author | Robert D. Finn |
  | Author | Alex Bateman |
  | Author | Marco Punta |
  | URL | http://www.ncbi.nlm.nih.gov/pmc/articles/PMC3630804/ |
  | Volume | 2013 |
  | Publication | Database: the journal of biological databases and curation |
  | Date | 2013 |
  | Accessed | 9/20/2013, 10:46:43 AM |
  | Library Catalog | Google Scholar |
  | Date Added | 2/20/2014, 12:24:01 PM |
  | Modified | 2/20/2014, 12:24:01 PM |

  ### Notes:

  - Computational study of Pfam coverage of human proteome.

    How SCOP is used:

    Use SUPERFAMILY to annotate SCOP domains, in order to determine how much of human proteome is classified into SCOP.

    SCOP reference:

    Coverage of the human proteome by structure-based protein family databases

    We looked at coverage of our set of human UniProtKB/ Swiss-Prot proteins by two structure-based protein family databases: SUPERFAMILY (19) and Gene3D (20) (Methods). These databases build their families starting from experi- mentally determined structural domains. SUPERFAMILY is based on the SCOP classification (21), and Gene3D is based on the CATH classification (22). SUPERFAMILY had a human sequence coverage of 72% and residue coverage of 41%, while Gene3D covered 69% of human sequences and 35% of human residues. This can be compared with 90% sequence and 45% residue coverage achieved by Pfam-A families. Interestingly, both SUPERFAMILY and Gene3D matched part of the 38% uncovered human resi- dues discussed above. SUPERFAMILY covered 18% of the 38% or an additional 7% of the entire proteome, while Gene3D covered 15% of the 38% or an additional 6% of the entire proteome.

  ### Attachments

  - bat023.pdf
  - [HTML] from nih.gov
- ## The chitinolytic machinery of Serratiamarcescens - a model system for enzymatic degradation of recalcitrant polysaccharides

  |  |  |
  | --- | --- |
  | Type | Journal Article |
  | Author | Gustav Vaaje-Kolstad |
  | Author | Svein J. Horn |
  | Author | Morten Sorlie |
  | Author | Vincent G. H. Eijsink |
  | Volume | 280 |
  | Issue | 13 |
  | Pages | 3028-3049 |
  | Publication | Febs Journal |
  | ISSN | 1742-464X |
  | Date | JUL 2013 |
  | Extra | WOS:000320557100007 |
  | DOI | 10.1111/febs.12181 |
  | Abstract | The chitinolytic machinery of Serratiamarcescens is one of the best known enzyme systems for the conversion of insoluble polysaccharides. This machinery includes four chitin-active enzymes: ChiC, an endo-acting non-processive chitinase; ChiA and ChiB, two processive chitinases moving along chitin chains in opposite directions; and CBP21, a surface-active CBM33-type lytic polysaccharide monooxygenase that introduces chain breaks by oxidative cleavage. Furthermore, an N-acetylhexosaminidase or chitobiase converts the oligomeric products from the other enzymes to monomeric N-acetylglucosamine. Here we discuss the catalytic mechanisms of these enzymes as well as the structural basis of each enzyme's specific role in the chitin degradation process. We also discuss how knowledge of this enzyme system may be extrapolated to other enzyme systems for conversion of insoluble polysaccharides, in particular conversion of cellulose by cellulases and GH61-type lytic polysaccharide monooxygenases. |
  | Date Added | 2/13/2014, 4:13:17 PM |
  | Modified | 3/7/2014, 12:08:43 PM |
- ## The coevolutionary roots of biochemistry and cellular organization challenge the RNA world paradigm

  |  |  |
  | --- | --- |
  | Type | Journal Article |
  | Author | Gustavo Caetano-Anollés |
  | Author | Manfredo J. Seufferheld |
  | URL | http://www.karger.com/Article/FullText/346551 |
  | Volume | 23 |
  | Issue | 1-2 |
  | Pages | 152–177 |
  | Publication | Journal of molecular microbiology and biotechnology |
  | Date | 2013 |
  | Accessed | 9/20/2013, 1:16:59 PM |
  | Library Catalog | Google Scholar |
  | Date Added | 10/11/2013, 10:29:15 AM |
  | Modified | 3/7/2014, 12:10:54 PM |

  ### Tags:

  - Acidocalcisome organelles
  - Coevolution
  - Origin of life
  - P-loop hydrolases
  - protein structure
  - Pyrophosphate
  - Ribonucleoprotein world
  - RNA world
  - Translation

  ### Notes:

  - Goal is to lay a framework to unravel origins and emergence of molecular and cellular structure with knowledge from structural, functional and evolutionary genomics.

    How SCOP is used:

    Use domain data from SCOP, and SUPERFAMILY, and SCOP family classification, to build phylogenetic trees.  Examine trees to study emergence of various functions.

    SCOP reference:

    Ele- ments of the matrix (g) represent genomic abundances of domains in proteomes, defined at different levels of classification of domain structure (e.g. SCOP F, FSF and FF). 

    ...

    The tree was reconstructed from a census of domain abundance in proteomes using SCOP 1.67 definitions [Wang et al., 2006]. The panel was modified from Caetano-Anollés and Wang [2008].

  ### Attachments

  - 346551.pdf
  - Snapshot

    Abstract

    ### Abstract

    The origin and evolution of modern biochemistry and cellular structure is a complex problem that has puzzled scientists for almost a century. While comparative, functional and structural genomics has unraveled considerable complexity at the molecular level, there is very little understanding of the origin, evolution and structure of the molecules responsible for cellular or viral features in life. Recent efforts, however, have dissected the emergence of the very early molecules that populated primordial cells. Deep historical signal was retrieved from a census of molecular structures and functions in thousands of nucleic acid and protein structures and hundreds of genomes using powerful phylogenomic methods. Together with structural, chemical and cell biology considerations, this information reveals that modern biochemistry is the result of the gradual evolutionary appearance and accretion of molecular parts and molecules. These patterns comply with the principle of continuity and lead to molecular and cellular complexity. Here, we review findings and report possible origins of molecular and cellular structure, the early rise of lipid biosynthetic pathways and components of cytoskeletal microstructures, the piecemeal accumulation of domains in ATP synthase complexes and the origin and evolution of the ribosome. Phylogenomic studies suggest the last universal common ancestor of life, the 'urancestor', had already developed complex cellular structure and bioenergetics. Remarkably, our findings falsify the existence of an ancient RNA world. Instead they are compatible with gradually coevolving nucleic acids and proteins in interaction with increasingly complex cofactors, lipid membrane structures and other cellular components. This changes the perception we have of the rise of modern biochemistry and prompts further analysis of the emergence of biological complexity in an ever-expanding coevolving world of macromolecules.
- ## The crystal structure of sterol carrier protein 2 from Yarrowia lipolytica and the evolutionary conservation of a large, non-specific lipid-binding cavity

  |  |  |
  | --- | --- |
  | Type | Journal Article |
  | Author | Federico Perez De Berti |
  | Author | Stefano Capaldi |
  | Author | Raul Ferreyra |
  | Author | Noelia Burgardt |
  | Author | Juan P. Acierno |
  | Author | Sebastian Klinke |
  | Author | Hugo L. Monaco |
  | Author | Mario R. Ermacora |
  | Volume | 14 |
  | Issue | 4 |
  | Pages | 145-153 |
  | Publication | Journal of Structural and Functional Genomics |
  | ISSN | 1345-711X; 1570-0267 |
  | Date | DEC 2013 |
  | Extra | BCI:BCI201400050835 |
  | DOI | 10.1007/s10969-013-9166-6 |
  | Abstract | Sterol carrier protein 2 (SCP2), a small intracellular domain present in all forms of life, binds with high affinity a broad spectrum of lipids. Due to its involvement in the metabolism of long-chain fatty acids and cholesterol uptake, it has been the focus of intense research in mammals and insects; much less characterized are SCP2 from other eukaryotic cells and microorganisms. We report here the X-ray structure of Yarrowia lipolytica SCP2 (YLSCP2) at 2.2 resolution in complex with palmitic acid. This is the first fungal SCP2 structure solved, and it consists of the canonical five-stranded beta-sheet covered on the internal face by a layer of five alpha-helices. The overall fold is conserved among the SCP2 family, however, YLSCP2 is most similar to the SCP2 domain of human MFE-2, a bifunctional enzyme acting on peroxisomal beta-oxidation. We have identified the common structural elements defining the shape and volume of the large binding cavity in all species characterized. Moreover, we found that the cavity of the SCP2 domains is distinctly formed by carbon atoms, containing neither organized water nor rigid polar interactions with the ligand. These features are in contrast with those of fatty acid binding proteins, whose internal cavities are more polar and contain bound water. The results will help to design experiments to unveil the SCP2 function in very different cellular contexts and metabolic conditions. |
  | Date Added | 2/12/2014, 1:36:22 PM |
  | Modified | 2/12/2014, 1:36:22 PM |

  ### Notes:

  - Present crystal structure of SCP2 protein.

    How SCOP is used:

    Classify the newly solved crystal structure into class, fold, and superfamily.

    SCOP reference:

    YLSCP2 fold

    YLSCP2 domain belongs to the a?b class and to the SCP-like fold (SCOP 1.75 release; [31]). The fold con- sists of a five-stranded b-sheet covered on the internal face by a layer of five a-helices. The external, solvent- exposed face is traversed by a crossover loop (Fig. 2). The b-sheet exhibits strand order 32,145, with all strands antiparallel except 1 and 4. The a-helices and the internal face of the b-sheet form a large cavity where the ligand binds.

    Based on sequence and structure similarity, YLSCP2 belongs to the SCP super family, one of the four super families with the SCP-like fold (Fig. 3). The structural superposition of characterized members of this superfamily indicates that the closest relative of YLSCP2 is the human MFE-2 SCP2 domain, the C-terminal domain of a complex bifunctional enzyme acting on the peroxisomal b-oxidation pathway for fatty acids. The backbones of YLSCP2 and MFE-2 SCP2 domains can be superposed with 0.94 A ̊ RMSD (87 residues out of 128). However, and despite a low sequential identity, the fold of all members of the superfamily is well preserved from bacteria to mammals (Table 2).

  ### Attachments

  - art%3A10.1007%2Fs10969-013-9166-6.pdf
- ## The dehaloperoxidase paradox

  |  |  |
  | --- | --- |
  | Type | Journal Article |
  | Author | Stefan Franzen |
  | Author | Matthew K. Thompson |
  | Author | Reza A. Ghiladi |
  | URL | http://www.sciencedirect.com/science/article/pii/S1570963911003256 |
  | Volume | 1824 |
  | Issue | 4 |
  | Pages | 578–588 |
  | Publication | Biochimica et Biophysica Acta (BBA)-Proteins and Proteomics |
  | Date | 2012 |
  | Accessed | 9/23/2013, 10:14:36 AM |
  | Library Catalog | Google Scholar |
  | Date Added | 2/20/2014, 12:24:01 PM |
  | Modified | 2/20/2014, 12:24:01 PM |

  ### Notes:

  - Review of studies of dehaloperoxidase-hemoglobin.

    How SCOP is used:

    Look up family-level classification of a DHP protein.

    SCOP reference:

    However, despite DHP being categorized as a globin according to the Structural Classification of Proteins (SCOP) database [18], DHP has little sequence homology to other known Hbs.

  ### Attachments

  - [PDF] from ncsu.edu
  - Snapshot
- ## The distribution of ligand-binding pockets around protein-protein interfaces suggests a general mechanism for pocket formation

  |  |  |
  | --- | --- |
  | Type | Journal Article |
  | Author | Mu Gao |
  | Author | Jeffrey Skolnick |
  | Volume | 109 |
  | Issue | 10 |
  | Pages | 3784-3789 |
  | Publication | Proceedings of the National Academy of Sciences of the United States of America |
  | ISSN | 0027-8424 |
  | Date | MAR 6 2012 |
  | Extra | WOS:000301117700041 |
  | DOI | 10.1073/pnas.1117768109 |
  | Abstract | Protein-protein and protein-ligand interactions are ubiquitous in a biological cell. Here, we report a comprehensive study of the distribution of protein-ligand interaction sites, namely ligand-binding pockets, around protein-protein interfaces where protein-protein interactions occur. We inspected a representative set of 1,611 representative protein-protein complexes and identified pockets with a potential for binding small molecule ligands. The majority of these pockets are within a 6 angstrom distance from protein interfaces. Accordingly, in about half of ligand-bound protein-protein complexes, amino acids from both sides of a protein interface are involved in direct contacts with at least one ligand. Statistically, ligands are closer to a protein-protein interface than a random surface patch of the same solvent accessible surface area. Similar results are obtained in an analysis of the ligand distribution around domain-domain interfaces of 1,416 nonredundant, two-domain protein structures. Furthermore, comparable sized pockets as observed in experimental structures are present in artificially generated protein complexes, suggesting that the prominent appearance of pockets around protein interfaces is mainly a structural consequence of protein packing and thus, is an intrinsic geometric feature of protein structure. Nature may take advantage of such a structural feature by selecting and further optimizing for biological function. We propose that packing nearby protein-protein or domain-domain interfaces is a major route to the formation of ligand-binding pockets. |
  | Date Added | 2/13/2014, 4:13:17 PM |
  | Modified | 3/7/2014, 12:14:55 PM |

  ### Notes:

  - Computational study of the distribution of protein-ligand interaction sites, namely ligand-binding pockets, around protein-protein interfaces where protein-protein interactions occur.   Show that domain-domain inter- faces of multi-domain proteins provide structural pockets that in- teract with ligands.

    How CATH is used:

    Compile a non-redundant data set of 2-domain proteins from CATH.

    Use to investigate "whether small molecule ligands preferably bind to the pockets adjacent to protein interfaces"

    CATH reference:

    Methods  
     Datasets. A nonredundant set of 1,611 dimeric protein complexes was taken from previous studies (13, 27). None of these dimers shares with another dimer more than one pair of monomers at a sequence identity of 35% or higher. A nonredundant set of 1,416 two-domain proteins were taken from the protein classification database CATH version 3.4 (37). They share less than 35% sequence identity among each other. The domain boundaries were manually defined by CATH curators. The complete lists of these two datasets are available at http://cssb.biology.gatech.edu/ppipocket.

  ### Attachments

  - PNAS-2012-Gao-3784-9.pdf
- ## The dynamic determinants of reaction specificity in the IMPDH/GMPR family of (beta/alpha)(8) barrel enzymes

  |  |  |
  | --- | --- |
  | Type | Journal Article |
  | Author | Lizbeth Hedstrom |
  | Volume | 47 |
  | Issue | 3 |
  | Pages | 250-263 |
  | Publication | Critical Reviews in Biochemistry and Molecular Biology |
  | ISSN | 1040-9238 |
  | Date | MAY-JUN 2012 |
  | Extra | WOS:000303244100004 |
  | DOI | 10.3109/10409238.2012.656843 |
  | Abstract | The inosine monophosphate dehydrogenase (IMPDH)/guanosine monophosphate reductase (GMPR) family of (beta/alpha)(8) enzymes presents an excellent opportunity to investigate how subtle changes in enzyme structure change reaction specificity. IMPDH and GMPR bind the same ligands with similar affinities and share a common set of catalytic residues. Both enzymes catalyze a hydride transfer reaction involving a nicotinamide cofactor hydride, and both reactions proceed via the same covalent intermediate. In the case of IMPDH, this intermediate reacts with water, while in GMPR it reacts with ammonia. In both cases, the two chemical transformations are separated by a conformational change. In IMPDH, the conformational change involves a mobile protein flap while in GMPR, the cofactor moves. Thus reaction specificity is controlled by differences in dynamics, which in turn are controlled by residues outside the active site. These findings have some intriguing implications for the evolution of the IMPDH/GMPR family. |
  | Date Added | 10/28/2013, 4:57:32 PM |
  | Modified | 3/7/2014, 12:14:46 PM |

  ### Notes:

  - Review studies how changes in enzyme structure change reaction specificity in GMPR family.

    How SCOP/CATH is used:

    Get count of number of superfamilies in TIM barrel fold.

    SCOP/CATH reference:

    The (β/α)8 barrel, also known as the TIM barrel, is the most common and versatile enzyme fold (Glasner et al., 2006; Soskine & Tawfik, 2010; Zalatan & Herschlag, 2009; Nagano et al., 2002; Gerlt & Raushel, 2003; Wise & Rayment, 2004). Approximately, 30 (β/α)8 barrel protein superfami- lies are listed in the current Structural Classification of Proteins (SCOP) and Class, Architecture, Topology and Homologous (CATH Superfamily databases (Lo Conte et al., 2002; Orengo et al., 1997), catalyzing over 25 dif- ferent reactions (Anantharaman et al., 2003). Therefore, these proteins present a particularly thorny annotation problem.

  ### Attachments

  - 10409238%2E2012%2E656843.pdf
- ## The Effect of Edge Definition of Complex Networks on Protein Structure Identification

  |  |  |
  | --- | --- |
  | Type | Journal Article |
  | Author | Jing Sun |
  | Author | Runyu Jing |
  | Author | Di Wu |
  | Author | Tuanfei Zhu |
  | Author | Menglong Li |
  | Author | Yizhou Li |
  | Publication | COMPUTATIONAL AND MATHEMATICAL METHODS IN MEDICINE |
  | ISSN | 1748-670X |
  | Date | 2013 |
  | DOI | 10.1155/2013/365410 |
  | Language | English |
  | Abstract | The main objective of this study is to explore the contribution of complex network together with its different definitions of vertexes and edges to describe the structure of proteins. Protein folds into a specific conformation for its function depending on interactions between residues. Consequently, in many studies, a protein structure was treated as a complex system comprised of individual components residues, and edges were interactions between residues. What is the proper time for representing a protein structure as a network? To confirm the effect of different definitions of vertexes and edges in constructing the amino acid interaction networks, protein domains and the structural unit of proteins were described using this method. The identification performance of 2847 proteins with domain/domains proved that the structure of proteins was described well when R-C alpha was around 5.0-7.5 angstrom, and the optimal cutoff value for constructing the protein structure networks was 5.0 angstrom (C-alpha-C-alpha distances) while the ideal community division method was community structure detection based on edge betweenness in this study. |
  | Date Added | 10/25/2013, 4:29:01 PM |
  | Modified | 3/7/2014, 12:08:46 PM |

  ### Notes:

  - Present method for representing protein as a connected graph and applying different algorithms for measuring "connectedness".  Apply to domain prediction.

    How SCOP is used:

    Validate domain boundary detection on ASTRAL data.

    SCOP reference:

    2. Materials and Methods

    2.1. Data Collection and Data Set Construction. The infor- mation on domains in proteins in this study were collected from ASTRAL SCOP [40] version 1.75 database. Protein domains in SCOP are grouped into species and hierarchically classified into families, superfamilies, folds, and classes [41]. This database organizes proteins hierarchically according to their families and folds, which is generally considered as the standard for protein structure classification [42]. In order to ensure the nonredundancy of the data, only these proteins with a pairwise sequence identity ≤30% were downloaded, and only those in which the structures were solved by X-ray crystallography with resolution ≤2.5 A ̊ were kept for the clear structure of the proteins. Finally, the remaining 2847 proteins were left for this research. The compositions of the dataset were listed in Table 1.

  ### Attachments

  - 365410.pdf
- ## The emerging dynamic view of proteins: Protein plasticity in allostery, evolution and self-assembly

  |  |  |
  | --- | --- |
  | Type | Journal Article |
  | Author | Ugo Bastolla |
  | Author | Markus Porto |
  | Author | H. Eduardo Roman |
  | URL | http://www.sciencedirect.com/science/article/pii/S1570963913001295 |
  | Series | The emerging dynamic view of proteins:Protein plasticity in allostery,evolution and self-assembly |
  | Volume | 1834 |
  | Issue | 5 |
  | Pages | 817-819 |
  | Publication | Biochimica et Biophysica Acta (BBA) - Proteins and Proteomics |
  | ISSN | 1570-9639 |
  | Date | May 2013 |
  | Journal Abbr | Biochimica et Biophysica Acta (BBA) - Proteins and Proteomics |
  | DOI | 10.1016/j.bbapap.2013.03.016 |
  | Accessed | 12/9/2014, 5:36:30 AM |
  | Library Catalog | ScienceDirect |
  | Short Title | The emerging dynamic view of proteins |
  | Date Added | 12/9/2014, 5:36:30 AM |
  | Modified | 12/9/2014, 5:36:30 AM |

  ### Notes:

  - Review of research on protein plasticity.

    How SCOP is used:

    Background on protein structure classification.

    SCOP reference:

    The assumption that the fold is almost always conserved during evolution, on which databases of protein structure classification [4,5] are based.

  ### Attachments

  - ScienceDirect Full Text PDF
- ## The Enzymatic and Metabolic Capabilities of Early Life

  |  |  |
  | --- | --- |
  | Type | Journal Article |
  | Author | Aaron David Goldman |
  | Author | John A. Baross |
  | Author | Ram Samudrala |
  | Volume | 7 |
  | Issue | 9 |
  | Pages | e39912 |
  | Publication | Plos One |
  | ISSN | 1932-6203 |
  | Date | SEP 10 2012 |
  | Extra | WOS:000308748400001 |
  | DOI | 10.1371/journal.pone.0039912 |
  | Abstract | We introduce the concept of metaconsensus and employ it to make high confidence predictions of early enzyme functions and the metabolic properties that they may have produced. Several independent studies have used comparative bioinformatics methods to identify taxonomically broad features of genomic sequence data, protein structure data, and metabolic pathway data in order to predict physiological features that were present in early, ancestral life forms. But all such methods carry with them some level of technical bias. Here, we cross-reference the results of these previous studies to determine enzyme functions predicted to be ancient by multiple methods. We survey modern metabolic pathways to identify those that maintain the highest frequency of metaconsensus enzymes. Using the full set of modern reactions catalyzed by these metaconsensus enzyme functions, we reconstruct a representative metabolic network that may reflect the core metabolism of early life forms. Our results show that ten enzyme functions, four hydrolases, three transferases, one oxidoreductase, one lyase, and one ligase, are determined by metaconsensus to be present at least as late as the last universal common ancestor. Subnetworks within central metabolic processes related to sugar and starch metabolism, amino acid biosynthesis, phospholipid metabolism, and CoA biosynthesis, have high frequencies of these enzyme functions. We demonstrate that a large metabolic network can be generated from this small number of enzyme functions. |
  | Date Added | 2/20/2014, 12:24:01 PM |
  | Modified | 2/20/2014, 12:24:01 PM |

  ### Notes:

  - Study of ancient folds and functions.

    How SCOP is used:

    Annotate data set with SCOP fold in order to determine if fold is catalytic or noncatalytic.

    SCOP reference:

    Figure 2 shows that many ancient folds are associated with these ten metaconsensus enzyme functions. All ten metaconsensus enzyme functions can be catalyzed by the triosephosphate isomerase (TIM) beta/alpha barrel (SCOP ID = c.1; ances- try = 1.9%). The TIM beta/alpha barrel is a very versatile fold architecture that is able to catalyze a number of disparate enzyme functions [40]. Other ancestral catalytic folds common among these EC groups include the Ferredoxin-like fold (SCOP ID = d.58, ancestry = 1.3%), the Ribonuclease H-like motif (SCOP ID=c.55; ancestry=3.7%), the S-adenosyl-L-methionine-depen- dent methyltransferase fold (SCOP ID=c.66; ancestry=5.0%), the Adenine nucleotide alpha hydrolase-like fold (SCOP ID=c.26; ancestry=5.7%), the UDP-glycosyltransferase/glyco- gen phosphorylase fold (SCOP ID = c.87; ancestry = 11.9%), and the Globin-like fold (SCOP ID = a.1; ancestry = 18.8%).

    Most proteins are composed of more than one fold within a single peptide chain. In many cases, some ancient folds associated with an EC group are not catalytic domains, themselves. By ancestry value, the P-loop containing nucleoside triphosphate fold (SCOP ID = c.37; ancestry = 0.0%) is the most ancient fold associated with any metaconsensus enzyme function, but this fold catalyzes NTP hydrolysis or NDP phosphorylation that is coupled to the enzyme rather than the specific catalytic function of the enzyme. Other ancient folds associated with metaconsensus enzyme functions that do not confer specific catalysis include the DNA/RNA-binding 3-helical bundle (SCOP ID=a.4, ancestry 0.6%), the NAD(P)-binding Rossmann fold (SCOP ID = c.2, ancestry=2.5%), and the Oligonucleotide/oligosaccharide bind- ing (OB) fold (SCOP ID = b.40; ancestry = 4.4%), to name a few.

    ...

    These folds were identified as catalytic or associated noncatalytic folds by their SCOP database functional annotations [4].

  ### Attachments

  - journal.pone.0039912.pdf
- ## The evolutionary history of protein fold families and proteomes confirms that the archaeal ancestor is more ancient than the ancestors of other superkingdoms

  |  |  |
  | --- | --- |
  | Type | Journal Article |
  | Author | Kyung Mo Kim |
  | Author | Gustavo Caetano-Anollés |
  | URL | http://www.biomedcentral.com/1471-2148/12/13 |
  | Volume | 12 |
  | Issue | 1 |
  | Pages | 13 |
  | Publication | BMC Evolutionary Biology |
  | Date | 2012 |
  | Accessed | 9/20/2013, 1:18:03 PM |
  | Library Catalog | Google Scholar |
  | Date Added | 10/11/2013, 10:29:15 AM |
  | Modified | 10/11/2013, 10:29:15 AM |

  ### Tags:

  - Interesting

  ### Notes:

  - Build phylogenies that describe the evolution of proteins and proteomes. These phylogenetic trees are derived from a genomic census of protein domains defined at the fold family (FF) level of structural classification.

    How SCOP is used:

    Use Superfamily. Use data sets derived from SCOP.

    Build a matrix for each proteome with counts of occurrences of each family in SCOP.

    SCOP reference:

    Several reliable classification systems of protein domains are available based on structural similarity and common evolutionary origin. For example, the Struc- tural Classification of Proteins (SCOP) is a high-quality

    taxonomical resource that groups protein domains that have known three-dimensional (3D) structures into fold families (FFs), fold superfamilies and folds [10]. FFs group domains that are closely related at the sequence level (> 30% pairwise amino acid identities) or that share similar structures and functions with lower sequence identity. Fold superfamilies unify FFs that share functional and structural features, suggesting that they probably have common evolutionary origins. Finally, folds group fold superfamilies that have similar arrangements of secondary structures in 3D space but that may not be evolutionarily related due to sequence divergence. As other protein classifications, SCOP was established based on hierarchical levels of structural complexity, each of which represents a certain extent of evolutionary conservation. SCOP currently describes known structures in Protein Data Bank (PDB) entries with about 1,200 folds, 2,000 fold superfamilies, and 4,000 FFs. The relatively small numbers of these domain structures indicate that they are more conserved than domains defined by other classification schemes, such as those of the Pfam database, with levels of molecular diversity that are closer to protein sequence. A recent version of Pfam contains 11,912 distinct domains repre- senting over 107 proteins [11]. While protein domains defined as groups of orthologous sequences share the same problems of sequence analysis, SCOP domain structures are highly conserved evolutionary units [12] that can be used effectively to uncover evolutionary pat- terns in the history of life [13].

    Methods

    Assigning FFs to proteomes

    We downloaded the local MYSQL database from SUPER- FAMILY ver. 1.73 [44] that assigned all known FFs to pro- teomes. At the time of this analysis, the genomes of the 645 organisms we analyzed were completely sequenced. SUPERFAMILY has built HMMs for all fold superfamilies that have been defined in SCOP. Proteomes deposited in the database were scanned with the HMMs using the itera- tive Sequence Alignment and Modeling System (SAM) method [45], which has generated fold superfamily assign- ments covering ~60% of amino acid residues of individual proteomes on the average [44]. Subsequently, protein domains in individual fold superfamilies are assigned to corresponding FFs using a hybrid method that compares the two profile alignments: (1) protein domains to fold superfamily HMMs; and (2) ASTRAL reference sequence of FF to fold superfamily HMMs [46]. FF assignments that meet the E-value of 10-4 were extracted from the individual proteomes. This E-value cutoff is optimal to maximize the rate of true positives in the HMM searches [46]. FFs were named using SCOP concise classification strings (ccs) (e.g., c.67.1.4, where c indicates the protein class, 67 the fold, 1 the fold superfamily, and 4 the FF). The lifestyles of the 645 organisms were manually determined based on various resources including public databases and literature review. Organisms were classified into free-living, facultative para- site, and obligate parasite categories.

    Phylogenomic analysis

    According to SCOP, protein sequences that have sequence identity of over 30% or that share a common ancestor in terms of structures and functions are grouped into FFs [10,18]. Individual FFs are expected to be pre- sent multiple times in a proteome. We thus counted how many times individual FFs were assigned to each of the sampled proteomes.

  ### Attachments

  - 1471-2148-12-13.pdf
  - [HTML] from biomedcentral.com
  - PubMed entry
- ## The evolution of filamin - A protein domain repeat perspective

  |  |  |
  | --- | --- |
  | Type | Journal Article |
  | Author | Sara Light |
  | Author | Rauan Sagit |
  | Author | Sujay S. Ithychanda |
  | Author | Jun Qin |
  | Author | Arne Elofsson |
  | Volume | 179 |
  | Issue | 3, SI |
  | Pages | 289-298 |
  | Publication | JOURNAL OF STRUCTURAL BIOLOGY |
  | ISSN | 1047-8477 |
  | Date | September 2012 |
  | DOI | 10.1016/j.jsb.2012.02.010 |
  | Language | English |
  | Abstract | Particularly in higher eukaryotes, some protein domains are found in tandem repeats, performing broad functions often related to cellular organization. For instance, the eukaryotic protein filamin interacts with many proteins and is crucial for the cytoskeleton. The functional properties of long repeat domains are governed by the specific properties of each individual domain as well as by the repeat copy number. To provide better understanding of the evolutionary and functional history of repeating domains, we investigated the mode of evolution of the filamin domain in some detail. Among the domains that are common in long repeat proteins, sushi and spectrin domains evolve primarily through cassette tandem duplications while scavenger and immunoglobulin repeats appear to evolve through clustered tandem duplications. Additionally, immunoglobulin and filamin repeats exhibit a unique pattern where every other domain shows high sequence similarity. This pattern may be the result of tandem duplications, serve to avert aggregation between adjacent domains or it is the result of functional constraints. In filamin, our studies confirm the presence of interspersed integrin binding domains in vertebrates, while invertebrates exhibit more varied patterns, including more clustered integrin binding domains. The most notable case is leech filamin, which contains a 20 repeat expansion and exhibits unique dimerization topology. Clearly, invertebrate filamins are varied and contain examples of similar adjacent integrin-binding domains. Given that invertebrate integrin shows more similarity to the weaker filamin binder, integrin beta 3, it is possible that the distance between integrin-binding domains is not as crucial for invertebrate filamins as for vertebrates. (C) 2012 Elsevier Inc. All rights reserved. |
  | Date Added | 10/11/2013, 10:29:15 AM |
  | Modified | 10/11/2013, 10:29:15 AM |

  ### Tags:

  - Aggregation
  - Filamin
  - Integrin
  - Protein domain evolution
  - Protein domain repeats
  - Tandem duplication

  ### Notes:

  - To provide better understanding of the evolutionary and functional history of repeating domains, we investigated the mode of evolution of the filamin domain in some detail.  Use computational methods to study properties such as sequence similarity and solvent accessibility.

     How SCOP is used:

    Cite SCOP as a reference for protein domain evolution.

     SCOP reference:

    The majority of known proteins are composed of at least one protein domain, functional units of common descent. Indeed, most proteins contain more than one domain – a tendency that is most pronounced in eukaryotes (Teichmann et al., 1998; Apic et al., 2001; Ekman et al., 2005; Gerstein, 1998). These findings are quite important, since they provide a clear path through which proteins can evolve in a modular fashion, adding and removing functionally distinct building blocks (Murzin et al., 1995). However, protein do- mains are not static, but evolve and can show a great deal of vari- ation, for instance in length (Grishin, 2001; Reeves et al., 2006).

  ### Attachments

  - 1-s2.0-S1047847712000639-main.pdf
- ## The four-transmembrane protein IP39 of Euglena forms strands by a trimeric unit repeat

  |  |  |
  | --- | --- |
  | Type | Journal Article |
  | Author | Hiroshi Suzuki |
  | Author | Yasuyuki Ito |
  | Author | Yuji Yamazaki |
  | Author | Katsuhiko Mineta |
  | Author | Masami Uji |
  | Author | Kazuhiro Abe |
  | Author | Kazutoshi Tani |
  | Author | Yoshinori Fujiyoshi |
  | Author | Sachiko Tsukita |
  | URL | http://www.nature.com/ncomms/journal/v4/n4/abs/ncomms2731.html |
  | Volume | 4 |
  | Pages | 1766 |
  | Publication | Nature communications |
  | Date | 2013 |
  | Accessed | 9/20/2013, 1:11:53 PM |
  | Library Catalog | Google Scholar |
  | Date Added | 10/11/2013, 10:29:15 AM |
  | Modified | 10/11/2013, 10:29:15 AM |

  ### Notes:

  - Reports the structure of the transmembrane protein IP39 in Euglena, which was obtained by electron crystallography. Also performed model fitting to study the trimeric unit.

    How SCOP is used:

    They selected candidate model from the structures found in the SCOP database (by manual inspection of the database.) Doesn't mention SCOP's hierarchy.

    SCOP Reference:

    To examine the arrangement of each molecule in the trimeric unit, we attempted to find a well-fitted template structure to our EM map from the known structures of the four-helical bundle of the membrane proteins selected by manual inspection from the database SCOP22 and membrane proteins of a known 3D structure (http://blanco.biomol.uci.edu/mpstruc/ listAll/list) (Fig. 4d,e). Twelve models, including connexin26 and the K subunit of V-type ATPase, were selected to dock into the EM map using the Situs program23. The model best fitted to the density of the IP39 protomer (the cross-correlation function: 0.816 to B-Mol1, 0.857 to B-Mol2 and 0.838 to B-Mol3) was the structure of leukotriene C4 synthase (Protein Data Bank ID: 2UUI), which had a MAPEG domain-like fold.

    ...  
      

    Model fitting. The candidate transmembrane model of IP39 was selected from the known structures of four-helical bundles of membrane proteins from the database SCOP22 and membrane proteins with a known 3D structure (http:// blanco.biomol.uci.edu/mpstruc/listAll/list). After selection by manual inspection, 12 models were selected: F1FO ATP synthase subunit A (PDB: 1C17), neurotransmitter-gated ion-channel transmembrane pore (PDB: 1OED), heme- binding four-helical bundle (PDB: 1PPJ, 1KQF, 1Q16, 2BS2, 1NEK), MAPEG domain-like (PDB: 2Q7R, 2H8A, 2UUI), connexin26 (PDB: 2ZW3) and V-ATPase K-ring (PDB: 2BL2).

  ### Attachments

  - [HTML] from nih.gov
  - ncomms2731.pdf
  - Snapshot
- ## The FSSP database: fold classification based on structure-structure alignment of proteins

  |  |  |
  | --- | --- |
  | Type | Journal Article |
  | Author | L. Holm |
  | Author | C. Sander |
  | Volume | 24 |
  | Issue | 1 |
  | Pages | 206-209 |
  | Publication | Nucleic Acids Research |
  | ISSN | 0305-1048 |
  | Date | Jan 1, 1996 |
  | Extra | PMID: 8594580 PMCID: PMC145583 |
  | Journal Abbr | Nucleic Acids Res. |
  | Library Catalog | NCBI PubMed |
  | Language | eng |
  | Abstract | The FSSP database presents a continuously updated classification of 3-D protein folds based on an all-against-all comparison of structures currently in the Protein Data Bank (PDB) [Bernstein et al. (1977) J. Mol. Biol., 112, 535- 542]. The database currently contains an extended structural family for each of 600 representative protein chains which have <25% mutual sequence identity. The results of the exhaustive pairwise structure comparisons are reported in the form of a fold tree generated by hierarchical clustering and as a series of structurally representative sets of folds at varying levels of uniqueness. For each query structure from the representative set, there is a database entry containing structure-structure alignments with its structural neighbours in the representative set and its sequence homologs in the PDB. All alignments are based purely on the 3-D co-ordinates of the proteins and are derived by an automatic structure comparison program (Dali). The FSSP database is accessible electronically on the World Wide Web and by anonymous ftp. |
  | Short Title | The FSSP database |
  | Date Added | 10/29/2014, 12:00:31 PM |
  | Modified | 10/29/2014, 12:00:31 PM |

  ### Tags:

  - Amino Acid Sequence
  - Animals
  - Computer Communication Networks
  - Databases, Factual
  - Humans
  - Molecular Sequence Data
  - Protein Folding
  - Sequence Alignment

  ### Attachments

  - PubMed entry
- ## The genome of the obligate intracellular parasite trachipleistophora hominis: new insights into microsporidian genome dynamics and reductive evolution

  |  |  |
  | --- | --- |
  | Type | Journal Article |
  | Author | Eva Heinz |
  | Author | Tom A. Williams |
  | Author | Sirintra Nakjang |
  | Author | Christophe J. Noël |
  | Author | Daniel C. Swan |
  | Author | Alina V. Goldberg |
  | Author | Simon R. Harris |
  | Author | Thomas Weinmaier |
  | Author | Stephanie Markert |
  | Author | Dörte Becher |
  | URL | http://dx.plos.org/10.1371/journal.ppat.1002979 |
  | Volume | 8 |
  | Issue | 10 |
  | Pages | e1002979 |
  | Publication | PLoS pathogens |
  | Date | 2012 |
  | Accessed | 9/20/2013, 1:11:53 PM |
  | Library Catalog | Google Scholar |
  | Short Title | The genome of the obligate intracellular parasite trachipleistophora hominis |
  | Date Added | 10/11/2013, 10:29:15 AM |
  | Modified | 10/11/2013, 10:29:15 AM |

  ### Tags:

  - Acquired Immunodeficiency Syndrome
  - Biological Evolution
  - Energy Metabolism
  - Evolution, Molecular
  - Genome, Fungal
  - Humans
  - Microsporidia
  - Mitochondria
  - Phylogeny
  - Proteome
  - Proteomics
  - RNA Interference
  - RNA, Small Interfering
  - Sequence Analysis, DNA

  ### Notes:

  - Computational study in which the authors examined the genome of Trachipleistophora hominis to examine the "gene content, genome architecture and intergenic regions of a larger microsporidian genome." They also studied the features of the common ancestor to microsporidia.

    **SCOP Use**

    SCOP was used for function annotation in the protein cluster analysis.  It was one of several databases that was searched against, but only if the cluster in question did not have yeast or human sequences.

    **SCOP Reference**

    To infer putative functions for the identified clusters, we used  
    the COG annotation of the human or yeast homologue present in the clusters. In cases were a cluster did not contain a human or  
    yeast sequences, all of the cluster members were searched with  
    BLASTP against all proteins in the COG database with an e-value  
    cutoff of #0.01. Clusters were assigned a functional COG  
    category if at least 2 members in the cluster hit the same COG.  
    In cases where no COG hit was obtained for clusters inferred to be  
    present in the ancestor of the microsporidians analysed, we tried to  
    infer putative functions using the highly sensitive HHsearch [44].  
    This was done with default settings and by searching against  
    protein profiles from COG, KOG, CDD, Pfam, Superfamily,  
    SMART, SCOP, PDB, and TIGRfams [36,107–114]; any  
    functional annotation was added based upon on an e-value cutoff  
    #0.01 and a probability of .90%.

  ### Attachments

  - [HTML] from plos.org
  - journal.ppat.1002979.pdf
  - PubMed entry
- ## The HHpred interactive server for protein homology detection and structure prediction

  |  |  |
  | --- | --- |
  | Type | Journal Article |
  | Author | Johannes Söding |
  | Author | Andreas Biegert |
  | Author | Andrei N Lupas |
  | Volume | 33 |
  | Issue | Web Server issue |
  | Pages | W244-248 |
  | Publication | Nucleic acids research |
  | ISSN | 1362-4962 |
  | Date | Jul 1, 2005 |
  | Extra | PMID: 15980461 |
  | Journal Abbr | Nucleic Acids Res. |
  | DOI | 10.1093/nar/gki408 |
  | Library Catalog | NCBI PubMed |
  | Language | eng |
  | Abstract | HHpred is a fast server for remote protein homology detection and structure prediction and is the first to implement pairwise comparison of profile hidden Markov models (HMMs). It allows to search a wide choice of databases, such as the PDB, SCOP, Pfam, SMART, COGs and CDD. It accepts a single query sequence or a multiple alignment as input. Within only a few minutes it returns the search results in a user-friendly format similar to that of PSI-BLAST. Search options include local or global alignment and scoring secondary structure similarity. HHpred can produce pairwise query-template alignments, multiple alignments of the query with a set of templates selected from the search results, as well as 3D structural models that are calculated by the MODELLER software from these alignments. A detailed help facility is available. As a demonstration, we analyze the sequence of SpoVT, a transcriptional regulator from Bacillus subtilis. HHpred can be accessed at http://protevo.eb.tuebingen.mpg.de/hhpred. |
  | Date Added | 10/11/2013, 10:29:15 AM |
  | Modified | 10/11/2013, 10:29:15 AM |

  ### Notes:

  - HHPred is a remote homolog detection and structure prediction server which relies on HMMs.

    How SCOP is used:

    Permits search of SCOP, among other databases, for homologues.

    SCOP references:

    Under abstract:

    It allows to search a wide choice of databases, such as the PDB, SCOP, Pfam, SMART, COGs and CDD.

  ### Attachments

  - Full Text PDF
  - PubMed entry
  - Snapshot
- ## The Impact of Computer Science in Molecular Medicine: Enabling High-Throughput Research

  |  |  |
  | --- | --- |
  | Type | Journal Article |
  | Author | Diana de la Iglesia |
  | Author | Miguel Garcia-Remesal |
  | Author | Guillermo de la Calle |
  | Author | Casimir Kulikowski |
  | Author | Ferran Sanz |
  | Author | Victor Maojo |
  | Volume | 13 |
  | Issue | 5 |
  | Pages | 526-575 |
  | Publication | CURRENT TOPICS IN MEDICINAL CHEMISTRY |
  | ISSN | 1568-0266 |
  | Date | March 2013 |
  | Language | English |
  | Abstract | The Human Genome Project and the explosion of high-throughput data have transformed the areas of molecular and personalized medicine, which are producing a wide range of studies and experimental results and providing new insights for developing medical applications. Research in many interdisciplinary fields is resulting in data repositories and computational tools that support a wide diversity of tasks: genome sequencing, genome-wide association studies, analysis of genotype-phenotype interactions, drug toxicity and side effects assessment, prediction of protein interactions and diseases, development of computational models, biomarker discovery, and many others. The authors of the present paper have developed several inventories covering tools, initiatives and studies in different computational fields related to molecular medicine: medical informatics, bioinformatics, clinical informatics and nanoinformatics. With these inventories, created by mining the scientific literature, we have carried out several reviews of these fields, providing researchers with a useful framework to locate, discover, search and integrate resources. In this paper we present an analysis of the state-of-the-art as it relates to computational resources for molecular medicine, based on results compiled in our inventories, as well as results extracted from a systematic review of the literature and other scientific media. The present review is based on the impact of their related publications and the available data and software resources for molecular medicine. It aims to provide information that can be useful to support ongoing research and work to improve diagnostics and therapeutics based on molecular-level insights. |
  | Date Added | 10/25/2013, 4:29:01 PM |
  | Modified | 10/8/2014, 12:50:51 PM |

  ### Tags:

  - Biomedical Informatics
  - Computational Resources
  - Data sources
  - Genotype-Phenotype
  - Medicinal Chemistry
  - Molecular Medicine
  - Personalized Medicine

  ### Notes:

  - Paper unavailable.
- ## The impact of splicing on protein domain architecture

  |  |  |
  | --- | --- |
  | Type | Journal Article |
  | Author | Sara Light |
  | Author | Arne Elofsson |
  | URL | http://www.sciencedirect.com/science/article/pii/S0959440X13000432 |
  | Volume | 23 |
  | Issue | 3 |
  | Pages | 451-458 |
  | Publication | Current Opinion in Structural Biology |
  | ISSN | 0959-440X |
  | Date | June 2013 |
  | Journal Abbr | Current Opinion in Structural Biology |
  | DOI | 10.1016/j.sbi.2013.02.013 |
  | Accessed | 9/20/2013, 12:47:33 PM |
  | Library Catalog | ScienceDirect |
  | Abstract | Many proteins are composed of protein domains, functional units of common descent. Multidomain forms are common in all eukaryotes making up more than half of the proteome and the evolution of novel domain architecture has been accelerated in metazoans. It is also becoming increasingly clear that alternative splicing is prevalent among vertebrates. Given that protein domains are defined as structurally, functionally and evolutionarily distinct units, one may speculate that some alternative splicing events may lead to clean excisions of protein domains, thus generating a number of different domain architectures from one gene template. However, recent findings indicate that smaller alternative splicing events, in particular in disordered regions, might be more prominent than domain architectural changes. The problem of identifying protein isoforms is, however, still not resolved. Clearly, many splice forms identified through detection of mRNA sequences appear to produce ‘nonfunctional’ proteins, such as proteins with missing internal secondary structure elements. Here, we review the state of the art methods for identification of functional isoforms and present a summary of what is known, thus far, about alternative splicing with regard to protein domain architectures. |
  | Date Added | 10/11/2013, 10:29:15 AM |
  | Modified | 3/7/2014, 12:09:22 PM |

  ### Notes:

  - Review of research into how alternative splicing affects the structure and expression of protein domains.

    How SCOP/CATH is used:

    Background on protein structure classification.

    SCOP reference:

    Protein domains are structural, functional and evolutionary  
    building blocks that, within one protein, can form  
    various architectures that may be composed of one or  
    several domains [1]. Domains can often be defined either  
    from a sequence similarity viewpoint as in the Pfam  
    database [2], from an evolutionary perspective as in SCOP  
    [3] or from a structural perspective as in CATH [4]. In  
    many cases these definitions overlap [5].

  ### Attachments

  - ScienceDirect Full Text PDF
- ## The impact of structural genomics: expectations and outcomes

  |  |  |
  | --- | --- |
  | Type | Journal Article |
  | Author | John-Marc Chandonia |
  | Author | Steven E. Brenner |
  | Volume | 311 |
  | Issue | 5759 |
  | Pages | 347-351 |
  | Publication | Science (New York, N.Y.) |
  | ISSN | 1095-9203 |
  | Date | Jan 20, 2006 |
  | Extra | PMID: 16424331 |
  | Journal Abbr | Science |
  | DOI | 10.1126/science.1121018 |
  | Library Catalog | NCBI PubMed |
  | Language | eng |
  | Abstract | Structural genomics (SG) projects aim to expand our structural knowledge of biological macromolecules while lowering the average costs of structure determination. We quantitatively analyzed the novelty, cost, and impact of structures solved by SG centers, and we contrast these results with traditional structural biology. The first structure identified in a protein family enables inference of the fold and of ancient relationships to other proteins; in the year ending 31 January 2005, about half of such structures were solved at a SG center rather than in a traditional laboratory. Furthermore, the cost of solving a structure at the most efficient SG center in the United States has dropped to one-quarter of the estimated cost of solving a structure by traditional methods. However, the efficiency of the top structural biology laboratories-even though they work on very challenging structures-is comparable to that of SG centers; moreover, traditional structural biology papers are cited significantly more often, suggesting greater current impact. |
  | Short Title | The impact of structural genomics |
  | Date Added | 10/13/2014, 2:13:49 PM |
  | Modified | 10/13/2014, 2:13:49 PM |

  ### Tags:

  - Amino Acid Sequence
  - Computational Biology
  - Costs and Cost Analysis
  - Databases, Protein
  - Financial Support
  - Genomics
  - Protein Conformation
  - Protein Folding
  - Proteins
  - Publishing

  ### Attachments

  - PubMed entry
- ## The Jpred 3 secondary structure prediction server

  |  |  |
  | --- | --- |
  | Type | Journal Article |
  | Author | Christian Cole |
  | Author | Jonathan D Barber |
  | Author | Geoffrey J Barton |
  | Volume | 36 |
  | Issue | Web Server issue |
  | Pages | W197-201 |
  | Publication | Nucleic acids research |
  | ISSN | 1362-4962 |
  | Date | Jul 1, 2008 |
  | Extra | PMID: 18463136 |
  | Journal Abbr | Nucleic Acids Res. |
  | DOI | 10.1093/nar/gkn238 |
  | Library Catalog | NCBI PubMed |
  | Language | eng |
  | Abstract | Jpred (http://www.compbio.dundee.ac.uk/jpred) is a secondary structure prediction server powered by the Jnet algorithm. Jpred performs over 1000 predictions per week for users in more than 50 countries. The recently updated Jnet algorithm provides a three-state (alpha-helix, beta-strand and coil) prediction of secondary structure at an accuracy of 81.5%. Given either a single protein sequence or a multiple sequence alignment, Jpred derives alignment profiles from which predictions of secondary structure and solvent accessibility are made. The predictions are presented as coloured HTML, plain text, PostScript, PDF and via the Jalview alignment editor to allow flexibility in viewing and applying the data. The new Jpred 3 server includes significant usability improvements that include clearer feedback of the progress or failure of submitted requests. Functional improvements include batch submission of sequences, summary results via email and updates to the search databases. A new software pipeline will enable Jnet/Jpred to continue to be updated in sync with major updates to SCOP and UniProt and so ensures that Jpred 3 will maintain high-accuracy predictions. |
  | Date Added | 10/11/2013, 10:29:15 AM |
  | Modified | 10/11/2013, 10:29:15 AM |

  ### Tags:

  - ASTRAL domain structures
  - ASTRAL sequences
  - ASTRAL subsets
  - Cite ASTRAL

  ### Notes:

  - Jpred server for secondary structure prediction.

    How SCOP is used:

     SCOP is used to improve domain boundaries where structural data is available.  Also offer a cross-reference between SCOP and Pfam domains.

    Use SCOP data in 7-fold cross validation method for secondary structure prediction.  Use ASTRAL representative data set of structural data.

    SCOP reference:

    The method was developed through 7-fold cross-validated training on a sequence and structure non-redundant dataset derived from the Astral compen- dium of SCOP domain data (release 1.71) (24,25) at the superfamily level.

    ...

    Hence, Jnet will now be linked to SCOP releases and will be retrained whenever a new SCOP release is announced or soon thereafter. The datasets used for training Jnet will be made available via the website.

    A new semi-automatic pipeline has been developed in Perl for creating the Jnet training data. The pipeline requires SCOP domain definition and sequence data as determined by Astral and will create (with some manual input) a structurally and sequence non-redundant dataset ready for input to Jnet neural networks for full-scale training and validation via the SNNS application. Once the training of Jnet is checked on an independent blind set it will be recompiled with the new networks and used in Jpred.

  ### Attachments

  - Full Text PDF
  - PubMed entry
  - Snapshot
- ## The Landscape of the Prion Protein's Structural Response to Mutation Revealed by Principal Component Analysis of Multiple NMR Ensembles

  |  |  |
  | --- | --- |
  | Type | Journal Article |
  | Author | Deena M. A. Gendoo |
  | Author | Paul M. Harrison |
  | Volume | 8 |
  | Issue | 8 |
  | Publication | PLoS computational biology |
  | ISSN | 1553-7358 |
  | Date | August 2012 |
  | DOI | 10.1371/journal.pcbi.1002646 |
  | Language | English |
  | Abstract | Prion Proteins (PrP) are among a small number of proteins for which large numbers of NMR ensembles have been resolved for sequence mutants and diverse species. Here, we perform a comprehensive principle components analysis (PCA) on the tertiary structures of PrP globular proteins to discern PrP subdomains that exhibit conformational change in response to point mutations and clade-specific evolutionary sequence mutation trends. This is to our knowledge the first such large-scale analysis of multiple NMR ensembles of protein structures, and the first study of its kind for PrPs. We conducted PCA on human (n = 11), mouse (n = 14), and wildtype (n = 21) sets of PrP globular structures, from which we identified five conformationally variable subdomains within PrP. PCA shows that different non-local patterns and rankings of variable subdomains arise for different pathogenic mutants. These subdomains may thus be key areas for initiating PrP conversion during disease. Furthermore, we have observed the conformational clustering of divergent TSE-non-susceptible species pairs; these non-phylogenetic clusterings indicate structural solutions towards TSE resistance that do not necessarily coincide with evolutionary divergence. We discuss the novelty of our approach and the importance of PrP subdomains in structural conversion during disease. |
  | Date Added | 10/25/2013, 4:17:08 PM |
  | Modified | 3/7/2014, 12:14:52 PM |

  ### Tags:

  - Cite ASTRAL

  ### Notes:

  - Study dynamics of Prion Proteins (PrP) using NMR data and principal component analysis.

    How SCOP/CATH is used:

    Collated data set of PrP structures.  "search" for all proteins within the Prion-like superfamily and family to collect a data set of 41 PDB structures that were all NMR derived.

    Cite ASTRAL, and not SCOP paper, but doesn't seem that ASTRAL was needed.

    Collect structures from Major Prion Protein architecture in CATH.

    SCOP reference:

    PDB Structures

    We collated all known PrP structures in the RCSB Protein Data Bank [42], by searching for all proteins within the ‘Prion-like’ family and superfamily of SCOP [43], proteins which match the architecture of the Major Prion Protein as specified in CATH [44] (Mainly alpha, orthogonal bundle, 1.10.790), as well as searches based on PFAM [45] Hidden Markov Models (HMMs) repre- senting the Prion-like protein Doppel [PF11466], Prion/Doppel alpha-helical domain [PF00377], and the major prion protein bPrP-N terminal [PF11587]. These searches yielded a total of 112 prion PDB structures, from which only PrP globular domains were selected. The list of PrP globular domains was further refined to exclude dimers (ex: [PDB 3O79]), domain-swapped structures (ex: [PDB 1I4M]), and pdb models representing the average minimized structure of an NMR ensemble (ex: [1E1J, 1E1S, 1E1W, 1FKC, 1HJM, 1QLX, 1QM0, 1QM2] in human PrP, [1AG2] in mouse PrP, and [1DWY], [1DX0] in bovine PrP). A total of 41 PDB structures, all of which are NMR-derived, were selected for analysis.

  ### Attachments

  - journal.pcbi.1002646.pdf
- ## The macrodomain family: Rethinking an ancient domain from evolutionary perspectives

  |  |  |
  | --- | --- |
  | Type | Journal Article |
  | Author | Li XiaoLei |
  | Author | Wu ZhiQiang |
  | Author | Han WeiDong |
  | Volume | 58 |
  | Issue | 9 |
  | Pages | 953-960 |
  | Publication | Chinese Science Bulletin |
  | ISSN | 1001-6538 |
  | Date | MAR 2013 |
  | Extra | WOS:000316219500001 |
  | DOI | 10.1007/s11434-013-5674-9 |
  | Abstract | The reasons why certain domains evolve much slower than others is unclear. The notion that functionally more important genes evolve more slowly than less important genes is one of the few commonly believed principles of molecular evolution. The macro-domain (also known as the X domain) is an ancient, slowly evolving and highly conserved structural domain found in proteins throughout all of the kingdoms and was first discovered nearly two decades ago with the isolation and cloning of macroH2A1. Macrodomains, which are functionally promiscuous, have been studied intensively for the past decade due to their importance in the regulation of cellular responses to DNA damage, chromatin remodeling, transcription and tumorigenesis. Recent structural, phylogenetic and biological analyses, however, suggest the need for some reconsideration of the evolutionary advantage of concentrating such a plethora of diverse functions into the macrodomain and of how macrodomains could perform so many functions. In this article, we focus on macrodomains that are evolving slowly and broadly discuss the potential relationship between the biological evolution and functional diversity of macrodomains. |
  | Date Added | 2/20/2014, 12:24:01 PM |
  | Modified | 2/20/2014, 12:24:01 PM |

  ### Notes:

  - Review of studies of macrodomains.

    How SCOP is used:

    Background on protein domains.

    SCOP reference:

    Accordingly, the domains can be viewed as the building blocks of pro- teins, and, with the exception of some disordered proteins, all proteins consist of one or more domains [11].

  ### Attachments

  - art%3A10.1007%2Fs11434-013-5674-9.pdf
- ## The Missing Linker: A Dimerization Motif Located within Polyketide Synthase Modules

  |  |  |
  | --- | --- |
  | Type | Journal Article |
  | Author | Jianting Zheng |
  | Author | Christopher D. Fage |
  | Author | Borries Demeler |
  | Author | David W. Hoffman |
  | Author | Adrian T. Keatinge-Clay |
  | Volume | 8 |
  | Issue | 6 |
  | Pages | 1263–1270 |
  | Publication | Acs Chemical Biology |
  | Date | June 2013 |
  | DOI | 10.1021/cb400047s |
  | Abstract | The dimerization of multimodular polyketide synthases is essential for their function. Motifs that supplement the contacts made by dimeric polyketide synthase enzymes have previously been characterized outside the boundaries of modules, at the N- and C-terminal ends of polyketide synthase subunits. Here we describe a heretofore uncharacterized dimerization motif located within modules. The dimeric state of this dimerization element was elucidated through the 2.6 angstrom resolution crystal structure of a fragment containing a dimerization element and a ketoreductase. The solution structure of a standalone dimerization element was revealed by nuclear magnetic resonance spectroscopy to be consistent with that of the crystal structure, and its dimerization constant was measured through analytical ultracentrifugation to be similar to 20 mu M. The dimer buries similar to 990 A(2) at its interface, and its C-terminal helices rigidly connect to ketoreductase domains to constrain their locations within a module. These structural restraints permitted the construction of a common type of polyketide synthase module. |
  | Date Added | 3/7/2014, 12:08:00 PM |
  | Modified | 3/7/2014, 12:08:00 PM |
- ## The modified Mahalanobis Discriminant for predicting outer membrane proteins by using Chou's pseudo amino acid composition

  |  |  |
  | --- | --- |
  | Type | Journal Article |
  | Author | Hao Lin |
  | Volume | 252 |
  | Issue | 2 |
  | Pages | 350-356 |
  | Publication | JOURNAL OF THEORETICAL BIOLOGY |
  | ISSN | 0022-5193 |
  | Date | MAY 21 2008 |
  | DOI | 10.1016/j.jtbi.2008.02.004 |
  | Language | English |
  | Abstract | The outer membrane proteins (OMPs) are beta-barrel membrane proteins that performed lots of biology functions. The discriminating OMPs from other non-OMPs is a very important task for understanding some biochemical process. In this study, a method that combines increment of diversity with modified Mahalanobis Discriminant, called IDQD, is presented to predict 208 OMPs, 206 transmembrane helical proteins (TMHPs) and 673 globular proteins (GPs) by using Chou's pseudo amino acid compositions as parameters. The overall accuracy of jackknife cross-validation is 93.2% and 96.1%, respectively, for three datasets (OMPs, TMHPs and GPs) and two datasets (OMPs and non-OMPs). These predicted results suggest that the method can be effectively applied to discriminate OMPs, TMHPs and GPs. And it also indicates that the pseudo amino acid composition can better reflect the core feature of membrane proteins than the classical amino acid composition. (C) 2008 Elsevier Ltd. All rights reserved. |
  | Date Added | 10/11/2013, 10:29:15 AM |
  | Modified | 10/11/2013, 10:29:15 AM |

  ### Tags:

  - Chou's pseudo amino acid composition
  - globular protein
  - increment of diversity
  - modified Mahalanobis Discriminant
  - outer membrane protein
  - transmembrane helical protein

  ### Notes:

  - Presents method to classify outer membrane proteins, transmembrane proteins, and globular proteins.

    How SCOP is used:

    Use SCOP to derive the data set of globular proteins.  Use SCOP 1.37 and the "PDB40D" data set.

    SCOP reference:

    Under Materials and theoretical algorithm

    2.1. Datasets

    A total of 208 OMPs, 206 TMHPs and 673 GPs studied here were constructed by Park et al. (2005), extracted from the PSORT-B database (Gardy et al., 2003) for membrane proteins and PDB40D\_1.37 database of SCOP (Murzin et al., 1995; Berman et al., 2000) for GPs. GPs dataset contains 154 all-a proteins, 156 all-b proteins, 184 a+b proteins and 179 a/b proteins. The sequence identity in each protein class is lesser than 40%. Therefore, the proteins are not similar to each other in each database.

  ### Attachments

  - 1-s2.0-S0022519308000556-main.pdf
- ## The natural history of ubiquitin and ubiquitin-related domains

  |  |  |
  | --- | --- |
  | Type | Journal Article |
  | Author | Alexander Maxwell Burroughs |
  | Author | Lakshminarayan M. Iyer |
  | Author | L. Aravind |
  | Volume | 17 |
  | Pages | 1433-1460 |
  | Publication | FRONTIERS IN BIOSCIENCE-LANDMARK |
  | ISSN | 1093-9946 |
  | Date | JAN 1 2012 |
  | DOI | 10.2741/3996 |
  | Language | English |
  | Abstract | The ubiquitin (Ub) system is centered on conjugation and deconjugation of Ub and Ub-like (Ubls) proteins by a system of ligases and peptidases, respectively. Ub/Ubls contain the beta-grasp fold, also found in numerous proteins with biochemically distinct roles unrelated to the conventional Ub-system. The beta-GF underwent an early radiation spawning at least seven clades prior to the divergence of extant organisms from their last universal common ancestor, first emerging in the context of translation-related RNA-interactions and subsequently exploding to occupy various functional niches. Most beta-GF diversification occurred in prokaryotes, with the Ubl clade showing dramatic expansion in the eukaryotes. Diversification of Ubl families in eukaryotes played a major role in emergence of characteristic eukaryotic cellular sub-structures and systems. Recent comparative genomics studies indicate precursors of the eukaryotic Ub-system emerged in prokaryotes. The simplest of these combine an Ubl and an E1-like enzyme in metabolic pathways. Sampylation in archaea and Urmylation in eukaryotes appear to represent recruitment of such systems as simple protein-tagging apparatuses. However, other prokaryotic systems incorporated further components and mirror the eukaryotic condition in possessing an E2, a RING-type E3 or both of these components. Additionally, prokaryotes have evolved conjugation systems independent of Ub ligases, such as the Pup system. |
  | Date Added | 10/11/2013, 10:29:15 AM |
  | Modified | 10/11/2013, 10:29:15 AM |

  ### Tags:

  - Beta-Grasp Fold
  - Non-Ribosomal Peptide Ligases
  - Prokaryotic Ubiquitin Conjugation
  - Review
  - Rna Modification
  - Sumo
  - Ubiquitin

  ### Notes:

  - Article unavailable.
- ## The novel structure of the cockroach allergen Bla g 1 has implications for allergenicity and exposure assessment

  |  |  |
  | --- | --- |
  | Type | Journal Article |
  | Author | Geoffrey A. Mueller |
  | Author | Lars C. Pedersen |
  | Author | Fred B. Lih |
  | Author | Jill Glesner |
  | Author | Andrea F. Moon |
  | Author | Martin D. Chapman |
  | Author | Kenneth B. Tomer |
  | Author | Robert E. London |
  | Author | Anna Pomes |
  | Volume | 132 |
  | Issue | 6 |
  | Pages | 1420-+ |
  | Publication | Journal of Allergy and Clinical Immunology |
  | ISSN | 0091-6749; 1097-6825 |
  | Date | DEC 2013 |
  | Extra | WOS:000327538200021 |
  | DOI | 10.1016/j.jaci.2013.06.014 |
  | Abstract | Background: Sensitization to cockroach allergens is a major risk factor for asthma. The cockroach allergen Bla g 1 has multiple repeats of approximately 100 amino acids, but the fold of the protein and its biological function are unknown. Objective: We sought to determine the structure of Bla g 1, investigate the implications for allergic disease, and standardize cockroach exposure assays. Methods: nBla g 1 and recombinant constructs were compared by using ELISA with specific murine IgG and human IgE. The structure of Bla g 1 was determined by x-ray crystallography. Mass spectrometry and nuclear magnetic resonance spectroscopy were used to examine the ligand-binding properties of the allergen. Results: The structure of an rBla g 1 construct with comparable IgE and IgG reactivity to the natural allergen was solved by x-ray crystallography. The Bla g 1 repeat forms a novel fold with 6 helices. Two repeats encapsulate a large and nearly spherical hydrophobic cavity, defining the basic structural unit. Lipids in the cavity varied depending on the allergen origin. Palmitic, oleic, and stearic acids were associated with nBla g 1 from cockroach frass. One unit of Bla g 1 was equivalent to 104 ng of allergen. Conclusions: Bla g 1 has a novel fold with a capacity to bind various lipids, which suggests a digestive function associated with nonspecific transport of lipid molecules in cockroaches. Defining the basic structural unit of Bla g 1 facilitates the standardization of assays in absolute units for the assessment of environmental allergen exposure. |
  | Date Added | 2/13/2014, 4:13:41 PM |
  | Modified | 3/7/2014, 1:06:39 PM |
- ## The Pfam protein families database

  |  |  |
  | --- | --- |
  | Type | Journal Article |
  | Author | Alex Bateman |
  | Author | Ewan Birney |
  | Author | Lorenzo Cerruti |
  | Author | Richard Durbin |
  | Author | Laurence Etwiller |
  | Author | Sean R. Eddy |
  | Author | Sam Griffiths-Jones |
  | Author | Kevin L. Howe |
  | Author | Mhairi Marshall |
  | Author | Erik LL Sonnhammer |
  | URL | http://nar.oxfordjournals.org/content/30/1/276.short |
  | Volume | 30 |
  | Issue | 1 |
  | Pages | 276–280 |
  | Publication | Nucleic acids research |
  | Date | 2002 |
  | Accessed | 2/28/2013, 3:33:53 PM |
  | Library Catalog | Google Scholar |
  | Date Added | 10/11/2013, 10:29:15 AM |
  | Modified | 10/11/2013, 10:29:15 AM |

  ### Tags:

  - likely ASTRAL
  - likely ASTRAL sequences

  ### Notes:

  - How SCOP is used:

    SCOP-defined domain boundaries are used, where available.

    SCOP reference:

    The domain boundaries used are currently those defined by the SCOP database (6) and a new web-based tool allows direct cross-linking from domains on the SCOP web site to the corresponding Pfam families.

  ### Attachments

  - Full Text PDF
  - [HTML] from oxfordjournals.org
- ## The Pfam protein families database

  |  |  |
  | --- | --- |
  | Type | Journal Article |
  | Author | Marco Punta |
  | Author | Penny C. Coggill |
  | Author | Ruth Y. Eberhardt |
  | Author | Jaina Mistry |
  | Author | John Tate |
  | Author | Chris Boursnell |
  | Author | Ningze Pang |
  | Author | Kristoffer Forslund |
  | Author | Goran Ceric |
  | Author | Jody Clements |
  | Author | Andreas Heger |
  | Author | Liisa Holm |
  | Author | Erik L. L. Sonnhammer |
  | Author | Sean R. Eddy |
  | Author | Alex Bateman |
  | Author | Robert D. Finn |
  | URL | http://nar.oxfordjournals.org/content/40/D1/D290.abstract |
  | Volume | 40 |
  | Issue | D1 |
  | Pages | D290-D301 |
  | Publication | Nucleic Acids Research |
  | Date | 2012 |
  | DOI | 10.1093/nar/gkr1065 |
  | Abstract | Pfam is a widely used database of protein families, currently containing more than 13 000 manually curated protein families as of release 26.0. Pfam is available via servers in the UK (http://pfam.sanger.ac.uk/), the USA (http://pfam.janelia.org/) and Sweden (http://pfam.sbc.su.se/). Here, we report on changes that have occurred since our 2010 NAR paper (release 24.0). Over the last 2 years, we have generated 1840 new families and increased coverage of the UniProt Knowledgebase (UniProtKB) to nearly 80%. Notably, we have taken the step of opening up the annotation of our families to the Wikipedia community, by linking Pfam families to relevant Wikipedia pages and encouraging the Pfam and Wikipedia communities to improve and expand those pages. We continue to improve the Pfam website and add new visualizations, such as the ‘sunburst’ representation of taxonomic distribution of families. In this work we additionally address two topics that will be of particular interest to the Pfam community. First, we explain the definition and use of family-specific, manually curated gathering thresholds. Second, we discuss some of the features of domains of unknown function (also known as DUFs), which constitute a rapidly growing class of families within Pfam. |
  | Date Added | 10/11/2013, 10:20:13 AM |
  | Modified | 10/11/2013, 10:20:13 AM |

  ### Notes:

  - Corresponding protein on the SCOP website linked in the infobox of a protein family/entry

    How SCOP is used:

    SCOP data is not used directly.  SCOP links are provided in Pfam site.

    SCOP reference:

    "Further information was  
    extracted from the Pfam database and added to the  
    infobox, such as the Pfam clan accession and links to  
    other database sites such as PROSITE (10), SCOP (11)  
    and CAZy (12)."

    11. Murzin,A.G., Brenner,S.E., Hubbard,T. and Chothia,C. (1995)  
    SCOP: a structural classification of proteins database for the  
    investigation of sequences and structures. J. Mol. Biol., 247,  
    536–540.

  ### Attachments

  - Attachment
  - [HTML] from oxfordjournals.org
  - Nucl. Acids Res.-2012-Punta-D290-301.pdf
  - PubMed entry
  - Snapshot
- ## The phylogenomic roots of modern biochemistry: origins of proteins, cofactors and protein biosynthesis

  |  |  |
  | --- | --- |
  | Type | Journal Article |
  | Author | Gustavo Caetano-Anollés |
  | Author | Kyung Mo Kim |
  | Author | Derek Caetano-Anollés |
  | URL | http://link.springer.com/article/10.1007/s00239-011-9480-1 |
  | Volume | 74 |
  | Issue | 1-2 |
  | Pages | 1–34 |
  | Publication | Journal of molecular evolution |
  | Date | 2012 |
  | Accessed | 9/20/2013, 1:17:19 PM |
  | Library Catalog | Google Scholar |
  | Short Title | The phylogenomic roots of modern biochemistry |
  | Date Added | 10/11/2013, 10:29:15 AM |
  | Modified | 3/7/2014, 12:15:21 PM |

  ### Tags:

  - Aminoacyl-tRNA synthetases
  - Non-ribosomal protein synthesis
  - Origin of life
  - Phylogenetic analysis
  - Protein domain structure
  - Ribonucleoprotein world

  ### Notes:

  - Study emergence of molecular functions by examining SCOP families

    How SCOP is used:

    Use SCOP and SUPERFAMILY data from 2,397 families from SCOP 1.73 to build a phylogenomic tree and determine the 54 "oldest" SCOP families.

    Then study the functions, ligands, and fold features of the 54 most ancient "FF domains" using SCOP/SUPERFAMILY domains.

    Description of SCOP use is very unclear.

     SCOP reference:

    In these studies, domains were defined at increasing levels of structural complexity and conservation. For example, trees of domain structure were generated (reviewed in Caetano-Anolle ́s et al. 2009a) at fold family (FF), fold superfamily (FSF), and fold (F) levels of the structural classification of proteins (SCOP; Murzin et al. 1995). FFs group protein structures that are homologous at sequence level and are unambiguously linked to specific molecular functions. FSFs group FFs with common structures and functions and offer high levels of certainty that proteins belonging to this hierarchical level share a common evolutionary origin (Yang et al. 2005). Fs group FSFs that share similarly arranged and topologically connected secondary structures, but that may not be neces- sarily related at the evolutionary level. FF and FSF levels are the most useful. Although proteins in FFs often diverge and obscure sequence similarities, the close packing of amino acid side chains in the buried core of the protein retains the same FSF folded structure.

    ...

    SCOP, the gold standard used to describe the complexity of proteins and to benchmark structural prediction methods, was used to define domain structure (Andreeva et al. 2008). SCOP was selected because it partitions proteins into fewer and larger components than other structural classifications and takes into account both functional and evolutionary considerations (Holland et al. 2006). The structures of 2,397 FFs (out of 3,464 defined by SCOP 1.73) were assigned to genomic sequences using linear HMMs of structural rec- ognition in SUPERFAMILY (Gough et al. 2001; Wilson et al. 2009) with a probability cutoff of 10-4.

  ### Attachments

  - art%3A10.1007%2Fs00239-011-9480-1.pdf
  - Snapshot

    ## Abstract

    The complexity of modern biochemistry developed gradually on early Earth as new molecules and structures populated the emerging cellular systems. Here, we generate a historical account of the gradual discovery of primordial proteins, cofactors, and molecular functions using phylogenomic information in the sequence of 420 genomes. We focus on structural and functional annotations of the 54 most ancient protein domains. We show how primordial functions are linked to folded structures and how their interaction with cofactors expanded the functional repertoire. We also reveal protocell membranes played a crucial role in early protein evolution and show translation started with RNA and thioester cofactor-mediated aminoacylation. Our findings allow elaboration of an evolutionary model of early biochemistry that is firmly grounded in phylogenomic information and biochemical, biophysical, and structural knowledge. The model describes how primordial α-helical bundles stabilized membranes, how these were decorated by layered arrangements of β-sheets and α-helices, and how these arrangements became globular. Ancient forms of aminoacyl-tRNA synthetase (aaRS) catalytic domains and ancient non-ribosomal protein synthetase (NRPS) modules gave rise to primordial protein synthesis and the ability to generate a code for specificity in their active sites. These structures diversified producing cofactor-binding molecular switches and barrel structures. Accretion of domains and molecules gave rise to modern aaRSs, NRPS, and ribosomal ensembles, first organized around novel emerging cofactors (tRNA and carrier proteins) and then more complex cofactor structures (rRNA). The model explains how the generation of protein structures acted as scaffold for nucleic acids and resulted in crystallization of modern translation.
- ## The plant proteome folding project: structure and positive selection in plant protein families

  |  |  |
  | --- | --- |
  | Type | Journal Article |
  | Author | M. M. Pentony |
  | Author | P. Winters |
  | Author | D. Penfold-Brown |
  | Author | K. Drew |
  | Author | A. Narechania |
  | Author | R. DeSalle |
  | Author | R. Bonneau |
  | Author | M. D. Purugganan |
  | URL | http://gbe.oxfordjournals.org/content/4/3/360.short |
  | Volume | 4 |
  | Issue | 3 |
  | Pages | 360–371 |
  | Publication | Genome biology and evolution |
  | Date | 2012 |
  | Accessed | 9/20/2013, 1:12:22 PM |
  | Library Catalog | Google Scholar |
  | Short Title | The plant proteome folding project |
  | Date Added | 10/11/2013, 10:29:15 AM |
  | Modified | 10/11/2013, 10:29:15 AM |

  ### Tags:

  - adaptation
  - Evolution, Molecular
  - fold prediction
  - plant evolution
  - Plant Proteins
  - Protein Folding
  - protein structure
  - Proteome
  - Selection, Genetic

  ### Notes:

  - Present the Plant Proteome Folding Project, "a database with predicted protein domains for five plant proteomes (http://pfp.bio.nyu.edu)"

    How SCOP is used:

    1. Use SCOP to label data in plant protein database.

    Get "structural information" for protein domains in the database.  Don't specify whether they're getting domain boundaries or what classification levels are used.

    2. Validated their de novo structure prediction method on SCOP data using the superfamily level.

    SCOP reference:

    We also obtained Structural Classification of Proteins (SCOP) structural infor- mation. The SCOP (Murzin et al. 1995) database uses manual inspection, with the help of automated methods, to predicted structural and evolutionary relatedness.

    ...

    Under Results - Database Overview:

    To evaluate the accuracy of high-confidence structure predictions, a double-blind benchmarking of the structural analyses methods were used, and these correctly predicted 47% of structures using SCOP (v1.67) superfamily classifications (Drew et al. 2011), which is high for computational structure prediction.

  ### Attachments

  - Genome Biol Evol-2012-Pentony-360-71.pdf
  - PubMed entry
- ## The prediction of protein structural class using averaged chemical shifts

  |  |  |
  | --- | --- |
  | Type | Journal Article |
  | Author | Hao Lin |
  | Author | Chen Ding |
  | Author | Qiang Song |
  | Author | Ping Yang |
  | Author | Hui Ding |
  | Author | Ke-Jun Deng |
  | Author | Wei Chen |
  | Volume | 29 |
  | Issue | 6 |
  | Pages | 643-649 |
  | Publication | Journal of Biomolecular Structure & Dynamics |
  | ISSN | 0739-1102 |
  | Date | 2012 |
  | Extra | WOS:000303566400005 |
  | DOI | 10.1080/07391102.2011.672628 |
  | Abstract | Knowledge of protein structural class can provide important information about its folding patterns. Many approaches have been developed for the prediction of protein structural classes. However, the information used by these approaches is primarily based on amino acid sequences. In this study, a novel method is presented to predict protein structural classes by use of chemical shift (CS) information derived from nuclear magnetic resonance spectra. Firstly, 399 non-homologue (about 15% identity) proteins were constructed to investigate the distribution of averaged CS values of six nuclei ((CO)-C-13, C-13 alpha, C-13 beta, (HN)-H-1, H-1 alpha and N-15) in three protein structural classes. Subsequently, support vector machine was proposed to predict three protein structural classes by using averaged CS information of six nuclei. Overall accuracy of jackknife cross-validation achieves 87.0%. Finally, the feature selection technique is applied to exclude redundant information and find out an optimized feature set. Results show that the overall accuracy increased to 88.0% by using the averaged CSs of (CO)-C-13, H-1 alpha and N-15. The proposed approach outperformed other state-of-the-art methods in terms of predictive accuracy in particular for low-similarity protein data. We expect that our proposed approach will be an excellent alternative to traditional methods for protein structural class prediction. |
  | Date Added | 2/13/2014, 4:13:17 PM |
  | Modified | 3/7/2014, 12:14:22 PM |

  ### Notes:

  - Present method for predicting protein structural class with chemical shift data.

    How SCOP/CATH is used:

    Background on protein structure classification.  Mention that in this study, they predicted only mixed ab class, but in the future, they would like to distinguish between a+b and a/b classes.

    SCOP reference:

    On the basis of classification by SCOP database (Andreeva et al., 2008), most works have focused on predicting four classes of protein structural classes: all α, all β, α+β and α/β. The latter two classes are different in the aspect of the secondary structure connectivity, which is considered at a lower level describing topology (Orengo et al., 1997). Thus, we studied and predicted three major classes: all α, all β, mixed αβ according to the classification defined by CATH database (Orengo et al., 1997). Another important reason is that the num- ber of α/β class (10 proteins in benchmark data-set) is too few to have statistical significance. In the future work, we shall collect sufficient α/β proteins to investi- gate the difference of ACSs between α + β and α/β.

  ### Attachments

  - 07391102%2E2011%2E672628.pdf
- ## The protein data bank

  |  |  |
  | --- | --- |
  | Type | Journal Article |
  | Author | Helen M. Berman |
  | Author | Tammy Battistuz |
  | Author | T. N. Bhat |
  | Author | Wolfgang F. Bluhm |
  | Author | Philip E. Bourne |
  | Author | Kyle Burkhardt |
  | Author | Zukang Feng |
  | Author | Gary L. Gilliland |
  | Author | Lisa Iype |
  | Author | Shri Jain |
  | URL | http://scripts.iucr.org/cgi-bin/paper?S0907444902003451 |
  | Volume | 58 |
  | Issue | 6 |
  | Pages | 899–907 |
  | Publication | Acta Crystallographica Section D: Biological Crystallography |
  | Date | 2002 |
  | Accessed | 10/10/2013, 1:19:17 PM |
  | Library Catalog | Google Scholar |
  | Date Added | 10/11/2013, 10:29:15 AM |
  | Modified | 10/11/2013, 10:29:15 AM |

  ### Notes:

  - The PDB was in 2002, and still is in 2013, the primary database for biomolecular structure data.

     How SCOP is used:

    Proved links to SCOP website for structural classification.

    SCOP reference:

    Since there is no agreement in the community at present as to a de facto standard method for protein structure alignment, the PDB's policy is to provide access to a variety of alignments and classification schemes (Murzin et al., 1995; Gibrat et al., 1996; Orengo et al., 1997; Holm & Sander, 1998; Shindyalov & Bourne, 1998).

  ### Attachments

  - [PDF] from researchgate.net
  - Snapshot
- ## The Protein Data Bank

  |  |  |
  | --- | --- |
  | Type | Journal Article |
  | Author | H M Berman |
  | Author | J Westbrook |
  | Author | Z Feng |
  | Author | G Gilliland |
  | Author | T N Bhat |
  | Author | H Weissig |
  | Author | I N Shindyalov |
  | Author | P E Bourne |
  | Volume | 28 |
  | Issue | 1 |
  | Pages | 235-242 |
  | Publication | Nucleic acids research |
  | ISSN | 0305-1048 |
  | Date | Jan 1, 2000 |
  | Extra | PMID: 10592235 |
  | Journal Abbr | Nucleic Acids Res. |
  | Library Catalog | NCBI PubMed |
  | Language | eng |
  | Abstract | The Protein Data Bank (PDB; http://www.rcsb.org/pdb/ ) is the single worldwide archive of structural data of biological macromolecules. This paper describes the goals of the PDB, the systems in place for data deposition and access, how to obtain further information, and near-term plans for the future development of the resource. |
  | Date Added | 10/11/2013, 10:29:15 AM |
  | Modified | 10/11/2013, 10:29:15 AM |

  ### Tags:

  - Databases, Factual
  - Information Storage and Retrieval
  - Internet
  - Magnetic Resonance Spectroscopy
  - Protein Conformation
  - Proteins

  ### Notes:

  - Describes updates to the PDB.

    How SCOP is used:

    PDB website provides cross-link to SCOP for "Structure classification".

    SCOP reference:

    listed in a table of "Static cross-links to other data resources currently provided by the PDB".  In particular, the "Information Content" listed for SCOP is "Structure classification".

  ### Attachments

  - [HTML] from oxfordjournals.org
  - Nucl. Acids Res.-2000-Berman-235-42.pdf
  - Snapshot
- ## The Protein-Folding Problem, 50 Years On

  |  |  |
  | --- | --- |
  | Type | Journal Article |
  | Author | Ken A. Dill |
  | Author | Justin L. MacCallum |
  | URL | http://www.sciencemag.org/content/338/6110/1042 |
  | Volume | 338 |
  | Issue | 6110 |
  | Pages | 1042-1046 |
  | Publication | Science |
  | ISSN | 0036-8075, 1095-9203 |
  | Date | 11/23/2012 |
  | Extra | PMID: 23180855 |
  | Journal Abbr | Science |
  | DOI | 10.1126/science.1219021 |
  | Accessed | 9/5/2013, 3:31:23 PM |
  | Library Catalog | www.sciencemag.org |
  | Language | en |
  | Abstract | The protein-folding problem was first posed about one half-century ago. The term refers to three broad questions: (i) What is the physical code by which an amino acid sequence dictates a protein’s native structure? (ii) How can proteins fold so fast? (iii) Can we devise a computer algorithm to predict protein structures from their sequences? We review progress on these problems. In a few cases, computer simulations of the physical forces in chemically detailed models have now achieved the accurate folding of small proteins. We have learned that proteins fold rapidly because random thermal motions cause conformational changes leading energetically downhill toward the native structure, a principle that is captured in funnel-shaped energy landscapes. And thanks in part to the large Protein Data Bank of known structures, predicting protein structures is now far more successful than was thought possible in the early days. What began as three questions of basic science one half-century ago has now grown into the full-fledged research field of protein physical science. |
  | Date Added | 2/20/2014, 12:24:01 PM |
  | Modified | 2/20/2014, 12:24:01 PM |

  ### Notes:

  - Surveys the research undertaken to solve "the protein-folding problem" and argues for protein folding is now its own large field of research.

    How SCOP is used:

    Mentions SCOP in order to point out the that there are only about 4000 distinct known folds.  Most structure prediction methods rely on homology modeling.

    SCOP reference:

    "Currently, all successful structure-prediction algorithms are based on assuming that similar sequences lead to similar structures. These methods draw heavily on the PDB, which now contains more than 80,000 structures. However, many of these structures are similar, and the PDB contains only ~4000 structural families and 1200 folds (*30*)."

  ### Attachments

  - Full Text PDF
  - [PDF] from psu.edu
  - PubMed entry
  - Snapshot
  - Snapshot
- ## The putative alpha/beta-hydrolases of Dietzia cinnamea P4 strain as potential enzymes for biocatalytic applications

  |  |  |
  | --- | --- |
  | Type | Journal Article |
  | Author | Luciano Procopio |
  | Author | Andrew Macrae |
  | Author | Jan Dirk van Elsas |
  | Author | Lucy Seldin |
  | Volume | 103 |
  | Issue | 3 |
  | Pages | 635–646 |
  | Publication | Antonie Van Leeuwenhoek International Journal of General and Molecular Microbiology |
  | Date | March 2013 |
  | DOI | 10.1007/s10482-012-9847-3 |
  | Abstract | The draft genome of the soil actinomycete Dietzia cinnamea P4 reveals a versatile group of alpha/beta-hydrolase fold enzymes. Phylogenetic and comparative sequence analyses were used to classify the alpha/beta-hydrolases of strain P4 into six different groups: (i) lipases, (ii) esterases, (iii) epoxide hydrolases, (iv) haloacid dehalogenases, (v) C-C breaking enzymes and (vi) serine peptidases. The high number of lipases/esterases (41) and epoxide hydrolase enzymes (14) present in the relatively small (3.6 Mb) P4 genome is unusual; it is likely to be linked to the survival of strain P4 in its natural environment. Strain P4 is thus equipped with a large number of genes which would appear to confer survivability in harsh hot tropical soil. As such, this highly resilient soil bacterial strain provides an interesting genome for enzyme mining for applications in the field of biotransformations of polymeric compounds. |
  | Date Added | 3/7/2014, 12:08:00 PM |
  | Modified | 3/7/2014, 12:08:00 PM |
- ## The RCSB Protein Data Bank: new resources for research and education

  |  |  |
  | --- | --- |
  | Type | Journal Article |
  | Author | Peter W. Rose |
  | Author | Chunxiao Bi |
  | Author | Wolfgang F. Bluhm |
  | Author | Cole H. Christie |
  | Author | Dimitris Dimitropoulos |
  | Author | Shuchismita Dutta |
  | Author | Rachel K. Green |
  | Author | David S. Goodsell |
  | Author | Andreas Prlić |
  | Author | Martha Quesada |
  | URL | http://nar.oxfordjournals.org/content/41/D1/D475.short |
  | Volume | 41 |
  | Issue | D1 |
  | Pages | D475–D482 |
  | Publication | Nucleic Acids Research |
  | Date | 2013 |
  | Accessed | 9/20/2013, 1:12:22 PM |
  | Library Catalog | Google Scholar |
  | Short Title | The RCSB Protein Data Bank |
  | Date Added | 10/11/2013, 10:20:13 AM |
  | Modified | 3/7/2014, 12:08:59 PM |

  ### Tags:

  - coverage

  ### Notes:

  - Research Collaboratory for Structural Bioinformatics  
    Protein Data Bank

    Paper detailing new resources for their databank

    -Uses the SCOP hierarchy as an option for browsing

    -SCOP 1.75 domains used for structural alignments where available using The Protein Comparison Tool

    -Each PDB entry includes a SCOP annotation of the domain (domain and protein)

    **Quotes**

    "The ‘Browse Database’  
    option allows exploration of the PDB archive using different  
    hierarchical trees. Browsers are available to search  
    for related terms and structures based on many different  
    classifications, such as Biological Process, Cellular  
    Component, Molecular Function (8), Enzyme Commission  
    number (http://www.chem.qmul.ac.uk/iubmb),  
    Transporter Classification System (9), and structure  
    classifications SCOP (10) and CATH (11). "

    **Under structural alignment**

    "The calculation based on domains extends our sequence  
    clustering approach. To remove redundancy, we start with a  
    40% sequence identity clustering procedure based on  
    complete polypeptide chains, and select a representative  
    chain from each sequence cluster (3). If the representative  
    chain contains multiple domains, each is included. SCOP  
    1.75 domain assignments are used when available; otherwise,  
    assignments are computed using ProteinDomain  
    Parser (PDP) (29). Pairwise alignments of the domains are  
    performed with the jFatCat version (24) of FatCat (22).  
    For each PDB entry, the ‘3D Similarity’ tab provides a  
    visual summary of the protein chains. Figure 2 highlights  
    how the residues listed in the sequence (SEQRES) and in  
    the atom records (ATOM) map onto the relevant parts of  
    the UniProtKB sequence, along with annotations from  
    DSSP (32), SCOP, PDP (29) and Pfam (33)."

    **Citation**10. Murzin,A.G., Brenner,S.E., Hubbard,T. and Chothia,C. (1995)  
    SCOP: a structural classification of proteins database for the  
    investigation of sequences and structures. J. Mol. Biol., 247,  
    536–540.

  ### Attachments

  - [HTML] from oxfordjournals.org
  - Nucl. Acids Res.-2013-Rose-D475-82.pdf
  - Snapshot
- ## The Repertoires of Ubiquitinating and Deubiquitinating Enzymes in Eukaryotic Genomes

  |  |  |
  | --- | --- |
  | Type | Journal Article |
  | Author | Andrew Paul Hutchins |
  | Author | Shaq Liu |
  | Author | Diego Diez |
  | Author | Diego Miranda-Saavedra |
  | Volume | 30 |
  | Issue | 5 |
  | Pages | 1172-1187 |
  | Publication | Molecular Biology and Evolution |
  | ISSN | 0737-4038 |
  | Date | MAY 2013 |
  | Extra | WOS:000318165700017 |
  | DOI | 10.1093/molbev/mst022 |
  | Abstract | Reversible protein ubiquitination regulates virtually all known cellular activities. Here, we present a quantitatively evaluated and broadly applicable method to predict eukaryotic ubiquitinating enzymes (UBE) and deubiquitinating enzymes (DUB) and its application to 50 distinct genomes belonging to four of the five major phylogenetic supergroups of eukaryotes: unikonts (including metazoans, fungi, choanozoa, and amoebozoa), excavates, chromalveolates, and plants. Our method relies on a collection of profile hidden Markov models, and we demonstrate its superior performance (coverage and classification accuracy > 99%) by identifying approximately 25% and approximately 35% additional UBE and DUB genes in yeast and human, which had not been reported before. In yeast, we predict 85 UBE and 24 DUB genes, for 814 UBE and 107 DUB genes in the human genome. Most UBE and DUB families are present in all eukaryotic lineages, with plants and animals harboring massively enlarged repertoires of ubiquitin ligases. Unicellular organisms, on the other hand, typically harbor less than 300 UBEs and less than 40 DUBs per genome. Ninety-one UBE/DUB genes are orthologous across all four eukaryotic supergroups, and these likely represent a primordial core of enzymes of the ubiquitination system probably dating back to the first eukaryotes approximately 2 billion years ago. Our genome-wide predictions are available through the Database of Ubiquitinating and Deubiquitinating Enzymes ( ext-link-type="uri" xlink:href="http://www.DUDE-db.org" xmlns:xlink="http://www.w3.org/1999/xlink">www.DUDE-db.org), where users can also perform advanced sequence and phylogenetic analyses and submit their own predictions. |
  | Date Added | 2/20/2014, 12:24:01 PM |
  | Modified | 2/20/2014, 12:24:01 PM |

  ### Notes:

  - Present method for predicting eukaryotic ubiquitinating enzymes (EUB) and deubiquitinating enzymes (DUB).

    How SCOP is used:

    Get SUPERFAMILY domains for their data set and use family-level classification.

    SCOP reference:

    Analysis of the protein domain signatures of the working  
     data set led to the identification of a minimal group of 33  
     distinct protein domain signatures that allows the automatic  
     retrieval and correct family-level classification of all the en-  
     zymes in the working data set (supplementary table S4, Supplementary Material online). We call this collection of  
     HMMs the “UBE/DUB HMM Library." We chose to combine  
     HMMs from Pfam (Punta et al. 2012), SMART (Letunic et al.  
     2012), and SUPERFAMILY (Wilson et al. 2009) as these data-  
     bases differ in their contents and therefore their coverage.  
     Pfam (release 26.0) is a large collection of more than 13,000  
     protein family alignments, whereas SMART (version 7) con-  
     tains models for more than 1,000 protein domains. Although  
     Pfam and SMART are built from manually curated alignments  
     of multiple protein sequences, **SUPERFAMILY is based on the sequences of domains with known three-dimensional struc-**  
     **ture as contained in the SCOP database (****Andreeva et al.  
     2008****).** Therefore, the integration of HMMs from the three  
     databases leads to an improvement in the predictive power of  
     the method when compared with using any individual data-  
     base in isolation.

  ### Attachments

  - Mol Biol Evol-2013-Hutchins-1172-87.pdf
- ## Thermal stability limits of proteins in solution and adsorbed on a hydrophobic surface

  |  |  |
  | --- | --- |
  | Type | Journal Article |
  | Author | Yevgeny Moskovitz |
  | Author | Simcha Srebnik |
  | URL | http://pubs.rsc.org/en/content/articlehtml/2012/cp/c2cp00005a |
  | Volume | 14 |
  | Issue | 22 |
  | Pages | 8013–8022 |
  | Publication | Physical Chemistry Chemical Physics |
  | Date | 2012 |
  | Accessed | 9/23/2013, 10:17:26 AM |
  | Library Catalog | Google Scholar |
  | Date Added | 10/11/2013, 10:29:15 AM |
  | Modified | 10/11/2013, 10:29:15 AM |

  ### Notes:

  - Study thermal denaturation of small proteins using Monte Carlo simulation.

    How SCOP is used:

    Not using SCOP data.  Cite SCOP when discussing representations of 3D proteins in 2D contact maps.  Not sure why.

    SCOP reference:

    (SCOP paper is 32.)

    The three-dimensional structure of proteins is often represented by a two-dimensional contact map of nearest neighbor monomers, where a residue–residue contact is defined according to some distance threshold, usually in the range of 7.5–10 A ̊ 32 and with different levels of details.33,34

  ### Attachments

  - C2CP00005A.pdf
  - Snapshot

    A coarse-grained Monte Carlo simulation is used to study thermal denaturation of small proteins in an infinitely dilute solution and adsorbed on a flat hydrophobic surface. Intermolecular interactions are modeled using the Miyazawa–Jernigan (MJ) knowledge-based potential for implicit solvent with the BULDG hydrophobicity scale. We analyze the thermal behavior of lysozyme for its prevalence of α-helices, fibronectin for its prevalence of β-sheets, and a short single helical peptide. Protein dimensions and contact maps are studied in detail before and during isothermal adsorption and heating. The MJ potential is shown to correctly predict the native conformation in solution under standard conditions, and the anticipated thermal stabilization of adsorbed proteins is observed when compared with heating in solution. The helix of the peptide is found to be much less stable thermally than the helices of lysozyme, reinforcing the importance of long-range forces in defining the protein structure. Contact map analysis of the adsorbed proteins shows correlation between the hydrophobicity of the secondary structure and their thermal stability on the surface.
- ## The role of DNA shape in protein-DNA recognition

  |  |  |
  | --- | --- |
  | Type | Journal Article |
  | Author | Remo Rohs |
  | Author | Sean M West |
  | Author | Alona Sosinsky |
  | Author | Peng Liu |
  | Author | Richard S Mann |
  | Author | Barry Honig |
  | Volume | 461 |
  | Issue | 7268 |
  | Pages | 1248-1253 |
  | Publication | Nature |
  | ISSN | 1476-4687 |
  | Date | Oct 29, 2009 |
  | Extra | PMID: 19865164 |
  | Journal Abbr | Nature |
  | DOI | 10.1038/nature08473 |
  | Library Catalog | NCBI PubMed |
  | Language | eng |
  | Abstract | The recognition of specific DNA sequences by proteins is thought to depend on two types of mechanism: one that involves the formation of hydrogen bonds with specific bases, primarily in the major groove, and one involving sequence-dependent deformations of the DNA helix. By comprehensively analysing the three-dimensional structures of protein-DNA complexes, here we show that the binding of arginine residues to narrow minor grooves is a widely used mode for protein-DNA recognition. This readout mechanism exploits the phenomenon that narrow minor grooves strongly enhance the negative electrostatic potential of the DNA. The nucleosome core particle offers a prominent example of this effect. Minor-groove narrowing is often associated with the presence of A-tracts, AT-rich sequences that exclude the flexible TpA step. These findings indicate that the ability to detect local variations in DNA shape and electrostatic potential is a general mechanism that enables proteins to use information in the minor groove, which otherwise offers few opportunities for the formation of base-specific hydrogen bonds, to achieve DNA-binding specificity. |
  | Date Added | 10/11/2013, 10:29:15 AM |
  | Modified | 10/11/2013, 10:29:15 AM |

  ### Tags:

  - Animals
  - Arginine
  - AT Rich Sequence
  - Base Sequence
  - Databases, Factual
  - DNA
  - DNA-Binding Proteins
  - Hydrogen Bonding
  - Lysine
  - Nucleic Acid Conformation
  - Nucleosomes
  - Protein Binding
  - Saccharomyces cerevisiae
  - Static Electricity

  ### Notes:

  - Study of 3D structures of protein-DNA complexes.

    How SCOP is used:

    To classify a data set of DNA binding proteins by superfamily.  if the protein was not in SCOP use ASTRAL (ASTEROIDs?).

    SCOP reference:

    Under methods:

    Structural annotation of DNA-binding proteins. The proteins in our data set of protein–DNA complexes were classified in SCOP46 superfamilies. Proteins for which SCOP annotations were not available were annotated manually or using the ASTRAL database49.

  ### Attachments

  - nature08473.pdf
  - PubMed entry
- ## The Role of Non-Native Interactions in the Folding of Knotted Proteins

  |  |  |
  | --- | --- |
  | Type | Journal Article |
  | Author | Tatjana Skrbic |
  | Author | Cristian Micheletti |
  | Author | Pietro Faccioli |
  | Volume | 8 |
  | Issue | 6 |
  | Pages | e1002504 |
  | Publication | Plos Computational Biology |
  | ISSN | 1553-7358 |
  | Date | JUN 2012 |
  | Extra | WOS:000305965300004 |
  | DOI | 10.1371/journal.pcbi.1002504 |
  | Abstract | Stochastic simulations of coarse-grained protein models are used to investigate the propensity to form knots in early stages of protein folding. The study is carried out comparatively for two homologous carbamoyltransferases, a natively-knotted N-acetylornithine carbamoyltransferase (AOTCase) and an unknotted ornithine carbamoyltransferase (OTCase). In addition, two different sets of pairwise amino acid interactions are considered: one promoting exclusively native interactions, and the other additionally including non-native quasi-chemical and electrostatic interactions. With the former model neither protein shows a propensity to form knots. With the additional non-native interactions, knotting propensity remains negligible for the natively-unknotted OTCase while for AOTCase it is much enhanced. Analysis of the trajectories suggests that the different entanglement of the two transcarbamylases follows from the tendency of the C-terminal to point away from (for OTCase) or approach and eventually thread (for AOTCase) other regions of partly-folded protein. The analysis of the OTCase/AOTCase pair clarifies that natively-knotted proteins can spontaneously knot during early folding stages and that non-native sequence-dependent interactions are important for promoting and disfavouring early knotting events. |
  | Date Added | 2/13/2014, 4:13:17 PM |
  | Modified | 3/7/2014, 12:11:04 PM |
- ## The role of structural bioinformatics resources in the era of integrative structural biology

  |  |  |
  | --- | --- |
  | Type | Journal Article |
  | Author | Aleksandras Gutmanas |
  | Author | Thomas J. Oldfield |
  | Author | Ardan Patwardhan |
  | Author | Sanchayita Sen |
  | Author | Sameer Velankar |
  | Author | Gerard J. Kleywegt |
  | Volume | 69 |
  | Pages | 710-721 |
  | Publication | Acta Crystallographica Section D-Biological Crystallography |
  | ISSN | 0907-4449 |
  | Date | MAY 2013 |
  | Extra | WOS:000318240200005 |
  | DOI | 10.1107/S0907444913001157 |
  | Abstract | The history and the current state of the PDB and EMDB archives is briefly described, as well as some of the challenges that they face. It seems natural that the role of structural biology archives will change from being a pure repository of historic data into becoming an indispensable resource for the wider biomedical community. As part of this transformation, it will be necessary to validate the biomacromolecular structure data and ensure the highest possible quality for the archive holdings, to combine structural data from different spatial scales into a unified resource and to integrate structural data with functional, genetic and taxonomic data as well as other information available in bioinformatics resources. Some recent developments and plans to address these challenges at PDBe are presented. |
  | Date Added | 2/20/2014, 12:24:01 PM |
  | Modified | 3/7/2014, 12:09:41 PM |

  ### Notes:

  - Review of history and use structural bioinformatics resources (namely PDB and EMDB).

    How SCOP/CATH is used:

    Background on protein structure classification.

    Mention that PDBe and Uniprot integrate SCOP data.

    SCOP reference:

    For over a decade, PDBe and UniProt (UniProt Consor- tium, 2012) have worked together to integrate information from protein sequences and structures, resulting in a data resource called SIFTS (Structure Integration with Function, Taxonomy and Sequences; Velankar et al., 2005, 2013). This resource provides up-to-date residue-level annotation of protein structures in the PDB with data available from UniProt, InterPro (Hunter et al., 2009), Pfam (Punta et al., 2012), GO (Ashburner et al., 2000), CATH (Cuff et al., 2011) and SCOP (Andreeva et al., 2008).

  ### Attachments

  - ic5087.pdf
- ## The sequence and structure of snake gourd (Trichosanthes anguina) seed lectin, a three-chain nontoxic homologue of type II RIPs

  |  |  |
  | --- | --- |
  | Type | Journal Article |
  | Author | Alok Sharma |
  | Author | Gottfried Pohlentz |
  | Author | Kishore Babu Bobbili |
  | Author | A. Arockia Jeyaprakash |
  | Author | Thyageshwar Chandran |
  | Author | Michael Mormann |
  | Author | Musti J. Swamy |
  | Author | M. Vijayan |
  | URL | http://scripts.iucr.org/cgi-bin/paper?be5225 |
  | Volume | 69 |
  | Issue | 8 |
  | Pages | 0–0 |
  | Publication | Acta Crystallographica Section D: Biological Crystallography |
  | Date | 2013 |
  | Accessed | 9/23/2013, 10:21:39 AM |
  | Library Catalog | Google Scholar |
  | Date Added | 10/11/2013, 10:29:15 AM |
  | Modified | 10/11/2013, 10:29:15 AM |

  ### Notes:

  - The sequence and structure of snake gourd seed lectin (SGSL), a nontoxic homologue of type II ribosome-inactivating proteins (RIPs), have been determined by mass spectrometry and X-ray crystallography, respectively.

    The sequence and structure of snake gourd seed lectin (SGSL), a nontoxic homologue of type II ribosome-inactivating proteins (RIPs), have been determined by mass spectrometry and X-ray crystallography, respectively.

    How SCOP is used:

    Use case: Provide background on the fold that the protein of interest falls under (beta trefoil).

    Description: Help guide search for homologs to narrow down to a smaller set. Did not use SCOP data to search for homologs.

    SCOP reference:

    2.8. Sequence homologues and evolutionary implications

    ...

    A global search of amino-acid sequences using the sequence of the lectin subunit of SGSL, employing the criteria mentioned in x2, resulted in the identification of 160 proteins with at least one carbohydrate-binding site. The ⬚⬚-trefoil fold is known to exhibit substantial functional diversity (Murzin et al., 1995). Therefore, the 160 sequences were further searched for the presence of the catalytic domain using the CDD web server available at the NCBI. This resulted in the identification of 30 proteins containing the lectin as well as the catalytic chains.

  ### Attachments

  - be5225.pdf
- ## The structure of a glycoside hydrolase family 81 endo-beta-1,3-glucanase

  |  |  |
  | --- | --- |
  | Type | Journal Article |
  | Author | Peng Zhou |
  | Author | Zhongzhou Chen |
  | Author | Qiaojuan Yan |
  | Author | Shaoqing Yang |
  | Author | Rolf Hilgenfeld |
  | Author | Zhengqiang Jiang |
  | Volume | 69 |
  | Pages | 2027-2038 |
  | Publication | Acta Crystallographica Section D-Biological Crystallography |
  | ISSN | 0907-4449 |
  | Date | OCT 2013 |
  | Extra | WOS:000325403900016 |
  | DOI | 10.1107/S090744491301799X |
  | Abstract | Endo-beta-1,3-glucanases catalyze the hydrolysis of beta-1,3-glycosidic linkages in glucans. They are also responsible for rather diverse physiological functions such as carbon utilization, cell-wall organization and pathogen defence. Glycoside hydrolase (GH) family 81 mainly consists of beta-1,3-glucanases from fungi, higher plants and bacteria. A novel GH family 81 beta-1,3-glucanase gene (RmLam81A) from Rhizomucor miehei was expressed in Escherichia coli. Purified RmLam81A was crystallized and the structure was determined in two crystal forms (form I-free and form II-Se) at 2.3 and 2.0 angstrom resolution, respectively. Here, the crystal structure of a member of GH family 81 is reported for the first time. The structure of RmLam81A is greatly different from all endo-beta-1,3-glucanase structures available in the Protein Data Bank. The overall structure of the RmLam81A monomer consists of an N-terminal beta-sandwich domain, a C-terminal (alpha/alpha)(6) domain and an additional domain between them. Glu553 and Glu557 are proposed to serve as the proton donor and basic catalyst, respectively, in a single-displacement mechanism. In addition, Tyr386, Tyr482 and Ser554 possibly contribute to both the position or the ionization state of the basic catalyst Glu557. The first crystal structure of a GH family 81 member will be helpful in the study of the |
  | Date Added | 2/12/2014, 2:18:08 PM |
  | Modified | 2/12/2014, 2:18:08 PM |

  ### Notes:

  - Present crystal structure of a glycoside hydrolase - RmLam81A - in e. coli.

    How SCOP is used:

    Listed out the superfamilies that the domains should be classified into.  Seems to have done this by browsing and using DALI.

    SCOP reference:

    Domain A of RmLam81A consists of a core of two eight-stranded antiparallel ⬚⬚-sheets with orders ⬚⬚2–⬚⬚3–⬚⬚4–⬚⬚7–⬚⬚12– ⬚⬚18–⬚⬚17–⬚⬚14 and ⬚⬚8–⬚⬚9–⬚⬚10–⬚⬚11–⬚⬚19–⬚⬚16–⬚⬚15–⬚⬚13 packed on top of one another. According to the structural classifica- tion of proteins (SCOP; http://scop.berkeley.edu), this ⬚⬚- sandwich is grouped into the supersandwich superfamily, which contains 18 strands in two sheets (Murzin et al., 1995).

    ...

    Domain C of RmLam81A is comprised of a core of (⬚⬚/⬚⬚)6- barrel topology consisting of a double barrel of ⬚⬚-helices with the C-terminus of the outer helix leading into the N-terminus of an inner helix. According to the structural classification of proteins (SCOP), this (⬚⬚/⬚⬚)6-barrel, which is common in glycosyl hydrolases, polysaccharide lyases and terpenoid cylases/protein prenyltransferases, is grouped into the six- hairpin enzyme superfamily (Murzin et al., 1995). **A structure- homologue search using the** **DALI server shows that domain C shares the highest structural similarity to the GH family 88 Bacillus sp. GL1 glycosaminoglycan (PDB entry 1vd5; Z-score 15.8; Itoh et al., 2004) and the GH family 8 xylanase of Pseudoalteromonas haloplanktis (PDB entry 1h14; Z-score 15.2; Van Petegem et al., 2003). Both these proteins show the same (⬚⬚/⬚⬚)6****-barrel fold found in the six-hairpin enzyme superfamily of the SCOP database.**

  ### Attachments

  - dz5286.pdf
- ## The structure of latherin, a surfactant allergen protein from horse sweat and saliva

  |  |  |
  | --- | --- |
  | Type | Journal Article |
  | Author | Steven J. Vance |
  | Author | Rhona E. McDonald |
  | Author | Alan Cooper |
  | Author | Brian O. Smith |
  | Author | Malcolm W. Kennedy |
  | Volume | 10 |
  | Issue | 85 |
  | Publication | JOURNAL OF THE ROYAL SOCIETY INTERFACE |
  | ISSN | 1742-5689 |
  | Date | AUG 6 2013 |
  | DOI | 10.1098/rsif.2013.0453 |
  | Language | English |
  | Abstract | Latherin is a highly surface-active allergen protein found in the sweat and saliva of horses and other equids. Its surfactant activity is intrinsic to the protein in its native form, and is manifest without associated lipids or glycosylation. Latherin probably functions as a wetting agent in evaporative cooling in horses, but it may also assist in mastication of fibrous food as well as inhibition of microbial biofilms. It is a member of the PLUNC family of proteins abundant in the oral cavity and saliva of mammals, one of which has also been shown to be a surfactant and capable of disrupting microbial biofilms. How these proteins work as surfactants while remaining soluble and cell membrane-compatible is not known. Nor have their structures previously been reported. We have used protein nuclear magnetic resonance spectroscopy to determine the conformation and dynamics of latherin in aqueous solution. The protein is a monomer in solution with a slightly curved cylindrical structure exhibiting a `super-roll' motif comprising a four-stranded anti-parallel beta-sheet and two opposing alpha-helices which twist along the long axis of the cylinder. One end of the molecule has prominent, flexible loops that contain a number of apolar amino acid side chains. This, together with previous biophysical observations, leads us to a plausible mechanism for surfactant activity in which the molecule is first localized to the non-polar interface via these loops, and then unfolds and flattens to expose its hydrophobic interior to the air or non-polar surface. Intrinsically surface-active proteins are relatively rare in nature, and this is the first structure of such a protein from mammals to be reported. Both its conformation and proposed method of action are different from other, non-mammalian surfactant proteins investigated so far. |
  | Date Added | 10/11/2013, 10:29:15 AM |
  | Modified | 3/7/2014, 12:08:41 PM |

  ### Tags:

  - horse
  - latherin
  - PLUNC proteins
  - surfactant protein
  - sweat

  ### Notes:

  - Use NMR to study dynamics and function of latherin, a surfactant allergen protein from horse sweat and saliva.

    How SCOP /CATHis used:

    Lookup fold classification of latherin.

    SCOP/CATH reference:

    As expected from amino acid sequence comparisons [17,23], database examinations identify the fold adopted by latherin as a BPI domain-like fold (SCOP; [52], code 55393) or as a super-roll (CATH; [53], code 3.15).

  ### Attachments

  - J. R. Soc. Interface-2013-Vance-.pdf
- ## The Transcriptome Profile of the Mosquito Culex quinquefasciatus following Permethrin Selection

  |  |  |
  | --- | --- |
  | Type | Journal Article |
  | Author | William R. Reid |
  | Author | Lee Zhang |
  | Author | Feng Liu |
  | Author | Nannan Liu |
  | Volume | 7 |
  | Issue | 10 |
  | Pages | e47163 |
  | Publication | Plos One |
  | ISSN | 1932-6203 |
  | Date | OCT 5 2012 |
  | Extra | WOS:000309827300085 |
  | Journal Abbr | PLoS One |
  | DOI | 10.1371/journal.pone.0047163 |
  | Library Catalog | ISI Web of Knowledge |
  | Language | English |
  | Abstract | To gain valuable insights into the gene interaction and the complex regulation system involved in the development of insecticide resistance in mosquitoes Culex quinquefasciatus, we conducted a whole transcriptome analysis of Culex mosquitoes following permethrin selection. Gene expression profiles for the lower resistant parental mosquito strain HAmCq(G0) and their permethrin-selected high resistant offspring HAmCq(G8) were compared and a total of 367 and 3982 genes were found to be up- and down-regulated, respectively, in HAmCq(G8), indicating that multiple genes are involved in response to permethrin selection. However, a similar overall cumulative gene expression abundance was identified between up- and down-regulated genes in HAmCq(G8) mosquitoes following permethrin selection, suggesting a homeostatic response to insecticides through a balancing of the up- and down-regulation of the genes. While structural and/or cuticular structural functions were the only two enriched GO terms for down-regulated genes, the enriched GO terms obtained for the up-regulated genes occurred primarily among the catalytic and metabolic functions where they represented three functional categories: electron carrier activity, binding, and catalytic activity. Interestingly, the functional GO terms in these three functional categories were overwhelmingly overrepresented in P450s and proteases/serine proteases. The important role played by P450s in the development of insecticide resistance has been extensively studied but the function of proteases/serine proteases in resistance is less well understood. Hence, the characterization of the functions of these proteins, including their digestive, catalytic and proteinase activities; regulation of signaling transduction and protein trafficking, immunity and storage; and their precise function in the development of insecticide resistance in mosquitoes will provide new insights into how genes are interconnected and regulated in resistance. |
  | Date Added | 10/8/2014, 12:34:47 PM |
  | Modified | 10/8/2014, 1:32:25 PM |

  ### Tags:

  - anopheles-gambiae
  - detoxification genes
  - differential expression
  - gene-expression
  - genomic analysis
  - insecticide resistance
  - musca-domestica
  - protease-activated receptors
  - pyrethroid-resistant
  - susceptible strains

  ### Attachments

  - PLoS Full Text PDF
  - PLoS Snapshot
- ## The UCSC Genome Browser database: 2014 update

  |  |  |
  | --- | --- |
  | Type | Journal Article |
  | Author | Donna Karolchik |
  | Author | Galt P. Barber |
  | Author | Jonathan Casper |
  | Author | Hiram Clawson |
  | Author | Melissa S. Cline |
  | Author | Mark Diekhans |
  | Author | Timothy R. Dreszer |
  | Author | Pauline A. Fujita |
  | Author | Luvina Guruvadoo |
  | Author | Maximilian Haeussler |
  | Author | Rachel A. Harte |
  | Author | Steve Heitner |
  | Author | Angie S. Hinrichs |
  | Author | Katrina Learned |
  | Author | Brian T. Lee |
  | Author | Chin H. Li |
  | Author | Brian J. Raney |
  | Author | Brooke Rhead |
  | Author | Kate R. Rosenbloom |
  | Author | Cricket A. Sloan |
  | Author | Matthew L. Speir |
  | Author | Ann S. Zweig |
  | Author | David Haussler |
  | Author | Robert M. Kuhn |
  | Author | W. James Kent |
  | Volume | 42 |
  | Issue | Database issue |
  | Pages | D764-770 |
  | Publication | Nucleic Acids Research |
  | ISSN | 1362-4962 |
  | Date | Jan 2014 |
  | Extra | PMID: 24270787 PMCID: PMC3964947 |
  | Journal Abbr | Nucleic Acids Res. |
  | DOI | 10.1093/nar/gkt1168 |
  | Library Catalog | NCBI PubMed |
  | Language | eng |
  | Abstract | The University of California Santa Cruz (UCSC) Genome Browser (http://genome.ucsc.edu) offers online public access to a growing database of genomic sequence and annotations for a large collection of organisms, primarily vertebrates, with an emphasis on the human and mouse genomes. The Browser's web-based tools provide an integrated environment for visualizing, comparing, analysing and sharing both publicly available and user-generated genomic data sets. As of September 2013, the database contained genomic sequence and a basic set of annotation 'tracks' for ∼90 organisms. Significant new annotations include a 60-species multiple alignment conservation track on the mouse, updated UCSC Genes tracks for human and mouse, and several new sets of variation and ENCODE data. New software tools include a Variant Annotation Integrator that returns predicted functional effects of a set of variants uploaded as a custom track, an extension to UCSC Genes that displays haplotype alleles for protein-coding genes and an expansion of data hubs that includes the capability to display remotely hosted user-provided assembly sequence in addition to annotation data. To improve European access, we have added a Genome Browser mirror (http://genome-euro.ucsc.edu) hosted at Bielefeld University in Germany. |
  | Short Title | The UCSC Genome Browser database |
  | Date Added | 10/10/2014, 4:35:16 PM |
  | Modified | 10/10/2014, 4:35:16 PM |

  ### Tags:

  - Alleles
  - Animals
  - Databases, Genetic
  - Genome
  - Genome, Human
  - Genomics
  - Humans
  - Internet
  - Mice
  - Molecular Sequence Annotation
  - Polymorphism, Single Nucleotide
  - Sequence Alignment
  - Software

  ### Attachments

  - PubMed entry
- ## The use of evolutionary patterns in protein annotation

  |  |  |
  | --- | --- |
  | Type | Journal Article |
  | Author | Angela D. Wilkins |
  | Author | Benjamin J. Bachman |
  | Author | Serkan Erdin |
  | Author | Olivier Lichtarge |
  | Volume | 22 |
  | Issue | 3 |
  | Pages | 316-325 |
  | Publication | Current Opinion in Structural Biology |
  | ISSN | 0959-440X |
  | Date | JUN 2012 |
  | Extra | WOS:000306347800010 |
  | DOI | 10.1016/j.sbi.2012.05.001 |
  | Abstract | With genomic data skyrocketing, their biological interpretation remains a serious challenge. Diverse computational methods address this problem by pointing to the existence of recurrent patterns among sequence, structure, and function. These patterns emerge naturally from evolutionary variation, natural selection, and divergence - the defining features of biological systems and they identify molecular events and shapes that underlie specificity of function and allosteric communication. Here we review these methods, and the patterns they identify in case studies and in proteome-wide applications, to infer and rationally redesign function. |
  | Date Added | 2/20/2014, 12:24:01 PM |
  | Modified | 3/7/2014, 12:10:35 PM |

  ### Notes:

  - Review of research using computational methods to infer function from evolutionary relationships.

    How SCOP/CATH is used:

    Background on protein structure classification.

    SCOP reference:

    Structure-based patterns

    Structural information adds another dimension to the search for functionally relevant similarities among proteins. First, global structure alignments will detect homologies that elude sequence searches [8]. Addition- ally, spatial correlation among key residues can reveal highly specific three-dimensional (3D) functional fea- tures [31]. Some structural comparisons treat the structure as a rigid body, as in DALI [32] and TM-align [33], while others tolerate flexibility, as in TOPS++FATCAT [34⬚⬚]. A challenge for these structural alignments is the lack of a universally accepted definition of structural similarity [35]. In order to address this, CATH [36] and SCOP [37] created manually curated protein structure classifi- cation codes based on both domain and evolutionary similarities. These classifications enable functional infer- ence of protein structure in many cases, but overall, and for the same reasons that a few amino acid prove deter- minant of function in sequence comparisons, the struc- ture-to-function relationship over protein domains is not one-to-one [38].

  ### Attachments

  - 1-s2.0-S0959440X12000759-main.pdf
- ## The utility of artificially evolved sequences in protein threading and fold recognition

  |  |  |
  | --- | --- |
  | Type | Journal Article |
  | Author | Michal Brylinski |
  | Volume | 328 |
  | Pages | 77-88 |
  | Publication | Journal of Theoretical Biology |
  | ISSN | 0022-5193 |
  | Date | JUL 7 2013 |
  | Extra | WOS:000318960200009 |
  | DOI | 10.1016/j.jtbi.2013.03.018 |
  | Abstract | Template-based protein structure prediction plays an important role in Functional Genomics by providing structural models of gene products, which can be utilized by structure-based approaches to function inference. From a systems level perspective, the high structural coverage of gene products in a given organism is critical. Despite continuous efforts towards the development of more sensitive threading approaches, confident structural models cannot be constructed for a considerable fraction of proteins due to difficulties in recognizing low-sequence identity templates with a similar fold to the target. Here we introduce a new modeling stratagem, which employs a library of synthetic sequences to improve template ranking in fold recognition by sequence profile-based methods. We developed a new method for the optimization of generic protein-like amino acid sequences to stabilize the respective structures using a combined empirical scoring function, which is compatible with these commonly used in protein threading and fold recognition. We show that the artificially evolved sequences, whose average sequence identity to the wild-type sequences is as low as 13.8%, have significant capabilities to recognize the correct structures. Importantly, the quality of the corresponding threading alignments is comparable to these constructed using conventional wild-type approaches (the average TM-score is 0.48 and 0.54, respectively). Fold recognition that uses data fusion to combine ranks calculated for both wild-type and synthetic template libraries systematically improves the detection of structural analogs. Depending on the threading algorithm used, it yields on average 4-16% higher recognition rates than using the wildtype template library alone. Synthetic sequences artificially evolved for the template structures provide an orthogonal source of signal that could be exploited to detect these templates unrecognized by standard modeling techniques. It opens up new directions in the development of more sensitive threading methods with the enhanced capabilities of targeting difficult, midnight zone templates. (C) 2013 Elsevier Ltd. All rights reserved. |
  | Date Added | 2/13/2014, 4:13:17 PM |
  | Modified | 3/7/2014, 12:10:56 PM |
- ## This Deja Vu Feeling-Analysis of Multidomain Protein Evolution in Eukaryotic Genomes

  |  |  |
  | --- | --- |
  | Type | Journal Article |
  | Author | Christian M. Zmasek |
  | Author | Adam Godzik |
  | Volume | 8 |
  | Issue | 11 |
  | Pages | e1002701 |
  | Publication | Plos Computational Biology |
  | ISSN | 1553-7358 |
  | Date | NOV 2012 |
  | Extra | WOS:000311897100003 |
  | DOI | 10.1371/journal.pcbi.1002701 |
  | Abstract | Evolutionary innovation in eukaryotes and especially animals is at least partially driven by genome rearrangements and the resulting emergence of proteins with new domain combinations, and thus potentially novel functionality. Given the random nature of such rearrangements, one could expect that proteins with particularly useful multidomain combinations may have been rediscovered multiple times by parallel evolution. However, existing reports suggest a minimal role of this phenomenon in the overall evolution of eukaryotic proteomes. We assembled a collection of 172 complete eukaryotic genomes that is not only the largest, but also the most phylogenetically complete set of genomes analyzed so far. By employing a maximum parsimony approach to compare repertoires of Pfam domains and their combinations, we show that independent evolution of domain combinations is significantly more prevalent than previously thought. Our results indicate that about 25% of all currently observed domain combinations have evolved multiple times. Interestingly, this percentage is even higher for sets of domain combinations in individual species, with, for instance, 70% of the domain combinations found in the human genome having evolved independently at least once in other species. We also show that previous, much lower estimates of this rate are most likely due to the small number and biased phylogenetic distribution of the genomes analyzed. The process of independent emergence of identical domain combination is widespread, not limited to domains with specific functional categories. Besides data from large-scale analyses, we also present individual examples of independent domain combination evolution. The surprisingly large contribution of parallel evolution to the development of the domain combination repertoire in extant genomes has profound consequences for our understanding of the evolution of pathways and cellular processes in eukaryotes and for comparative functional genomics. |
  | Date Added | 2/20/2014, 12:24:01 PM |
  | Modified | 2/20/2014, 12:24:01 PM |

  ### Notes:

  - Computational study of emergence of different combinations of domains in multidomain proteins within a set of 172 eukaryotic genomes.  Find a core set of domain combinations keep reemerging independently.

    How SCOP is used:

    Get statistics on ratio of multidomain proteins in SCOP to study whether there are biases.

    SCOP reference:

    Similarly, we can show that other differences between our results and that of the previous analyses are mostly due to the changes in the number of genomes and the size of the domain database. For instance, analysis of five eukaryotic genomes and domain definitions from the SCOP 1.53 database [47] led to the estimate that 80% of all eukaryotic proteins are multidomain proteins [48] (similar numbers were reported

    in Liu), while our results suggest that this number is around 32% (Table 1). Two reasons are likely to contribute to these discrepancies. First, here we used much-more-stringent cutoff values than the unrealistically low E-value of 1022 used in [48]. But even performing our analysis with an E-value cutoff of 1022 instead of the domain- specific ‘‘gathering’’ thresholds results in a multidomain protein percentage of 52 (and a protein match range of 52% to 97%), which is still lower than reported in [33] and [49]. This effect is due to the growth of the domain databases over the last 10 years—the SCOP database has more than doubled during that time—and the specific bias in the order in which domains are added to databases such as SCOP or Pfam. For instance, central and highly promiscuous domains [10], such as kinase, PH (Pleckstrin homology), PDZ, SH3 (Src Homology 3), and AAA (ATPases Associated with diverse cellular Activities), have been studied and, as a consequence, added to the domain databases earlier than rare and less-central domains. Confirming this trend are two more-recent studies based on seven eukaryotic genomes in which the percentage of eukaryotic multido- main proteins is estimated to be 65% [49].

  ### Attachments

  - journal.pcbi.1002701.pdf
- ## ThreaDom: extracting protein domain boundary information from multiple threading alignments

  |  |  |
  | --- | --- |
  | Type | Journal Article |
  | Author | Zhidong Xue |
  | Author | Dong Xu |
  | Author | Yan Wang |
  | Author | Yang Zhang |
  | URL | http://bioinformatics.oxfordjournals.org/content/29/13/i247.abstract |
  | Volume | 29 |
  | Issue | 13 |
  | Pages | i247–i256 |
  | Publication | Bioinformatics |
  | Date | 2013 |
  | Accessed | 9/23/2013, 10:14:18 AM |
  | Library Catalog | Google Scholar |
  | Short Title | ThreaDom |
  | Date Added | 10/11/2013, 10:29:15 AM |
  | Modified | 3/7/2014, 12:08:36 PM |

  ### Tags:

  - negative reference

  ### Notes:

  - THREA-Dom is a new method for detecting domains from sequence data, which relies on multiple threading alignments.  One advantage of ThreaDom is that it can detect domains of non-contiguous regions of sequence.

    ThreaDom is a domain prediction method that is trained on CATH data. They mention that there might be bias in their evaluation because methods they compare with are trained on SCOP data.

    How SCOP and CATH are used:

    In order to show that, although trained with CATH data, the predicted domains are still consistent with SCOP domains, compared THREA-Doms predicted domains with SCOP domains (version 1.75).  Used their own data set and retrieved domain data from SCOP.  Because not all of the proteins were found in SCOP, they resorted to using a different method (DomainParser) for these cases.

    SCOP reference:

    One concern of the aforementioned data analyses is on the pos- sible bias of distinctive domain definitions of the training and test proteins, as some methods (e.g. FIEFDom) were trained by do- mains defined in the SCOP database (Murzin et al., 1995), but the analyses are mainly on CATH definitions, which is what ThreaDom was trained on. In Supplementary Table S2, we present a quantitative analysis of the domain predictions on the 315 test protein pairs with the domains defined by SCOP1.75. Similarly, if a protein cannot be seen in the SCOP library, a definition from DomainParser is used instead. Although some small variations are seen in specific score values, there is no qualitative difference between Supplementary Table S2 and the data shown in Table 1 and Figure 3. These results demonstrate that the distinctive domain definitions of different databases have no impact on the training and testing procedures of domain predictions.

  ### Attachments

  - Full Text PDF
  - [HTML] from oxfordjournals.org
  - PubMed entry
  - Snapshot
- ## Three-dimensional domain swapping in the protein structure space

  |  |  |
  | --- | --- |
  | Type | Journal Article |
  | Author | Yongqi Huang |
  | Author | Huaiqing Cao |
  | Author | Zhirong Liu |
  | URL | http://onlinelibrary.wiley.com/doi/10.1002/prot.24055/full |
  | Volume | 80 |
  | Issue | 6 |
  | Pages | 1610–1619 |
  | Publication | Proteins: Structure, Function, and Bioinformatics |
  | Date | 2012 |
  | Accessed | 9/23/2013, 10:16:21 AM |
  | Library Catalog | Google Scholar |
  | Date Added | 10/11/2013, 10:29:15 AM |
  | Modified | 10/11/2013, 10:29:15 AM |

  ### Tags:

  - dataset
  - domain swapping
  - hinge loop
  - interface
  - protein-protein interaction
  - SCOP

  ### Notes:

  - 3D domain swapping happens when protein monomers exchange identical domains during oligomerization.  This paper investigate the question of whether 3D domain swapping is a general mechanism for protein assembly.  In particular, is domain swapping uniformly distributed across  protein domain structure space?

    They first collected a dataset of proteins from the literature and other databases that are 3D-domain swapped structures, then got SCOP classification for the single-domain chain.

    How used SCOP:

    Categorized a data set of domain-swapped structures by fold, superfamily, family, etc. Then analyzed the distribution of fold class to see if there was a preference for some folds over others.

    Reference to SCOP:

    The hierarchical organization (Species, Protein, Family, Superfamily, Fold, and Class) of SCOP facilitates the discovery of protein relationship. By constructing a large dataset of domain-swapped structures and assigning the fold types according to the SCOP classification, we assembled all domain-swapped structures into the protein structure space and systematically evaluated the relationship between domain swapping and protein structure.

    ...

    Swapping of single-domain structures was observed in six SCOP fold classes (all α, all β, α + β, α/β, designed proteins, and membrane and cell surface proteins and peptides), with the α + β class having the largest number of structures (Fig. 2). ... This is the first quantitative estimate of how frequently 3D domain swapping occurs in the protein structure space and the obtained frequency was surprisingly high. Furthermore, the distribution of domain-swapped structures (Fig. 2) and the fold types (families, superfamilies) with swapped proteins (Fig. 3) showed that there is no distinct preference of one fold class over another.

  ### Attachments

  - 24055\_ftp.pdf
  - Snapshot
- ## Three-dimensional structural view of the central metabolic network of Thermotoga maritima

  |  |  |
  | --- | --- |
  | Type | Journal Article |
  | Author | Ying Zhang |
  | Author | Ines Thiele |
  | Author | Dana Weekes |
  | Author | Zhanwen Li |
  | Author | Lukasz Jaroszewski |
  | Author | Krzysztof Ginalski |
  | Author | Ashley M Deacon |
  | Author | John Wooley |
  | Author | Scott A Lesley |
  | Author | Ian A Wilson |
  | Author | Bernhard Palsson |
  | Author | Andrei Osterman |
  | Author | Adam Godzik |
  | Volume | 325 |
  | Issue | 5947 |
  | Pages | 1544-1549 |
  | Publication | Science (New York, N.Y.) |
  | ISSN | 1095-9203 |
  | Date | Sep 18, 2009 |
  | Extra | PMID: 19762644 |
  | Journal Abbr | Science |
  | DOI | 10.1126/science.1174671 |
  | Library Catalog | NCBI PubMed |
  | Language | eng |
  | Abstract | Metabolic pathways have traditionally been described in terms of biochemical reactions and metabolites. With the use of structural genomics and systems biology, we generated a three-dimensional reconstruction of the central metabolic network of the bacterium Thermotoga maritima. The network encompassed 478 proteins, of which 120 were determined by experiment and 358 were modeled. Structural analysis revealed that proteins forming the network are dominated by a small number (only 182) of basic shapes (folds) performing diverse but mostly related functions. Most of these folds are already present in the essential core (approximately 30%) of the network, and its expansion by nonessential proteins is achieved with relatively few additional folds. Thus, integration of structural data with networks analysis generates insight into the function, mechanism, and evolution of biological networks. |
  | Date Added | 10/11/2013, 10:29:15 AM |
  | Modified | 10/11/2013, 10:29:15 AM |

  ### Notes:

  - Derived a metabolic network for bacterium Thermotoga maritima.  Network encompasses 478 proteins: 120 with experimental structures and 358 with modeled structures.  Found that integration of structural data with network analysis gives insight into function, mechanism, and evolution of biological networks.

    How SCOP is used:

    Use SCOP to classify their protein structures by fold.

    SCOP references:

    Use SCOP in two figures.

  ### Attachments

  - PubMed entry
  - Science-2009-Zhang-1544-9.pdf
- ## Three structural representatives of the PF06855 protein domain family from Staphyloccocus aureus and Bacillus subtilis have SAM domain-like folds and different functions

  |  |  |
  | --- | --- |
  | Type | Journal Article |
  | Author | G. V. T. Swapna |
  | Author | Paolo Rossi |
  | Author | Alexander F. Montelione |
  | Author | Jordi Benach |
  | Author | Bomina Yu |
  | Author | Mariam Abashidze |
  | Author | Jayaraman Seetharaman |
  | Author | Rong Xiao |
  | Author | Thomas B. Acton |
  | Author | Liang Tong |
  | URL | http://link.springer.com/article/10.1007/s10969-012-9134-6 |
  | Volume | 13 |
  | Issue | 3 |
  | Pages | 163–170 |
  | Publication | Journal of structural and functional genomics |
  | Date | 2012 |
  | Accessed | 9/20/2013, 1:20:11 PM |
  | Library Catalog | Google Scholar |
  | Date Added | 2/20/2014, 12:24:01 PM |
  | Modified | 2/20/2014, 12:24:01 PM |

  ### Notes:

  - Experimental and computational study of three structural representatives from the PF06855 protein domain family.  Clone, express, and purify proteins, then use NMR or crystallize the structures.  Use functional and structural analysis to compare the three representatives.  These are the first structural representatives of Pfam domain famaly PF06855.

    How SCOP is used:

    Locate the closest fold to the MW1311 and MW0776 structures.

    SCOP reference:

    Three-dimensional structural analysis was used to complement the CRSH analysis for identification of orthologous proteins. The structures of MW1311 and MW0776 consist of a four a-helix bundle comprised of two orthogonal helix-turn-helix motifs, or helix hairpins (Fig. 1b, c), similar to the SAM domain-like fold in SCOP [35].

  ### Attachments

  - art%3A10.1007%2Fs10969-012-9134-6.pdf
- ## TnpPred: AWeb Service for the Robust Prediction of Prokaryotic Transposases

  |  |  |
  | --- | --- |
  | Type | Journal Article |
  | Author | Gonzalo Riadi |
  | Author | Cristobal Medina-Moenne |
  | Author | David S. Holmes |
  | Pages | 678761 |
  | Publication | Comparative and Functional Genomics |
  | ISSN | 1531-6912 |
  | Date | 2012 |
  | Extra | WOS:000311744100001 |
  | DOI | 10.1155/2012/678761 |
  | Abstract | Transposases (Tnps) are enzymes that participate in the movement of insertion sequences (ISs) within and between genomes. Genes that encode Tnps are amongst the most abundant and widely distributed genes in nature. However, they are difficult to predict bioinformatically and given the increasing availability of prokaryotic genomes and metagenomes, it is incumbent to develop rapid, high quality automatic annotation of ISs. This need prompted us to develop a web service, termed TnpPred for Tnp discovery. It provides better sensitivity and specificity for Tnp predictions than given by currently available programs as determined by ROC analysis. TnpPred should be useful for improving genome annotation. The TnpPred web service is freely available for noncommercial use. |
  | Date Added | 2/20/2014, 12:24:01 PM |
  | Modified | 2/20/2014, 12:24:01 PM |

  ### Notes:

  - Present TnPred method for transposases (Tnps) predictions. Tnps are enzymes that participate in the movement of insertion sequences within and between genomes.  In particular, host HMM profiles for IS families not covered by Superfamily or other resources.

    How SCOP is used:

    Do not use SCOP data.  Background on protein structure classification

    SCOP reference:

    An additional bioinformatic resource for IS prediction is the Superfamily database [14] of structural and functional annotation of genomes based on a library of HMM profiles derived from structural domains in SCOP database [15]. Currently, Superfamily hosts 6 HMM profiles from domains belonging to two prokaryotic families of transposases, mu bacteriophage transposase, and IS200. A third HMM profile in Superfamily recognizes the eukaryotic Hermes transposase.

  ### Attachments

  - 678761.pdf
- ## Topology and structural self-organization in folded proteins

  |  |  |
  | --- | --- |
  | Type | Journal Article |
  | Author | M. Lundgren |
  | Author | Andrey Krokhotin |
  | Author | Antti J. Niemi |
  | Volume | 88 |
  | Issue | 4 |
  | Pages | 042709 |
  | Publication | Physical Review E |
  | ISSN | 1539-3755; 1550-2376 |
  | Date | OCT 28 2013 |
  | Extra | WOS:000326163800009 |
  | DOI | 10.1103/PhysRevE.88.042709 |
  | Abstract | Topological methods are indispensable in theoretical studies of particle physics, condensed matter physics, and gravity. These powerful techniques have also been applied to biological physics. For example, knowledge of DNA topology is pivotal to the understanding as to how living cells function. Here, the biophysical repertoire of topological methods is extended, with the aim to understand and characterize the global structure of a folded protein. For this, the elementary concept of winding number of a vector field on a plane is utilized to introduce a topological quantity called the folding index of a crystallographic protein. It is observed that in the case of high resolution protein crystals, the folding index, when evaluated over the entire length of the crystallized protein backbone, has a very clear and strong propensity towards integer values. The observation proposes that the way how a protein folds into its biologically active conformation is a structural self-organization process with a topological facet that relates to the concept of solitons. It is proposed that the folding index has a potential to become a useful tool for the global, topological characterization of the folding pathways. |
  | Date Added | 2/12/2014, 1:36:22 PM |
  | Modified | 3/7/2014, 1:06:41 PM |

  ### Notes:

  - Present a knot-theory based method for describing protein backbone conformation that may be used to compare two protein chains.

    How SCOP/CATH is used:

    Provide background on protein structure classification.

    SCOP/CATH reference:

    For backbone fragments with several amino acids, taxonomy approaches such as SCOP and CATH [3–5] provide a classification in terms of the geometry of short protein segments: The modular building blocks of a folded protein are supersecondary structures which are made of regular helices and strands, and apparently irregular loops that join them together.

  ### Attachments

  - PhysRevE.88.042709.pdf
- ## Touring protein fold space with Dali/FSSP

  |  |  |
  | --- | --- |
  | Type | Journal Article |
  | Author | L Holm |
  | Author | C Sander |
  | Volume | 26 |
  | Issue | 1 |
  | Pages | 316-319 |
  | Publication | Nucleic acids research |
  | ISSN | 0305-1048 |
  | Date | Jan 1, 1998 |
  | Extra | PMID: 9399863 |
  | Journal Abbr | Nucleic Acids Res. |
  | Library Catalog | NCBI PubMed |
  | Language | eng |
  | Abstract | The FSSP database and its new supplement, the Dali Domain Dictionary, present a continuously updated classification of all known 3D protein structures. The classification is derived using an automatic structure alignment program (Dali) for the all-against-all comparison of structures in the Protein Data Bank. From the resulting enumeration of structural neighbours (which form a surprisingly continuous distribution in fold space) we derive a discrete fold classification in three steps: (i) sequence-related families are covered by a representative set of protein chains; (ii) protein chains are decomposed into structural domains based on the recurrence of structural motifs; (iii) folds are defined as tight clusters of domains in fold space. The fold classification, domain definitions and test sets for sequence-structure alignment (threading) are accessible on the web at www.embl-ebi.ac.uk/dali . The web interface provides a rich network of links between neighbours in fold space, between domains and proteins, and between structures and sequences leading, for example, to a database of explicit multiple alignments of protein families in the twilight zone of sequence similarity. The Dali/FSSP organization of protein structures provides a map of the currently known regions of the protein universe that is useful for the analysis of folding principles, for the evolutionary unification of protein families and for maximizing the information return from experimental structure determination. |
  | Date Added | 10/11/2013, 10:29:15 AM |
  | Modified | 10/11/2013, 10:29:15 AM |

  ### Tags:

  - Computer Communication Networks
  - Databases, Factual
  - Information Storage and Retrieval
  - Protein Conformation
  - Protein Folding
  - Proteins

  ### Notes:

  - Describe FSSP/DALI database which is a classification of all protein structures using DALI for structure comparison.

    How SCOP is used:

    Not using SCOP.  Just listed for comparison.

    SCOP reference:

    There are a number of other classification schemes for protein structures available on the web. Although they are based on the same data, the presentations differ in their basic philosophy regarding automation and organization (4–9)....Scop (5) and CATH (6) are strictly hierarchical classifications based on the abstractions of class (4–10 categories at the top of the hierarchy), architecture/topology or fold, and superfamily (519 in scop). Both classifications are curated by experts, with emphasis in scop on the definition of functionally related superfamilies and in CATH on the definition of architectural types.

  ### Attachments

  - Nucl. Acids Res.-1998-Holm-316-9.pdf
  - PubMed entry
- ## Touring Protein Space with Matt

  |  |  |
  | --- | --- |
  | Type | Journal Article |
  | Author | Noah M. Daniels |
  | Author | Anoop Kumar |
  | Author | Lenore J. Cowen |
  | Author | Matt Menke |
  | Volume | 9 |
  | Issue | 1 |
  | Pages | 286-293 |
  | Publication | Ieee-Acm Transactions on Computational Biology and Bioinformatics |
  | ISSN | 1545-5963 |
  | Date | JAN-FEB 2012 |
  | Extra | WOS:000296782200024 |
  | DOI | 10.1109/TCBB.2011.70 |
  | Abstract | Using the Matt structure alignment program, we take a tour of protein space, producing a hierarchical clustering scheme that divides protein structural domains into clusters based on geometric dissimilarity. While it was known that purely structural, geometric, distance-based measures of structural similarity, such as Dali/FSSP, could largely replicate hand-curated schemes such as SCOP at the family level, it was an open question as to whether any such scheme could approximate SCOP at the more distant superfamily and fold levels. We partially answer this question in the affirmative, by designing a clustering scheme based on Matt that approximately matches SCOP at the superfamily level, and demonstrates qualitative differences in performance between Matt and DaliLite. Implications for the debate over the organization of protein fold space are discussed. Based on our clustering of protein space, we introduce the Mattbench benchmark set, a new collection of structural alignments useful for testing sequence aligners on more distantly homologous proteins. |
  | Date Added | 10/28/2013, 4:53:08 PM |
  | Modified | 3/7/2014, 1:07:10 PM |

  ### Notes:

  - Perform protein structure classification using the Matt structure alignment program and compare with SCOP.

    How SCOP is used:

    Validate against SCOP data (families,superfamilies,and folds).

    How CATH is used:

    Not using CATH data.  Just discuss CATH in context of how it differs with SCOP and other databases.

    SCOP reference:

    Abstract—Using the Matt structure alignment program, we take a tour of protein space, producing a hierarchical clustering scheme that divides protein structural domains into clusters based on geometric dissimilarity. While it was known that purely structural, geometric, distance-based measures of structural similarity, such as Dali/FSSP, could largely replicate hand-curated schemes such as SCOP at the family level, it was an open question as to whether any such scheme could approximate SCOP at the more distant superfamily and fold levels. We partially answer this question in the affirmative, by designing a clustering scheme based on Matt that approximately matches SCOP at the superfamily level, and demonstrates qualitative differences in performance between Matt and DaliLite. Implications for the debate over the organization of protein fold space are discussed. Based on our clustering of protein space, we introduce the Mattbench benchmark set, a new collection of structural alignments useful for testing sequence aligners on more distantly homologous proteins.

  ### Attachments

  - 06078456.pdf
- ## Toward a "Structural BLAST": Using structural relationships to infer function

  |  |  |
  | --- | --- |
  | Type | Journal Article |
  | Author | Fabian Dey |
  | Author | Qiangfeng Cliff Zhang |
  | Author | Donald Petrey |
  | Author | Barry Honig |
  | Volume | 22 |
  | Issue | 4 |
  | Pages | 359-366 |
  | Publication | Protein Science |
  | ISSN | 0961-8368 |
  | Date | APR 2013 |
  | Extra | WOS:000316623900002 |
  | DOI | 10.1002/pro.2225 |
  | Abstract | We outline a set of strategies to infer protein function from structure. The overall approach depends on extensive use of homology modeling, the exploitation of a wide range of global and local geometric relationships between protein structures and the use of machine learning techniques. The combination of modeling with broad searches of protein structure space defines a structural BLAST approach to infer function with high genomic coverage. Applications are described to the prediction of proteinprotein and proteinligand interactions. In the context of proteinprotein interactions, our structure-based prediction algorithm, PrePPI, has comparable accuracy to high-throughput experiments. An essential feature of PrePPI involves the use of Bayesian methods to combine structure-derived information with non-structural evidence (e.g. co-expression) to assign a likelihood for each predicted interaction. This, combined with a structural BLAST approach significantly expands the range of applications of protein structure in the annotation of protein function, including systems level biological applications where it has previously played little role. |
  | Date Added | 10/28/2013, 4:53:08 PM |
  | Modified | 3/7/2014, 12:09:59 PM |

  ### Notes:

  - Present structure-based function prediction method, PrePPI.

    How SCOP/CATH are used:

    Background on protein structure classificaiton.

    SCOP/CATH reference:

    The exploitation of structural relationships has often been based on similarity in the overall shape, or ‘‘fold’’, of proteins. For example, the largely man- ually curated SCOP1 and partially curated CATH2 databases classify proteins into discrete groups. It is frequently possible to deduce function for a given protein based on membership in such predefined cat- egories which can also be determined with tools such as DALI which provide structural similarity scores that have been shown to correlate well with database classifications.3

  ### Attachments

  - 2225\_ftp.pdf
- ## Towards creating complete proteomic structural databases of whole organisms

  |  |  |
  | --- | --- |
  | Type | Journal Article |
  | Author | B. Jayaram |
  | Author | Priyanka Dhingra |
  | URL | http://www.ingentaconnect.com/content/ben/cbio/2012/00000007/00000004/art00010 |
  | Volume | 7 |
  | Issue | 4 |
  | Pages | 424–435 |
  | Publication | Current Bioinformatics |
  | Date | 2012 |
  | Accessed | 9/23/2013, 10:22:49 AM |
  | Library Catalog | Google Scholar |
  | Date Added | 10/11/2013, 10:29:15 AM |
  | Modified | 3/7/2014, 12:14:37 PM |

  ### Tags:

  - Critical assessment of protein structure prediction (CASP)
  - database
  - homology modeling
  - protein structure prediction
  - root mean square deviation (RMSD)
  - template based modelling

  ### Notes:

  - No access to article.

  ### Attachments

  - Snapshot
- ## Towards human-computer synergetic analysis of large-scale biological data

  |  |  |
  | --- | --- |
  | Type | Journal Article |
  | Author | Rahul Singh |
  | Author | Hui Yang |
  | Author | Ben Dalziel |
  | Author | Daniel Asarnow |
  | Author | William Murad |
  | Author | David Foote |
  | Author | Matthew Gormley |
  | Author | Jonathan Stillman |
  | Author | Susan Fisher |
  | Volume | 14 |
  | Pages | S10 |
  | Publication | BMC bioinformatics |
  | ISSN | 1471-2105 |
  | Date | OCT 9 2013 |
  | Extra | WOS:000326747100010 |
  | DOI | 10.1186/1471-2105-14-S14-S10 |
  | Abstract | Background: Advances in technology have led to the generation of massive amounts of complex and multifarious biological data in areas ranging from genomics to structural biology. The volume and complexity of such data leads to significant challenges in terms of its analysis, especially when one seeks to generate hypotheses or explore the underlying biological processes. At the state-of-the-art, the application of automated algorithms followed by perusal and analysis of the results by an expert continues to be the predominant paradigm for analyzing biological data. This paradigm works well in many problem domains. However, it also is limiting, since domain experts are forced to apply their instincts and expertise such as contextual reasoning, hypothesis formulation, and exploratory analysis after the algorithm has produced its results. In many areas where the organization and interaction of the biological processes is poorly understood and exploratory analysis is crucial, what is needed is to integrate domain expertise during the data analysis process and use it to drive the analysis itself. Results: In context of the aforementioned background, the results presented in this paper describe advancements along two methodological directions. First, given the context of biological data, we utilize and extend a design approach called experiential computing from multimedia information system design. This paradigm combines information visualization and human-computer interaction with algorithms for exploratory analysis of large-scale and complex data. In the proposed approach, emphasis is laid on: (1) allowing users to directly visualize, interact, experience, and explore the data through interoperable visualization-based and algorithmic components, (2) supporting unified query and presentation spaces to facilitate experimentation and exploration, (3) providing external contextual information by assimilating relevant supplementary data, and (4) encouraging user-directed information visualization, data exploration, and hypotheses formulation. Second, to illustrate the proposed design paradigm and measure its efficacy, we describe two prototype web applications. The first, called XMAS (Experiential Microarray Analysis System) is designed for analysis of time-series transcriptional data. The second system, called PSPACE (Protein Space Explorer) is designed for holistic analysis of structural and structure-function relationships using interactive low-dimensional maps of the protein structure space. Both these systems promote and facilitate human-computer synergy, where cognitive elements such as domain knowledge, contextual reasoning, and purpose-driven exploration, are integrated with a host of powerful algorithmic operations that support large-scale data analysis, multifaceted data visualization, and multi-source information integration. Conclusions: The proposed design philosophy, combines visualization, algorithmic components and cognitive expertise into a seamless processing-analysis-exploration framework that facilitates sense-making, exploration, and discovery. Using XMAS, we present case studies that analyze transcriptional data from two highly complex domains: gene expression in the placenta during human pregnancy and reaction of marine organisms to heat stress. With PSPACE, we demonstrate how complex structure-function relationships can be explored. These results demonstrate the novelty, advantages, and distinctions of the proposed paradigm. Furthermore, the results also highlight how domain insights can be combined with algorithms to discover meaningful knowledge and formulate evidence-based hypotheses during the data analysis process. Finally, user studies against comparable systems indicate that both XMAS and PSPACE deliver results with better interpretability while placing lower cognitive loads on the users. XMAS is available at: http://tintin.sfsu.edu:8080/xmas. PSPACE is available at: http://pspace.info/. |
  | Date Added | 2/20/2014, 12:24:01 PM |
  | Modified | 5/5/2014, 3:10:42 PM |

  ### Notes:

  - Present two prototype web applications for exploratory data analysis.  PSPACE (Protein space explorer) provides an interactive view of the protein universe.

    How SCOP/CATH is used:

    Proteins in PSPACE are annotated with their classification (superfamily level).

    SCOP/CATH reference:

    PSPACE is a web based software system for experiential exploration of protein structure-function relationships through low (two or three) dimensional maps of the protein fold space, displayed as interactive scatter plots. PSPACE allows for interactive visual data analysis and user driven exploration of annotations from external data sources (e.g. CATH [38] and SCOP [39]), which may be mapped to attributes such as the color of points in the MPSS.

    ...

    For example, PSPACE interactively maps CATH and SCOP annotations to pro- vide perspective on structure-function properties of a pro- tein.

    ...

    • Perform functional inference through analysis and exploration of structural proximity of adjacent structures annotated with protein properties (CATH and SCOP annotations).

    ...

    • User-driven analysis of MPSS using interactive visua- lizations. MPSS allow for broad and localized topological analysis of structure-function patterns using interactively mapped CATH and SCOP annotations to provide various protein property views. Molecular structure views of indi- vidual structures and pair

    ...

  ### Attachments

  - 1471-2105-14-S14-S10.pdf
- ## Towards the virtual screening of BIK inhibitors with the homology-modeled protein structure

  |  |  |
  | --- | --- |
  | Type | Journal Article |
  | Author | Bhargavi Kondagari |
  | Author | Ramasree Dulapalli |
  | Author | Dwarkanath Krishna Murthy |
  | Author | Uma Vuruputuri |
  | URL | http://link.springer.com/article/10.1007/s00044-012-0105-z |
  | Volume | 22 |
  | Issue | 3 |
  | Pages | 1184–1196 |
  | Publication | Medicinal Chemistry Research |
  | Date | 2013 |
  | Accessed | 9/23/2013, 10:24:20 AM |
  | Library Catalog | Google Scholar |
  | Date Added | 10/11/2013, 10:29:15 AM |
  | Modified | 3/7/2014, 12:09:26 PM |

  ### Tags:

  - ADME
  - BIK
  - Cancer
  - Glide
  - homology modeling
  - Virtual screening

  ### Notes:

  - Computational drug design study, to find inhibitors of BIK protein.  Performed homology modeling to get a plausible structure, and then examined ligands would bind in the structure.  BIK is involved in apostosis.

    How SCOP/CATH is used:

    Search SCOP, the PDB, and CATH for homologs using several different programs.

    SCOP reference:

    Homology modeling

    The 3D model of the BIK was built by homology modeling based on the high-resolution crystal structures of homolo- gous proteins. Similar method of generating 3D models was applied in generating the model of Bcl-2L10 and SigK (Bhargavi et al., 2010, Vasavi et al., 2011). The complete amino acid sequence of the target protein BIK was retrieved from Swiss-Prot database (accession number: Q13323) in FASTA format (Boeckmann et al., 2003). Sequence similarity search of target was used for searching the crystal structures of the closest homologs through structural databases PDB (Bernstein et al., 1977), SCOP (Murzin et al., 1995), CATH (Orengo et al., 1997) using several programs like PDB-Blast (Altschul et al., 1990), JPRED (Cuff et al., 1998), 3D-PSSM (Kelley et al., 2000), FUGUE (Shi et al., 2001), and Domain Fishing (Contreras- Moreira and Bates 2002).

  ### Attachments

  - art%3A10.1007%2Fs00044-012-0105-z.pdf
  - Snapshot

    ## Abstract

    Induction of apoptosis in tumor cells through direct triggering of the Bcl-2 regulated intrinsic pathway by small molecules carries great potential to overcome the shortcomings of current anticancer therapies. The Bcl-2 family members are crucial regulators of apoptosis. The BIK protein, is an important member of the Bcl-2 family and an attractive drug target. Homology model of BIK was developed based on the crystal structures of appropriate template. We have employed structure-based virtual screening techniques using Glide 5.6 to identify lead like molecules from an in-house library. The database has yielded 345 hits, the top scoring 60 ligands were selected and a pharmacokinetic analysis (ADME) was performed. We have identified six ligands from the combined approach of virtual screening followed by ADME that can work against BIK.
- ## Toxic and nontoxic components of botulinum neurotoxin complex are evolved from a common ancestral zinc protein

  |  |  |
  | --- | --- |
  | Type | Journal Article |
  | Author | Ken Inui |
  | Author | Yoshimasa Sagane |
  | Author | Keita Miyata |
  | Author | Shin-Ichiro Miyashita |
  | Author | Tomonori Suzuki |
  | Author | Yasuyuki Shikamori |
  | Author | Tohru Ohyama |
  | Author | Koichi Niwa |
  | Author | Toshihiro Watanabe |
  | URL | http://www.sciencedirect.com/science/article/pii/S0006291X12002781 |
  | Volume | 419 |
  | Issue | 3 |
  | Pages | 500–504 |
  | Publication | Biochemical and biophysical research communications |
  | Date | 2012 |
  | Accessed | 2/28/2013, 1:38:04 PM |
  | Library Catalog | Google Scholar |
  | Date Added | 10/11/2013, 10:29:15 AM |
  | Modified | 3/7/2014, 12:14:43 PM |

  ### Tags:

  - Botulinum neurotoxin
  - Inductively coupled plasma-mass
  - Molecular modeling
  - Nontoxic nonhemagglutinin
  - Protein domain architecture
  - spectrometry
  - Zinc coordination

  ### Notes:

  - Use SCOP database to verify that their NTNHA and BoNT proteins had similar structures.

    This paper doesn't make too much sense to me, because it looks like they took the sequence of NTNHA and then used BoNT to homology model its structure.

    How SCOP is used:

    Search CATH and SCOP for structure classification of BoNT and NTNHA structures, and find they have the same domain architecture.

    See figure 2B.

    Why is CATH cited:

    Look up classification in CATH for more information.

    SCOP references:

    From Abstract:

    "A protein structure classification database search indicated that BoNT and NTNHA share a similar domain architecture, comprising a zinc-dependent metalloproteinase-like, BoNT coiled-coil motif and concanavalin A-like domains."

    2. Materials and methods

    2.1. In silico analyses

    To analyze protein structure classifications, amino acid se- quences of NTNHA [serotype A strain Hall (A-Hall), GenBank ID: ABS37375.1; serotype B strain Okra (B-Okra), GenBank ID: ACA47084.1; serotype C strain Stockholm (C-St), GenBank ID: CAA44262.1; serotype D strain 4947 (D-4947), GenBank ID: BAA90660.1; serotype E strain Alaska (E-Alaska), GenBank ID: ACD52603.1; serotype F strain Langeland (F-Langeland), GenBank ID: CAA67511.1] and BoNT (A-Hall, GenBank ID: ABS38337.1; B- Okra, GenBank ID: ACA46990.1; C-St, GenBank ID: CAA44263.1; D-4947, GenBank ID: BAA90661.1; E-Alaska, GenBank ID: ACD53549.1; F-Langeland, ADA79551.1) were examined with pro- tein structure classification database CATH (http://www.cath- db.info/) [15] and with SCOP (http://scop.mrc-lmb.cam.ac.uk/ scop/) [13]. The amino acid sequences of serotype A–F NTNHAs were aligned (ClustalW) with those of serotypes A–F BoNT pro- teins, in order to predict the zinc-coordinating site in the NTNHA molecule.

    "The SCOP database search showed that three domain structures, e.g. the zincin catalytic domain, the BoNT “coiled-coil” domain and the concanavalin A-like lectin domain, were assigned to all serotypes of the NTNHA molecules (Fig 2B). These three domains were also found in all serotypes of BoNT molecules, and the domain architecture of NTNHA and BoNT are quite similar."

  ### Attachments

  - [PDF] from researchgate.net
  - Snapshot
- ## Transmembrane helix: simple or complex

  |  |  |
  | --- | --- |
  | Type | Journal Article |
  | Author | Wing-Cheong Wong |
  | Author | Sebastian Maurer-Stroh |
  | Author | Georg Schneider |
  | Author | Frank Eisenhaber |
  | URL | http://nar.oxfordjournals.org/content/40/W1/W370.short |
  | Volume | 40 |
  | Issue | W1 |
  | Pages | W370–W375 |
  | Publication | Nucleic Acids Research |
  | Date | 2012 |
  | Accessed | 9/20/2013, 1:17:19 PM |
  | Library Catalog | Google Scholar |
  | Short Title | Transmembrane helix |
  | Date Added | 10/11/2013, 10:20:13 AM |
  | Modified | 11/12/2013, 4:28:33 PM |

  ### Notes:

  - Web server for identifying and classifying simple and complex transmembrane helical segments.

    Simple TMs: products of convergent evolution.  hydrophobic anchors that will have overrepresentation of aliphatic hydrophobic residues, and therefore, will show false homology with other sequences

    Complex TMs: have structural and functional roles beyond membrane immersion

    Motivation: would like to mask segments of sequences from TMs from BLAST searches

    How SCOP is used:

    Gather alpha helices in SCOP domains, which tend to be globular.  Then the user's TM helices can be compared with these.

    Plot complexity vs. hydrophobicity of SCOP alpha helices, membrane anchors (simple), functional tm helices (complex), and the user's predicted TMs.

    It's very unclear what they mean by 'SCOP alpha helices', but my best guess is that they downloaded data from the all-alpha class.

    Input:  "(i) a fasta-formatted sequence as a mandatory  
    input and (ii) the associated TM segments as an  
    optional input."

    Output: In the third section of the output (complexity/hydrophobicity plot) the alpha-helices are used from the SCOP database

    -Uses the sequences to determine complexity and hydrophobicity, which is then plotted on the graph

    ". The third section outputs a sequence complexity/  
    hydrophobicity plot of the predicted/user-defined  
    TM segments (in black) against the background of  
    membrane anchors (in blue), functional TMs (in red)  
    and a-helices (in green) from the SCOP (23,24) database  
    (see Figure 1C)."

    23. Andreeva,A., Howorth,D., Chandonia,J.M., Brenner,S.E.,  
    Hubbard,T.J., Chothia,C. and Murzin,A.G. (2008) Data growth  
    and its impact on the SCOP database: new developments.  
    Nucleic Acids Res., 36, D419–D425.  
    24. Murzin,A.G., Brenner,S.E., Hubbard,T. and Chothia,C. (1995)  
    SCOP: a structural classification of proteins database for the  
    investigation of sequences and structures. J. Mol. Biol., 247,  
    536–540.

  ### Attachments

  - Nucl. Acids Res.-2012-Wong-W370-5.pdf
- ## Trends in structural coverage of the protein universe and the impact of the Protein Structure Initiative

  |  |  |
  | --- | --- |
  | Type | Journal Article |
  | Author | Kamil Khafizov |
  | Author | Carlos Madrid-Aliste |
  | Author | Steven C. Almo |
  | Author | Andras Fiser |
  | URL | http://www.pnas.org/content/early/2014/02/19/1321614111 |
  | Pages | 201321614 |
  | Publication | Proceedings of the National Academy of Sciences |
  | ISSN | 0027-8424, 1091-6490 |
  | Date | 2014-02-24 |
  | Extra | PMID: 24567391 |
  | Journal Abbr | PNAS |
  | DOI | 10.1073/pnas.1321614111 |
  | Accessed | 2/27/2014, 1:29:51 PM |
  | Library Catalog | www.pnas.org |
  | Language | en |
  | Abstract | The exponential growth of protein sequence data provides an ever-expanding body of unannotated and misannotated proteins. The National Institutes of Health-supported Protein Structure Initiative and related worldwide structural genomics efforts facilitate functional annotation of proteins through structural characterization. Recently there have been profound changes in the taxonomic composition of sequence databases, which are effectively redefining the scope and contribution of these large-scale structure-based efforts. The faster-growing bacterial genomic entries have overtaken the eukaryotic entries over the last 5 y, but also have become more redundant. Despite the enormous increase in the number of sequences, the overall structural coverage of proteins—including proteins for which reliable homology models can be generated—on the residue level has increased from 30% to 40% over the last 10 y. Structural genomics efforts contributed ∼50% of this new structural coverage, despite determining only ∼10% of all new structures. Based on current trends, it is expected that ∼55% structural coverage (the level required for significant functional insight) will be achieved within 15 y, whereas without structural genomics efforts, realizing this goal will take approximately twice as long. |
  | Date Added | 2/27/2014, 1:29:51 PM |
  | Modified | 2/27/2014, 1:29:51 PM |

  ### Attachments

  - PubMed entry
  - Snapshot
- ## T-RMSD: a web server for automated fine-grained protein structural classification

  |  |  |
  | --- | --- |
  | Type | Journal Article |
  | Author | Cedrik Magis |
  | Author | Paolo Di Tommaso |
  | Author | Cedric Notredame |
  | URL | http://nar.oxfordjournals.org/content/early/2013/05/28/nar.gkt383.short |
  | Publication | Nucleic Acids Research |
  | Date | 2013 |
  | Accessed | 9/20/2013, 1:12:22 PM |
  | Library Catalog | Google Scholar |
  | Short Title | T-RMSD |
  | Date Added | 10/11/2013, 10:20:13 AM |
  | Modified | 3/7/2014, 12:09:16 PM |

  ### Notes:

  - Article introduces T-RMSD server, which is a new computational method of classifying proteins using structure and function

    How SCOP/CATH is used:

    Not using SCOP or CATH data.

    SCOP/CATH reference:

    "This observation has triggered the development of several  
    structural classification schemes, such as SCOP (6),  
    CATH (7)"

    **Citation**

    6. Murzin,A.G., Brenner,S.E., Hubbard,T. and Chothia,C. (1995)  
    SCOP: a structural classification of proteins database for the  
    investigation of sequences and structures. J. Mol. Biol., 247,  
    536–540.

  ### Attachments

  - Nucl. Acids Res.-2013-Magis-nar\_gkt383.pdf
- ## Twilight zone of protein sequence alignments

  |  |  |
  | --- | --- |
  | Type | Journal Article |
  | Author | Burkhard Rost |
  | URL | http://peds.oxfordjournals.org/content/12/2/85.short |
  | Volume | 12 |
  | Issue | 2 |
  | Pages | 85–94 |
  | Publication | Protein engineering |
  | Date | 1999 |
  | Accessed | 10/10/2013, 1:19:17 PM |
  | Library Catalog | Google Scholar |
  | Date Added | 10/11/2013, 10:29:15 AM |
  | Modified | 10/11/2013, 10:29:15 AM |

  ### Tags:

  - alignment quality analysis
  - evolutionary conservation
  - genome analysis
  - protein sequence alignment
  - sequence space hopping

  ### Notes:

  - With long alignments with sequence ID >40% have the predictive power to distinguish between similar and non-similar structures is very high.

    The "twilight zone" of protein sequence alignment is where identity is around 20-35% and its ambiguous whether the structures will be similar or not.

    The paper describes an attempt to better characterize and define the boundary of this "twilight zone".

    How SCOP is used:

    Do not use SCOP data.

    Negative reference.  Point out that CATH, SCOP, and FSSP can disagree on domain structure similarity, and have chosen to use FSSP for their "standard of truth".

  ### Attachments

  - [HTML] from oxfordjournals.org
  - Protein Eng.-1999-Rost-85-94.pdf
  - Snapshot
- ## Two Fe-S clusters catalyze sulfur insertion by radical-SAM methylthiotransferases

  |  |  |
  | --- | --- |
  | Type | Journal Article |
  | Author | Farhad Forouhar |
  | Author | Simon Arragain |
  | Author | Mohamed Atta |
  | Author | Serge Gambarelli |
  | Author | Jean-Marie Mouesca |
  | Author | Munif Hussain |
  | Author | Rong Xiao |
  | Author | Sylvie Kieffer-Jaquinod |
  | Author | Jayaraman Seetharaman |
  | Author | Thomas B. Acton |
  | Author | Gaetano T. Montelione |
  | Author | Etienne Mulliez |
  | Author | John F. Hunt |
  | Author | Marc Fontecave |
  | Volume | 9 |
  | Issue | 5 |
  | Pages | 333-+ |
  | Publication | Nature Chemical Biology |
  | ISSN | 1552-4450 |
  | Date | MAY 2013 |
  | Extra | WOS:000317727600011 |
  | DOI | 10.1038/NCHEMBIO.1229 |
  | Abstract | How living organisms create carbon-sulfur bonds during the biosynthesis of critical sulfur-containing compounds is still poorly understood. The methylthiotransferases MiaB and RimO catalyze sulfur insertion into tRNAs and ribosomal protein S12, respectively. Both belong to a subgroup of radical-S-adenosylmethionine (radical-SAM) enzymes that bear two [4Fe-4S] clusters. One cluster binds S-adenosylmethionine and generates an Ado radical via a well-established mechanism. However, the precise role of the second cluster is unclear. For some sulfur-inserting radical-SAM enzymes, this cluster has been proposed to act as a sacrificial source of sulfur for the reaction. In this paper, we report parallel enzymological, spectroscopic and crystallographic investigations of RimO and MiaB, which provide what is to our knowledge the first evidence that these enzymes are true catalysts and support a new sulfation mechanism involving activation of an exogenous sulfur cosubstrate at an exchangeable coordination site on the second cluster, which remains intact during the reaction. |
  | Date Added | 2/20/2014, 12:24:01 PM |
  | Modified | 2/20/2014, 12:24:01 PM |

  ### Notes:

  - Study two mehtyltriotransferases, MiaB and RimO.

    How SCOP is used:

    Both proteins share a conserved N-terminal domain UPF0004, whose structure was previously not solved. The authors crystallize the structure and classify its fold in SCOP.

    Although they don't explicitly describe its use in the Methods section, it seems they have used DALI to perform the structure comparison.

    SCOP reference:

    Reference 20 is DALI.  Reference 21 is SCOP.

    Our crystal structure provides what is to our knowledge the first experimental data on the UPF0004 domain fold. The fold seems to comprise a five-stranded parallel β-sheet created by four α-β supersecondary motifs followed by a final β-strand....The UPF0004 domain is struc- turally similar to proteins in the CheY-related fold family20,21, which have a flavodoxin-like α-β fold.

  ### Attachments

  - nchembio.1229.pdf
- ## Tyrosyl Radicals in Dehaloperoxidase HOW NATURE DEALS WITH EVOLVING AN OXYGEN-BINDING GLOBIN TO A BIOLOGICALLY RELEVANT PEROXIDASE

  |  |  |
  | --- | --- |
  | Type | Journal Article |
  | Author | Rania Dumarieh |
  | Author | Jennifer D'Antonio |
  | Author | Alexandria Deliz-Liang |
  | Author | Tatyana Smirnova |
  | Author | Dimitri A. Svistunenko |
  | Author | Reza A. Ghiladi |
  | Volume | 288 |
  | Issue | 46 |
  | Pages | 33470-33482 |
  | Publication | Journal of Biological Chemistry |
  | ISSN | 0021-9258; 1083-351X |
  | Date | NOV 15 2013 |
  | Extra | WOS:000328841700059 |
  | DOI | 10.1074/jbc.M113.496497 |
  | Abstract | Dehaloperoxidase (DHP) from Amphitrite ornata, having been shown to catalyze the hydrogen peroxide-dependent oxidation of trihalophenols to dihaloquinones, is the first oxygen binding globin that possesses a biologically relevant peroxidase activity. The catalytically competent species in DHP appears to be Compound ES, a reactive intermediate that contains both a ferryl heme and a tyrosyl radical. By simulating the EPR spectra of DHP activated by H2O2, Thompson et al. (Thompson, M. K., Franzen, S., Ghiladi, R. A., Reeder, B. J., and Svistunenko, D. A. (2010) J. Am. Chem. Soc. 132, 17501-17510) proposed that two different radicals, depending on the pH, are formed, one located on either Tyr-34 or Tyr-28 and the other on Tyr-38. To provide additional support for these simulation-based assignments and to deduce the role(s) that tyrosyl radicals play in DHP, stopped-flow UV-visible and rapid-freeze-quench EPR spectroscopic methods were employed to study radical formation in DHP when three tyrosine residues, Tyr-28, Tyr-34, and Tyr-38, were replaced either individually or in combination with phenylalanines. The results indicate that radicals form on all three tyrosines in DHP. Evidence for the formation of DHP Compound I in several tyrosine mutants was obtained. Variants that formed Compound I showed an increase in the catalytic rate for substrate oxidation but also an increase in heme bleaching, suggesting that the tyrosines are necessary for protecting the enzyme from oxidizing itself. This protective role of tyrosines is likely an evolutionary adaptation allowing DHP to avoid self-inflicted damage in the oxidative environment. |
  | Date Added | 2/12/2014, 1:36:22 PM |
  | Modified | 2/12/2014, 1:36:22 PM |

  ### Notes:

  - Experimental study of dehaloperoxidase (DHP).

    How SCOP is used:

    look up fold classification of DHP.

    SCOP reference:

    (Reference number is 15)

    As a globin, DHP has a characteristic fold composed of the canonical 3/3 ⬚⬚-helical structure (11, 13–15) ..

  ### Attachments

  - J. Biol. Chem.-2013-Dumarieh-33470-82.pdf
- ## UCSF Chimera—a visualization system for exploratory research and analysis

  |  |  |
  | --- | --- |
  | Type | Journal Article |
  | Author | Eric F. Pettersen |
  | Author | Thomas D. Goddard |
  | Author | Conrad C. Huang |
  | Author | Gregory S. Couch |
  | Author | Daniel M. Greenblatt |
  | Author | Elaine C. Meng |
  | Author | Thomas E. Ferrin |
  | URL | http://onlinelibrary.wiley.com/doi/10.1002/jcc.20084/full |
  | Volume | 25 |
  | Issue | 13 |
  | Pages | 1605–1612 |
  | Publication | Journal of computational chemistry |
  | Date | 2004 |
  | Accessed | 10/10/2013, 1:18:50 PM |
  | Library Catalog | Google Scholar |
  | Date Added | 10/11/2013, 10:29:15 AM |
  | Modified | 10/11/2013, 10:29:15 AM |

  ### Tags:

  - extensibility
  - molecular graphics
  - multiscale modeling
  - Sequence Alignment
  - visualization

  ### Notes:

  - Present Chimera software.

    How SCOP is used:

    Software supports loading SCOP domains into multiple sequence alignment viewer.

    SCOP reference:

    Conversely, if the alignment sequence names are recognizable as including SCOP19,20 or PDB4 identifiers using a few simple criteria, the researcher can use a Multalign Viewer menu item or preference setting to load all of the corresponding structures into Chimera.

  ### Attachments

  - chimera\_2004.pdf
  - Snapshot
- ## Understanding Protein-Protein Interactions Using Local Structural Features

  |  |  |
  | --- | --- |
  | Type | Journal Article |
  | Author | Joan Planas-Iglesias |
  | Author | Jaume Bonet |
  | Author | Javier Garcia-Garcia |
  | Author | Manuel A. Marin-Lopez |
  | Author | Elisenda Feliu |
  | Author | Baldo Oliva |
  | Volume | 425 |
  | Issue | 7 |
  | Pages | 1210-1224 |
  | Publication | Journal of Molecular Biology |
  | ISSN | 0022-2836 |
  | Date | APR 12 2013 |
  | Extra | WOS:000316924600010 |
  | DOI | 10.1016/j.jmb.2013.01.014 |
  | Abstract | Protein-protein interactions (PPIs) play a relevant role among the different functions of a cell. Identifying the PPI network of a given organism (interactome) is useful to shed light on the key molecular mechanisms within a biological system. In this work, we show the role of structural features (loops and domains) to comprehend the molecular mechanisms of PPIs. A paradox in protein protein binding is to explain how the unbound proteins of a binary complex recognize each other among a large population within a cell and how they find their best docking interface in a short timescale. We use interacting and non-interacting protein pairs to classify the structural features that sustain the binding (or non-binding) behavior. Our study indicates that not only the interacting region but also the rest of the protein surface are important for the interaction fate. The interpretation of this classification suggests that the balance between favoring and disfavoring structural features determines if a pair of proteins interacts or not. Our results are in agreement with previous works and support the funnel-like intermolecular energy landscape theory that explains PPIs. We have used these features to score the likelihood of the interaction between two proteins and to develop a method for the prediction of PPIs. We have tested our method on several sets with unbalanced ratios of interactions and non-interactions to simulate real conditions, obtaining accuracies higher than 25% in the most unfavorable circumstances. (C) 2013 Elsevier Ltd. All rights reserved. |
  | Date Added | 2/20/2014, 12:24:01 PM |
  | Modified | 2/20/2014, 12:24:01 PM |

  ### Notes:

  - Computational study of structural features (loops and domains) involved in molecular mechanisms of PPIs.

    How SCOP is used:

    Use SCOP to get domain boundaries for their data set.

    SCOP reference:

    Assignment of classified loops and domains to proteins

    We obtained two reference sets of pairs of proteins—one for PPIs and the other for NIPs—with some structure associated (pairs of proteins to which we could assign standardized loops or domains, see Methods). These sets were referred to as the positive reference set (PRS) and the negative reference set (NRS), respectively. Local structural features, namely, SCOP domains21 or loops classified in ArchDB,22 were assigned to proteins in these sets (see Supplementary Table S1).

    ...

    Assignment of loops and domains

    We assigned loops and domains to each protein in the PPI and NIP sets. Protein loops were defined by the super- secondary structures classified in ArchDB22 and protein domains were defined as classified in SCOP.21

    ...

    We defined as protein signature any group of up to three local structural features. We considered three different types of local structural features. Groups of ArchDB loops were named loop signatures, which were denoted by {L}; groups of SCOP domains were named domain signatures, which were denoted by {D}; and groups of ArchDB loops located in the same SCOP domain were denoted by {LD}. For a pair of proteins (A,B), we defined an interaction signature as a pair of protein signatures of the same type, one from protein A and the other from protein B (see Supplementary Note 1).

    ...

    The PES was obtained by annotating loops from ArchDB and domains from SCOP in the set of curated PPIs by means of sequence similarity as described above. We were able to annotate domains and loops to 8207 and 7264 PPIs, respectively. To extend the set of NIPs, we needed to define some conditions ensuring that a pair of proteins would not interact. First, we considered all proteins of the previous sets PRS and NRS and generated all possible pairs. Next, we removed all PPIs and any pair that could be predicted to interact by means of sequence similarity using BIPS55 (Supplementary Note 3) with a non- restrictive criterion (40%ID sequence similarity). We also ensured that the proteins of the pair were co-localized (sharing the same cellular component GO terms56). We obtained with this protocol 21,155 pairs of proteins with unreported interactions. Finally, loops and domains were annotated for all the protein pairs by means of sequence similarity (as described above) and pairs without structural features were removed. We were able to annotate domains of SCOP for 20,229 protein pairs and loops of ArchDB for 3361 pairs. This set was named NES.

  ### Attachments

  - 1-s2.0-S0022283613000302-main.pdf
- ## Understanding protein structure: using scop for fold interpretation

  |  |  |
  | --- | --- |
  | Type | Journal Article |
  | Author | S. E. Brenner |
  | Author | C. Chothia |
  | Author | T. J. Hubbard |
  | Author | A. G. Murzin |
  | Volume | 266 |
  | Pages | 635-643 |
  | Publication | Methods in Enzymology |
  | ISSN | 0076-6879 |
  | Date | 1996 |
  | Extra | PMID: 8743710 |
  | Journal Abbr | Meth. Enzymol. |
  | Library Catalog | NCBI PubMed |
  | Language | eng |
  | Short Title | Understanding protein structure |
  | Date Added | 11/3/2014, 2:48:03 PM |
  | Modified | 11/3/2014, 2:48:03 PM |

  ### Tags:

  - Amino Acid Sequence
  - Automation
  - Databases, Factual
  - Enzymes
  - NAD
  - NADP
  - Protein Folding
  - Proteins
  - Protein Structure, Secondary
  - Sequence Homology, Amino Acid
  - Software

  ### Attachments

  - PubMed entry
- ## Understanding the Folding-Function Tradeoff in Proteins

  |  |  |
  | --- | --- |
  | Type | Journal Article |
  | Author | Shachi Gosavi |
  | Volume | 8 |
  | Issue | 4 |
  | Pages | e61222 |
  | Publication | Plos One |
  | ISSN | 1932-6203 |
  | Date | APR 12 2013 |
  | Extra | WOS:000317385300063 |
  | DOI | 10.1371/journal.pone.0061222 |
  | Abstract | When an amino-acid sequence cannot be optimized for both folding and function, folding can get compromised in favor of function. To understand this tradeoff better, we devise a novel method for extracting the "function-less" folding-motif of a protein fold from a set of structurally similar but functionally diverse proteins. We then obtain the beta-trefoil folding-motif, and study its folding using structure-based models and molecular dynamics simulations. Comparison with the folding of wild-type beta-trefoil proteins shows that function affects folding in two ways: In the slower folding interleukin-1 beta, binding sites make the fold more complex, increase contact order and slow folding. In the faster folding hisactophilin, residues which could have been part of the folding-motif are used for function. This reduces the density of native contacts in functional regions and increases folding rate. The folding-motif helps identify subtle structural deviations which perturb folding. These may then be used for functional annotation. Further, the folding-motif could potentially be used as a first step in the sequence design of function-less scaffold proteins. Desired function can then be engineered into these scaffolds. |
  | Date Added | 2/20/2014, 12:24:01 PM |
  | Modified | 2/20/2014, 12:24:01 PM |

  ### Notes:

  - Computational study of folding of proteins in b-trefoil fold using structural bioinformatics and molecular dynamics.

    How is SCOP used:

    Curate a data set of representatives from the b-trefoil fold.

    SCOP reference:

    Choosing the residues of the FM using a structural alignment of a set of functionally diverse proteins from the b-trefoil fold

    The SCOP database [32] classifies proteins into different folds. Within these folds, proteins from different families ‘‘have related sequences but distinct functions’’ [32]. One protein is picked at random from each of the 13 families of the b-trefoil fold included in the database. The Multiseq extension [49] (the STAMP algorithm [50]) of VMD [34] is then used to create a structural alignment of the chosen proteins. The pdb IDs of the proteins, the total number of residues and the number of calculated contacts (if a specific chain or a specific set of residues from the pdb file are used, then this information is appended at the end) are: 2AFG (129:374:chain-A), 6I1B (153:430), 1T9F (178:532), 1SR4 (154:367:chain-C), 1DQG (134:388), 1UPS (131:380: chain- A:290–420), 1JLY (153:457:chain-A:1–153), 1WBA (171:499), 1DFQ (193:528: 1123–1315), 1DFC (119:347:chain-A:1141– 1259), 1HCD (118:324), 1TTU (161:428: chain-A:381–541), 1WD4 (162:481:338–499).

  ### Attachments

  - journal.pone.0061222.pdf
- ## Understanding the general packing rearrangements required for successful template based modeling of protein structure from a CASP experiment

  |  |  |
  | --- | --- |
  | Type | Journal Article |
  | Author | Ryan Day |
  | Author | Hyun Joo |
  | Author | Archana C. Chavan |
  | Author | Kristin P. Lennox |
  | Author | Y. Ann Chen |
  | Author | David B. Dahl |
  | Author | Marina Vannucci |
  | Author | Jerry W. Tsai |
  | Volume | 42 |
  | Pages | 40-48 |
  | Publication | COMPUTATIONAL BIOLOGY AND CHEMISTRY |
  | ISSN | 1476-9271 |
  | Date | February 2013 |
  | DOI | 10.1016/j.compbiolchem.2012.10.008 |
  | Language | English |
  | Abstract | As an alternative to the common template based protein structure prediction methods based on main-chain position, a novel side-chain centric approach has been developed. Together with a Bayesian loop modeling procedure and a combination scoring function, the Stone Soup algorithm was applied to the CASP9 set of template based modeling targets. Although the method did not generate as large of perturbations to the template structures as necessary, the analysis of the results gives unique insights into the differences in packing between the target structures and their templates. Considerable variation in packing is found between target and template structures even when the structures are close, and this variation is found due to 2 and 3 body packing interactions. Outside the inherent restrictions in packing representation of the PDB, the first steps in correctly defining those regions of variable packing have been mapped primarily to local interactions, as the packing at the secondary and tertiary structure are largely conserved. Of the scoring functions used, a loop scoring function based on water structure exhibited some promise for discrimination. These results present a clear structural path for further development of a side-chain centered approach to template based modeling. (C) 2012 Elsevier Ltd. All rights reserved. |
  | Date Added | 10/25/2013, 4:17:08 PM |
  | Modified | 10/25/2013, 4:17:08 PM |

  ### Tags:

  - loop modeling
  - Protein packing
  - Protein statistical function
  - Template-based protein structure prediction

  ### Notes:

  - Present method for side-chain packing prediction for protein structure prediction.

    How SCOP is used:

    Derive parameters for algorithm from domain geometry data from ASTRAL representative subset.

    SCOP reference:

    2.1.1. 3SP:side-chaindrivenbackbonerefinement

    The underlying concept of 3SP is to drive backbone perturba- tions based on the interactions of side-chains. This is accomplished by creating a move-set library that relates side-chain packing vari- ations in Cartesian space to the ⬚⬚,⬚⬚ torsion angle space of the backbone main-chain. This library is generated by clustering the maximal contact cliques (Bron and Kerbosch, 1973) computed from the 95% sequence unique ASTRAL (Chandonia et al., 2004) set of known protein structures (hereafter referred to as move- set cliques) based on the relative positions of their C⬚⬚ atoms and side-chain centers of mass (centroids) (Day et al., 2010). These move-set cliques represent the maximally self-interacting clusters of residues (all residues in the set are in contact with all other residues in the set).

  ### Attachments

  - 1-s2.0-S1476927112000783-main.pdf
- ## Understanding the role of domain-domain linkers in the spatial orientation of domains in multi-domain proteins

  |  |  |
  | --- | --- |
  | Type | Journal Article |
  | Author | Ramachandra M. Bhaskara |
  | Author | Alexandre G. de Brevern |
  | Author | Narayanaswamy Srinivasan |
  | Volume | 31 |
  | Issue | 12 |
  | Pages | 1467-1480 |
  | Publication | Journal of Biomolecular Structure & Dynamics |
  | ISSN | 0739-1102; 1538-0254 |
  | Date | DEC 1 2013 |
  | Extra | WOS:000326014900009 |
  | DOI | 10.1080/07391102.2012.743438 |
  | Abstract | Inter-domain linkers (IDLs)' bridge flanking domains and support inter-domain communication in multi-domain proteins. Their sequence and conformational preferences enable them to carry out varied functions. They also provide sufficient flexibility to facilitate domain motions and, in conjunction with the interacting interfaces, they also regulate the inter-domain geometry (IDG). In spite of the basic intuitive understanding of the inter-domain orientations with respect to linker conformations and interfaces, we still do not entirely understand the precise relationship among the three. We show that IDG is evolutionarily well conserved and is constrained by the domain-domain interface interactions. The IDLs modulate the interactions by varying their lengths, conformations and local structure, thereby affecting the overall IDG. Results of our analysis provide guidelines in modelling of multi-domain proteins from the tertiary structures of constituent domain components. |
  | Date Added | 2/12/2014, 1:36:22 PM |
  | Modified | 2/12/2014, 1:36:22 PM |

  ### Notes:

  - Computational study of inter-domain linkers.

    How SCOP is used:

    Dataset is composed of all high-resolution 2-domain proteins in SCOP (mention that they excluded all proteins that were not classified in SCOP).

    SCOP reference:

    Methods 

    Data-set of 3D structures of multi-domain proteins

    The data-set of multi-domain proteins was obtained by mining the PDB (http://www.pdb.org) (Berman, Kleywegt, Nakamura, & Markley, 2012) using the following criteria: the presence of a single polypeptide chain in the asymmetric unit and the biological unit; the crystallographic resolution ≤ 3.0 Å and the presence of only two continuous Structural Classification of Proteins (SCOP) (Murzin, Brenner, Hubbard, & Chothia, 1995) domains within each polypeptide chain. We did not consider protein structures without SCOP domain annotations in this data-set. The PDB accession codes are provided in Table S4. The table also gives the details and criteria used to curate the PDB to obtain (n = 290) the final data-set of two-domain proteins. This set was non-redundant at 30%.

    Data-sets of homologous structures and sequences

    Each of the 290 proteins sequences were queried against the entire PDB, using Position specific iterative blast (PSI- BLAST) (Altschul et al., 1997) at an E-value cut-off of 105 with low-complexity regions masked for five itera- tions. We ensured that the sets of homologous protein sequences picked by BLAST for every query was reliable by filtering the hits using the following criteria: sequence identity ≥ 30% and query and hit coverage ≥ 80%. A total of 691 homologous protein sequences with known 3D structure for 255 sequences were picked. Thirty-five sequences were unique in the initial data-set and had no homologous proteins matching our criteria. This summed up to a total of 928 unique multi-domain protein–homo- logue pairs. This data-set was used to compare the features of multi-domain proteins with their homologous proteins. Domain definitions for the homologous proteins were taken from SCOP. In the absence of SCOP domain defini- tions, domain boundaries were marked from the align- ments of these homologues with their corresponding SCOP annotated multi-domain protein. Apart from having homologues of known structure from the PDB, we also queried each of the 290 sequences to obtain homologues from UniProt database (Consortium, 2012) using PSI- BLAST (Altschul et al., 1997). We used the same criteria for selection and pruning of hits to obtain the final set of homologues sequences as described above. We were not able to obtain homologues for three sequences using the above-mentioned criteria.

    Identification of IDLs in multi-domain proteins

    The IDLs definition was guided by the SCOP domain definitions for the multi-domain protein data-set. The rationale is that IDLs have very little or no interactions with either of the domains which they tether. Linker fragments connecting the two domains for each of the multi-domain contain the ith (i.e. C-ter residue of the 1st SCOP domain) and i + 1 residue (i.e. N-ter residue of the 2nd SCOP domain); they can have a maximum length of 40 residues. We scanned 20 residues towards the N- and C-terminus of the ith residue to generate all possible fragments. We then computed average number of heavy atom contacts for each residue within a sphere of 4.5Å for all the fragments. The contacts within i+3 and i3 residues, while computing averages for the ith residue, were neglected. The fragments generated using SCOP boundaries showed fewer contacts per residue than when a random boundary position was chosen. The fragment with the lowest average contacts was chosen as the IDL.

  ### Attachments

  - 07391102%2E2012%2E743438.pdf
- ## Use of comparative genomics approaches to characterize interspecies differences in response to environmental chemicals: Challenges, opportunities, and research needs

  |  |  |
  | --- | --- |
  | Type | Journal Article |
  | Author | Sarah L. Burgess-Herbert |
  | Author | Susan Y. Euling |
  | Volume | 271 |
  | Issue | 3 |
  | Pages | 372-385 |
  | Publication | TOXICOLOGY AND APPLIED PHARMACOLOGY |
  | ISSN | 0041-008X |
  | Date | SEP 15 2013 |
  | DOI | 10.1016/j.taap.2011.11.011 |
  | Language | English |
  | Abstract | A critical challenge for environmental chemical risk assessment is the characterization and reduction of uncertainties introduced when extrapolating inferences from one species to another. The purpose of this article is to explore the challenges, opportunities, and research needs surrounding the issue of how genomics data and computational and systems level approaches can be applied to inform differences in response to environmental chemical exposure across species. We propose that the data, tools, and evolutionary framework of comparative genomics be adapted to inform interspecies differences in chemical mechanisms of action. We compare and contrast existing approaches, from disciplines as varied as evolutionary biology, systems biology, mathematics, and computer science, that can be used, modified, and combined in new ways to discover and characterize interspecies differences in chemical mechanism of action which, in turn, can be explored for application to risk assessment. We consider how genetic, protein, pathway, and network information can be interrogated from an evolutionary biology perspective to effectively characterize variations in biological processes of toxicological relevance among organisms. We conclude that comparative genomics approaches show promise for characterizing interspecies differences in mechanisms of action, and further, for improving our understanding of the uncertainties inherent in extrapolating inferences across species in both ecological and human health risk assessment. To achieve long-term relevance and consistent use in environmental chemical risk assessment, improved bioinformatics tools, computational methods robust to data gaps, and quantitative approaches for conducting extrapolations across species are critically needed. Specific areas ripe for research to address these needs are recommended. (C) 2011 Elsevier Inc. All rights reserved. |
  | Date Added | 10/25/2013, 4:17:08 PM |
  | Modified | 10/8/2014, 12:50:49 PM |

  ### Tags:

  - -omics
  - Biological pathway
  - Cite ASTRAL
  - Cross-species
  - Human health risk assessment
  - Molecular network
  - Selective constraints
  - Systems biology

  ### Notes:

  - Review of how comparative genomics can help to reveal differences in responses to chemical exposure in different species.

    Discussion of "challenges, opportunities, and research needs surrounding the issue of how genomics data and computational and systems level approaches can be applied to inform differences in response to environmental chemical exposure across species."

    How SCOP is used:

    background on protein structure classification

    SCOP reference:

    Examples of online resources that implement such algo- rithms, find and predict protein structures, and make 3D comparisons include: the Structural Classification of Proteins (SCOP) database (scop.mrc-lmb.com.ac.uk/scop) (Murzin et al., 1995; Lo Conte et al., 2000; Andreeva et al., 2004; Andreeva et al., 2008) with its associated collection of manually curated structural alignments, SISYPHUS (Andreeva et al., 2007); the ASTRAL Compendium for Sequence and Structure Analysis (astral.berkeley.edu) (Brenner et al., 2000; Chandonia et al., 2002, 2004); MATRAS Protein 3D Structure Compar- ison (biunit.naist.jp/matras/) (Kawabata, 2003); and, MinRMS: A Tool for Determining Protein Similarity (www.cgl.ucsf.edu/research/ minrms) (Huang et al., 2000; Jewett et al., 2003). Additional tools and resources can be found on the RCSB Protein Database (RCSB PDB) archive (www.pdb.org) (Berman et al., 2000).

  ### Attachments

  - 1-s2.0-S0041008X11004479-main.pdf
- ## Use of structural phylogenetic networks for classification of the ferritin-like superfamily

  |  |  |
  | --- | --- |
  | Type | Journal Article |
  | Author | Daniel Lundin |
  | Author | Anthony M. Poole |
  | Author | Britt-Marie Sjöberg |
  | Author | Martin Högbom |
  | URL | http://www.jbc.org/content/287/24/20565.short |
  | Volume | 287 |
  | Issue | 24 |
  | Pages | 20565–20575 |
  | Publication | Journal of Biological Chemistry |
  | Date | 2012 |
  | Accessed | 9/20/2013, 1:16:59 PM |
  | Library Catalog | Google Scholar |
  | Date Added | 10/11/2013, 10:29:15 AM |
  | Modified | 3/7/2014, 12:14:20 PM |

  ### Tags:

  - Interesting

  ### Notes:

  - Study to "bridge the gap" between sequence-based phylogenetic methods and high level protein structure classification databases.  Study 80 structures from the Ferritin-like superfamily using phylogenetic methods.  Show that sequence-based methods can help to build an internal phylogeny.

    How SCOP is used:

    Compare SCOP, CATH, and Pfam data for the 80 structures in Ferritin-like superfamily. 

    SCOP reference:

    Several important databases exist that attempt to provide high level organization to the protein universe, including Pfam (5), which is sequence-based, and SCOP (6) and CATH (7), which both use protein structural information. These databases all use measures of either sequence or structural similarity as a means of organizing proteins into families or superfamilies. Although these databases are invaluable in charting broad structural relationships, in many cases they offer conflicting classifications, and known evolutionary relationships between individual superfamily constituents are not included. To gain a better appreciation of this issue, we examined the classification of ferritin-like proteins across these three databases and sought to establish whether the application of phylogenetic methods to protein structural data (8–10) can augment classification within a well sampled superfamily, rich in data on biological function of its members. We report that structural phylogenies of the ferritin-like superfamily recover informative relation- ships between superfamily members that reflect known evolu- tionary relationships and functional roles. We conclude that phylogenetic tools can provide an important complement to established structural classifications.

  ### Attachments

  - J. Biol. Chem.-2012-Lundin-20565-75.pdf
- ## Using Amino Acid Physicochemical Distance Transformation for Fast Protein Remote Homology Detection

  |  |  |
  | --- | --- |
  | Type | Journal Article |
  | Author | Bin Liu |
  | Author | Xiaolong Wang |
  | Author | Qingcai Chen |
  | Author | Qiwen Dong |
  | Author | Xun Lan |
  | Volume | 7 |
  | Issue | 9 |
  | Publication | PLOS ONE |
  | ISSN | 1932-6203 |
  | Date | SEP 28 2012 |
  | DOI | 10.1371/journal.pone.0046633 |
  | Language | English |
  | Abstract | Protein remote homology detection is one of the most important problems in bioinformatics. Discriminative methods such as support vector machines (SVM) have shown superior performance. However, the performance of SVM-based methods depends on the vector representations of the protein sequences. Prior works have demonstrated that sequence-order effects are relevant for discrimination, but little work has explored how to incorporate the sequence-order information along with the amino acid physicochemical properties into the prediction. In order to incorporate the sequence-order effects into the protein remote homology detection, the physicochemical distance transformation (PDT) method is proposed. Each protein sequence is converted into a series of numbers by using the physicochemical property scores in the amino acid index (AAIndex), and then the sequence is converted into a fixed length vector by PDT. The sequence-order information can be efficiently included into the feature vector with little computational cost by this approach. Finally, the feature vectors are input into a support vector machine classifier to detect the protein remote homologies. Our experiments on a well-known benchmark show the proposed method SVM-PDT achieves superior or comparable performance with current state-of-the-art methods and its computational cost is considerably superior to those of other methods. When the evolutionary information extracted from the frequency profiles is combined with the PDT method, the profile-based PDT approach can improve the performance by 3.4% and 11.4% in terms of ROC score and ROC50 score respectively. The local sequence-order information of the protein can be efficiently captured by the proposed PDT and the physicochemical properties extracted from the amino acid index are incorporated into the prediction. The physicochemical distance transformation provides a general framework, which would be a valuable tool for protein-level study. |
  | Date Added | 10/25/2013, 4:29:01 PM |
  | Modified | 10/25/2013, 4:29:01 PM |

  ### Notes:

  - Present SVM-PDT method for remote homology detection.

    How SCOP is used:

    Train and validate method on SCOP data using superfamily and family levels.  Dataset was derived from ASTRAL.

    SCOP reference:

    Comparative results of the methods based on sequence composition information

    In order to compare the proposed sequence-based PDT vectorization approach with other relevant protein remote homology detection methods, the proposed method SVM-PDT was evaluated on the widely used SCOP 1.53 dataset to give an unbiased comparison with prior methods that are based on sequence composition information.

    ...

    Ten most discriminative features of SVM-PDT were selected from each of the four target SCOP 1.53 families and the results are shown in Table 2. We observed a few family-specific l variables and indices, majority of which are highly consistent with current understanding of the structure of the protein families.

    ...

    All the 4352 protein sequences in the SCOP 1.53 dataset can be converted into fixed length vectors via using PDT with b value of 8 in 200 seconds

    ...

    Methods

    Dataset description

    A common benchmark [1] was used to evaluate the perfor- mance of our method for protein remote homology detection, which is available at http://noble.gs.washington.edu/proj/svm- pairwise/. This benchmark has been used by many studies of remote homology detection methods [8,20,34], which can provide good comparability with previous methods. The benchmark contains 54 families and 4352 proteins selected from SCOP version 1.53. These proteins are extracted from the Astral database [47] and include no pair with a sequence similarity higher than an E-value of 10225. For each family, the proteins within the family are taken as positive test samples, and the proteins outside the family but within the same superfamily are taken as positive training samples. Negative samples are selected from outside of the superfamily and are separated into training and test sets.

  ### Attachments

  - journal.pone.0046633.pdf
- ## VariBench: A Benchmark Database for Variations

  |  |  |
  | --- | --- |
  | Type | Journal Article |
  | Author | Preethy Sasidharan Nair |
  | Author | Mauno Vihinen |
  | Volume | 34 |
  | Issue | 1 |
  | Pages | 42-49 |
  | Publication | Human Mutation |
  | ISSN | 1059-7794 |
  | Date | JAN 2013 |
  | Extra | WOS:000314476900005 |
  | DOI | 10.1002/humu.22204 |
  | Abstract | Several computational methods have been developed for predicting the effects of rapidly expanding variation data. Comparison of the performance of tools has been very difficult as the methods have been trained and tested with different datasets. Until now, unbiased and representative benchmark datasets have been missing. We have developed a benchmark database suite, VariBench, to overcome this problem. VariBench contains datasets of experimentally verified high-quality variation data carefully chosen from literature and relevant databases. It provides the mapping of variation position to different levels (protein, RNA and DNA sequences, protein three-dimensional structure), along with identifier mapping to relevant databases. VariBench contains the first benchmark datasets for variation effect analysis, a field which is of high importance and where many developments are currently going on. VariBench datasets can be used, for example, to test performance of prediction tools as well as to train novel machine learning-based tools. New datasets will be included and the community is encouraged to submit high-quality datasets to the service. VariBench is freely available at http://structure.bmc.lu.se/VariBench. Hum Mutat 34:42-49, 2013. (C) 2012 Wiley Periodicals, Inc. |
  | Date Added | 2/13/2014, 4:13:17 PM |
  | Modified | 10/8/2014, 1:32:38 PM |

  ### Tags:

  - benchmark
  - genetic variation
  - Mutation
  - variant effect analysis
  - variant effect prediction
  - variant position mapping

  ### Notes:

  - Varibench is a database of variation data for benchmarks.

    How SCOP is used:

    Not using SCOP.

    Just mentioned as an example of a benchmark.

    SCOP reference:

    Some other bioin- formatic benchmarks include those for protein three-dimensional structure prediction [Kolodny et al., 2005; Lo Conte et al., 2000; Orengo et al., 1997], protein function annotation [Sonego et al., 2007], protein–protein docking [Hwang et al., 2010], and gene ex- pression analysis [Cope et al., 2004; Zhu et al., 2010].

  ### Attachments

  - humu22204.pdf
  - Snapshot
- ## Verification of the PREFAB alignment database

  |  |  |
  | --- | --- |
  | Type | Journal Article |
  | Author | T. V. Astakhova |
  | Author | M. N. Lobanov |
  | Author | I. V. Poverennaya |
  | Author | M. A. Roytberg |
  | Author | V. V. Yacovlev |
  | URL | http://link.springer.com/article/10.1134/S0006350912020030 |
  | Volume | 57 |
  | Issue | 2 |
  | Pages | 133–137 |
  | Publication | Biophysics |
  | Date | 2012 |
  | Accessed | 9/23/2013, 10:21:39 AM |
  | Library Catalog | Google Scholar |
  | Date Added | 2/13/2014, 4:13:17 PM |
  | Modified | 3/7/2014, 12:15:29 PM |

  ### Notes:

  - PREFAB is a protein sequence alignment database.

    How SCOP is used:

    Get domain boundaries.

    How CATH is used:

    Do not use CATH data.

    SCOP reference:

    2.5. Domain determination. As the source of domain classification, we took SCOP v. 1.75. For every PREFAB sequence, all possible SCOP domains of the given protein chain are determined. Further identifi⬚⬚ cation of the SCOP domain(s) consists in comparing the corresponding coordinates, i.e. the coordinates of the domain and PREFAB sequence according to the protein sequence. For each domain its overlap with the PREFAB sequence is calculated. The overlap is calcu⬚⬚ lated as the length of intersection of the given SCOP domain and the PREFAB sequence divided by the length of the PREFAB sequence. If the overlap is greater than 0.95 (95%), it is taken that the PREFAB sequence is uniquely specified by the given SCOP domain. Domains for this the overlap equals zero are excluded from consideration. If there are several pos⬚⬚ sible SCOP domains, then every domain is first con⬚⬚ sidered separately, and if the sequence is not uniquely determined by one of the putative domains, then we consider the sum overlap of the remaining domains. Domains are accepted if it is greater than 0.95 (95%). If for a PREFAB sequence not a single SCOP domain is determined, then such sequence and the corre⬚⬚ sponding alignment are removed.

    ...

    We have conducted PREFAB preprocessing and selected only those alignments the sequences of which are homologous to each other. It has been disclosed that some PREFAB alignments present sequences for which the SCOP classification diverges not only at the family level but also at higher levels, such as superfam⬚⬚ ily, fold and even class.

  ### Attachments

  - art%3A10.1134%2FS0006350912020030.pdf
  - Snapshot
- ## VipD of Legionella pneumophila Targets Activated Rab5 and Rab22 to Interfere with Endosomal Trafficking in Macrophages

  |  |  |
  | --- | --- |
  | Type | Journal Article |
  | Author | Bonsu Ku |
  | Author | Kwang-Hoon Lee |
  | Author | Wei Sun Park |
  | Author | Chul-Su Yang |
  | Author | Jianning Ge |
  | Author | Seong-Gyu Lee |
  | Author | Sun-Shin Cha |
  | Author | Feng Shao |
  | Author | Won Do Heo |
  | Author | Jae U. Jung |
  | Author | Byung-Ha Oh |
  | Volume | 8 |
  | Issue | 12 |
  | Pages | e1003082 |
  | Publication | Plos Pathogens |
  | Date | December 2012 |
  | DOI | 10.1371/journal.ppat.1003082 |
  | Abstract | Upon phagocytosis, Legionella pneumophila translocates numerous effector proteins into host cells to perturb cellular metabolism and immunity, ultimately establishing intracellular survival and growth. VipD of L. pneumophila belongs to a family of bacterial effectors that contain the N-terminal lipase domain and the C-terminal domain with an unknown function. We report the crystal structure of VipD and show that its C-terminal domain robustly interferes with endosomal trafficking through tight and selective interactions with Rab5 and Rab22. This domain, which is not significantly similar to any known protein structure, potently interacts with the GTP-bound active form of the two Rabs by recognizing a hydrophobic triad conserved in Rabs. These interactions prevent Rab5 and Rab22 from binding to downstream effectors Rabaptin-5, Rabenosyn-5 and EEA1, consequently blocking endosomal trafficking and subsequent lysosomal degradation of endocytic materials in macrophage cells. Together, this work reveals endosomal trafficking as a target of L. pneumophila and delineates the underlying molecular mechanism. |
  | Date Added | 3/7/2014, 1:06:24 PM |
  | Modified | 3/7/2014, 1:06:24 PM |
- ## Viral Capsid Proteins Are Segregated in Structural Fold Space

  |  |  |
  | --- | --- |
  | Type | Journal Article |
  | Author | Shanshan Cheng |
  | Author | Charles L. Brooks |
  | URL | http://dx.doi.org/10.1371/journal.pcbi.1002905 |
  | Volume | 9 |
  | Issue | 2 |
  | Pages | e1002905 |
  | Publication | PLoS computational biology |
  | Date | February 7, 2013 |
  | Journal Abbr | PLoS Comput Biol |
  | DOI | 10.1371/journal.pcbi.1002905 |
  | Accessed | 9/19/2013, 6:31:11 PM |
  | Library Catalog | PLoS Journals |
  | Abstract | Author SummaryViruses are increasingly viewed not as pathogens that parasitize all domains of life, but as useful nanoplatforms for synthetic maneuvers in a wide range of biomedical and materials science applications. One of the most well-known examples of virus-based nanotools developed so far features viral capsules as therapeutic agents, which protect and deliver drug molecules to targeted disease sites in the human body before the drug molecules are released. In order to optimize these nano-designs to best fulfill their purposes, we first have to understand properties of the constitutive building blocks of these viral containers, so as to rationalize and guide the synthetic modification attempts. Based on the observation that viral shells are functionally unique to viruses, we hypothesize that the structure of the building blocks must also be distinct from generic proteins, given that function follows form. Our computational modeling and statistical analysis support this novel hypothesis, and recognize the folded topology of these ‘Lego’ proteins as a differentiating factor to ensure correct geometry, and consequently, proper tiling into the large complex architecture. Our findings highlight an important design principle: efforts on imparting new functionalities to virus templates should restrain from disrupting the fundamental protein fold. |
  | Date Added | 10/11/2013, 10:29:15 AM |
  | Modified | 2/20/2014, 12:38:15 PM |

  ### Tags:

  - ASTRAL
  - ASTRAL domain structures
  - Cite ASTRAL
  - Interesting

  ### Notes:

  - Diagram.

    Step 1:

    Derived viral capsid domain dataset: a nonredundant data (<40% sequence identity) of 151 virus capsid domains from ViperDB and SCOP.  Then, performed clustering by structure similarity on set of 151 and got medoids from 56 clusters as representatives.

    Step 2:

    Derived non-viral capsid dataset: nonredundant dataset (<40% sequence identity) of non virus capsid domains from ASTRAL.

    Step 3:

    Determined 210 shared folds between the capsid and non-capsid set.  For each domain in the non-capsid set, selected its nearest neighbor (via structural similarity) from the capsid set, and removed all domains from the non-capsid set whose nearest neighbor distance was above some cutoff.  Then counted the number of SCOP folds remaining in the non-capsid set.

    Step 4:

    Perfomed permutation tests to determine statistical significance of these 210 folds.  Found more folds with random subsets of 56 domains and their complements, there were more folds.  The results were consistent even when the similarity cutoff for determining nearest neighbors was varied.

    Results:

    Found 210 folds covered by nearest neighbor non-capsid set.
  - SCOP coverage insufficent.

    SCOP's classified domains were insufficient.  They had to annotate own domains using homologues.

    Excerpt:

    We collected the viral capsid protein set from the VIrus Particle ExploreR (VIPERdb) [23], which is a database of icosahedral virus capsid structures, with 319 entries in total. Altogether 1174 protein chains having at least 80 residues were extracted from these entries, as short peptides are known to assume very simple topologies. These 1174 were further cut into domains; while 452 proteins have domain annotations in SCOP, 637 proteins have homologues (sharing a sequence identity of at least 40%) that are well-annotated by SCOP. The remaining 85 were examined visually and dissected into individual domains.
  - Use SCOP's classification of protein folds to compare the 21-folds found in viral capsid proteins against "generic proteins" (all domains in SCOP that are not in viral capsid proteins).  They cluster all folds using some structure similarity score, and conclude that viral capsids are significantly segregated in structural space.

    How SCOP is used:

    Use ASTRAL representative set (filtered at 40% sequence identity) from SCOP 1.75 and label the folds under which viral capsid proteins are found.  Perform clustering on the data set using structure similarity.

    SCOP reference:

    Abstract:

    we applied a structure-alignment based clustering of all protein chains in VIPERdb filtered at 40% sequence identity to identify distinct capsid folds, and compared the cluster medoids with a non-redundant subset of protein domains in the SCOP database, not including the viral capsid entries.

    Materials and Methods:

    Data collection

    In our work, we included all of capsid, nucleocapsid and envelope proteins for analysis, which we collectively call capsid proteins, because of their common structural role in forming the viral shell despite differentiated functions in a few cases. We collected the viral capsid protein set from the VIrus Particle ExploreR (VIPERdb) [23], which is a database of icosahedral virus capsid structures, with 319 entries in total. Altogether 1174 protein chains having at least 80 residues were extracted from these entries, as short peptides are known to assume very simple topologies. These 1174 were further cut into domains; while 452 proteins have domain annotations in SCOP, 637 proteins have homologues (sharing a sequence identity of at least 40%) that are well-annotated by SCOP. The remaining 85 were examined visually and dissected into individual domains. Lastly, the non- compact domains (extended structure with little secondary structure content) are removed, leaving 1447 domains in total.

    We used the non-redundant set of 10569 proteins covering 1195 folds from the database Structural Classification Of Proteins (SCOP) 1.75 [24] filtered at 40% sequence identity, available from the ASTRAL compendium [25], to constitute our total protein set. This set was further reduced to 8921 proteins covering 1047 folds after removal of short peptides with fewer than 80 residues. The viral capsid protein set was then subtracted from the total protein set to yield the non-capsid protein set. In addition, 24 capsid proteins in the total protein set that were originally not deposited in VIPERdb were added to the capsid set and removed from the non-capsid set (Table S1). A sequence filter of 40% identity was then applied to the domains of the capsid set, which resulted in 151 domains that are sequence-wise non-redundant.

  ### Attachments

  - PLoS Full Text PDF
- ## Visualisation of variable binding pockets on protein surfaces by probabilistic analysis of related structure sets

  |  |  |
  | --- | --- |
  | Type | Journal Article |
  | Author | Paul Ashford |
  | Author | David S. Moss |
  | Author | Alexander Alex |
  | Author | Siew K. Yeap |
  | Author | Alice Povia |
  | Author | Irene Nobeli |
  | Author | Mark A. Williams |
  | Volume | 13 |
  | Pages | 39 |
  | Publication | Bmc Bioinformatics |
  | ISSN | 1471-2105 |
  | Date | MAR 14 2012 |
  | Extra | WOS:000304348400001 |
  | DOI | 10.1186/1471-2105-13-39 |
  | Abstract | Background: Protein structures provide a valuable resource for rational drug design. For a protein with no known ligand, computational tools can predict surface pockets that are of suitable size and shape to accommodate a complementary small-molecule drug. However, pocket prediction against single static structures may miss features of pockets that arise from proteins' dynamic behaviour. In particular, ligand-binding conformations can be observed as transiently populated states of the apo protein, so it is possible to gain insight into ligand-bound forms by considering conformational variation in apo proteins. This variation can be explored by considering sets of related structures: computationally generated conformers, solution NMR ensembles, multiple crystal structures, homologues or homology models. It is non-trivial to compare pockets, either from different programs or across sets of structures. For a single structure, difficulties arise in defining particular pocket's boundaries. For a set of conformationally distinct structures the challenge is how to make reasonable comparisons between them given that a perfect structural alignment is not possible. Results: We have developed a computational method, Provar, that provides a consistent representation of predicted binding pockets across sets of related protein structures. The outputs are probabilities that each atom or residue of the protein borders a predicted pocket. These probabilities can be readily visualised on a protein using existing molecular graphics software. We show how Provar simplifies comparison of the outputs of different pocket prediction algorithms, of pockets across multiple simulated conformations and between homologous structures. We demonstrate the benefits of use of multiple structures for protein-ligand and protein-protein interface analysis on a set of complexes and consider three case studies in detail: i) analysis of a kinase superfamily highlights the conserved occurrence of surface pockets at the active and regulatory sites; ii) a simulated ensemble of unliganded Bcl2 structures reveals extensions of a known ligand-binding pocket not apparent in the apo crystal structure; iii) visualisations of interleukin-2 and its homologues highlight conserved pockets at the known receptor interfaces and regions whose conformation is known to change on inhibitor binding. Conclusions: Through post-processing of the output of a variety of pocket prediction software, Provar provides a flexible approach to the analysis and visualization of the persistence or variability of pockets in sets of related protein structures. |
  | Date Added | 2/13/2014, 4:13:17 PM |
  | Modified | 3/7/2014, 12:15:30 PM |
- ## Vivaldi: Visualization and validation of biomacromolecular NMR structures from the PDB

  |  |  |
  | --- | --- |
  | Type | Journal Article |
  | Author | Pieter Hendrickx |
  | Author | Aleksandras Gutmanas |
  | Author | Gerard J. Kleywegt |
  | URL | http://onlinelibrary.wiley.com/doi/10.1002/prot.24213/full |
  | Publication | Proteins: Structure, Function, and Bioinformatics |
  | Date | 2013 |
  | Accessed | 9/20/2013, 11:10:25 AM |
  | Library Catalog | Google Scholar |
  | Short Title | Vivaldi |
  | Date Added | 2/20/2014, 12:24:01 PM |
  | Modified | 3/7/2014, 12:09:38 PM |

  ### Notes:

  - Describe Vivaldi server for the analysis, visualization, and validation of NMR structures in the PDB.

    How SCOP/CATH is used:

    Compare coverage of whole PDB and NMR entries alone.  Count the number of SCOP and CATH folds present.

    SCOP reference:

    In a table.

  ### Attachments

  - 24213\_ftp.pdf
- ## Web Tools for Predicting Metal Binding Sites in Proteins

  |  |  |
  | --- | --- |
  | Type | Journal Article |
  | Author | Vladimir Sobolev |
  | Author | Marvin Edelman |
  | URL | http://onlinelibrary.wiley.com/doi/10.1002/ijch.201200084/full |
  | Volume | 53 |
  | Issue | 3-4 |
  | Pages | 166–172 |
  | Publication | Israel Journal of Chemistry |
  | Date | 2013 |
  | Accessed | 9/20/2013, 1:19:04 PM |
  | Library Catalog | Google Scholar |
  | Date Added | 2/20/2014, 12:24:01 PM |
  | Modified | 2/20/2014, 12:24:01 PM |

  ### Notes:

  - Present methods for predicting metal binding sites.

    How SCOP is used:

    Refer to previous study in which SCOP was used for benchmarking.

    SCOP reference:

    MetSite (http://bioinf.cs.ucl.ac.uk/metsite)15 was the first method for predicting metal binding residues that was available as a web server. This method performed satisfactorily for SCOP database superfamilies,16 which are composed of large sets of evolutionarily related proteins. As output, the server maps the neural network scores for individual residues that can be easily visualized. However, it suffers from difficulties in identification of the location of metal binding sites, since MetSite is based solely on the distribution of individual residues.

  ### Attachments

  - Snapshot
- ## What can we learn from the evolution of protein-ligand interactions to aid the design of new therapeutics?

  |  |  |
  | --- | --- |
  | Type | Journal Article |
  | Author | Alicia P. Higueruelo |
  | Author | Adrian Schreyer |
  | Author | G. Richard J. Bickerton |
  | Author | Tom L. Blundell |
  | Author | Will R. Pitt |
  | URL | http://dx.plos.org/10.1371/journal.pone.0051742 |
  | Volume | 7 |
  | Issue | 12 |
  | Pages | e51742 |
  | Publication | PloS one |
  | Date | 2012 |
  | Accessed | 9/20/2013, 1:17:33 PM |
  | Library Catalog | Google Scholar |
  | Date Added | 10/11/2013, 10:29:15 AM |
  | Modified | 10/11/2013, 10:29:15 AM |

  ### Tags:

  - Drug Design
  - Evolution, Molecular
  - Humans
  - Interesting
  - Ligands
  - Models, Molecular
  - Molecular Targeted Therapy
  - Protein Binding
  - Proteins
  - Software
  - Water

  ### Notes:

  - Computational study of protein-ligand interactions in evolutionarily related proteins to compare interactions between natural and synthetic ligands.

    How SCOP is used:

    Classify their data set by SCOP family and compare the polar ratio (#polar contacts over total #contacts) for small synthetic ligands in six different SCOP families.

    SCOP reference:

    Synthetic small molecule complexes. Calculated properties versus interaction profile

    An analysis of the distribution of the polar ratio plotted against molecular weight, AlogP, surface area buried upon binding and sum of contacts has been carried out for the synthetic small molecules. Figure 4 shows these distributions color-coded by SCOP [24] family.

    ...

    The proteins belonging to the reverse transcriptase SCOP family have similar characteristics to nuclear receptor ligand-binding domain, and bind to molecules with AlogP.1, all of which have less than 15% polar contacts.

  ### Attachments

  - journal.pone.0051742.pdf
- ## What Makes a Protein Fold Amenable to Functional Innovation? Fold Polarity and Stability Trade-offs

  |  |  |
  | --- | --- |
  | Type | Journal Article |
  | Author | Eynat Dellus-Gur |
  | Author | Agnes Toth-Petroczy |
  | Author | Mikael Elias |
  | Author | Dan S. Tawfik |
  | Volume | 425 |
  | Issue | 14 |
  | Pages | 2609–2621 |
  | Publication | Journal of Molecular Biology |
  | Date | July 2013 |
  | DOI | 10.1016/j.jmb.2013.03.033 |
  | Abstract | Protein evolvability includes two elements-robustness (or neutrality, mutations having no effect) and innovability (mutations readily inducing new functions). How are these two conflicting demands bridged? Does the ability to bridge them relate to the observation that certain folds, such as TIM barrels, accommodate numerous functions, whereas other folds support only one? Here, we hypothesize that the key to innovability is polarity-an active site composed of flexible, loosely packed loops alongside a well-separated, highly ordered scaffold. We show that highly stabilized variants of TEM-1 beta-lactamase exhibit selective rigidification of the enzyme's scaffold while the active-site loops maintained their conformational plasticity. Polarity therefore results in stabilizing, compensatory mutations not trading off, but instead promoting the acquisition of new activities. Indeed, computational analysis indicates that in folds that accommodate only one function throughout evolution, for example, dihydrofolate reductase, >= 60% of the active-site residues belong to the scaffold. In contrast, folds associated with multiple functions such as the TIM barrel show high scaffold-active-site polarity (similar to 20% of the active site comprises scaffold residues) and >2-fold higher rates of sequence divergence at active-site positions. Our work suggests structural measures of fold polarity that appear to be correlated with innovability, thereby providing new insights regarding protein evolution, design, and engineering. (C) 2013 Elsevier Ltd. All rights reserved. |
  | Date Added | 3/7/2014, 12:08:00 PM |
  | Modified | 3/7/2014, 12:08:00 PM |
- ## When a domain is not a domain, and why it is important to properly filter proteins in databases

  |  |  |
  | --- | --- |
  | Type | Journal Article |
  | Author | Clare-Louise Towse |
  | Author | Valerie Daggett |
  | URL | http://onlinelibrary.wiley.com/doi/10.1002/bies.201200116/full |
  | Volume | 34 |
  | Issue | 12 |
  | Pages | 1060–1069 |
  | Publication | BioEssays |
  | Date | 2012 |
  | Accessed | 9/20/2013, 1:18:37 PM |
  | Library Catalog | Google Scholar |
  | Date Added | 10/11/2013, 10:29:15 AM |
  | Modified | 3/7/2014, 12:10:59 PM |

  ### Tags:

  - computational biology
  - dynameome
  - intrinsic disorder
  - protein structure

  ### Notes:

  - Computational study of protein domains.  Define a domain as "an autonomous folded unit".  Analyze proteins in the Consensus Dynamics Database (CDD) and find that 40% have local or global disorder.  The CDD contains consensus domains from SCOP, CATH, and Dali.

    How SCOP /CATHis used:

    The CDD uses SCOP, CATH, and Dali. Compare CDD domains with those predicted by their method for detecting autonomous folding units, and find about 40% aren't "true" domains.

    SCOP references:

    Although similarities between protein structures are often visibly apparent, classifying protein structure suffers from three main hurdles: deciding where the boundaries of a domain lie, how to group similar structures together, and when a structure is ‘‘too’’ different to be part of a group [11]. The first comprehensive attempt to group protein domains by structural similarity was in 1976 when patterns were cate- gorized across a set of 31 globular proteins [4]. Since then, there have been three leading databases that have specialized in categorization of protein structure into fold families: SCOP [12], CATH [13], and Dali [14].

    The Structural Classification of Proteins database (SCOP) started as a manual effort, with visual inspection used to identify domains and classify them based on evolutionary relationships [12]. The structures were first placed into all-a, all-b, or mixed ab classes based on the overall secondary structure content, then grouped by shared function or struc- tural features irrespective of their sequence similarity. Next, they were grouped based on sequence similarity, and finally by the nature of the conserved topologies. Due to increasing speed with which protein structures were being solved, this eventually became a partially automated effort along with refinement of the classification definitions [15].

    ...

    The consensus domain dictionary consolidated the visible landscape of protein fold space

    The motivation behind the generation of a consensus domain dictionary (CDD) [19, 20] was to gather a set of representative protein structures that could be used to systematically inves- tigate all of protein fold space and determine the principles of protein dynamics and folding. This structure-based dictionary should not be confused with the Conserved Domain Database [1, 21] that categorizes the primary sequences of protein domains from an evolutionary standpoint.

    The collation of all identified structural domains currently in SCOP, CATH, and Dali into a consensus set was initially done in 2003 using a metadata approach [19]. This was then updated in 2009 to include new protein folds discovered in that interim period [20]. The total number of metafolds increased by 595, reflecting not just newly discovered folds but also the refinement of structural classifications that occurred during this 6-year period [15, 22]. Consequently, there were some domains made obsolete, re-delineation of some domain boundaries, as well as a merging of domains and metafolds within the v2009 CDD. Once complete, there had been inclusion of 976 ‘‘new’’ metafolds, composed entirely of

  ### Attachments

  - 1060\_ftp.pdf
- ## Whole-genome Trees Based on the Occurrence of Folds and Orthologs: Implications for Comparing Genomes on Different Levels

  |  |  |
  | --- | --- |
  | Type | Journal Article |
  | Author | Jimmy Lin |
  | Author | Mark Gerstein |
  | Volume | 19 |
  | Issue | 11 |
  | Pages | 808-818 |
  | Publication | Rna-a Publication of the Rna Society |
  | ISSN | 1355-8382; 1469-9001 |
  | Date | NOV 2013 |
  | Extra | WOS:000325813900008 |
  | Abstract | We built whole-genome trees based on the presence or absence of particular molecular features, either orthologs or folds, in the genomes of a number of recently sequenced microorganisms. To put these genomic trees into perspective, we compared them to the traditional ribosomal phylogeny and also to trees based on the sequence similarity of individual orthologous proteins. We found that our genomic trees based on the overall occurrence of orthologs did not agree well with the traditional tree. This discrepancy, however, vanished when one restricted the tree to proteins involved in transcription and translation, not including problematic proteins involved in metabolism. Protein folds unite superficially unrelated sequence families and represent a most fundamental molecular unit described by genomes. We found that our genomic occurrence tree based on folds agreed fairly well with the traditional ribosomal phylogeny. Surprisingly, despite this overall agreement, certain classes of folds, particularly all-beta ones, had a somewhat different phylogenetic distribution. We also compared our occurrence trees to whole-genome clusters based on the composition of amino acids and di-nucleotides. Finally, we analyzed some technical aspects of genomic trees-e. g., comparing parsimony versus distance-based approaches and examining the effects of increasing numbers of organisms. Additional information (e. g. clickable trees) is available from http://bioinfo.mbb.yale.edu/genome/trees. |
  | Date Added | 2/12/2014, 1:36:22 PM |
  | Modified | 2/12/2014, 1:36:22 PM |

  ### Tags:

  - coverage
  - Interesting

  ### Notes:

  - Present methods for building genomic trees using presence of folds and orthologs in genomes.

    How SCOP is used:

    Use the SCOP classification to classify gene sequences from the 7 genomes studied into folds.  For each organism, create a bit string representation of the occurrence of folds and measure the distances between the strings.  Use this information to build genomic trees.

    SCOP reference:

    Folds were assigned to the genome sequences based on a previously described approach (Gerstein 1997, 1998b; Teichmann and Mitchison 1999b; Hegyi and Gerstein 1999). We compared the structure data- bank (the PDB) against the genome sequences by using both pairwise and multiple-sequence methods and standard thresholds (FASTA and PSI-BLAST, Lipman and Pearson 1985; Pearson 1996; Altschul et al. 1997). We used the SCOP classification to group the domain- level structure matches into different fold families (Murzin et al. 1995). The SCOP (structural classifica- tion of proteins) classification is assembled based on expert manual judgement, and we have augmented it with our automatically derived protein-structural alignments (Gerstein and Levitt 1998). Like the COG scheme, the SCOP classification is in wide use and ac- cepted as a reliable classification of a protein’s fold.

  ### Attachments

  - x6.pdf
- ## Yahoo! Media Relations

  |  |  |
  | --- | --- |
  | Type | Journal Article |
  | Author | Yahoo! |
  | URL | https://archive.today/20120712130315/http://docs.yahoo.com/info/misc/history.html |
  | Date | 2005 |
  | Accessed | 9/30/2005, 5:00:00 PM |
  | Date Added | 10/29/2014, 11:52:33 AM |
  | Modified | 11/3/2014, 3:30:57 PM |

  ### Attachments

  - Yahoo! Media Relations
- ## β-sheet topology prediction with high precision and recall for β and mixed α/β proteins

  |  |  |
  | --- | --- |
  | Type | Journal Article |
  | Author | Ashwin Subramani |
  | Author | Christodoulos A Floudas |
  | Volume | 7 |
  | Issue | 3 |
  | Pages | e32461 |
  | Publication | PloS one |
  | ISSN | 1932-6203 |
  | Date | 2012 |
  | Extra | PMID: 22427840 |
  | Journal Abbr | PLoS ONE |
  | DOI | 10.1371/journal.pone.0032461 |
  | Library Catalog | NCBI PubMed |
  | Language | eng |
  | Abstract | The prediction of the correct β-sheet topology for pure β and mixed α/β proteins is a critical intermediate step toward the three dimensional protein structure prediction. The predicted beta sheet topology provides distance constraints between sequentially separated residues, which reduces the three dimensional search space for a protein structure prediction algorithm. Here, we present a novel mixed integer linear optimization based framework for the prediction of β-sheet topology in β and mixed α/β proteins. The objective is to maximize the total strand-to-strand contact potential of the protein. A large number of physical constraints are applied to provide biologically meaningful topology results. The formulation permits the creation of a rank-ordered list of preferred β-sheet arrangements. Finally, the generated topologies are re-ranked using a fully atomistic approach involving torsion angle dynamics and clustering. For a large, non-redundant data set of 2102 β and mixed α/β proteins with at least 3 strands taken from the PDB, the proposed approach provides the top 5 solutions with average precision and recall greater than 78%. Consistent results are obtained in the β-sheet topology prediction for blind targets provided during the CASP8 and CASP9 experiments, as well as for actual and predicted secondary structures. The β-sheet topology prediction algorithm, BeST, is available to the scientific community at http://selene.princeton.edu/BeST/. |
  | Date Added | 10/11/2013, 10:29:15 AM |
  | Modified | 3/7/2014, 1:06:56 PM |

  ### Tags:

  - Algorithms
  - Databases, Protein
  - Models, Chemical
  - Models, Molecular
  - Proteins
  - Protein Structure, Secondary

  ### Notes:

  - Present BeST method for beta-sheet topology prediction.

    How SCOP is used:

    Background on protein structure classification.

    SCOP reference:

    In order to determine rules based on conformational and biological observations of proteins, b-sheet topologies observed in nature have been categorized into a broad set of categories. Some of the earliest work in this direction classified proteins based on tertiary structure patterns [16,17]. Subsequently, protein struc- tures have been classified in large databases like SCOP and CATH, based on the structural family that they belong to [18–21].

  ### Attachments

  - journal.pone.0032461.pdf
